# Piecewise Stereoselective Assembly of Multisubstituted Alkenes

**DOI:** 10.1021/jacs.6c09135

**Published:** 2026-06-08

**Authors:** Eli Jones, Robert T. Martin, David W. C. MacMillan

**Affiliations:** 6740 Merck Center for Catalysis at Princeton University, Princeton, New Jersey 08544, United States

## Abstract

Multisubstituted alkenes are valuable structural motifs that also serve as key intermediates in complexity-building transformations; however, their stereoselective synthesis remains a longstanding challenge. Alkenyl bromides are appealing coupling handles for alkene synthesis, though their application would be greatly enhanced if they could be coupled directly with alkyl radicals derived from feedstock alcohols. Herein, we report a nickel metallaphotoredox-enabled deoxygenative cross-coupling of alkenyl bromides with alcohols, providing a direct, modular approach to multisubstituted alkenes. This transformation exhibits a broad substrate scope across both alcohol and alkenyl bromide partners, delivering over 30 multisubstituted alkenes. Moreover, we developed a complementary stereoselective bromoalkenylation from readily synthesized *gem*-dibromoolefins to address the limited availability of stereodefined alkenyl bromides. In the presence of a phthalimide additive, selective monofunctionalization enables access to over 40 stereodefined alkenyl bromides, providing a general and stereoselective pathway to these otherwise difficult-to-access intermediates. Direct access to alkenyl bromides enables piecewise stereoselective assembly of multisubstituted alkenes through sequential alcohol coupling or stereoretentive Suzuki–Miyaura reactions. These strategies are illustrated via iterative cross-coupling sequences and the total synthesis of (+)-sponalisolide B.

## Graphical abstract

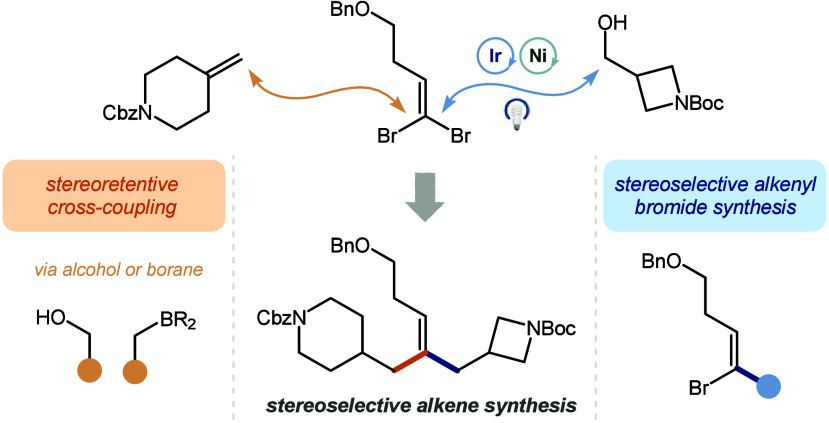

Multisubstituted alkenes are ubiquitous motifs in pharmaceuticals, natural products,^1^ and functional materials and also serve as versatile synthetic building blocks.^2,3^ Although classical alkene syntheses, including carbonyl olefination^4^ and olefin metathesis,^5^ have been reliably used to access disubstituted alkenes, the stereoselective synthesis of tri- and tetrasubstituted alkenes remains challenging.^6^ Established approaches to these motifs include alkyne difunctionalization,^7,8^ carbonyl olefination,^9^ and elimination-based strategies.^10^ However, these methods often fail to offer broad generality across substitution patterns. Cross-coupling has emerged as a powerful strategy for the modular construction of such multisubstituted alkene frameworks. In particular, highly substituted alkenes can be accessed stereoselectively via the direct coupling of structurally diverse vinyl and alkyl or aryl coupling partners.^11,12^ Representative examples include our laboratory’s decarboxylative vinylation,^13,14^ Weix’s cross-electrophile coupling of vinyl and alkyl halides,^15^ and Watson’s coupling of tetrasubstituted vinyl silanes with aryl halides.^16^ Despite these advances, preparation of the corresponding coupling partners remains challenging: alkyl substrates can be limited in generality while vinyl partners are often difficult to prepare with stereoselectivity.^12^

Recently, our group identified benzoxazolium salt reagents (termed “**NHC**”) as mild, robust activators of alcohols in deoxygenative metallaphotoredox cross-coupling reactions.^17–22^ We reasoned that we could pair this activation strategy with alkenyl bromide electrophiles to achieve alkene synthesis via direct cross-coupling. Given the commercial abundance and structural diversity of alcohols,^23^ this approach could enable a modular strategy for the assembly of multisubstituted alkenes from alkenyl bromides (Figure 1B). Alkenyl bromides are typically obtained through multistep routes from enolizable carbonyl compounds,^24^ alkynes,^25^ or functionalized alkenyl fragments.^26,27^ These strategies are often ineffective for all-alkyl alkenyl bromides, where both regio- and stereoselective synthesis can be challenging. To accomplish our desired modular alkene assembly strategy, we also sought a general and stereoselective route to the alkenyl bromide partners themselves.

**Figure 1:**
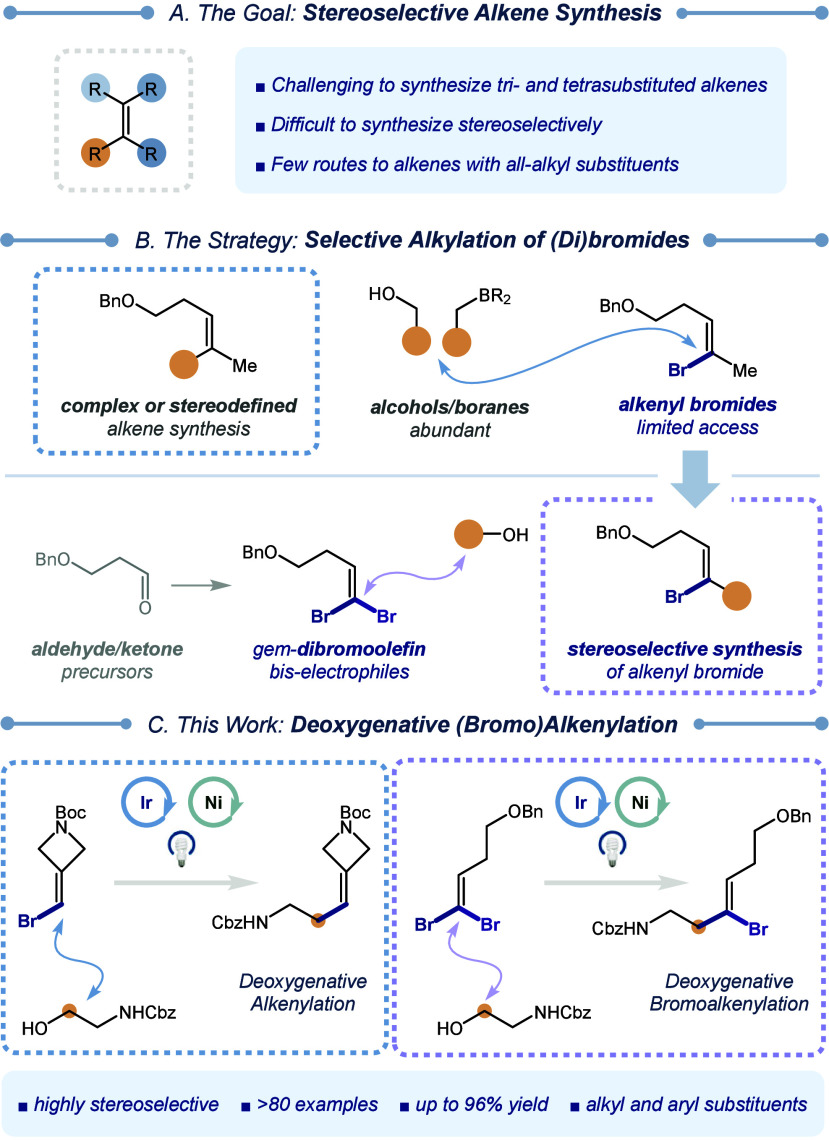
Strategic approach to stereoselective multisubstituted alkene synthesis.

*gem*-Dibromoolefins are readily accessible in a single step^28^ from commercially abundant aldehydes and ketones (Figure 1B). We envisioned that these *gem*-dibromoolefins could serve as suitable electrophiles for selective cross-coupling with alcohol-derived alkyl radicals to furnish alkenyl bromide products, which can serve as intermediates for subsequent, stereocontrolled coupling steps (Figure 1C).^29–32^ If successful, this strategy would provide one of the first general methods to stereoselectively access all-alkyl trisubstituted alkenyl bromides. We recognized two major challenges associated with such a transformation. (1) The reaction must proceed with high chemoselectivity to furnish the alkenyl bromide product, which can itself serve as a competent electrophile. Indeed, Suzuki–Miyaura functionalization of *gem*-dibromoolefins has typically shown limited chemoselectivity, with predominant double coupling observed.^30–32^ (2) The reaction must be stereoselective, functionalizing only one of the two bromides on the substrate.

Herein, we overcome both of these challenges using nickel metallaphotoredox catalysis. We further demonstrate that the resulting alkenyl bromide products—and alkenyl bromides more generally—undergo efficient cross-coupling with alkyl radicals or alkylboranes, enabling the piecewise stereoselective assembly of multisubstituted alkenes from aldehydes or ketones and alcohols.

We first set out to develop a general alcohol–alkenyl bromide cross-coupling. Following an initial optimization campaign (see Supporting Information, Figures S01–S03), we identified conditions to couple *tert*-butyl 3-(bromomethylene)­azetidine-1-carboxylate with benzyl 4-(2-hydroxyethyl)­piperidine-1-carboxylate in 87% yield (Table 1, **20**). Encouraged by these results, we evaluated the scope of the transformation with a range of alcohol coupling partners (Table 1). Primary alcohols bearing heterocyclic side chains are well tolerated (**4**, **5**), as are substrates containing alkyl and aryl bromides (**6**, **1**), highlighting selective functionalization of the alkenyl bromide over other potential electrophiles. Allylic and benzylic alcohols (**3**, **9**, **10**) are also competent substrates, furnishing skipped diene products that are often challenging to access.^33^ The reaction accommodates secondary alcohols, with broad functional-group tolerance for hydridic C–H bonds (**11**), free alcohols (**17**), and ketones (**18**). Complex alcohols are excellent substrates, including sugars (**8**), steroids (**18**), and a derivative of the antiplatelet agent ticagrelor (**19**), all of which undergo vinylation in >70% yield.

**Table 1:**
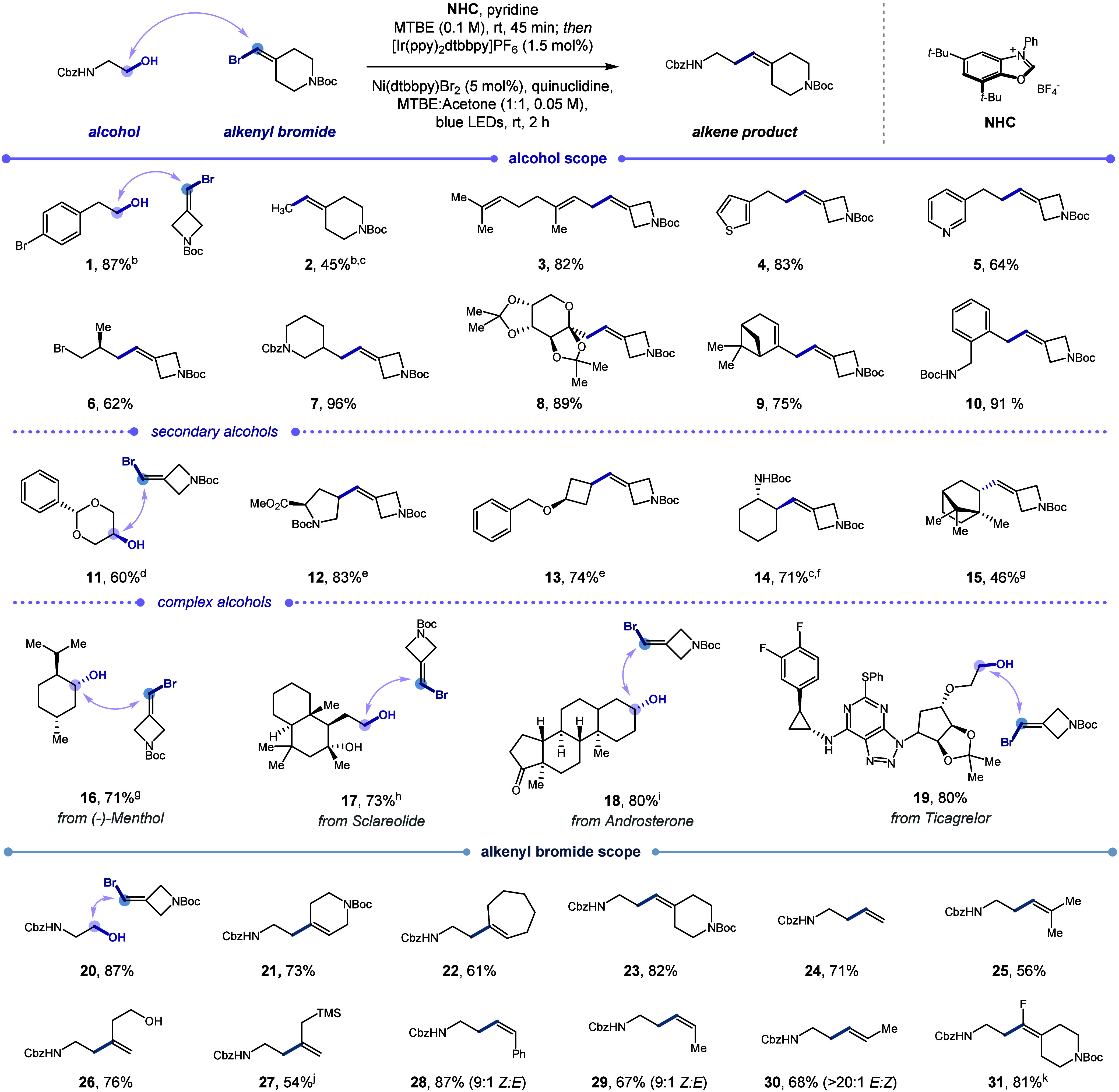
Alkylation of Alkenyl Bromides^a^

a Reactions performed with alcohol (1.75 equiv), alkenyl bromide (0.5 mmol, 1.0 equiv), 5,7-di-*tert*-butyl-3-phenylbenzo­[*d*]­oxazol-3-ium tetrafluoroborate (**NHC**, 1.9 equiv), pyridine (1.9 equiv), quinuclidine (1.9 equiv), [Ir­(ppy)_2_(dtbbpy)]­PF_6_ (1.5 mol %), Ni­(dtbbpy)­Br_2_ (5 mol %), MTBE/Acetone (1:1, 0.05 M), integrated photoreactor (450 nm, m1 plate, 100% light intensity), 2 h.

b Assay yield (by NMR vs. mesitylene as internal standard).

c 7.5 mol % Ni­(dtbbpy)­Br_2_, 0.225 equiv. phthalimide used.

d d.r. = 1.9:1.

e d.r. = 1.4:1.

f d.r. = 5:1.

g d.r. > 20:1.

h Alcohol condensation performed in 1,4-dioxane, 1.0 equiv. phthalimide used.

i d.r. = 3.2:1.

j 0.225 equiv. phthalimide used.

k 1.0 equiv. phthalimide used. See Supporting Information for experimental details.

We then explored the scope of the alkenyl bromide coupling partner. Alkenyl bromides embedded within six- (**21**) and seven-membered rings (**22**), as well as trisubstituted alkenyl bromides (**23**, **25**), furnish products in good yields. The reaction tolerates pendant functionalities on the alkenyl bromide, including a free alcohol (**26**) and a trimethylsilyl group (**27**). We were pleased to find that the deoxygenative vinylation proceeds with retention of stereochemistry for unsymmetric alkenyl bromides, although partial erosion of stereochemical fidelity was observed with (*Z*)-1-bromoprop-1-ene (**29**). Finally, a 1-bromo-1-fluoroalkene furnishes the fluoroalkene product in 81% yield, with full chemoselectivity for the bromide (**31**).

Next, we set out to achieve the stereoselective synthesis of alkenyl bromides from *gem*-dibromoolefin precursors, themselves readily prepared from commercially available aldehydes and ketones (see the Supporting Information). Under optimized alcohol–alkenyl bromide coupling conditions (*vide supra*) with increased nickel loading, the alkenyl bromide product was obtained, along with significant amounts of the corresponding dialkylated product (Figure S04). To suppress this undesired pathway, we evaluated additives that could impact the oxidative addition step. Our group has previously shown that phthalimide is an effective additive in nickel-catalyzed metallaphotoredox reactions, where it is proposed to stabilize the oxidative addition adduct and suppress off-cycle reactivity.^34^ Gratifyingly, we observed that addition of 1.0 equiv of phthalimide both enhanced the yield of the desired alkenyl bromide and suppressed dialkylation (see Supporting Information for further details). We next sought to optimize for product stereoselectivity. Following extensive evaluation of conditions using an aldehyde-derived unsymmetric *gem*-dibromoolefin (**51**, Figures S05–S10), we found that changing the solvent system to 1:2 MTBE:1,2-dichlorobenzene led to marked improvements in both yield and stereoselectivity.

With optimized conditions in hand, we explored the scope of this transformation (Table 2). Primary alcohols are particularly well-tolerated, including those bearing potentially challenging motifs, such as phosphonate (**36**), pyridine (**38**), and trifluoromethyl groups (**41**). Excitingly, an alcohol substrate bearing an aryl bromide reacted with high efficiency—even in the presence of three potential sites for oxidative addition, only a single stereodefined product is observed (**39**). Benzylic alcohols are also efficient coupling partners, affording 2-bromoallylarene products (**40**, **41**). A variety of secondary alcohols bearing four- (**42**), five- (**44**, **46**), six- (**43**), and seven-membered rings (**45**) furnish the desired products in good yields. Finally, even complex alcohols, including sugars (**47**, **50**) and alcohol-containing drugs (**48**, **49**), serve as effective alkyl radical sources.

**Table 2:**
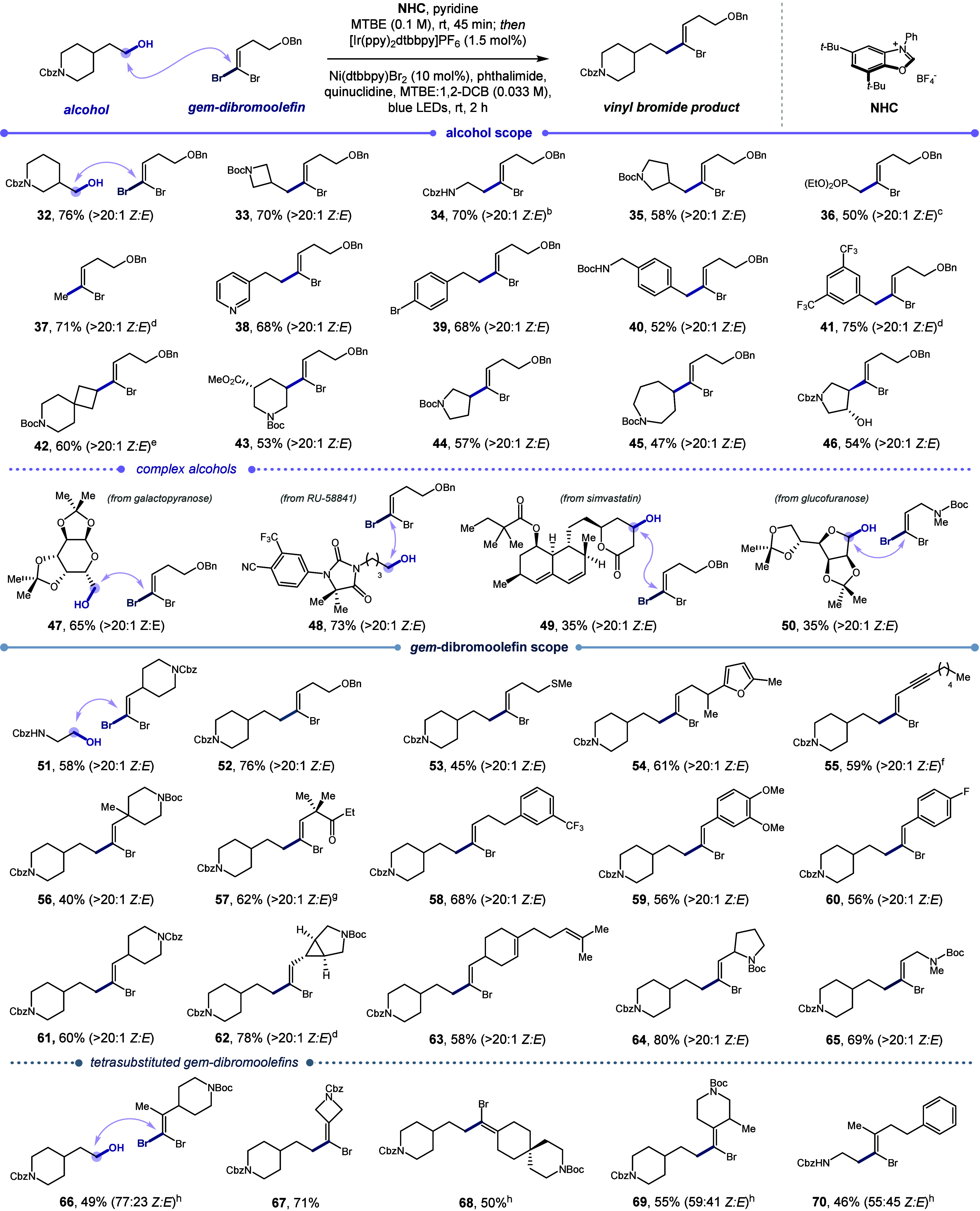
Deoxygenative Alkylation of *gem*-Dibromoolefins^a^

a Reactions performed with alcohol (1.3 equiv), *gem*-dibromoolefin (0.5 mmol, 1.0 equiv), **NHC** (1.4 equiv), pyridine (1.4 equiv), [Ir­(ppy)_2_(dtbbpy)]­PF_6_ (1.5 mol %), Ni­(dtbbpy)­Br_2_ (10 mol %), quinuclidine (1.4 equiv), phthalimide (1.0 equiv), MTBE:1,2-dichlorobenzene (1:2, 0.033 M), integrated photoreactor (450 nm, m1 plate, 100% light intensity), 2 h.

b 15 mL DCB used.

c 1.75 equiv of alcohol used.

d Assay yield (by NMR vs mesitylene as internal standard).

e 1.5 equiv of alcohol used.

f 0.45 mmol scale.

g 0.4 mmol scale.

h Trifluorotoluene used as cosolvent with MTBE. See Supporting Information for experimental details.

We next examined the scope of the *gem*-dibromoolefin component. Substrates containing tertiary amides (**51**), thioethers (**53**), and furans (**54**) underwent coupling with 4-(2-hydroxyethyl)­piperidine-1-carboxylate in good yields, as did conjugated *gem*-dibromoolefins, which efficiently formed the corresponding bromostyrenes (**59**, **60**) and bromoenynes (**55**). Tertiary substitution at the olefin α-carbon (**56**, **57**) did not meaningfully impair reactivity. Notably, substrates bearing a free carbonyl group were compatible with both the *gem*-dibromoolefin synthesis and subsequent cross-coupling conditions (**57**). Finally, *gem*-dibromoolefins bearing six- (**61**), three- (**62**), and five-membered rings (**64**) were well-tolerated.

We then applied these conditions to the synthesis of tetra-substituted alkenes from ketone-derived *gem*-dibromoolefins. A variety of *gem*-dibromoolefins underwent cross-coupling in good yields, although asymmetric tetra-substituted *gem*-dibromoolefins (**66**, **69**, **70**) suffered erosion of stereoselectivity compared to aldehyde-derived *gem*-dibromoolefins. This may arise from unselective oxidative addition owing to the similar steric environments of the two Br atoms in the *gem*-dibromoolefin, or from an XAT-type oxidative addition^35^ at more hindered C–Br bonds, producing a vinyl radical that can rapidly isomerize before nickel radical capture.

To showcase the synthetic utility of this transformation, we undertook the piecewise, stereoselective assembly of multisubstituted alkenes (Figure 2). Stereo*selective* synthesis of the alkenyl bromide could be followed by a stereo*retentive* Suzuki–Miyaura cross-coupling^11^ with an alkylborane to yield *all*-alkyl trisubstituted alkenes. Delightfully, either of the two stereoisomers can be accessed with excellent selectivity by exchanging the alcohol and alkylborane partners ((*E*)-**71**, *(Z)* **-71**). This piecewise procedure is also effective with primary and secondary alcohol partners in conjunction with trimethylboroxine^36^ (**72**) or alkylboranes (**73**).

**Figure 2:**
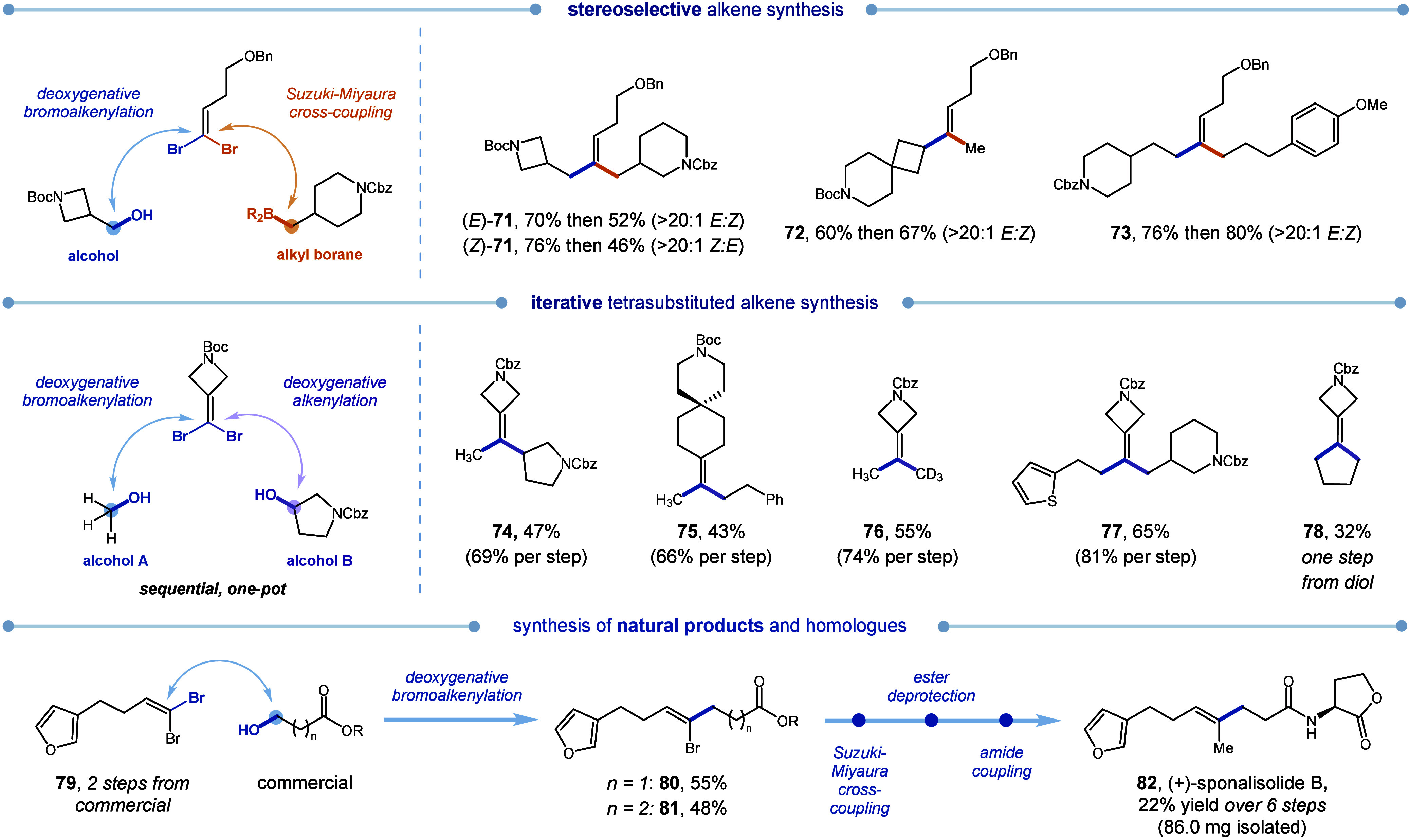
Sequential strategies toward piecewise assembly of multisubstituted alkenes. (See the Supporting Information for experimental details.)

We next envisioned a one-flask synthesis of tetrasubstituted alkenes via sequential addition of two distinct **NHC**-activated alcohol partners to a reaction vessel containing the *gem*-dibromoolefin substrate. To our delight, this iterative procedure can be deployed using methyl (**74**, **75**, **76**), primary (**75**, **77**), and secondary (**74**) alcohol partners, producing tetrasubstituted alkenes in high yields with excellent cross-selectivity. Additionally, 1,4-butanediol can form a tetrasubstituted alkene in a single step (**78**), wherein the doubly **NHC**-activated diol can engage in coupling and subsequent 5-*exo*-trig cyclization to furnish the desired product.

Stereodefined alkenes are prevalent motifs in natural products, yet their stereoselective synthesis often relies on substrate-specific strategies tailored to the alkene target. To highlight the modularity of our approach, we envisioned its application to the synthesis of (+)-sponalisolide B, a natural product isolated from *Spongia officinalis* that exhibits quorum-sensing inhibitory activity.^37^ Deoxygenative alkylation enables direct synthesis of (+)-sponalisolide B from simple starting materials. Moreover, by using an alternative commercially available alcohol, we synthesized a homologue (**81**) that is inaccessible via previously reported protocols (Figure 2). More broadly, this modular approach provides common alkenyl bromide intermediates en route to stereodefined alkene-containing natural products and their analogues.

A plausible mechanism for the deoxygenative bromoalkenylation of *gem*-dibromoolefins is shown in Figure 3. Following activation of the alcohol by **NHC**, blue light irradiation generates an excited-state iridium photocatalyst, which undergoes reductive single-electron transfer (SET) with the alcohol-NHC adduct **83** to form the reduced photocatalyst and the alcohol-**NHC** radical cation, **83** ^**•+**^. Subsequent deprotonation and rapid β-scission of **83** ^**•+**^ generates the desired alkyl radical (**85** ^**•**^). Concurrently, reduced nickel­(I) species **86** undergoes facile oxidative addition with the *gem*-dibromoolefin to generate a bromovinyl nickel­(III) species, **87**. Separately, the reduced iridium photocatalyst can undergo SET with a nickel­(II) species (**88**) to yield nickel­(I) species **89**. Comproportionation^35^ of **89** and **87** furnishes a nickel­(II) species (**90**), primed for radical capture of alkyl radical **85** ^**•**^. Rapid reductive elimination of the resultant nickel­(III) species (**91**) generates the desired deoxygenative bromoalkenylation product and reforms **86**. We propose that the high stereoselectivity observed with aldehyde-derived *gem*-dibromoolefins can be attributed to high selectivity in the oxidative addition toward the C–Br bond furthest from the alkene substituent. We further propose that phthalimide may suppress subsequent oxidative addition of the deoxygenative bromoalkenylation product to the nickel by reducing Ni­(I) reactivity (see Supporting Information).

**Figure 3:**
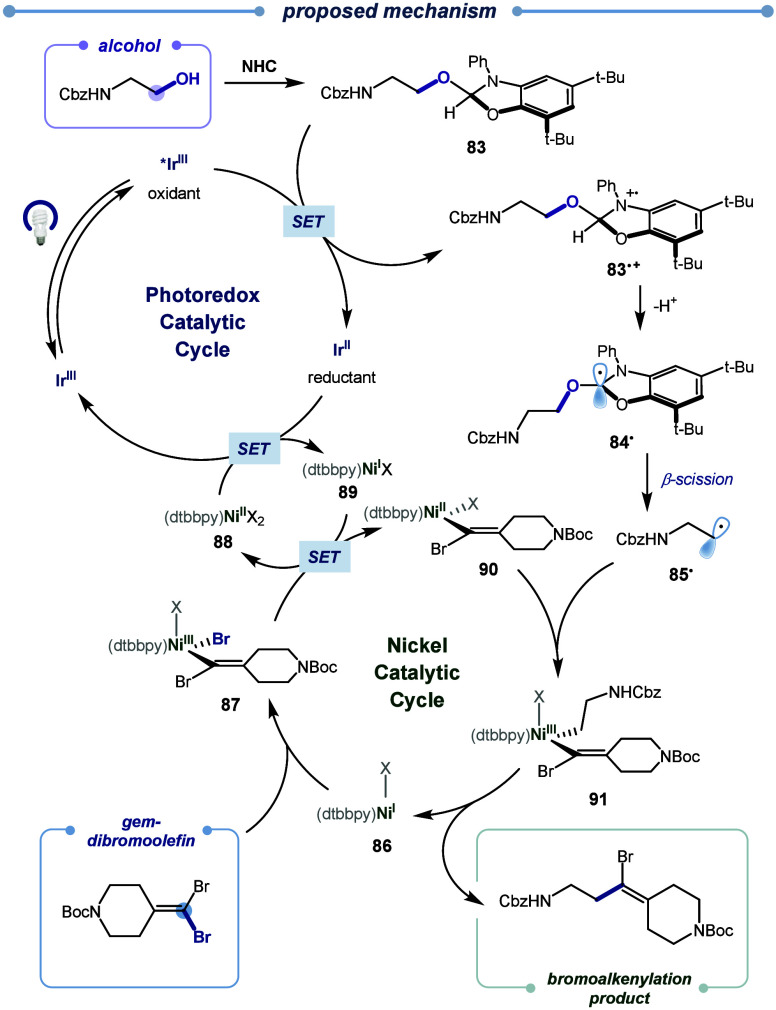
Proposed mechanism.

Herein we have reported the direct deoxygenative nickel metallaphotoredox-catalyzed cross-coupling of alcohols with alkenyl bromides and *gem*-dibromoolefins to generate multisubstituted alkenes and stereodefined alkenyl bromides. These reactions proceed with excellent functional group tolerance and—in the case of trisubstituted alkenes and alkenyl bromides—high stereoselectivity. This platform enables iterative, piecewise assembly of multisubstituted alkenes, providing a new pathway for the synthesis of these highly valued motifs from feedstock building blocks. We anticipate that the modularity and functional group tolerance of this platform will find broad application in the synthesis of complex alkene-containing molecules.

## Supplementary Material



## Notes

Additional experimental details, optimization studies, mechanistic studies, compound characterization, and spectra (PDF)
