# Supplementary material for: Piecewise Stereoselective Assembly of Multisubstituted Alkenes
Source: J Am Chem Soc. 2026 Jun 8;148(23):23499–505. doi: 10.1021/jacs.6c09135 (PMC13281536; doi:10.1021/jacs.6c09135)
Supplement: Supplementary file 1 [file ja6c09135_si_001.pdf]

Supporting Information

Piecewise Stereoselective Assembly of Multi-Substituted Alkenes

Eli Jones†, Robert T. Martin†, and David W. C. MacMillan*

Merck Center for Catalysis at Princeton University, Princeton, New Jersey 08544, USA

*Corresponding author. Email: dmacmill@princeton.edu

†These authors contributed equally

Table of Contents

1) General Information S3

2) General Procedures S5

3) Optimizations and Control Experiments S10

4) Additional Experiments S21

5) Experimental Data for Deoxygenative Alkenylation Products S26

6) Experimental Data for Deoxygenative Bromoalkenylation Products S57

7) Experimental Data for *gem*-Dibromoolefin Starting Materials S102

8) Experimental Data for Stereoselective Alkene Synthesis S120

9) Experimental Data for Iterative Tetrasubstituted Alkene Synthesis S126

10) Experimental Data for Total Synthesis of (+)-sponalisolide B S132

11) Spectral Data for Isolated Products S137

12) References S327

1) General Information

Commercial reagents were used without prior purification unless otherwise indicated. All solvents were obtained commercially in Sure/Seal™ packaged bottles. Alcohol activation reagent **NHC** (deoxazole, 5,7-di-*tert*-butyl-3-phenylbenzo[*d*]oxazol-3-ium tetrafluoroborate) was prepared according to literature procedures.[1] Organic solutions were concentrated under reduced pressure on a Büchi™ rotary evaporator using a water bath. Crude reaction mixtures containing high boiling point solvents were concentrated on a *Genevac*™ HT-4X Centrifugal Vacuum Evaporator Series II machine at 45°C under 1.5 mbar for 5 to 10 hours.

Chromatographic purification of products was performed on an automated Teledyne ISCO CombiFlash® NextGen 300+ system using RediSep Rf Gold® Silica Gel Disposable Flash Columns (20–40 microns). Preparative High Performance Liquid Chromatography (prepHPLC) purification was performed on a Teledyne ISCO ACCQPrep® HP150 system using Waters™ XBridge BEH C18 OBD Prep Column (30 mm x 150 mm, 130 Å, 5 μm) with 0.1% ammonium hydroxide buffered water and acetonitrile solutions. Thin-layer chromatography (TLC) was performed on Silicycle® 0.25 mm or Supelco® 0.20 mm silica gel F-254 plates. Visualization of the developed chromatogram was performed by fluorescence quenching or $KMnO_4$ staining.

^{1}H and ^{13}C NMR spectra were recorded on a Bruker™ Avance III NMR 500 MHz instrument and are internally referenced to the residual proteo-solvent signals ($CDCl_3$ referenced at 7.26 and 77.16 ppm, respectively; DMSO-d_6 referenced at 2.50 and 39.52 ppm, respectively; benzene-d_6 referenced at 7.16 and 128.06 ppm, respectively). ^{19}F NMR spectra were recorded on a Bruker™ Avance III NMR 500 MHz instrument and are reported unreferenced. Data for ^{1}H and ^{19}F NMR are reported as follows: chemical shift (δ ppm), multiplicity (s = singlet, d = doublet, t = triplet, q = quartet, p = pentet, h = hextet, m = multiplet, br = broad), coupling constant (Hz), and integration. Data for ^{13}C NMR were reported in terms of chemical shift; multiplicity and coupling constants are included only in the case of coupling with ^{19}F nuclei.

Liquid chromatography (LC) analysis was performed on an Agilent™ 1200 or Agilent™ 1290 Infinity II LC system. Infrared (IR) spectroscopy was performed on a Thermo™ Nicolet 6700

FTIR spectrometer with diamond Smart Orbit ATR accessory, and spectra are reported in wavenumbers (cm^{-1}). High resolution mass spectra (HRMS) were obtained from the Princeton University Mass Spectral Facility on Agilent™ 6220 ESI-TOF LC/MS or Agilent™ 7200 EI-QTOF GC/MS systems.

2) General Procedures

General Procedure A: Deoxygenative alkenylation

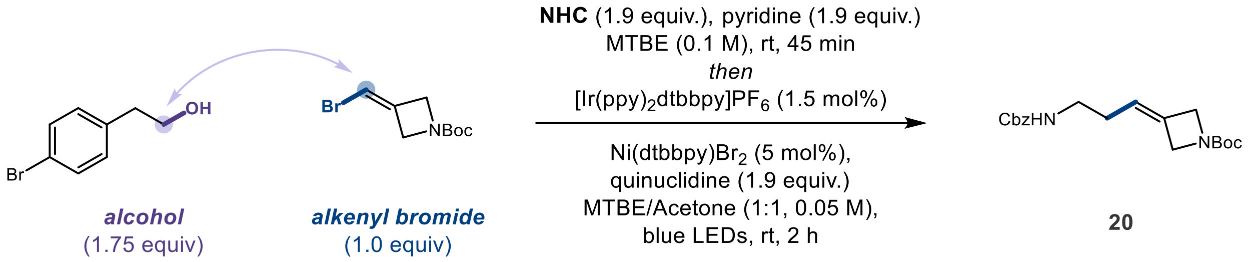

20

***NHC Condensation* (Stock A):** To an oven-dried 40 mL vial equipped with a cross-shaped stir bar was added the alcohol (1.75 equiv.) and 5,7-di-*tert*-butyl-3-phenylbenzo[*d*]oxazol-3-ium tetrafluoroborate (**NHC**, 1.90 equiv.) followed by the addition of anhydrous *t*-BuOMe. (If the alcohol was a liquid, the alcohol would be dissolved in *t*-BuOMe before **NHC** was added). Pyridine (1.90 equiv.) was added dropwise at room temperature, and the suspension was stirred under nitrogen atmosphere for 45 minutes. The resulting suspension was then syringe filtered, yielding a homogenous solution (**Stock A**). This solution was used immediately.

***Catalyst stock solution* (Stock B)** To an oven-dried 40 mL vial was added $NiBr_2$(dtbbpy) (0.005 equiv.), photocatalyst (0.015 equiv.), quinuclidine (1.9 equiv.), and alkenyl bromide (0.5 mmol, 1.0 equiv.). Acetone was then added (5 mL, 0.10 M relative to dibromide). The mixture was sonicated until homogenous.

Stock A was added by syringe to the vial containing **Stock B**. The reaction mixture was then sparged with nitrogen for 5 minutes. The vial was then sealed with parafilm and placed in a PennPhD m1 integrated photoreactor (m1 450 nm LED plate, 100% intensity, 5200 rpm fans, 1000 rpm stirring) and irradiated for 2 hours. These procedures were modified as necessary to screen the variables outlined *vide infra*.

General Procedure B: Deoxygenative bromoalkenylation

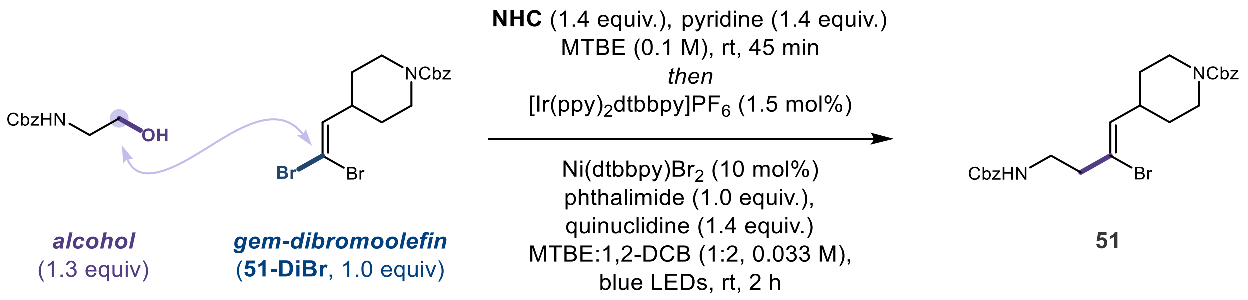

***NHC Condensation* (Stock A):** To an oven-dried 40 mL vial equipped with a cross-shaped stir bar was added the alcohol (1.30 equiv.) and 5,7-di-*tert*-butyl-3-phenylbenzo[*d*]oxazol-3-ium tetrafluoroborate (**NHC**, 1.40 equiv.) followed by the addition of anhydrous t-BuOMe. (If the alcohol was a liquid, the alcohol would be dissolved in *t*-BuOMe before **NHC** was added). Pyridine (1.40 equiv.) was added dropwise at room temperature, and the suspension was stirred under nitrogen atmosphere for 45 minutes. The resulting suspension was then syringe filtered, yielding a homogenous solution (**Stock A**). This solution was used immediately.

***Catalyst stock solution* (Stock B):** To an oven-dried 40 mL vial was added $NiBr_2$(dtbbpy) (0.01 equiv.), photocatalyst (0.015 equiv.), quinuclidine (1.4 equiv.), and *gem*-dibromoolefin (0.5 mmol, 1.0 equiv.). 1,2-dichlorobenzene was then added (10 mL, 0.05 M relative to dibromide). The mixture was sonicated until homogenous.

Stock A (5 mL) was added by syringe to the vial containing **Stock B**. The reaction mixture was then sparged with nitrogen for 5 minutes. The vial was then sealed with parafilm and placed in a PennPhD m1 integrated photoreactor (m1 450 nm LED plate, 100% intensity, 5200 rpm fans, 1000 rpm stirring) and irradiated for 2 hours. These procedures were modified as necessary to screen the variables outlined *vide infra*.

General Procedure C: *gem*-dibromoolefin synthesis from aldehydes

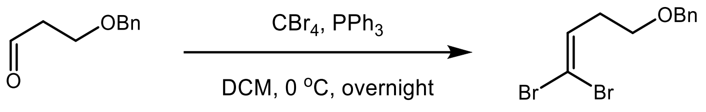

52-DiBr

A solution of carbon tetrabromide (2.25 equiv.) in CH_2Cl_2 (0.25 M) at 0 °C was prepared. To this solution, triphenylphosphine (4.5 equiv.) flake was added portionwise over the course of 15 minutes. During this time, the solution turned from clear yellow to clear orange. This solution was stirred at 0 °C for 1 hour. After 1 hour, a solution of the aldehyde (1.0 equiv.) in CH_2Cl_2 (0.667 M relative to the aldehyde) was added dropwise over 30 minutes using a syringe pump. This mixture was allowed to warm to room temperature with stirring overnight. The solution was then poured into stirring hexane (0.05 M relative to the aldehyde), filtered, and the filtrate concentrated under reduced pressure via rotary evaporation. The residue was purified by automated flash chromatography. The pure fractions were combined and concentrated under reduced pressure to afford the desired *gem*-dibromoolefin product.

General Procedure D: *gem*-dibromoolefin synthesis from hindered ketones

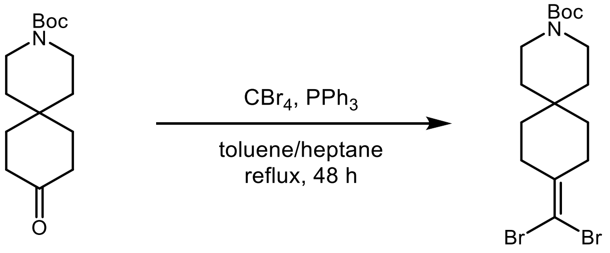

68-DiBr

A solution of ketone (1.0 equiv.), carbon tetrabromide (3.0 equiv.), and triphenylphosphine (6.0 equiv.) in toluene or heptane (0.05 M) was prepared. This solution was refluxed under nitrogen atmosphere for 48 hours. The solution was then poured into stirring hexane (0.05 M relative to the ketone), filtered, and the filtrate concentrated under reduced pressure via rotary evaporation. The residue was purified by automated flash chromatography. The pure fractions were combined and concentrated under reduced pressure to afford the desired *gem*-dibromoolefin product.

General Procedure E: Iterative tetrasubstituted alkene synthesis

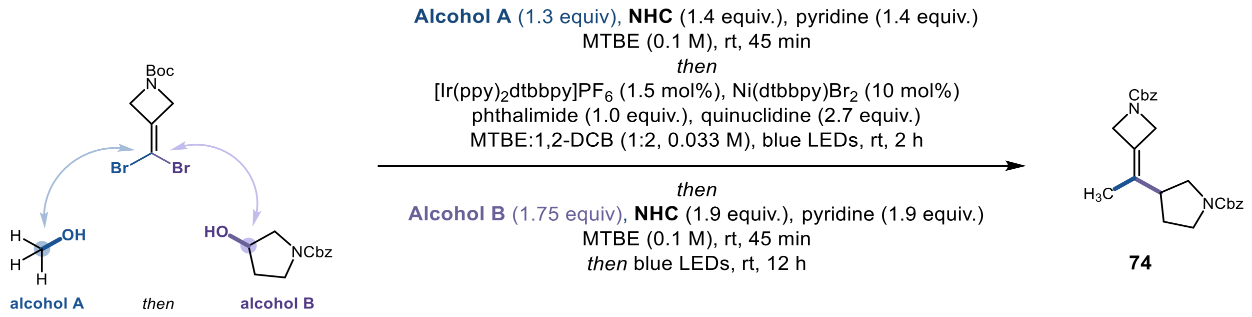

NHC Condensation **(Stock A):** To an oven-dried 40 mL vial equipped with a cross-shaped stir bar was added alcohol A (1.30 equiv.) and 5,7-di-*tert*-butyl-3-phenylbenzo[*d*]oxazol-3-ium tetrafluoroborate (**NHC**, 1.40 equiv.) followed by the addition of anhydrous *t*-BuOMe. (If the alcohol was a liquid, the alcohol would be dissolved in *t*-BuOMe before **NHC** was added). Pyridine (1.40 equiv.) was added dropwise at room temperature, and the suspension was stirred under nitrogen atmosphere for 45 minutes. The resulting suspension was then syringe filtered, yielding a homogenous solution (**Stock A**). This solution was used immediately.

Catalyst stock solution **(Stock B):** To an oven-dried 40 mL vial was added $NiBr_2$(dtbbpy) (0.01 equiv.), photocatalyst (0.015 equiv.), quinuclidine (1.4 equiv.), and *gem*-dibromoolefin (0.5 mmol, 1.0 equiv.). 1,2-dichlorobenzene was then added (10 mL, 0.05 M relative to dibromide). The mixture was sonicated until homogenous.

Stock A (5 mL) was added by syringe to the vial containing **Stock B**. The reaction mixture was then sparged with nitrogen for 5 minutes. The vial was then sealed with parafilm and placed in a PennPhD m1 integrated photoreactor (m1 450 nm LED plate, 100% intensity, 5200 rpm fans, 1000 rpm stirring) and irradiated for 2 hours.

NHC Condensation **(Stock C):** To an oven-dried 40 mL vial equipped with a cross-shaped stir bar was added alcohol B (1.75 equiv.) and 5,7-di-*tert*-butyl-3-phenylbenzo[*d*]oxazol-3-ium tetrafluoroborate (**NHC**, 1.90 equiv.) followed by the addition of anhydrous *t*-BuOMe. (If the alcohol was a liquid, the alcohol would be dissolved in *t*-BuOMe before **NHC** was added). Pyridine (1.90 equiv.) was added dropwise at room temperature, and the suspension was stirred

under nitrogen atmosphere for 45 minutes. The resulting suspension was then syringe filtered, yielding a homogenous solution (**Stock C**). This solution was used immediately.

After the reaction mixture finished 2 hours of irradiation, **Stock C** (5 mL) was added by syringe to the vial. The reaction mixture was then sparged with nitrogen for 5 minutes. The vial was then sealed with parafilm and placed in a PennPhD m1 integrated photoreactor (m1 450 nm LED plate, 100% intensity, 5200 rpm fans, 1000 rpm stirring) and irradiated for an additional 12 hours.

3) Optimizations and Control Experiments

Optimization of deoxygenative alkenylation

Figure S01: Iridium photocatalyst x Nickel catalyst screen for deoxygenative alkenylation.

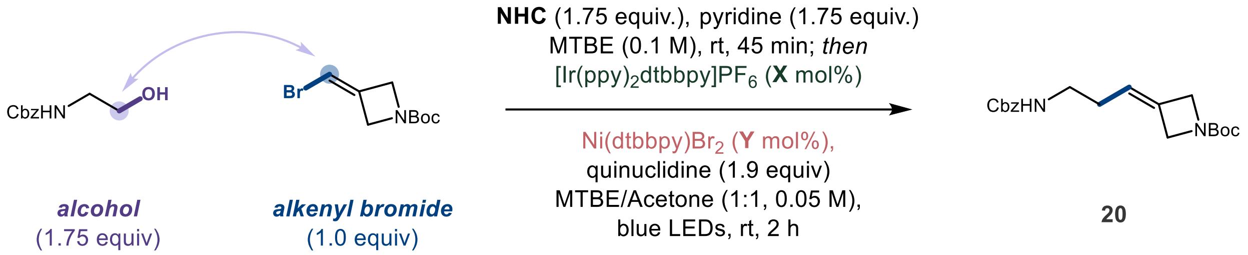

Ir loading, X mol% \ Ni loading, Y mol%	1.0%	2.5%	5.0%	10.0%
0.25%	19%	64%	80%	84%
0.50%	68%	65%	81%	81%
1.0%	70%	67%	85%	83%
1.5%	64%	57%	**87%**	79%

Yields determined by UPLC vs. mesitylene

In an initial set of optimization experiments, iridium photocatalyst and nickel catalyst loading were screened. Decreasing the photocatalyst loading does not dramatically reduce reaction efficiency. Decreasing nickel loading reduces reactivity significantly, and increasing the nickel loading beyond 5.0 mol% does not improve reactivity. Decreasing both photocatalyst loading and nickel catalyst loading does significantly impair reactivity.

Figure S02: Cosolvent screen for deoxygenative alkenylation.

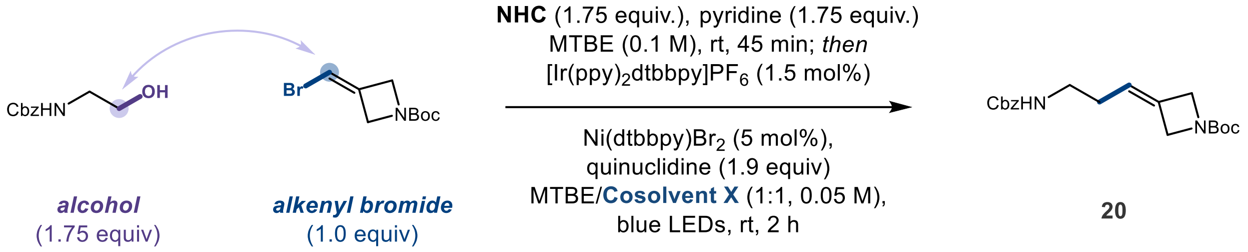

20

Cosolvent X	*Assay Yield* (20)
**Acetone**	**84%**
DMA	73%
DMF	80%
MeCN	61%
DMSO	72%
NMP	72%

Yields determined by UPLC vs. mesitylene

Deoxygenative alkenylation was found to be effective with various reaction cosolvents, though acetone was found to be the most enabling cosolvent with *t*-BuOMe in a 1:1 ratio.

Figure S03: Control experiments for deoxygenative alkenylation.

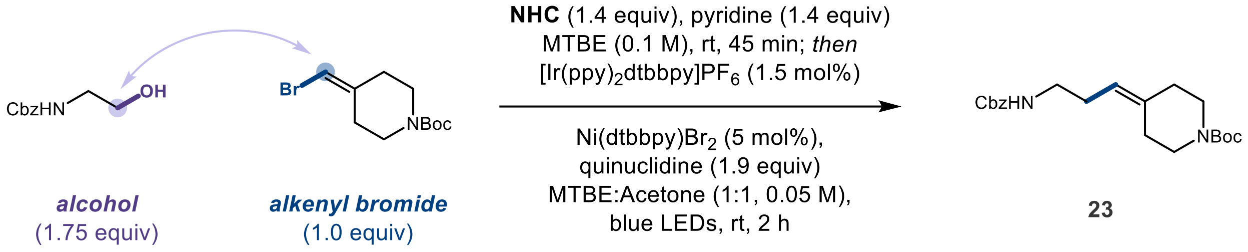

Deviation from Standard Conditions	*Assay Yield* (23)
**no deviation**	**82%**
no $[Ir(ppy)_2dtbbpy]PF_6$	0%
no $Ni(dtbbpy)Br_2$	0%
Add phthalimide (1 equiv)	50%
No quinuclidine	0%
No light	0%

Yields determined by ^{1}H NMR vs. mesitylene

Control experiments confirmed that all components of the reaction are necessary for optimal cross-coupling reactivity. Notably, addition of one equivalent of phthalimide did not inhibit reactivity completely, though efficiency was reduced.

Optimization of deoxygenative bromoalkenylation

With optimized conditions for deoxygenative alkenylation in hand, initial experiments had determined that bromoalkenylation can proceed with 40% yield with the following changes to the optimal alkenylation conditions: Reduction of alcohol equivalents (to 1.3 equiv.) as well as corresponding **NHC** (1.4 equiv.), pyridine (1.4 equiv.), and quinuclidine (1.4 equiv.); Increase of Ni(dtbbpy)Br_2 loading (to 10 mol%); and DMA as cosolvent (1:1 with *t*-BuOMe). These conditions, however, also resulted in the formation of 18% of dialkylation product (benzyl 3-(3,11-dioxo-1,13-diphenyl-2,12-dioxa-4,10-diazatridecan-7-ylidene)azetidine-1-carboxylate).

Figure S04: Optimization of phthalimide as an additive for deoxygenative bromoalkenylation.

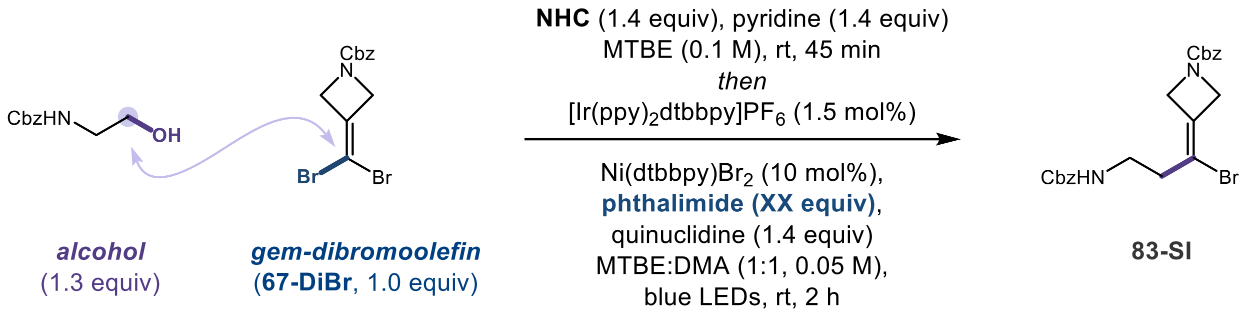

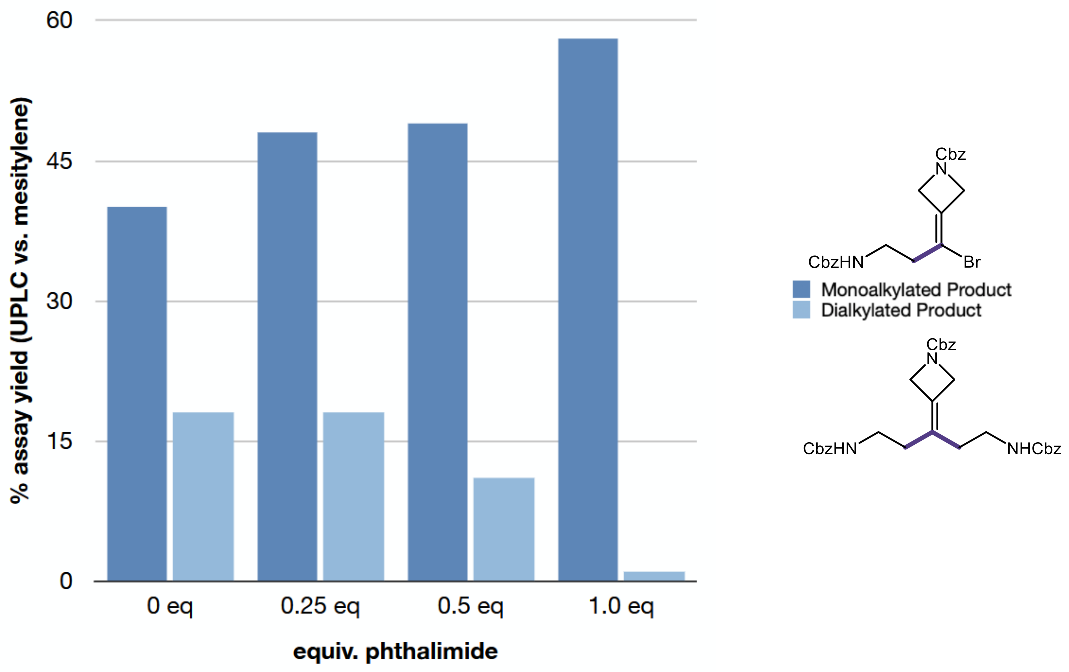

We sought to optimize the deoxygenative bromoalkenylation while preventing dialkylation of the *gem*-dibromoolefin. We determined that addition of phthalimide could solve both of these problems, as it resulted in higher yields of desired bromoalkenylation product and reduction in the yield of the dialkylated product. 1 equivalent of phthalimide as an additive was found to be optimal.

Figure S05: Optimization of nickel catalyst ligand for deoxygenative bromoalkenylation of aldehyde derivatives.

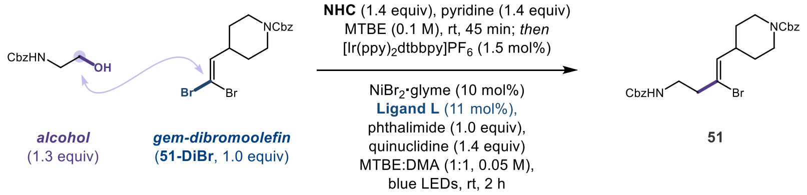

Ligand L (11 mol%)	*Assay Yield* **51** *(d.r.)*	*Ligand L (11 mol%)*	*Assay Yield* **51** *(d.r.)*	*Ligand L (11 mol%)*	*Assay Yield* **51** *(d.r.)*
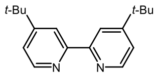	**58%** *(90:10)*	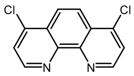	40% *(83:17)*	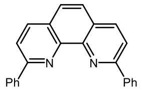	0% *(N/A)*
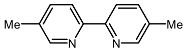	58% *(91:9)*	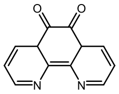	28% *(71:29)*	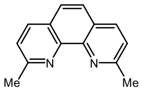	0% *(N/A)*
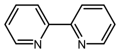	54% *(89:11)*	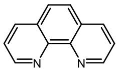	49% *(90:10)*	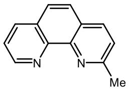	0% *(N/A)*
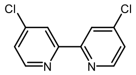	44% *(89:11)*	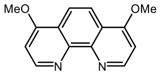	53% *(81:19)*	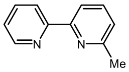	0% *(N/A)*
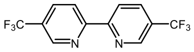	0% *(N/A)*	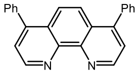	50% *(88:12)*	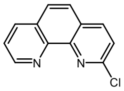	0% *(N/A)*
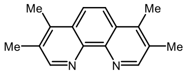	52% *(85:15)*	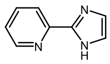	42% *(83:17)*	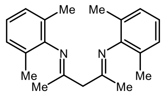	0% *(N/A)*
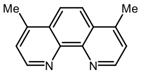	50% *(86:14)*	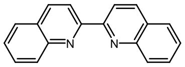	0% *(N/A)*	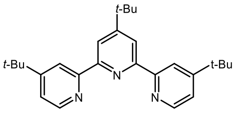	0% *(N/A)*

Yields and d.r.'s determined by ^{1}H NMR vs. mesitylene

Given the newly found enabling phthalimide additive, we moved on to optimize the deoxygenative bromoalkenylation of an aldehyde-derived *gem*-dibromoolefin. To this end, we sought to optimize both the yield and the diastereoselectivity of the bromoalkenylation. We began with a screen of the ligand on the nickel catalyst. We determined that 4,4'-di-tert-butyl-2,2'-bipyridine (dtbbpy)

resulted in the highest yield of the ligands tested, while also maintaining good stereoselectivity (Z:E = 90:10). Other bipyridine ligands worked well, though introduction of trifluoromethyl groups did inhibit reactivity completely. Phenanthroline ligands and a pyox ligand were also effective, though the diastereoselectivity was typically significantly reduced with these ligand classes. Notably, increase of steric hindrance at the *ortho*-position in the pyridyl rings completely suppressed reactivity and led to full recovery of the *gem*-dibromoolefin starting material. This suggests that the reaction system, and particularly the oxidative addition, is very sensitive to sterics.

Figure S06: Screen of photocatalysts for deoxygenative bromoalkenylation of aldehyde derivatives.

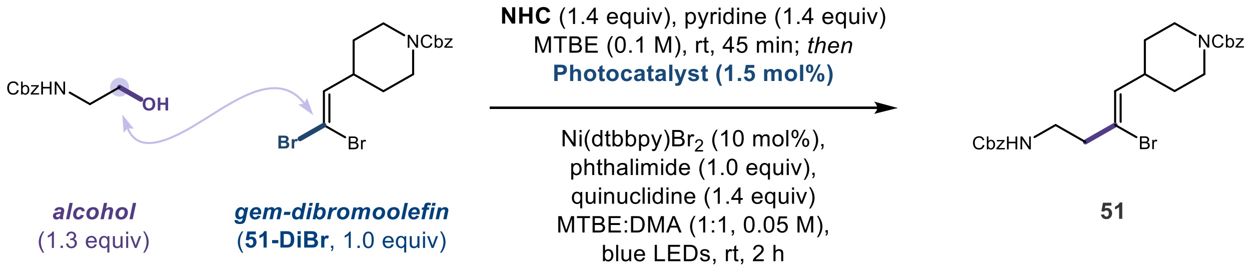

Photocatalyst (1.5 mol%)	*Assay Yield* **51** *(d.r.)*
**[Ir(ppy)$_2$dtbbpy]PF$_6$**	**67% *(94:6)***
[Ir(F(Me)ppy)$_2$dtbbpy]PF$_6$	66% *(92:8)*
[Ir(dF(Me)ppy)$_2$dtbbpy]PF$_6$	61% *(93:7)*
[Ir(dFFppy)$_2$dtbbpy]PF$_6$	63% *(94:6)*
[Ir(dFCF$_3$ppy)$_2$dtbbpy]PF$_6$	61% *(93:7)*
4-CzIPN	65% *(92:8)*

Yields and d.r.'s determined by ^{1}H NMR vs. mesitylene

We found that this reaction was effective with a variety of different iridium photocatalysts, as well as an organic photocatalyst (4-CzIPN).

Figure S07: Cosolvent screen for deoxygenative bromoalkenylation of aldehyde derivatives.

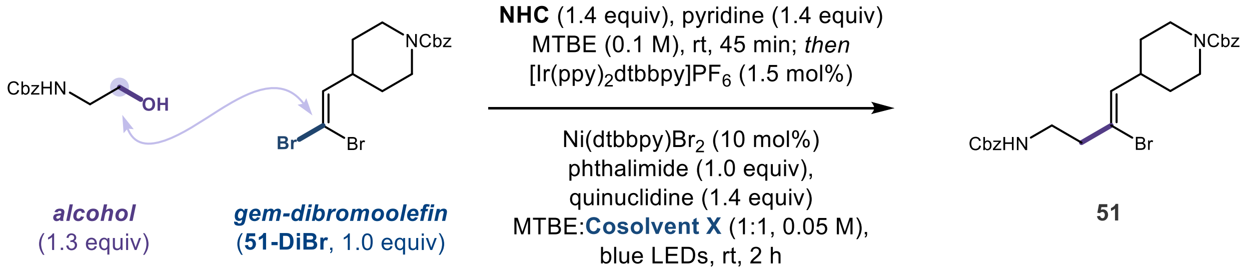

Cosolvent X	*Assay Yield 51 (d.r.)*	*Cosolvent X*	*Assay Yield 51 (d.r.)*
**1,2-dichlorobenzene**	**67% *(96:4)***	EtOAc	20% *(91:9)*
3-methyl-2-butanone	0% *(N/A)*	dimethyl carbonate	33% *(94:6)*
Acetone	25% *(95:5)*	ethylene glycol	1% *(N/A)*
2-butanone	35% *(95:5)*	fluorobenzene	64% *(95:5)*
DMA	47% *(89:11)*	1,4-difluorobenzene	57% *(93:7)*
DMA (no MTBE)	49% *(92:8)*	trifluoromethoxybenzene	57% *(95:5)*
MeCN	30% *(95:5)*	1,3-bisbenzotrifluoride	56% *(95:5)*
DMSO	30% *(92:8)*	phenyl ether	58% *(93:7)*
DMSO (no MTBE)	45% *(95:5)*	chlorobenzene	47% *(96:4)*
tert-amyl alcohol	20% *(90:10)*	DCM	0% *(N/A)*
DMF	51% *(90:10)*	1,2-DCE	67% *(96:4)*
tert-butanol	18% *(93:7)*	dioxane	50% *(92:8)*
trifluorotoluene	47% *(96:4)*	*tert*-butylbenzene	65% *(89:11)*
HMPA	4% *(N/A)*	toluene	53% *(91:9)*

Yields and d.r.'s determined by ^{1}H NMR vs. benzyl benzoate

We screened a variety of cosolvents with *t*-BuOMe for this reaction to see if we could improve reactivity and stereoselectivity. Gratifyingly, we found that 1,2-dichlorobenzene (1,2-DCB) was able to improve both the reaction yield and the stereoselectivity of the transformation. The reaction can proceed efficiently in a variety of different solvents, though more polar solvents tended to reduce reactivity. Interestingly, halogenated benzene derivatives tended to result in improved yields, and often improved stereoselectivity.

Figure S08: Concentration screen for deoxygenative bromoalkenylation of aldehyde derivatives.

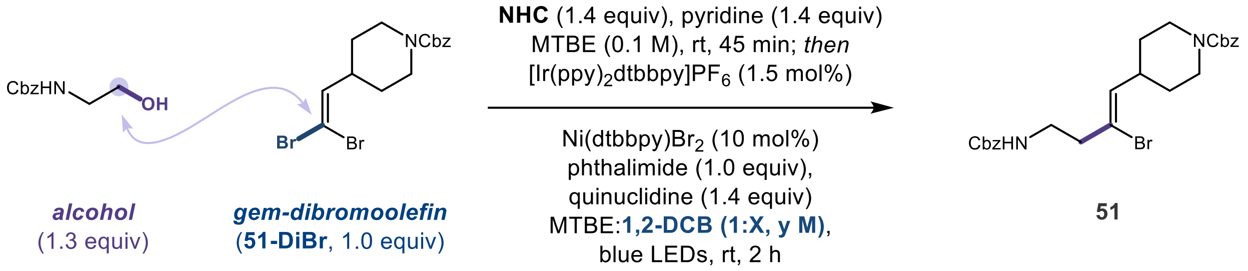

51

MTBE : 1,2-DCB (concentration)	***Assay Yield 51*** *(d.r.)*
1 mL : 0.5 mL (0.067 M)	55% *(96:4)*
1 mL : 1.0 mL (0.050 M)	62% *(97:3)*
**1 mL : 2.0 mL (0.033 M)**	**65% *(95:5)***
1 mL : 3.0 mL (0.025 M)	64% *(95:5)*
1 mL : 4.0 mL (0.020 M)	64% *(94:6)*
1 mL : 5.0 mL (0.017 M)	60% *(92:8)*

Yields and d.r.'s determined by ^{1}H NMR vs. benzyl benzoate

Following our discovery of 1,2-DCB as an optimal reaction solvent, we determined that decreasing the concentration of the reaction by addition of an extra mL of 1,2-DCB (relative to the *t*-BuOMe) further improved reactivity and stereoselectivity.

Figure S09: Nickel catalyst screen for deoxygenative bromoalkenylation of aldehyde derivatives.

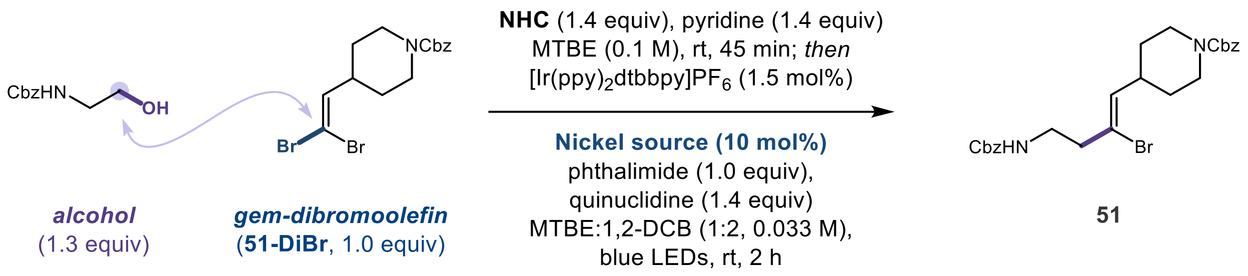

Nickel source (10 mol%)	***Assay Yield 51*** *(d.r.)*
**Ni(dtbbpy)Br_2**	**67% *(96:4)***
Ni(dtbbpy)Cl_2	65% *(97:3)*
$NiBr_2$ · glyme + dtbbpy	69% *(96:4)*
$NiCl_2$ · glyme + dtbbpy	67% *(96:4)*
$Ni(BF_4)_2$ · H_2O + dtbbpy	43% *(88:11)*
$Ni(OAc)_2$ · 4 H_2O + dtbbpy	47% *(94:6)*

Yields and d.r.'s determined by ^{1}H NMR vs. benzyl benzoate

We determined that nickel(II) catalysts were very effective for this reaction, and that changing the anion did not improve reactivity or selectivity. Additionally, using precomplexed Ni(dtbbpy)Br_2 or having $NiBr_2$·glyme with dtbbpy added to form the catalyst in situ resulted in similar yield and selectivity.

Figure S10: Time study for deoxygenative bromoalkenylation of aldehyde derivatives.

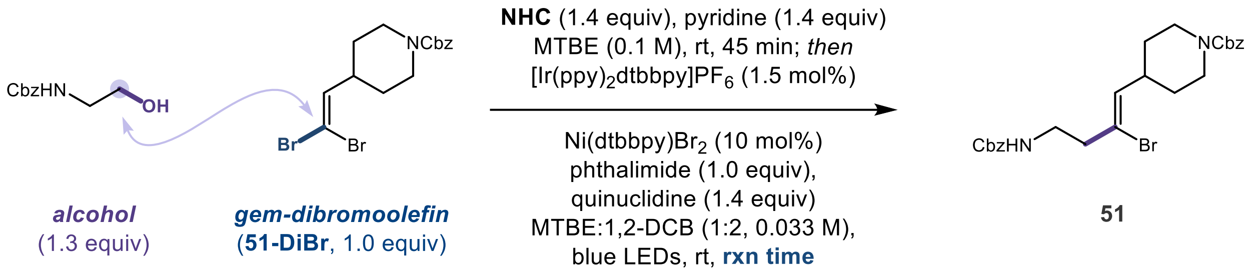

Reaction time	***Assay Yield* 51** *(d.r.)*
15 minutes	32% *(97:3)*
1 hour	66% *(94:6)*
**2 hours**	**67% *(96:4)***
4 hours	61% *(95:5)*

Yields and d.r.'s determined by ^{1}H NMR vs. benzyl benzoate

We then sought to determine the amount of time necessary for the reaction to reach completion. On performing a time study, we found that the reaction is typically complete after 2 hours.

Figure S11: Control experiments for deoxygenative bromoalkenylation of aldehyde derivatives.

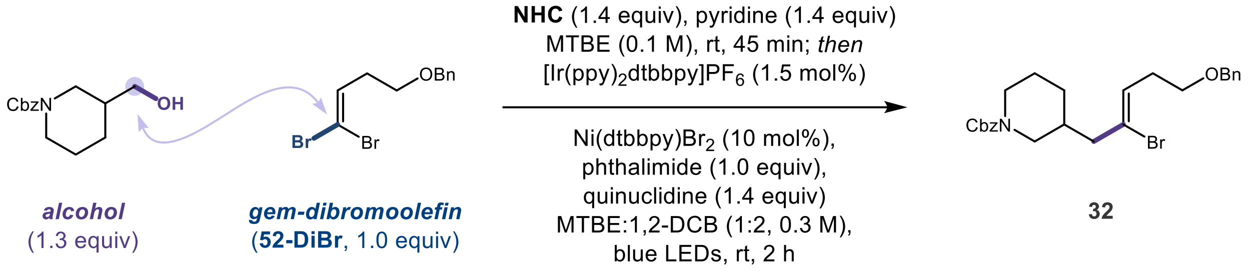

Deviation from Standard Conditions	***Assay Yield* (32)**
**no deviation**	**76%**
no $[Ir(ppy)_2dtbbpy]PF_6$	0%
no $Ni(dtbbpy)Br_2$	0%
No phthalimide	38%
No quinuclidine	0%
No light	0%

Yields determined by ^{1}H NMR vs. mesitylene

Control experiments revealed that all components of the reaction are necessary for desirable reactivity and selectivity. Removal of phthalimide did not inhibit reactivity completely, though efficiency was reduced significantly and resulted in formation of undesirable dialkylated product.

4) Additional Experiments

Role of phthalimide in inducing chemoselectivity

During optimization of the deoxygenative bromoalkenylation, we found that addition of phthalimide suppressed dialkylation of the *gem*-dibromoolefins. To further probe the effects of phthalimide in our system, we conducted time studies on the bromoalkenylation of **67-DiBr** to form **83-SI** (monoalkylated product), and the alkylation of **83-SI** (to form the dialkylated product).

Figure S12: Time study of deoxygenative (bromo)alkenylation.

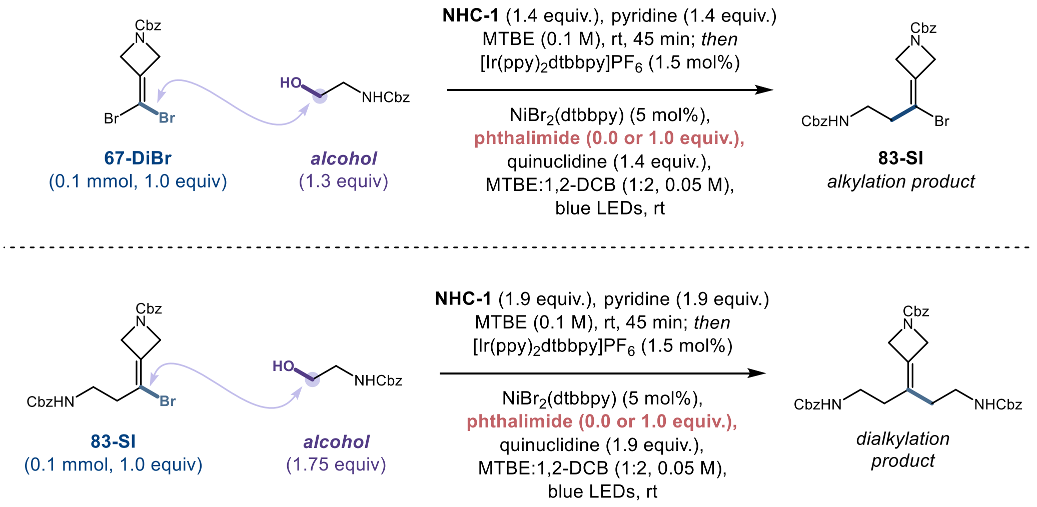

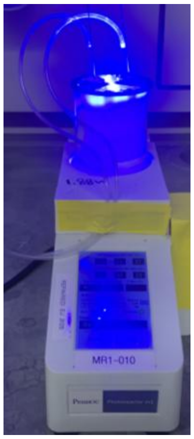

Experimental setup

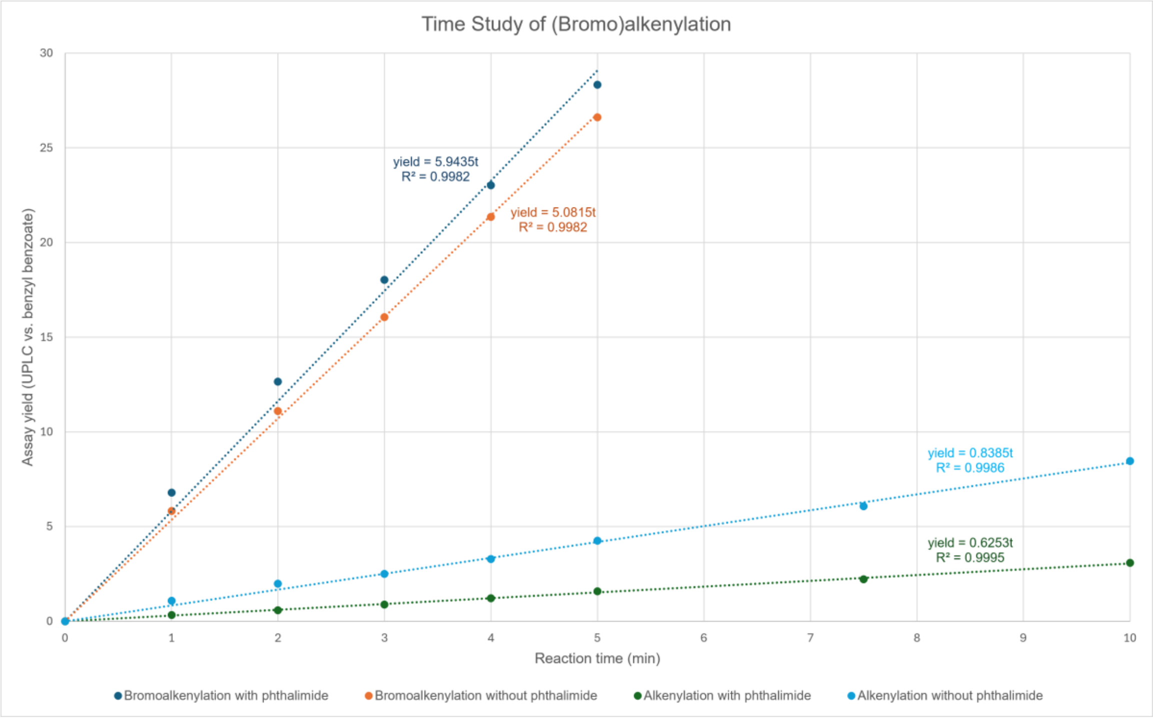

Notably, addition of phthalimide did not significantly hinder the bromoalkenylation of **67-DiBr**. We propose that the highly electrophilic nature of the gem-dibromoolefin allows for enhanced reactivity regardless of the presence of phthalimide. This is further supported by the faster initial rate of reaction for the bromoalkenylation conditions (~6.1x faster without phthalimide, ~9.5x faster with phthalimide), compared to the alkenylation reactions tested. On the other hand, we found that *addition of phthalimide significantly slowed the rate of alkenylation of* **83-SI**. Specifically, addition of phthalimide resulted in approximately a 1.3x reduction in reaction rate.

Based on this time study, and the observation in **Figure S03** that addition of phthalimide reducing efficiency of deoxygenative alkenylation, we propose that the role of phthalimide in inducing chemoselectivity is to slow the rate of oxidative addition. This may be due to steric hindrance in a Ni(I)-phthalimido complex or steric hindrance from phthalimide stabilizing the ligand sphere of the catalytically active nickel species. Slower oxidative addition would allow for off-cycle reactivity of the alkyl radicals derived from the alcohol partner (e.g. dimerization). This would result in consumption of the alcohol before it can be engaged in the cross-coupling, thereby preventing overalkylation.

Limitations of tertiary alcohols as coupling partners

Following optimization of deoxygenative alkenylation with primary and secondary alcohols, we sought to determine if tertiary alcohols could be competent coupling partners. To facilitate condensation of these tertiary alcohols, we used **NHC-5** (5,7-Di-tert-butyl-3-(4-(trifluoromethoxy)phenyl)benzo[d]oxazol-3-ium tetrafluoroborate) in trifluorotoluene, rather than the typical **NHC** in MTBE. Unfortunately, following screens of reaction conditions with various tertiary alcohol and alkenyl bromide partners (**Figure S13-S14**), we found tertiary alcohols were not viable substrates with our transformation.

Figure S13: Nickel catalyst screen for deoxygenative alkenylation conditions for tertiary alcohols.

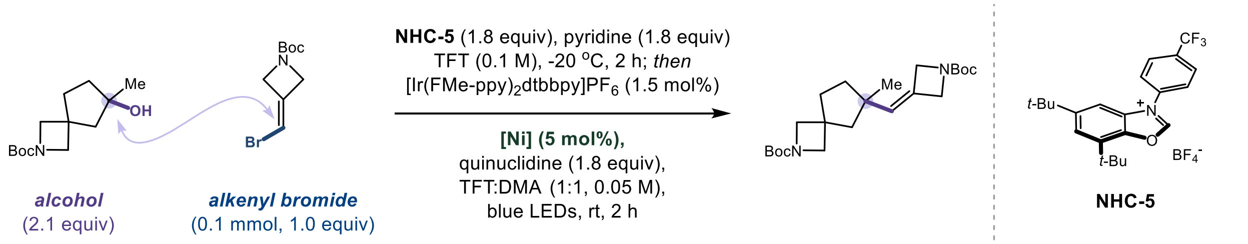

	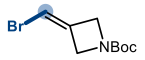		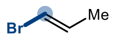		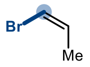	
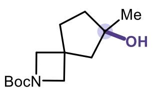	**$Ni(TMHD)_2$**	*n.d.*	**$Ni(TMHD)_2$**	*n.d.*	**$Ni(TMHD)_2$**	*n.d.*
	**$Ni(acac)_2$**	*n.d.*	**$Ni(acac)_2$**	*n.d.*	**$Ni(acac)_2$**	*n.d.*
	**$NiBr_2$·glyme + dtbbpy**	*n.d.*	**$NiBr_2$·glyme + dtbbpy**	*n.d.*	**$NiBr_2$·glyme + dtbbpy**	*n.d.*
	**$Ni(BF_4)_2$·6 H_2O + dtbbpy**	*n.d.*	**$Ni(BF_4)_2$·6 H_2O + dtbbpy**	*n.d.*	**$Ni(BF_4)_2$·6 H_2O + dtbbpy**	*n.d.*
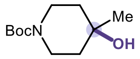	**$Ni(TMHD)_2$**	*n.d.*	**$Ni(TMHD)_2$**	*n.d.*	**$Ni(TMHD)_2$**	*n.d.*
	**$Ni(acac)_2$**	*n.d.*	**$Ni(acac)_2$**	*n.d.*	**$Ni(acac)_2$**	*n.d.*
	**$NiBr_2$·glyme + dtbbpy**	*n.d.*	**$NiBr_2$·glyme + dtbbpy**	*n.d.*	**$NiBr_2$·glyme + dtbbpy**	*n.d.*
	**$Ni(BF_4)_2$·6 H_2O + dtbbpy**	*n.d.*	**$Ni(BF_4)_2$·6 H_2O + dtbbpy**	*n.d.*	**$Ni(BF_4)_2$·6 H_2O + dtbbpy**	*n.d.*
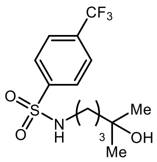	**$Ni(TMHD)_2$**	*n.d.*	**$Ni(TMHD)_2$**	*n.d.*	**$Ni(TMHD)_2$**	*n.d.*
	**$Ni(acac)_2$**	*n.d.*	**$Ni(acac)_2$**	*n.d.*	**$Ni(acac)_2$**	*n.d.*
	**$NiBr_2$·glyme + dtbbpy**	*n.d.*	**$NiBr_2$·glyme + dtbbpy**	*n.d.*	**$NiBr_2$·glyme + dtbbpy**	*n.d.*
	**$Ni(BF_4)_2$·6 H_2O + dtbbpy**	*n.d.*	**$Ni(BF_4)_2$·6 H_2O + dtbbpy**	*n.d.*	**$Ni(BF_4)_2$·6 H_2O + dtbbpy**	*n.d.*

(Assay yield determined by UPLC vs. Mesitylene)

Figure S14: Nickel catalyst and additive screen for deoxygenative alkenylation conditions for tertiary alcohols.

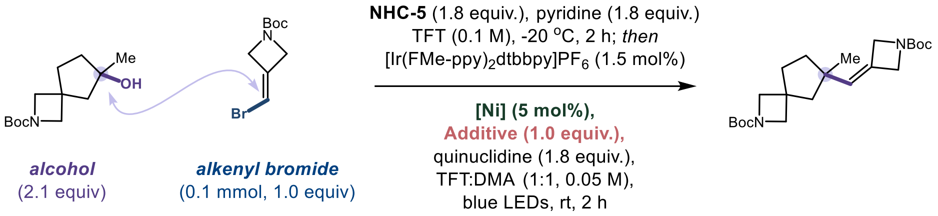

Nickel source (5 mol%)	*Additive (1.0 equiv.)*	*Assay Yield*
$Ni(TMHD)_2$	LiBr	*n.d.*
	phthalimide	*n.d.*
$Ni(acac)_2$	LiBr	*n.d.*
	phthalimide	*n.d.*
$NiBr_2$·glyme + dtbbpy	LiBr	*n.d.*
	phthalimide	*n.d.*
$Ni(BF_4)_2$·6 H_2O + dtbbpy	LiBr	*n.d.*
	phthalimide	*n.d.*

(Assay yield determined by UPLC vs. Mesitylene)

vic-dibromoolefins as coupling partners

We were interested in determining whether *vic*-dibromoolefins could also be viable substrates using our reaction conditions. This would provide an alternative route to multi-substituted alkenes and enhance the synthetic utility of this transformation. Towards this end, we synthesized a number of *vic*-dibromoolefins (according to the method of He et al.[2]) and subjected them to our standard reaction conditions. The results of these experiments are summarized in **Figure S15** below.

Figure S15: *vic*-Dibromoolefins as substrates for deoxygenative bromoalkenylation.

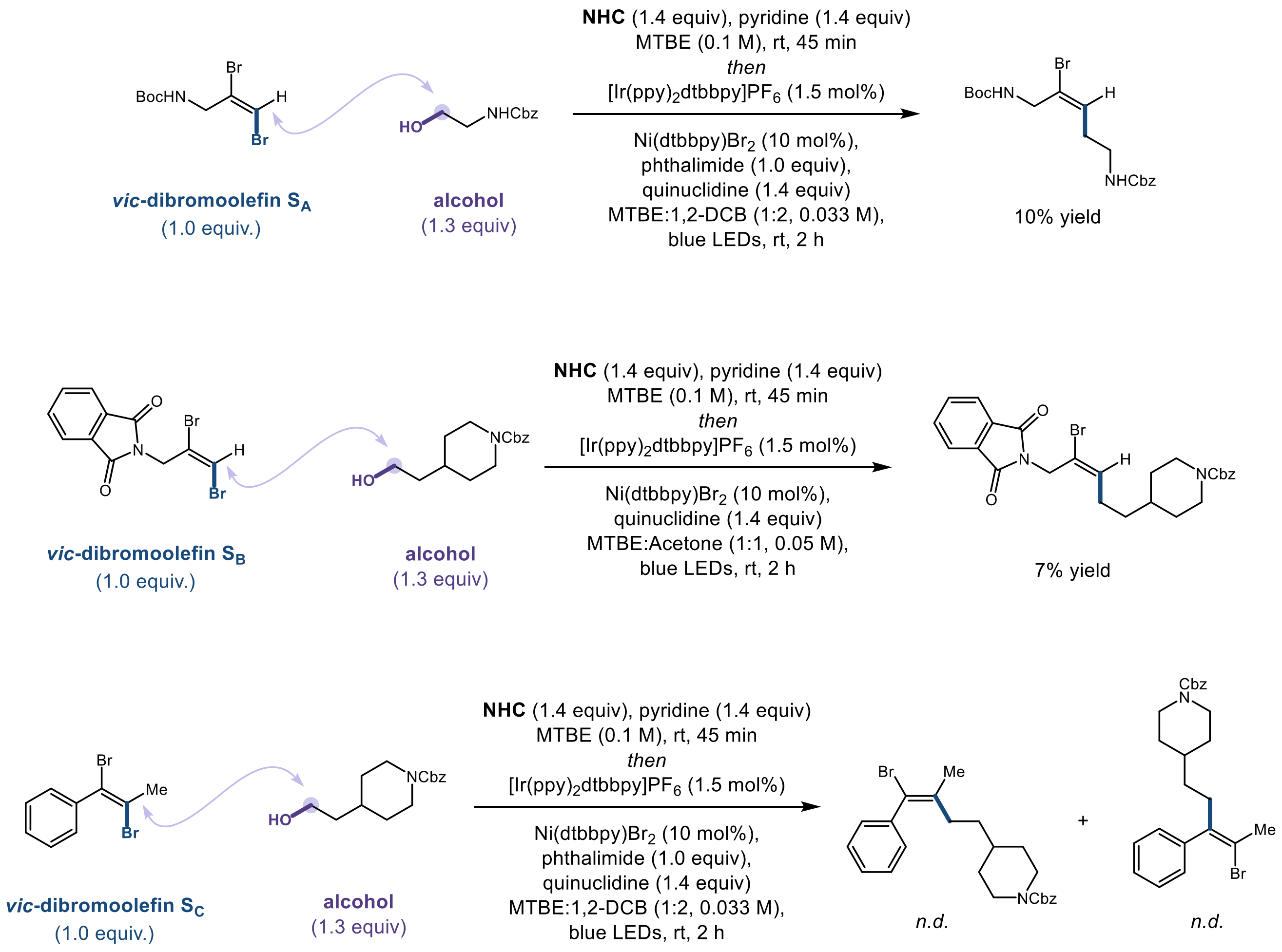

Excitingly, without additional optimization of reaction conditions, $\mathbf{S_A}$ can undergo deoxygenative bromoalkenylation in 10% yield. Additionally, using conditions similar to those of the deoxygenative alkenylation (without phthalimide, using acetone instead of 1,2-DCB), $\mathbf{S_B}$ can undergo deoxygenative bromoalkenylation in 7% yield. We believe that these yields could certainly be improved with further optimization of the reaction conditions. Unfortunately, internal *vic*-dibromoolefin $\mathbf{S_C}$ was not a viable substrate with our reaction conditions. We propose this may be due to steric hindrance inhibiting oxidative addition to either C–Br bond. However, further optimization may ultimately enable bromoalkenylation with internal *vic*-dibromoolefins.

5) Experimental Data for Deoxygenative Alkenylation Products

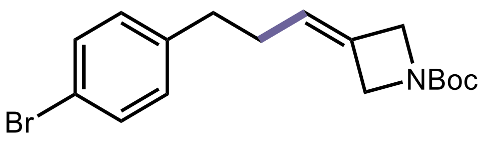

tert-butyl 3-(3-(4-bromophenyl)propylidene)azetidine-1-carboxylate (1)

The title compound was prepared according to **General Procedure A** using **NHC** (375.5 mg, 0.95 mmol, 1.9 equiv.), 2-(4-bromophenyl)ethan-1-ol (175.9 mg, 0.87 mmol, 1.75 equiv.), pyridine (76.8 μL, 0.95 mmol, 1.9 equiv.), and *t*-BuOMe (5 mL) for **Stock A** and Ir[(ppy)$_2$(dtbbpy)]PF$_6$ (6.9 mg, 7.5 μmol, 0.015 equiv.), NiBr$_2$(dtbbpy) (12.2 mg, 25.0 μmol, 0.05 equiv.), quinuclidine (105.6 mg, 0.95 mmol, 1.9 equiv.), tert-butyl 3-(bromomethylene)azetidine-1-carboxylate (124.1 mg, 0.50 mmol, 1.0 equiv.), and acetone (5 mL) for **Stock B**. Due to challenges in purification, the title compound was isolated only in analytical quantities for characterization. The pure material, obtained as a viscous brown oil, was subsequently used to determine the assay yield of the crude reaction mixture by ^{1}H NMR analysis with mesitylene as an internal standard, which indicated a yield of 87%.

^{1}H NMR (500 MHz, CDCl$_3$) δ 7.40 (d, *J* = 8.4 Hz, 2H), 7.03 (d, *J* = 8.3 Hz, 2H), 5.28 (tt, *J* = 7.3, 2.3 Hz, 1H), 4.40 (s, 2H), 4.34 (s, 2H), 2.62 (t, *J* = 7.5 Hz, 2H), 2.18 (q, *J* = 7.5 Hz, 2H), 1.44 (s, 9H).

^{13}C NMR (126 MHz, CDCl$_3$) δ 156.53, 140.49, 131.56, 130.31, 129.07, 121.17, 119.90, 79.70, 57.61, 56.80, 34.92, 30.35, 28.53.

IR (film) ν_{max} 2975.29, 2929.81, 2861.82, 1696.31, 1487.67, 1451.89, 1388.54, 1364.69, 1276.35, 1252.05, 1179.38, 1150.05, 1116.59, 1071.51, 1010.62, 940.06, 860.57, 839.22, 813.91, 792.79, 771.54, 629.87, 597.92, 558.71 cm^{-1}.

HRMS (ESI-TOF) *m/z* calcd. for $C_{12}H_{15}BrN^+$ ($[M-C_5H_9O_2+H]^+$) 252.0382, found 252.0381.

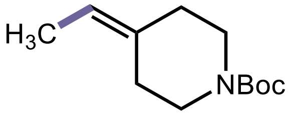

***tert*-butyl 4-ethylidenepiperidine-1-carboxylate (2)**

The title compound was prepared according to **General Procedure A** using **NHC** (375.5 mg, 0.95 mmol, 1.9 equiv.), methanol (35.2 µL, 0.87 mmol, 1.75 equiv.), pyridine (76.8 µL, 0.95 mmol, 1.9 equiv.), and *t*-BuOMe (5 mL) for **Stock A** and Ir[(ppy)$_2$(dtbbpy)]PF$_6$ (6.9 mg, 7.5 µmol, 0.015 equiv.), NiBr$_2$(dtbbpy) (18.3 mg, 37.5 µmol, 0.075 equiv.), quinuclidine (105.6 mg, 0.95 mmol, 1.9 equiv.), *tert*-butyl 4-(bromomethylene)piperidine-1-carboxylate (124.1 mg, 0.50 mmol, 1.0 equiv.), phthalimide (16.6 mg, 0.113 mmol, 0.225 equiv.), and acetone (5 mL) for **Stock B**. Due to decomposition of the product and coelution with *tert*-butyl 4-(bromomethylene)piperidine-1-carboxylate, the yield was determined by ^{1}H NMR analysis of the crude reaction mixture using mesitylene as an internal standard, which indicated a yield of 45%. The ^{1}H NMR shifts of the crude ^{1}H NMR matched literature reports[3] and a commercial standard.

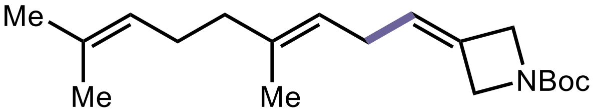

***tert*-butyl (*E*)-3-(4,8-dimethylnona-3,7-dien-1-ylidene)azetidine-1-carboxylate (3)**

The title compound was prepared according to **General Procedure A** using **NHC** (375.5 mg, 0.95 mmol, 1.9 equiv.), geraniol (135.0 mg, 0.87 mmol, 1.75 equiv.), pyridine (76.8 µL, 0.95 mmol, 1.9 equiv.), and *t*-BuOMe (5 mL) for **Stock A** and Ir[(ppy)$_2$(dtbbpy)]PF$_6$ (6.9 mg, 7.5 µmol, 0.015 equiv.), NiBr$_2$(dtbbpy) (12.2 mg, 25.0 µmol, 0.05 equiv.), quinuclidine (105.6 mg, 0.95 mmol, 1.9 equiv.), tert-butyl 3-(bromomethylene)azetidine-1-carboxylate (124.1 mg, 0.50 mmol, 1.0 equiv.), and acetone (5 mL) for **Stock B.** The crude mixture was concentrated via *Genevac* then purified by automated flash chromatography (25 g high performance silica column, 0-25% ethyl acetate/hexanes gradient) to yield impure product. This was further purified by preparative HPLC (30-70% MeCN in water with a 0.1% NH$_4$OH modifier) to provide the desired compound as a viscous yellow oil (124.7 mg, 0.408 mmol, 82% yield).

^{1}H NMR (500 MHz, $CDCl_3$) δ 5.27 (tt, *J* = 7.0, 2.3 Hz, 1H), 5.09 (q, *J* = 1.4 Hz, 2H), 4.46 (s, 2H), 4.42 (s, 2H), 2.67 – 2.57 (m, 2H), 2.13 – 2.02 (m, 2H), 2.02 – 1.93 (m, 2H), 1.68 (s, 3H), 1.63 – 1.58 (m, 6H), 1.45 (s, 9H).

^{13}C NMR (126 MHz, $CDCl_3$) δ 156.57, 136.66, 131.67, 127.71, 124.30, 121.30, 121.10, 79.62, 57.36 (br), 39.77, 28.55, 27.70, 26.72, 25.84, 17.84, 16.17.

IR (film) ν_{max} 2973.50, 2928.73, 1702.47, 1477.98, 1390.45, 1365.75, 1250.00, 1152.32, 969.05, 941.09, 858.62, 771.65, 562.52 cm^{-1}.

HRMS (ESI-TOF) *m/z* calcd. for $C_{19}H_{32}NO_2^+$ ($[M+H]^+$) 306.2428, found 306.2430.

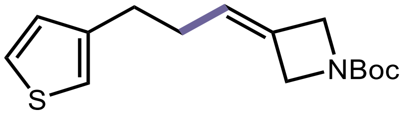

***tert*-butyl 3-(3-(thiophen-3-yl)propylidene)azetidine-1-carboxylate (4)**

The title compound was prepared according to **General Procedure A** using **NHC** (375.5 mg, 0.95 mmol, 1.9 equiv.), 2-(thiophen-3-yl)ethan-1-ol (112.2 mg, 0.87 mmol, 1.75 equiv.), pyridine (76.8 µL, 0.95 mmol, 1.9 equiv.), and *t*-BuOMe (5 mL) for **Stock A** and Ir[(ppy)$_2$(dtbbpy)]PF_6 (6.9 mg, 7.5 µmol, 0.015 equiv.), $NiBr_2$(dtbbpy) (12.2 mg, 25.0 µmol, 0.05 equiv.), quinuclidine (105.6 mg, 0.95 mmol, 1.9 equiv.), *tert*-butyl 3-(bromomethylene)azetidine-1-carboxylate (124.1 mg, 0.50 mmol, 1.0 equiv.), and acetone (5 mL) for **Stock B**. The crude mixture was concentrated via *Genevac* then purified by automated flash chromatography (25 g high performance silica column, 0-25% ethyl acetate/hexanes gradient) to yield impure product. This was further purified by preparative HPLC (30-70% MeCN in water with a 0.1% NH_4OH modifier) to provide the desired compound as a viscous yellow oil (115.3 mg, 0.413 mmol, 83% yield).

^{1}H NMR (500 MHz, $CDCl_3$) δ 7.25 (dd, *J* = 4.9, 3.0 Hz, 1H), 6.96 – 6.90 (m, 2H), 5.32 (tp, *J* = 7.2, 2.3 Hz, 1H), 4.41 (s, 1H), 4.37 (s, 1H), 2.69 (t, *J* = 7.5 Hz, 2H), 2.21 (q, *J* = 7.6 Hz, 2H), 1.44 (s, 9H).

^{13}C NMR (126 MHz, CDCl$_3$) δ 156.55, 141.93, 128.75, 128.22, 125.54, 121.62, 120.47, 79.65, 57.65, 56.82, 29.85, 29.72, 28.53.

IR (film) ν_{max} 3339.76, 3099.45, 2975.62, 2930.22, 2863.33, 1694.72, 1501.07, 1477.73, 1390.69, 1365.52, 1247.94, 1151.59, 1080.13, 939.05, 856.54, 834.99, 770.93, 681.77, 634.47, 577.28 cm^{-1}.

HRMS (ESI-TOF) *m/z* calcd. for $C_{11}H_{14}NO_2S^+$ ($[M\text{-}C_4H_9\text{+}H]^+$) 224.0740, found 224.0740.

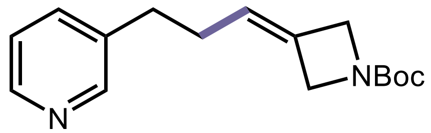

***tert*-butyl 3-(3-(pyridin-3-yl)propylidene)azetidine-1-carboxylate (5)**

The title compound was prepared according to **General Procedure A** using **NHC** (375.5 mg, 0.95 mmol, 1.9 equiv.), 2-(pyridin-3-yl)ethan-1-ol (107.8 mg, 0.87 mmol, 1.75 equiv.), pyridine (76.8 µL, 0.95 mmol, 1.9 equiv.), and *t*-BuOMe (5 mL) for **Stock A** and Ir[(ppy)$_2$(dtbbpy)]PF$_6$ (6.9 mg, 7.5 µmol, 0.015 equiv.), NiBr$_2$(dtbbpy) (12.2 mg, 25.0 µmol, 0.05 equiv.), quinuclidine (105.6 mg, 0.95 mmol, 1.9 equiv.), *tert*-butyl 3-(bromomethylene)azetidine-1-carboxylate (124.1 mg, 0.50 mmol, 1.0 equiv.), and acetone (5 mL) for **Stock B**. The crude mixture was concentrated via *Genevac* then purified by automated flash chromatography (25 g high performance silica column, 0-25% ethyl acetate/hexanes gradient) to yield impure product. This was further purified by preparative HPLC (30-70% MeCN in water with a 0.1% NH_4OH modifier) to provide the desired compound as a viscous brown oil (88.2 mg, 0.322 mmol, 64% yield).

^{1}H NMR (500 MHz, CDCl$_3$) δ 8.50 (s, 2H), 7.48 (d, *J* = 7.8 Hz, 1H), 7.25 (s, 1H), 5.33 – 5.26 (m, 1H), 4.40 (s, 2H), 4.32 (s, 2H), 2.68 (t, *J* = 7.5 Hz, 2H), 2.22 (q, *J* = 7.5 Hz, 2H), 1.43 (s, 9H).

^{13}C NMR (126 MHz, CDCl$_3$) δ 156.48, 149.93, 147.58, 137.06 (br), 135.90, 129.52, 123.82 (br), 120.79, 79.72, 57.17 (br), 32.66, 30.20, 28.51.

IR (film) ν_{max} 2975.74, 2930.77, 2863.61, 1791.46, 1695.23, 1575.49, 1478.28, 1453.14, 1390.28, 1365.35, 1331.46, 1251.49, 1151.39, 1118.75, 1075.75, 1027.16, 940.02, 859.32, 771.04, 713.04, 628.98, 556.85, 463.22, 424.52 cm^{-1}.

HRMS (ESI-TOF) *m/z* calcd. for $C_{16}H_{22}N_2O_2Na^+$ ($[M+Na]^+$) 297.1573, found 297.1575.

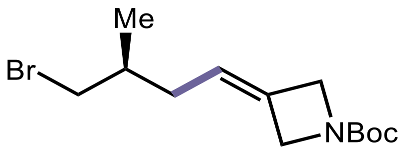

***tert*-butyl (*S*)-3-(4-bromo-3-methylbutylidene)azetidine-1-carboxylate (6)**

The title compound was prepared according to **General Procedure A** using **NHC** (375.5 mg, 0.95 mmol, 1.9 equiv.), (*S*)-3-bromo-2-methylpropan-1-ol (133.9 mg, 0.87 mmol, 1.75 equiv.), pyridine (76.8 µL, 0.95 mmol, 1.9 equiv.), and *t*-BuOMe (5 mL) for **Stock A** and Ir[(ppy)$_2$(dtbbpy)]PF$_6$ (6.9 mg, 7.5 µmol, 0.015 equiv.), NiBr$_2$(dtbbpy) (12.2 mg, 25.0 µmol, 0.05 equiv.), quinuclidine (105.6 mg, 0.95 mmol, 1.9 equiv.), *tert*-butyl 3-(bromomethylene)azetidine-1-carboxylate (124.1 mg, 0.50 mmol, 1.0 equiv.), and acetone (5 mL) for **Stock B**. The crude mixture was concentrated via *Genevac* then purified by automated flash chromatography (25 g high performance silica column, 0-25% ethyl acetate/hexanes gradient) to yield impure product. This was further purified by preparative HPLC (30-70% MeCN in water with a 0.1% NH_4OH modifier) to provide the desired compound as a transparent oil (94.1 mg, 0.310 mmol, 62% yield).

^{1}H NMR (500 MHz, CDCl$_3$) δ 5.30 – 5.22 (m, 1H), 4.54 – 4.39 (m, 4H), 3.34 (dd, *J* = 5.2, 1.8 Hz, 2H), 2.09 – 1.99 (m, 1H), 1.94 – 1.82 (m, 2H), 1.45 (s, 9H), 1.02 (d, *J* = 6.4 Hz, 3H).

^{13}C NMR (126 MHz, CDCl$_3$) δ 156.51, 130.27, 119.65, 79.72, 57.60, 57.04, 40.45, 35.38, 33.80, 28.53, 18.79.

IR (film) ν_{max} 2973.58, 2931.42, 2870.37, 1789.99, 1695.63, 1501.27, 1478.18, 1455.33, 1391.16, 1365.69, 1333.35, 1247.08, 1152.79, 940.15, 857.27, 772.03, 650.00, 619.83, 563.61 cm^{-1}.

HRMS (ESI-TOF) *m/z* calcd. for $C_8H_{15}BrN^+$ ($[M-C_5H_9O_2+H]^+$) 204.0382, found 204.0381.

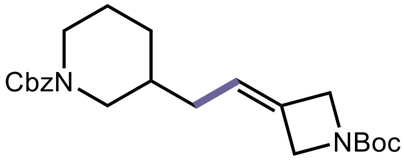

(±)-benzyl 3-(2-(1-(*tert*-butoxycarbonyl)azetidin-3-ylidene)ethyl)piperidine-1-carboxylate (7)

The title compound was prepared according to **General Procedure A** using **NHC** (375.5 mg, 0.95 mmol, 1.9 equiv.), (±)-benzyl 3-(hydroxymethyl)piperidine-1-carboxylate (218.1 mg, 0.87 mmol, 1.75 equiv.), pyridine (76.8 µL, 0.95 mmol, 1.9 equiv.), and *t*-BuOMe (5 mL) for **Stock A** and Ir[(ppy)$_2$(dtbbpy)]PF$_6$ (6.9 mg, 7.5 µmol, 0.015 equiv.), NiBr$_2$(dtbbpy) (12.2 mg, 25.0 µmol, 0.05 equiv.), quinuclidine (105.6 mg, 0.95 mmol, 1.9 equiv.), *tert*-butyl 3-(bromomethylene)azetidine-1-carboxylate (124.1 mg, 0.50 mmol, 1.0 equiv.), and acetone (5 mL) for **Stock B**. The crude mixture was concentrated via *Genevac* then purified by automated flash chromatography (25 g high performance silica column, 0-25% ethyl acetate/hexanes gradient) to yield impure product. This was further purified by preparative HPLC (30-70% MeCN in water with a 0.1% NH_4OH modifier) to provide the desired compound as a light brown solid (192.4 mg, 0.481 mmol, 96% yield).

^{1}H NMR (500 MHz, CDCl$_3$) δ 7.40 – 7.30 (m, 5H), 5.28 (t, *J* = 7.7 Hz, 1H), 5.12 (s, 2H), 4.42 (s, 4H), 4.05 – 3.95 (m, 2H), 2.86 – 2.77 (m, 1H), 2.50 (s, 1H), 1.88 – 1.73 (m, 3H), 1.69 – 1.61 (m, 2H), 1.58 – 1.49 (m, 1H), 1.45 (s, 9H), 1.15 – 1.03 (m, 1H).

^{13}C NMR (126 MHz, CDCl$_3$) δ 156.37, 155.30, 136.97, 129.40, 128.49, 127.95, 127.80, 119.81, 79.58, 66.99, 57.48, 56.78, 49.53, 44.59, 36.05, 32.68 (br), 30.60, 28.41, 24.90 (br).

IR (film) ν_{max} 2974.15, 2930.92, 2859.63, 1692.57, 1497.75, 1468.36, 1428.41, 1389.95, 1364.68, 1256.94, 1235.05, 1180.51, 1149.14, 1118.46, 1028.16, 962.56, 939.05, 918.69, 856.15, 824.69, 764.52, 731.15, 696.69, 646.68, 605.32, 561.66 cm^{-1}.

HRMS (ESI-TOF) *m/z* calcd. for $C_{18}H_{25}N_2O_2^+$ ([M-$C_5H_9O_2$+H]$^+$) 301.1911, found 301.1915.

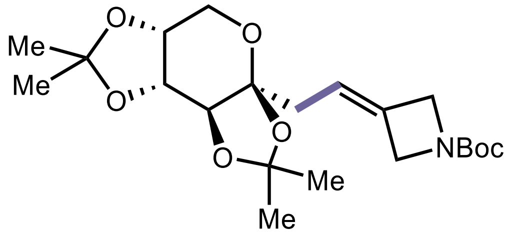

***tert*-butyl 3-(2-((3a*S*,5a*R*,8a*R*,8b*S*)-2,2,7,7-tetramethyltetrahydro-3a*H*-bis([1,3]dioxolo)[4,5-*b*:4',5'-*d*]pyran-3a-yl)ethylidene)azetidine-1-carboxylate (8)**

The title compound was prepared according to **General Procedure A** using **NHC** (375.5 mg, 0.95 mmol, 1.9 equiv.), diacetonefructose (227.8 mg, 0.87 mmol, 1.75 equiv.), pyridine (76.8 µL, 0.95 mmol, 1.9 equiv.), and *t*-BuOMe (5 mL) for **Stock A** and Ir[(ppy)$_2$(dtbbpy)]PF$_6$ (6.9 mg, 7.5 µmol, 0.015 equiv.), NiBr$_2$(dtbbpy) (12.2 mg, 25.0 µmol, 0.05 equiv.), quinuclidine (105.6 mg, 0.95 mmol, 1.9 equiv.), *tert*-butyl 3-(bromomethylene)azetidine-1-carboxylate (124.1 mg, 0.50 mmol, 1.0 equiv.), and acetone (5 mL) for **Stock B**. The crude mixture was concentrated via *Genevac* then purified by automated flash chromatography (25 g high performance silica column, 0-25% ethyl acetate/hexanes gradient) to yield impure product. This was further purified by preparative HPLC (30-70% MeCN in water with a 0.1% NH$_4$OH modifier) to provide the desired compound as a viscous light brown oil (182.2 mg, 0.443 mmol, 89% yield).

^{1}H NMR (500 MHz, CDCl$_3$) δ 5.50 (tp, *J* = 7.0, 2.3 Hz, 1H), 4.57 (dd, *J* = 8.0, 2.5 Hz, 1H), 4.53 – 4.44 (m, 4H), 4.24 – 4.19 (m, 1H), 4.09 (d, *J* = 2.5 Hz, 1H), 3.86 (dd, *J* = 13.0, 1.9 Hz, 1H), 3.73 (d, *J* = 13.1 Hz, 1H), 2.55 – 2.47 (m, 1H), 2.28 (dd, *J* = 14.2, 8.6 Hz, 1H), 1.52 (s, 3H), 1.48 (s, 3H), 1.45 (s, 9H), 1.34 (s, 6H).

^{13}C NMR (126 MHz, CDCl$_3$) δ 156.58, 131.92, 116.54, 109.10, 108.03, 103.59, 79.70, 72.84, 70.94, 70.51, 61.37, 57.80, 56.94, 39.19, 28.53, 26.65, 25.99, 25.34, 24.17.

IR (film) ν_{max} 2978.71, 2933.74, 2866.41, 1700.99, 1479.05, 1455.06, 1381.00, 1366.18, 1315.67, 1249.45, 1209.58, 1152.65, 1114.69, 1063.45, 1040.13, 1014.28, 991.75, 939.72, 898.21, 862.75, 758.13, 701.01, 633.78, 576.88, 538.98, 522.42, 507.89, 413.24 cm^{-1}.

HRMS (ESI-TOF) *m/z* calcd. for $C_{21}H_{33}NO_7Na^+$ ([M+Na]$^+$) 434.2149, found 434.2150.

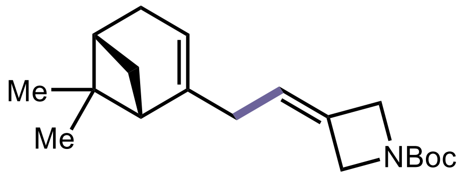

***tert*-butyl 3-(2-((1*S*,5*R*)-6,6-dimethylbicyclo[3.1.1]hept-2-en-2-yl)ethylidene)azetidine-1-carboxylate (9)**

The title compound was prepared according to **General Procedure A** using **NHC** (375.5 mg, 0.95 mmol, 1.9 equiv.), (-)-myrtenol (133.2 mg, 0.87 mmol, 1.75 equiv.), pyridine (76.8 µL, 0.95 mmol, 1.9 equiv.), and *t*-BuOMe (5 mL) for **Stock A** and Ir[(ppy)$_2$(dtbbpy)]PF$_6$ (6.9 mg, 7.5 µmol, 0.015 equiv.), NiBr$_2$(dtbbpy) (12.2 mg, 25.0 µmol, 0.05 equiv.), quinuclidine (105.6 mg, 0.95 mmol, 1.9 equiv.), *tert*-butyl 3-(bromomethylene)azetidine-1-carboxylate (124.1 mg, 0.50 mmol, 1.0 equiv.), and acetone (5 mL) for **Stock B**. The crude mixture was concentrated via *Genevac* then purified by automated flash chromatography (25 g high performance silica column, 0-25% ethyl acetate/hexanes gradient) to yield impure product. This was further purified by preparative HPLC (30-70% MeCN in water with a 0.1% NH_4OH modifier) to provide the desired compound as a viscous yellow oil (113.7 mg, 0.375 mmol, 75% yield).

^{1}H NMR (500 MHz, CDCl$_3$) δ 5.27 (tt, *J* = 7.6, 2.3 Hz, 1H), 5.20 (dp, *J* = 3.0, 1.5 Hz, 1H), 4.44 (s, 4H), 2.55 – 2.49 (m, 2H), 2.34 (dt, *J* = 8.5, 5.6 Hz, 1H), 2.30 – 2.14 (m, 2H), 2.07 (dtd, *J* = 5.9, 2.9, 1.3 Hz, 1H), 1.94 (td, *J* = 5.6, 1.5 Hz, 1H), 1.45 (s, 9H), 1.26 (s, 3H), 1.13 (d, *J* = 8.6 Hz, 1H), 0.80 (s, 3H).

^{13}C NMR (126 MHz, CDCl$_3$) δ 156.56, 146.07, 128.78, 119.85, 116.85, 79.64, 57.31 (br), 45.89, 40.89, 38.17, 35.99, 31.69, 31.37, 28.51, 26.40, 21.17.

IR (film) ν_{max} 2976.18, 2917.41, 1701.77, 1390.63, 1365.30, 1249.39, 1151.18, 940.86, 886.33, 859.21, 771.53, 576.10, 462.71 cm^{-1}.

HRMS (ESI-TOF) *m/z* calcd. for $C_{19}H_{29}NO_2Na^+$ ([M+Na]$^+$) 326.2090, found 326.2088.

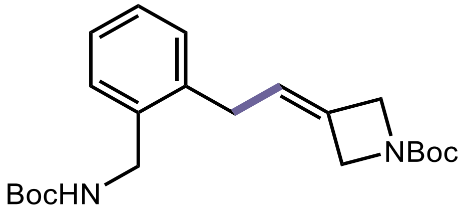

tert-butyl 3-(2-(2-(((_tert_-butoxycarbonyl)amino)methyl)phenyl)ethylidene)azetidine-1-carboxylate (10)

The title compound was prepared according to **General Procedure A** using **NHC** (375.5 mg, 0.95 mmol, 1.9 equiv.), *tert*-butyl (2-(hydroxymethyl)benzyl)carbamate (207.6 mg, 0.87 mmol, 1.75 equiv.), pyridine (76.8 µL, 0.95 mmol, 1.9 equiv.), and *t*-BuOMe (5 mL) for **Stock A** and Ir[(ppy)$_2$(dtbbpy)]PF$_6$ (6.9 mg, 7.5 µmol, 0.015 equiv.), NiBr$_2$(dtbbpy) (12.2 mg, 25.0 µmol, 0.05 equiv.), quinuclidine (105.6 mg, 0.95 mmol, 1.9 equiv.), *tert*-butyl 3-(bromomethylene)azetidine-1-carboxylate (124.1 mg, 0.50 mmol, 1.0 equiv.), and acetone (5 mL) for **Stock B**. The crude mixture was concentrated via *Genevac* then purified by automated flash chromatography (25 g high performance silica column, 0-25% ethyl acetate/hexanes gradient) to yield impure product. This was further purified by preparative HPLC (30-70% MeCN in water with a 0.1% NH_4OH modifier) to provide the desired compound as a viscous yellow oil (176.7 mg, 0.455 mmol, 91% yield).

^{1}H NMR (500 MHz, CDCl$_3$) δ 7.29 – 7.13 (m, 4H), 5.45 (tt, *J* = 7.0, 2.3 Hz, 1H), 4.70 (s, 1H), 4.40 (d, *J* = 45.3 Hz, 4H), 4.32 (d, *J* = 5.6 Hz, 2H), 3.29 (d, *J* = 6.3 Hz, 2H), 1.46 (s, 9H), 1.44 (s, 9H).

^{13}C NMR (126 MHz, CDCl$_3$) δ 156.40, 155.76, 137.94, 136.43, 129.87, 129.57, 128.79, 128.06, 127.07, 120.55, 79.74, 57.78, 56.82, 42.54, 32.06, 28.55, 28.53.

IR (film) ν_{max} 3340.93, 2975.68, 2931.18, 2865.29, 1685.68, 1511.32, 1452.88, 1389.78, 1364.50, 1246.89, 1154.28, 1120.51, 1047.24, 939.76, 859.93, 753.82, 611.38, 561.07, 521.70, 462.05, 406.47 cm^{-1}.

HRMS (ESI-TOF) *m/z* calcd. for $C_{22}H_{32}N_2O_4Na^+$ ([M+Na]$^+$) 411.2254, found 411.2256.

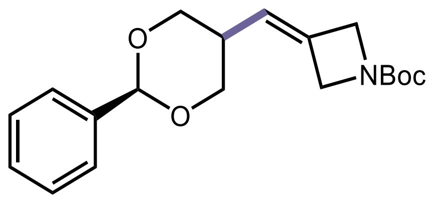

(±)-*tert*-butyl 3-(((2*r*,5*r*)-2-phenyl-1,3-dioxan-5-yl)methylene)azetidine-1-carboxylate (11)

The title compound was prepared according to **General Procedure A** using **NHC** (375.5 mg, 0.95 mmol, 1.9 equiv.), 2-phenyl-1,3-dioxan-5-ol (157.7 mg, 0.87 mmol, 1.75 equiv.), pyridine (76.8 µL, 0.95 mmol, 1.9 equiv.), and *t*-BuOMe (5 mL) for **Stock A** and Ir[(ppy)$_2$(dtbbpy)]PF$_6$ (6.9 mg, 7.5 µmol, 0.015 equiv.), NiBr$_2$(dtbbpy) (12.2 mg, 25.0 µmol, 0.05 equiv.), quinuclidine (105.6 mg, 0.95 mmol, 1.9 equiv.), *tert*-butyl 3-(bromomethylene)azetidine-1-carboxylate (124.1 mg, 0.50 mmol, 1.0 equiv.), and acetone (5 mL) for **Stock B.** The crude mixture was concentrated via *Genevac* then purified by automated flash chromatography (25 g high performance silica column, 0-25% ethyl acetate/hexanes gradient) to yield impure product. This was further purified by preparative HPLC (30-70% MeCN in water with a 0.1% NH$_4$OH modifier) to provide the desired compound as a viscous clear oil (98.9 mg, 0.299 mmol, 60% yield). The desired compound was isolated as an inseparable, unassigned 66:34 mixture of diastereomers (ratio determined by integration of the crude ^{1}H NMR). Spectral data is provided for the mixture of diastereomers.

^{1}H NMR (500 MHz, CDCl$_3$) δ 7.50 – 7.47 (m, 2H), 7.39 – 7.35 (m, 3H), 5.99 (dt, *J* = 9.1, 2.3 Hz, 1H), 5.55 (s, 1H), 4.50 (d, *J* = 2.3 Hz, 4H), 4.19 – 4.15 (m, 2H), 4.08 – 4.03 (m, 2H), 2.11 (d, *J* = 9.0 Hz, 1H), 1.45 (s, 9H).

^{13}C NMR (126 MHz, CDCl$_3$) δ 156.42, 138.29, 129.36, 128.98, 128.34, 126.02, 122.21, 101.86, 79.69, 70.98, 56.36, 56.36 (br, 2C), 34.79, 28.40.

IR (film) ν_{max} 2973.57, 2930.95, 2860.62, 1696.11, 1477.99, 1454.42, 1390.33, 1374.87, 1365.51, 1277.50, 1231.81, 1150.12, 1107.06, 1027.92, 1003.85, 974.58, 953.76, 940.54, 860.10, 824.42, 746.46, 697.22, 675.03, 647.41, 588.14, 540.83, 463.07, 427.29 cm^{-1}.

HRMS (ESI-TOF) *m/z* calcd. for $C_{19}H_{25}NO_4Na^+$ ([M+Na]$^+$) 354.1676, found 354.1676.

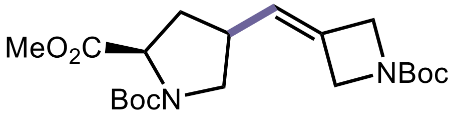

1-(*tert*-butyl) 2-methyl (2*R*,4*R*)-4-((1-(*tert*-butoxycarbonyl)azetidin-3-ylidene)methyl)pyrrolidine-1,2-dicarboxylate (12)

The title compound was prepared according to **General Procedure A** using **NHC** (375.5 mg, 0.95 mmol, 1.9 equiv.), 1-(*tert*-butyl) 2-methyl (2*R*,4*S*)-4-hydroxypyrrolidine-1,2-dicarboxylate (214.6 mg, 0.87 mmol, 1.75 equiv.), pyridine (76.8 µL, 0.95 mmol, 1.9 equiv.), and *t*-BuOMe (5 mL) for **Stock A** and Ir[(ppy)$_2$(dtbbpy)]PF$_6$ (6.9 mg, 7.5 µmol, 0.015 equiv.), NiBr$_2$(dtbbpy) (12.2 mg, 25.0 µmol, 0.05 equiv.), quinuclidine (105.6 mg, 0.95 mmol, 1.9 equiv.), *tert*-butyl 3-(bromomethylene)azetidine-1-carboxylate (124.1 mg, 0.50 mmol, 1.0 equiv.), and acetone (5 mL) for **Stock B**. The crude mixture was concentrated via *Genevac* then purified by automated flash chromatography (25 g high performance silica column, 0-25% ethyl acetate/hexanes gradient) to yield impure product. This was further purified by preparative HPLC (30-70% MeCN in water with a 0.1% NH_4OH modifier) to provide the desired compound as a viscous brown oil (164.2 mg, 0.414 mmol, 83% yield). The desired compound was isolated as an inseparable, unassigned 58:42 mixture of diastereomers (ratio determined by integration of the crude ^{1}H NMR). Spectral data is provided for the mixture of diastereomers.

^{1}H NMR (500 MHz, $CDCl_3$) δ 5.18 (s, 1H), 4.50 – 4.40 (m, 4H), 4.39 – 4.24 (m, 1H), 3.73 (s, 4H), 3.16 – 2.78 (m, 2H), 2.13 – 1.86 (m, 2H), 1.46 – 1.39 (m, 18H).

^{13}C NMR (126 MHz, $CDCl_3$) δ 173.49, 173.28, 156.45, 154.30, 153.66, 130.93, 130.85, 121.71, 121.68, 80.30, 79.90, 79.88, 58.92, 58.66, 57.68 (br), 56.95 (br), 52.42, 52.24, 51.38, 51.06, 37.26, 36.76, 36.38, 35.91, 28.54, 28.51, 28.41.

IR (film) ν_{max} 2975.21, 2932.58, 2869.14, 1747.78, 1694.39, 1478.61, 1453.71, 1388.63, 1364.01, 1254.39, 1198.89, 1154.84, 1114.40, 1033.01, 985.14, 940.88, 893.43, 860.24, 823.63, 770.67, 560.26 cm^{-1}.

HRMS (ESI-TOF) *m/z* calcd. for $C_{15}H_{25}N_2O_4^+$ ($[M-C_5H_9O_2+H]^+$) 297.1809, found 297.1810.

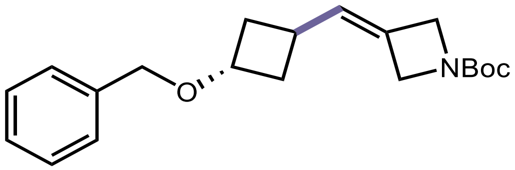

(±)-*tert*-butyl 3-(((1*r*,3*r*)-3-(benzyloxy)cyclobutyl)methylene)azetidine-1-carboxylate (13)

The title compound was prepared according to **General Procedure A** using **NHC** (375.5 mg, 0.95 mmol, 1.9 equiv.), **(±)**-3-(benzyloxy)cyclobutan-1-ol (156.0 mg, 0.87 mmol, 1.75 equiv.), pyridine (76.8 µL, 0.95 mmol, 1.9 equiv.), and *t*-BuOMe (5 mL) for **Stock A** and Ir[(ppy)$_2$(dtbbpy)]PF$_6$ (6.9 mg, 7.5 µmol, 0.015 equiv.), NiBr$_2$(dtbbpy) (18.3 mg, 37.5 µmol, 0.075 equiv.), quinuclidine (105.6 mg, 0.95 mmol, 1.9 equiv.), *tert*-butyl 3-(bromomethylene)azetidine-1-carboxylate (124.1 mg, 0.50 mmol, 1.0 equiv.), phthalimide (16.6 mg, 0.113 mmol, 0.225 equiv.), and acetone (5 mL) for **Stock B**. The crude mixture was concentrated via *Genevac* then purified by automated flash chromatography (25 g high performance silica column, 0-25% ethyl acetate/hexanes gradient) to yield impure product. This was further purified by preparative HPLC (30-70% MeCN in water with a 0.1% NH_4OH modifier) to provide the desired compound as a viscous light yellow oil (121.7 mg, 0.370 mmol, 74% yield). The desired compound was isolated as an inseparable, unassigned 58:42 mixture of diastereomers (ratio determined by integration of the crude ^{1}H NMR). Spectral data is provided for the mixture of diastereomers.

^{1}H NMR (500 MHz, $CDCl_3$) δ 7.37 – 7.26 (m, 5H), 5.51 – 5.26 (m, 1H), 4.46 – 3.86 (m, 7H), 2.51 – 1.58 (m, 5H), 1.44 (s, 9H).

^{13}C NMR (126 MHz, $CDCl_3$) δ 156.55, 156.10, 138.36, 138.32, 128.55, 128.53, 128.00, 127.96, 127.80, 127.78, 127.36, 127.25, 126.92, 126.33, 79.68, 71.87, 70.29, 70.27, 69.55, 57.37 (br), 37.20, 35.96, 28.53, 27.93, 25.57.

IR (film) ν_{max} 2974.45, 2932.03, 2863.75, 1698.46, 1495.88, 1478.25, 1453.22, 1390.15, 1364.95, 1275.02, 1251.73, 1151.43, 1110.23, 1027.59, 940.32, 860.29, 821.57, 771.93, 735.36, 697.47, 610.51, 562.22, 457.81, 424.77, 412.07 cm^{-1}.

HRMS (ESI-TOF) *m/z* calcd. for $C_{20}H_{27}NO_3Na^+$ ($[M+Na]^+$) 352.1883, found 352.1879.

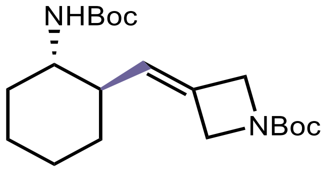

(±)-*tert*-butyl 3-(((1*R*,2*S*)-2-((*tert*-butoxycarbonyl)amino)cyclohexyl)methylene)azetidine-1-carboxylate (14)

The title compound was prepared according to **General Procedure A** using **NHC** (375.5 mg, 0.95 mmol, 1.9 equiv.), **(±)-***tert*-butyl ((1*S*,2*S*)-2-hydroxycyclohexyl)carbamate (188.4 mg, 0.87 mmol, 1.75 equiv.), pyridine (76.8 µL, 0.95 mmol, 1.9 equiv.), and *t*-BuOMe (5 mL) for **Stock A** and $Ir[(ppy)_2(dtbbpy)]PF_6$ (6.9 mg, 7.5 µmol, 0.015 equiv.), $NiBr_2(dtbbpy)$ (18.3 mg, 37.5 µmol, 0.075 equiv.), quinuclidine (105.6 mg, 0.95 mmol, 1.9 equiv.), *tert*-butyl 3-(bromomethylene)azetidine-1-carboxylate (124.1 mg, 0.50 mmol, 1.0 equiv.), phthalimide (16.6 mg, 0.113 mmol, 0.225 equiv.), and acetone (5 mL) for **Stock B**. The crude mixture was concentrated via *Genevac* then purified by automated flash chromatography (25 g high performance silica column, 0-25% ethyl acetate/hexanes gradient) to yield impure product. This was further purified by preparative HPLC (30-70% MeCN in water with a 0.1% NH_4OH modifier) to provide the desired compound as a white solid (129.7 mg, 0.354 mmol, 71% yield). The desired compound was isolated as an inseparable, 83:17 mixture of diastereomers (ratio determined by integration of the crude ^1H NMR and configuration determined by allylic NOE correlations). Spectral data is provided for the mixture of diastereomers.

^{1}H NMR (500 MHz, $CDCl_3$) δ 1H NMR (500 MHz, CDCl3) δ 5.20 (dt, *J* = 9.4, 2.3 Hz, 1H), 4.52 – 4.35 (m, 4H), 4.34 – 4.12 (m, 1H), 3.23 (d, *J* = 10.8 Hz, 1H), 2.04 – 1.95 (m, 1H), 1.75 – 1.63 (m, 4H), 1.43 (d, *J* = 9.5 Hz, 18H), 1.33 – 1.03 (m, 4H).

^{13}C NMR (126 MHz, $CDCl_3$) δ 156.53, 155.57, 128.29, 125.53, 79.65, 79.24, 57.39 (br, 2C), 53.52, 45.72, 33.52, 32.09, 28.53, 28.52, 25.41, 25.28.

IR (film) ν_{max} 3320.71, 2977.15, 2930.20, 2857.71, 1705.84, 1680.86, 1525.86, 1478.83, 1449.42, 1407.31, 1388.94, 1364.21, 1312.50, 1279.08, 1252.37, 1168.82, 1146.53, 1128.31, 1040.11, 1010.29, 943.54, 907.35, 859.41, 809.88, 770.54, 715.62, 668.17, 660.94, 646.36, 568.88, 549.01, 534.14, 464.14, 440.78, 424.75 cm^{-1}.

HRMS (ESI-TOF) *m/z* calcd. for $C_{20}H_{34}N_2O_4Na^+$ ($[M+Na]^+$) 389.2411, found 389.2412.

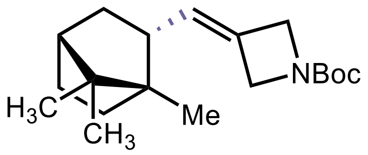

***tert*-butyl 3-(((1*S*,2*R*,4*R*)-1,7,7-trimethylbicyclo[2.2.1]heptan-2-yl)methylene)azetidine-1-carboxylate (15)**

The title compound was prepared according to **General Procedure A** using **NHC** (375.5 mg, 0.95 mmol, 1.9 equiv.), isoborneol (135.0 mg, 0.87 mmol, 1.75 equiv.), pyridine (76.8 µL, 0.95 mmol, 1.9 equiv.), and *t*-BuOMe (5 mL) for **Stock A** and $Ir[(ppy)_2(dtbbpy)]PF_6$ (6.9 mg, 7.5 µmol, 0.015 equiv.), $NiBr_2(dtbbpy)$ (12.2 mg, 25.0 µmol, 0.05 equiv.), quinuclidine (105.6 mg, 0.95 mmol, 1.9 equiv.), *tert*-butyl 3-(bromomethylene)azetidine-1-carboxylate (124.1 mg, 0.50 mmol, 1.0 equiv.), and acetone (5 mL) for **Stock B**. The crude mixture was concentrated via *Genevac* then purified by automated flash chromatography (25 g high performance silica column, 0-25% ethyl acetate/hexanes gradient) to yield impure product. This was further purified by preparative HPLC (30-70% MeCN in water with a 0.1% NH_4OH modifier) to provide the desired compound as an oily yellow solid (70.3 mg, 0.230 mmol, 46% yield). The product was isolated as a single diastereomer (configuration determined by allylic NOE correlations).

^{1}H NMR (500 MHz, $CDCl_3$) δ 5.32 (dt, *J* = 9.5, 2.3 Hz, 1H), 4.52 – 4.35 (m, 4H), 2.22 (t, *J* = 11.4 Hz, 1H), 2.09 – 1.98 (m, 1H), 1.71 (ddt, *J* = 12.3, 8.2, 4.0 Hz, 1H), 1.61 (t, *J* = 4.5 Hz, 1H), 1.51 (td, *J* = 9.3, 4.7 Hz, 1H), 1.45 (s, 9H), 1.32 – 1.24 (m, 1H), 1.15 – 1.08 (m, 1H), 0.92 – 0.87 (m, 4H), 0.86 (s, 3H), 0.74 (s, 3H).

^{13}C NMR (126 MHz, $CDCl_3$) δ 156.59, 128.11, 125.75, 79.59, 57.26 (br), 50.15, 48.57, 45.56, 43.78, 36.30, 29.17, 28.69, 28.56, 19.54, 18.69, 14.67.

IR (film) ν_{max} 2948.31, 2874.67, 1702.15, 1478.78, 1456.53, 1388.63, 1365.21, 1276.37, 1248.88, 1153.52, 1119.50, 941.07, 860.72, 772.55, 564.94 cm^{-1}.

HRMS (ESI-TOF) *m/z* calcd. for $C_{19}H_{31}NO_2Na^+$ ([M+Na]$^+$) 328.2247, found 328.2245.

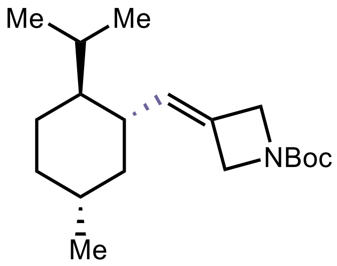

***tert*-butyl 3-(((1*R*,2*S*,5*R*)-2-isopropyl-5-methylcyclohexyl)methylene)azetidine-1-carboxylate (16)**

The title compound was prepared according to **General Procedure A** using **NHC** (375.5 mg, 0.95 mmol, 1.9 equiv.), (-)-menthol (136.7 mg, 0.87 mmol, 1.75 equiv.), pyridine (76.8 µL, 0.95 mmol, 1.9 equiv.), and *t*-BuOMe (5 mL) for **Stock A** and Ir[(ppy)$_2$(dtbbpy)]PF$_6$ (6.9 mg, 7.5 µmol, 0.015 equiv.), NiBr$_2$(dtbbpy) (12.2 mg, 25.0 µmol, 0.05 equiv.), quinuclidine (105.6 mg, 0.95 mmol, 1.9 equiv.), *tert*-butyl 3-(bromomethylene)azetidine-1-carboxylate (124.1 mg, 0.50 mmol, 1.0 equiv.), and acetone (5 mL) for **Stock B.** The crude mixture was concentrated via *Genevac* then purified by automated flash chromatography (25 g high performance silica column, 0-25% ethyl acetate/hexanes gradient) to yield impure product. This was further purified by preparative HPLC (30-70% MeCN in water with a 0.1% NH_4OH modifier) to provide the desired compound as a light brown solid (108.7 mg, 0.354 mmol, 71% yield). The product was isolated as a single diastereomer (configuration determined by vinylic NOE correlations).

^{1}H NMR (500 MHz, CDCl$_3$) δ 5.13 (d, *J* = 9.6 Hz, 1H), 4.43 (dq, *J* = 12.2, 2.0 Hz, 4H), 2.06 (qd, *J* = 9.1, 5.3 Hz, 1H), 1.89 (ddt, *J* = 11.2, 7.7, 3.8 Hz, 1H), 1.78 (pd, *J* = 6.9, 4.1 Hz, 1H), 1.45 (s, 9H), 1.41 – 1.20 (m, 4H), 1.01 – 0.94 (m, 1H), 0.93 (d, *J* = 7.1 Hz, 3H), 0.87 (d, *J* = 6.9 Hz, 3H), 0.75 (d, *J* = 6.9 Hz, 3H).

^{13}C NMR (126 MHz, $CDCl_3$) δ 156.38, 128.00, 126.08, 79.46, 57.10 (br), 47.54, 38.38, 35.04, 31.20, 28.42, 28.21, 27.30, 21.37, 19.06, 18.79, 16.96.

IR (film) ν_{max} 2957.71, 2926.09, 1688.54, 1461.43, 1393.50, 1364.20, 1339.10, 1180.93, 1148.67, 1119.72, 940.27, 861.39 cm^{-1}.

HRMS (ESI-TOF) *m/z* calcd. for $C_{19}H_{33}NO_2Na^+$ ($[M+Na]^+$) 330.2404, found 330.2403.

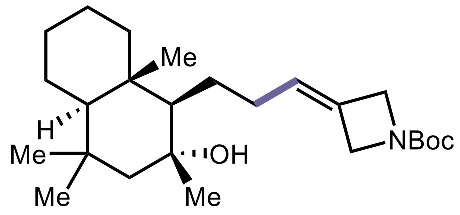

***tert*-butyl 3-(3-((1*R*,2*R*,4a*R*,8a*S*)-2-hydroxy-2,4,4,8a-tetramethyldecahydronaphthalen-1-yl)propylidene)azetidine-1-carboxylate (17)**

The title compound was prepared according to **General Procedure A** using **NHC** (375.5 mg, 0.95 mmol, 1.9 equiv.), (1*R*,2*R*,4a*R*,8a*S*)-1-(2-hydroxyethyl)-2,4,4,8a-tetramethyldecahydro-naphthalen-2-ol (136.7 mg, 0.87 mmol, 1.75 equiv.), pyridine (76.8 µL, 0.95 mmol, 1.9 equiv.), and 1,4-dioxane (5 mL, due to alcohol insolubility in t-BuOMe) for **Stock A**. and Ir[$(ppy)_2$(dtbbpy)]PF_6 (6.9 mg, 7.5 µmol, 0.015 equiv.), $NiBr_2$(dtbbpy) (12.2 mg, 25.0 µmol, 0.05 equiv.), quinuclidine (105.6 mg, 0.95 mmol, 1.9 equiv.), *tert*-butyl 3-(bromomethylene)azetidine-1-carboxylate (124.1 mg, 0.50 mmol, 1.0 equiv.), phthalimide (73.6 mg, 0.50 mmol, 1.0 equiv.), and acetone (5 mL) for **Stock B**. The crude mixture was concentrated via *Genevac* then purified by automated flash chromatography (25 g high performance silica column, 0-25% ethyl acetate/hexanes gradient) to yield impure product. This was further purified by preparative HPLC (30-70% MeCN in water with a 0.1% NH_4OH modifier) to provide the desired compound as a white solid (148.0 mg, 0.365 mmol, 73% yield).

^{1}H NMR (500 MHz, $CDCl_3$) δ 5.32 (tt, *J* = 7.4, 2.4 Hz, 1H), 4.45 (s, 2H), 4.42 (s, 2H), 2.03 – 1.93 (m, 2H), 1.86 (dt, *J* = 12.2, 3.2 Hz, 1H), 1.69 – 1.55 (m, 3H), 1.56 – 1.16 (m, 16H), 1.13 (m, 4H), 1.01 (t, *J* = 4.1 Hz, 1H), 0.97 – 0.88 (m, 2H), 0.87 (s, 3H), 0.78 (s, 3H), 0.78 (s, 3H).

^{13}C NMR (126 MHz, CDCl$_3$) δ 156.59, 127.49, 123.35, 79.61, 74.25, 61.55, 57.69, 56.98, 56.26, 44.82, 42.07, 39.92, 39.24, 33.53, 33.39, 32.19, 28.55, 25.11, 24.05, 21.64, 20.71, 18.58, 15.52.

IR (film) ν_{max} 3425.35, 2930.46, 2866.60, 1680.58, 1456.00, 1387.80, 1365.37, 1251.55, 1152.38, 1123.46, 1083.61, 964.20, 938.43, 909.55, 859.94, 812.20, 771.36, 559.53 cm^{-1}.

HRMS (ESI-TOF) *m/z* calcd. for $C_{20}H_{36}NO^+$ ($[M-C_5H_9O_2+H]^+$) 306.2791, found 306.2793.

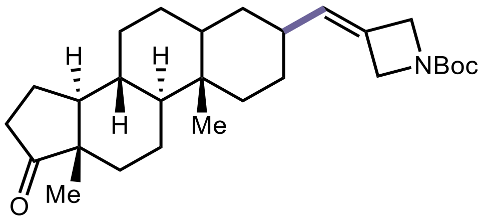

***tert*-butyl 3-(((3*R*,8*R*,9*S*,10*S*,13*S*,14*S*)-10,13-dimethyl-17-oxohexadecahydro-1H-cyclopenta[*a*]phenanthren-3-yl)methylene)azetidine-1-carboxylate (18)**

The title compound was prepared according to **General Procedure A** using **NHC** (375.5 mg, 0.95 mmol, 1.9 equiv.), androsterone (254.1 mg, 0.87 mmol, 1.75 equiv.), pyridine (76.8 µL, 0.95 mmol, 1.9 equiv.), and *t*-BuOMe (5 mL) for **Stock A** and Ir[(ppy)$_2$(dtbbpy)]PF$_6$ (6.9 mg, 7.5 µmol, 0.015 equiv.), NiBr$_2$(dtbbpy) (12.2 mg, 25.0 µmol, 0.05 equiv.), quinuclidine (105.6 mg, 0.95 mmol, 1.9 equiv.), *tert*-butyl 3-(bromomethylene)azetidine-1-carboxylate (124.1 mg, 0.50 mmol, 1.0 equiv.), and acetone (5 mL) for **Stock B**. The crude mixture was concentrated via *Genevac* then purified by automated flash chromatography (25 g high performance silica column, 0-25% ethyl acetate/hexanes gradient) to yield impure product. This was further purified by preparative HPLC (30-70% MeCN in water with a 0.1% NH_4OH modifier) to provide the desired compound as a yellow solid (176.3 mg, 0.399 mmol, 80% yield). The desired compound was isolated as an inseparable, unassigned 76:24 mixture of diastereomers (ratio determined by integration of the crude ^{1}H NMR). Spectral data is provided for the mixture of diastereomers.

^{1}H NMR (500 MHz, CDCl$_3$) δ 5.37 (ddt, *J* = 217.2, 8.4, 2.4 Hz, 1H), 4.45 (q, *J* = 11.4 Hz, 4H), 2.52 – 1.35 (m, 22H), 1.33 – 0.63 (m, 16H).

^{13}C NMR (126 MHz, $CDCl_3$) δ 221.59, 156.54, 128.09, 126.98, 126.38, 79.63, 79.60, 58.01, 57.90, 57.03, 57.00, 54.88, 54.82, 51.65, 47.98, 47.96, 46.43, 41.38, 38.90, 38.25, 36.52, 36.00, 35.92, 35.23, 35.20, 34.99, 33.51, 33.46, 31.73, 31.06, 31.03, 28.70, 28.63, 28.56, 28.28, 21.91, 21.89, 20.36, 20.18, 12.40, 11.97.

IR (film) ν_{max} 2923.70, 2853.21, 1739.47, 1696.94, 1452.34, 1387.41, 1363.19, 1251.89, 1179.06, 1151.13, 1121.98, 1055.44, 1037.50, 1006.14, 944.61, 860.44, 819.53, 778.86, 412.58 cm^{-1}.

HRMS (ESI-TOF) *m/z* calcd. for $C_{28}H_{43}NO_3Na^+$ ($[M+Na]^+$) 464.3135, found 464.3137.

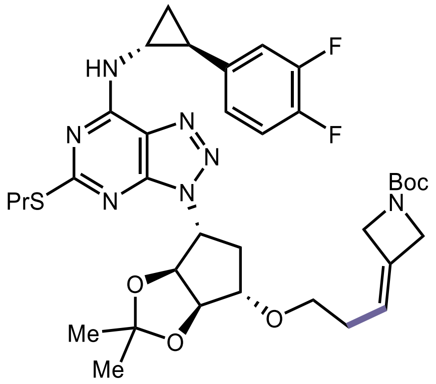

***tert*-butyl 3-(3-(((1*S*,2*S*,3*S*,4*R*)-4-(7-(((1*R*,2*S*)-2-(3,4-difluorophenyl)cyclopropyl)amino)-5-(phenylthio)-3H-[1,2,3]triazolo[4,5-*d*]pyrimidin-3-yl)-2,3-dihydroxycyclopentyl)oxy)propylidene)azetidine-1-carboxylate (19)**

The title compound was prepared according to **General Procedure A** using **NHC** (375.5 mg, 0.95 mmol, 1.9 equiv.), Ticagrelor acetonide (487.0 mg, 0.87 mmol, 1.75 equiv.), pyridine (76.8 µL, 0.95 mmol, 1.9 equiv.), and *t*-BuOMe (5 mL) for **Stock A** and Ir[(ppy)$_2$(dtbbpy)]PF_6 (6.9 mg, 7.5 µmol, 0.015 equiv.), $NiBr_2$(dtbbpy) (12.2 mg, 25.0 µmol, 0.05 equiv.), quinuclidine (105.6 mg, 0.95 mmol, 1.9 equiv.), *tert*-butyl 3-(bromomethylene)azetidine-1-carboxylate (124.1 mg, 0.50 mmol, 1.0 equiv.), and acetone (5 mL) for **Stock B.** The crude mixture was concentrated via *Genevac* then purified by automated flash chromatography (25 g high performance silica column, 0-25% ethyl acetate/hexanes gradient) to yield impure product. This was further purified by preparative HPLC (30-70% MeCN in water with a 0.1% NH_4OH modifier) to provide the desired compound as a yellow solid (286.7 mg, 0.402 mmol, 80% yield).

^{1}H NMR (500 MHz, DMSO) δ 9.18 (dd, *J* = 190.0, 4.6 Hz, 1H), 7.36 – 7.19 (m, 2H), 7.09 – 6.98 (m, 1H), 5.28 (ddd, *J* = 31.5, 7.1, 4.2 Hz, 1H), 5.17 (ddd, *J* = 12.4, 6.1, 3.6 Hz, 1H), 5.02 (ddd, *J* = 8.7, 6.8, 4.0 Hz, 1H), 4.65 (ddd, *J* = 19.3, 7.1, 2.8 Hz, 1H), 4.33 (d, *J* = 15.6 Hz, 4H), 3.95 (td, *J* = 6.7, 2.9 Hz, 1H), 3.48 – 3.32 (m, 2H), 3.23 – 2.81 (m, 3H), 2.64 – 2.51 (m, 2H), 2.28 – 2.09 (m, 1H), 1.99 (q, *J* = 6.9 Hz, 2H), 1.77 – 1.48 (m, 3H), 1.47 (s, 3H), 1.37 (s, 10H), 1.26 (d, *J* = 6.8 Hz, 3H), 0.92 (dt, *J* = 74.8, 7.3 Hz, 3H).

^{13}C NMR (126 MHz, DMSO) δ 169.89, 156.19, 154.39, 150.03 (dd, *J* = 200.4, 12.6 Hz), 149.53, 148.09 (dd, *J* = 198.9, 12.7 Hz), 139.68 (dd, *J* = 6.2, 3.3 Hz), 129.68, 123.60, 123.34 (dd, *J* = 6.3, 3.2 Hz), 119.21, 117.49 (d, *J* = 16.9 Hz), 115.41 (d, *J* = 17.3 Hz), 112.64, 84.21, 82.60, 82.22, 79.19, 68.24, 62.20, 57.48 (br), 35.55, 34.44, 32.84, 29.01, 28.46, 27.24, 25.09, 24.42, 22.87, 15.39, 13.52.

^{19}F NMR (471 MHz, DMSO) δ -139.29 – -139.46 (m, 1F), -143.03 – -143.25 (m, 1F).

IR (film) ν_{max} 3264.17, 2974.51, 2931.98, 2867.43, 1698.11, 1605.54, 1588.13, 1519.66, 1453.59, 1381.94, 1366.19, 1318.81, 1275.17, 1208.44, 1157.29, 1113.24, 1045.12, 989.84, 940.53, 862.52, 811.80, 791.82, 770.85, 715.86, 689.31, 657.18, 619.31, 579.71 cm^{-1}.

HRMS (ESI-TOF) *m/z* calcd. for $C_{35}H_{46}F_2N_7O_5S^+$ ([M+H]$^+$) 714.3244, found 714.3242.

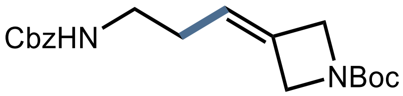

***tert*-butyl 3-(3-(((benzyloxy)carbonyl)amino)propylidene)azetidine-1-carboxylate (20)**

The title compound was prepared according to **General Procedure A** using **NHC** (375.5 mg, 0.95 mmol, 1.9 equiv.), benzyl (2-hydroxyethyl)carbamate (170.8 mg, 0.87 mmol, 1.75 equiv.), pyridine (76.8 µL, 0.95 mmol, 1.9 equiv.), and *t*-BuOMe (5 mL) for **Stock A** and Ir[(ppy)-$_2$(dtbbpy)]PF_6 (6.9 mg, 7.5 µmol, 0.015 equiv.), $NiBr_2$(dtbbpy) (12.2 mg, 25.0 µmol, 0.05 equiv.), quinuclidine (105.6 mg, 0.95 mmol, 1.9 equiv.), *tert*-butyl 3-(bromomethylene)azetidine-1-carboxylate (124.1 mg, 0.50 mmol, 1.0 equiv.), and acetone (5 mL) for **Stock B**. The crude mixture was concentrated via *Genevac* then purified by automated flash chromatography (25 g high

performance silica column, 0-25% ethyl acetate/hexanes gradient) to yield impure product. This was further purified by preparative HPLC (30-70% MeCN in water with a 0.1% NH_4OH modifier) to provide the desired compound as an oily solid (150.9 mg, 0.436 mmol, 87% yield).

^{1}H NMR (500 MHz, $CDCl_3$) δ 7.38 – 7.28 (m, 5H), 5.25 (d, *J* = 7.9 Hz, 1H), 5.09 (s, 2H), 4.81 (s, 1H), 4.45 – 4.40 (m, 4H), 3.23 (q, *J* = 6.6 Hz, 2H), 2.11 (q, *J* = 7.2 Hz, 2H), 1.45 (s, 9H).

^{13}C NMR (126 MHz, $CDCl_3$) δ 156.47, 136.62, 130.97, 128.69, 128.31, 128.28, 128.24, 118.92, 79.77, 66.87, 57.65, 56.86, 40.42, 29.41, 28.52.

IR (film) ν_{max} 3329.23, 2975.11, 2931.93, 2865.15, 1683.24, 1527.44, 1477.12, 1454.06, 1391.15, 1365.20, 1243.29, 1120.86, 1035.91, 939.41, 859.40, 808.67, 772.98, 736.23, 696.14, 606.58, 576.57, 456.86 cm^{-1}.

HRMS (ESI-TOF) *m/z* calcd. for $C_{19}H_{26}N_2O_4Na^+$ ($[M+Na]^+$) 369.1785, found 369.1789.

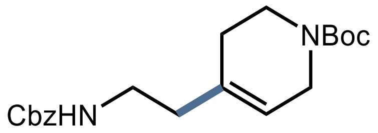

***tert*-butyl 4-(2-(((benzyloxy)carbonyl)amino)ethyl)-3,6-dihydropyridine-1(*2H*)-carboxylate (21)**

The title compound was prepared according to **General Procedure A** using **NHC** (375.5 mg, 0.95 mmol, 1.9 equiv.), benzyl (2-hydroxyethyl)carbamate (170.8 mg, 0.87 mmol, 1.75 equiv.), pyridine (76.8 µL, 0.95 mmol, 1.9 equiv.), and *t*-BuOMe (5 mL) for **Stock A** and Ir[(ppy)-$_2$(dtbbpy)]PF_6 (6.9 mg, 7.5 µmol, 0.015 equiv.), $NiBr_2$(dtbbpy) (12.2 mg, 25.0 µmol, 0.05 equiv.), quinuclidine (105.6 mg, 0.95 mmol, 1.9 equiv.), *tert*-butyl 4-bromo-3,6-dihydropyridine-1(*2H*)-carboxylate (131.1 mg, 0.50 mmol, 1.0 equiv.), and acetone (5 mL) for **Stock B.** The crude mixture was concentrated via *Genevac* then purified by automated flash chromatography (25 g high performance silica column, 0-25% ethyl acetate/hexanes gradient) to yield impure product. This was further purified by preparative HPLC (30-70% MeCN in water with a 0.1% NH_4OH modifier) to provide the desired compound as an yellow solid (130.6 mg, 0.363 mmol, 73% yield).

^{1}H NMR (500 MHz, CDCl$_3$) δ 7.41 – 7.27 (m, 5H), 5.40 (s, 1H), 5.08 (s, 2H), 4.75 (s, 1H), 3.83 (s, 2H), 3.46 (t, J = 5.7 Hz, 2H), 3.30 (q, J = 6.5 Hz, 2H), 2.20 (t, J = 6.9 Hz, 2H), 2.03 (s, 2H), 1.46 (s, 9H).

^{13}C NMR (126 MHz, CDCl$_3$) δ 156.38, 155.01, 136.66, 133.45, 128.66, 128.28, 120.79 (br), 120.37 (br), 79.68, 66.80, 43.50 – 43.05 (rotamers), 40.95 – 39.72 (rotamers), 38.71, 37.46, 28.59, 28.10.

IR (film) ν_{max} 3319.05, 2975.02, 2924.12, 2864.13, 1712.80, 1673.76, 1545.01, 1452.39, 1430.76, 1390.56, 1364.75, 1339.65, 1303.72, 1282.75, 1239.99, 1153.00, 1137.51, 1111.07, 1049.18, 1029.84, 999.66, 911.34, 871.56, 856.67, 832.40, 817.15, 777.41, 759.57, 698.37, 671.57, 632.13, 581.30, 549.49, 528.88 cm^{-1}.

HRMS (ESI-TOF) *m/z* calcd. for $C_{15}H_{21}N_2O_2^+$ ([M-$C_5H_9O_2$+H]$^+$) 261.1598, found 261.1599.

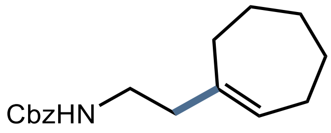

benzyl (2-(cyclohept-1-en-1-yl)ethyl)carbamate (22)

The title compound was prepared according to **General Procedure A** using **NHC** (375.5 mg, 0.95 mmol, 1.9 equiv.), benzyl (2-hydroxyethyl)carbamate (170.8 mg, 0.87 mmol, 1.75 equiv.), pyridine (76.8 µL, 0.95 mmol, 1.9 equiv.), and *t*-BuOMe (5 mL) for **Stock A** and Ir[(ppy)$_2$(dtbbpy)]PF$_6$ (6.9 mg, 7.5 µmol, 0.015 equiv.), NiBr$_2$(dtbbpy) (12.2 mg, 25.0 µmol, 0.05 equiv.), quinuclidine (105.6 mg, 0.95 mmol, 1.9 equiv.), 1-bromocyclohept-1-ene (65.6 µL, 0.50 mmol, 1.0 equiv.), and acetone (5 mL) for **Stock B**. The vinyl bromide was added via syringe after sparging the reaction due to its volatility. The crude mixture was concentrated via *Genevac* then purified by automated flash chromatography (25 g high performance silica column, 0-25% ethyl acetate/hexanes gradient) to yield impure product. This was further purified by preparative HPLC (30-70% MeCN in water with a 0.1% NH_4OH modifier) to provide the desired compound as a white solid (83.0 mg, 0.304 mmol, 61% yield).

^{1}H NMR (500 MHz, CDCl$_3$) δ 7.38 – 7.28 (m, 5H), 5.60 (t, *J* = 6.4 Hz, 1H), 5.10 (s, 2H), 4.72 (s, 1H), 3.26 (q, *J* = 6.3 Hz, 2H), 2.16 (t, *J* = 6.7 Hz, 2H), 2.13 – 2.03 (m, 4H), 1.72 (p, *J* = 6.0 Hz, 2H), 1.50 – 1.41 (m, 4H).

^{13}C NMR (126 MHz, CDCl$_3$) δ 156.41, 141.25, 136.83, 129.09, 128.65, 128.28, 128.22, 66.70, 40.21, 39.00, 32.63, 32.38, 28.46, 27.35, 26.90.

IR (film) ν_{max} 3335.42, 2917.46, 2846.11, 1696.83, 1512.89, 1453.12, 1243.33, 1215.57, 1132.21, 1086.25, 1026.28, 1002.07, 907.88, 845.82, 774.03, 733.92, 695.19, 605.32, 575.66, 536.36, 459.12, 412.81 cm^{-1}.

HRMS (ESI-TOF) *m/z* calcd. for $C_{17}H_{24}NO_2^+$ ($[M+H]^+$) 274.1802, found 274.1798.

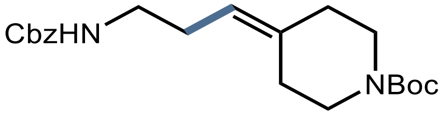

***tert*-butyl 4-(3-(((benzyloxy)carbonyl)amino)propylidene)piperidine-1-carboxylate (23)**

The title compound was prepared according to **General Procedure A** using **NHC** (375.5 mg, 0.95 mmol, 1.9 equiv.), benzyl (2-hydroxyethyl)carbamate (170.8 mg, 0.87 mmol, 1.75 equiv.), pyridine (76.8 µL, 0.95 mmol, 1.9 equiv.), and *t*-BuOMe (5 mL) for **Stock A** and Ir[(ppy)-$_2$(dtbbpy)]PF$_6$ (6.9 mg, 7.5 µmol, 0.015 equiv.), NiBr$_2$(dtbbpy) (12.2 mg, 25.0 µmol, 0.05 equiv.), quinuclidine (105.6 mg, 0.95 mmol, 1.9 equiv.), *tert*-butyl 4-(bromomethylene)piperidine-1-carboxylate (138.1 mg, 0.50 mmol, 1.0 equiv.), and acetone (5 mL) for **Stock B**. The crude mixture was concentrated via *Genevac* then purified by automated flash chromatography (25 g high performance silica column, 0-25% ethyl acetate/hexanes gradient) to yield impure product. This was further purified by preparative HPLC (30-70% MeCN in water with a 0.1% NH_4OH modifier) to provide the desired compound as a viscous yellow oil (152.7 mg, 0.408 mmol, 82% yield).

^{1}H NMR (500 MHz, CDCl$_3$) δ 7.37 – 7.29 (m, 5H), 5.13 (d, *J* = 39.0 Hz, 3H), 4.78 (s, 1H), 3.36 (dt, *J* = 11.7, 5.7 Hz, 4H), 3.20 (q, *J* = 6.7 Hz, 2H), 2.36 – 1.94 (m, 6H), 1.46 (s, 9H).

^{13}C NMR (126 MHz, CDCl$_3$) δ 156.34, 154.75, 138.26, 136.57, 128.55, 128.19, 128.17, 119.91, 79.51, 66.66, 45.14 (br), 40.93, 35.92, 28.47, 27.68.

IR (film) ν_{max} 3333.18, 2971.21, 1691.60, 1528.91, 1453.50, 1421.28, 1364.18, 1325.26, 1233.35, 1163.12, 1115.78, 1088.24, 1027.75, 1003.94, 862.94, 771.21, 735.99, 696.33, 672.15, 625.02, 535.52, 456.75 cm^{-1}.

HRMS (ESI-TOF) *m/z* calcd. for $C_{21}H_{30}N_2O_4Na^+$ ($[M+Na]^+$) 397.2098, found 397.2099.

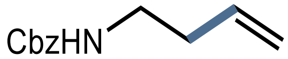

benzyl but-3-en-1-ylcarbamate (24)

The title compound was prepared according to **General Procedure A** using **NHC** (375.5 mg, 0.95 mmol, 1.9 equiv.), benzyl (2-hydroxyethyl)carbamate (170.8 mg, 0.87 mmol, 1.75 equiv.), pyridine (76.8 µL, 0.95 mmol, 1.9 equiv.), and *t*-BuOMe (5 mL) for **Stock A** and Ir[(ppy)-$_2$(dtbbpy)]PF_6 (6.9 mg, 7.5 µmol, 0.015 equiv.), $NiBr_2$(dtbbpy) (12.2 mg, 25.0 µmol, 0.05 equiv.), quinuclidine (105.6 mg, 0.95 mmol, 1.9 equiv.), 1.0 M vinyl bromide in THF (500 µL, 0.50 mmol, 1.0 equiv.), and acetone (5 mL) for **Stock B**. The vinyl bromide THF solution was added via syringe after sparging the reaction due to its volatility. The crude mixture was concentrated via *Genevac* then purified by automated flash chromatography (25 g high performance silica column, 0-25% ethyl acetate/hexanes gradient) to yield impure product. This was further purified by preparative HPLC (30-70% MeCN in water with a 0.1% NH_4OH modifier) to provide the desired compound as a white solid (72.9 mg, 0.355 mmol, 71% yield).

^{1}H NMR (500 MHz, CDCl$_3$) δ 7.41 – 7.31 (m, 5H), 5.75 (ddt, *J* = 17.1, 10.2, 6.8 Hz, 1H), 5.14 – 5.05 (m, 4H), 4.78 (s, 1H), 3.28 (q, *J* = 6.4 Hz, 2H), 2.27 (q, *J* = 6.8 Hz, 2H).

^{13}C NMR (126 MHz, CDCl$_3$) δ 156.45, 136.73, 135.20, 128.66, 128.27, 128.25, 117.49, 66.78, 40.21, 34.25.

IR (film) ν_{max} 3332.16, 3066.47, 3033.22, 2938.57, 1694.37, 1641.04, 1586.34, 1519.24, 1454.08, 1437.75, 1414.86, 1363.71, 1331.48, 1244.29, 1215.81, 1132.11, 1025.26, 991.40, 914.32, 823.89, 775.29, 735.12, 695.43, 639.75, 604.76, 576.15 cm^{-1}.

HRMS (ESI-TOF) *m/z* calcd. for $C_{12}H_{16}NO_2^+$ ($[M+H]^+$) 206.1176, found 206.1178.

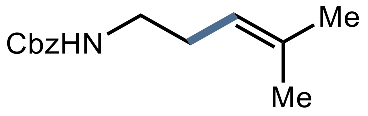

benzyl (4-methylpent-3-en-1-yl)carbamate (25)

The title compound was prepared according to **General Procedure A** using **NHC** (375.5 mg, 0.95 mmol, 1.9 equiv.), benzyl (2-hydroxyethyl)carbamate (170.8 mg, 0.87 mmol, 1.75 equiv.), pyridine (76.8 µL, 0.95 mmol, 1.9 equiv.), and *t*-BuOMe (5 mL) for **Stock A** and Ir[(ppy)-$_2$(dtbbpy)]PF_6 (6.9 mg, 7.5 µmol, 0.015 equiv.), $NiBr_2$(dtbbpy) (12.2 mg, 25.0 µmol, 0.05 equiv.), quinuclidine (105.6 mg, 0.95 mmol, 1.9 equiv.), 1-bromo-2-methylprop-1-ene (51.2 µL, 0.50 mmol, 1.0 equiv.), and acetone (5 mL) for **Stock B**. The vinyl bromide was added via syringe after sparging the reaction due to its volatility. The crude mixture was concentrated via *Genevac* then purified by automated flash chromatography (25 g high performance silica column, 0-25% ethyl acetate/hexanes gradient) to yield impure product. This was further purified by preparative HPLC (30-70% MeCN in water with a 0.1% NH_4OH modifier) to provide the desired compound as a transparent liquid (65.7 mg, 0.282 mmol, 56% yield).

^{1}H NMR (500 MHz, $CDCl_3$) δ 7.41 – 7.29 (m, 5H), 5.31 – 4.92 (m, 3H), 4.74 (s, 1H), 3.20 (q, *J* = 6.5 Hz, 2H), 2.20 (q, *J* = 7.1 Hz, 2H), 1.70 (s, 3H), 1.61 (s, 3H).

^{13}C NMR (126 MHz, $CDCl_3$) δ 156.50, 136.83, 134.86, 128.66, 128.29, 128.23, 120.73, 66.73, 41.09, 28.64, 25.91, 17.95.

IR (film) ν_{max} 3334.60, 2967.47, 2928.45, 1695.63, 1517.87, 1453.57, 1376.66, 1245.94, 1133.04, 1111.51, 1065.65, 1023.06, 907.49, 825.12, 775.71, 734.42, 695.46, 605.71, 576.08, 536.52, 475.26, 455.15, 437.67, 412.85 cm^{-1}.

HRMS (ESI-TOF) *m/z* calcd. for $C_{14}H_{20}NO_2^+$ ($[M+H]^+$) 234.1489, found 234.1486.

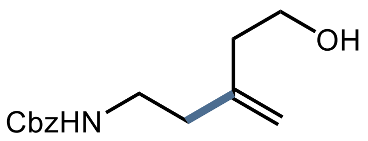

benzyl (5-hydroxy-3-methylenepentyl)carbamate (26)

The title compound was prepared according to **General Procedure A** using **NHC** (375.5 mg, 0.95 mmol, 1.9 equiv.), benzyl (2-hydroxyethyl)carbamate (170.8 mg, 0.87 mmol, 1.75 equiv.), pyridine (76.8 µL, 0.95 mmol, 1.9 equiv.), and *t*-BuOMe (5 mL) for **Stock A** and Ir[(ppy)-$_2$(dtbbpy)]PF_6 (6.9 mg, 7.5 µmol, 0.015 equiv.), $NiBr_2$(dtbbpy) (12.2 mg, 25.0 µmol, 0.05 equiv.), quinuclidine (105.6 mg, 0.95 mmol, 1.9 equiv.), 3-bromobut-3-en-1-ol (75.5 mg, 0.50 mmol, 1.0 equiv.), and acetone (5 mL) for **Stock B**. The crude mixture was concentrated via *Genevac* then purified by automated flash chromatography (25 g high performance silica column, 0-25% ethyl acetate/hexanes gradient) to yield impure product. This was further purified by preparative HPLC (30-70% MeCN in water with a 0.1% NH_4OH modifier) to provide the desired compound as a white solid (94.3 mg, 0.378 mmol, 76% yield).

^{1}H NMR (500 MHz, $CDCl_3$) δ 7.39 – 7.28 (m, 5H), 5.09 (s, 2H), 4.91 (dd, *J* = 9.7, 1.5 Hz, 2H), 4.84 (s, 1H), 3.73 (t, *J* = 6.3 Hz, 2H), 3.34 (t, *J* = 6.9 Hz, 2H), 2.30 (t, *J* = 6.2 Hz, 2H), 2.25 (t, *J* = 6.8 Hz, 2H), 1.62 (s, 1H).

^{13}C NMR (126 MHz, $CDCl_3$) δ 156.55, 142.97, 136.70, 128.67, 128.28, 114.05, 66.85, 60.55, 39.09, 38.88, 36.36.

IR (film) ν_{max} 3320.56, 3062.26, 2946.06, 1685.26, 1644.93, 1541.85, 1499.27, 1453.02, 1374.07, 1321.78, 1300.63, 1261.40, 1216.14, 1170.30, 1139.15, 1072.82, 1046.84, 1014.27, 894.60, 868.11, 840.65, 780.78, 746.35, 694.73, 670.93, 620.55, 574.10, 487.56, 462.98, 433.62, 413.05 cm^{-1}.

HRMS (ESI-TOF) *m/z* calcd. for $C_{14}H_{19}NO_3Na^+$ ($[M+Na]^+$) 272.1257, found 272.1257.

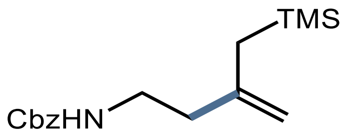

benzyl (3-((trimethylsilyl)methyl)but-3-en-1-yl)carbamate (27)

The title compound was prepared according to **General Procedure A** using **NHC** (375.5 mg, 0.95 mmol, 1.9 equiv.), benzyl (2-hydroxyethyl)carbamate (170.8 mg, 0.87 mmol, 1.75 equiv.), pyridine (76.8 µL, 0.95 mmol, 1.9 equiv.), and *t*-BuOMe (5 mL) for **Stock A** and Ir[(ppy)-$_2$(dtbbpy)]PF_6 (6.9 mg, 7.5 µmol, 0.015 equiv.), $NiBr_2$(dtbbpy) (12.2 mg, 25.0 µmol, 0.05 equiv.), quinuclidine (105.6 mg, 0.95 mmol, 1.9 equiv.), (2-bromoallyl)trimethylsilane (86.1 µL, 0.50 mmol, 1.0 equiv.), phthalimide (16.6 mg, 0.113 mmol, 0.225 equiv.), and acetone (5 mL) for **Stock B**. The vinyl bromide was added via syringe after sparging the reaction due to its volatility. The crude mixture was concentrated via *Genevac* then purified by automated flash chromatography (25 g high performance silica column, 0-25% ethyl acetate/hexanes gradient) to yield impure product. This was further purified by preparative HPLC (30-70% MeCN in water with a 0.1% NH_4OH modifier) to provide the desired compound as a light brown oil (78.7 mg, 0.270 mmol, 54% yield).

^{1}H NMR (500 MHz, C_6D_6) δ 7.27 – 7.25 (m, 2H), 7.13 – 7.04 (m, 3H), 5.11 (s, 2H), 4.55 (s, 2H), 4.36 (s, 1H), 3.18 (q, J = 6.6 Hz, 2H), 1.92 (t, J = 7.0 Hz, 2H), 1.35 (s, 2H), -0.04 (s, 9H).

^{13}C NMR (126 MHz, C_6D_6) δ 156.24, 144.61, 137.67, 128.65, 128.52, 128.13, 109.28, 66.60, 39.31, 38.52, 26.44, -1.38.

IR (film) ν_{max} 3339.09, 3068.68, 3033.61, 2952.62, 2895.11, 1697.87, 1632.71, 1513.86, 1454.35, 1417.51, 1363.35, 1246.07, 1135.00, 1053.97, 1027.03, 1001.89, 837.30, 772.49, 751.08, 733.97, 694.49, 656.02, 625.74, 574.80, 531.80 cm^{-1}.

HRMS (ESI-TOF) *m/z* calcd. for $C_{16}H_{26}NO_2Si^+$ ([M+H]$^+$) 292.1727, found 292.1728.

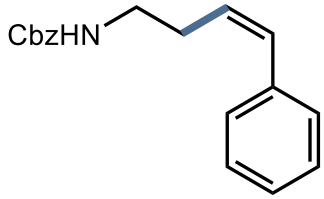

benzyl (*E/Z*)-(4-phenylbut-3-en-1-yl)carbamate (28)

The title compound was prepared according to **General Procedure A** using **NHC** (375.5 mg, 0.95 mmol, 1.9 equiv.), benzyl (2-hydroxyethyl)carbamate (170.8 mg, 0.87 mmol, 1.75 equiv.), pyridine (76.8 µL, 0.95 mmol, 1.9 equiv.), and *t*-BuOMe (5 mL) for **Stock A** and Ir[(ppy)-$_2$(dtbbpy)]PF_6 (6.9 mg, 7.5 µmol, 0.015 equiv.), $NiBr_2$(dtbbpy) (12.2 mg, 25.0 µmol, 0.05 equiv.), quinuclidine (105.6 mg, 0.95 mmol, 1.9 equiv.), (*E/Z*)-β-bromostyrene (64.1 µL, 0.50 mmol, 1.0 equiv.), and acetone (5 mL) for **Stock B**. The vinyl bromide was added via syringe after sparging the reaction due to its volatility. The crude mixture was concentrated via *Genevac* then purified by automated flash chromatography (25 g high performance silica column, 0-25% ethyl acetate/hexanes gradient) to yield impure product. This was further purified by preparative HPLC (30-70% MeCN in water with a 0.1% NH_4OH modifier) to provide the desired compound as a white solid (122.2 mg, 0.435 mmol, 87% yield). The compound was isolated as *90:10* mixture of *Z:E* isomers (configuration determined by NOE correlations). Spectral data is provided for the mixture of diastereomers.

^{1}H NMR (500 MHz, $CDCl_3$) δ 7.38 – 7.21 (m, 10H), 6.55 (d, *J* = 11.6 Hz, 1H), 5.62 (dt, *J* = 12.0, 7.3 Hz, 1H), 5.10 (d, *J* = 6.2 Hz, 2H), 4.79 (s, 1H), 3.32 (q, *J* = 6.6 Hz, 2H), 2.56 (q, *J* = 7.1 Hz, 2H).

^{13}C NMR (126 MHz, $CDCl_3$) δ 156.48, 137.21, 136.69, 131.63, 128.81, 128.65, 128.38, 128.23, 127.01, 126.23, 66.78, 41.06, 29.23.

IR (film) ν_{max} 3332.62, 3060.44, 3032.27, 3014.85, 2968.26, 2940.42, 2878.24, 1687.34, 1654.19, 1599.79, 1539.20, 1492.76, 1461.99, 1448.17, 1425.65, 1362.32, 1323.59, 1268.18, 1210.09, 1160.69, 1144.02, 1080.02, 1066.17, 1047.61, 1027.49, 1014.99, 960.15, 908.09, 845.83, 786.43, 774.80, 747.18, 718.57, 697.39, 689.16, 664.27, 620.73, 575.41, 564.25, 534.40 cm^{-1}.

HRMS (ESI-TOF) *m/z* calcd. for $C_{18}H_{20}NO_2^+$ ($[M+H]^+$) 282.1489, found 282.1485.

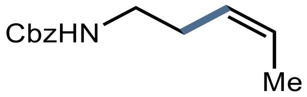

benzyl (*Z*)-pent-3-en-1-ylcarbamate (29)

The title compound was prepared according to **General Procedure A** using **NHC** (375.5 mg, 0.95 mmol, 1.9 equiv.), benzyl (2-hydroxyethyl)carbamate (170.8 mg, 0.87 mmol, 1.75 equiv.), pyridine (76.8 µL, 0.95 mmol, 1.9 equiv.), and *t*-BuOMe (5 mL) for **Stock A** and Ir[(ppy)-$_2$(dtbbpy)]PF_6 (6.9 mg, 7.5 µmol, 0.015 equiv.), $NiBr_2$(dtbbpy) (12.2 mg, 25.0 µmol, 0.05 equiv.), quinuclidine (105.6 mg, 0.95 mmol, 1.9 equiv.), (*Z*)-1-bromoprop-1-ene (42.5 µL, 0.50 mmol, 1.0 equiv.), and acetone (5 mL) for **Stock B**. The vinyl bromide was added via syringe after sparging the reaction due to its volatility. The crude mixture was concentrated via *Genevac* then purified by automated flash chromatography (25 g high performance silica column, 0-25% ethyl acetate/hexanes gradient) to yield impure product. This was further purified by preparative HPLC (30-70% MeCN in water with a 0.1% NH_4OH modifier) to provide the desired compound as a light yellow liquid (73.7 mg, 0.336 mmol, 67% yield). Some stereo-erosion in the desired product was observed, which was isolated as *89:11* mixture of *cis*- and *trans*- isomers (configuration determined by NOE correlations). Spectral data is provided for the mixture of diastereomers.

^{1}H NMR (500 MHz, $CDCl_3$) δ 7.40 – 7.28 (m, 5H), 5.65 – 5.47 (m, 1H), 5.39 – 5.30 (m, 1H), 5.10 (s, 2H), 4.78 (s, 1H), 3.23 (p, *J* = 6.7 Hz, 2H), 2.45 – 2.11 (m, 2H), 1.61 (d, *J* = 6.7 Hz, 3H).

^{13}C NMR (126 MHz, $CDCl_3$) δ 156.50, 136.78, 128.65, 128.26, 128.23, 127.10, 126.61, 66.74, 40.77, 27.49, 13.01.

IR (film) ν_{max} 3334.48, 3064.92, 3015.28, 2936.68, 1694.64, 1586.42, 1518.62, 1453.92, 1404.60, 1361.95, 1326.95, 1240.16, 1211.95, 1134.36, 1026.79, 997.24, 909.20, 823.86, 775.51, 735.09, 695.00, 606.92 cm^{-1}.

HRMS (ESI-TOF) *m/z* calcd. for $C_{13}H_{18}NO_2^+$ ($[M+H]^+$) 220.1332, found 220.1332.

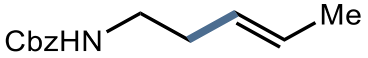

benzyl (*E*)-pent-3-en-1-ylcarbamate (30)

The title compound was prepared according to **General Procedure A** using **NHC** (375.5 mg, 0.95 mmol, 1.9 equiv.), benzyl (2-hydroxyethyl)carbamate (170.8 mg, 0.87 mmol, 1.75 equiv.), pyridine (76.8 µL, 0.95 mmol, 1.9 equiv.), and *t*-BuOMe (5 mL) for **Stock A** and Ir[(ppy)-$_2$(dtbbpy)]PF_6 (6.9 mg, 7.5 µmol, 0.015 equiv.), $NiBr_2$(dtbbpy) (12.2 mg, 25.0 µmol, 0.05 equiv.), quinuclidine (105.6 mg, 0.95 mmol, 1.9 equiv.), (*E*)-1-bromoprop-1-ene (43.0 µL, 0.50 mmol, 1.0 equiv.), and acetone (5 mL) for **Stock B**. The vinyl bromide was added via syringe after sparging the reaction due to its volatility. The crude mixture was concentrated via *Genevac* then purified by automated flash chromatography (25 g high performance silica column, 0-25% ethyl acetate/hexanes gradient) to yield impure product. This was further purified by preparative HPLC (30-70% MeCN in water with a 0.1% NH_4OH modifier) to provide the desired compound as a white solid (74.7 mg, 0.341 mmol, 68% yield). No stereo-erosion in the desired product was observed, which was formed as a *trans*-isomer (configuration determined by NOE correlations).

^{1}H NMR (500 MHz, $CDCl_3$) δ 7.40 – 7.28 (m, 5H), 5.57 – 5.46 (m, 1H), 5.40 – 5.30 (m, 1H), 5.10 (s, 2H), 4.75 (s, 1H), 3.22 (q, *J* = 6.4 Hz, 2H), 2.18 (q, *J* = 6.9 Hz, 2H), 1.66 (dd, *J* = 6.5, 1.4 Hz, 3H).

^{13}C NMR (126 MHz, $CDCl_3$) δ 156.44, 136.79, 128.67, 128.30, 128.25, 128.19, 127.66, 66.75, 40.73, 33.06, 18.11.

IR (film) ν_{max} 3314.24, 3061.79, 3029.06, 2916.98, 2855.49, 1685.02, 1537.50, 1498.56, 1452.68, 1370.66, 1247.55, 1140.45, 1069.62, 1015.91, 964.83, 906.88, 841.26, 780.16, 746.07, 695.38, 619.40, 572.67, 523.64, 469.26 cm^{-1}.

HRMS (ESI-TOF) *m/z* calcd. for $C_{13}H_{18}NO_2^+$ ($[M+H]^+$) 220.1332, found 220.1329.

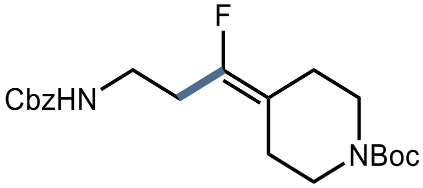

***tert*-butyl 4-(3-(((benzyloxy)carbonyl)amino)-1-fluoropropylidene)piperidine-1-carboxylate (31)**

The title compound was prepared according to **General Procedure A** using **NHC** (375.5 mg, 0.95 mmol, 1.9 equiv.), benzyl (2-hydroxyethyl)carbamate (170.8 mg, 0.87 mmol, 1.75 equiv.), pyridine (76.8 µL, 0.95 mmol, 1.9 equiv.), and *t*-BuOMe (5 mL) for **Stock A** and Ir[(ppy)-$_2$(dtbbpy)]PF_6 (6.9 mg, 7.5 µmol, 0.015 equiv.), $NiBr_2$(dtbbpy) (12.2 mg, 25.0 µmol, 0.05 equiv.), quinuclidine (105.6 mg, 0.95 mmol, 1.9 equiv.), *tert*-butyl 4-(bromofluoromethylene)piperidine-1-carboxylate (147.1 mg, 0.50 mmol, 1.0 equiv.), phthalimide (73.6 mg, 0.50 mmol, 1.0 equiv.), and acetone (5 mL) for **Stock B**. The vinyl bromide was added via syringe after sparging the reaction due to its volatility. The crude mixture was concentrated via *Genevac* then purified by automated flash chromatography (25 g high performance silica column, 0-25% ethyl acetate/hexanes gradient) to yield impure product. This was further purified by preparative HPLC (30-70% MeCN in water with a 0.1% NH_4OH modifier) to provide the desired compound as a white solid (158.0 mg, 0.403 mmol, 81% yield).

^{1}H NMR (500 MHz, $CDCl_3$) δ 7.39 – 7.28 (m, 5H), 5.09 (s, 2H), 4.94 – 4.56 (m, 1H), 3.39 – 3.30 (m, 6H), 2.49 (dt, *J* = 23.4, 6.5 Hz, 2H), 2.26 (d, *J* = 6.2 Hz, 2H), 2.05 (t, *J* = 5.7 Hz, 2H), 1.47 (s, 9H).

^{13}C NMR (126 MHz, $CDCl_3$) δ 156.29, 154.67, 150.57 (d, *J* = 244.4 Hz), 136.44, 128.56, 128.25, 128.21, 114.17 (d, *J* = 16.2 Hz), 79.63, 66.76, 44.32 (br), 38.25, 28.70 (d, *J* = 28.9 Hz), 28.46, 27.40, 25.14.

^{19}F NMR (471 MHz, $CDCl_3$) δ -113.88 (d, *J* = 23.3 Hz, 1F).

IR (film) ν_{max} 3306.16, 3067.22, 2969.01, 2934.14, 2906.28, 2865.56, 1708.21, 1671.31, 1540.59, 1499.05, 1474.96, 1434.26, 1392.28, 1365.27, 1331.42, 1279.18, 1253.45, 1235.63, 1217.74,

1165.99, 1132.55, 1078.63, 1052.42, 1017.83, 957.77, 946.09, 911.38, 889.69, 851.95, 820.70, 783.14, 767.54, 751.26, 730.47, 697.44, 650.02, 618.81, 600.34, 577.50, 559.20, 542.37 cm^{-1}.

HRMS (ESI-TOF) *m/z* calcd. for $C_{17}H_{22}FN_2O_4^+$ ($[M-C_4H_9+H]^+$) 337.1558, found 337.1555.

6) Experimental Data for Deoxygenative Bromoalkenylation Products

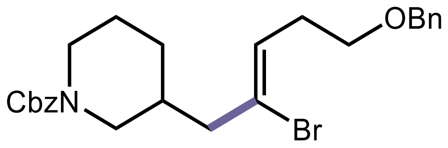

(±)-benzyl (*Z*)-3-(5-(benzyloxy)-2-bromopent-2-en-1-yl)piperidine-1-carboxylate (32)

The title compound was prepared according to **General Procedure B** using **NHC** (276.7 mg, 0.70 mmol, 1.4 equiv.), benzyl 3-(hydroxymethyl)piperidine-1-carboxylate (162.0 mg, 0.65 mmol, 1.3 equiv.), pyridine (56.6 µL, 0.70 mmol, 1.4 equiv.), and *t*-BuOMe (5 mL) for **Stock A** and Ir[(ppy)-$_2$(dtbbpy)]PF_6 (6.9 mg, 7.5 µmol, 0.015 equiv.), $NiBr_2$(dtbbpy) (24.3 mg, 50.0 µmol, 0.10 equiv.), quinuclidine (77.8 mg, 0.70 mmol, 1.4 equiv.), phthalimide (73.6 mg, 0.50 mmol, 1.0 equiv.), **52-DiBr** (152.0 mg, 0.50 mmol, 1.0 equiv.), and 1,2-dichlorobenzene (10 mL) for **Stock B**. The crude mixture was concentrated via *Genevac* then purified by automated flash chromatography (25 g high performance silica column, 0-25% ethyl acetate/hexanes gradient) to yield impure product. This was further purified by preparative HPLC (30-70% MeCN in water with a 0.1% NH_4OH modifier) to provide the desired compound as a yellow oil (178.8 mg, 0.379 mmol, 76% yield). The product was isolated in a >20:1 *Z:E* ratio of diastereomers (configuration determined by vinylic NOE correlations).

^{1}H NMR (500 MHz, $CDCl_3$) δ 7.51 – 7.27 (m, 10H), 5.74 (t, *J* = 6.7 Hz, 1H), 5.19 – 5.08 (m, 2H), 4.50 (s, 2H), 3.96 (dt, *J* = 13.3, 4.2 Hz, 2H), 3.50 (s, 2H), 2.89 (ddd, *J* = 13.8, 11.0, 3.1 Hz, 1H), 2.60 (s, 1H), 2.48 (q, *J* = 6.5 Hz, 2H), 2.37 (s, 1H), 2.30 (dd, *J* = 14.3, 7.2 Hz, 1H), 1.92 (th, *J* = 14.3, 4.2 Hz, 1H), 1.81 (dq, *J* = 13.1, 4.2 Hz, 1H), 1.69 – 1.61 (m, 1H), 1.51 – 1.40 (m, 1H), 1.11 (d, *J* = 19.0 Hz, 1H).

^{13}C NMR (126 MHz, $CDCl_3$) δ 155.38, 138.46, 137.06, 128.58, 128.50, 128.01, 127.88, 127.76, 127.71, 127.29, 126.95, 72.95, 68.70, 67.08, 49.26, 49.25, 49.12, 45.46, 44.71, 34.01, 32.15, 29.95, 24.61.

IR (film) ν_{max} 3030.06, 2931.73, 2853.36, 1694.30, 1496.56, 1466.76, 1427.41, 1360.91, 1257.97, 1234.62, 1182.45, 1139.53, 1100.06, 1027.41, 963.42, 911.94, 853.07, 763.44, 733.08, 695.48, 604.23, 558.52, 459.18, 405.79 cm^{-1}.

HRMS (ESI-TOF) *m/z* calcd. for $C_{25}H_{30}BrNO_3Na^+$ ($[M+Na]^+$) 494.1301, found 494.1301.

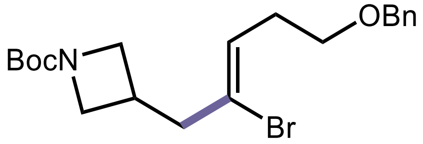

***tert*-butyl (*Z*)-3-(5-(benzyloxy)-2-bromopent-2-en-1-yl)azetidine-1-carboxylate (33)**

The title compound was prepared according to **General Procedure B** using **NHC** (276.7 mg, 0.70 mmol, 1.4 equiv.), *tert*-butyl 3-(hydroxymethyl)azetidine-1-carboxylate (121.7 mg, 0.65 mmol, 1.3 equiv.), pyridine (56.6 µL, 0.70 mmol, 1.4 equiv.), and *t*-BuOMe (5 mL) for **Stock A** and $Ir[(ppy)_2(dtbbpy)]PF_6$ (6.9 mg, 7.5 µmol, 0.015 equiv.), $NiBr_2(dtbbpy)$ (24.3 mg, 50.0 µmol, 0.10 equiv.), quinuclidine (77.8 mg, 0.70 mmol, 1.4 equiv.), phthalimide (73.6 mg, 0.50 mmol, 1.0 equiv.), **52-DiBr** (152.0 mg, 0.50 mmol, 1.0 equiv.), and 1,2-dichlorobenzene (10 mL) for **Stock B**. The crude mixture was concentrated via *Genevac* then purified by automated flash chromatography (25 g high performance silica column, 0-25% ethyl acetate/hexanes gradient) to yield impure product. This was further purified by preparative HPLC (30-70% MeCN in water with a 0.1% NH_4OH modifier) to provide the desired compound as a yellow oil (144.4 mg, 0.352 mmol, 70% yield). The product was isolated in a >20:1 *Z:E* ratio of diastereomers (configuration determined by vinylic NOE correlations).

^{1}H NMR (500 MHz, $CDCl_3$) δ 7.38 – 7.27 (m, 5H), 5.78 (t, *J* = 6.7 Hz, 1H), 4.51 (s, 2H), 4.01 (t, *J* = 8.4 Hz, 2H), 3.56 (dd, *J* = 8.7, 5.3 Hz, 2H), 3.51 (t, *J* = 6.5 Hz, 2H), 2.89 – 2.78 (m, 1H), 2.71 (d, *J* = 7.6 Hz, 2H), 2.47 (q, *J* = 6.5 Hz, 2H), 1.44 (s, 9H).

^{13}C NMR (126 MHz, $CDCl_3$) δ 156.50, 138.40, 128.56, 127.80, 127.79, 127.03, 126.26, 79.50, 73.04, 68.59, 53.79, 46.10, 32.14, 28.55, 27.73.

IR (film) ν_{max} 2966.40, 2932.51, 2876.69, 1694.59, 1495.08, 1477.90, 1453.76, 1389.06, 1362.69, 1290.67, 1252.67, 1133.03, 1097.56, 1027.96, 928.09, 859.70, 770.60, 734.70, 696.68, 606.13, 561.32, 463.75, 417.58 cm^{-1}.

HRMS (ESI-TOF) *m/z* calcd. for $C_{15}H_{21}BrNO^+$ ($[M-C_5H_9O_2+H]^+$) 310.0801, found 310.0801.

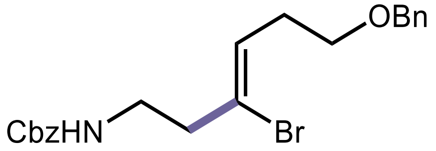

benzyl (*Z*)-(6-(benzyloxy)-3-bromohex-3-en-1-yl)carbamate (34)

The title compound was prepared according to **General Procedure B** using **NHC** (276.7 mg, 0.70 mmol, 1.4 equiv.), benzyl (2-hydroxyethyl)carbamate (126.9 mg, 0.65 mmol, 1.3 equiv.), pyridine (56.6 µL, 0.70 mmol, 1.4 equiv.), and *t*-BuOMe (5 mL) for **Stock A** and Ir[(ppy)$_2$(dtbbpy)]PF_6 (6.9 mg, 7.5 µmol, 0.015 equiv.), $NiBr_2$(dtbbpy) (24.3 mg, 50.0 µmol, 0.10 equiv.), quinuclidine (77.8 mg, 0.70 mmol, 1.4 equiv.), phthalimide (73.6 mg, 0.50 mmol, 1.0 equiv.), **52-DiBr** (152.0 mg, 0.50 mmol, 1.0 equiv.), and 1,2-dichlorobenzene (15 mL) for **Stock B**. The crude mixture was concentrated via *Genevac* then purified by automated flash chromatography (25 g high performance silica column, 0-25% ethyl acetate/hexanes gradient) to yield impure product. This was further purified by preparative HPLC (30-70% MeCN in water with a 0.1% NH_4OH modifier) to provide the desired compound as a viscous clear oil (145.6 mg, 0.349 mmol, 70% yield). The product was isolated in a >20:1 *Z:E* ratio of diastereomers (configuration determined by vinylic NOE correlations).

^{1}H NMR (500 MHz, $CDCl_3$) δ 7.38 – 7.27 (m, 10H), 5.81 (t, *J* = 6.7 Hz, 1H), 5.09 (s, 2H), 4.84 (d, *J* = 6.3 Hz, 1H), 4.50 (s, 2H), 3.51 (t, *J* = 6.5 Hz, 2H), 3.41 (q, *J* = 6.2 Hz, 2H), 2.63 (t, *J* = 8.1 Hz, 2H), 2.48 (q, *J* = 6.6 Hz, 2H).

^{13}C NMR (126 MHz, $CDCl_3$) δ 156.39, 138.42, 136.68, 130.69, 128.67, 128.55, 128.46, 128.28, 127.79, 127.77, 125.98, 73.03, 68.52, 66.85, 41.76, 39.25, 32.23.

IR (film) ν_{max} 3340.49, 3063.60, 3031.95, 2949.97, 1698.75, 1600.64, 1584.10, 1519.17, 1497.18, 1453.06, 1411.09, 1362.33, 1313.35, 1246.88, 1204.58, 1176.18, 1108.99, 1069.37, 1025.63, 1001.64, 910.02, 827.70, 774.93, 737.04, 712.67, 695.84, 648.93, 603.19, 575.78, 545.76, 492.05, 456.30, 419.35, 410.23 cm^{-1}.

HRMS (ESI-TOF) *m/z* calcd. for $C_{21}H_{25}BrNO_3^+$ ($[M+H]^+$) 418.1012, found 418.1015.

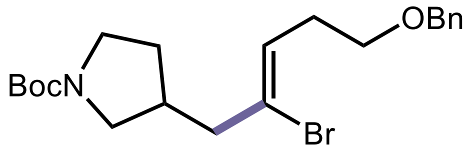

(±)-*tert*-butyl (*Z*)-3-(5-(benzyloxy)-2-bromopent-2-en-1-yl)pyrrolidine-1-carboxylate (35)

The title compound was prepared according to **General Procedure B** using **NHC** (276.7 mg, 0.70 mmol, 1.4 equiv.), (±)-*tert*-butyl 3-(hydroxymethyl)pyrrolidine-1-carboxylate (130.8 mg, 0.65 mmol, 1.3 equiv.), pyridine (56.6 µL, 0.70 mmol, 1.4 equiv.), and *t*-BuOMe (5 mL) for **Stock A** and Ir[(ppy)$_2$(dtbbpy)]PF_6 (6.9 mg, 7.5 µmol, 0.015 equiv.), $NiBr_2$(dtbbpy) (24.3 mg, 50.0 µmol, 0.10 equiv.), quinuclidine (77.8 mg, 0.70 mmol, 1.4 equiv.), phthalimide (73.6 mg, 0.50 mmol, 1.0 equiv.), **52-DiBr** (152.0 mg, 0.50 mmol, 1.0 equiv.), and 1,2-dichlorobenzene (10 mL) for **Stock B**. The crude mixture was concentrated via *Genevac* then purified by automated flash chromatography (25 g high performance silica column, 0-25% ethyl acetate/hexanes gradient) to yield impure product. This was further purified by preparative HPLC (30-70% MeCN in water with a 0.1% NH_4OH modifier) to provide the desired compound as an opaque oil (123.1 mg, 0.290 mmol, 58% yield). The product was isolated in a >20:1 *Z:E* ratio of diastereomers (configuration determined by vinylic NOE correlations).

^{1}H NMR (500 MHz, CDCl$_3$) δ 7.38 – 7.25 (m, 5H), 5.78 (t, *J* = 6.6 Hz, 1H), 4.52 (s, 2H), 3.55 (t, *J* = 6.6 Hz, 2H), 3.41 (ddd, *J* = 12.1, 8.1, 3.9 Hz, 1H), 3.28 (dt, *J* = 10.9, 7.8 Hz, 1H), 2.92 (dd, *J* = 10.8, 7.3 Hz, 1H), 2.59 – 2.43 (m, 5H), 1.95 (dtd, *J* = 13.4, 6.8, 4.3 Hz, 1H), 1.52 (dq, *J* = 12.5, 8.2 Hz, 1H), 1.45 (s, 9H).

^{13}C NMR (126 MHz, CDCl$_3$) δ 154.72, 138.43, 128.53, 127.80, 127.76, 127.62, 126.91, 79.23, 73.02, 68.66, 50.65, 45.39, 45.09, 37.03 (br), 32.15, 30.57 (br), 28.68.

IR (film) ν_{max} 3003.29, 2972.60, 2930.65, 2862.68, 1688.64, 1602.01, 1477.26, 1452.52, 1390.70, 1364.00, 1313.56, 1271.82, 1254.39, 1203.28, 1164.20, 1110.76, 1070.54, 1026.35, 880.36, 827.71, 772.13, 737.33, 712.46, 697.44, 648.90, 615.89, 588.02, 542.58, 462.08, 433.40, 423.96, 415.64, 406.88 cm^{-1}.

HRMS (ESI-TOF) *m/z* calcd. for $C_{16}H_{23}BrNO^+$ ($[M-C_5H_9O_2+H]^+$) 324.0958, found 324.0960.

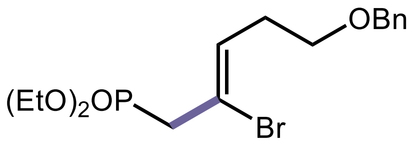

diethyl (*Z*)-(5-(benzyloxy)-2-bromopent-2-en-1-yl)phosphonate (36)

The title compound was prepared according to **General Procedure B** using **NHC** (375.5 mg, 0.95 mmol, 1.9 equiv.), diethyl (hydroxymethyl)phosphonate (147.1 mg, 0.65 mmol, 1.75 equiv.), pyridine (76.8 µL, 0.95 mmol, 1.9 equiv.), and *t*-BuOMe (5 mL) for **Stock A** and Ir[(ppy)-$_2$(dtbbpy)]PF_6 (6.9 mg, 7.5 µmol, 0.015 equiv.), $NiBr_2$(dtbbpy) (24.3 mg, 50.0 µmol, 0.10 equiv.), quinuclidine (77.8 mg, 0.70 mmol, 1.4 equiv.), phthalimide (73.6 mg, 0.50 mmol, 1.0 equiv.), **52-DiBr** (152.0 mg, 0.50 mmol, 1.0 equiv.), and 1,2-dichlorobenzene (10 mL) for **Stock B**. The crude mixture was concentrated via *Genevac* then purified by automated flash chromatography (25 g high performance silica column, 0-25% ethyl acetate/hexanes gradient) to yield impure product. This was further purified by preparative HPLC (30-70% MeCN in water with a 0.1% NH_4OH modifier) to provide the desired compound as a yellow oil (97.5 mg, 0.249 mmol, 50% yield). The product was isolated in a >20:1 *Z:E* ratio of diastereomers (configuration determined by vinylic NOE correlations).

^{1}H NMR (500 MHz, $CDCl_3$) δ 7.39 – 7.29 (m, 5H), 6.06 – 6.01 (m, 1H), 4.54 (s, 2H), 4.15 (dqd, *J* = 7.9, 7.1, 1.1 Hz, 4H), 3.57 (t, *J* = 6.6 Hz, 2H), 3.11 (dq, *J* = 20.9, 0.9 Hz, 2H), 2.56 – 2.50 (m, 2H), 1.34 (t, *J* = 7.1 Hz, 6H).

^{13}C NMR (126 MHz, CDCl$_3$) δ 138.39, 130.95 (d, *J* = 10.7 Hz), 128.53, 127.79, 127.76, 116.24 (d, *J* = 12.2 Hz), 73.05, 68.43 (d, *J* = 3.5 Hz), 62.53 (d, *J* = 6.7 Hz), 39.41 (d, *J* = 141.4 Hz), 32.74 (d, J = 2.7 Hz), 16.54 (d, *J* = 6.0 Hz).

IR (film) ν_{max} 3029.38, 2980.62, 2905.07, 2859.86, 1650.95, 1495.76, 1477.90, 1453.51, 1391.78, 1363.70, 1253.07, 1220.57, 1162.61, 1097.37, 1049.24, 1019.99, 959.31, 839.37, 778.35, 736.32, 697.58, 605.93, 526.00, 485.73, 419.48 cm^{-1}.

HRMS (ESI-TOF) *m/z* calcd. for $C_{16}H_{25}BrO_4P^+$ ([M+H]$^+$) 391.0668, found 391.0671.

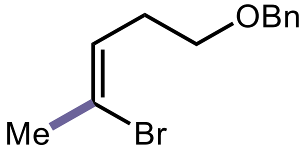

(*Z*)-(((4-bromopent-3-en-1-yl)oxy)methyl)benzene (37)

The title compound was prepared according to **General Procedure B** using **NHC** (276.7 mg, 0.70 mmol, 1.4 equiv.), methanol (26.3 µL, 0.65 mmol, 1.3 equiv.), pyridine (56.6 µL, 0.70 mmol, 1.4 equiv.), and *t*-BuOMe (5 mL) for **Stock A** and Ir[(ppy)$_2$(dtbbpy)]PF$_6$ (6.9 mg, 7.5 µmol, 0.015 equiv.), NiBr$_2$(dtbbpy) (24.3 mg, 50.0 µmol, 0.10 equiv.), quinuclidine (77.8 mg, 0.70 mmol, 1.4 equiv.), phthalimide (73.6 mg, 0.50 mmol, 1.0 equiv.), **52-DiBr** (152.0 mg, 0.50 mmol, 1.0 equiv.), and 1,2-dichlorobenzene (10 mL) for **Stock B**. Due to challenges in purification, the title compound was isolated only in analytical quantities for characterization. The pure material, obtained as a clear oil, was subsequently used to determine the assay yield of the crude reaction mixture by ^{1}H NMR analysis with mesitylene as an internal standard, which indicated a yield of 71%. The product was isolated in a >20:1 *Z:E* ratio of diastereomers (configuration determined by vinylic NOE correlations).

^{1}H NMR (500 MHz, CDCl$_3$) δ 7.39 – 7.27 (m, 5H), 5.72 (tq, *J* = 6.7, 1.4 Hz, 1H), 4.53 (s, 2H), 3.53 (t, *J* = 6.6 Hz, 2H), 2.47 (qq, *J* = 6.7, 1.4 Hz, 2H), 2.30 (d, *J* = 1.4 Hz, 3H).

^{13}C NMR (126 MHz, CDCl$_3$) δ 138.50, 128.53, 127.81, 127.73, 125.71, 124.07, 72.98, 68.76, 32.32, 28.97.

IR (film) ν_{max} 3063.64, 3030.20, 2952.40, 2917.46, 2858.96, 1719.50, 1702.65, 1664.51, 1597.56, 1584.13, 1495.43, 1453.01, 1426.86, 1377.93, 1360.92, 1310.86, 1272.67, 1247.00, 1203.13, 1175.70, 1166.96, 1099.77, 1072.20, 1027.24, 937.07, 908.83, 827.22, 742.22, 712.58, 696.95, 688.41, 649.59, 614.19, 579.25, 558.61, 532.14, 484.98, 450.97, 433.34, 420.17, 407.75 cm^{-1}.

HRMS (EI-QTOF) *m/z* calcd. for $C_{12}H_{15}BrO^{\bullet+}$ ($[M]^{\bullet+}$) 254.0301, found 254.0300.

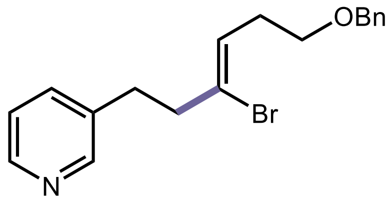

(*Z*)-3-(6-(benzyloxy)-3-bromohex-3-en-1-yl)pyridine (38)

The title compound was prepared according to **General Procedure B** using **NHC** (276.7 mg, 0.70 mmol, 1.4 equiv.), 2-(pyridin-3-yl)ethan-1-ol (80.1 mg, 0.65 mmol, 1.3 equiv.), pyridine (56.6 µL, 0.70 mmol, 1.4 equiv.), and *t*-BuOMe (5 mL) for **Stock A** and Ir[(ppy)$_2$(dtbbpy)]PF_6 (6.9 mg, 7.5 µmol, 0.015 equiv.), $NiBr_2$(dtbbpy) (24.3 mg, 50.0 µmol, 0.10 equiv.), quinuclidine (77.8 mg, 0.70 mmol, 1.4 equiv.), phthalimide (73.6 mg, 0.50 mmol, 1.0 equiv.), **52-DiBr** (152.0 mg, 0.50 mmol, 1.0 equiv.), and 1,2-dichlorobenzene (10 mL) for **Stock B**. The crude mixture was concentrated via *Genevac* then purified by automated flash chromatography (25 g high performance silica column, 0-25% ethyl acetate/hexanes gradient) to yield impure product. This was further purified by preparative HPLC (30-70% MeCN in water with a 0.1% NH_4OH modifier) to provide the desired compound as a viscous brown oil (117.5 mg, 0.340 mmol, 68% yield). The product was isolated in a >20:1 *Z:E* ratio of diastereomers (configuration determined by vinylic NOE correlations).

^{1}H NMR (500 MHz, $CDCl_3$) δ 8.50 – 8.39 (m, 2H), 7.49 (dt, *J* = 7.8, 2.0 Hz, 1H), 7.38 – 7.26 (m, 5H), 7.18 (dd, *J* = 7.8, 4.8 Hz, 1H), 5.65 (t, *J* = 6.7 Hz, 1H), 4.48 (s, 2H), 3.44 (t, *J* = 6.5 Hz, 2H), 2.88 (dd, *J* = 8.2, 6.5 Hz, 2H), 2.76 – 2.68 (m, 2H), 2.44 (q, *J* = 6.5 Hz, 2H).

^{13}C NMR (126 MHz, CDCl$_3$) δ 150.09, 147.70, 138.42, 136.30, 136.01, 128.51, 127.85, 127.78, 127.74, 127.04, 123.37, 72.97, 68.59, 43.18, 32.11, 31.73.

IR (film) ν_{max} 3028.05, 2857.42, 1727.84, 1657.12, 1574.68, 1495.32, 1478.15, 1452.61, 1422.59, 1360.57, 1210.84, 1157.70, 1100.01, 1026.86, 909.59, 884.28, 845.42, 798.08, 735.46, 713.15, 696.61, 652.64, 621.44, 568.51, 536.06, 515.01, 459.51 cm^{-1}.

HRMS (ESI-TOF) *m/z* calcd. for $C_{18}H_{21}BrNO^+$ ($[M+H]^+$) 346.0801, found 346.0801.

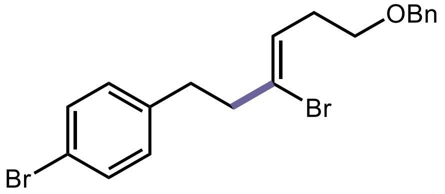

(*Z*)-1-(6-(benzyloxy)-3-bromohex-3-en-1-yl)-4-bromobenzene (39)

The title compound was prepared according to **General Procedure B** using **NHC** (276.7 mg, 0.70 mmol, 1.4 equiv.), 2-(4-bromophenyl)ethan-1-ol (130.7 mg, 0.65 mmol, 1.3 equiv.), pyridine (56.6 µL, 0.70 mmol, 1.4 equiv.), and *t*-BuOMe (5 mL) for **Stock A** and Ir[(ppy)$_2$(dtbbpy)]PF$_6$ (6.9 mg, 7.5 µmol, 0.015 equiv.), NiBr$_2$(dtbbpy) (24.3 mg, 50.0 µmol, 0.10 equiv.), quinuclidine (77.8 mg, 0.70 mmol, 1.4 equiv.), phthalimide (73.6 mg, 0.50 mmol, 1.0 equiv.), **52-DiBr** (152.0 mg, 0.50 mmol, 1.0 equiv.), and 1,2-dichlorobenzene (10 mL) for **Stock B**. The crude mixture was concentrated via *Genevac* then purified by automated flash chromatography (25 g high performance silica column, 0-25% ethyl acetate/hexanes gradient) to yield impure product. This was further purified by preparative HPLC (30-70% MeCN in water with a 0.1% NH_4OH modifier) to provide the desired compound as a viscous brown oil (143.7 mg, 0.339 mmol, 68% yield). The product was isolated in a >20:1 *Z:E* ratio of diastereomers (configuration determined by vinylic NOE correlations).

^{1}H NMR (500 MHz, CDCl$_3$) δ 7.42 – 7.27 (m, 7H), 7.08 – 7.02 (m, 2H), 5.65 (t, *J* = 6.7 Hz, 1H), 4.49 (s, 2H), 3.45 (t, *J* = 6.6 Hz, 2H), 2.83 (dd, *J* = 8.5, 6.4 Hz, 2H), 2.73 – 2.65 (m, 2H), 2.50 – 2.37 (m, 2H).

^{13}C NMR (126 MHz, $CDCl_3$) δ 139.68, 138.44, 131.49, 130.51, 128.52, 128.26, 127.79, 127.74, 126.58, 119.99, 72.98, 68.63, 43.41, 34.07, 32.12.

IR (film) ν_{max} 3027.98, 2857.14, 1657.11, 1487.05, 1452.55, 1428.19, 1403.73, 1360.23, 1211.02, 1160.91, 1100.10, 1071.28, 1027.76, 1010.54, 907.97, 810.96, 768.23, 733.56, 696.04, 653.45, 605.51, 557.34, 515.55 cm^{-1}.

HRMS (EI-QTOF) *m/z* calcd. for $C_{19}H_{20}Br^{81}BrO^{\bullet+}$ ([M, ^{81}Br isotope]$^{\bullet+}$) 423.9855, found 423.9851.

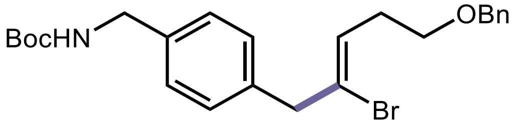

***tert*-butyl (*Z*)-(4-(5-(benzyloxy)-2-bromopent-2-en-1-yl)benzyl)carbamate (40)**

The title compound was prepared according to **General Procedure B** using **NHC** (276.7 mg, 0.70 mmol, 1.4 equiv.), *tert*-butyl (4-(hydroxymethyl)benzyl)carbamate (154.2 mg, 0.65 mmol, 1.3 equiv.), pyridine (56.6 µL, 0.70 mmol, 1.4 equiv.), and *t*-BuOMe (5 mL) for **Stock A** and Ir[(ppy)-$_2$(dtbbpy)]PF_6 (6.9 mg, 7.5 µmol, 0.015 equiv.), $NiBr_2$(dtbbpy) (24.3 mg, 50.0 µmol, 0.10 equiv.), quinuclidine (77.8 mg, 0.70 mmol, 1.4 equiv.), phthalimide (73.6 mg, 0.50 mmol, 1.0 equiv.), **52-DiBr** (152.0 mg, 0.50 mmol, 1.0 equiv.), and 1,2-dichlorobenzene (10 mL) for **Stock B**. The crude mixture was concentrated via *Genevac* then purified by automated flash chromatography (25 g high performance silica column, 0-25% ethyl acetate/hexanes gradient) to yield impure product. This was further purified by preparative HPLC (30-70% MeCN in water with a 0.1% NH_4OH modifier) to provide the desired compound as a viscous clear oil (120.7 mg, 0.262 mmol, 52% yield). The product was isolated in a >20:1 *Z:E* ratio of diastereomers (configuration determined by vinylic NOE correlations).

^{1}H NMR (500 MHz, $CDCl_3$) δ 7.38 – 7.27 (m, 5H), 7.22 (d, *J* = 7.9 Hz, 2H), 7.18 (d, *J* = 8.1 Hz, 2H), 5.84 (tt, *J* = 6.7, 1.2 Hz, 1H), 4.87 – 4.73 (m, 1H), 4.52 (s, 2H), 4.35 – 4.25 (m, 2H), 3.74 (s, 2H), 3.55 (t, *J* = 6.5 Hz, 2H), 2.56 – 2.45 (m, 2H), 1.47 (s, 9H).

^{13}C NMR (126 MHz, CDCl$_3$) δ 156.01, 138.47, 137.55, 137.05, 129.32, 128.53, 128.01, 127.78, 127.75, 127.21, 79.61, 72.97, 68.67, 47.56, 44.57, 32.23, 28.56.

IR (film) ν_{max} 3351.15, 3060.19, 2975.96, 2893.12, 2851.75, 2788.67, 1702.78, 1679.99, 1530.76, 1512.62, 1477.68, 1452.28, 1422.40, 1389.09, 1362.12, 1268.37, 1247.89, 1209.21, 1164.55, 1142.32, 1117.75, 1071.43, 1040.61, 1026.44, 1004.86, 957.52, 944.32, 896.93, 872.84, 841.63, 818.55, 767.70, 727.67, 694.03, 645.79, 623.98, 613.52, 587.25, 570.16, 535.00, 462.82, 434.50 cm^{-1}.

HRMS (ESI-TOF) *m/z* calcd. for $C_{24}H_{30}BrNaNO_3^+$ ([M+Na]$^+$) 482.1301, found 482.1299.

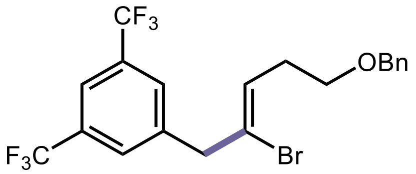

(*Z*)-1-(5-(benzyloxy)-2-bromopent-2-en-1-yl)-3,5-bis(trifluoromethyl)benzene (41)

The title compound was prepared according to **General Procedure B** using **NHC** (276.7 mg, 0.70 mmol, 1.4 equiv.), (3,5-bis(trifluoromethyl)phenyl)methanol (158.7 mg, 0.65 mmol, 1.3 equiv.), pyridine (56.6 µL, 0.70 mmol, 1.4 equiv.), and *t*-BuOMe (5 mL) for **Stock A** and Ir[(ppy)-$_2$(dtbbpy)]PF$_6$ (6.9 mg, 7.5 µmol, 0.015 equiv.), NiBr$_2$(dtbbpy) (24.3 mg, 50.0 µmol, 0.10 equiv.), quinuclidine (77.8 mg, 0.70 mmol, 1.4 equiv.), phthalimide (73.6 mg, 0.50 mmol, 1.0 equiv.), **52-DiBr** (152.0 mg, 0.50 mmol, 1.0 equiv.), and 1,2-dichlorobenzene (10 mL) for **Stock B**. The crude mixture was concentrated via *Genevac* then purified by automated flash chromatography (25 g high performance silica column, 0-25% ethyl acetate/hexanes gradient) to yield impure product. This was further purified by preparative HPLC (30-70% MeCN in water with a 0.1% NH_4OH modifier) to provide the desired compound as a viscous clear oil (147.2 mg, 0.315 mmol, 63% yield). The product was isolated in a >20:1 *Z:E* ratio of diastereomers (configuration determined by vinylic NOE correlations).

^{1}H NMR (500 MHz, CDCl$_3$) δ 7.79 (s, 1H), 7.68 (d, *J* = 1.8 Hz, 2H), 7.41 – 7.26 (m, 5H), 6.01 (t, *J* = 6.7 Hz, 1H), 4.54 (s, 2H), 3.88 (s, 2H), 3.58 (t, *J* = 6.4 Hz, 2H), 2.55 (q, *J* = 6.4 Hz, 2H).

^{13}C NMR (126 MHz, CDCl$_3$) δ 140.50, 138.32, 131.87 (q, *J* = 33.1 Hz), 129.40, 129.23, 129.19, 128.57, 127.84, 127.82, 123.45 (q, *J* = 272.8 Hz), 121.13 (p, *J* = 3.9 Hz), 73.12, 68.46, 47.35, 32.28.

^{19}F NMR (471 MHz, CDCl$_3$) δ -62.80 (s, 6F).

IR (film) ν_{max} 3032.37, 2862.77, 1704.65, 1656.37, 1623.53, 1496.26, 1454.41, 1428.04, 1375.50, 1274.75, 1168.22, 1125.58, 1027.99, 952.61, 893.44, 841.73, 736.12, 704.77, 698.11, 681.93, 584.08, 562.04, 498.12, 486.77, 458.40, 415.03 cm^{-1}.

HRMS (EI-QTOF) *m/z* calcd. for $C_{20}H_{17}BrF_6O^{\bullet+}$ ($[M]^{\bullet+}$) 466.0361, found 466.0357.

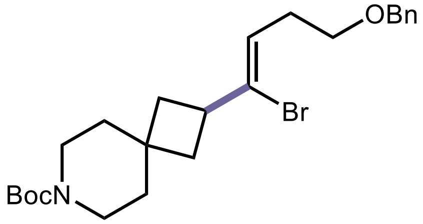

***tert*-butyl (*Z*)-2-(4-(benzyloxy)-1-bromobut-1-en-1-yl)-7-azaspiro[3.5]nonane-7-carboxylate (42)**

The title compound was prepared according to **General Procedure B** using **NHC** (316.2 mg, 0.80 mmol, 1.6 equiv.), *tert*-butyl 2-hydroxy-7-azaspiro[3.5]nonane-7-carboxylate (196.1 mg, 0.75 mmol, 1.5 equiv.), pyridine (64.7 µL, 0.80 mmol, 1.6 equiv.), and *t*-BuOMe (5 mL) for **Stock A** and Ir[(ppy)$_2$(dtbbpy)]PF$_6$ (6.9 mg, 7.5 µmol, 0.015 equiv.), NiBr$_2$(dtbbpy) (24.3 mg, 50.0 µmol, 0.10 equiv.), quinuclidine (77.8 mg, 0.70 mmol, 1.4 equiv.), phthalimide (73.6 mg, 0.50 mmol, 1.0 equiv.), **52-DiBr** (152.0 mg, 0.50 mmol, 1.0 equiv.), and 1,2-dichlorobenzene (10 mL) for **Stock B**. The crude mixture was concentrated via *Genevac* then purified by automated flash chromatography (25 g high performance silica column, 0-25% ethyl acetate/hexanes gradient) to yield impure product. This was further purified by preparative HPLC (30-70% MeCN in water with a 0.1% NH_4OH modifier) to provide the desired compound as a clear oil (139.8 mg, 0.301

mmol, 60% yield). The product was isolated in a >20:1 *Z:E* ratio of diastereomers (configuration determined by vinylic NOE correlations).

^{1}H NMR (500 MHz, CDCl$_3$) δ 7.40 – 7.27 (m, 5H), 5.73 (td, *J* = 6.6, 1.4 Hz, 1H), 4.52 (s, 2H), 3.53 (t, *J* = 6.6 Hz, 2H), 3.38 – 3.34 (m, 2H), 3.29 – 3.25 (m, 2H), 3.17 (pd, *J* = 8.8, 1.4 Hz, 1H), 2.49 (qd, *J* = 6.6, 1.2 Hz, 2H), 2.05 – 1.98 (m, 2H), 1.83 (td, *J* = 9.4, 2.5 Hz, 2H), 1.60 – 1.56 (m, 2H), 1.49 – 1.46 (m, 2H), 1.45 (s, 8H).

^{13}C NMR (126 MHz, CDCl$_3$) δ 155.10, 138.50, 134.74, 128.54, 127.81, 127.76, 123.13, 79.45, 72.97, 68.76, 40.96, 40.73, 39.03, 38.64, 37.60, 33.16, 32.01, 28.61.

IR (film) ν_{max} 3062.82, 3028.76, 2963.74, 2922.86, 2846.12, 1957.13, 1684.64, 1494.87, 1476.56, 1452.60, 1416.53, 1390.98, 1363.26, 1297.90, 1269.05, 1240.55, 1172.67, 1144.63, 1097.36, 1027.81, 994.74, 967.22, 925.36, 883.55, 860.02, 823.35, 734.28, 714.38, 696.46, 649.08, 536.92, 459.36, 439.54, 423.49, 402.75 cm^{-1}.

HRMS (ESI-TOF) *m/z* calcd. for $C_{24}H_{34}BrNNaO_3^+$ ([M+Na]$^+$) 486.1614, found 486.1612.

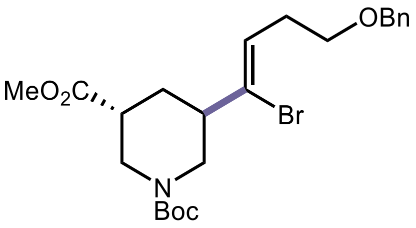

1-(*tert*-butyl) 3-methyl (3*R*,5*R*)-5-((*Z*)-4-(benzyloxy)-1-bromobut-1-en-1-yl)piperidine-1,3-dicarboxylate (43)

The title compound was prepared according to **General Procedure B** using **NHC** (276.7 mg, 0.70 mmol, 1.4 equiv.), 1-(*tert*-butyl) 3-methyl (3*R*,5*R*)-5-hydroxypiperidine-1,3-dicarboxylate (168.5 mg, 0.65 mmol, 1.3 equiv.), pyridine (56.6 µL, 0.70 mmol, 1.4 equiv.), and *t*-BuOMe (5 mL) for **Stock A** and Ir[(ppy)$_2$(dtbbpy)]PF$_6$ (6.9 mg, 7.5 µmol, 0.015 equiv.), NiBr$_2$(dtbbpy) (24.3 mg, 50.0 µmol, 0.10 equiv.), quinuclidine (77.8 mg, 0.70 mmol, 1.4 equiv.), phthalimide (73.6 mg, 0.50 mmol, 1.0 equiv.), **52-DiBr** (152.0 mg, 0.50 mmol, 1.0 equiv.), and 1,2-dichlorobenzene (10

mL) for **Stock B**. The crude mixture was concentrated via *Genevac* then purified by automated flash chromatography (25 g high performance silica column, 0-25% ethyl acetate/hexanes gradient) to yield impure product. This was further purified by preparative HPLC (30-70% MeCN in water with a 0.1% NH_4OH modifier) to provide the desired compound as a viscous light brown oil (126.6 mg, 0.263 mmol, 53% yield). The desired compound was isolated as an inseparable, unassigned 18:82 mixture of diastereomers (ratio determined by integration of the crude ^{1}H NMR). Spectral data is provided for the mixture of diastereomers. The product was isolated in a >20:1 *Z:E* ratio of diastereomers (configuration determined by vinylic NOE correlations).

^{1}H NMR (500 MHz, $CDCl_3$) δ 7.39 – 7.27 (m, 5H), 5.88 (t, *J* = 6.5 Hz, 1H), 4.51 (s, 2H), 4.41 – 4.14 (m, 2H), 3.70 (s, 3H), 3.52 (t, *J* = 6.5 Hz, 2H), 2.79 – 2.59 (m, 2H), 2.56 – 2.45 (m, 3H), 2.39 (tt, *J* = 10.9, 3.3 Hz, 1H), 2.23 (ddt, *J* = 12.8, 3.7, 1.8 Hz, 1H), 1.73 (q, *J* = 12.4 Hz, 1H), 1.45 (d, *J* = 10.4 Hz, 9H).

^{13}C NMR (126 MHz, $CDCl_3$) δ 173.31, 154.62, 138.38, 130.10, 128.55, 127.82, 127.78, 126.40, 80.34, 73.03, 68.50, 52.04, 45.88, 45.52, 42.56, 41.33, 33.26, 32.09, 28.53.

IR (film) ν_{max} 2974.40, 2862.58, 1734.42, 1690.99, 1453.19, 1418.92, 1365.14, 1296.39, 1250.32, 1146.03, 1099.77, 1027.58, 941.48, 889.22, 858.70, 765.64, 735.87, 697.33, 610.32, 514.84, 459.10, 405.73 cm^{-1}.

HRMS (ESI-TOF) *m/z* calcd. for $C_{23}H_{32}BrNaNO_5^+$ ($[M+Na]^+$) 504.1356, found 504.1355.

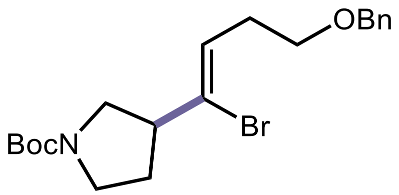

(±)-*tert*-butyl (*Z*)-3-(4-(benzyloxy)-1-bromobut-1-en-1-yl)pyrrolidine-1-carboxylate (44)

The title compound was prepared according to **General Procedure B** using **NHC** (276.7 mg, 0.70 mmol, 1.4 equiv.), (±)-*tert*-butyl 3-hydroxypyrrolidine-1-carboxylate (121.7 mg, 0.65 mmol, 1.3 equiv.), pyridine (56.6 µL, 0.70 mmol, 1.4 equiv.), and *t*-BuOMe (5 mL) for **Stock A** and Ir[(ppy)-

$_2$(dtbbpy)]PF_6 (6.9 mg, 7.5 µmol, 0.015 equiv.), $NiBr_2$(dtbbpy) (24.3 mg, 50.0 µmol, 0.10 equiv.), quinuclidine (77.8 mg, 0.70 mmol, 1.4 equiv.), phthalimide (73.6 mg, 0.50 mmol, 1.0 equiv.), **52-DiBr** (152.0 mg, 0.50 mmol, 1.0 equiv.), and 1,2-dichlorobenzene (10 mL) for **Stock B**. The crude mixture was concentrated via *Genevac* then purified by automated flash chromatography (25 g high performance silica column, 0-25% ethyl acetate/hexanes gradient) to yield impure product. This was further purified by preparative HPLC (30-70% MeCN in water with a 0.1% NH_4OH modifier) to provide the desired compound as a light brown oil (116.9 mg, 0.286 mmol, 57% yield). The product was isolated in a >20:1 *Z:E* ratio of diastereomers (configuration determined by vinylic NOE correlations).

^{1}H NMR (500 MHz, $CDCl_3$) δ 7.40 – 7.26 (m, 5H), 5.89 (t, *J* = 0.9 Hz, 1H), 4.52 (s, 2H), 3.59 – 3.49 (m, 4H), 3.34 – 3.24 (m, 2H), 3.05 (p, *J* = 8.3 Hz, 1H), 2.50 (q, *J* = 0.7 Hz, 2H), 2.04 – 1.98 (m, 2H), 1.46 (s, 9H).

^{13}C NMR (126 MHz, $CDCl_3$) δ 154.57, 138.40, 129.41, 128.55, 127.80, 127.79, 126.29, 79.44, 73.01, 68.52, 50.26 (br), 48.09 – 48.72 (rotamers), 45.37, 32.10, 30.45 – 31.21 (rotamers), 28.67.

IR (film) ν_{max} 2974.28, 2930.44, 2874.79, 1690.54, 1494.87, 1477.32, 1453.10, 1398.60, 1363.79, 1341.16, 1247.60, 1213.57, 1166.15, 1121.43, 1100.01, 1028.08, 982.40, 950.84, 886.99, 873.40, 770.83, 734.97, 696.76, 660.27, 611.70, 578.39, 564.28, 519.82, 459.73, 420.02 cm^{-1}.

HRMS (ESI-TOF) *m/z* calcd. for $C_{20}H_{28}BrNaNO_3^+$ ($[M+Na]^+$) 432.1145, found 432.1141.

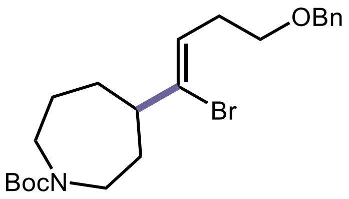

(±)-*tert*-butyl (*Z*)-4-(4-(benzyloxy)-1-bromobut-1-en-1-yl)azepane-1-carboxylate (45)

The title compound was prepared according to **General Procedure B** using **NHC** (276.7 mg, 0.70 mmol, 1.4 equiv.), (±)-*tert*-butyl 4-hydroxyazepane-1-carboxylate (139.9 mg, 0.65 mmol, 1.3 equiv.), pyridine (56.6 µL, 0.70 mmol, 1.4 equiv.), and *t*-BuOMe (5 mL) for **Stock A** and Ir[(ppy)-

$_2$(dtbbpy)]PF_6 (6.9 mg, 7.5 μmol, 0.015 equiv.), $NiBr_2$(dtbbpy) (24.3 mg, 50.0 μmol, 0.10 equiv.), quinuclidine (77.8 mg, 0.70 mmol, 1.4 equiv.), phthalimide (73.6 mg, 0.50 mmol, 1.0 equiv.), **52-DiBr** (152.0 mg, 0.50 mmol, 1.0 equiv.), and 1,2-dichlorobenzene (10 mL) for **Stock B**. The crude mixture was concentrated via *Genevac* then purified by automated flash chromatography (25 g high performance silica column, 0-25% ethyl acetate/hexanes gradient) to yield impure product. This was further purified by preparative HPLC (30-70% MeCN in water with a 0.1% NH_4OH modifier) to provide the desired compound as a light yellow oil (103.9 mg, 0.237 mmol, 47% yield). The product was isolated in a >20:1 *Z:E* ratio of diastereomers (configuration determined by vinylic NOE correlations).

^{1}H NMR (500 MHz, $CDCl_3$) δ 7.37 – 7.27 (m, 5H), 5.77 (t, *J* = 6.6 Hz, 1H), 4.52 (s, 2H), 3.52 (t, *J* = 5.6 Hz, 4H), 3.33 – 3.18 (m, 2H), 2.46 (q, *J* = 6.5 Hz, 2H), 2.33 (tdd, *J* = 10.3, 3.7, 1.8 Hz, 1H), 1.97 – 1.81 (m, 3H), 1.75 (dtd, *J* = 14.5, 10.2, 4.5 Hz, 1H), 1.65 – 1.53 (m, 2H), 1.47 (s, 9H).

^{13}C NMR (126 MHz, $CDCl_3$) δ 155.67, 138.49, 136.36, 128.53, 127.80, 127.75, 123.32, 79.37, 72.97, 68.75, 49.93 (br), 46.57, 44.85, 34.41, 33.25 (br), 31.88, 28.67, 27.17.

IR (film) ν_{max} 2972.63, 2928.85, 2859.88, 1686.24, 1477.68, 1453.06, 1412.32, 1390.58, 1363.77, 1310.34, 1247.64, 1161.17, 1098.85, 1027.65, 981.47, 897.59, 868.46, 771.26, 734.95, 696.73, 648.97, 610.93, 543.67, 460.30, 413.11 cm^{-1}.

HRMS (ESI-TOF) *m/z* calcd. for $C_{22}H_{32}BrNNaO_3^+$ ([M+Na]$^+$) 460.1458, found 460.1456.

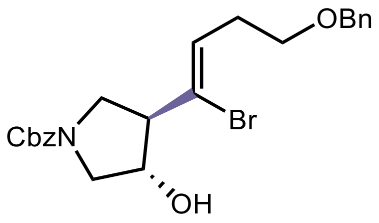

(±)-benzyl (3*R*,4*S*)-3-((*Z*)-4-(benzyloxy)-1-bromobut-1-en-1-yl)-4-hydroxypyrrolidine-1-carboxylate (46)

The title compound was prepared according to **General Procedure B** using **NHC** (276.7 mg, 0.70 mmol, 1.4 equiv.), benzyl (3*R*,4*R*)-3,4-dihydroxypyrrolidine-1-carboxylate (154.2 mg, 0.65 mmol,

1.3 equiv.), pyridine (56.6 µL, 0.70 mmol, 1.4 equiv.), and *t*-BuOMe (5 mL) for **Stock A** and Ir[$(ppy)_2$(dtbbpy)]PF_6 (6.9 mg, 7.5 µmol, 0.015 equiv.), $NiBr_2$(dtbbpy) (24.3 mg, 50.0 µmol, 0.10 equiv.), quinuclidine (77.8 mg, 0.70 mmol, 1.4 equiv.), phthalimide (73.6 mg, 0.50 mmol, 1.0 equiv.), **52-DiBr** (152.0 mg, 0.50 mmol, 1.0 equiv.), and 1,2-dichlorobenzene (10 mL) for **Stock B**. The crude mixture was concentrated via *Genevac* then purified by automated flash chromatography (25 g high performance silica column, 0-25% ethyl acetate/hexanes gradient) to yield impure product. This was further purified by preparative HPLC (30-70% MeCN in water with a 0.1% NH_4OH modifier) to provide the desired compound as a viscous clear oil (123.4 mg, 0.268 mmol, 54% yield). The desired compound was isolated as a single diastereomer, with the hydroxyl and vinyl groups possessing a *trans*- configuration (as assigned by NOE correlations).

^{1}H NMR (500 MHz, $CDCl_3$) δ 7.41 – 7.26 (m, 10H), 5.99 (t, *J* = 5.6 Hz, 1H), 5.13 (s, 2H), 4.51 (s, 2H), 4.40 (q, *J* = 7.0 Hz, 1H), 3.90 – 3.77 (m, 1H), 3.75 – 3.64 (m, 1H), 3.59 – 3.46 (m, 3H), 3.26 (dt, *J* = 10.7, 6.8 Hz, 1H), 2.93 (dq, *J* = 16.1, 8.0 Hz, 1H), 2.59 – 2.45 (m, 2H), 2.02 – 1.81 (m, 1H).

^{13}C NMR (126 MHz, $CDCl_3$) δ 154.86, 154.77, 138.23, 136.83, 136.79, 129.40, 128.63, 128.59, 128.18, 128.09, 128.03, 127.87, 127.83, 126.48, 73.15, 73.07, 72.44, 68.35, 67.14, 67.08, 56.42, 55.80, 51.83, 51.50, 48.66, 48.45, 32.20.

IR (film) ν_{max} 3402.27, 3030.72, 2883.09, 1675.90, 1496.47, 1452.57, 1418.49, 1357.99, 1318.48, 1209.16, 1173.96, 1092.42, 1028.03, 950.40, 913.97, 846.44, 767.21, 735.09, 695.49, 610.73, 571.46, 457.49 cm^{-1}.

HRMS (ESI-TOF) *m/z* calcd. for $C_{23}H_{26}BrNNaO_4^+$ ([M+Na]$^+$) 482.0937, found 482.0941.

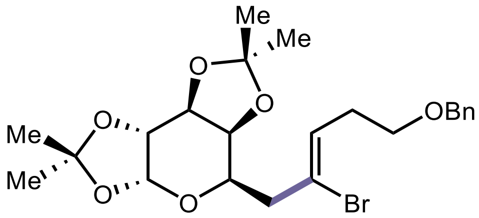

(3a*R*,5*R*,5a*S*,8a*S*,8b*R*)-5-((*Z*)-5-(benzyloxy)-2-bromopent-2-en-1-yl)-2,2,7,7-tetramethyltetrahydro-5H-bis([1,3]dioxolo)[4,5-b:4',5'-d]pyran (47)

The title compound was prepared according to **General Procedure B** using **NHC** (276.7 mg, 0.70 mmol, 1.4 equiv.), 1,2:3,4-di-O-isopropylidene-α-D-galactopyranose (169.2 mg, 0.65 mmol, 1.3 equiv.), pyridine (56.6 µL, 0.70 mmol, 1.4 equiv.), and *t*-BuOMe (5 mL) for **Stock A** and Ir[(ppy)-$_2$(dtbbpy)]PF_6 (6.9 mg, 7.5 µmol, 0.015 equiv.), $NiBr_2$(dtbbpy) (24.3 mg, 50.0 µmol, 0.10 equiv.), quinuclidine (77.8 mg, 0.70 mmol, 1.4 equiv.), phthalimide (73.6 mg, 0.50 mmol, 1.0 equiv.), **52-DiBr** (152.0 mg, 0.50 mmol, 1.0 equiv.), and 1,2-dichlorobenzene (10 mL) for **Stock B**. The crude mixture was concentrated via *Genevac* then purified by automated flash chromatography (25 g high performance silica column, 0-25% ethyl acetate/hexanes gradient) to yield impure product. This was further purified by preparative HPLC (30-70% MeCN in water with a 0.1% NH_4OH modifier) to provide the desired compound as a yellow oil (158.0 mg, 0.327 mmol, 65% yield). The product was isolated in a >20:1 *Z:E* ratio of diastereomers (configuration determined by vinylic NOE correlations).

^{1}H NMR (500 MHz, CDCl$_3$) δ 7.38 – 7.26 (m, 5H), 5.93 (t, *J* = 6.7 Hz, 1H), 5.51 (d, *J* = 5.0 Hz, 1H), 4.59 (dd, *J* = 7.8, 2.4 Hz, 1H), 4.52 (s, 2H), 4.29 (dd, *J* = 5.0, 2.4 Hz, 1H), 4.18 – 4.11 (m, 2H), 3.54 (t, *J* = 6.7 Hz, 2H), 2.82 – 2.69 (m, 2H), 2.51 (qd, *J* = 7.0, 3.8 Hz, 2H), 1.57 (s, 3H), 1.46 (s, 3H), 1.32 (d, *J* = 3.7 Hz, 6H).

^{13}C NMR (126 MHz, CDCl$_3$) δ 138.55, 128.50, 128.23, 127.77, 127.69, 125.00, 109.28, 108.79, 96.77, 72.98, 72.25, 71.05, 70.70, 68.71, 65.65, 42.49, 32.35, 26.17, 26.13, 25.16, 24.52.

IR (film) ν_{max} 3062.75, 2985.38, 2909.29, 2861.36, 1496.10, 1453.99, 1380.77, 1370.83, 1307.90, 1252.28, 1208.02, 1165.35, 1099.74, 1062.83, 996.40, 947.78, 917.46, 900.24, 864.07, 804.20, 772.71, 734.89, 696.62, 651.86, 611.72, 561.05, 510.89, 408.72 cm^{-1}.

HRMS (ESI-TOF) *m/z* calcd. for $C_{23}H_{31}BrNaO_6^+$ ($[M+Na]^+$) 505.1196, found 505.1195.

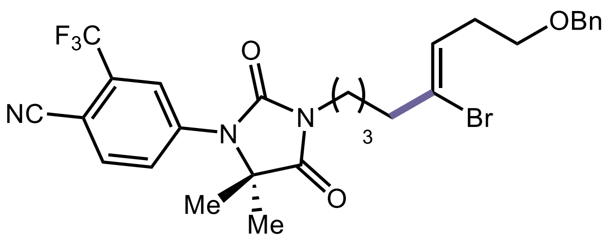

(*Z*)-4-(3-(8-(benzyloxy)-5-bromooct-5-en-1-yl)-5,5-dimethyl-2,4-dioxoimidazolidin-1-yl)-2-(trifluoromethyl)benzonitrile (48)

The title compound was prepared according to **General Procedure B** using **NHC** (276.7 mg, 0.70 mmol, 1.4 equiv.), 4-(3-(4-hydroxybutyl)-5,5-dimethyl-2,4-dioxoimidazolidin-1-yl)-2-(trifluoromethyl)benzonitrile (240.1 mg, 0.65 mmol, 1.3 equiv.), pyridine (56.6 µL, 0.70 mmol, 1.4 equiv.), and *t*-BuOMe (5 mL) for **Stock A** and Ir[(ppy)$_2$(dtbbpy)]PF$_6$ (6.9 mg, 7.5 µmol, 0.015 equiv.), NiBr$_2$(dtbbpy) (24.3 mg, 50.0 µmol, 0.10 equiv.), quinuclidine (77.8 mg, 0.70 mmol, 1.4 equiv.), phthalimide (73.6 mg, 0.50 mmol, 1.0 equiv.), **52-DiBr** (152.0 mg, 0.50 mmol, 1.0 equiv.), and 1,2-dichlorobenzene (10 mL) for **Stock B**. The crude mixture was concentrated via *Genevac* then purified by automated flash chromatography (25 g high performance silica column, 0-25% ethyl acetate/hexanes gradient) to yield impure product. This was further purified by preparative HPLC (30-70% MeCN in water with a 0.1% NH_4OH modifier) to provide the desired compound as a viscous brown oil (215.7 mg, 0.364 mmol, 73% yield). The product was isolated in a >20:1 *Z:E* ratio of diastereomers (configuration determined by vinylic NOE correlations).

^{1}H NMR (500 MHz, CDCl$_3$) δ 8.15 (s, 1H), 7.99 (dd, *J* = 8.4, 2.0 Hz, 1H), 7.90 (d, *J* = 8.4 Hz, 1H), 7.37 – 7.27 (m, 5H), 5.79 (t, *J* = 6.6 Hz, 1H), 4.51 (s, 2H), 3.53 (t, *J* = 6.5 Hz, 2H), 3.35 (t, *J* = 7.4 Hz, 2H), 2.49 (p, *J* = 6.7 Hz, 4H), 1.67 (ddt, *J* = 21.5, 8.5, 4.8 Hz, 4H), 1.51 (s, 6H).

^{13}C NMR (126 MHz, CDCl$_3$) δ 174.74, 152.89, 138.44, 136.62, 135.37, 133.72 (q, *J* = 33.3 Hz), 128.97, 128.52, 127.97, 127.76, 127.75, 126.29, 123.09 (q, *J* = 4.8 Hz), 122.10 (q, *J* = 274.2 Hz), 115.17, 108.33 (q, *J* = 2.2 Hz), 72.97, 68.71, 61.96, 41.01, 40.25, 32.13, 28.26, 25.54, 23.63.

^{19}F NMR (471 MHz, CDCl$_3$) δ -62.01 (s, 3F).

IR (film) ν_{max} 2937.74, 2862.20, 2231.66, 1777.7, 1719.35, 1613.00, 1504.14, 1435.93, 1406.47, 1372.91, 1311.63, 1175.72, 1133.85, 1053.56, 1028.08, 894.19, 839.49, 761.88, 736.50, 697.79, 674.55, 635.39, 615.10, 598.31, 571.29, 556.70, 534.11, 479.82, 438.41 cm^{-1}.

HRMS (ESI-TOF) *m/z* calcd. for $C_{28}H_{30}BrF_3N_3O_3^+$ ($[M+H]^+$) 592.1417, found 592.1416.

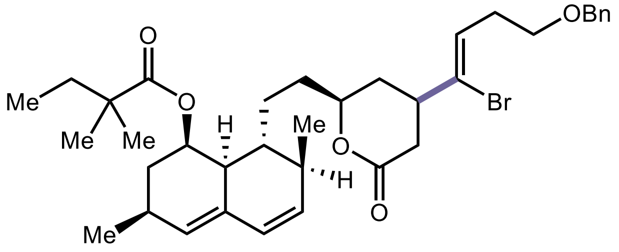

(1*R*,3*S*,7*S*,8*R*,8a*S*)-8-(2-((2*S*,4*R/S*)-4-((*Z*)-4-(benzyloxy)-1-bromobut-1-en-1-yl)-6-oxotetrahydro-2H-pyran-2-yl)ethyl)-3,7-dimethyl-1,2,3,7,8,8a-hexahydronaphthalen-1-yl 2,2-dimethylbutanoate (49)

The title compound was prepared according to **General Procedure B** using **NHC** (276.7 mg, 0.70 mmol, 1.4 equiv.), Simvastatin (272.1 mg, 0.65 mmol, 1.3 equiv.), pyridine (56.6 µL, 0.70 mmol, 1.4 equiv.), and *t*-BuOMe (5 mL) for **Stock A** and Ir[(ppy)$_2$(dtbbpy)]PF$_6$ (6.9 mg, 7.5 µmol, 0.015 equiv.), NiBr$_2$(dtbbpy) (24.3 mg, 50.0 µmol, 0.10 equiv.), quinuclidine (77.8 mg, 0.70 mmol, 1.4 equiv.), phthalimide (73.6 mg, 0.50 mmol, 1.0 equiv.), **52-DiBr** (152.0 mg, 0.50 mmol, 1.0 equiv.), and 1,2-dichlorobenzene (10 mL) for **Stock B**. The crude mixture was concentrated via *Genevac* then purified by automated flash chromatography (25 g high performance silica column, 0-25% ethyl acetate/hexanes gradient) to yield impure product. This was further purified by preparative HPLC (30-70% MeCN in water with a 0.1% NH_4OH modifier) to provide the desired compound as a viscous yellow oil (111.2 mg, 0.174 mmol, 35% yield). The product was isolated in a >20:1 *Z:E* ratio of diastereomers (configuration determined by vinylic NOE correlations).

^{1}H NMR (500 MHz, CDCl$_3$) δ 7.33 (td, J = 15.6, 7.4 Hz, 5H), 5.98 (d, J = 9.6 Hz, 1H), 5.91 (dt, J = 21.7, 6.5 Hz, 1H), 5.77 (t, J = 6.9 Hz, 1H), 5.51 (s, 1H), 5.37 – 5.30 (m, 1H), 4.52 (s, 2H), 4.25 (dtd, J = 46.9, 8.6, 4.4 Hz, 1H), 3.53 (td, J = 6.4, 2.3 Hz, 2H), 2.92 (dtt, J = 61.6, 10.8, 4.8 Hz, 1H), 2.81 – 2.67 (m, 1H), 2.60 – 2.52 (m, 1H), 2.52 – 2.46 (m, 2H), 2.46 – 2.39 (m, 1H), 2.35 (q, J =

6.0 Hz, 1H), 2.28 – 2.21 (m, 1H), 2.01 – 1.93 (m, 3H), 1.88 – 1.61 (m, 3H), 1.59 – 1.52 (m, 2H), 1.44 (ddt, J = 24.9, 12.0, 5.9 Hz, 2H), 1.27 (dd, J = 13.6, 7.3 Hz, 1H), 1.12 (d, J = 4.3 Hz, 6H), 1.08 (dd, J = 7.4, 2.0 Hz, 3H), 0.88 (d, J = 7.0 Hz, 3H), 0.82 (t, J = 7.4 Hz, 3H).

^{13}C NMR (126 MHz, CDCl$_3$) δ 177.83, 177.80, 171.74, 170.21, 138.32, 132.98, 132.97, 131.76, 131.67, 131.64, 131.15, 129.88, 129.87, 128.56, 128.54, 127.83, 127.82, 127.80, 127.12, 126.73, 80.27, 78.12, 73.08, 73.06, 68.39, 68.34, 68.17, 68.06, 43.13, 43.11, 42.24, 39.69, 37.60, 37.51, 36.83, 35.29, 34.81, 33.75, 33.24, 33.17, 33.14, 32.87, 32.83, 31.96, 30.80, 30.77, 27.43, 27.42, 24.94, 24.87, 24.79, 23.16, 23.13, 14.04, 14.02, 9.52, 9.51.

IR (film) ν_{max} 3015.01, 2958.42, 2922.88, 2867.87, 1712.73, 1452.58, 1388.14, 1361.83, 1310.28, 1237.18, 1157.73, 1096.35, 1055.43, 1027.26, 1009.97, 979.79, 944.76, 860.66, 791.48, 736.60, 697.22, 655.68, 620.16, 582.25, 521.20, 455.83, 430.88, 421.81 cm^{-1}.

HRMS (ESI-TOF) *m/z* calcd. for $C_{36}H_{49}BrNaO_5^+$ ([M+Na]$^+$) 663.2656, found 663.2654.

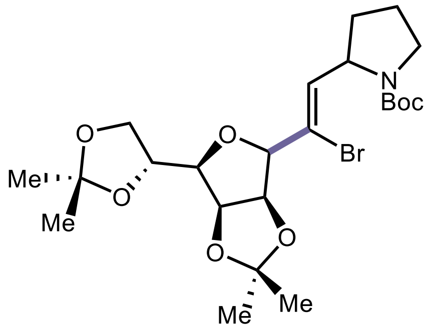

***tert*-butyl 2-((*Z*)-2-bromo-2-((3a*S*,4*R*,6*R*,6a*S*)-6-((*R*)-2,2-dimethyl-1,3-dioxolan-4-yl)-2,2-dimethyltetrahydrofuro[3,4-*d*][1,3]dioxol-4-yl)vinyl)pyrrolidine-1-carboxylate (50)**

The title compound was prepared according to **General Procedure B** using **NHC** (276.7 mg, 0.70 mmol, 1.4 equiv.), 2,3:5,6-di-O-isopropylidene-α-D-mannofuranose (169.2 mg, 0.65 mmol, 1.3 equiv.), pyridine (56.6 μL, 0.70 mmol, 1.4 equiv.), and *t*-BuOMe (5 mL) for **Stock A** and Ir[(ppy)-$_2$(dtbbpy)]PF$_6$ (6.9 mg, 7.5 μmol, 0.015 equiv.), NiBr$_2$(dtbbpy) (24.3 mg, 50.0 μmol, 0.10 equiv.), quinuclidine (77.8 mg, 0.70 mmol, 1.4 equiv.), phthalimide (73.6 mg, 0.50 mmol, 1.0 equiv.), **64-DiBr** (177.5 mg, 0.50 mmol, 1.0 equiv.), and 1,2-dichlorobenzene (10 mL) for **Stock B**. The crude mixture was concentrated via *Genevac* then purified by automated flash chromatography (25 g

high performance silica column, 0-25% ethyl acetate/hexanes gradient) to yield impure product. This was further purified by preparative HPLC (30-70% MeCN in water with a 0.1% NH_4OH modifier) to provide thne desired compound as a viscous clear oil (86.5 mg, 0.176 mmol, 35% yield). The product was isolated in a >20:1 *Z:E* ratio of diastereomers (configuration determined by vinylic NOE correlations).

^{1}H NMR (500 MHz, $CDCl_3$) δ 6.04 (td, *J* = 6.2, 1.6 Hz, 1H), 4.92 (dd, *J* = 6.0, 1.6 Hz, 1H), 4.79 (dd, *J* = 6.0, 3.7 Hz, 1H), 4.64 (t, *J* = 1.6 Hz, 1H), 4.40 (ddd, *J* = 7.3, 6.3, 4.7 Hz, 1H), 4.12 (dd, *J* = 8.7, 6.3 Hz, 1H), 4.08 – 4.03 (m, 2H), 4.03 – 3.88 (m, 2H), 2.83 (s, 3H), 1.52 (s, 3H), 1.46 (s, 9H), 1.43 (s, 3H), 1.37 (d, *J* = 5.7 Hz, 6H).

^{13}C NMR (126 MHz, $CDCl_3$) δ 155.62, 126.22, 125.63, 113.38, 109.38, 88.30, 85.27, 82.91, 81.03, 80.05, 73.53, 66.94, 49.55 (br), 48.88 (br), 34.46, 28.57, 26.99, 26.48, 25.31, 25.05.

IR (film) ν_{max} 2979.98, 2932.44, 2878.07, 1693.75, 1658.17, 1479.84, 1453.94, 1419.62, 1389.79, 1380.54, 1366.8, 1247.59, 1209.30, 1146.42, 1118.96, 1091.05, 1062.41, 1044.25, 978.26, 943.95, 878.35, 846.14, 771.20, 667.14, 623.74, 511.95, 460.68, 445.33, 430.84, 424.17, 414.43, 401.13 cm^{-1}.

HRMS (ESI-TOF) *m/z* calcd. for $C_{16}H_{27}BrNO_5^+$ ($[M-C_5H_9O_2+H]^+$) 392.1067, found 392.1068.

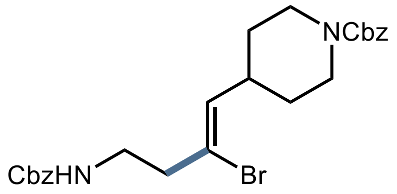

benzyl (Z)-4-(4-(((benzyloxy)carbonyl)amino)-2-bromobut-1-en-1-yl)piperidine-1-carboxylate (51)

The title compound was prepared according to **General Procedure B** using **NHC** (276.7 mg, 0.70 mmol, 1.4 equiv.), benzyl (2-hydroxyethyl)carbamate (126.9 mg, 0.65 mmol, 1.3 equiv.), pyridine (56.6 µL, 0.70 mmol, 1.4 equiv.), and *t*-BuOMe (5 mL) for **Stock A** and $Ir[(ppy)_2(dtbbpy)]PF_6$ (6.9 mg, 7.5 µmol, 0.015 equiv.), $NiBr_2(dtbbpy)$ (24.3 mg, 50.0 µmol, 0.10 equiv.), quinuclidine

(77.8 mg, 0.70 mmol, 1.4 equiv.), phthalimide (73.6 mg, 0.50 mmol, 1.0 equiv.), **51-DiBr** (201.6 mg, 0.5 mmol, 1.0 equiv.), and 1,2-dichlorobenzene (10 mL) for **Stock B**. The crude mixture was concentrated via *Genevac* then purified by automated flash chromatography (25 g high performance silica column, 0-25% ethyl acetate/hexanes gradient) to yield impure product. This was further purified by preparative HPLC (30-70% MeCN in water with a 0.1% NH_4OH modifier) to provide the desired compound as a yellow oil (146.3 mg, 0.292 mmol, 58% yield). The product was isolated in a >20:1 *Z:E* ratio of diastereomers (configuration determined by vinylic NOE correlations).

^{1}H NMR (500 MHz, CDCl$_3$) δ 7.40 – 7.27 (m, 10H), 5.51 (d, *J* = 8.4 Hz, 1H), 5.13 (s, 2H), 5.09 (s, 2H), 4.78 (t, *J* = 6.3 Hz, 1H), 4.13 (s, br, 2H), 3.39 (q, *J* = 6.3 Hz, 2H), 2.85 (s, 2H), 2.61 (t, *J* = 6.4 Hz, 2H), 2.55 (dtd, *J* = 11.5, 7.9, 4.1 Hz, 1H), 1.73 – 1.60 (m, 2H), 1.33 – 1.17 (m, 2H).

^{13}C NMR (126 MHz, CDCl$_3$) δ 156.37, 155.38, 137.01, 136.60, 134.87, 128.69, 128.64, 128.36, 128.34, 128.13, 128.02, 123.86, 67.20, 66.87, 43.75, 41.59, 39.27, 38.77, 30.67.

IR (film) ν_{max} 3332.15, 3031.71, 2937.47, 1682.33, 1527.09, 1497.59, 1469.07, 1429.48, 1362.95, 1313.55, 1250.15, 1219.26, 1141.16, 1087.77, 1002.46, 969.80, 910.10, 824.87, 734.21, 695.31, 604.85, 554.39, 456.59 cm^{-1}.

HRMS (ESI-TOF) *m/z* calcd. for $C_{25}H_{29}BrN_2NaO_4^+$ ([M+Na]$^+$) 523.1203, found 523.1206.

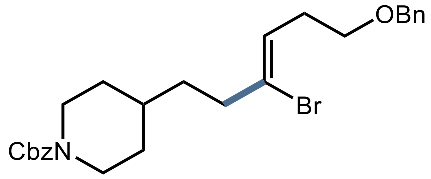

benzyl (*Z*)-4-(6-(benzyloxy)-3-bromohex-3-en-1-yl)piperidine-1-carboxylate (52)

The title compound was prepared according to **General Procedure B** using **NHC** (276.7 mg, 0.70 mmol, 1.4 equiv.), benzyl 4-(2-hydroxyethyl)piperidine-1-carboxylate (171.1 mg, 0.65 mmol, 1.3 equiv.), pyridine (56.6 μL, 0.70 mmol, 1.4 equiv.), and *t*-BuOMe (5 mL) for **Stock A** and Ir[(ppy)$_2$(dtbbpy)]PF_6 (6.9 mg, 7.5 μmol, 0.015 equiv.), $NiBr_2$(dtbbpy) (24.3 mg, 50.0 μmol, 0.10

equiv.), quinuclidine (77.8 mg, 0.70 mmol, 1.4 equiv.), phthalimide (73.6 mg, 0.50 mmol, 1.0 equiv.), **52-DiBr** (160.0 mg, 0.50 mmol, 1.0 equiv.), and 1,2-dichlorobenzene (10 mL) for **Stock B**. The crude mixture was concentrated via *Genevac* then purified by automated flash chromatography (25 g high performance silica column, 0-25% ethyl acetate/hexanes gradient) to yield impure product. This was further purified by preparative HPLC (30-70% MeCN in water with a 0.1% NH_4OH modifier) to provide the desired compound as a viscous yellow oil (183.7 mg, 0.378 mmol, 76% yield). The product was isolated in a >20:1 *Z:E* ratio of diastereomers (configuration determined by vinylic NOE correlations).

^{1}H NMR (500 MHz, $CDCl_3$) δ 7.40 – 7.25 (m, 11H), 5.74 (tt, *J* = 6.7, 1.1 Hz, 1H), 5.12 (s, 2H), 4.52 (s, 2H), 4.15 (s, 2H), 3.52 (t, *J* = 6.5 Hz, 2H), 2.80 – 2.69 (m, 2H), 2.51 – 2.41 (m, 4H), 1.67 (d, *J* = 13.1 Hz, 2H), 1.55 – 1.46 (m, 2H), 1.41 (ddq, *J* = 14.4, 6.9, 3.6 Hz, 1H), 1.12 (qd, *J* = 12.4, 4.2 Hz, 2H).

^{13}C NMR (126 MHz, $CDCl_3$) δ 155.41, 138.46, 137.12, 129.78, 128.60, 128.53, 128.05, 127.96, 127.78, 127.76, 125.44, 72.98, 68.75, 67.08, 44.28, 38.85, 34.96, 34.91, 32.15, 32.05 (br).

IR (film) ν_{max} 3030.14, 2920.54, 2852.77, 1694.57, 1496.49, 1468.23, 1427.61, 1361.88, 1275.25, 1231.59, 1174.12, 1073.91, 1026.22, 963.89, 910.07, 846.91, 733.43, 695.60, 603.86, 555.81, 457.83 cm^{-1}.

HRMS (ESI-TOF) *m/z* calcd. for $C_{26}H_{32}BrNNaO_3^+$ ($[M+Na]^+$) 508.1458, found 508.1455.

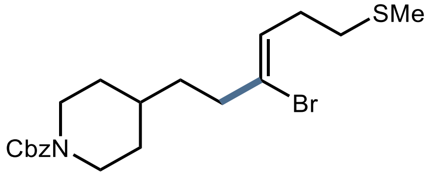

benzyl (*Z*)-4-(3-bromo-6-(methylthio)hex-3-en-1-yl)piperidine-1-carboxylate (53)

The title compound was prepared according to **General Procedure B** using **NHC** (276.7 mg, 0.70 mmol, 1.4 equiv.), benzyl 4-(2-hydroxyethyl)piperidine-1-carboxylate (171.1 mg, 0.65 mmol, 1.3 equiv.), pyridine (56.6 µL, 0.70 mmol, 1.4 equiv.), and *t*-BuOMe (5 mL) for **Stock A** and Ir[(ppy)-

$_2$(dtbbpy)]PF_6 (6.9 mg, 7.5 μmol, 0.015 equiv.), $NiBr_2$(dtbbpy) (24.3 mg, 50.0 μmol, 0.10 equiv.), quinuclidine (77.8 mg, 0.70 mmol, 1.4 equiv.), phthalimide (73.6 mg, 0.50 mmol, 1.0 equiv.), **53-DiBr** (130.0 mg, 0.50 mmol, 1.0 equiv.), and 1,2-dichlorobenzene (10 mL) for **Stock B**. The crude mixture was concentrated via *Genevac* then purified by automated flash chromatography (25 g high performance silica column, 0-25% ethyl acetate/hexanes gradient) to yield impure product. This was further purified by preparative HPLC (30-70% MeCN in water with a 0.1% NH_4OH modifier) to provide the desired compound as a viscous brown oil (96.8 mg, 0.227 mmol, 45% yield). The product was isolated in a >20:1 *Z:E* ratio of diastereomers (configuration determined by vinylic NOE correlations).

^{1}H NMR (500 MHz, $CDCl_3$) δ 7.42 – 7.27 (m, 5H), 5.72 (tt, *J* = 6.9, 1.2 Hz, 1H), 5.12 (s, 2H), 4.16 (d, *J* = 12.9 Hz, 2H), 2.76 (t, *J* = 13.4 Hz, 2H), 2.56 (td, *J* = 7.1, 1.0 Hz, 2H), 2.49 – 2.43 (m, 4H), 2.12 (s, 3H), 1.67 (d, *J* = 12.9 Hz, 2H), 1.51 (q, *J* = 7.0 Hz, 2H), 1.48 – 1.38 (m, 1H), 1.13 (qd, *J* = 12.4, 4.3 Hz, 2H).

^{13}C NMR (126 MHz, $CDCl_3$) δ 155.41, 137.11, 129.75, 128.60, 128.05, 127.96, 126.74, 67.09, 44.30, 38.80, 34.89, 34.87, 32.99, 32.05, 30.96, 15.50.

IR (film) ν_{max} 2914.75, 2850.27, 1693.79, 1497.09, 1468.23, 1426.48, 1362.75, 1274.70, 1231.67, 1197.61, 1122.08, 1074.25, 1020.71, 963.22, 919.87, 837.69, 785.83, 762.55, 733.06, 696.09, 603.69, 546.26, 457.72 cm^{-1}.

HRMS (ESI-TOF) *m/z* calcd. for $C_{20}H_{29}BrNO_2S^+$ ($[M+H]^+$) 426.1097, found 426.1098.

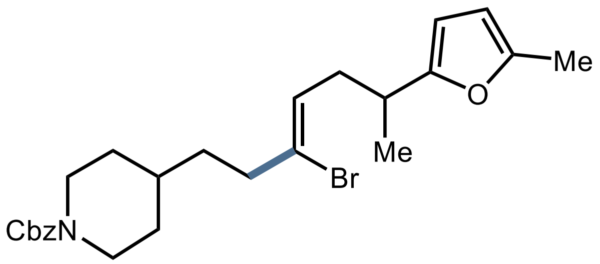

benzyl (*Z*)-4-(3-bromo-6-(5-methylfuran-2-yl)hept-3-en-1-yl)piperidine-1-carboxylate (54)

The title compound was prepared according to **General Procedure B** using **NHC** (276.7 mg, 0.70 mmol, 1.4 equiv.), benzyl 4-(2-hydroxyethyl)piperidine-1-carboxylate (171.1 mg, 0.65 mmol, 1.3 equiv.), pyridine (56.6 µL, 0.70 mmol, 1.4 equiv.), and *t*-BuOMe (5 mL) for **Stock A** and Ir[(ppy)-$_2$(dtbbpy)]PF_6 (6.9 mg, 7.5 µmol, 0.015 equiv.), $NiBr_2$(dtbbpy) (24.3 mg, 50.0 µmol, 0.10 equiv.), quinuclidine (77.8 mg, 0.70 mmol, 1.4 equiv.), phthalimide (73.6 mg, 0.50 mmol, 1.0 equiv.), **54-DiBr** (154.0 mg, 0.50 mmol, 1.0 equiv.), and 1,2-dichlorobenzene (10 mL) for **Stock B**. The crude mixture was concentrated via *Genevac* then purified by automated flash chromatography (25 g high performance silica column, 0-25% ethyl acetate/hexanes gradient) to yield impure product. This was further purified by preparative HPLC (30-70% MeCN in water with a 0.1% NH_4OH modifier) to provide the desired compound as a brown oil (144.3 mg, 0.304 mmol, 61% yield). The product was isolated in a >20:1 *Z:E* ratio of diastereomers (configuration determined by vinylic NOE correlations).

^{1}H NMR (500 MHz, $CDCl_3$) δ 7.41 – 7.26 (m, 5H), 5.84 (d, *J* = 1.3 Hz, 2H), 5.60 (t, *J* = 6.7 Hz, 1H), 5.12 (s, 2H), 4.15 (s, 2H), 2.88 (h, *J* = 6.9 Hz, 1H), 2.73 (t, *J* = 13.0 Hz, 2H), 2.55 – 2.46 (m, 1H), 2.46 – 2.35 (m, 3H), 2.25 (s, 3H), 1.66 (d, 2H), 1.53 – 1.44 (m, 2H), 1.39 (dqt, *J* = 12.4, 8.8, 4.4 Hz, 1H), 1.22 (d, *J* = 7.0 Hz, 3H), 1.16 – 1.06 (m, 2H).

^{13}C NMR (126 MHz, $CDCl_3$) δ 157.89, 155.41, 150.46, 137.11, 129.35, 128.60, 128.05, 127.96, 126.84, 105.76, 104.55, 67.08, 44.28, 38.79, 37.34, 34.85, 34.69, 32.73, 32.01 (br), 18.93, 13.68.

IR (film) ν_{max} 2920.83, 2852.10, 1696.05, 1565.49, 1497.40, 1468.04, 1428.02, 1362.89, 1300.46, 1275.64, 1232.68, 1198.30, 1173.36, 1126.73, 1073.91, 1017.50, 961.64, 939.31, 911.04, 866.32, 780.59, 763.24, 732.43, 696.20, 603.88, 556.09, 457.70, 405.84 cm^{-1}.

HRMS (ESI-TOF) *m/z* calcd. for $C_{25}H_{33}BrNO_3^+$ ($[M+H]^+$) 474.1638, found 474.1638.

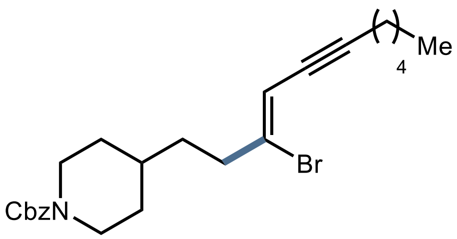

benzyl (*Z*)-4-(3-bromoundec-3-en-5-yn-1-yl)piperidine-1-carboxylate (55)

The title compound was prepared according to **General Procedure B** using **NHC** (251.2 mg, 0.64 mmol, 1.4 equiv.), benzyl 4-(2-hydroxyethyl)piperidine-1-carboxylate (155.4 mg, 0.59 mmol, 1.3 equiv.), pyridine (51.4 μL, 0.64 mmol, 1.4 equiv.), and *t*-BuOMe (4.5 mL) for **Stock A** and Ir[(ppy)$_2$(dtbbpy)]PF$_6$ (6.3 mg, 6.8 μmol, 0.015 equiv.), NiBr$_2$(dtbbpy) (22.1 mg, 45.4 μmol, 0.10 equiv.), quinuclidine (70.6 mg, 0.64 mmol, 1.4 equiv.), phthalimide (66.8 mg, 0.454 mmol, 1.0 equiv.), **55-DiBr** (127.1 mg, 0.454 mmol, 1.0 equiv.), and 1,2-dichlorobenzene (9.1 mL) for **Stock B**. The crude mixture was concentrated via *Genevac* then purified by automated flash chromatography (25 g high performance silica column, 0-25% ethyl acetate/hexanes gradient) to yield impure product. This was further purified by preparative HPLC (30-70% MeCN in water with a 0.1% NH$_4$OH modifier) to provide the desired compound as a light brown oil (127.9 mg, 0.269 mmol, 59% yield). The product was isolated in a >20:1 *Z:E* ratio of diastereomers (configuration determined by vinylic NOE correlations).

^{1}H NMR (500 MHz, CDCl$_3$) δ 7.41 – 7.29 (m, 5H), 5.89 (s, 1H), 5.12 (s, 2H), 4.16 (s, 2H), 2.75 (t, *J* = 12.7 Hz, 2H), 2.51 (t, *J* = 7.3 Hz, 2H), 2.34 (td, *J* = 7.1, 2.1 Hz, 2H), 1.66 (d, *J* = 12.6 Hz, 2H), 1.54 (dq, *J* = 20.9, 7.3 Hz, 4H), 1.50 – 1.39 (m, 3H), 1.39 – 1.26 (m, 2H), 1.12 (qd, *J* = 12.2, 4.1 Hz, 2H), 0.90 (t, *J* = 7.3 Hz, 3H).

^{13}C NMR (126 MHz, CDCl$_3$) δ 155.39, 137.38, 137.08, 128.60, 128.05, 127.96, 111.20, 95.95, 77.86, 67.10, 44.23, 38.36, 34.96, 34.82, 31.99, 31.14, 28.38, 22.32, 19.73, 14.12.

IR (film) ν_{max} 2928.98, 2856.51, 1695.56, 1497.33, 1467.19, 1427.13, 1363.21, 1275.50, 1229.67, 1193.72, 1166.79, 1124.47, 1073.17, 1022.81, 963.86, 909.63, 823.29, 763.14, 731.54, 696.21, 603.63, 546.58, 458.86 cm^{-1}.

HRMS (ESI-TOF) *m/z* calcd. for $C_{24}H_{32}BrNNaO_2^+$ ([M+Na]$^+$) 468.1509, found 468.1506.

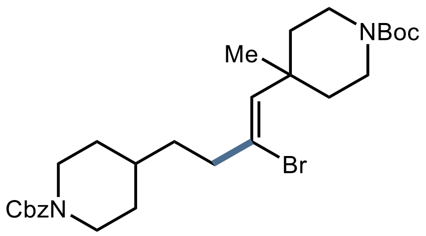

***tert*-butyl (*Z*)-4-(4-(1-((benzyloxy)carbonyl)piperidin-4-yl)-2-bromobut-1-en-1-yl)-4-methylpiperidine-1-carboxylate (56)**

The title compound was prepared according to **General Procedure B** using **NHC** (276.7 mg, 0.70 mmol, 1.4 equiv.), benzyl 4-(2-hydroxyethyl)piperidine-1-carboxylate (171.1 mg, 0.65 mmol, 1.3 equiv.), pyridine (56.6 µL, 0.70 mmol, 1.4 equiv.), and *t*-BuOMe (5 mL) for **Stock A** and Ir[(ppy)-$_2$(dtbbpy)]PF_6 (6.9 mg, 7.5 µmol, 0.015 equiv.), $NiBr_2$(dtbbpy) (24.3 mg, 50.0 µmol, 0.10 equiv.), quinuclidine (77.8 mg, 0.70 mmol, 1.4 equiv.), phthalimide (73.6 mg, 0.50 mmol, 1.0 equiv.), **56-DiBr** (191.6 mg, 0.5 mmol, 1.0 equiv.), and 1,2-dichlorobenzene (10 mL) for **Stock B**. The crude mixture was concentrated via *Genevac* then purified by automated flash chromatography (25 g high performance silica column, 0-25% ethyl acetate/hexanes gradient) to yield impure product. This was further purified by preparative HPLC (30-70% MeCN in water with a 0.1% NH_4OH modifier) to provide the desired compound as an oily brown solid (110.6 mg, 0.202 mmol, 40% yield). The product was isolated in a >20:1 *Z:E* ratio of diastereomers (configuration determined by vinylic NOE correlations).

^{1}H NMR (500 MHz, $CDCl_3$) δ 7.38 – 7.29 (m, 5H), 5.70 (s, 1H), 5.12 (s, 2H), 4.26 – 4.07 (m, 2H), 3.73 (dt, *J* = 13.9, 4.2 Hz, 2H), 2.97 (ddd, *J* = 13.7, 10.8, 2.8 Hz, 2H), 2.74 (t, *J* = 12.6 Hz, 2H), 2.44 (t, *J* = 6.4 Hz, 2H), 2.04 – 1.96 (m, 3H), 1.67 (d, *J* = 12.9 Hz, 2H), 1.50 (q, *J* = 7.1 Hz, 2H), 1.45 (s, 9H), 1.36 (ddd, *J* = 14.2, 10.9, 4.0 Hz, 2H), 1.21 (s, 3H), 1.14 (qt, *J* = 11.8, 6.2 Hz, 2H).

^{13}C NMR (126 MHz, $CDCl_3$) δ 155.41, 155.13, 137.09, 133.75, 128.61, 128.06, 127.97, 126.73, 79.47, 67.11, 44.31, 41.12, 40.68 (br), 37.63, 35.49, 35.28, 34.96, 32.07, 28.61, 25.65.

IR (film) ν_{max} 2924.90, 2852.12, 1689.09, 1421.96, 1364.12, 1276.46, 1243.63, 1159.57, 1119.65, 1086.09, 1018.33, 965.42, 927.42, 862.13, 828.42, 763.19, 696.50, 592.28, 541.71, 459.44 cm^{-1}.

HRMS (ESI-TOF) *m/z* calcd. for $C_{28}H_{41}BrN_2NaO_4^+$ ($[M+Na]^+$) 571.2142, found 571.2141.

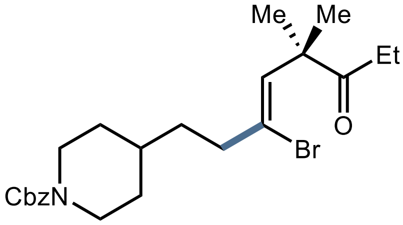

benzyl (*Z*)-4-(3-bromo-5,5-dimethyl-6-oxooct-3-en-1-yl)piperidine-1-carboxylate (57)

The title compound was prepared according to **General Procedure B** using **NHC** (221.4 mg, 0.56 mmol, 1.4 equiv.), benzyl 4-(2-hydroxyethyl)piperidine-1-carboxylate (136.9 mg, 0.52 mmol, 1.3 equiv.), pyridine (56.6 µL, 0.56 mmol, 1.4 equiv.), and *t*-BuOMe (4 mL) for **Stock A** and Ir[(ppy)-$_2$(dtbbpy)]PF_6 (5.5 mg, 6 µmol, 0.015 equiv.), $NiBr_2$(dtbbpy) (19.4 mg, 40.0 µmol, 0.10 equiv.), quinuclidine (62.3 mg, 0.56 mmol, 1.4 equiv.), phthalimide (58.9 mg, 0.40 mmol, 1.0 equiv.), **57-DiBr** (113.6 mg, 0.4 mmol, 1.0 equiv.), and 1,2-dichlorobenzene (8 mL) for **Stock B**. The crude mixture was concentrated via *Genevac* then purified by automated flash chromatography (25 g high performance silica column, 0-25% ethyl acetate/hexanes gradient) to yield impure product. This was further purified by preparative HPLC (30-70% MeCN in water with a 0.1% NH_4OH modifier) to provide the desired compound as a yellow oil (112.5 mg, 0.247 mmol, 62% yield). The product was isolated in a >20:1 *Z:E* ratio of diastereomers (configuration determined by vinylic NOE correlations).

^{1}H NMR (500 MHz, $CDCl_3$) 7.40 – 7.27 (m, 5H), 5.95 (s, 1H), 5.12 (s, 2H), 4.17 (s, br, 2H), 2.75 (s, br, 2H), 2.53 (q, J = 7.3 Hz, 2H), 2.44 (t, J = 7.6 Hz, 2H), 1.67 (d, J = 12.9 Hz, 2H), 1.50 (q, J = 7.3 Hz, 2H), 1.42 (th, J = 10.5, 3.4 Hz, 1H), 1.29 (s, 6H), 1.19 – 1.10 (m, 2H), 1.07 (t, J = 7.2 Hz, 3H).

^{13}C NMR (126 MHz, $CDCl_3$) δ 213.33, 155.41, 137.07, 134.42, 128.95, 128.61, 128.07, 127.97, 67.12, 50.15, 44.30, 39.73, 35.08, 35.05, 32.03 (br), 31.16, 25.58, 8.70.

IR (film) ν_{max} 2972.08, 2933.24, 2852.45, 1695.01, 1497.49, 1467.01, 1427.54, 1382.11, 1361.42, 1346.41, 1303.50, 1275.62, 1235.71, 1192.88, 1173.77, 1151.86, 1121.59, 1095.88, 1023.13, 967.75, 936.22, 867.03, 823.35, 785.51, 762.88, 733.72, 696.53, 604.36, 555.65, 458.77, 413.24 cm^{-1}.

HRMS (ESI-TOF) *m/z* calcd. for $C_{23}H_{32}BrNNaO_3^+$ ($[M+Na]^+$) 472.1458, found 472.1456.

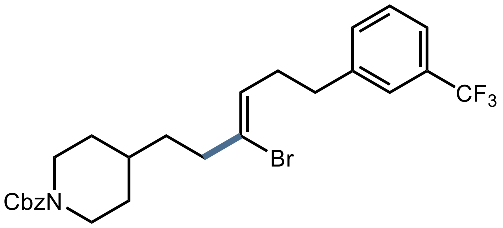

benzyl (*Z*)-4-(3-bromo-6-(3-(trifluoromethyl)phenyl)hex-3-en-1-yl)piperidine-1-carboxylate (58)

The title compound was prepared according to **General Procedure B** using **NHC** (276.7 mg, 0.70 mmol, 1.4 equiv.), benzyl 4-(2-hydroxyethyl)piperidine-1-carboxylate (171.1 mg, 0.65 mmol, 1.3 equiv.), pyridine (56.6 µL, 0.70 mmol, 1.4 equiv.), and *t*-BuOMe (5 mL) for Stock A and Ir[(ppy)-$_2$(dtbbpy)]PF_6 (6.9 mg, 7.5 µmol, 0.015 equiv.), $NiBr_2$(dtbbpy) (24.3 mg, 50.0 µmol, 0.10 equiv.), quinuclidine (77.8 mg, 0.70 mmol, 1.4 equiv.), phthalimide (73.6 mg, 0.50 mmol, 1.0 equiv.), **58-DiBr** (179.0 mg, 0.5 mmol, 1.0 equiv.), and 1,2-dichlorobenzene (10 mL) for **Stock B**. The crude mixture was concentrated via *Genevac* then purified by automated flash chromatography (25 g high performance silica column, 0-25% ethyl acetate/hexanes gradient) to yield impure product. This was further purified by preparative HPLC (30-70% MeCN in water with a 0.1% NH_4OH modifier) to provide the desired compound as a brown oil (178.9 mg, 0.341 mmol, 68% yield). The product was isolated in a >20:1 *Z:E* ratio of diastereomers (configuration determined by vinylic NOE correlations).

^{1}H NMR (500 MHz, $CDCl_3$) δ 7.49 – 7.28 (m, 9H), 5.63 (t, *J* = 6.8 Hz, 1H), 5.13 (s, 2H), 4.26 – 4.03 (m, 2H), 2.80 – 2.66 (m, 4H), 2.50 (q, 2H), 2.43 (t, *J* = 7.6 Hz, 2H), 1.66 (d, *J* = 12.1 Hz, 2H), 1.47 (q, *J* = 7.2 Hz, 2H), 1.38 (tp, *J* = 10.8, 3.3 Hz, 1H), 1.11 (qd, *J* = 12.3, 4.2 Hz, 2H).

^{13}C NMR (126 MHz, CDCl$_3$) δ 155.40, 142.26, 137.10, 131.97 (d, J = 1.5 Hz), 130.76 (q, J = 31.9 Hz), 129.70, 128.90, 128.60, 128.05, 127.96, 127.06, 125.44 (t, J = 272.4 Hz), 125.29 (q, J = 3.8 Hz), 123.05 (q, J = 3.9 Hz), 67.08, 44.24, 38.77, 34.97, 34.88, 34.43, 32.68, 32.00 (br).

^{19}F NMR (471 MHz, CDCl$_3$) δ -62.52 (s, 3F).

IR (film) ν_{max} 2930.30, 2853.63, 1695.08, 1497.13, 1468.85, 1428.79, 1325.71, 1275.77, 1232.97, 1196.77, 1160.96, 1119.74, 1096.32, 1072.00, 1024.05, 963.90, 902.54, 799.00, 763.32, 748.94, 734.06, 698.44, 659.39, 603.75, 556.41, 458.03, 405.22 cm^{-1}.

HRMS (ESI-TOF) *m/z* calcd. for $C_{26}H_{30}BrF_3NO_2^+$ ([M+H]$^+$) 524.1407, found 524.1403.

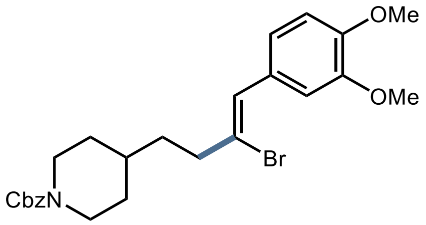

benzyl (*Z*)-4-(3-bromo-4-(3,4-dimethoxyphenyl)but-3-en-1-yl)piperidine-1-carboxylate (59)

The title compound was prepared according to **General Procedure B** using **NHC** (276.7 mg, 0.70 mmol, 1.4 equiv.), benzyl 4-(2-hydroxyethyl)piperidine-1-carboxylate (171.1 mg, 0.65 mmol, 1.3 equiv.), pyridine (56.6 µL, 0.70 mmol, 1.4 equiv.), and *t*-BuOMe (5 mL) for **Stock A** and Ir[(ppy)-$_2$(dtbbpy)]PF_6 (6.9 mg, 7.5 µmol, 0.015 equiv.), $NiBr_2$(dtbbpy) (24.3 mg, 50.0 µmol, 0.10 equiv.), quinuclidine (77.8 mg, 0.70 mmol, 1.4 equiv.), phthalimide (73.6 mg, 0.50 mmol, 1.0 equiv.), **59-DiBr** (161.0 mg, 0.5 mmol, 1.0 equiv.), and 1,2-dichlorobenzene (10 mL) for **Stock B**. The crude mixture was concentrated via *Genevac* then purified by automated flash chromatography (25 g high performance silica column, 0-25% ethyl acetate/hexanes gradient) to yield impure product. This was further purified by preparative HPLC (30-70% MeCN in water with a 0.1% NH_4OH modifier) to provide the desired compound as a yellow oil (136.5 mg, 0.280 mmol, 56% yield). The product was isolated in a >20:1 *Z:E* ratio of diastereomers (configuration determined by vinylic NOE correlations).

^{1}H NMR (500 MHz, CDCl$_3$) δ 7.39 – 7.29 (m, 5H), 7.27 (d, J = 2.0 Hz, 1H), 7.10 (dd, J = 8.4, 2.0 Hz, 1H), 6.85 (d, J = 8.4 Hz, 1H), 6.67 (s, 1H), 5.13 (s, 2H), 4.18 (s, 2H), 3.89 (d, J = 3.9 Hz, 6H), 2.78 (s, 2H), 2.62 (t, 2H), 1.74 (s, br, 2H), 1.62 (q, J = 7.3 Hz, 2H), 1.49 (ddh, J = 14.4, 7.0, 3.4 Hz, 1H), 1.24 – 1.10 (m, 2H).

^{13}C NMR (126 MHz, CDCl$_3$) δ 155.42, 148.73, 148.49, 137.10, 128.73, 128.61, 128.06, 127.97, 127.24, 125.93, 122.17, 111.91, 110.79, 67.10, 56.00, 44.29, 40.65, 35.29, 35.04, 32.02 (br).

IR (film) ν_{max} 3057.50, 2994.04, 2910.63, 2831.64, 1689.50, 1600.19, 1581.78, 1511.01, 1462.89, 1418.11, 1362.22, 1305.04, 1270.30, 1227.63, 1196.74, 1172.57, 1158.83, 1140.24, 1120.91, 1074.05, 1022.27, 963.62, 867.35, 804.21, 785.23, 762.13, 734.10, 696.13, 638.21, 603.50, 537.19, 460.22, 410.82 cm^{-1}.

HRMS (ESI-TOF) *m/z* calcd. for $C_{25}H_{30}BrNNaO_4^+$ ($[M+Na]^+$) 510.1250, found 510.1250.

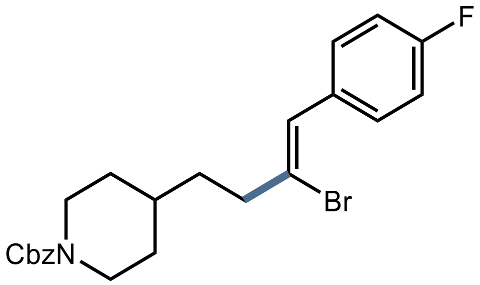

benzyl (Z)-4-(3-bromo-4-(4-fluorophenyl)but-3-en-1-yl)piperidine-1-carboxylate (60)

The title compound was prepared according to **General Procedure B** using **NHC** (276.7 mg, 0.70 mmol, 1.4 equiv.), benzyl 4-(2-hydroxyethyl)piperidine-1-carboxylate (171.1 mg, 0.65 mmol, 1.3 equiv.), pyridine (56.6 µL, 0.70 mmol, 1.4 equiv.), and *t*-BuOMe (5 mL) for **Stock A** and Ir[(ppy)-$_2$(dtbbpy)]PF_6 (6.9 mg, 7.5 µmol, 0.015 equiv.), $NiBr_2$(dtbbpy) (24.3 mg, 50.0 µmol, 0.10 equiv.), quinuclidine (77.8 mg, 0.70 mmol, 1.4 equiv.), phthalimide (73.6 mg, 0.50 mmol, 1.0 equiv.), **60-DiBr** (140.0 mg, 0.5 mmol, 1.0 equiv.), and 1,2-dichlorobenzene (10 mL) for **Stock B**. The crude mixture was concentrated via *Genevac* then purified by automated flash chromatography (25 g high performance silica column, 0-25% ethyl acetate/hexanes gradient) to yield impure product.

This was further purified by preparative HPLC (30-70% MeCN in water with a 0.1% NH_4OH modifier) to provide the desired compound as a pale yellow oil (123.9 mg, 0.278 mmol, 56% yield). The product was isolated in a >20:1 *Z:E* ratio of diastereomers (configuration determined by vinylic NOE correlations).

^{1}H NMR (500 MHz, $CDCl_3$) δ 7.57 – 7.50 (m, 2H), 7.39 – 7.29 (m, 5H), 7.03 (t, *J* = 8.7 Hz, 2H), 6.69 (s, 1H), 5.13 (s, 2H), 4.19 (s, br, 2H), 2.78 (t, *J* = 12.3 Hz, 2H), 2.62 (t, *J* = 7.2 Hz, 2H), 1.72 (d, *J* = 13.5 Hz, 2H), 1.62 (q, *J* = 7.1 Hz, 2H), 1.50 (dtq, *J* = 14.6, 6.9, 3.5 Hz, 1H), 1.18 (qd, *J* = 10.7, 5.8 Hz, 2H).

^{13}C NMR (126 MHz, $CDCl_3$) δ 163.13, 161.16, 155.42, 137.09, 132.12 (d, *J* = 3.5 Hz), 130.74 (d, *J* = 8.0 Hz), 128.61, 128.07, 127.76 (d, *J* = 1.7 Hz), 126.65, 115.18 (d, *J* = 21.5 Hz), 67.12, 44.29, 40.47, 35.19, 35.07, 32.09 (br).

^{19}F NMR (471 MHz, $CDCl_3$) δ -113.62 (tt, *J* = 8.6, 5.5 Hz, 1F).

IR (film) ν_{max} 2931.15, 2851.79, 1692.52, 1601.28, 1506.27, 1468.37, 1427.19, 1362.88, 1275.16, 1226.26, 1172.36, 1159.55, 1122.88, 1084.55, 1014.06, 963.98, 910.31, 864.93, 822.85, 786.96, 762.78, 732.55, 695.98, 593.16, 548.05, 523.35, 457.07 cm^{-1}.

HRMS (ESI-TOF) *m/z* calcd. for $C_{23}H_{26}BrFNO_2^+$ ($[M+H]^+$) 446.1125, found 446.1122.

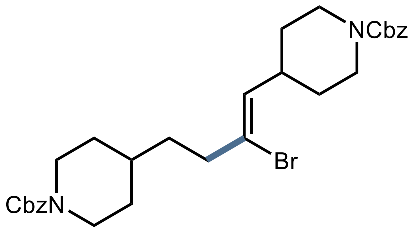

dibenzyl 4,4'-(2-bromobut-1-ene-1,4-diyl)(*Z*)-bis(piperidine-1-carboxylate) (61)

The title compound was prepared according to **General Procedure B** using **NHC** (276.7 mg, 0.70 mmol, 1.4 equiv.), benzyl 4-(2-hydroxyethyl)piperidine-1-carboxylate (171.1 mg, 0.65 mmol, 1.3 equiv.), pyridine (56.6 µL, 0.70 mmol, 1.4 equiv.), and *t*-BuOMe (5 mL) for **Stock A** and Ir[(ppy)-

$_2$(dtbbpy)]PF_6 (6.9 mg, 7.5 μmol, 0.015 equiv.), $NiBr_2$(dtbbpy) (24.3 mg, 50.0 μmol, 0.10 equiv.), quinuclidine (77.8 mg, 0.70 mmol, 1.4 equiv.), phthalimide (73.6 mg, 0.50 mmol, 1.0 equiv.), **51-DiBr** (201.6 mg, 0.5 mmol, 1.0 equiv.), and 1,2-dichlorobenzene (10 mL) for **Stock B**. The crude mixture was concentrated via *Genevac* then purified by automated flash chromatography (25 g high performance silica column, 0-25% ethyl acetate/hexanes gradient) to yield impure product. This was further purified by preparative HPLC (30-70% MeCN in water with a 0.1% NH_4OH modifier) to provide the desired compound as a viscous brown oil (170.0 mg, 0.299 mmol, 60% yield). The product was isolated in a >20:1 *Z:E* ratio of diastereomers (configuration determined by vinylic NOE correlations).

^{1}H NMR (500 MHz, $CDCl_3$) δ 7.39 – 7.28 (m, 10H), 5.44 (d, *J* = 8.4 Hz, 1H), 5.12 (d, *J* = 3.5 Hz, 4H), 4.22 – 4.09 (m, 4H), 2.86 (t, *J* = 11.9 Hz, 2H), 2.74 (ddd, *J* = 14.8, 12.6, 2.7 Hz, 2H), 2.57 (tdt, *J* = 11.8, 8.0, 3.9 Hz, 1H), 2.45 – 2.39 (m, 2H), 1.68 (td, *J* = 14.5, 7.5 Hz, 4H), 1.49 (q, *J* = 7.0 Hz, 2H), 1.40 (ddt, *J* = 10.9, 7.4, 3.7 Hz, 1H), 1.28 (qd, *J* = 12.1, 4.3 Hz, 2H), 1.13 (qd, *J* = 12.6, 4.4 Hz, 2H).

^{13}C NMR (126 MHz, $CDCl_3$) δ 155.42, 155.40, 137.10, 137.03, 132.01, 128.63, 128.61, 128.11, 128.07, 128.01, 127.98, 127.85, 67.18, 67.11, 44.29, 43.84, 38.81, 38.75, 35.03, 34.95, 32.05, 30.82.

IR (film) ν_{max} 2929.06, 2858.17, 1679.28, 1496.82, 1467.29, 1420.64, 1386.84, 1357.44, 1327.78, 1310.44, 1296.20, 1275.60, 1249.36, 1231.98, 1214.08, 1170.55, 1140.44, 1118.47, 1109.02, 1100.42, 1083.05, 1066.04, 1051.03, 1020.62, 1000.16, 981.21, 959.28, 941.55, 911.55, 864.62, 846.96, 827.35, 786.24, 777.00, 754.96, 696.74, 645.93, 622.16, 604.43, 596.47, 584.82, 551.61, 539.11, 492.16, 471.41, 462.62 cm^{-1}.

HRMS (ESI-TOF) *m/z* calcd. for $C_{30}H_{37}BrN_2NaO_4^+$ ([M+Na]$^+$) 591.1829, found 591.1830.

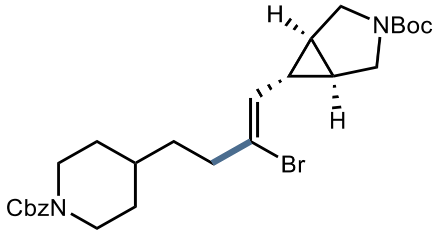

(±)-*tert*-butyl (1*R*,5*S*,6*s*)-6-((*Z*)-4-(1-((benzyloxy)carbonyl)piperidin-4-yl)-2-bromobut-1-en-1-yl)-3-azabicyclo[3.1.0]hexane-3-carboxylate (62)

The title compound was prepared according to **General Procedure B** using **NHC** (276.7 mg, 0.70 mmol, 1.4 equiv.), benzyl 4-(2-hydroxyethyl)piperidine-1-carboxylate (171.1 mg, 0.65 mmol, 1.3 equiv.), pyridine (56.6 µL, 0.70 mmol, 1.4 equiv.), and *t*-BuOMe (5 mL) for **Stock A** and Ir[(ppy)-$_2$(dtbbpy)]PF_6 (6.9 mg, 7.5 µmol, 0.015 equiv.), $NiBr_2$(dtbbpy) (24.3 mg, 50.0 µmol, 0.10 equiv.), quinuclidine (77.8 mg, 0.70 mmol, 1.4 equiv.), phthalimide (73.6 mg, 0.50 mmol, 1.0 equiv.), **62-DiBr** (183.5 mg, 0.5 mmol, 1.0 equiv.), and 1,2-dichlorobenzene (10 mL) for **Stock B**. Due to challenges in purification, the title compound was isolated only in analytical quantities for characterization. The pure material, obtained as a viscous yellow oil, was subsequently used to determine the assay yield of the crude reaction mixture by ^{1}H NMR analysis with mesitylene as an internal standard, which indicated a yield of 78%, with a >20:1 *Z:E* diastereomer ratio.

^{1}H NMR (500 MHz, $CDCl_3$) δ 7.39 – 7.28 (m, 5H), 5.17 – 5.06 (m, 3H), 4.16 (s, 2H), 3.63 (dd, *J* = 42.6, 11.0 Hz, 2H), 3.38 (t, *J* = 10.7 Hz, 2H), 2.75 (s, 2H), 2.44 – 2.37 (m, 2H), 1.66 (d, *J* = 13.0 Hz, 2H), 1.60 – 1.52 (m, 4H), 1.49 (q, *J* = 7.3 Hz, 2H), 1.45 (s, 9H), 1.12 (qd, *J* = 12.3, 4.1 Hz, 2H).

^{13}C NMR (126 MHz, $CDCl_3$) δ 155.41, 155.08, 137.10, 128.74, 128.61, 128.07, 127.97, 126.84, 79.61, 67.10, 48.40 – 48.14 (rotamers), 44.27, 38.66, 35.07, 35.00, 28.63, 25.33, 25.00 – 24.26 (rotamers).

IR (film) ν_{max} 3030.44, 2971.62, 2927.06, 2863.29, 1687.81, 1586.36, 1497.18, 1469.52, 1413.30, 1383.34, 1363.25, 1348.09, 1320.35, 1303.25, 1275.32, 1230.89, 1164.79, 1110.08, 1020.74, 963.52, 910.49, 851.06, 763.07, 696.34, 603.32, 593.51, 578.04, 554.01, 459.88, 427.65, 411.13, 402.52 cm^{-1}.

HRMS (ESI-TOF) *m/z* calcd. for $C_{22}H_{30}BrN_2O_2^+$ ($[M-C_5H_9O_2+H]^+$) 433.1485, found 433.1481.

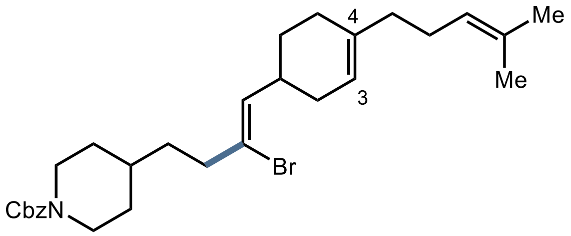

benzyl (*Z*)-4-(3-bromo-4-(4-(4-methylpent-3-en-1-yl)cyclohex-3-en-1-yl)but-3-en-1-yl)piperidine-1-carboxylate (63)

The title compound was prepared according to **General Procedure B** using **NHC** (276.7 mg, 0.70 mmol, 1.4 equiv.), benzyl 4-(2-hydroxyethyl)piperidine-1-carboxylate (171.1 mg, 0.65 mmol, 1.3 equiv.), pyridine (56.6 µL, 0.70 mmol, 1.4 equiv.), and *t*-BuOMe (5 mL) for **Stock A** and Ir[(ppy)-$_2$(dtbbpy)]PF_6 (6.9 mg, 7.5 µmol, 0.015 equiv.), $NiBr_2$(dtbbpy) (24.3 mg, 50.0 µmol, 0.10 equiv.), quinuclidine (77.8 mg, 0.70 mmol, 1.4 equiv.), phthalimide (73.6 mg, 0.50 mmol, 1.0 equiv.), **63-DiBr** (174.1 mg, 0.5 mmol, 1.0 equiv.), and 1,2-dichlorobenzene (10 mL) for **Stock B**. The crude mixture was concentrated via *Genevac* then purified by automated flash chromatography (25 g high performance silica column, 0-25% ethyl acetate/hexanes gradient) to yield impure product. The impure product was isolated as a 64:36 mixture of the 4- and 3- isomers. This was further purified by preparative HPLC (30-70% MeCN in water with a 0.1% NH_4OH modifier) to provide the isomers of the desired compound, each as clear oils (148.6 mg, 0.289 mmol, 58% yield). The products were both isolated in >20:1 *Z:E* ratios of diastereomers (configuration determined by vinylic NOE correlations).

4-Isomer ^{1}H NMR (500 MHz, $CDCl_3$) δ 7.38 – 7.28 (m, 5H), 5.54 (d, *J* = 8.6 Hz, 1H), 5.37 (s, 1H), 5.16 – 5.05 (m, 3H), 4.16 (s, 2H), 2.75 (s, 2H), 2.60 (ddtd, *J* = 24.3, 12.4, 5.9, 3.0 Hz, 1H), 2.43 (t, *J* = 7.5 Hz, 2H), 2.19 – 2.11 (m, 1H), 2.11 – 2.00 (m, 3H), 1.98 – 1.90 (m, 3H), 1.82 – 1.71 (m, 2H), 1.70 – 1.65 (m, 4H), 1.60 (s, 3H), 1.50 (q, *J* = 7.2 Hz, 2H), 1.46 (s, 1H), 1.42 (dtd, *J* = 13.1, 7.1, 3.9 Hz, 2H), 1.20 – 1.06 (m, 2H).

3-Isomer ^{1}H NMR (500 MHz, CDCl$_3$) δ 7.39 – 7.28 (m, 5H), 5.54 (d, *J* = 8.5 Hz, 1H), 5.40 (s, 1H), 5.11 (d, *J* = 13.0 Hz, 3H), 4.17 (s, br, 2H), 2.84 – 2.62 (m, 3H), 2.43 (t, *J* = 7.5 Hz, 2H), 2.05 (dq, *J* = 14.7, 5.2 Hz, 4H), 1.94 (t, *J* = 7.9 Hz, 2H), 1.77 – 1.62 (m, 6H), 1.60 (s, 3H), 1.54 – 1.29 (m, 6H), 1.21 – 1.06 (m, 2H).

4-Isomer ^{13}C NMR (126 MHz, CDCl$_3$) δ 155.43, 137.79, 137.11, 133.57, 131.56, 128.61, 128.05, 127.96, 126.83, 124.46, 119.49, 67.09, 44.32, 38.83, 37.88, 36.42, 34.94 – 34.93 (rotamers), 32.07, 30.60, 28.59, 28.16, 27.65, 26.58, 25.86, 17.84.

3-Isomer ^{13}C NMR (126 MHz, CDCl$_3$) δ 155.44, 137.10, 136.52, 133.51, 131.57, 128.60, 128.06, 127.96, 126.77, 124.41, 120.66, 67.10, 44.31, 38.82, 37.95, 36.80, 34.95, 33.62, 32.07, 28.59, 27.68, 26.56, 25.85, 24.48, 17.85.

IR (film) ν_{max} 2919.74, 2905.07, 2877.46, 2849.98, 1700.60, 1467.43, 1432.62, 1275.63, 1240.18, 1125.25 cm^{-1}.

HRMS (ESI-TOF) *m/z* calcd. for $C_{29}H_{41}BrNO_2^+$ ([M+H]$^+$) 514.2315, found 514.2312.

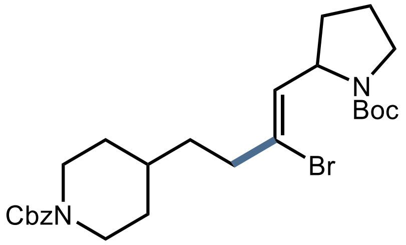

(±)-benzyl (*Z*)-4-(3-bromo-4-(1-(*tert*-butoxycarbonyl)pyrrolidin-2-yl)but-3-en-1-yl)piperidine-1-carboxylate (64)

The title compound was prepared according to **General Procedure B** using **NHC** (276.7 mg, 0.70 mmol, 1.4 equiv.), benzyl 4-(2-hydroxyethyl)piperidine-1-carboxylate (171.1 mg, 0.65 mmol, 1.3 equiv.), pyridine (56.6 μL, 0.70 mmol, 1.4 equiv.), and *t*-BuOMe (5 mL) for **Stock A** and Ir[(ppy)-$_2$(dtbbpy)]PF_6 (6.9 mg, 7.5 μmol, 0.015 equiv.), $NiBr_2$(dtbbpy) (24.3 mg, 50.0 μmol, 0.10 equiv.), quinuclidine (77.8 mg, 0.70 mmol, 1.4 equiv.), phthalimide (73.6 mg, 0.50 mmol, 1.0 equiv.), **64-DiBr** (177.3 mg, 0.5 mmol, 1.0 equiv.), and 1,2-dichlorobenzene (10 mL) for **Stock B**. The crude

mixture was concentrated via *Genevac* then purified by automated flash chromatography (25 g high performance silica column, 0-25% ethyl acetate/hexanes gradient) to yield impure product. This was further purified by preparative HPLC (30-70% MeCN in water with a 0.1% NH_4OH modifier) to provide the desired compound as a viscous brown oil (207.3 mg, 0.398 mmol, 80% yield). The product was isolated in a >20:1 *Z:E* ratio of diastereomers (configuration determined by vinylic NOE correlations).

^{1}H NMR (500 MHz, $CDCl_3$) δ 7.38 – 7.28 (m, 5H), 5.65 (d, *J* = 7.5 Hz, 1H), 5.12 (s, 2H), 4.52 (td, *J* = 7.6, 4.6 Hz, 1H), 4.16 (s, 2H), 3.48 – 3.35 (m, 2H), 2.75 (t, *J* = 12.9 Hz, 2H), 2.43 (t, *J* = 7.3 Hz, 2H), 2.16 (dq, *J* = 12.5, 7.5 Hz, 1H), 1.82 (p, *J* = 6.8 Hz, 2H), 1.74 – 1.60 (m, 3H), 1.55 – 1.44 (m, 3H), 1.43 (s, 9H), 1.12 (qd, *J* = 12.0, 6.2 Hz, 2H).

^{13}C NMR (126 MHz, $CDCl_3$) δ 155.40, 154.66, 137.09, 131.52, 128.60, 128.05, 127.96, 126.57, 79.37, 67.09, 58.69, 46.78, 44.26, 38.61, 34.90, 32.62, 32.04 (br), 28.71, 24.05.

IR (film) ν_{max} 2972.91, 2928.52, 1689.32, 1497.33, 1469.79, 1428.14, 1389.18, 1363.55, 1275.61, 1240.54, 1161.10, 1118.42, 1022.33, 964.39, 916.11, 883.64, 859.51, 763.36, 734.11, 696.70, 604.12, 547.28, 460.10 cm^{-1}.

HRMS (ESI-TOF) *m/z* calcd. for $C_{26}H_{37}BrN_2NaO_4^+$ ($[M+Na]^+$) 543.1829, found 543.1825.

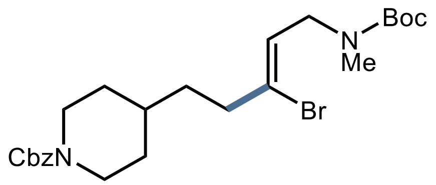

benzyl (*Z*)-4-(3-bromo-5-((*tert*-butoxycarbonyl)(methyl)amino)pent-3-en-1-yl)piperidine-1-carboxylate (65)

The title compound was prepared according to **General Procedure B** using **NHC** (276.7 mg, 0.70 mmol, 1.4 equiv.), benzyl 4-(2-hydroxyethyl)piperidine-1-carboxylate (171.1 mg, 0.65 mmol, 1.3 equiv.), pyridine (56.6 µL, 0.70 mmol, 1.4 equiv.), and *t*-BuOMe (5 mL) for **Stock A** and Ir[(ppy)-$_2$(dtbbpy)]PF_6 (6.9 mg, 7.5 µmol, 0.015 equiv.), $NiBr_2$(dtbbpy) (24.3 mg, 50.0 µmol, 0.10 equiv.),

quinuclidine (77.8 mg, 0.70 mmol, 1.4 equiv.), phthalimide (73.6 mg, 0.50 mmol, 1.0 equiv.), **65-DiBr** (164.5 mg, 0.50 mmol, 1.0 equiv.), and 1,2-dichlorobenzene (10 mL) for **Stock B**. The crude mixture was concentrated via *Genevac* then purified by automated flash chromatography (25 g high performance silica column, 0-25% ethyl acetate/hexanes gradient) to yield impure product. This was further purified by preparative HPLC (30-70% MeCN in water with a 0.1% NH_4OH modifier) to provide the desired compound as a pale yellow oil (170.9 mg, 0.345 mmol, 69% yield). The product was isolated in a >20:1 *Z:E* ratio of diastereomers (configuration determined by vinylic NOE correlations).

^{1}H NMR (500 MHz, $CDCl_3$) δ 7.40 – 7.28 (m, 5H), 5.67 (t, *J* = 6.3 Hz, 1H), 5.12 (s, 2H), 4.16 (d, *J* = 13.1 Hz, 2H), 3.95 (d, *J* = 6.2 Hz, 2H), 2.82 (s, 3H), 2.75 (t, *J* = 12.8 Hz, 2H), 2.47 (t, *J* = 7.3 Hz, 2H), 1.67 (d, *J* = 12.7 Hz, 2H), 1.51 (q, *J* = 7.2 Hz, 2H), 1.46 (s, 9H), 1.44 – 1.37 (m, 1H), 1.13 (qd, *J* = 12.3, 4.3 Hz, 2H).

^{13}C NMR (126 MHz, $CDCl_3$) δ 155.75, 155.39, 137.08, 130.39, 128.60, 128.06, 127.96, 125.05, 79.81, 67.10, 49.71 (br), 44.26, 38.85, 34.98, 34.85, 34.20, 32.02, 28.59.

IR (film) ν_{max} 2928.20, 2852.86, 1690.67, 1426.95, 1389.38, 1364.34, 1275.79, 1240.06, 1171.95, 1146.41, 1123.58, 1076.11, 1023.02, 964.12, 921.44, 878.08, 763.69, 733.72, 696.62, 604.05, 555.67, 458.95 cm^{-1}.

HRMS (ESI-TOF) *m/z* calcd. for $C_{24}H_{35}BrN_2NaO_4^+$ ([M+Na]$^+$) 517.1672, found 517.1676.

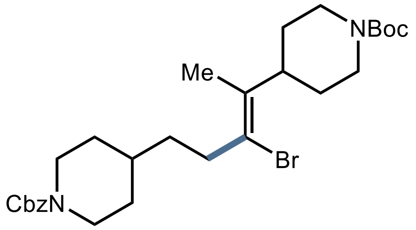

benzyl (*Z*)-4-(3-bromo-4-(1-(*tert*-butoxycarbonyl)piperidin-4-yl)pent-3-en-1-yl)piperidine-1-carboxylate (66)

The title compound was prepared according to **General Procedure B** using **NHC** (276.7 mg, 0.70 mmol, 1.4 equiv.), benzyl 4-(2-hydroxyethyl)piperidine-1-carboxylate (171.1 mg, 0.65 mmol, 1.3 equiv.), pyridine (56.6 μL, 0.70 mmol, 1.4 equiv.), and *t*-BuOMe (5 mL) for **Stock A** and Ir[(ppy)-$_2$(dtbbpy)]PF_6 (6.9 mg, 7.5 μmol, 0.015 equiv.), $NiBr_2$(dtbbpy) (24.3 mg, 50.0 μmol, 0.10 equiv.), quinuclidine (77.8 mg, 0.70 mmol, 1.4 equiv.), phthalimide (73.6 mg, 0.50 mmol, 1.0 equiv.), **66-DiBr** (191.6 mg, 0.5 mmol, 1.0 equiv.), and trifluorotoluene (10 mL) for **Stock B**. The crude mixture was concentrated via *Genevac* then purified by automated flash chromatography (25 g high performance silica column, 0-25% ethyl acetate/hexanes gradient) to yield impure product. This was further purified by preparative HPLC (30-70% MeCN in water with a 0.1% NH_4OH modifier) to provide the desired compound as viscous brown oil (134.4 mg, 0.245 mmol, 49% yield). The desired compound was isolated as a 77:23 mixture of *Z:E* diastereomers (ratio determined by integration of the crude ^{1}H NMR and assigned by 2D NMR correlations). Spectral data is provided for the each diastereomer.

(*Z*)-Isomer ^{1}H NMR (500 MHz, $CDCl_3$) δ 7.38 – 7.28 (m, 5H), 5.12 (s, 2H), 4.14 (d, *J* = 19.1 Hz, 4H), 2.91 (tt, *J* = 12.0, 3.7 Hz, 1H), 2.82 – 2.66 (m, 4H), 2.54 – 2.48 (m, 2H), 1.72 – 1.63 (m, 2H), 1.60 (s, 3H), 1.53 – 1.47 (m, 4H), 1.46 (s, 9H), 1.44 – 1.34 (m, 3H), 1.14 (d, *J* = 12.7 Hz, 2H).

(*Z*)-Isomer ^{13}C NMR (126 MHz, $CDCl_3$) δ 155.42, 154.97, 137.09, 135.83, 128.61, 128.06, 127.97, 122.14, 79.53, 67.10, 44.31, 44.29, 44.07 (br), 35.40, 35.33, 35.02, 32.16 (br), 29.04 (br), 28.61, 14.39.

(*E*)-Isomer ^{1}H NMR (500 MHz, $CDCl_3$) δ 7.38 – 7.28 (m, 5H), 5.12 (s, 2H), 4.17 (s, br, 4H), 3.48 (s, 1H), 2.82 – 2.61 (m, 4H), 2.57 (tt, *J* = 8.7, 4.2 Hz, 3H), 1.75 (s, 3H), 1.69 (d, *J* = 12.1 Hz, 2H), 1.58 – 1.48 (m, 4H), 1.46 (s, 10H), 1.42 – 1.36 (m, 2H), 1.22 – 1.10 (m, 2H).

(*E*)-Isomer ^{13}C NMR (126 MHz, $CDCl_3$) δ 155.39, 154.86, 137.05, 137.03, 128.59, 128.07, 127.98, 124.14, 79.72, 67.12, 44.25, 40.61, 44.12 (br), 35.85, 35.24, 34.56, 32.16 (br), 30.07 (br), 28.57, 19.18.

IR (film) ν_{max} 2975.50, 2922.39, 2851.44, 1680.73, 1497.54, 1467.57, 1422.27, 1392.56, 1364.44, 1305.93, 1293.92, 1275.20, 1232.03, 1164.32, 1125.00, 1086.23, 1012.01, 963.21, 906.92, 862.60, 764.68, 725.64, 696.08, 645.76, 603.64, 556.11, 532.91, 455.80, 411.22 cm^{-1}.

HRMS (ESI-TOF) *m/z* calcd. for $C_{23}H_{34}BrN_2O_2^+$ ($[M-C_5H_9O_2+H]^+$) 449.1798, found 449.1795.

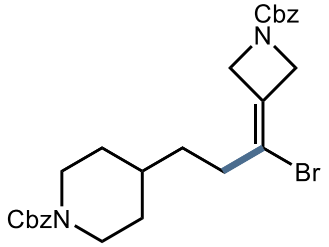

benzyl 4-(3-(1-((benzyloxy)carbonyl)azetidin-3-ylidene)-3-bromopropyl)piperidine-1-carboxylate (67)

The title compound was prepared according to **General Procedure B** using **NHC** (276.7 mg, 0.70 mmol, 1.4 equiv.), benzyl 4-(2-hydroxyethyl)piperidine-1-carboxylate (171.1 mg, 0.65 mmol, 1.3 equiv.), pyridine (56.6 µL, 0.70 mmol, 1.4 equiv.), and *t*-BuOMe (5 mL) for **Stock A** and Ir[(ppy)-$_2$(dtbbpy)]PF_6 (6.9 mg, 7.5 µmol, 0.015 equiv.), $NiBr_2$(dtbbpy) (24.3 mg, 50.0 µmol, 0.10 equiv.), quinuclidine (77.8 mg, 0.70 mmol, 1.4 equiv.), phthalimide (73.6 mg, 0.50 mmol, 1.0 equiv.), **67-DiBr** (180.5 mg, 0.5 mmol, 1.0 equiv.), and 1,2-dichlorobenzene (10 mL) for **Stock B**. The crude mixture was concentrated via *Genevac* then purified by automated flash chromatography (25 g high performance silica column, 0-25% ethyl acetate/hexanes gradient) to yield impure product. This was further purified by preparative HPLC (30-70% MeCN in water with a 0.1% NH_4OH modifier) to provide the desired compound as viscous yellow oil (185.8 mg, 0.353 mmol, 71% yield).

^{1}H NMR (500 MHz, $CDCl_3$) δ 7.41 – 7.26 (m, 10H), 5.12 (s, 4H), 4.51 – 4.39 (m, 4H), 4.17 (s, 2H), 2.76 (t, *J* = 12.8 Hz, 2H), 2.30 (t, *J* = 7.6 Hz, 2H), 1.65 (d, *J* = 13.1 Hz, 2H), 1.48 (q, *J* = 7.1 Hz, 2H), 1.41 (ddq, *J* = 10.9, 7.3, 3.8 Hz, 1H), 1.14 (td, *J* = 11.9, 4.1 Hz, 2H).

^{13}C NMR (126 MHz, $CDCl_3$) δ 156.45, 155.37, 137.04, 136.50, 128.66, 128.60, 128.32, 128.21, 128.07, 127.97, 127.24, 118.97, 67.16, 67.12, 59.35 (br), 56.47 (br), 44.21, 34.98, 34.35, 33.51, 32.00 (br).

IR (film) ν_{max} 2929.59, 2858.61, 1693.67, 1497.26, 1408.23, 1347.97, 1275.70, 1233.81, 1162.73, 1123.47, 1026.42, 962.65, 862.34, 733.13, 695.48, 605.70, 556.39, 457.33 cm^{-1}.

HRMS (ESI-TOF) *m/z* calcd. for $C_{27}H_{31}BrN_2NaO_4^+$ ($[M+Na]^+$) 549.1359, found 549.1360.

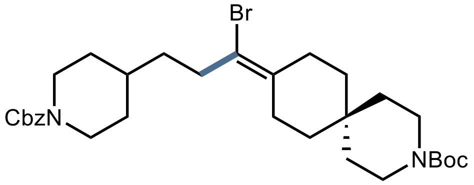

***tert*-butyl 9-(3-(1-((benzyloxy)carbonyl)piperidin-4-yl)-1-bromopropylidene)-3-azaspiro[5.5]undecane-3-carboxylate (68)**

The title compound was prepared according to **General Procedure B** using **NHC** (276.7 mg, 0.70 mmol, 1.4 equiv.), benzyl 4-(2-hydroxyethyl)piperidine-1-carboxylate (171.1 mg, 0.65 mmol, 1.3 equiv.), pyridine (56.6 µL, 0.70 mmol, 1.4 equiv.), and *t*-BuOMe (5 mL) for **Stock A** and Ir[(ppy)-$_2$(dtbbpy)]PF_6 (6.9 mg, 7.5 µmol, 0.015 equiv.), $NiBr_2$(dtbbpy) (24.3 mg, 50.0 µmol, 0.10 equiv.), quinuclidine (77.8 mg, 0.70 mmol, 1.4 equiv.), phthalimide (73.6 mg, 0.50 mmol, 1.0 equiv.), **68-DiBr** (211.6 mg, 0.5 mmol, 1.0 equiv.), and trifluorotoluene (10 mL) for **Stock B**. The crude mixture was concentrated via *Genevac* then purified by automated flash chromatography (25 g high performance silica column, 0-25% ethyl acetate/hexanes gradient) to yield impure product. This was further purified by preparative HPLC (30-70% MeCN in water with a 0.1% NH_4OH modifier) to provide the desired compound as viscous light brown oil (147.3 mg, 0.250 mmol, 50% yield).

^{1}H NMR (500 MHz, $CDCl_3$) δ 7.39 – 7.28 (m, 5H), 5.12 (s, 2H), 4.16 (s, 2H), 3.38 (t, J = 5.8 Hz, 4H), 2.74 (s, br, 2H), 2.53 (t, J = 7.7 Hz, 2H), 2.39 (t, J = 6.4 Hz, 2H), 2.22 (d, J = 6.0 Hz, 2H), 1.72 – 1.63 (m, 2H), 1.51 – 1.47 (m, 2H), 1.45 (s, 9H), 1.44 – 1.33 (m, 7H), 1.20 – 1.07 (m, 2H).

^{13}C NMR (126 MHz, $CDCl_3$) δ 155.42, 155.12, 137.09, 137.05, 128.60, 128.06, 127.97, 119.71, 79.44, 67.10, 44.31, 39.74 (br), 36.87, 36.01, 35.45 (br), 35.37, 35.22, 34.55, 32.10 (br), 31.21, 30.19, 28.61, 25.91.

IR (film) ν_{max} 2970.48, 2919.05, 2849.42, 1688.81, 1469.08, 1422.87, 1363.59, 1304.86, 1276.82, 1234.20, 1163.42, 1120.76, 1085.59, 1013.64, 982.47, 963.71, 928.23, 862.18, 823.46, 806.26, 762.97, 733.23, 696.41, 604.06, 593.34, 549.36, 458.95 cm^{-1}.

HRMS (ESI-TOF) *m/z* calcd. for $C_{31}H_{45}BrN_2NaO_4^+$ ($[M+Na]^+$) 611.2455, found 611.2451.

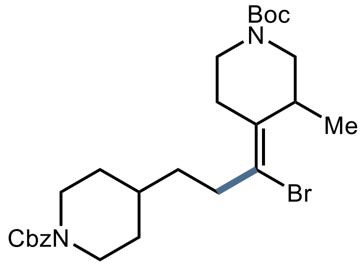

tert-butyl (_Z_)-4-(3-(1-((benzyloxy)carbonyl)piperidin-4-yl)-1-bromopropylidene)-3-methylpiperidine-1-carboxylate (69)

The title compound was prepared according to **General Procedure B** using **NHC** (276.7 mg, 0.70 mmol, 1.4 equiv.), benzyl 4-(2-hydroxyethyl)piperidine-1-carboxylate (171.1 mg, 0.65 mmol, 1.3 equiv.), pyridine (56.6 µL, 0.70 mmol, 1.4 equiv.), and *t*-BuOMe (5 mL) for **Stock A** and Ir[(ppy)-$_2$(dtbbpy)]PF_6 (6.9 mg, 7.5 µmol, 0.015 equiv.), $NiBr_2$(dtbbpy) (24.3 mg, 50.0 µmol, 0.10 equiv.), quinuclidine (77.8 mg, 0.70 mmol, 1.4 equiv.), phthalimide (73.6 mg, 0.50 mmol, 1.0 equiv.), **69-DiBr** (184.5 mg, 0.5 mmol, 1.0 equiv.), and trifluorotoluene (10 mL) for **Stock B**. The crude mixture was concentrated via *Genevac* then purified by automated flash chromatography (25 g high performance silica column, 0-25% ethyl acetate/hexanes gradient) to yield impure product. This was further purified by preparative HPLC (30-70% MeCN in water with a 0.1% NH_4OH modifier) to provide the desired compound as viscous yellow oil (146.6 mg, 0.274 mmol, 55% yield). The desired compound was isolated as a 59:41 mixture of *Z:E* diastereomers (ratio determined by chiral HPLC and assigned by 2D NMR correlations). To assign the identity of each diastereomer, analytical quantities were isolated with chiral supercritical fluid chromatography (ColumnTek Enantiocel A6-5 (250 x 4.6 mm), 2.0 mL/min, 40% ethanol (0.1% diethylamine)/60%

CO_2 (100 bar)). The (*Z*)-isomer could not be fully isolated from the (*E*)-isomer. As such, trace quantities of the (*E*)-isomer are present in the NMR spectra of the (*Z*)-isomer. Spectral data is provided for each diastereomer.

(*Z*)-isomer ^{1}H NMR (500 MHz, $CDCl_3$) δ 7.40 – 7.27 (m, 5H), 5.12 (s, 2H), 4.30 – 3.77 (m, 4H), 3.11 (s, 1H), 2.97 – 2.84 (m, 1H), 2.83 – 2.56 (m, 3H), 2.52 (t, *J* = 7.7 Hz, 2H), 2.42 (dt, *J* = 14.6, 3.3 Hz, 1H), 2.21 (ddd, *J* = 14.6, 12.4, 5.3 Hz, 1H), 1.72 – 1.62 (m, 2H), 1.61 – 1.50 (m, 2H), 1.47 (s, 9H), 1.40 (dtt, *J* = 14.0, 6.8, 3.9 Hz, 1H), 1.31 – 1.19 (m, 1H), 1.18 – 1.11 (m, 1H), 1.04 (d, *J* = 7.0 Hz, 3H).

(*Z*)-isomer ^{13}C NMR (126 MHz, $CDCl_3$) δ 155.42, 155.33, 137.09, 136.96, 128.62, 128.60, 128.08, 128.03, 127.98, 127.96, 121.50, 79.75, 67.12, 67.05, 49.52, 48.32 (br), 44.44, 44.30, 43.74, 37.78, 36.60, 36.04, 35.36, 35.30, 34.50, 32.16 (br), 28.57, 26.94, 26.43, 16.48.

(*E*)-isomer ^{1}H NMR (500 MHz, $CDCl_3$) δ 7.40 – 7.27 (m, 5H), 5.12 (s, 2H), 4.31 – 3.80 (m, 3H), 2.90 – 2.64 (m, 5H), 2.54 (t, *J* = 7.7 Hz, 2H), 2.28 (td, *J* = 14.1, 5.6 Hz, 1H), 1.69 (d, *J* = 10.3 Hz, 2H), 1.57 – 1.48 (m, 4H), 1.46 (s, 9H), 1.42 (h, *J* = 3.4 Hz, 1H), 1.16 (d, *J* = 11.0 Hz, 2H), 1.07 (d, *J* = 7.3 Hz, 3H).

(*E*)-isomer ^{13}C NMR (126 MHz, $CDCl_3$) δ 155.42, 137.36, 137.08, 128.62, 128.09, 128.00, 122.20, 79.76, 67.14, 49.87, 48.61 (br), 44.29, 43.65, 35.62, 35.41, 34.37, 32.13 (br), 30.23 (br), 28.58, 17.67.

IR (film) ν_{max} 2970.09, 2926.89, 2853.91, 1690.18, 1497.36, 1422.90, 1363.82, 1324.53, 1274.96, 1234.39, 1162.77, 1124.38, 1076.86, 1021.62, 962.55, 887.72, 861.21, 827.91, 762.71, 733.43, 696.40, 603.95, 556.16, 458.44, 434.49, 414.26 cm^{-1}.

HRMS (ESI-TOF) *m/z* calcd. for $C_{27}H_{39}BrN_2NaO_4^+$ ($[M+Na]^+$) 557.1985, found 557.1982.

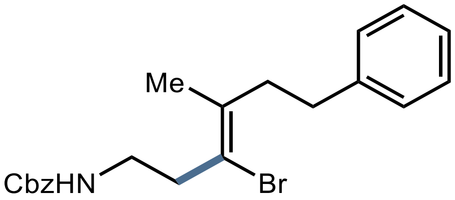

benzyl (*E/Z*)-(3-bromo-4-methyl-6-phenylhex-3-en-1-yl)carbamate (70)

The title compound was prepared according to **General Procedure B** using **NHC** (276.7 mg, 0.70 mmol, 1.4 equiv.), benzyl 4-(2-hydroxyethyl)piperidine-1-carboxylate (171.1 mg, 0.65 mmol, 1.3 equiv.), pyridine (56.6 µL, 0.70 mmol, 1.4 equiv.), and *t*-BuOMe (5 mL) for **Stock A** and Ir[(ppy)-$_2$(dtbbpy)]PF_6 (6.9 mg, 7.5 µmol, 0.015 equiv.), $NiBr_2$(dtbbpy) (24.3 mg, 50.0 µmol, 0.10 equiv.), quinuclidine (77.8 mg, 0.70 mmol, 1.4 equiv.), phthalimide (73.6 mg, 0.50 mmol, 1.0 equiv.), **70-DiBr** (152.0 mg, 0.5 mmol, 1.0 equiv.), and trifluorotoluene (10 mL) for **Stock B**. The crude mixture was concentrated via *Genevac* then purified by automated flash chromatography (25 g high performance silica column, 0-25% ethyl acetate/hexanes gradient) to yield impure product. This was further purified by preparative HPLC (30-70% MeCN in water with a 0.1% NH_4OH modifier) to provide the desired compound as a light green oil (107.3 mg, 0.267 mmol, 53% yield). The desired compound was isolated as an inseparable 55:45 mixture of *Z:E* diastereomers (ratio determined by integration of the crude ^{1}H NMR and assigned by NOE correlations). Spectral data is provided for the mixture of diastereomers.

^{1}H NMR (500 MHz, $CDCl_3$) δ 7.40 – 7.26 (m, 7H), 7.21 – 7.13 (m, 3H), 5.08 (d, *J* = 19.7 Hz, 2H), 4.75 (d, *J* = 31.0 Hz, 1H), 3.33 (dq, *J* = 24.2, 6.3 Hz, 2H), 2.76 – 2.68 (m, 3H), 2.66 – 2.37 (m, 3H), 1.80 (d, *J* = 107.0 Hz, 3H).

^{13}C NMR (126 MHz, $CDCl_3$) δ 156.47, 156.38, 141.49, 141.20, 136.72, 136.61, 136.53, 136.50, 128.68, 128.65, 128.63, 128.60, 128.59, 128.48, 128.35 (s, 2C), 128.31, 128.28, 126.29, 126.16, 120.36, 119.04, 66.85, 66.82, 40.96, 39.75, 39.45, 37.80, 37.08, 36.82, 34.64, 33.47, 23.77, 18.93.

IR (film) ν_{max} 3028.01, 2943.71, 1701.37, 1515.79, 1453.67, 1246.22, 1137.85, 1076.70, 1027.44, 748.09, 698.15 cm^{-1}.

HRMS (ESI-TOF) *m/z* calcd. for $C_{21}H_{24}BrNNaO_2^+$ ($[M+Na]^+$) 424.0883, found 424.0883.

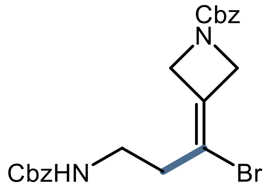

benzyl 3-(3-(((benzyloxy)carbonyl)amino)-1-bromopropylidene)azetidine-1-carboxylate (83-SI)

The title compound was prepared according to **General Procedure B** using **NHC** (276.7 mg, 0.70 mmol, 1.4 equiv.), benzyl (2-hydroxyethyl)carbamate (126.9 mg, 0.65 mmol, 1.3 equiv.), pyridine (56.6 μL, 0.70 mmol, 1.4 equiv.), and *t*-BuOMe (5 mL) for **Stock A** and Ir[(ppy)$_2$(dtbbpy)]PF$_6$ (6.9 mg, 7.5 μmol, 0.015 equiv.), NiBr$_2$(dtbbpy) (24.3 mg, 50.0 μmol, 0.10 equiv.), quinuclidine (77.8 mg, 0.70 mmol, 1.4 equiv.), phthalimide (73.6 mg, 0.50 mmol, 1.0 equiv.), **67-DiBr** (180.5 mg, 0.5 mmol, 1.0 equiv.), and 1,2-dichlorobenzene (10 mL) for **Stock B**. The crude mixture was concentrated via *Genevac* then purified by automated flash chromatography (25 g high performance silica column, 0-25% ethyl acetate/hexanes gradient) to yield impure product. This was further purified by preparative HPLC (30-70% MeCN in water with a 0.1% NH_4OH modifier). The pure material, obtained as a white solid, was subsequently used to determine the assay yield of the crude reaction mixture by ^{1}H NMR analysis with mesitylene as an internal standard, which indicated a yield of 78%.

^{1}H NMR (500 MHz, CDCl$_3$) δ 7.38 – 7.27 (m, 10H), 5.12 (s, 2H), 5.09 (s, 2H), 4.87 (s, 1H), 4.42 (s, 4H), 3.39 (q, *J* = 6.4 Hz, 2H), 2.51 (t, *J* = 6.4 Hz, 2H).

^{13}C NMR (126 MHz, CDCl$_3$) δ 156.38, 156.33, 136.51, 136.46, 130.33, 128.74, 128.69, 128.44, 128.34, 128.32, 128.22, 115.29, 67.18, 67.02, 59.23 (br), 56.56 (br), 38.99, 36.44.

IR (film) ν_{max} 3308.73, 3029.24, 2968.29, 2939.50, 2898.18, 2863.78, 1709.24, 1679.85, 1518.71, 1428.19, 1350.38, 1270.71, 1245.59, 1173.73, 1131.13, 994.67, 896.67, 751.29, 694.01, 605.35, 561.57, 543.71, 466.24, 440.74, 423.90, 413.75 cm^{-1}.

HRMS (ESI-TOF) *m/z* calcd. for $C_{22}H_{23}BrN_2NaO_4^+$ ([M+Na]$^+$) 481.0733, found 481.0731.

7) Experimental Data for *gem*-Dibromoolefin Starting Materials

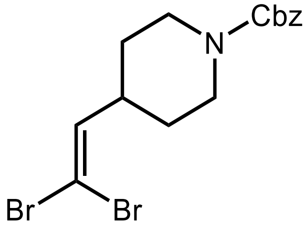

benzyl 4-(2,2-dibromovinyl)piperidine-1-carboxylate (51-DiBr)

The title compound was prepared according to **General Procedure C** using carbon tetrabromide (14.92 g, 45.0 mmol, 2.25 equiv.), triphenylphosphine (23.60 g, 90.0 mmol, 4.5 equiv.), benzyl 4-formylpiperidine-1-carboxylate (4.05 mL, 20 mmol, 1.0 equiv.), and CH_2Cl_2 (180 mL + 20 mL). This mixture was allowed to warm to room temperature with stirring overnight. The solution was then poured into stirring hexane (~800 mL), filtered, and the filtrate concentrated under reduced pressure via rotary evaporation. The residue was purified by automated flash chromatography (100 g high performance silica column, 0-100% ethyl acetate/hexanes gradient) to provide the desired compounds as a white solid (4.8460 g, 12.02 mmol, 60% yield).

^{1}H NMR (500 MHz, $CDCl_3$) δ 7.41 – 7.28 (m, 5H), 6.22 (d, *J* = 8.9 Hz, 1H), 5.13 (s, 2H), 4.16 (s, 2H), 2.86 (s, 2H), 2.47 (tdt, *J* = 11.4, 8.9, 3.9 Hz, 1H), 1.73 (d, *J* = 13.1 Hz, 2H), 1.34 (q, *J* = 12.6 Hz, 2H).

^{13}C NMR (126 MHz, $CDCl_3$) δ 155.32, 141.45, 136.91, 128.64, 128.16, 128.03, 89.07, 67.26, 43.60, 40.69, 30.15.

IR (film) ν_{max} 2932.93, 2850.50, 1683.64, 1605.79, 1497.49, 1475.05, 1456.72, 1434.16, 1367.76, 1309.58, 1275.65, 1244.58, 1214.48, 1189.35, 1145.51, 1095.41, 1068.85, 1029.52, 1010.63, 977.11, 963.10, 929.41, 911.11, 872.72, 845.47, 789.74, 775.15, 763.65, 739.15, 697.89, 642.70, 601.14, 560.59, 484.45, 465.57, 438.31, 412.22 cm^{-1}.

HRMS (ESI-TOF) *m/z* calcd. for $C_{15}H_{18}Br_2NO_2^+$ ($[M+H]^+$) 401.9699, found 401.9695.

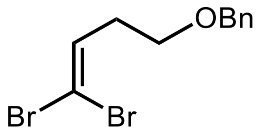

(((4,4-dibromobut-3-en-1-yl)oxy)methyl)benzene (52-DiBr)

The title compound was prepared according to **General Procedure C** using carbon tetrabromide (6.714 g, 20.3 mmol, 2.25 equiv.), triphenylphosphine (10.62 g, 40.5 mmol, 4.5 equiv.), 3-(benzyloxy)propanal (1.4 mL, 9 mmol, 1.0 equiv.), and CH_2Cl_2 (81 mL + 10 mL). This mixture was allowed to warm to room temperature with stirring overnight. The solution was then poured into stirring hexane (~400 mL), filtered, and the filtrate concentrated under reduced pressure via rotary evaporation. The residue was purified by automated flash chromatography (100 g high performance silica column, 0-100% ethyl acetate/hexanes gradient) to provide the desired compound as a clear-pale yellow oil (1.61 g, 5.03 mmol, 56% yield).

^{1}H NMR (500 MHz, $CDCl_3$) δ 7.40 – 7.28 (m, 5H), 6.51 (t, J = 7.0 Hz, 1H), 4.52 (s, 2H), 3.54 (t, J = 6.4 Hz, 2H), 2.42 (q, J = 6.6 Hz, 2H).

^{13}C NMR (126 MHz, $CDCl_3$) δ 138.20, 135.68, 128.58, 127.85, 127.80, 90.26, 73.12, 67.72, 33.80.

IR (film) ν_{max} 3029.27, 2857.22, 1702.80, 1623.57, 1495.24, 1477.67, 1453.08, 1360.34, 1309.63, 1239.58, 1205.33, 1094.00, 1027.59, 911.34, 803.96, 769.80, 733.32, 695.64, 649.56, 611.41, 457.72, 416.23 cm^{-1}.

HRMS (EI-QTOF) *m/z* calcd. for $C_{11}H_{12}Br_2O^{\bullet+}$ ($[M]^{\bullet+}$) 317.9249, found 317.9250.

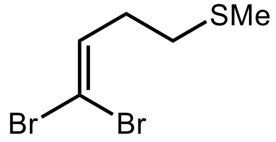

(4,4-dibromobut-3-en-1-yl)(methyl)sulfane (53-DiBr)

The title compound was prepared according to **General Procedure C** using carbon tetrabromide (6.714 g, 20.3 mmol, 2.25 equiv.), triphenylphosphine (10.62 g, 40.5 mmol, 4.5 equiv.), 3-(methylthio)propanal (0.90 mL, 9 mmol, 1.0 equiv.), and CH_2Cl_2 (81 mL + 10 mL). This mixture was allowed to warm to room temperature with stirring overnight. The solution was then poured into stirring hexane (~400 mL), filtered, and the filtrate concentrated under reduced pressure via rotary evaporation. The residue was purified by automated flash chromatography (100 g high performance silica column, 0-100% ethyl acetate/hexanes gradient) to provide the desired compound as a yellow oil (1.46 g, 5.62 mmol, 62% yield).

^{1}H NMR (500 MHz, $CDCl_3$) δ 6.48 (t, J = 7.1 Hz, 1H), 2.58 (t, J = 7.3 Hz, 2H), 2.40 (q, J = 7.2 Hz, 2H), 2.13 (s, 3H).

^{13}C NMR (126 MHz, $CDCl_3$) δ 136.65, 90.39, 32.68, 32.13, 15.57.

IR (film) ν_{max} 2913.12, 2831.99, 1624.10, 1430.62, 1317.17, 1280.21, 1266.30, 1266.30, 1201.23, 1159.01, 1056.95, 1017.10, 956.31, 932.14, 838.12, 797.39, 779.54, 696.67, 651.63, 566.34, 459.68, 413.74 cm^{-1}.

HRMS (EI-QTOF) *m/z* calcd. for $C_5H_8BrS^{\bullet+}$ ($[M-Br]^{\bullet+}$) 178.9525, found 178.9526.

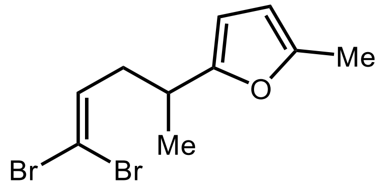

(±)-2-(5,5-dibromopent-4-en-2-yl)-5-methylfuran (54-DiBr)

The title compound was prepared according to **General Procedure C** using carbon tetrabromide (6.714 g, 20.3 mmol, 2.25 equiv.), triphenylphosphine (10.62 g, 40.5 mmol, 4.5 equiv.), 3-(5-methylfuran-2-yl)butanal (1.37 mL, 9 mmol, 1.0 equiv.), and CH_2Cl_2 (81 mL + 10 mL). This mixture was allowed to warm to room temperature with stirring overnight. The solution was then poured into stirring hexane (~400 mL), filtered, and the filtrate concentrated under reduced

pressure via rotary evaporation. The residue was purified by automated flash chromatography (100 g high performance silica column, 0-100% ethyl acetate/hexanes gradient) to provide the desired compound as an orange-brown oil (2.01 g, 6.24 mmol, 73% yield).

^{1}H NMR (500 MHz, CDCl$_3$) δ 6.36 (t, *J* = 7.1 Hz, 1H), 5.89 – 5.83 (m, 2H), 2.93 (h, *J* = 6.9 Hz, 1H), 2.44 (dt, *J* = 14.9, 6.8 Hz, 1H), 2.33 (dt, *J* = 14.8, 7.1 Hz, 1H), 2.26 (s, 3H), 1.25 (d, *J* = 7.0 Hz, 3H).

^{13}C NMR (126 MHz, CDCl$_3$) δ 156.99, 150.77, 136.82, 105.87, 104.95, 89.70, 38.87, 32.23, 18.55, 13.68.

IR (film) ν_{max} 2965.87, 2929.17, 1707.82, 1649.92, 1618.64, 1543.68, 1452.76, 1375.59, 1214.97, 1018.55, 939.98, 879.73, 779.67, 415.62, 405.39 cm^{-1}.

HRMS (EI-QTOF) *m/z* calcd. for $C_{10}H_{12}Br_2O^{\bullet+}$ ([M]$^{\bullet+}$) 305.2949, found 305.9247.

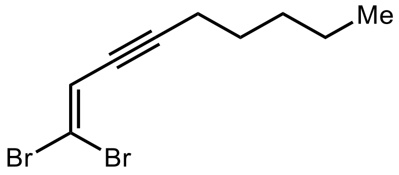

1,1-dibromonon-1-en-3-yne (55-DiBr)

The title compound was prepared according to **General Procedure C** using carbon tetrabromide (3.77 g, 11.4 mmol, 2.25 equiv.), triphenylphosphine (5.96 g, 22.7 mmol, 4.5 equiv.), oct-2-ynal (0.72 mL, 5.05 mmol, 1.0 equiv.), and CH_2Cl_2 (45 mL + 5 mL). This mixture was allowed to warm to room temperature with stirring overnight. The solution was then poured into stirring hexane (~200 mL), filtered, and the filtrate concentrated under reduced pressure via rotary evaporation. The residue was purified by automated flash chromatography (100 g high performance silica column, 0-100% ethyl acetate/hexanes gradient) to provide the desired compound as a dark yellow-brown oil (845.1 mg, 3.02 mmol, 60% yield).

^{1}H NMR (500 MHz, CDCl$_3$) δ 6.53 (t, *J* = 2.2 Hz, 1H), 2.31 (td, *J* = 7.0, 2.2 Hz, 2H), 1.61 – 1.52 (m, 2H), 1.42 – 1.31 (m, 4H), 0.90 (t, *J* = 7.2 Hz, 3H).

^{13}C NMR (126 MHz, CDCl$_3$) δ 120.29, 100.20, 99.68, 77.91, 31.12, 28.06, 22.29, 19.88, 14.11.

IR (film) ν_{max} 3017.85, 2955.61, 2929.02, 2858.75, 2216.34, 1709.02, 1565.08, 1465.29, 1426.74, 1378.31, 1325.77, 1262.22, 1169.16, 1106.77, 935.71, 858.30, 823.25, 797.18, 712.96, 568.49, 539.65, 456.91 cm^{-1}.

HRMS (EI-QTOF) *m/z* calcd. for $C_9H_{12}Br_2^{\bullet+}$ ($[M]^{\bullet+}$) 277.9300, found 277.9298.

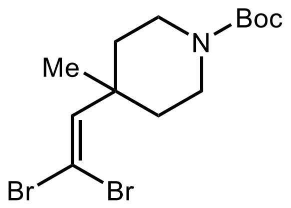

***tert*-butyl 4-(2,2-dibromovinyl)-4-methylpiperidine-1-carboxylate (56-DiBr)**

The title compound was prepared according to **General Procedure C** using carbon tetrabromide (2.80 g, 8.44 mmol, 2.25 equiv.), triphenylphosphine (4.43 g, 16.9 mmol, 4.5 equiv.), *tert*-butyl 4-formyl-4-methylpiperidine-1-carboxylate (852.4 mg, 3.75 mmol, 1.0 equiv.), and CH_2Cl_2 (34 mL + 4 mL). This mixture was allowed to warm to room temperature with stirring overnight. The solution was then poured into stirring hexane (~180 mL), filtered, and the filtrate concentrated under reduced pressure via rotary evaporation. The residue was purified by automated flash chromatography (100 g high performance silica column, 0-100% ethyl acetate/hexanes gradient) to provide the desired compound as a white solid (705.9 mg, 1.84 mmol, 49% yield).

^{1}H NMR (500 MHz, CDCl$_3$) δ 6.55 (s, 1H), 3.72 (d, *J* = 12.3 Hz, 2H), 3.03 (ddd, *J* = 13.7, 10.7, 3.0 Hz, 2H), 1.97 (d, *J* = 13.6 Hz, 2H), 1.45 (s, 9H), 1.37 (ddd, *J* = 13.6, 10.7, 4.1 Hz, 2H), 1.23 (s, 3H).

^{13}C NMR (126 MHz, CDCl$_3$) δ 155.01, 143.91, 87.16, 79.61, 40.62 (br), 38.24, 37.14, 28.59, 24.80.

IR (film) ν_{max} 3009.70, 2985.30, 2968.44, 2919.85, 2869.53, 2846.63, 1668.73, 1465.45, 1440.07, 1420.16, 1386.48, 1376.44, 1363.72, 1344.56, 1278.10, 1259.28, 1241.27, 1162.42, 1105.95, 1080.42, 1026.19, 1017.81, 988.80, 976.40, 916.90, 896.59, 869.17, 849.18, 808.59, 793.78, 766.00, 708.48, 603.22, 581.95, 420.17 cm^{-1}.

HRMS (ESI-TOF) *m/z* calcd. for $C_8H_{14}Br_2N^+$ ($[M-C_5H_9O_2+H]^+$) 281.9488, found 281.9487.

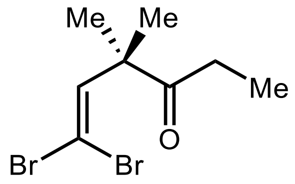

6,6-dibromo-4,4-dimethylhex-5-en-3-one (57-DiBr)

The title compound was prepared according to **General Procedure C** using carbon tetrabromide (3.73 g, 11.3 mmol, 2.25 equiv.), triphenylphosphine (5.90 g, 22.5 mmol, 4.5 equiv.), 2,2-dimethyl-3-oxopentanal (640.9 mg, 5.00 mmol, 1.0 equiv.), and CH_2Cl_2 (40 mL + 8 mL). This mixture was allowed to warm to room temperature with stirring overnight. The solution was then poured into stirring hexane (~180 mL), filtered, and the filtrate concentrated under reduced pressure via rotary evaporation. The residue was purified by automated flash chromatography (100 g high performance silica column, 0-100% ethyl acetate/hexanes gradient) to provide the desired compound as a clear liquid (151.7 mg, 0.534 mmol, 11% yield).

^{1}H NMR (500 MHz, $CDCl_3$) δ 6.74 (s, 1H), 2.56 (q, *J* = 7.3 Hz, 2H), 1.32 (s, 6H), 1.09 (t, *J* = 7.2 Hz, 3H).

^{13}C NMR (126 MHz, $CDCl_3$) δ 211.53, 143.42, 90.09, 52.18, 31.40, 24.92, 8.63.

IR (film) ν_{max} 3003.26, 2973.61, 2927.20, 2851.98, 1681.48, 1475.99, 1465.37, 1447.18, 1417.95, 1391.30, 1363.61, 1306.42, 1275.29, 1234.98, 1215.98, 1158.85, 1120.67, 1089.19, 1031.47, 983.90, 937.60, 905.49, 863.15, 832.84, 819.45, 787.27, 767.91, 728.69, 645.90, 534.08, 447.57, 432.09, 411.83 cm^{-1}.

HRMS (EI-QTOF) *m/z* calcd. for $C_8H_{12}Br_2O^{\bullet+}$ ($[M]^{\bullet+}$) 281.9249, found 281.9247.

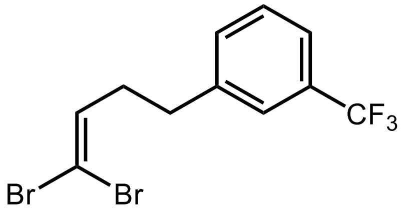

1-(4,4-dibromobut-3-en-1-yl)-3-(trifluoromethyl)benzene (58-DiBr)

The title compound was prepared according to **General Procedure C** using carbon tetrabromide (6.714 g, 20.3 mmol, 2.25 equiv.), triphenylphosphine (10.62 g, 40.5 mmol, 4.5 equiv.), 3-(3-(trifluoromethyl)phenyl)propanal (1.53 mL, 9 mmol, 1.0 equiv.), and CH_2Cl_2 (81 mL + 10 mL). This mixture was allowed to warm to room temperature with stirring overnight. The solution was then poured into stirring hexane (~180 mL), filtered, and the filtrate concentrated under reduced pressure via rotary evaporation. The residue was purified by automated flash chromatography (100 g high performance silica column, 0-100% ethyl acetate/hexanes gradient) to provide the desired compound as a brown oil (2.6911 g, 7.52 mmol, 84% yield).

^{1}H NMR (500 MHz, CDCl$_3$) δ 7.49 (d, *J* = 7.7 Hz, 1H), 7.46 – 7.39 (m, 2H), 7.37 (d, *J* = 7.7 Hz, 1H), 6.40 (t, *J* = 7.3 Hz, 1H), 2.80 (t, *J* = 7.7 Hz, 2H), 2.44 (q, *J* = 7.5 Hz, 2H).

^{13}C NMR (126 MHz, CDCl$_3$) δ 141.49, 136.98, 131.94, 131.00 (q, *J* = 32.0 Hz), 129.10, 125.26 (q, *J* = 3.8 Hz), 124.30 (q, *J* = 272.3 Hz), 123.37 (q, *J* = 3.8 Hz), 90.40, 34.49, 33.78.

^{19}F NMR (471 MHz, CDCl$_3$) δ -62.59 (s, 3F).

IR (film) ν_{max} 2931.79, 1718.01, 1596.99, 1492.23, 1450.25, 1324.72, 1197.83, 1160.05, 1118.34, 1097.91, 1071.85, 1002.20, 917.91, 899.76, 881.05, 795.04, 780.62, 749.85, 734.42, 700.69, 659.78, 588.27, 504.54, 445.93, 418.50 cm^{-1}.

HRMS (EI-QTOF) *m/z* calcd. for $C_{11}H_9Br_2F_3^{\bullet+}$ ($[M]^{\bullet+}$) 355.9018, found 355.9022.

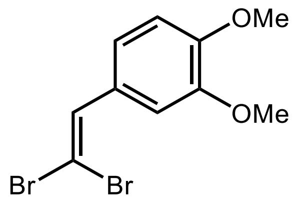

4-(2,2-dibromovinyl)-1,2-dimethoxybenzene (59-DiBr)

The title compound was prepared according to **General Procedure C** using carbon tetrabromide (6.714 g, 20.3 mmol, 2.25 equiv.), triphenylphosphine (10.62 g, 40.5 mmol, 4.5 equiv.), 3,4-dimethoxybenzaldehyde (1.496 g, 9.00 mmol, 1.0 equiv.), and CH_2Cl_2 (81 mL + 10 mL). This mixture was allowed to warm to room temperature with stirring overnight. The solution was then poured into stirring hexane (~400 mL), filtered, and the filtrate concentrated under reduced pressure via rotary evaporation. The residue was purified by automated flash chromatography (100 g high performance silica column, 0-100% ethyl acetate/hexanes gradient) to provide the desired compound as an orange oil (2.6712 g, 8.30 mmol, 92% yield).

^{1}H NMR (500 MHz, CDCl$_3$) δ 7.41 (s, 1H), 7.19 (d, *J* = 2.1 Hz, 1H), 7.09 (dd, *J* = 8.3, 1.4 Hz, 1H), 6.85 (d, *J* = 8.4 Hz, 1H), 3.89 (s, 3H), 3.89 (s, 3H).

^{13}C NMR (126 MHz, CDCl$_3$) δ 149.43, 148.70, 136.53, 128.09, 122.03, 111.25, 110.89, 87.51, 56.03, 56.01.

IR (film) ν_{max} 3001.19, 2954.51, 2932.29, 2905.06, 2833.69, 1598.67, 1510.36, 1461.43, 1439.69, 1417.60, 1407.37, 1334.47, 1257.68, 1233.20, 1194.14, 1155.32, 1139.82, 1022.28, 960.23, 944.09, 868.93, 837.39, 820.00, 798.75, 766.11, 717.02, 629.81, 612.21, 569.05, 551.17, 514.58, 480.32, 460.01, 438.76 cm^{-1}.

HRMS (EI-QTOF) *m/z* calcd. for $C_{10}H_{10}Br_2O_2^{\bullet+}$ ($[M]^{\bullet+}$) 319.9042, found 319.9040.

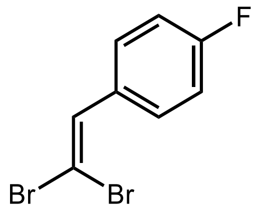

1-(2,2-dibromovinyl)-4-fluorobenzene (60-DiBr)

The title compound was prepared according to **General Procedure C** using carbon tetrabromide (6.714 g, 20.3 mmol, 2.25 equiv.), triphenylphosphine (10.62 g, 40.5 mmol, 4.5 equiv.), 4-fluorobenzaldehyde (0.97 mL, 9 mmol, 1.0 equiv.), and CH_2Cl_2 (81 mL + 10 mL). This mixture was allowed to warm to room temperature with stirring overnight. The solution was then poured into stirring hexane (~400 mL), filtered, and the filtrate concentrated under reduced pressure via rotary evaporation. The residue was purified by automated flash chromatography (100 g high performance silica column, 0-100% ethyl acetate/hexanes gradient) to provide the desired compounds as a yellow oil (2.0512 g, 7.33 mmol, 81% yield).

^{1}H NMR (500 MHz, $CDCl_3$) δ 7.56 – 7.49 (m, 2H), 7.44 (s, 1H), 7.10 – 7.02 (m, 2H).

^{13}C NMR (126 MHz, $CDCl_3$) δ 162.58 (d, *J* = 249.6 Hz), 135.88, 131.55 (d, *J* = 3.3 Hz), 130.43 (d, *J* = 8.1 Hz), 115.64 (d, *J* = 21.8 Hz), 89.78 (d, *J* = 2.3 Hz).

^{19}F NMR (471 MHz, $CDCl_3$) δ -111.70 (tt, *J* = 8.4, 5.3 Hz, 1F).

IR (film) ν_{max} 3013.68, 1892.82, 1601.36, 1584.96, 1504.06, 1457.76, 1410.11, 1316.57, 1266.06, 1226.04, 1159.09, 1098.07, 1014.05, 941.17, 872.22, 808.92, 739.09, 707.58, 644.06, 573.53, 515.23, 495.29, 441.98, 407.58 cm^{-1}.

HRMS (EI-QTOF) *m/z* calcd. for $C_8H_5Br_2F^{\bullet+}$ ($[M]^{\bullet+}$) 277.8737, found 277.8738.

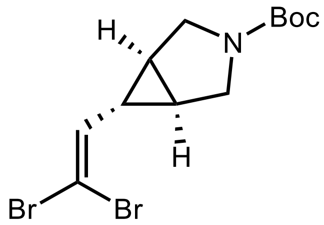

***tert*-butyl (1*R*,5*S*,6*s*)-6-(2,2-dibromovinyl)-3-azabicyclo[3.1.0]hexane-3-carboxylate (62-DiBr)**

The title compound was prepared according to **General Procedure C** using carbon tetrabromide (1.823 g, 5.50 mmol, 2.25 equiv.), triphenylphosphine (2.8845 g, 11.0 mmol, 4.5 equiv.), *tert*-butyl (1R,5S,6r)-6-formyl-3-azabicyclo[3.1.0]hexane-3-carboxylate (516.5 mg, 2.444 mmol, 1.0 equiv.), and CH_2Cl_2 (22 mL + 3 mL). This mixture was allowed to warm to room temperature with stirring overnight. The solution was then poured into stirring hexane (~100 mL), filtered, and the filtrate concentrated under reduced pressure via rotary evaporation. The residue was purified by automated flash chromatography (50 g high performance silica column, 0-100% ethyl acetate/hexanes gradient) to provide the desired compounds as an orange oil (653.6 g, 1.78 mmol, 73% yield).

^{1}H NMR (500 MHz, $CDCl_3$) δ 5.87 (d, *J* = 9.1 Hz, 1H), 3.63 (d, *J* = 31.6 Hz, 2H), 3.38 (d, *J* = 11.0 Hz, 2H), 1.66 (td, *J* = 3.1, 1.2 Hz, 2H), 1.49 – 1.46 (m, 1H), 1.44 (s, 9H).

^{13}C NMR (126 MHz, $CDCl_3$) δ 155.28, 138.56, 87.20, 80.06, 49.00 – 47.55 (rotamers), 28.86, 27.04, 25.47 – 24.03 (rotamers).

IR (film) ν_{max} 2974.13, 2929.71, 2869.87, 1688.92, 1475.83, 1454.63, 1410.97, 1382.77, 1364.96, 1347.44, 1288.78, 1244.53, 1207.29, 1168.55, 1111.76, 1050.03, 999.99, 963.20, 897.92, 859.15, 776.65, 661.63, 569.13, 542.92, 498.72, 440.82, 406.84 cm^{-1}.

HRMS (ESI-TOF) *m/z* calcd. for $C_7H_{10}Br_2N^+$ ($[M-C_5H_9O_2+H]^+$) 265.9175, found 265.9172.

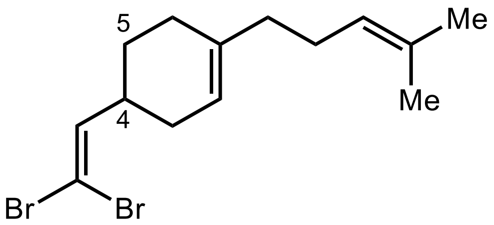

4/5-(2,2-dibromovinyl)-1-(4-methylpent-3-en-1-yl)cyclohex-1-ene (63-DiBr)

The title compound was prepared according to **General Procedure C** using carbon tetrabromide (6.714 g, 20.3 mmol, 2.25 equiv.), triphenylphosphine (10.62 g, 40.5 mmol, 4.5 equiv.), 3/4-(4-methylpent-3-en-1-yl)cyclohex-3-ene-1-carbaldehyde (1.86 mL, 9 mmol, 1.0 equiv., approx. 60:40 mixture of 4 and 3 isomers), and CH_2Cl_2 (81 mL + 10 mL). This mixture was allowed to warm to room temperature with stirring overnight. The solution was then poured into stirring hexane (~400 mL), filtered, and the filtrate concentrated under reduced pressure via rotary evaporation. The residue was purified by automated flash chromatography (100 g high performance silica column, 0-100% ethyl acetate/hexanes gradient) to provide the desired compound as a yellow oil (1.9700 g, 5.66 mmol, 63% yield). The product was isolated as an inseparable 63:37 mixture of 4- and 5- isomers (ratio determined by integration of the crude ^{1}H NMR and assigned by 2D NMR correlations). Spectral data represents the mixture of products.

^{1}H NMR (500 MHz, CDCl$_3$) δ 6.35 – 6.28 (m, 1H), 5.49 – 5.33 (m, 1H), 5.09 (ddq, *J* = 8.5, 5.7, 1.5 Hz, 1H), 2.66 – 2.46 (m, 1H), 2.16 – 1.74 (m, 9H), 1.69 (s, 3H), 1.61 (s, 3H), 1.52 – 1.40 (m, 1H).

^{13}C NMR (126 MHz, CDCl$_3$) δ 143.13, 143.08, 137.87, 136.05, 131.71, 131.68, 124.35, 124.28, 120.72, 118.99, 87.76, 87.72, 38.87, 38.45, 37.83, 37.80, 32.89, 29.88, 27.49, 27.36, 27.03, 26.52, 25.86, 24.22, 17.86, 17.85.

IR (film) ν_{max} 2964.96, 2913.30, 2835.34, 1607.51, 1436.24, 1375.32, 1265.32, 1217.08, 1200.42, 1137.39, 1105.75, 983.63, 916.01, 836.69, 784.56, 767.12, 695.70, 558.15, 492.81, 433.75 cm^{-1}.

HRMS (EI-QTOF) *m/z* calcd. for $C_{14}H_{20}Br_2^{\bullet+}$ ([$M^{\bullet+}$]) 345.9926, found 345.9929.

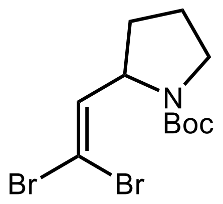

(±)-*tert*-butyl 2-(2,2-dibromovinyl)pyrrolidine-1-carboxylate (64-DiBr)

The title compound was prepared according to **General Procedure C** using carbon tetrabromide (6.714 g, 20.3 mmol, 2.25 equiv.), triphenylphosphine (10.62 g, 40.5 mmol, 4.5 equiv.), *tert*-butyl 2-formylpyrrolidine-1-carboxylate (1.7933 g, 9.00 mmol, 1.0 equiv.), and CH_2Cl_2 (81 mL + 10 mL). This mixture was allowed to warm to room temperature with stirring overnight. The solution was then poured into stirring hexane (~400 mL), filtered, and the filtrate concentrated under reduced pressure via rotary evaporation. The residue was purified by automated flash chromatography (100 g high performance silica column, 0-100% ethyl acetate/hexanes gradient) to provide the desired compounds as a white solid (1.8249 g, 5.14 mmol, 57% yield).

^{1}H NMR (500 MHz, CDCl$_3$) δ 6.36 (s, 1H), 4.36 (s, 1H), 3.53 – 3.31 (m, 2H), 2.17 (dq, *J* = 12.6, 7.4 Hz, 1H), 1.85 (pd, *J* = 6.7, 2.8 Hz, 2H), 1.73 (dq, *J* = 12.3, 6.2 Hz, 1H), 1.46 (s, 9H).

^{13}C NMR (126 MHz, CDCl$_3$) δ 154.47, 140.56, 88.39, 79.91, 59.65, 46.63, 31.94, 28.65, 23.84.

IR (film) ν_{max} 2975.33, 2929.73, 2883.27, 1674.69, 1608.71, 1475.51, 1447.35, 1396.61, 1361.36, 1309.23, 1274.08, 1252.93, 1160.93, 1105.27, 1007.62, 969.65, 908.66, 900.00, 880.24, 860.05, 791.19, 766.92, 744.31, 711.18, 617.35, 597.04, 568.16, 538.73, 489.04, 453.48, 414.18 cm^{-1}.

HRMS (ESI-TOF) *m/z* calcd. for $C_6H_{10}Br_2N^+$ ($[M-C_5H_9O_2+H]^+$) 253.9175, found 253.9172.

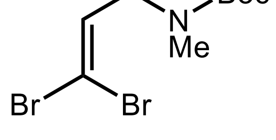

***tert*-butyl (3,3-dibromoallyl)(methyl)carbamate (65-DiBr)**

The title compound was prepared according to **General Procedure C** using carbon tetrabromide (6.714 g, 20.3 mmol, 2.25 equiv.), triphenylphosphine (10.62 g, 40.5 mmol, 4.5 equiv.), *tert*-butyl methyl(2-oxoethyl)carbamate (1.52 mL, 9 mmol, 1.0 equiv.), and CH_2Cl_2 (81 mL + 10 mL). This mixture was allowed to warm to room temperature with stirring overnight. The solution was then poured into stirring hexane (~400 mL), filtered, and the filtrate concentrated under reduced pressure via rotary evaporation. The residue was purified by automated flash chromatography (100 g high performance silica column, 0-100% ethyl acetate/hexanes gradient) to provide the desired compound as a pale yellow oil (2.18 g, 4.31 mmol, 48% yield).

^{1}H NMR (500 MHz, $CDCl_3$) δ 6.43 (s, 1H), 3.86 (s, 2H), 2.85 (s, 3H), 1.46 (s, 9H).

^{13}C NMR (126 MHz, $CDCl_3$) δ 155.48, 134.74, 91.38, 80.25, 51.96 – 49.23 (rotamers), 34.46, 28.55.

IR (film) ν_{max} 2975.34, 2930.35, 1690.99, 1619.33, 1478.36, 1449.95, 1419.29, 1388.42, 1365.25, 1297.38, 1228.57, 1174.05, 1142.64, 1048.27, 952.66, 880.55, 782.59, 669.43, 579.40, 520.70, 461.06, 433.64, 415.19 cm^{-1}.

HRMS (ESI-TOF) *m/z* calcd. for $C_4H_8Br_2N^+$ ($[M-C_5H_9O_2+H]^+$) 227.9018, found 227.9016.

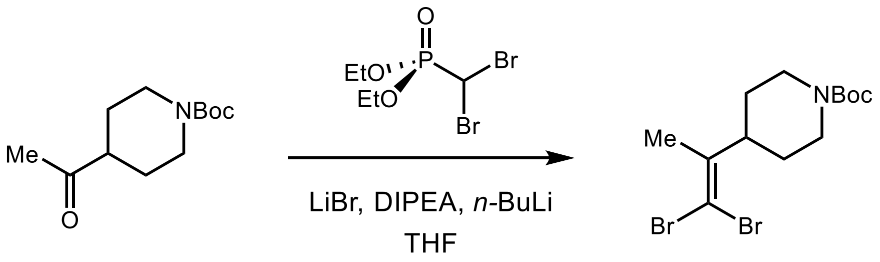

***tert*-butyl 4-(1,1-dibromoprop-1-en-2-yl)piperidine-1-carboxylate (66-DiBr)**

Lithium bromide (868.5 mg, 10 mmol, 2.0 equiv.) was dissolved in THF (25 mL, 0.20 M) under nitrogen atmosphere. N,N-diisopropylethylamine (0.71 mL, 5.0 mmol, 1.0 equiv.) was added dropwise at 0 °C. *n*-BuLi (2 mL of 2.5 M solution in hexanes, 5.0 mmol, 1.0 equiv.) was added dropwise at 0 °C. This solution was stirred at 0 °C for 40 minutes, before being cooled to -78 °C.

Diethyl (dibromomethyl)phosphonate (1.5496 g, 5.0 mmol, 1.0 equiv.) was dissolved in THF (10 mL, 0.50 M). This solution was added dropwise at -78 ºC and stirred for 30 minutes. After 30 minutes, *tert*-butyl 4-acetylpiperidine-1-carboxylate (1.08 mL, 5.0 mmol, 1.0 equiv.) was added via syringe. This reaction mixture was stirred at -78 ºC for 3 hours, before being allowed warm to room temperature over 1 hour. H_2O (10 mL) was added and the organic layer was extracted with Et_2O (3 x 15 mL). The organic layer was dried with $MgSO_4$, filtered, and the filtrate concentrated under reduced pressure via rotary evaporation. The residue was purified by automated flash chromatography (100 g high performance silica column, 0-100% ethyl acetate/hexanes gradient) to provide the desired product as a yellowish oil (1.0353 g, 2.702 mmol, 54% yield).

^{1}H NMR (500 MHz, $CDCl_3$) δ 4.17 (d, *J* = 13.2 Hz, 2H), 2.90 (tt, *J* = 12.0, 3.7 Hz, 1H), 2.83 – 2.64 (m, 2H), 1.77 (s, 3H), 1.56 – 1.51 (m, 2H), 1.47 – 1.38 (m, 11H).

^{13}C NMR (126 MHz, $CDCl_3$) δ 154.84, 143.91, 85.89, 79.69, 44.12, 43.99 (br), 29.00, 28.58, 18.91.

IR (film) ν_{max} 2974.59, 2928.66, 2852.67, 1689.10, 1476.35, 1418.94, 1363.88, 1306.69, 1275.51, 1235.42, 1216.93, 1161.27, 1121.19, 1088.66, 1032.24, 984.00, 938.47, 904.91, 863.76, 832.88, 819.61, 787.53, 767.86, 646.83, 529.80, 447.76, 415.76 cm^{-1}.

HRMS (ESI-TOF) *m/z* calcd. for $C_8H_{14}Br_2N^+$ ($[M-C_5H_9O_2+H]^+$) 281.9488, found 281.9488.

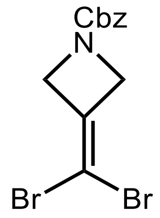

benzyl 3-(dibromomethylene)azetidine-1-carboxylate (67-DiBr)

The title compound was prepared according to **General Procedure C** using carbon tetrabromide (33.16 g, 100 mmol, 2.0 equiv.), triphenylphosphine (32.83 g, 125 mmol, 2.5 equiv.), benzyl 3-oxoazetidine-1-carboxylate (10.26 g, 50.0 mmol, 1.0 equiv.), and CH_2Cl_2 (20 mL + 50 mL). This mixture was allowed to warm to room temperature with stirring overnight. The solution was then

filtered directly and the filtrate concentrated under reduced pressure via rotary evaporation. The residue was purified by silica gel flash chromatography (4” diameter column, 25% ethyl acetate/hexanes) to provide the desired compounds as an off-white solid (11.32 g, 31.35 mmol, 63% yield).

^{1}H NMR (500 MHz, CDCl$_3$) δ 7.40 – 7.29 (m, 5H), 5.13 (s, 2H), 4.40 (s, 4H).

^{13}C NMR (126 MHz, CDCl$_3$) δ 156.33, 136.34, 135.53, 128.71, 128.41, 128.27, 81.49, 67.36, 59.18 (br).

IR (film) ν_{max} 2968.37, 2941.14, 2922.52, 2855.47, 1709.75, 1696.74, 1493.17, 1447.53, 1438.62, 1400.70, 1352.71, 1332.44, 1284.89, 1259.65, 1213.38, 1158.96, 1141.66, 1107.28, 1095.30, 1029.71, 982.95, 952.66, 927.84, 907.09, 826.26, 806.67, 764.82, 753.75, 699.28, 618.98, 607.00, 557.45, 512.35, 450.79 cm^{-1}.

HRMS (EI-QTOF) *m/z* calcd. for $C_{12}H_{11}Br_2NO_2^{\bullet+}$ ($[M]^{\bullet+}$) 358.9151, found 358.9154.

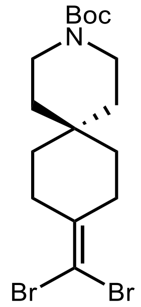

***tert*-butyl 9-(dibromomethylene)-3-azaspiro[5.5]undecane-3-carboxylate (68-DiBr)**

The title compound was prepared according to **General Procedure D** using carbon tetrabromide (5.0430 g, 15.0 mmol, 3.0 equiv.), triphenylphosphine (7.9770 g, 30.0 mmol, 6.0 equiv.), *tert*-butyl 9-oxo-3-azaspiro[5.5]undecane-3-carboxylate (1.3368 g, 5.00 mmol, 1.0 equiv.), and toluene (100 mL). This mixture was refluxed under nitrogen atmosphere for 48 hours. The solution was then poured into stirring hexane (~250 mL), filtered, and the filtrate concentrated under reduced pressure via rotary evaporation. The residue was purified by automated flash chromatography (100

g high performance silica column, 0-100% ethyl acetate/hexanes gradient) to provide the desired compounds as a white solid (1.4469 g, 3.42 mmol, 68% yield).

^{1}H NMR (500 MHz, CDCl$_3$) δ 3.45 – 3.34 (m, 4H), 2.43 – 2.35 (m, 4H), 1.49 – 1.41 (m, 17H).

^{13}C NMR (126 MHz, CDCl$_3$) δ 155.09, 144.30, 82.40, 79.51, 39.73 (br), 35.63, 35.22, 30.94, 29.72, 28.61.

IR (film) ν_{max} 2975.23, 2918.41, 2863.36, 1671.35, 1424.84, 1387.94, 1363.27, 1303.49, 1267.14, 1243.90, 1234.85, 1161.57, 1146.78, 1088.46, 1005.14, 985.73, 935.23, 900.03, 862.24, 823.55, 792.03, 779.65, 763.59, 650.72, 578.97, 527.62, 436.59 cm^{-1}.

HRMS (ESI-TOF) *m/z* calcd. for $C_{11}H_{18}Br_2N^+$ ([M-$C_5H_9O_2$+H]$^+$) 321.9801, found 321.9803.

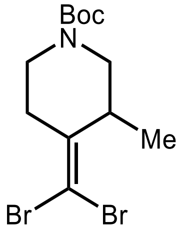

(±)-*tert*-butyl 4-(dibromomethylene)-3-methylpiperidine-1-carboxylate (69-DiBr)

The title compound was prepared according to **General Procedure D** using carbon tetrabromide (5.043 g, 15.0 mmol, 3.0 equiv.), triphenylphosphine (7.977 g, 30.0 mmol, 6.0 equiv.), (±)-*tert*-butyl 3-methyl-4-oxopiperidine-1-carboxylate (1.0664 g, 5.0 mmol, 1.0 equiv.), and heptane (100 mL). This mixture was refluxed under nitrogen atmosphere for 48 hours. The solution was then poured into stirring hexane (~250 mL), filtered, and the filtrate concentrated under reduced pressure via rotary evaporation. The residue was purified by automated flash chromatography (100 g high performance silica column, 0-100% ethyl acetate/hexanes gradient) to provide the desired compounds as an off-white solid (776.4 mg, 2.10 mmol, 42% yield).

^{1}H NMR (500 MHz, CDCl$_3$) δ 4.05 (d, *J* = 156.2 Hz, 2H), 3.10 (d, *J* = 9.5 Hz, 1H), 3.02 – 2.59 (m, 3H), 2.24 (ddd, *J* = 14.9, 12.3, 5.6 Hz, 1H), 1.47 (s, 9H), 1.08 (d, *J* = 7.1 Hz, 3H).

^{13}C NMR (126 MHz, CDCl$_3$) δ 155.20, 144.38, 84.87, 79.96, 49.55 – 47.37 (rotamers), 44.10 – 42.27 (rotamers), 37.98, 30.32, 28.55, 16.35.

IR (film) ν_{max} 2973.09, 2930.53, 2868.55, 1681.39, 1422.41, 1362.61, 1326.05, 1272.33, 1245.93, 1202.69, 1162.08, 1122.64, 1085.92, 1027.95, 981.21, 955.41, 901.90, 880.82, 861.24, 832.94, 793.95, 764.06, 717.86, 636.30, 530.31, 494.43, 463.85, 424.66 cm^{-1}.

HRMS (ESI-TOF) *m/z* calcd. for $C_7H_{12}Br_2N^+$ ($[M+H]^+$) 367.9855, found 367.9857.

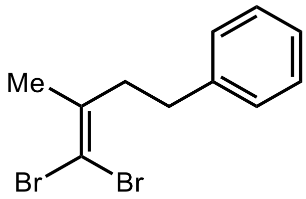

(4,4-dibromo-3-methylbut-3-en-1-yl)benzene (70-DiBr)

The title compound was prepared according to **General Procedure D** using carbon tetrabromide (4.9745 g, 15 mmol, 3.0 equiv.), triphenylphosphine (7.8687 g, 30.0 mmol, 6.0 equiv.), 4-phenylbutan-2-one (741.0 mg, 5.00 mmol, 1.0 equiv.), and heptane (100 mL). This mixture was refluxed under nitrogen atmosphere for 6 hours. The solution was then poured into stirring hexane (~100 mL), filtered, and the filtrate concentrated under reduced pressure via rotary evaporation. The residue was purified by automated flash chromatography (100 g high performance silica column, 0-100% ethyl acetate/hexanes gradient) to provide the desired compounds as a pale yellow oil (1.0130 g, 3.330 mmol, 67% yield).

^{1}H NMR (500 MHz, CDCl$_3$) δ 7.35 – 7.28 (m, 2H), 7.25 – 7.18 (m, 3H), 2.81 – 2.73 (m, 2H), 2.61 – 2.52 (m, 2H), 1.91 (s, 3H).

^{13}C NMR (126 MHz, CDCl$_3$) δ 141.52, 140.99, 128.62, 128.50, 126.33, 85.93, 40.40, 33.29, 23.20.

IR (film) ν_{max} 3025.70, 2924.54, 2860.29, 1600.79, 1494.60, 1453.43, 1373.24, 1214.39, 1150.54, 1078.90, 1030.36, 908.96, 812.45, 744.73, 696.10, 593.09, 539.10, 492.96, 473.10 cm^{-1}.

HRMS (EI-QTOF) *m/z* calcd. for $C_{11}H_{12}Br_2^{\bullet+}$ ($[M]^{\bullet+}$) 301.9300, found 301.9303.

8) Experimental Data for Stereoselective Alkene Synthesis

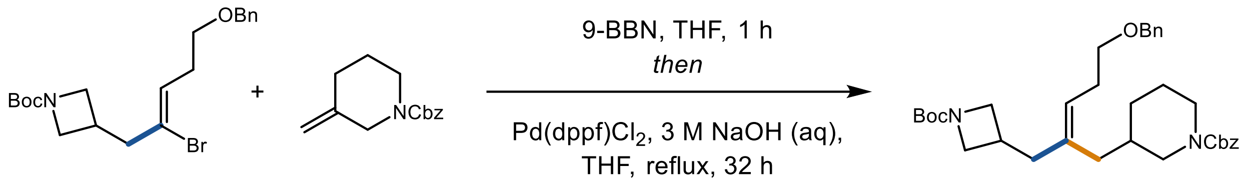

benzyl (*E*)-3-(5-(benzyloxy)-2-((1-(*tert*-butoxycarbonyl)azetidin-3-yl)methyl)pent-2-en-1-yl)piperidine-1-carboxylate (*(E)*-71)

A dry 8 mL vial was charged with a 0.5 M solution of 9-borabicyclo(3.3.1)nonane (2 mL, 1 mmol) under N_2 atmosphere at 0 °C. benzyl 3-methylenepiperidine-1-carboxylate (207.6 µL, 1 mmol) was added dropwise. The mixture was allowed to warm to room temperature with stirring for 1 hour under N_2 atmosphere. This yielded a 0.5 M stock solution of the 9-BBN adduct benzyl 3-((9-borabicyclo[3.3.1]nonan-9-yl)methyl)piperidine-1-carboxylate (**9-BBN Stock *(E)*-71**).

A dry round-bottom flask was charged with *tert*-butyl (*Z*)-3-(5-(benzyloxy)-2-bromopent-2-en-1-yl)azetidine-1-carboxylate (**33**, 102.6 mg, 0.25 mmol, 1.0 equiv.), [1,1′-bis(diphenylphosphino)ferrocene]dichloropalladium(II) (5.49 mg, 7.5 µmol, 0.003 equiv.), aqueous 3 M sodium hydroxide (0.30 mL, 0.9 mmol, 3.6 equiv.), and THF (1.5 mL). The flask was degassed and placed under N_2 atmosphere. **9-BBN Stock *(E)*-71** (0.55 mL, 0.275 mmol, 1.1 equiv.) was added dropwise via syringe. The reaction mixture was refluxed under N_2 atmosphere for 16 hours. After 16 hours, additional freshly prepared **9-BBN Stock *(E)*-71** (0.55 mL, 0.275 mmol, 1.1 equiv.) and [1,1′-bis(diphenylphosphino)ferrocene]dichloropalladium(II) (5.49 mg, 7.5 µmol, 0.003 equiv.) were added and the mixture was refluxed for an additional 16 hours.

Diethyl ether (10 mL) was added to the crude reaction mixture. The organic layer was extracted, washed with brine, dried with $MgSO_4$, and filtered. The filtrate was concentrated via rotary evaporation and then purified by automated flash chromatography (25 g high performance silica column, 0-100% ethyl acetate/hexanes gradient) to yield impure product. This was further purified by preparative HPLC (40-90% MeCN in water with a 0.1% NH_4OH modifier) to provide the

desired compound as a yellow oil (73.4 mg, 0.130 mmol, 52% yield). *E:Z* ratio determined to be > 20:1 by quantitative ^{13}C NMR.

^{1}H NMR (500 MHz, $CDCl_3$) δ 7.40 – 7.26 (m, 10H), 5.18 – 5.06 (m, 3H), 4.49 (s, 2H), 4.13 – 3.89 (m, 4H), 3.49 (dt, *J* = 8.7, 4.6 Hz, 2H), 3.45 – 3.34 (m, 2H), 2.73 (t, *J* = 13.4 Hz, 1H), 2.68 – 2.56 (m, 1H), 2.47 – 2.16 (m, 5H), 1.90 (s, 2H), 1.81 – 1.72 (m, 1H), 1.66 – 1.54 (m, 2H), 1.44 (s, 9H), 1.32 – 1.19 (m, 1H), 1.07 – 0.95 (m, 1H).

^{13}C NMR (126 MHz, $CDCl_3$) δ 156.44, 155.27, 138.49, 137.05, 135.80, 128.53, 128.45, 127.99, 127.86, 127.71, 127.65, 123.55, 79.26, 73.02, 70.02, 66.98, 56.05, 54.37 (d, *J* = 107.5 Hz), 50.03, 44.70, 41.50, 34.80 (br), 34.52, 30.98, 28.84, 28.52, 26.97, 25.26 (d, *J* = 32.2 Hz).

IR (film) ν_{max} 2972.88, 2929.93, 2895.19, 2876.97, 2852.00, 1685.74, 1559.43, 1496.41, 1467.80, 1452.68, 1404.81, 1364.85, 1312.68, 1288.02, 1257.37, 1236.41, 1135.29, 1100.40, 1060.71, 1028.17, 964.47, 907.21, 856.82, 806.61, 766.06, 725.80, 696.30, 645.64, 604.67, 559.61, 511.60, 502.08, 461.03, 421.84, 415.12, 408.23, 403.42 cm^{-1}.

HRMS (ESI-TOF) *m/z* calcd. for $C_{29}H_{39}N_2O_3^+$ ($[M-C_5H_9O_2+H]^+$) 463.2955, found 463.2956.

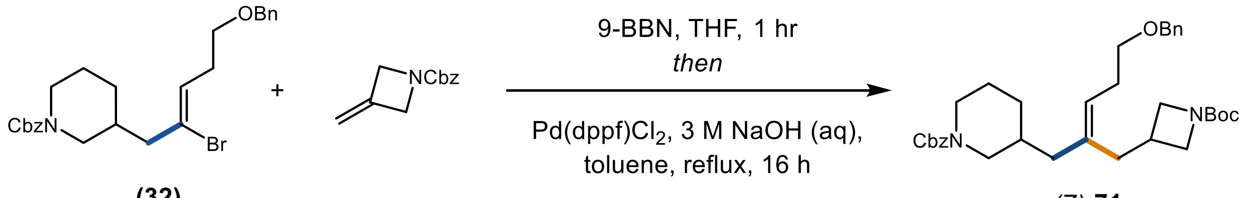

(32) **(Z)-71**

benzyl (*Z*)-3-(5-(benzyloxy)-2-((1-(*tert*-butoxycarbonyl)azetidin-3-yl)methyl)pent-2-en-1-yl)piperidine-1-carboxylate (*(Z)*-71)

A dry 8 mL vial was charged with a 0.5 M solution of 9-borabicyclo(3.3.1)nonane (2 mL, 1 mmol) under N_2 atmosphere at 0 °C. Benzyl 3-methyleneazetidine-1-carboxylate (166 µL, 1 mmol) was added dropwise. The mixture was allowed to warm to room temperature with stirring for 1 hour

under N_2 atmosphere. This yielded a 0.5 M stock solution of the 9-BBN adduct benzyl benzyl 3-((9-borabicyclo[3.3.1]nonan-9-yl)methyl)azetidine-1-carboxylate (**9-BBN Stock *(Z)*-71**).

A dry round-bottom flask was charged with benzyl *(Z)*-3-(5-(benzyloxy)-2-bromopent-2-en-1-yl)piperidine-1-carboxylate (**32**, 118.11 mg, 0.25 mmol, 1.0 equiv.), [1,1′-bis(diphenylphosphino)ferrocene]dichloropalladium(II) (8.3 mg, 0.025 mmol, 0.01 equiv.), aqueous 3 M sodium hydroxide (0.35 mL, 1.05 mmol, 4.2 equiv.), and toluene (1.6 mL). The flask was degassed and placed under N_2 atmosphere. **9-BBN Stock *(Z)*-71** (1.25 mL, 0.625 mmol, 2.5 equiv.) was added dropwise via syringe. The reaction mixture was heated to 90 °C under N_2 atmosphere for 16 hours.

Diethyl ether (10 mL) was added to the crude reaction mixture. The organic layer was extracted, washed with brine, dried with $MgSO_4$, and filtered. The filtrate was concentrated via rotary evaporation and then purified by automated flash chromatography (25 g high performance silica column, 0-100% ethyl acetate/hexanes gradient) to yield impure product. This was further purified by preparative HPLC (40-90% MeCN in water with a 0.1% NH_4OH modifier) to provide the desired compound as a yellow oil (64.3 mg, 0.114 mmol, 46% yield). *Z:E* ratio determined to be > 20:1 by quantitative ^{13}C NMR.

^1H NMR (500 MHz, $CDCl_3$) δ 7.40 – 7.24 (m, 10H), 5.23 (t, *J* = 7.3 Hz, 1H), 5.16 – 5.06 (m, 2H), 4.59 – 4.40 (m, 2H), 4.02 (d, *J* = 13.5 Hz, 2H), 3.94 (t, *J* = 8.4 Hz, 2H), 3.55 – 3.48 (m, 2H), 3.44 (t, *J* = 11.8 Hz, 2H), 2.79 (td, *J* = 12.5, 3.0 Hz, 1H), 2.63 – 2.57 (m, 1H), 2.37 (s, 2H), 2.28 (s, 2H), 1.92 – 1.82 (m, 1H), 1.82 – 1.72 (m, 2H), 1.63 (d, *J* = 12.6 Hz, 1H), 1.56 (s, 2H), 1.44 (s, 9H), 1.26 (s, 1H), 1.01 (s, 1H).

^{13}C NMR (126 MHz, $CDCl_3$) δ 156.44, 155.29, 138.51, 137.04, 135.54, 128.52, 128.42, 127.98, 127.82, 127.69, 127.61, 125.00, 79.29, 72.99, 70.09, 66.97, 56.03, 54.28, 49.92, 44.77, 41.09, 34.18, 33.95, 30.88, 28.76, 28.49, 27.67.

IR (film) ν_{max} 3065.37, 3028.65, 3001.59, 2923.27, 2875.56, 2848.70, 1691.67, 1606.74, 1585.89, 1552.13, 1496.43, 1466.80, 1452.30, 1425.98, 1390.23, 1363.27, 1347.79, 1309.89, 1287.57,

1256.07, 1234.30, 1130.79, 1100.18, 1027.48, 964.42, 930.40, 914.89, 855.66, 821.28, 764.45, 734.18, 696.14, 604.66, 560.94, 511.04, 460.45, 431.38, 420.18, 411.84 cm^{-1}.

HRMS (ESI-TOF) *m/z* calcd. for $C_{29}H_{39}N_2O_3^+$ ($[M-C_5H_9O_2+H]^+$) 463.2955, found 463.2956.

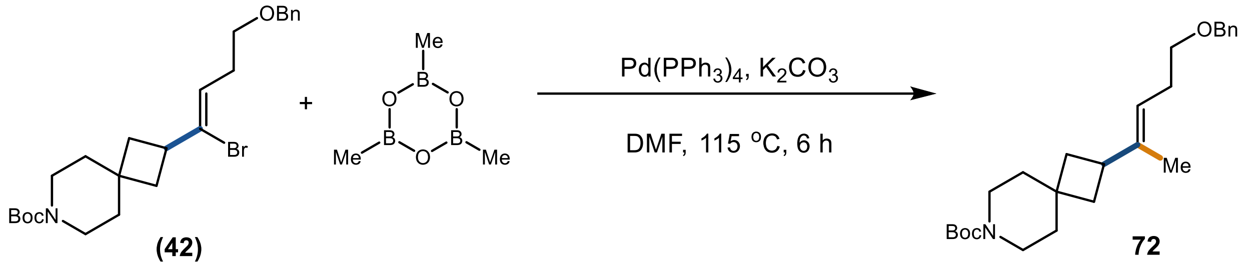

***tert*-butyl (*E*)-2-(5-(benzyloxy)pent-2-en-2-yl)-7-azaspiro[3.5]nonane-7-carboxylate (72)**

A dry 8 mL vial was charged with *tert*-butyl (*Z*)-2-(4-(benzyloxy)-1-bromobut-1-en-1-yl)-7-azaspiro[3.5]nonane-7-carboxylate (**42**, 116.1 mg, 0.25 mmol, 1.0 equiv.), trimethylboroxine (40.2 µL, 0.288 mmol, 1.15 equiv.), tetrakis(triphenylphosphine)palladium(0) (28.9 mg, 0.025 mmol, 0.10 equiv.), potassium carbonate (103.6 mg, 0.750 mmol, 3.0 equiv.), and DMF (1.0 mL). The vial was sparged with N_2 for 15 minutes. The reaction mixture was heated to 115 °C under N_2 atmosphere for 6 hours. The reaction mixture was then left to cool to room temperature with stirring overnight.

The crude reaction mixture was filtered through a celite plug, and the plug was washed with THF (25 mL). The filtrate was concentrated via rotary evaporation and then purified by automated flash chromatography (25 g high performance silica column, 0-100% ethyl acetate/hexanes gradient) to yield impure product. This was further purified by preparative HPLC (40-90% MeCN in water with a 0.1% NH_4OH modifier) to provide the desired compound as a yellow oil (67.2 mg, 0.168 mmol, 67% yield). *E:Z* ratio determined to be > 20:1 by the vinylic proton in ^{1}H NMR.

^{1}H NMR (500 MHz, $CDCl_3$) δ 7.36 – 7.25 (m, 5H), 5.11 (t, *J* = 7.0 Hz, 1H), 4.52 (s, 2H), 3.46 (t, *J* = 7.1 Hz, 2H), 3.36 (t, *J* = 5.8 Hz, 2H), 3.26 (t, *J* = 5.7 Hz, 2H), 2.83 (q, *J* = 9.0 Hz, 1H), 2.35 (q, *J* = 7.1 Hz, 2H), 1.94 (td, *J* = 8.9, 2.6 Hz, 2H), 1.75 – 1.58 (m, 5H), 1.56 (s, 3H), 1.45 (s, 9H), 1.41 (t, *J* = 5.7 Hz, 2H).

^{13}C NMR (126 MHz, CDCl$_3$) δ 155.12, 140.31, 138.71, 128.45, 127.73, 127.61, 118.01, 79.30, 72.94, 70.20, 40.94 (br), 39.44, 36.36, 36.06, 33.30, 28.59, 14.16.

IR (film) ν_{max} 3027.68, 3001.10, 2970.48, 2917.26, 2846.31, 1687.37, 1494.81, 1476.95, 1463.44, 1452.61, 1417.78, 1390.78, 1363.21, 1297.12, 1269.06, 1240.56, 1173.40, 1144.19, 1113.03, 1100.91, 1027.96, 998.14, 968.89, 924.44, 910.55, 892.32, 862.07, 822.22, 768.14, 733.72, 696.36, 632.94, 611.90, 563.52, 552.94, 528.67, 460.60, 430.77, 406.80 cm^{-1}.

HRMS (ESI-TOF) *m/z* calcd. for $C_{20}H_{30}NO^+$ ($[M-C_5H_9O_2+H]^+$) 300.2322, found 300.2323.

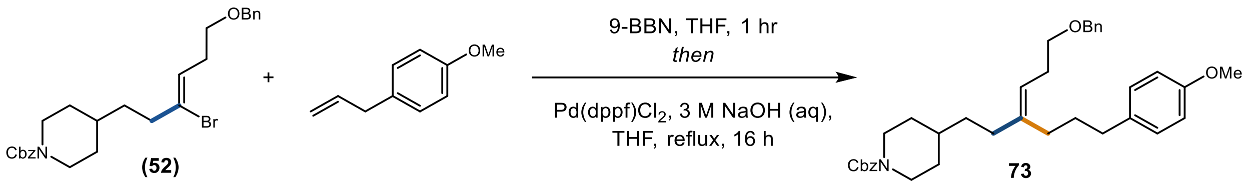

benzyl (*E*)-3-(5-(benzyloxy)-2-((1-(*tert*-butoxycarbonyl)azetidin-3-yl)methyl)pent-2-en-1-yl)piperidine-1-carboxylate (73)

A dry 8 mL vial was charged with a 0.5 M solution of 9-borabicyclo(3.3.1)nonane (2 mL, 1 mmol) under N_2 atmosphere at 0 °C. 1-allyl-4-methoxybenzene (156.7 µL, 1 mmol) was added dropwise. The mixture was allowed to warm to room temperature with stirring for 1 hour under N_2 atmosphere. This yielded a 0.5 M stock solution of the 9-BBN adduct 9-(3-(4-methoxyphenyl)propyl)-9-borabicyclo[3.3.1]nonane (**9-BBN Stock 73**).

A dry round-bottom flask was charged with benzyl *(Z)*-4-(6-(benzyloxy)-3-bromohex-3-en-1-yl)piperidine-1-carboxylate (**52**, 121.6 mg, 0.25 mmol, 1.0 equiv.), [1,1′-bis(diphenylphosphino)ferrocene]dichloropalladium(II) (5.49 mg, 7.5 µmol, 0.003 equiv.), aqueous 3 M sodium hydroxide (0.30 mL, 0.9 mmol, 3.6 equiv.), and THF (1.5 mL). The flask was degassed and placed under N_2 atmosphere. **9-BBN Stock 73** (0.55 mL, 0.275 mmol, 1.1 equiv.) was added dropwise via syringe. The reaction mixture was refluxed under N_2 atmosphere for 16 hours.

Diethyl ether (10 mL) was added to the crude reaction mixture. The organic layer was extracted, washed with brine, dried with $MgSO_4$, and filtered. The filtrate was concentrated via rotary evaporation and then purified by automated flash chromatography (25 g high performance silica column, 0-100% ethyl acetate/hexanes gradient) to yield impure product. This was further purified by preparative HPLC (40-90% MeCN in water with a 0.1% NH_4OH modifier) to provide the desired compound as a yellow oil (110.4 mg, 0.199 mmol, 80% yield). *E:Z* ratio determined to be > 20:1 by the vinylic proton in ^{1}H NMR.

^{1}H NMR (500 MHz, $CDCl_3$) δ 7.36 (d, *J* = 4.5 Hz, 4H), 7.33 (d, *J* = 4.6 Hz, 4H), 7.32 – 7.27 (m, 2H), 7.10 – 7.05 (m, 2H), 6.84 – 6.80 (m, 2H), 5.17 – 5.10 (m, 3H), 4.50 (s, 2H), 4.20 – 4.08 (m, 2H), 3.78 (s, 3H), 3.43 (t, *J* = 7.1 Hz, 2H), 2.73 (t, *J* = 12.2 Hz, 2H), 2.53 (t, *J* = 7.8 Hz, 2H), 2.31 (q, *J* = 7.1 Hz, 2H), 2.08 – 1.96 (m, 4H), 1.70 – 1.59 (m, 4H), 1.39 – 1.25 (m, 3H), 1.14 – 1.02 (m, 2H).

^{13}C NMR (126 MHz, $CDCl_3$) δ 157.85, 155.44, 141.25, 138.70, 137.17, 134.61, 129.34, 128.60, 128.50, 128.03, 127.95, 127.76, 127.67, 120.91, 113.85, 72.99, 70.45, 67.05, 55.39, 44.38, 35.75, 35.18, 35.03, 33.93, 32.21, 30.54, 29.96, 28.59.

IR (film) ν_{max} 3029.31, 2917.60, 2847.23, 1694.41, 1610.56, 1510.85, 1497.01, 1426.95, 1385.87, 1360.91, 1299.60, 1275.00, 1238.56, 1173.95, 1075.02, 1028.04, 963.55, 827.16, 732.93, 695.61, 604.00, 555.37, 516.27, 457.31, 408.87 cm^{-1}.

HRMS (ESI-TOF) *m/z* calcd. for $C_{36}H_{45}NNaO_4^+$ ($[M+Na]^+$) 578.3241, found 578.3242.

9) Experimental Data for Iterative Tetrasubstituted Alkene Synthesis

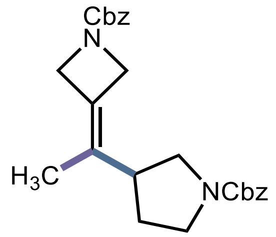

(±)-benzyl 3-(1-(1-((benzyloxy)carbonyl)azetidin-3-ylidene)ethyl)pyrrolidine-1-carboxylate (74)

The title compound was prepared according to **General Procedure E** using **NHC** (276.7 mg, 0.70 mmol, 1.4 equiv.), methanol (26.3 µL, 0.65 mmol, 1.3 equiv.), pyridine (56.6 µL, 0.70 mmol, 1.4 equiv.), and *t*-BuOMe (5 mL) for **Stock A** and Ir[(ppy)$_2$(dtbbpy)]PF_6 (6.9 mg, 7.5 µmol, 0.015 equiv.), $NiBr_2$(dtbbpy) (24.3 mg, 50.0 µmol, 0.10 equiv.), quinuclidine (150.0 mg, 1.35 mmol, 2.7 equiv.), phthalimide (73.6 mg, 0.50 mmol, 1.0 equiv.), benzyl 3-(dibromomethylene)azetidine-1-carboxylate (180.5 mg, 0.50 mmol, 1.0 equiv.), and 1,2-dichlorobenzene (10 mL) for **Stock B**. **Stock C** was made using **NHC** (375.5 mg, 0.95 mmol, 1.9 equiv.), **(±)-**benzyl 3-hydroxypyrrolidine-1-carboxylate (194.7 mg, 0.88 mmol, 1.75 equiv.), pyridine (76.8 µL, 0.95 mmol, 1.9 equiv.), and *t*-BuOMe (5 mL). The crude mixture was concentrated via *Genevac* then purified by automated flash chromatography (25 g high performance silica column, 0-25% ethyl acetate/hexanes gradient) to yield impure product. This was further purified by preparative HPLC (30-70% MeCN in water with a 0.1% NH_4OH modifier) to provide the desired compound as a yellow oil (98.6 mg, 0.235 mmol, 47% yield, 69% yield per step).

^{1}H NMR (500 MHz, $CDCl_3$) δ 7.41 – 7.32 (m, 10H), 5.15 – 5.11 (m, 4H), 4.59 – 4.48 (m, 4H), 3.66 – 3.50 (m, 2H), 3.34 (dq, *J* = 18.6, 8.4 Hz, 1H), 3.14 (dt, *J* = 16.4, 10.2 Hz, 1H), 2.75 (s, 1H), 1.99 – 1.72 (m, 2H), 1.50 (s, 3H).

^{13}C NMR (126 MHz, $CDCl_3$) δ 156.58, 154.91, 137.08, 137.01, 136.78, 128.64, 128.61, 128.21, 128.13, 128.10, 128.03, 127.19, 127.16, 123.43, 66.95, 66.90, 58.45 – 55.98 (br), 50.15 – 47.11 (rotamers), 46.73 – 44.76 (rotamers), 43.55 – 39.84 (rotamers), 31.57 – 26.55 (rotamers), 16.06 – 12.05 (rotamers).

IR (film) ν_{max} 3059.12, 3033.10, 2936.60, 2860.72, 1693.05, 1497.06, 1402.72, 1347.05, 1238.16, 1210.41, 1157.77, 1109.85, 1028.23, 972.36, 914.42, 875.51, 765.43, 734.54, 695.03, 604.97, 554.64, 455.27, 411.07 cm^{-1}.

HRMS (ESI-TOF) *m/z* calcd. for $C_{25}H_{29}N_2O_4^+$ ($[M+H]^+$) 421.2122, found 421.2122.

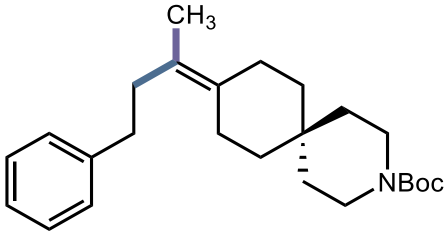

***tert*-butyl 9-(4-phenylbutan-2-ylidene)-3-azaspiro[5.5]undecane-3-carboxylate (75)**

The title compound was prepared according to **General Procedure E** using **NHC** (276.7 mg, 0.70 mmol, 1.4 equiv.), methanol (26.3 µL, 0.65 mmol, 1.3 equiv.), pyridine (56.6 µL, 0.70 mmol, 1.4 equiv.), and *t*-BuOMe (5 mL) for **Stock A** and Ir[(ppy)$_2$(dtbbpy)]PF_6 (6.9 mg, 7.5 µmol, 0.015 equiv.), $NiBr_2$(dtbbpy) (24.3 mg, 50.0 µmol, 0.10 equiv.), quinuclidine (150.0 mg, 1.35 mmol, 2.7 equiv.), phthalimide (73.6 mg, 0.50 mmol, 1.0 equiv.), *tert*-butyl 9-(dibromomethylene)-3-azaspiro[5.5]undecane-3-carboxylate (211.6 mg, 0.50 mmol, 1.0 equiv.), and 1,2-dichlorobenzene (10 mL) for **Stock B**. **Stock C** was made using **NHC** (375.5 mg, 0.95 mmol, 1.9 equiv.), 2-phenylethan-1-ol (107.5 mg, 0.88 mmol, 1.75 equiv.), pyridine (76.8 µL, 0.95 mmol, 1.9 equiv.), and *t*-BuOMe (5 mL). The crude mixture was concentrated via *Genevac* then purified by automated flash chromatography (25 g high performance silica column, 0-25% ethyl acetate/hexanes gradient) to yield impure product. This was further purified by preparative HPLC (30-70% MeCN in water with a 0.1% NH_4OH modifier) to provide the desired compound as a yellow oil (83.3 mg, 0.217 mmol, 43% yield, 66% yield per step).

^{1}H NMR (500 MHz, $CDCl_3$) δ 7.29 – 7.24 (m, 2H), 7.20 – 7.12 (m, 3H), 3.36 (td, *J* = 5.4, 3.1 Hz, 4H), 2.64 (dd, *J* = 9.2, 6.6 Hz, 2H), 2.33 (dd, *J* = 9.1, 6.7 Hz, 2H), 2.16 – 2.00 (m, 4H), 1.69 (s, 3H), 1.45 (s, 9H), 1.40 – 1.33 (m, 6H), 1.23 – 1.15 (m, 2H).

^{13}C NMR (126 MHz, $CDCl_3$) δ 155.07, 142.46, 132.89, 128.51, 128.21, 125.68, 124.13, 79.14, 39.66 (br), 36.86, 36.81, 36.18, 35.58, 34.95, 31.03, 28.51, 25.12, 24.74, 18.10.

IR (film) ν_{max} 3025.36, 3002.49, 2970.21, 2912.44, 2842.30, 1688.47, 1602.96, 1494.46, 1473.71, 1418.34, 1390.62, 1363.03, 1302.11, 1277.92, 1241.60, 1209.51, 1155.32, 1092.82, 1051.76, 1029.41, 995.85, 981.76, 927.70, 899.29, 862.00, 821.36, 766.14, 746.72, 713.29, 697.24, 565.41, 522.82, 490.56, 461.59, 433.39, 421.35, 415.21, 408.55 cm^{-1}.

HRMS (ESI-TOF) *m/z* calcd. for $C_{20}H_{30}N^+$ ($[M-C_5H_9O_2+H]^+$) 284.2373, found 284.2373.

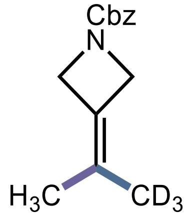

benzyl 3-(propan-2-ylidene-1,1,1-d3)azetidine-1-carboxylate (76)

The title compound was prepared according to **General Procedure E** using **NHC** (276.7 mg, 0.70 mmol, 1.4 equiv.), methanol (26.3 µL, 0.65 mmol, 1.3 equiv.), pyridine (56.6 µL, 0.70 mmol, 1.4 equiv.), and *t*-BuOMe (5 mL) for **Stock A** and $Ir[(ppy)_2(dtbbpy)]PF_6$ (6.9 mg, 7.5 µmol, 0.015 equiv.), $NiBr_2(dtbbpy)$ (24.3 mg, 50.0 µmol, 0.10 equiv.), quinuclidine (150.0 mg, 1.35 mmol, 2.7 equiv.), phthalimide (73.6 mg, 0.50 mmol, 1.0 equiv.), benzyl 3-(dibromomethylene)azetidine-1-carboxylate (180.5 mg, 0.50 mmol, 1.0 equiv.), and 1,2-dichlorobenzene (10 mL) for **Stock B**. **Stock C** was made using **NHC** (375.5 mg, 0.95 mmol, 1.9 equiv.), methanol-d_4 (35.7 µL, 0.88 mmol, 1.75 equiv.), pyridine (76.8 µL, 0.95 mmol, 1.9 equiv.), and *t*-BuOMe (5 mL). The crude mixture was concentrated via *Genevac* then purified by automated flash chromatography (25 g high performance silica column, 0-25% ethyl acetate/hexanes gradient) to yield impure product. This was further purified by preparative HPLC (30-70% MeCN in water with a 0.1% NH_4OH modifier) to provide the desired compound as a yellow oil (64.4 mg, 0.275 mmol, 55% yield, 74% yield per step).

^{1}H NMR (500 MHz, CDCl$_3$) δ 7.43 – 7.27 (m, 5H), 5.12 (s, 2H), 4.49 (q, *J* = 1.8 Hz, 4H), 1.53 (p, *J* = 1.6 Hz, 3H).

^{13}C NMR (126 MHz, CDCl$_3$) δ 156.71, 136.95, 128.60, 128.12, 128.06, 125.11, 120.25, 66.79, 57.00 (br), 18.87, 18.82.

IR (film) ν_{max} 3035.80, 2944.21, 1691.03, 1498.17, 1410.03, 1327.07, 1211.94, 1160.59, 1075.69, 966.19, 735.35, 695.84, 608.66, 455.42 cm^{-1}.

HRMS (ESI-TOF) *m/z* calcd. for $C_{14}H_{15}D_3NO_2^+$ ($[M+H]^+$) 235.1520, found 235.1520.

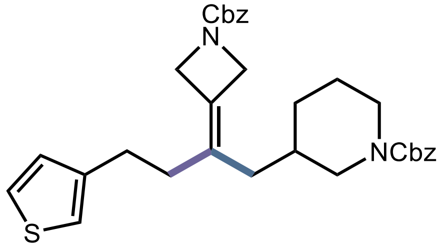

(±)-benzyl 3-(2-(1-((benzyloxy)carbonyl)azetidin-3-ylidene)-4-(thiophen-3-yl)butyl)piperidine-1-carboxylate (77)

The title compound was prepared according to **General Procedure E** using **NHC** (276.7 mg, 0.70 mmol, 1.4 equiv.), 2-(thiophen-3-yl)ethan-1-ol (83.3 mg, 0.65 mmol, 1.3 equiv.), pyridine (56.6 µL, 0.70 mmol, 1.4 equiv.), and *t*-BuOMe (5 mL) for **Stock A** and Ir[(ppy)$_2$(dtbbpy)]PF$_6$ (6.9 mg, 7.5 µmol, 0.015 equiv.), NiBr$_2$(dtbbpy) (24.3 mg, 50.0 µmol, 0.10 equiv.), quinuclidine (150.0 mg, 1.35 mmol, 2.7 equiv.), phthalimide (73.6 mg, 0.50 mmol, 1.0 equiv.), benzyl 3-(dibromomethylene)azetidine-1-carboxylate (180.5 mg, 0.50 mmol, 1.0 equiv.), and 1,2-dichlorobenzene (10 mL) for **Stock B**. **Stock C** was made using **NHC** (375.5 mg, 0.95 mmol, 1.9 equiv.), (±)-benzyl 3-(hydroxymethyl)piperidine-1-carboxylate (219.4 mg, 0.88 mmol, 1.75 equiv.), pyridine (76.8 µL, 0.95 mmol, 1.9 equiv.), and *t*-BuOMe (5 mL). The crude mixture was concentrated via *Genevac* then purified by automated flash chromatography (25 g high performance silica column, 0-25% ethyl acetate/hexanes gradient) to yield impure product. This was further purified by preparative HPLC (30-70% MeCN in water with a 0.1% NH_4OH modifier)

to provide the desired compound as a yellow oil (176.9 mg, 0.275 mmol, 65% yield, 81% yield per step).

^{1}H NMR (500 MHz, CDCl$_3$) δ 7.35 – 7.29 (m, 10H), 7.23 (dd, *J* = 4.9, 3.0 Hz, 1H), 6.97 – 6.71 (m, 2H), 5.11 (d, *J* = 5.0 Hz, 4H), 4.46 (d, *J* = 2.6 Hz, 2H), 4.35 (s, 2H), 4.04 (d, *J* = 12.4 Hz, 2H), 2.81 – 2.75 (m, 1H), 2.71 – 2.59 (m, 2H), 2.43 (t, *J* = 11.7 Hz, 1H), 2.17 (s, 1H), 1.79 (dd, *J* = 14.5, 5.1 Hz, 3H), 1.66 – 1.55 (m, 3H), 1.47 – 1.41 (m, 1H), 1.04 – 0.98 (m, 1H).

^{13}C NMR (126 MHz, CDCl$_3$) δ 156.51, 155.35, 141.77, 137.03, 136.80, 129.87, 128.63, 128.62, 128.21, 128.19, 128.15, 128.11, 127.94, 125.68, 124.04, 120.50, 67.16, 66.91, 57.14, 56.90, 49.99, 44.74, 34.89, 34.28, 31.84, 31.11, 28.20, 25.13.

IR (film) ν_{max} 3062.54, 3030.32, 2915.55, 2852.59, 1690.61, 1585.51, 1497.10, 1466.82, 1406.65, 1347.53, 1288.14, 1256.12, 1231.65, 1149.72, 1114.23, 1027.58, 964.56, 913.51, 854.24, 832.71, 733.18, 695.08, 604.93, 560.95, 455.22 cm^{-1}.

HRMS (ESI-TOF) *m/z* calcd. for $C_{32}H_{36}N_2NaO_4S^+$ ([M+Na]$^+$) 567.2288, found 567.2287.

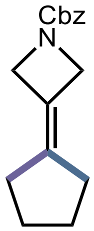

benzyl 3-cyclopentylideneazetidine-1-carboxylate (78)

The title compound was prepared according to **General Procedure C** using **NHC** (434.8 mg, 1.10 mmol, 2.2 equiv.), 1,4-butanediol (44.2 µL, 0.50 mmol, 1.0 equiv.), pyridine (69.3 µL, 1.10 mmol, 2.2 equiv.), and *t*-BuOMe (5 mL) for **Stock A** and Ir[(ppy)$_2$(dtbbpy)]PF$_6$ (6.9 mg, 7.5 µmol, 0.015 equiv.), NiBr$_2$(dtbbpy) (24.3 mg, 50.0 µmol, 0.10 equiv.), quinuclidine (122.3 mg, 1.10 mmol, 2.2 equiv.), phthalimide (73.6 mg, 0.50 mmol, 1.0 equiv.), **67-DiBr** (315.90 mg, 0.875 mmol, 1.75 equiv.), and 1,2-dichlorobenzene (10 mL) for **Stock B**. The crude mixture was concentrated via *Genevac* then purified by automated flash chromatography (25 g high performance silica column,

0-25% ethyl acetate/hexanes gradient) to yield impure product. This was further purified by preparative HPLC (30-70% MeCN in water with a 0.1% NH_4OH modifier) to provide the desired compound as a white solid (82.3 mg, 0.160 mmol, 32% yield).

^{1}H NMR (500 MHz, $CDCl_3$) δ 7.41 – 7.28 (m, 5H), 5.12 (s, 2H), 4.53 – 4.41 (m, 4H), 2.08 (s, 4H), 1.66 (ddd, *J* = 7.3, 4.4, 3.0 Hz, 4H).

^{13}C NMR (126 MHz, $CDCl_3$) δ 156.72, 136.98, 136.07, 128.60, 128.11, 128.06, 116.68, 66.78, 56.81 – 58.45 (rotamers), 29.75, 26.72.

IR (film) ν_{max} 2958.56, 2923.84, 2907.23, 2851.14, 1691.42, 1495.52, 1450.40, 1409.69, 1358.76, 1331.77, 1316.12, 1297.10, 1284.49, 1261.06, 1209.76, 1162.41, 1147.18, 1108.08, 1098.88, 1020.86, 969.55, 918.14, 767.71, 748.94, 697.20, 607.79, 565.56, 477.86 cm^{-1}.

HRMS (ESI-TOF) *m/z* calcd. for $C_{16}H_{20}NO_2^+$ ($[M+H]^+$) 258.1489, found 258.1493.

10) Experimental Data for Total Synthesis of (+)-sponalisolide B

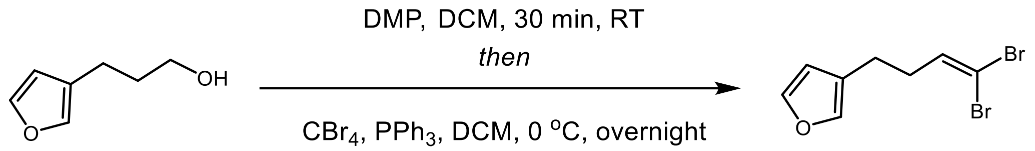

80-DiBr

3-(4,4-dibromobut-3-en-1-yl)furan (80-DiBr)

3-(furan-3-yl)propan-1-ol was synthesized according to the method of Bower et al.[4] 3-(furan-3-yl)propan-1-ol (620.69 mg, 5 mmol, 1 equiv.) was dissolved in DCM (12 mL) in a dry 40 mL vial. Dess-Martin periodinane (2.1207 g, 5.5 mmol, 1.1 equiv.) was added slowly. The solution was stirred for 30 minutes. The reaction mixture was then quenched with addition of a solution of saturated sodium thiosulfate in water ($Na_2S_2O_3$, 10 mL). The mixture was then washed with saturated sodium bicarbonate in water. The extracted organic layer was dried with Na_2SO_4, filtered, and concentrated under reduced pressure via rotary evaporation. The aldehyde (3-(furan-3-yl)propanal) was then used without further purification.

The title compound was then prepared according to **General Procedure C** using carbon tetrabromide (3.73 g, 11.25 mmol, 2.25 equiv.), triphenylphosphine (5.90 g, 22.5 mmol, 4.5 equiv.), 3-(furan-3-yl)propanal, and CH_2Cl_2 (45 mL + 5 mL). This mixture was allowed to warm to room temperature with stirring overnight. The solution was then poured into stirring hexane (~200 mL), filtered, and the filtrate concentrated under reduced pressure via rotary evaporation. The residue was purified by automated flash chromatography (50 g high performance silica column, 0-100% ethyl acetate/hexanes gradient) to provide the desired compound as a yellow oil (905.4 mg, 3.235 mmol, 65% yield). Although decomposition of the remaining material precluded complete NMR characterization, its identity was supported by IR, HRMS, and successful conversion in the synthesis of (+)-sponalisolide B.

IR (film) ν_{max} 2922.39, 2853.45, 1762.39, 1500.85, 1448.26, 1378.54, 1260.2, 1202.95, 1170.65, 1155.34, 1066.55, 1024.56, 904.34, 873.57, 806.11, 779.62, 724.69, 648.92, 598.67, 541.78, 515.48, 479.64, 452.93, 431.52, 420.59, 414.66, 408.42 cm^{-1}.

HRMS (EI-QTOF) *m/z* calcd. for $C_8H_8BrO^{•+}$ ($[M-Br]^{•+}$) 198.9753, found 198.9758.

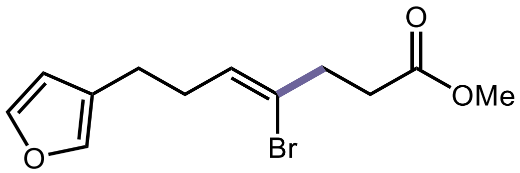

methyl (*Z*)-4-bromo-7-(furan-3-yl)hept-4-enoate (80)

The title compound was prepared according to **General Procedure B** using **NHC** (345.85 mg, 0.875 mmol, 1.75 equiv.), methyl 3-hydroxypropanoate (71.7 µL, 0.75 mmol, 1.5 equiv.), pyridine (69.3 µL, 0.875 mmol, 1.75 equiv.), and *t*-BuOMe (5 mL) for **Stock A** and Ir[(ppy)$_2$(dtbbpy)]PF$_6$ (6.9 mg, 7.5 µmol, 0.015 equiv.), NiBr$_2$(dtbbpy) (24.3 mg, 50.0 µmol, 0.10 equiv.), quinuclidine (97.3 mg, 0.875 mmol, 1.75 equiv.), phthalimide (73.6 mg, 0.50 mmol, 1.0 equiv.), **80-DiBr** (140.0 mg, 0.50 mmol, 1.0 equiv.), and 1,2-dichlorobenzene (10 mL) for **Stock B**. The crude mixture was concentrated via *Genevac* then purified by automated flash chromatography (25 g high performance silica column, 0-25% ethyl acetate/hexanes gradient) to yield impure product. This was further purified by preparative HPLC (30-70% MeCN in water with a 0.1% NH_4OH modifier) to provide the desired compound as a yellow oil (74.2 mg, 0.260 mmol, 52% yield).

^{1}H NMR (500 MHz, CDCl$_3$) δ 7.37 (s, 1H), 7.25 (s, 1H), 6.30 (s, 1H), 5.77 (t, *J* = 6.7 Hz, 1H), 3.69 (s, 3H), 2.77 (t, *J* = 7.5 Hz, 2H), 2.56 (dt, *J* = 23.8, 7.5 Hz, 4H), 2.43 (q, *J* = 7.2 Hz, 2H).

^{13}C NMR (126 MHz, CDCl$_3$) δ 172.81, 142.94, 139.11, 129.29, 126.67, 124.21, 111.05, 51.82, 36.98, 33.26, 31.73, 23.71.

IR (film) ν_{max} 3001.23, 2994.95, 2951.76, 2920.89, 2851.61, 1733.57, 1657.56, 1555.76, 1500.76, 1435.46, 1365.25, 1285.26, 1253.25, 1195.46, 1158.90, 1065.64, 1023.54, 984.71, 890.69, 872.57, 842.95, 776.53, 725.98, 661.99, 628.52, 598.66, 575.61, 530.57, 491.56, 461.19, 435.39, 422.90, 410.15 cm^{-1}.

HRMS (EI-QTOF) *m/z* calcd. for $C_{12}H_{15}O_3^{•+}$ ($[M-Br]^{•+}$) 207.1016, found 207.1012.

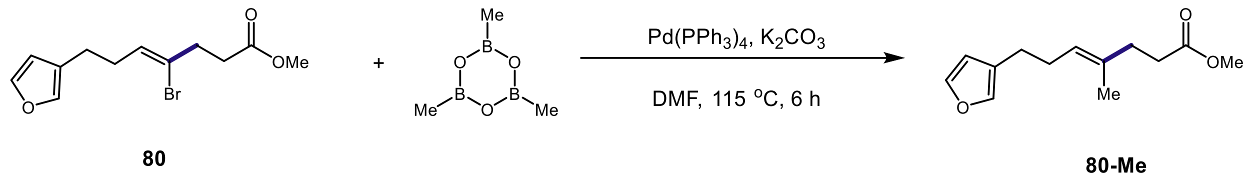

benzyl (*E*)-2-(5-(benzyloxy)pent-2-en-2-yl)-7-azaspiro[3.5]nonane-7-carboxylate (80-Me)

A dry 8 mL vial was charged with methyl (*Z*)-4-bromo-7-(furan-3-yl)hept-4-enoate (**80**, 77.0 mg, 0.27 mmol, 1.0 equiv.), trimethylboroxine (43.4 μL, 0.311 mmol, 1.15 equiv.), tetrakis(triphenylphosphine)palladium(0) (31.2 mg, 0.027 mmol, 0.10 equiv.), potassium carbonate (111.89 mg, 0.81 mmol, 3.0 equiv.), and DMF (1.1 mL). The vial was sparged with N_2 for 15 minutes. The reaction mixture was heated to 115 °C under N_2 atmosphere for 6 hours. The reaction mixture was then left to cool to room temperature with stirring overnight.

The crude reaction mixture was filtered through a celite plug, and the plug was washed with THF (25 mL). The filtrate was concentrated under reduced pressure via rotary evaporation and then purified by automated flash chromatography (25 g high performance silica column, 0-100% ethyl acetate/hexanes gradient) to yield impure product. This was further purified by preparative HPLC (40-90% MeCN in water with a 0.1% NH_4OH modifier) to provide the desired compound as a yellow oil (78.8 mg, 0.213 mmol, 79% yield).

^{1}H NMR (500 MHz, $CDCl_3$) δ 7.33 (t, *J* = 1.7 Hz, 1H), 7.20 (s, 1H), 6.26 (s, 1H), 5.19 (tq, *J* = 7.0, 1.4 Hz, 1H), 3.66 (s, 3H), 2.42 (ddd, *J* = 15.9, 7.9, 6.4 Hz, 4H), 2.31 (q, *J* = 7.7 Hz, 2H), 2.24 (q, *J* = 7.2 Hz, 2H), 1.59 (s, 3H).

^{13}C NMR (126 MHz, $CDCl_3$) δ 174.02, 142.74, 138.99, 134.23, 124.93, 124.71, 111.19, 51.65, 34.74, 33.10, 28.53, 25.02, 16.12.

IR (film) ν_{max} 2971.77, 2946.73, 2915.90, 2871.32, 2852.68, 1732.73, 1500.63, 1435.71, 1348.06, 1292.45, 1255.44, 1194.82, 1157.20, 1091.30, 1064.08, 1022.87, 893.81, 872.88, 848.97, 779.19, 723.70, 696.94, 661.86, 629.13, 599.23, 542.30, 510.87, 445.35, 431.79, 419.66, 408.77 cm^{-1}.

HRMS (EI-QTOF) *m/z* calcd. for $C_{13}H_{18}O_3^{\bullet+}$ ($[M]^{\bullet+}$) 222.1250, found 222.1246.

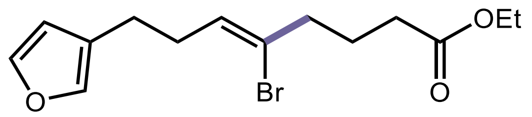

ethyl (*Z*)-5-bromo-8-(furan-3-yl)oct-5-enoate (81)

The title compound was prepared according to **General Procedure B** using **NHC** (345.85 mg, 0.875 mmol, 1.75 equiv.), ethyl 4-hydroxybutanoate (99.1 mg, 0.75 mmol, 1.5 equiv.), pyridine (69.3 µL, 0.875 mmol, 1.75 equiv.), and *t*-BuOMe (5 mL) for **Stock A** and Ir[(ppy)$_2$(dtbbpy)]PF$_6$ (6.9 mg, 7.5 µmol, 0.015 equiv.), NiBr$_2$(dtbbpy) (24.3 mg, 50.0 µmol, 0.10 equiv.), quinuclidine (97.3 mg, 0.875 mmol, 1.75 equiv.), phthalimide (73.6 mg, 0.50 mmol, 1.0 equiv.), **80-DiBr** (140.0 mg, 0.50 mmol, 1.0 equiv.), and 1,2-dichlorobenzene (10 mL) for **Stock B**. The crude mixture was concentrated via *Genevac* then purified by automated flash chromatography (25 g high performance silica column, 0-25% ethyl acetate/hexanes gradient) to yield impure product. This was further purified by preparative HPLC (30-70% MeCN in water with a 0.1% NH$_4$OH modifier) to provide the desired compound as a yellow oil (75.1 mg, 0.239 mmol, 48% yield).

^{1}H NMR (500 MHz, CDCl$_3$) δ 7.35 (t, *J* = 1.7 Hz, 1H), 7.23 (dd, *J* = 1.6, 0.9 Hz, 1H), 6.29 (s, 1H), 5.70 – 5.64 (m, 1H), 4.13 (q, *J* = 7.1 Hz, 2H), 2.54 – 2.40 (m, 6H), 2.26 (t, *J* = 7.4 Hz, 2H), 1.87 (p, *J* = 7.4 Hz, 2H), 1.26 (t, *J* = 7.1 Hz, 3H).

^{13}C NMR (126 MHz, CDCl$_3$) δ 173.40, 142.96, 139.11, 128.88, 127.86, 124.29, 111.06, 60.48, 40.73, 32.75, 31.75, 23.82, 23.38, 14.40.

IR (film) ν_{max} 2975.48, 2953.78, 2923.51, 2860.36, 1770.61, 1731.66, 1679.97, 1501.17, 1450.25, 1375.71, 1335.21, 1301.24, 1246.92, 1178.77, 1158.82, 1094.48, 1066.10, 1025.12, 873.50, 779.37, 600.07, 406.93 cm^{-1}.

HRMS (EI-QTOF) *m/z* calcd. for $C_{14}H_{19}O_3^{\bullet+}$ ($[M-Br]^{\bullet+}$) 235.1329, found 235.1326.

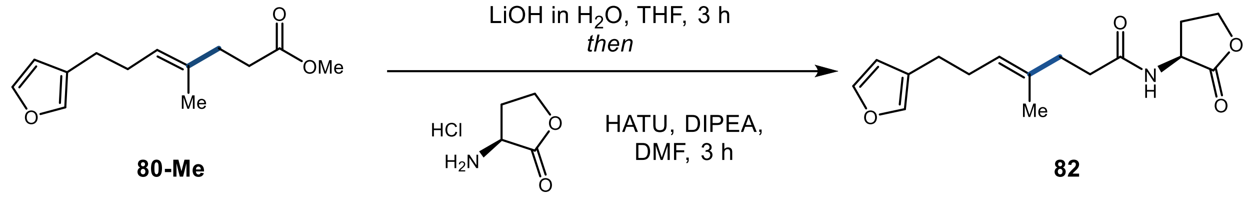

(+)-sponalisolide B (82)

Benzyl (*E*)-2-(5-(benzyloxy)pent-2-en-2-yl)-7-azaspiro[3.5]nonane-7-carboxylate (**80-Me**, 78.8 mg, 0.35 mmol, 1.0 equiv.) was dissolved in THF (2.8 mL). 1N LiOH in water was added (2.9 mL). The mixture was stirred at room temperature for 3 hours, before being acidified to pH 2 with 1 N HCl. This mixture was further stirred at room temperature for 2 hours. EtOAc was added and the organic layer was extracted (3x), then washed with brine (3x). The combined organic layers were dried with $MgSO_4$, filtered, and concentrated under reduced pressure via rotary evaporation. This crude carboxylic acid product was used without further purification.

The crude *(E)*-7-(furan-3-yl)-4-methylhept-4-enoic acid was dissolved in dry DMF (5 mL). The solution was then treated with L-homoserine lactone hydrochloride (48.8 mg, 0.35 mmol, 1.0 equiv.), HATU (148.5 mg, 0.385 mmol, 1.1 equiv.), and N,N-diisopropylethylamine (0.247 mL, 1.4 mmol, 4 equiv.). This mixture was stirred for 3 hours at room temperature. 1 N citric acid in water was added (6 mL). The solution was extracted with Et_2O and brine, before drying the organic with $MgSO_4$. The crude mixture was concentrated via *Genevac* then purified by automated flash chromatography (25 g high performance silica column, 0-100% ethyl acetate/hexanes gradient) to yield impure product. This was further purified by preparative HPLC (30-70% MeCN in water with a 0.1% NH_4OH modifier) to provide the desired compound as an amorphous white solid (86.0 mg, 0.235 mmol, 84% yield). Spectra are in accordance with literature.[5]

^{1}H NMR (500 MHz, $CDCl_3$) δ 7.33 (s, 1H), 7.20 (s, 1H), 6.26 (s, 1H), 6.01 (d, *J* = 5.7 Hz, 1H), 5.22 (t, *J* = 7.1 Hz, 1H), 4.57 – 4.42 (m, 2H), 4.27 (ddd, *J* = 11.3, 9.3, 5.8 Hz, 1H), 2.85 (ddd, *J* = 13.5, 8.5, 5.9 Hz, 1H), 2.45 (t, *J* = 7.5 Hz, 2H), 2.40 – 2.29 (m, 4H), 2.27 (d, *J* = 7.4 Hz, 1H), 2.24 (d, *J* = 7.4 Hz, 1H), 2.08 (qd, *J* = 11.8, 8.7 Hz, 1H), 1.61 (s, 3H).

11) Spectral Data for Isolated Products

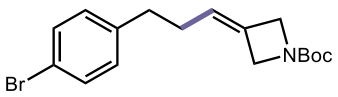

1-^{1}H-NMR-CDCl$_3$

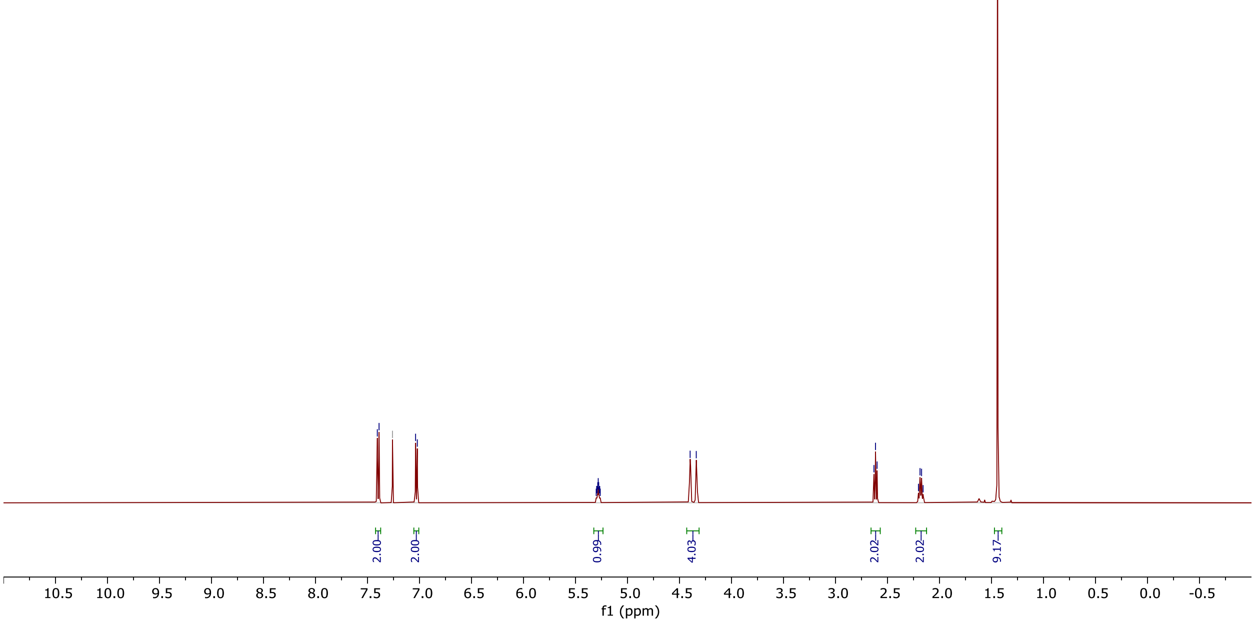

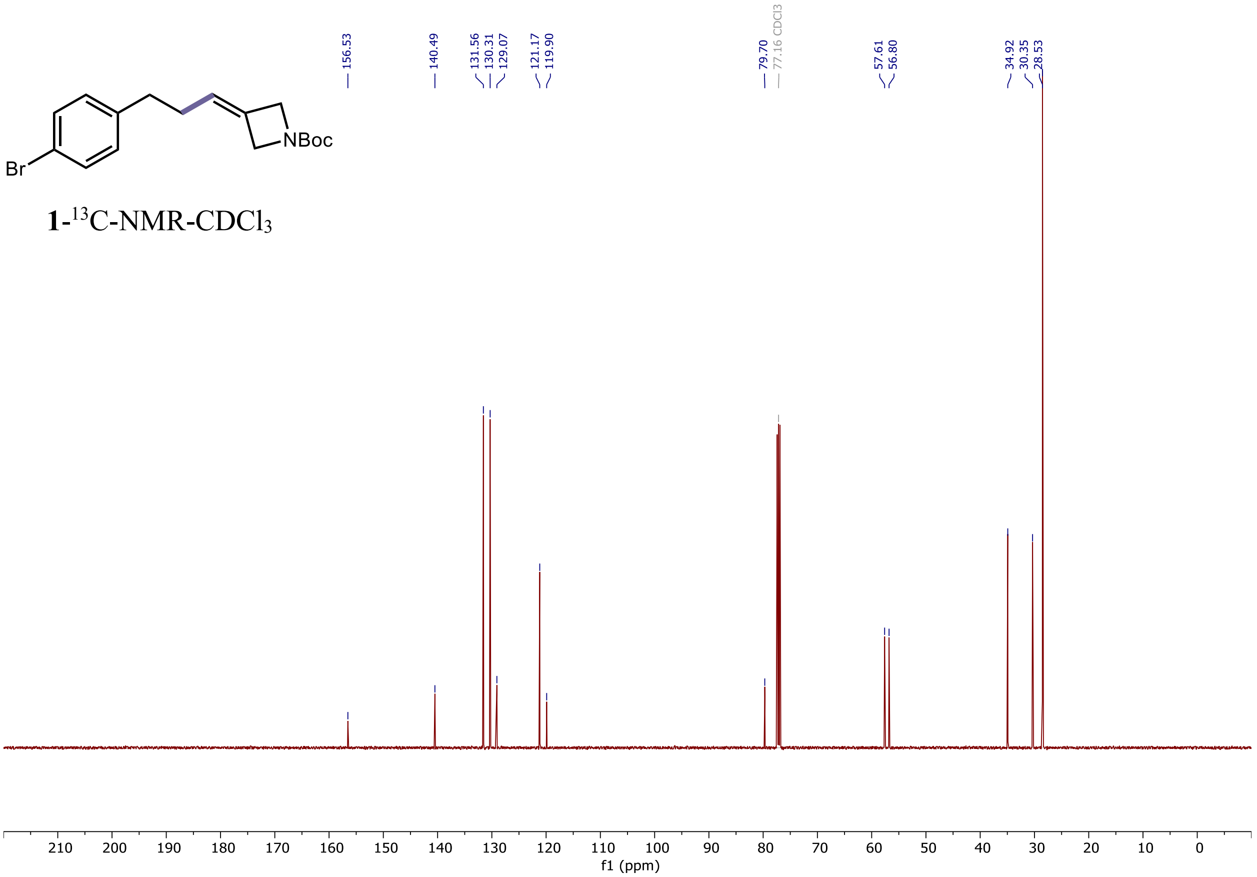

1-^{13}C-NMR-$CDCl_3$

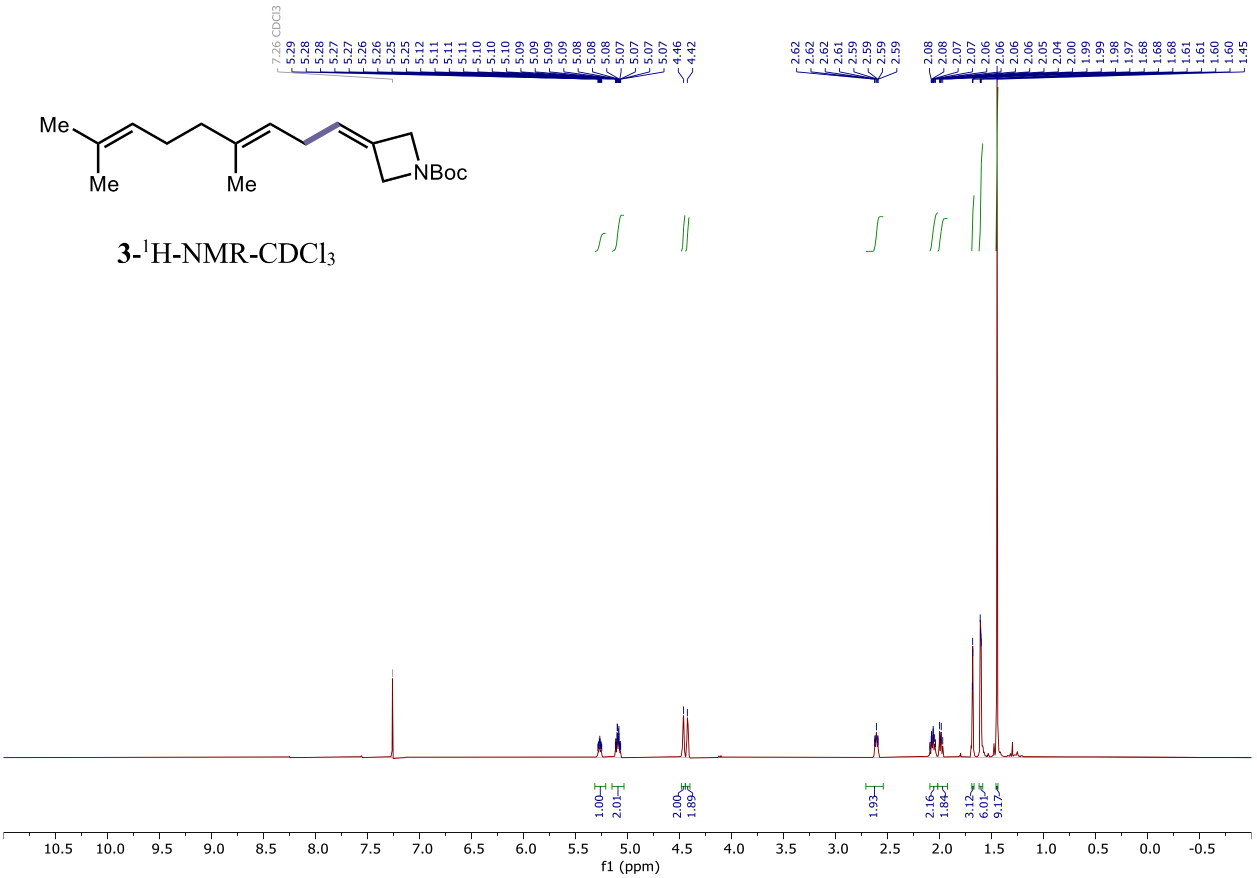

3-^{1}H-NMR-$CDCl_3$

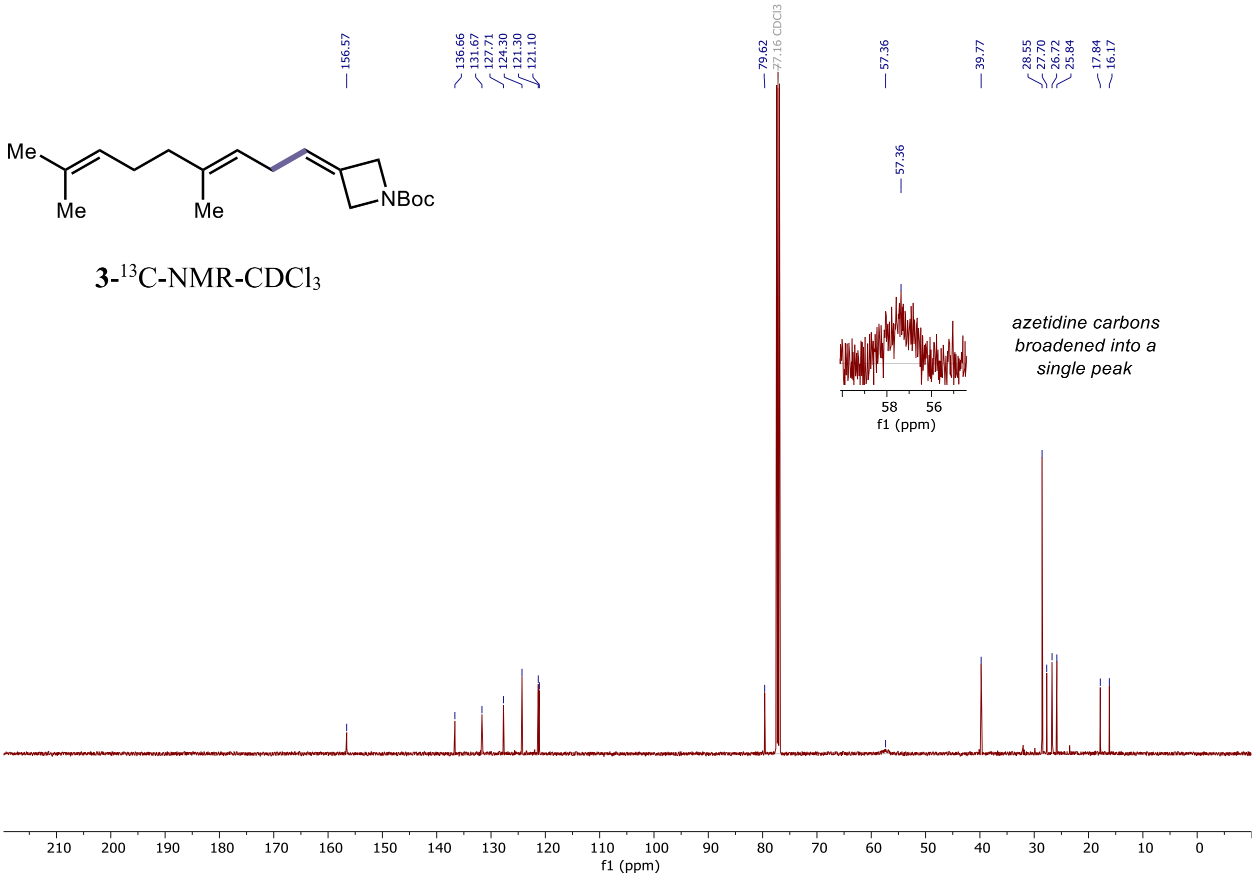

3-^{13}C-NMR-$CDCl_3$

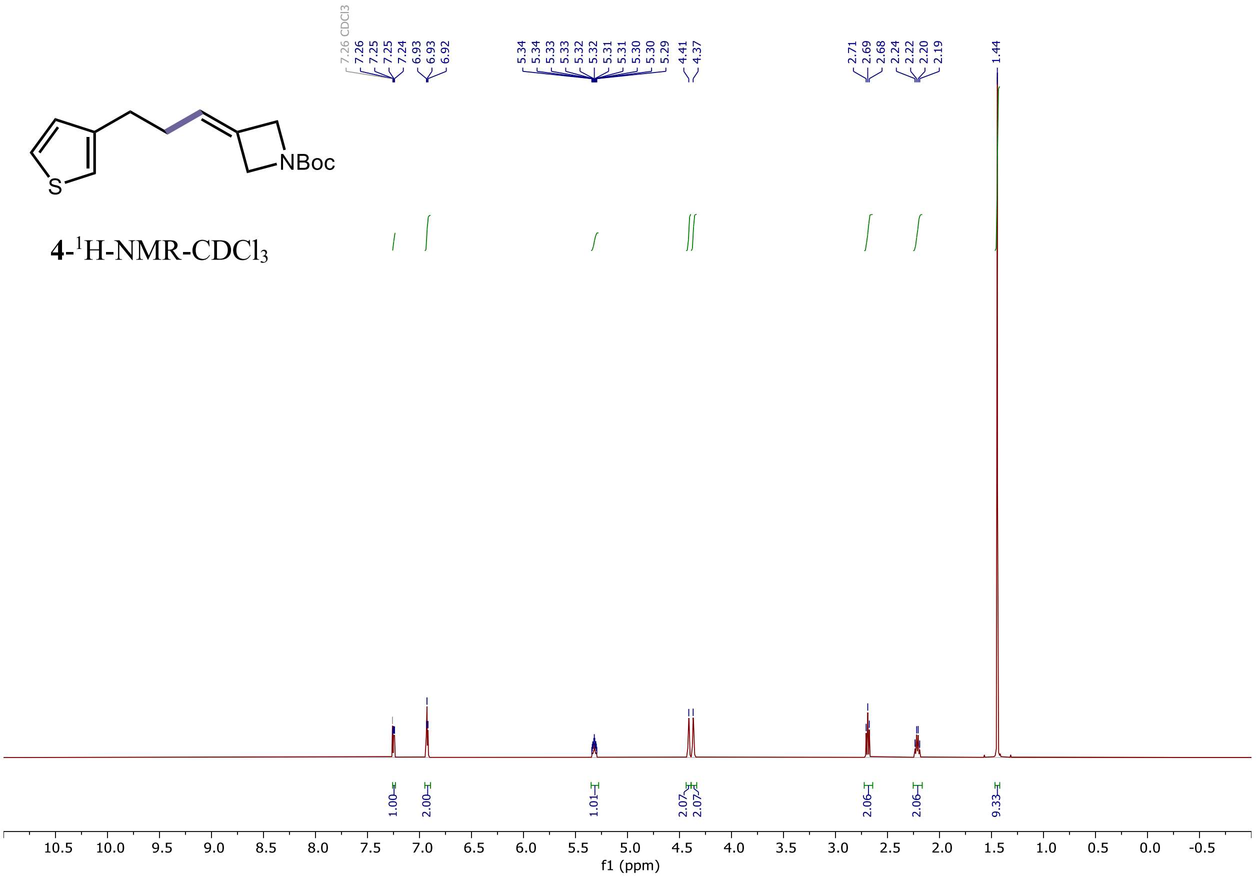

4-^{1}H-NMR-$CDCl_3$

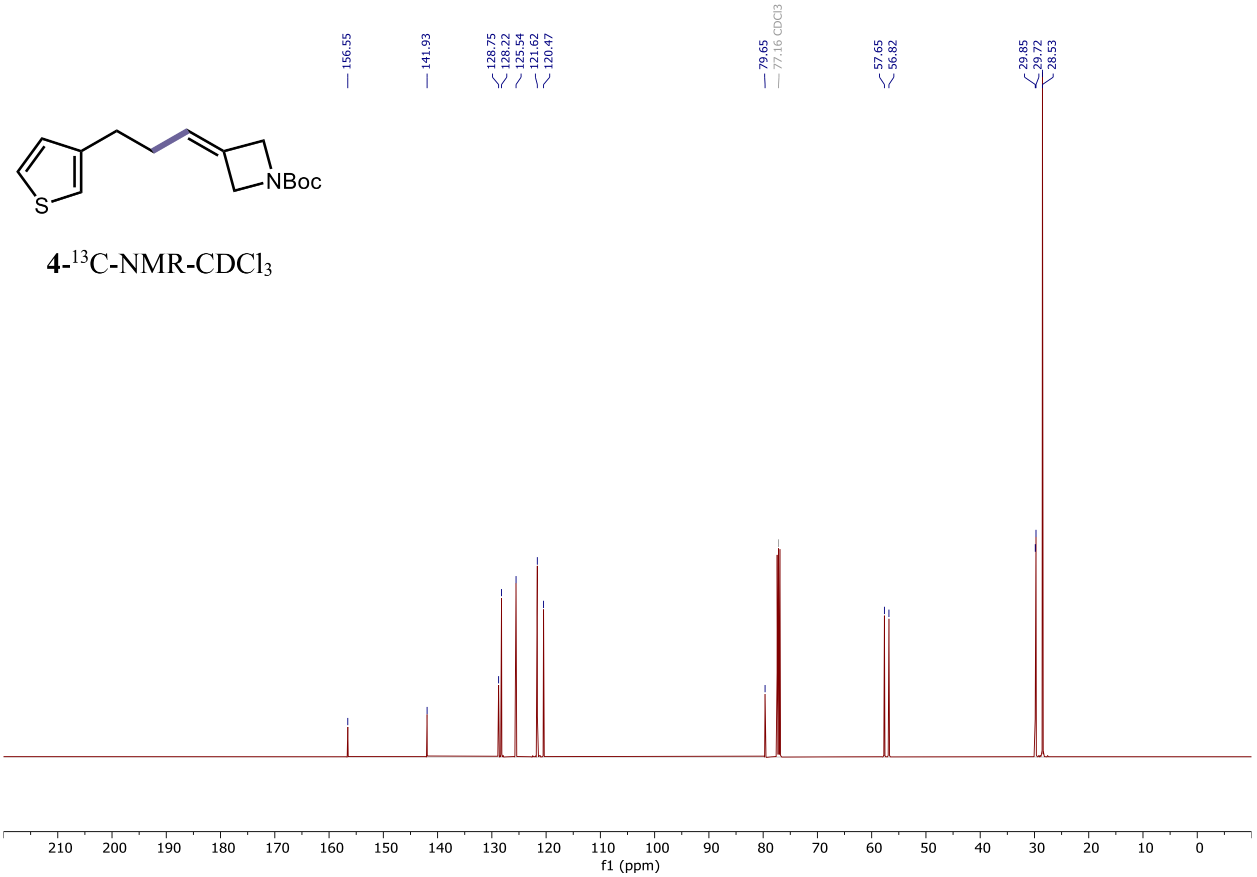

4-^{13}C-NMR-$CDCl_3$

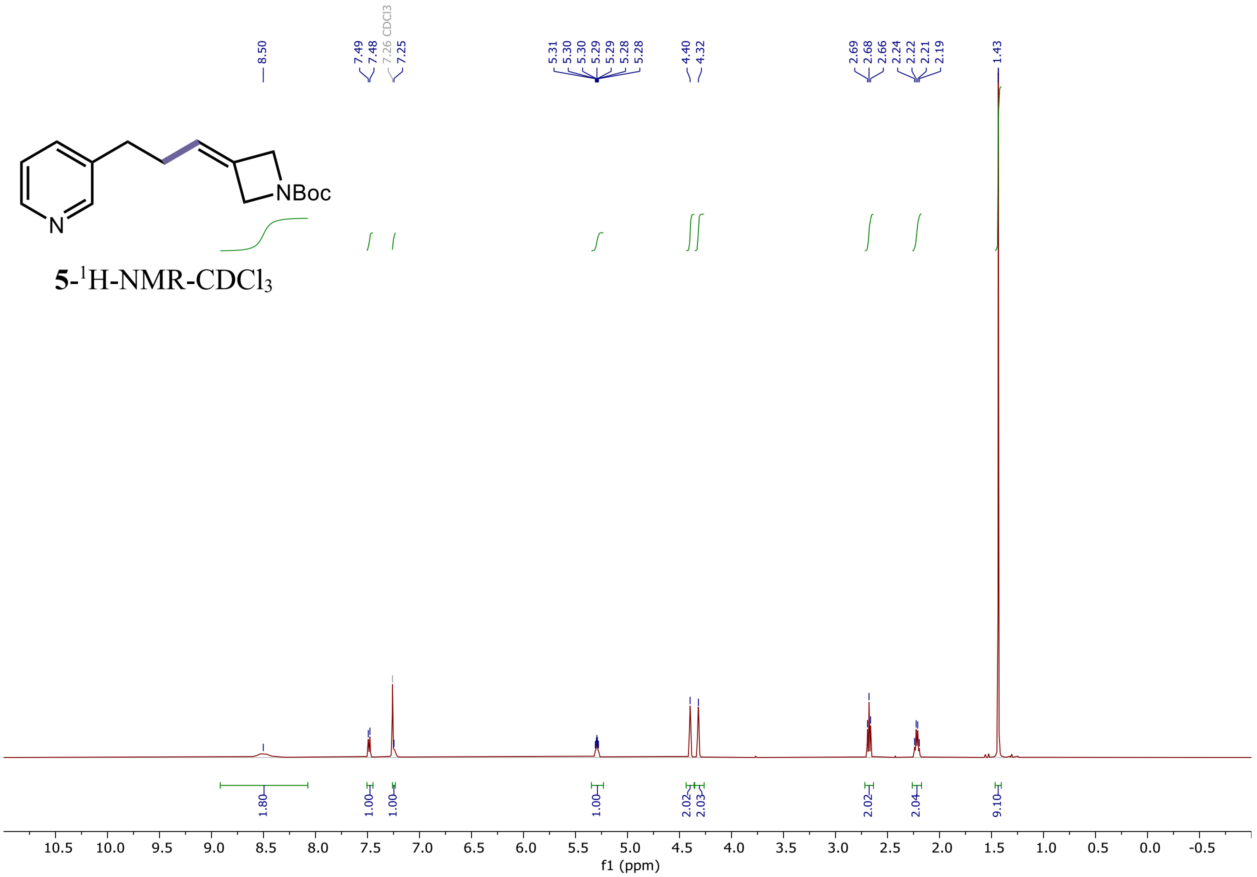

5-^{1}H-NMR-$CDCl_3$

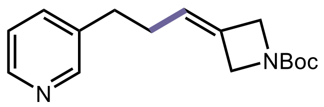

5-^{13}C-NMR-CDCl$_3$

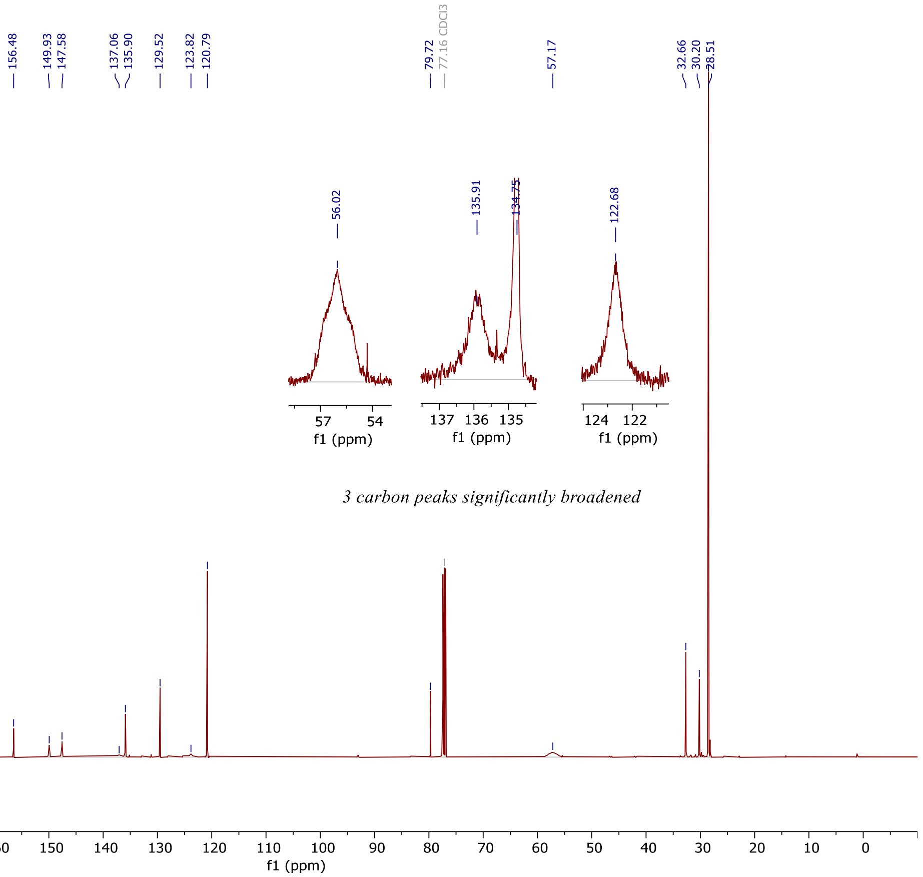

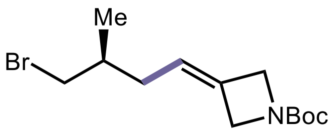

6-^{1}H-NMR-CDCl$_3$

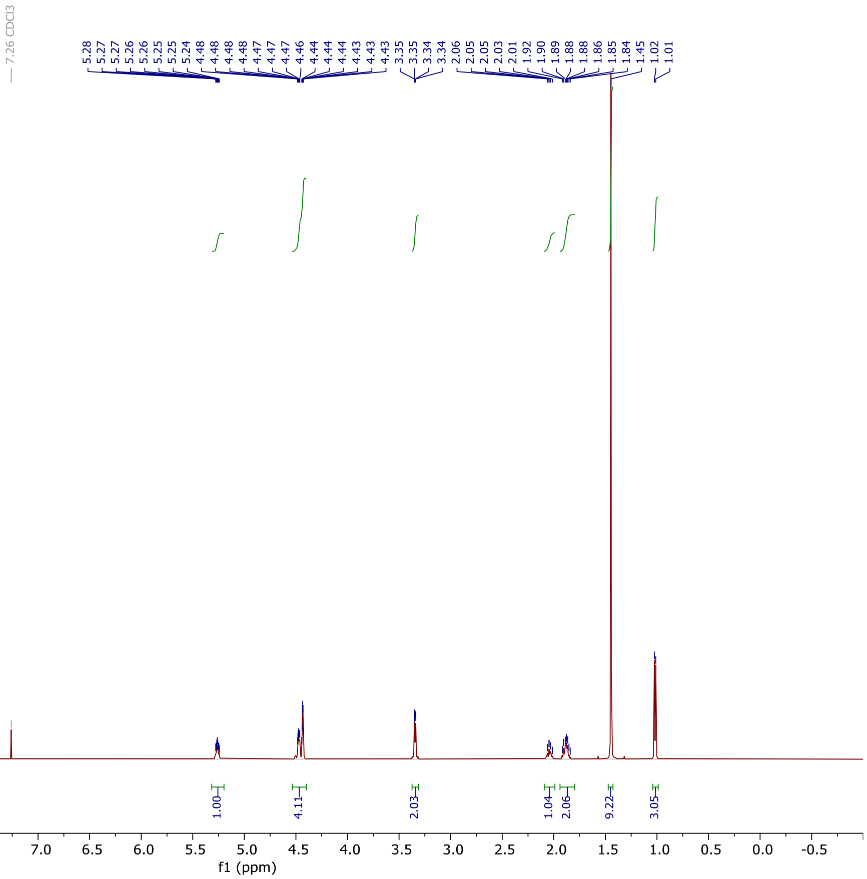

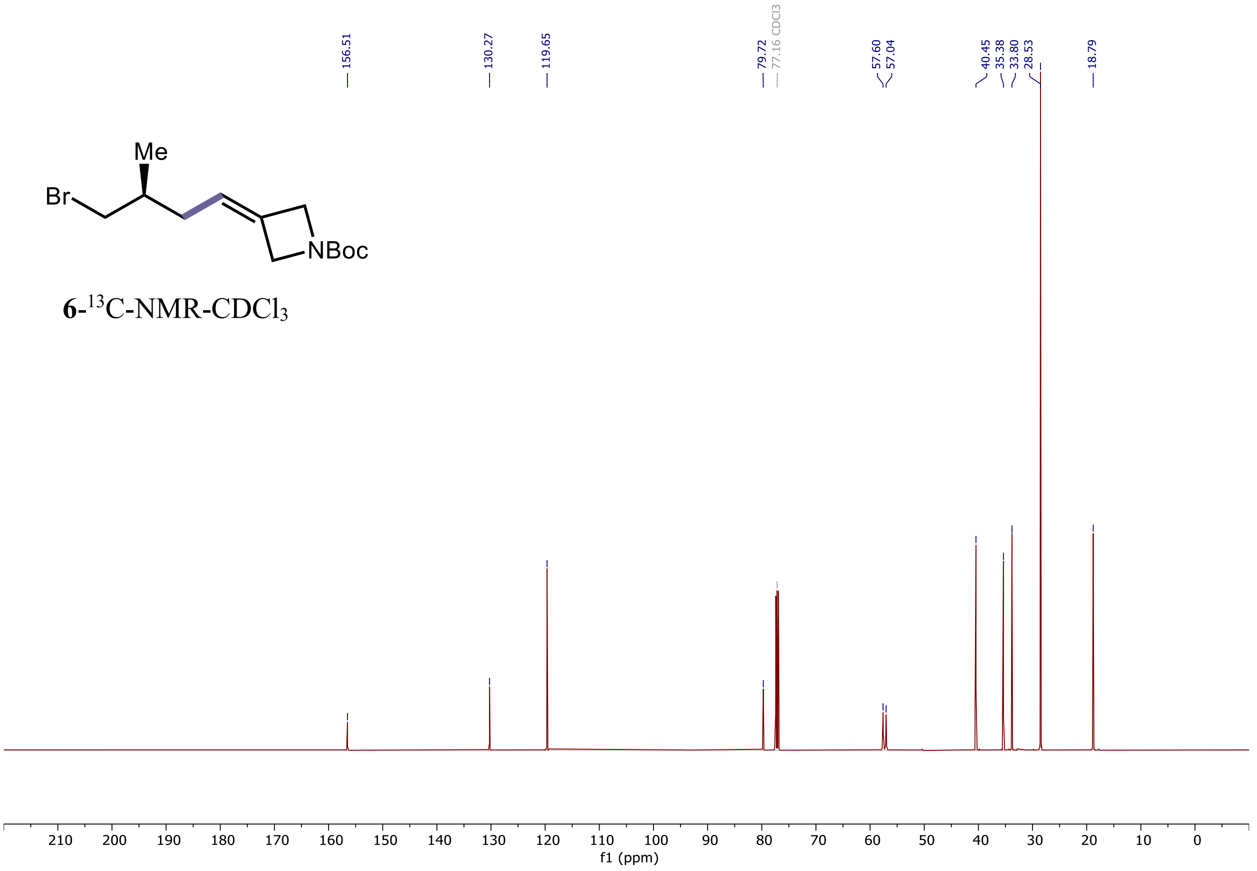

6-^{13}C-NMR-$CDCl_3$

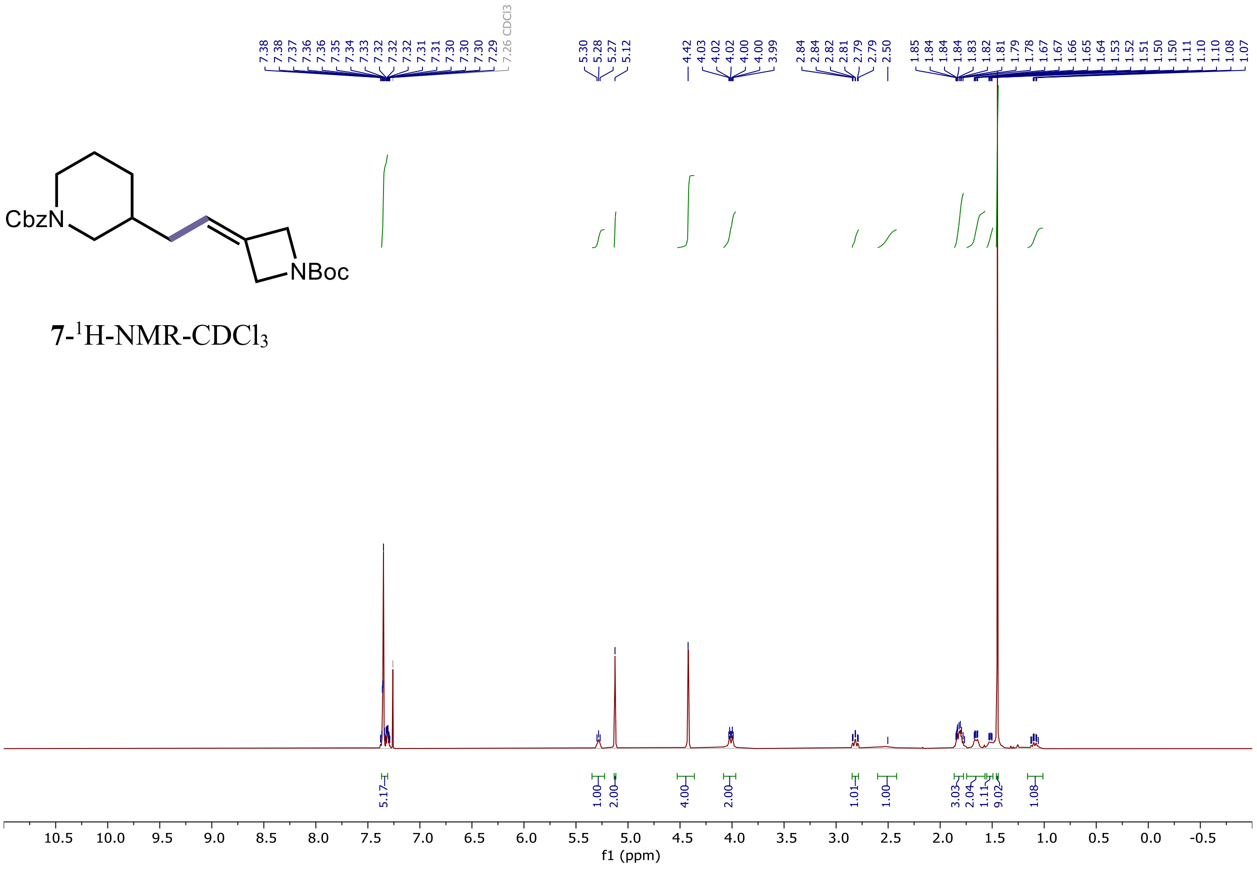

7-^{1}H-NMR-$CDCl_3$

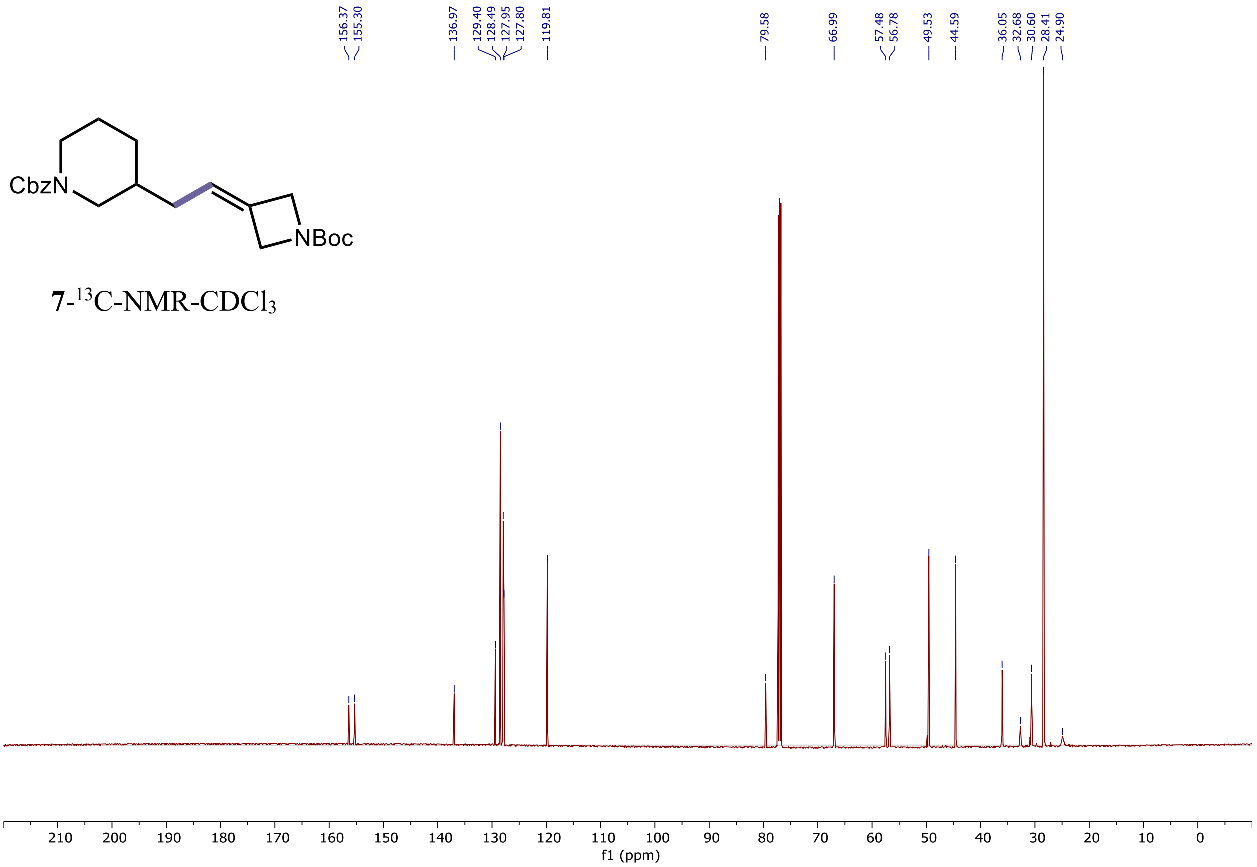

7-^{13}C-NMR-CDCl$_3$

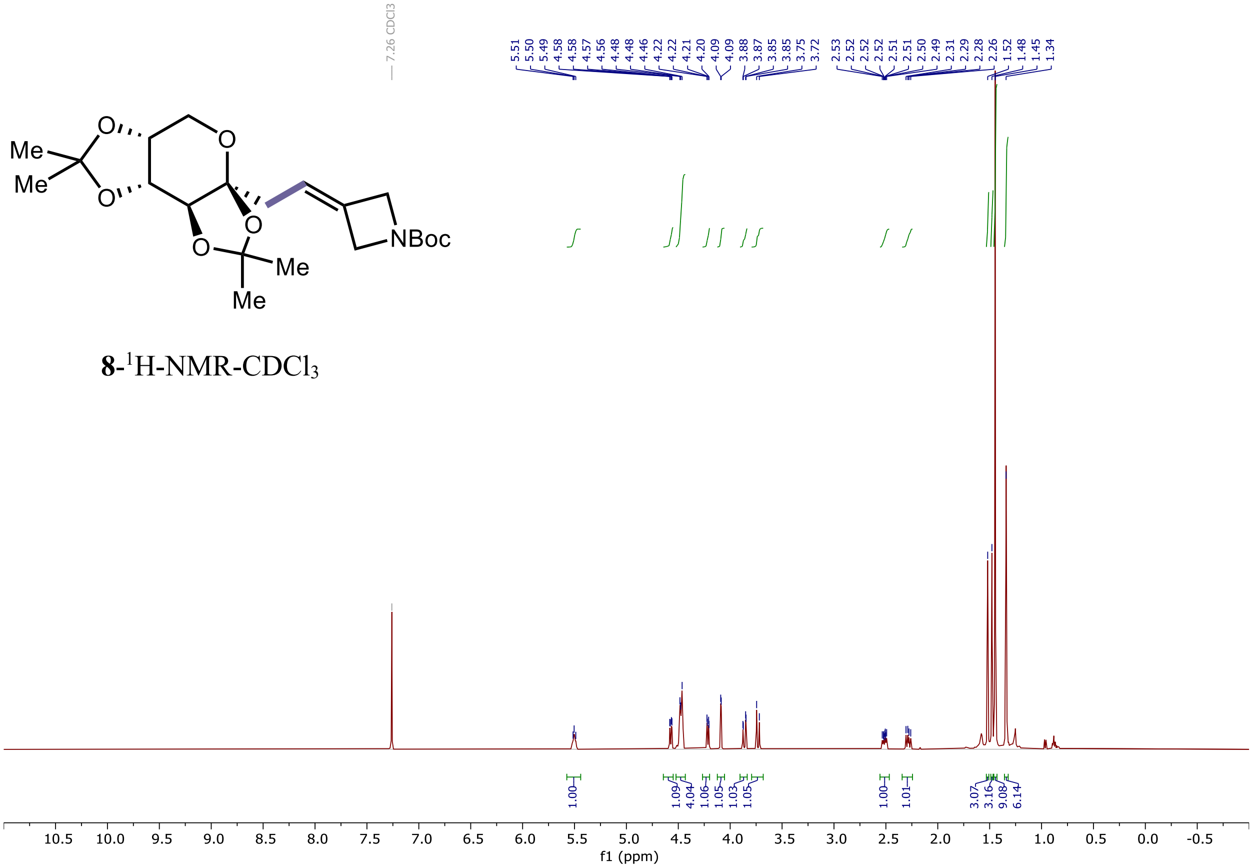

8-^{1}H-NMR-CDCl$_3$

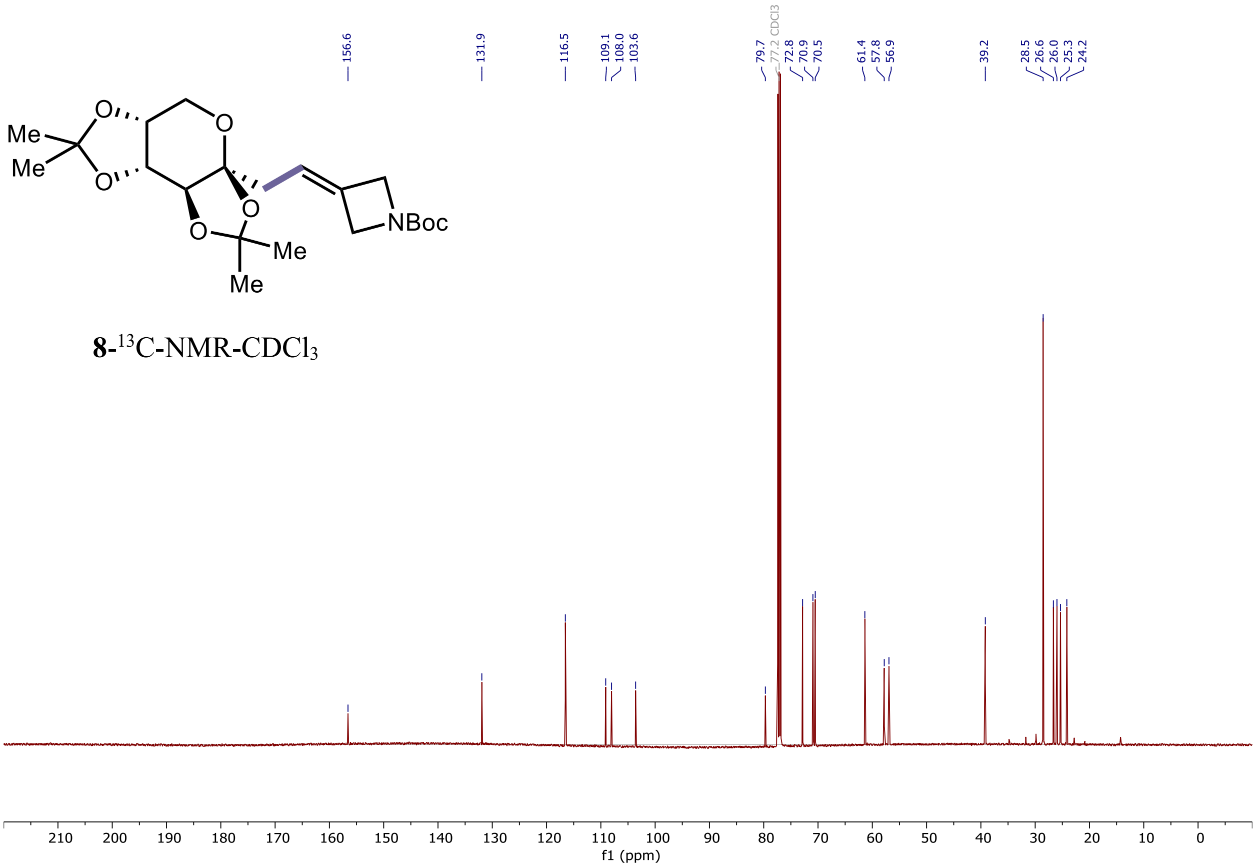

8-^{13}C-NMR-$CDCl_3$

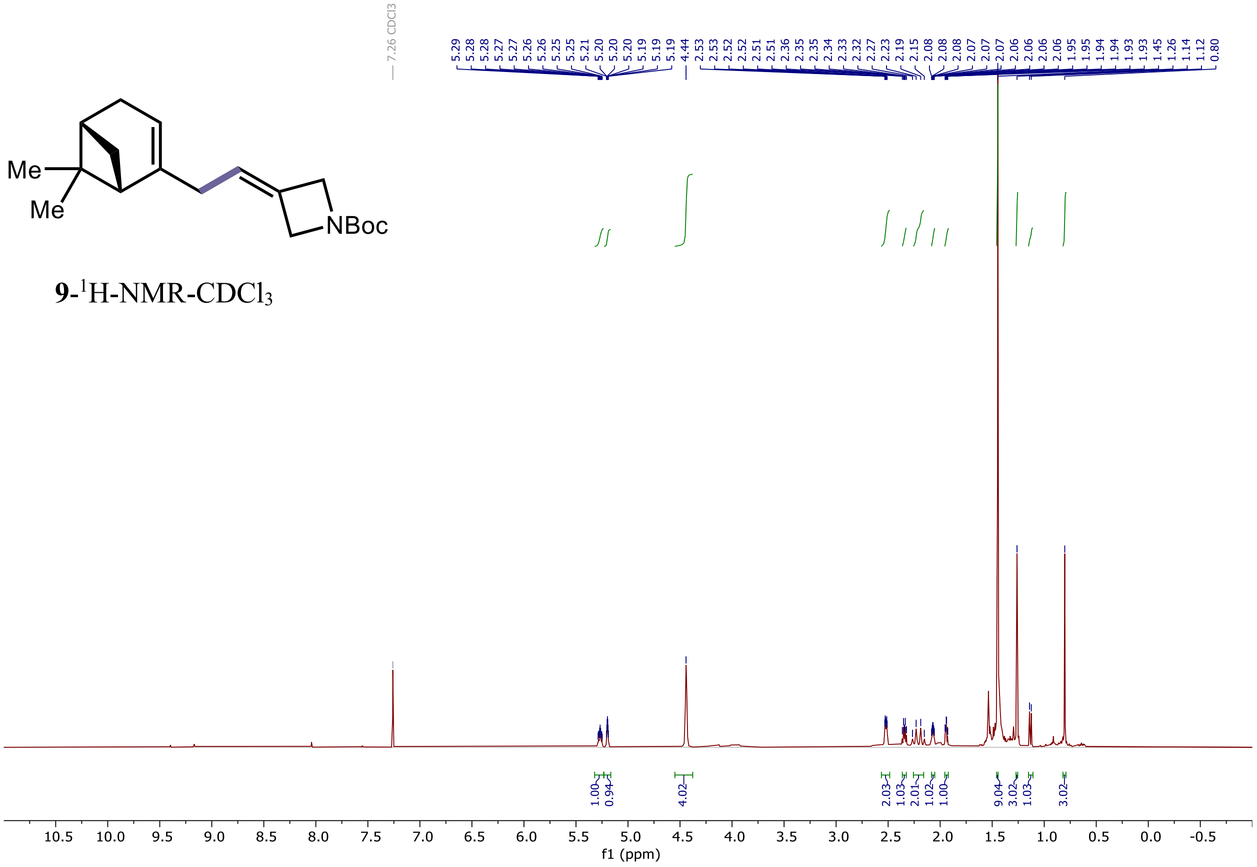

9-^{1}H-NMR-$CDCl_3$

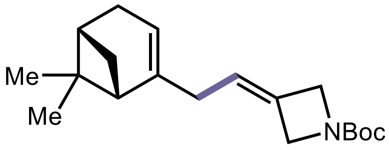

9-^{13}C-NMR-$CDCl_3$

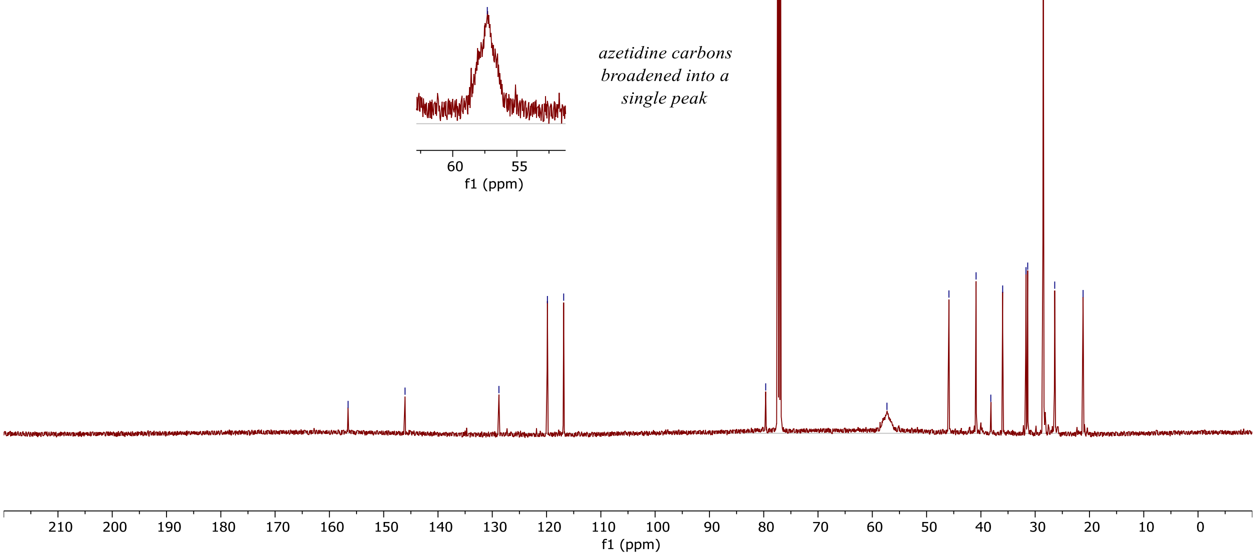

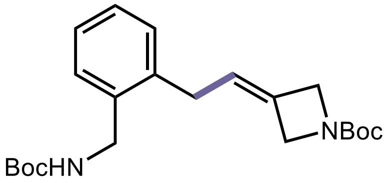

10-^{1}H-NMR-$CDCl_3$

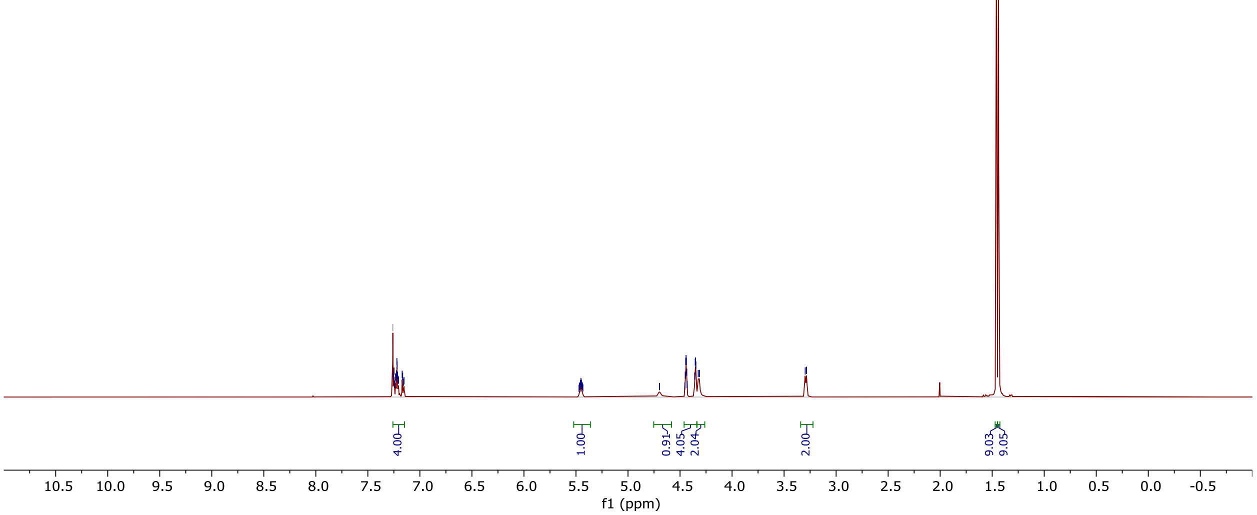

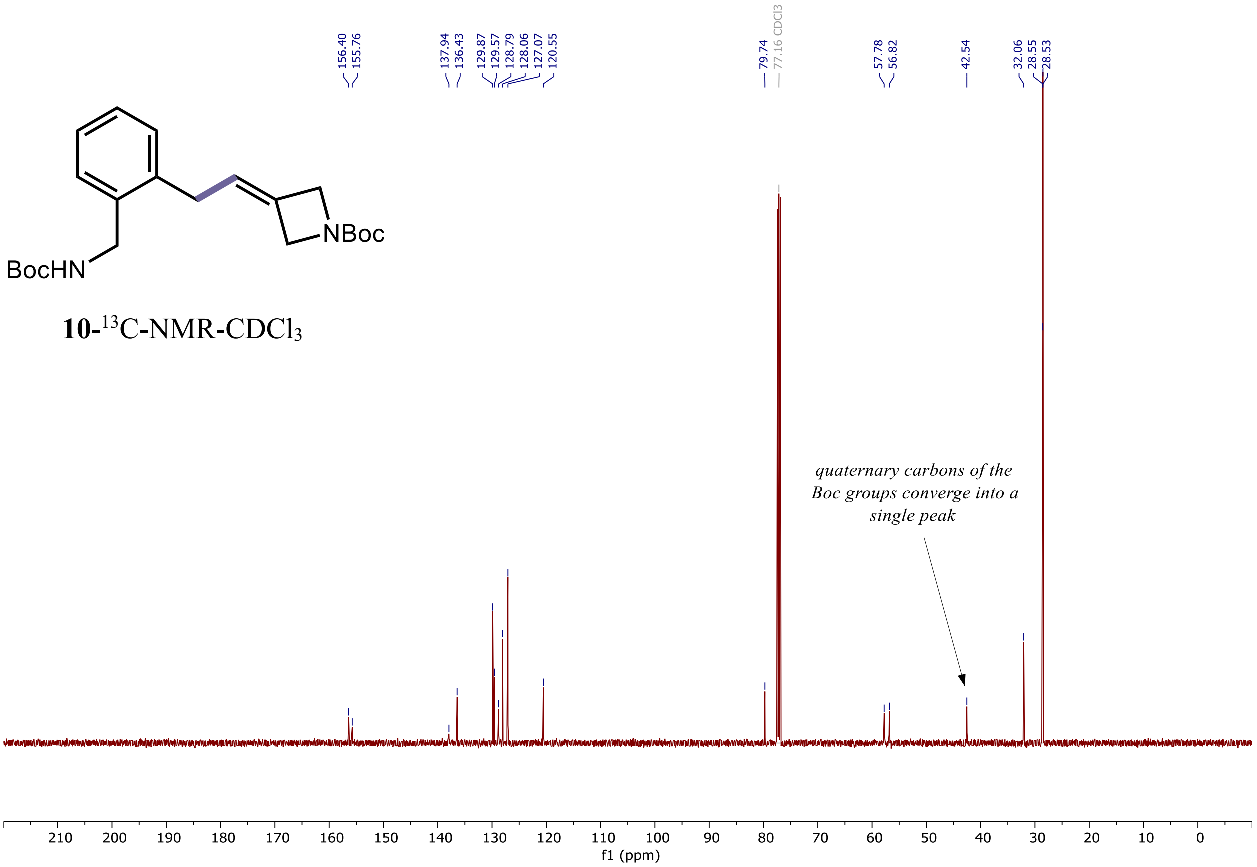

10-^{13}C-NMR-$CDCl_3$

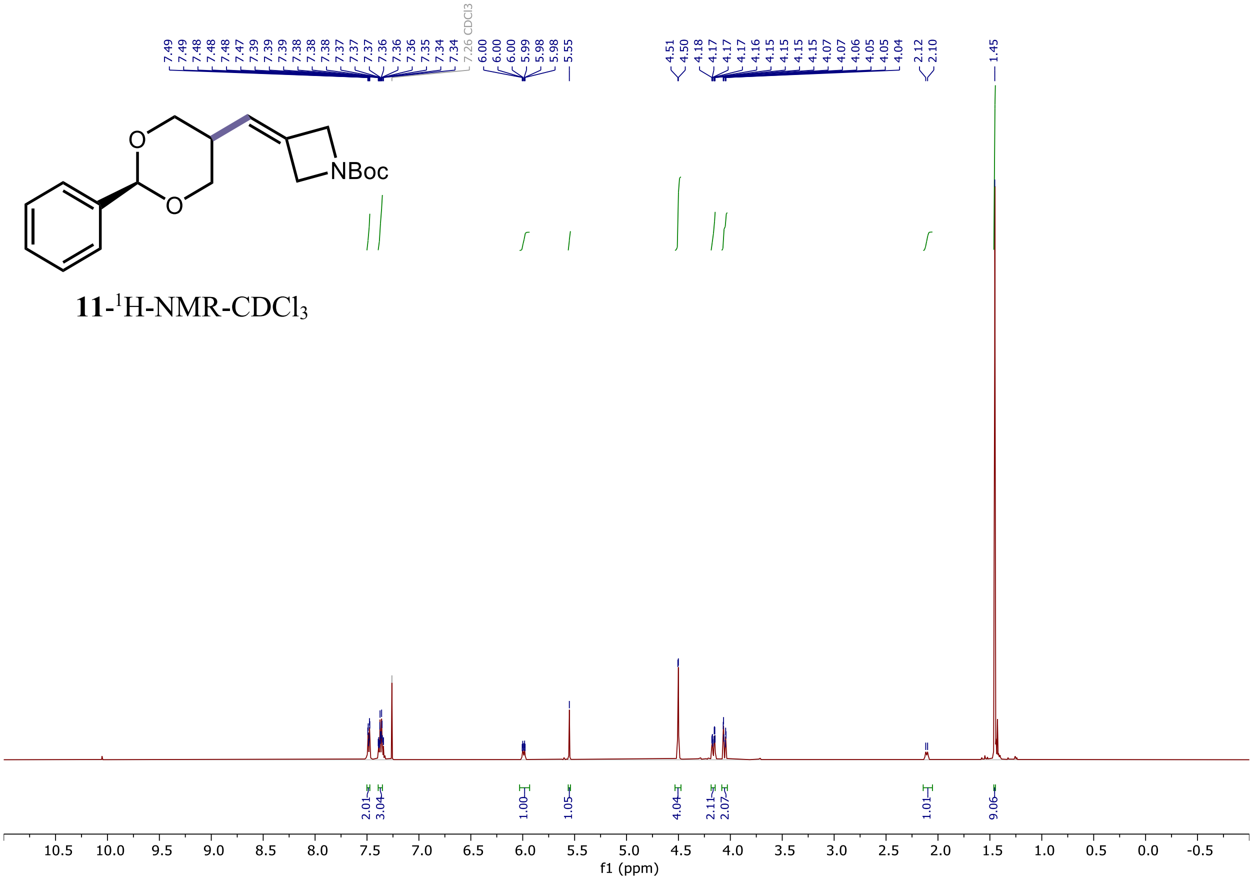

11-^{1}H-NMR-$CDCl_3$

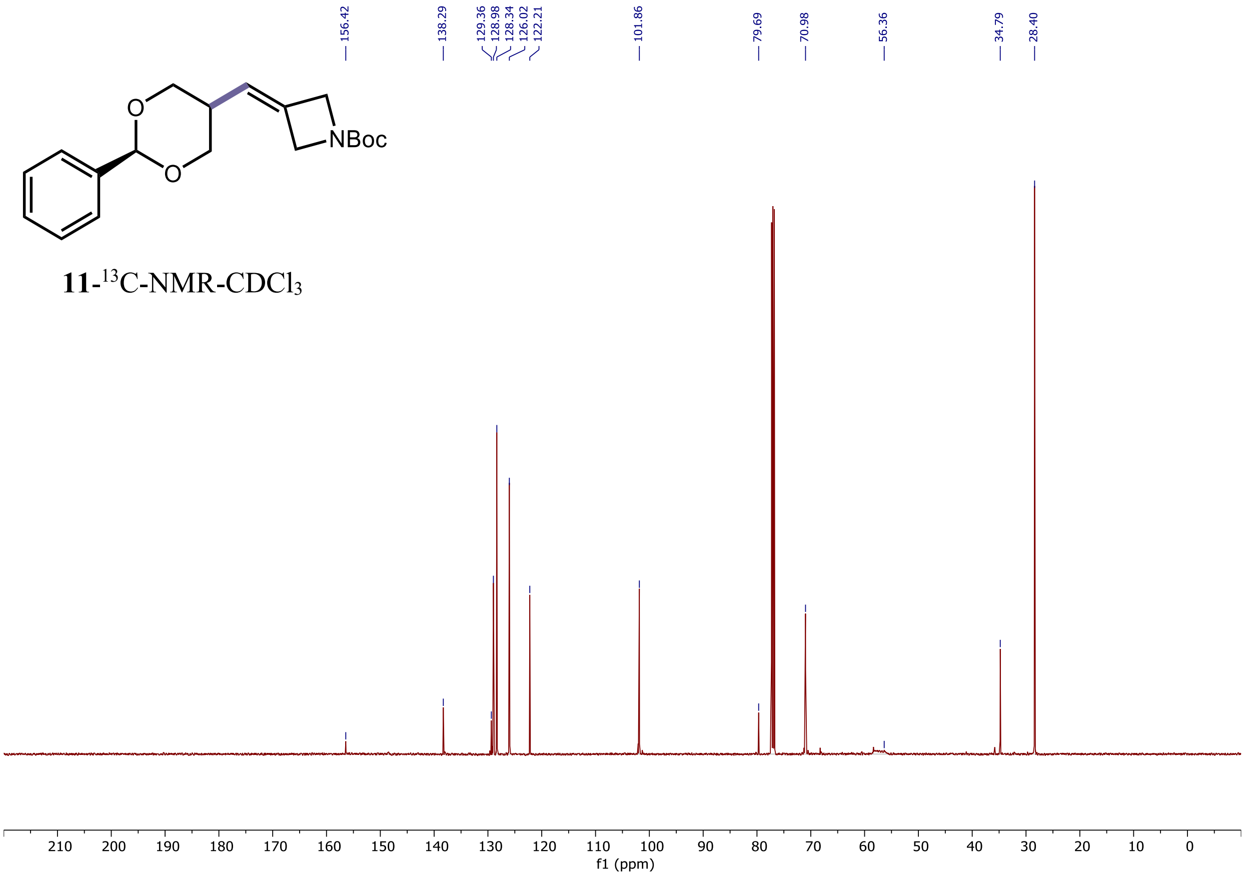

11-^{13}C-NMR-$CDCl_3$

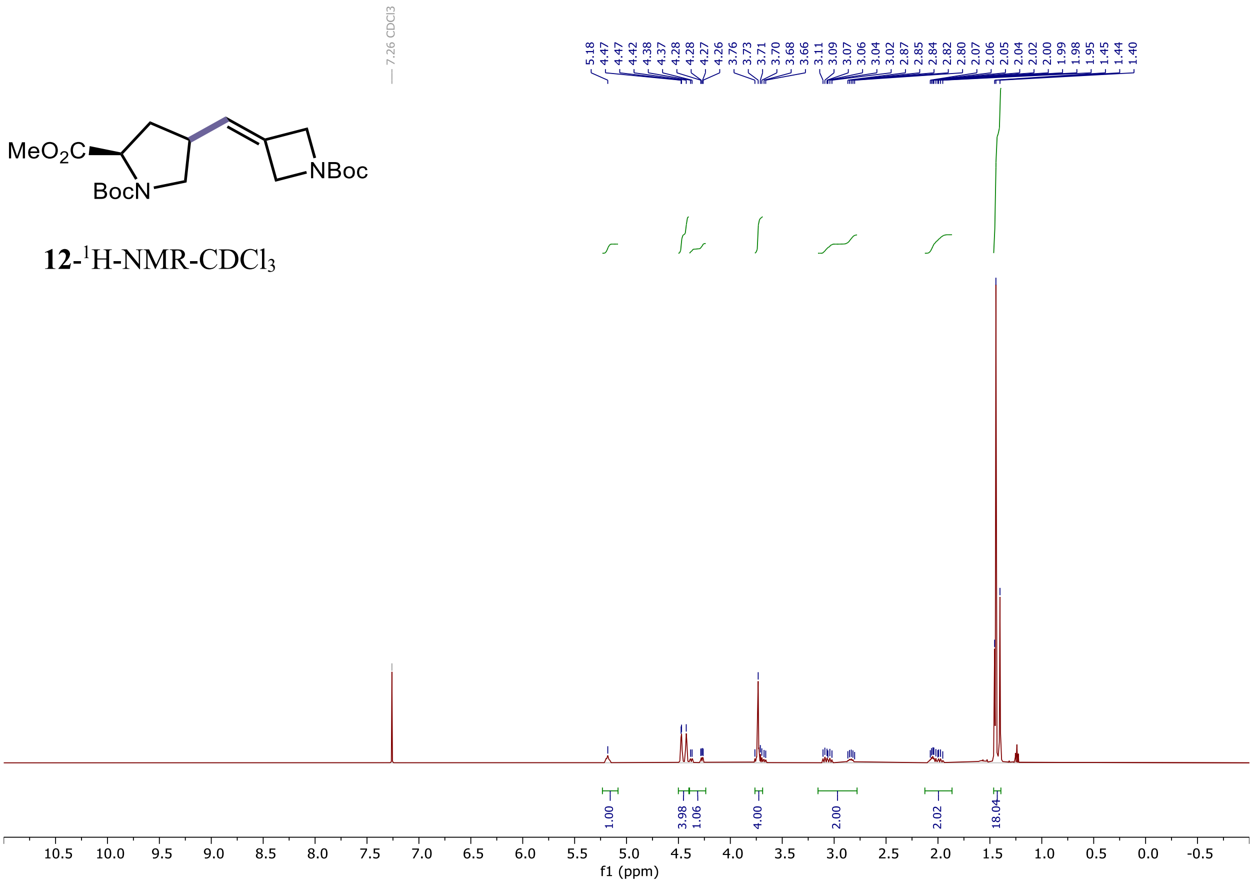

12-^{1}H-NMR-$CDCl_3$

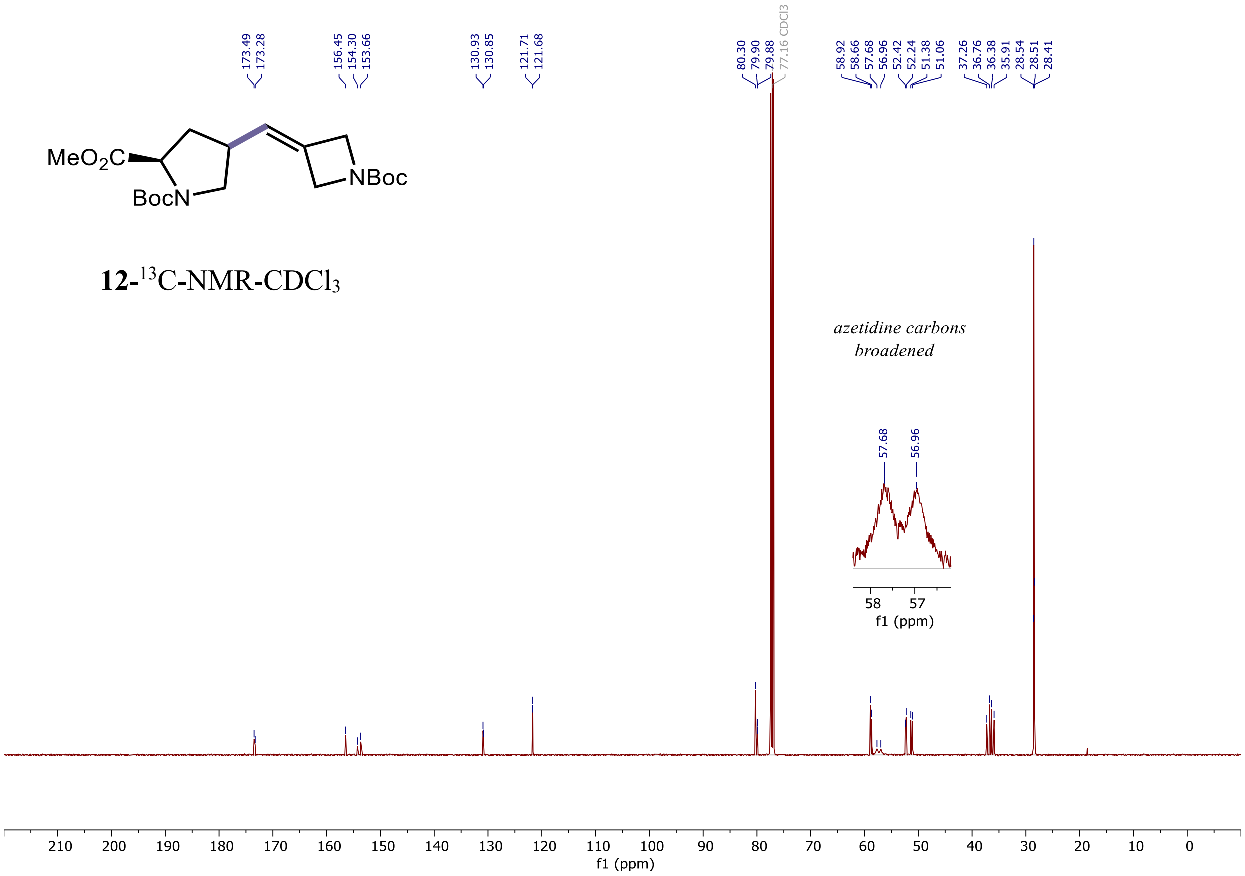

12-^{13}C-NMR-CDCl$_3$

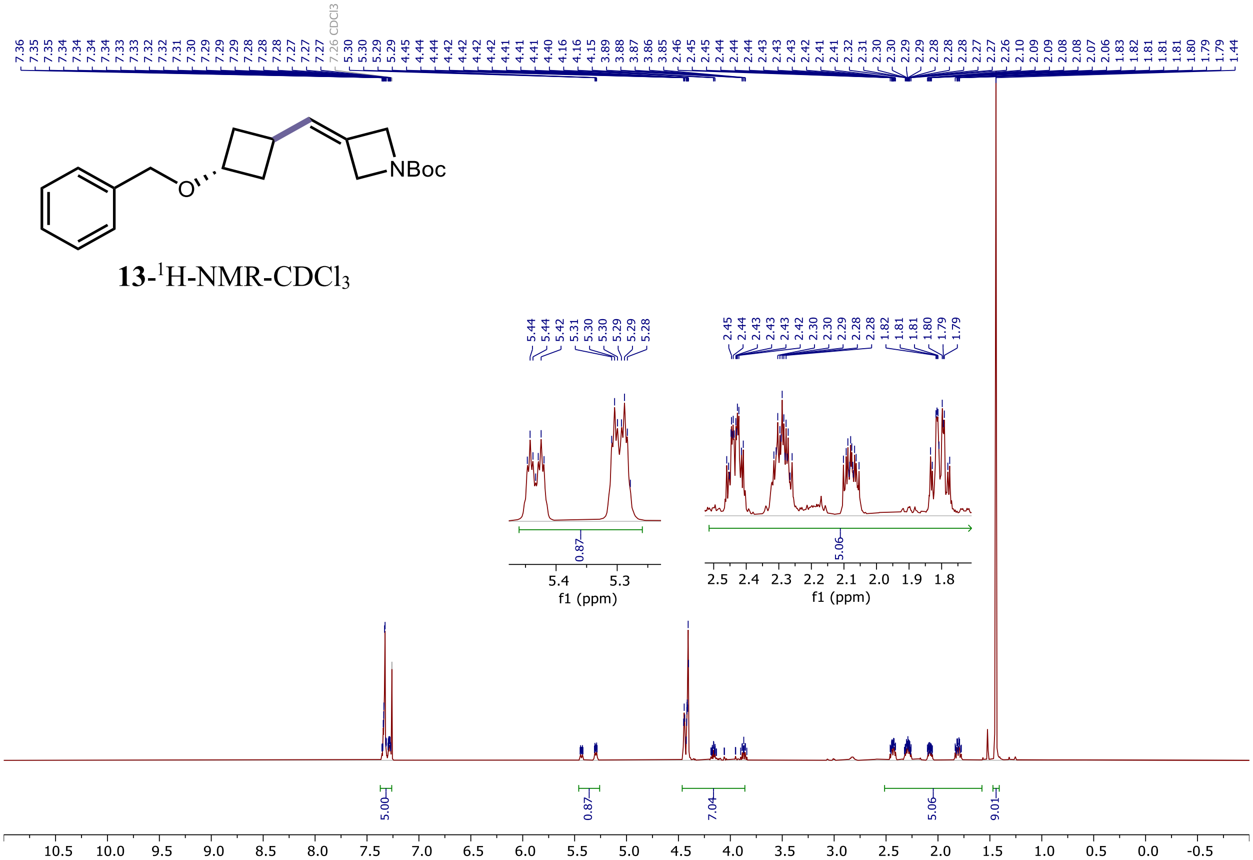

13-^{1}H-NMR-CDCl$_3$

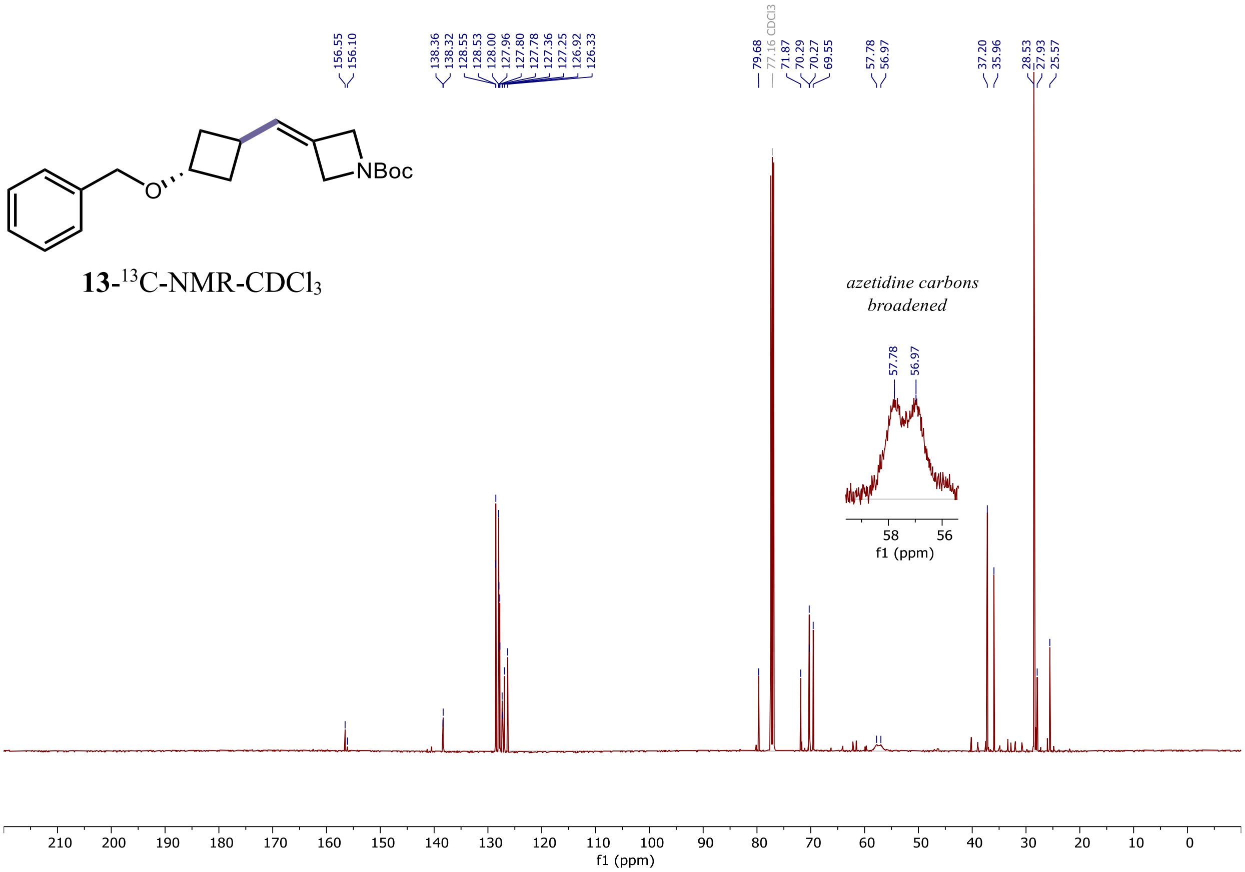

13-^{13}C-NMR-$CDCl_3$

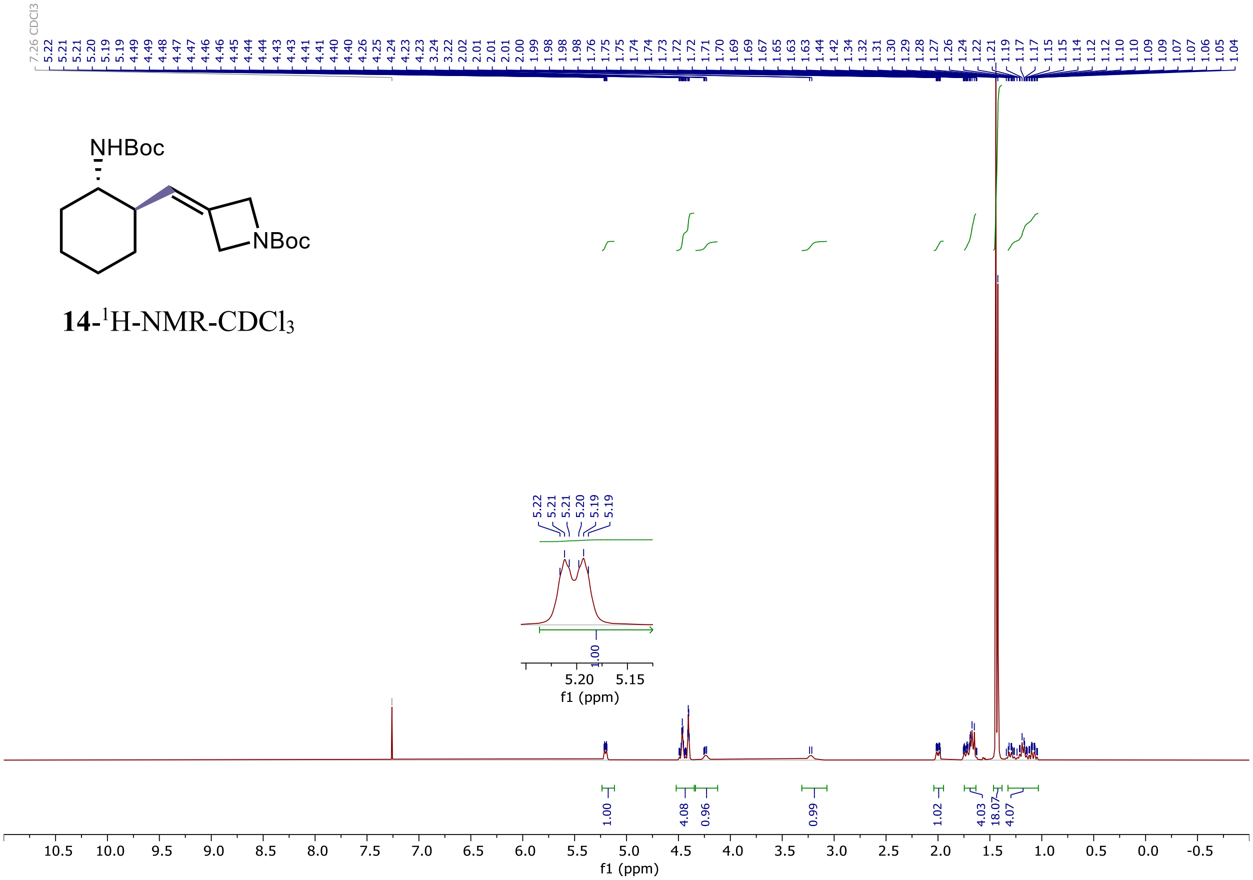

14-^{1}H-NMR-$CDCl_3$

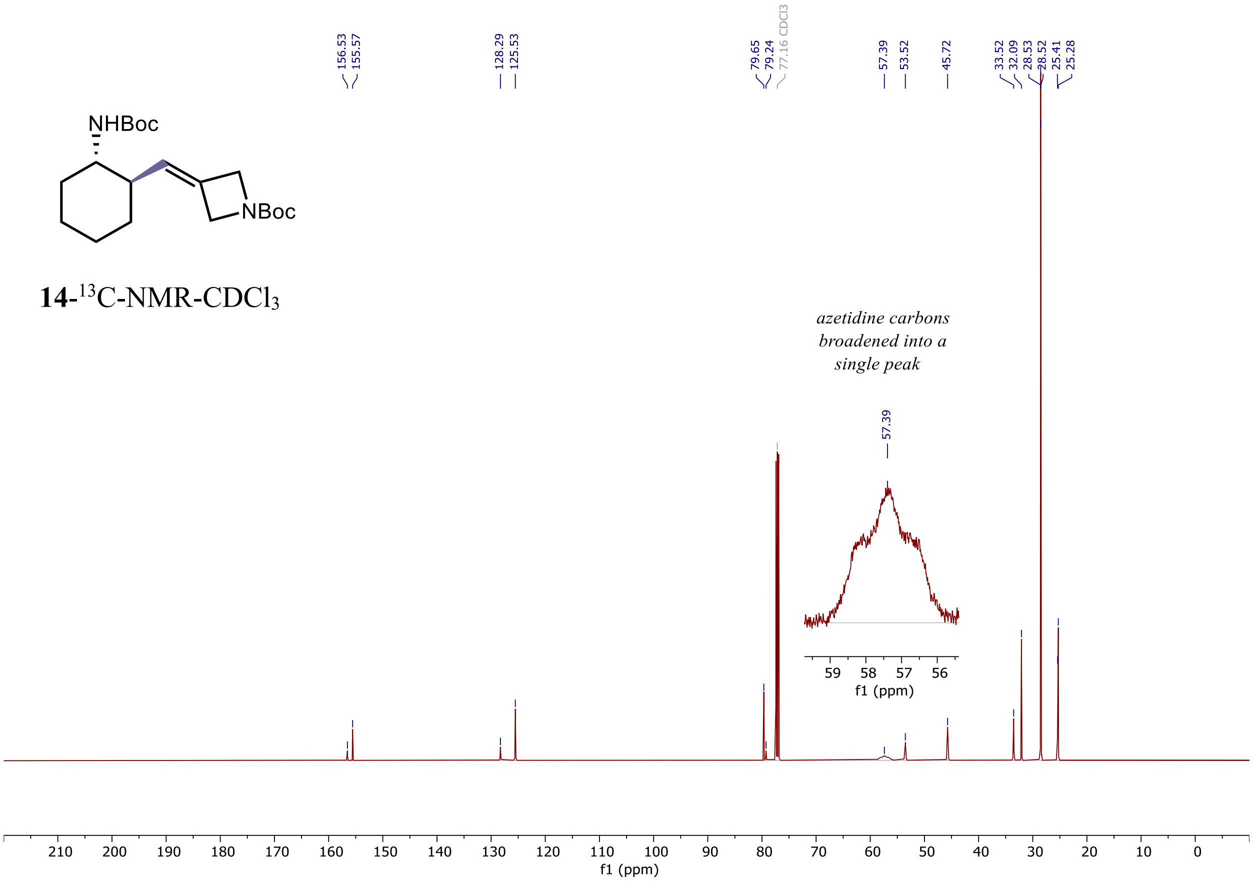

14-^{13}C-NMR-CDCl$_3$

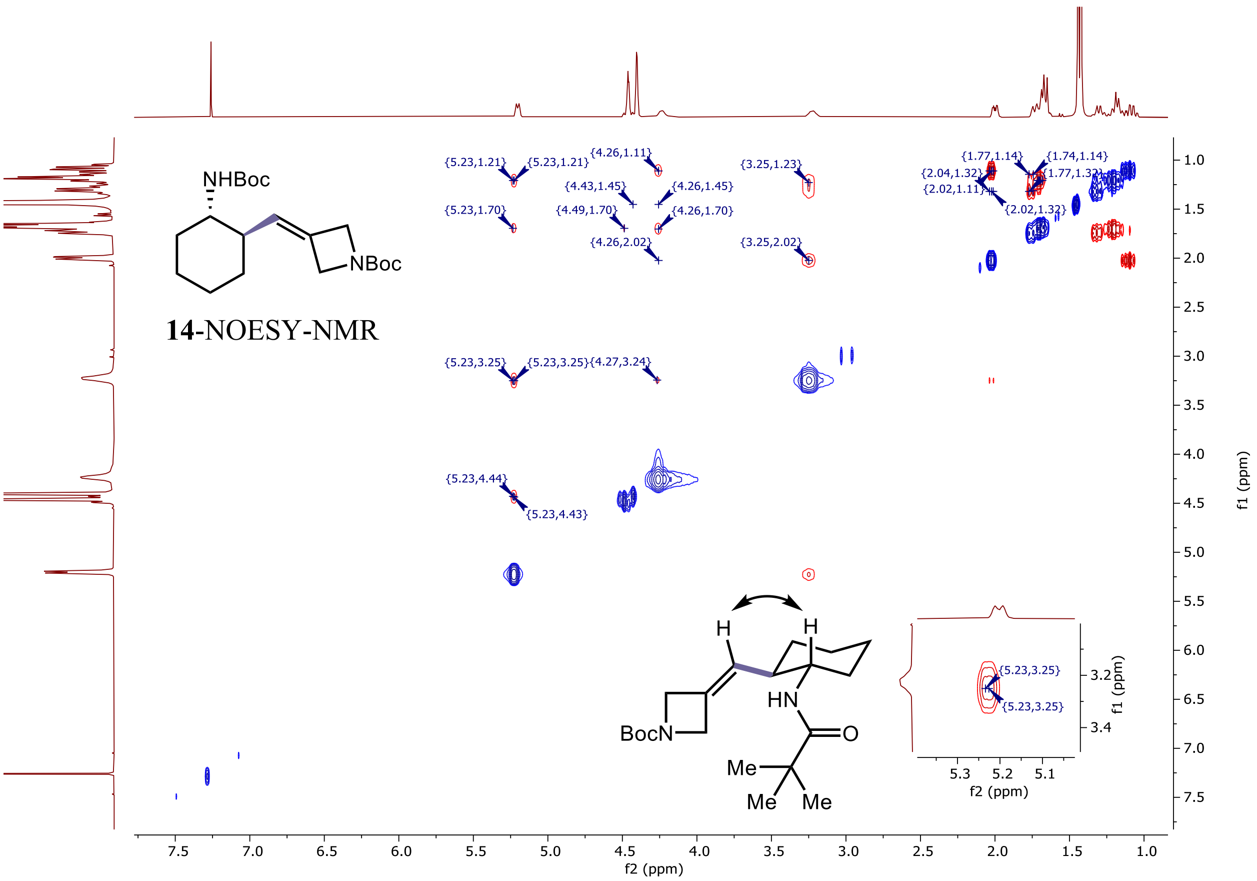

14-NOESY-NMR

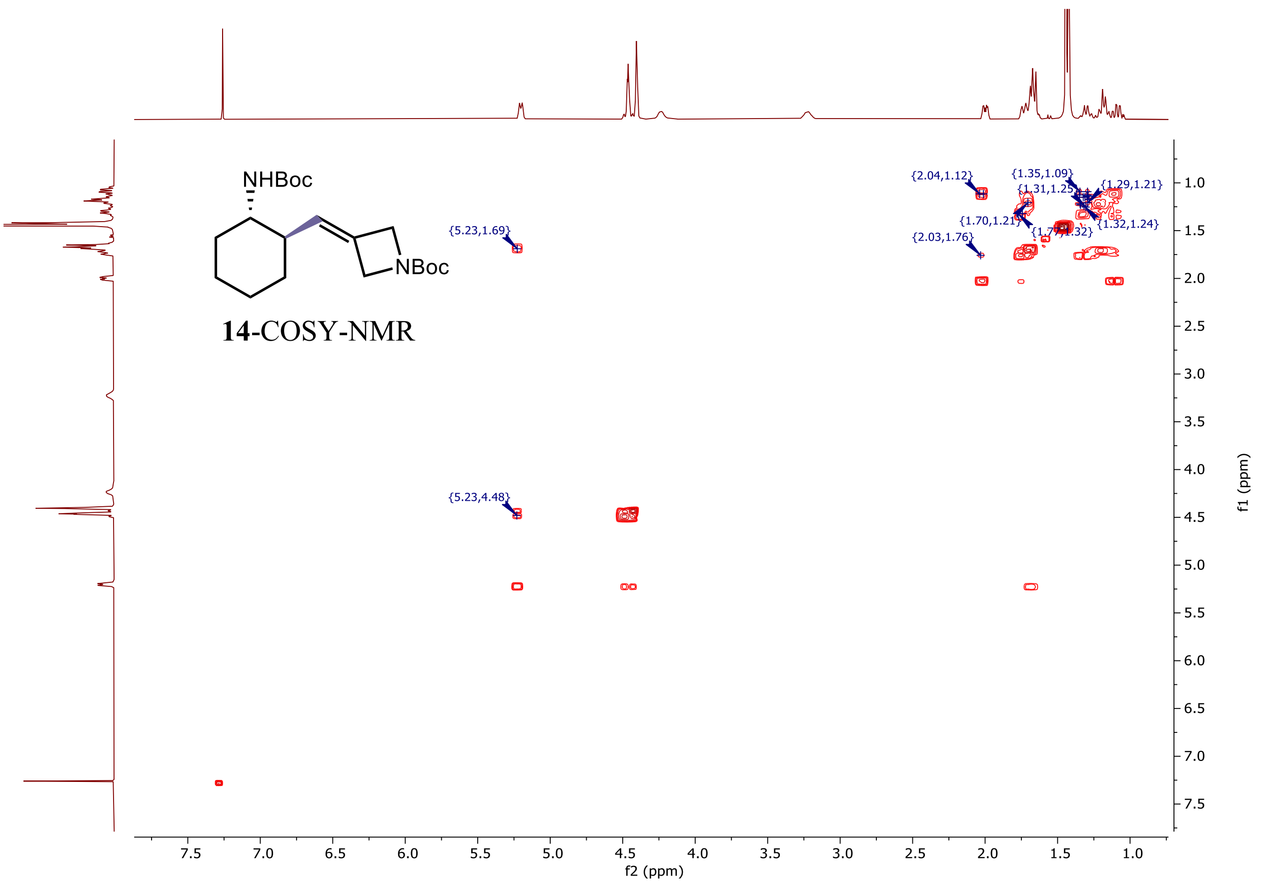
NHBoc
NBoc
14-COSY-NMR
f2 (ppm)
f1 (ppm)

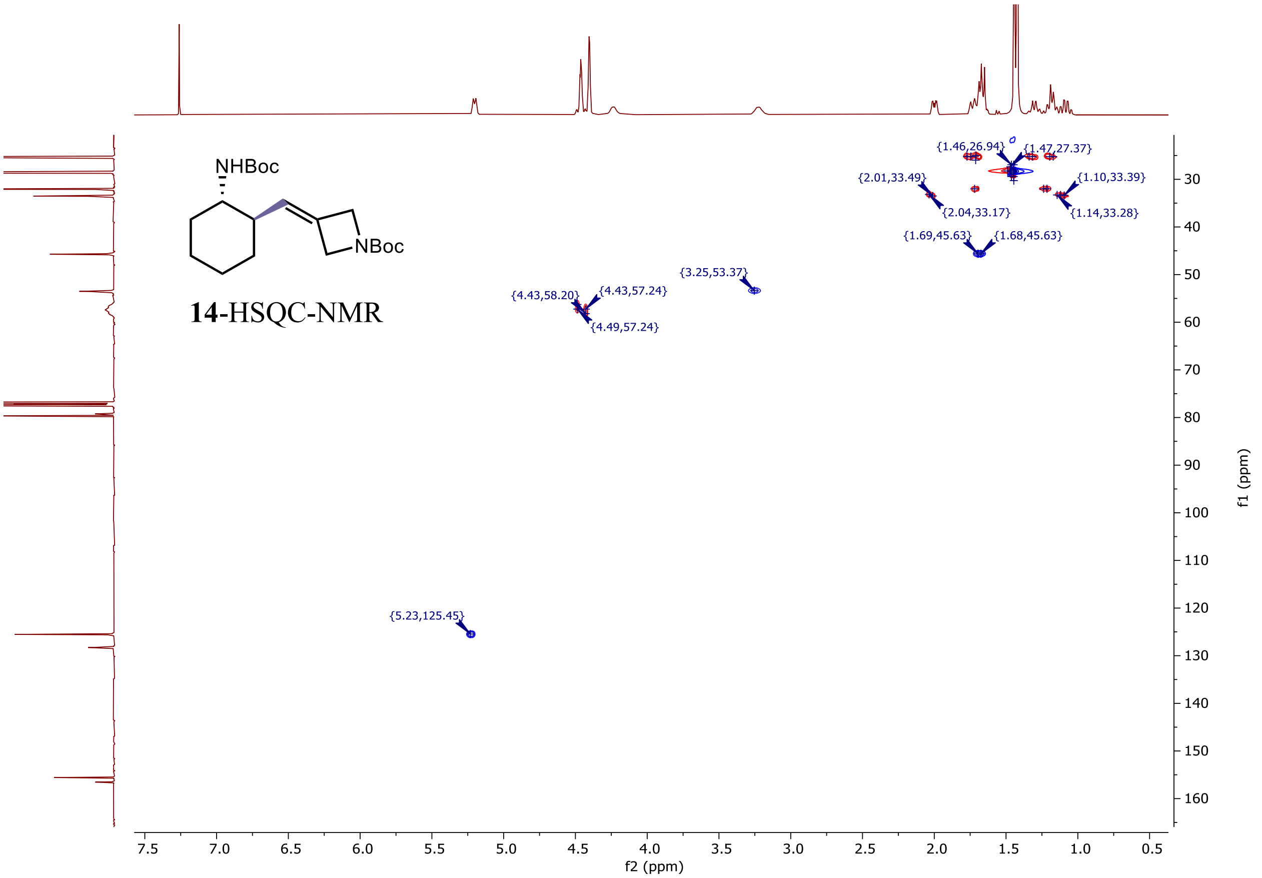
NHBoc
NBoc
14-HSQC-NMR
f2 (ppm)
f1 (ppm)

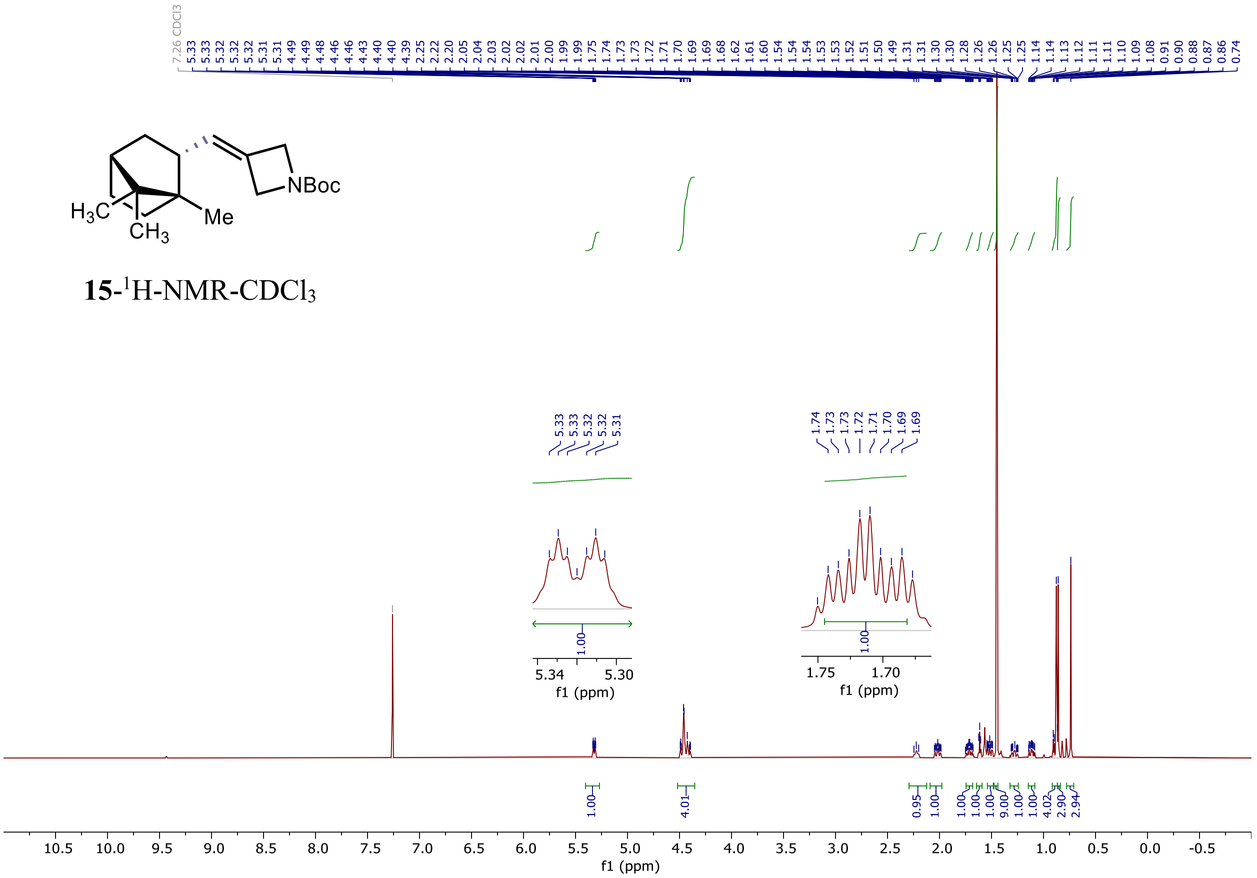

7.26 CDCl3
5.33 5.33 5.32 5.32 5.32 5.31 5.31 4.49 4.49 4.48 4.46 4.46 4.43 4.40 4.40 4.39 2.25 2.22 2.20 2.05 2.04 2.03 2.02 2.02 2.01 2.00 1.99 1.99 1.75 1.74 1.73 1.73 1.72 1.71 1.70 1.69 1.69 1.68 1.62 1.61 1.60 1.54 1.54 1.54 1.53 1.53 1.52 1.51 1.50 1.49 1.31 1.31 1.30 1.30 1.28 1.26 1.26 1.25 1.25 1.14 1.14 1.13 1.12 1.11 1.11 1.10 1.09 1.08 0.91 0.90 0.88 0.87 0.86 0.74
H_3C
CH_3
Me
NBoc
15-^{1}H-NMR-$CDCl_3$
1.00
4.01
0.95
1.00
1.00
1.00
1.00
9.00
1.00
1.00
4.02
2.90
2.94
f1 (ppm)

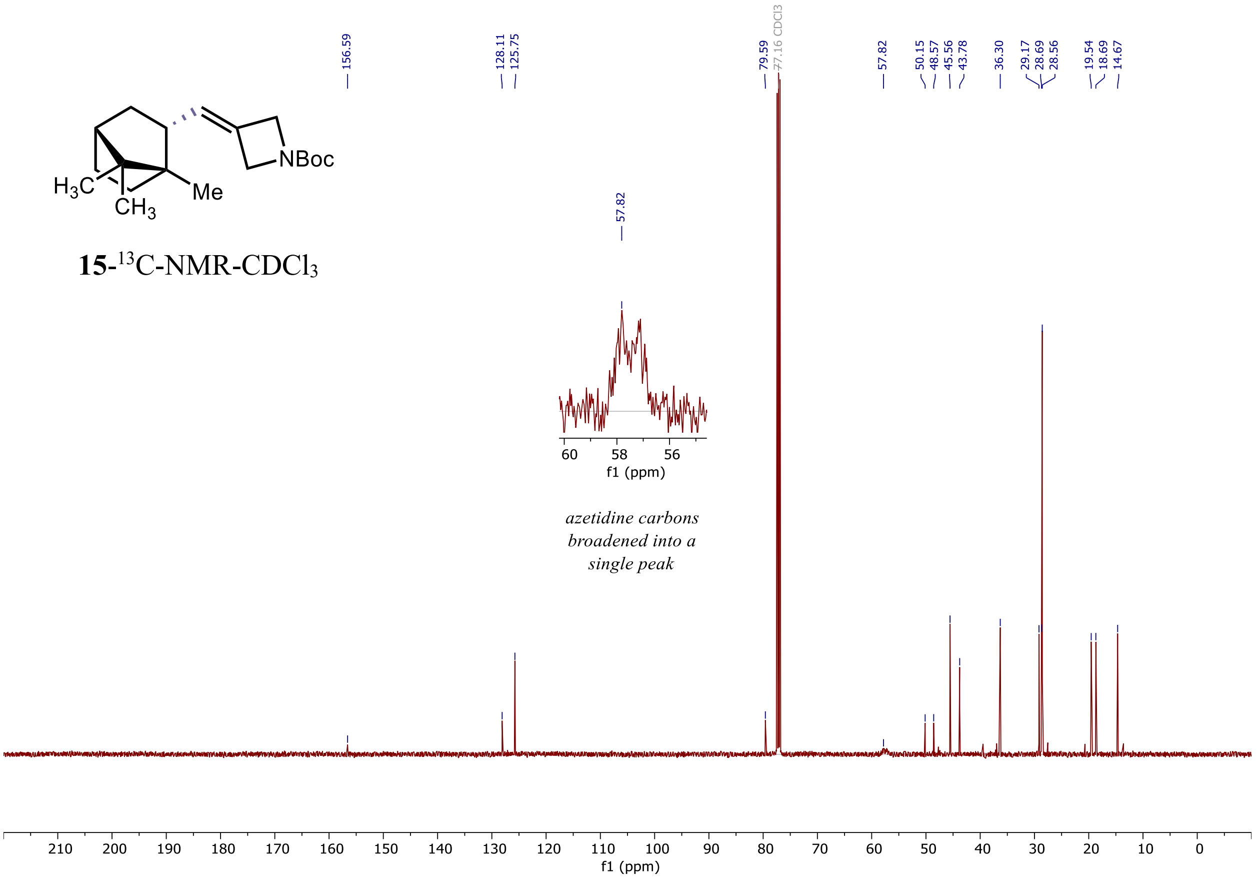

156.59
128.11
125.75
79.59
77.16 CDCl3
57.82
50.15
48.57
45.56
43.78
36.30
29.17
28.69
28.56
19.54
18.69
14.67
H_3C
CH_3
Me
NBoc
15-^{13}C-NMR-$CDCl_3$
azetidine carbons broadened into a single peak
f1 (ppm)

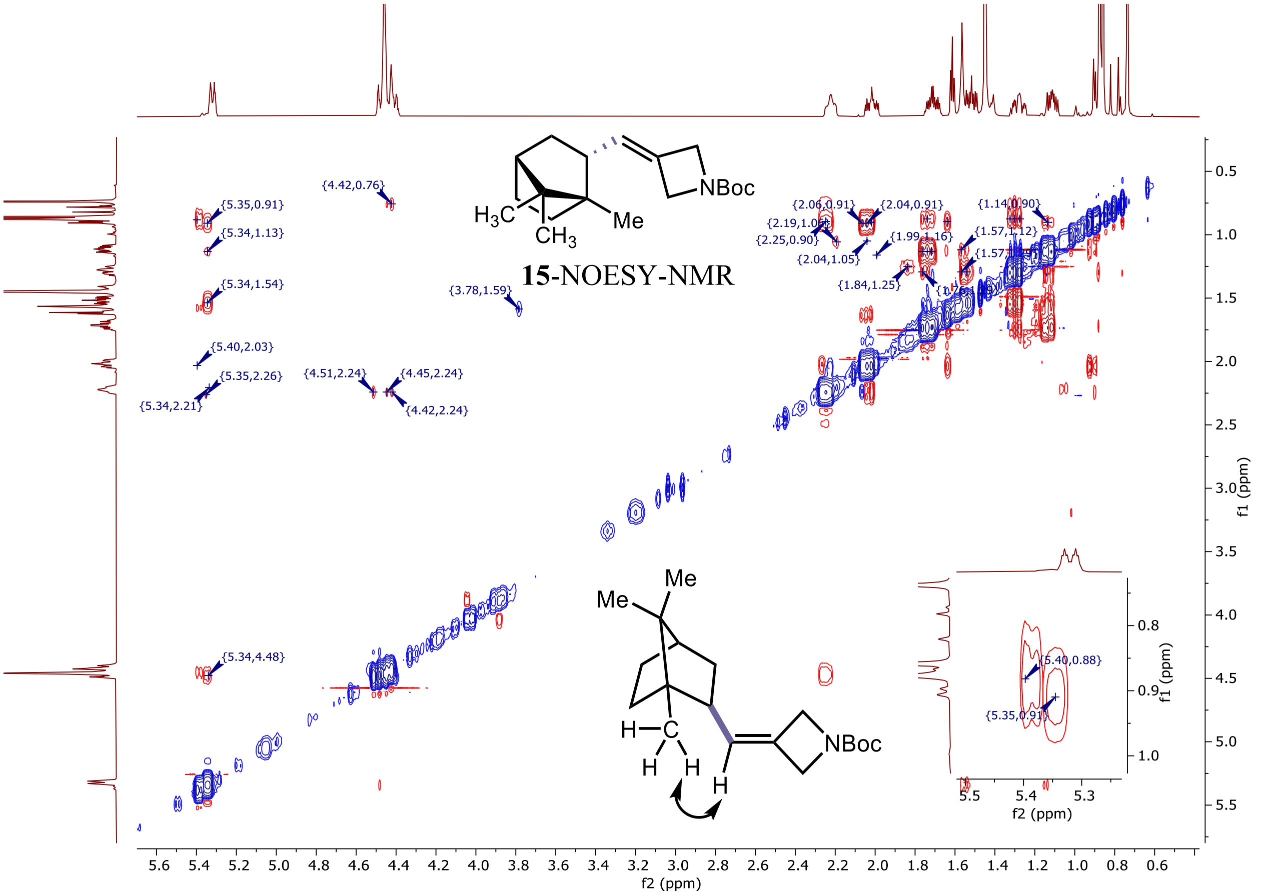
NBoc
H3C
CH3
Me
15-NOESY-NMR
Me
Me
H—C
H H
H
NBoc
f2 (ppm)
f1 (ppm)

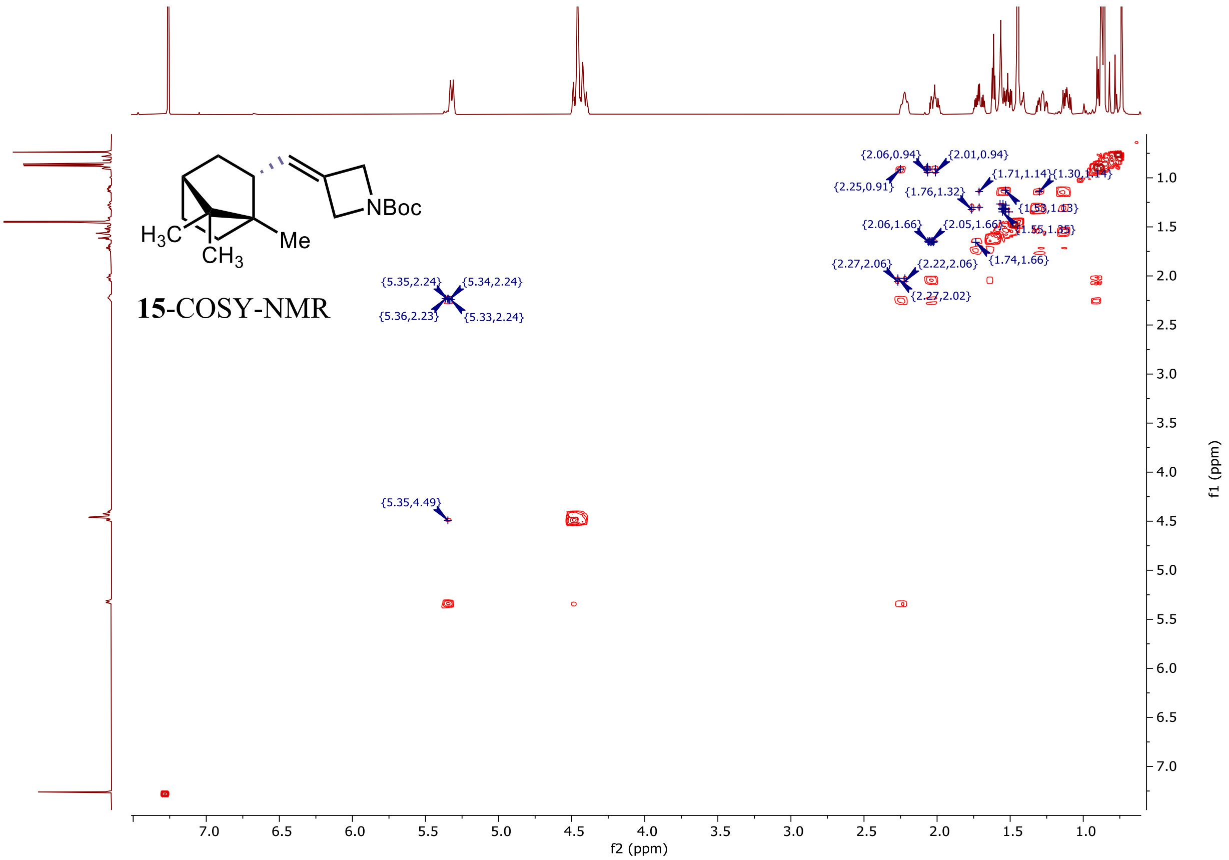
NBoc
H3C
CH3
Me
15-COSY-NMR
f2 (ppm)
f1 (ppm)

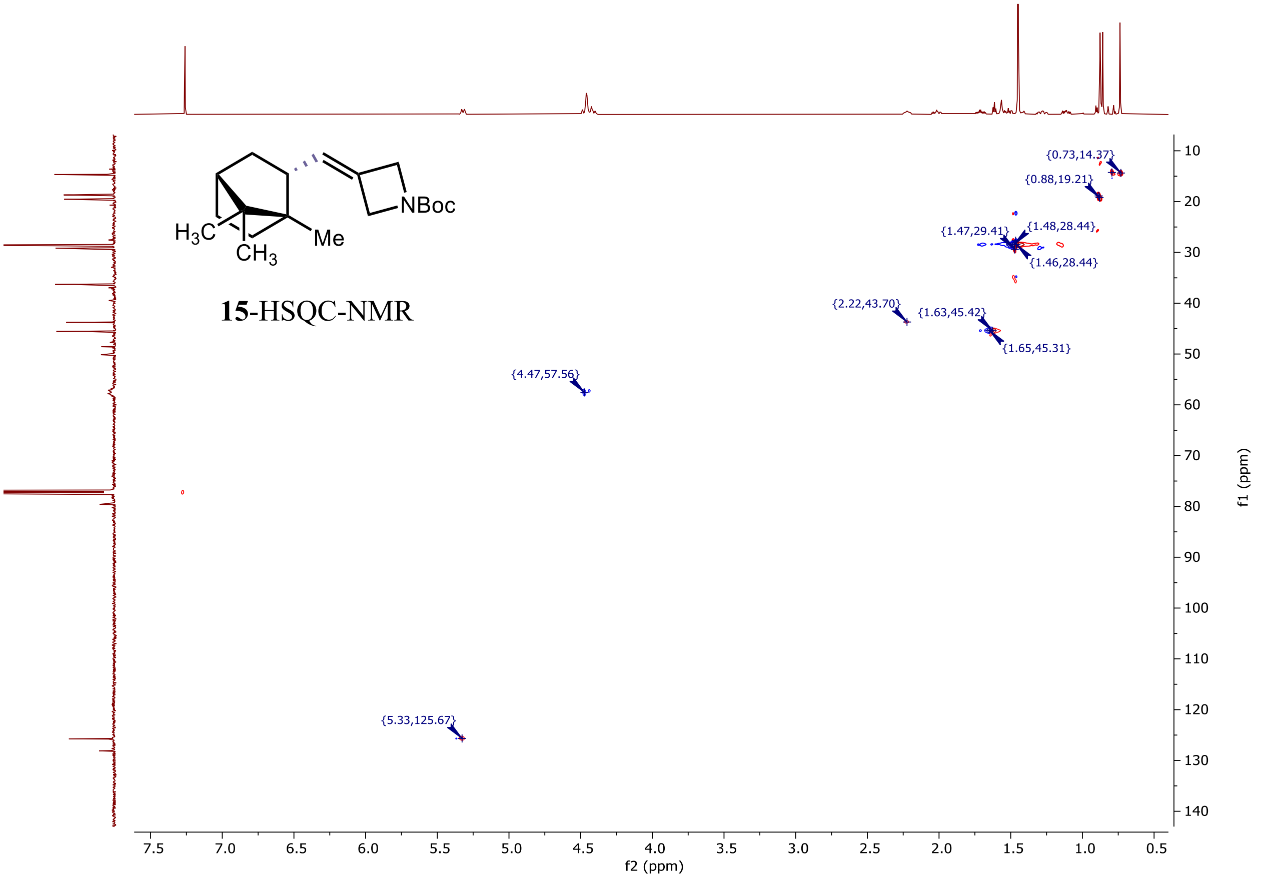
NBoc
H3C
CH3
Me
15-HSQC-NMR
f2 (ppm)
f1 (ppm)

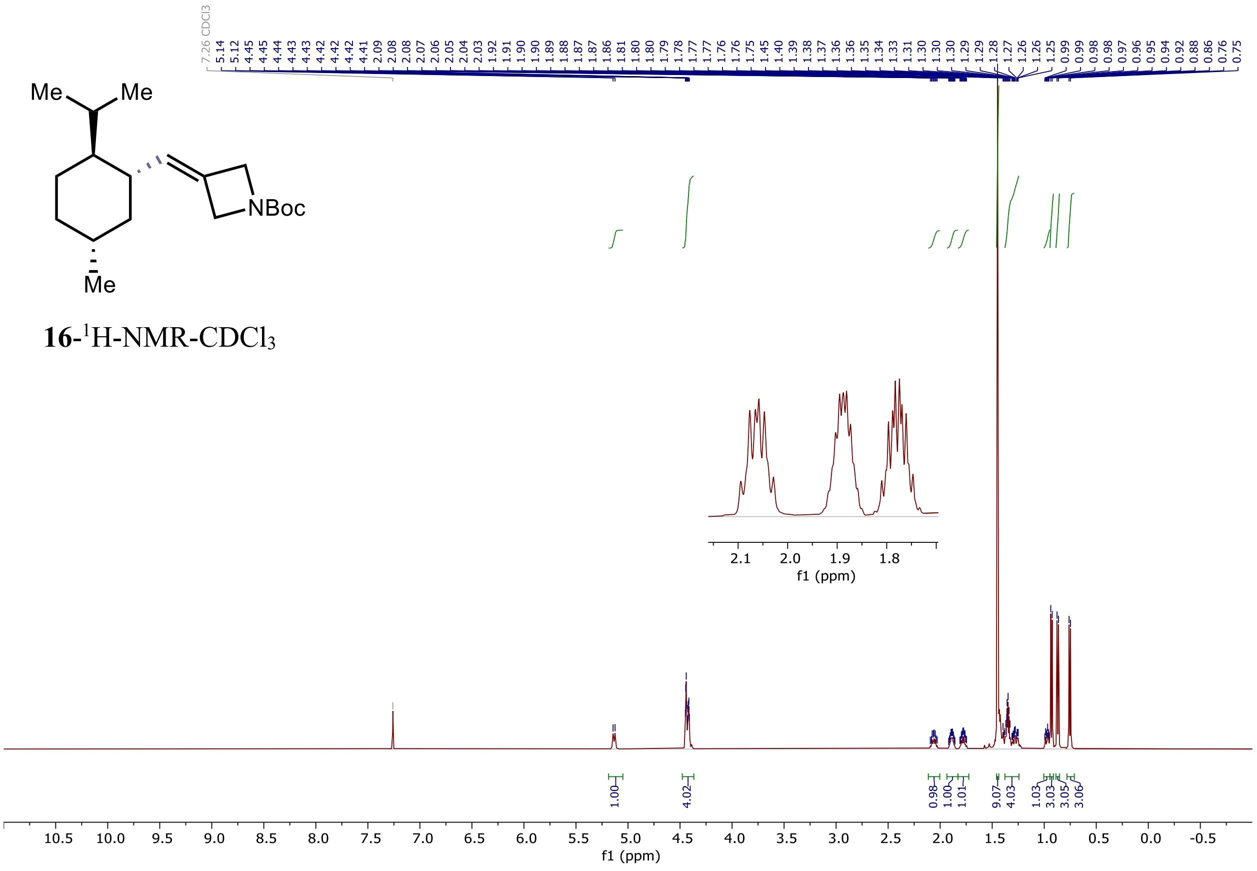
Me
Me
NBoc
Me
16-1H-NMR-CDCl3
f1 (ppm)

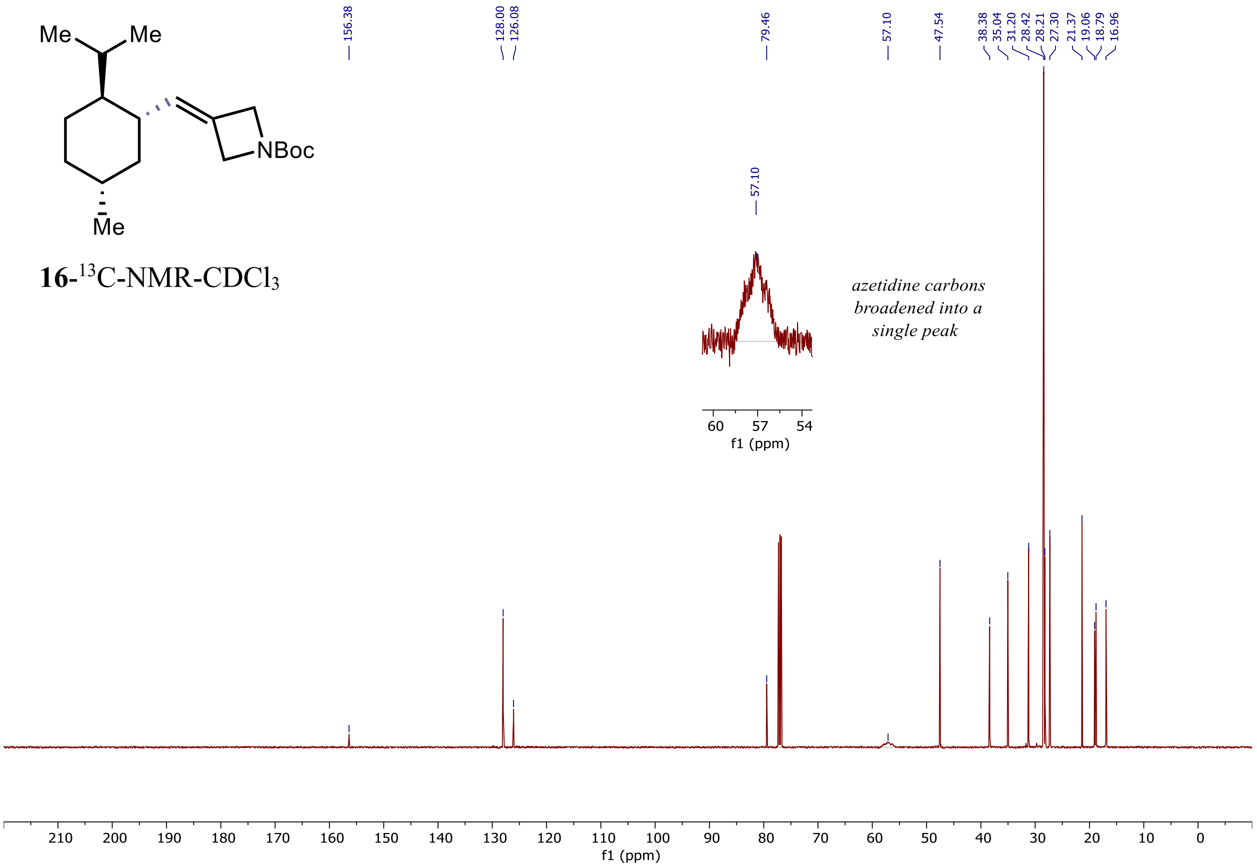

Me
Me
NBoc
Me
16-^{13}C-NMR-CDCl$_3$
156.38
128.00
126.08
79.46
57.10
47.54
38.38
35.04
31.20
28.42
28.21
27.30
21.37
19.06
18.79
16.96
azetidine carbons broadened into a single peak
f1 (ppm)

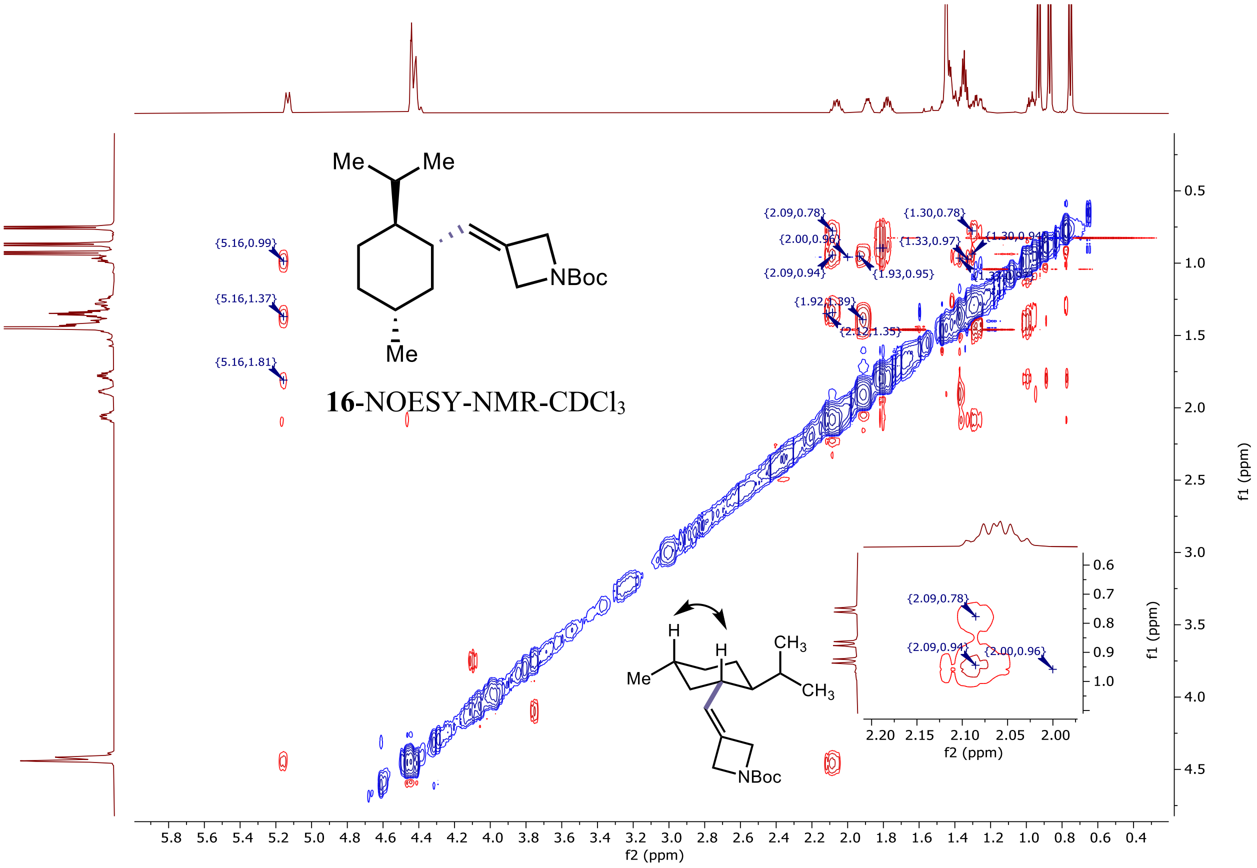

Me
Me
NBoc
Me
16-NOESY-NMR-CDCl$_3$
{5.16,0.99}
{5.16,1.37}
{5.16,1.81}
{2.09,0.78}
{2.00,0.96}
{2.09,0.94}
{1.93,0.95}
{1.30,0.78}
{1.33,0.97}
{1.92,1.39}
H
H
CH$_3$
CH$_3$
Me
NBoc
f2 (ppm)
f1 (ppm)

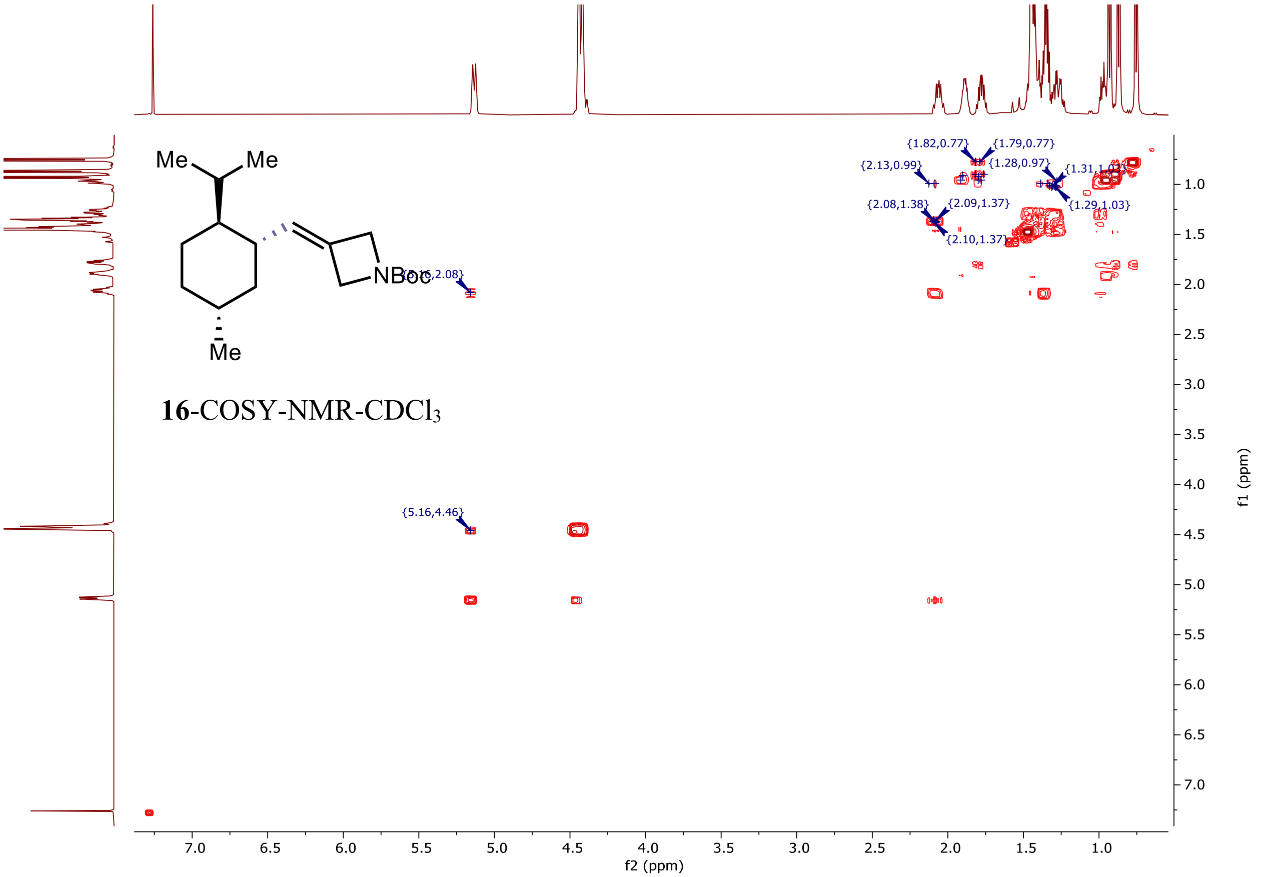

16-COSY-NMR-$CDCl_3$

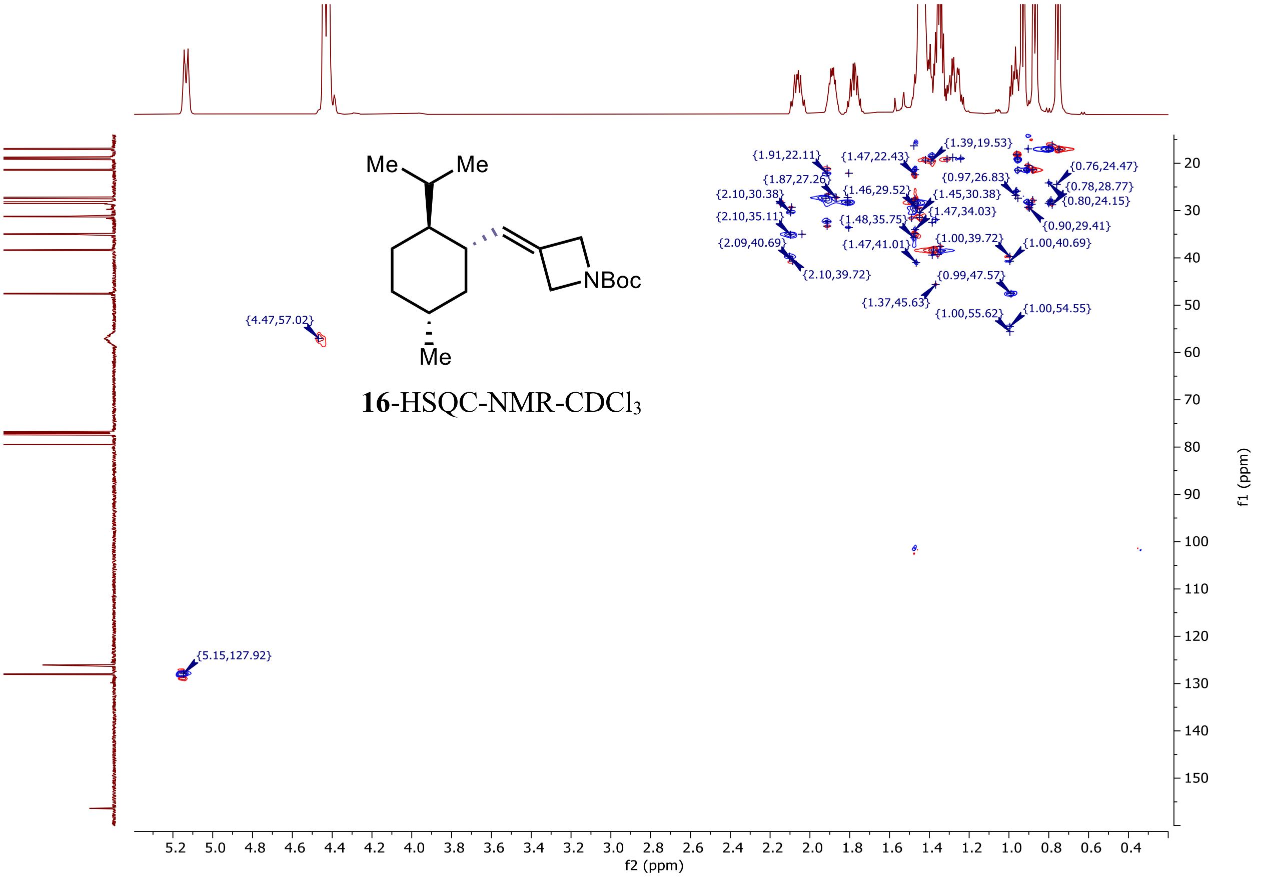

16-HSQC-NMR-$CDCl_3$

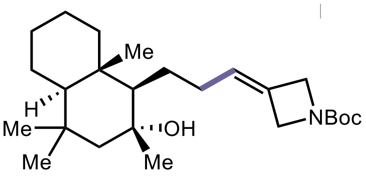

17-^{1}H-NMR-$CDCl_3$

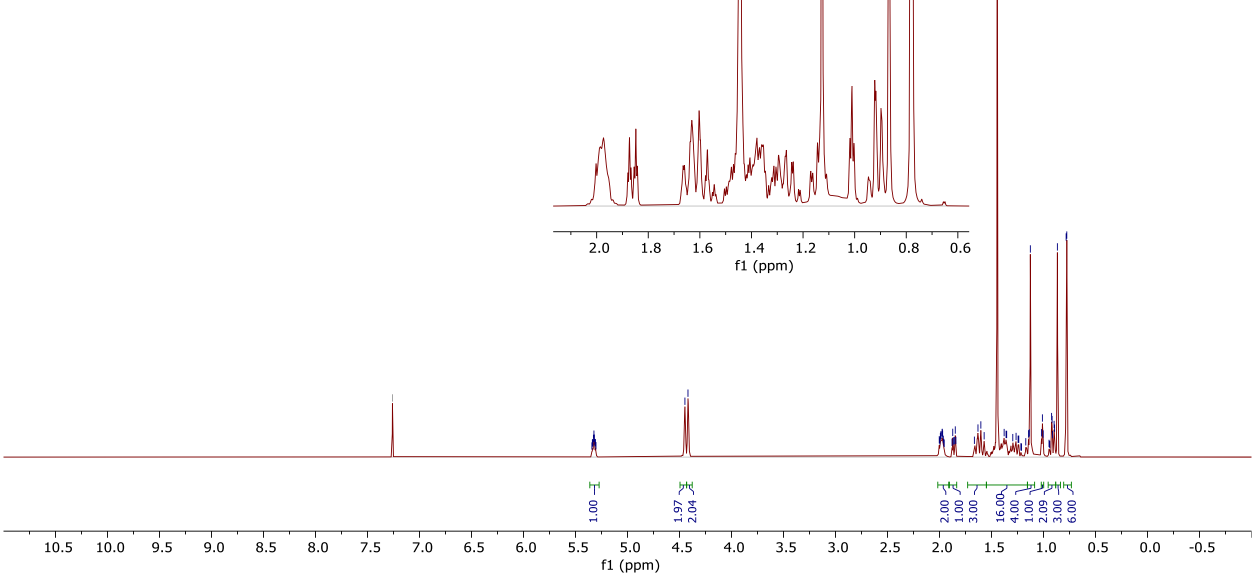

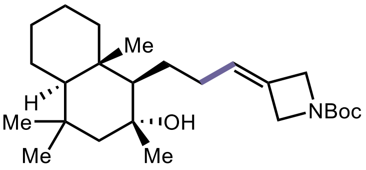

17-^{13}C-NMR-$CDCl_3$

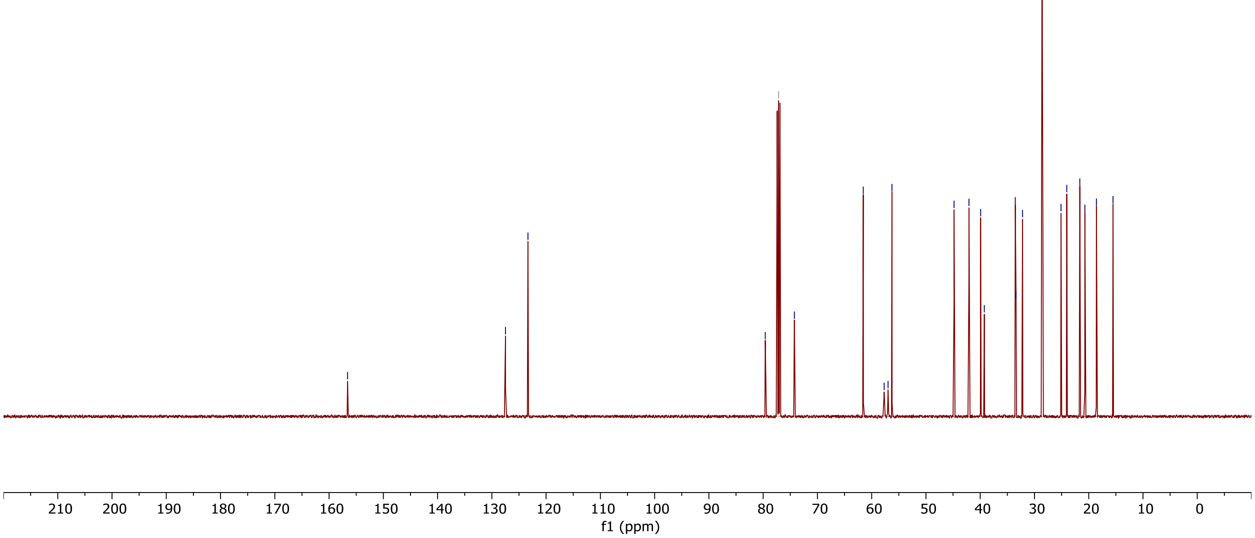

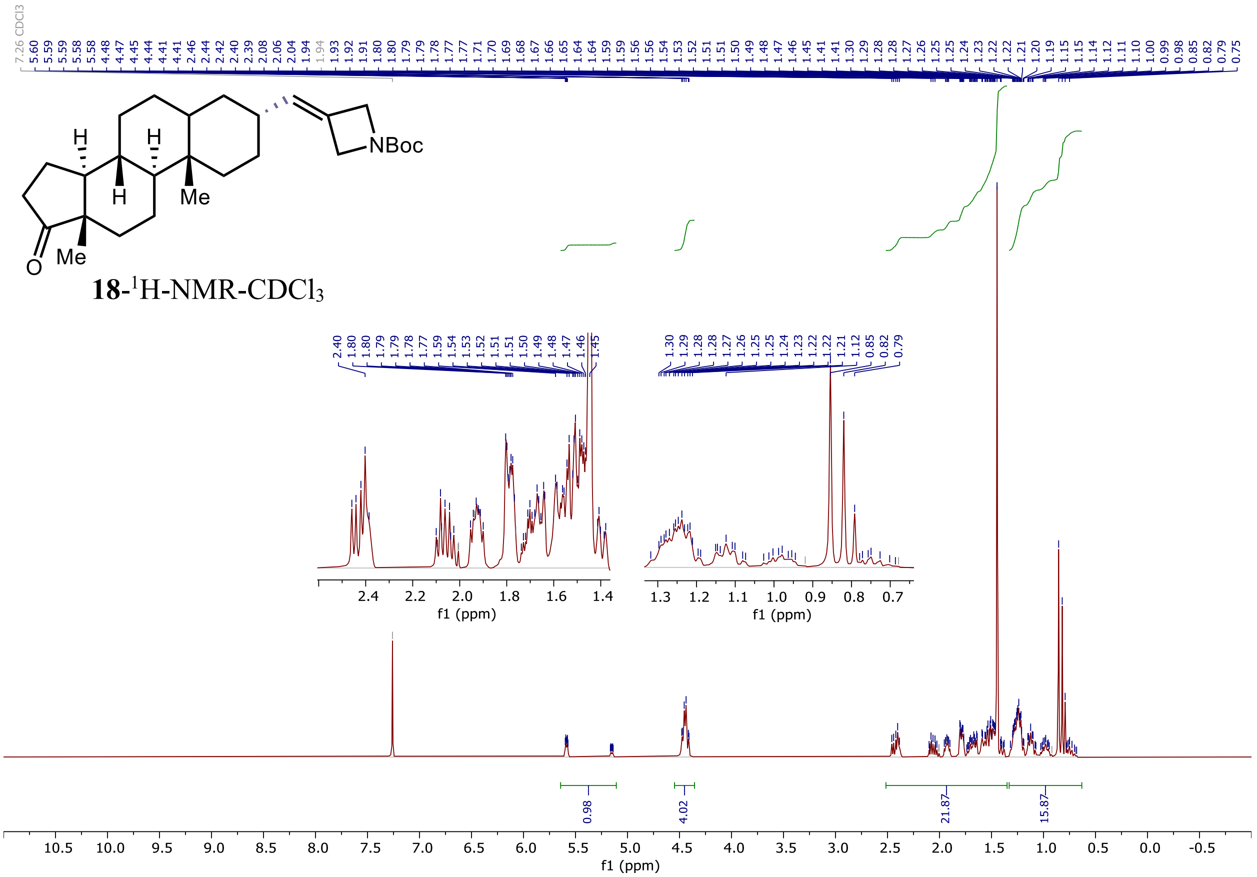

18-1H-NMR-CDCl3

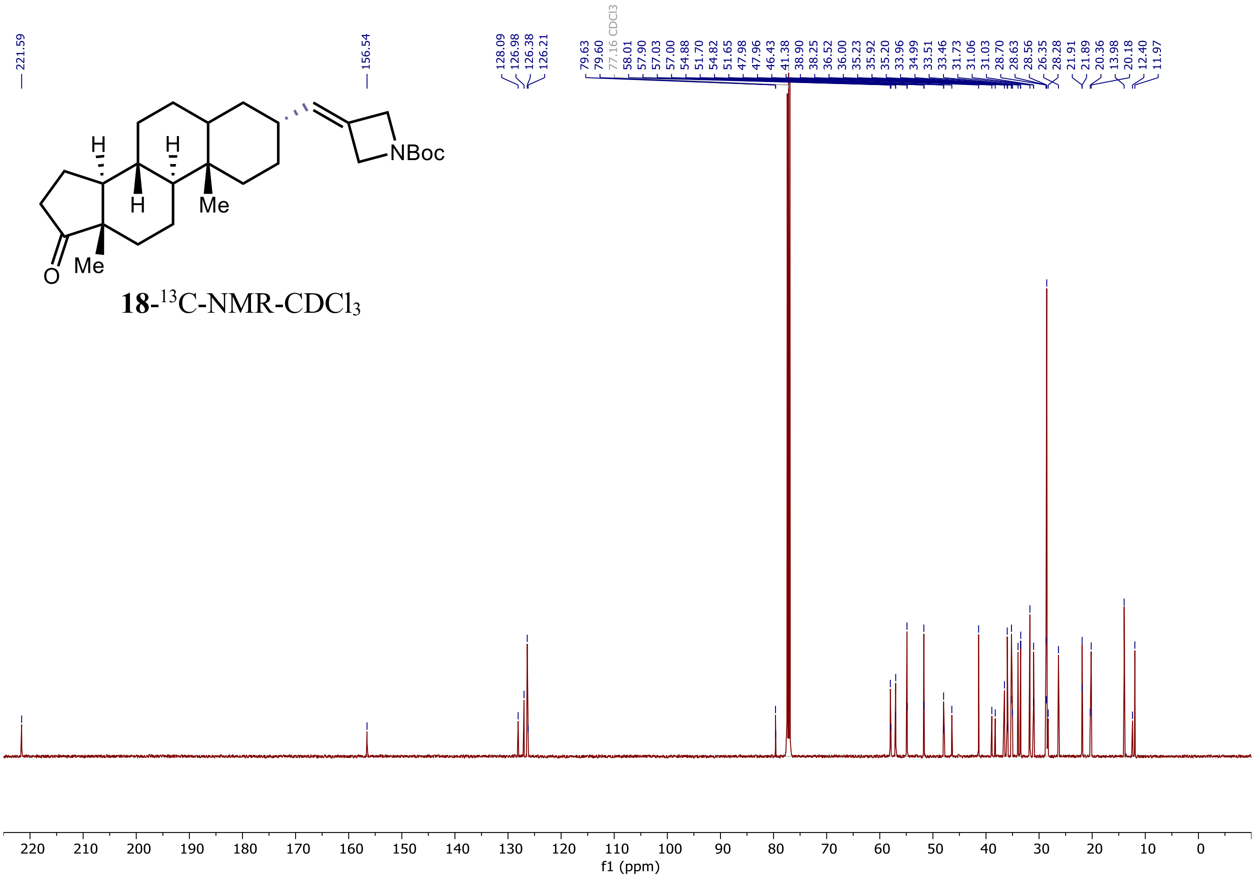

18-13C-NMR-CDCl3

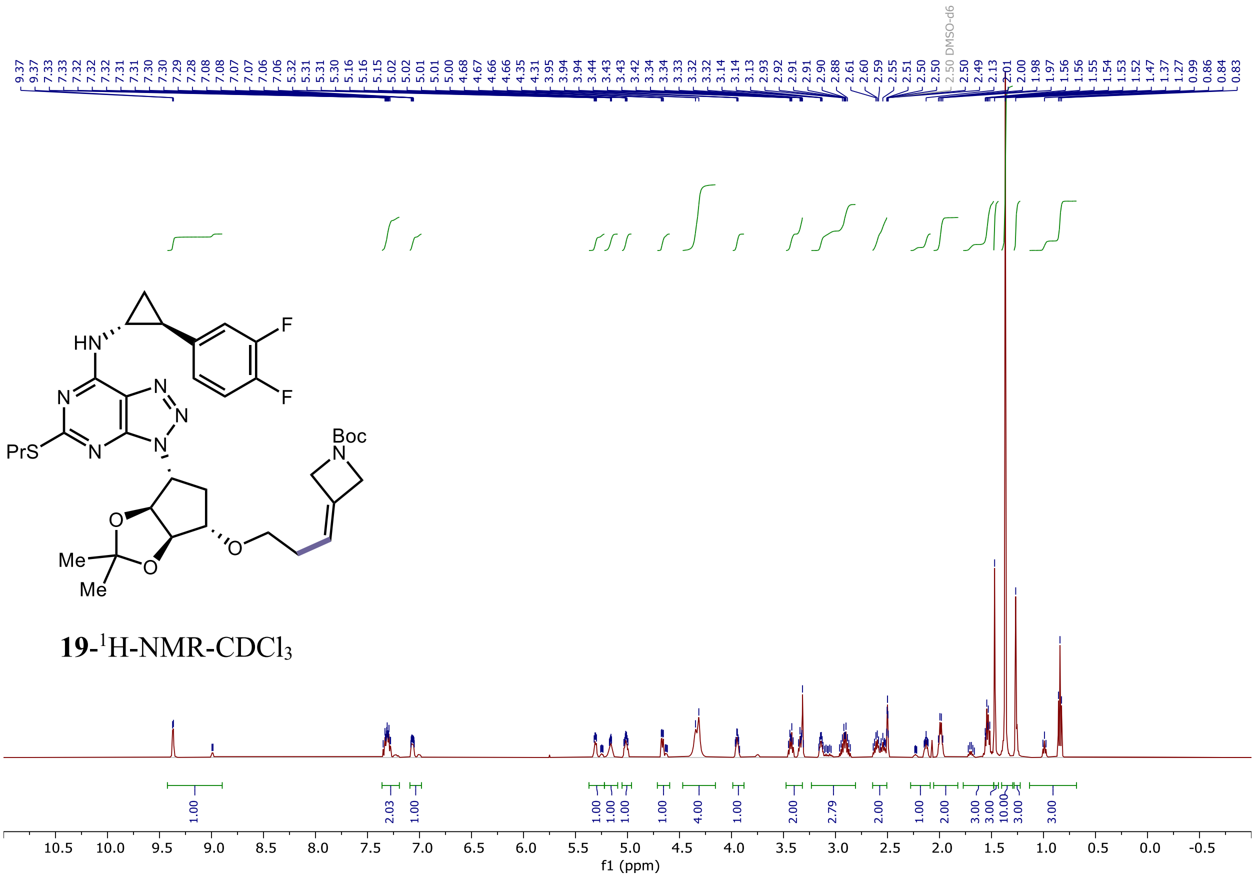

19-^{1}H-NMR-$CDCl_3$

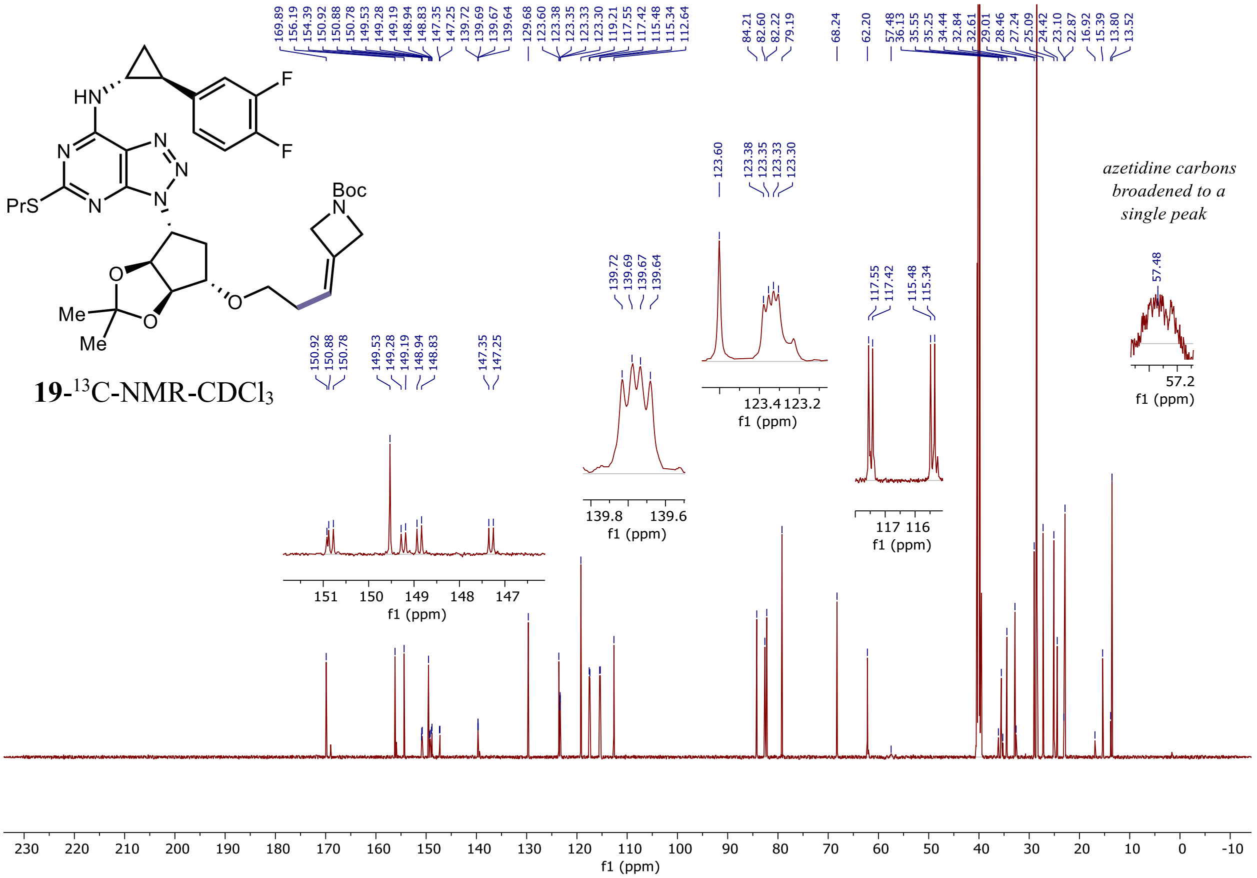

19-^{13}C-NMR-$CDCl_3$

azetidine carbons broadened to a single peak

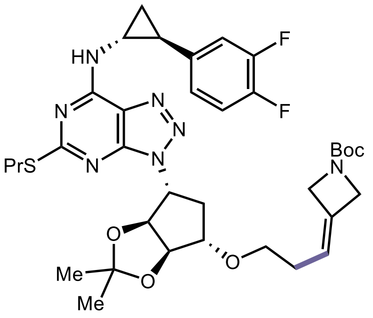

19-^{19}F-NMR-$CDCl_3$

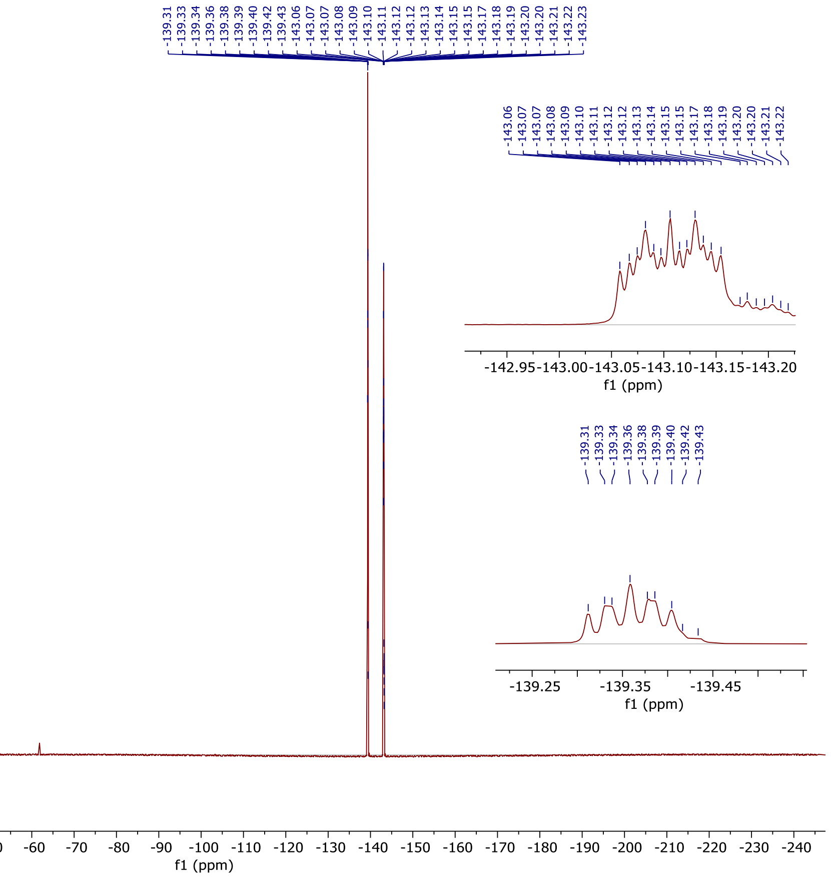

20-^{1}H-NMR-$CDCl_3$

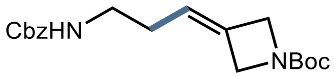

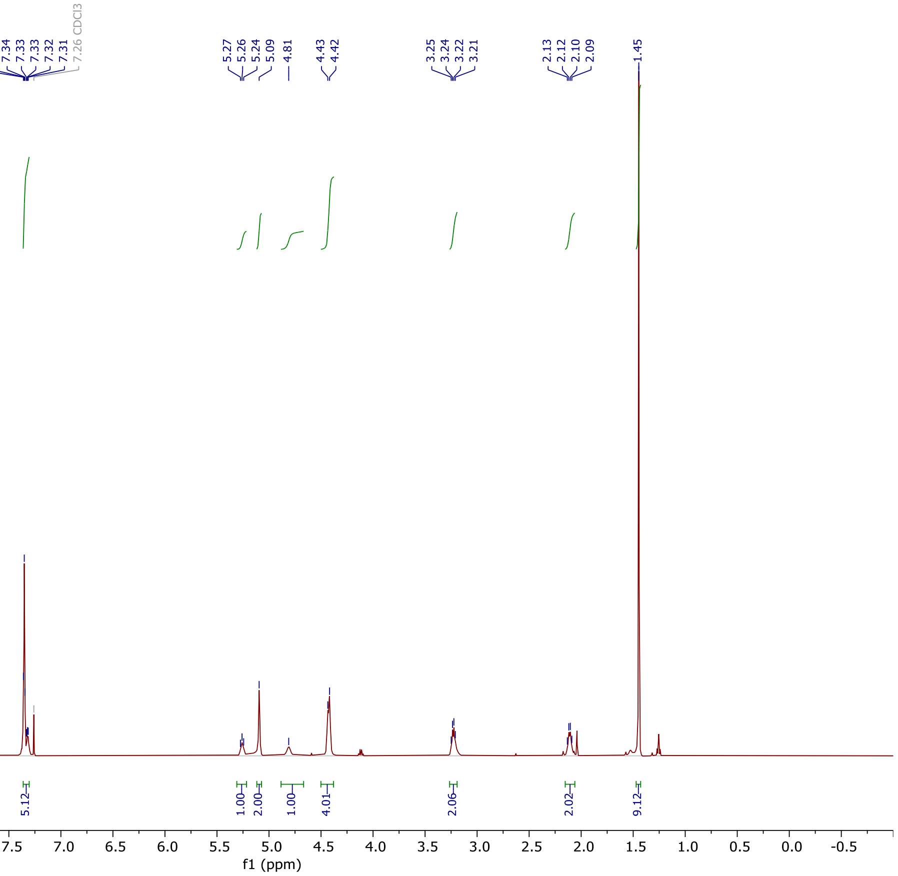

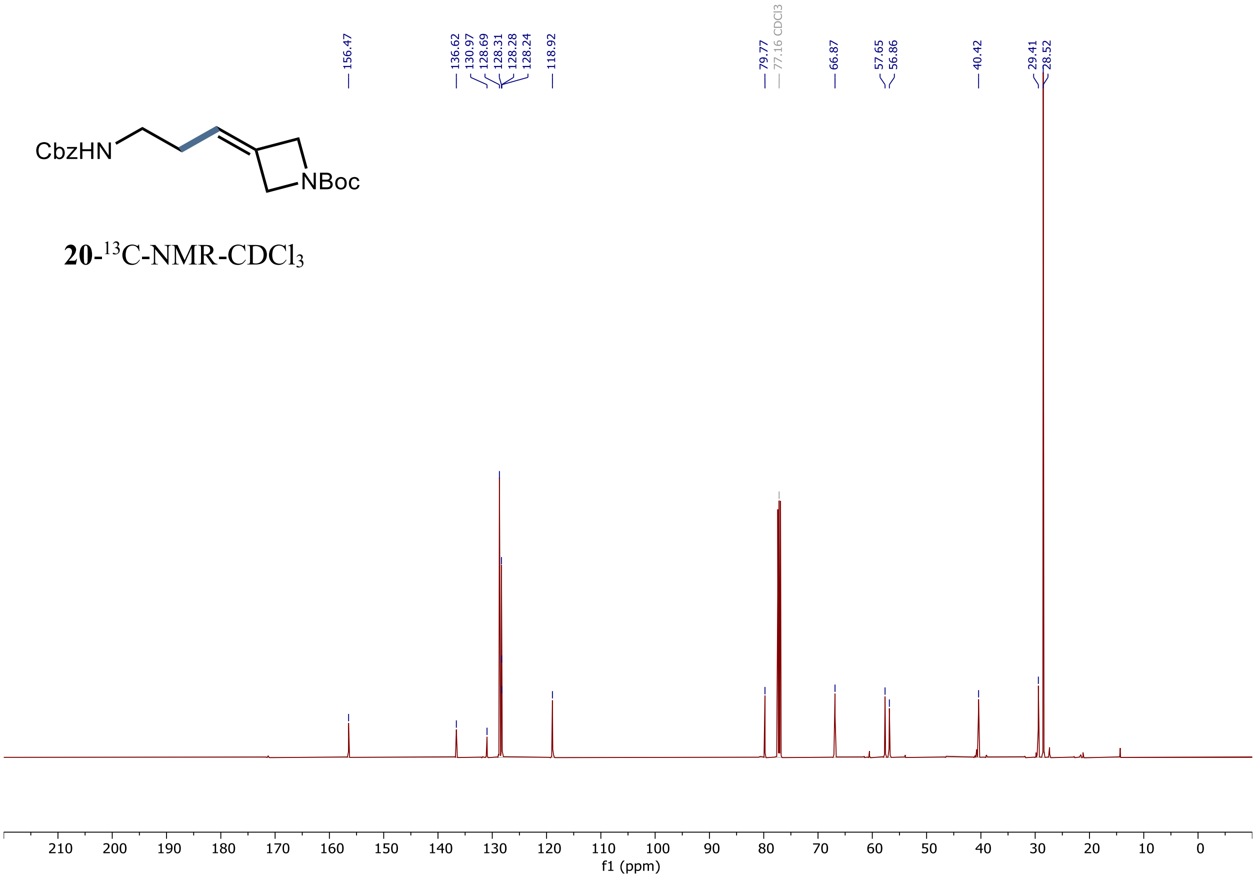

20-^{13}C-NMR-$CDCl_3$

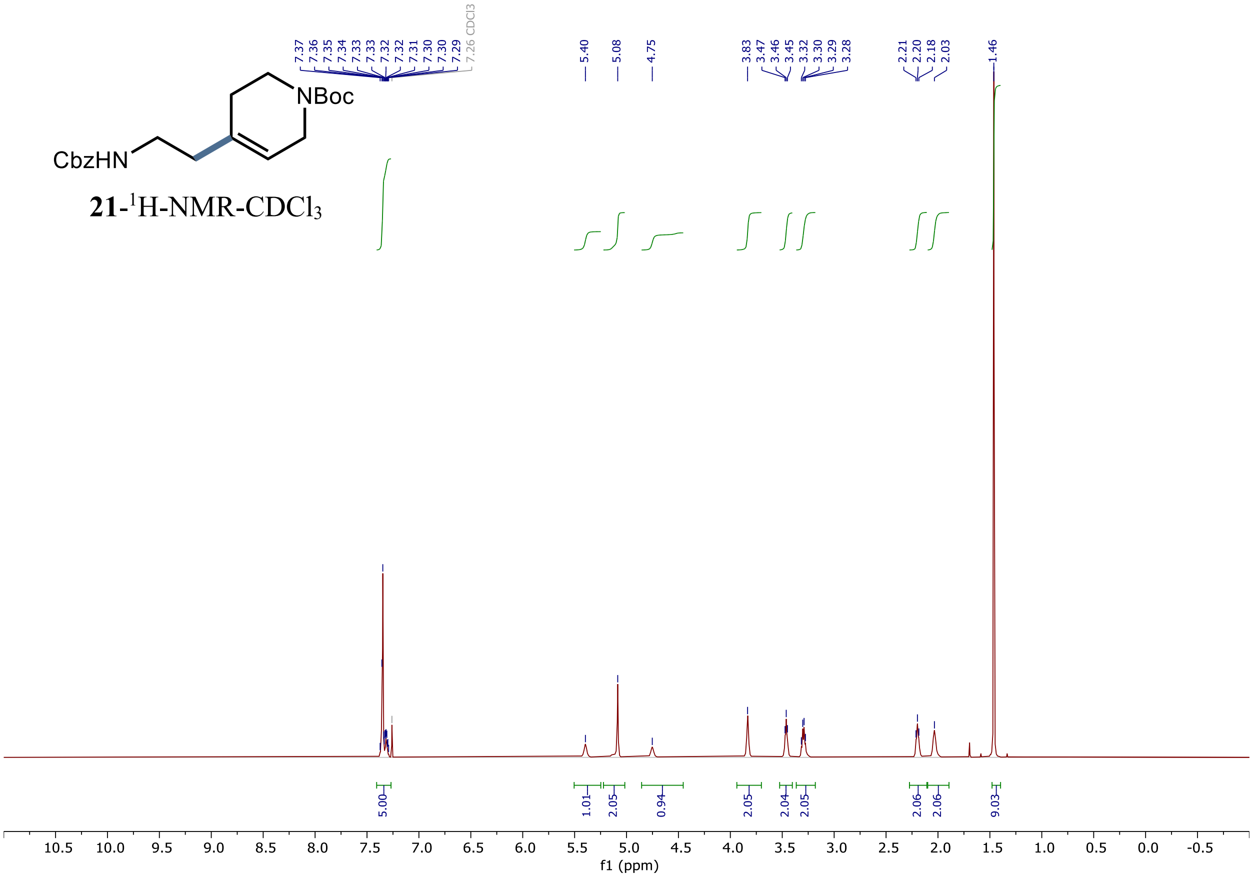

21-^{1}H-NMR-$CDCl_3$

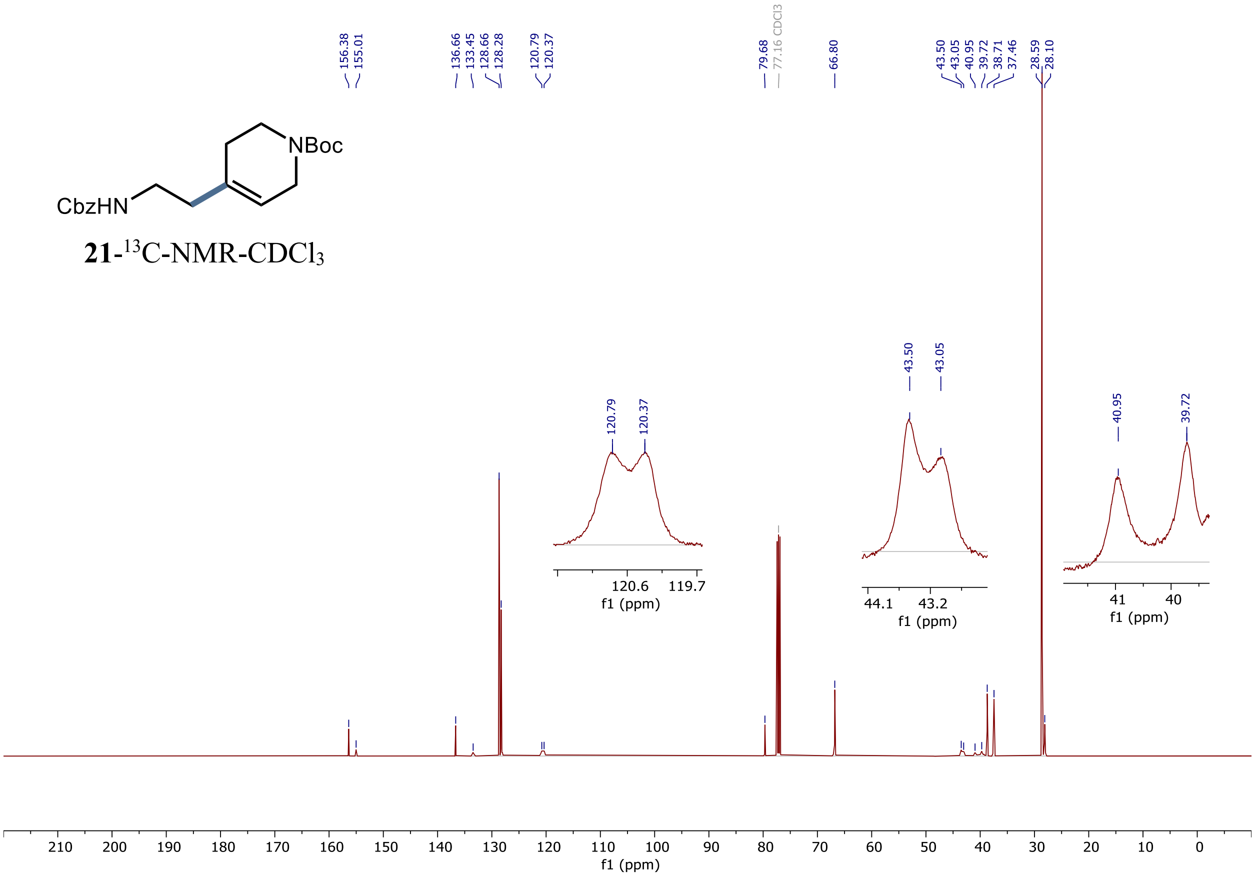

21-^{13}C-NMR-CDCl$_3$

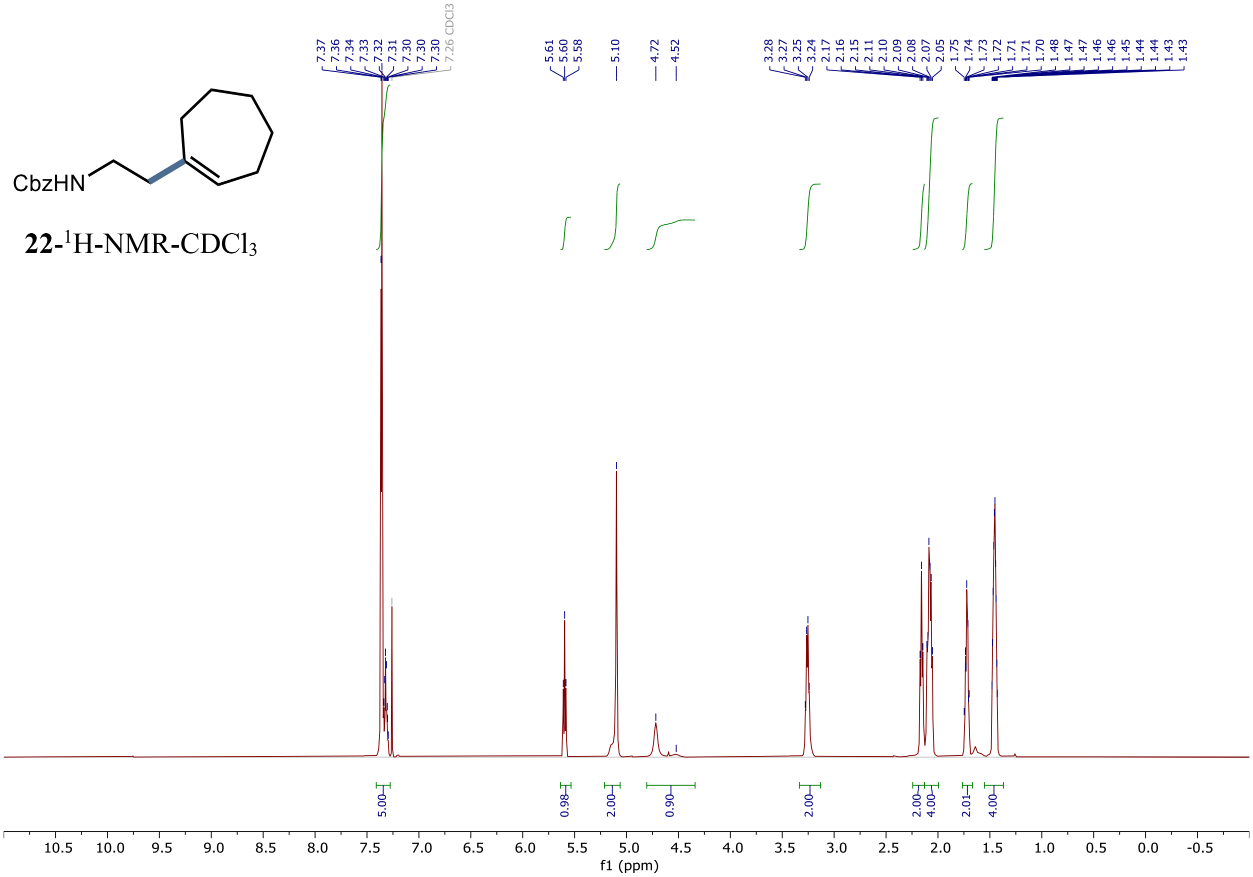

22-^{1}H-NMR-CDCl$_3$

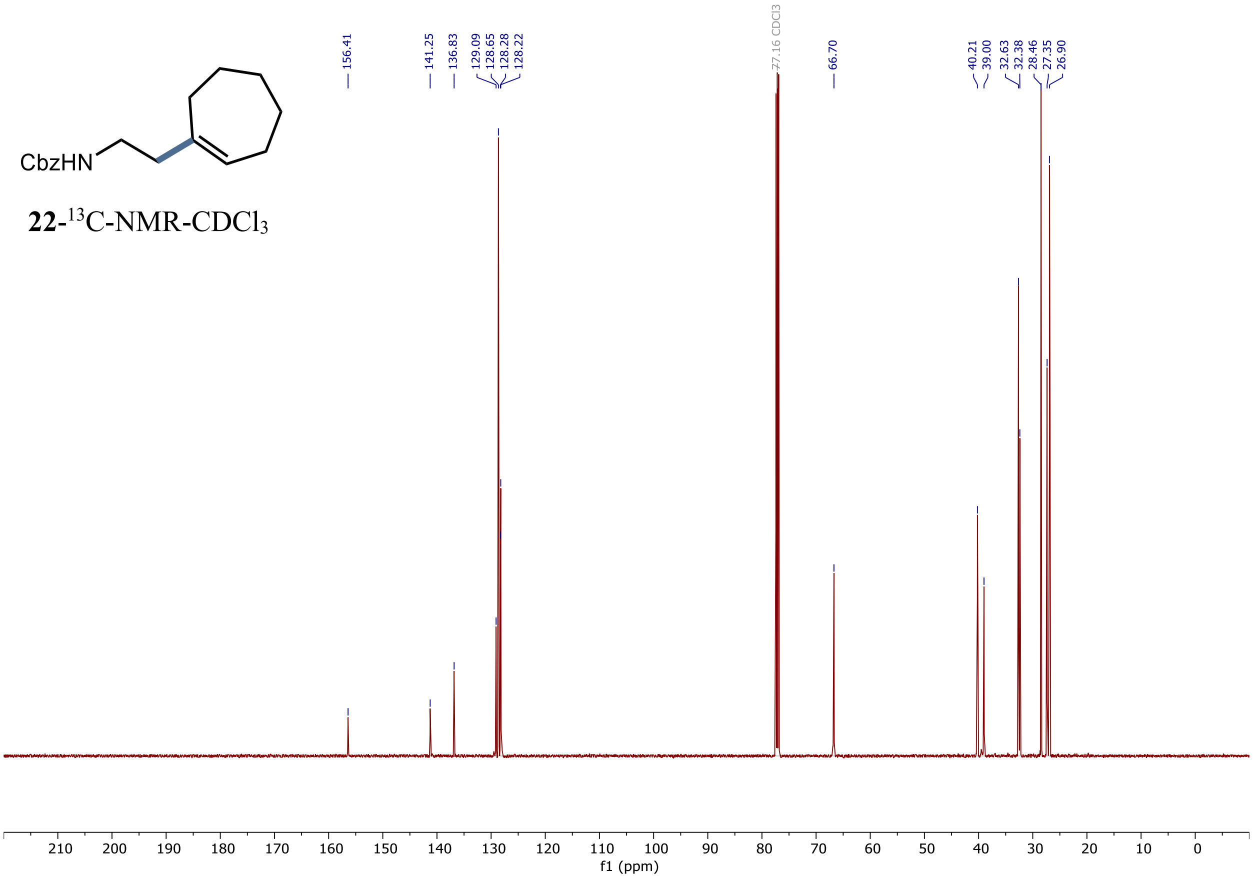

22-^{13}C-NMR-CDCl$_3$

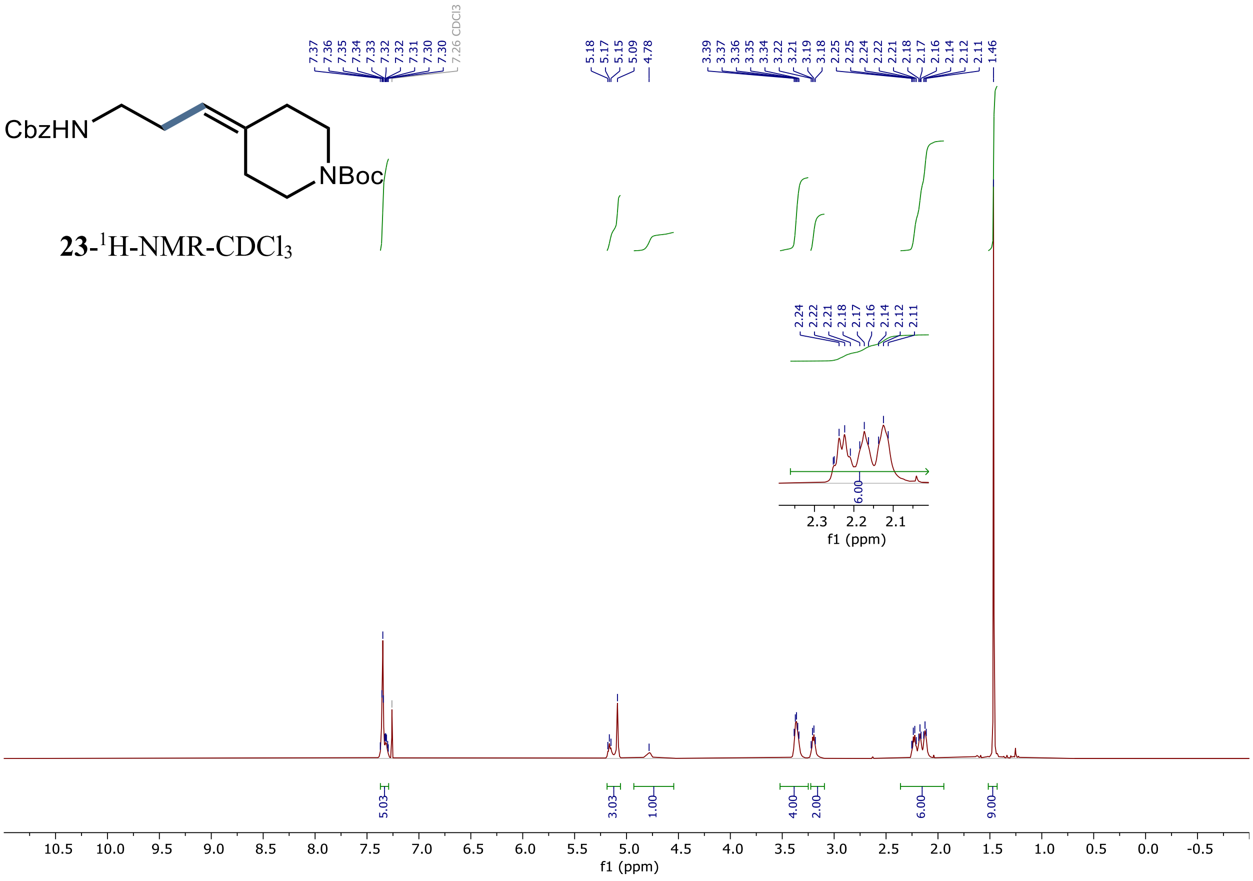

23-^{1}H-NMR-CDCl$_3$

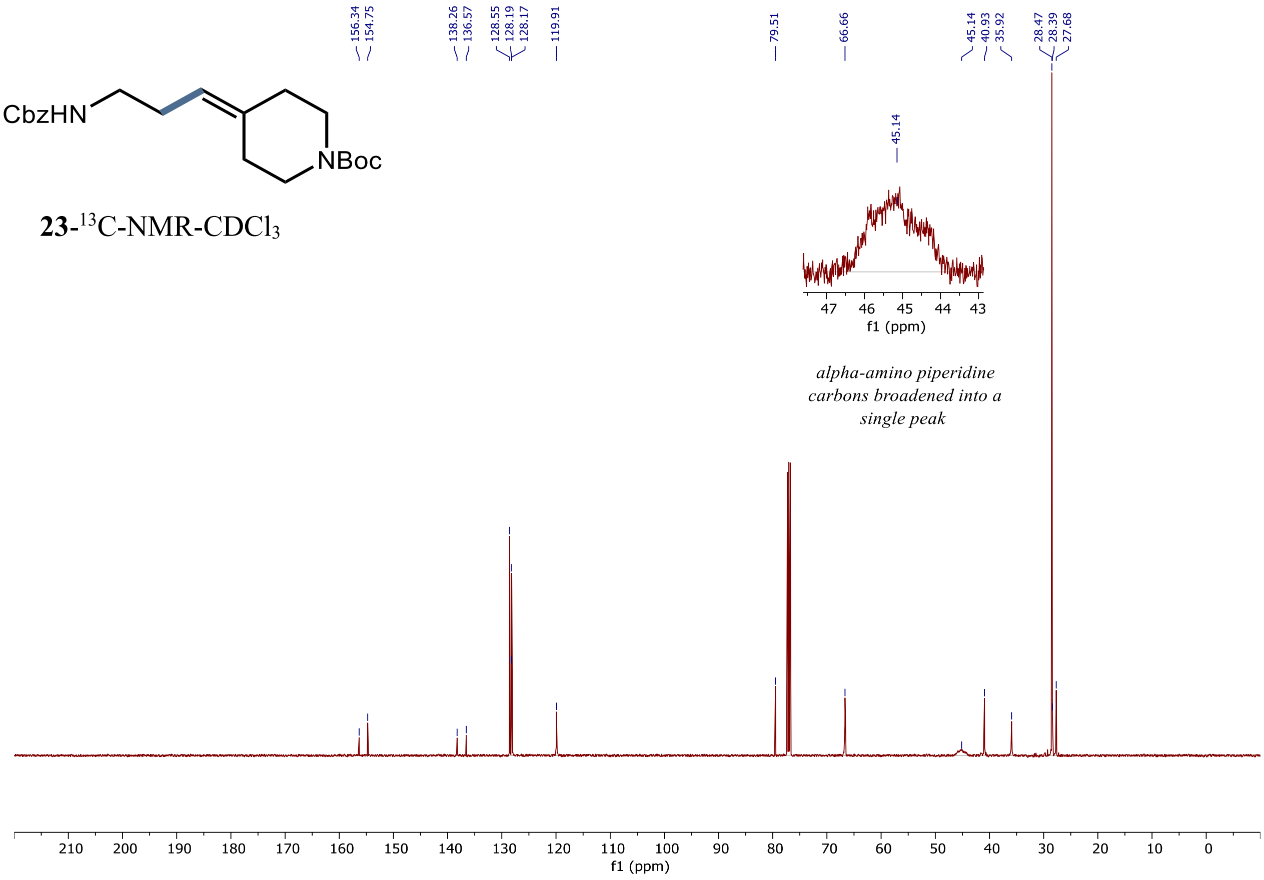

23-^{13}C-NMR-$CDCl_3$

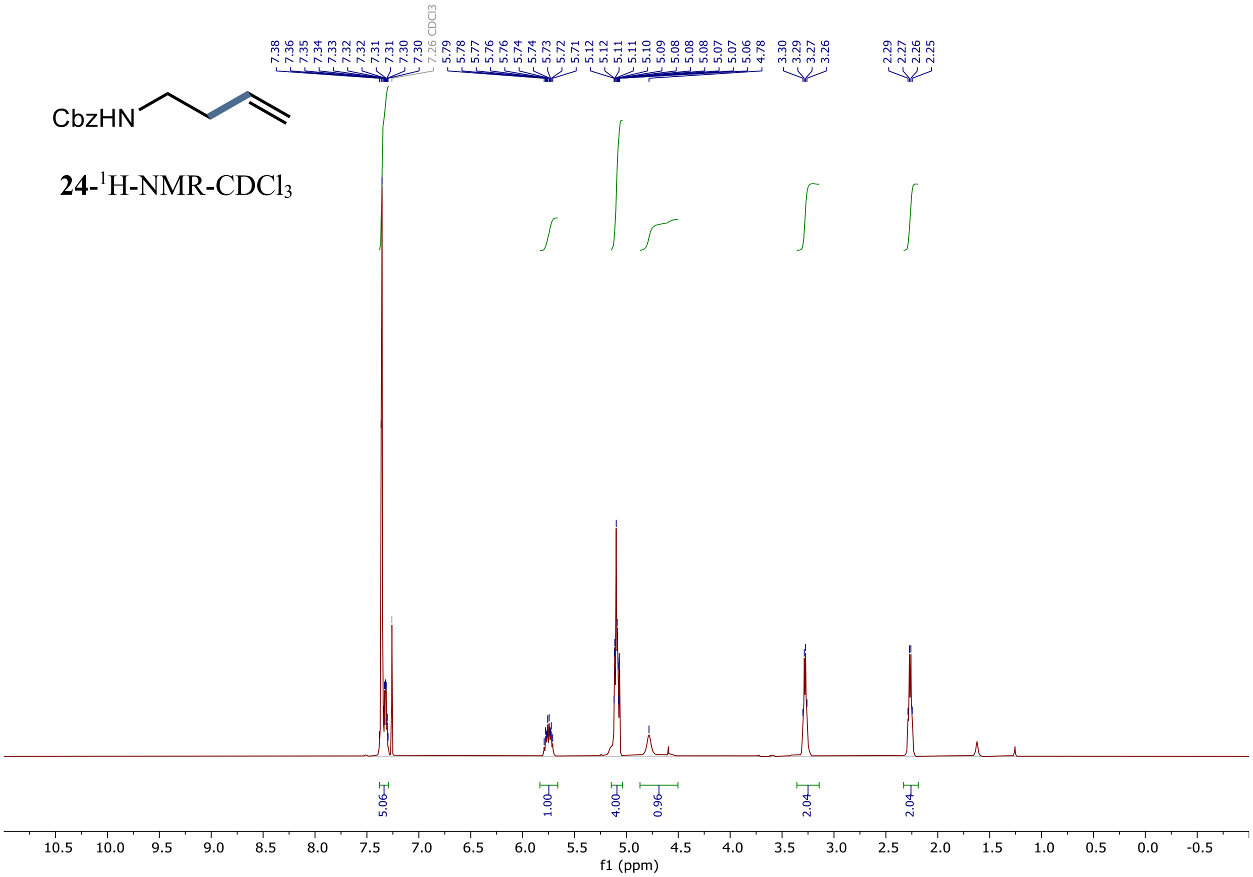

24-^{1}H-NMR-$CDCl_3$

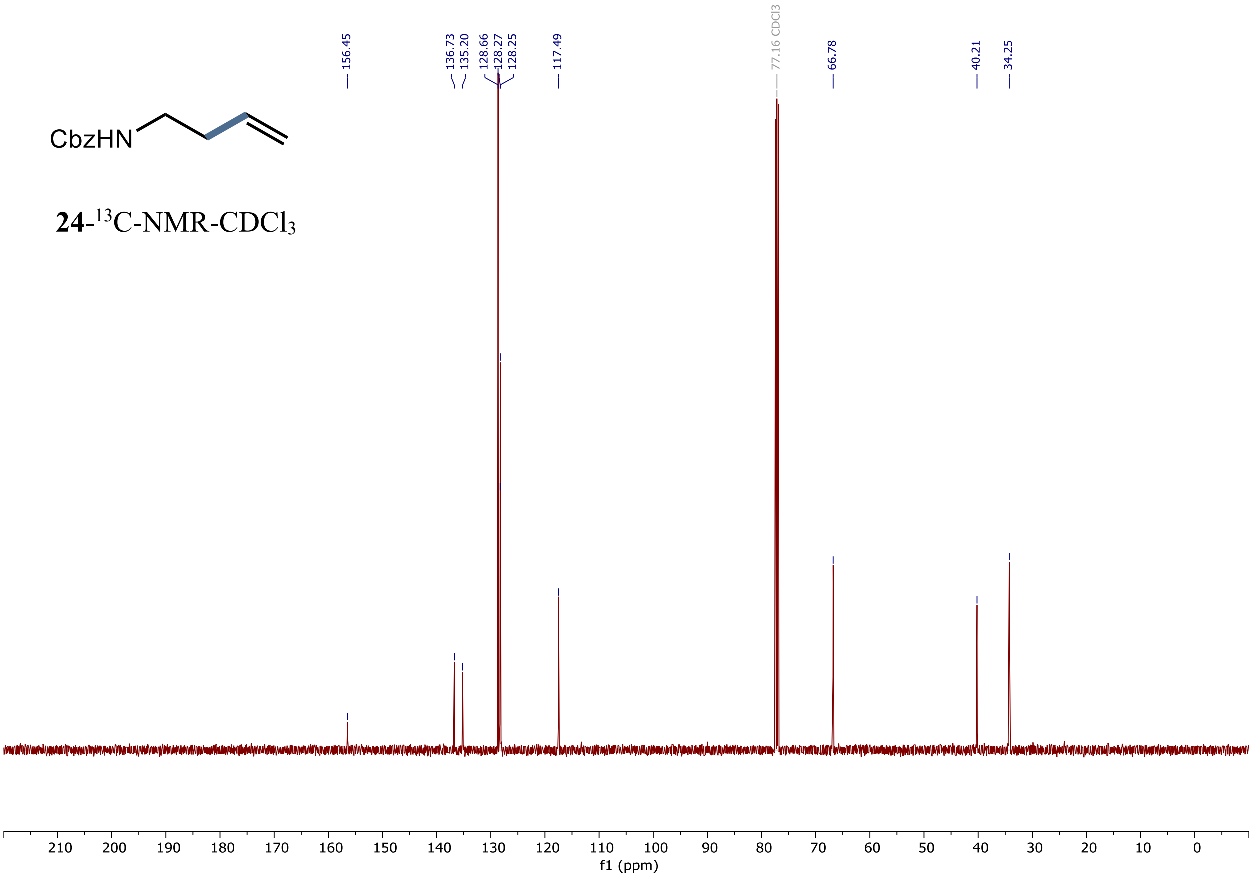
CbzHN
24-13C-NMR-CDCl3
156.45
136.73
135.20
128.66
128.27
128.25
117.49
77.16 CDCl3
66.78
40.21
34.25
210 200 190 180 170 160 150 140 130 120 110 100 90 80 70 60 50 40 30 20 10 0
f1 (ppm)

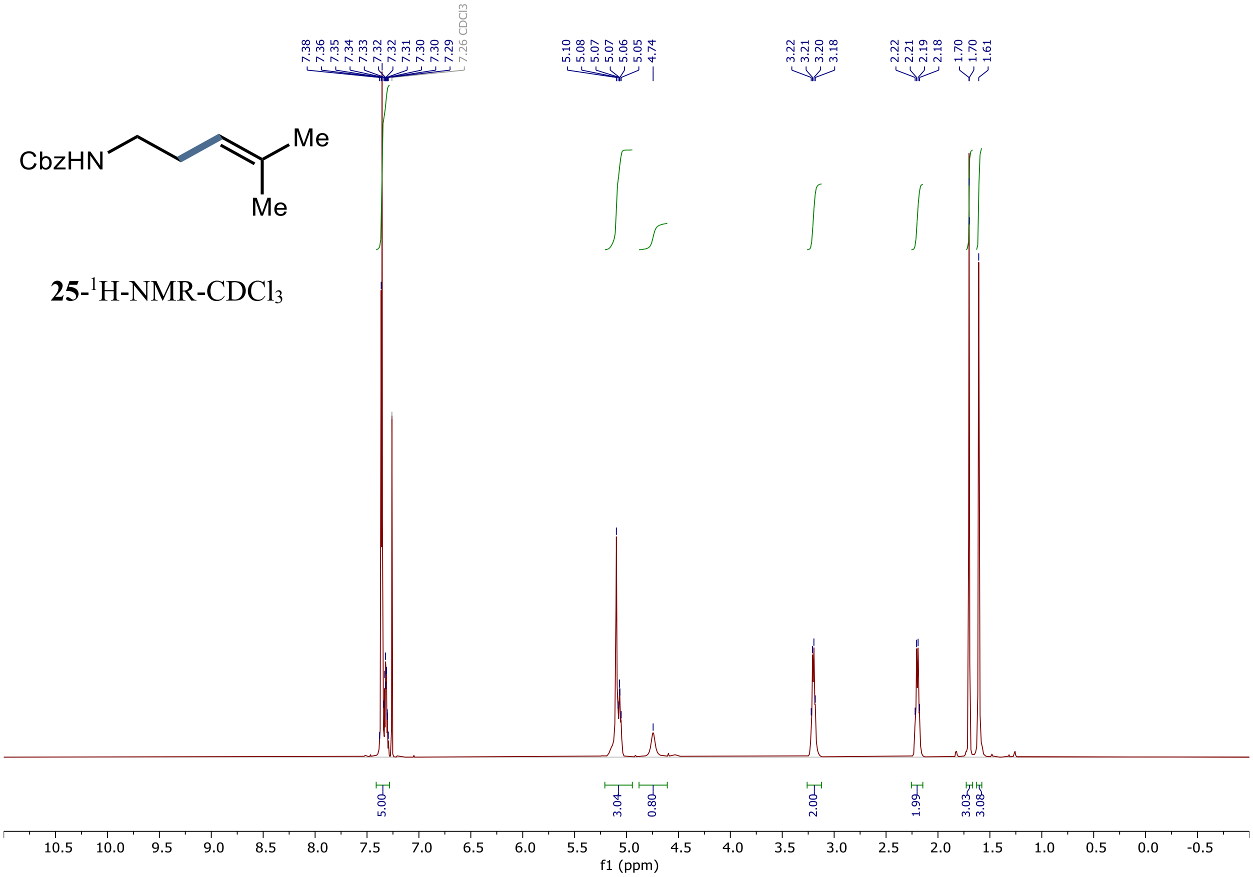
CbzHN
Me
Me
25-1H-NMR-CDCl3
7.38 7.36 7.35 7.34 7.33 7.32 7.32 7.31 7.30 7.30 7.29
7.26 CDCl3
5.10 5.08 5.07 5.07 5.06 5.05
4.74
3.22 3.21 3.20 3.18
2.22 2.21 2.19 2.18
1.70 1.70 1.61
5.00
3.04
0.80
2.00
1.99
3.03
3.08
10.5 10.0 9.5 9.0 8.5 8.0 7.5 7.0 6.5 6.0 5.5 5.0 4.5 4.0 3.5 3.0 2.5 2.0 1.5 1.0 0.5 0.0 -0.5
f1 (ppm)

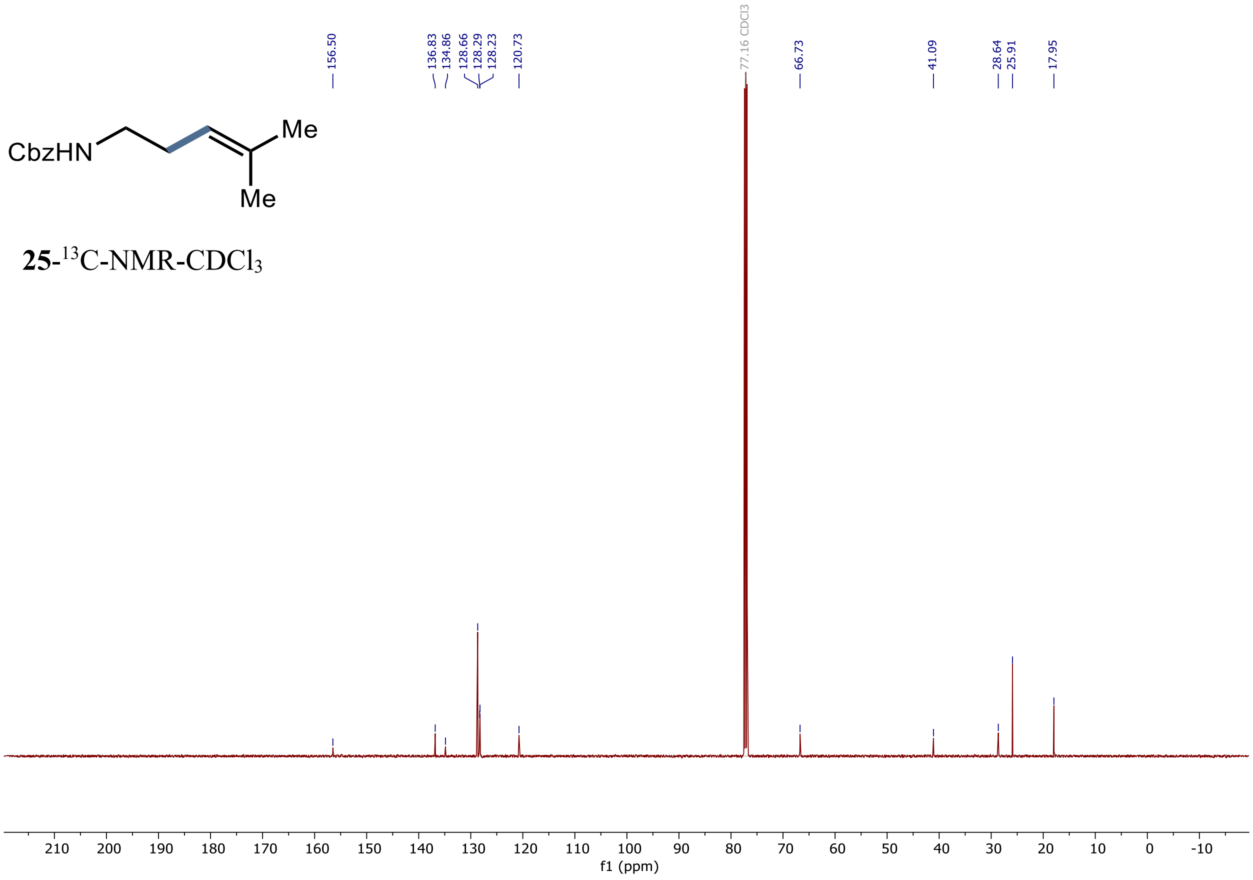

25-^{13}C-NMR-$CDCl_3$

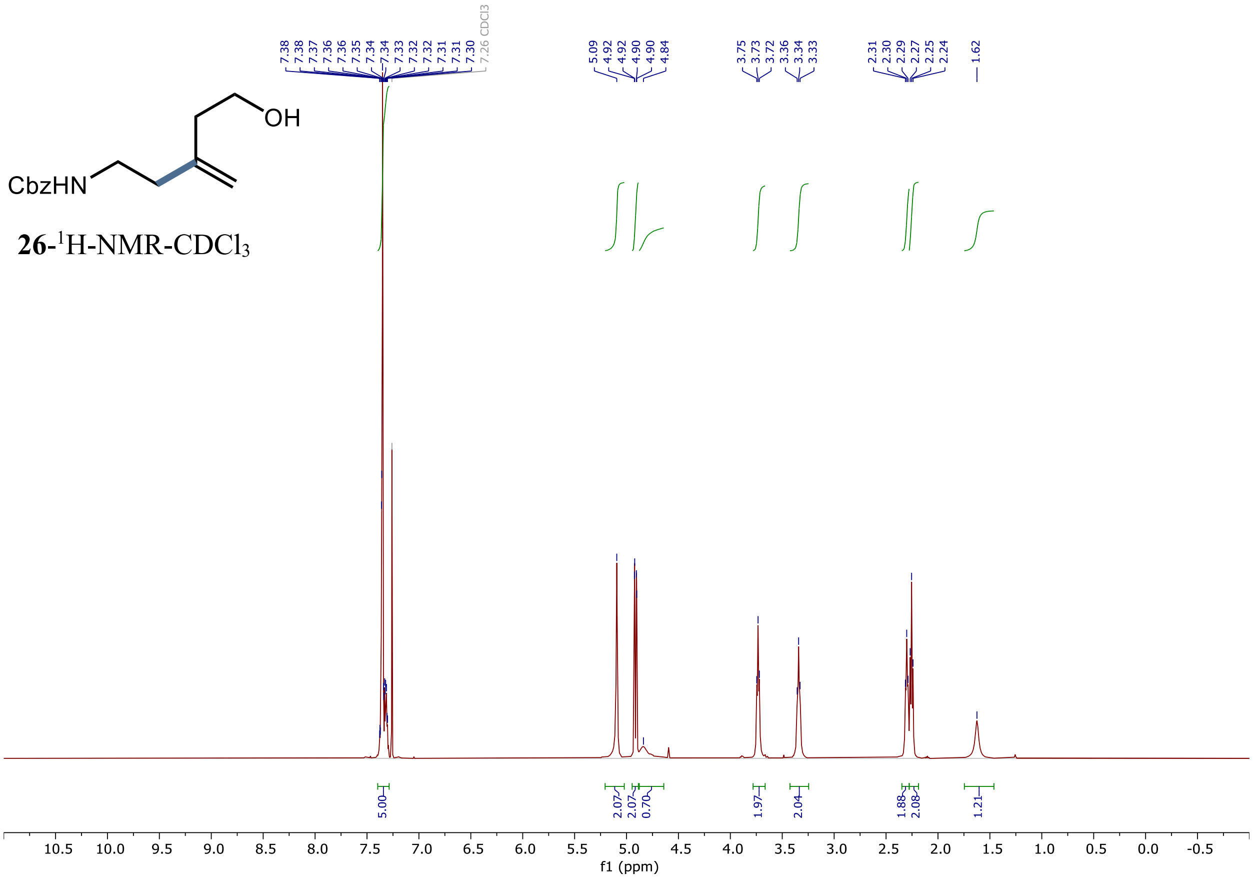

26-^{1}H-NMR-$CDCl_3$

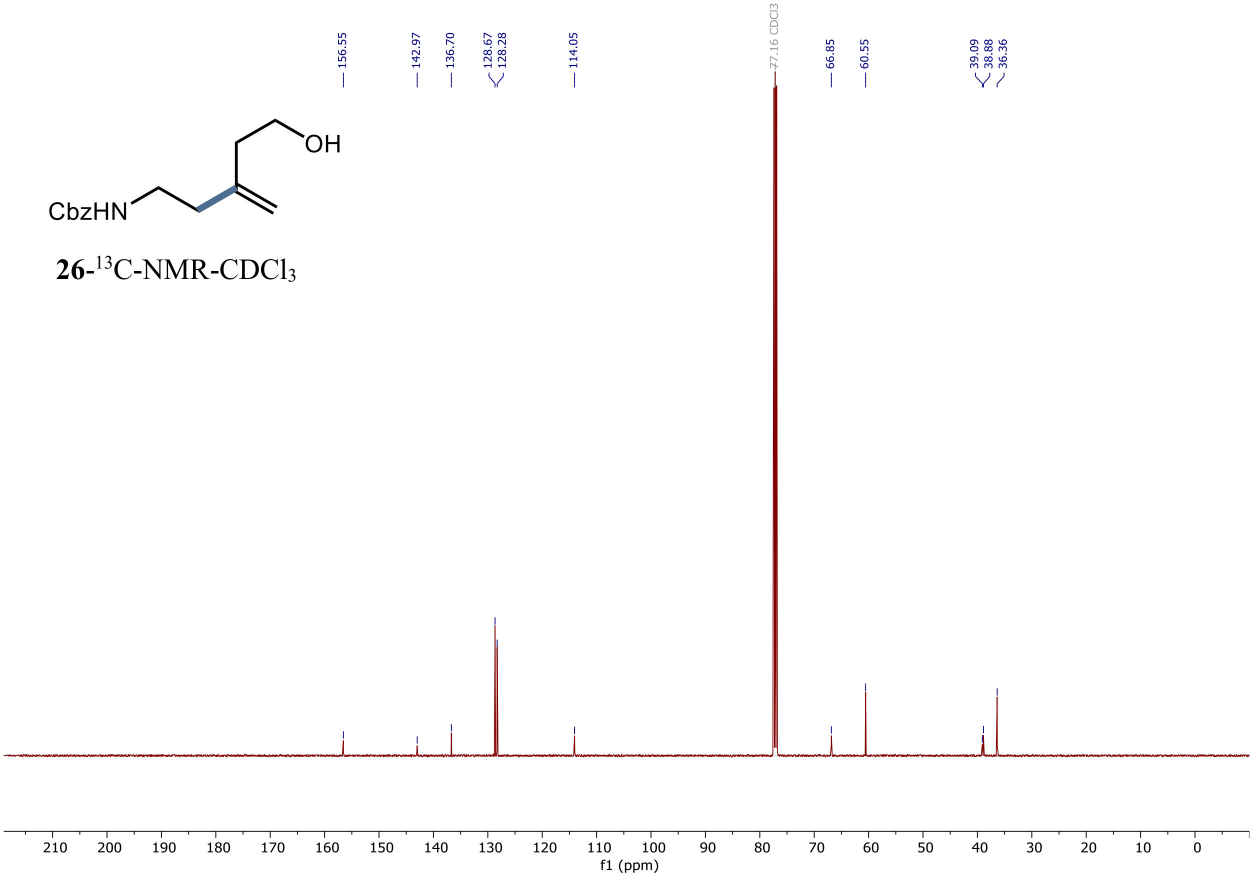

26-^{13}C-NMR-$CDCl_3$

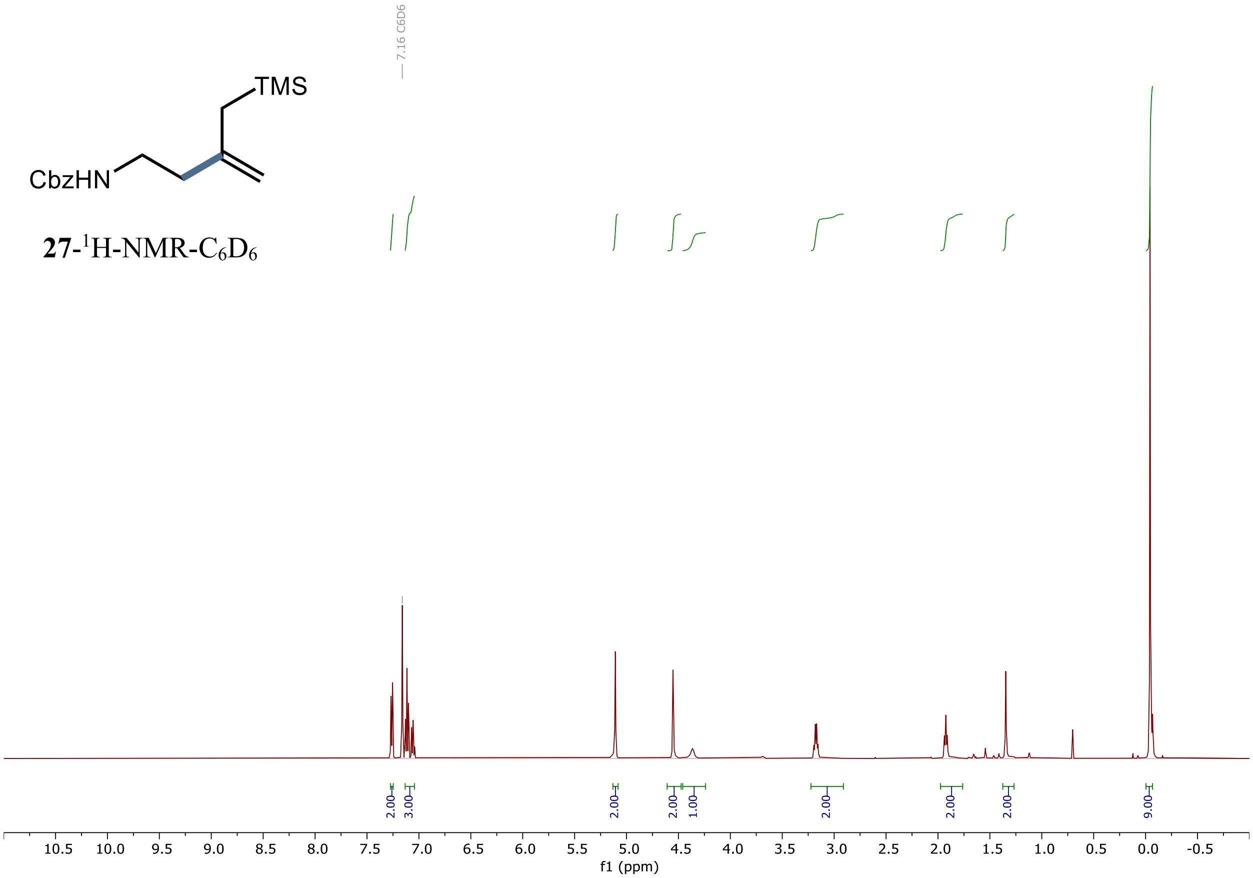

27-^{1}H-NMR-C_6D_6

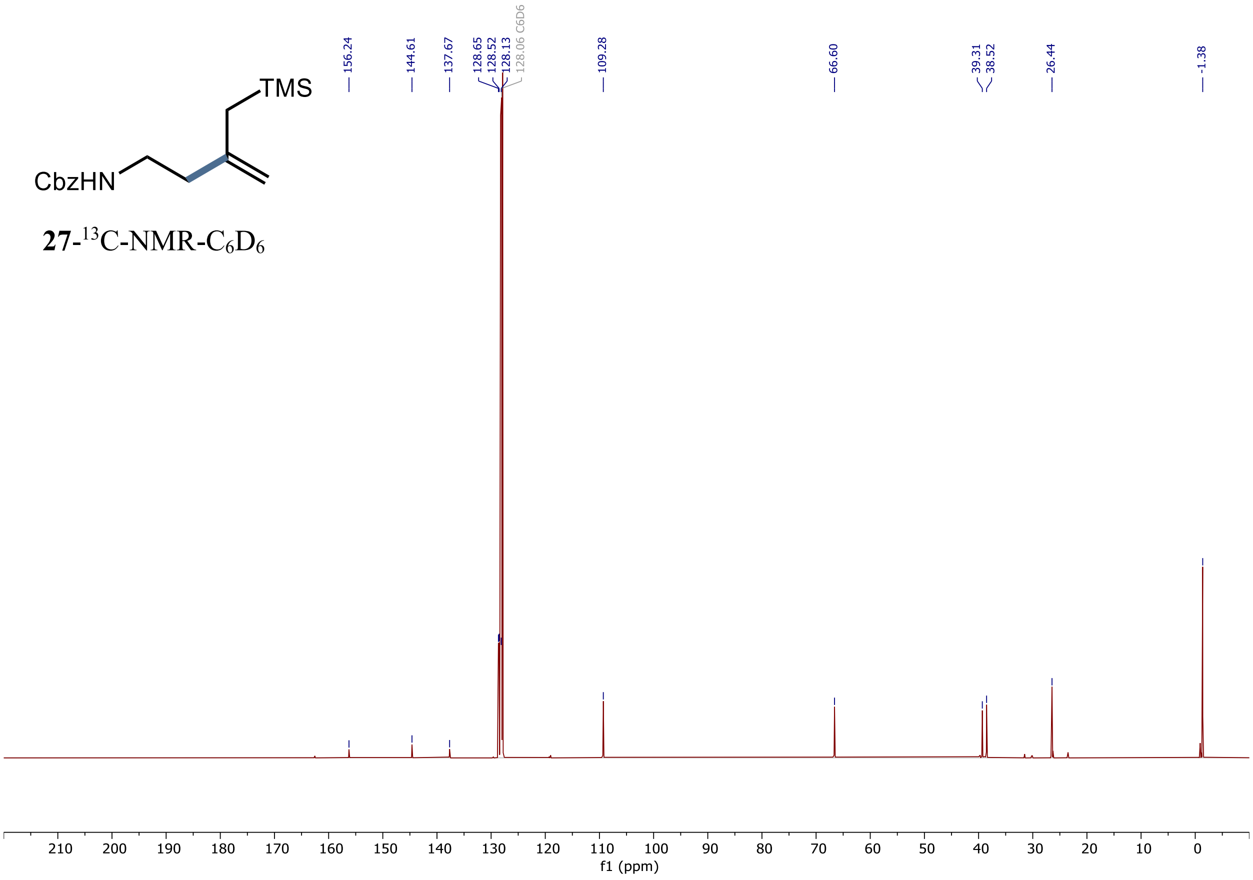
TMS
CbzHN
27-^{13}C-NMR-C_6D_6
f1 (ppm)

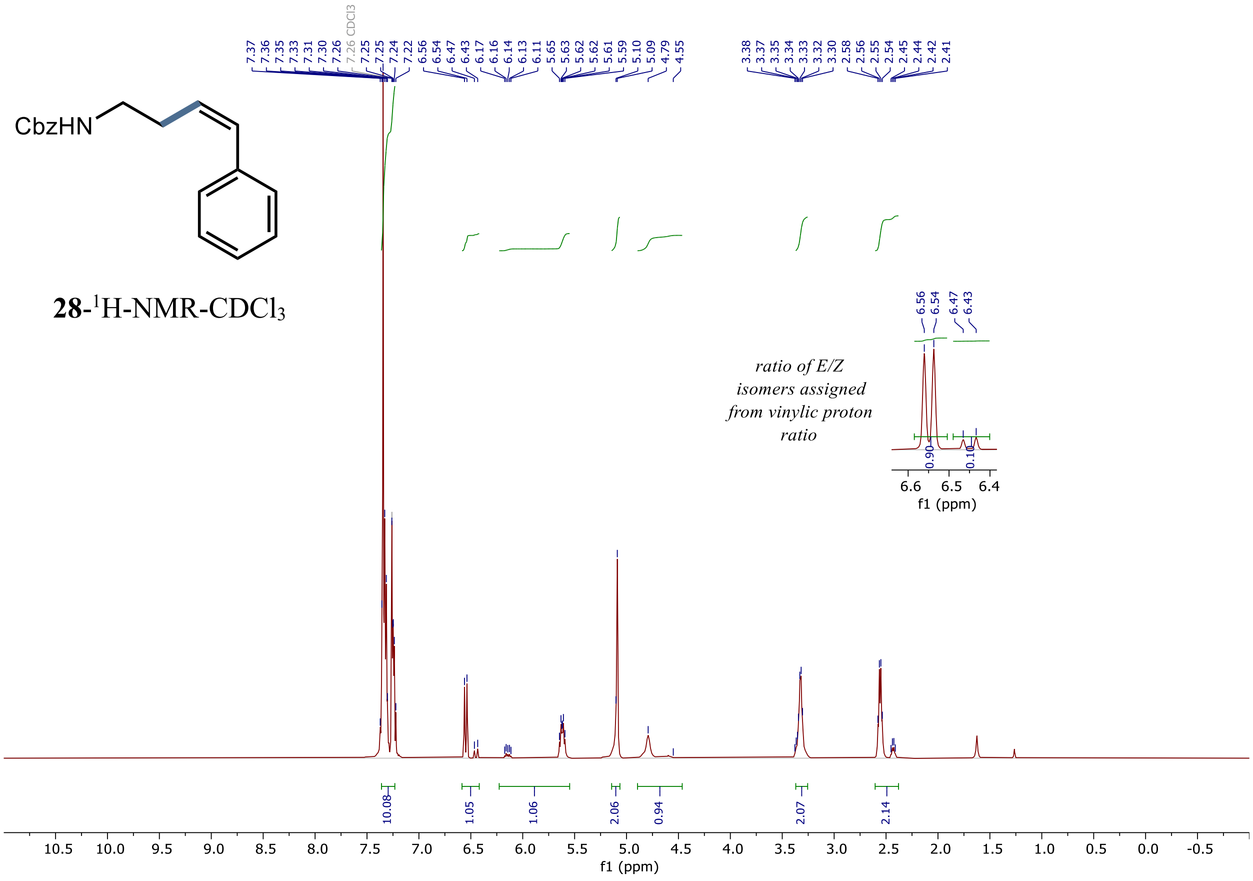
CbzHN
28-^{1}H-NMR-$CDCl_3$
ratio of E/Z
isomers assigned
from vinylic proton
ratio
f1 (ppm)

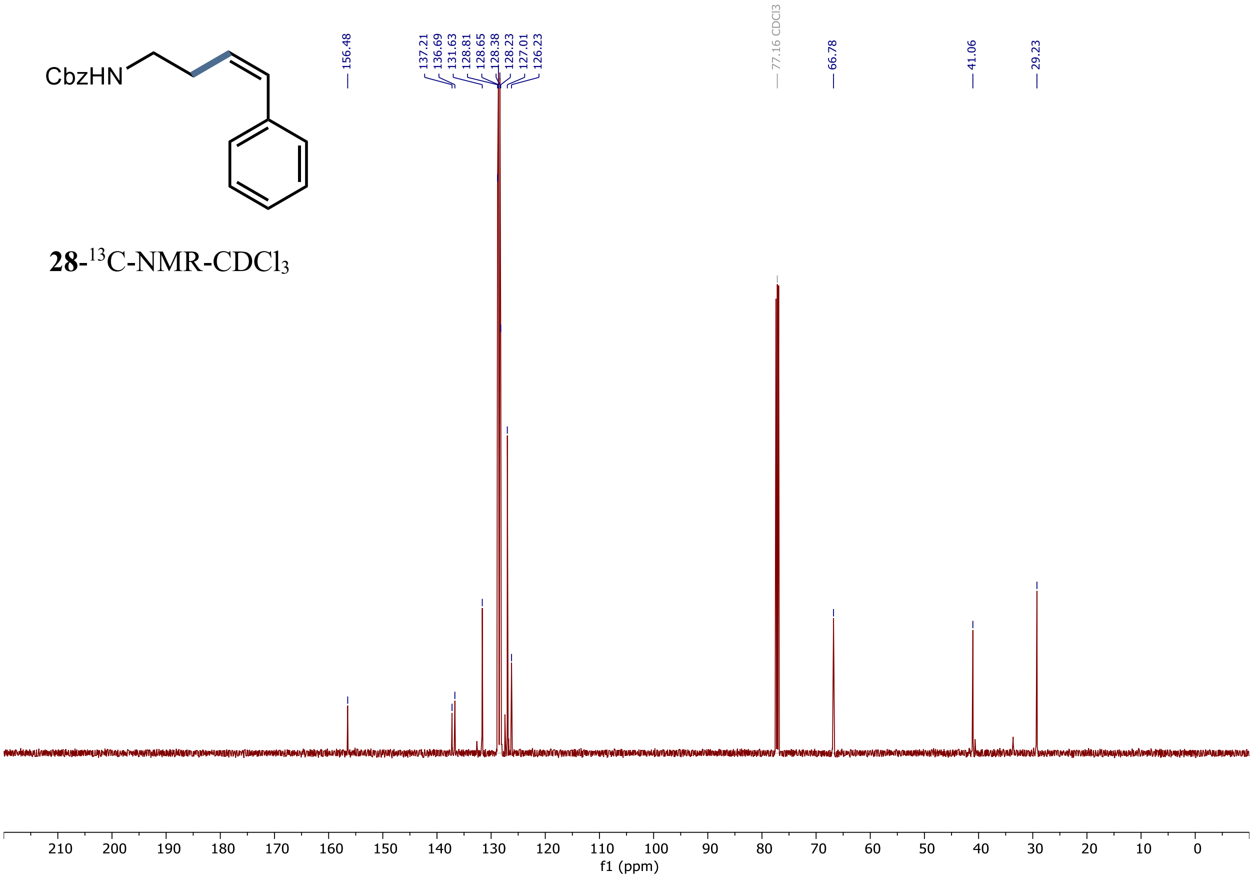
CbzHN
28-13C-NMR-CDCl3
156.48
137.21
136.69
131.63
128.81
128.65
128.38
128.23
127.01
126.23
77.16 CDCl3
66.78
41.06
29.23
f1 (ppm)

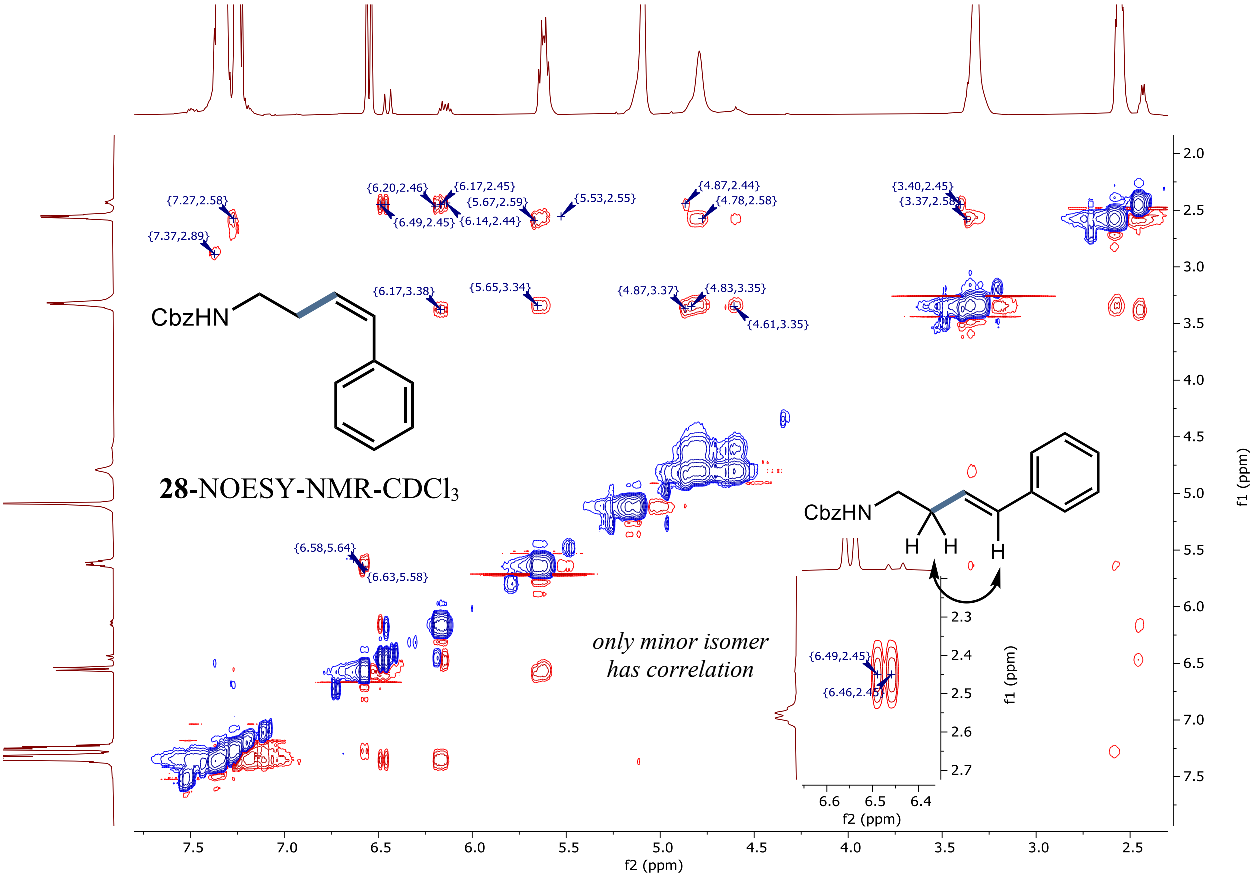
CbzHN
28-NOESY-NMR-CDCl3
only minor isomer
has correlation
H
f2 (ppm)
f1 (ppm)

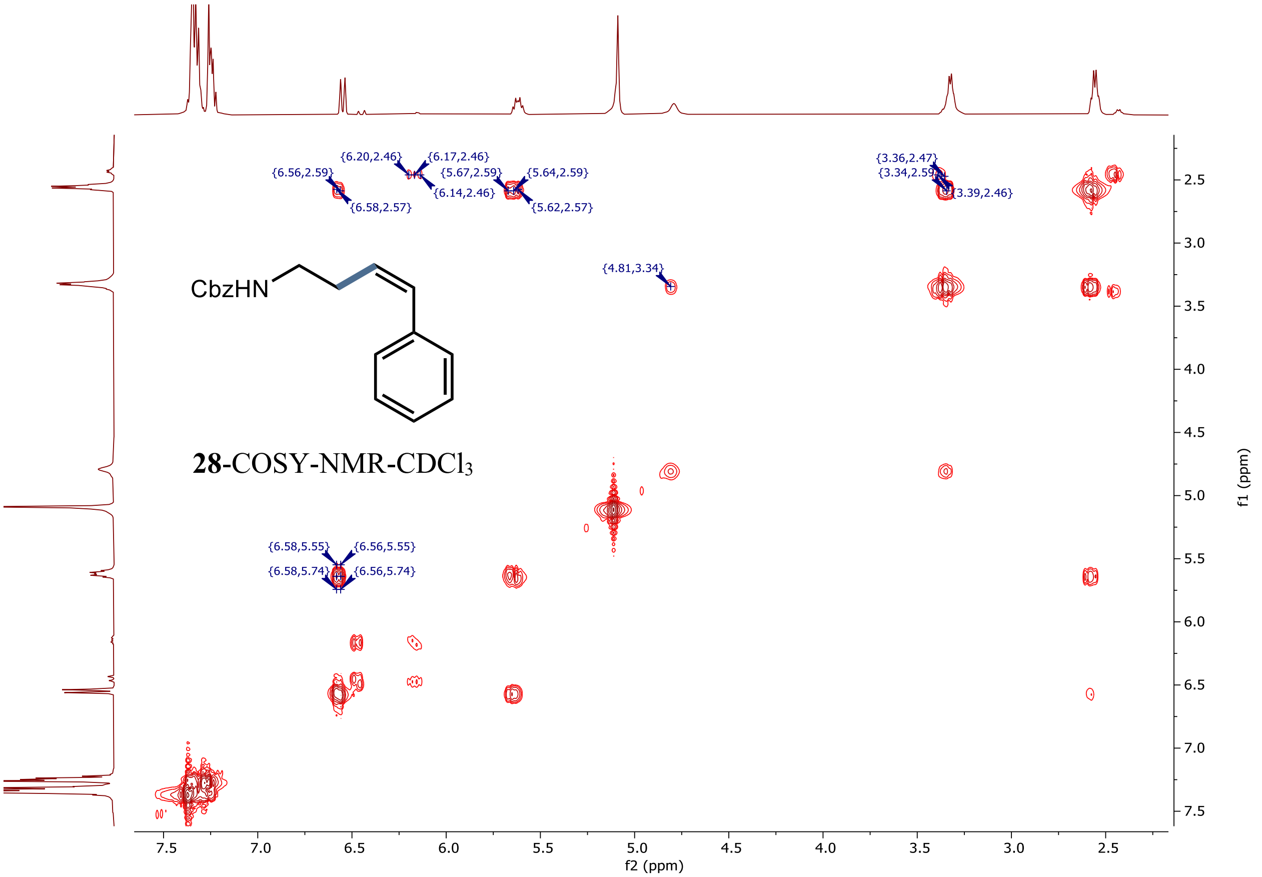
CbzHN
28-COSY-NMR-CDCl3
f2 (ppm)
f1 (ppm)

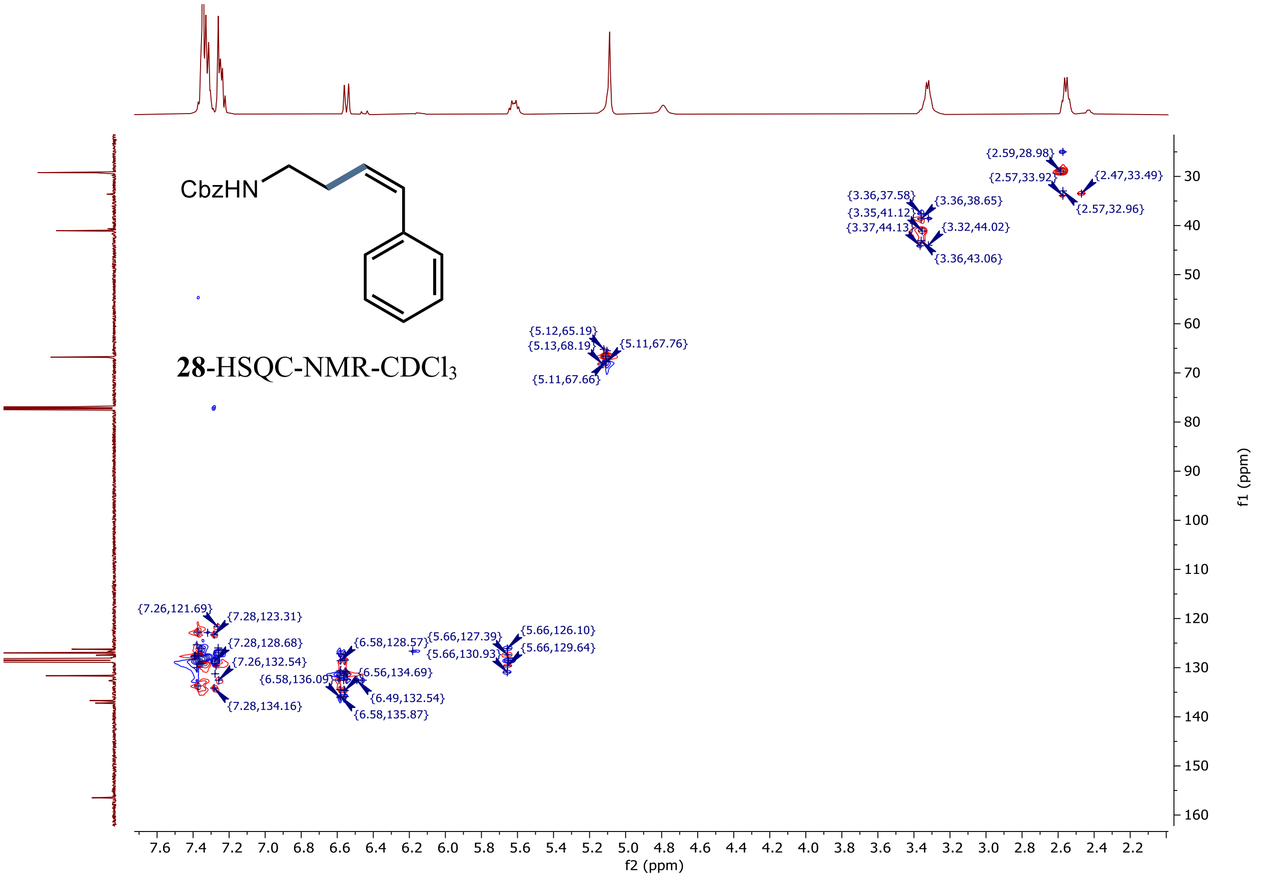
CbzHN
28-HSQC-NMR-CDCl3
f2 (ppm)
f1 (ppm)

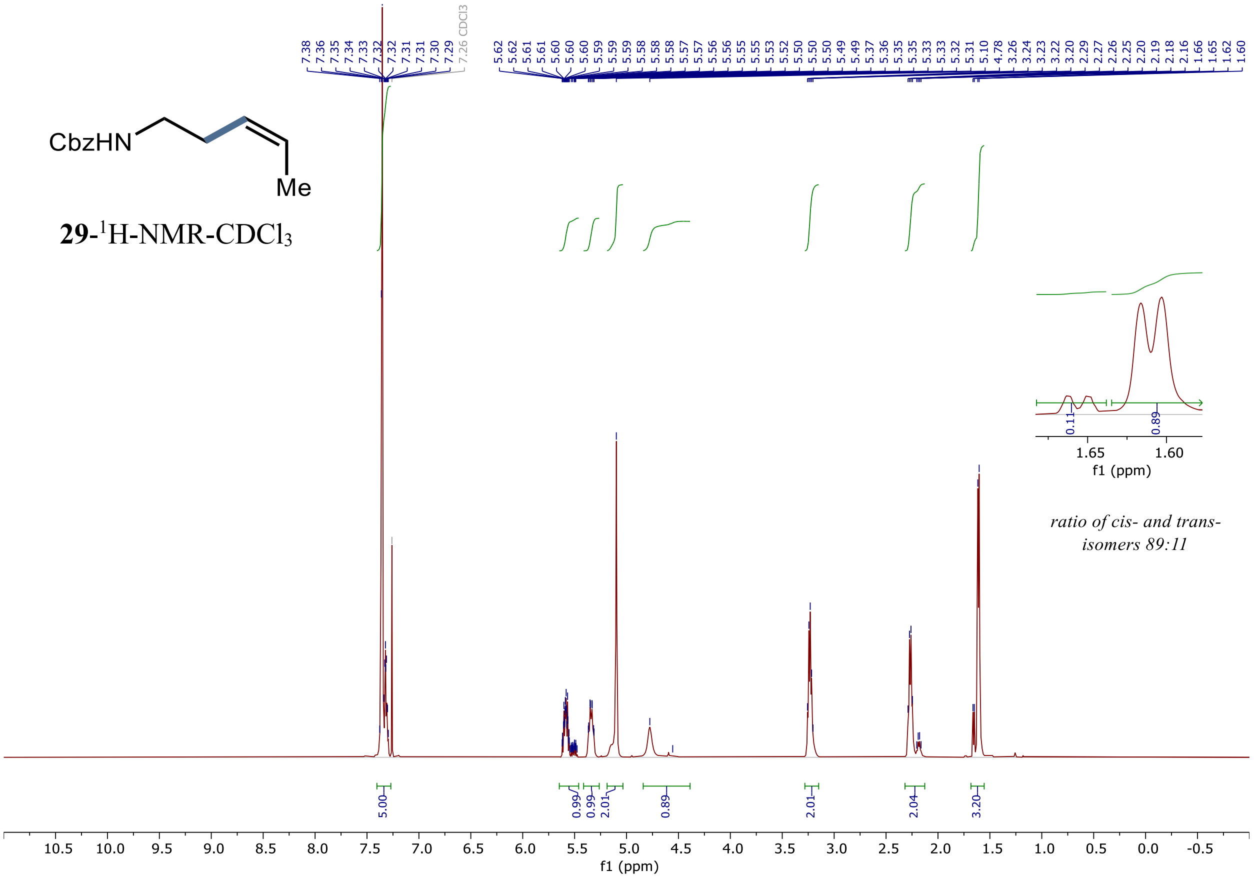
CbzHN
Me
29-¹H-NMR-CDCl₃
ratio of cis- and trans-isomers 89:11
f1 (ppm)

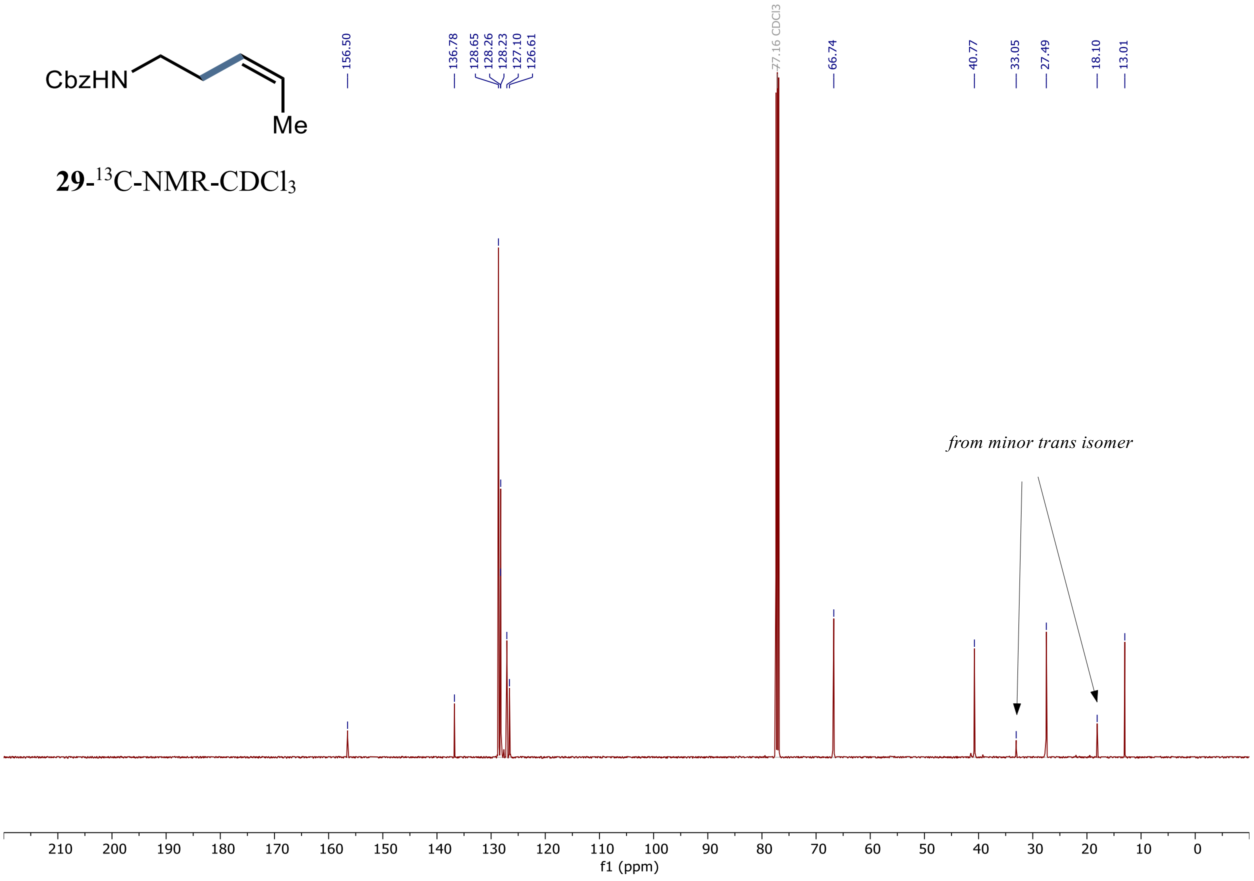
CbzHN
Me
29-¹³C-NMR-CDCl₃
156.50
136.78
128.65
128.26
128.23
127.10
126.61
77.16 CDCl3
66.74
40.77
33.05
27.49
18.10
13.01
from minor trans isomer
f1 (ppm)

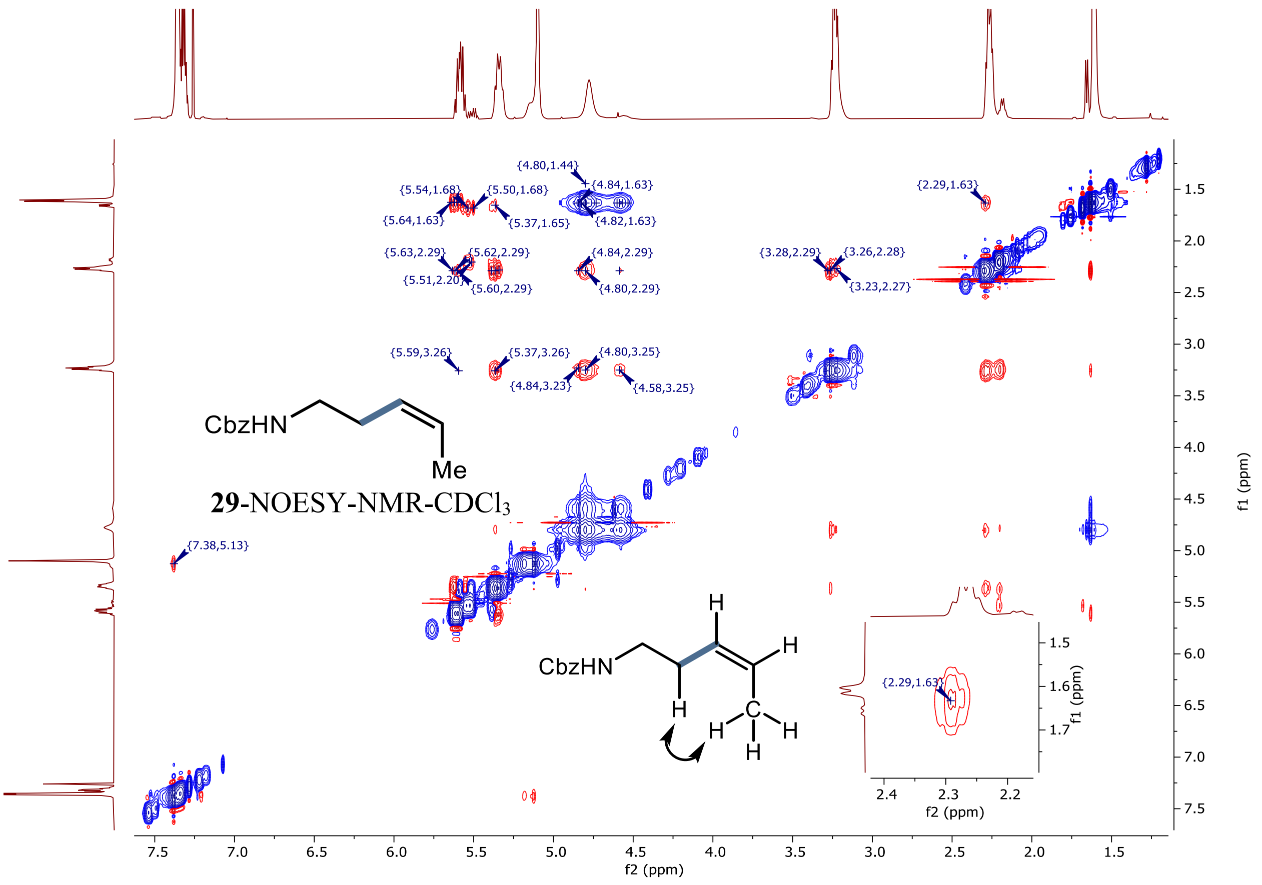
CbzHN
Me
29-NOESY-NMR-CDCl3
CbzHN
f2 (ppm)
f1 (ppm)

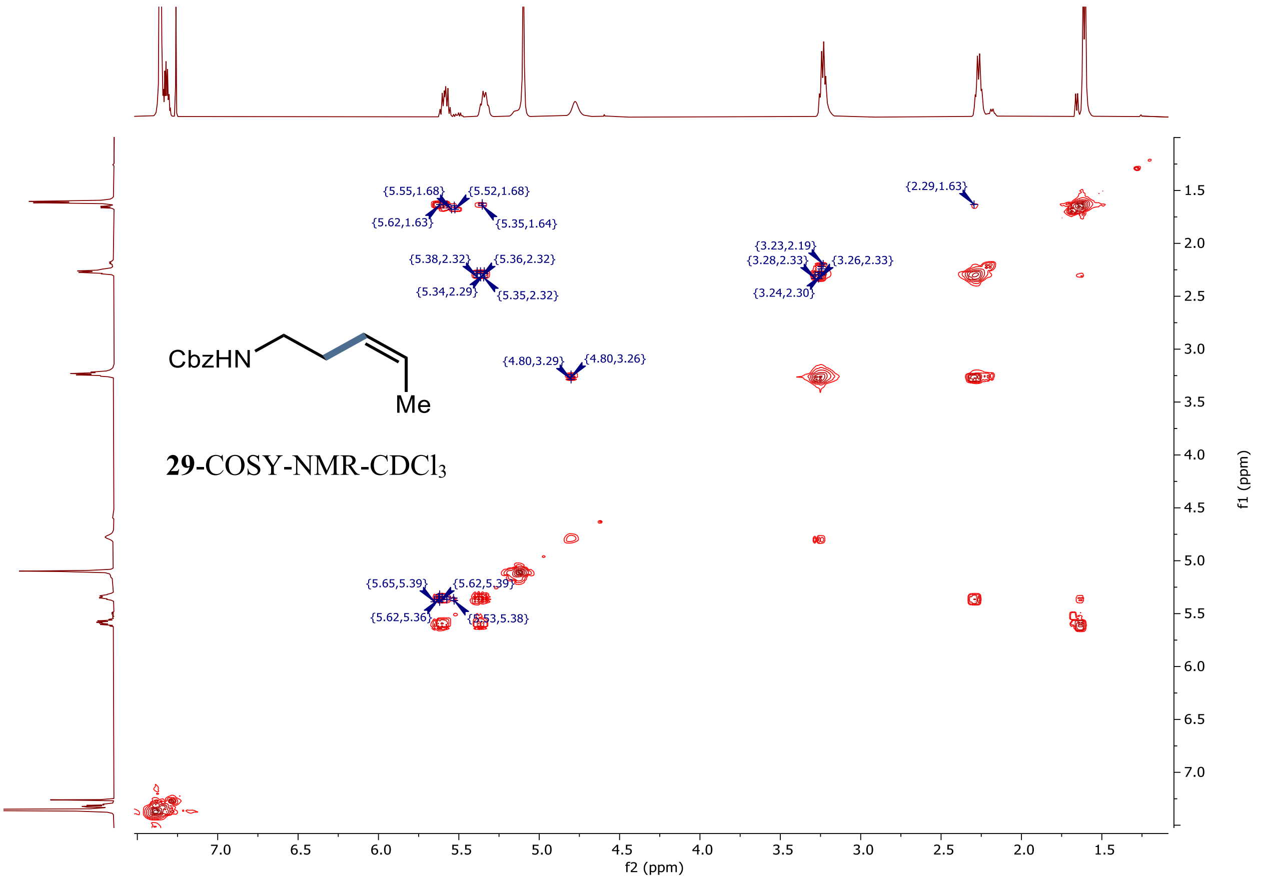
CbzHN
Me
29-COSY-NMR-CDCl3
f2 (ppm)
f1 (ppm)

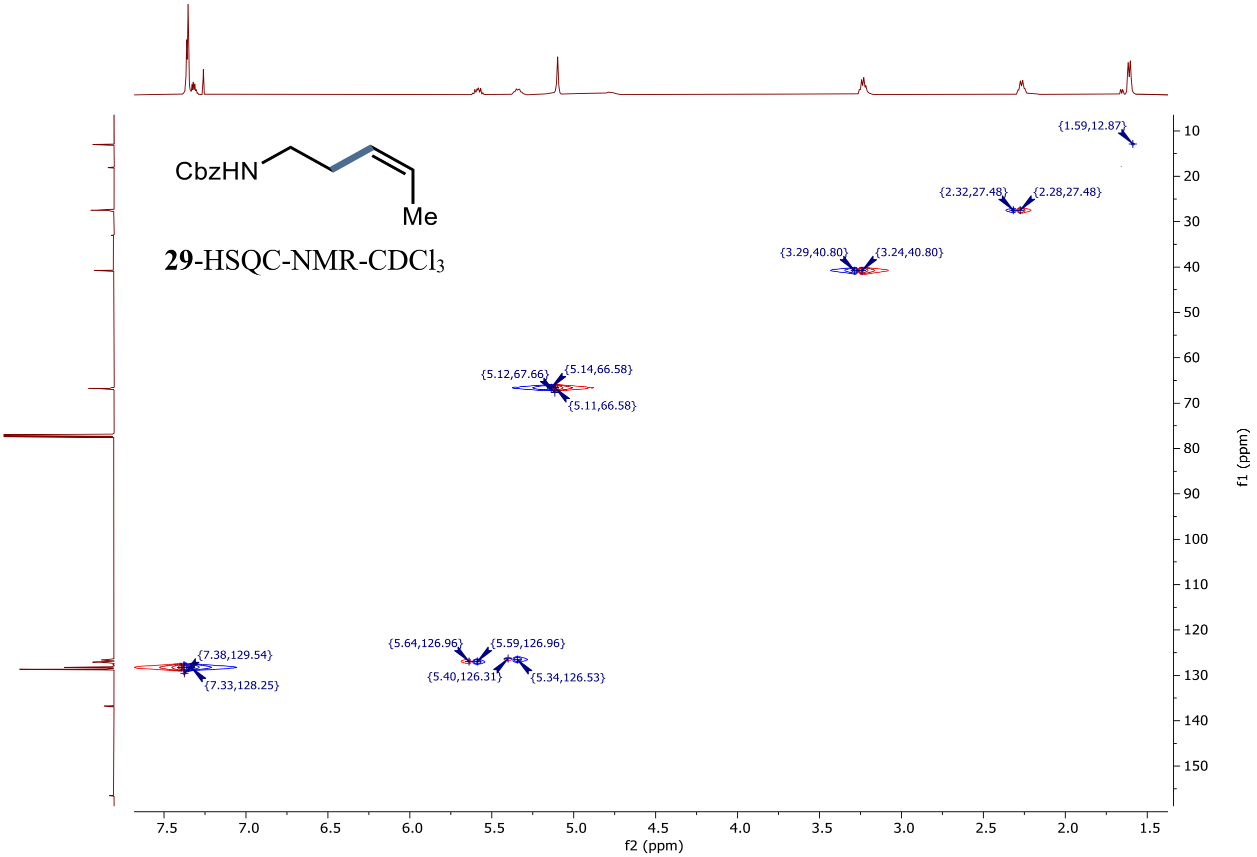
CbzHN
Me
29-HSQC-NMR-CDCl3
{1.59,12.87}
{2.32,27.48}
{2.28,27.48}
{3.29,40.80}
{3.24,40.80}
{5.12,67.66}
{5.14,66.58}
{5.11,66.58}
{7.38,129.54}
{7.33,128.25}
{5.64,126.96}
{5.59,126.96}
{5.40,126.31}
{5.34,126.53}
f2 (ppm)
f1 (ppm)

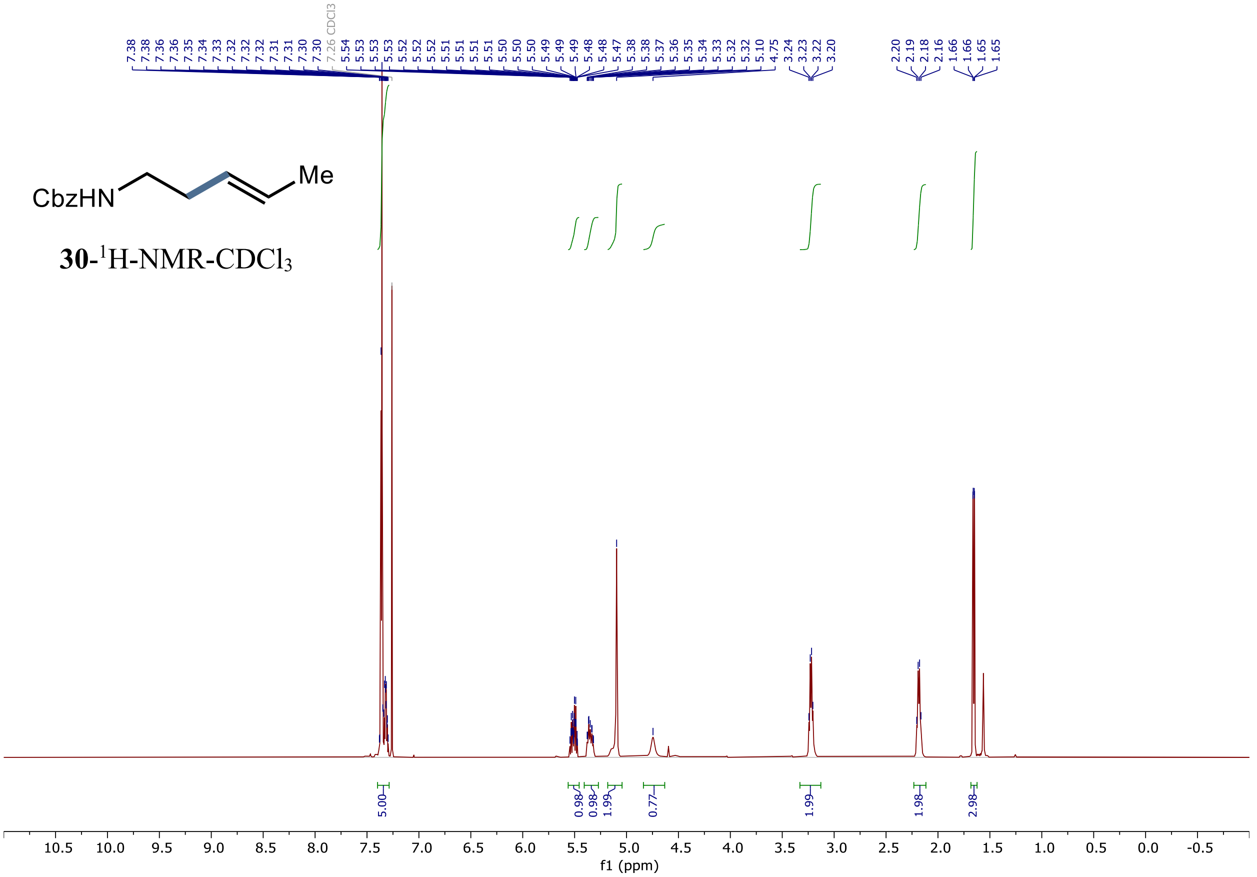
CbzHN
Me
30-1H-NMR-CDCl3
7.26 CDCl3
5.00
0.98
0.98
1.99
0.77
1.99
1.98
2.98
f1 (ppm)

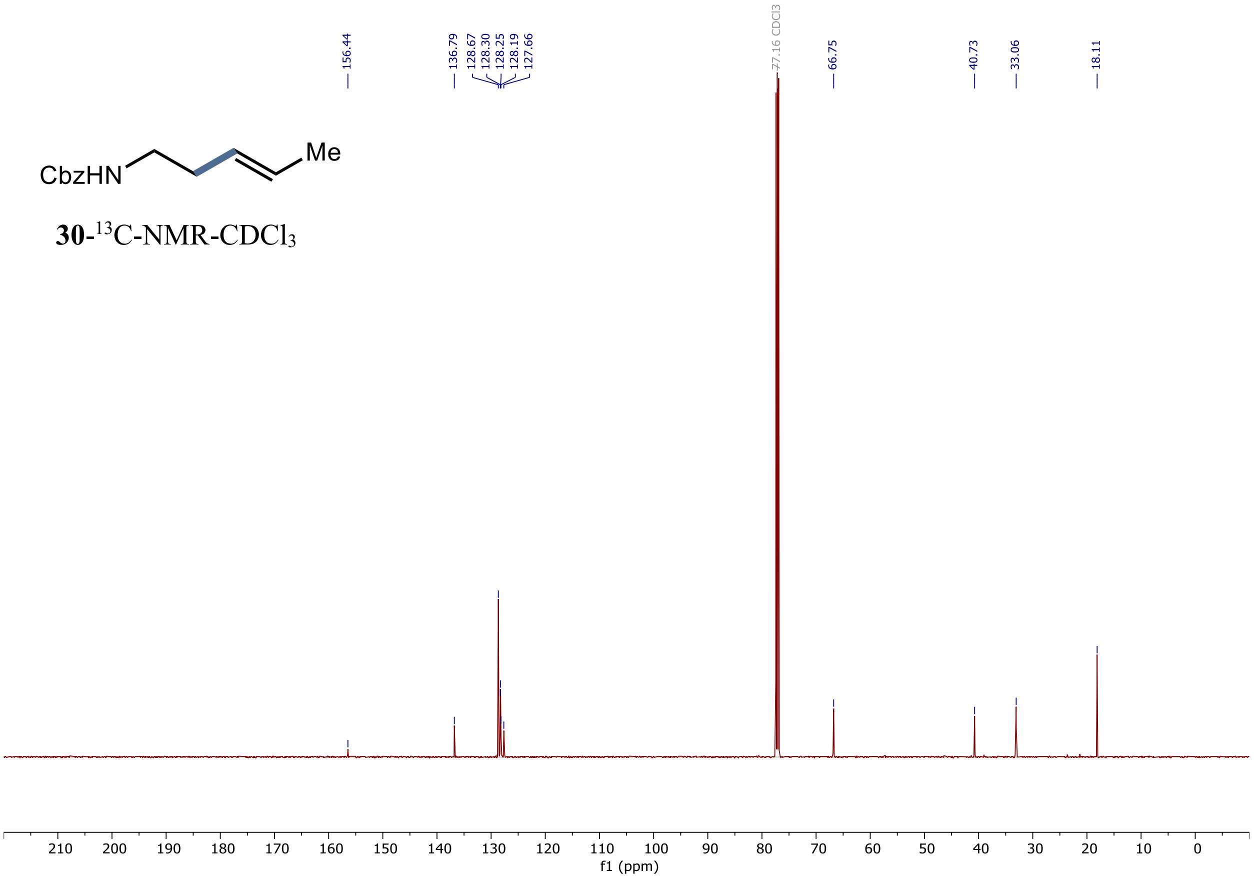

156.44
136.79
128.67
128.30
128.25
128.19
127.66
77.16 CDCl3
66.75
40.73
33.06
18.11
CbzHN
Me
30-13C-NMR-CDCl3
f1 (ppm)

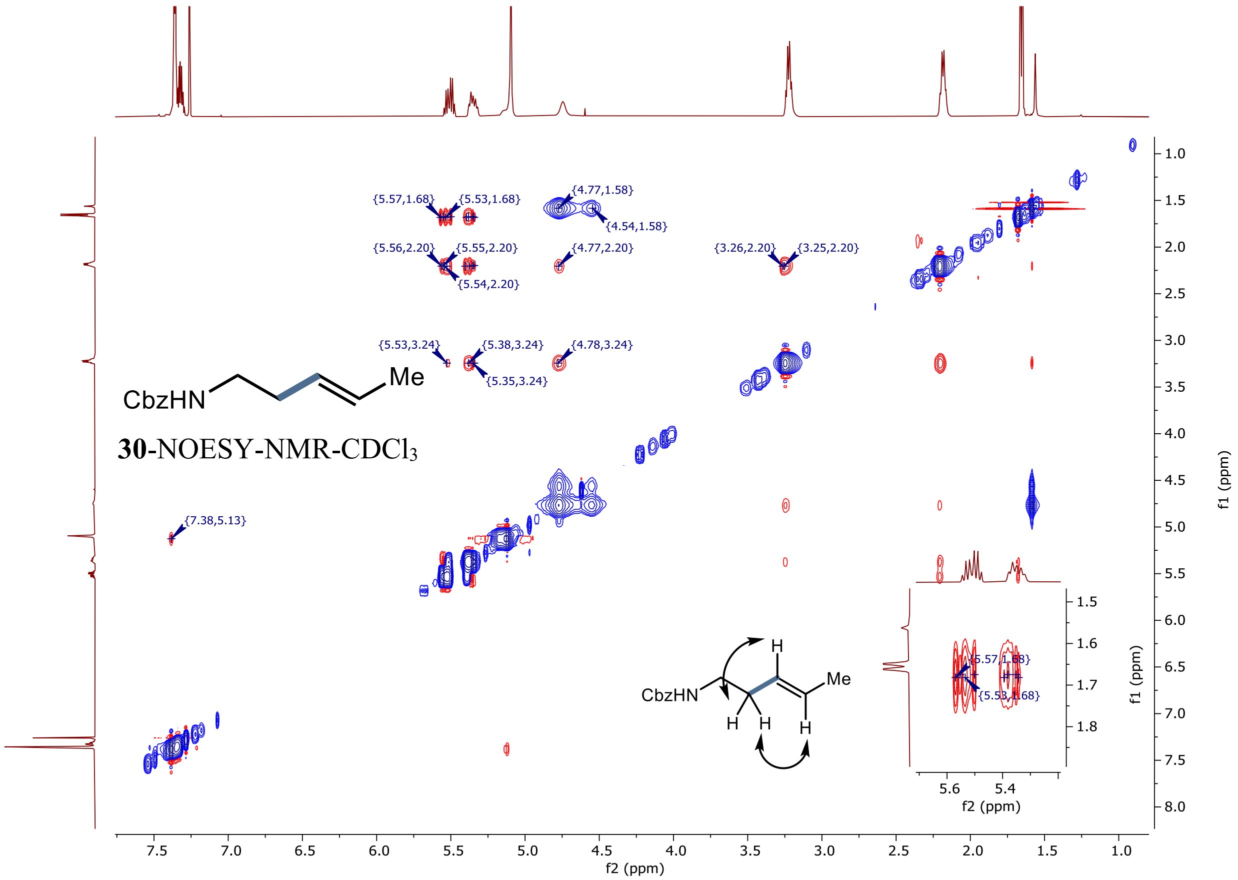

CbzHN
Me
30-NOESY-NMR-CDCl3
{5.57,1.68}
{5.53,1.68}
{4.77,1.58}
{4.54,1.58}
{5.56,2.20}
{5.55,2.20}
{5.54,2.20}
{4.77,2.20}
{3.26,2.20}
{3.25,2.20}
{5.53,3.24}
{5.38,3.24}
{5.35,3.24}
{4.78,3.24}
{7.38,5.13}
H
H
H
H
f2 (ppm)
f1 (ppm)

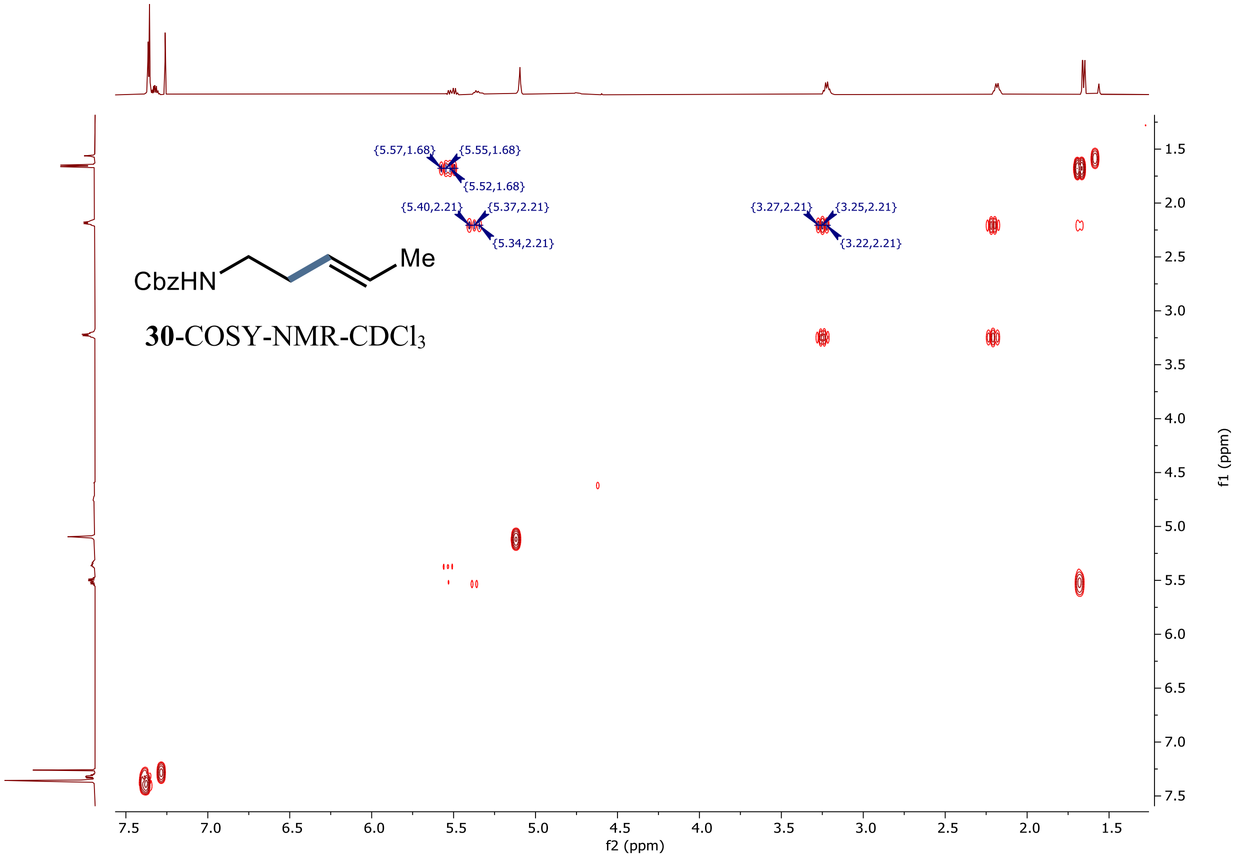

CbzHN
Me
30-COSY-NMR-CDCl3
{5.57,1.68}
{5.55,1.68}
{5.52,1.68}
{5.40,2.21}
{5.37,2.21}
{5.34,2.21}
{3.27,2.21}
{3.25,2.21}
{3.22,2.21}
f2 (ppm)
f1 (ppm)

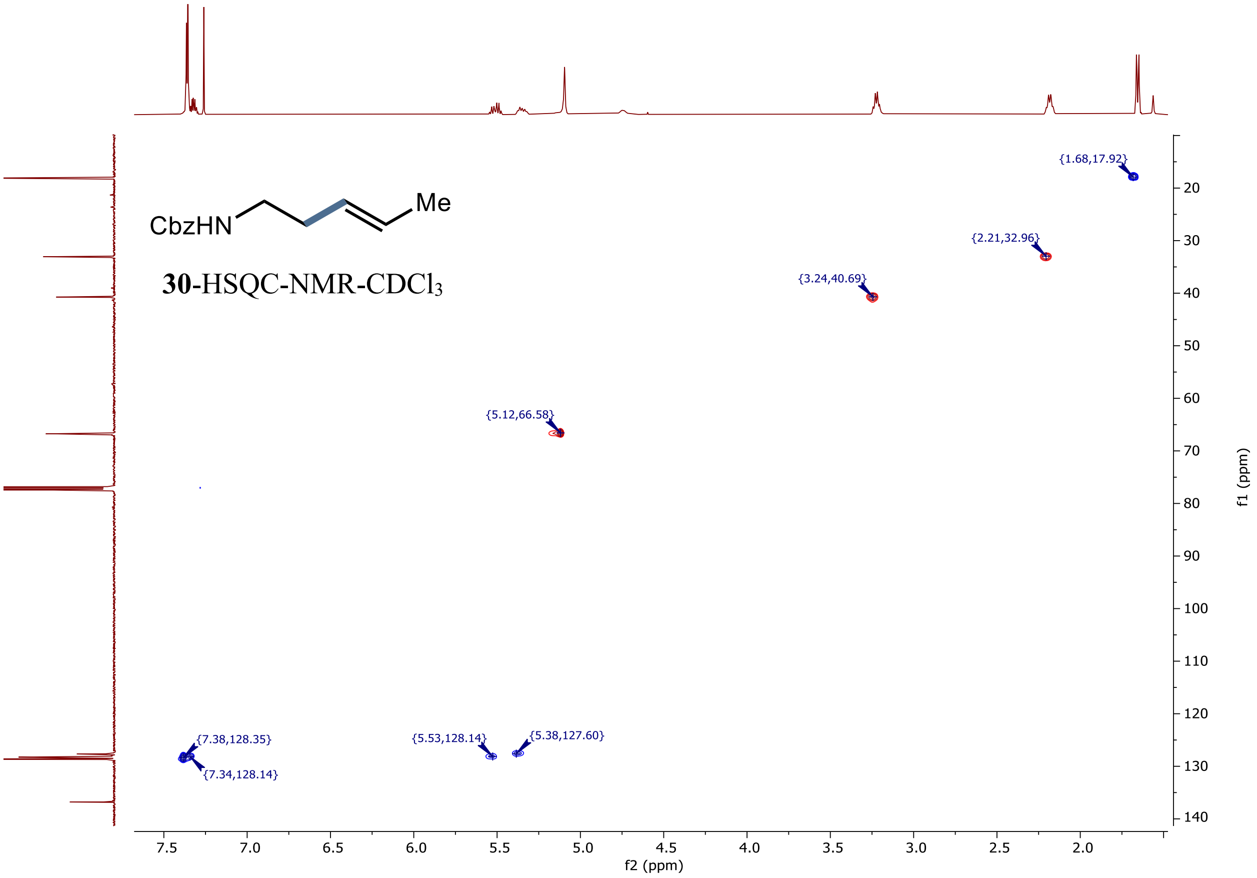

CbzHN
Me
30-HSQC-NMR-CDCl3
{1.68,17.92}
{2.21,32.96}
{3.24,40.69}
{5.12,66.58}
{7.38,128.35}
{7.34,128.14}
{5.53,128.14}
{5.38,127.60}
f2 (ppm)
f1 (ppm)

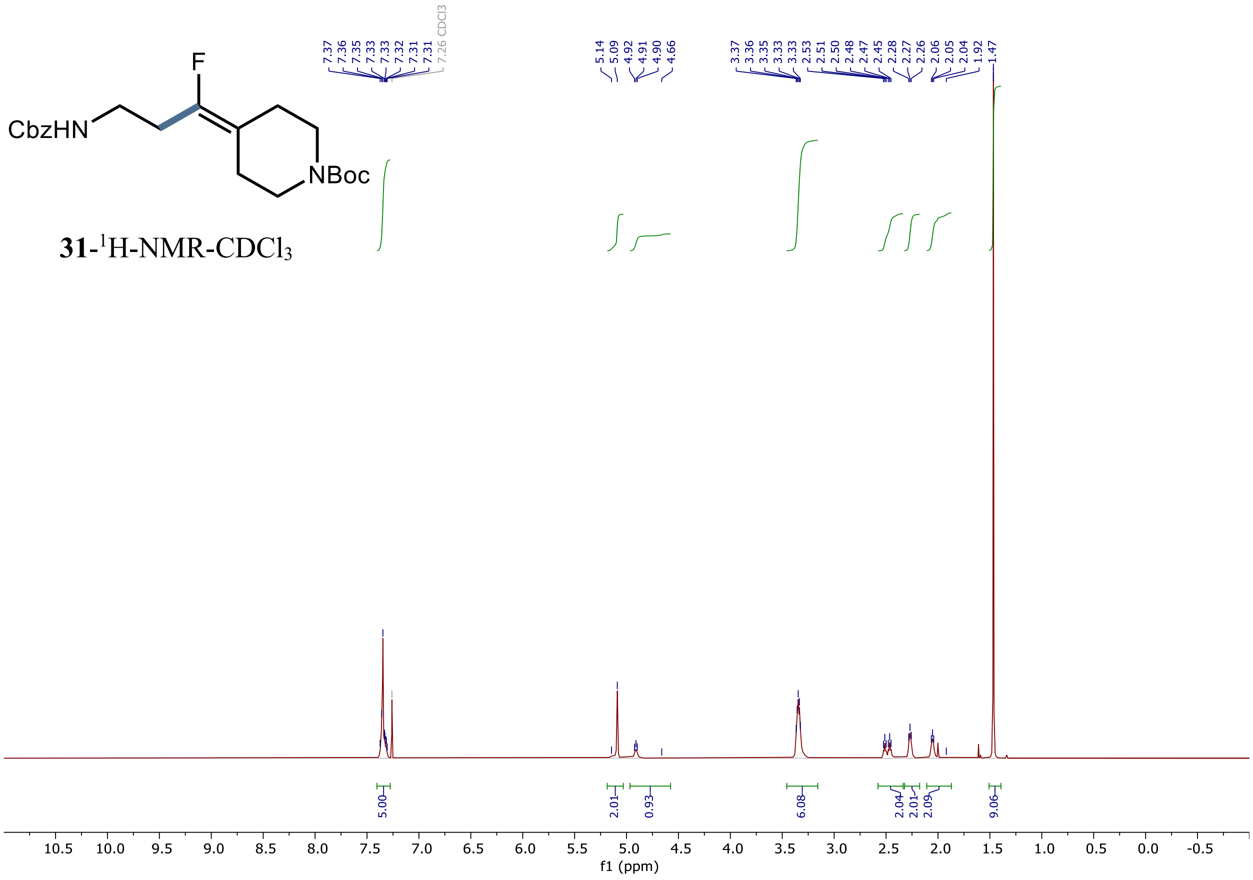
CbzHN
F
NBoc
31-¹H-NMR-CDCl3
7.37 7.36 7.35 7.33 7.33 7.32 7.31 7.31 7.26 CDCl3
5.14 5.09 4.92 4.91 4.90 4.66
3.37 3.36 3.35 3.33 3.33 2.53 2.51 2.50 2.48 2.47 2.45 2.28 2.27 2.26 2.06 2.05 2.04 1.92 1.47
5.00 2.01 0.93 6.08 2.04 2.01 2.09 9.06
f1 (ppm)

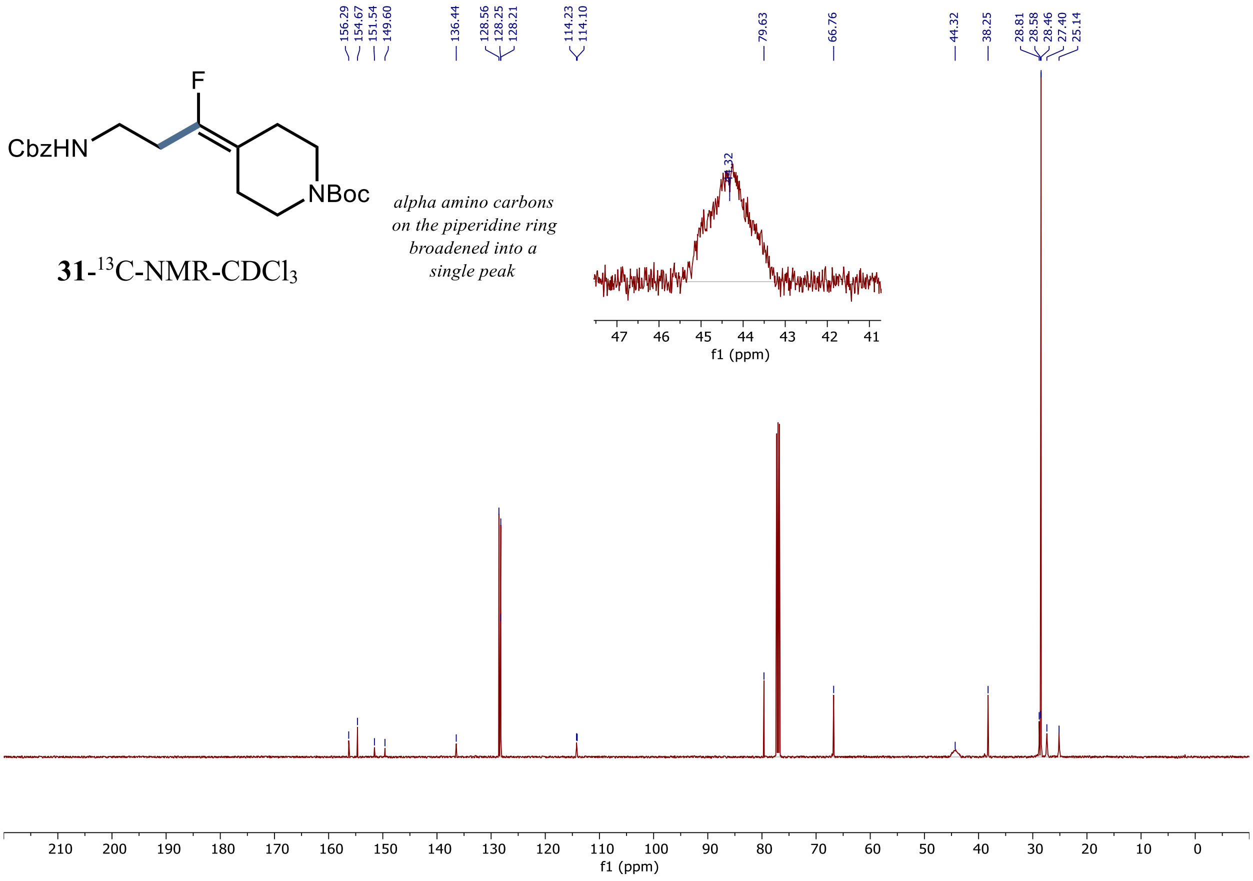
CbzHN
F
NBoc
31-¹³C-NMR-CDCl3
alpha amino carbons on the piperidine ring broadened into a single peak
156.29 154.67 151.54 149.60 136.44 128.56 128.25 128.21 114.23 114.10 79.63 66.76 44.32 38.25 28.81 28.58 28.46 27.40 25.14
f1 (ppm)

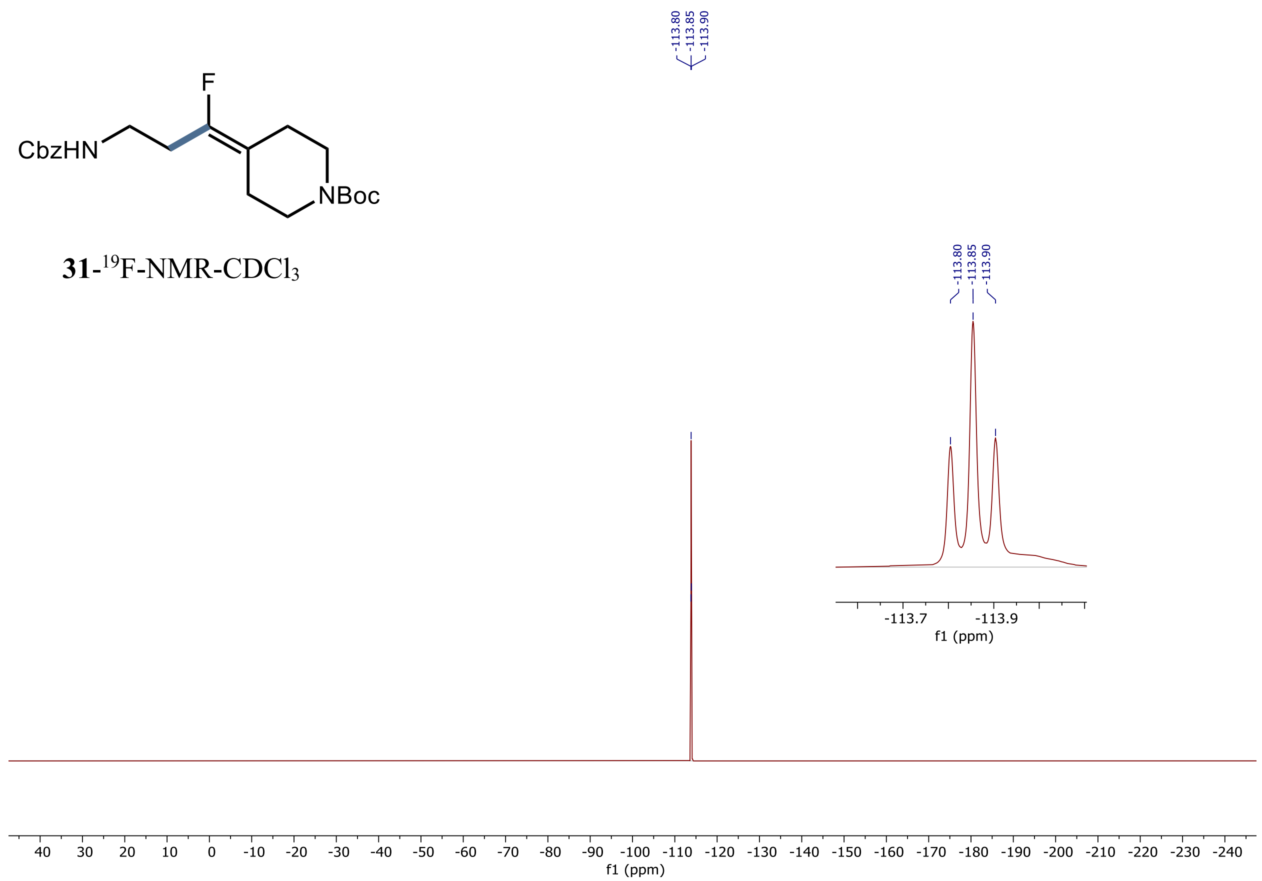

31-^{19}F-NMR-CDCl$_3$

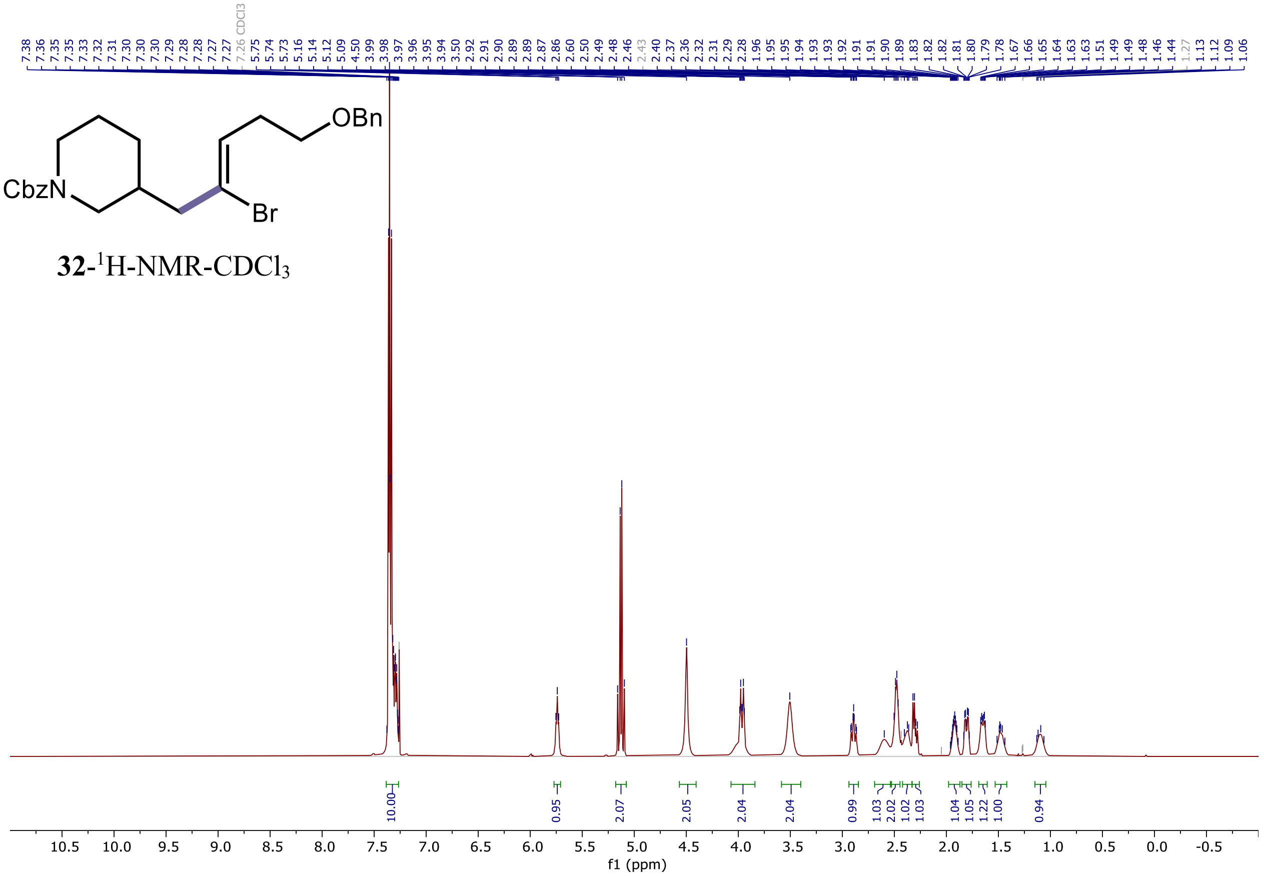

32-^{1}H-NMR-CDCl$_3$

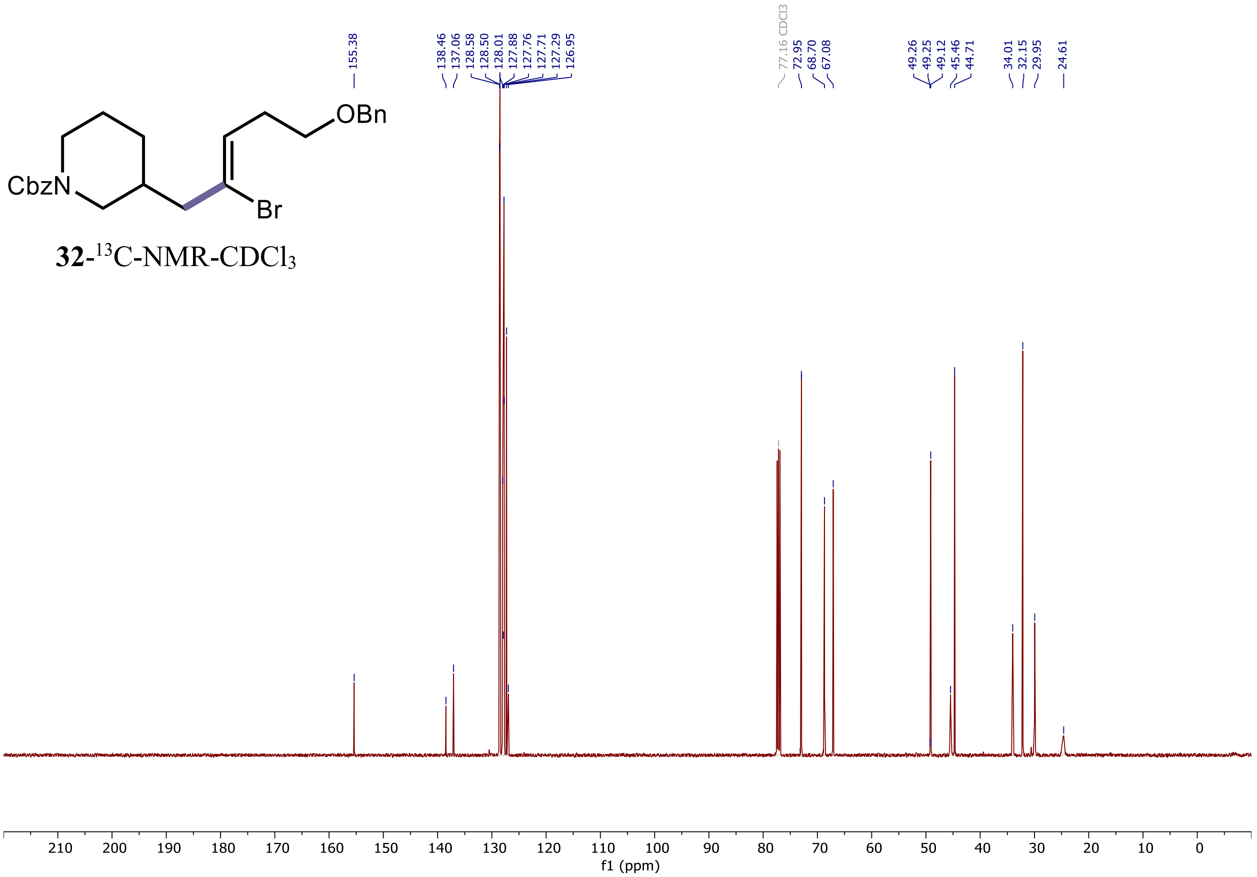
155.38
138.46
137.06
128.58
128.50
128.01
127.88
127.76
127.71
127.29
126.95
77.16 CDCl3
72.95
68.70
67.08
49.26
49.25
49.12
45.46
44.71
34.01
32.15
29.95
24.61
OBn
CbzN
Br
32-13C-NMR-CDCl3
f1 (ppm)

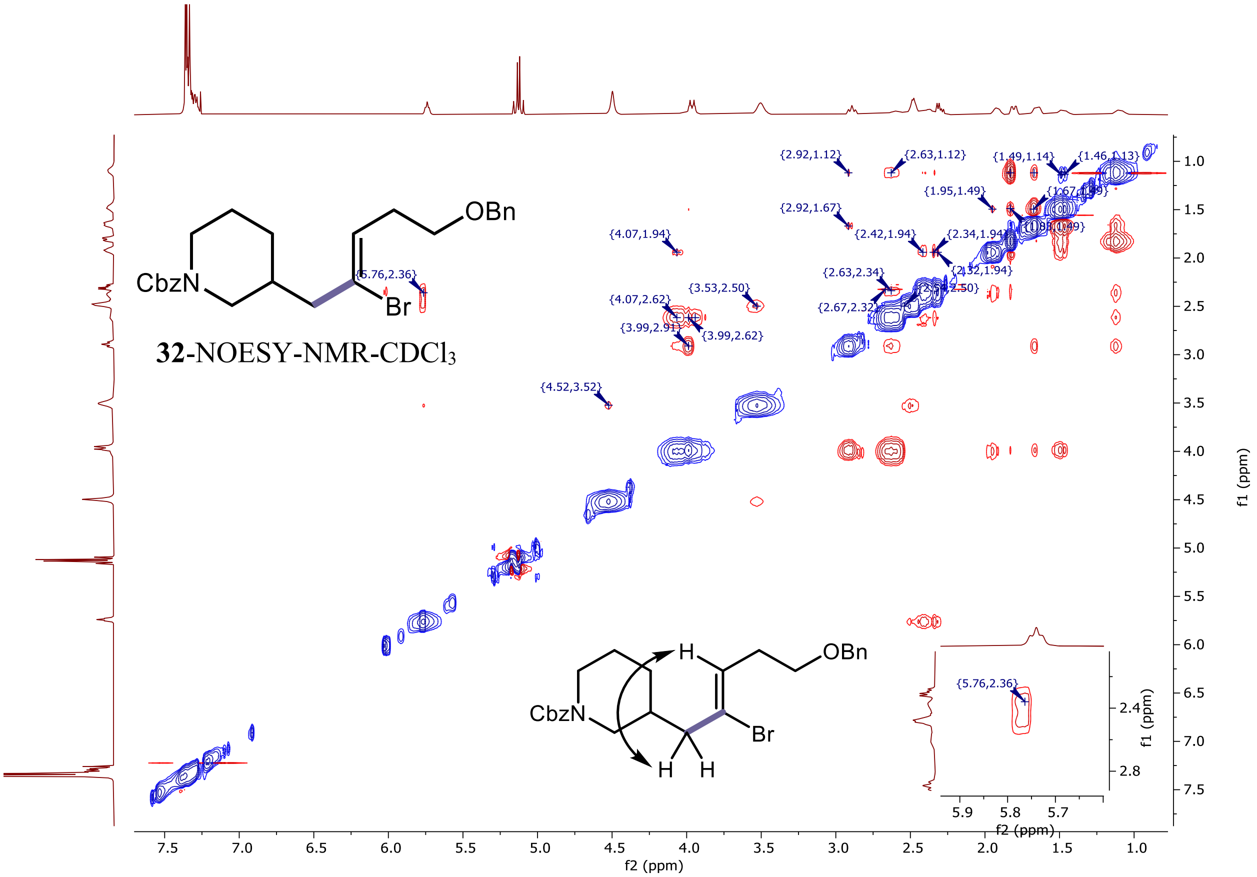
OBn
CbzN
Br
32-NOESY-NMR-CDCl3
H
H
H
f2 (ppm)
f1 (ppm)

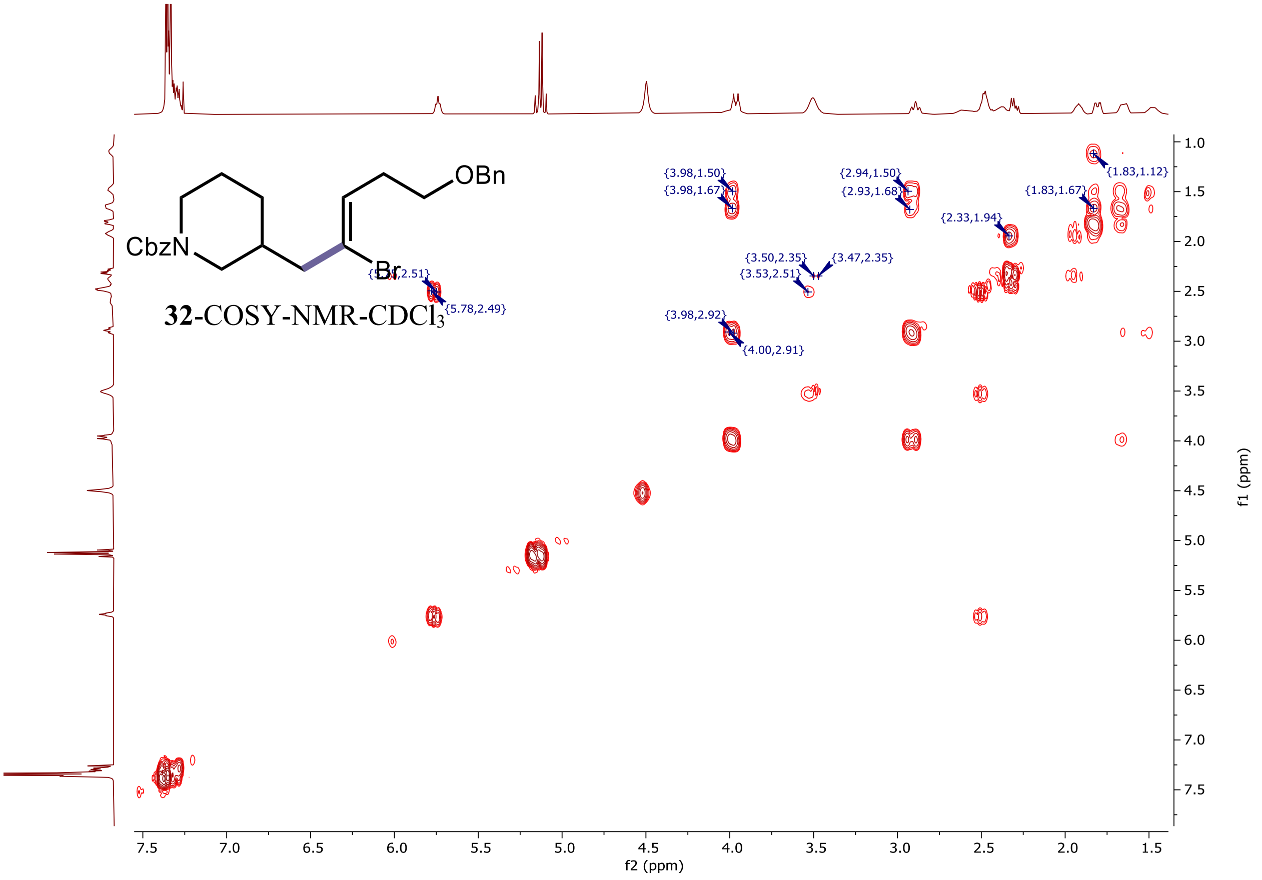

OBn
CbzN
Br
32-COSY-NMR-CDCl3
f2 (ppm)
f1 (ppm)

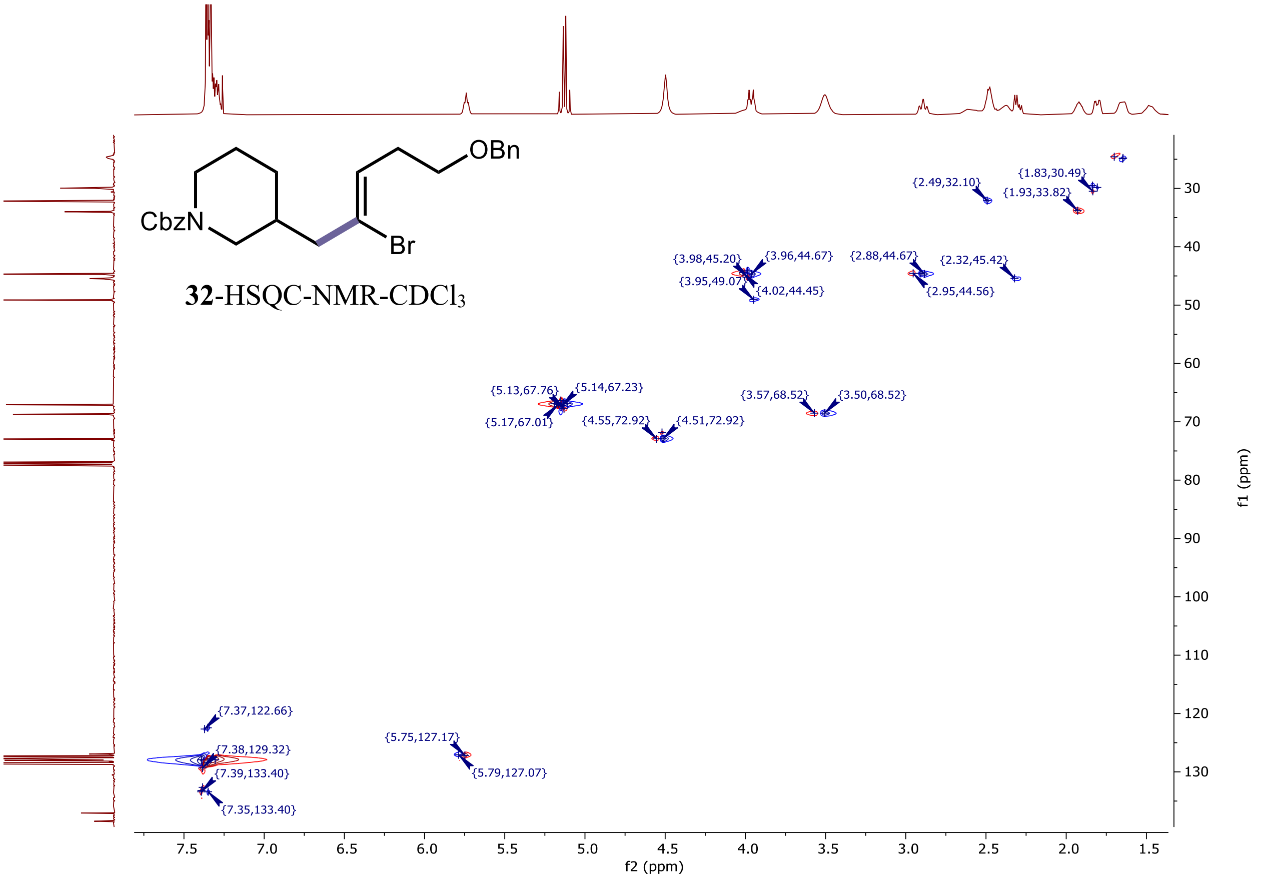

OBn
CbzN
Br
32-HSQC-NMR-CDCl3
f2 (ppm)
f1 (ppm)

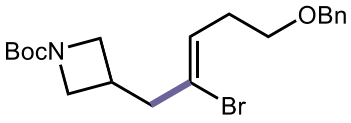

33-^{1}H-NMR-$CDCl_3$

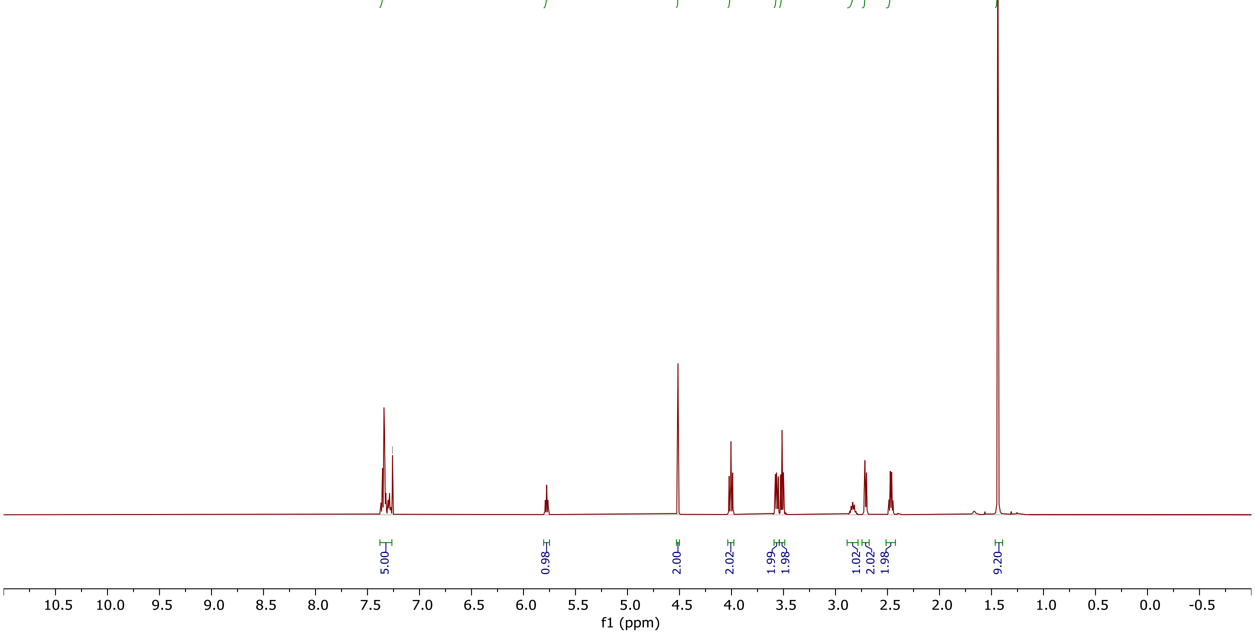

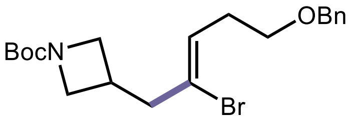

33-^{13}C-NMR-$CDCl_3$

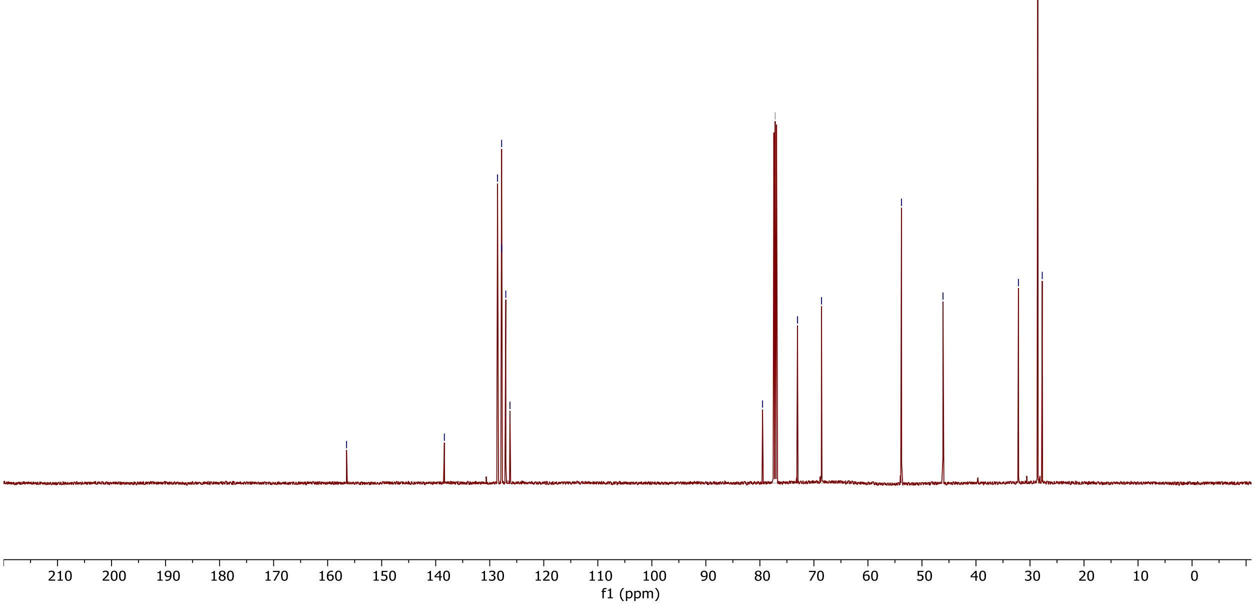

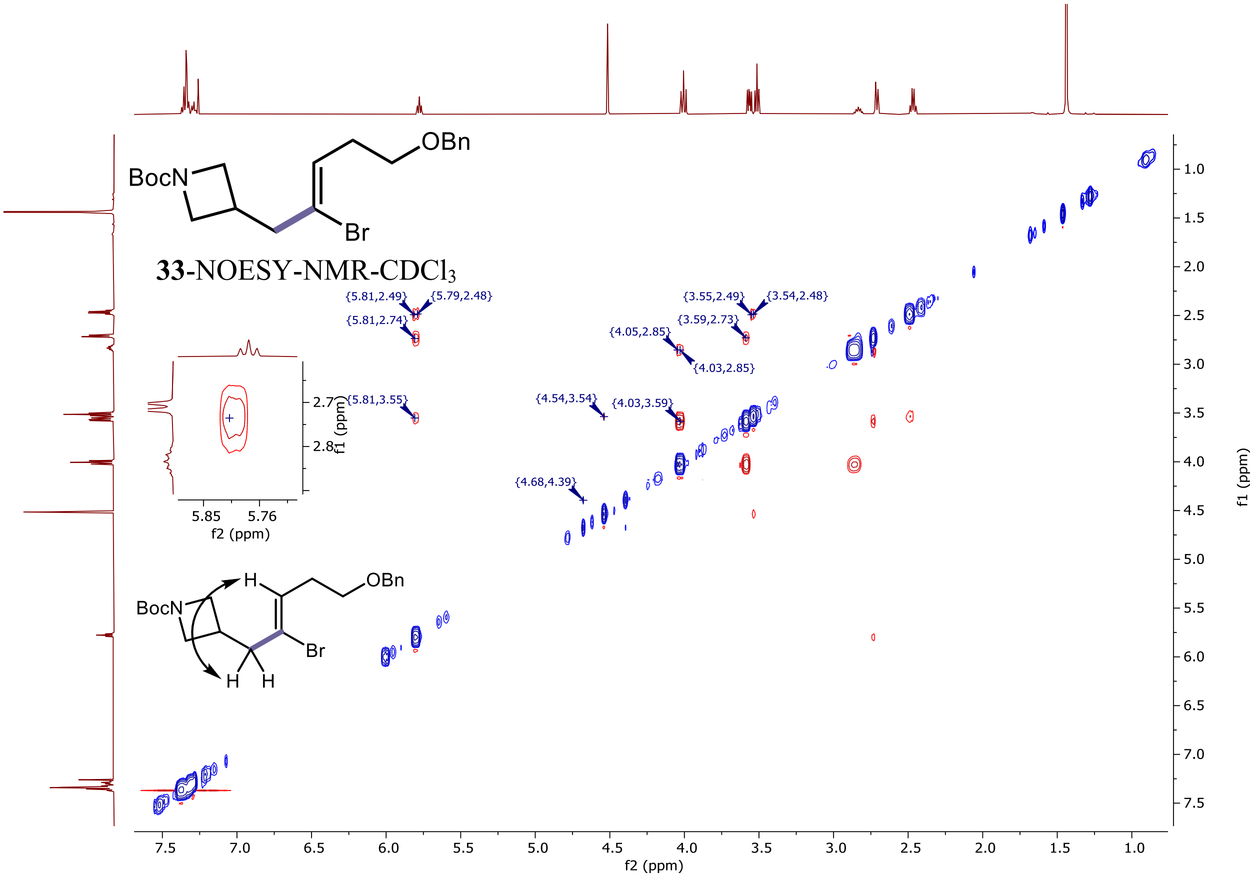
BocN
OBn
Br
33-NOESY-NMR-CDCl3
f2 (ppm)
f1 (ppm)

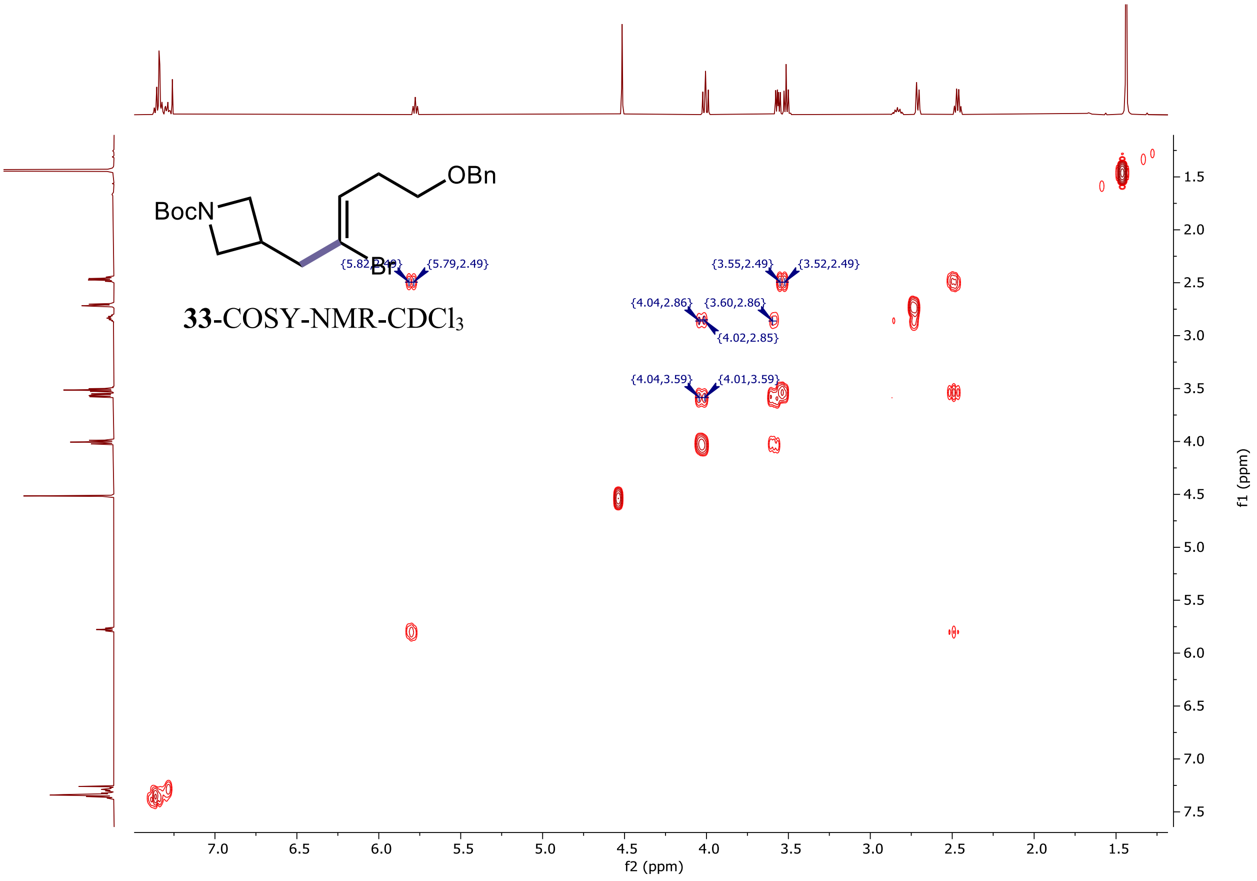
BocN
OBn
Br
33-COSY-NMR-CDCl3
f2 (ppm)
f1 (ppm)

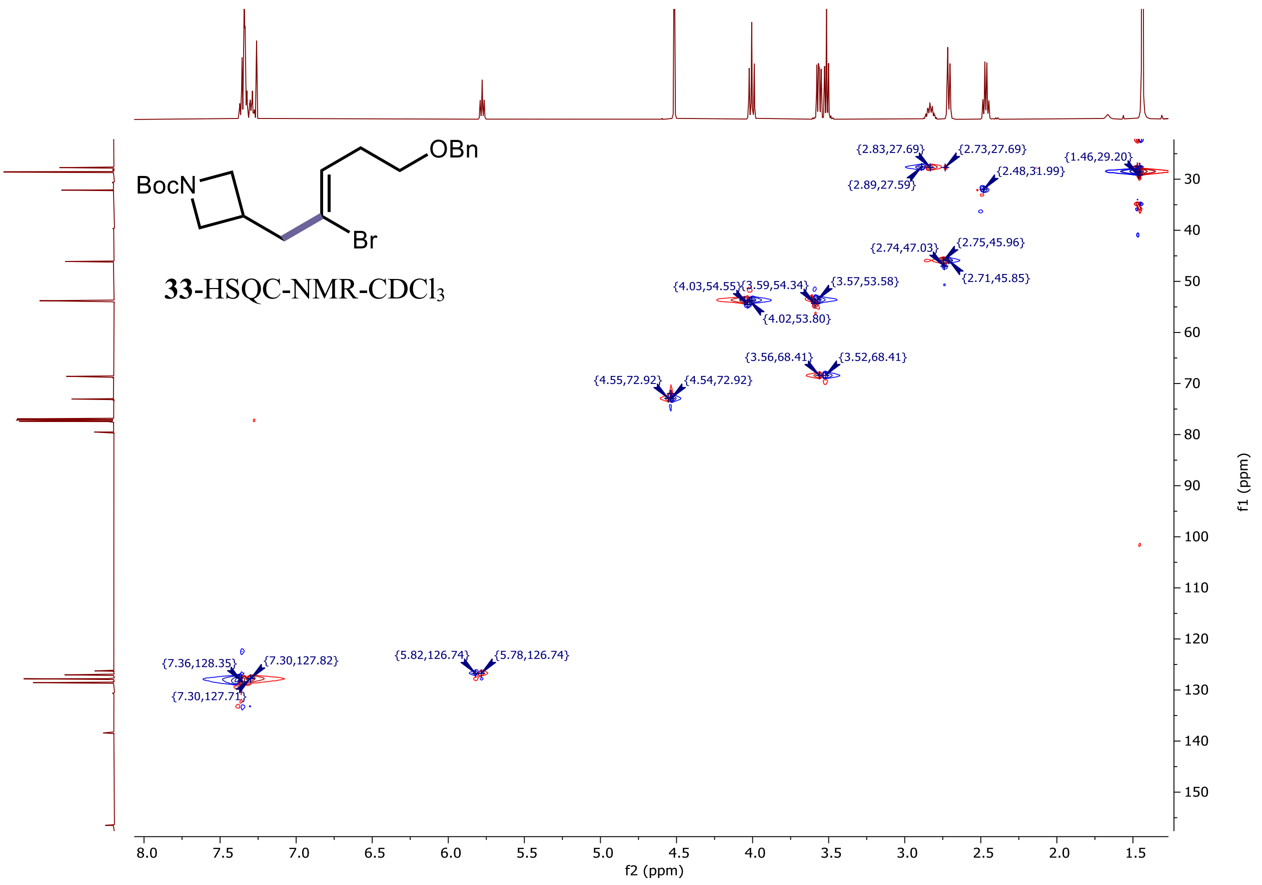

33-HSQC-NMR-CDCl$_3$

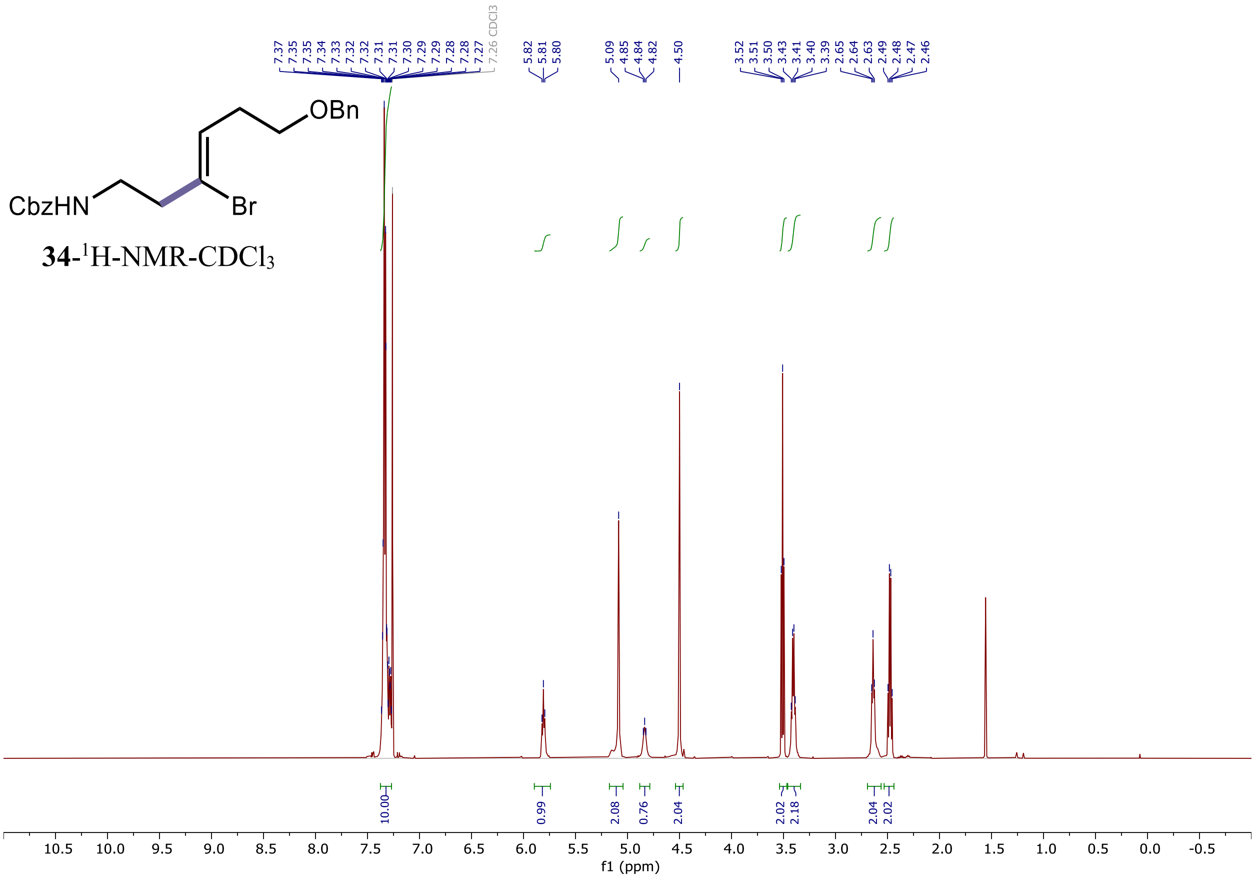

34-^{1}H-NMR-CDCl$_3$

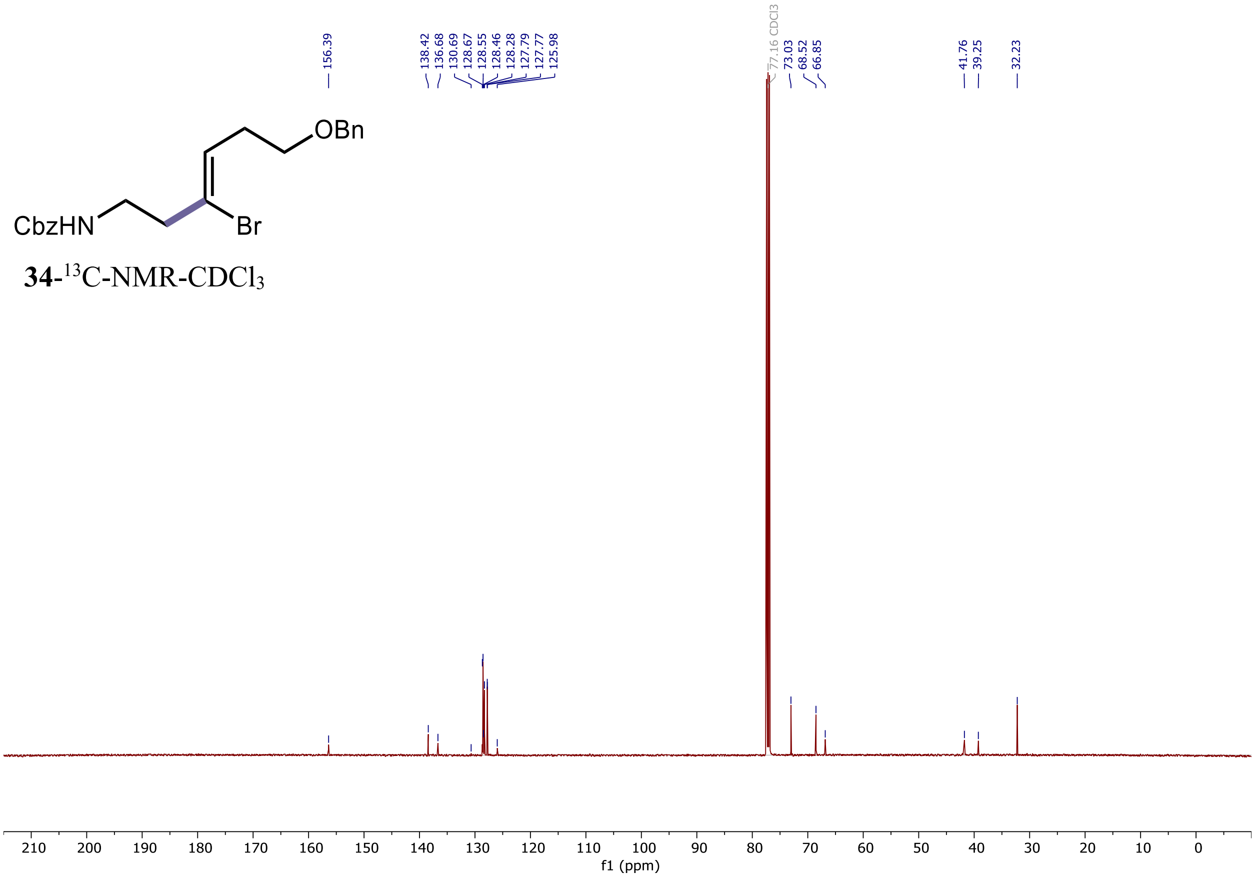

156.39
138.42
136.68
130.69
128.67
128.55
128.46
128.28
127.79
127.77
125.98
77.16 CDCl3
73.03
68.52
66.85
41.76
39.25
32.23
OBn
CbzHN
Br
34-^{13}C-NMR-CDCl$_3$
f1 (ppm)

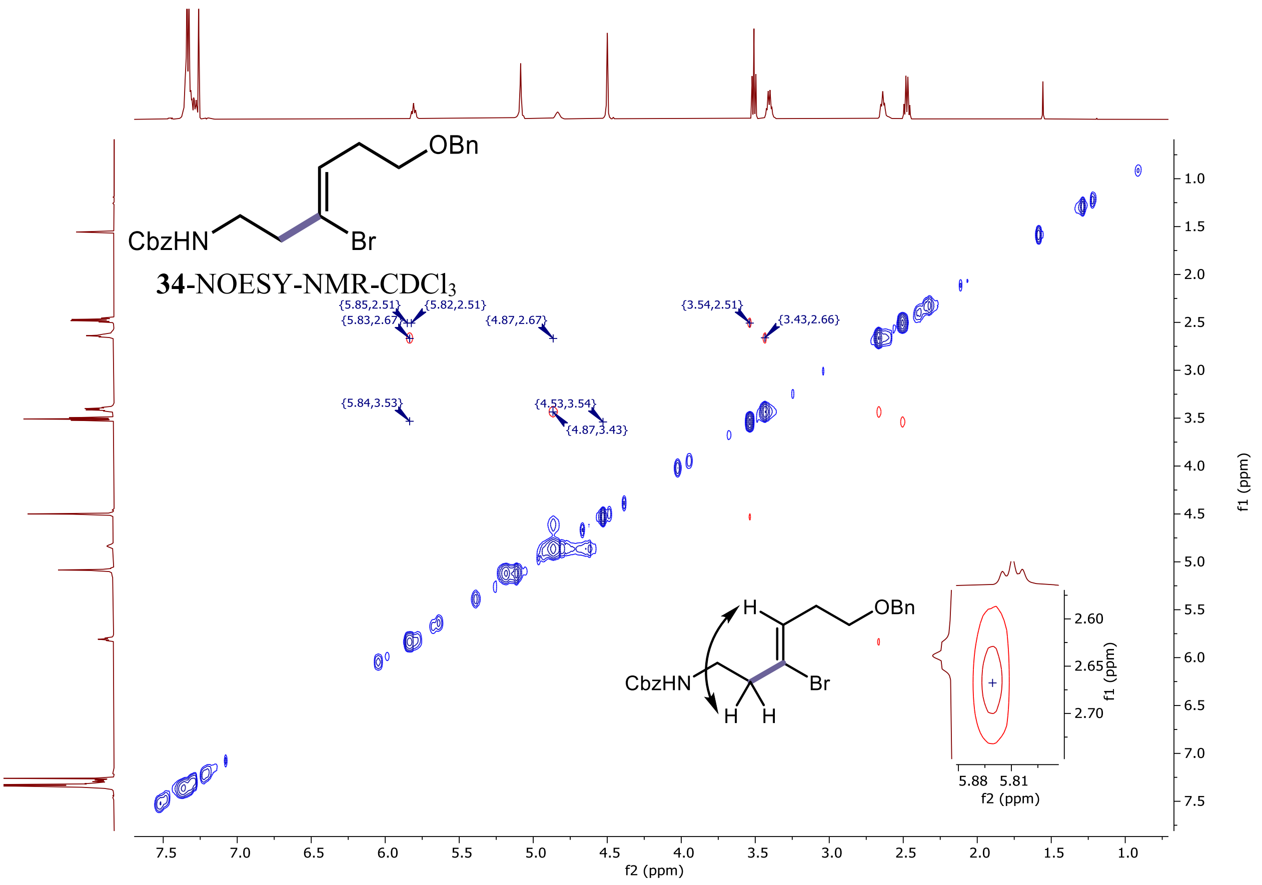

OBn
CbzHN
Br
34-NOESY-NMR-CDCl$_3$
{5.85,2.51}
{5.82,2.51}
{5.83,2.67}
{4.87,2.67}
{3.54,2.51}
{3.43,2.66}
{5.84,3.53}
{4.53,3.54}
{4.87,3.43}
H
H
H
f2 (ppm)
f1 (ppm)

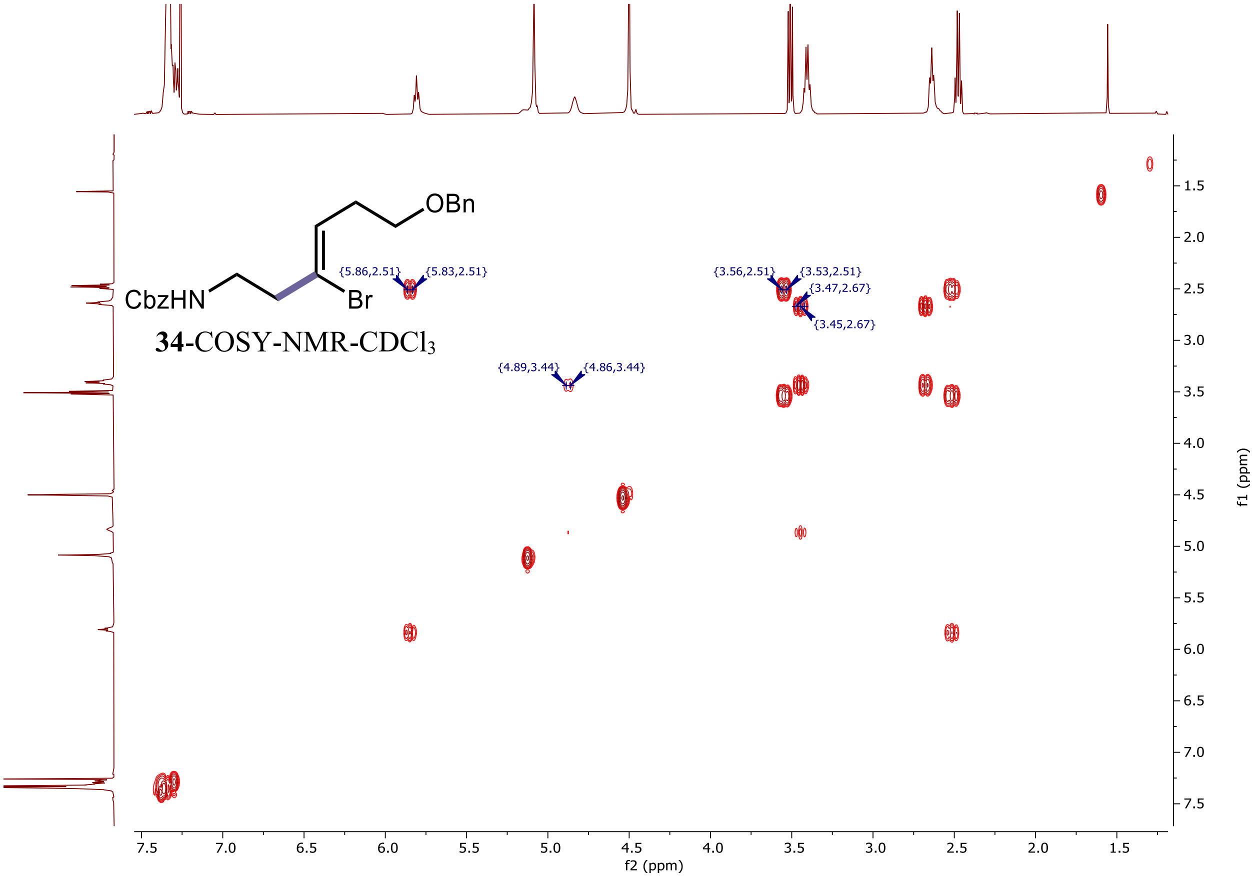

34-COSY-NMR-CDCl$_3$

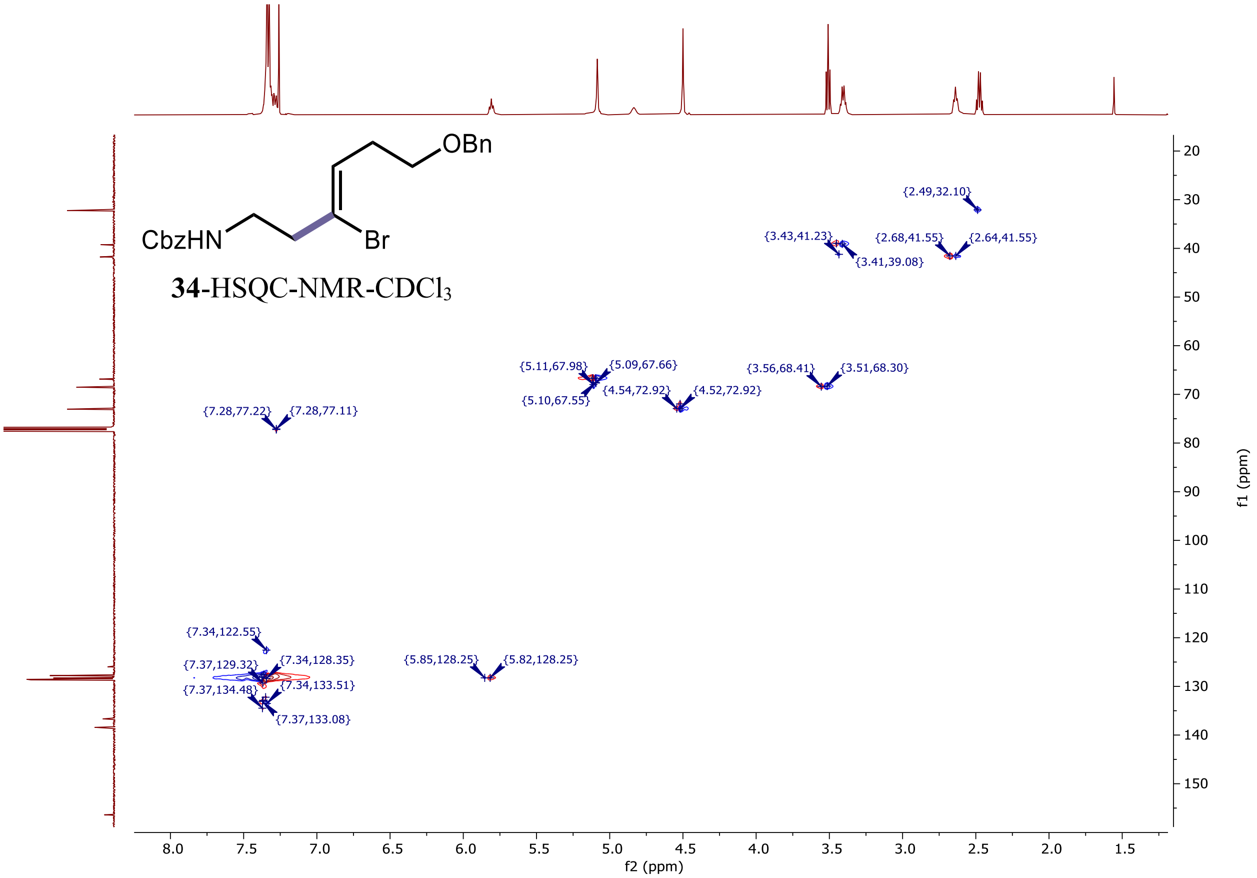

34-HSQC-NMR-CDCl$_3$

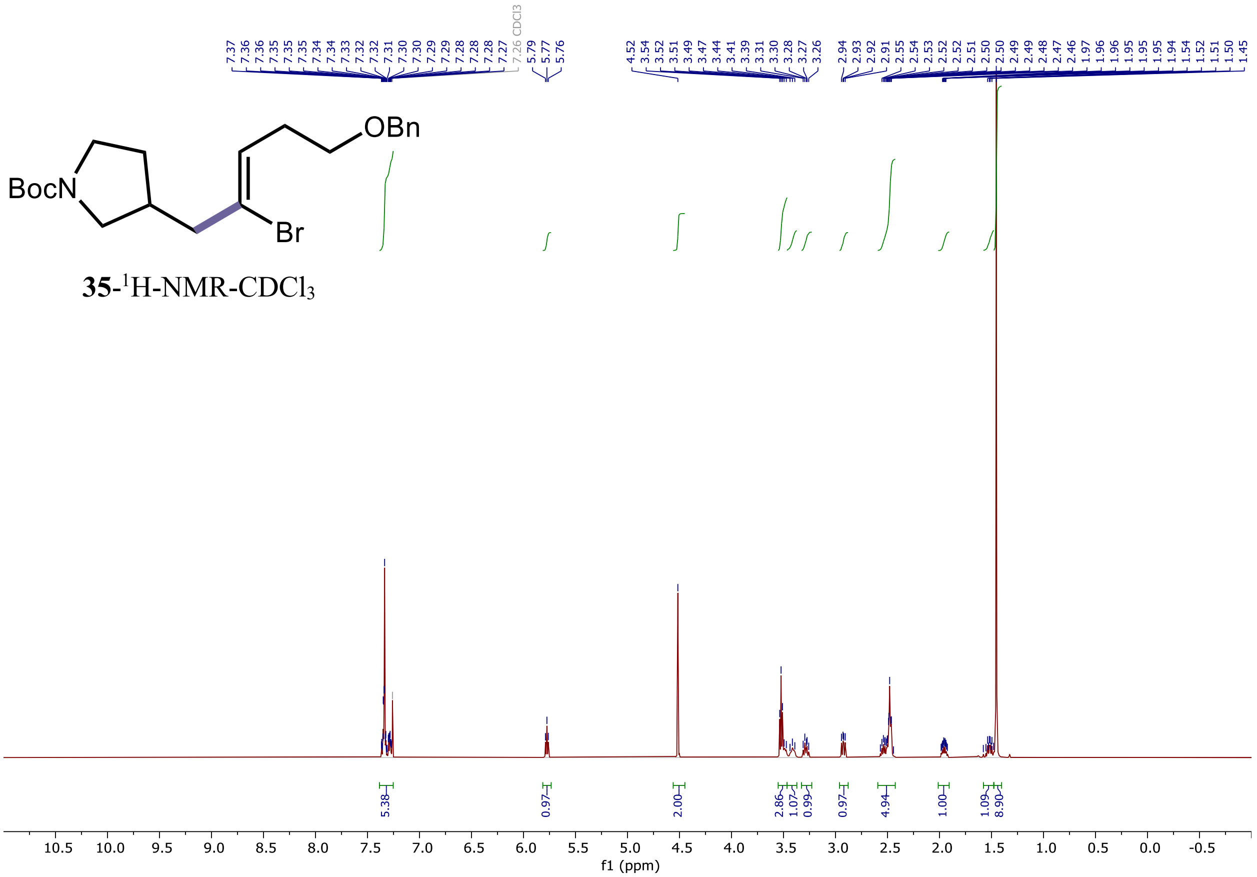

35-^{1}H-NMR-$CDCl_3$

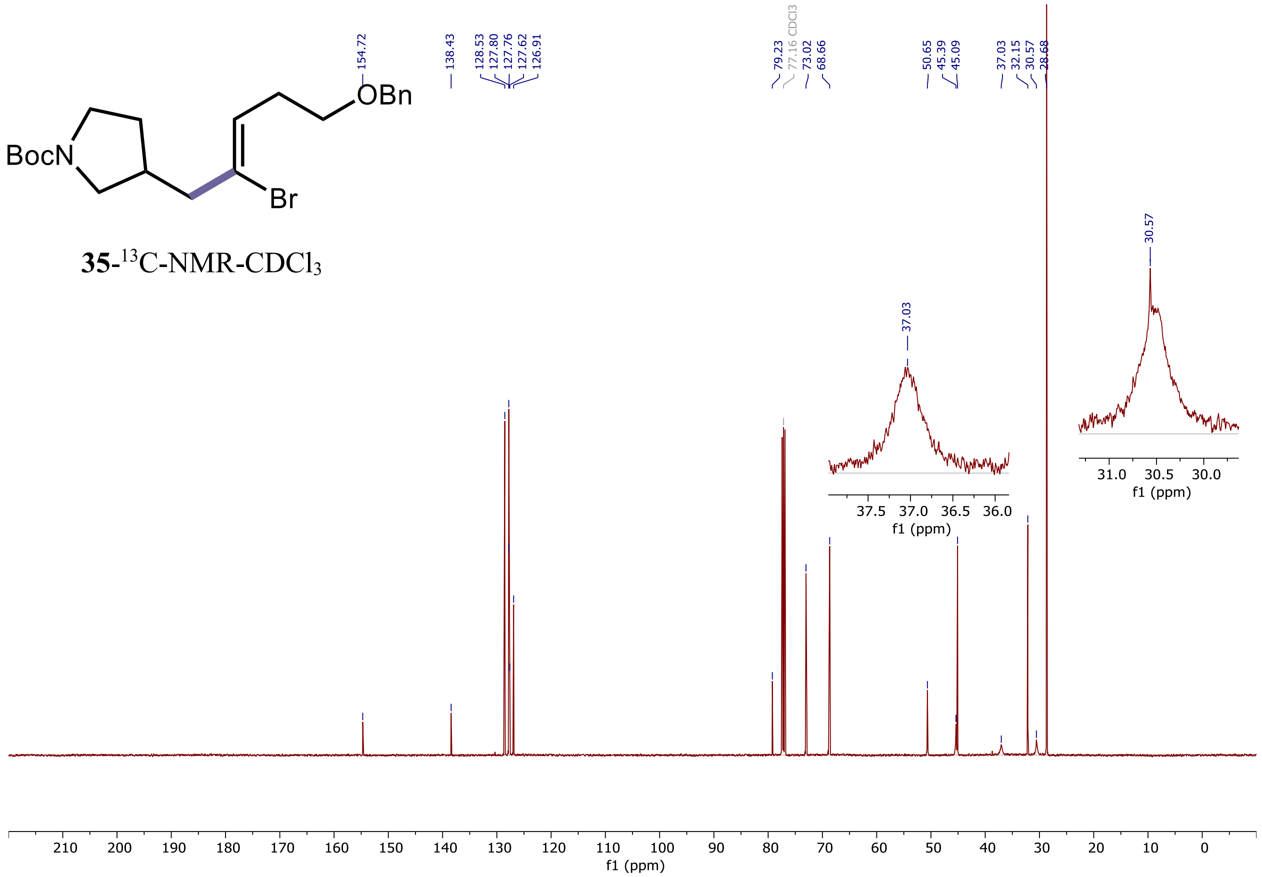

35-^{13}C-NMR-$CDCl_3$

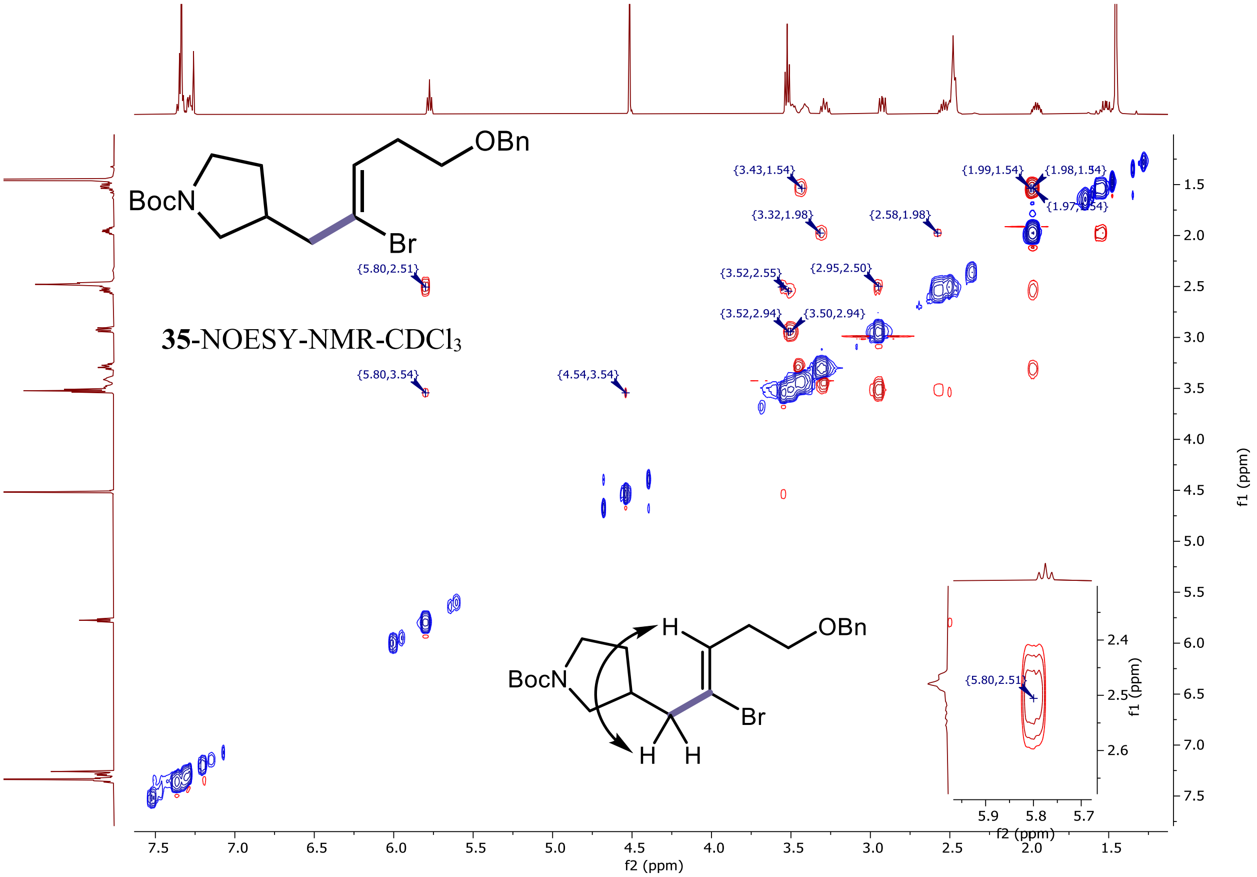

35-NOESY-NMR-CDCl$_3$

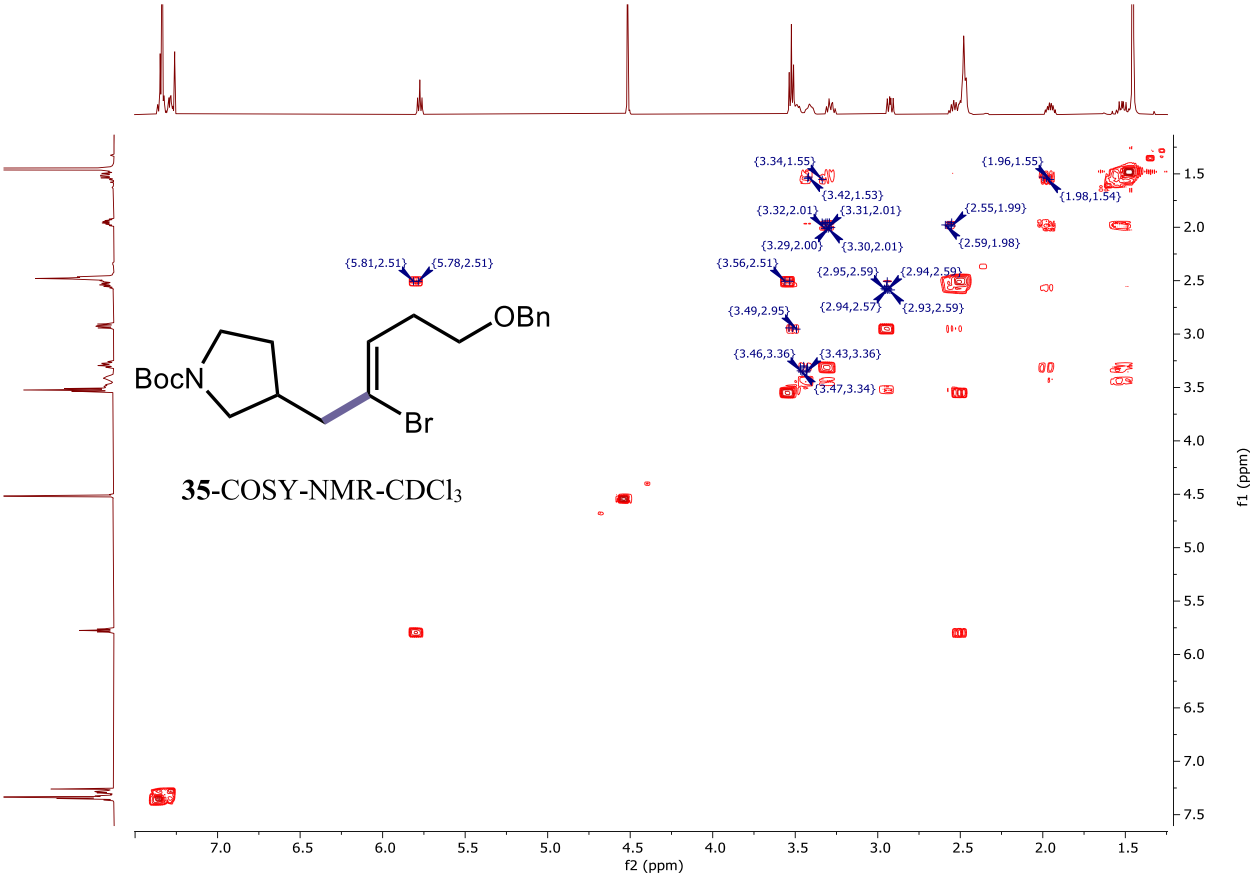

35-COSY-NMR-CDCl$_3$

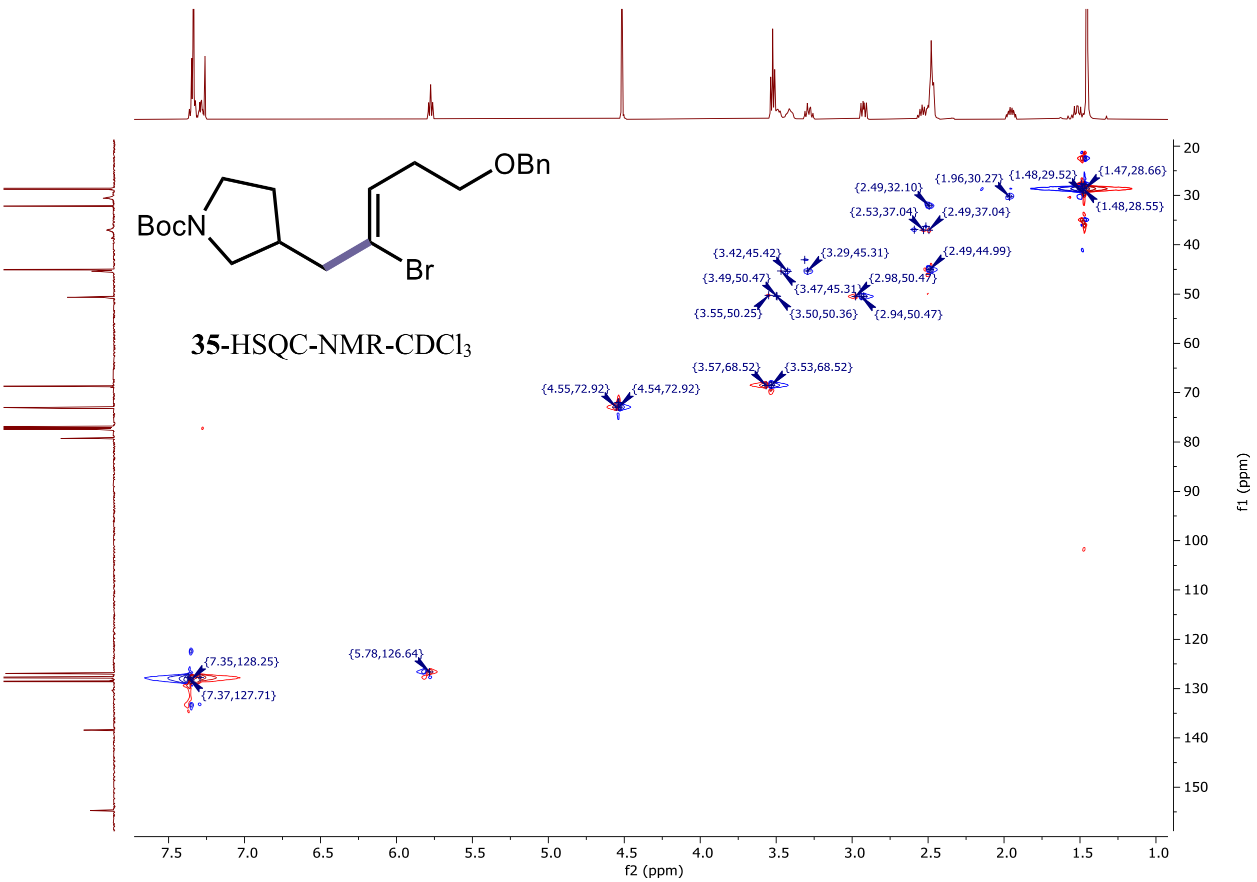

35-HSQC-NMR-CDCl$_3$

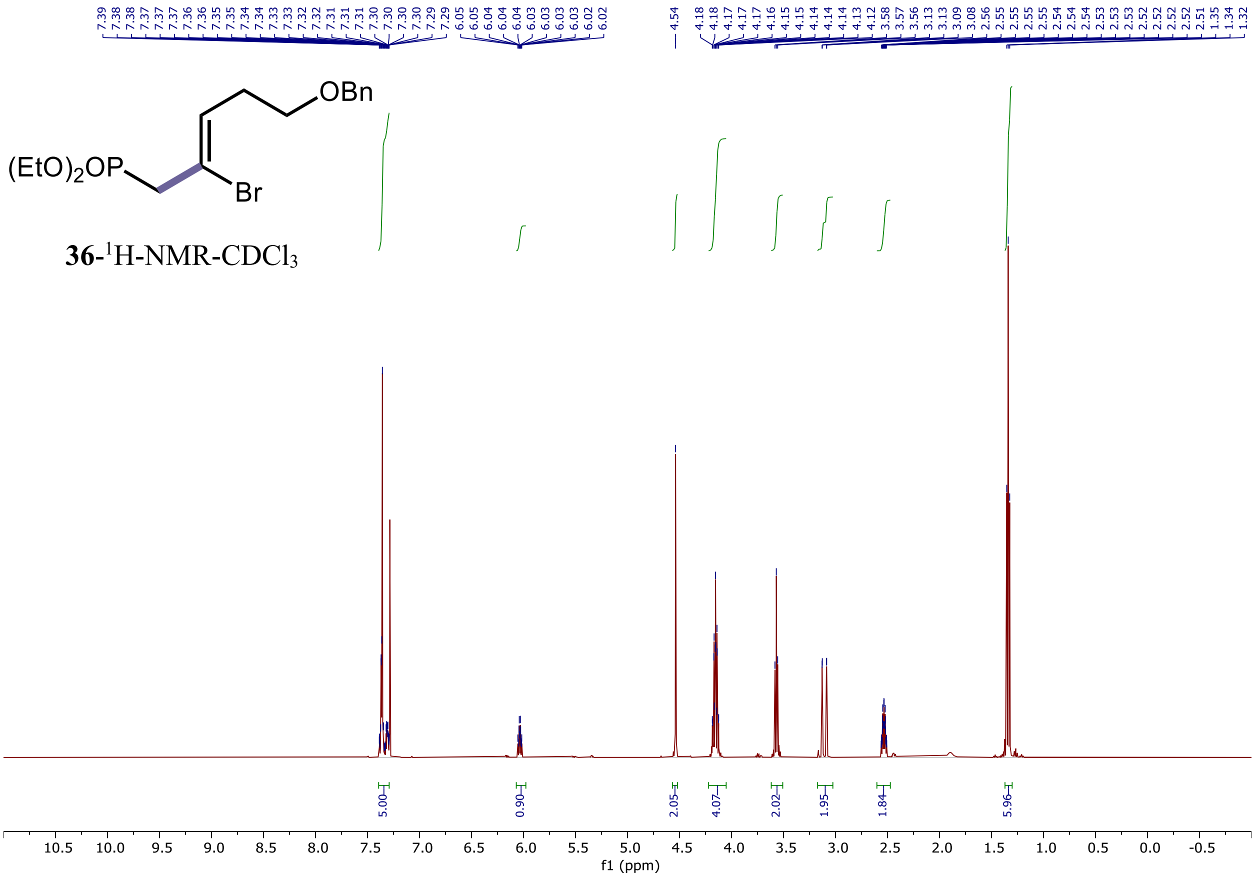

36-^{1}H-NMR-CDCl$_3$

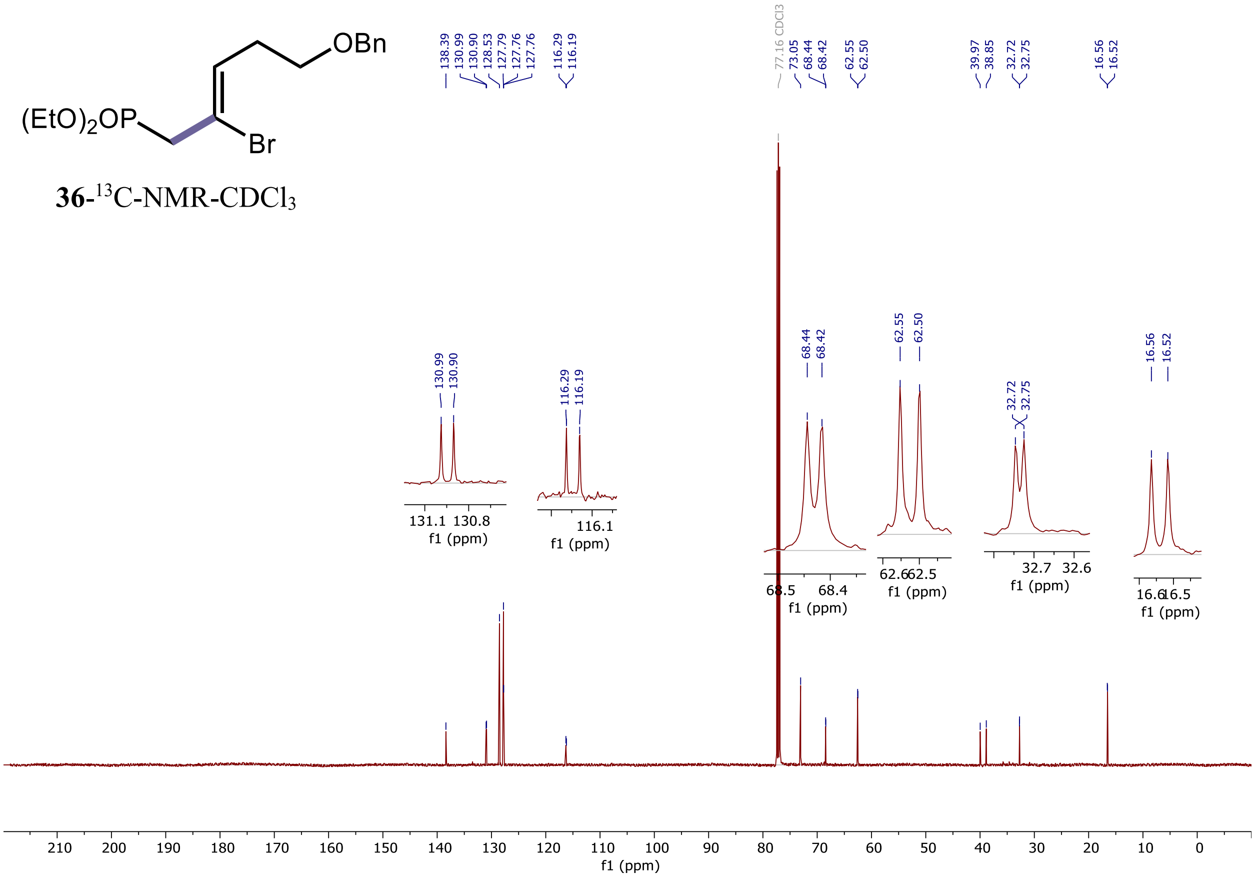
(EtO)2OP
Br
OBn
36-13C-NMR-CDCl3
138.39
130.99
130.90
128.53
127.79
127.76
127.76
116.29
116.19
77.16 CDCl3
73.05
68.44
68.42
62.55
62.50
39.97
38.85
32.72
32.75
16.56
16.52
f1 (ppm)

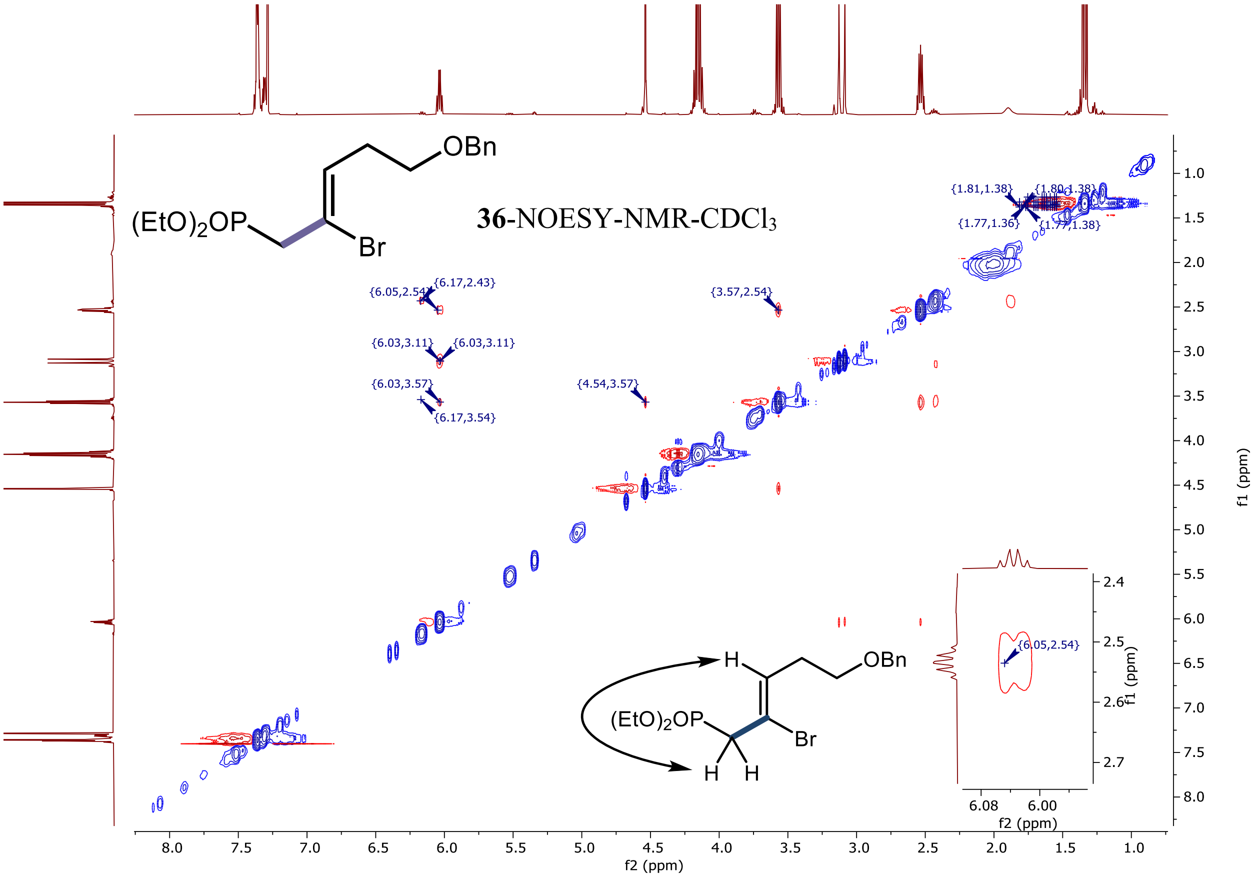
(EtO)2OP
Br
OBn
36-NOESY-NMR-CDCl3
{6.05,2.54}
{6.17,2.43}
{3.57,2.54}
{6.03,3.11}
{6.03,3.11}
{6.03,3.57}
{6.17,3.54}
{4.54,3.57}
{1.81,1.38}
{1.80,1.38}
{1.77,1.36}
{1.77,1.38}
H
H
H
f2 (ppm)
f1 (ppm)

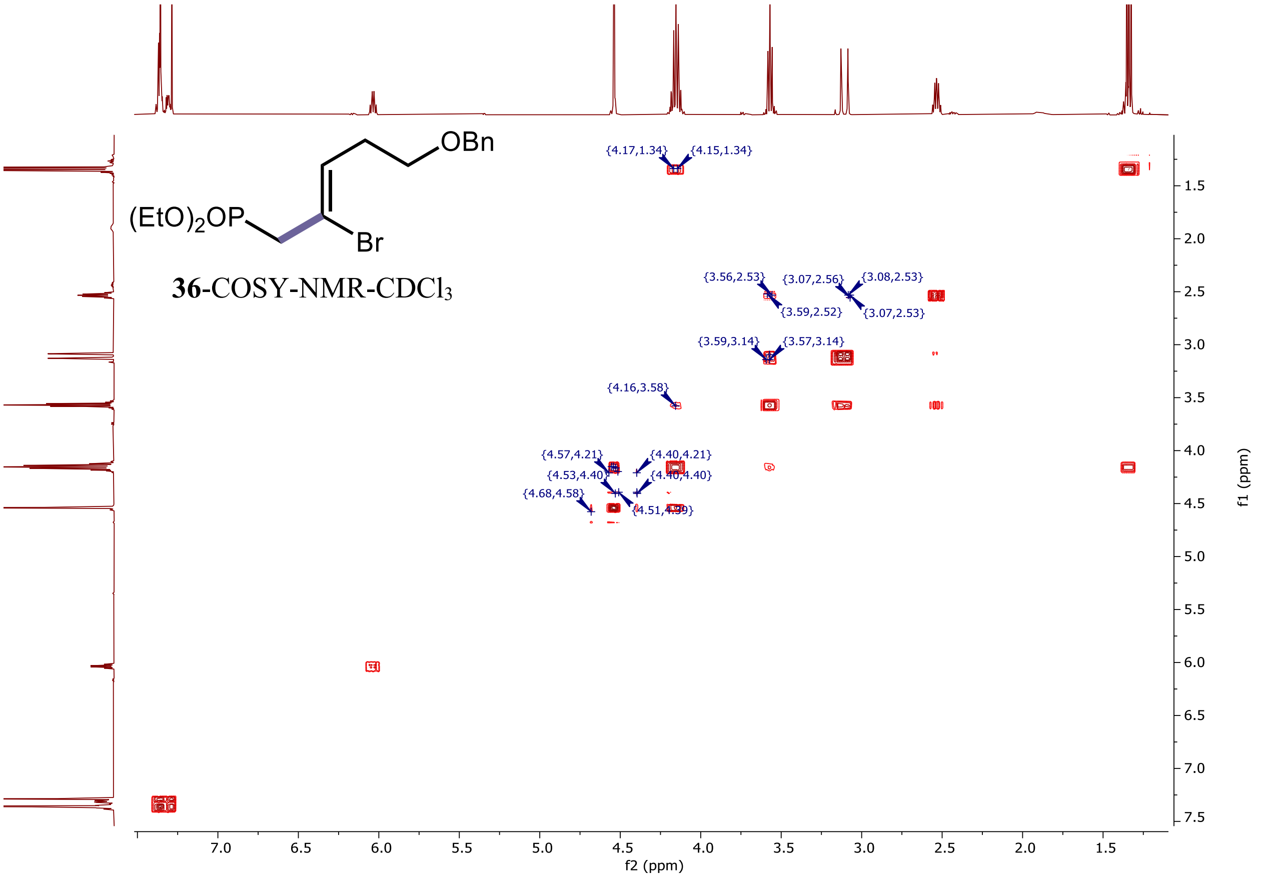
OBn
(EtO)2OP
Br
36-COSY-NMR-CDCl3
f2 (ppm)
f1 (ppm)

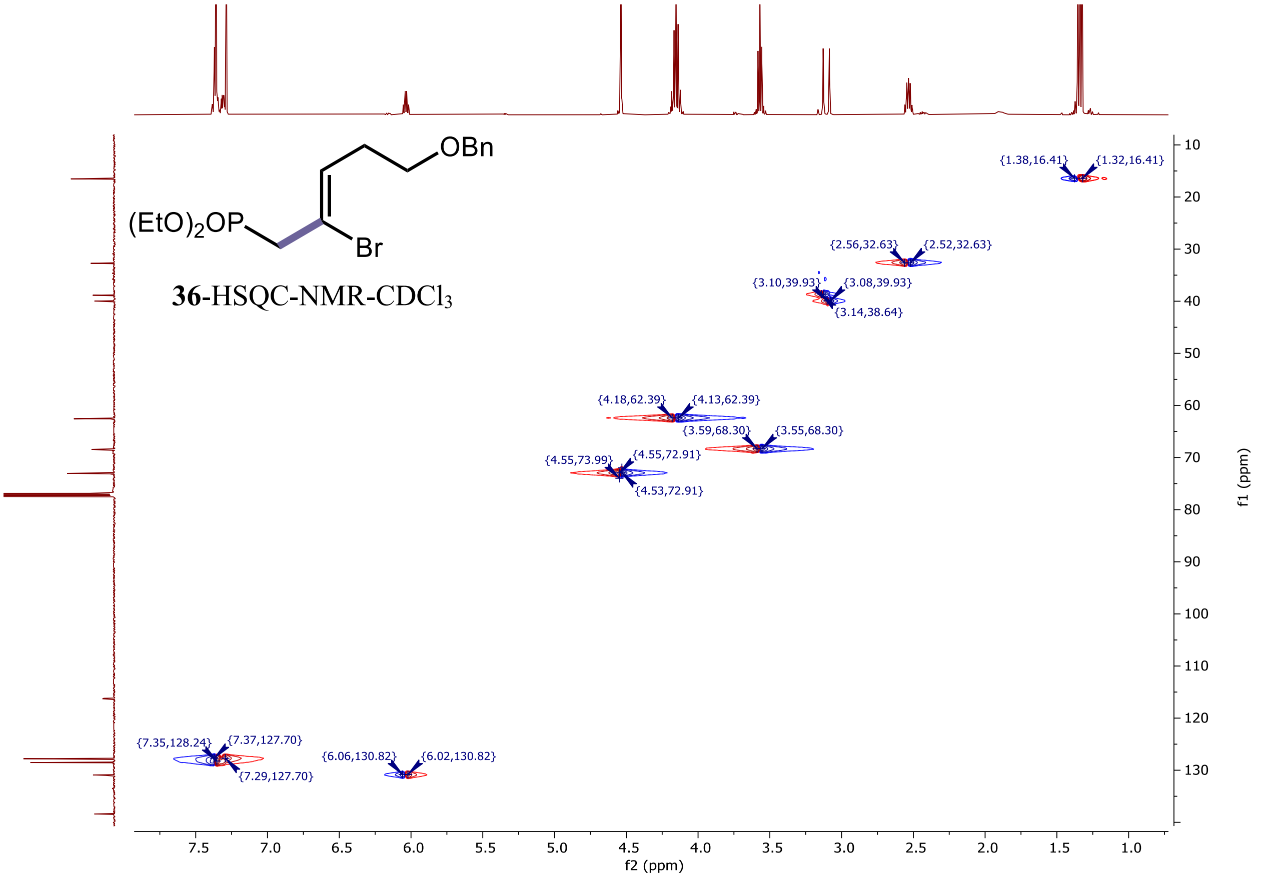
OBn
(EtO)2OP
Br
36-HSQC-NMR-CDCl3
f2 (ppm)
f1 (ppm)

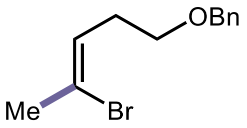

37-¹H-NMR-CDCl₃

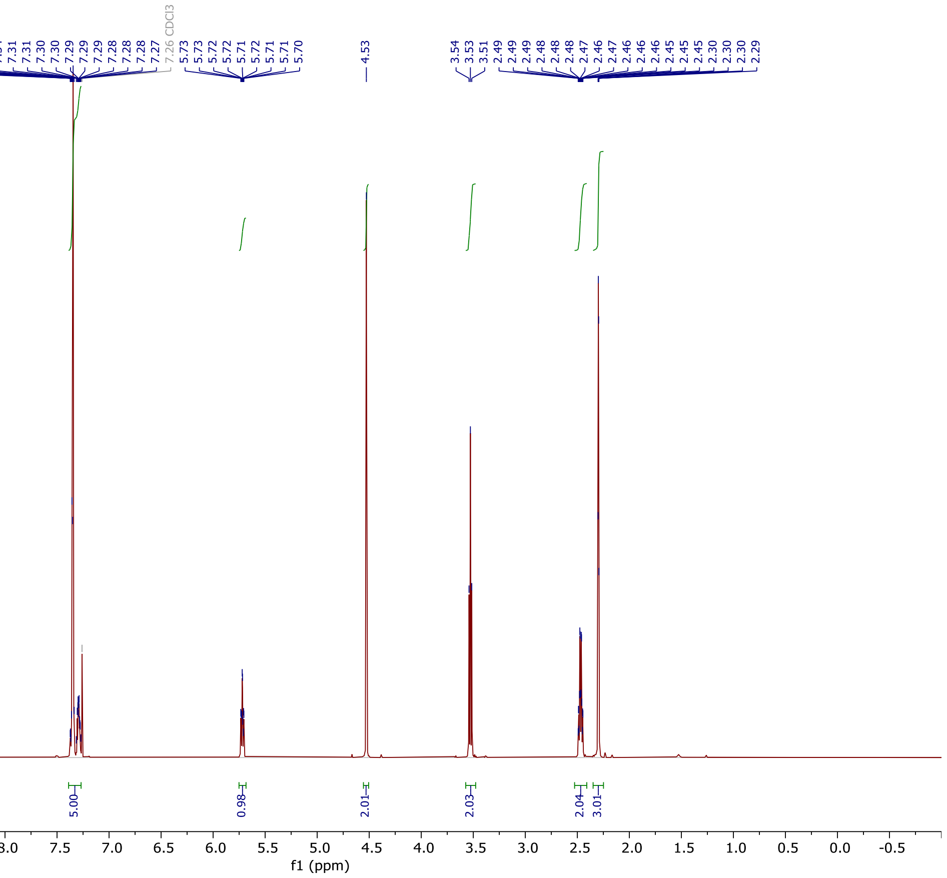

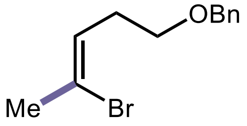

37-¹³C-NMR-CDCl₃

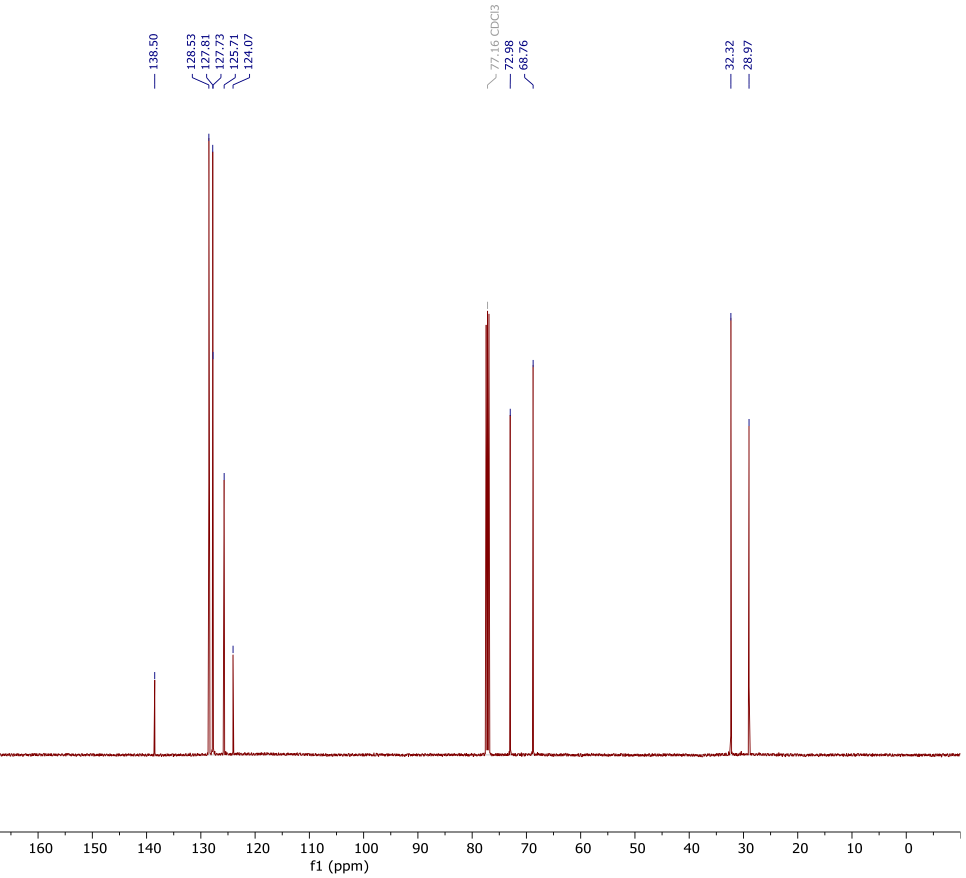

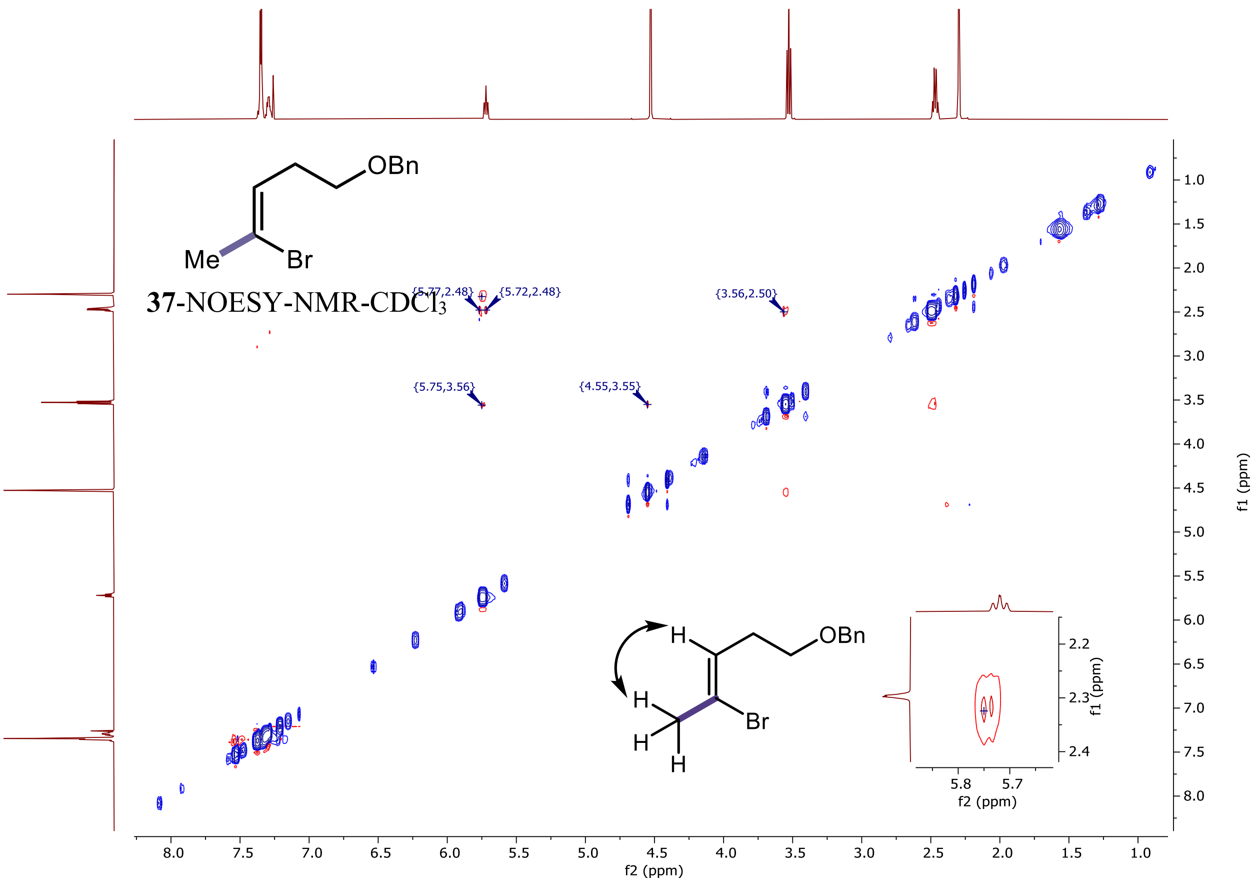
OBn
Me
Br
37-NOESY-NMR-CDCl3
{5.77,2.48}
{5.72,2.48}
{3.56,2.50}
{5.75,3.56}
{4.55,3.55}
H
H
H
H
Br
OBn
f2 (ppm)
f1 (ppm)

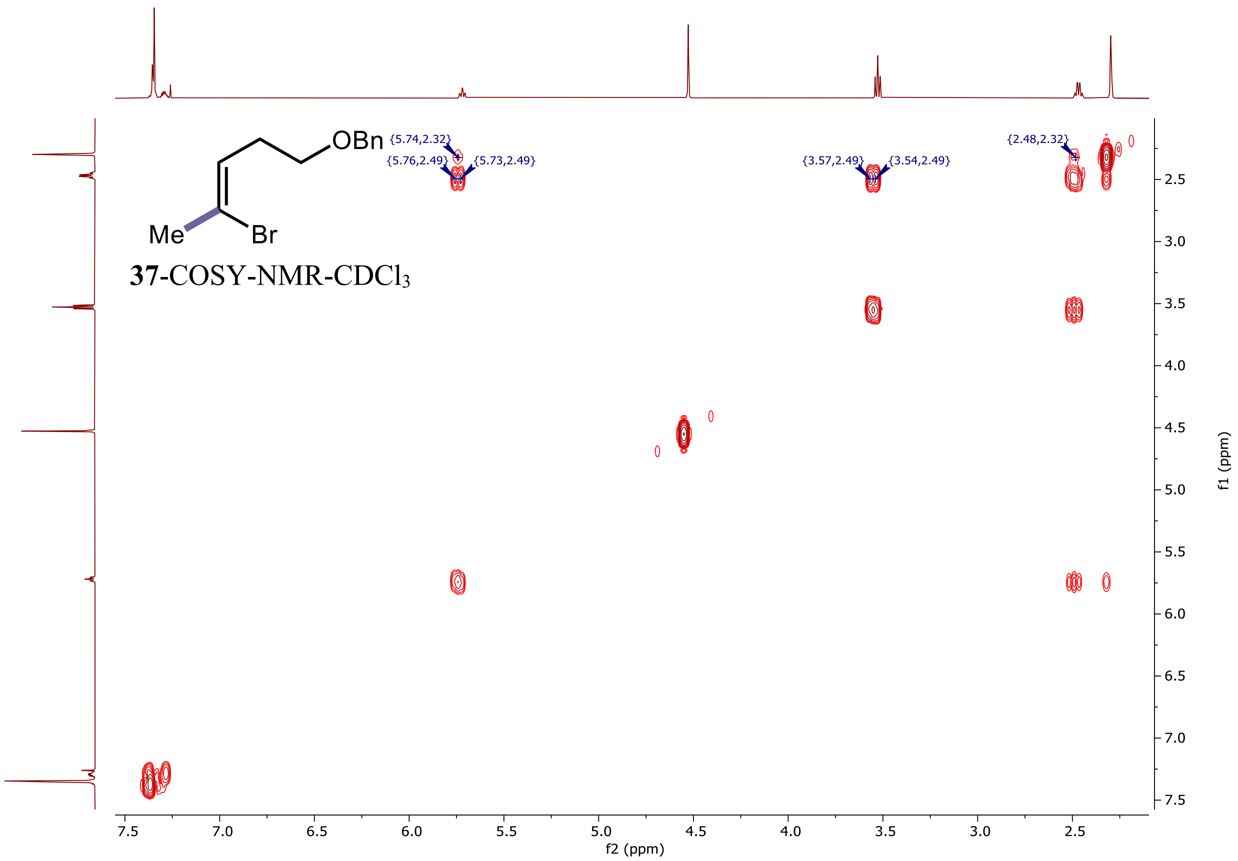
OBn
Me
Br
37-COSY-NMR-CDCl3
{5.74,2.32}
{5.76,2.49}
{5.73,2.49}
{3.57,2.49}
{3.54,2.49}
{2.48,2.32}
f2 (ppm)
f1 (ppm)

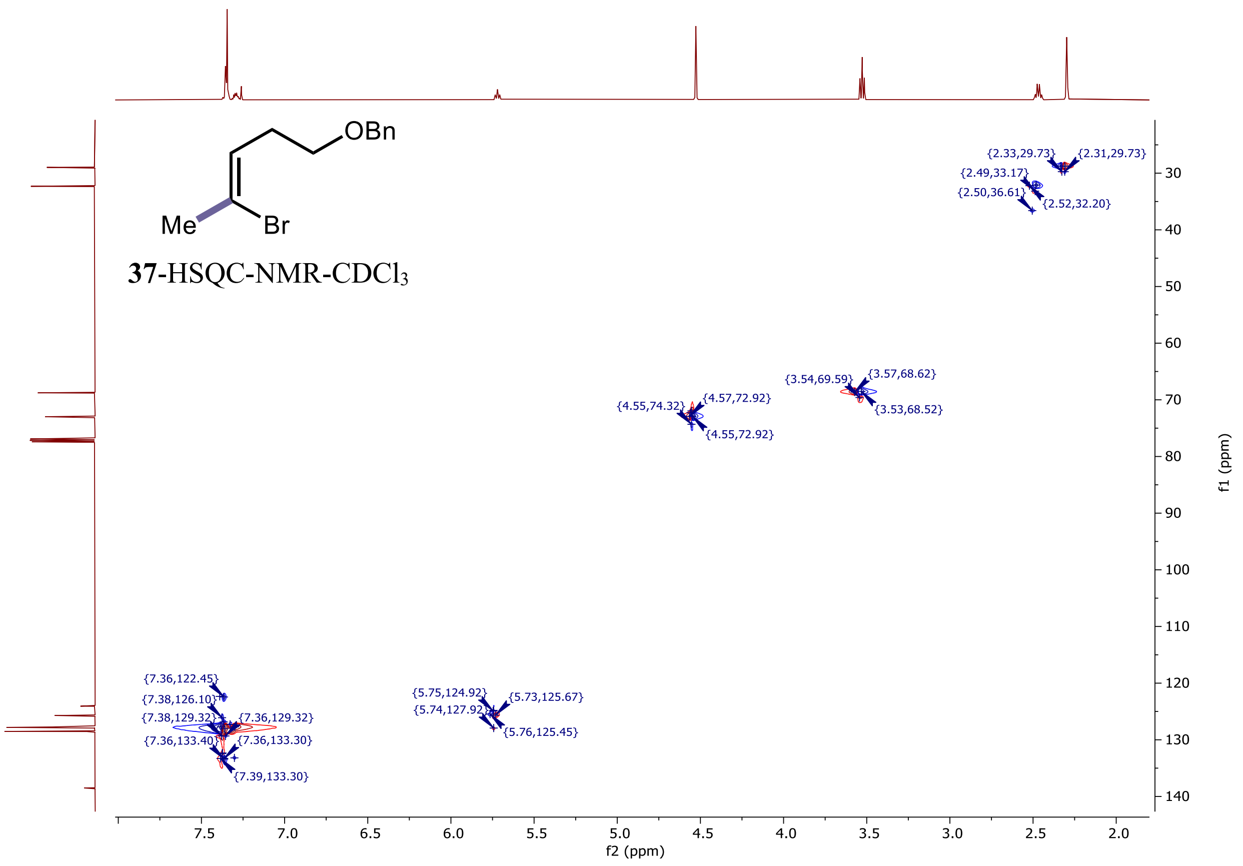

37-HSQC-NMR-$CDCl_3$

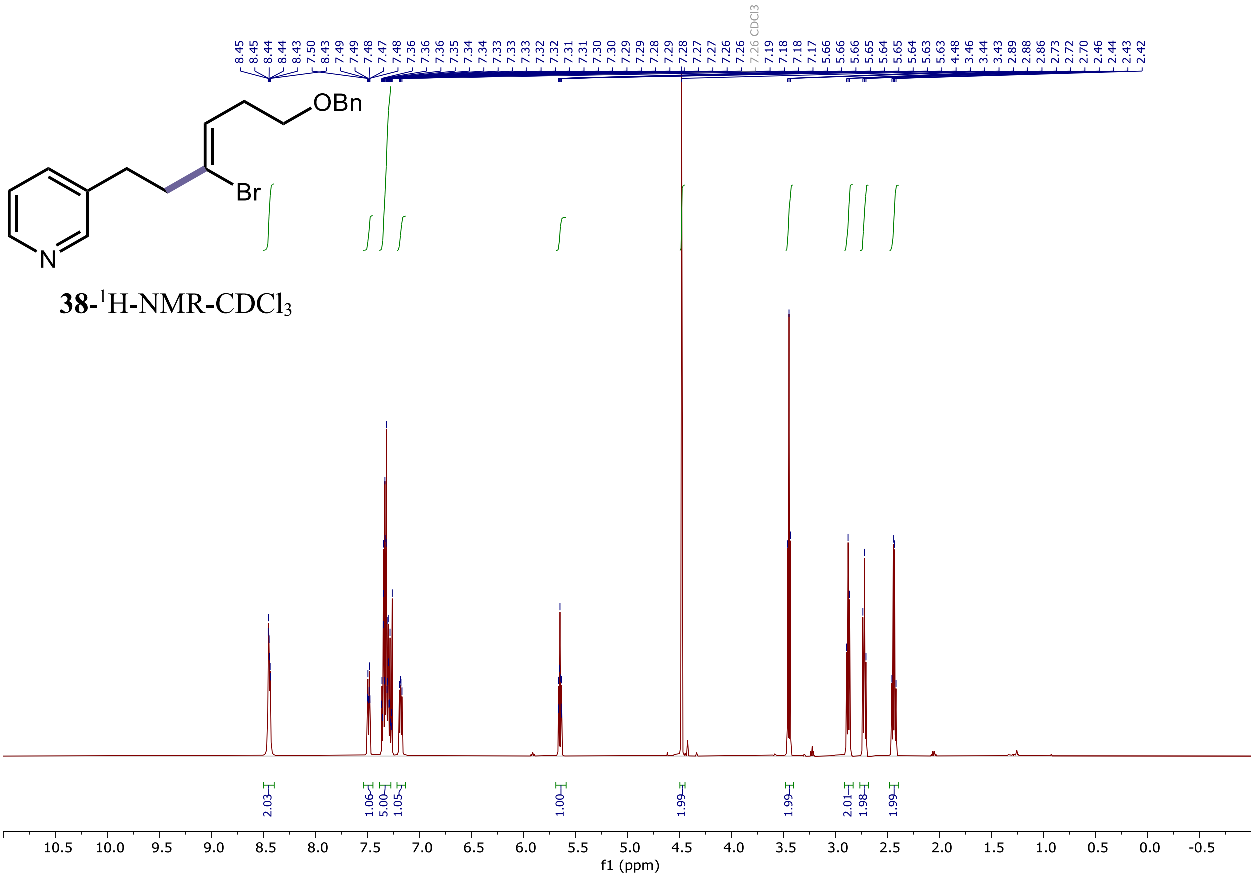

38-^{1}H-NMR-$CDCl_3$

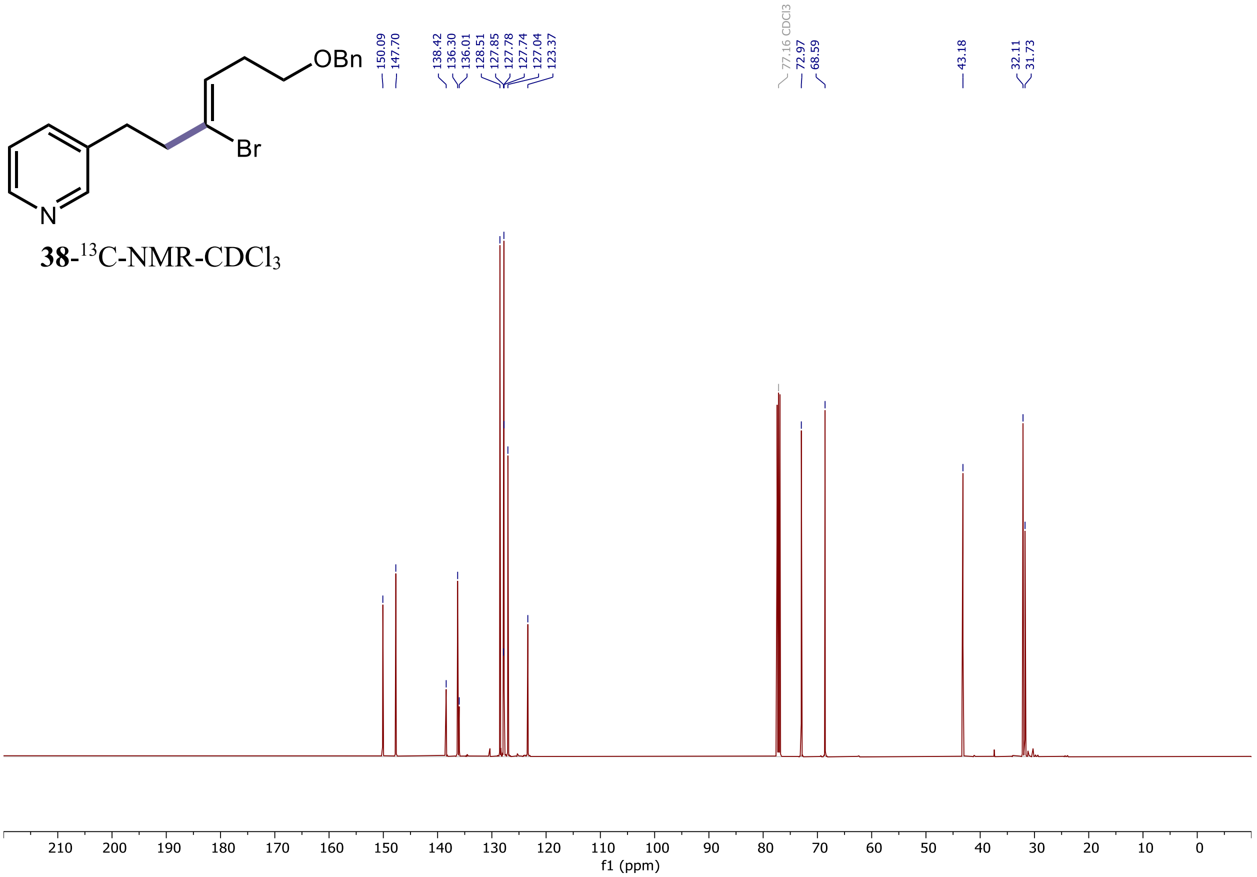

38-^{13}C-NMR-CDCl$_3$

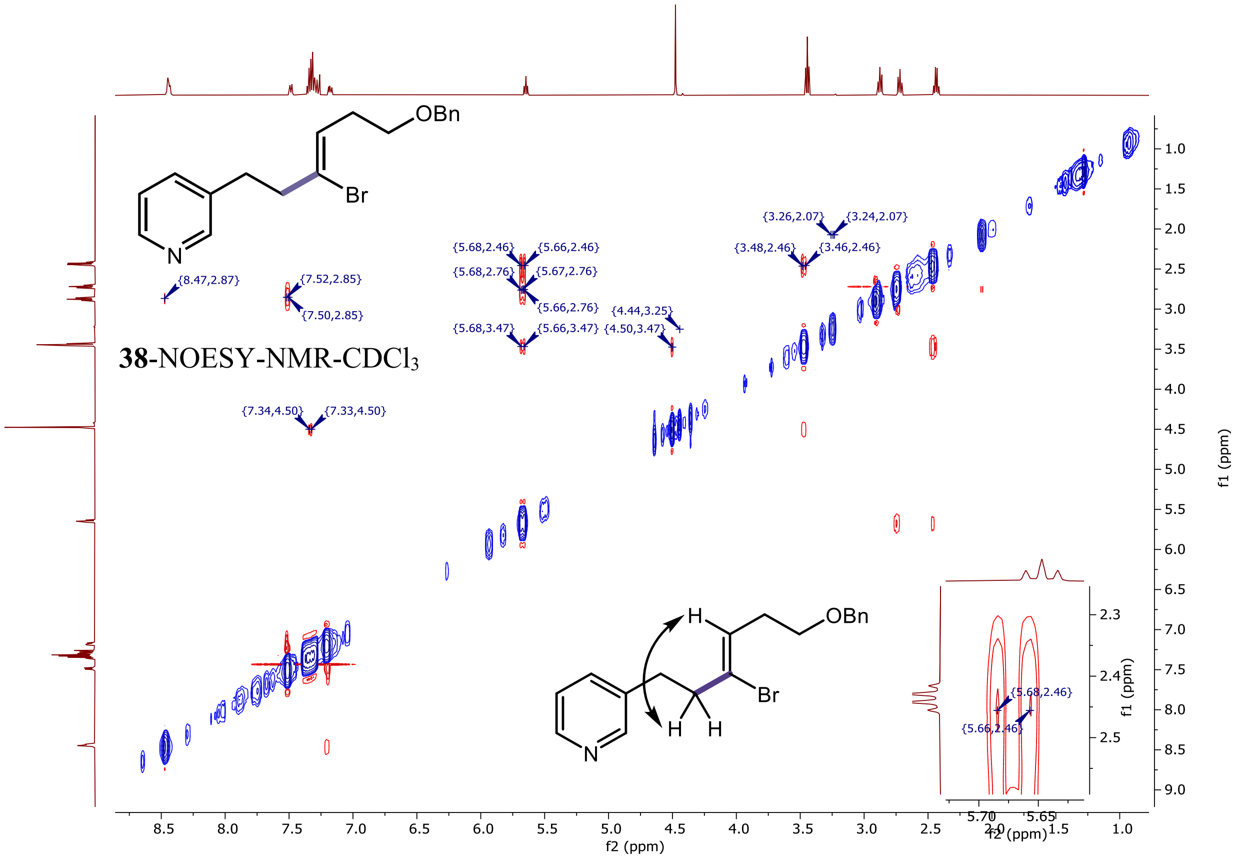

38-NOESY-NMR-CDCl$_3$

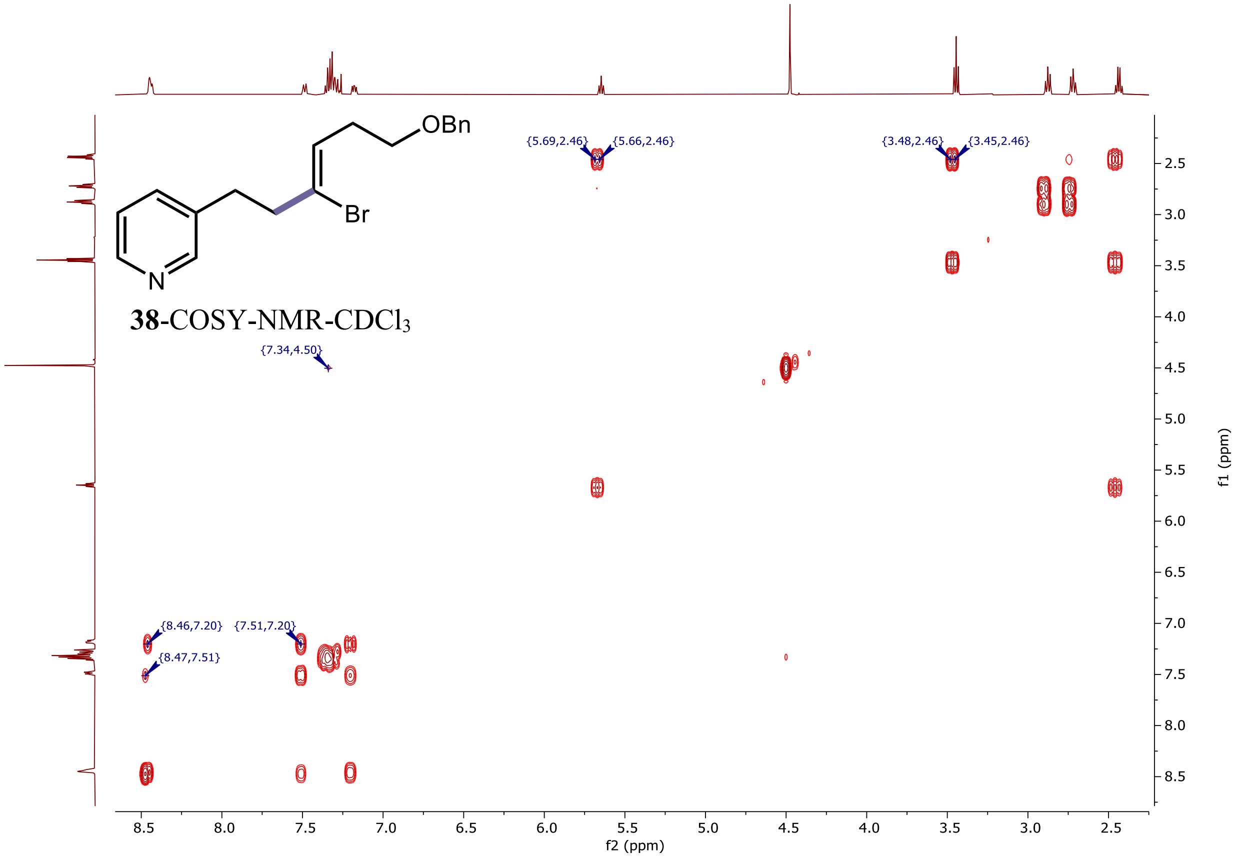
OBn
Br
N
38-COSY-NMR-CDCl3
{5.69,2.46}
{5.66,2.46}
{3.48,2.46}
{3.45,2.46}
{7.34,4.50}
{8.46,7.20}
{7.51,7.20}
{8.47,7.51}
f2 (ppm)
f1 (ppm)

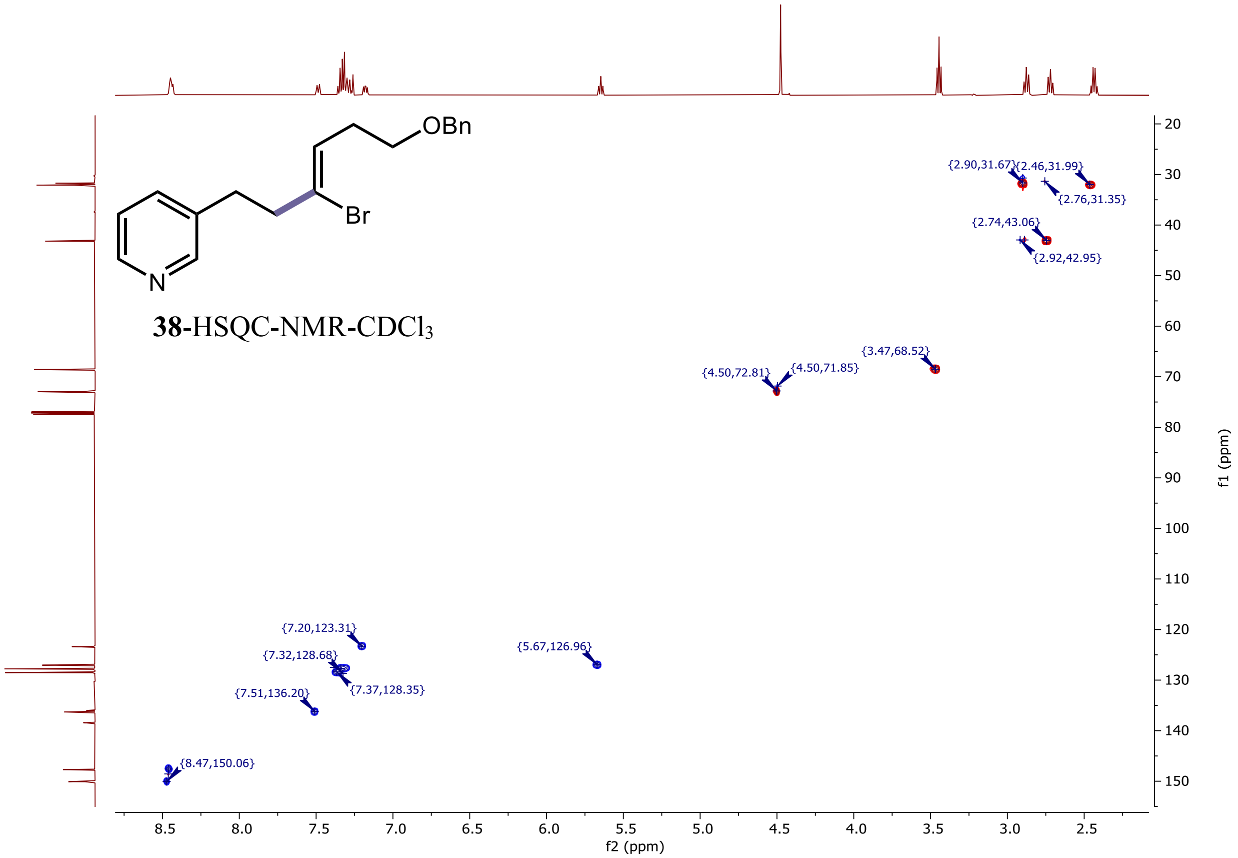
OBn
Br
N
38-HSQC-NMR-CDCl3
{2.90,31.67}
{2.46,31.99}
{2.76,31.35}
{2.74,43.06}
{2.92,42.95}
{3.47,68.52}
{4.50,72.81}
{4.50,71.85}
{7.20,123.31}
{7.32,128.68}
{7.37,128.35}
{5.67,126.96}
{7.51,136.20}
{8.47,150.06}
f2 (ppm)
f1 (ppm)

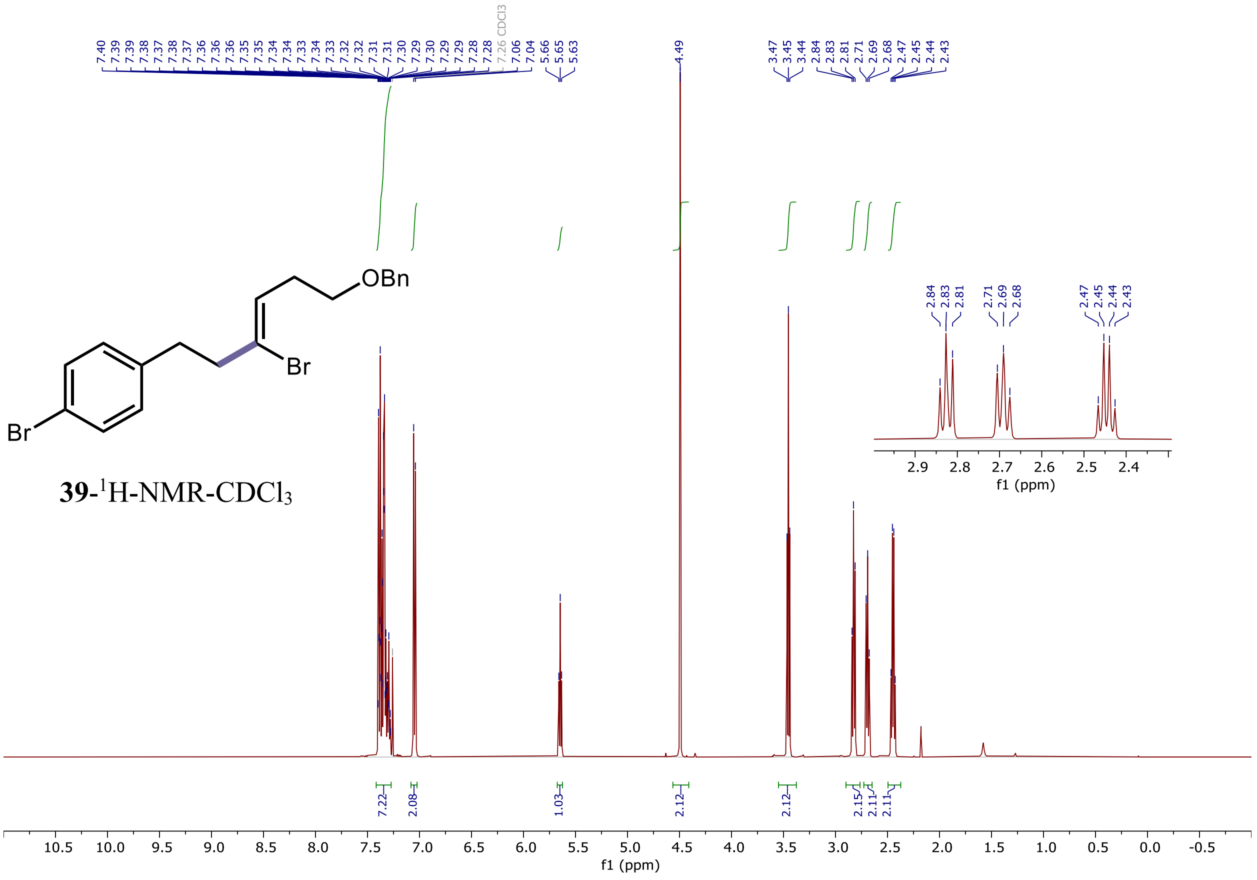

39-^{1}H-NMR-$CDCl_3$

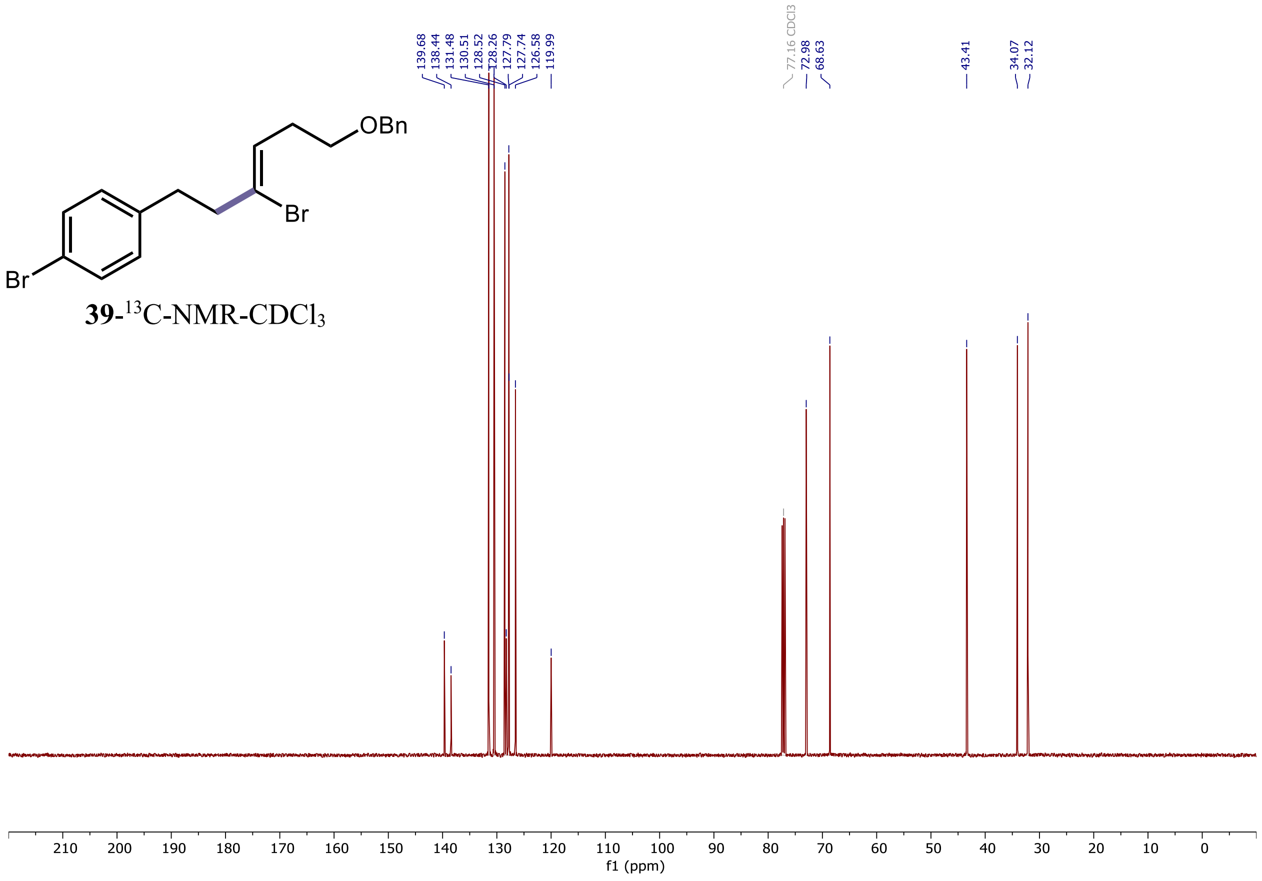

39-^{13}C-NMR-$CDCl_3$

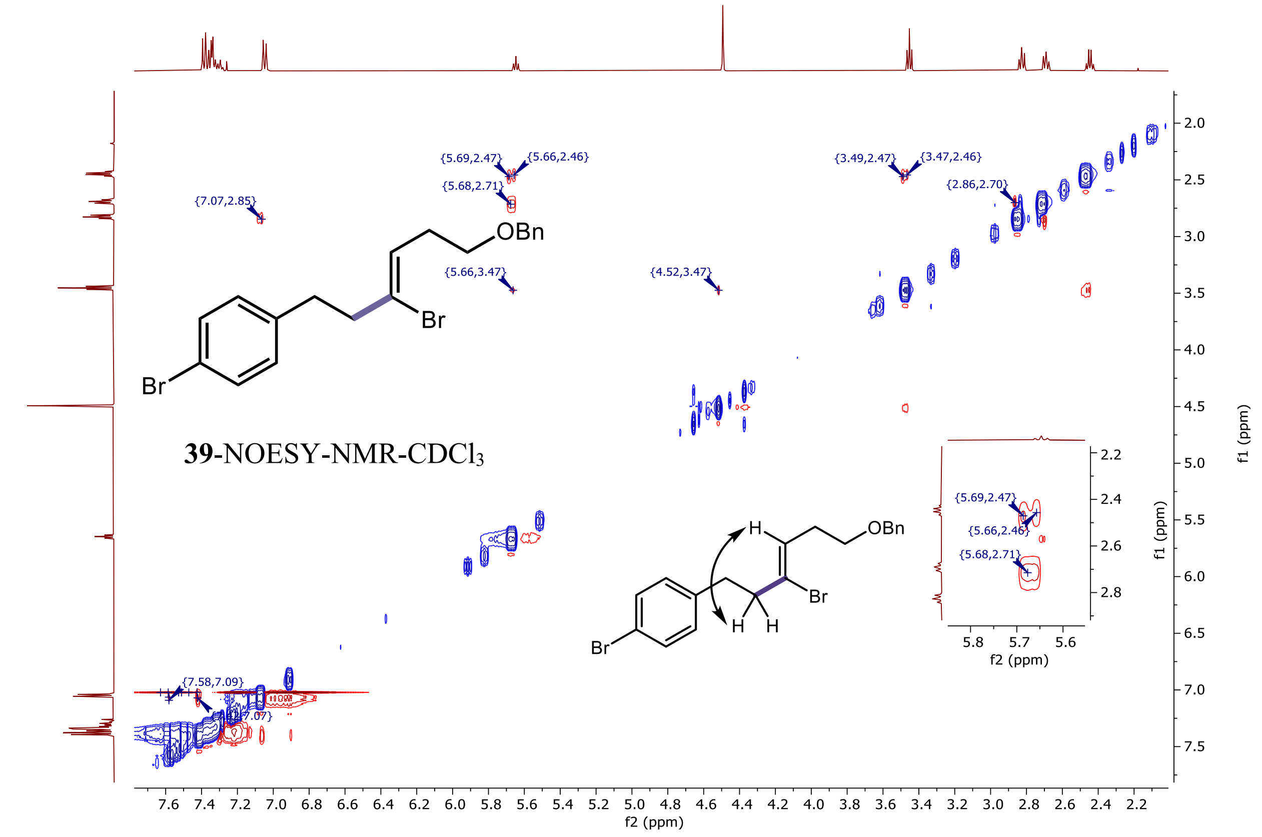

39-NOESY-NMR-CDCl$_3$

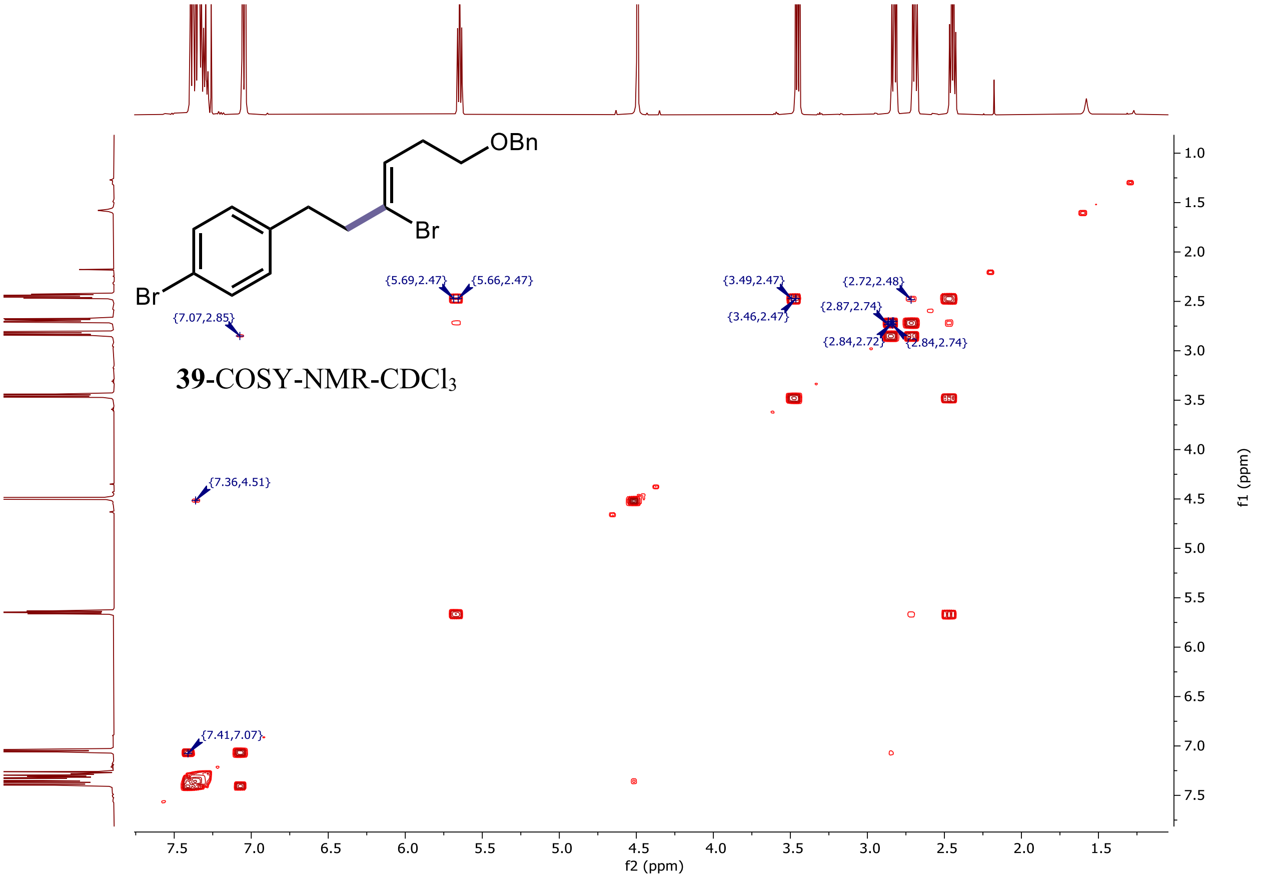

39-COSY-NMR-CDCl$_3$

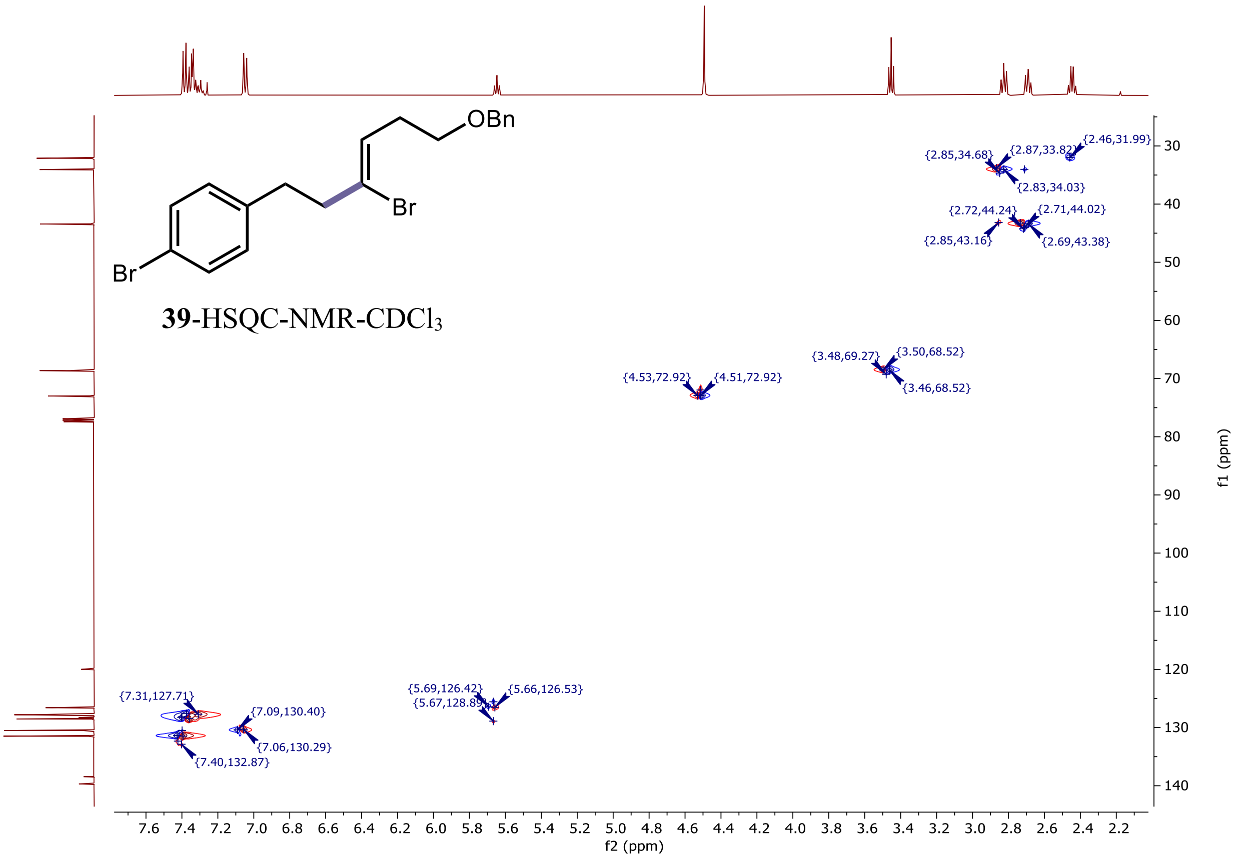

39-HSQC-NMR-$CDCl_3$

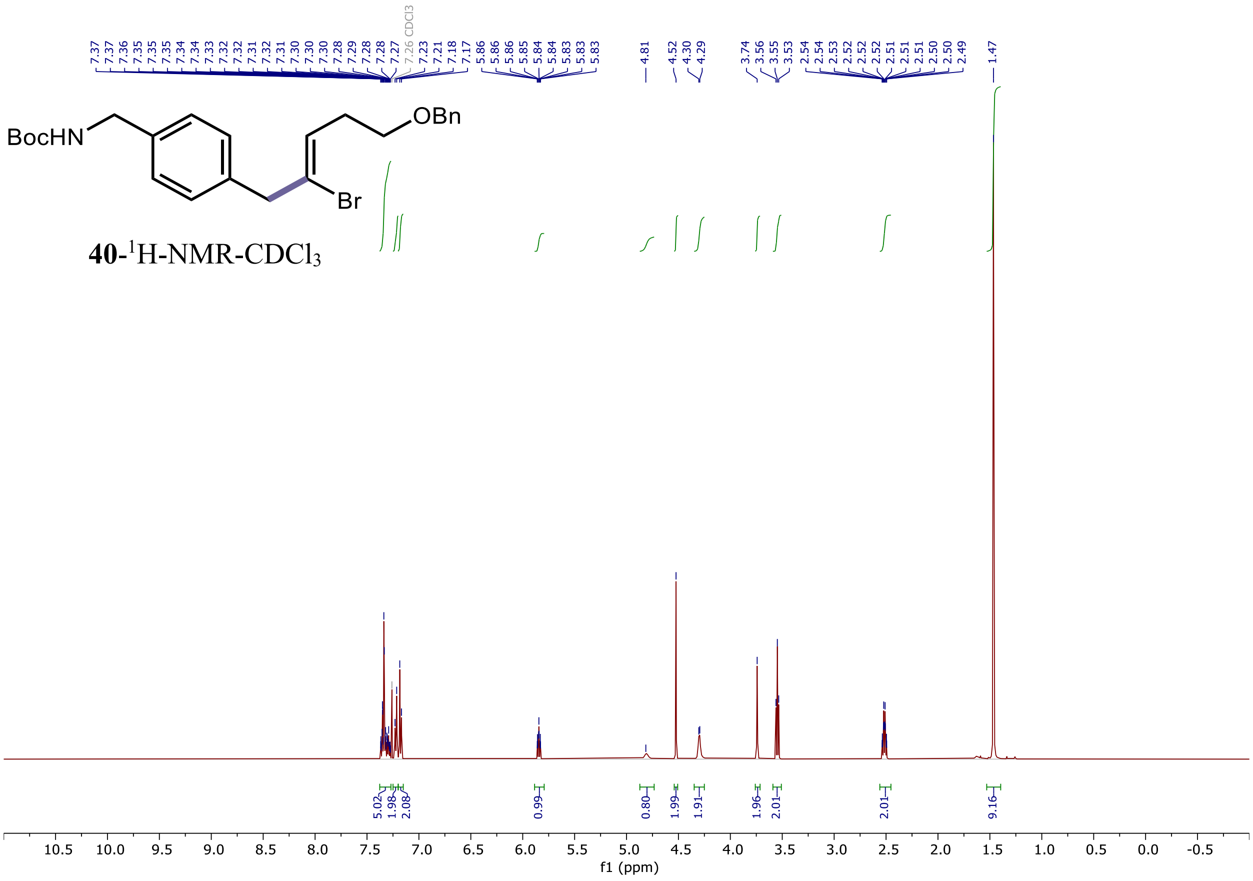

40-^{1}H-NMR-$CDCl_3$

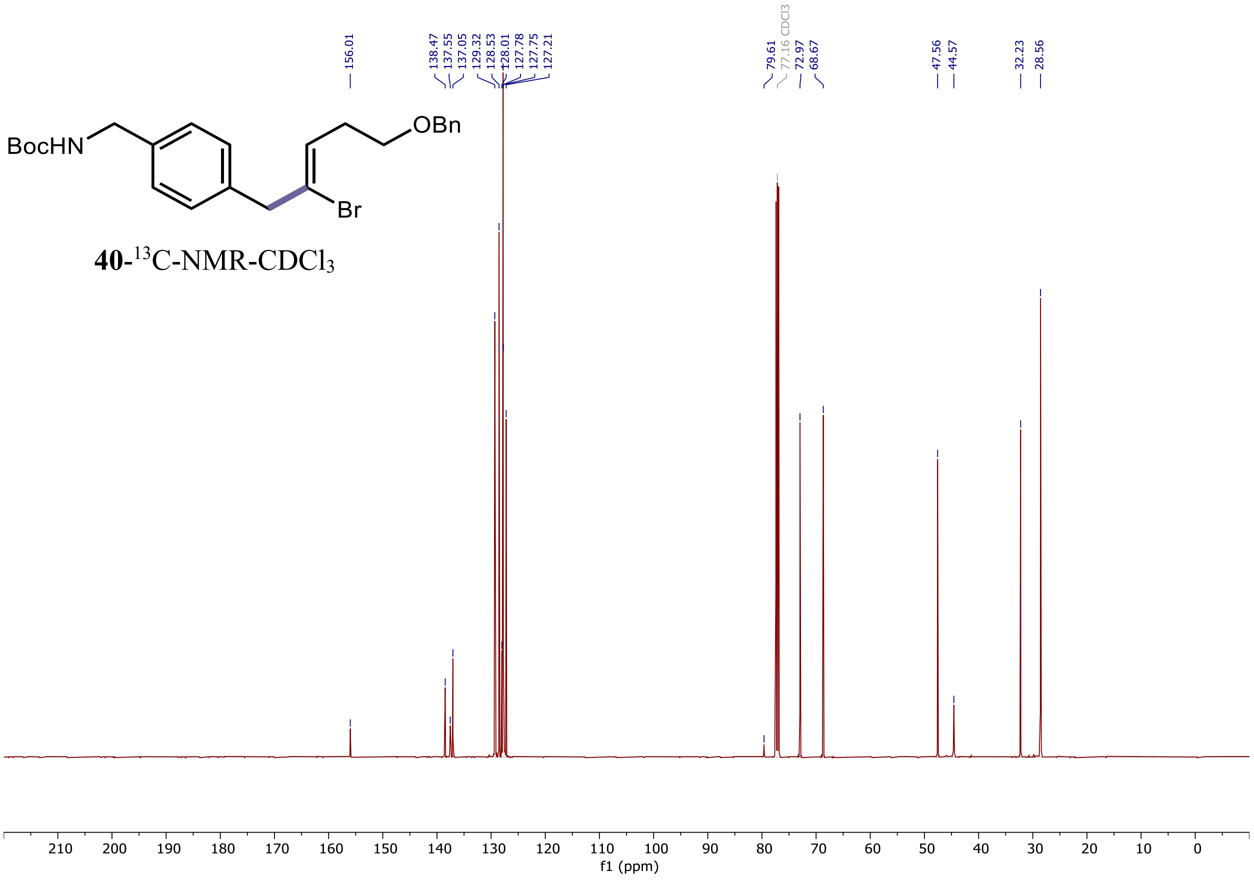
BocHN
OBn
Br
40-13C-NMR-CDCl3
156.01
138.47
137.55
137.05
129.32
128.53
128.01
127.78
127.75
127.21
79.61
77.16 CDCl3
72.97
68.67
47.56
44.57
32.23
28.56
f1 (ppm)

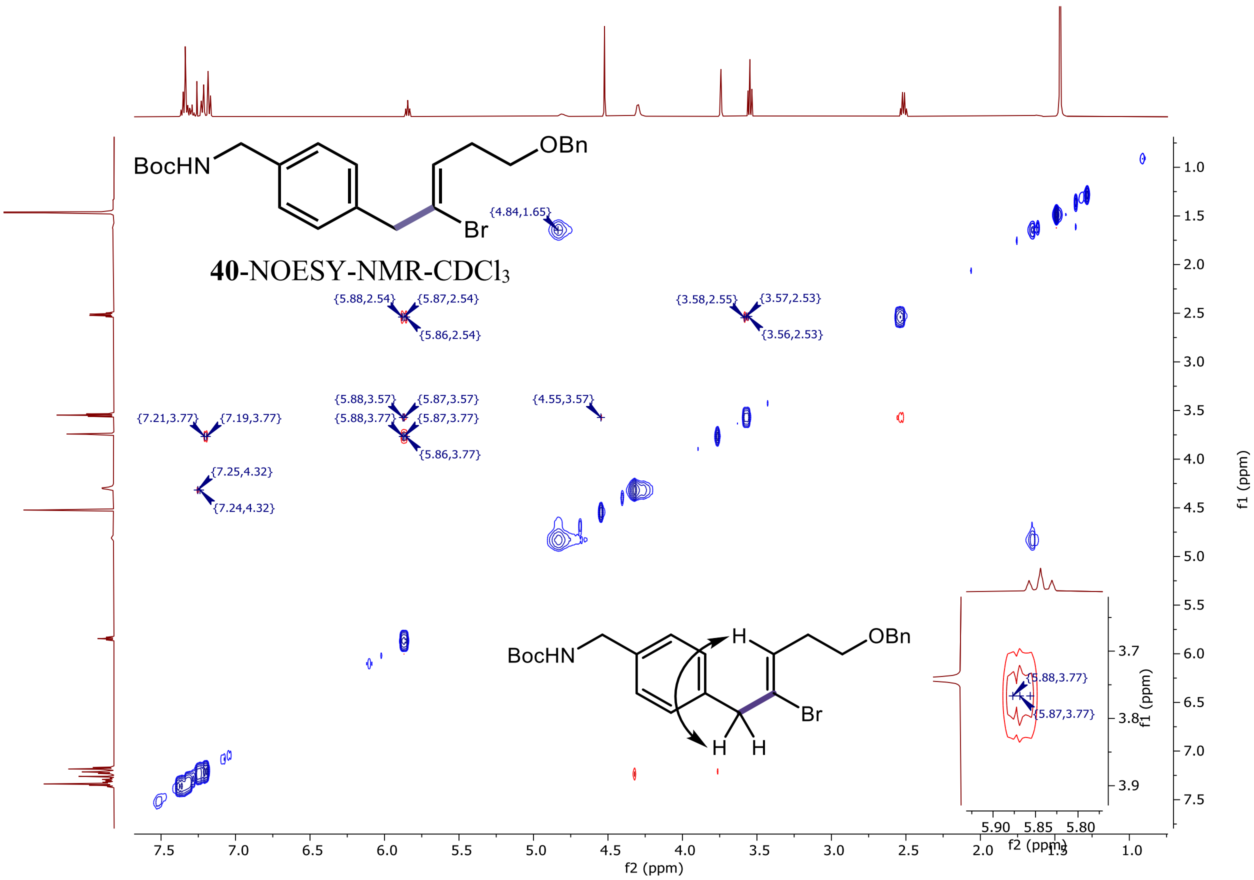
BocHN
OBn
Br
40-NOESY-NMR-CDCl3
{4.84,1.65}
{5.88,2.54}
{5.87,2.54}
{5.86,2.54}
{3.58,2.55}
{3.57,2.53}
{3.56,2.53}
{5.88,3.57}
{5.87,3.57}
{5.88,3.77}
{5.87,3.77}
{5.86,3.77}
{4.55,3.57}
{7.21,3.77}
{7.19,3.77}
{7.25,4.32}
{7.24,4.32}
H
H
H
f2 (ppm)
f1 (ppm)

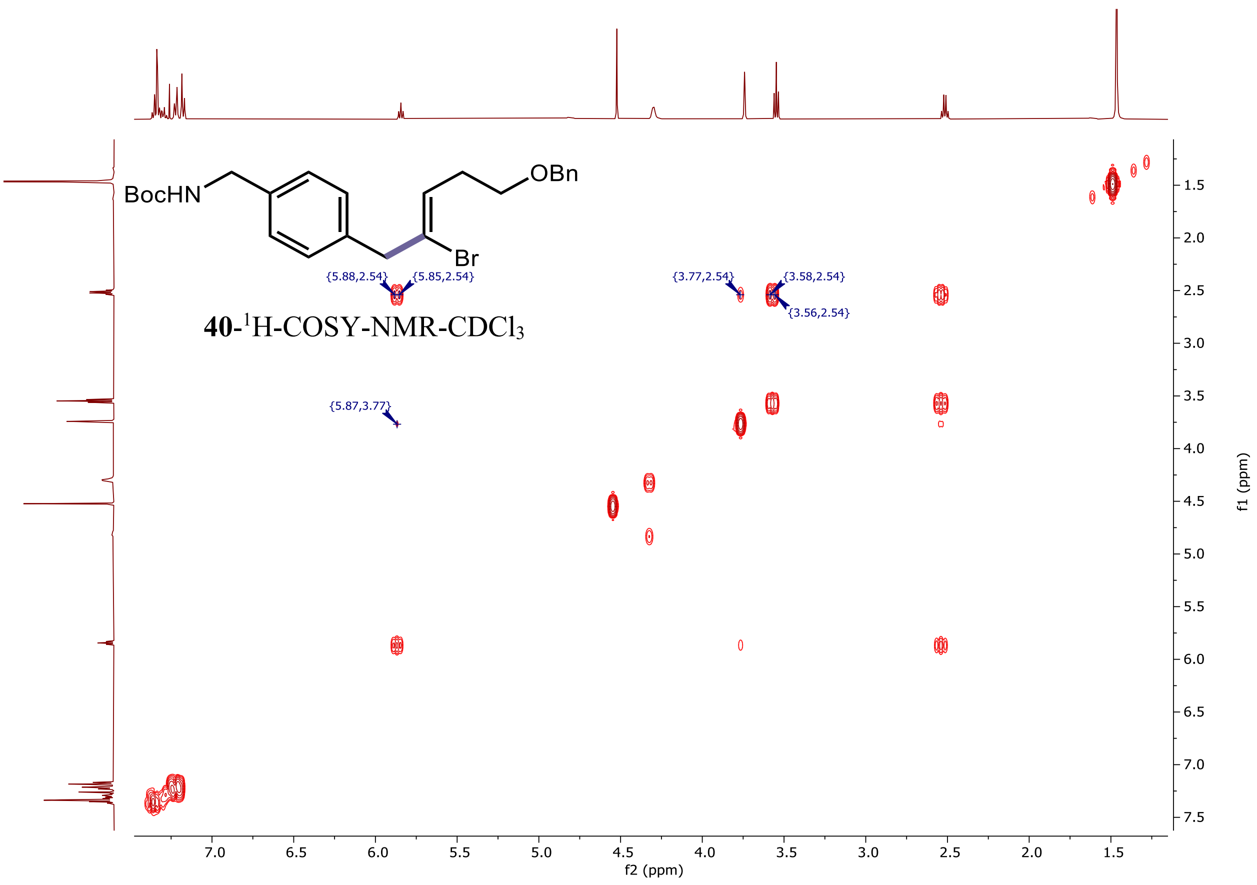

BocHN
OBn
Br
40-¹H-COSY-NMR-CDCl3
{5.88,2.54}
{5.85,2.54}
{3.77,2.54}
{3.58,2.54}
{3.56,2.54}
{5.87,3.77}
f2 (ppm)
f1 (ppm)

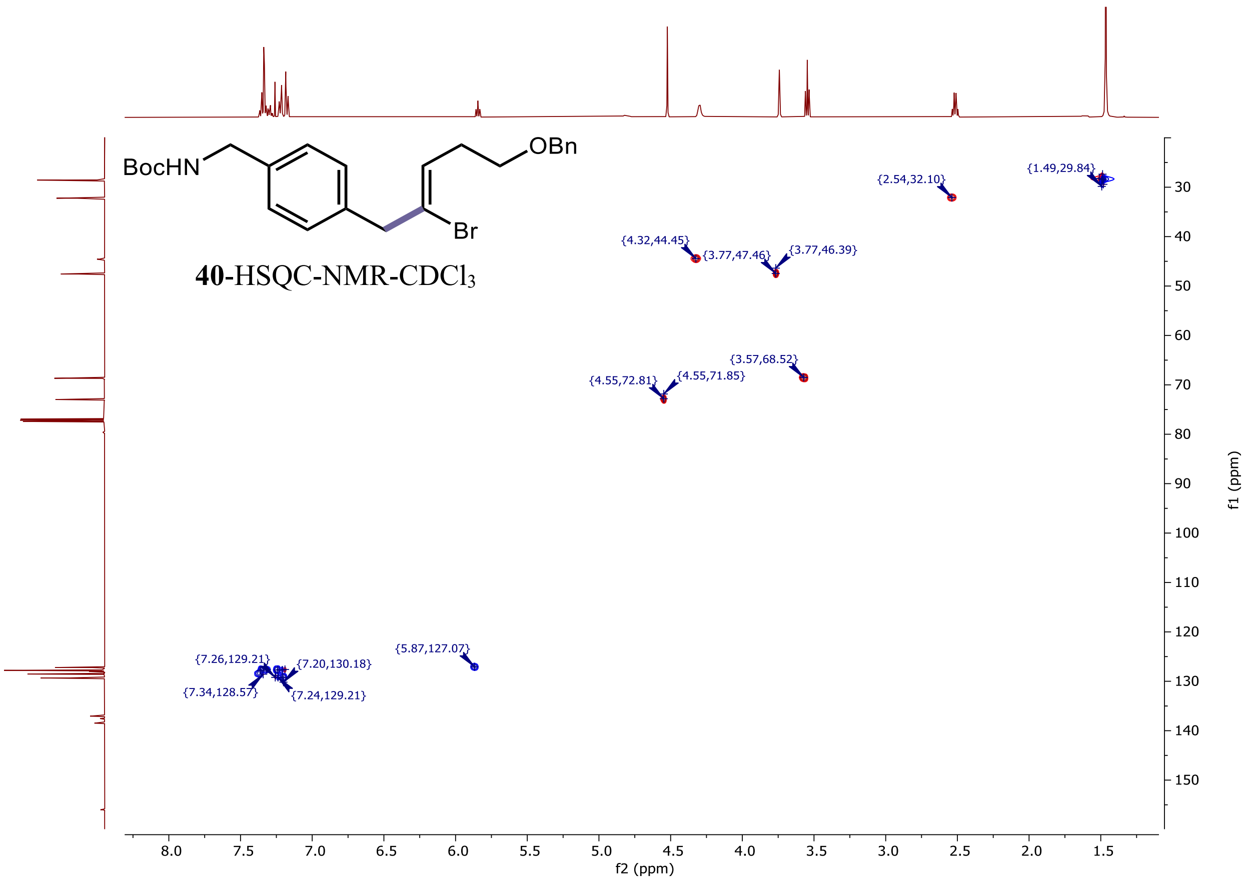

BocHN
OBn
Br
40-HSQC-NMR-CDCl3
{2.54,32.10}
{1.49,29.84}
{4.32,44.45}
{3.77,47.46}
{3.77,46.39}
{3.57,68.52}
{4.55,72.81}
{4.55,71.85}
{7.26,129.21}
{7.20,130.18}
{5.87,127.07}
{7.34,128.57}
{7.24,129.21}
f2 (ppm)
f1 (ppm)

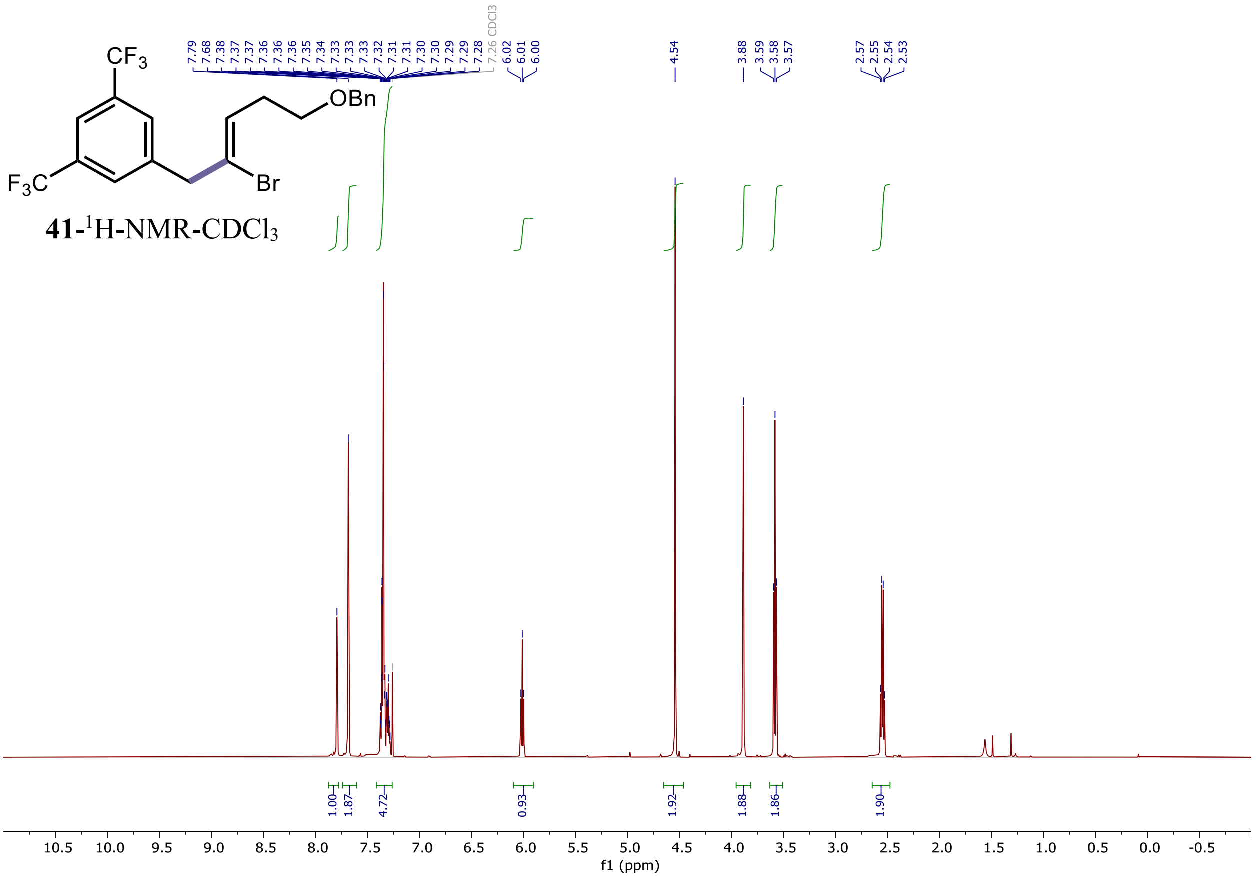
41-¹H-NMR-CDCl3
f1 (ppm)

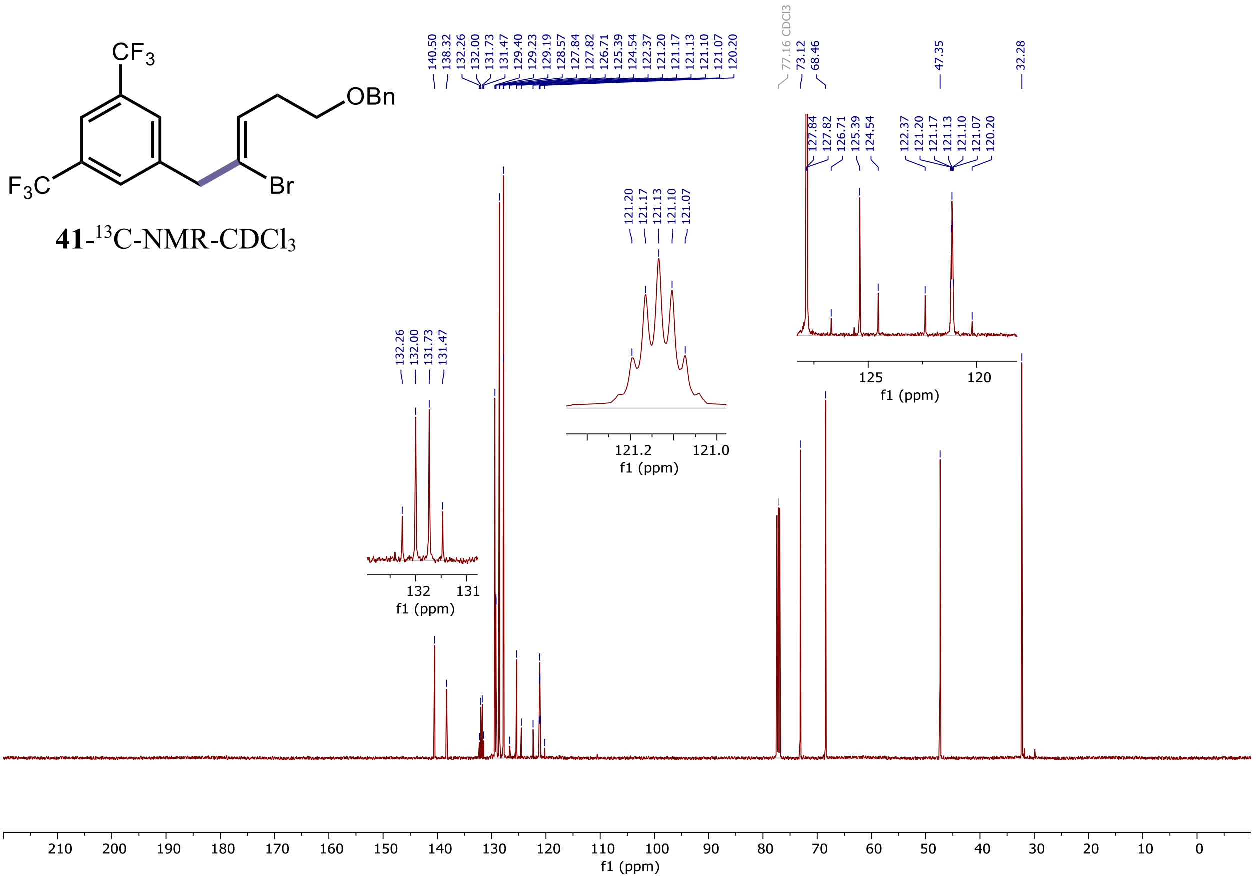
41-¹³C-NMR-CDCl3
f1 (ppm)

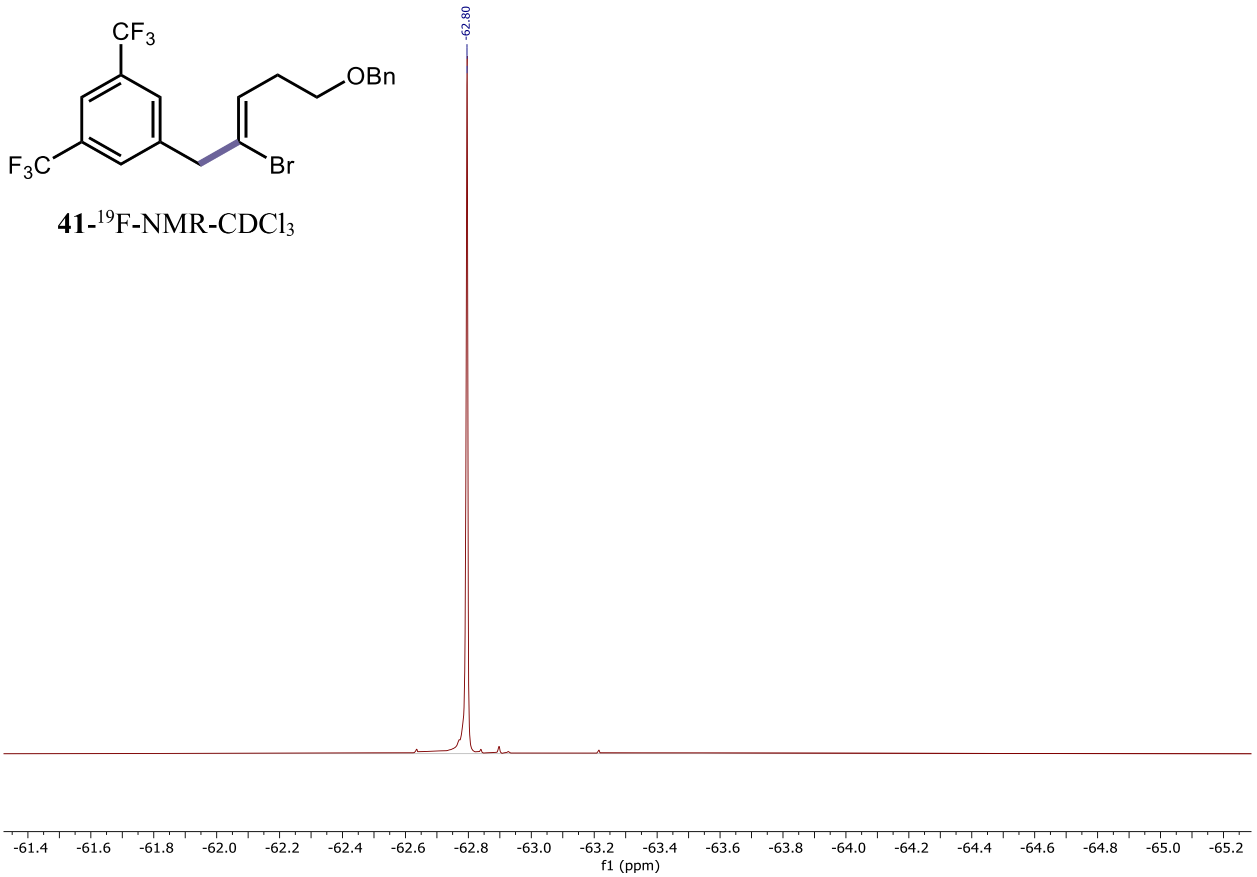
CF3
OBn
F3C
Br
41-19F-NMR-CDCl3
-62.80
f1 (ppm)

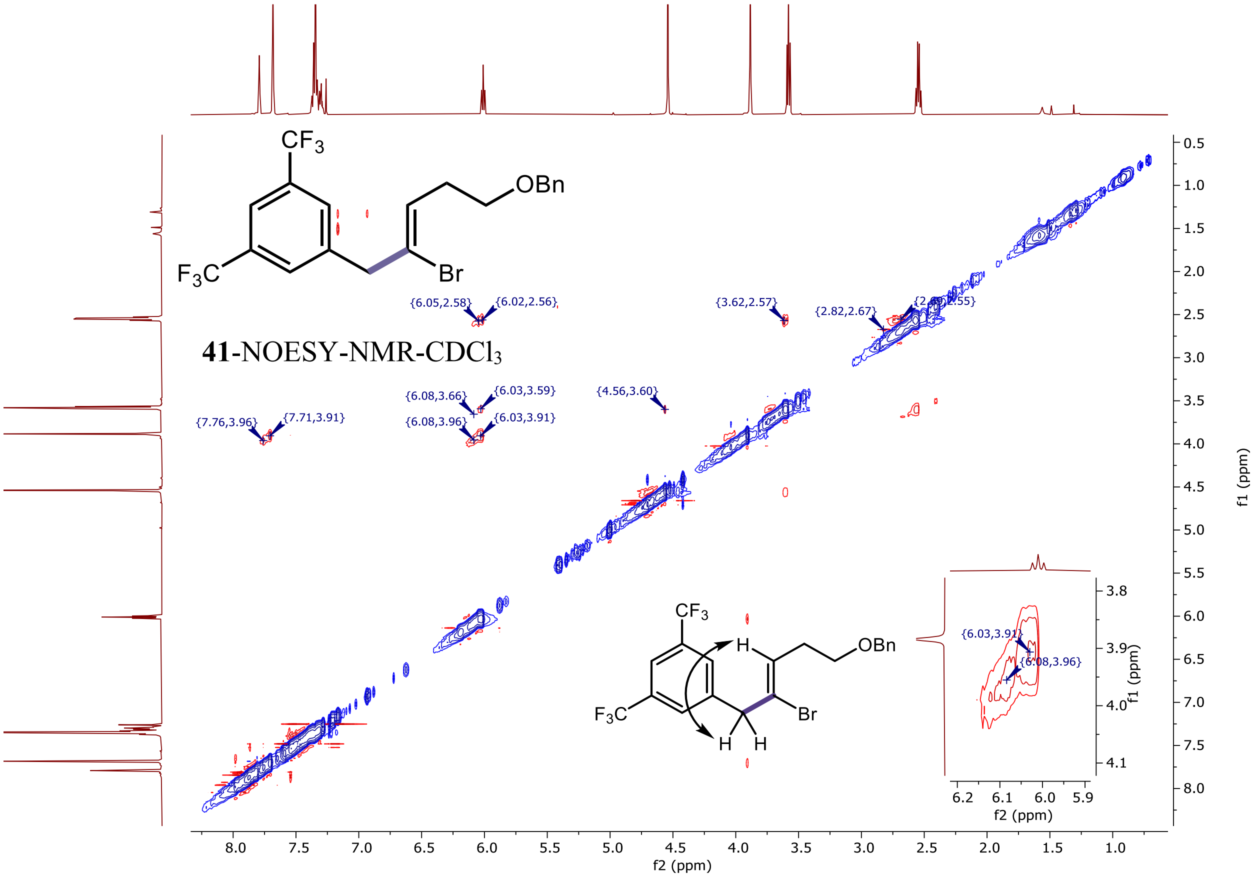
CF3
OBn
F3C
Br
41-NOESY-NMR-CDCl3
{6.05,2.58}
{6.02,2.56}
{3.62,2.57}
{2.82,2.67}
{6.08,3.66}
{6.03,3.59}
{4.56,3.60}
{7.76,3.96}
{7.71,3.91}
{6.08,3.96}
{6.03,3.91}
H
H
H
f2 (ppm)
f1 (ppm)

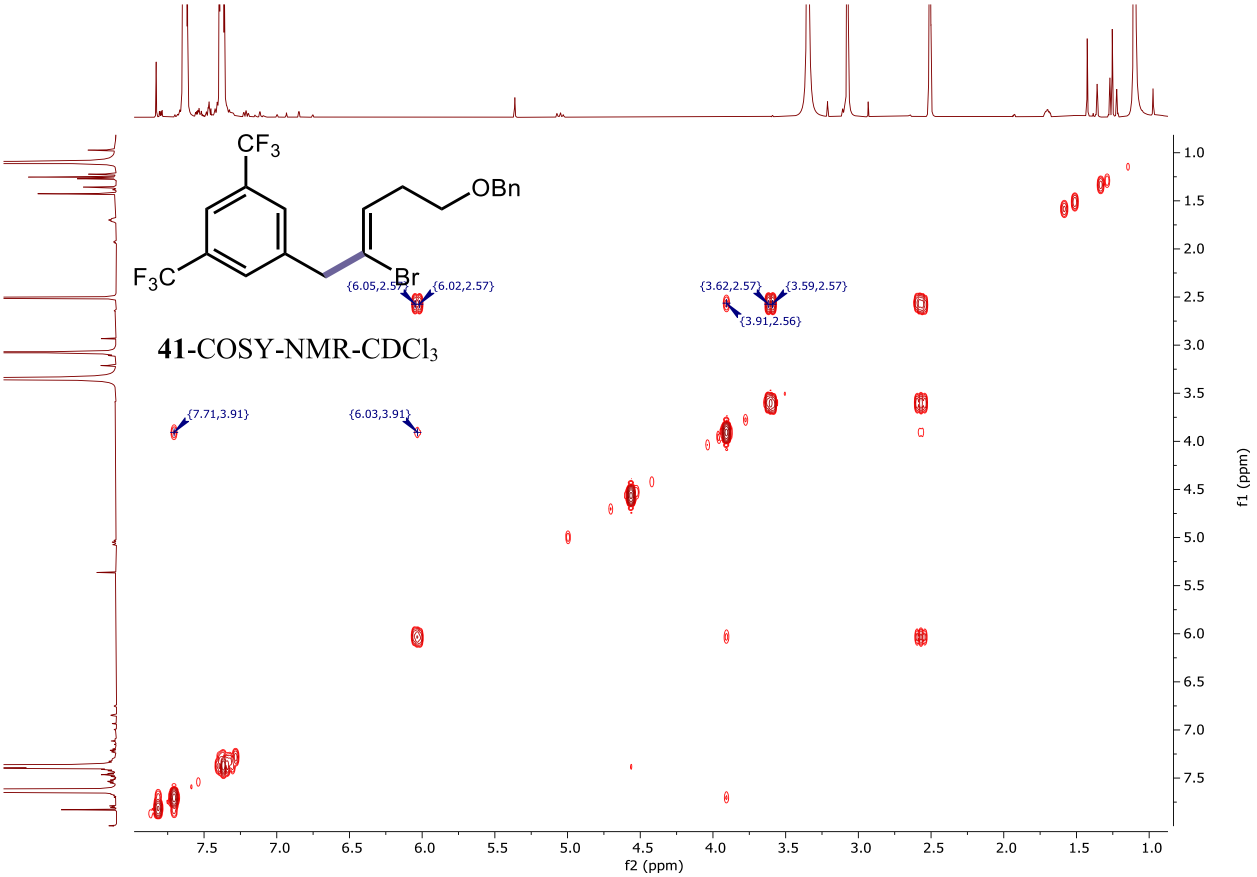
CF3
OBn
F3C
Br
41-COSY-NMR-CDCl3
{6.05,2.57}
{6.02,2.57}
{3.62,2.57}
{3.59,2.57}
{3.91,2.56}
{7.71,3.91}
{6.03,3.91}
f2 (ppm)
f1 (ppm)

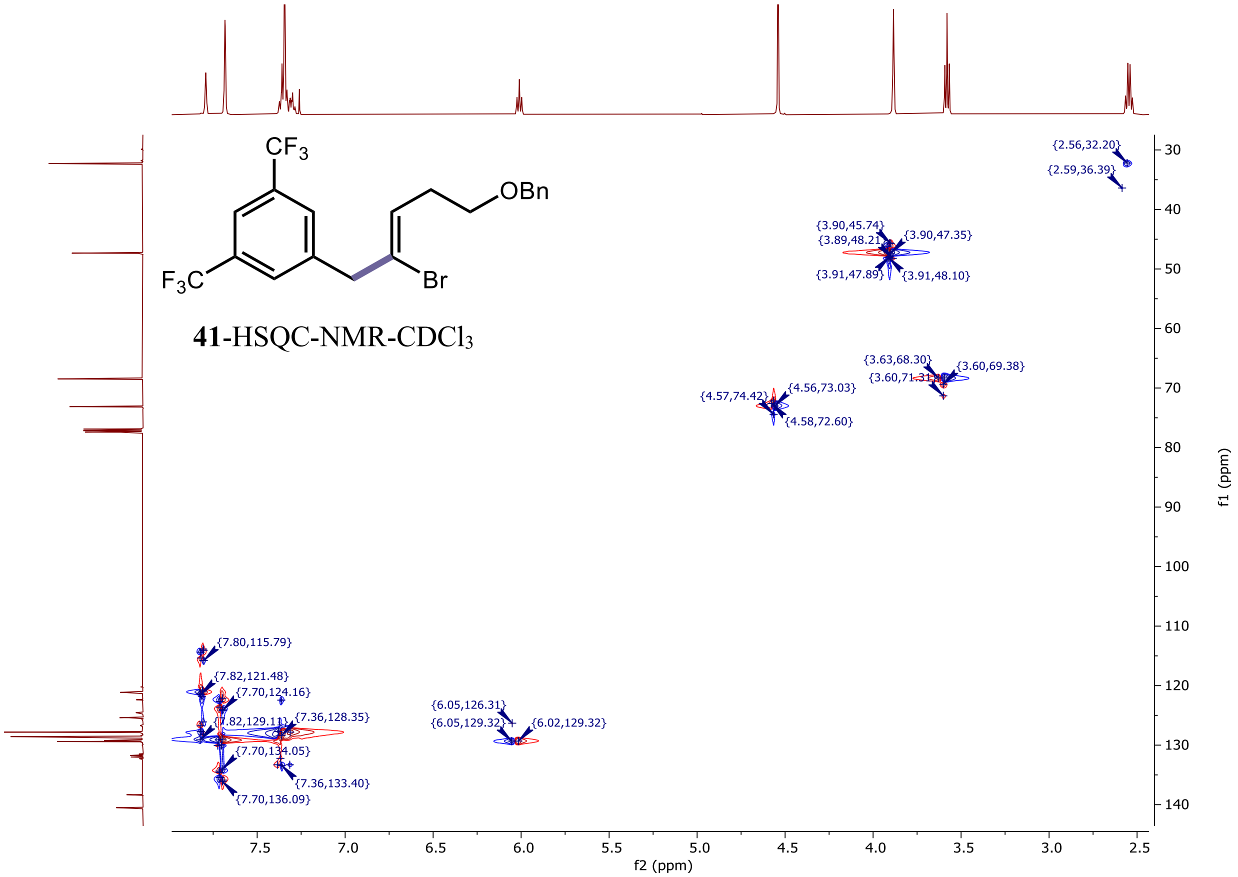
CF3
OBn
F3C
Br
41-HSQC-NMR-CDCl3
{2.56,32.20}
{2.59,36.39}
{3.90,45.74}
{3.89,48.21}
{3.90,47.35}
{3.91,47.89}
{3.91,48.10}
{3.63,68.30}
{3.60,69.38}
{4.56,73.03}
{4.57,74.42}
{4.58,72.60}
{7.80,115.79}
{7.82,121.48}
{7.70,124.16}
{7.36,128.35}
{6.05,126.31}
{6.05,129.32}
{6.02,129.32}
{7.70,134.05}
{7.36,133.40}
{7.70,136.09}
f2 (ppm)
f1 (ppm)

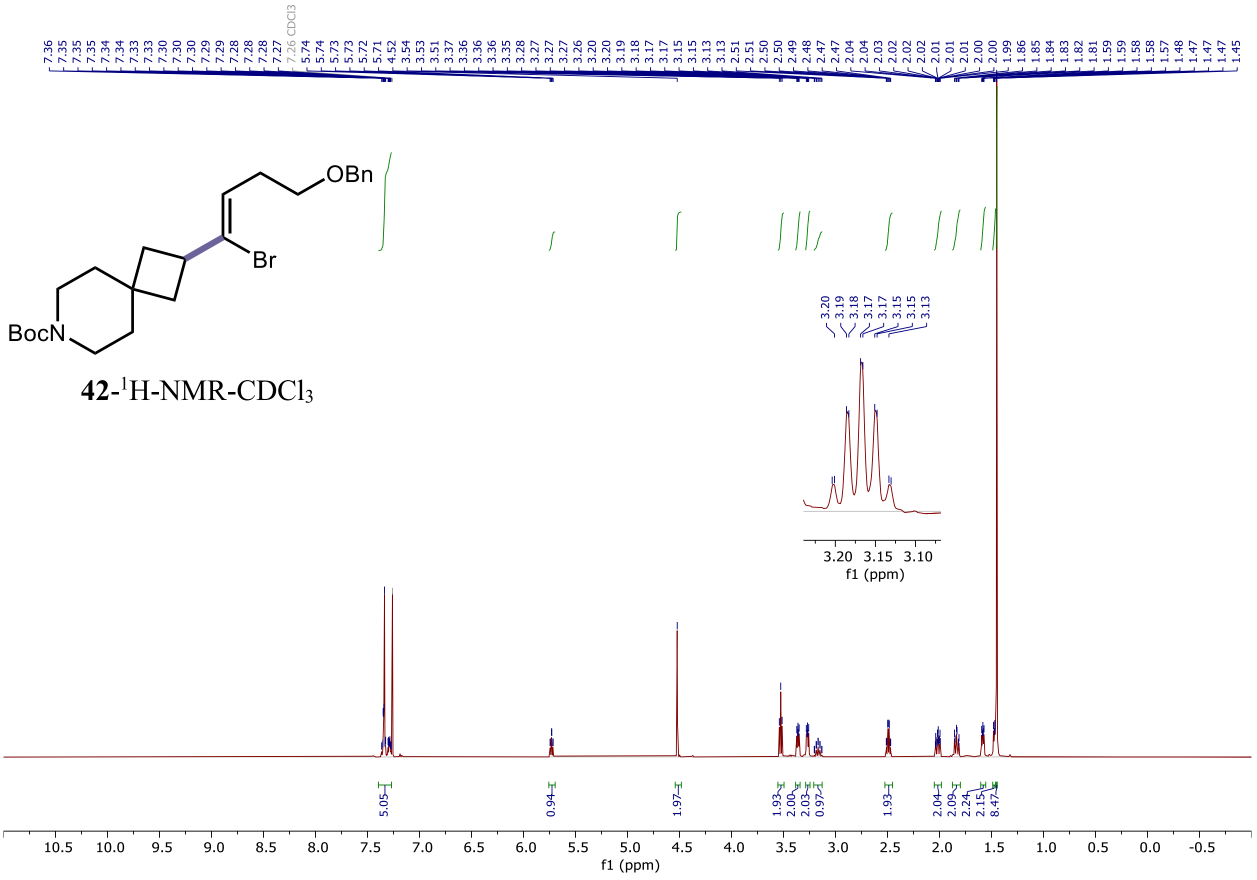

42-^{1}H-NMR-CDCl$_3$

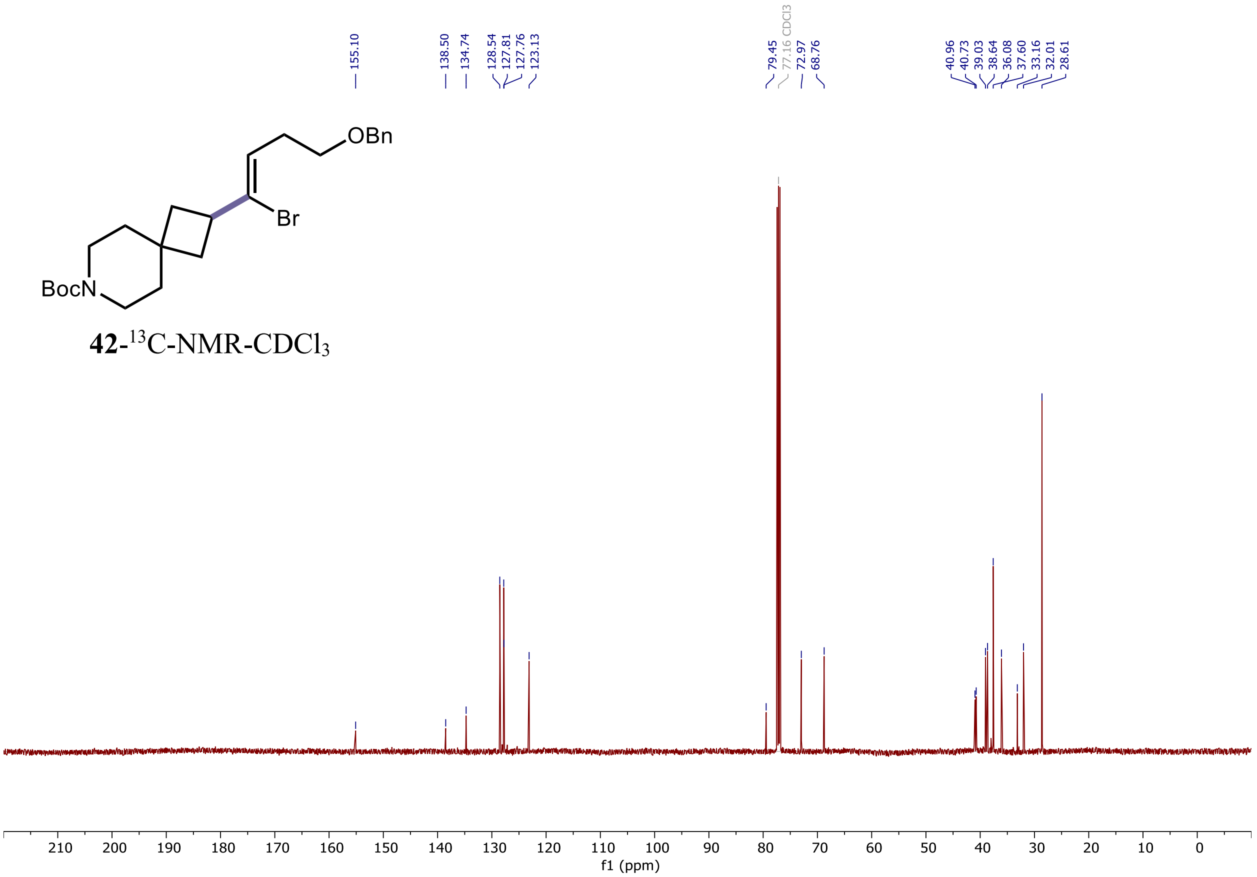

42-^{13}C-NMR-CDCl$_3$

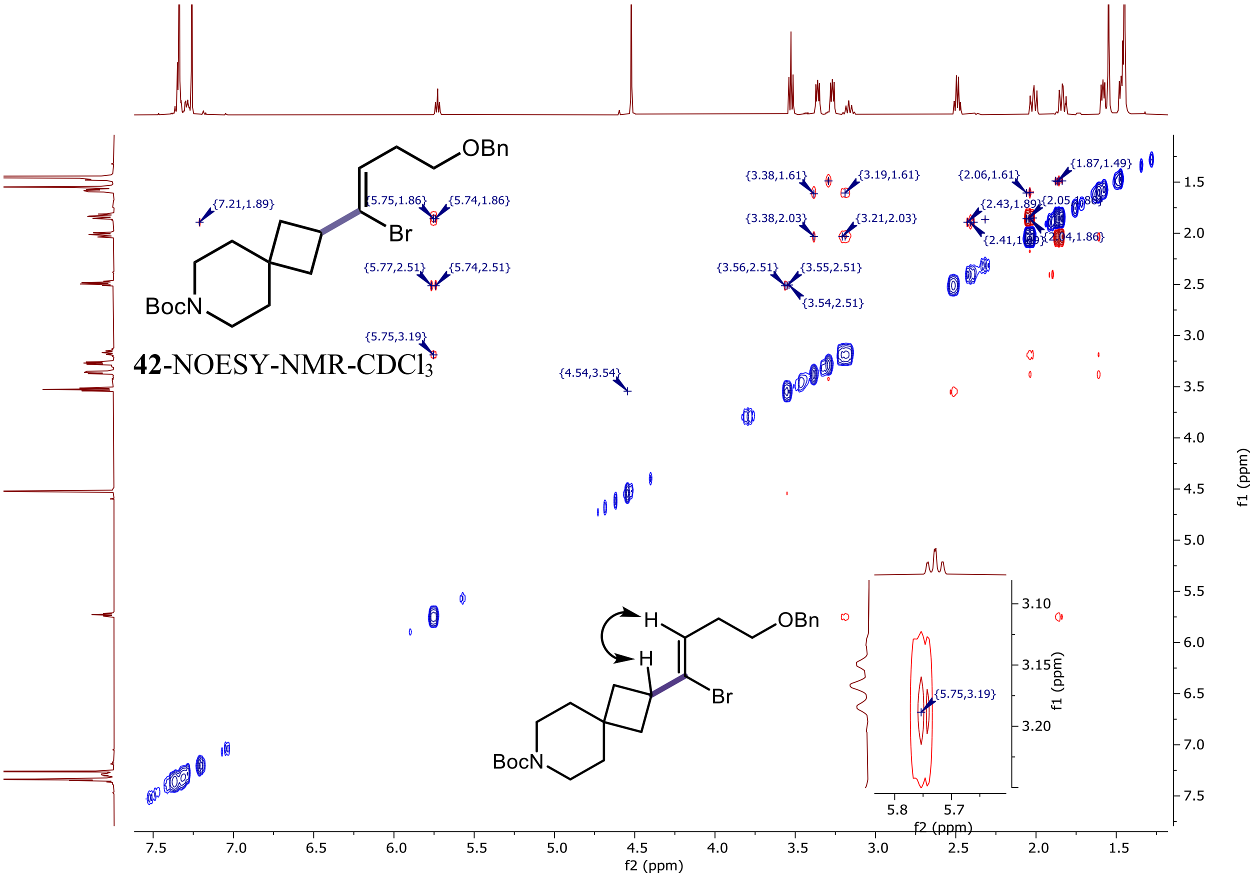
OBn
Br
BocN
42-NOESY-NMR-CDCl3
H
H
f1 (ppm)
f2 (ppm)

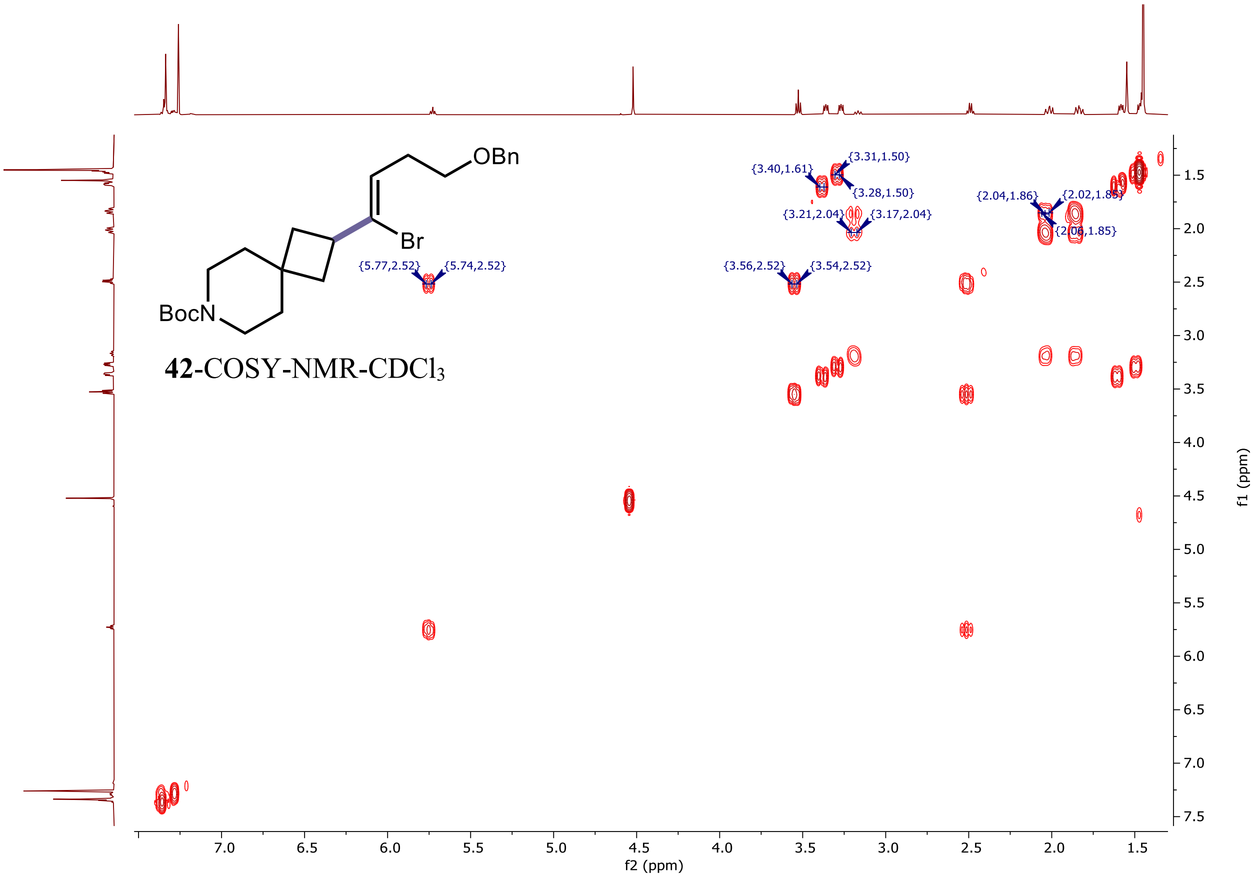
OBn
Br
BocN
42-COSY-NMR-CDCl3
f1 (ppm)
f2 (ppm)

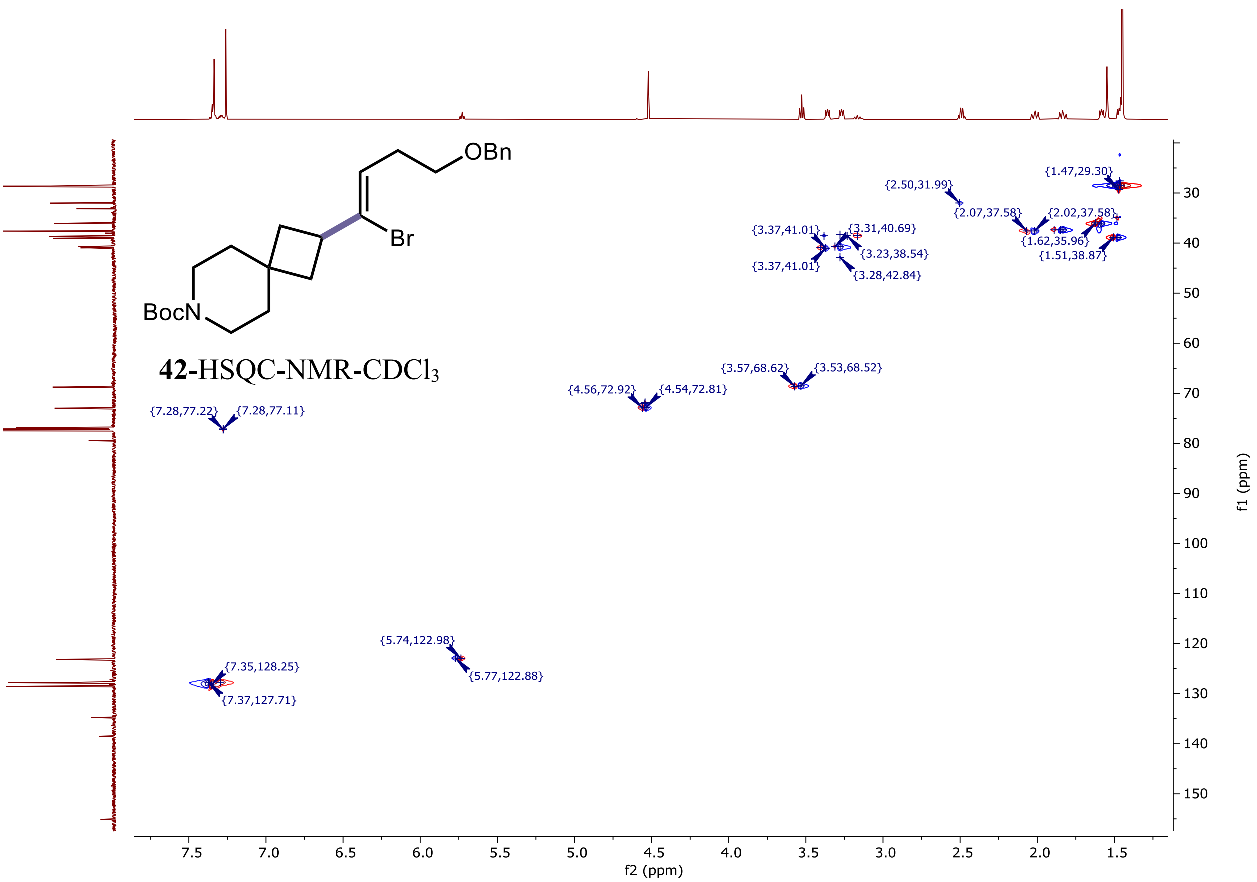
OBn
Br
BocN
42-HSQC-NMR-CDCl3
f2 (ppm)
f1 (ppm)

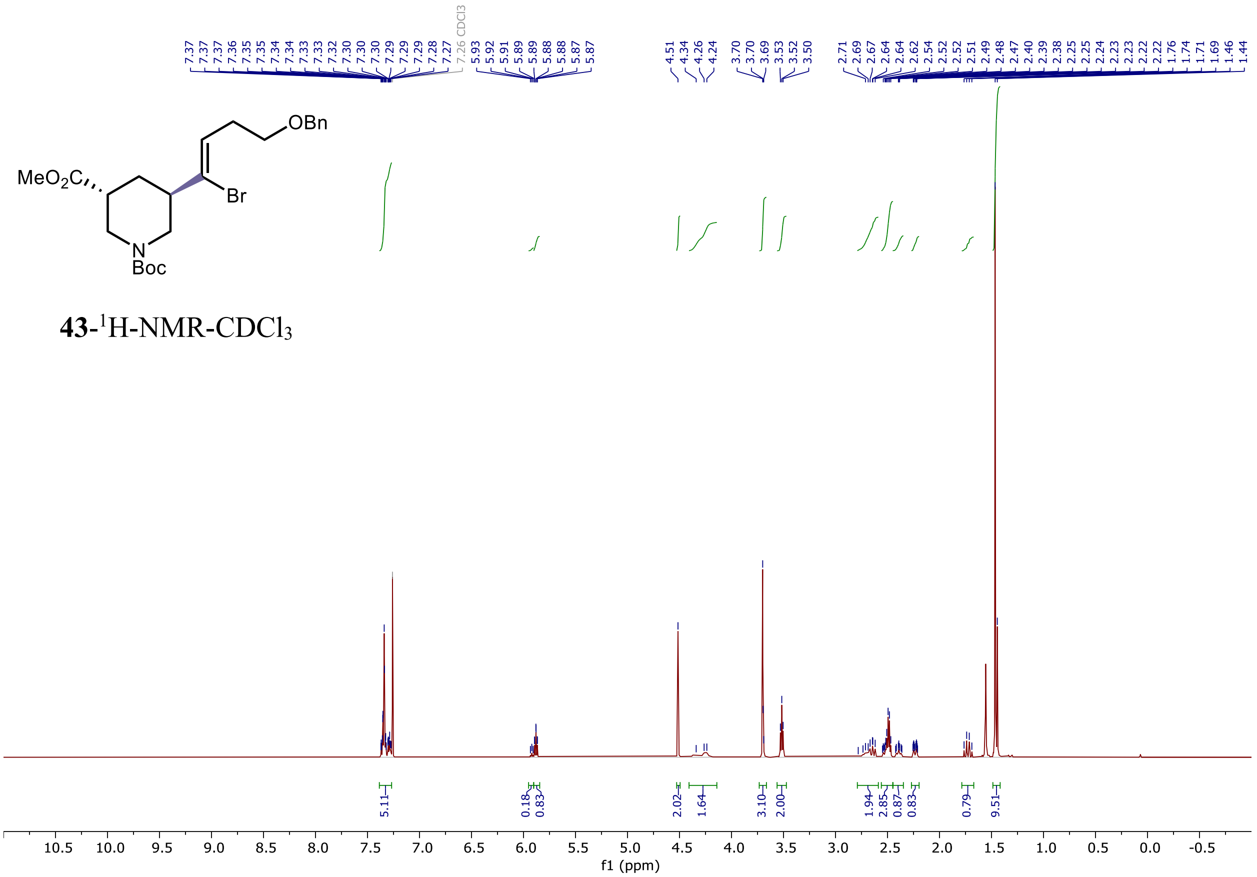
OBn
MeO2C
Br
N
Boc
43-1H-NMR-CDCl3
f1 (ppm)

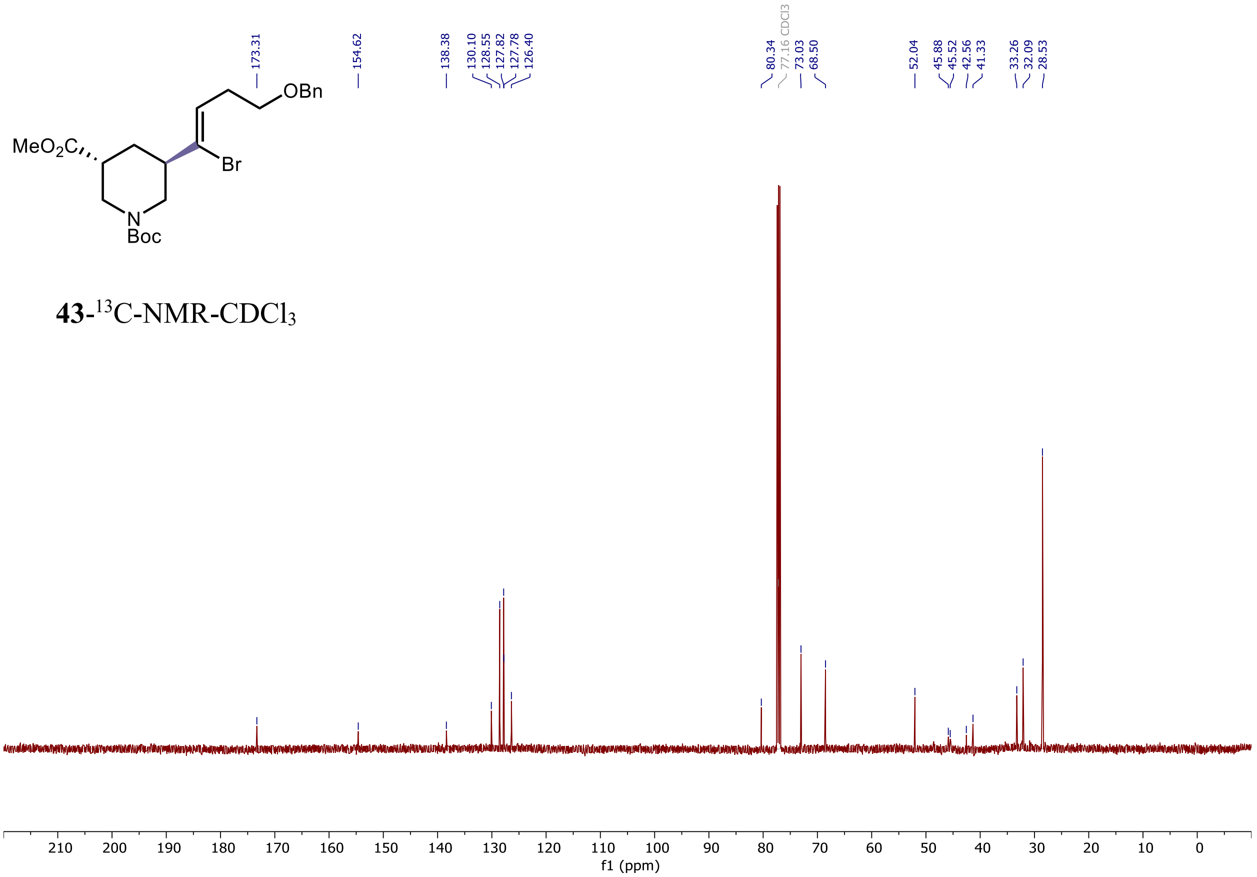

43-^{13}C-NMR-CDCl$_3$

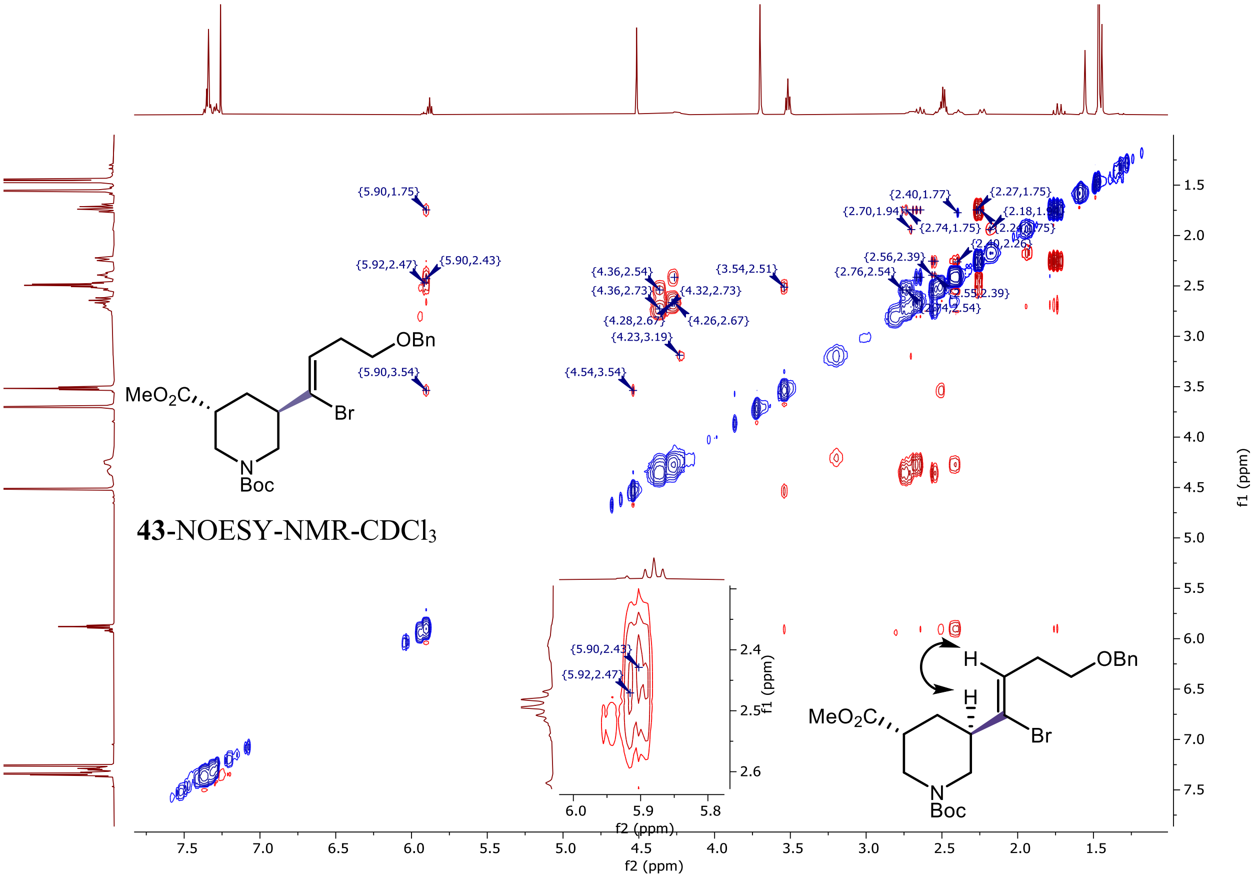

43-NOESY-NMR-CDCl$_3$

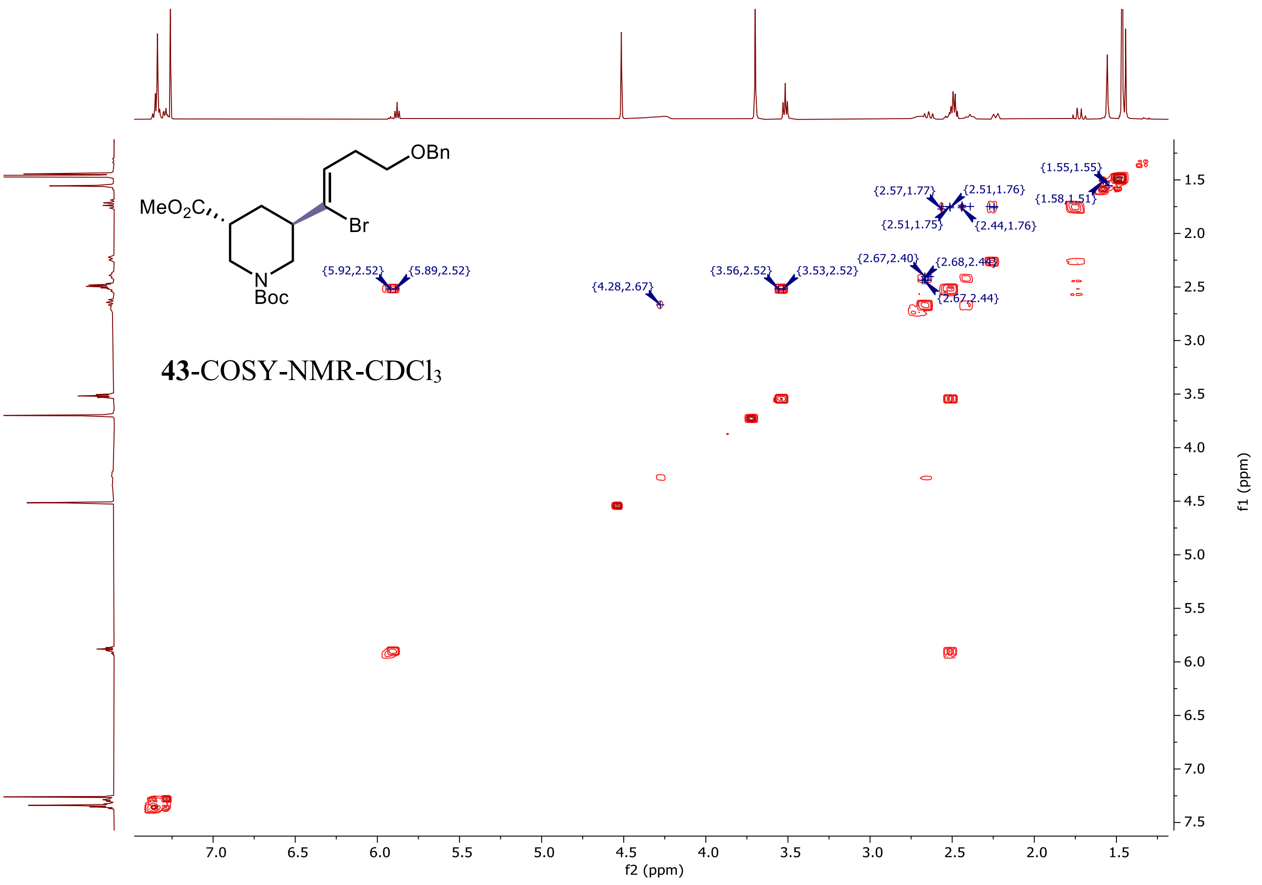
OBn
MeO2C
Br
N
Boc
43-COSY-NMR-CDCl3
f2 (ppm)
f1 (ppm)

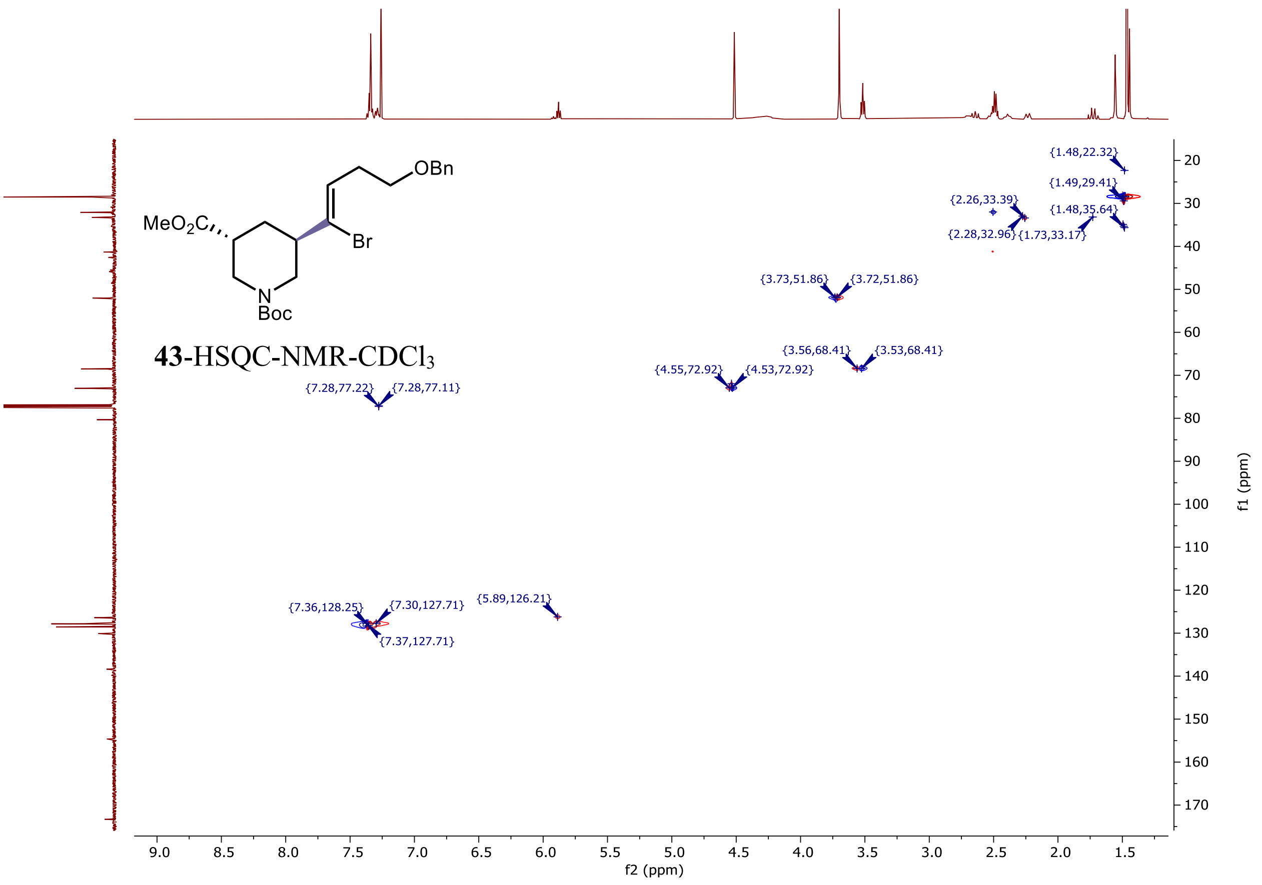
OBn
MeO2C
Br
N
Boc
43-HSQC-NMR-CDCl3
f2 (ppm)
f1 (ppm)

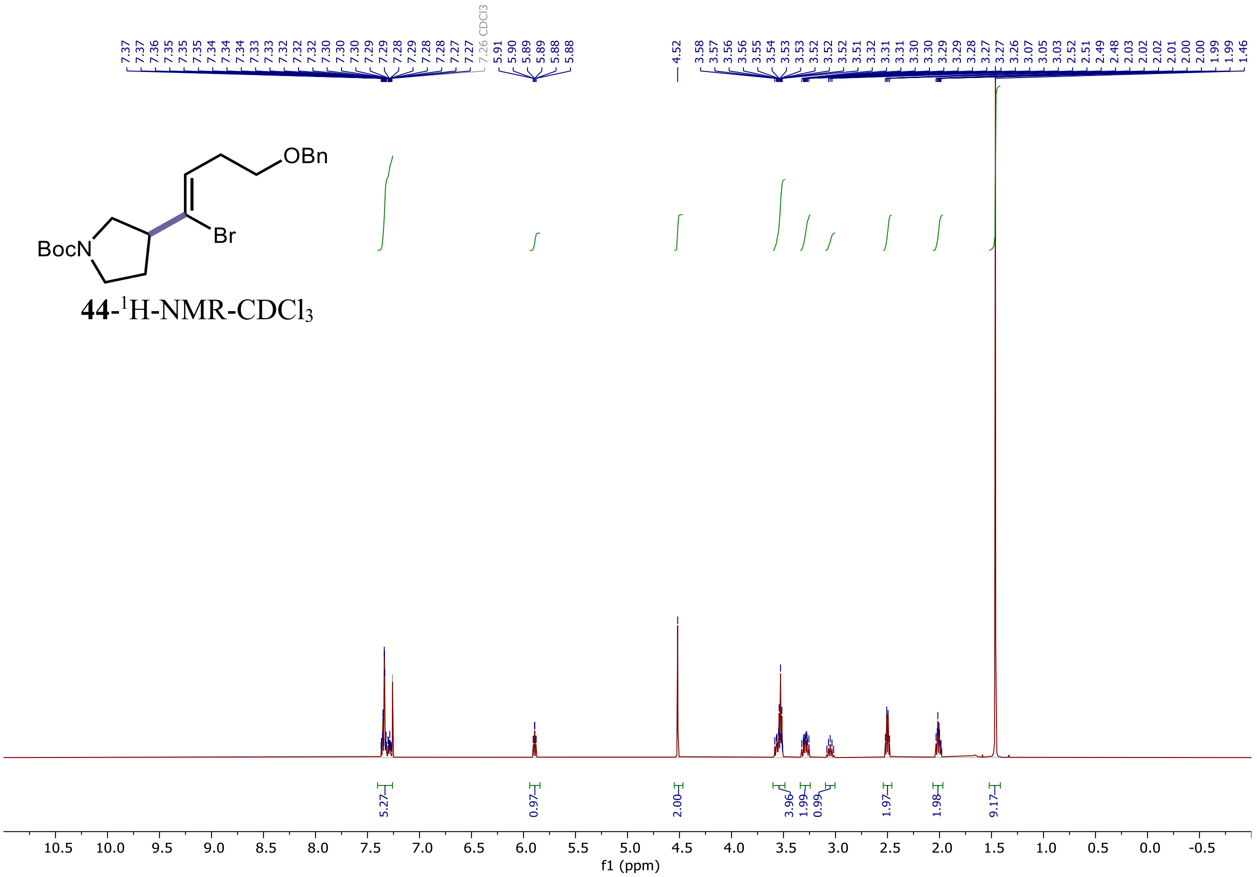

44-^{1}H-NMR-$CDCl_3$

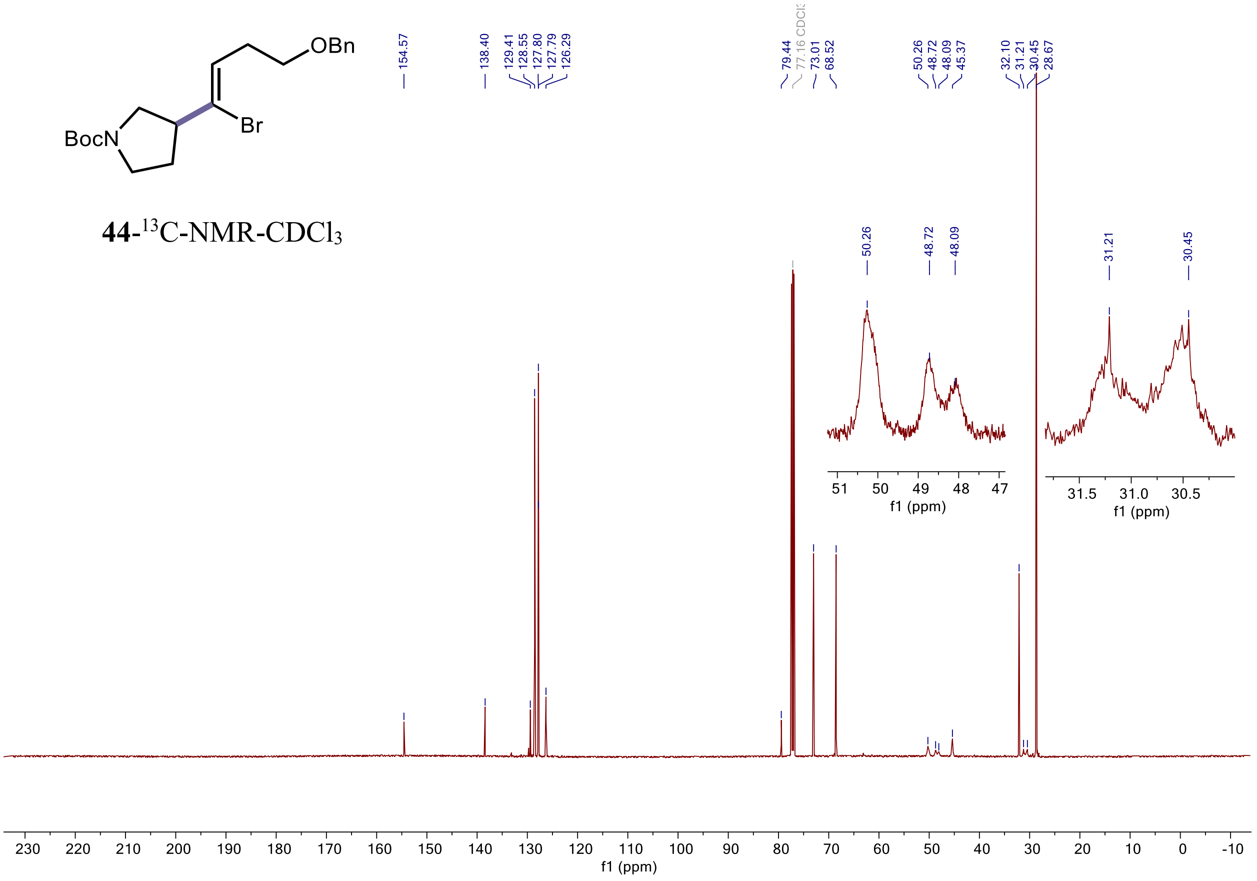

44-^{13}C-NMR-$CDCl_3$

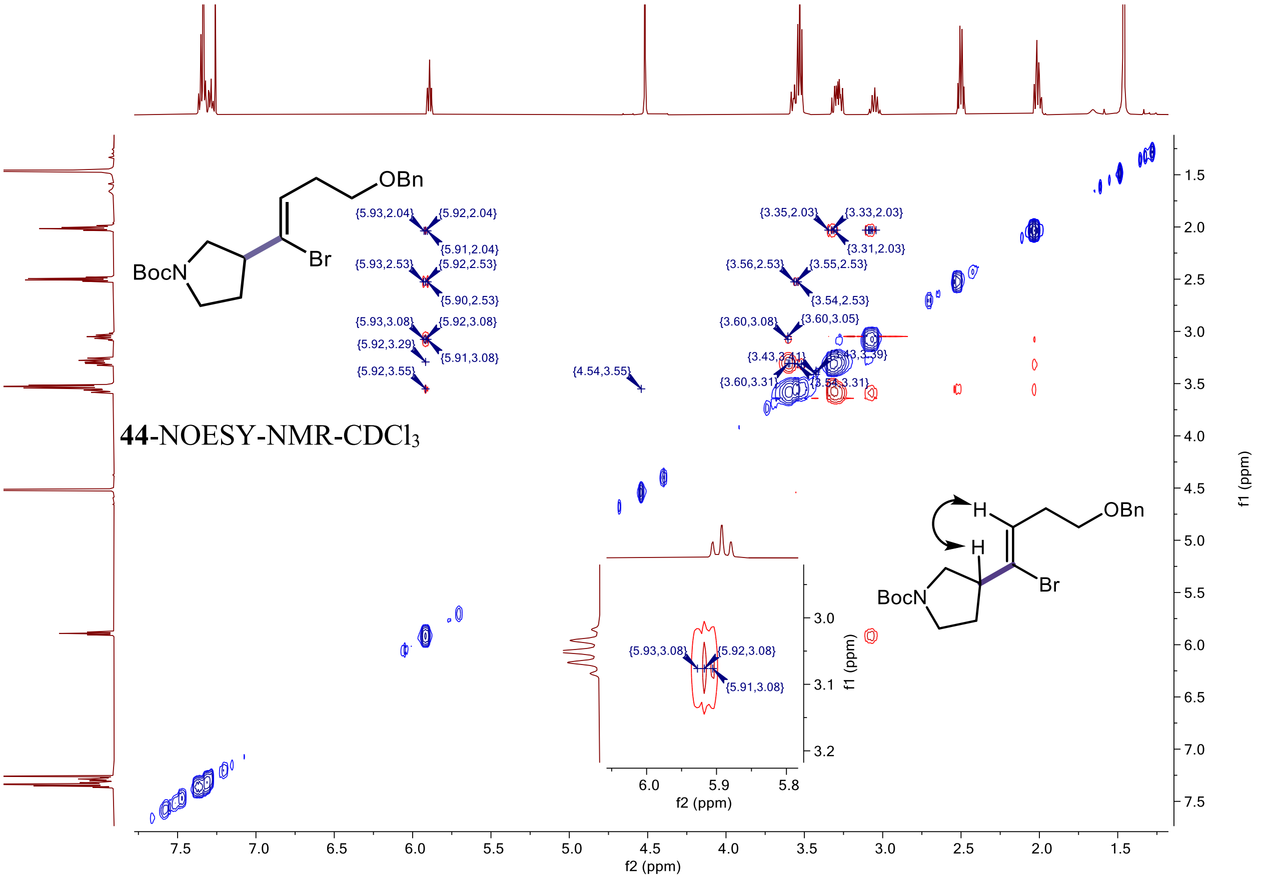
44-NOESY-NMR-CDCl3
BocN
OBn
Br
H
H
f1 (ppm)
f2 (ppm)

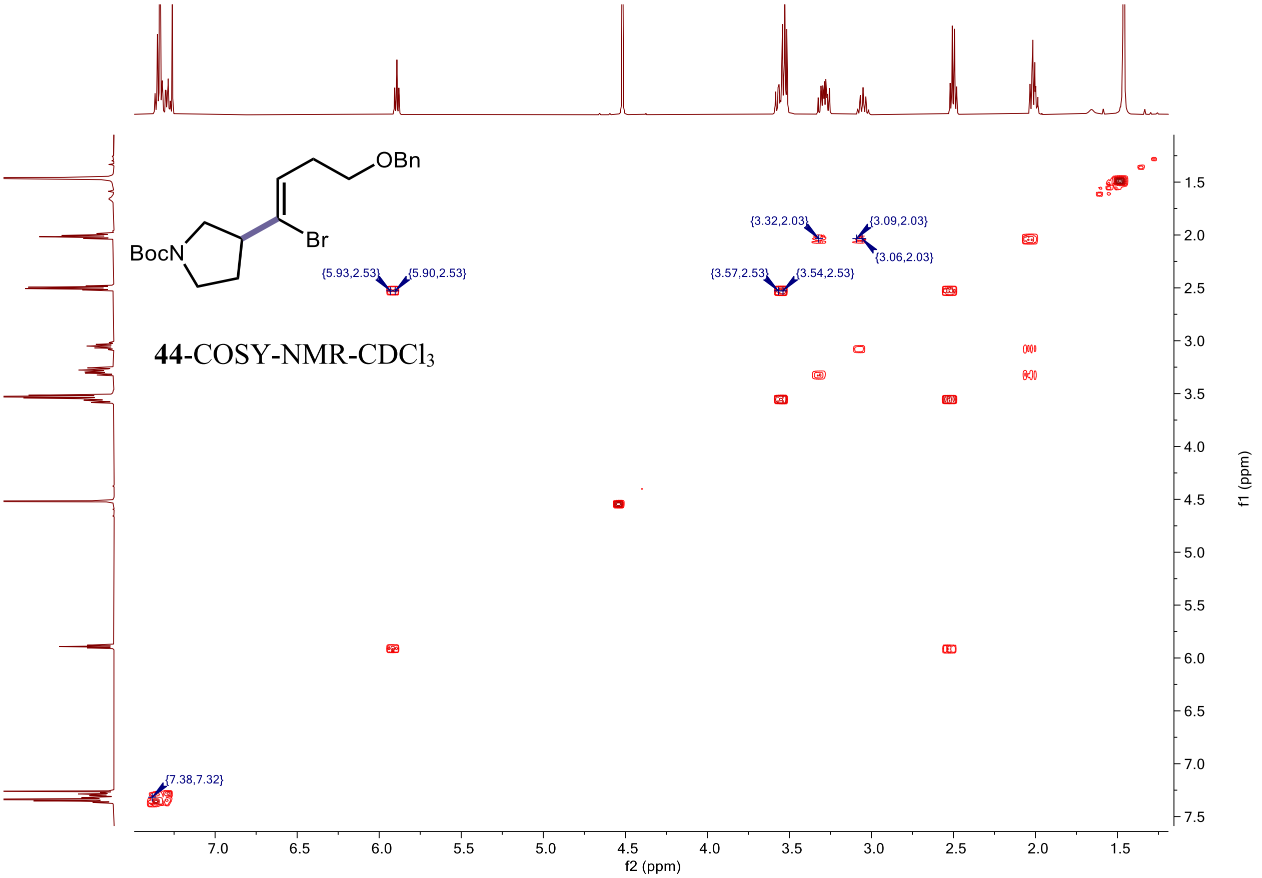
44-COSY-NMR-CDCl3
BocN
OBn
Br
f1 (ppm)
f2 (ppm)

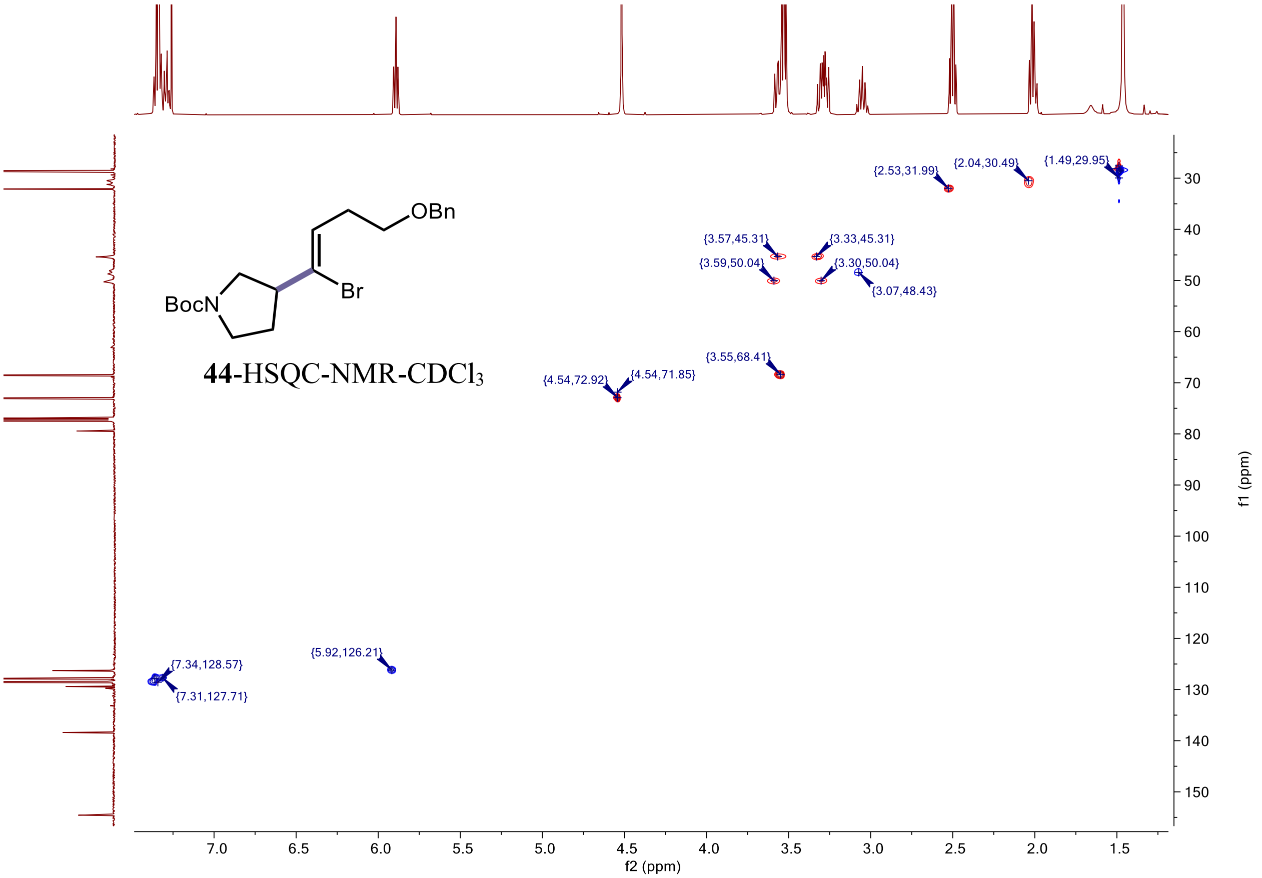

OBn
BocN
Br
44-HSQC-NMR-CDCl3
{2.53,31.99}
{2.04,30.49}
{1.49,29.95}
{3.57,45.31}
{3.33,45.31}
{3.59,50.04}
{3.30,50.04}
{3.07,48.43}
{3.55,68.41}
{4.54,72.92}
{4.54,71.85}
{5.92,126.21}
{7.34,128.57}
{7.31,127.71}
f2 (ppm)
f1 (ppm)

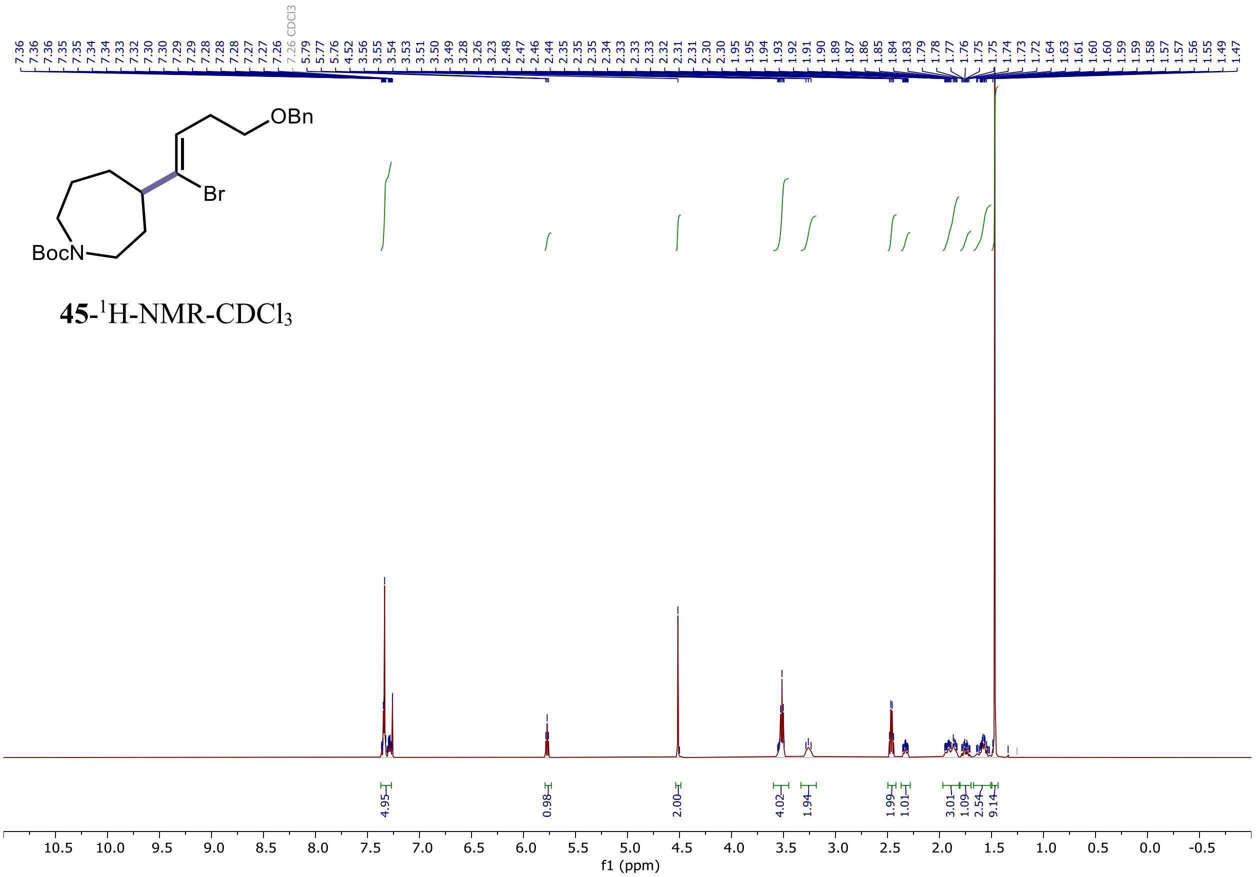

7.36 7.36 7.35 7.35 7.34 7.34 7.33 7.32 7.30 7.30 7.29 7.29 7.28 7.28 7.27 7.27 7.26 7.26 CDCl3 5.79 5.77 5.76 4.52 3.56 3.55 3.54 3.53 3.51 3.50 3.49 3.28 3.26 3.23 2.48 2.47 2.46 2.44 2.35 2.35 2.34 2.33 2.33 2.32 2.32 2.31 2.31 2.30 2.30 1.95 1.95 1.94 1.93 1.92 1.91 1.90 1.89 1.87 1.85 1.84 1.83 1.79 1.78 1.77 1.76 1.75 1.75 1.74 1.73 1.72 1.64 1.63 1.61 1.60 1.59 1.59 1.58 1.57 1.57 1.56 1.55 1.49 1.47
OBn
Br
BocN
45-1H-NMR-CDCl3
4.95
0.98
2.00
4.02
1.94
1.99
1.01
3.01
1.09
2.54
9.14
f1 (ppm)

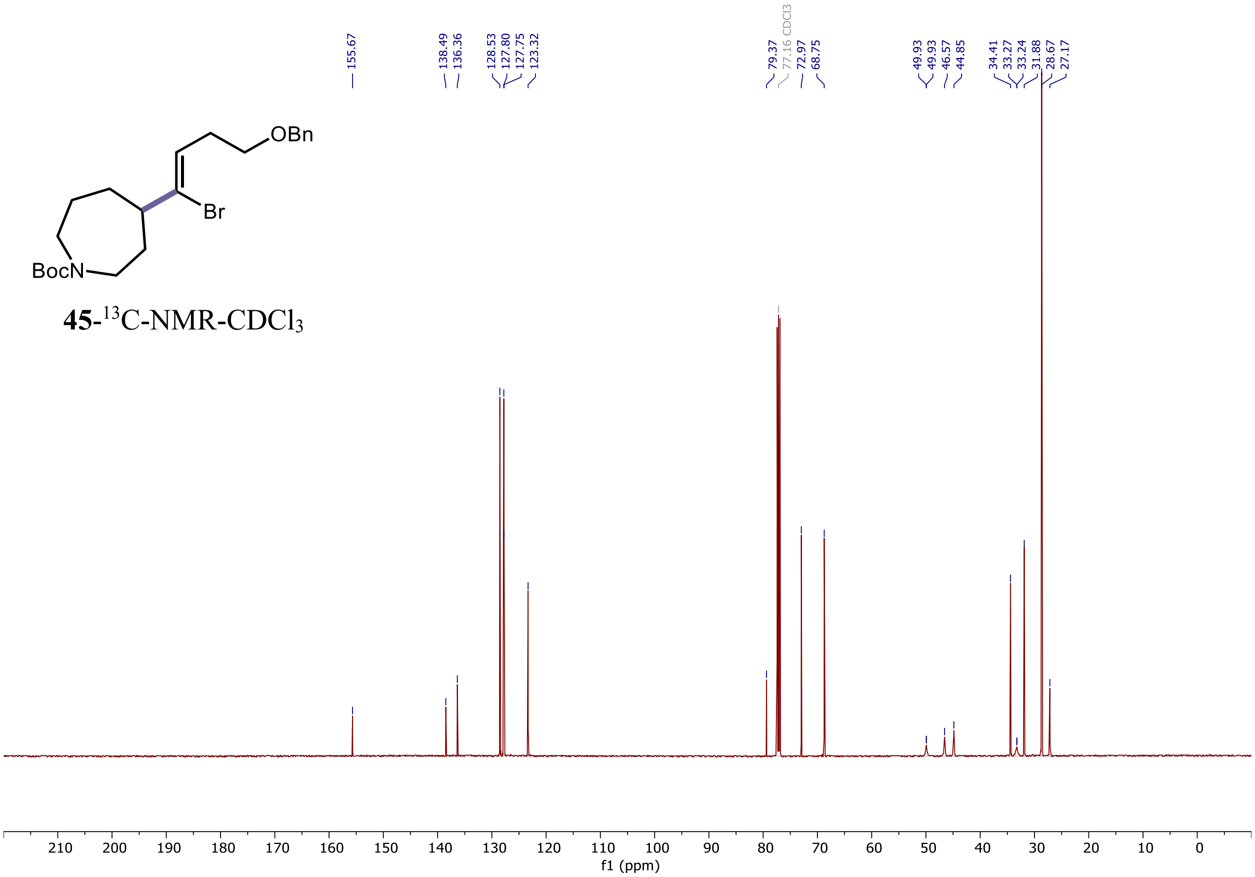
155.67
138.49
136.36
128.53
127.80
127.75
123.32
79.37
77.16 CDCl3
72.97
68.75
49.93
49.93
46.57
44.85
34.41
33.27
33.24
31.88
28.67
27.17
OBn
Br
BocN
45-^{13}C-NMR-CDCl$_3$
f1 (ppm)

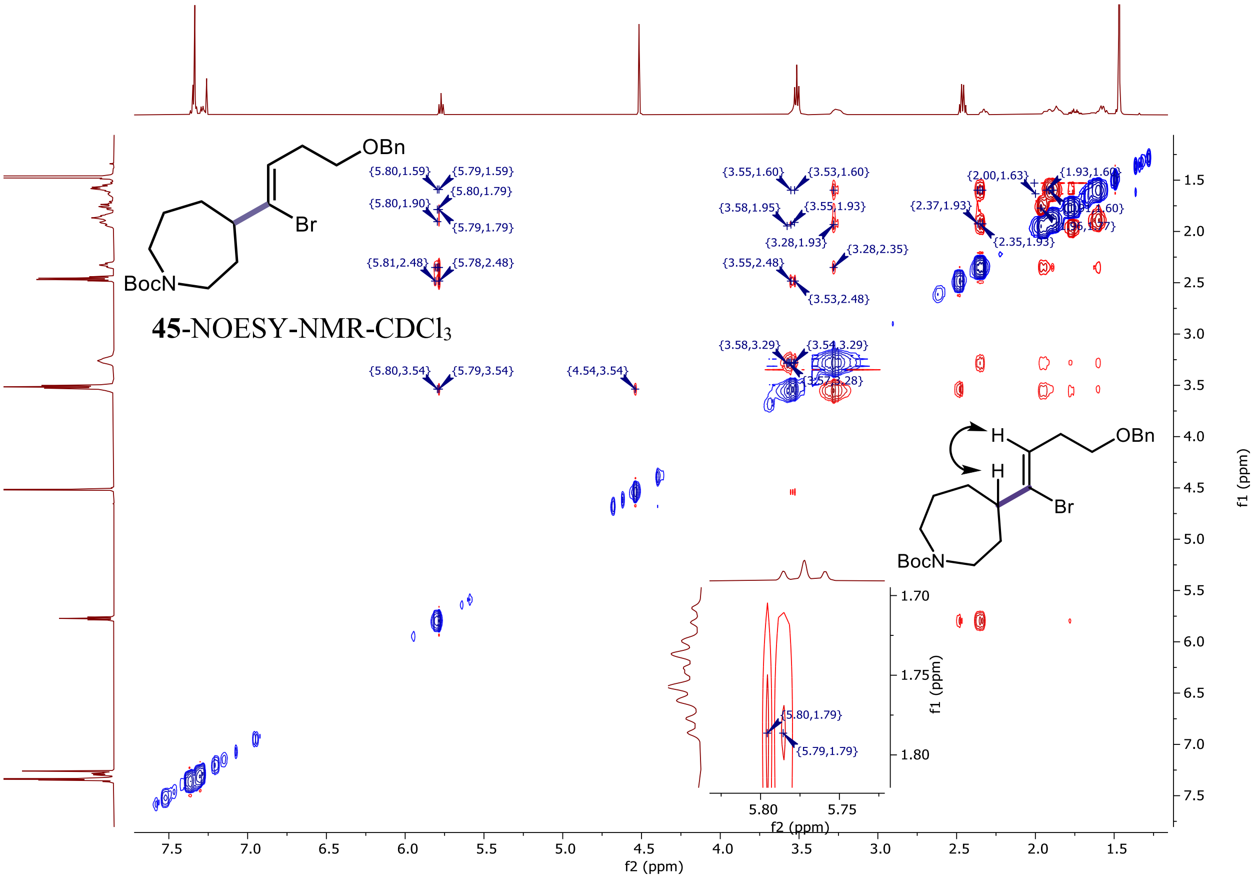
OBn
Br
BocN
45-NOESY-NMR-CDCl$_3$
H
H
f2 (ppm)
f1 (ppm)

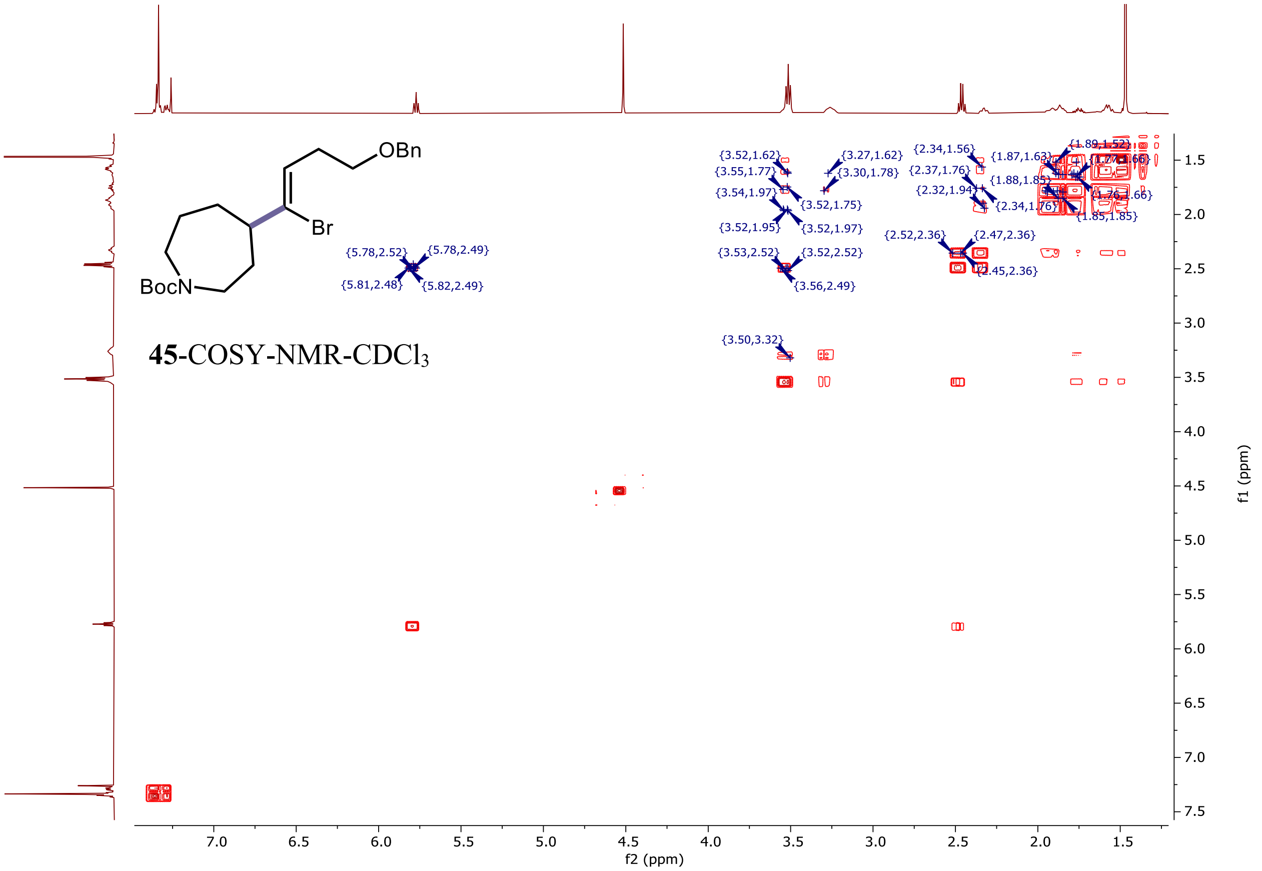
45-COSY-NMR-CDCl3
OBn
Br
BocN
f2 (ppm)
f1 (ppm)

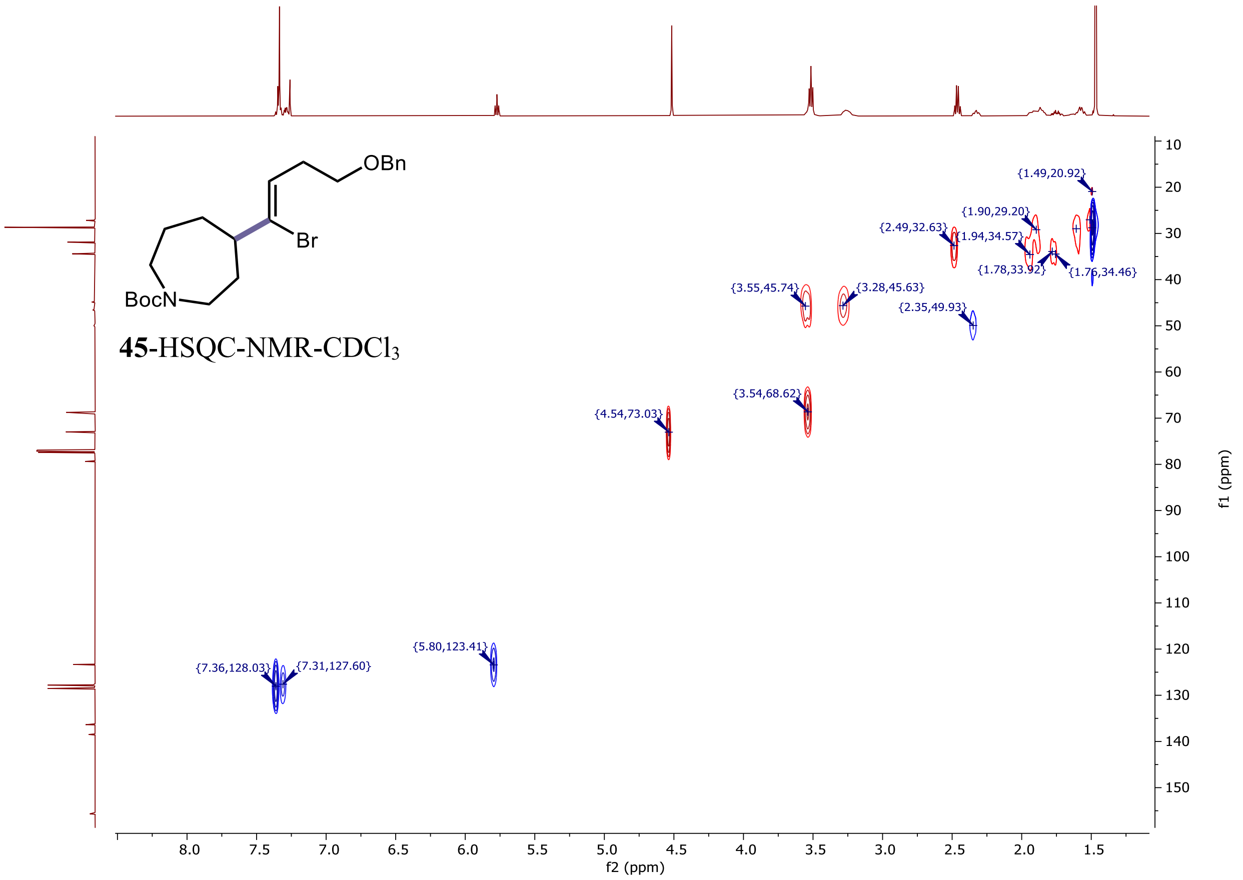
45-HSQC-NMR-CDCl3
OBn
Br
BocN
f2 (ppm)
f1 (ppm)

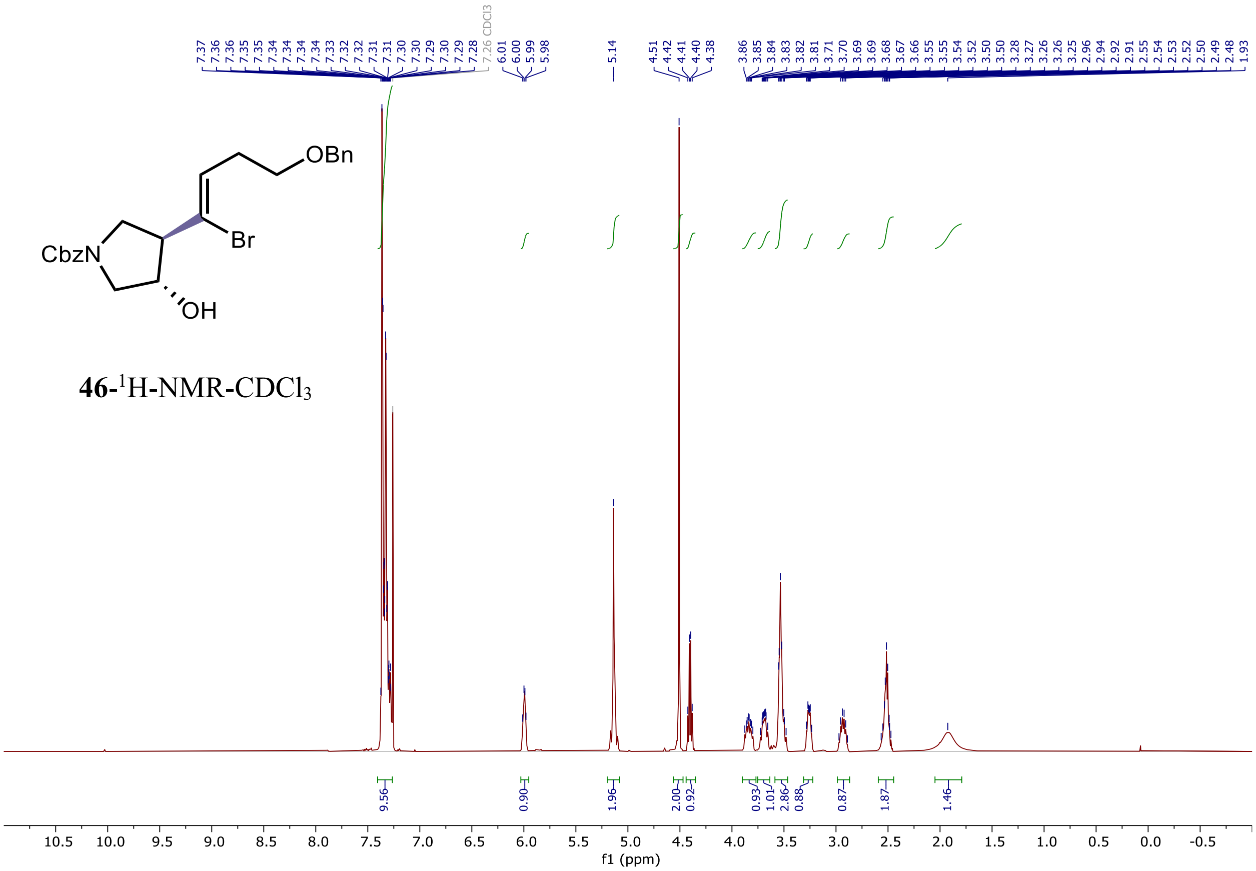
7.37 7.36 7.36 7.35 7.35 7.34 7.34 7.34 7.34 7.33 7.32 7.32 7.31 7.31 7.30 7.30 7.29 7.30 7.29 7.28
7.26 CDCl3
6.01 6.00 5.99 5.98
5.14
4.51 4.42 4.41 4.40 4.38
3.86 3.85 3.84 3.83 3.82 3.81 3.71 3.70 3.69 3.69 3.68 3.67 3.66 3.55 3.55 3.54 3.52 3.50 3.50 3.28 3.27 3.26 3.26 3.25 2.96 2.94 2.92 2.91 2.55 2.54 2.53 2.52 2.50 2.49 2.48 1.93
OBn
CbzN
Br
OH
46-¹H-NMR-CDCl₃
9.56 0.90 1.96 2.00 0.92 0.93 1.01 2.86 0.88 0.87 1.87 1.46
10.5 10.0 9.5 9.0 8.5 8.0 7.5 7.0 6.5 6.0 5.5 5.0 4.5 4.0 3.5 3.0 2.5 2.0 1.5 1.0 0.5 0.0 -0.5
f1 (ppm)

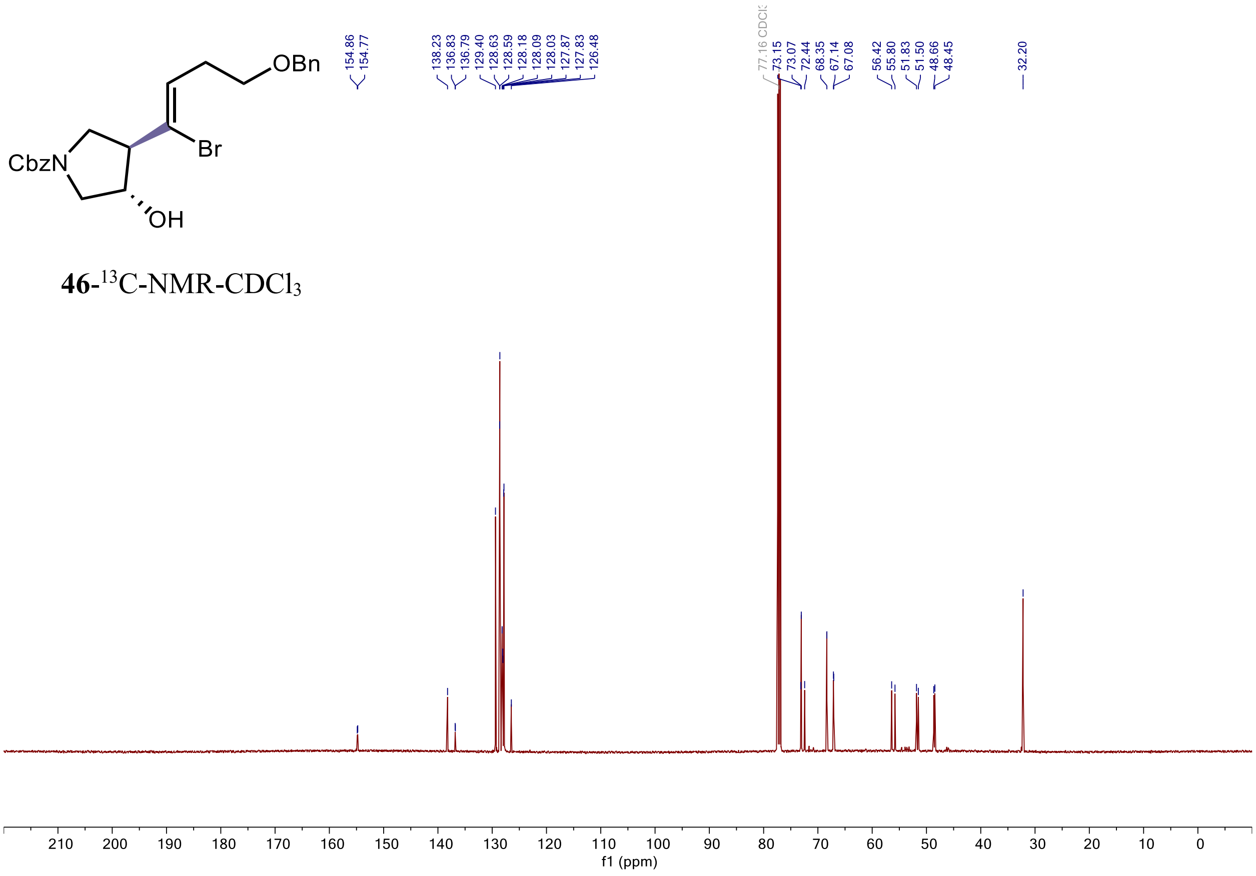
154.86 154.77
138.23 136.83 136.79 129.40 128.63 128.59 128.18 128.09 128.03 127.87 127.83 126.48
77.16 CDCl3
73.15 73.07 72.44 68.35 67.14 67.08
56.42 55.80 51.83 51.50 48.66 48.45
32.20
OBn
CbzN
Br
OH
46-¹³C-NMR-CDCl₃
210 200 190 180 170 160 150 140 130 120 110 100 90 80 70 60 50 40 30 20 10 0
f1 (ppm)

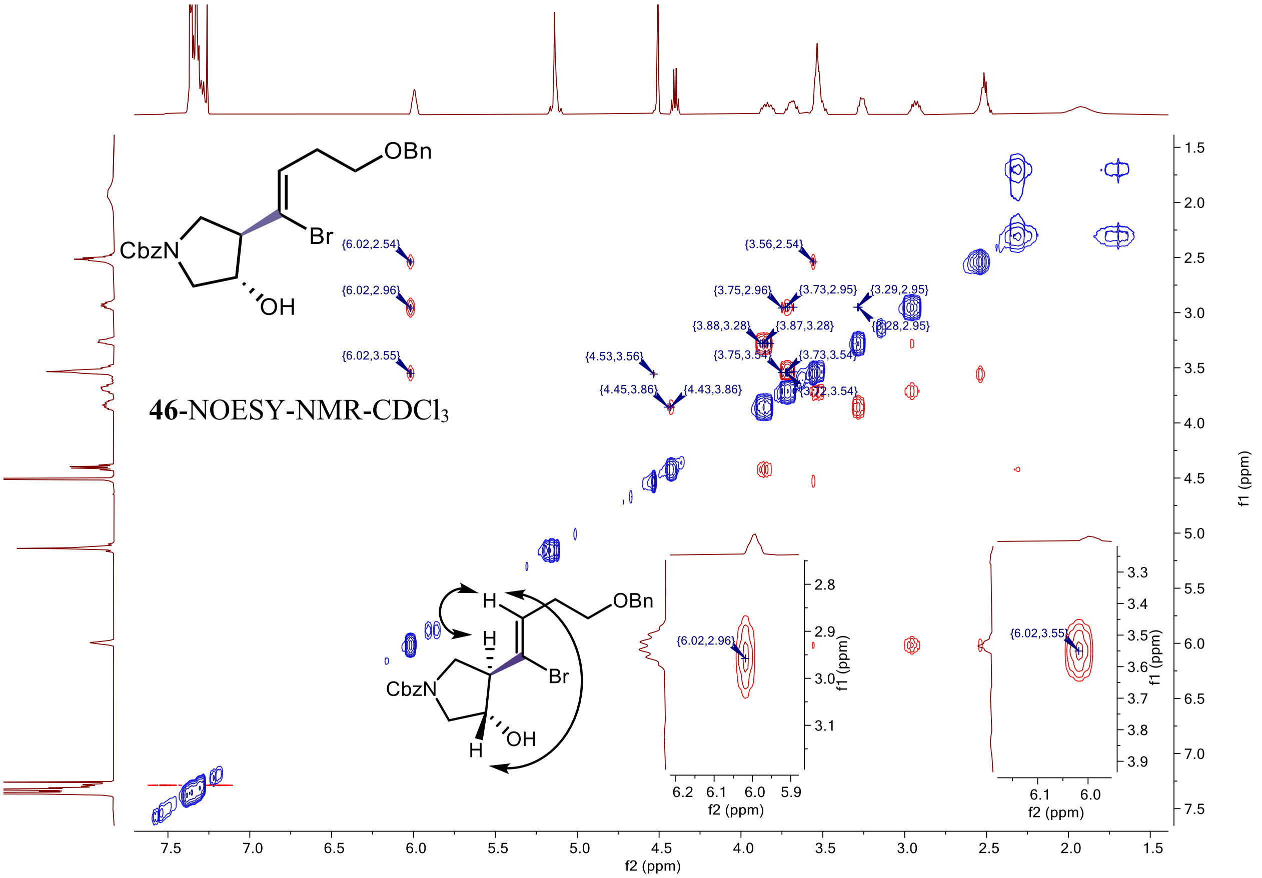
46-NOESY-NMR-CDCl3
OBn
CbzN
Br
OH
H
f2 (ppm)
f1 (ppm)

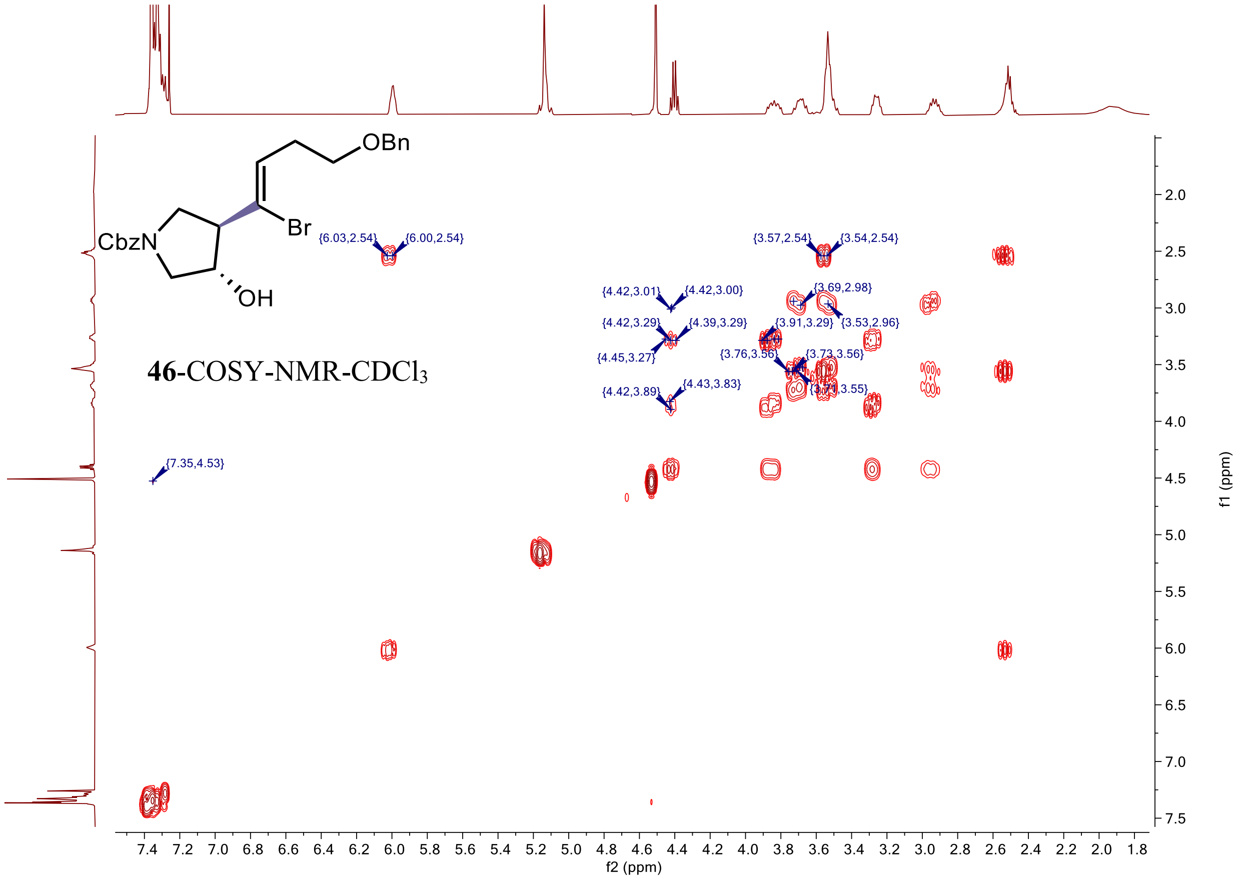
46-COSY-NMR-CDCl3
OBn
CbzN
Br
OH
f2 (ppm)
f1 (ppm)

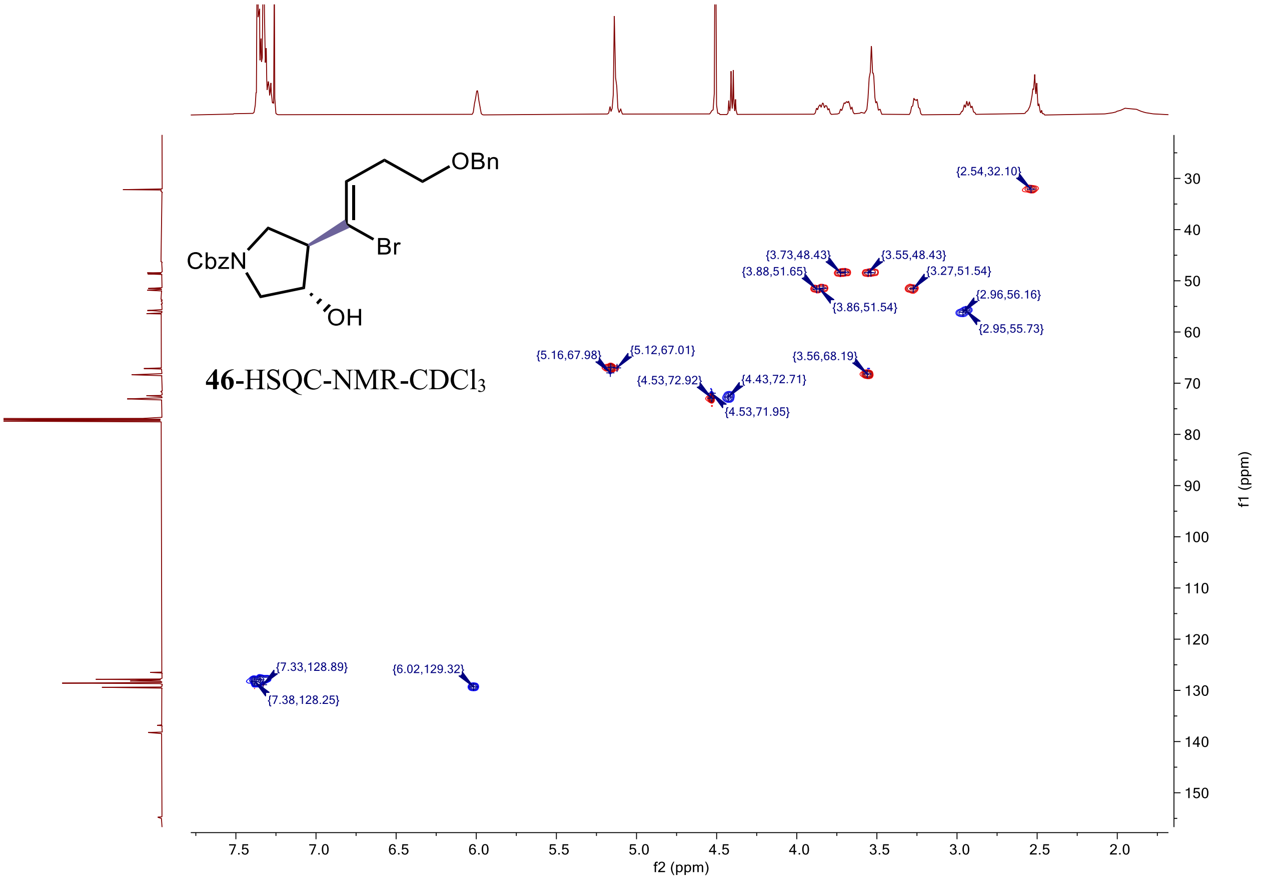

46-HSQC-NMR-$CDCl_3$

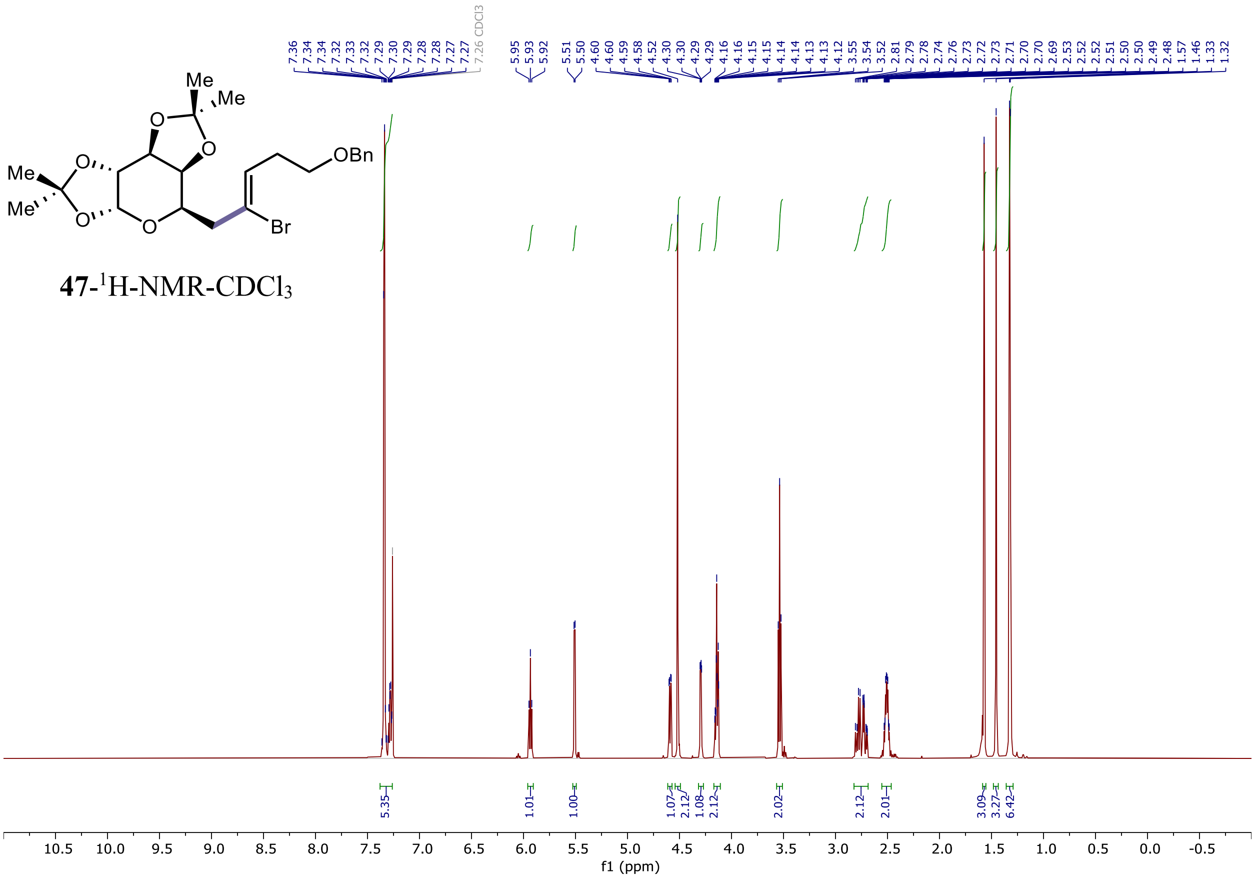

47-^{1}H-NMR-$CDCl_3$

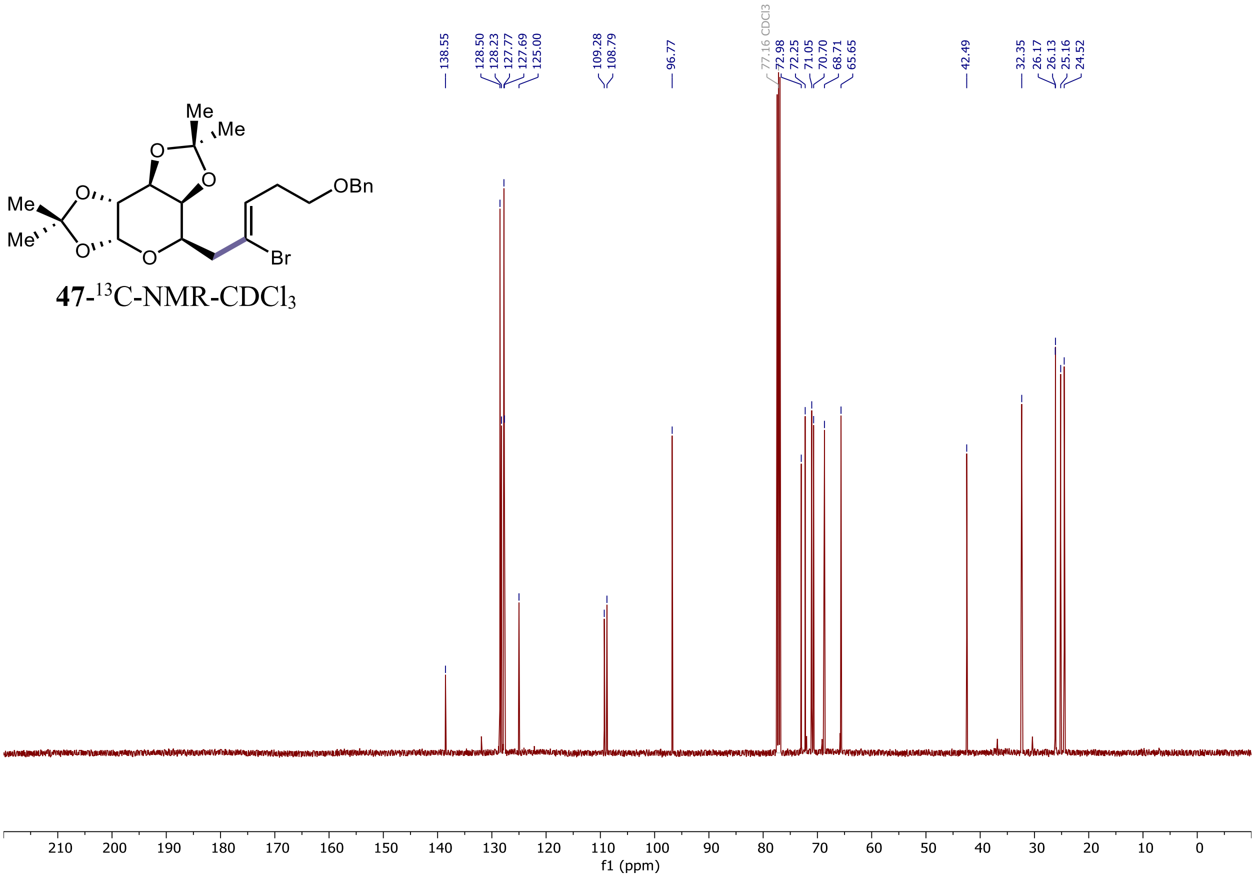
47-13C-NMR-CDCl3
f1 (ppm)

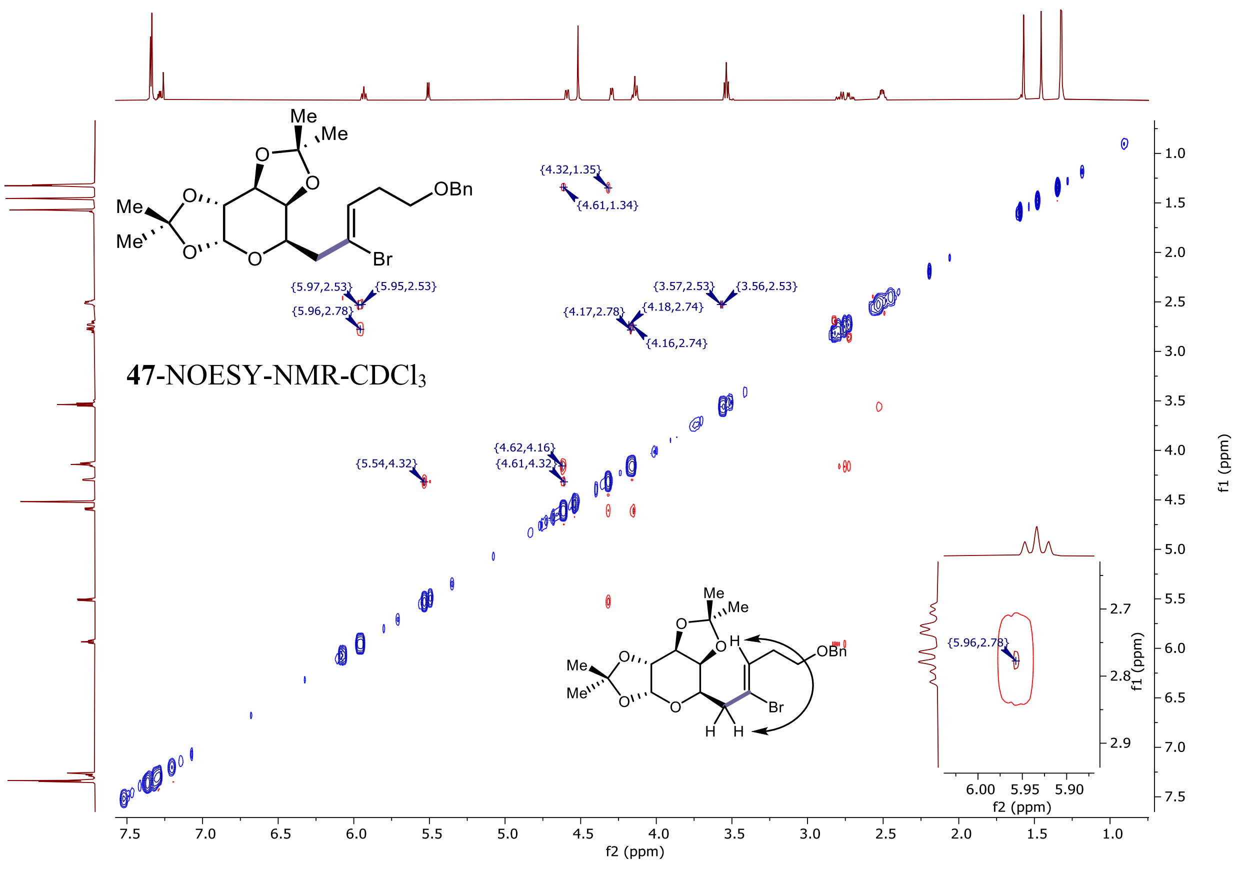
47-NOESY-NMR-CDCl3
f2 (ppm)
f1 (ppm)

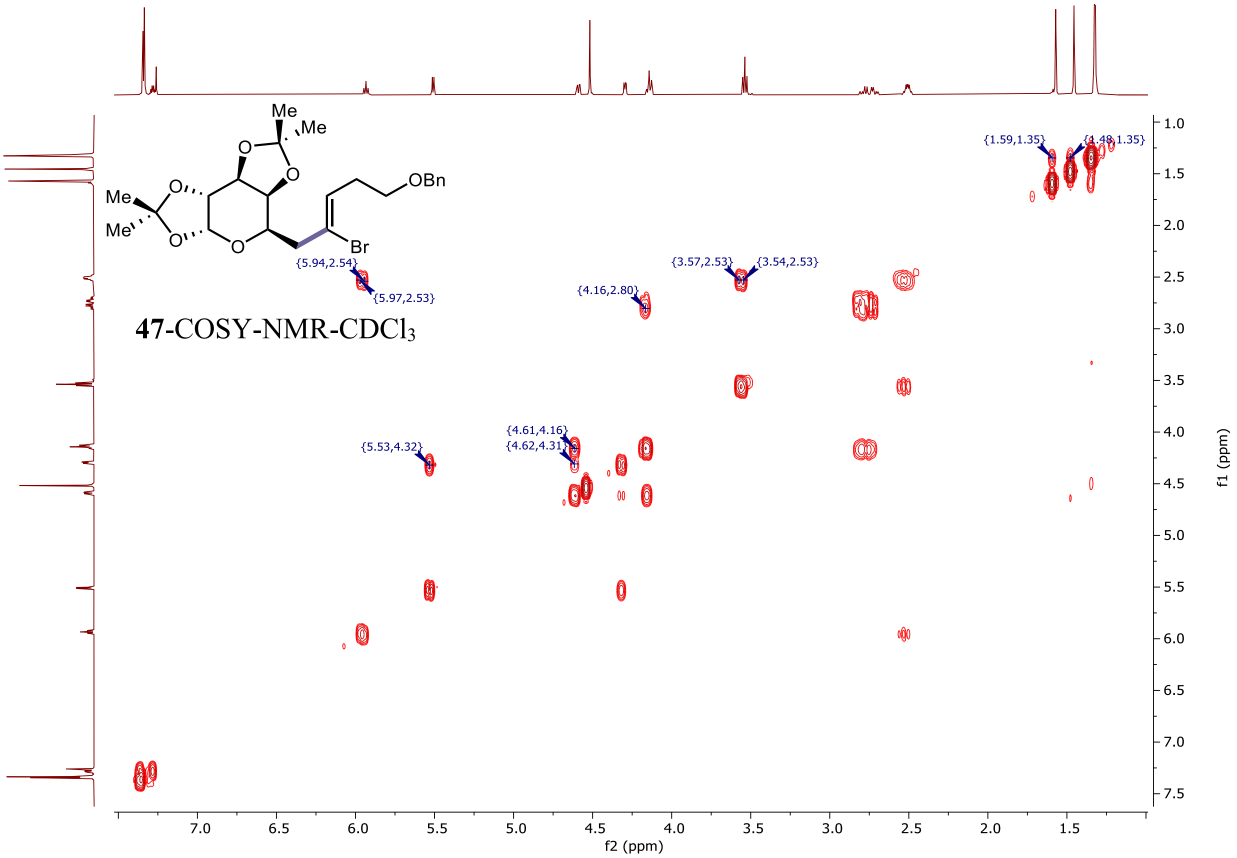

47-COSY-NMR-CDCl3
f2 (ppm)
f1 (ppm)

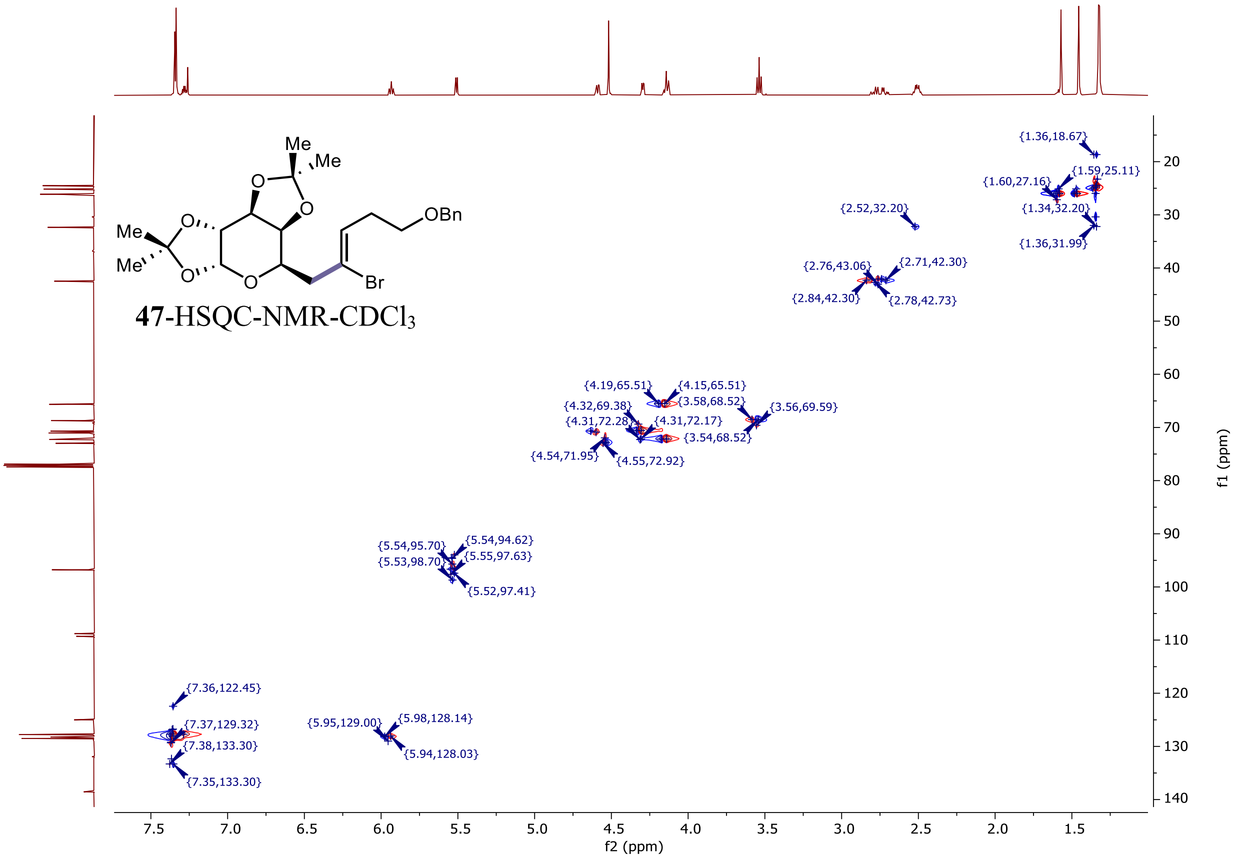

47-HSQC-NMR-CDCl3
f2 (ppm)
f1 (ppm)

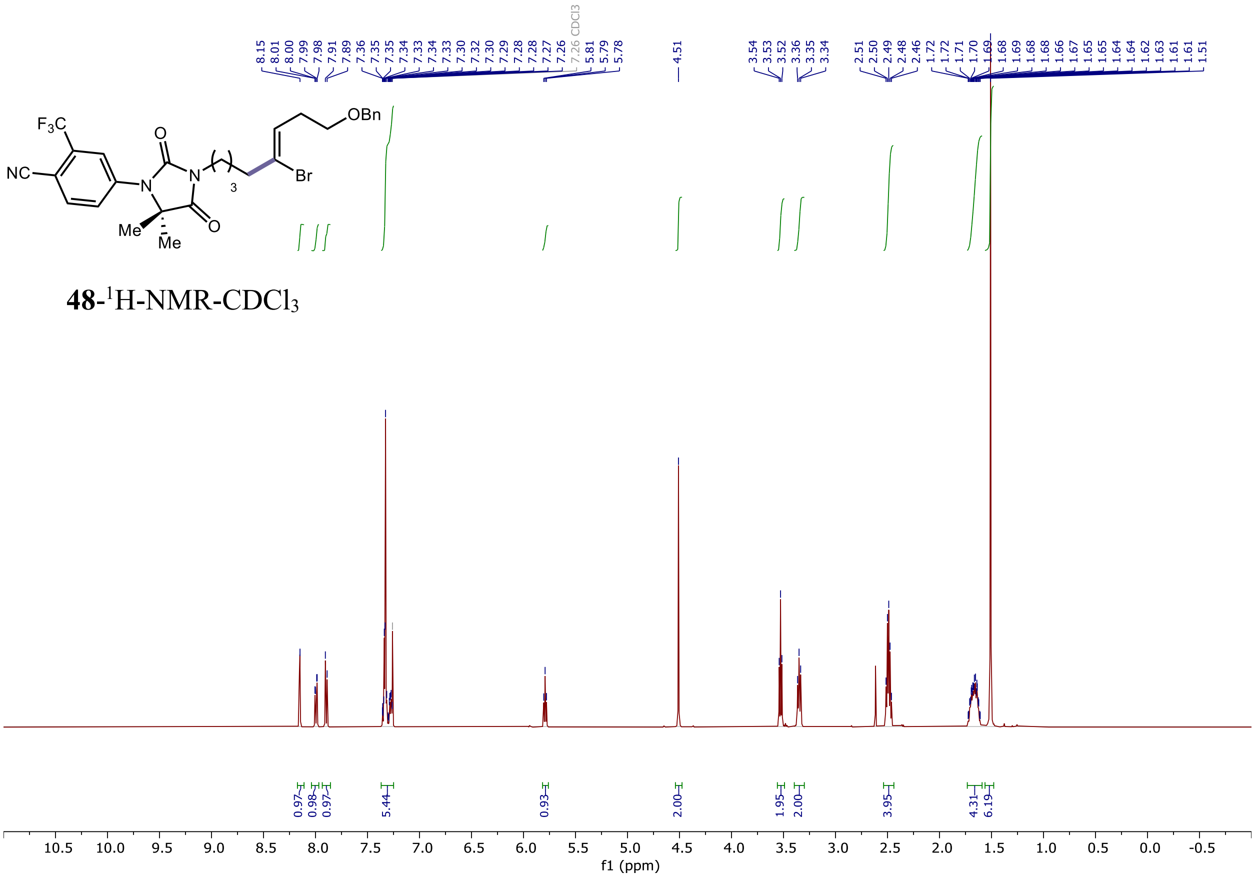

48-^{1}H-NMR-$CDCl_3$

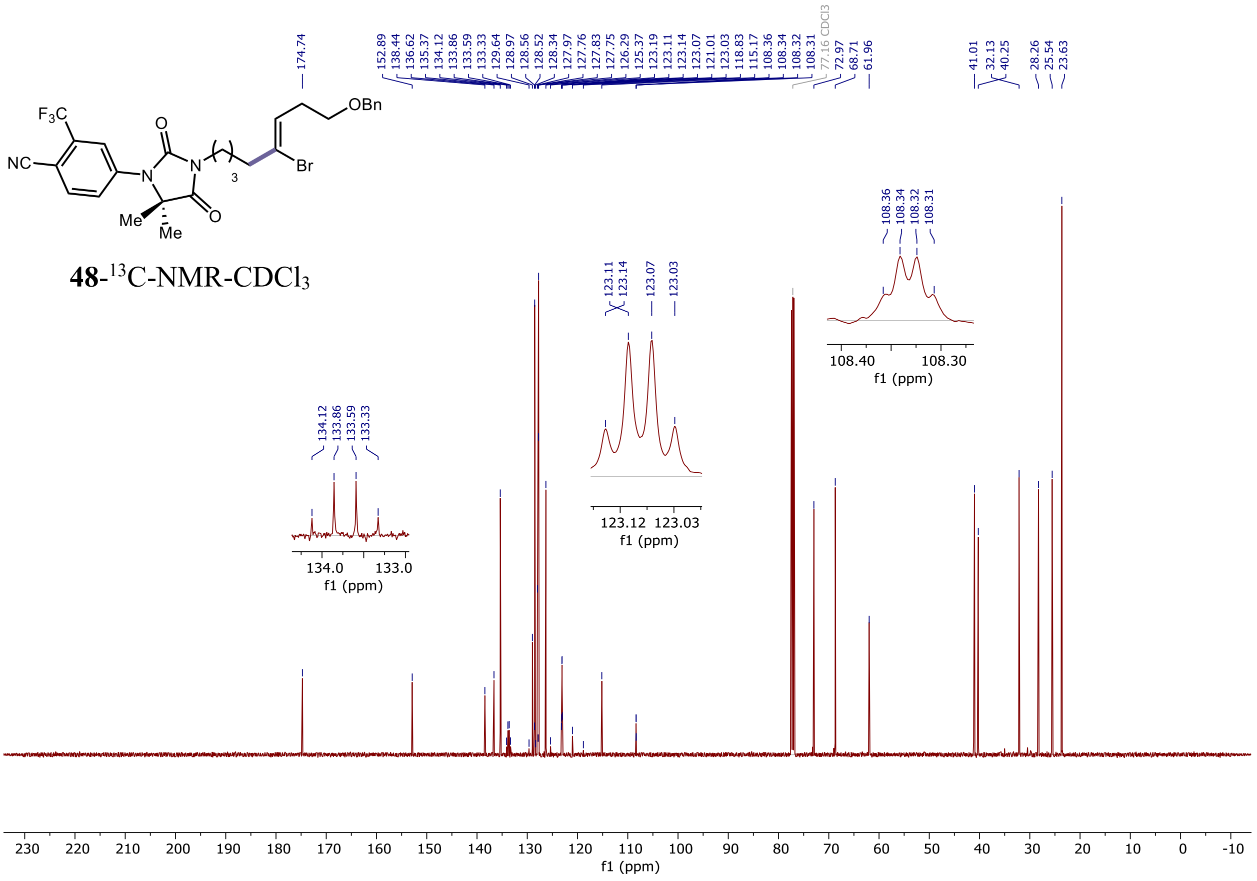

48-^{13}C-NMR-$CDCl_3$

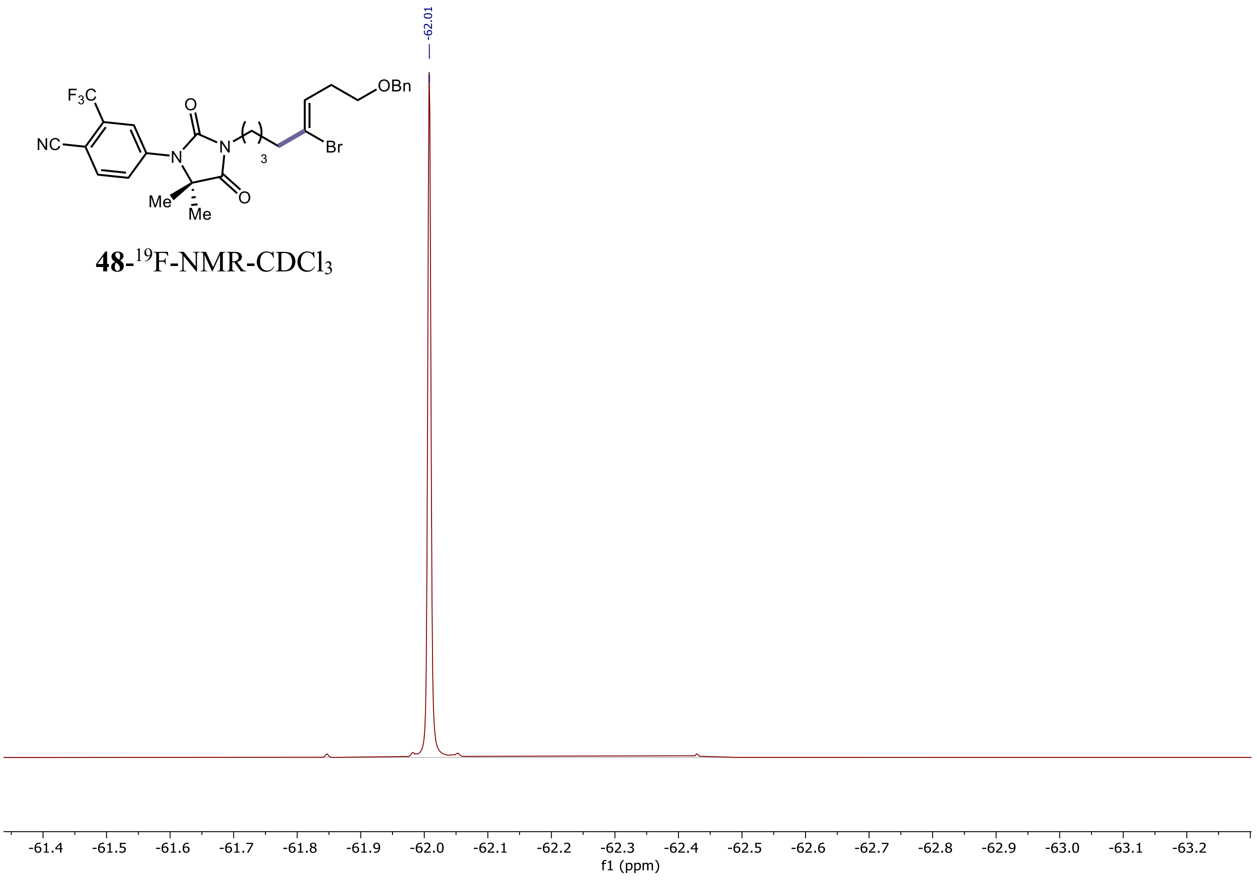
-62.01
F3C
NC
O
N
N
3
Br
OBn
O
Me
Me
48-19F-NMR-CDCl3
-61.4
-61.5
-61.6
-61.7
-61.8
-61.9
-62.0
-62.1
-62.2
-62.3
-62.4
-62.5
-62.6
-62.7
-62.8
-62.9
-63.0
-63.1
-63.2
f1 (ppm)

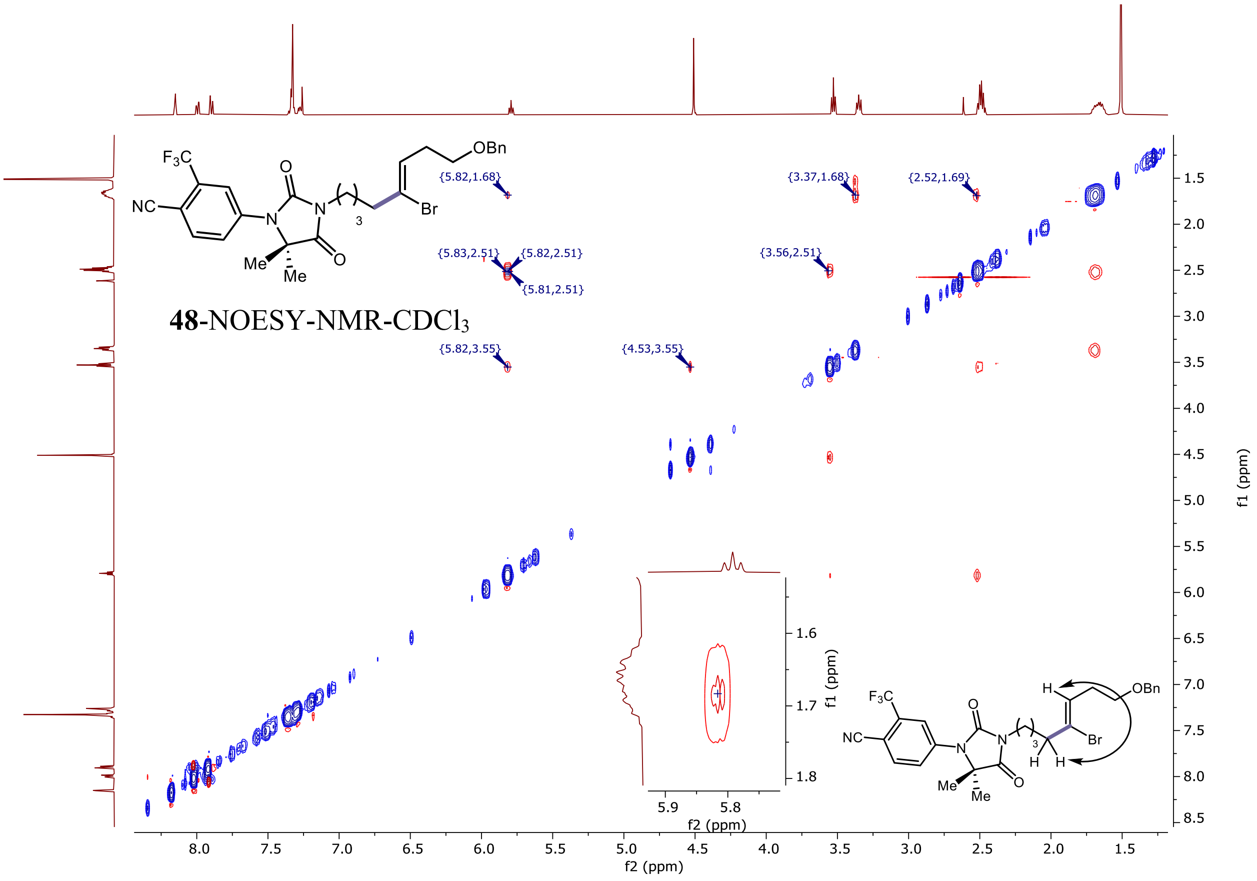
48-NOESY-NMR-CDCl3
{5.82,1.68}
{3.37,1.68}
{2.52,1.69}
{5.83,2.51}
{5.82,2.51}
{5.81,2.51}
{3.56,2.51}
{5.82,3.55}
{4.53,3.55}
f2 (ppm)
f1 (ppm)
1.6
1.7
1.8
5.9
5.8
OBn
Br
H
H
H
3
F3C
NC
Me
Me

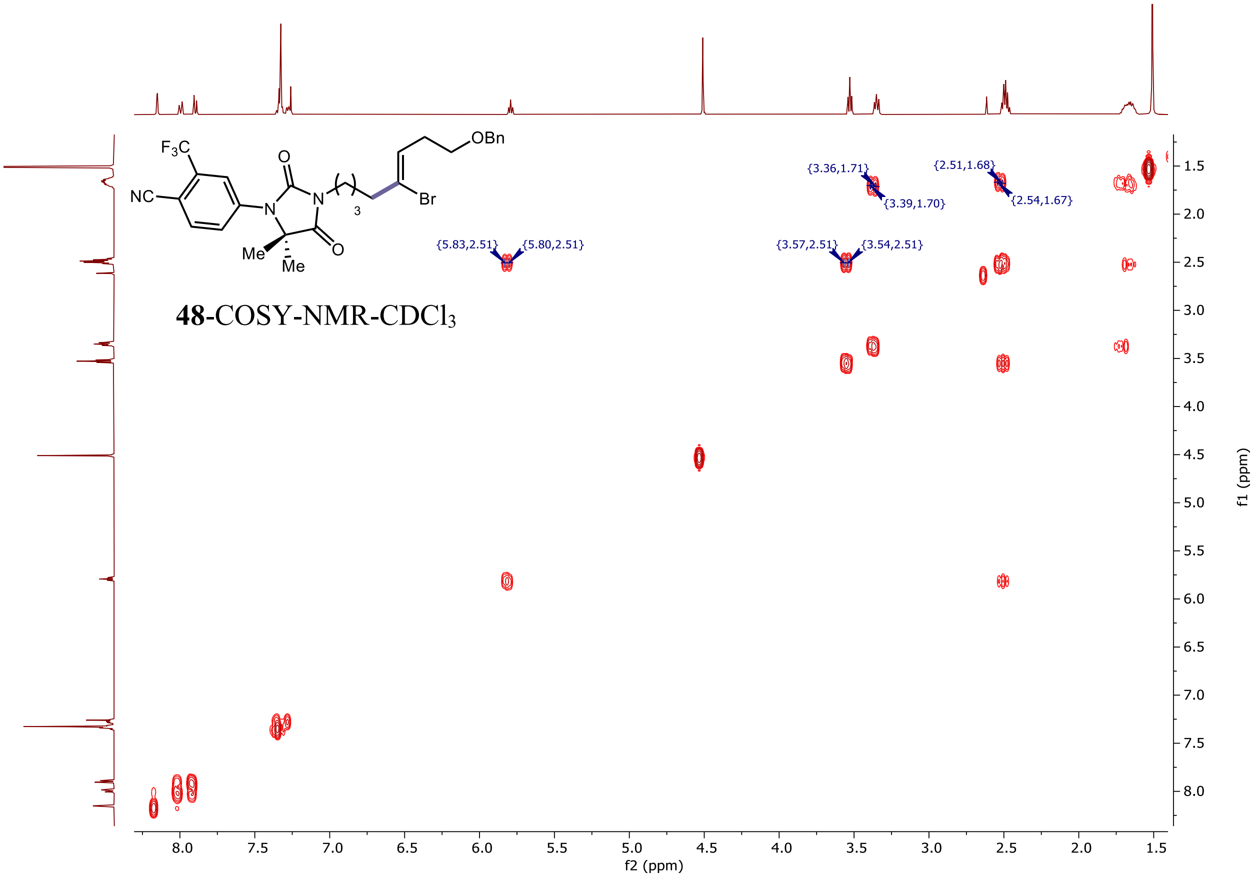
F3C
NC
OBn
Br
Me
Me
48-COSY-NMR-CDCl3
f2 (ppm)
f1 (ppm)

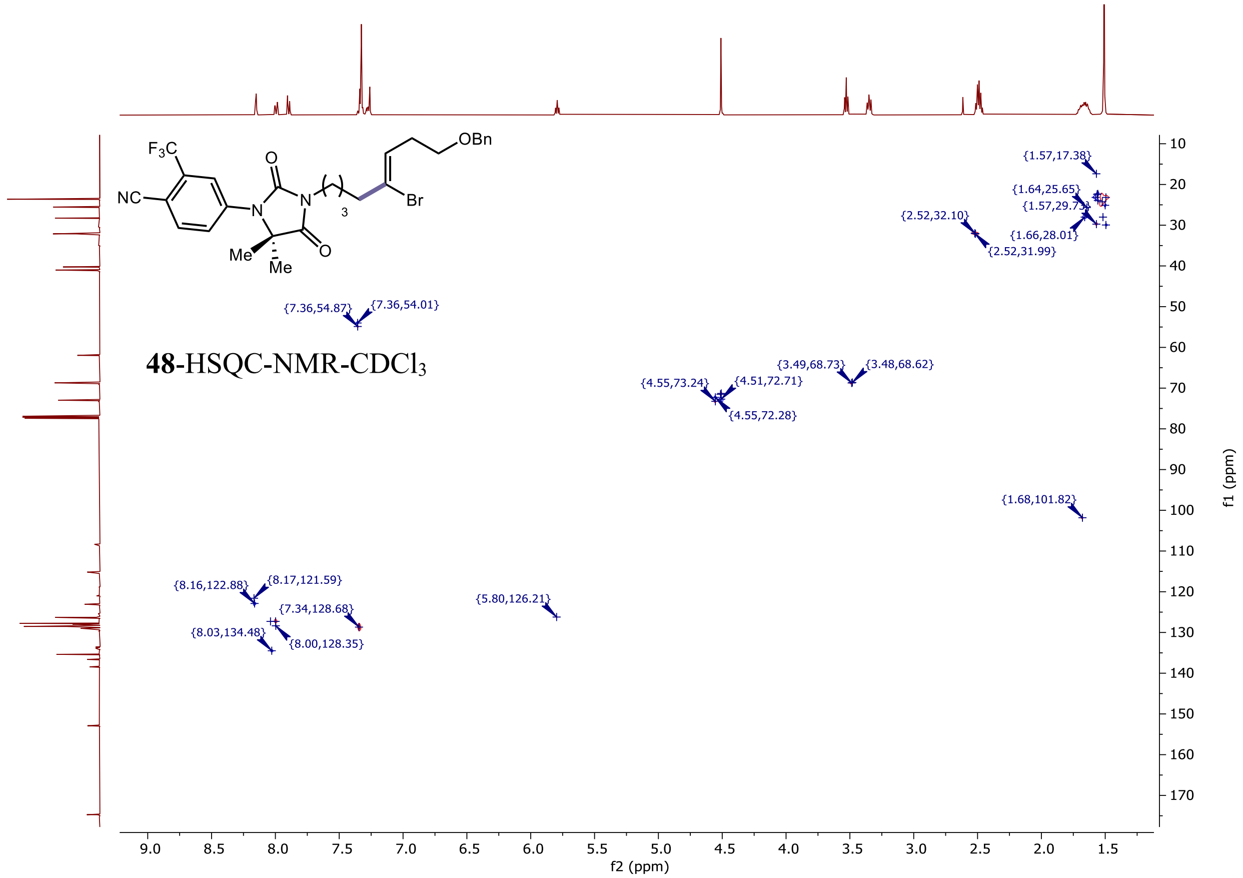
F3C
NC
OBn
Br
Me
Me
48-HSQC-NMR-CDCl3
f2 (ppm)
f1 (ppm)

49-^{1}H-NMR-$CDCl_3$

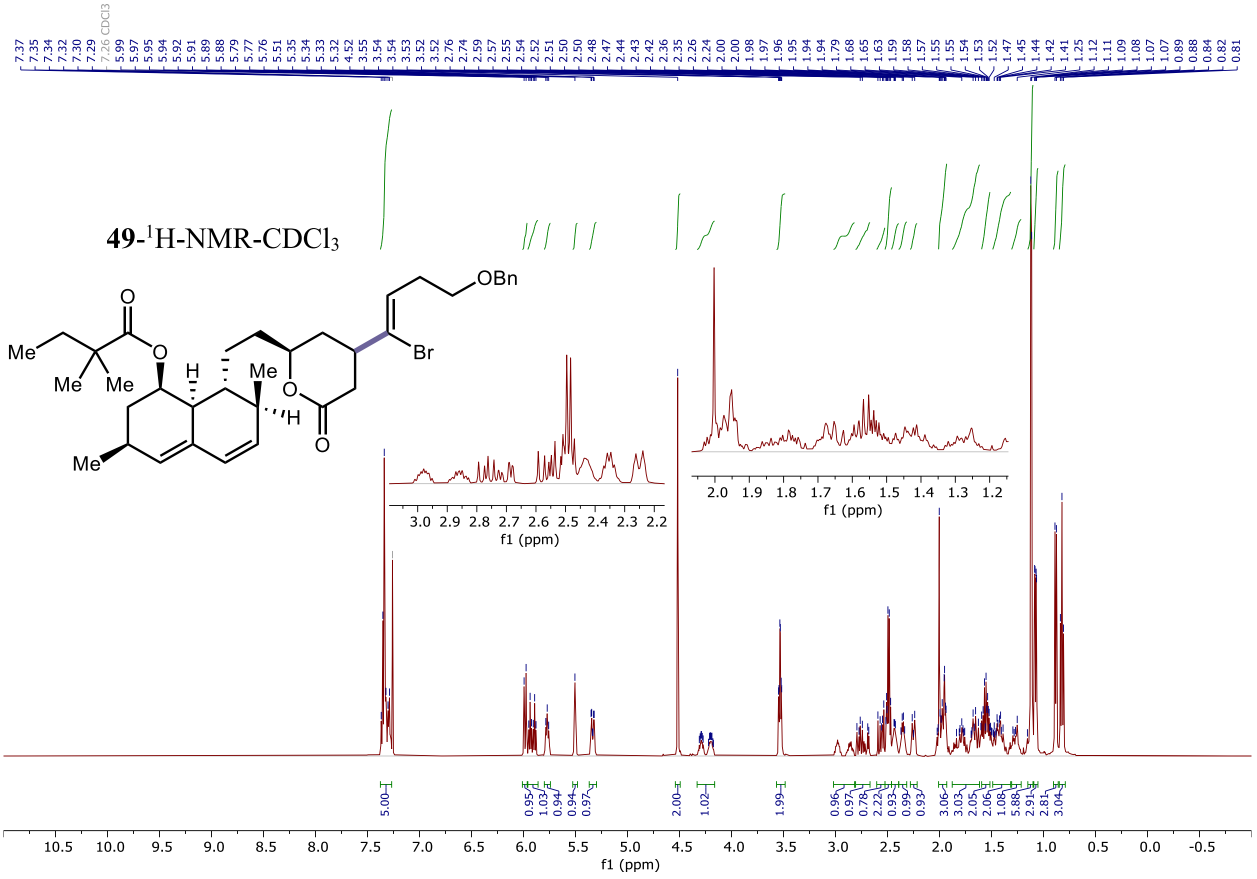

49-^{13}C-NMR-$CDCl_3$

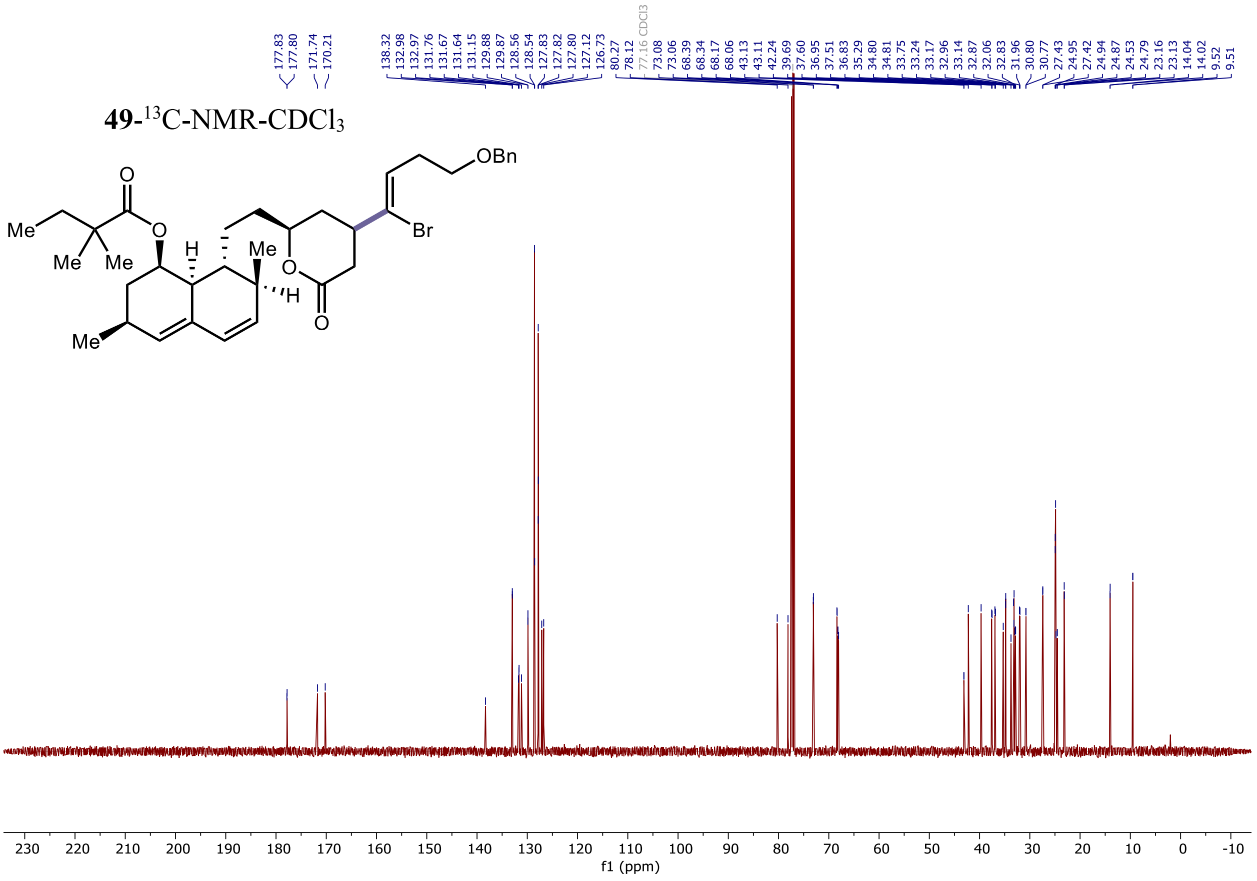

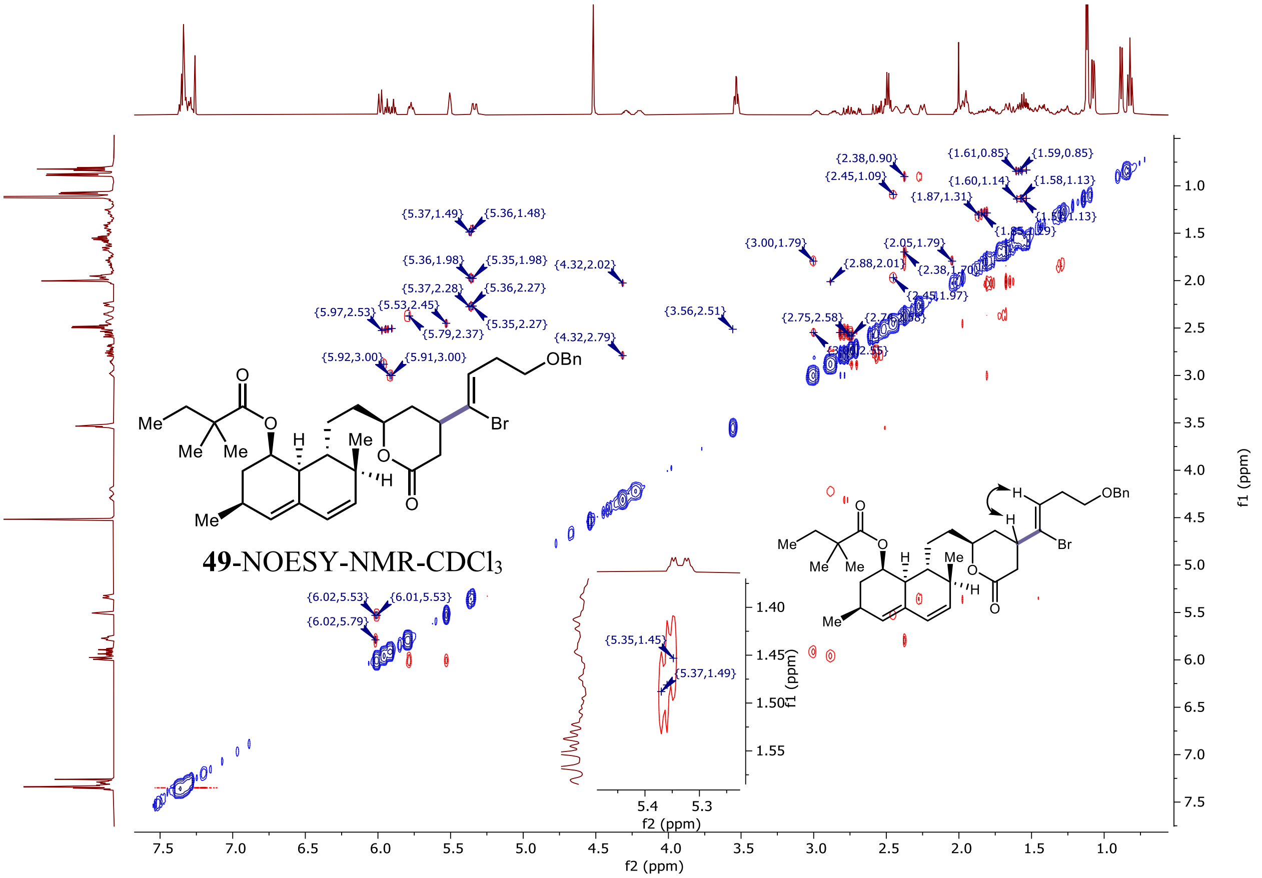
49-NOESY-NMR-CDCl3
f2 (ppm)
f1 (ppm)
Me
OBn
Br
H
O

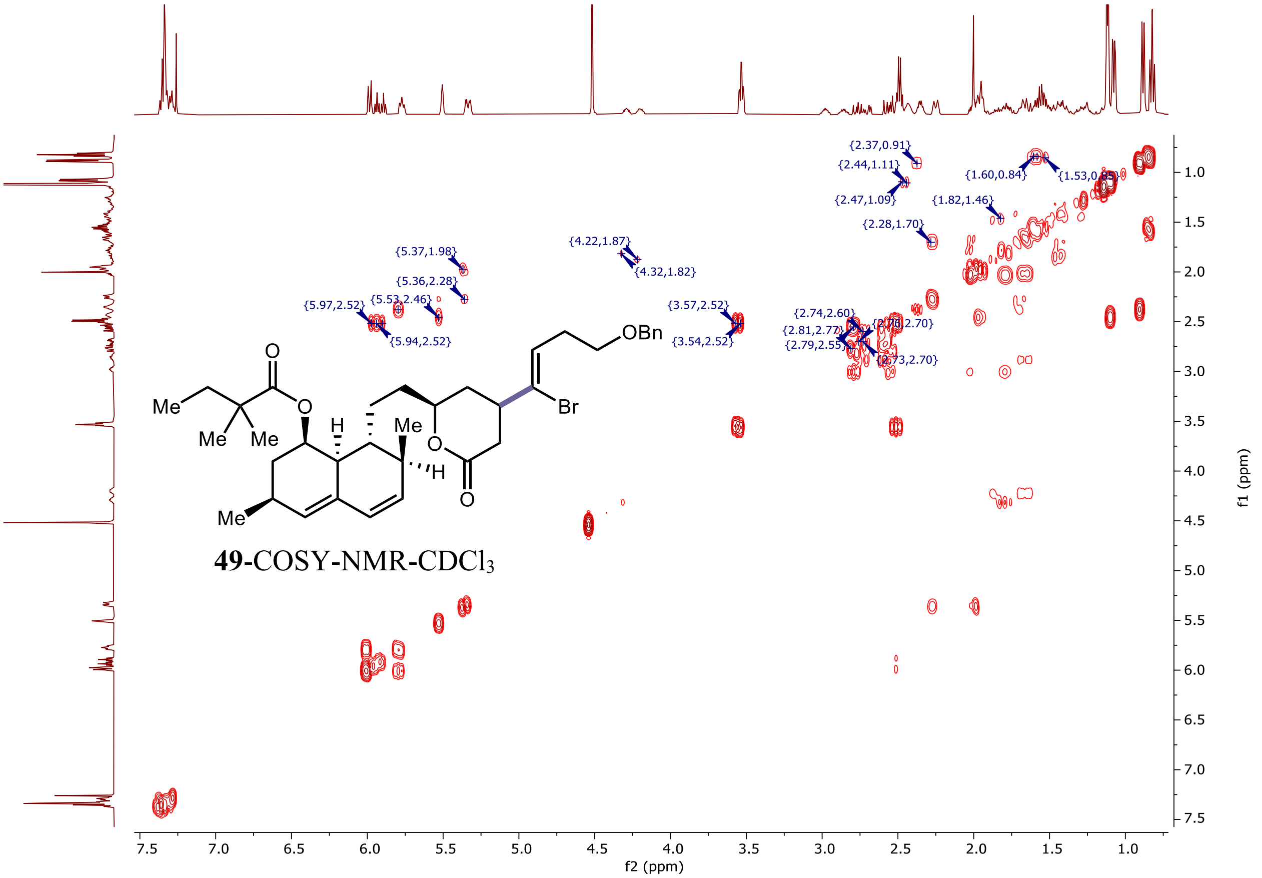
49-COSY-NMR-CDCl3
f2 (ppm)
f1 (ppm)
Me
OBn
Br
H
O

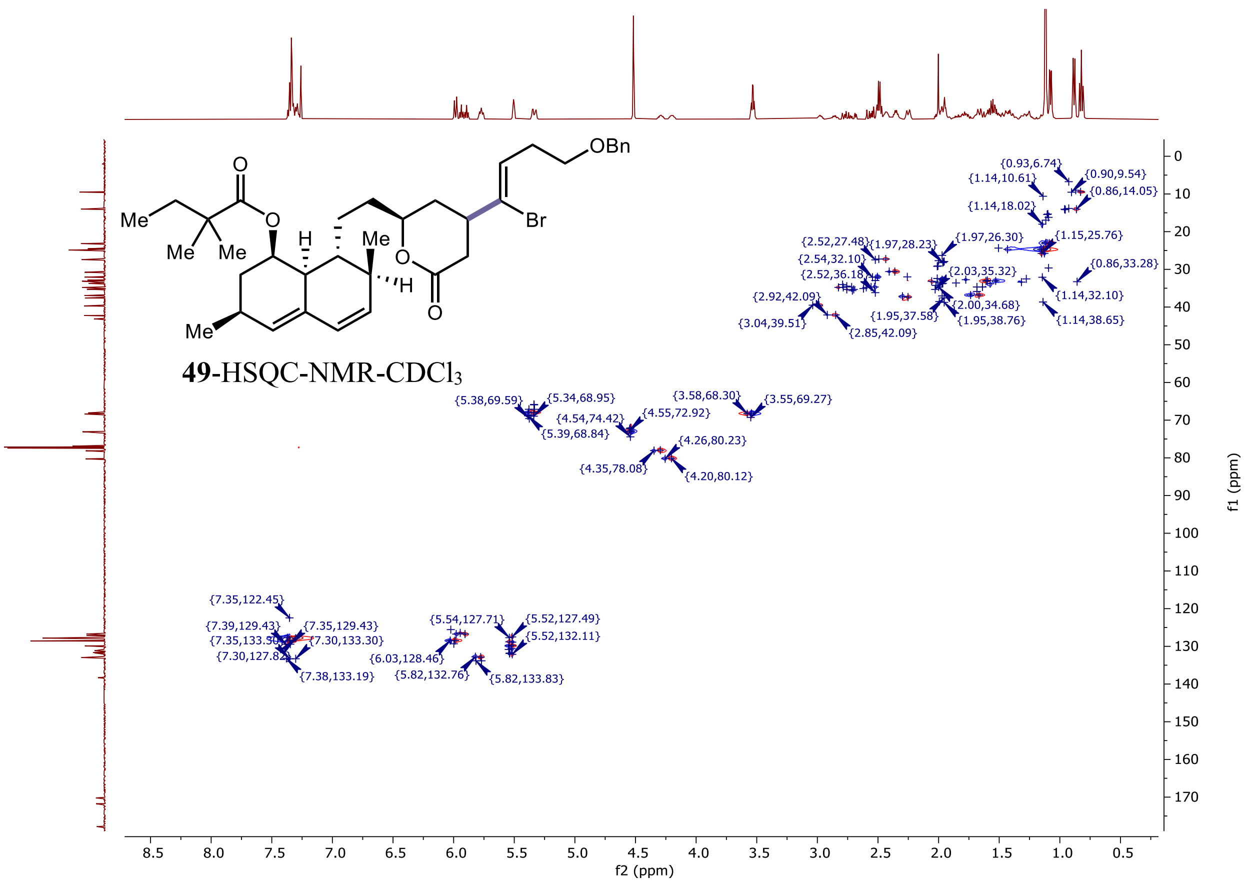

49-HSQC-NMR-$CDCl_3$

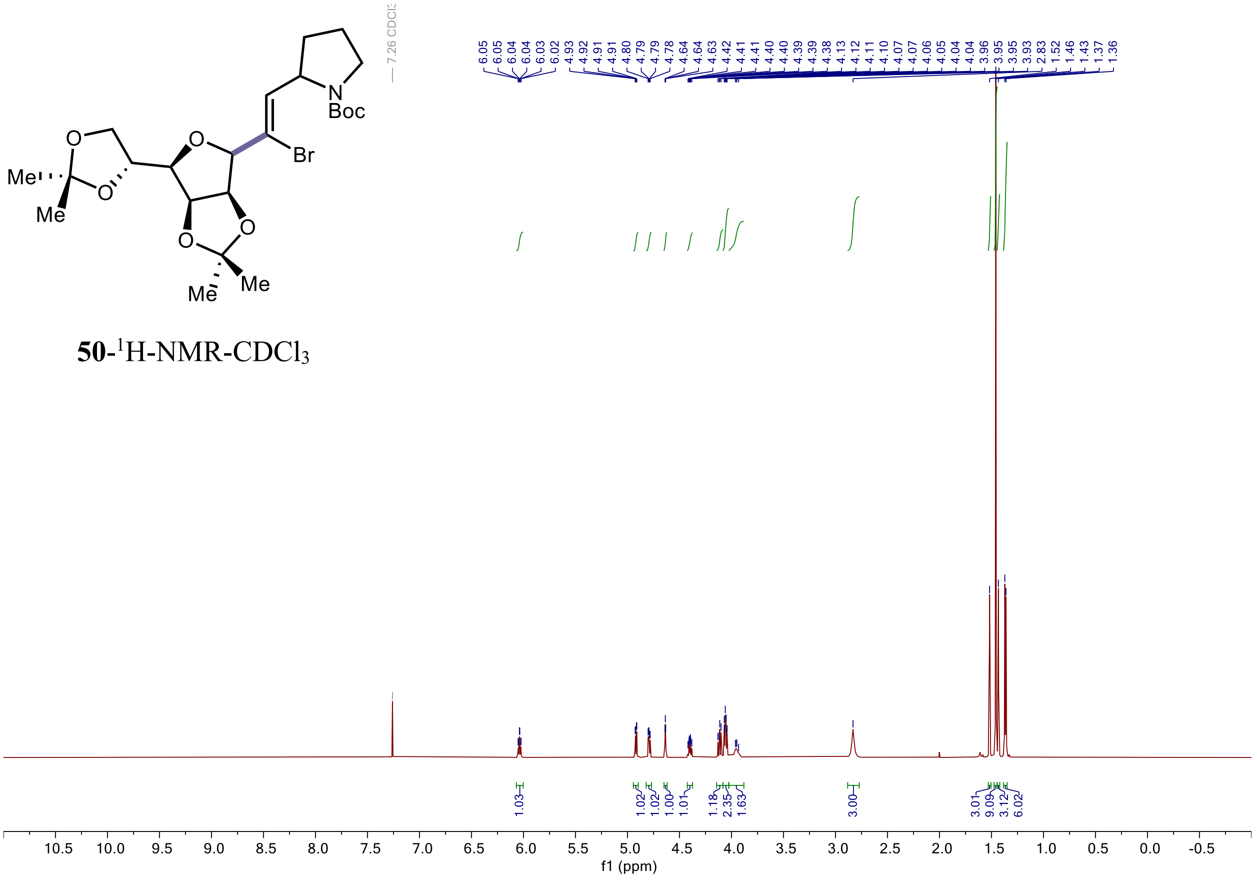

50-^{1}H-NMR-$CDCl_3$

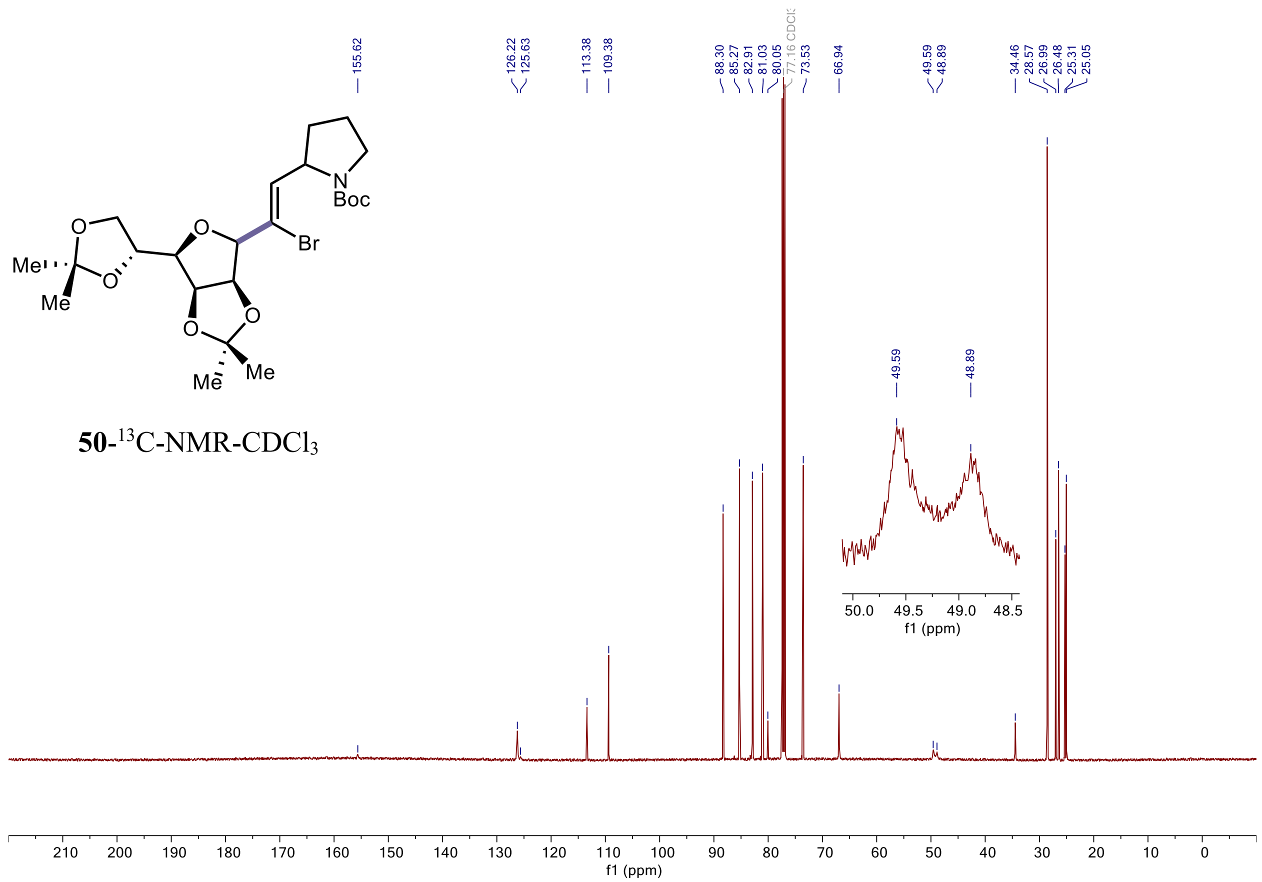
50-13C-NMR-CDCl3
155.62
126.22
125.63
113.38
109.38
88.30
85.27
82.91
81.03
80.05
77.16 CDCl3
73.53
66.94
49.59
48.89
34.46
28.57
26.99
26.48
25.31
25.05
f1 (ppm)

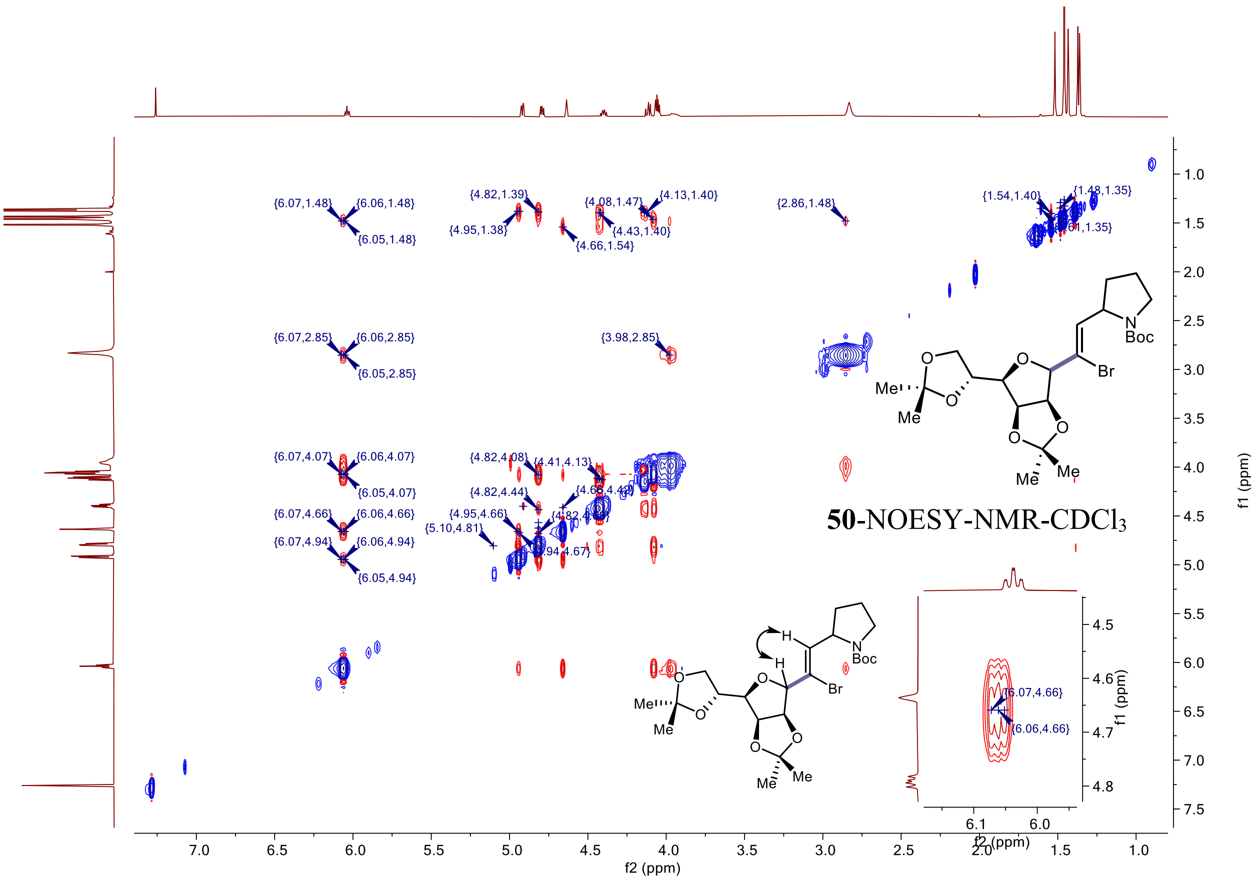
50-NOESY-NMR-CDCl3
f2 (ppm)
f1 (ppm)

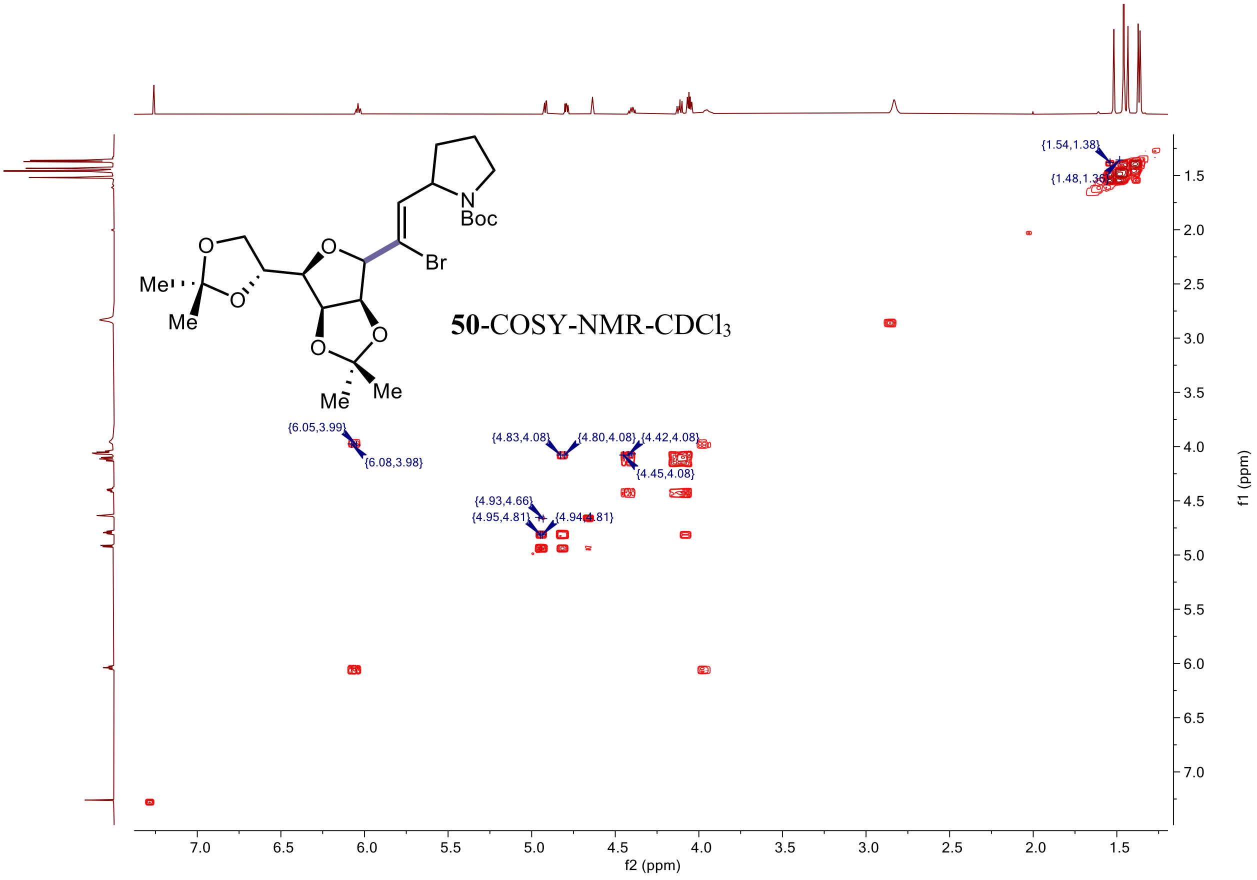
50-COSY-NMR-CDCl3
Boc
N
Br
O
Me
{1.54,1.38}
{1.48,1.36}
{6.05,3.99}
{6.08,3.98}
{4.83,4.08}
{4.80,4.08}
{4.42,4.08}
{4.45,4.08}
{4.93,4.66}
{4.95,4.81}
{4.94,4.81}
f2 (ppm)
f1 (ppm)

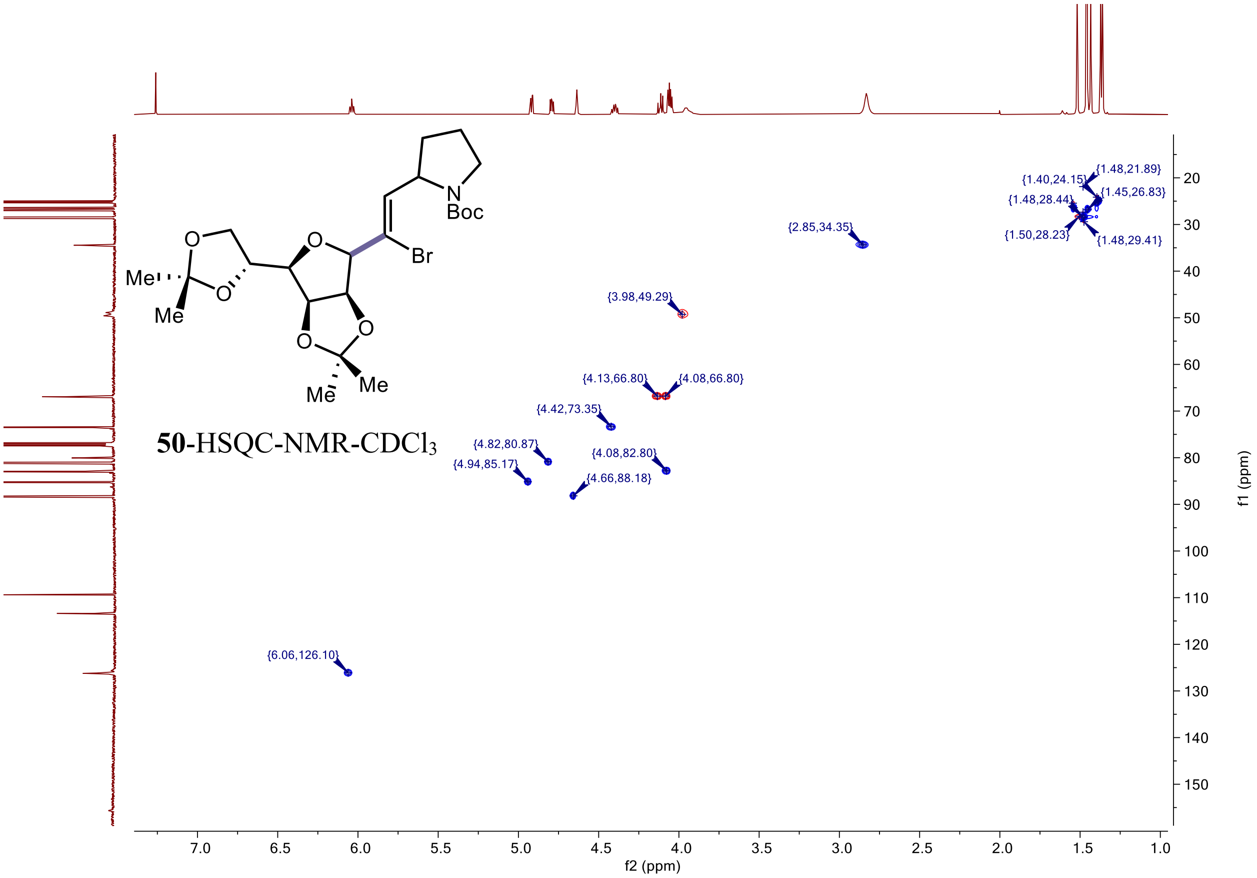
50-HSQC-NMR-CDCl3
Boc
N
Br
O
Me
{1.48,21.89}
{1.40,24.15}
{1.45,26.83}
{1.48,28.44}
{1.50,28.23}
{1.48,29.41}
{2.85,34.35}
{3.98,49.29}
{4.13,66.80}
{4.08,66.80}
{4.42,73.35}
{4.82,80.87}
{4.08,82.80}
{4.94,85.17}
{4.66,88.18}
{6.06,126.10}
f2 (ppm)
f1 (ppm)

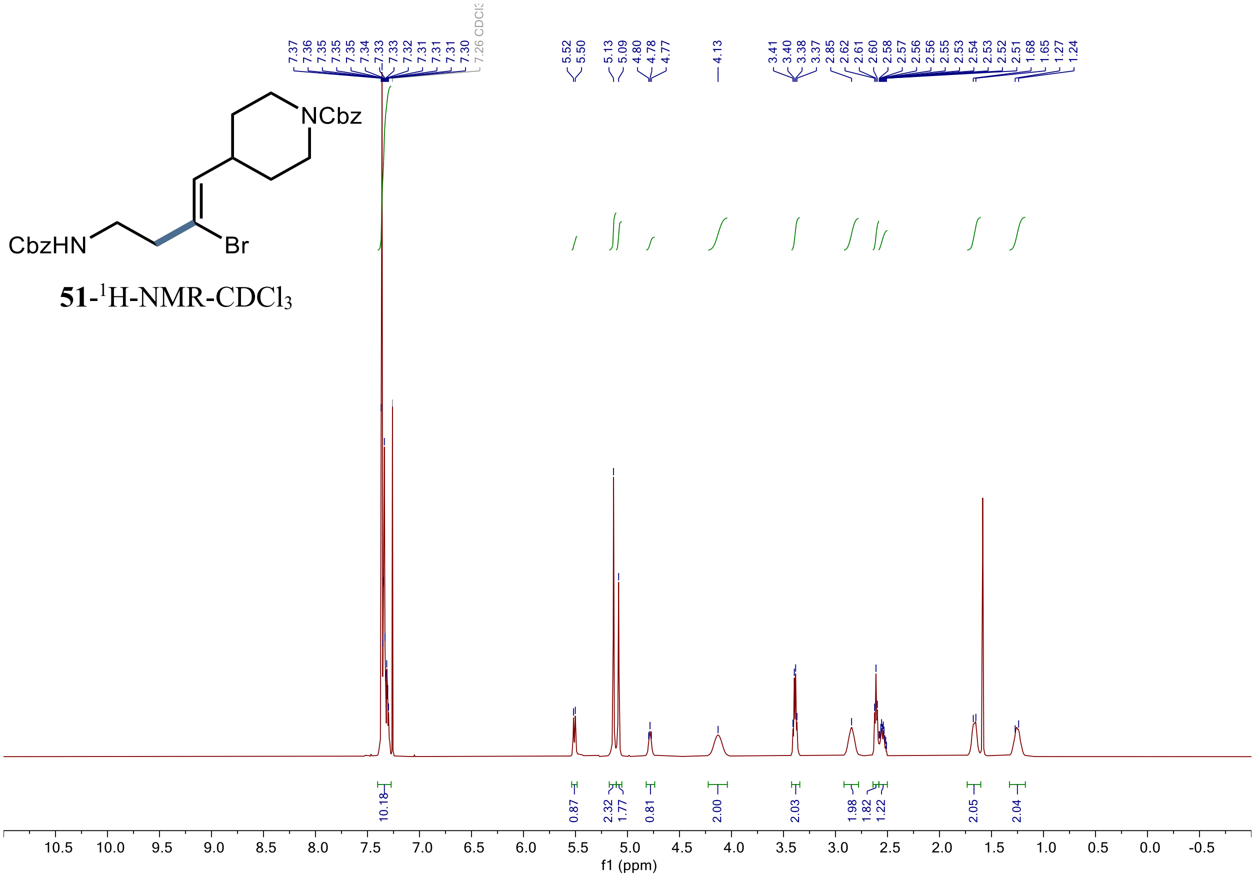

51-^{1}H-NMR-CDCl$_3$

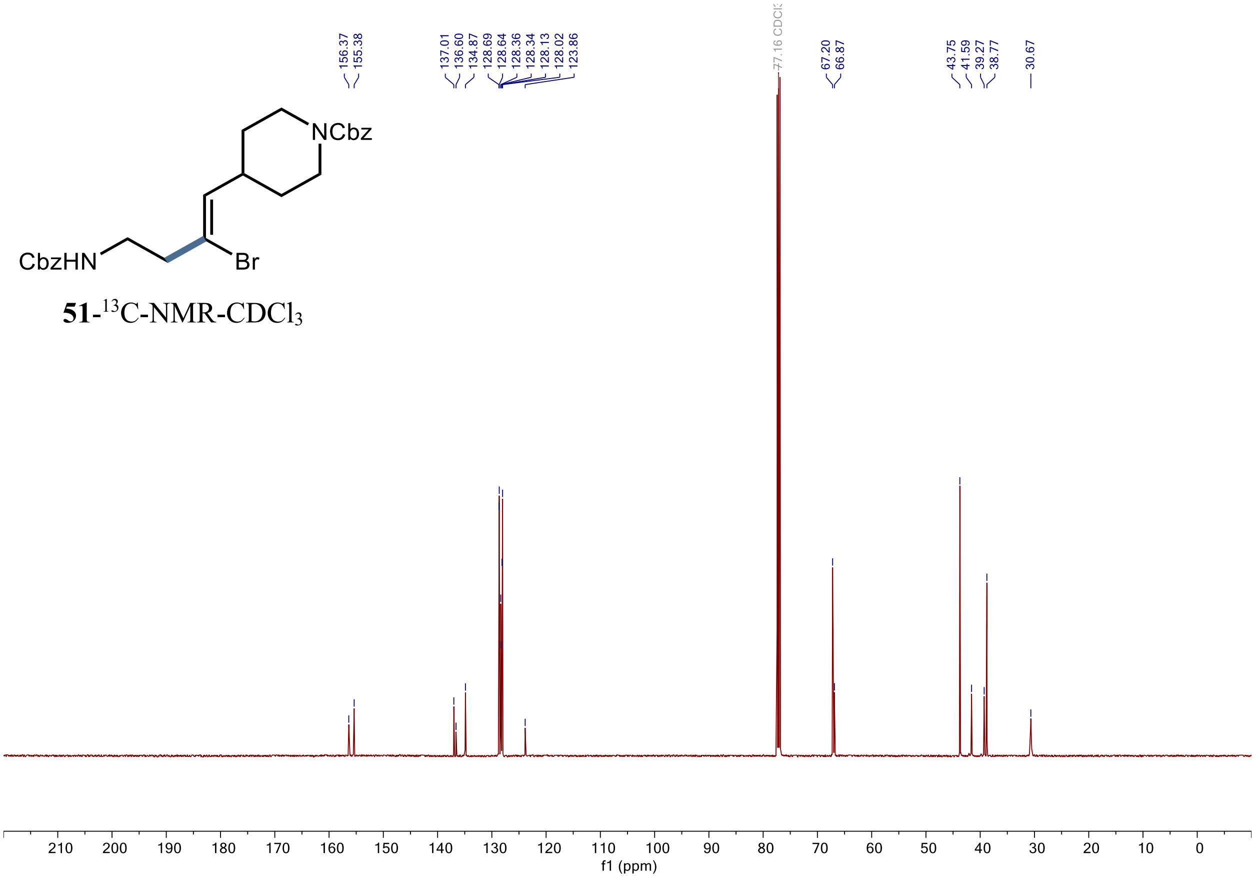

51-^{13}C-NMR-CDCl$_3$

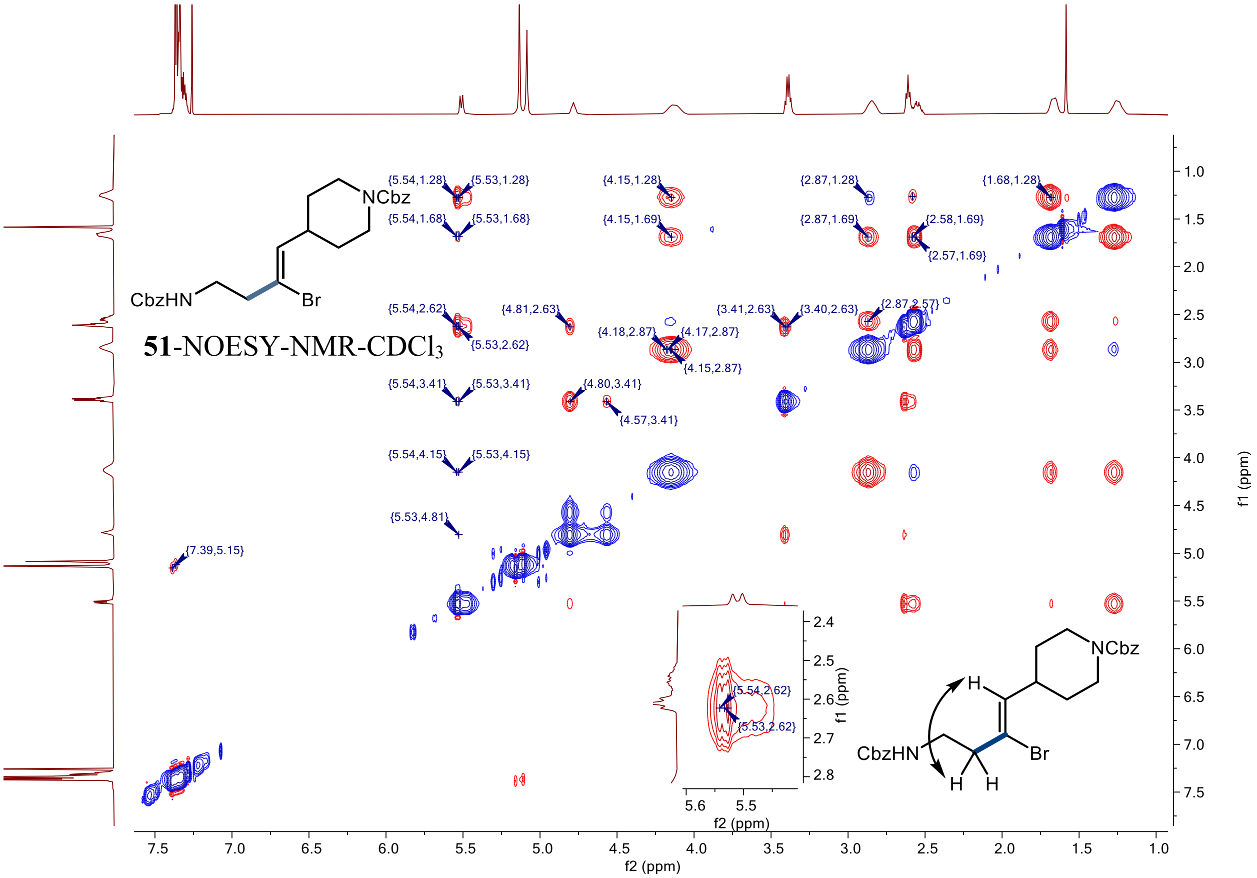
NCbz
CbzHN
Br
51-NOESY-NMR-CDCl3
{5.54,1.28} {5.53,1.28}
{5.54,1.68} {5.53,1.68}
{4.15,1.28}
{4.15,1.69}
{2.87,1.28}
{2.87,1.69}
{1.68,1.28}
{2.58,1.69}
{2.57,1.69}
{5.54,2.62}
{5.53,2.62}
{4.81,2.63}
{4.18,2.87} {4.17,2.87}
{4.15,2.87}
{3.41,2.63} {3.40,2.63}
{2.87,2.57}
{5.54,3.41} {5.53,3.41}
{4.80,3.41}
{4.57,3.41}
{5.54,4.15} {5.53,4.15}
{5.53,4.81}
{7.39,5.15}
{5.54,2.62}
{5.53,2.62}
f1 (ppm)
f2 (ppm)
NCbz
H
CbzHN
Br
H H
f1 (ppm)
f2 (ppm)

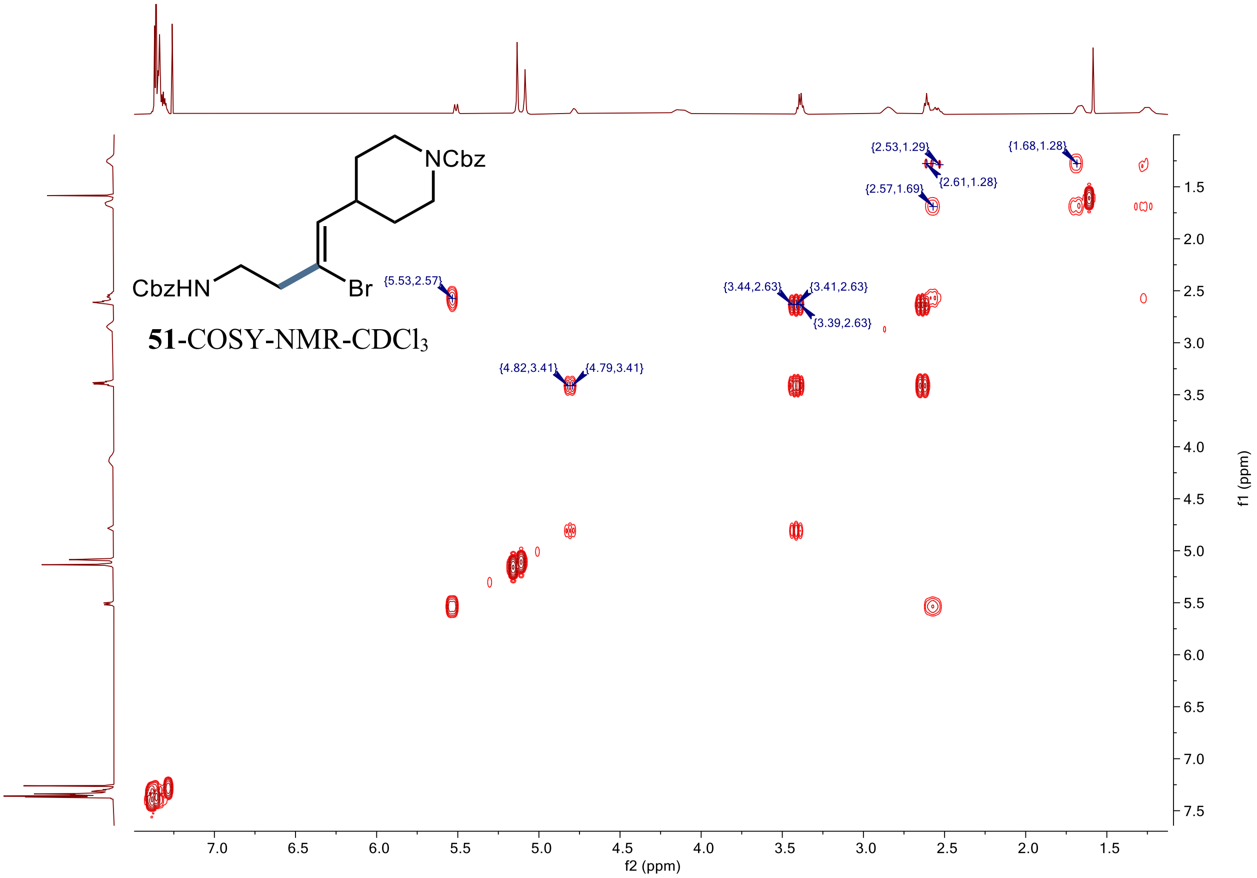
NCbz
CbzHN
Br
51-COSY-NMR-CDCl3
{2.53,1.29}
{2.61,1.28}
{1.68,1.28}
{2.57,1.69}
{5.53,2.57}
{3.44,2.63} {3.41,2.63}
{3.39,2.63}
{4.82,3.41} {4.79,3.41}
f1 (ppm)
f2 (ppm)

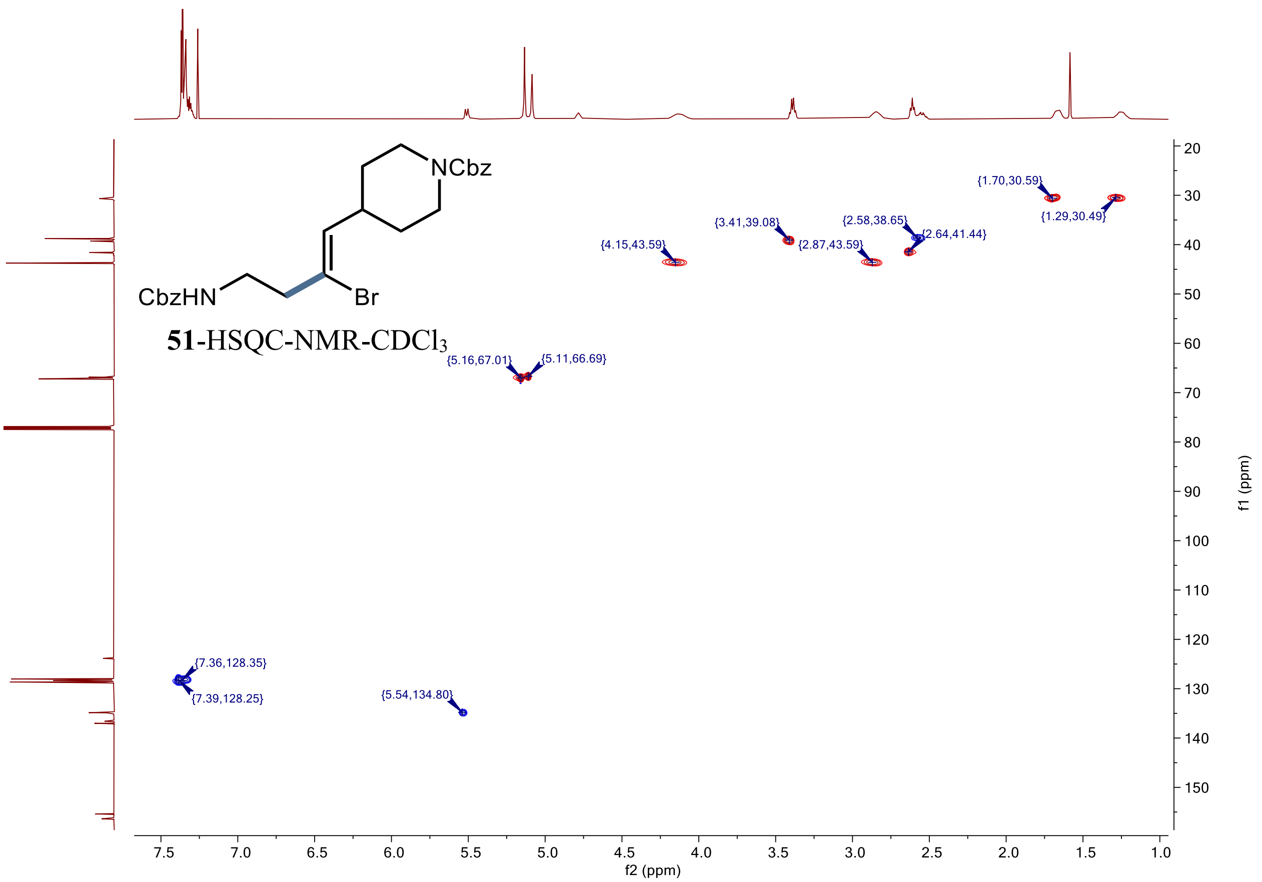

NCbz
CbzHN
Br
51-HSQC-NMR-CDCl3
{1.70,30.59}
{1.29,30.49}
{3.41,39.08}
{2.58,38.65}
{2.64,41.44}
{4.15,43.59}
{2.87,43.59}
{5.16,67.01}
{5.11,66.69}
{7.36,128.35}
{7.39,128.25}
{5.54,134.80}
f2 (ppm)
f1 (ppm)

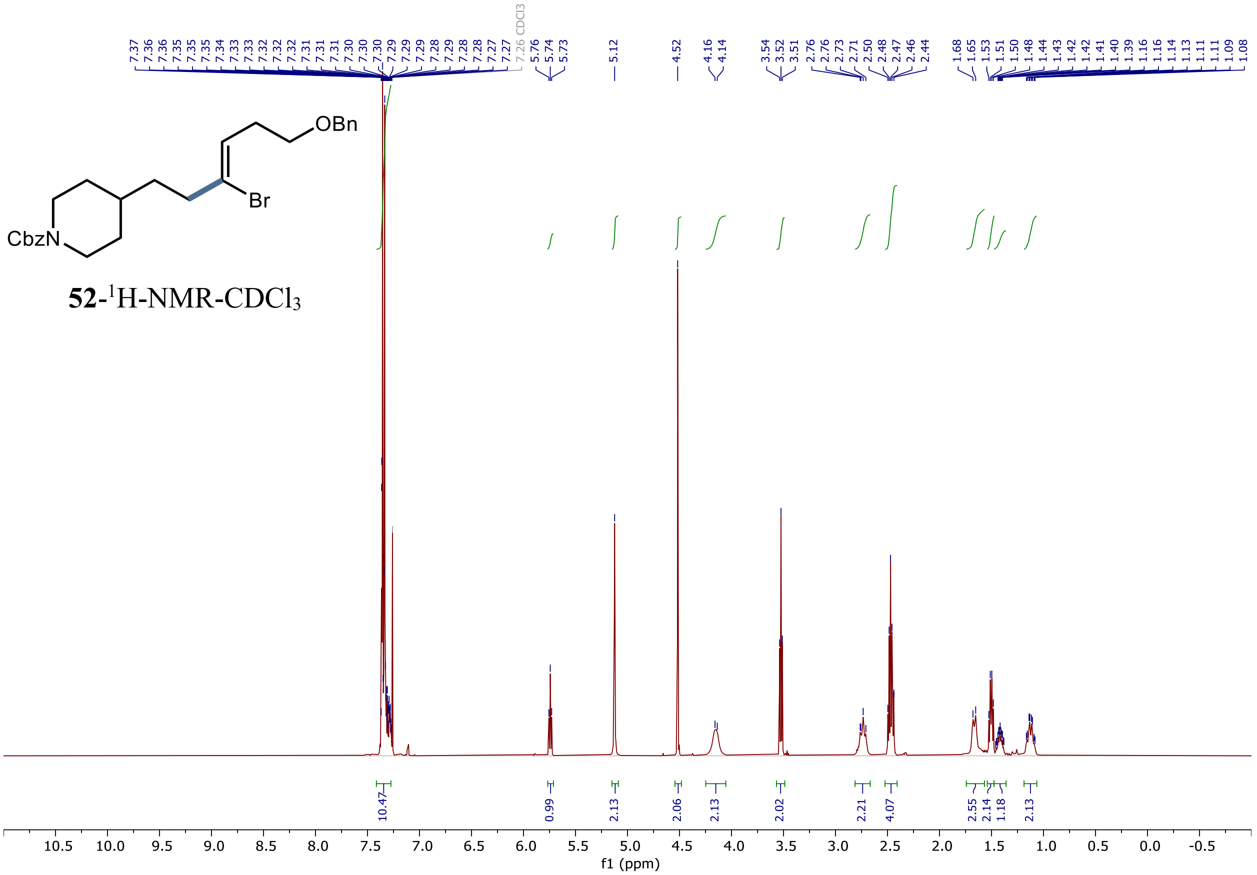

OBn
Br
CbzN
52-1H-NMR-CDCl3
7.26 CDCl3
10.47
0.99
2.13
2.06
2.13
2.02
2.21
4.07
2.55
2.14
1.18
2.13
f1 (ppm)

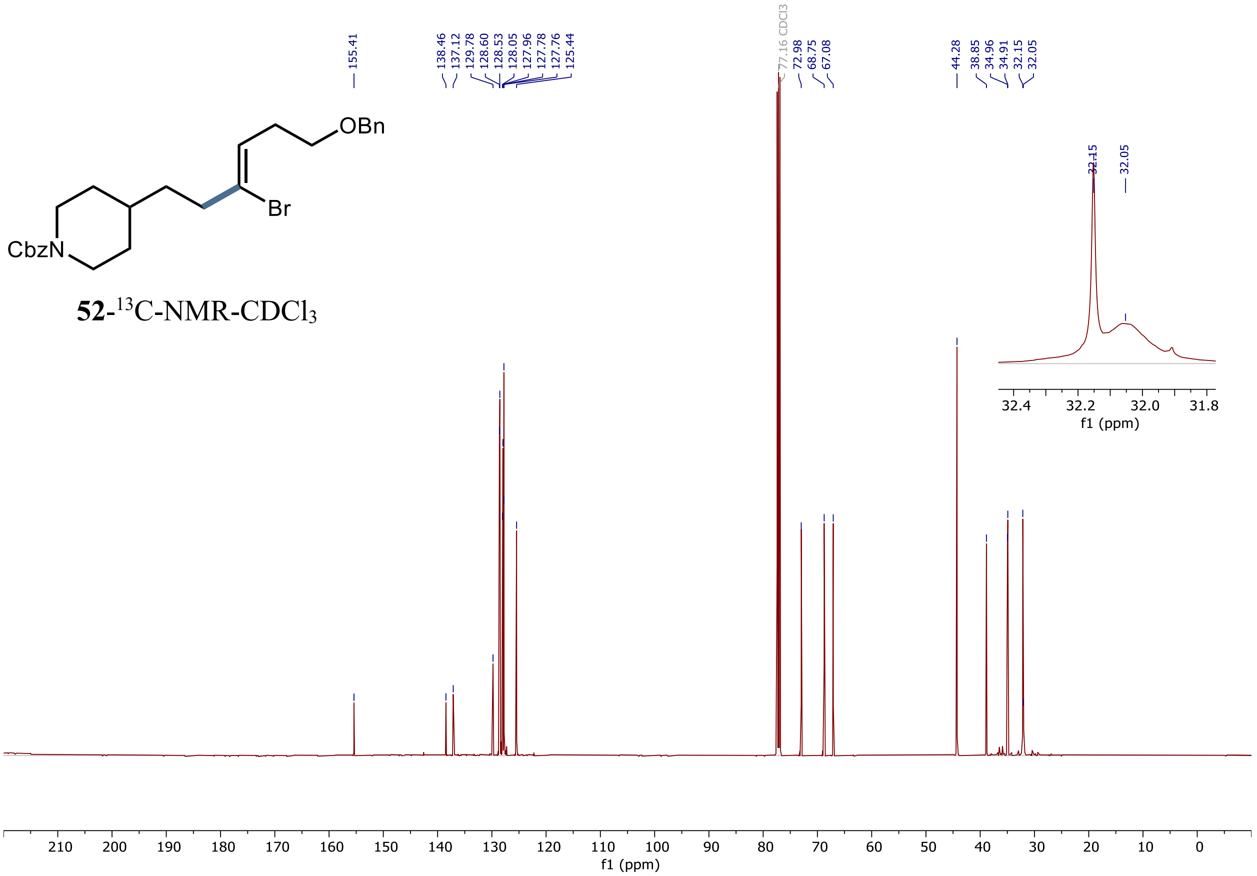

52-^{13}C-NMR-CDCl$_3$

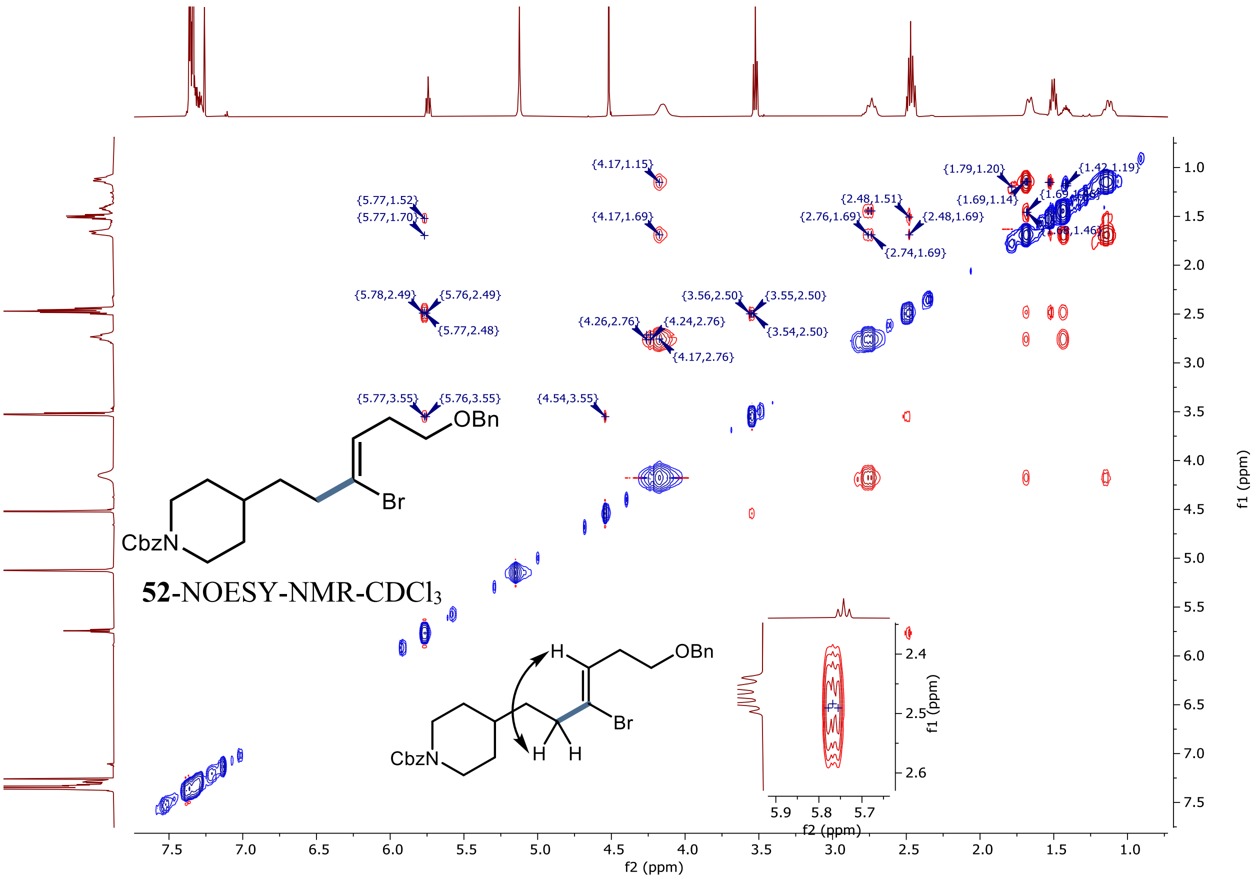

52-NOESY-NMR-CDCl$_3$

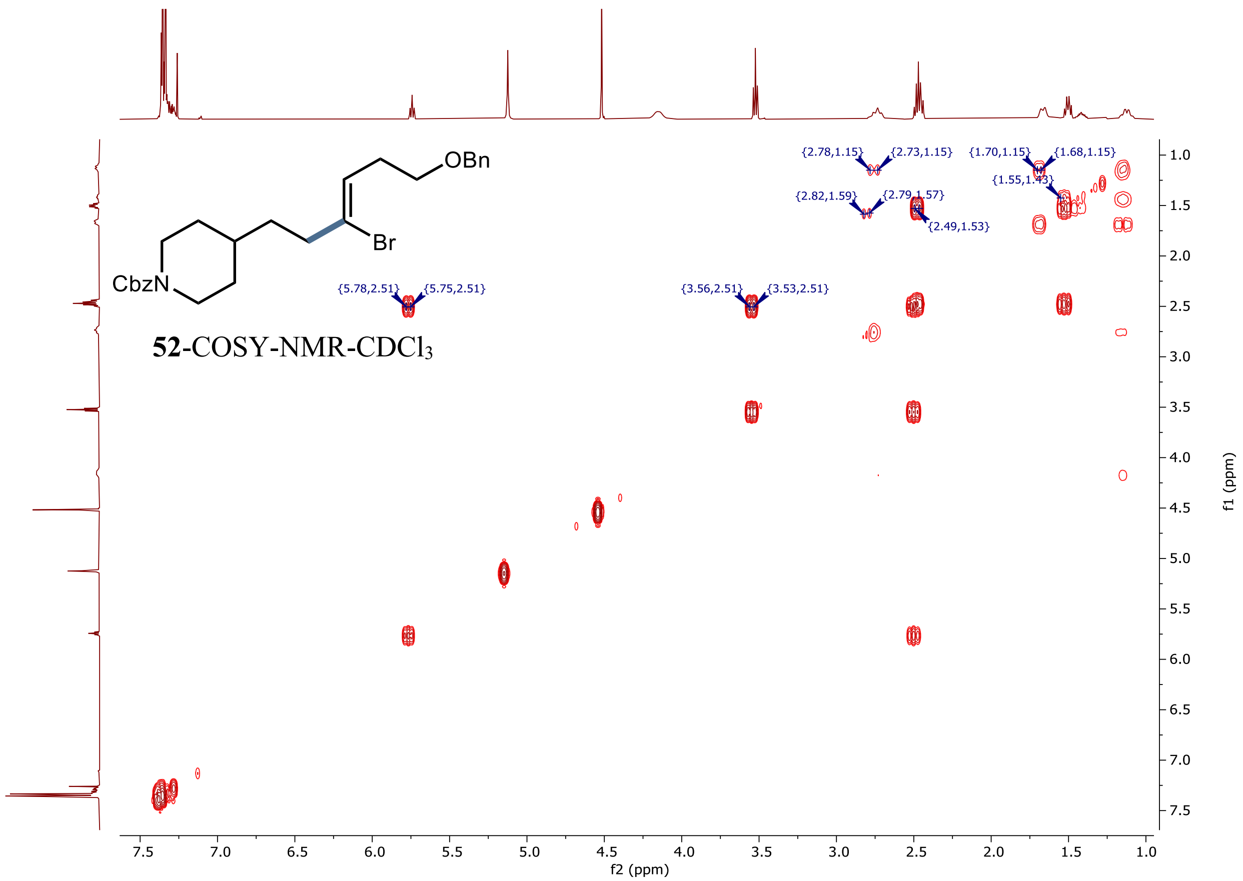
OBn
Br
CbzN
52-COSY-NMR-CDCl3
{2.78,1.15}
{2.73,1.15}
{1.70,1.15}
{1.68,1.15}
{1.55,1.43}
{2.82,1.59}
{2.79,1.57}
{2.49,1.53}
{5.78,2.51}
{5.75,2.51}
{3.56,2.51}
{3.53,2.51}
f2 (ppm)
f1 (ppm)

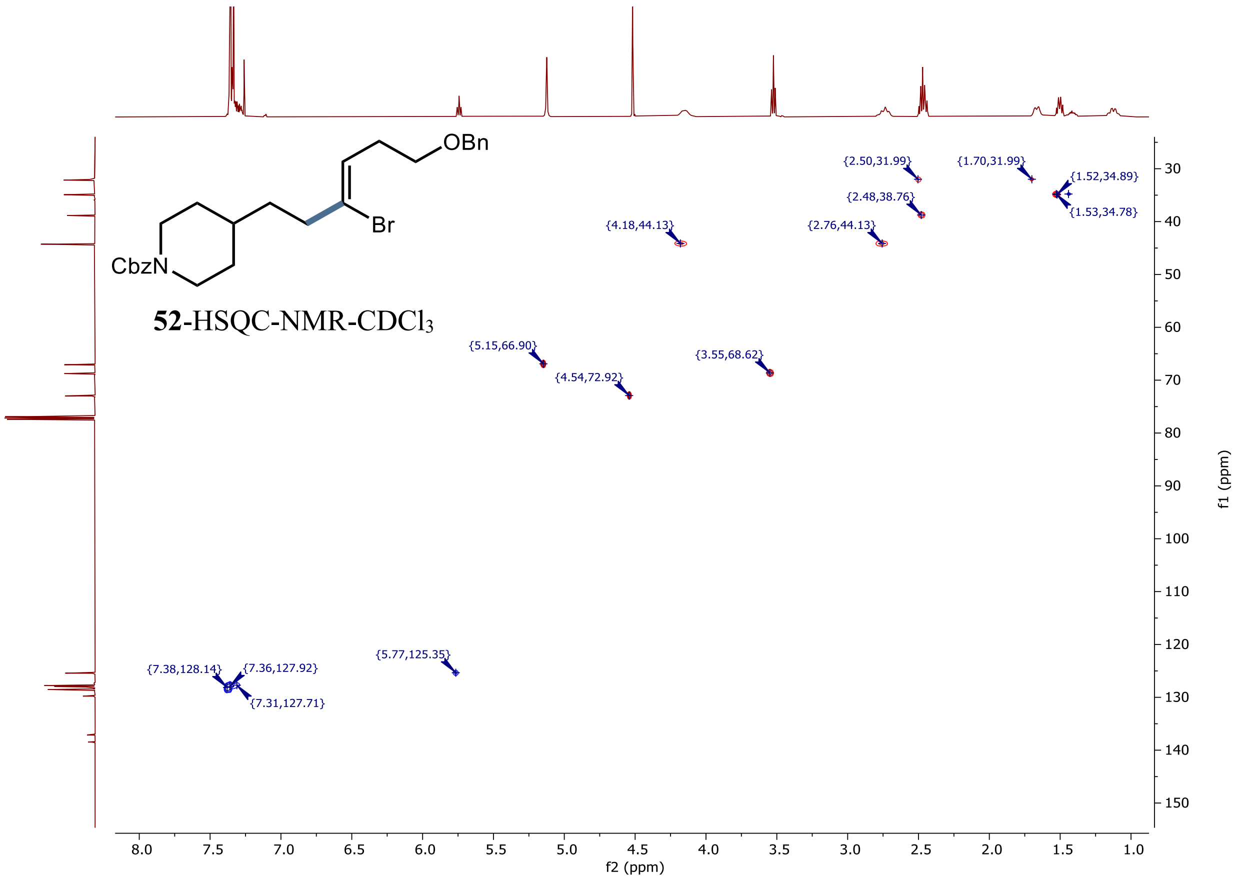
OBn
Br
CbzN
52-HSQC-NMR-CDCl3
{2.50,31.99}
{1.70,31.99}
{1.52,34.89}
{2.48,38.76}
{1.53,34.78}
{4.18,44.13}
{2.76,44.13}
{5.15,66.90}
{3.55,68.62}
{4.54,72.92}
{5.77,125.35}
{7.38,128.14}
{7.36,127.92}
{7.31,127.71}
f2 (ppm)
f1 (ppm)

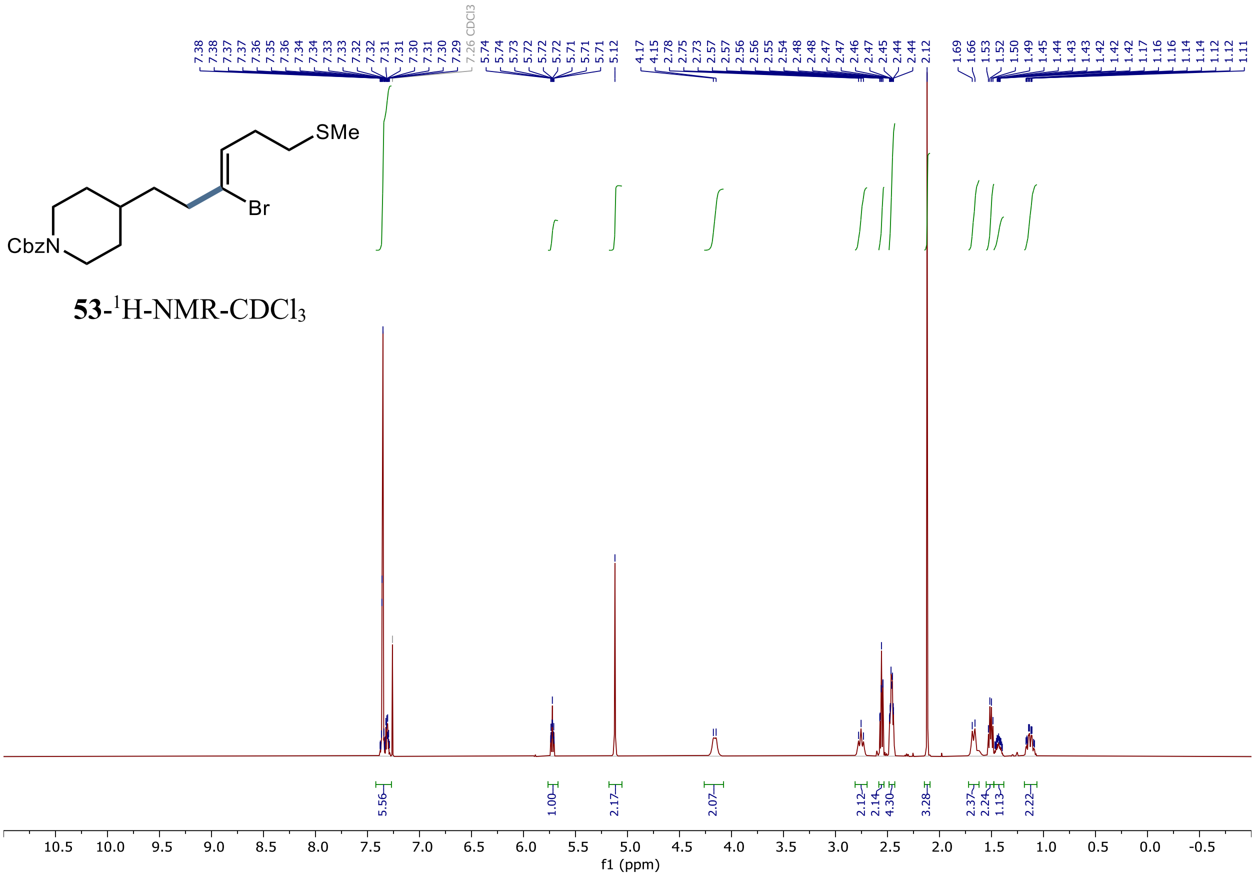
53-^{1}H-NMR-CDCl$_3$
SMe
Br
CbzN
f1 (ppm)

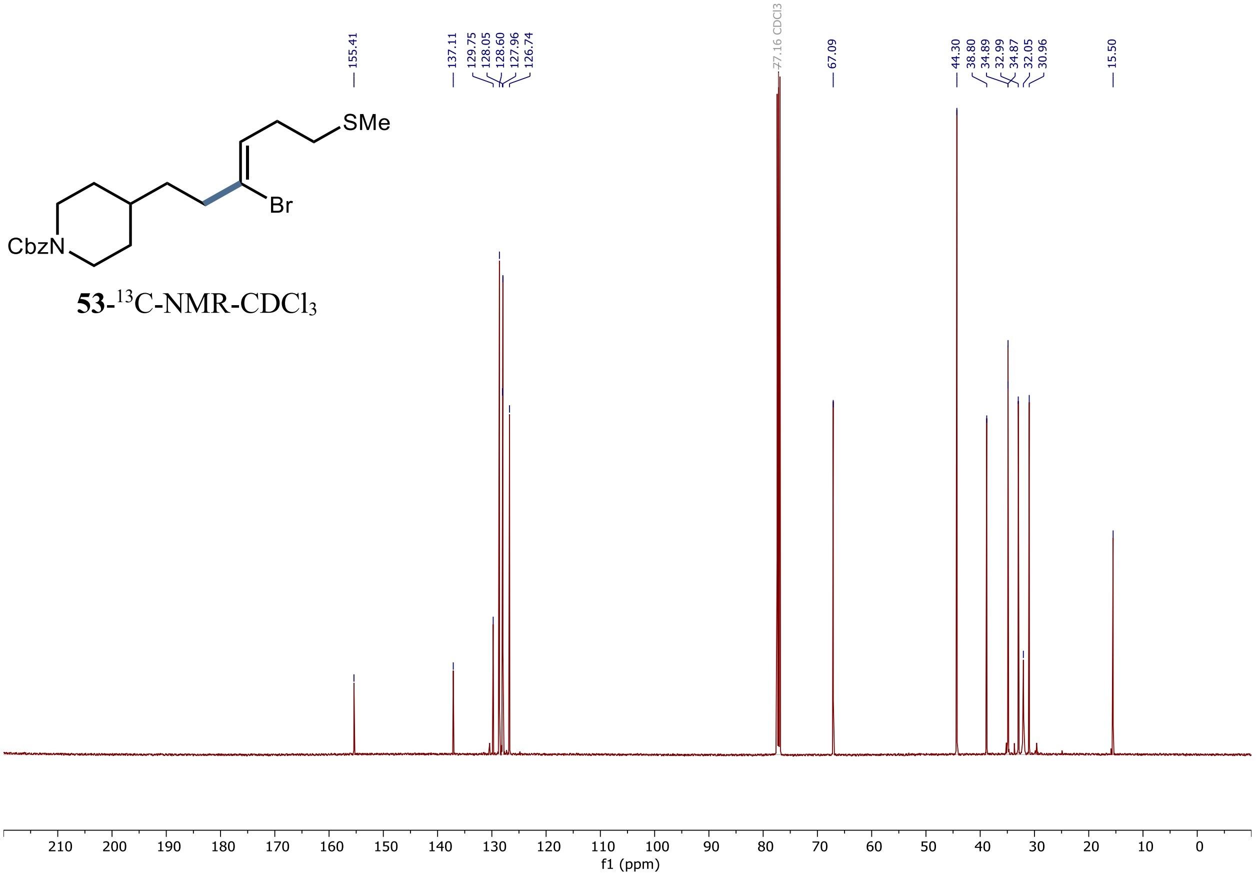
53-^{13}C-NMR-CDCl$_3$
SMe
Br
CbzN
f1 (ppm)

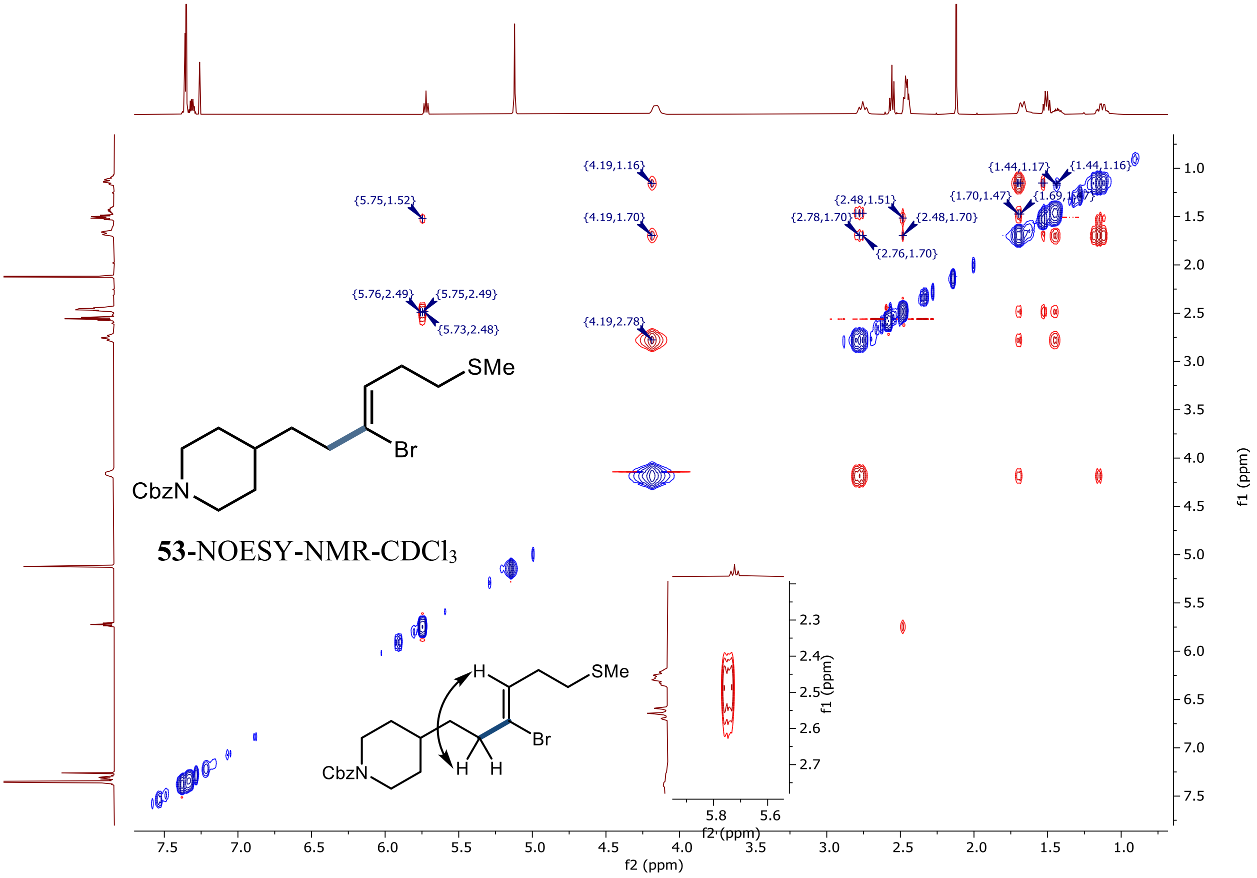
SMe
CbzN
Br
53-NOESY-NMR-CDCl3
H
H H
f2 (ppm)
f1 (ppm)

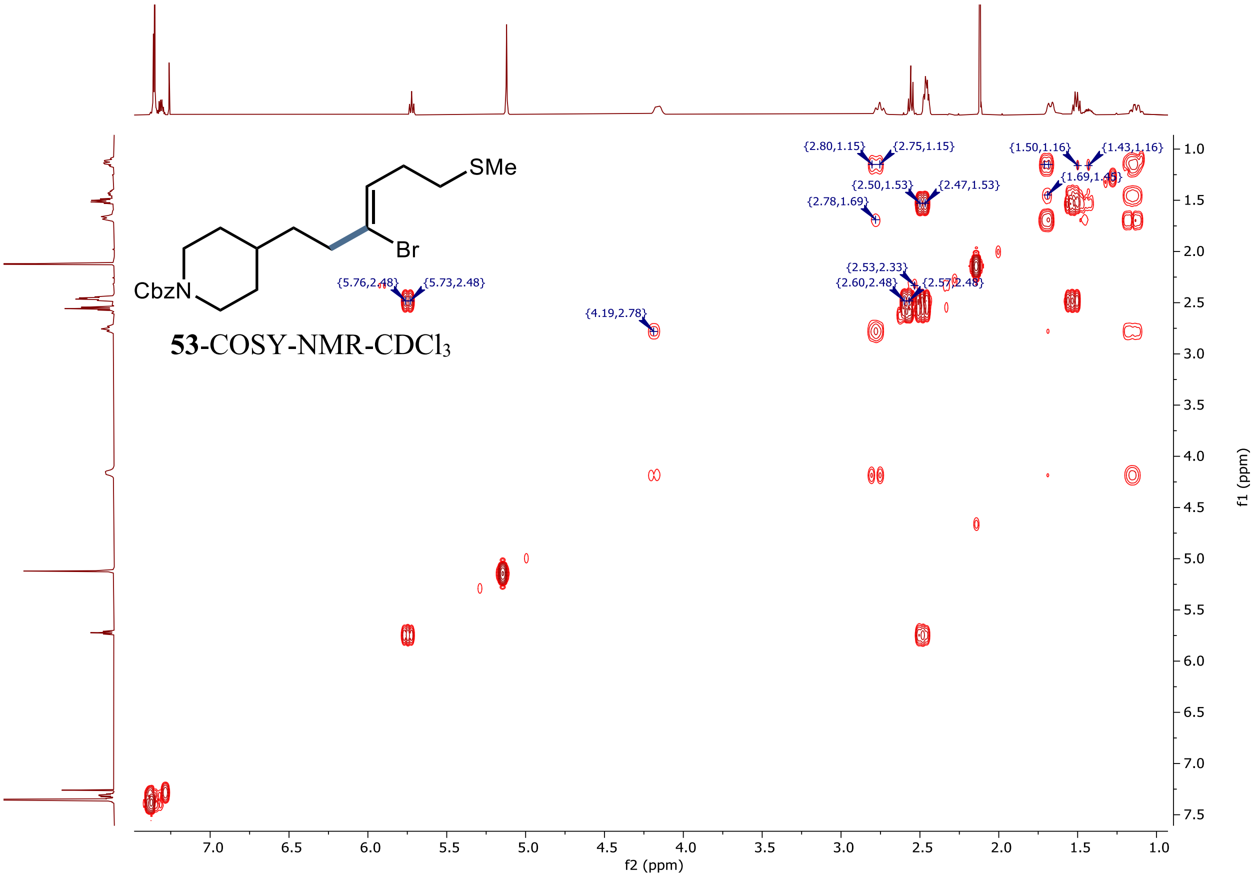
SMe
CbzN
Br
53-COSY-NMR-CDCl3
f2 (ppm)
f1 (ppm)

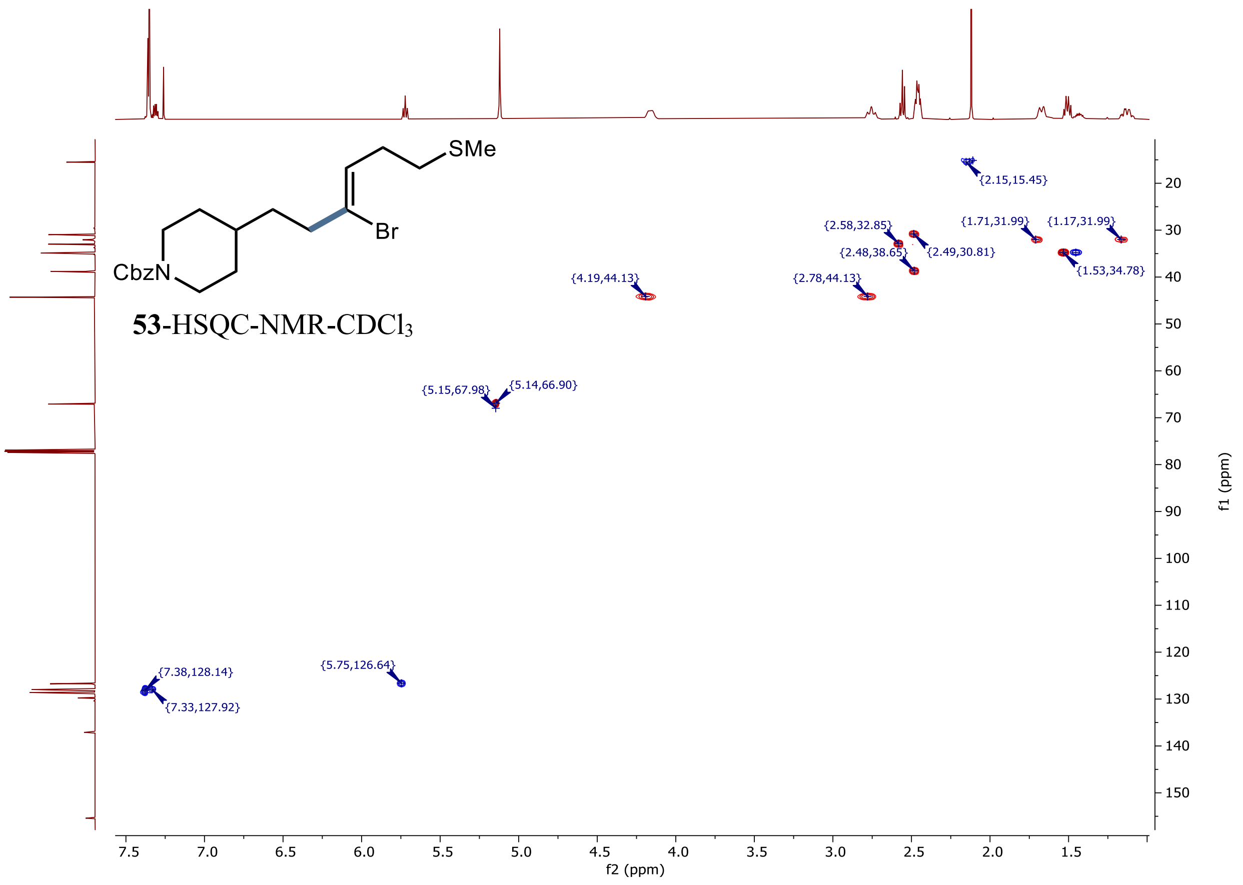

53-HSQC-NMR-$CDCl_3$

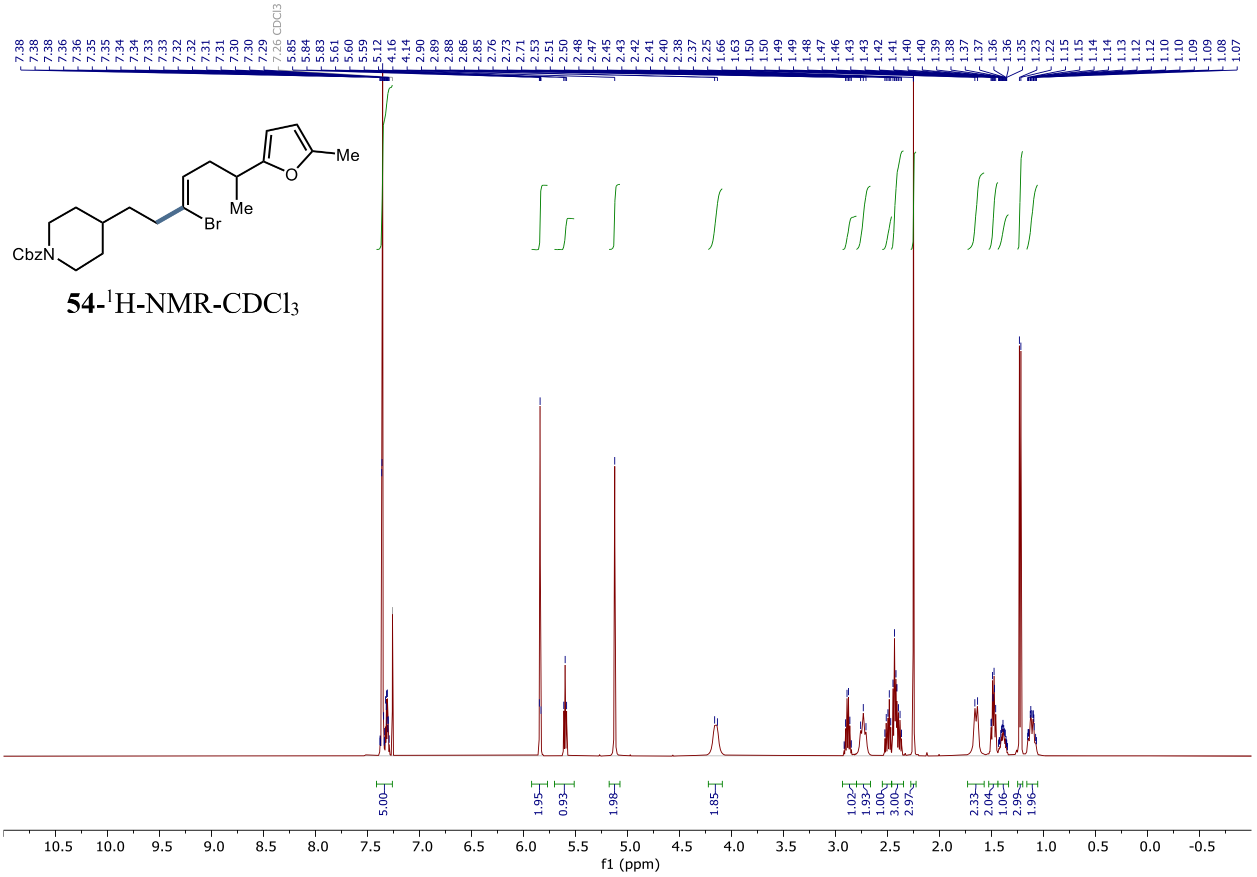

54-^1H-NMR-$CDCl_3$

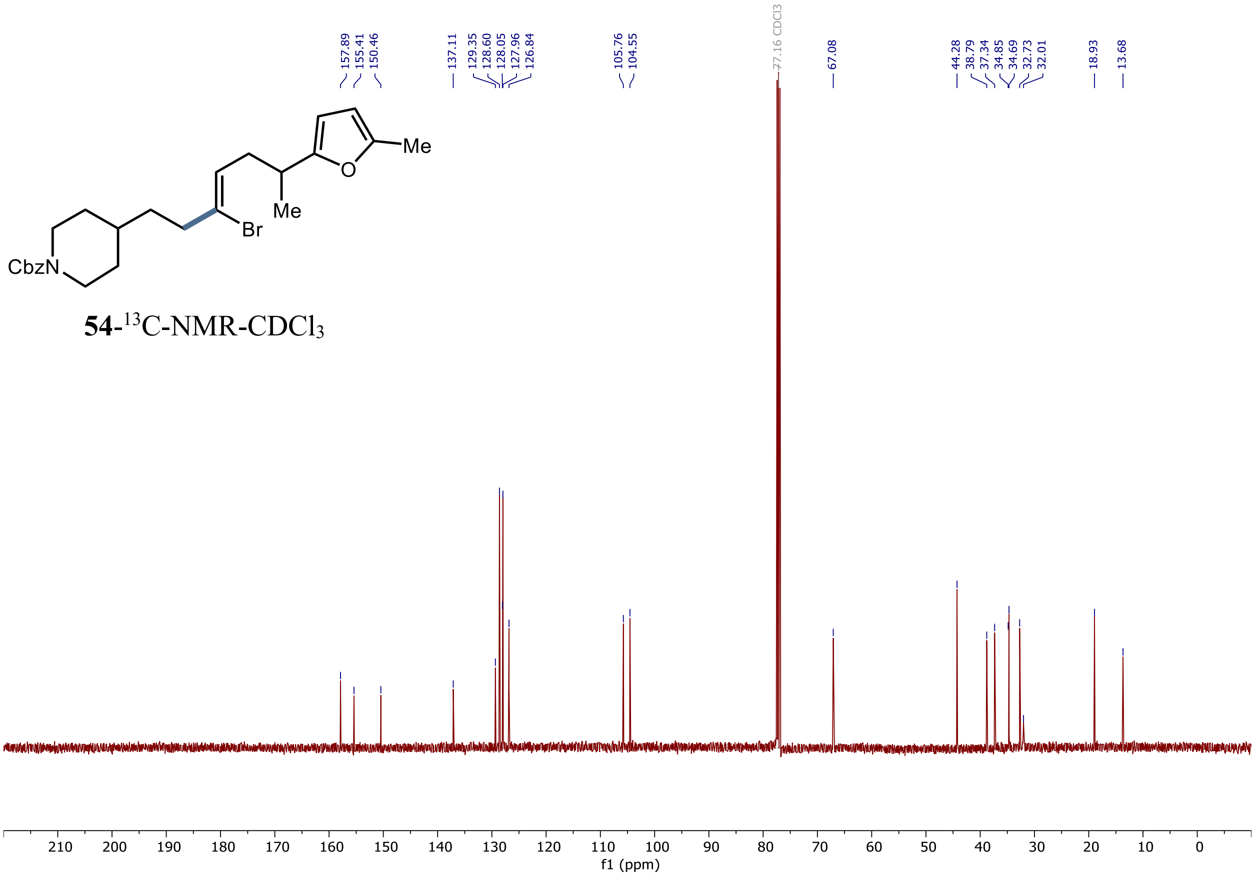

54-^{13}C-NMR-$CDCl_3$

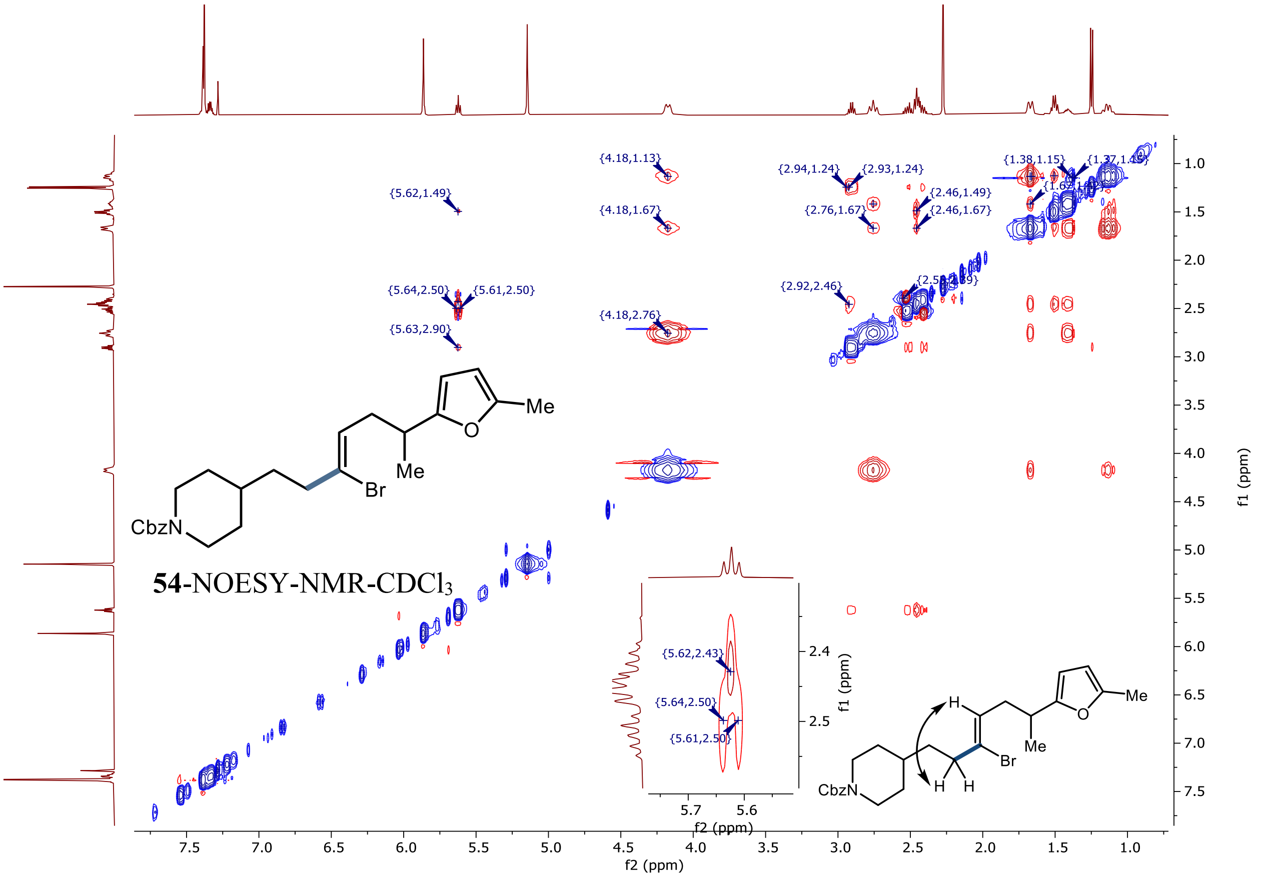

54-NOESY-NMR-$CDCl_3$

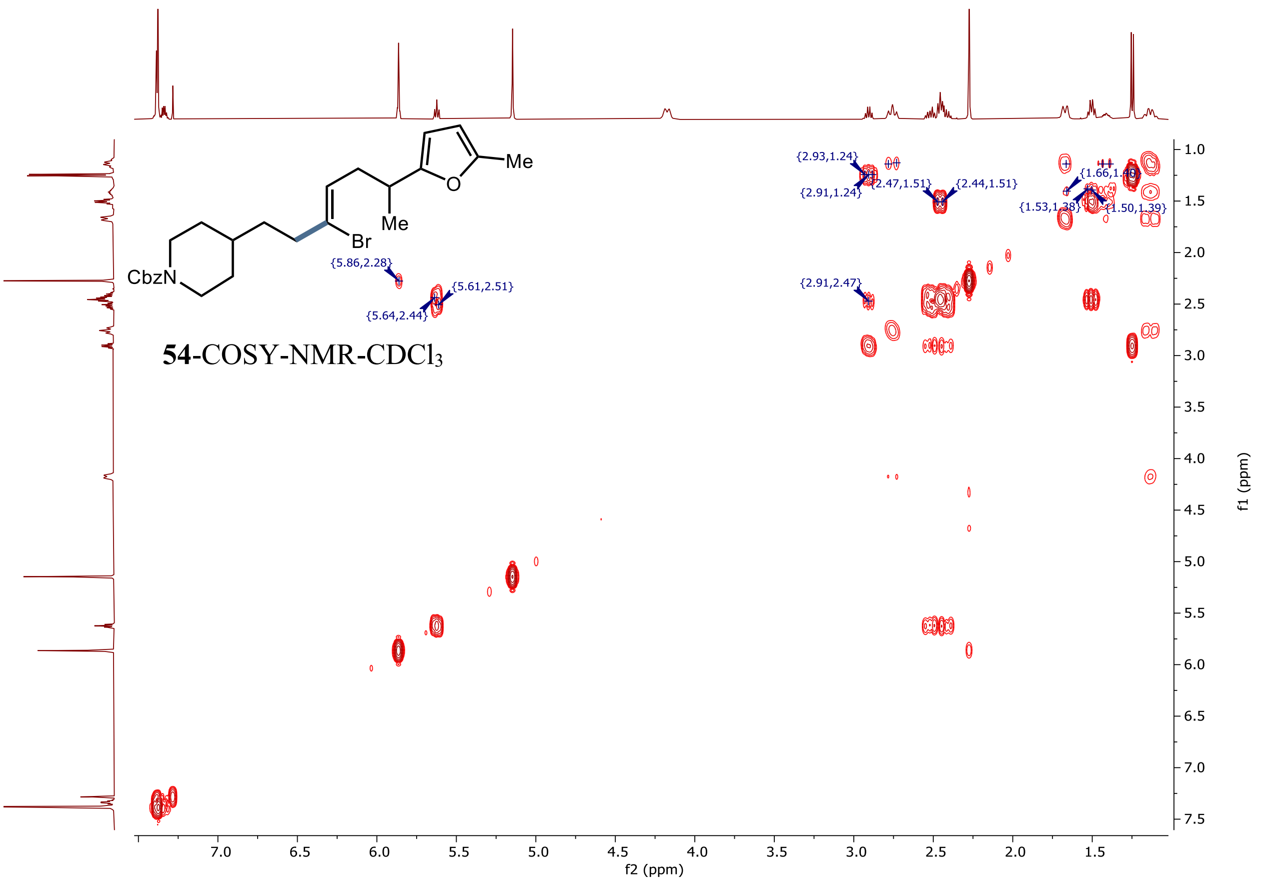
54-COSY-NMR-CDCl3
f2 (ppm)
f1 (ppm)

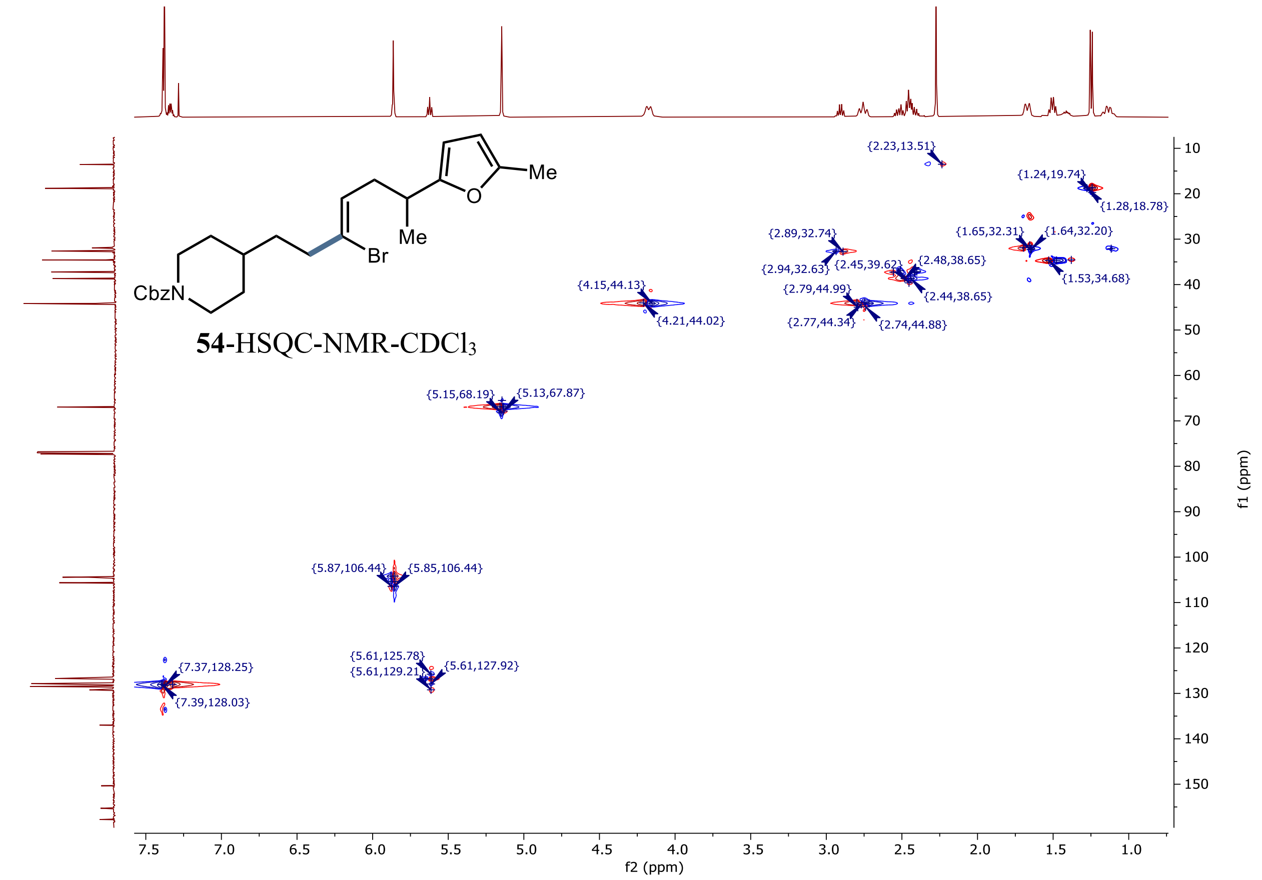
54-HSQC-NMR-CDCl3
f2 (ppm)
f1 (ppm)

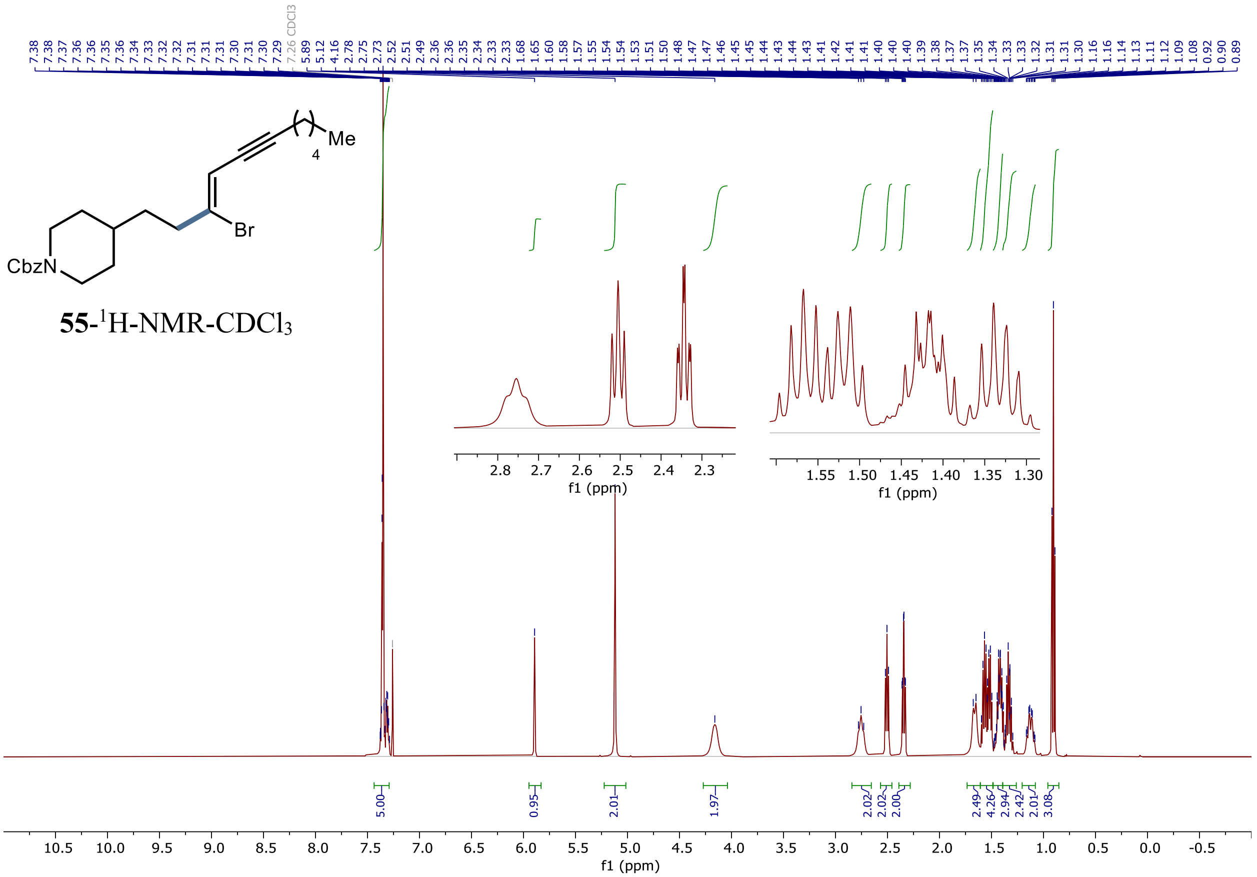

55-¹H-NMR-CDCl$_3$

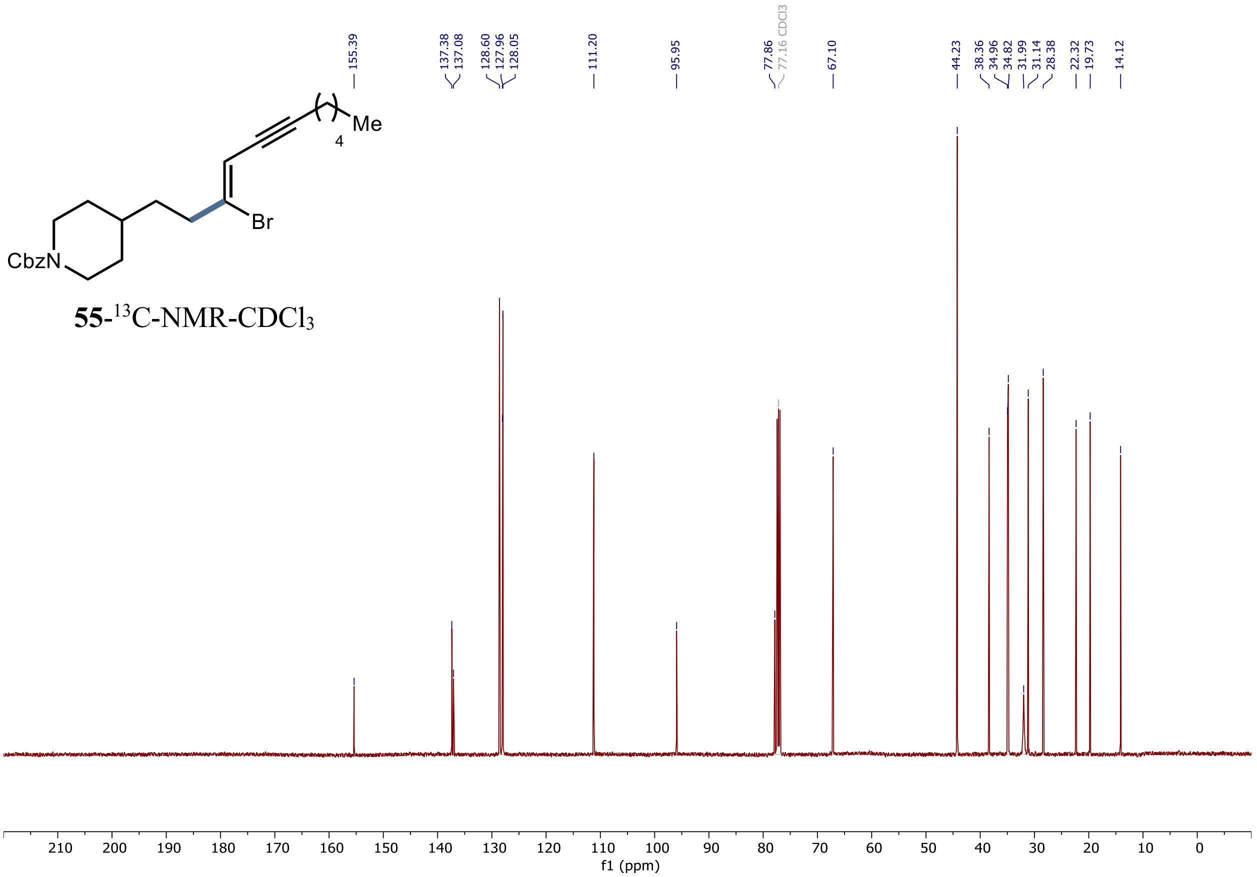

55-¹³C-NMR-CDCl$_3$

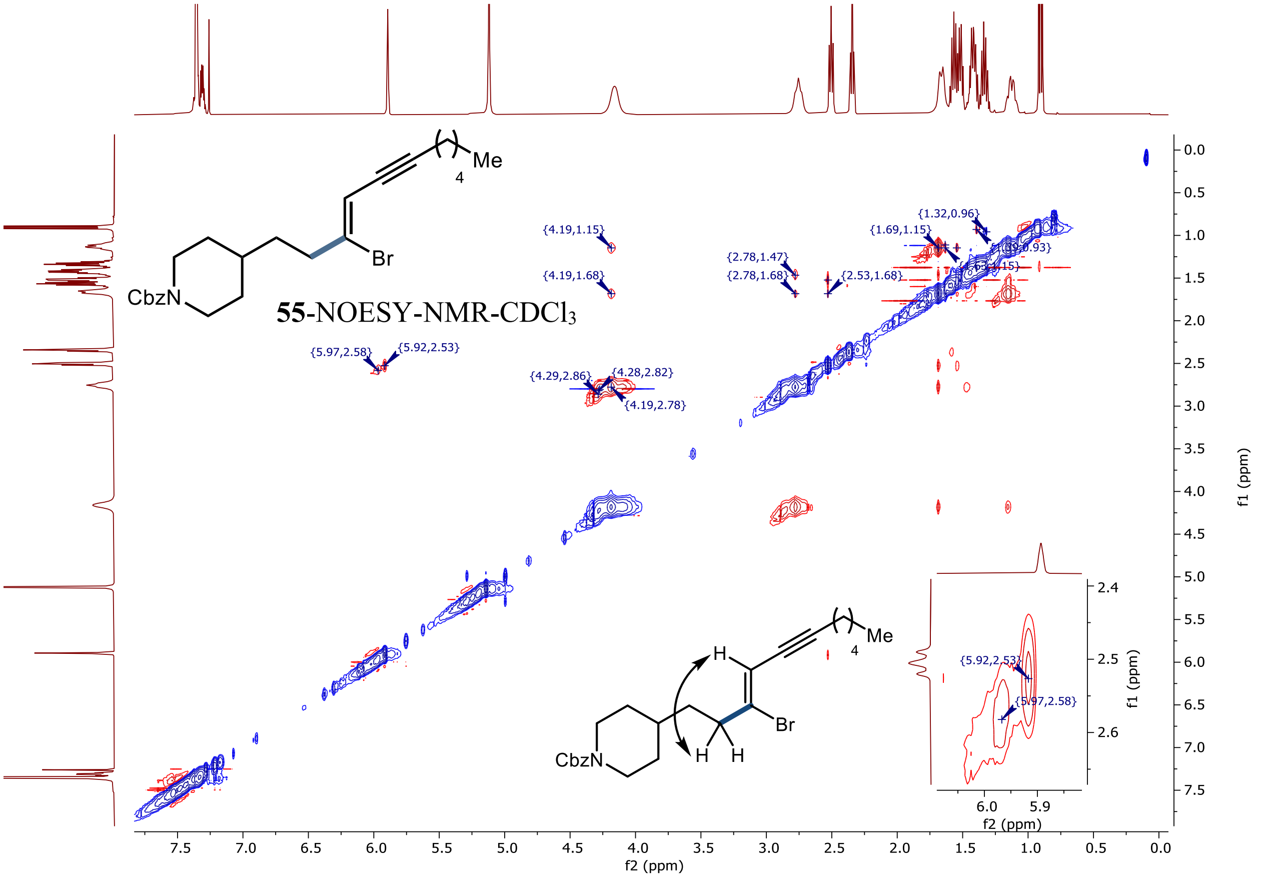
Me
4
Br
CbzN
55-NOESY-NMR-CDCl3
H
H H
f2 (ppm)
f1 (ppm)

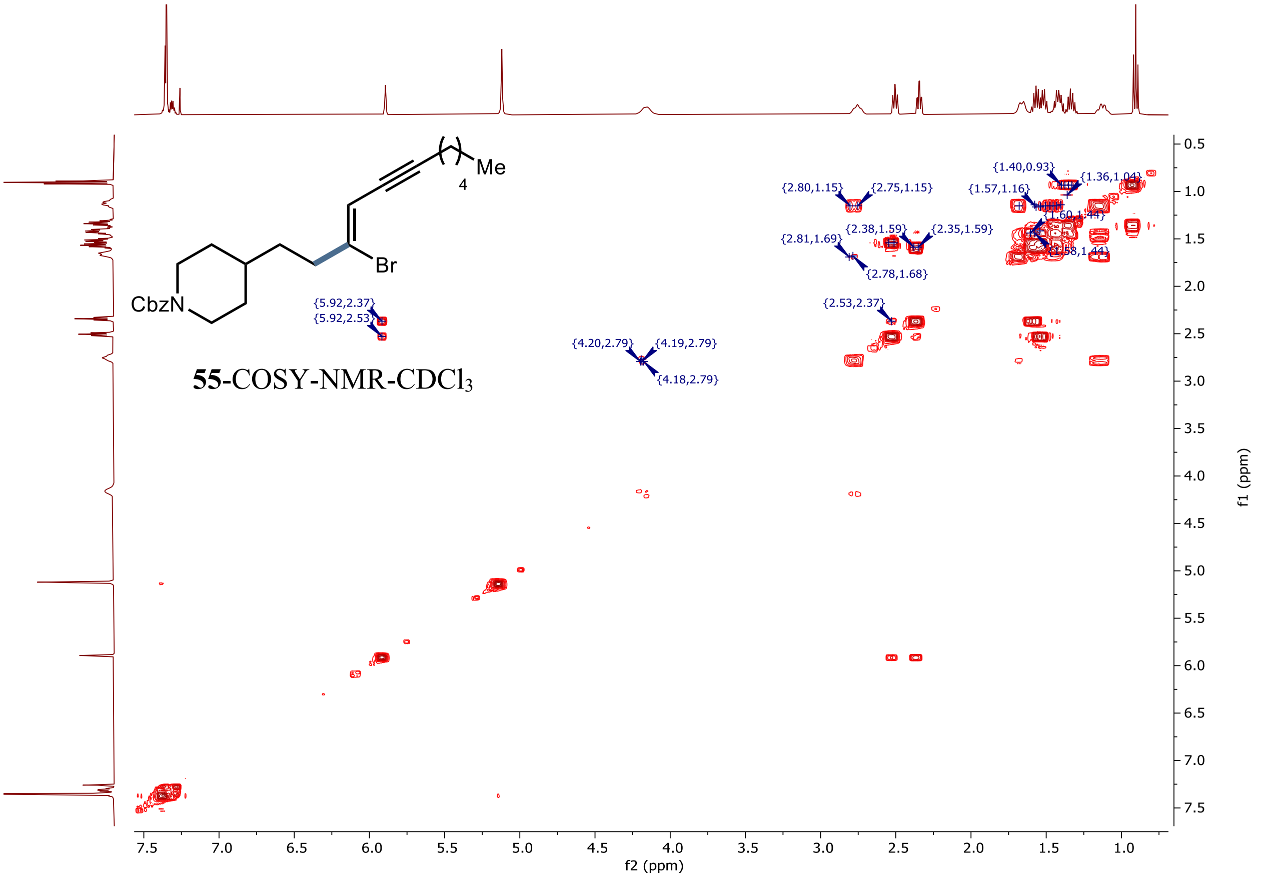
Me
4
Br
CbzN
55-COSY-NMR-CDCl3
f2 (ppm)
f1 (ppm)

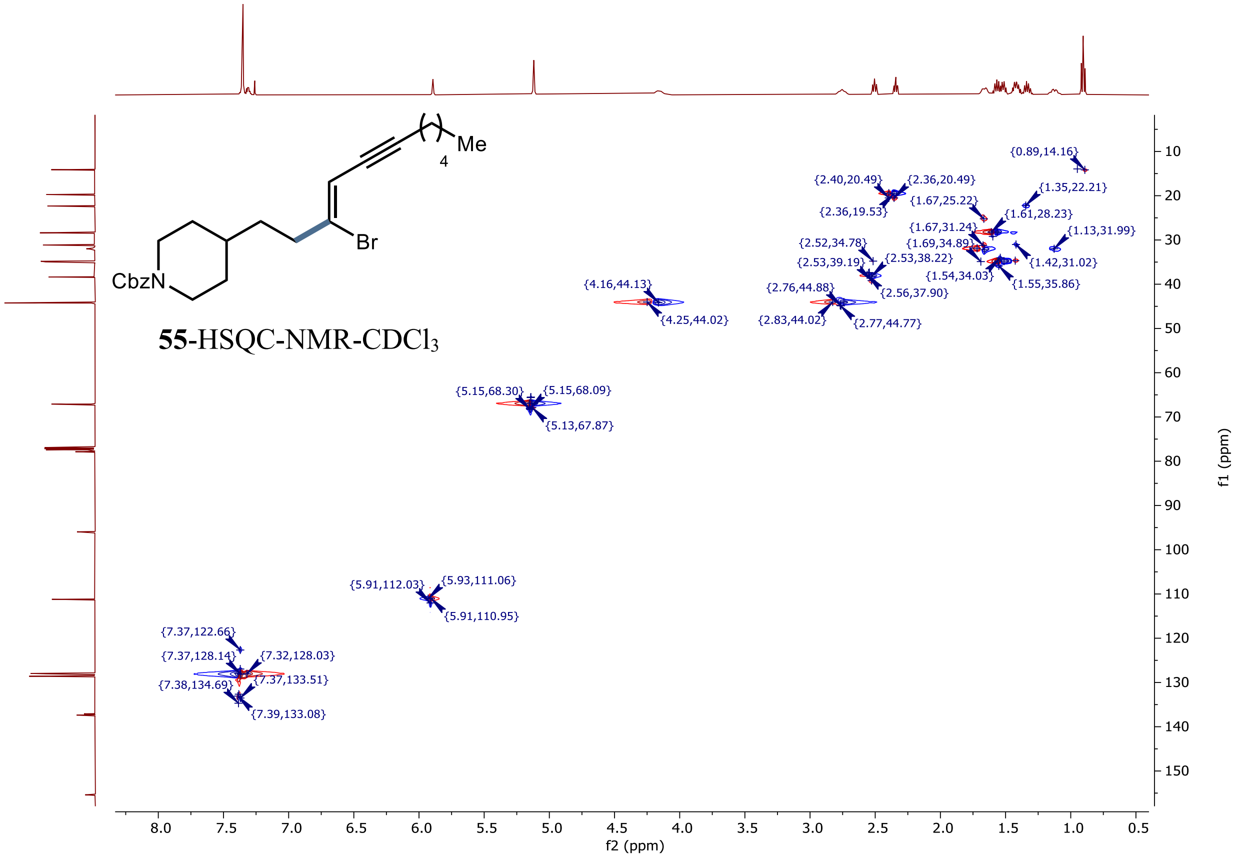

55-HSQC-NMR-$CDCl_3$

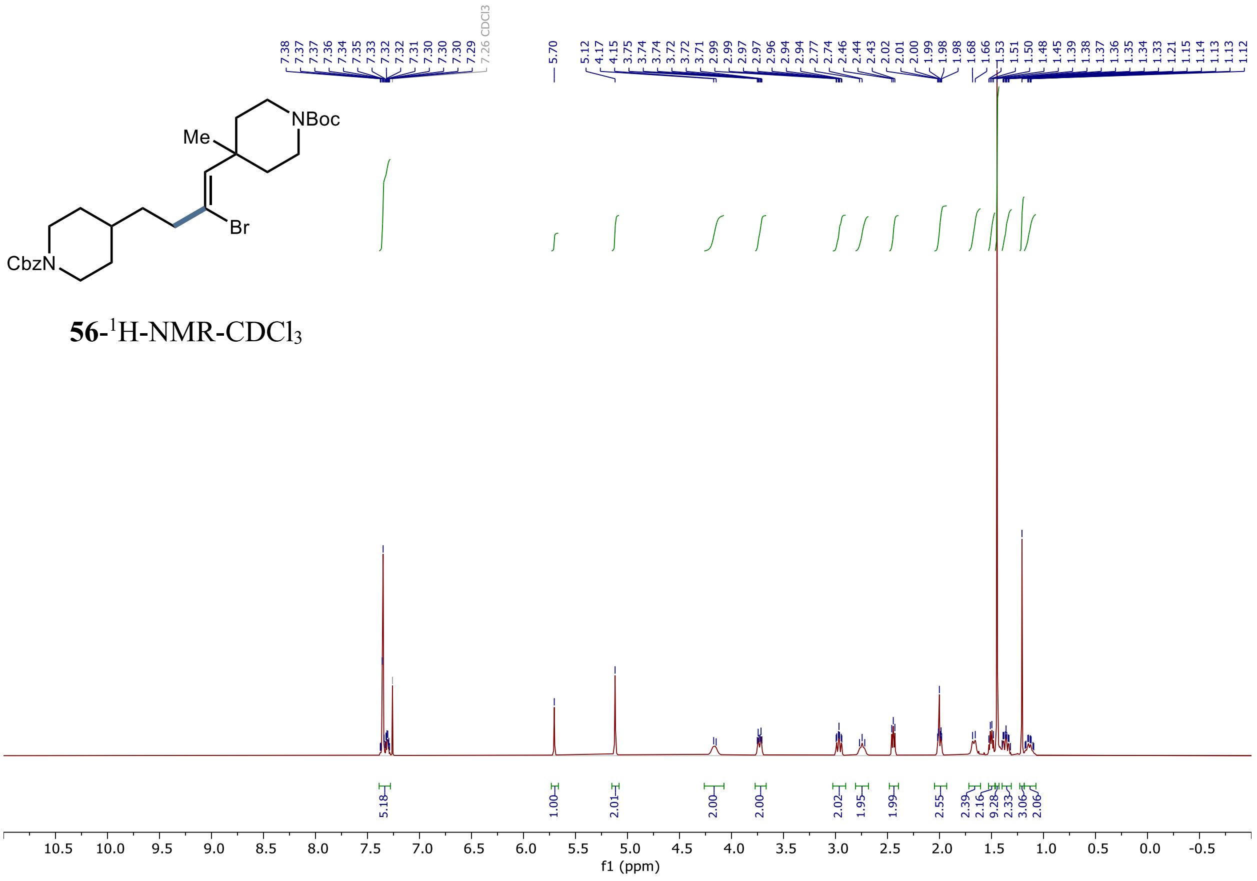

56-^{1}H-NMR-$CDCl_3$

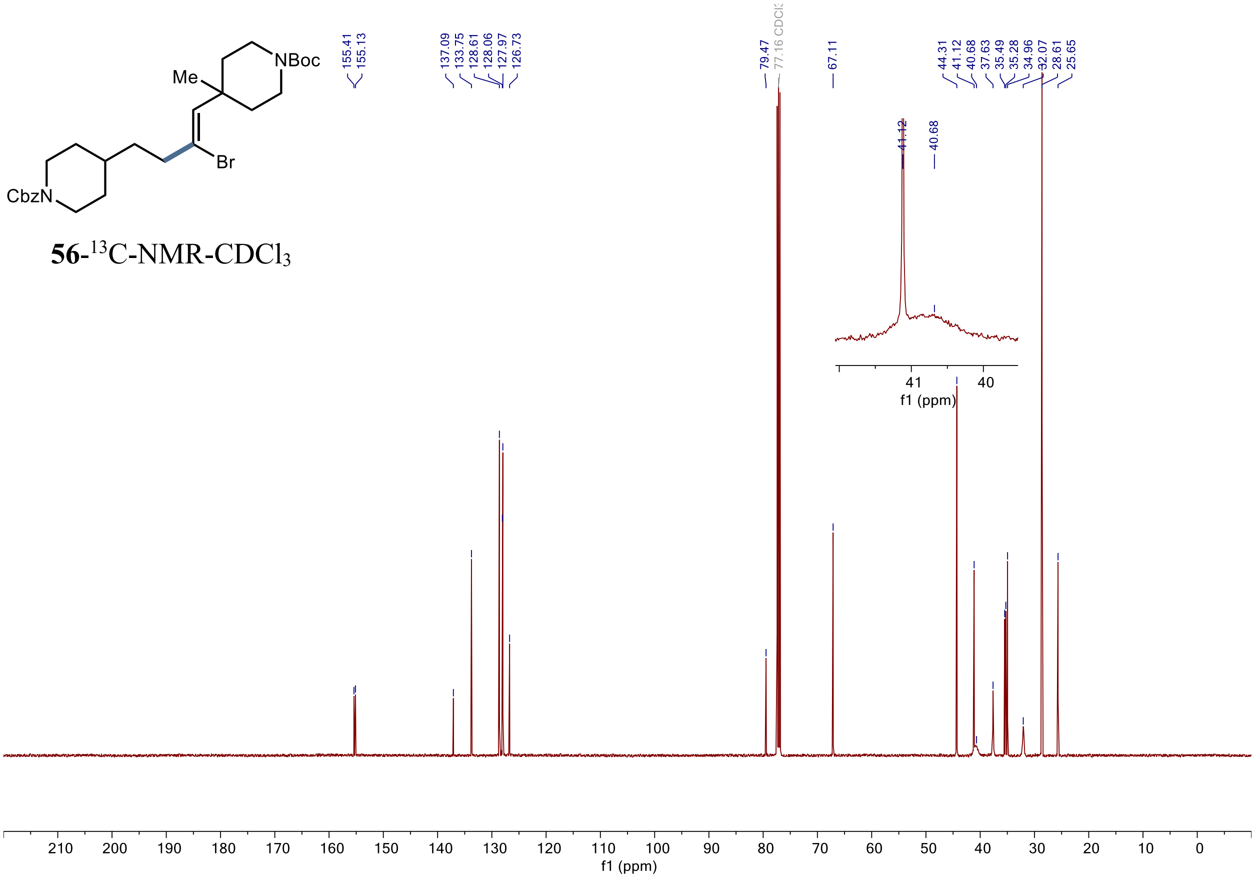
Me
NBoc
Br
CbzN
56-13C-NMR-CDCl3
155.41
155.13
137.09
133.75
128.61
128.06
127.97
126.73
79.47
77.16 CDCl3
67.11
44.31
41.12
40.68
37.63
35.49
35.28
34.96
32.07
28.61
25.65
f1 (ppm)

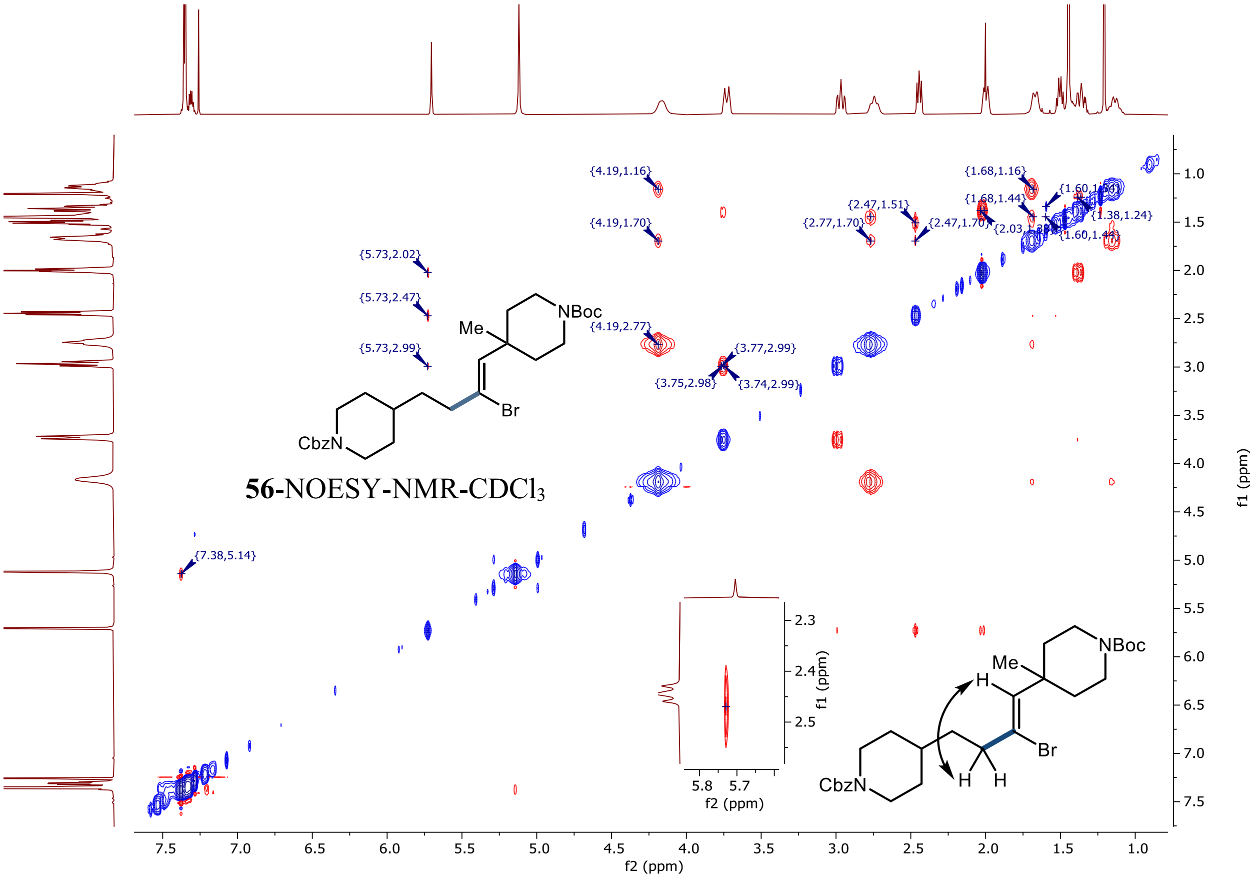
Me
NBoc
Br
CbzN
56-NOESY-NMR-CDCl3
{4.19,1.16}
{4.19,1.70}
{5.73,2.02}
{5.73,2.47}
{5.73,2.99}
{4.19,2.77}
{3.77,2.99}
{3.75,2.98}
{3.74,2.99}
{7.38,5.14}
{2.47,1.51}
{2.77,1.70}
{2.47,1.70}
{1.68,1.16}
{1.68,1.44}
{1.38,1.24}
{1.60,1.44}
H
H
H
f2 (ppm)
f1 (ppm)

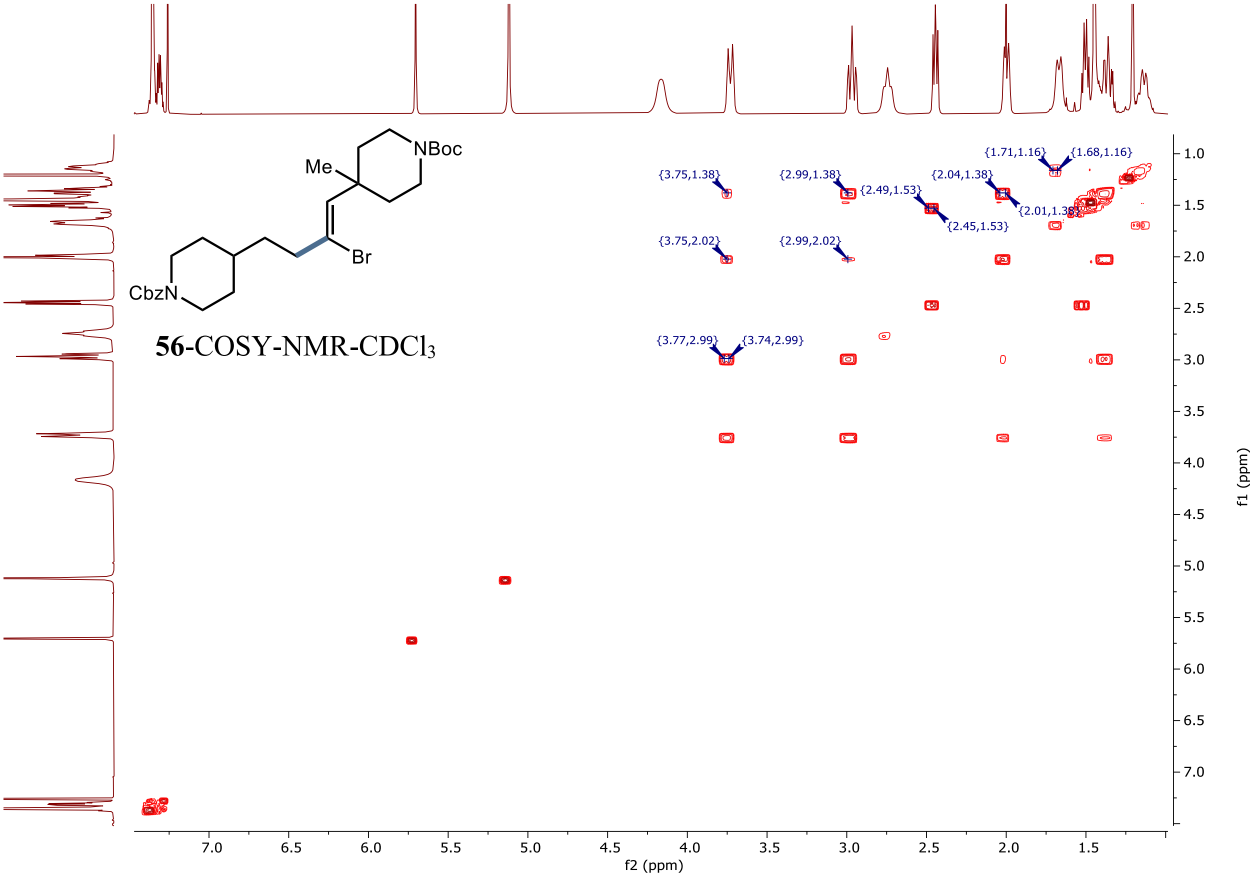
NBoc
Me
Br
CbzN
56-COSY-NMR-CDCl3
f2 (ppm)
f1 (ppm)

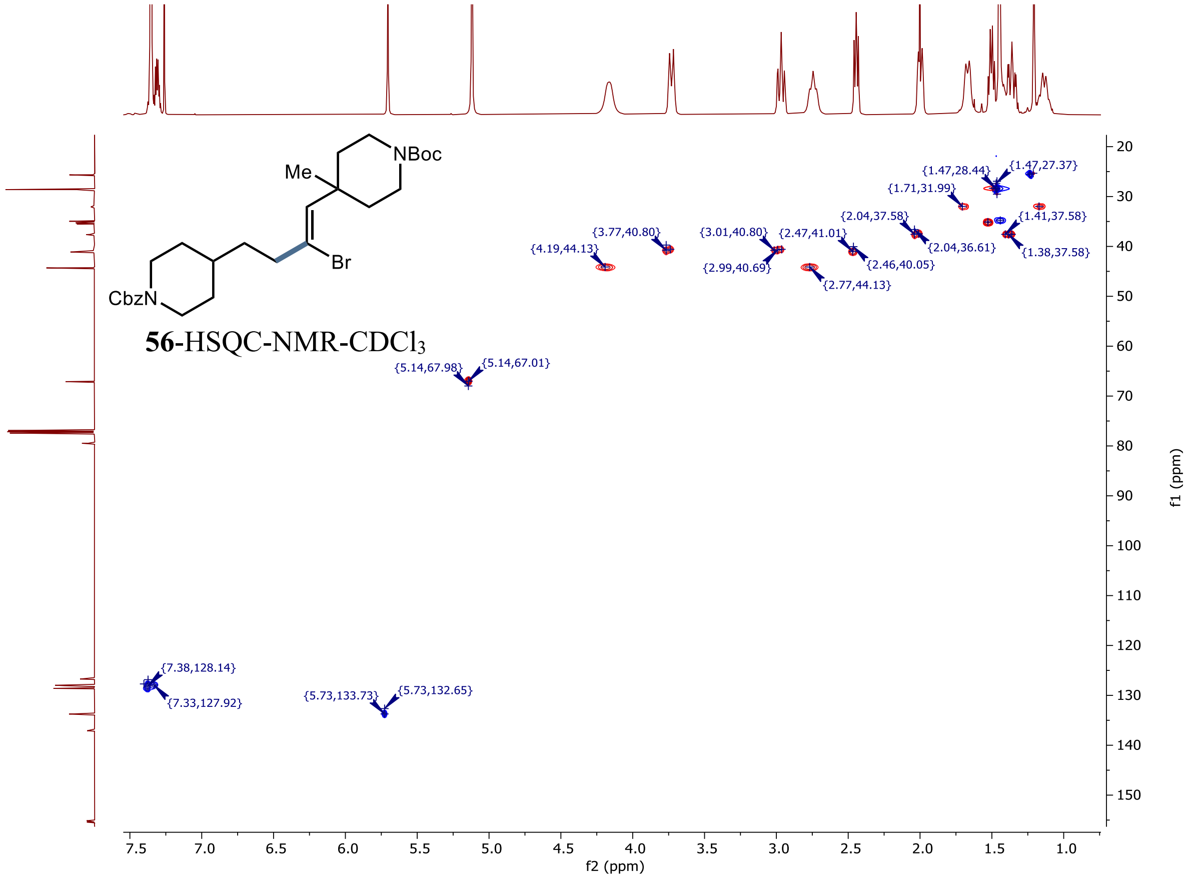
NBoc
Me
Br
CbzN
56-HSQC-NMR-CDCl3
f2 (ppm)
f1 (ppm)

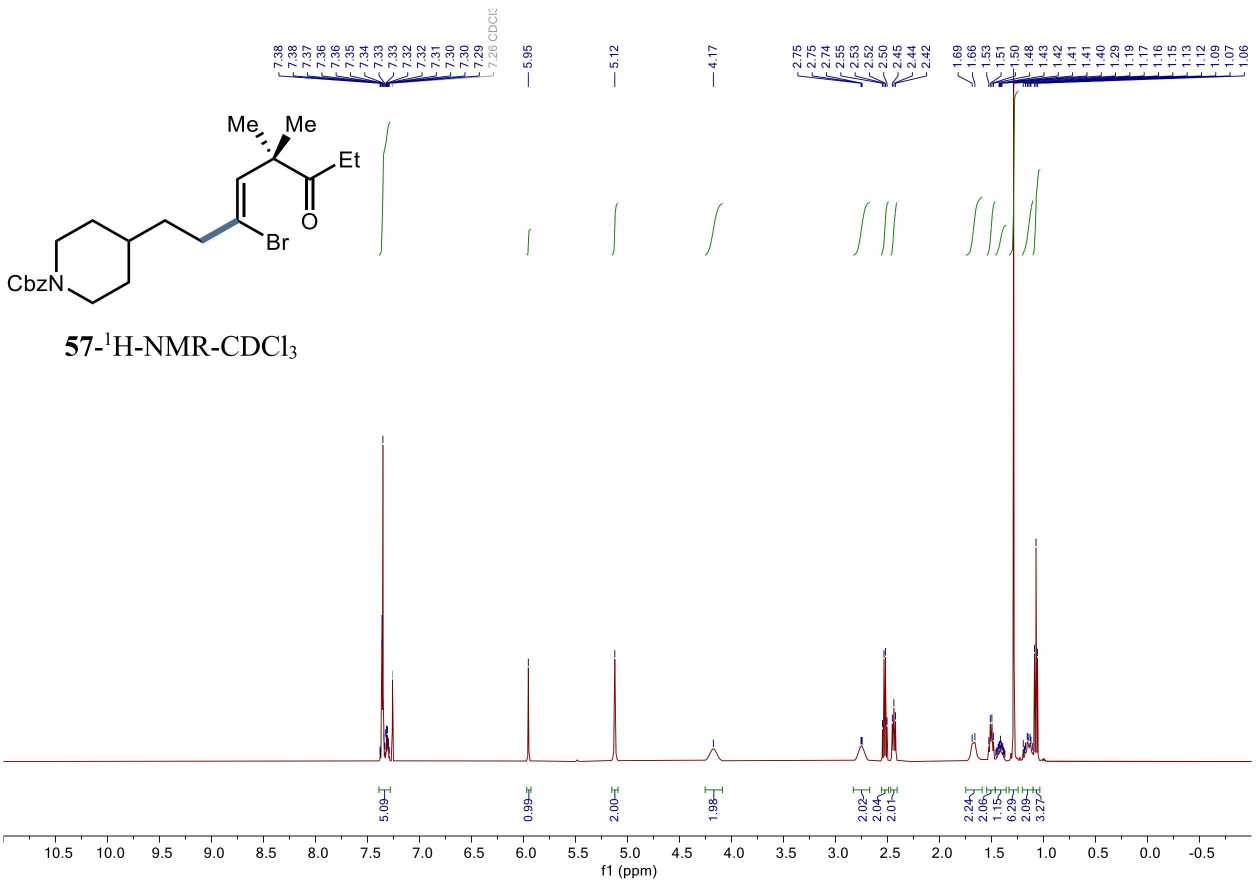

57-^{1}H-NMR-$CDCl_3$

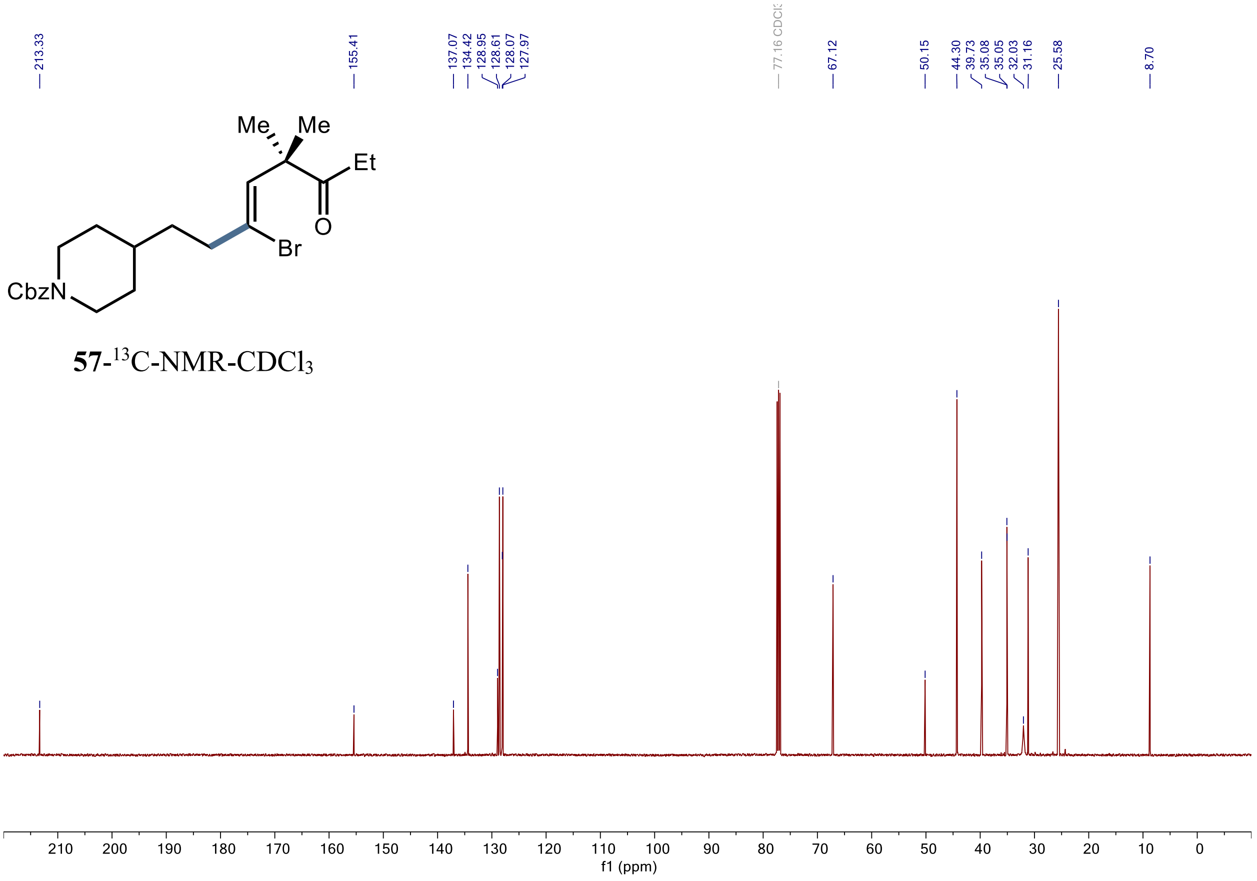

57-^{13}C-NMR-$CDCl_3$

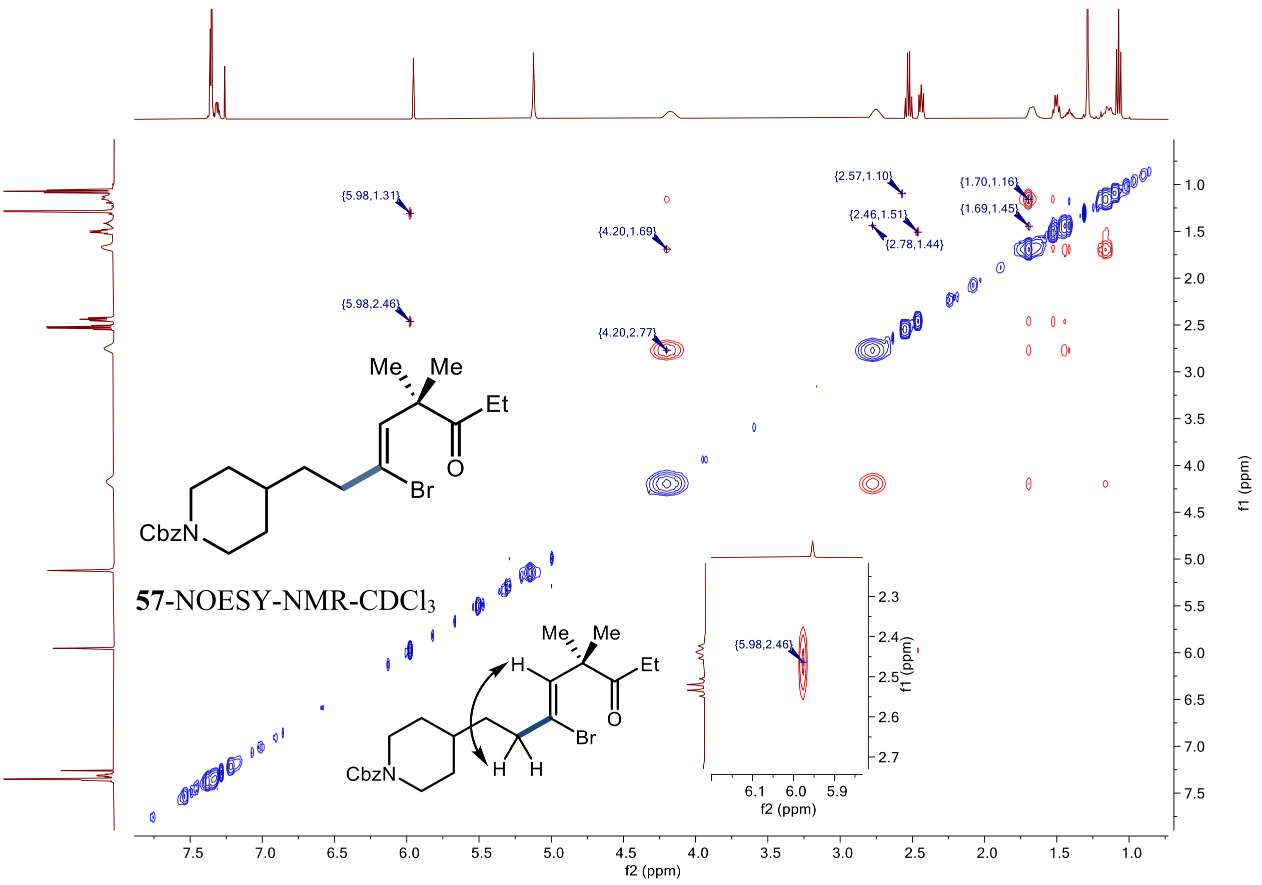
57-NOESY-NMR-CDCl3
Me
Me
Et
O
Br
CbzN
H
H
H
{5.98,1.31}
{4.20,1.69}
{5.98,2.46}
{4.20,2.77}
{2.57,1.10}
{2.46,1.51}
{2.78,1.44}
{1.70,1.16}
{1.69,1.45}
f2 (ppm)
f1 (ppm)

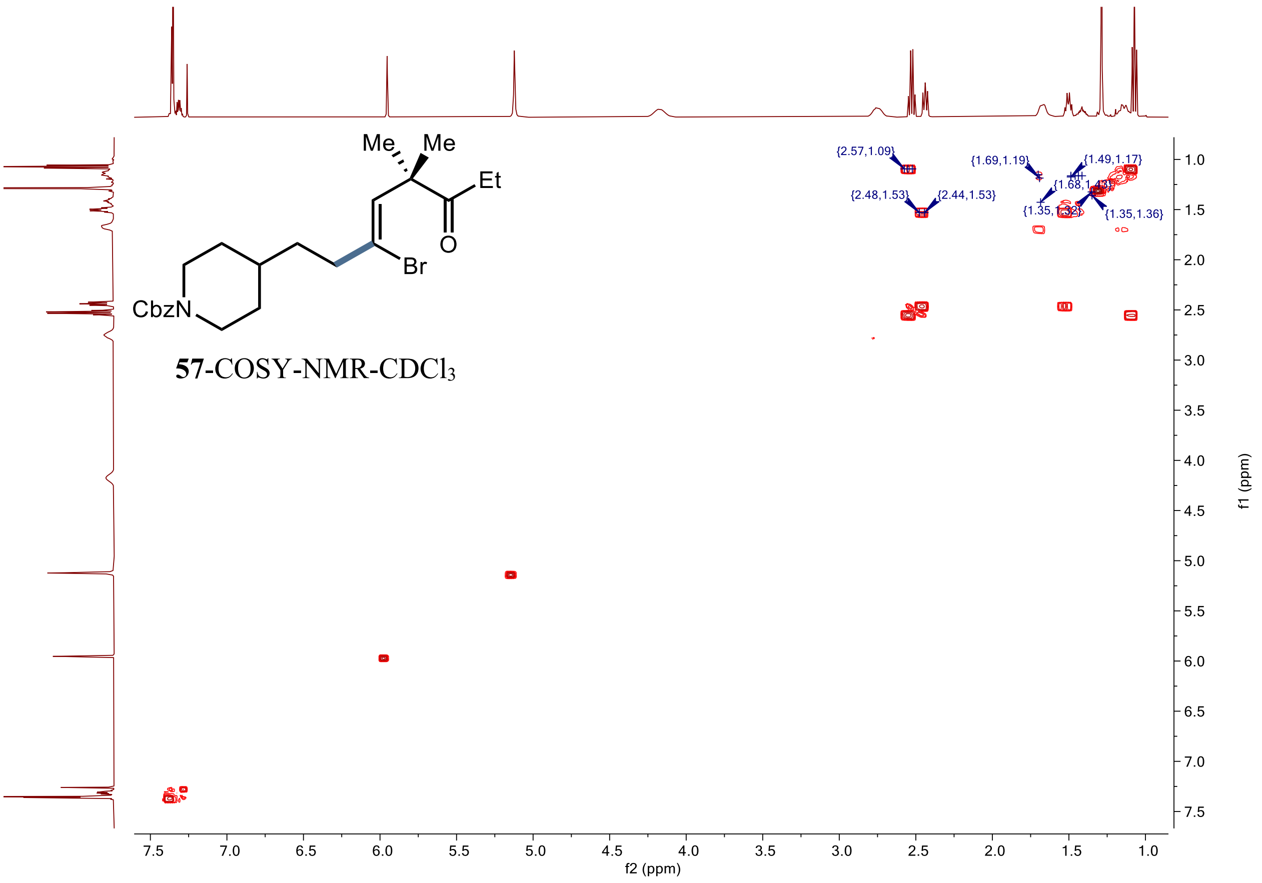
57-COSY-NMR-CDCl3
Me
Me
Et
O
Br
CbzN
{2.57,1.09}
{2.48,1.53}
{2.44,1.53}
{1.69,1.19}
{1.49,1.17}
{1.35,1.36}
f2 (ppm)
f1 (ppm)

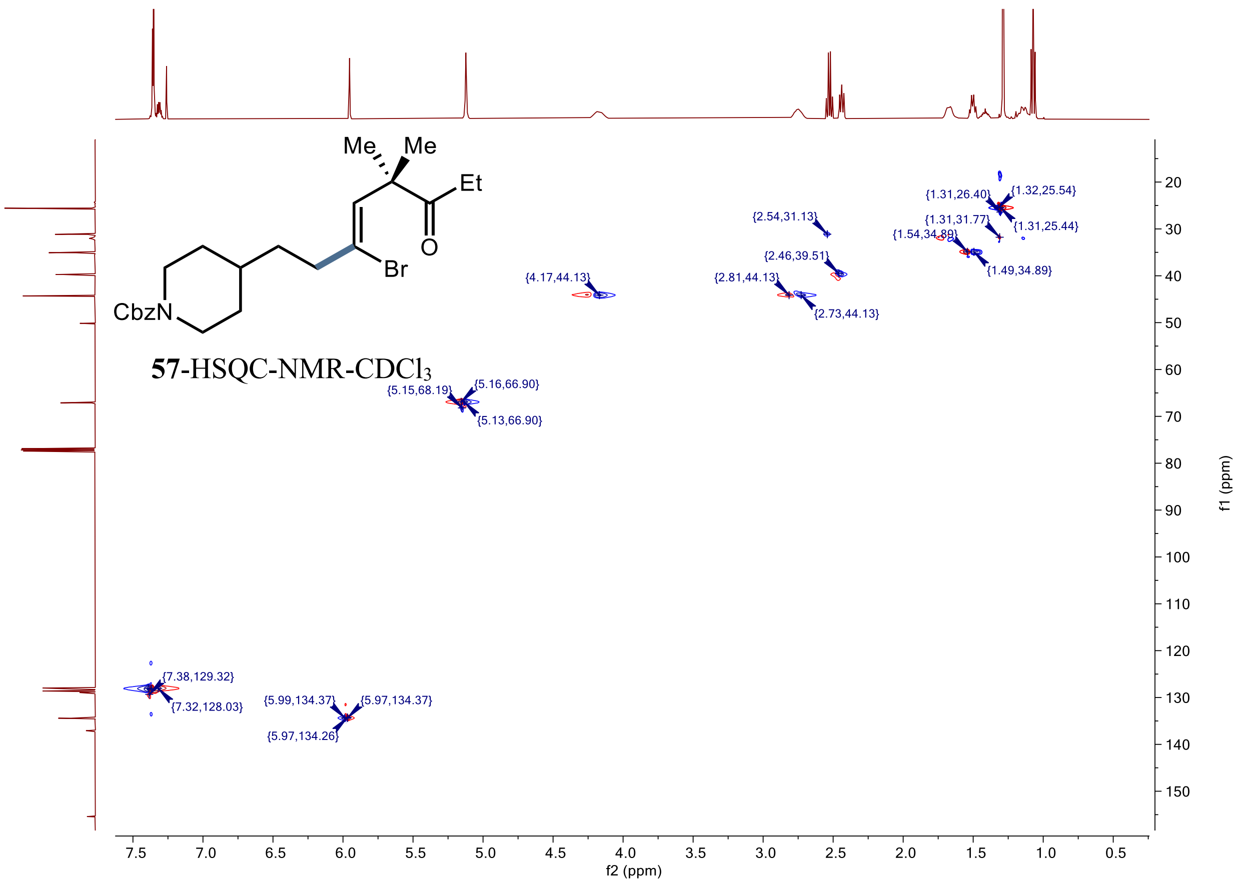
Me
Me
Et
O
Br
CbzN
57-HSQC-NMR-CDCl3
f2 (ppm)
f1 (ppm)

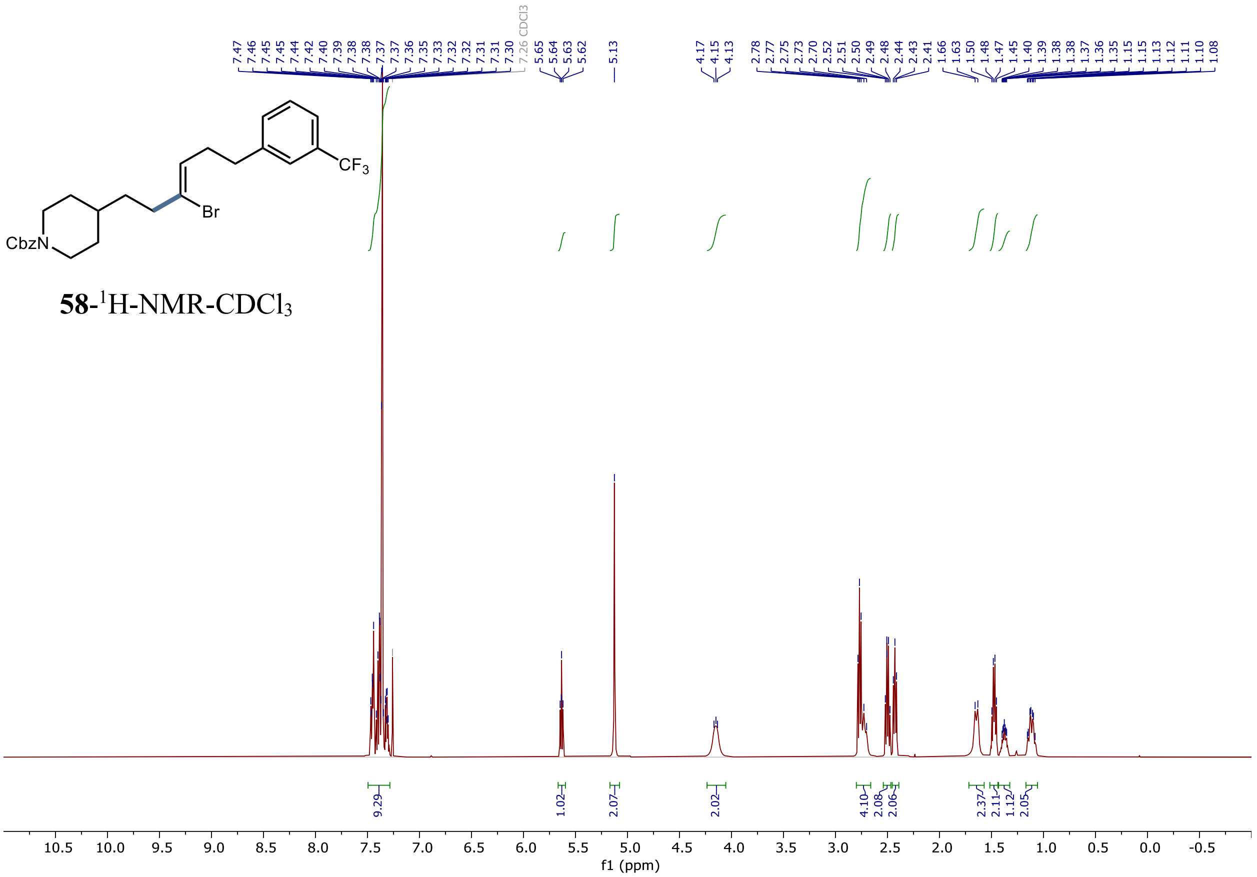
CF3
Br
CbzN
58-1H-NMR-CDCl3
f1 (ppm)

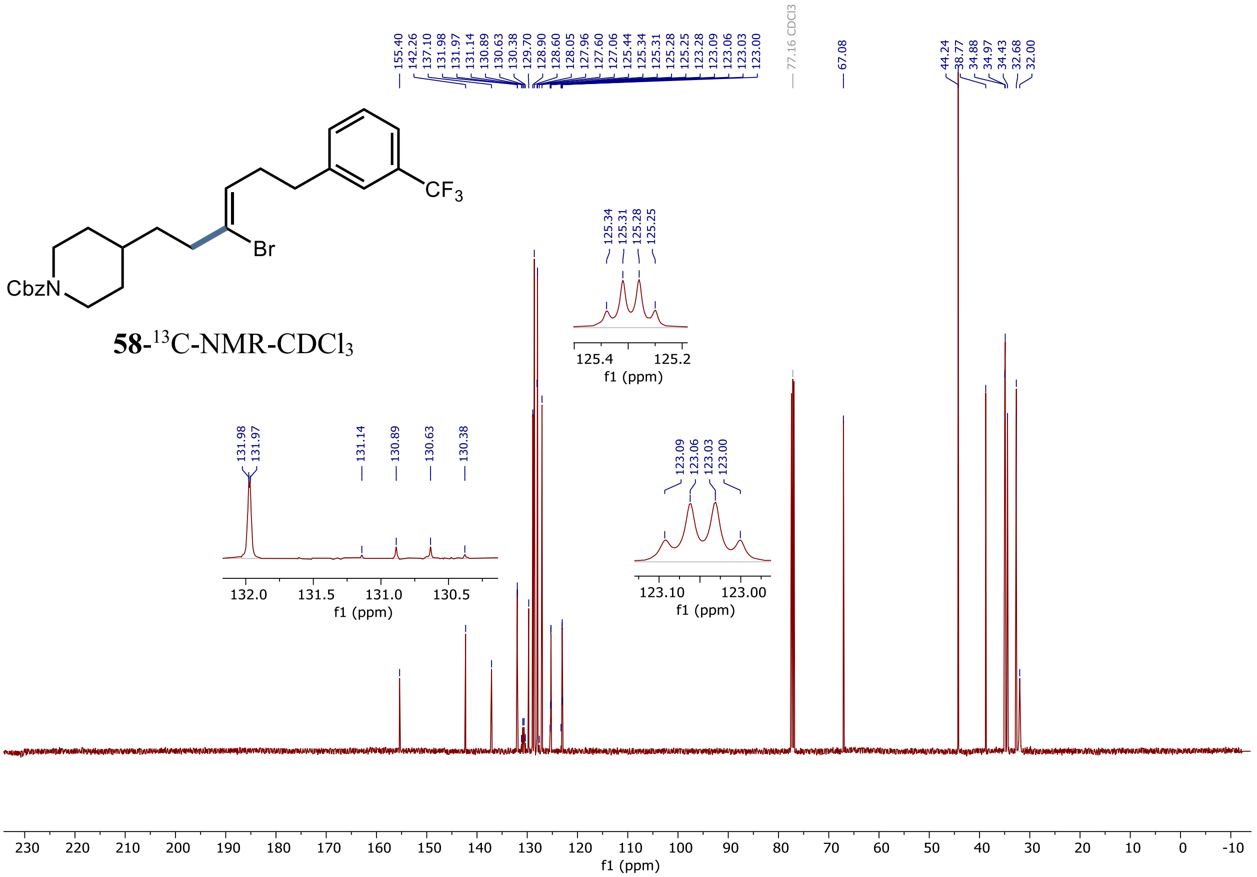

58-^{13}C-NMR-CDCl$_3$

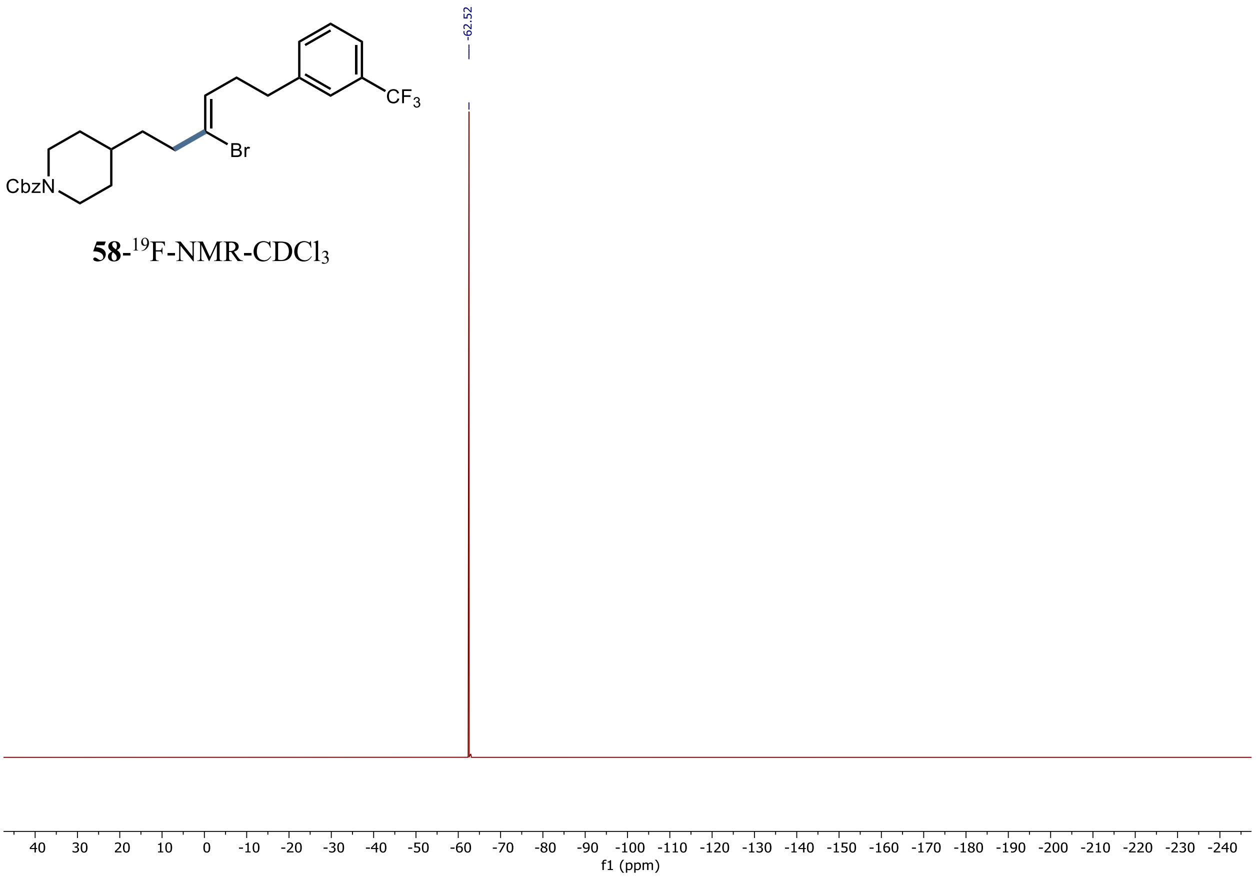

58-^{19}F-NMR-CDCl$_3$

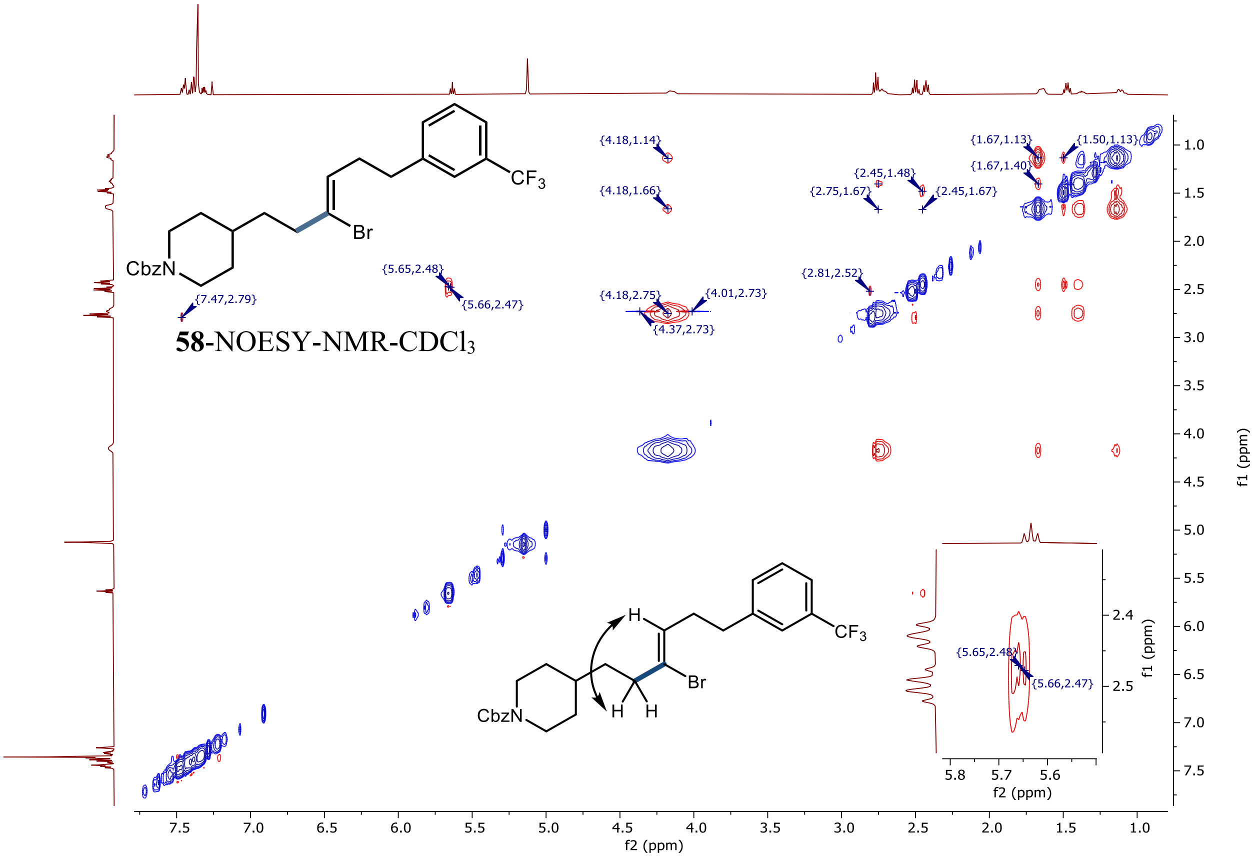
58-NOESY-NMR-CDCl3
f2 (ppm)
f1 (ppm)

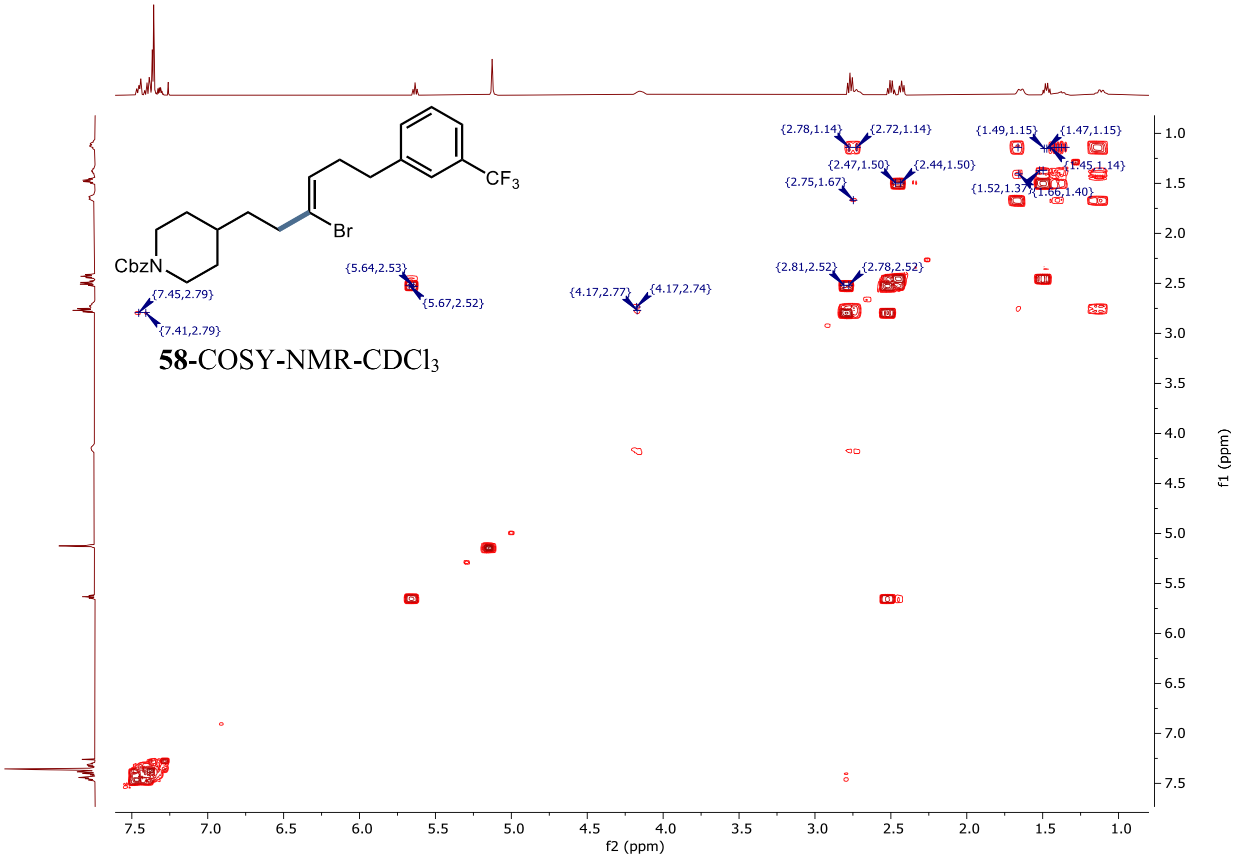
58-COSY-NMR-CDCl3
f2 (ppm)
f1 (ppm)

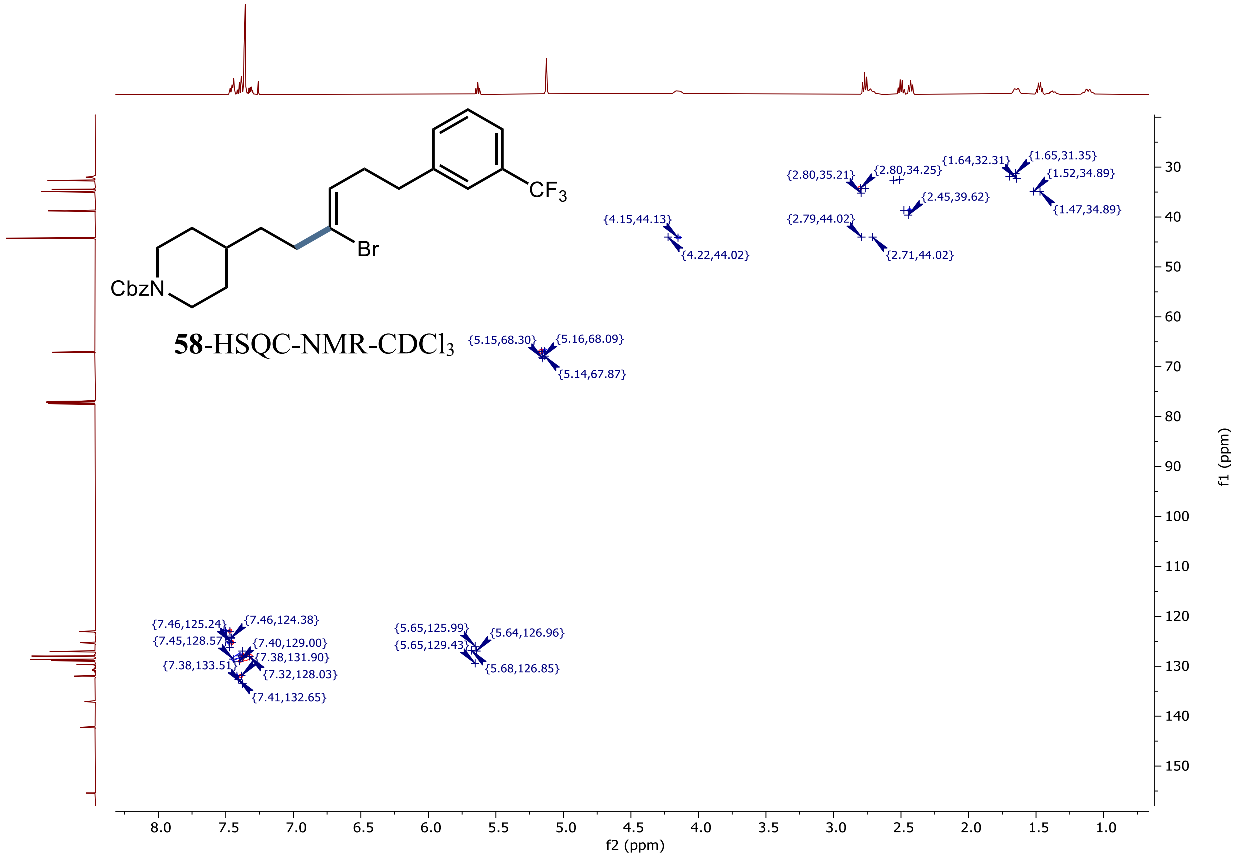

CbzN
Br
CF3
58-HSQC-NMR-CDCl3
f2 (ppm)
f1 (ppm)

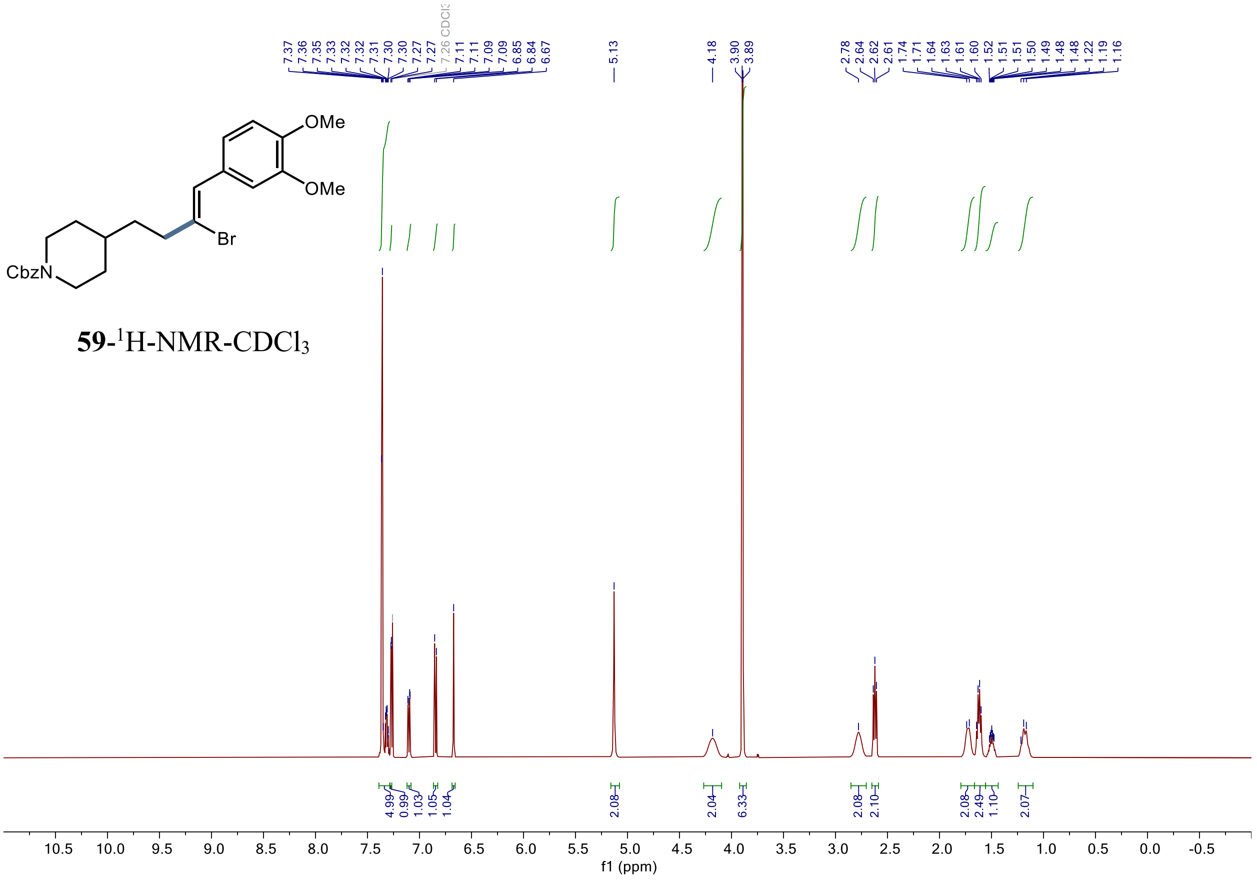

OMe
OMe
Br
CbzN
59-1H-NMR-CDCl3
f1 (ppm)

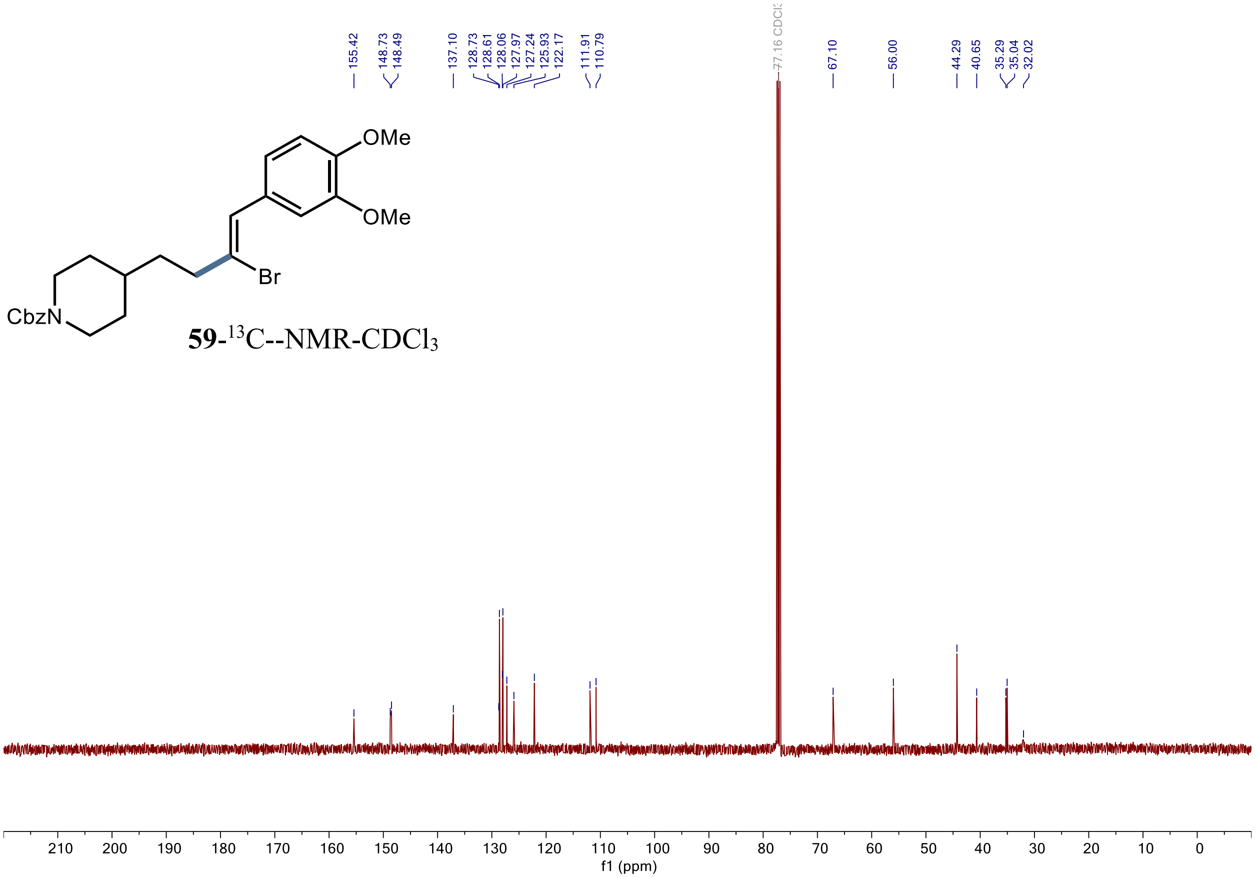

59-^{13}C--NMR-CDCl$_3$

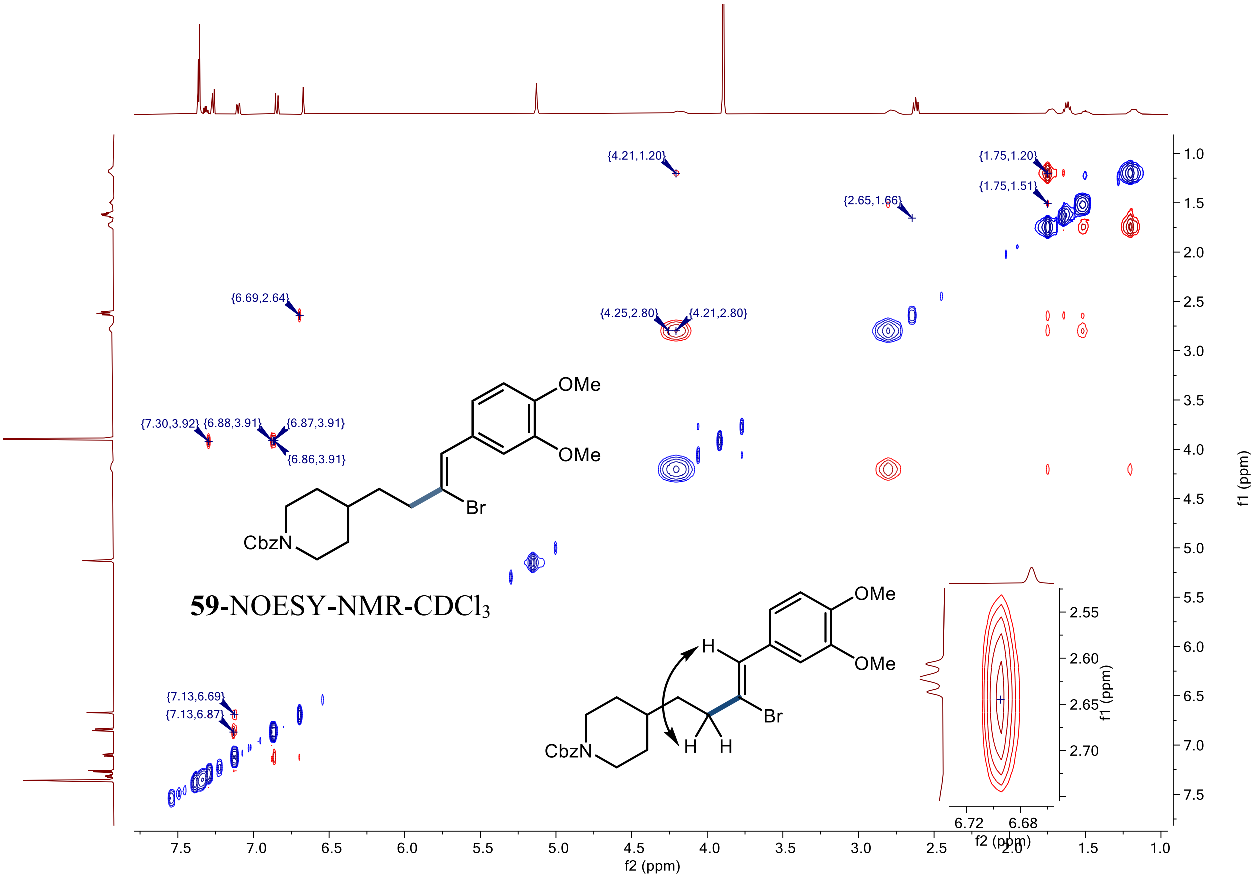

59-NOESY-NMR-CDCl$_3$

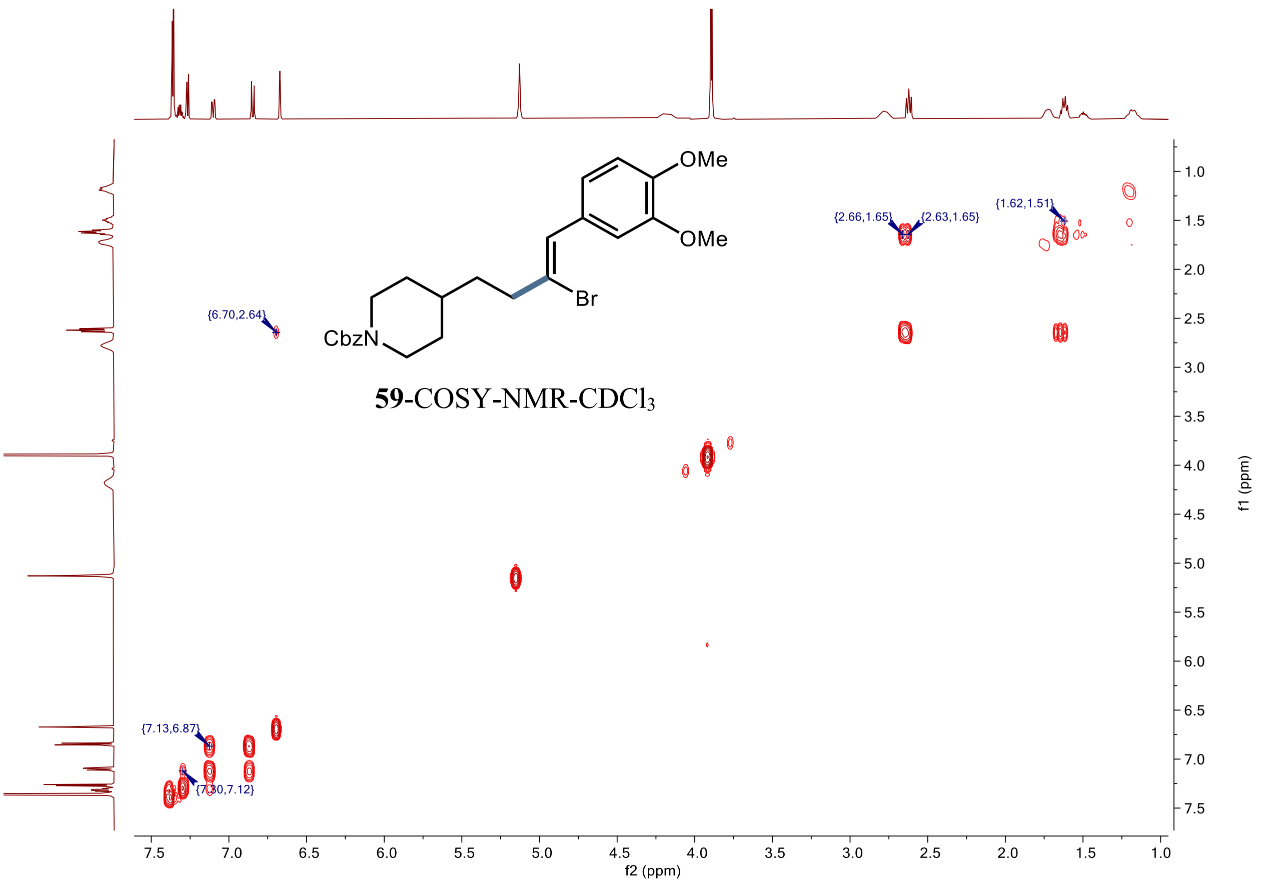

59-COSY-NMR-$CDCl_3$

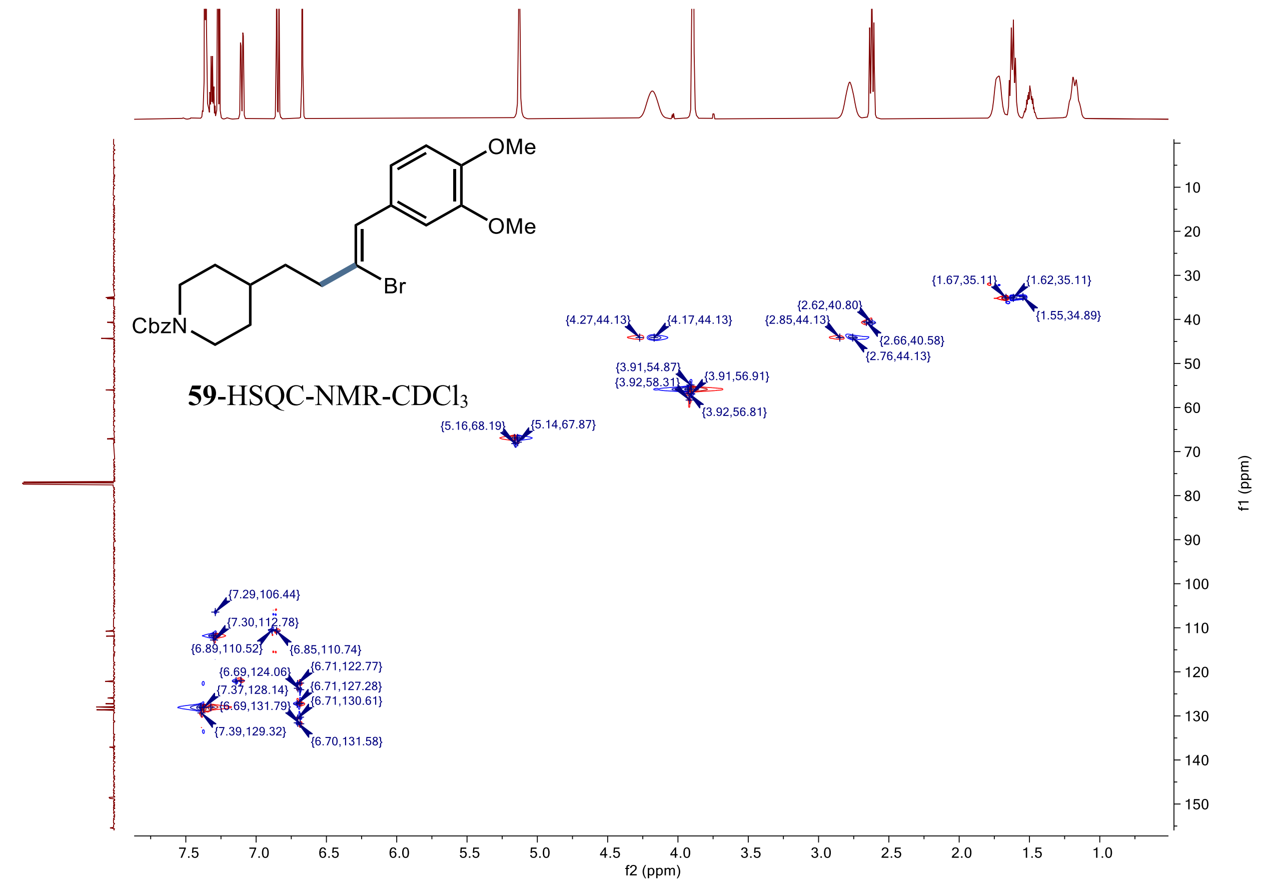

59-HSQC-NMR-$CDCl_3$

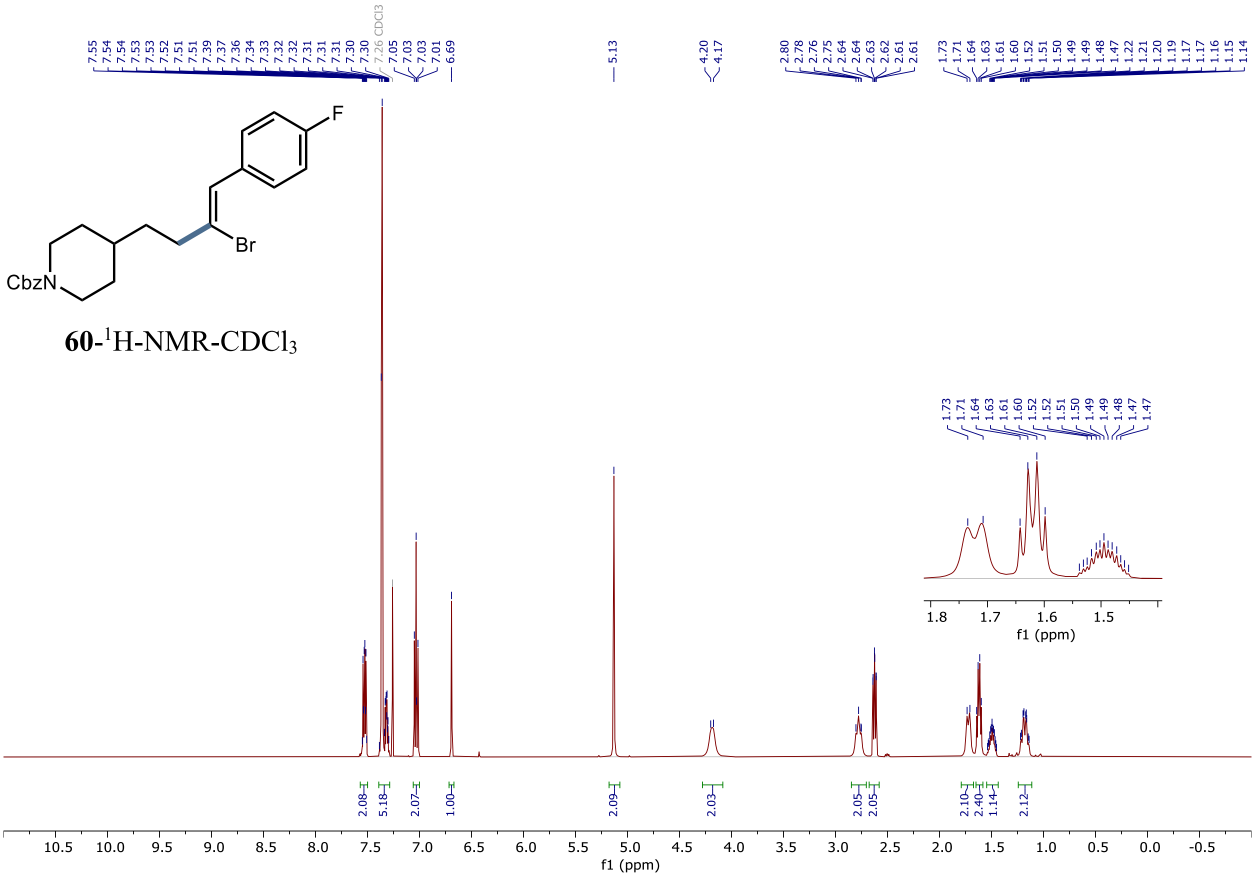

60-^{1}H-NMR-$CDCl_3$

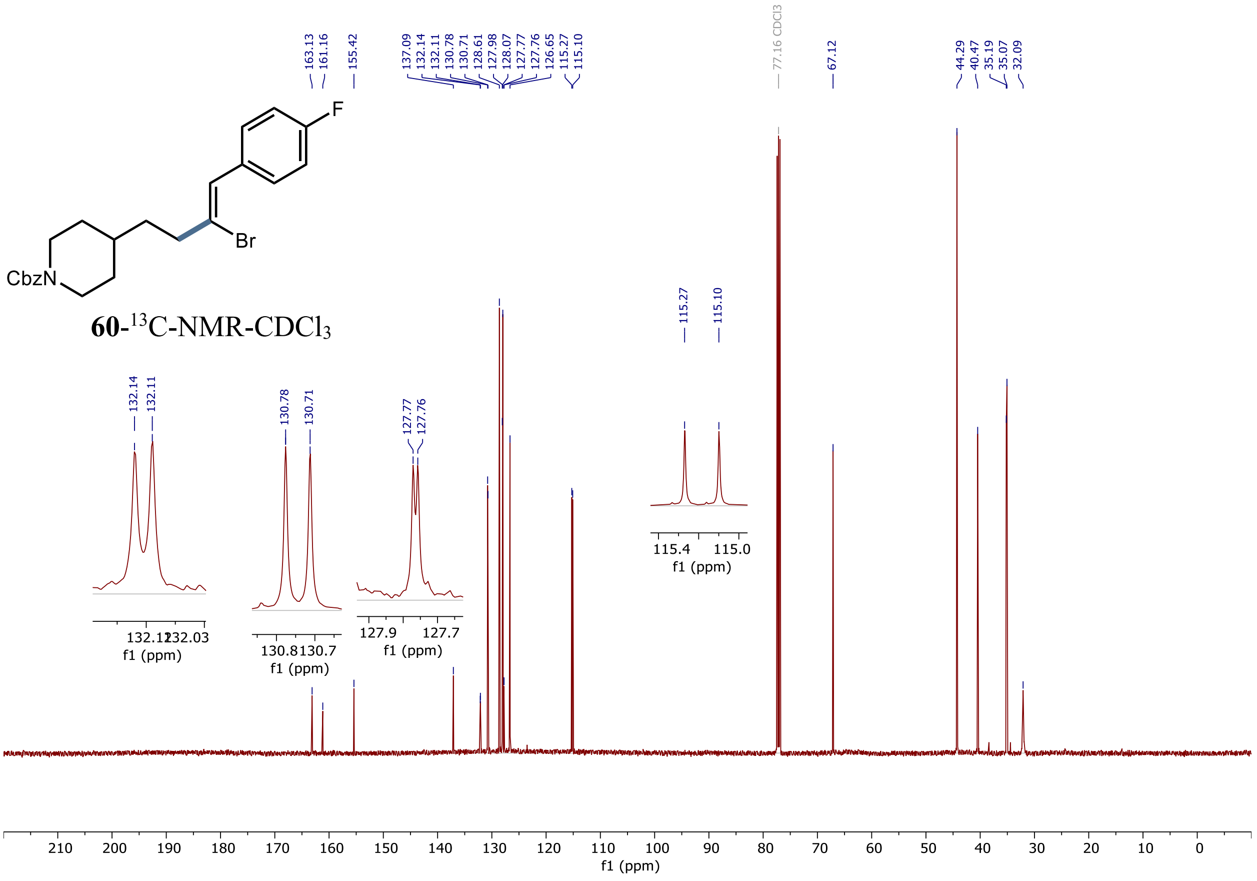

60-^{13}C-NMR-$CDCl_3$

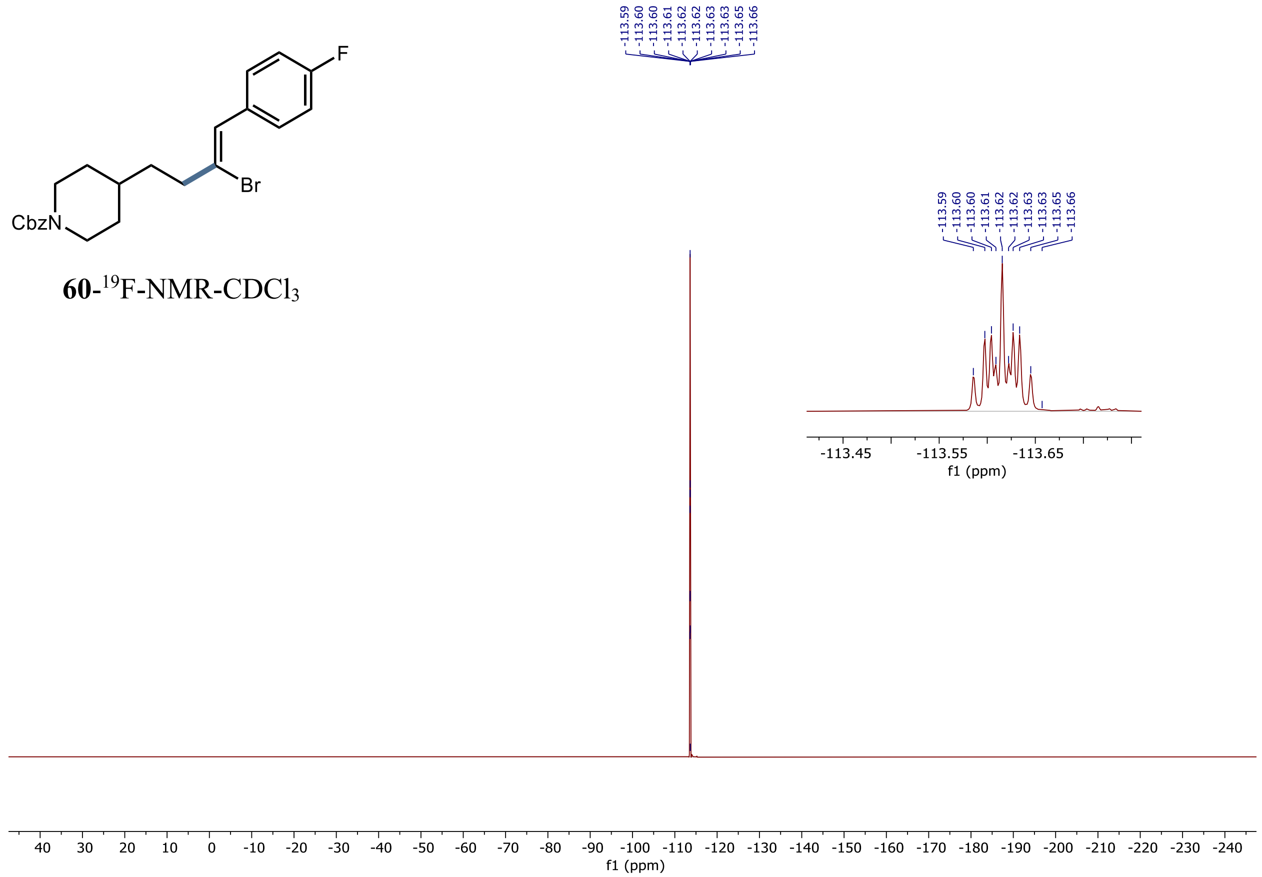
60-19F-NMR-CDCl3
CbzN
Br
F
-113.59
-113.60
-113.60
-113.61
-113.62
-113.62
-113.63
-113.63
-113.65
-113.66
-113.45
-113.55
-113.65
f1 (ppm)
40 30 20 10 0 -10 -20 -30 -40 -50 -60 -70 -80 -90 -100 -110 -120 -130 -140 -150 -160 -170 -180 -190 -200 -210 -220 -230 -240
f1 (ppm)

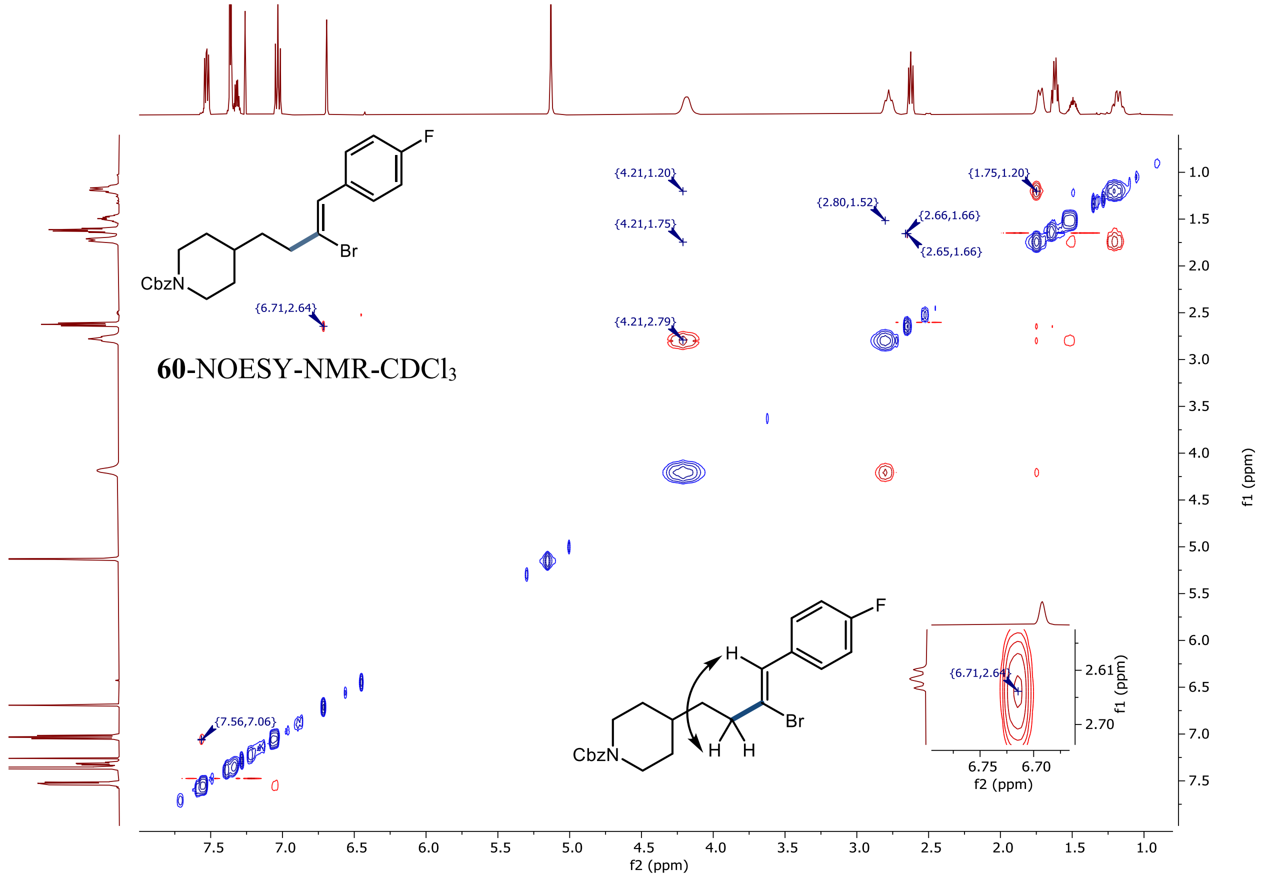
60-NOESY-NMR-CDCl3
CbzN
Br
F
H
H
H
{4.21,1.20}
{1.75,1.20}
{2.80,1.52}
{2.66,1.66}
{4.21,1.75}
{2.65,1.66}
{6.71,2.64}
{4.21,2.79}
{7.56,7.06}
6.75
6.70
f2 (ppm)
2.61
2.70
f1 (ppm)
7.5 7.0 6.5 6.0 5.5 5.0 4.5 4.0 3.5 3.0 2.5 2.0 1.5 1.0
f2 (ppm)
1.0 1.5 2.0 2.5 3.0 3.5 4.0 4.5 5.0 5.5 6.0 6.5 7.0 7.5
f1 (ppm)

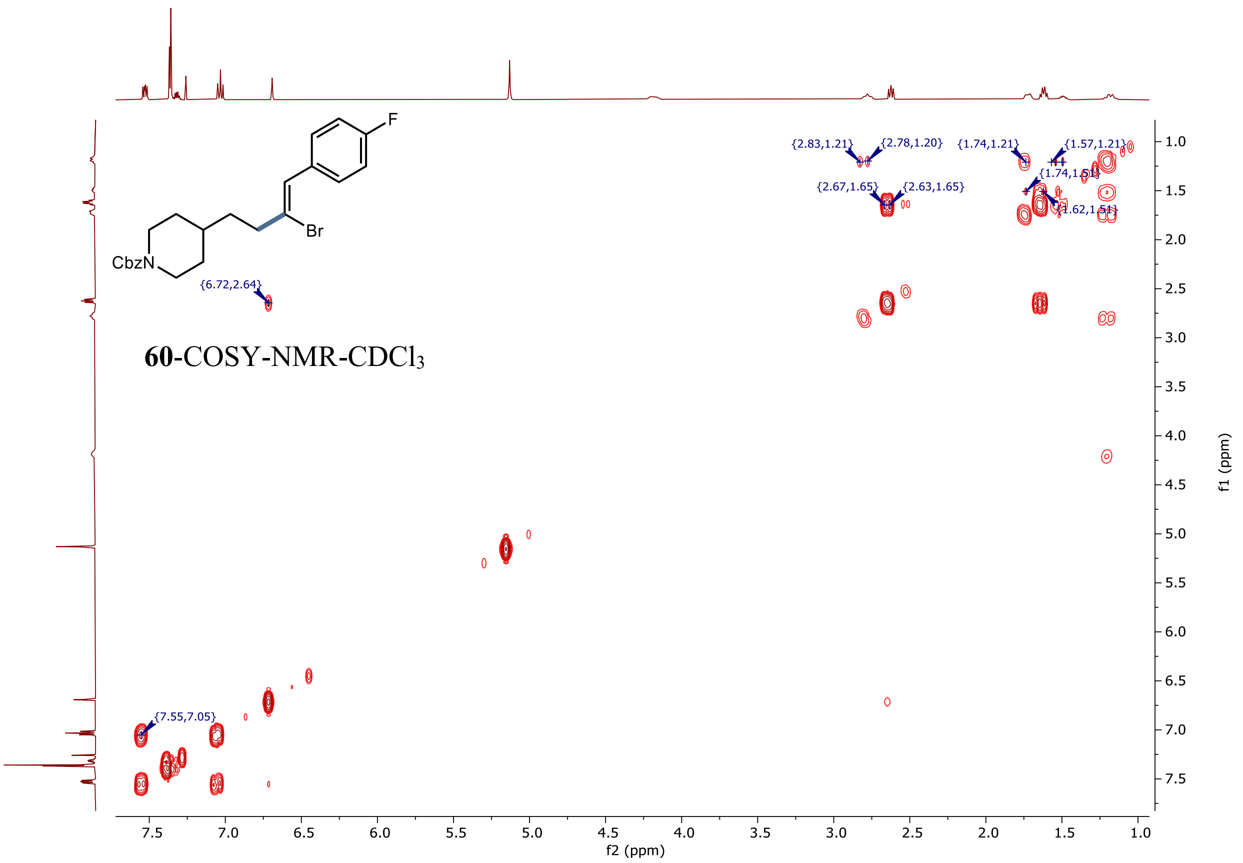

F
Br
CbzN
60-COSY-NMR-CDCl3
f2 (ppm)
f1 (ppm)

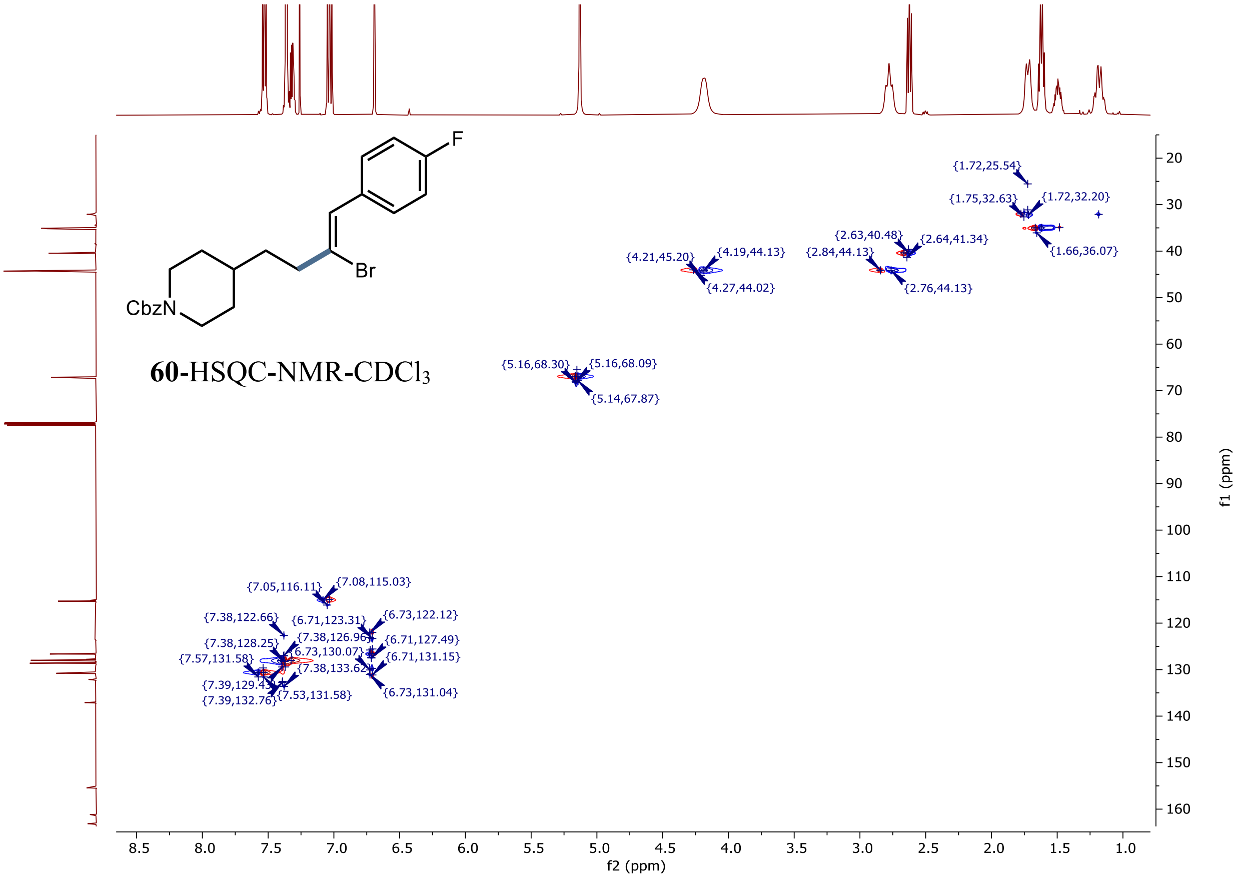

F
Br
CbzN
60-HSQC-NMR-CDCl3
f2 (ppm)
f1 (ppm)

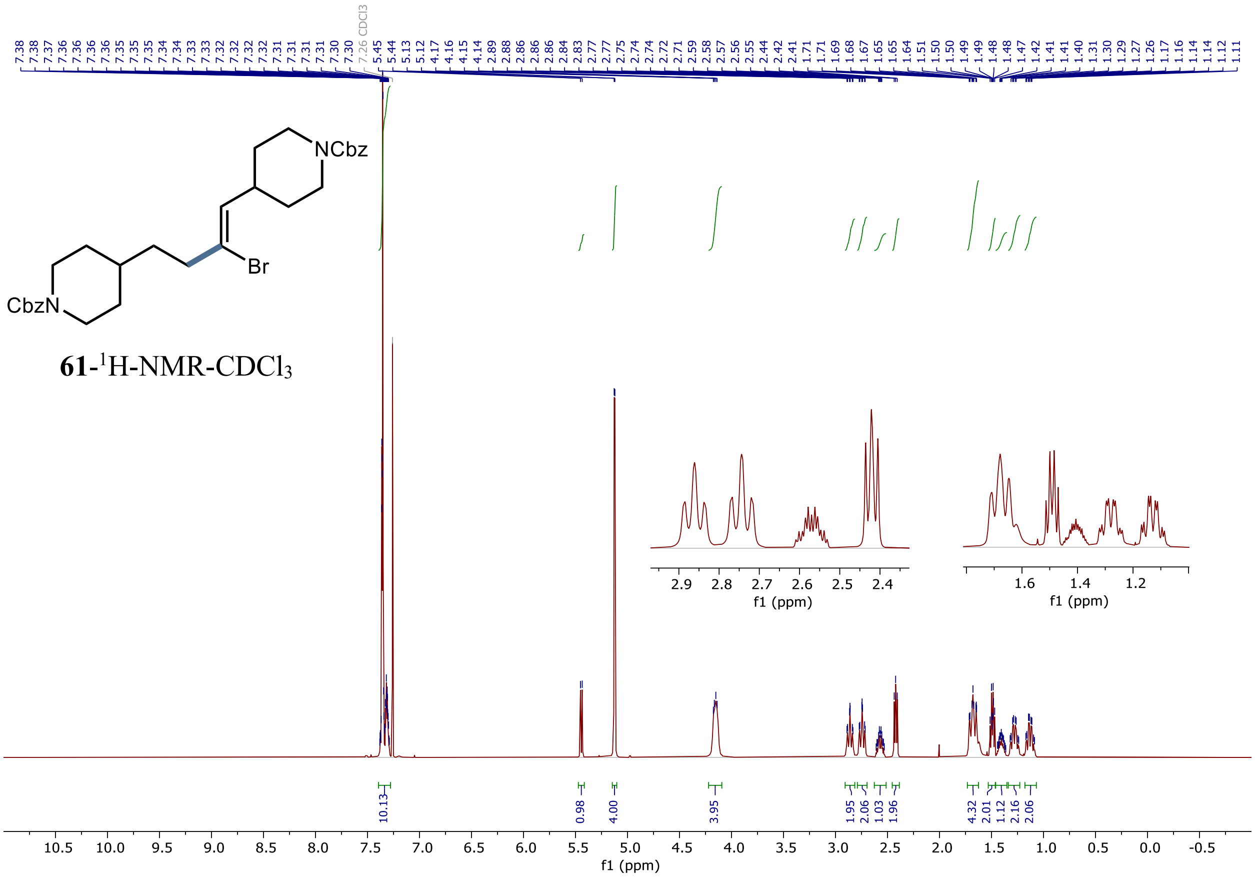

61-^{1}H-NMR-CDCl$_3$

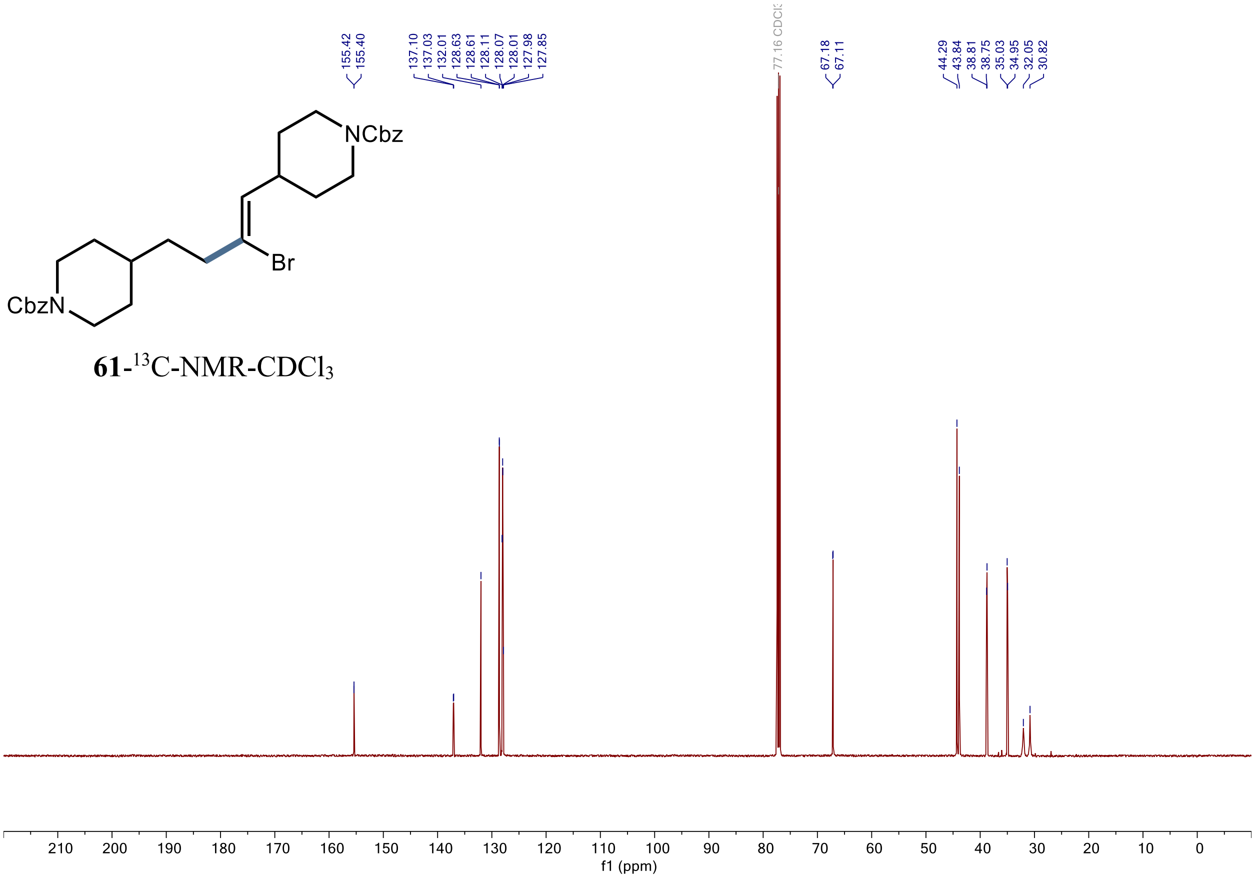

61-^{13}C-NMR-CDCl$_3$

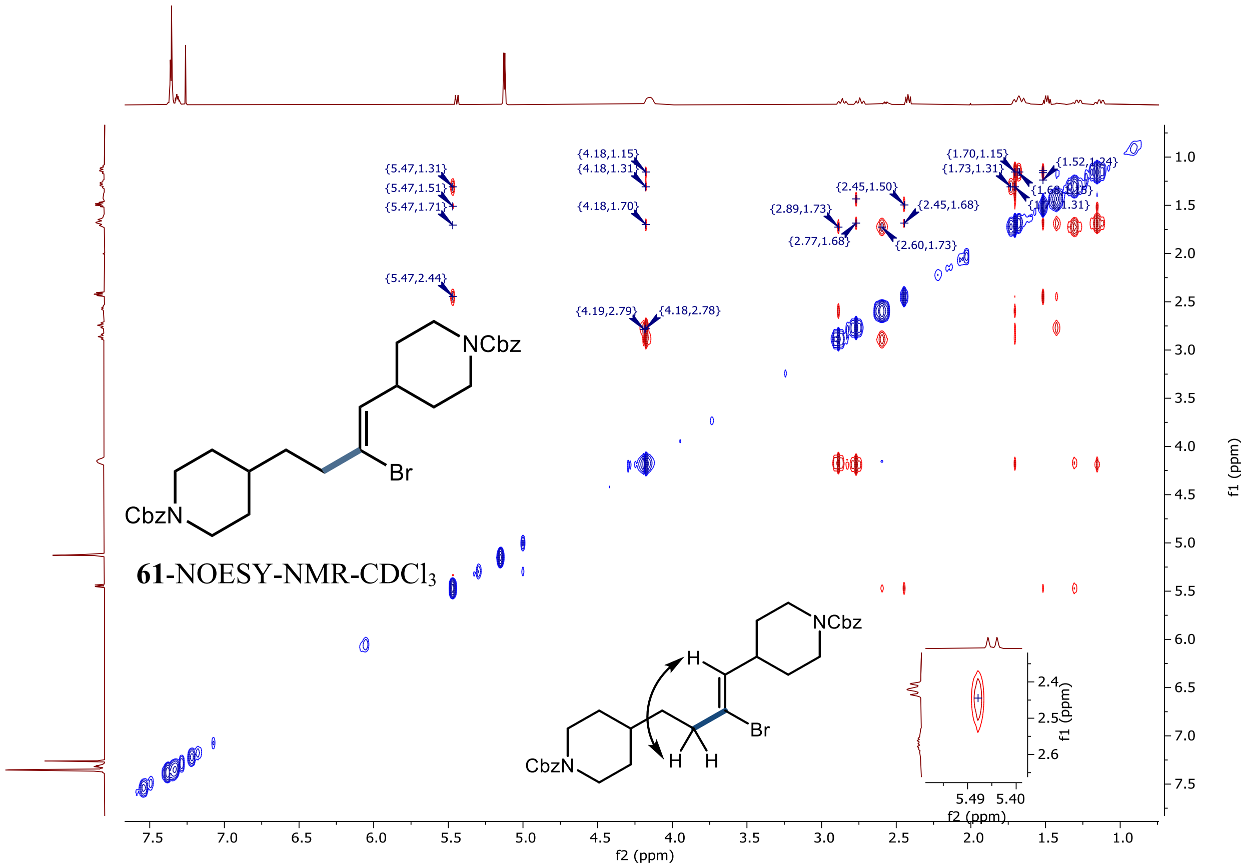
61-NOESY-NMR-CDCl3
NCbz
Br
CbzN
f2 (ppm)
f1 (ppm)

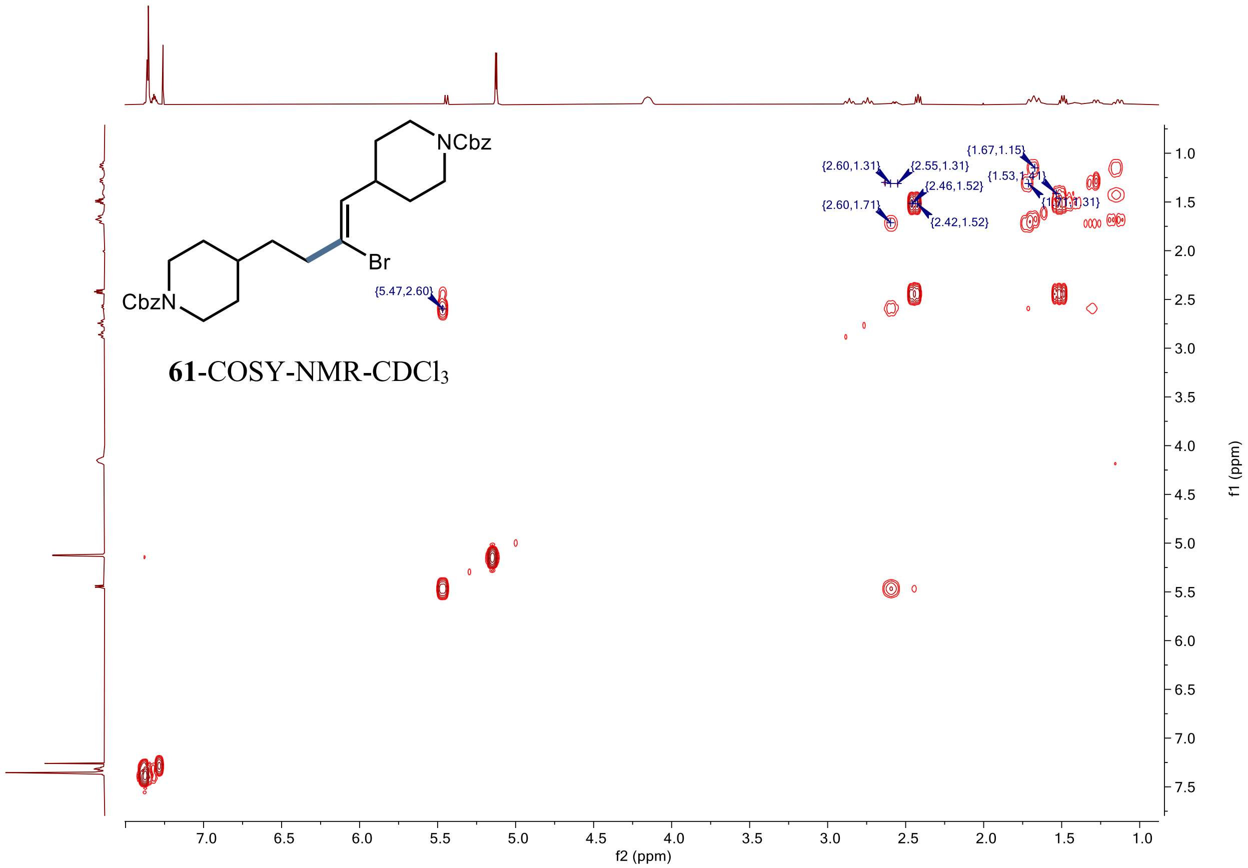
61-COSY-NMR-CDCl3
NCbz
Br
CbzN
f2 (ppm)
f1 (ppm)

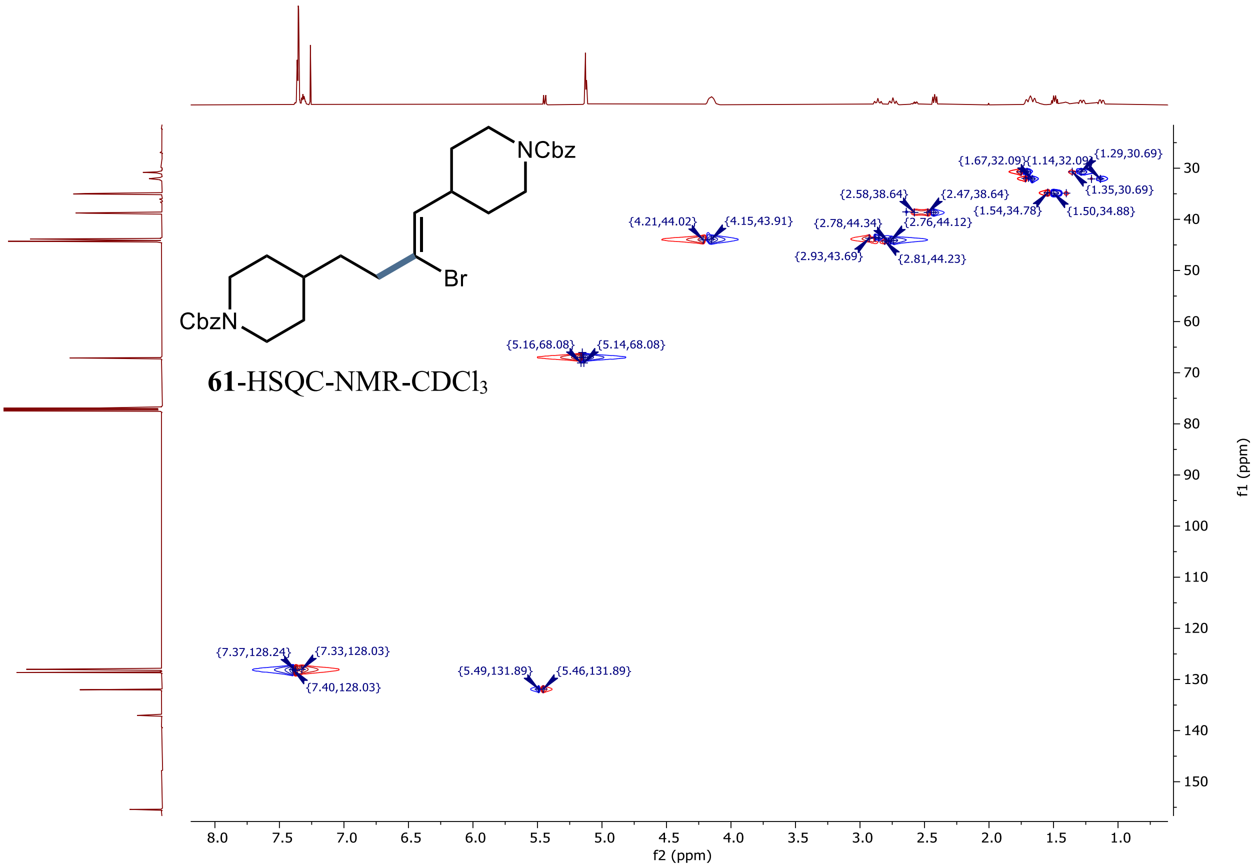
NCbz
Br
CbzN
61-HSQC-NMR-CDCl3
f2 (ppm)
f1 (ppm)

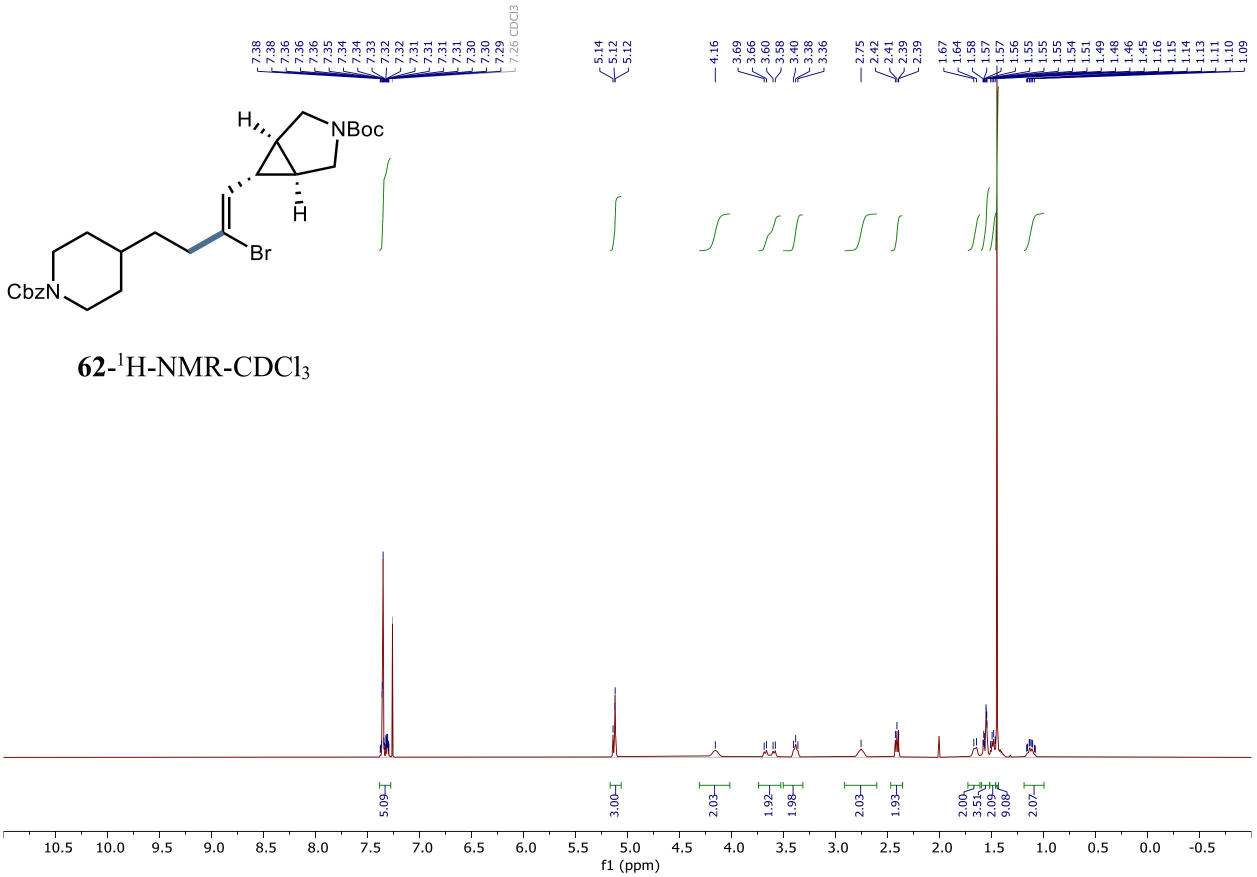
NBoc
H
H
Br
CbzN
62-1H-NMR-CDCl3
f1 (ppm)

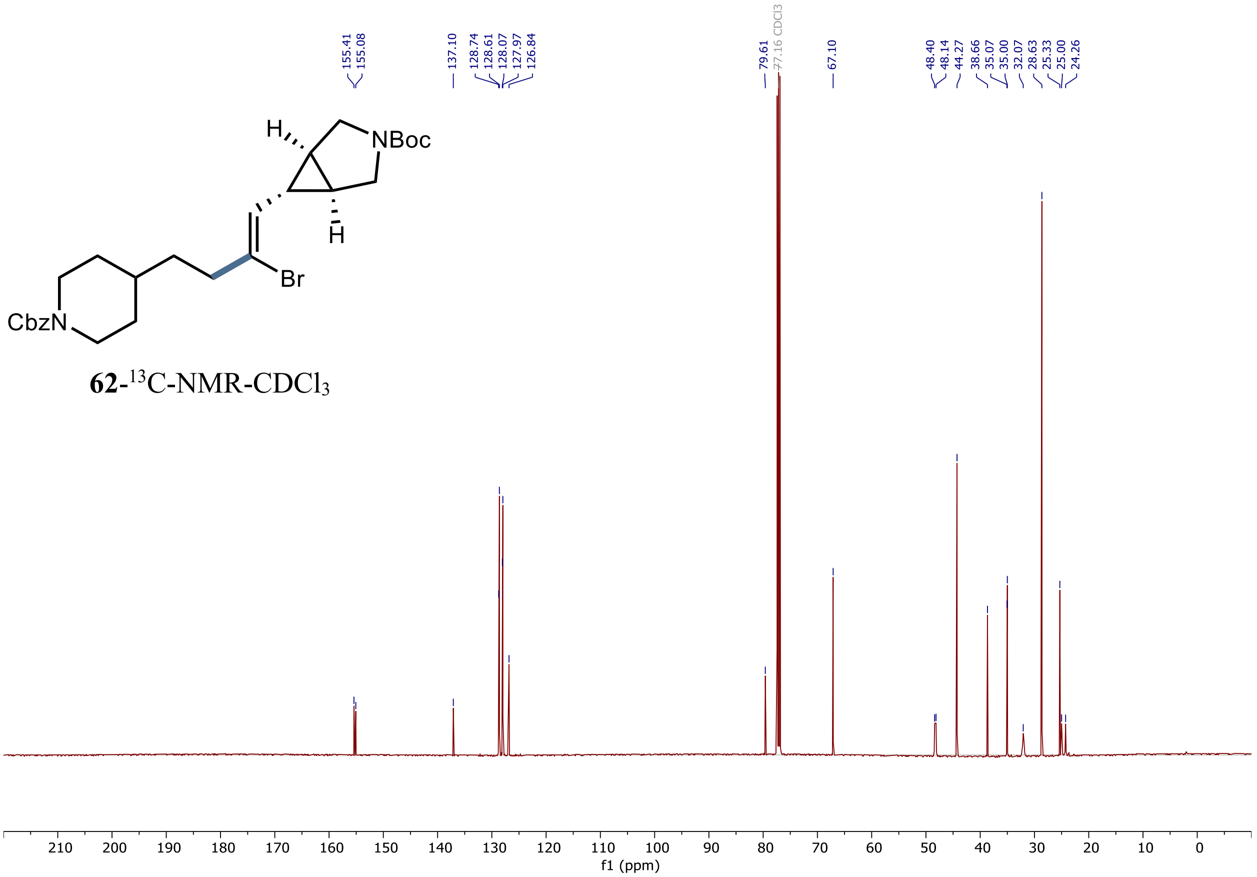

62-^{13}C-NMR-CDCl$_3$

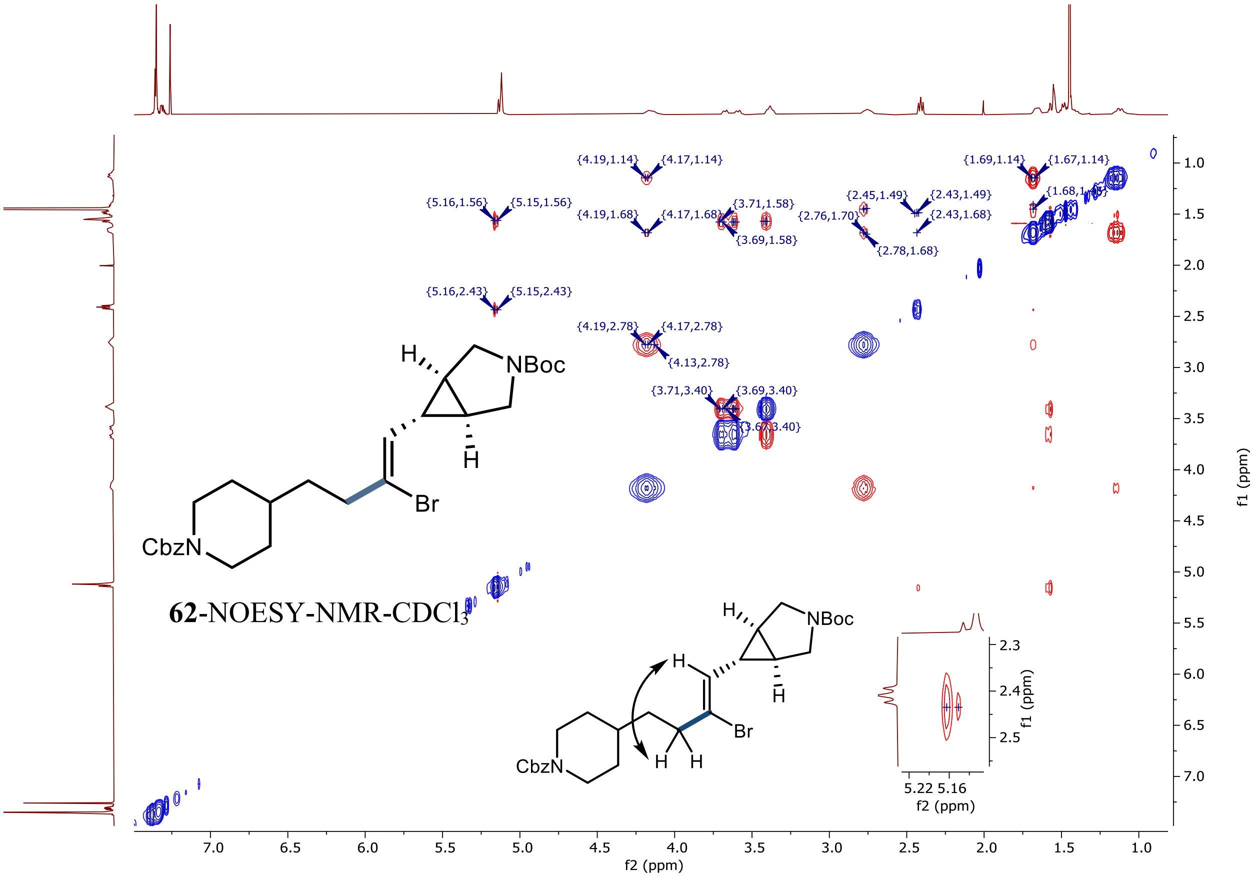

62-NOESY-NMR-CDCl$_3$

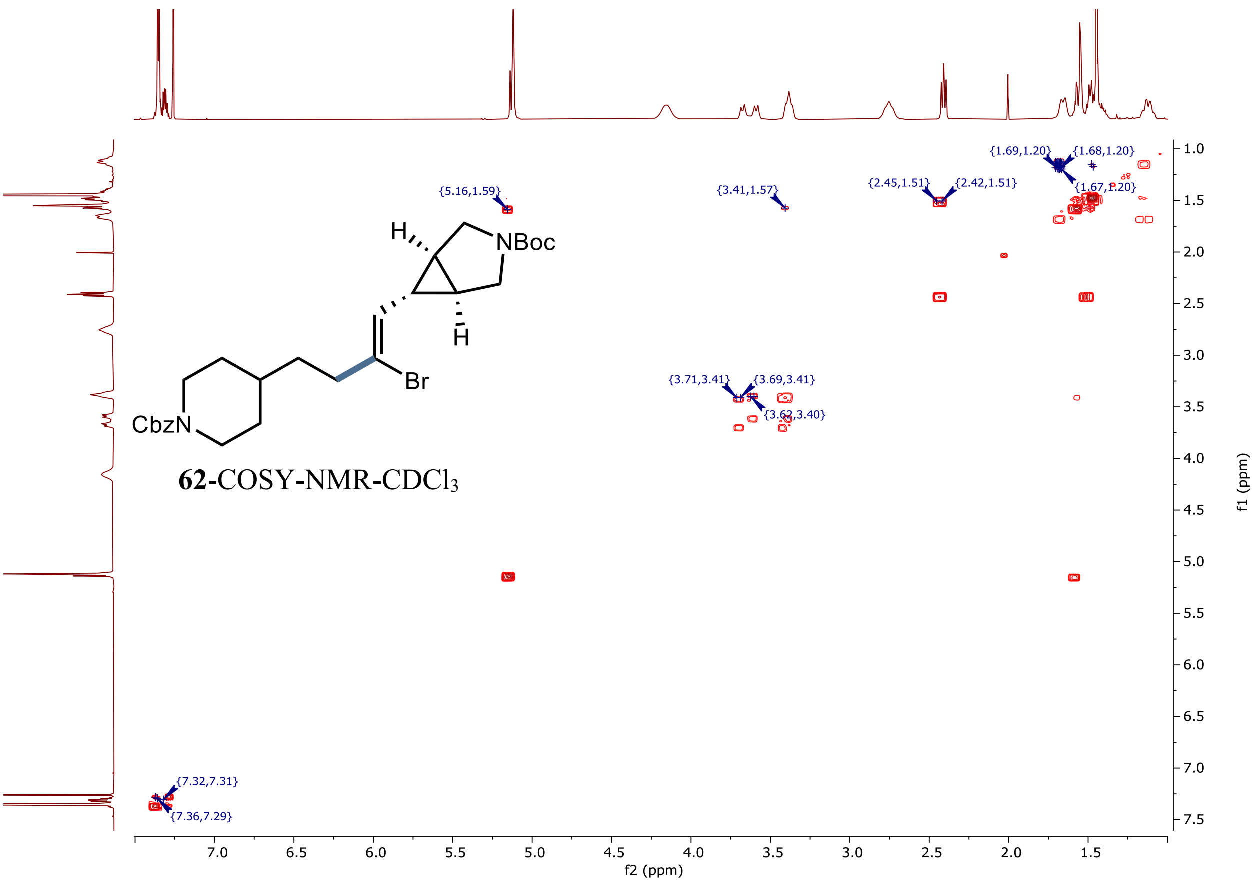
62-COSY-NMR-CDCl3
NBoc
Br
CbzN
f2 (ppm)
f1 (ppm)
{5.16,1.59}
{3.41,1.57}
{2.45,1.51}
{2.42,1.51}
{1.69,1.20}
{1.68,1.20}
{3.71,3.41}
{3.69,3.41}
{3.62,3.40}
{7.32,7.31}
{7.36,7.29}

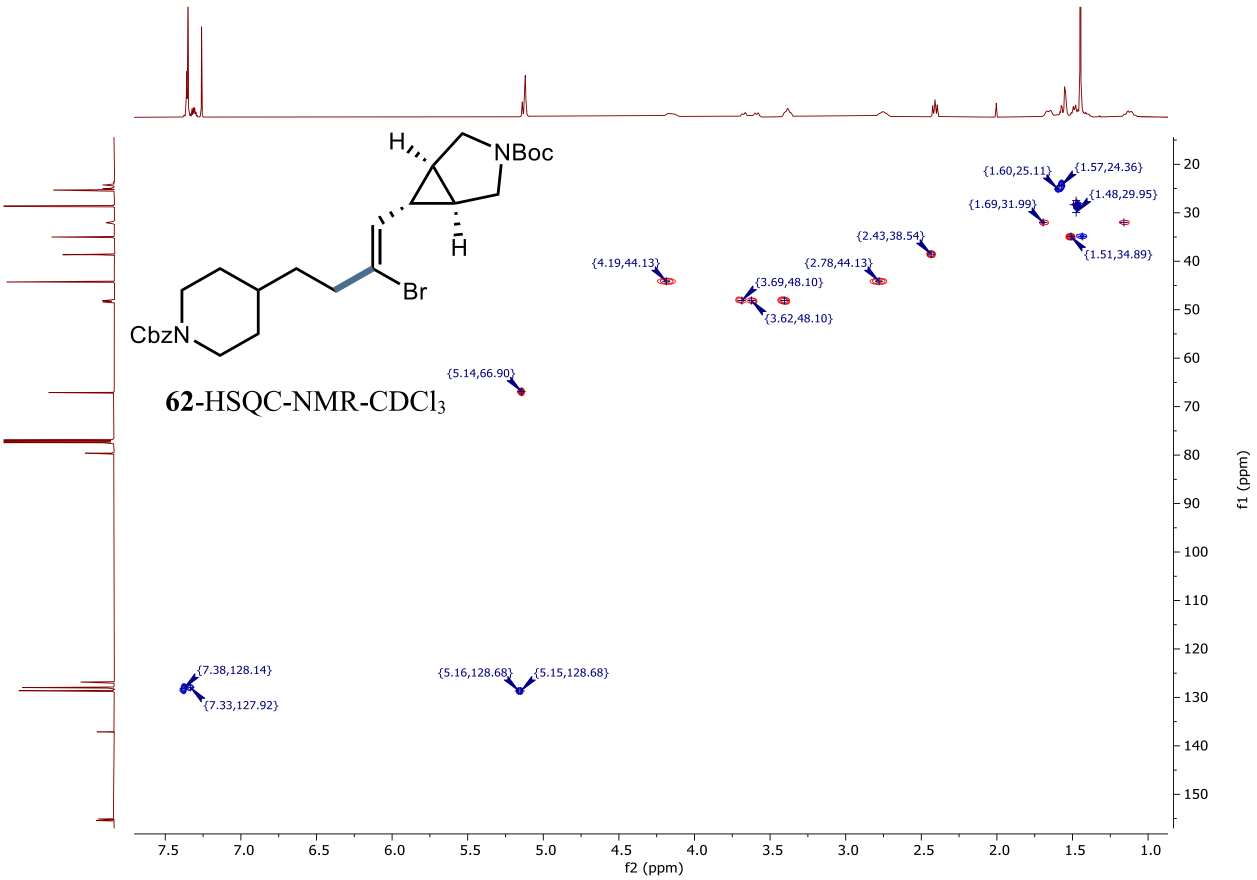
62-HSQC-NMR-CDCl3
NBoc
Br
CbzN
f2 (ppm)
f1 (ppm)
{1.60,25.11}
{1.57,24.36}
{1.48,29.95}
{1.69,31.99}
{2.43,38.54}
{1.51,34.89}
{4.19,44.13}
{2.78,44.13}
{3.69,48.10}
{3.62,48.10}
{5.14,66.90}
{7.38,128.14}
{7.33,127.92}
{5.16,128.68}
{5.15,128.68}

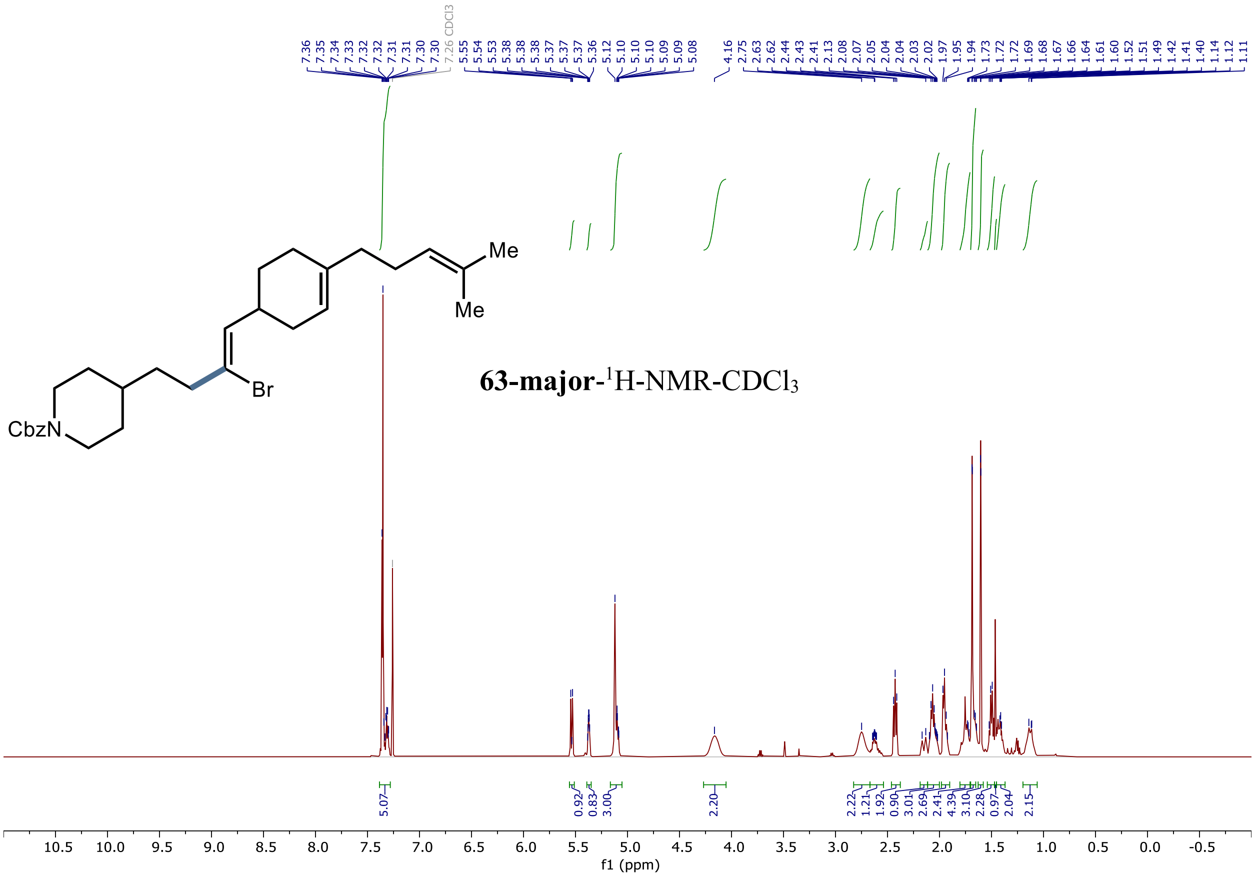
63-major-^{1}H-NMR-CDCl$_3$
Me
Me
Br
CbzN
f1 (ppm)

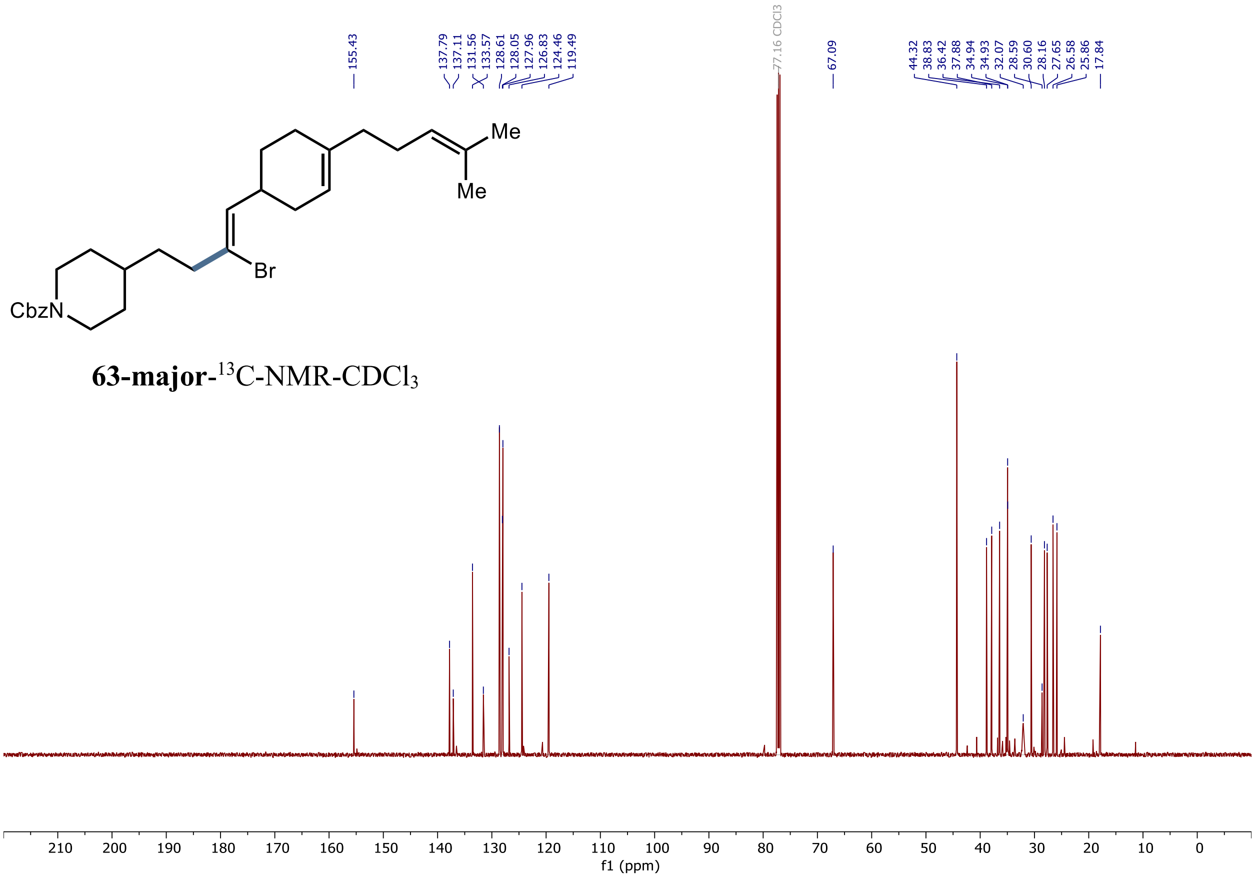
63-major-^{13}C-NMR-CDCl$_3$
Me
Me
Br
CbzN
f1 (ppm)

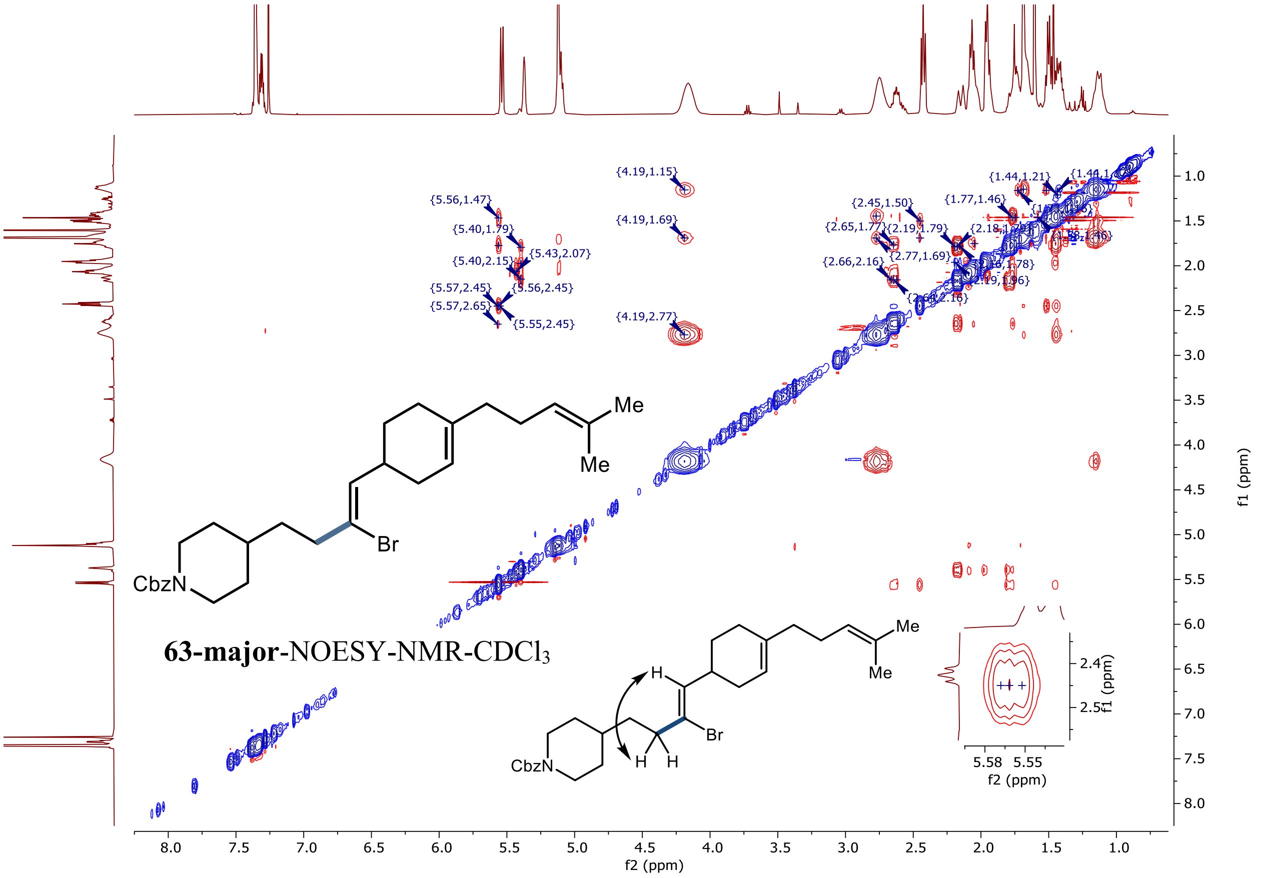
63-major-NOESY-NMR-CDCl3
CbzN
Br
Me
Me
f2 (ppm)
f1 (ppm)

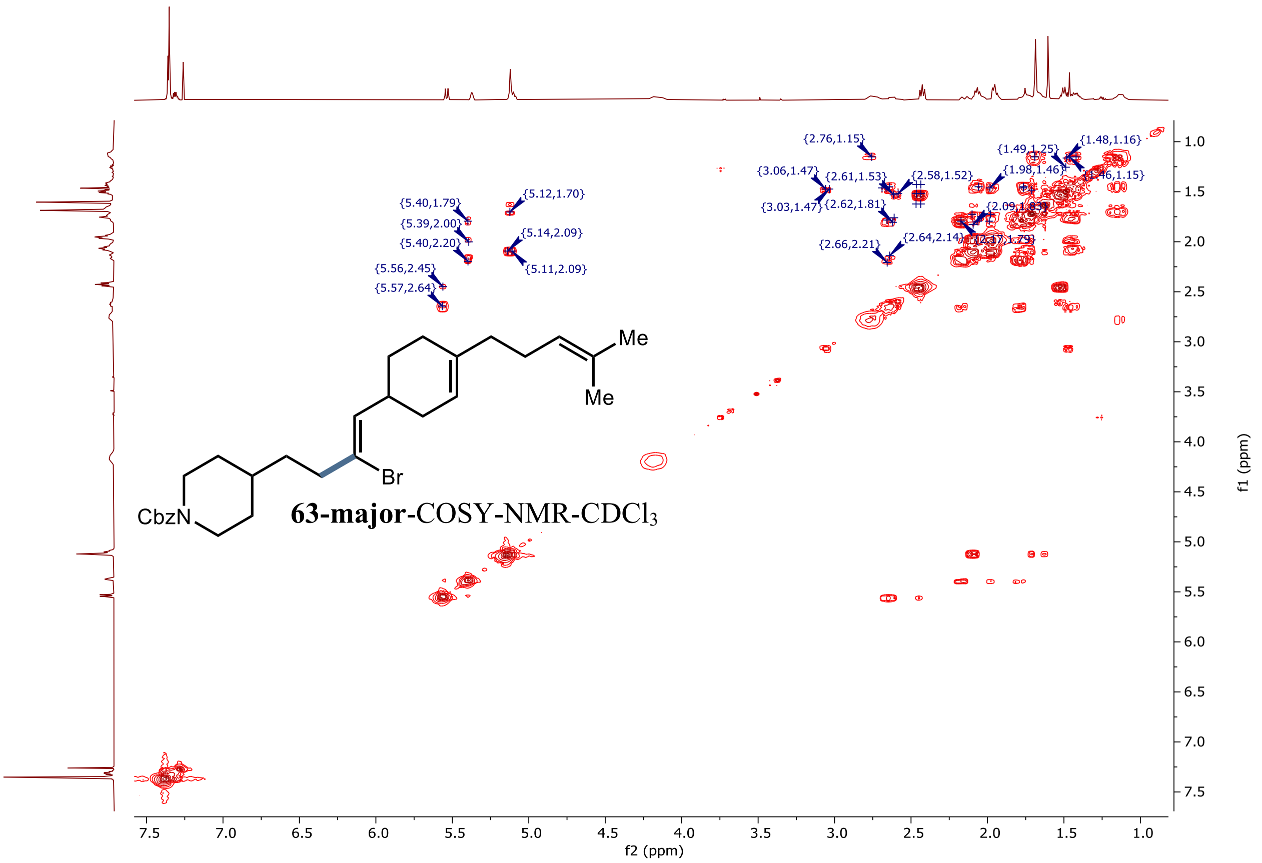
63-major-COSY-NMR-CDCl3
CbzN
Br
Me
Me
f2 (ppm)
f1 (ppm)

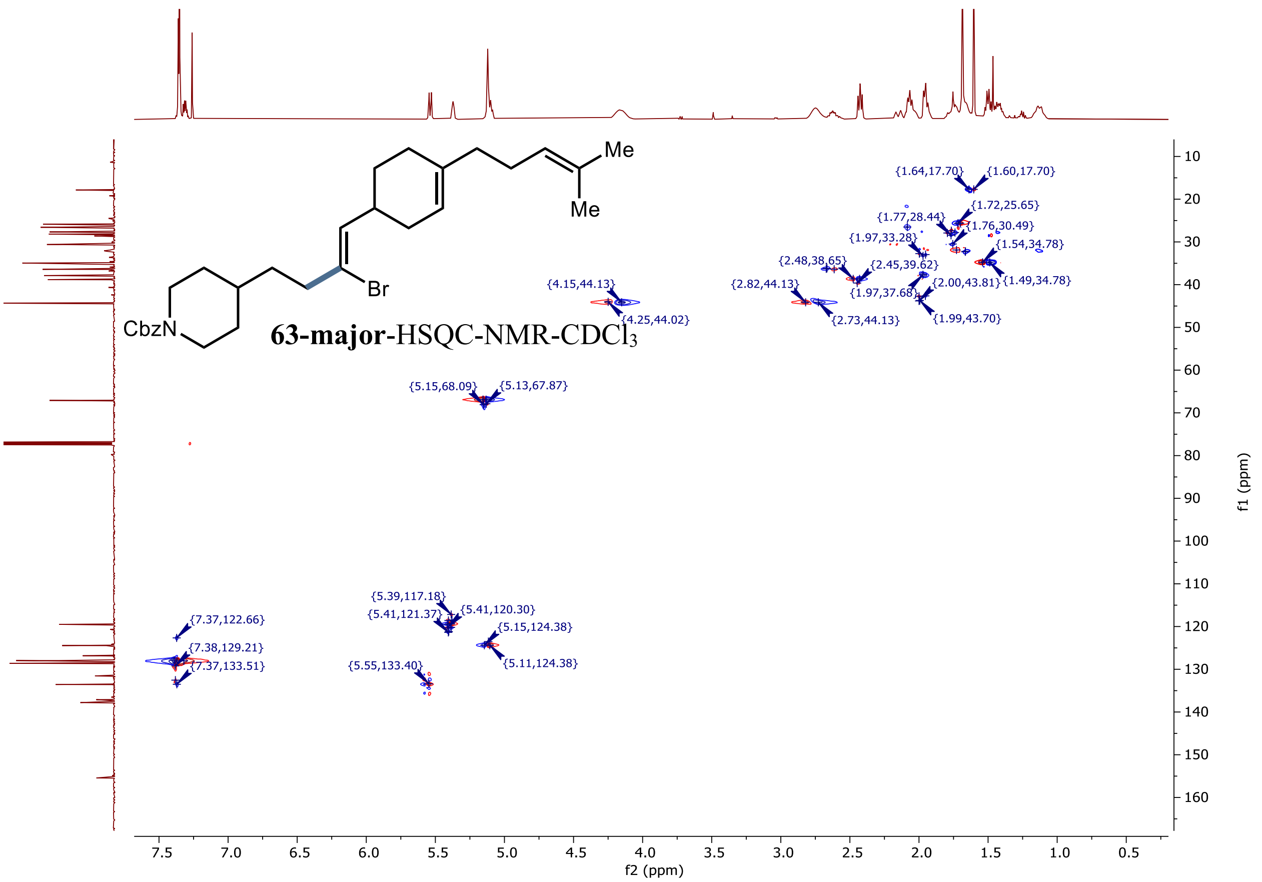
Me
Me
Br
CbzN
63-major-HSQC-NMR-CDCl3
f2 (ppm)
f1 (ppm)

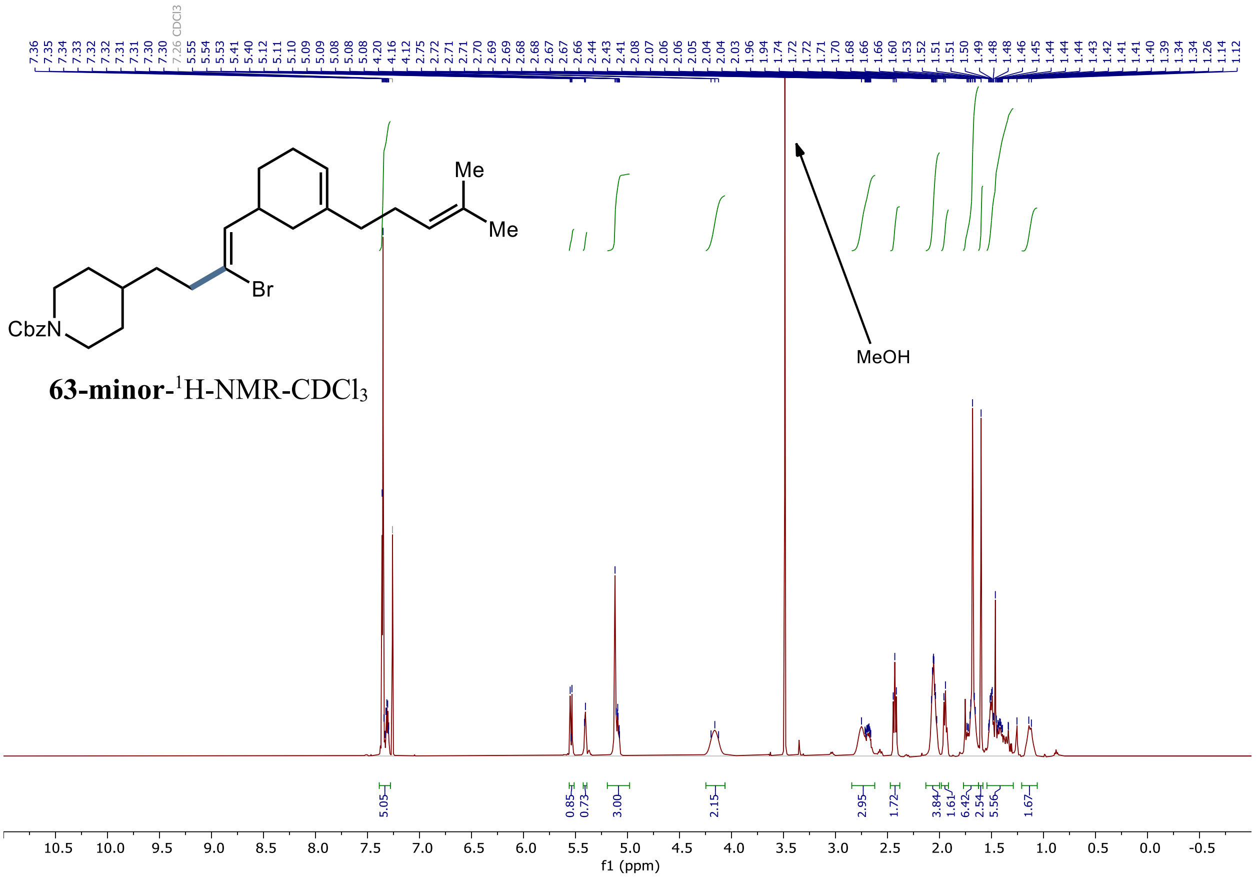
Me
Me
Br
CbzN
63-minor-1H-NMR-CDCl3
MeOH
f1 (ppm)

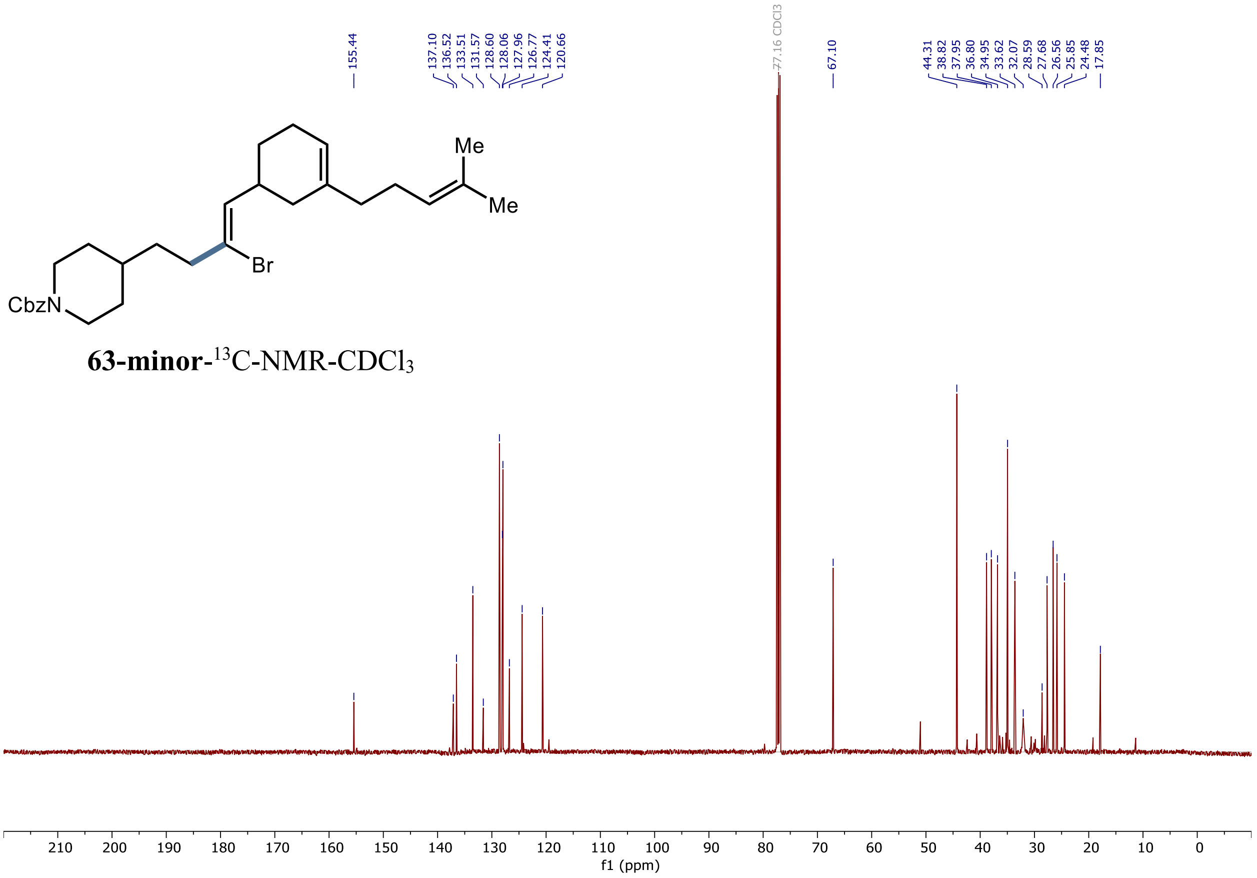

63-minor-^{13}C-NMR-$CDCl_3$

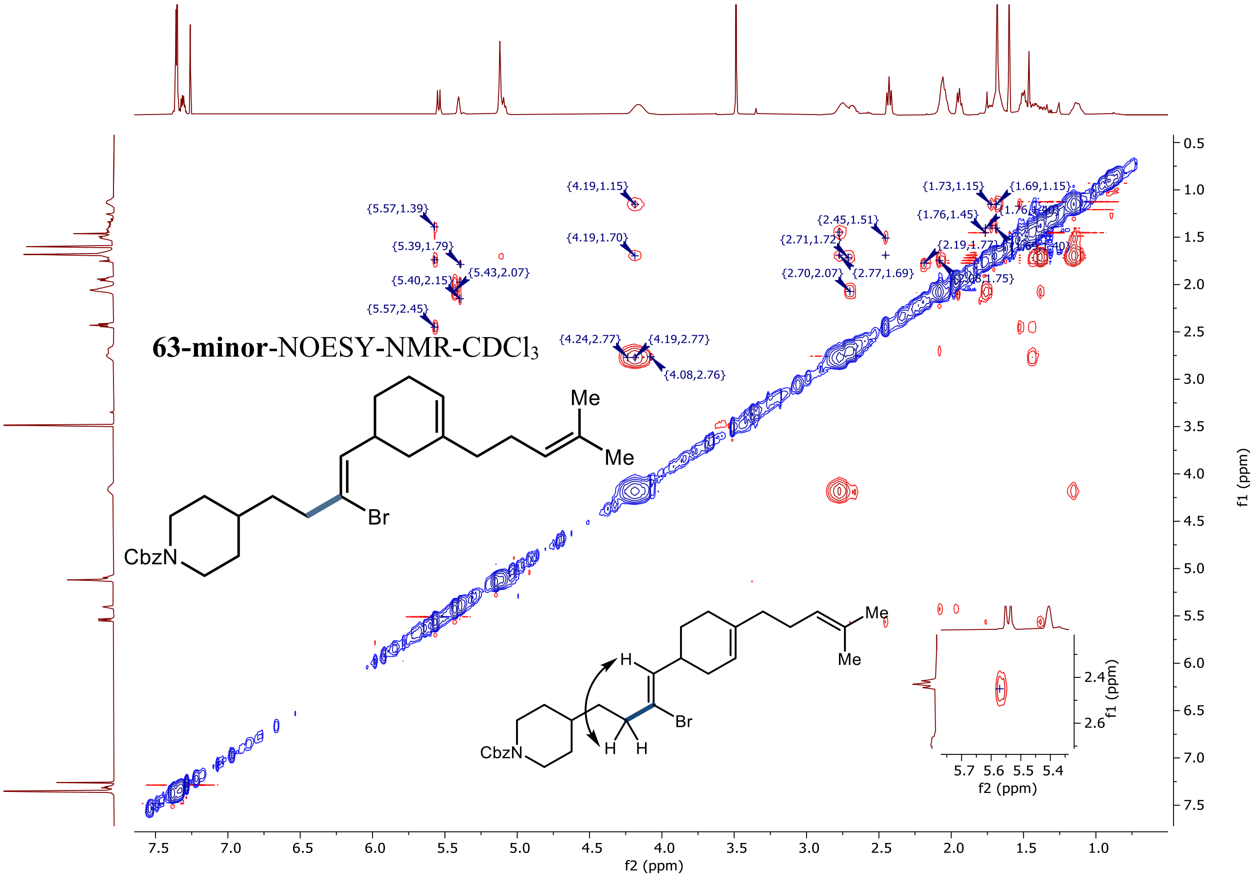

63-minor-NOESY-NMR-$CDCl_3$

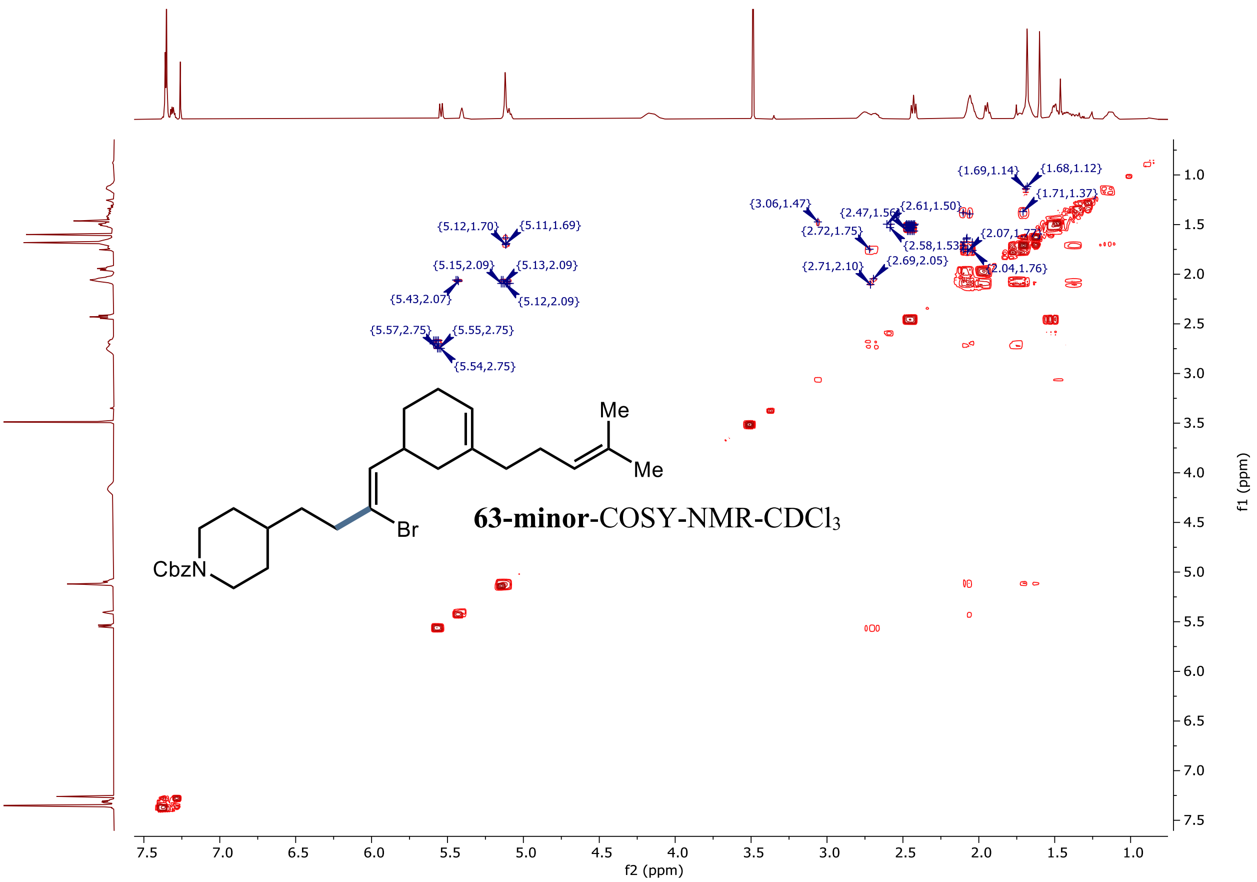
63-minor-COSY-NMR-CDCl3
f2 (ppm)
f1 (ppm)
CbzN
Br
Me
Me

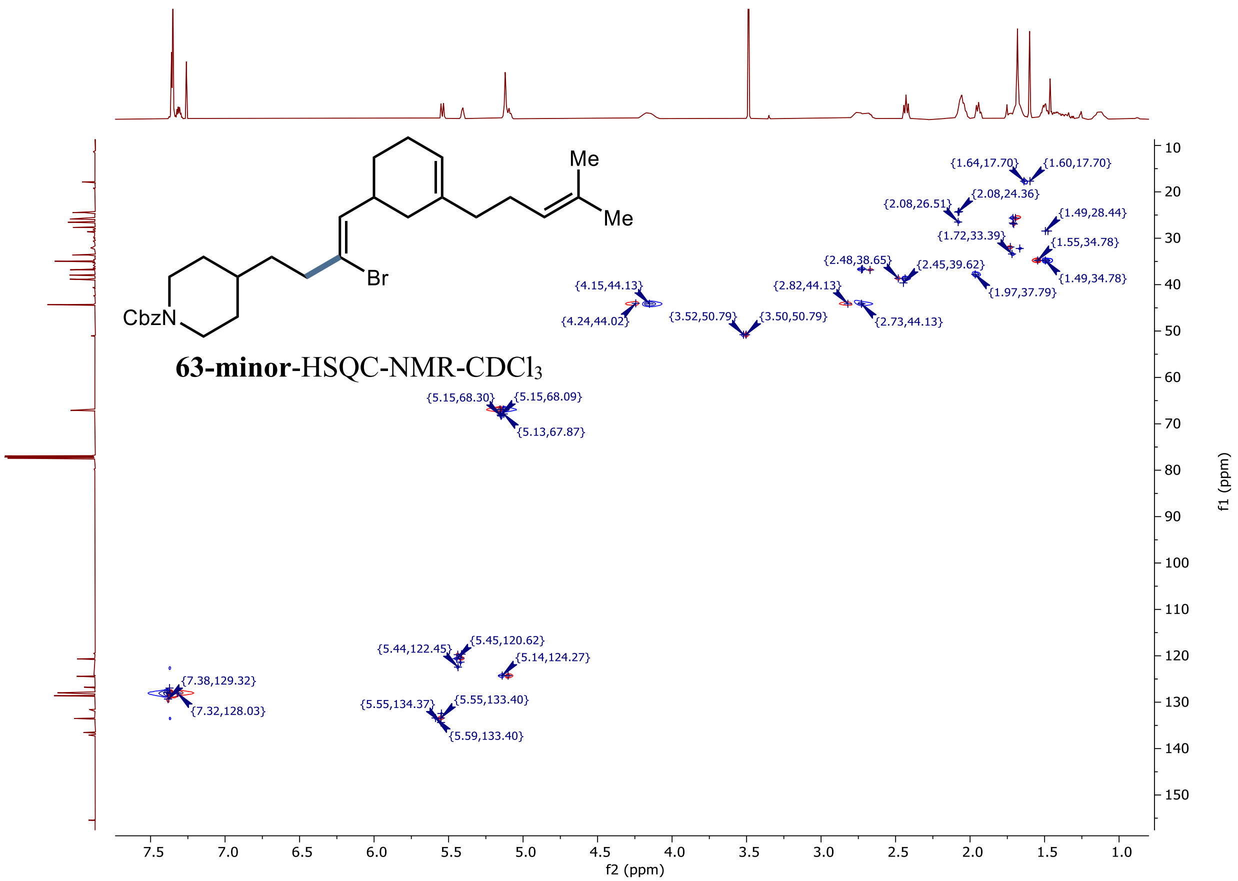
63-minor-HSQC-NMR-CDCl3
f2 (ppm)
f1 (ppm)
CbzN
Br
Me
Me

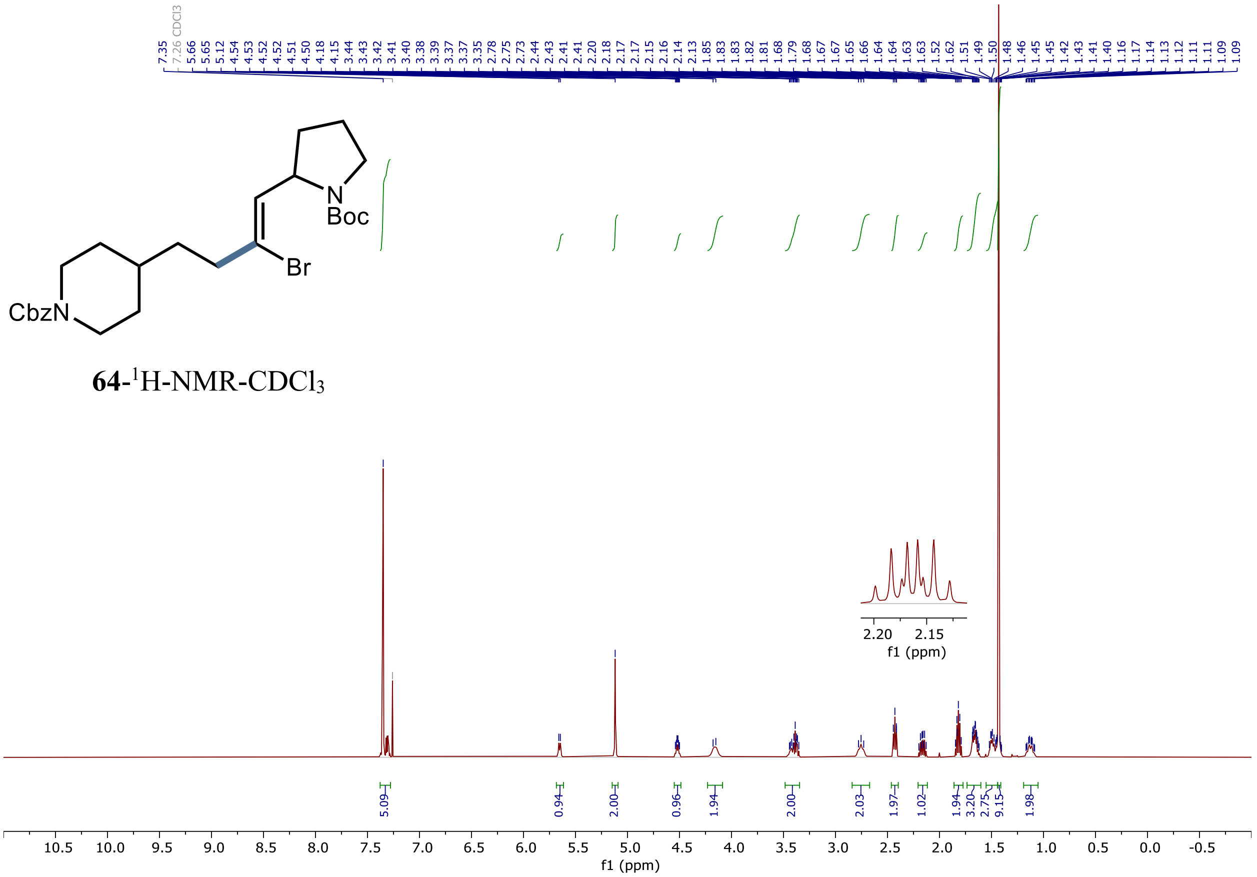

64-^{1}H-NMR-$CDCl_3$

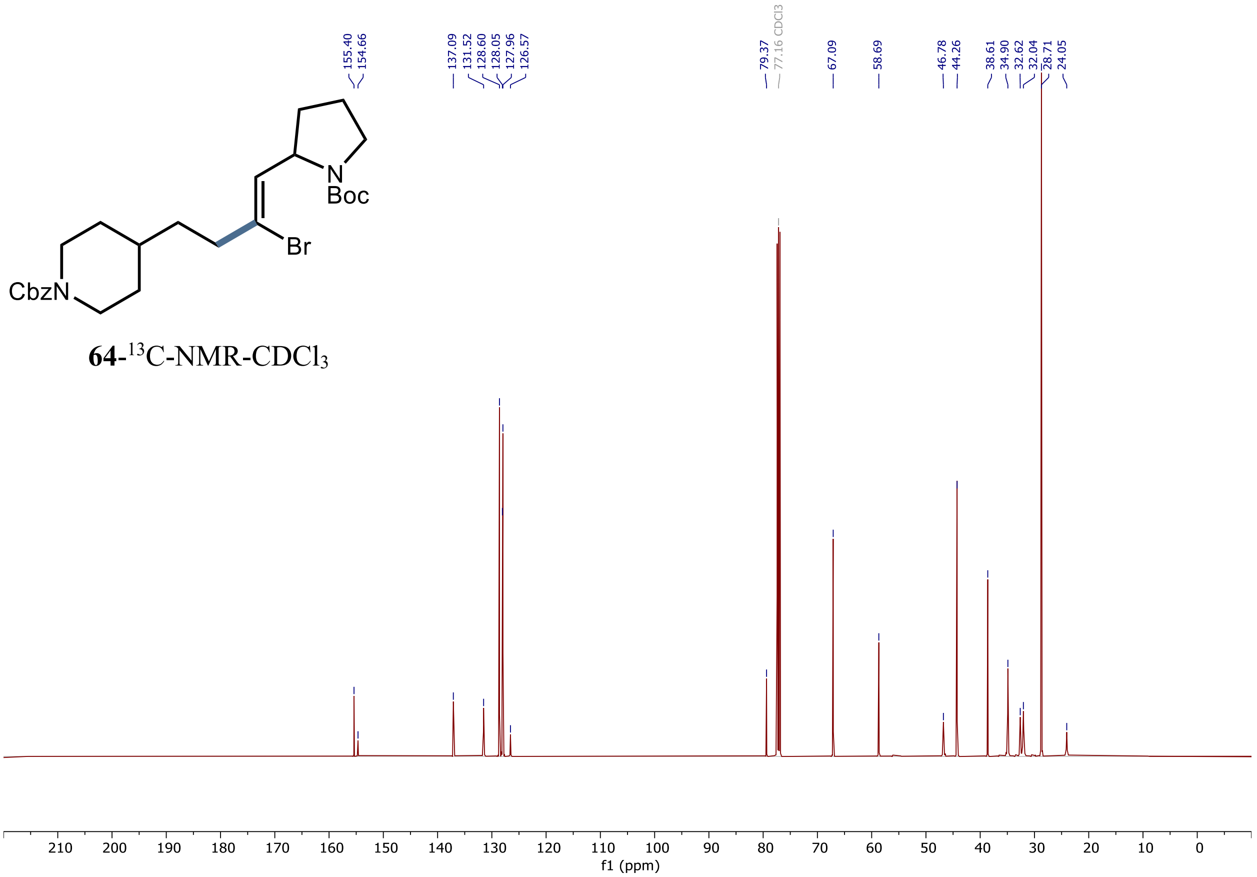

64-^{13}C-NMR-$CDCl_3$

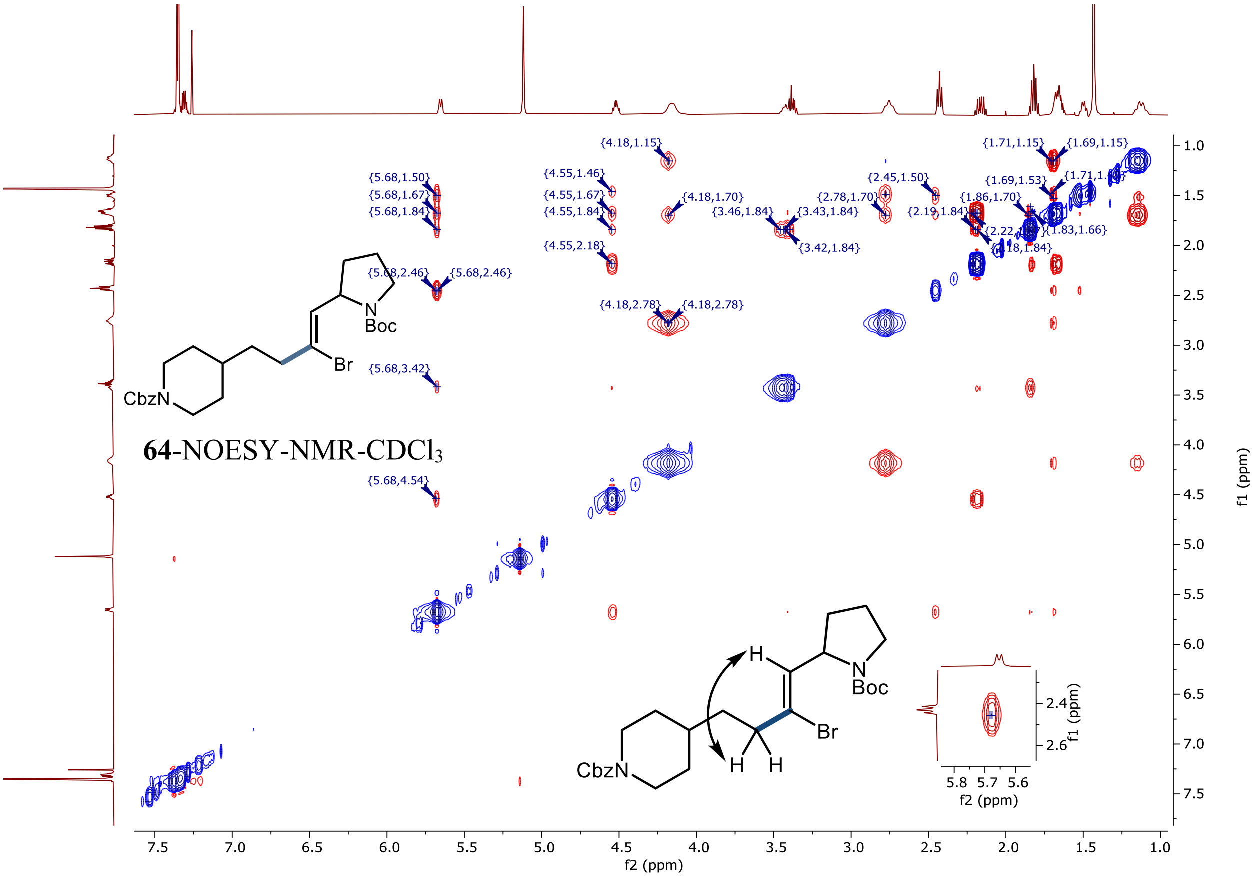
64-NOESY-NMR-CDCl3
CbzN
Boc
Br
f2 (ppm)
f1 (ppm)

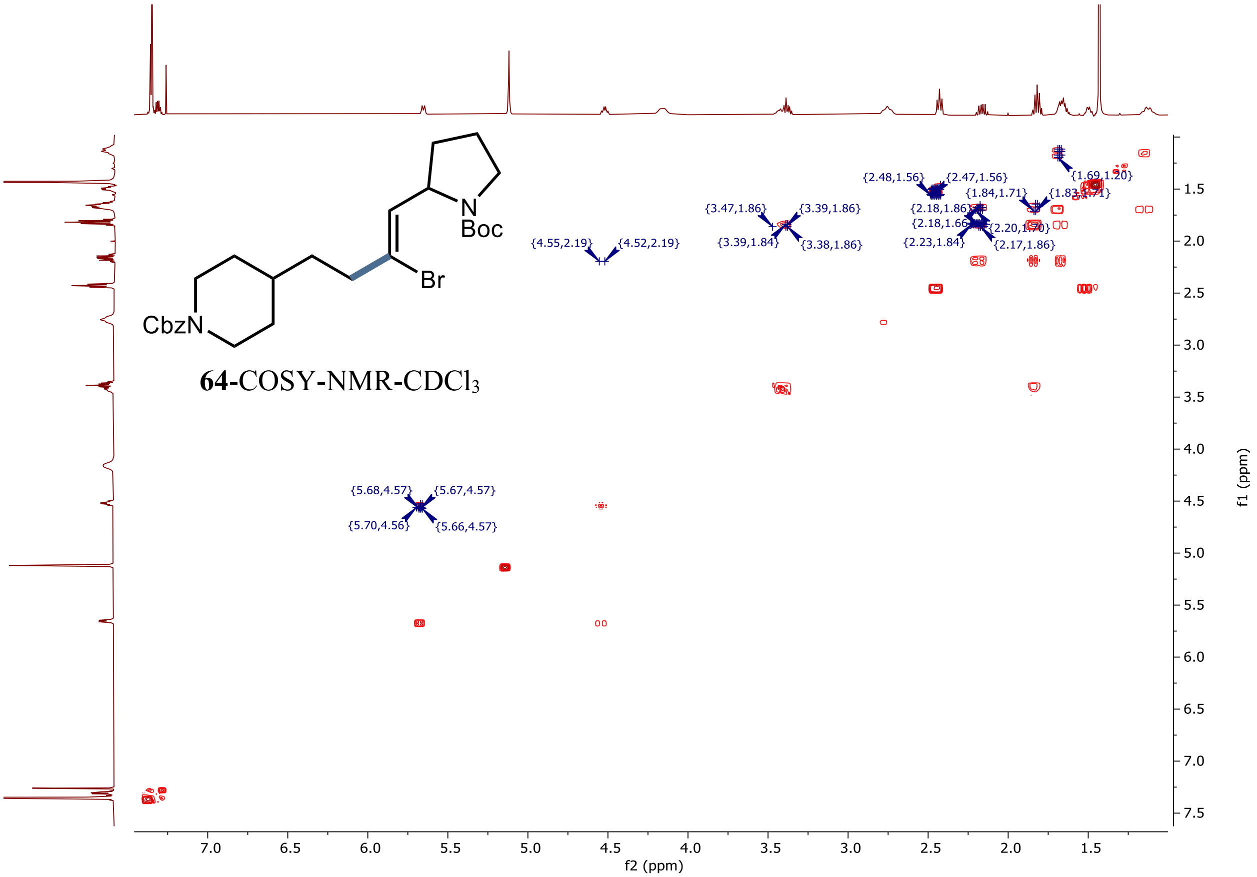
64-COSY-NMR-CDCl3
CbzN
Boc
Br
f2 (ppm)
f1 (ppm)

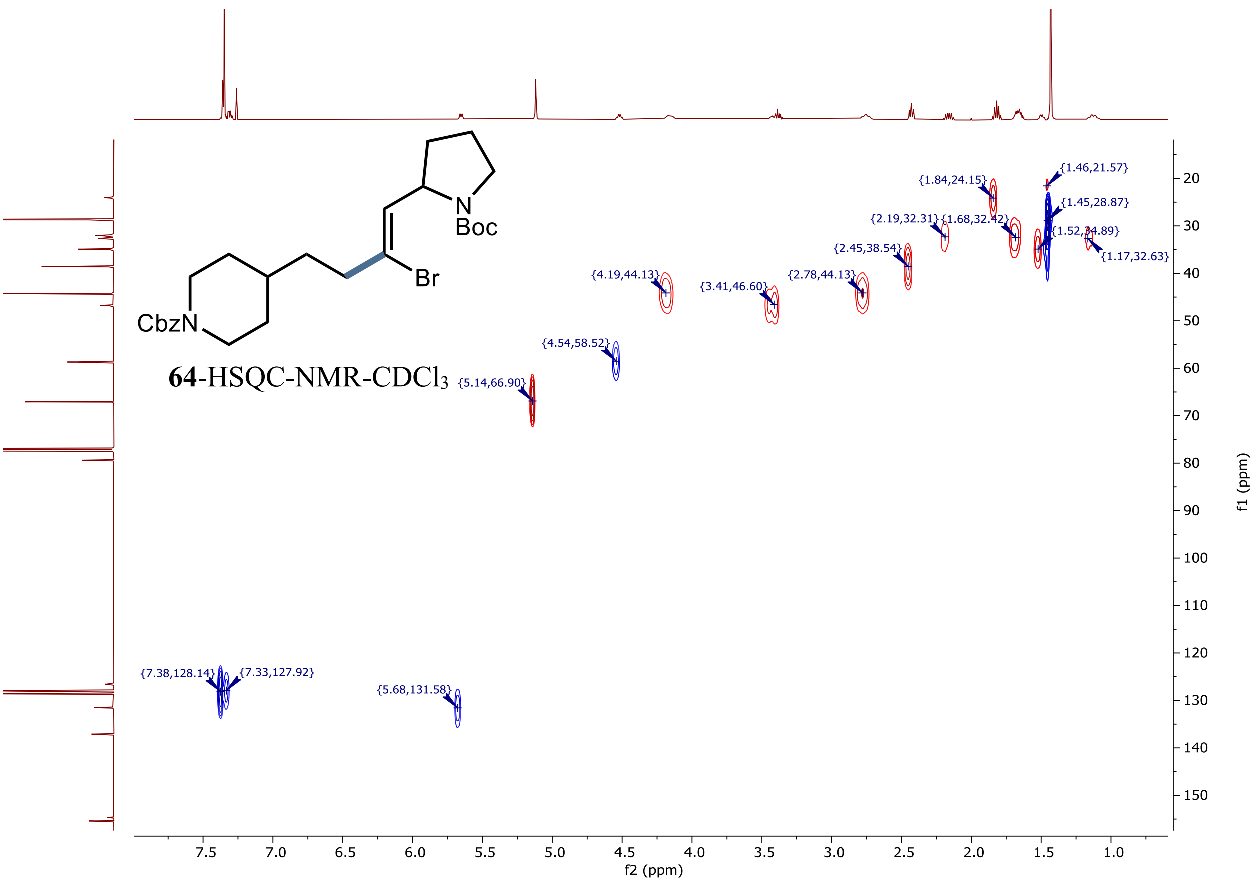

64-HSQC-NMR-$CDCl_3$

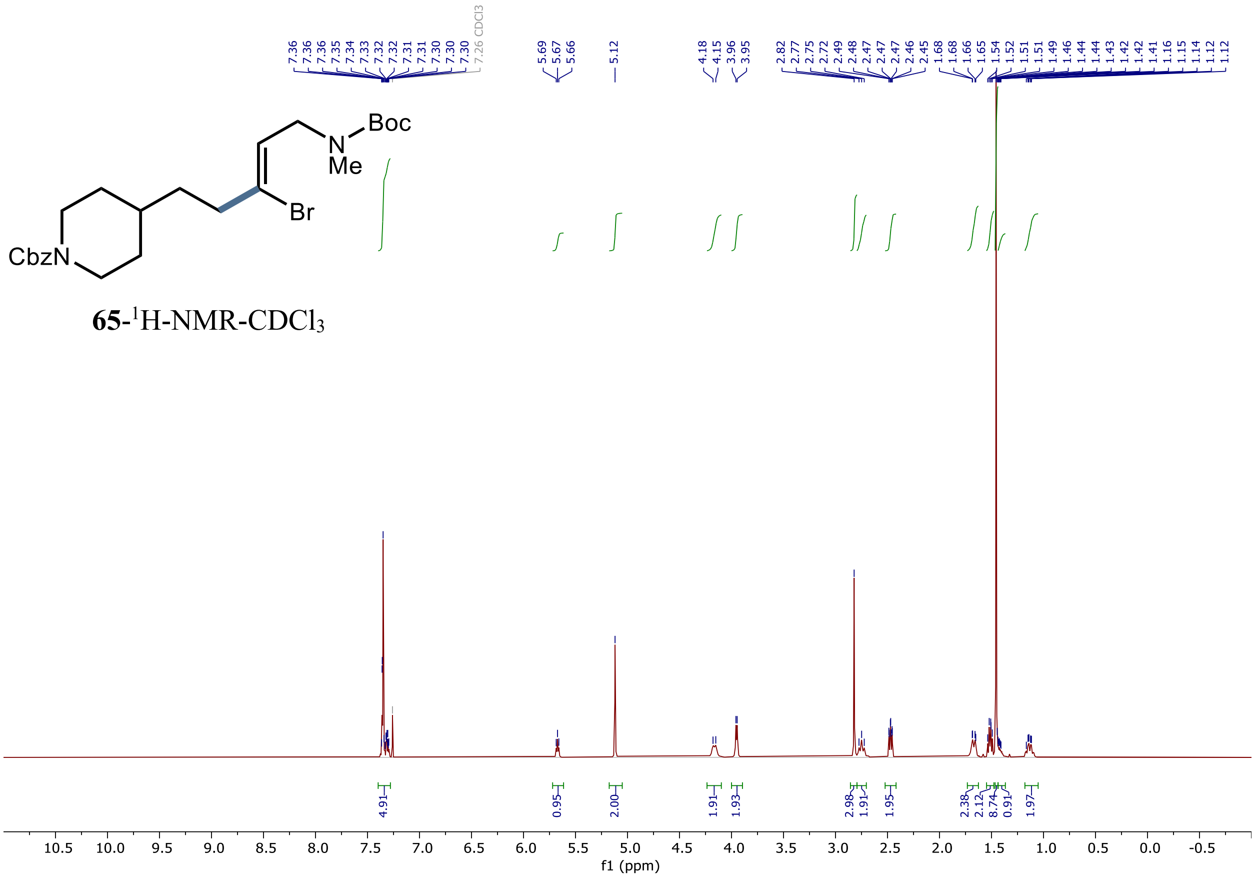

65-^{1}H-NMR-$CDCl_3$

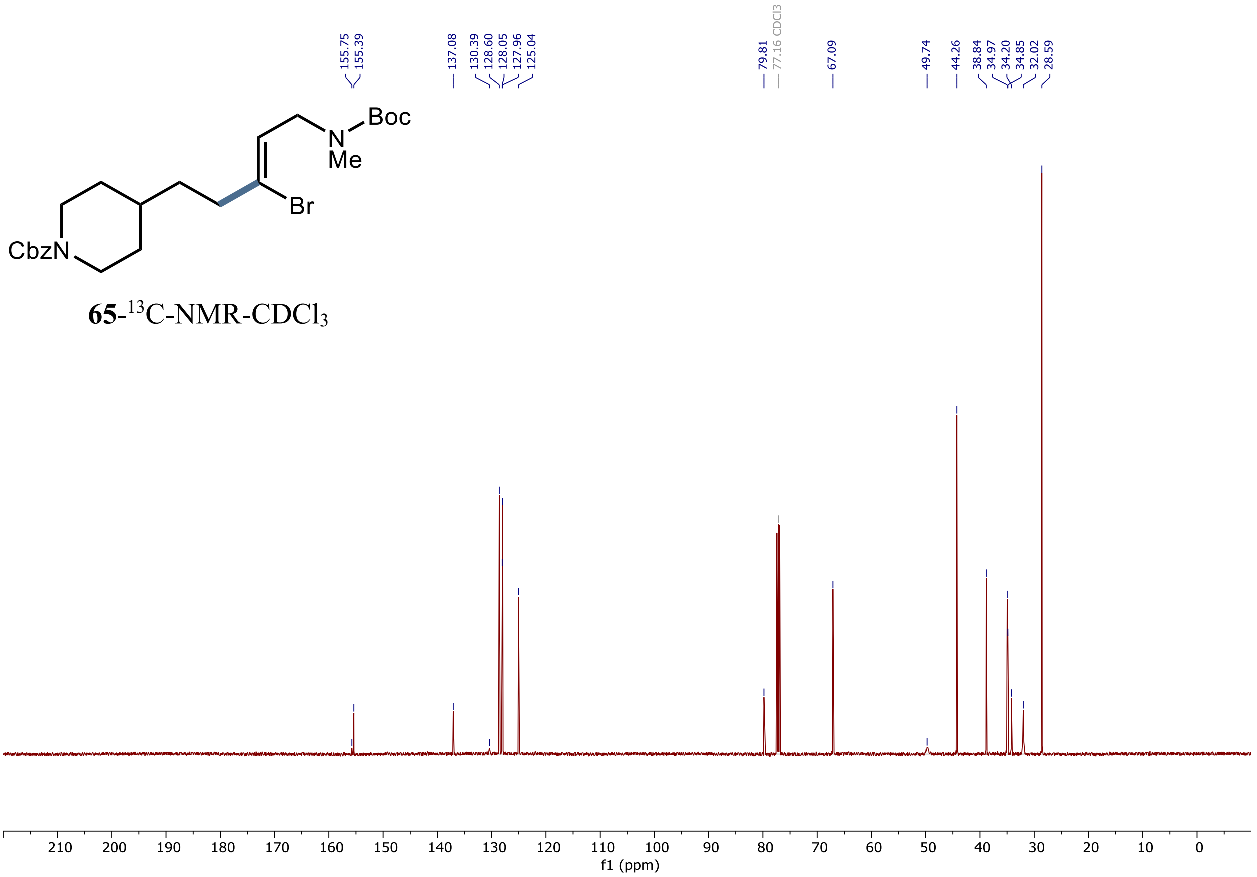

65-^{13}C-NMR-CDCl$_3$

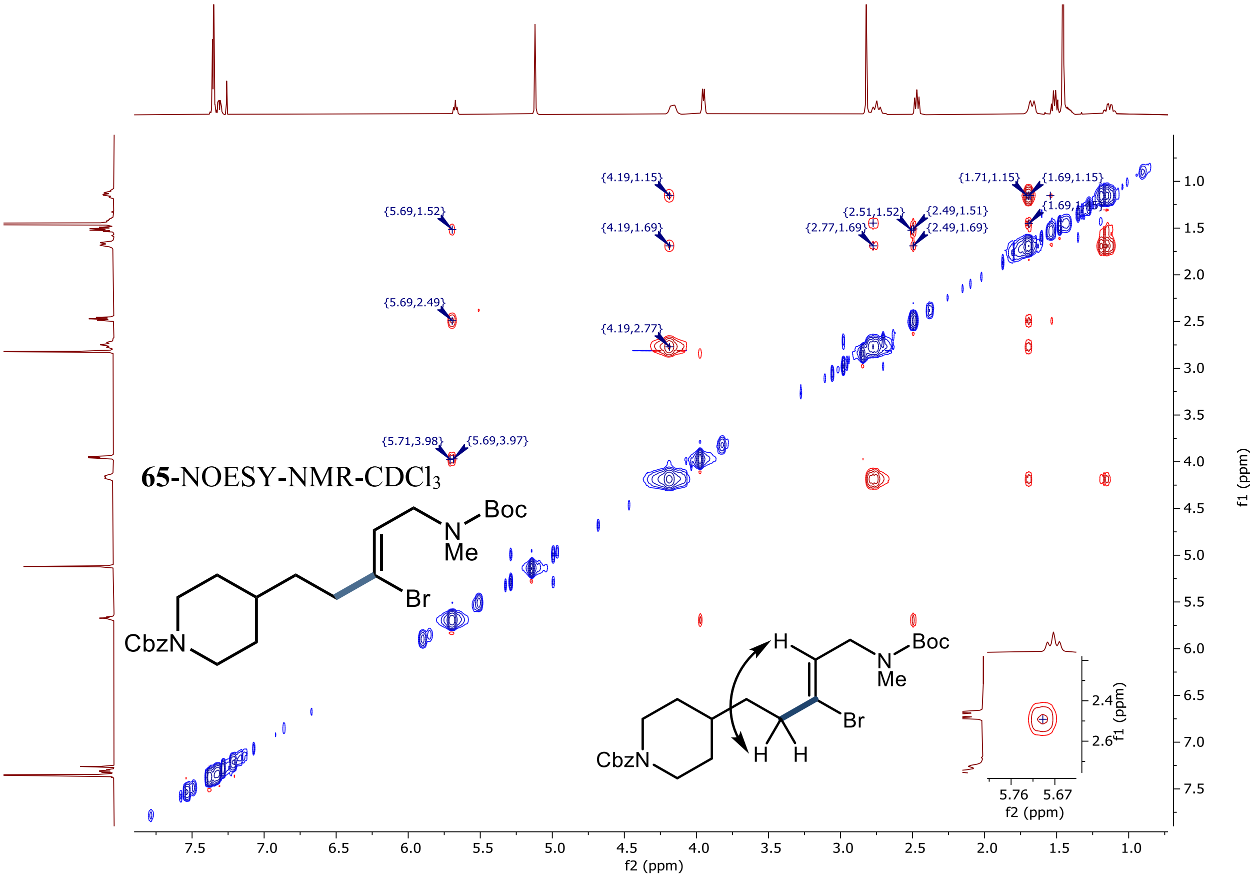

65-NOESY-NMR-CDCl$_3$

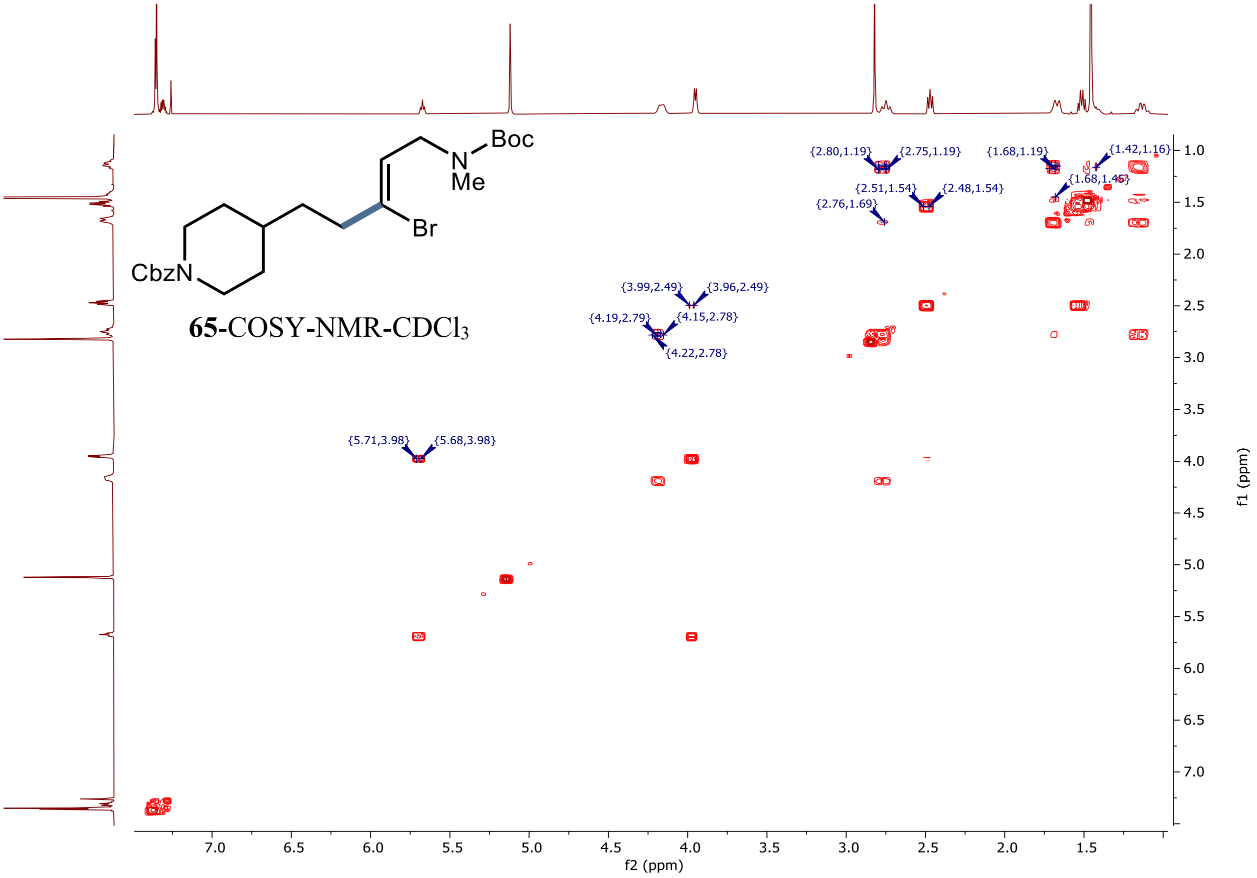
Boc
N
Me
Br
CbzN
65-COSY-NMR-CDCl3
f1 (ppm)
f2 (ppm)

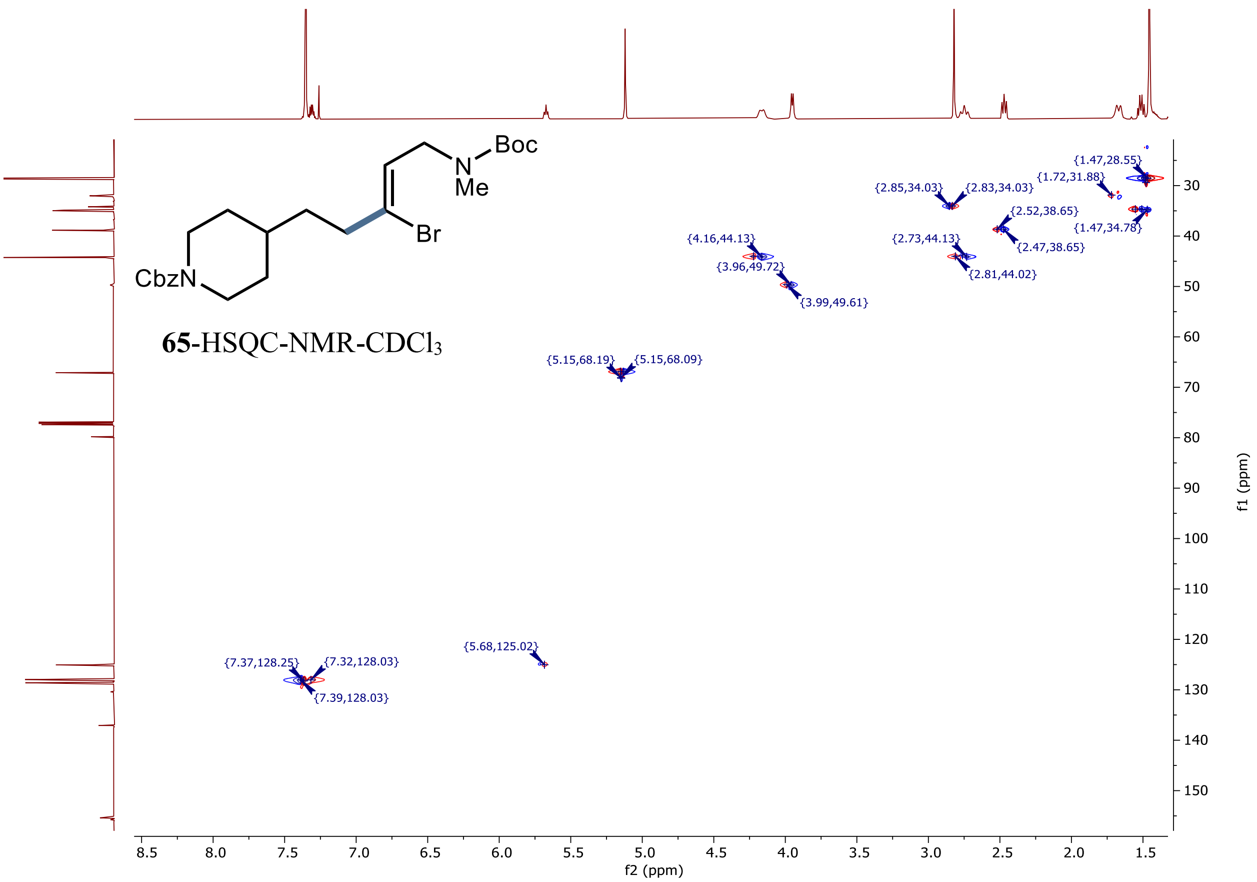
65-HSQC-NMR-CDCl3
f2 (ppm)
f1 (ppm)

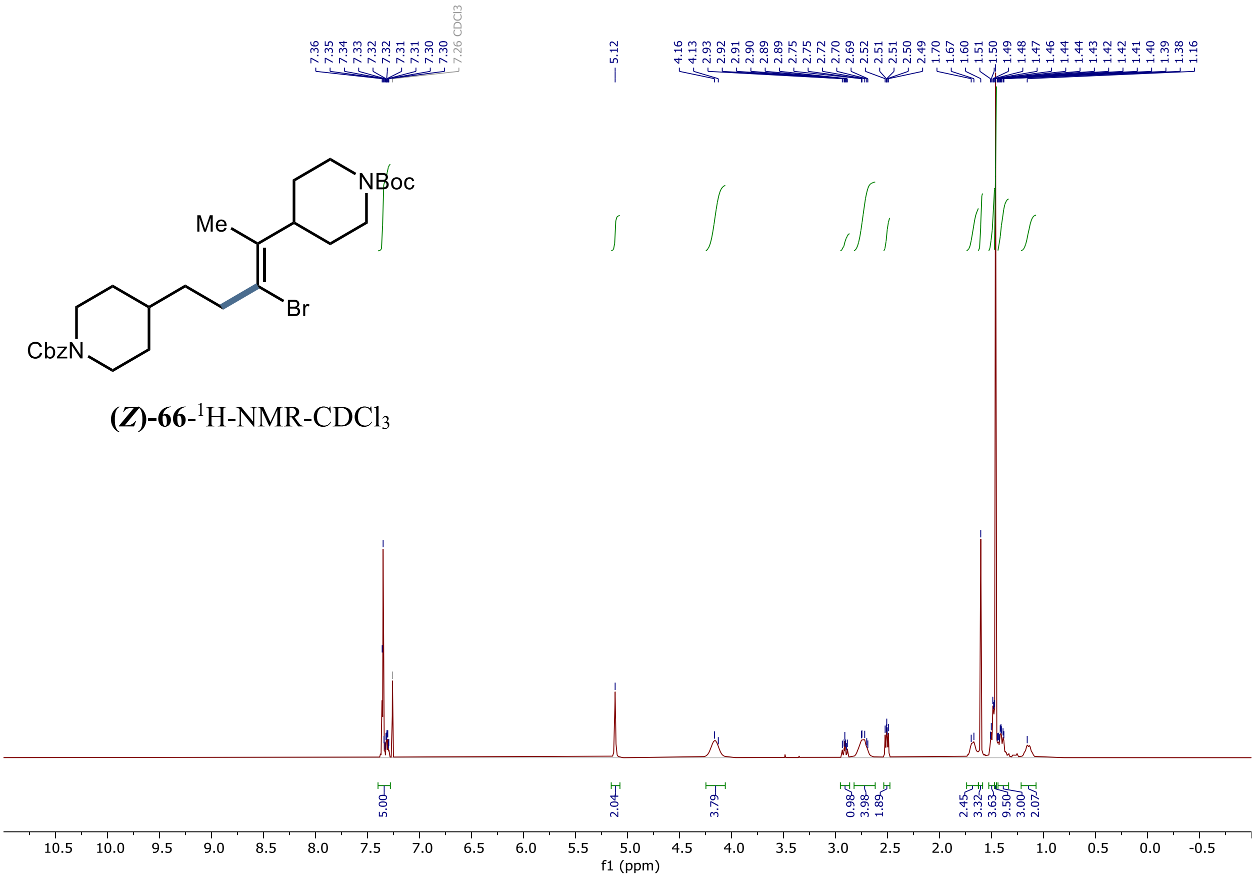
(Z)-66-1H-NMR-CDCl3
f1 (ppm)

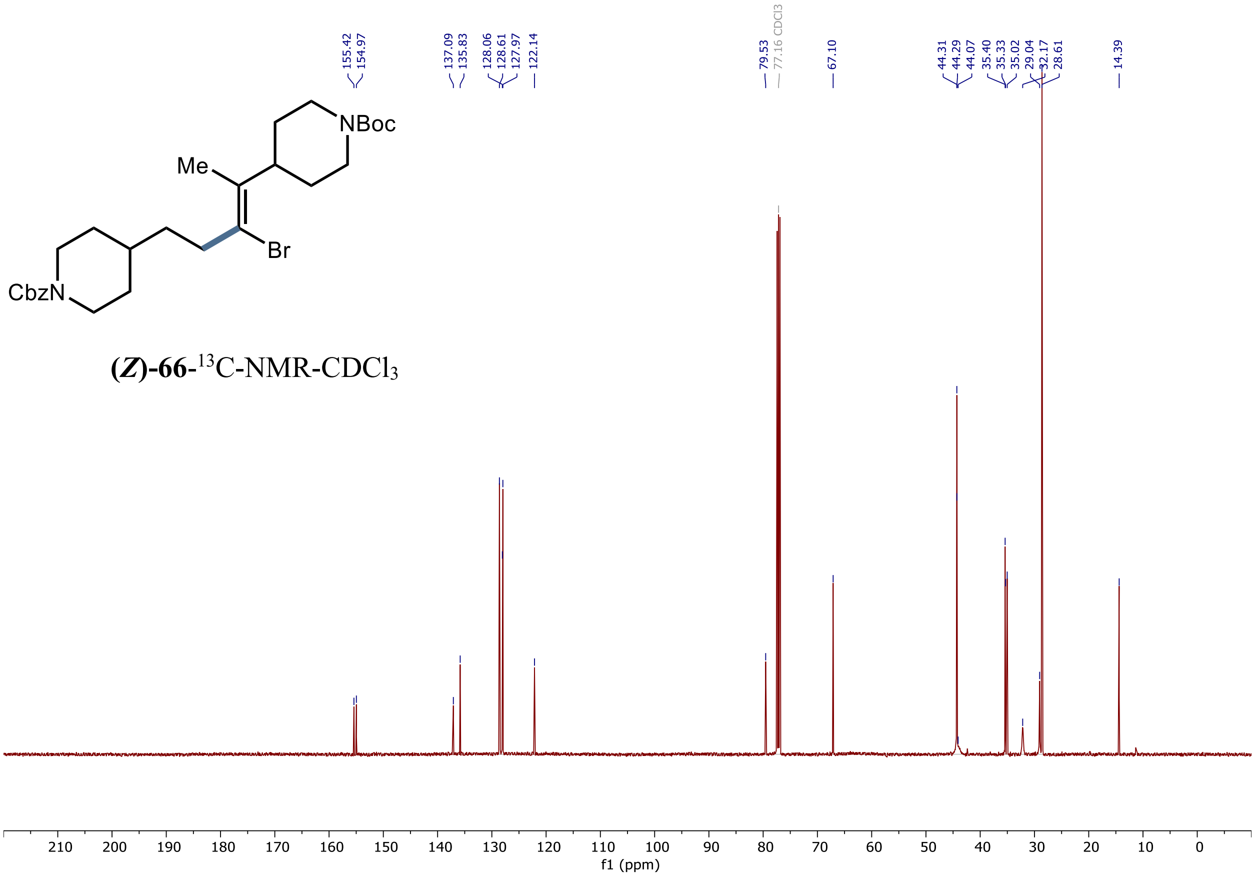

(*Z*)-66-^{13}C-NMR-$CDCl_3$

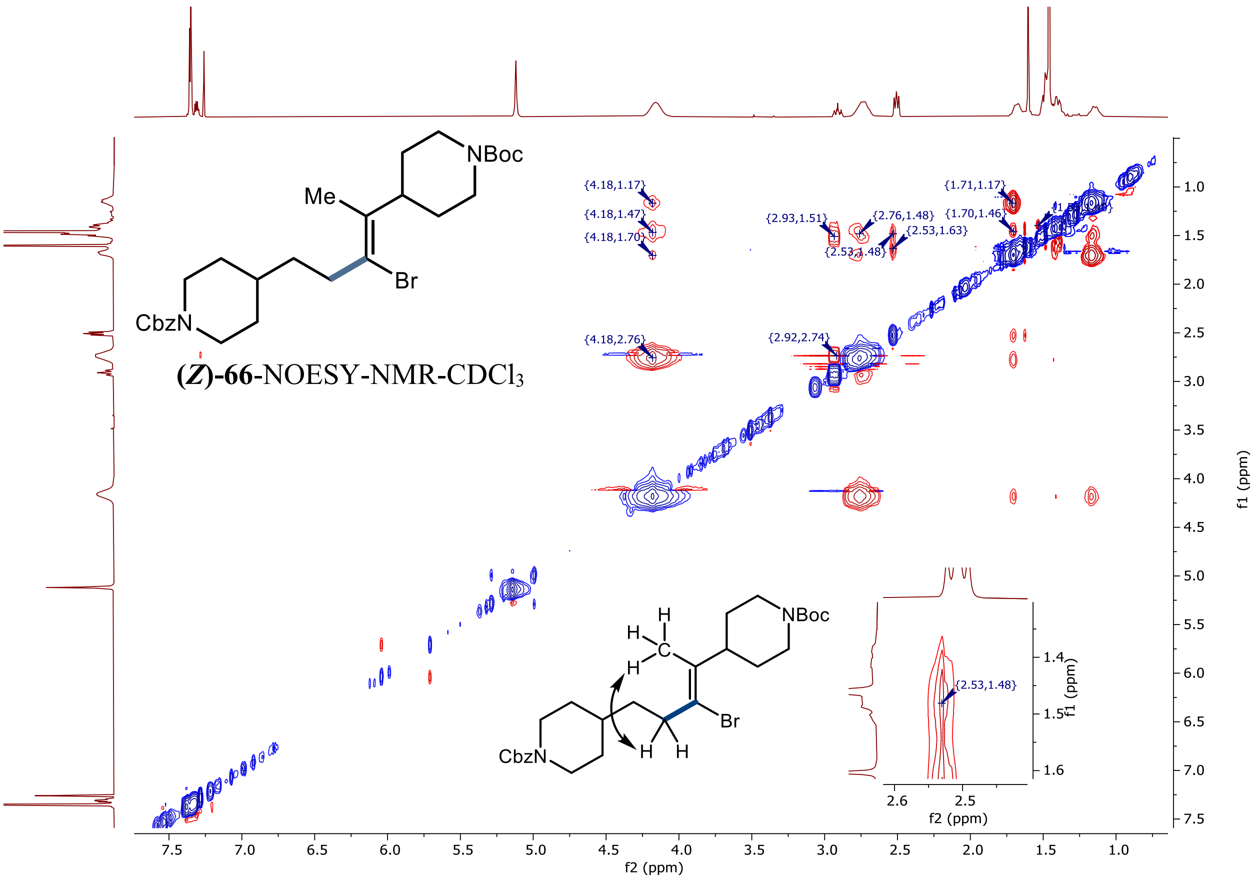

(*Z*)-66-NOESY-NMR-$CDCl_3$

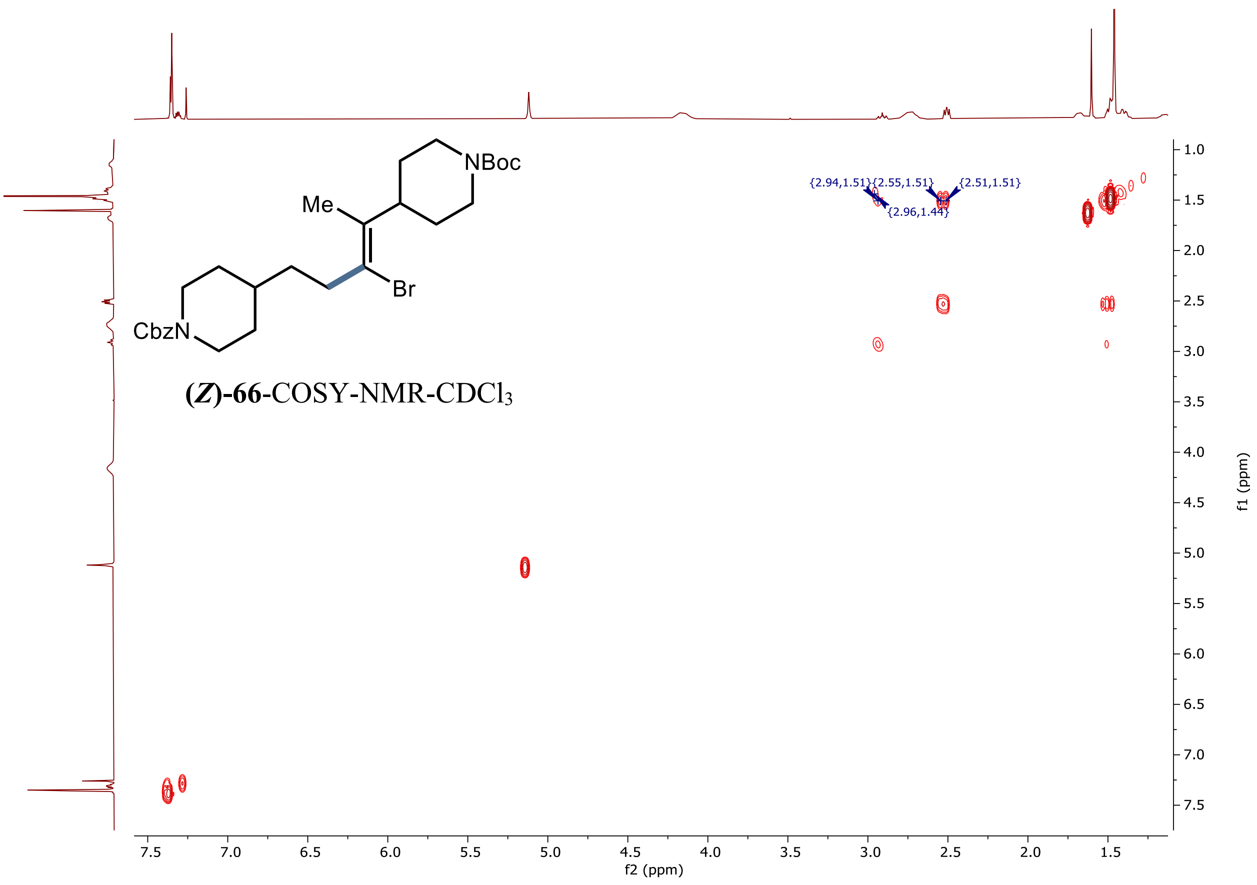
NBoc
Me
Br
CbzN
(Z)-66-COSY-NMR-CDCl3
{2.94,1.51}
{2.55,1.51}
{2.51,1.51}
{2.96,1.44}
f1 (ppm)
f2 (ppm)

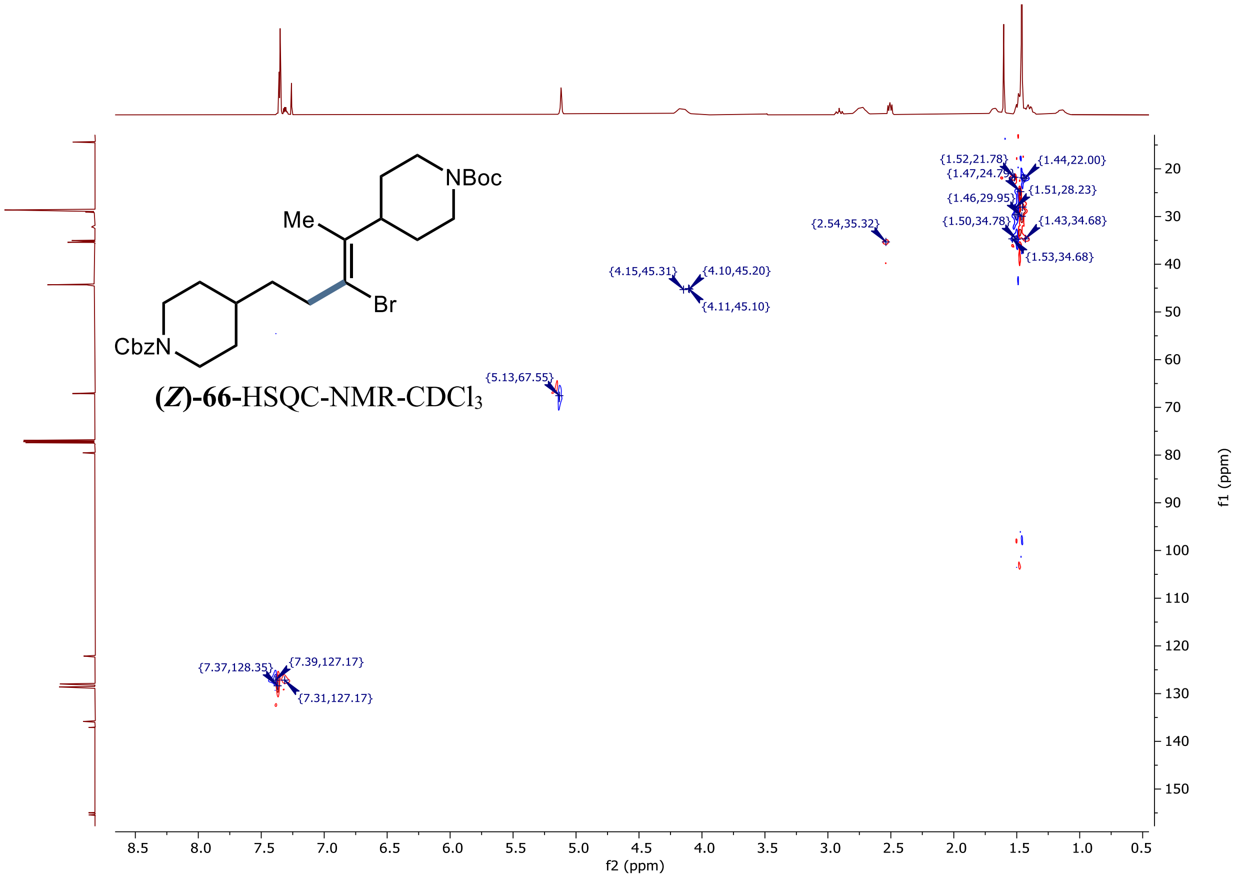
NBoc
Me
Br
CbzN
(Z)-66-HSQC-NMR-CDCl3
{1.52,21.78}
{1.44,22.00}
{1.51,28.23}
{1.46,29.95}
{1.50,34.78}
{1.43,34.68}
{1.53,34.68}
{2.54,35.32}
{4.15,45.31}
{4.10,45.20}
{4.11,45.10}
{5.13,67.55}
{7.37,128.35}
{7.39,127.17}
{7.31,127.17}
f1 (ppm)
f2 (ppm)

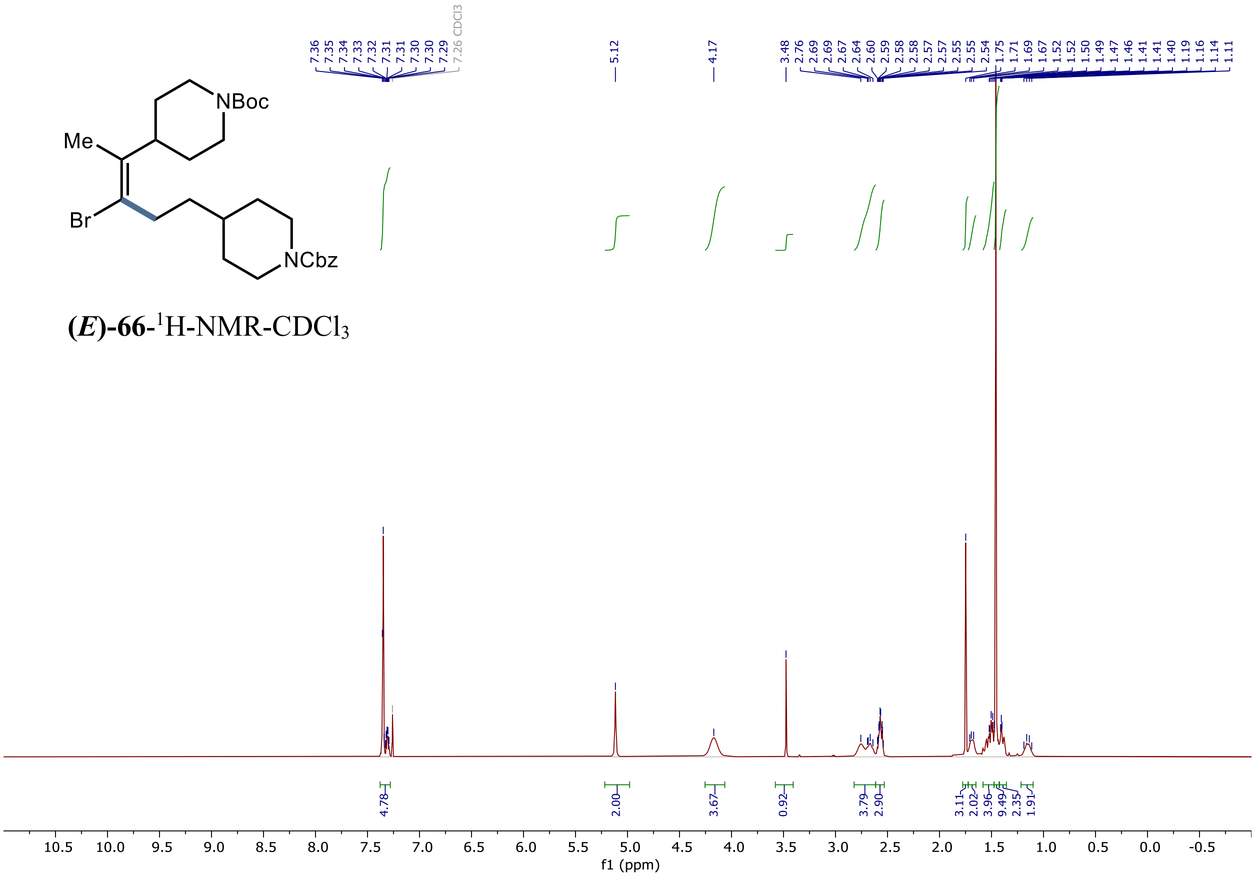
NBoc
Me
Br
NCbz
(E)-66-1H-NMR-CDCl3
f1 (ppm)

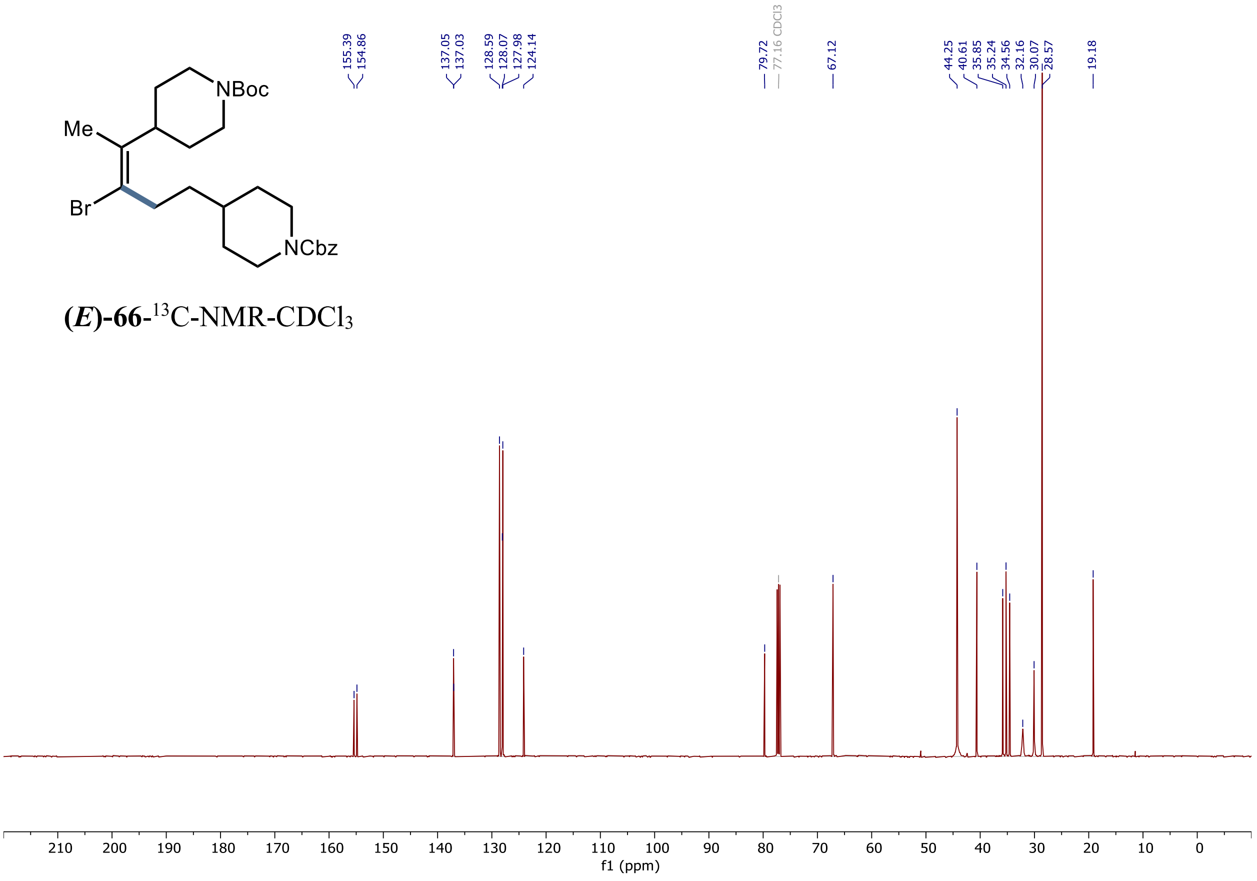
NBoc
Me
Br
NCbz
(E)-66-13C-NMR-CDCl3
f1 (ppm)

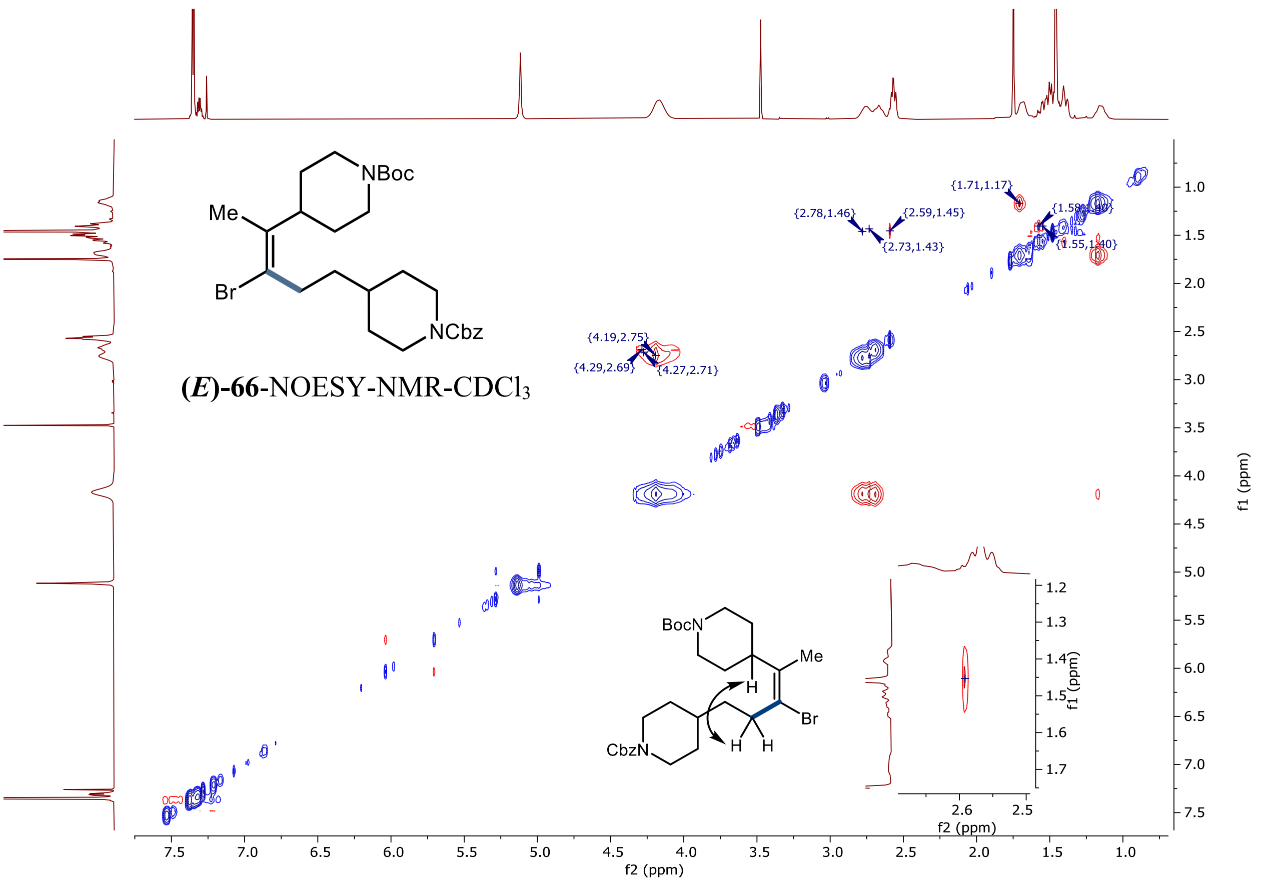
NBoc
Me
Br
NCbz
(E)-66-NOESY-NMR-CDCl3
{1.71,1.17}
{2.78,1.46}
{2.59,1.45}
{2.73,1.43}
{1.55,1.40}
{4.19,2.75}
{4.29,2.69}
{4.27,2.71}
BocN
CbzN
H
f2 (ppm)
f1 (ppm)

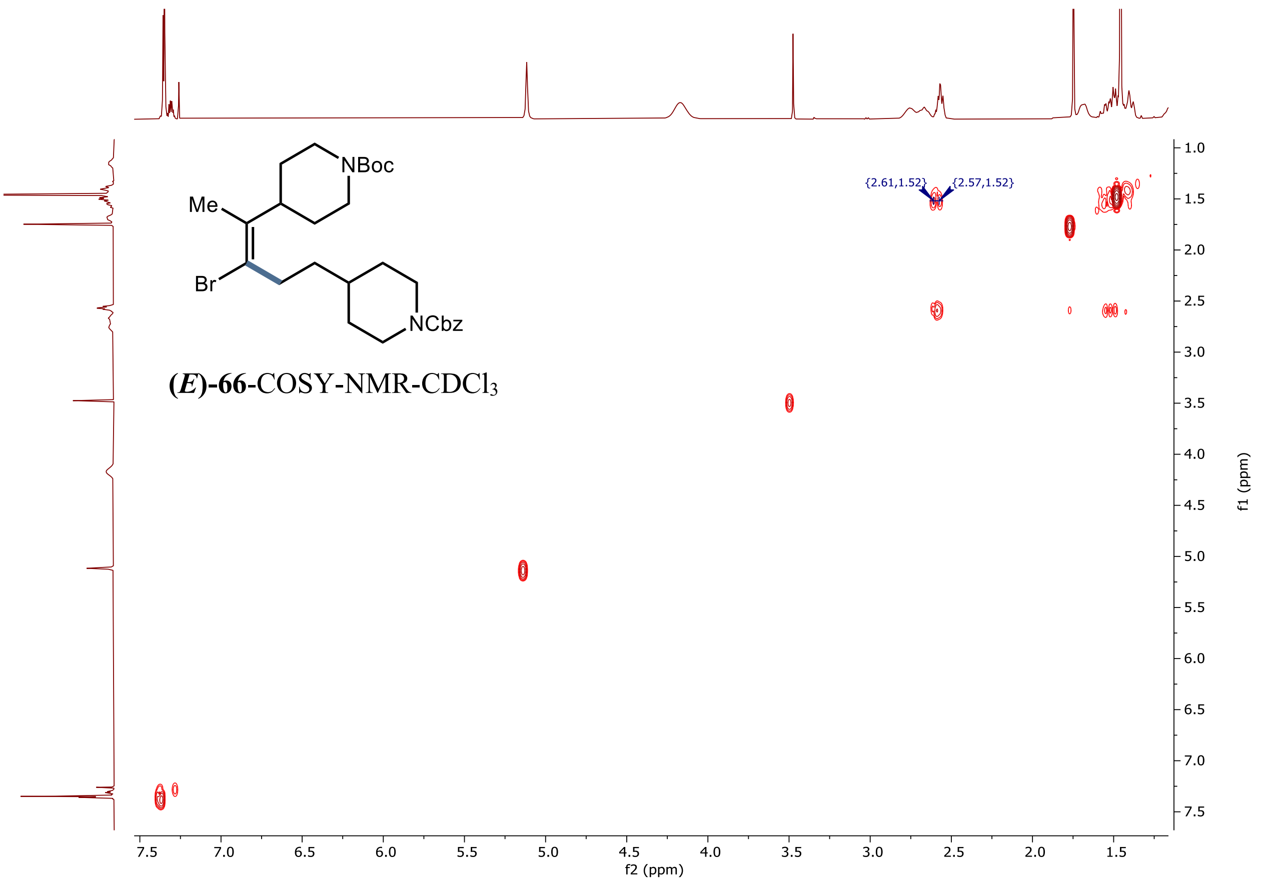
NBoc
Me
Br
NCbz
(E)-66-COSY-NMR-CDCl3
{2.61,1.52}
{2.57,1.52}
f2 (ppm)
f1 (ppm)

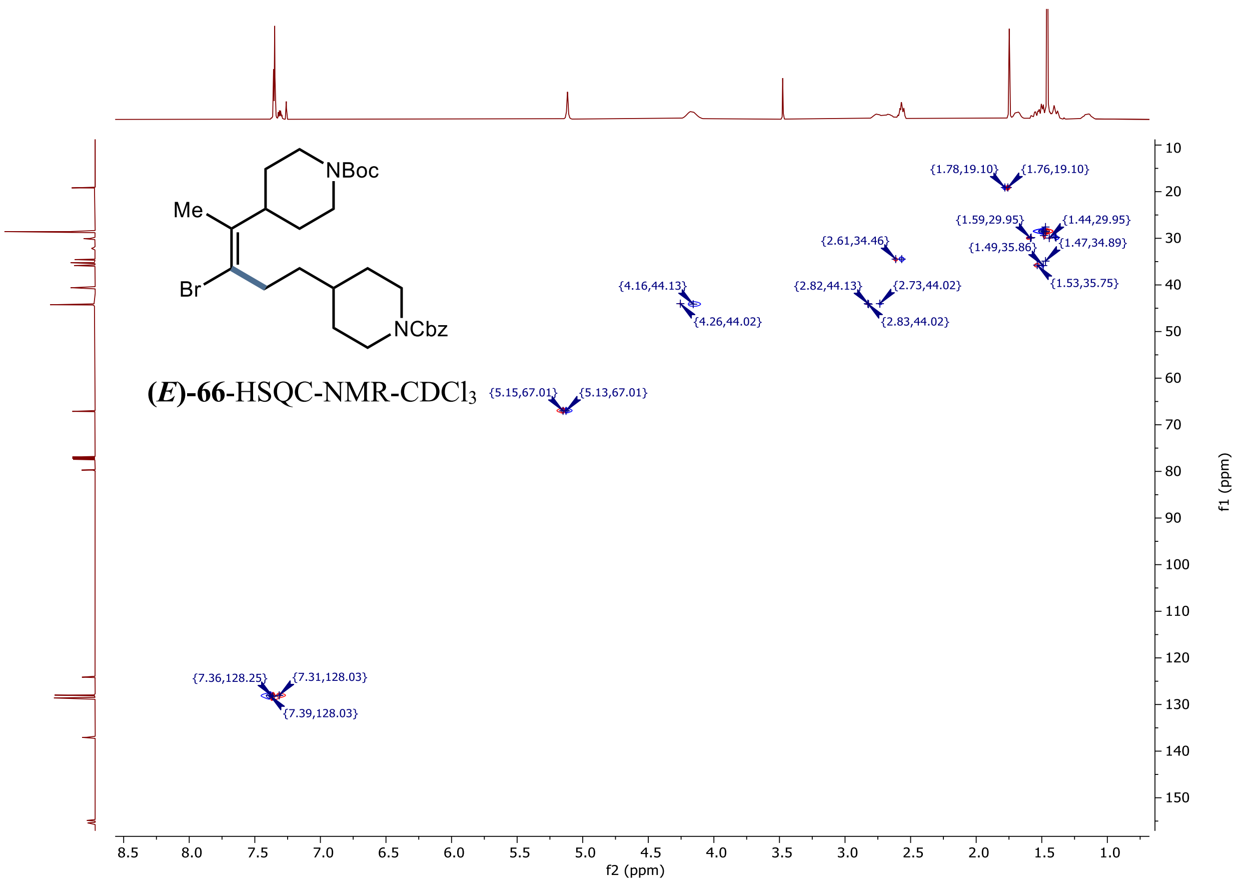
NBoc
Me
Br
NCbz
(E)-66-HSQC-NMR-CDCl3
f2 (ppm)
f1 (ppm)

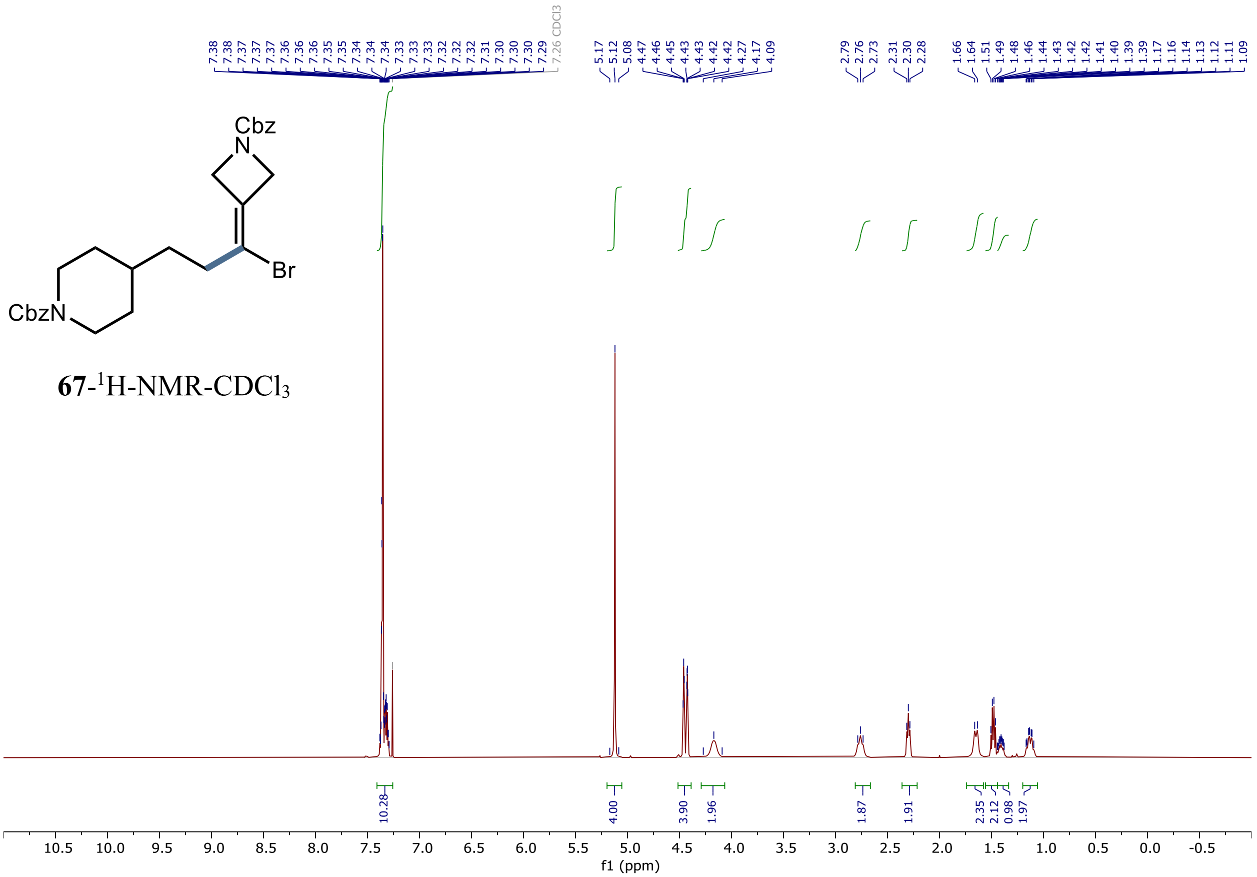
Cbz
N
Br
CbzN
67-1H-NMR-CDCl3
f1 (ppm)
10.28
4.00
3.90
1.96
1.87
1.91
2.35
2.12
0.98
1.97

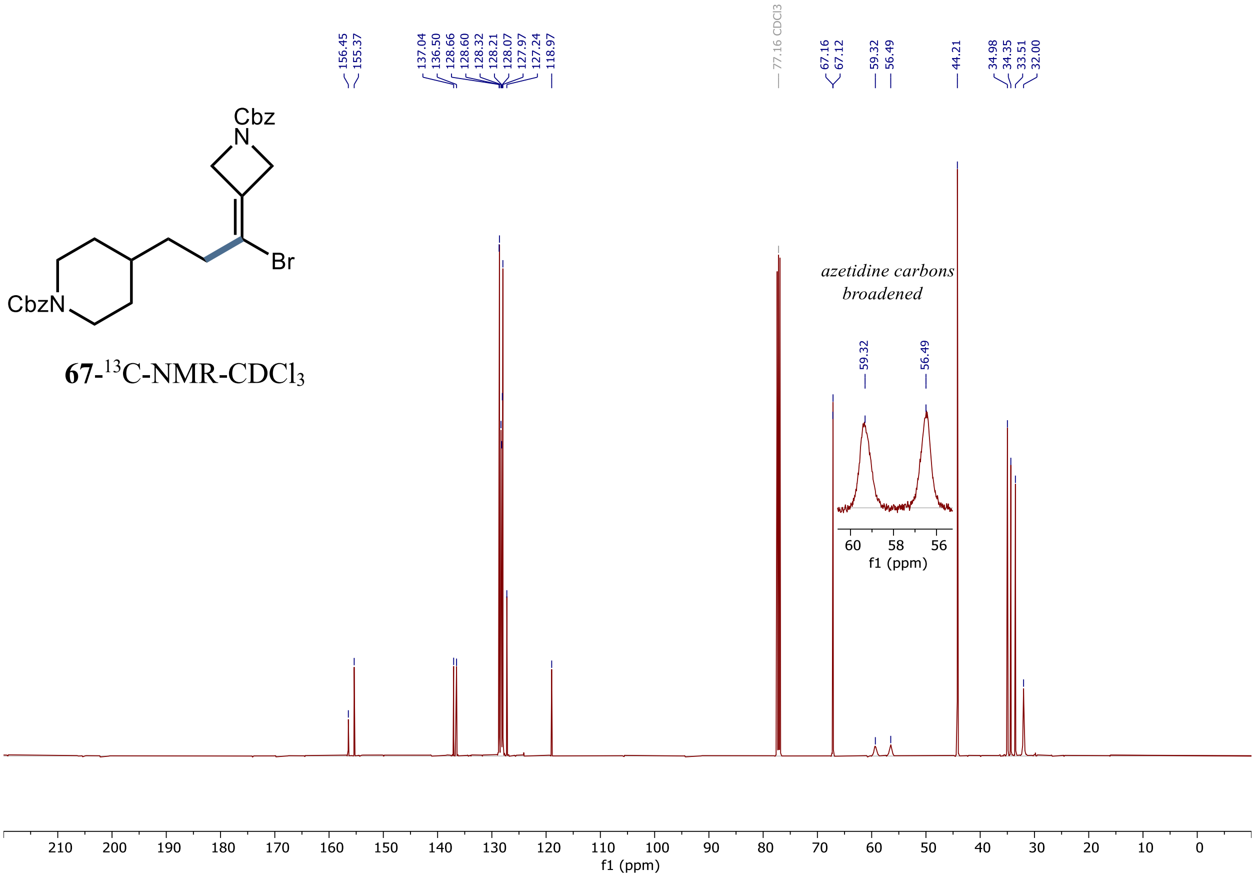

67-^{13}C-NMR-$CDCl_3$

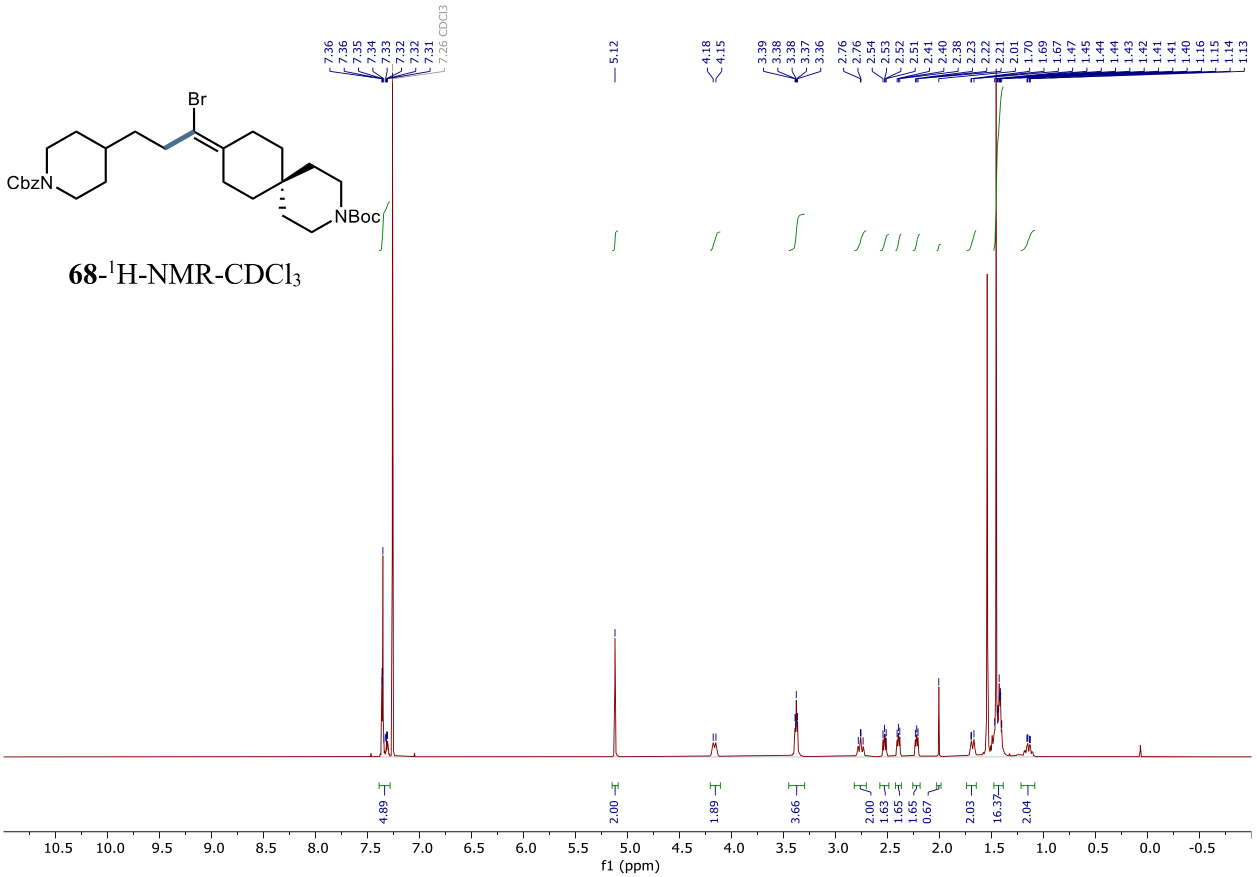

68-^{1}H-NMR-$CDCl_3$

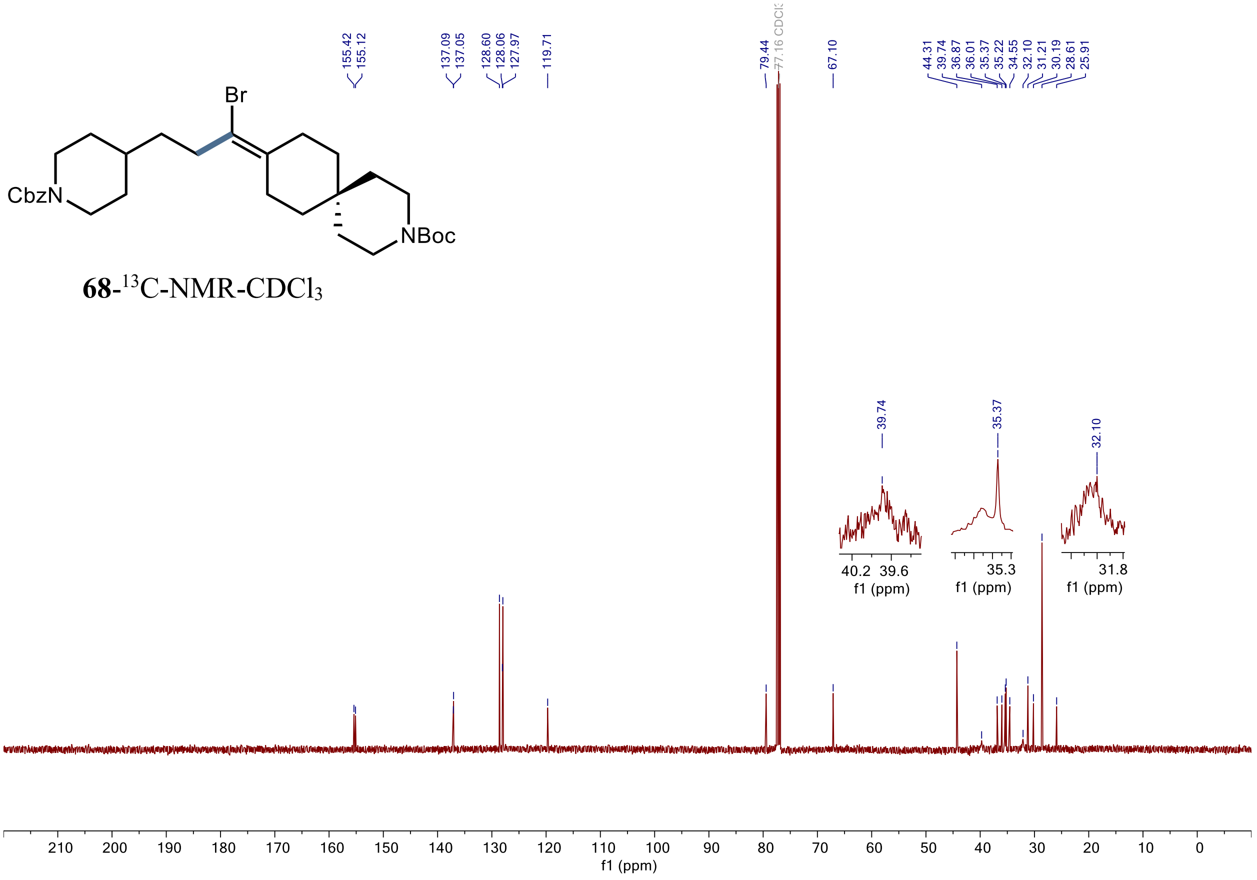

68-^{13}C-NMR-$CDCl_3$

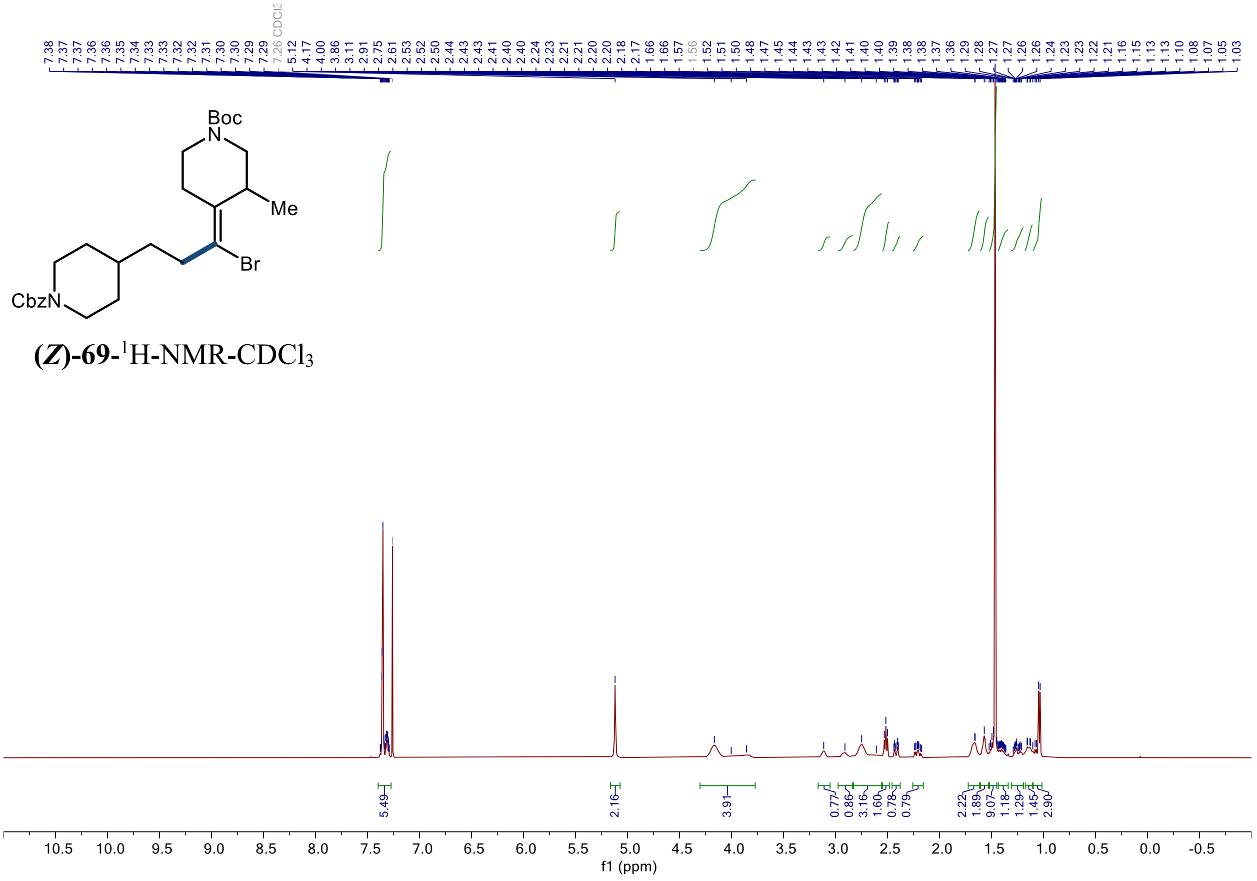

(*Z*)-69-^{1}H-NMR-$CDCl_3$

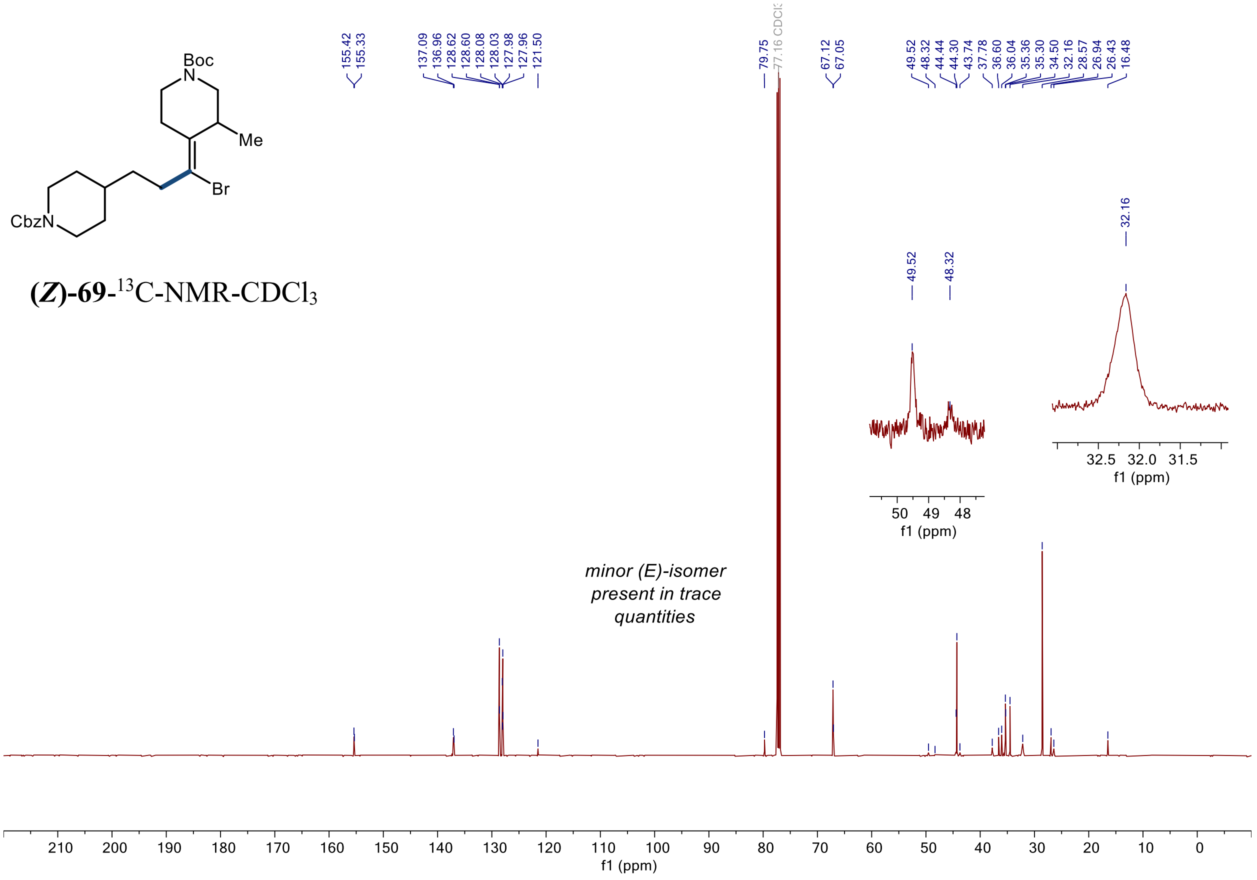

(***Z***)**-69**-^{13}C-NMR-CDCl$_3$

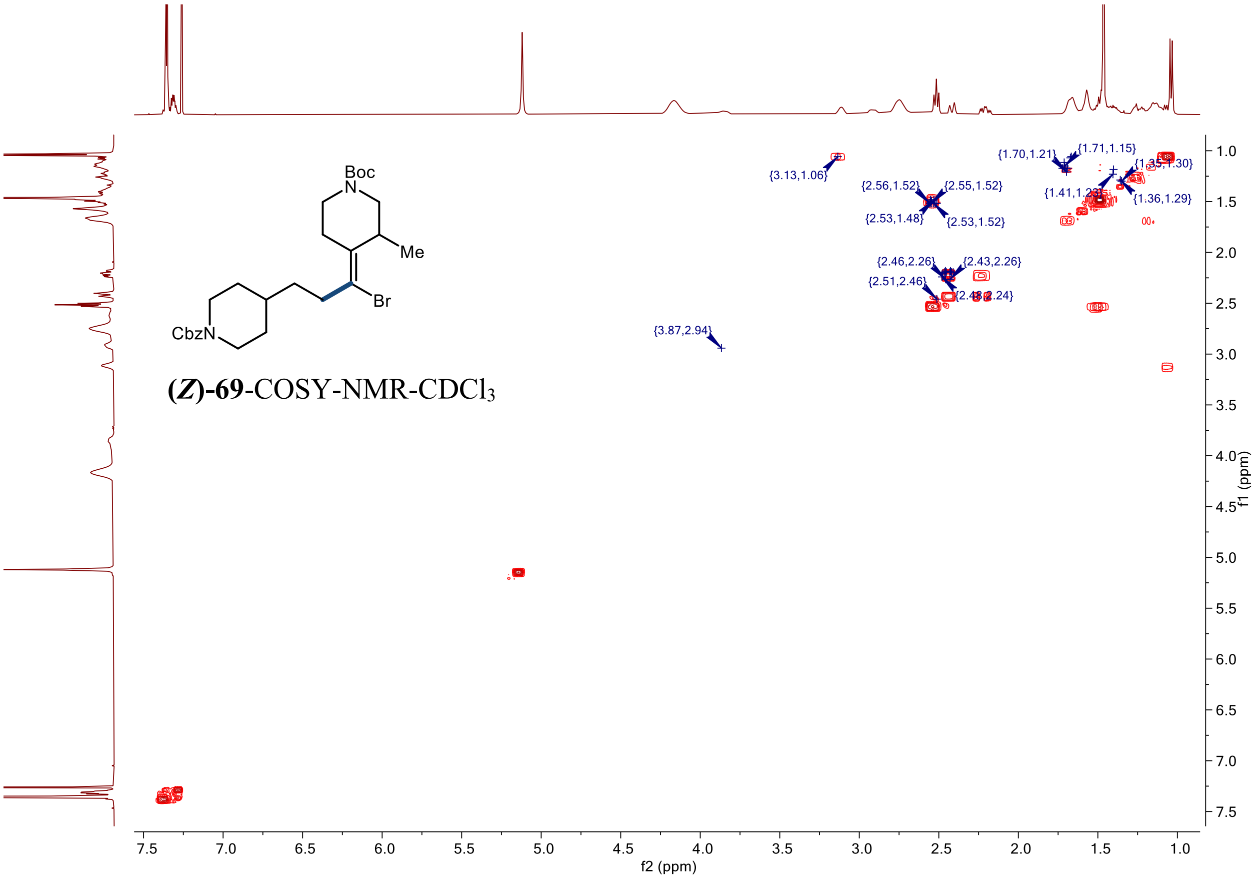

(***Z***)**-69**-COSY-NMR-CDCl$_3$

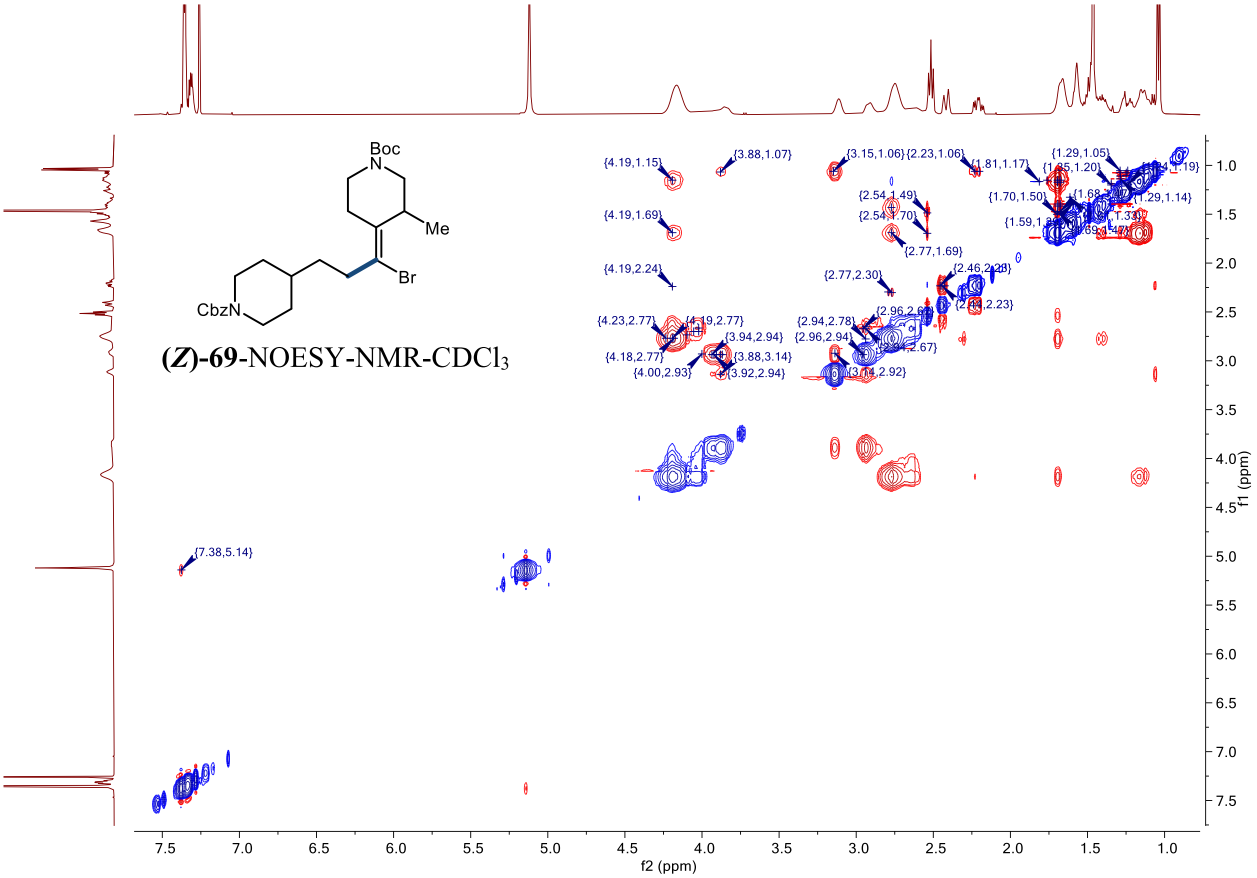
Boc
Me
Br
CbzN
(Z)-69-NOESY-NMR-CDCl3
f2 (ppm)
f1 (ppm)

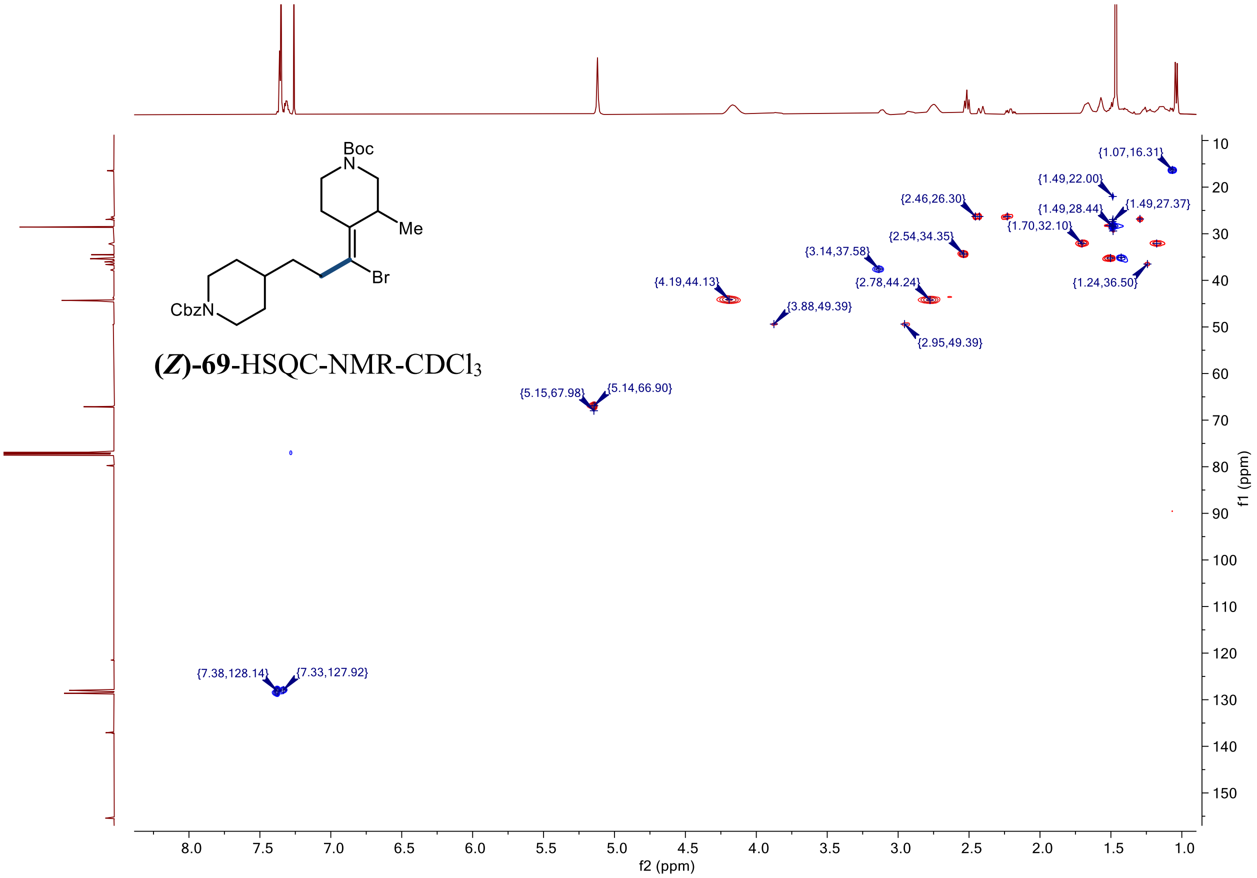
Boc
Me
Br
CbzN
(Z)-69-HSQC-NMR-CDCl3
f2 (ppm)
f1 (ppm)

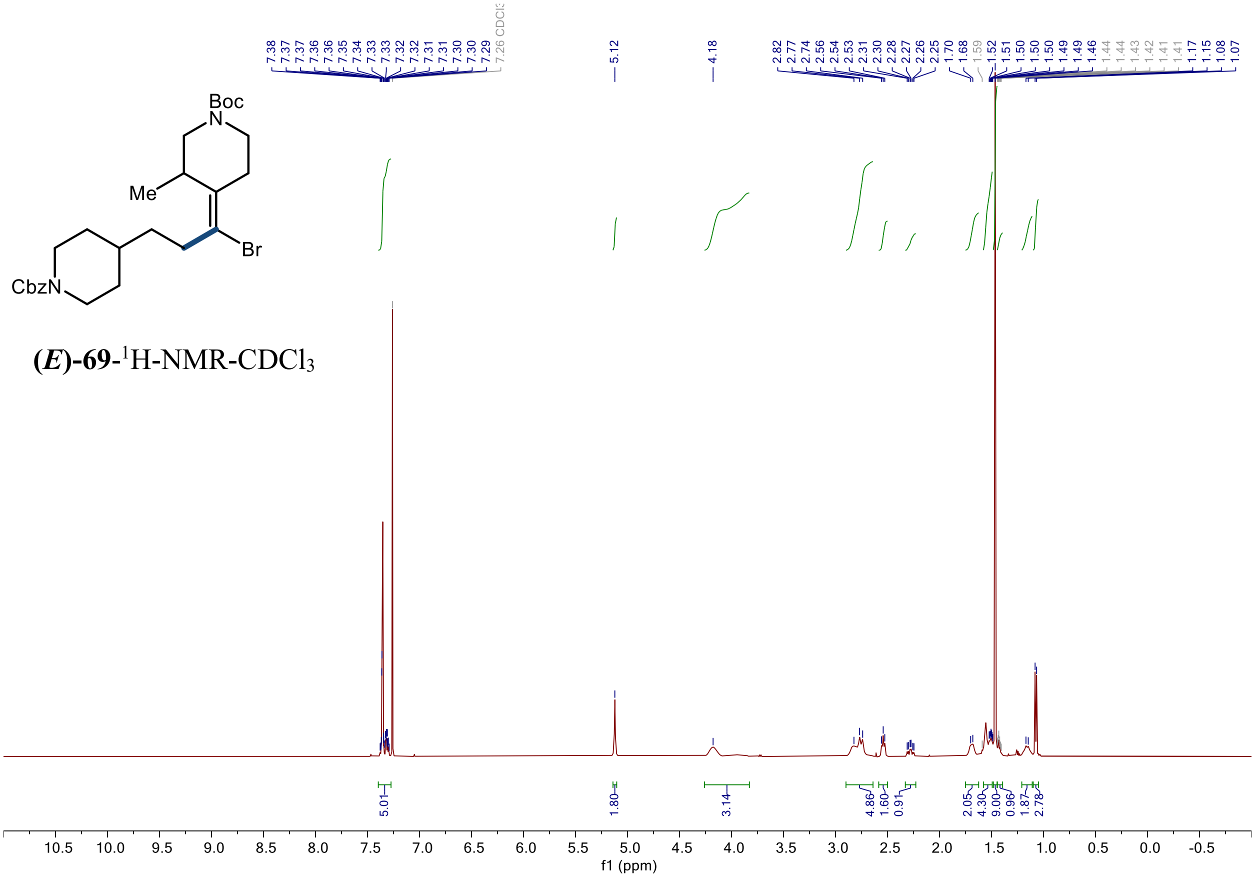

(*E*)-69-^{1}H-NMR-$CDCl_3$

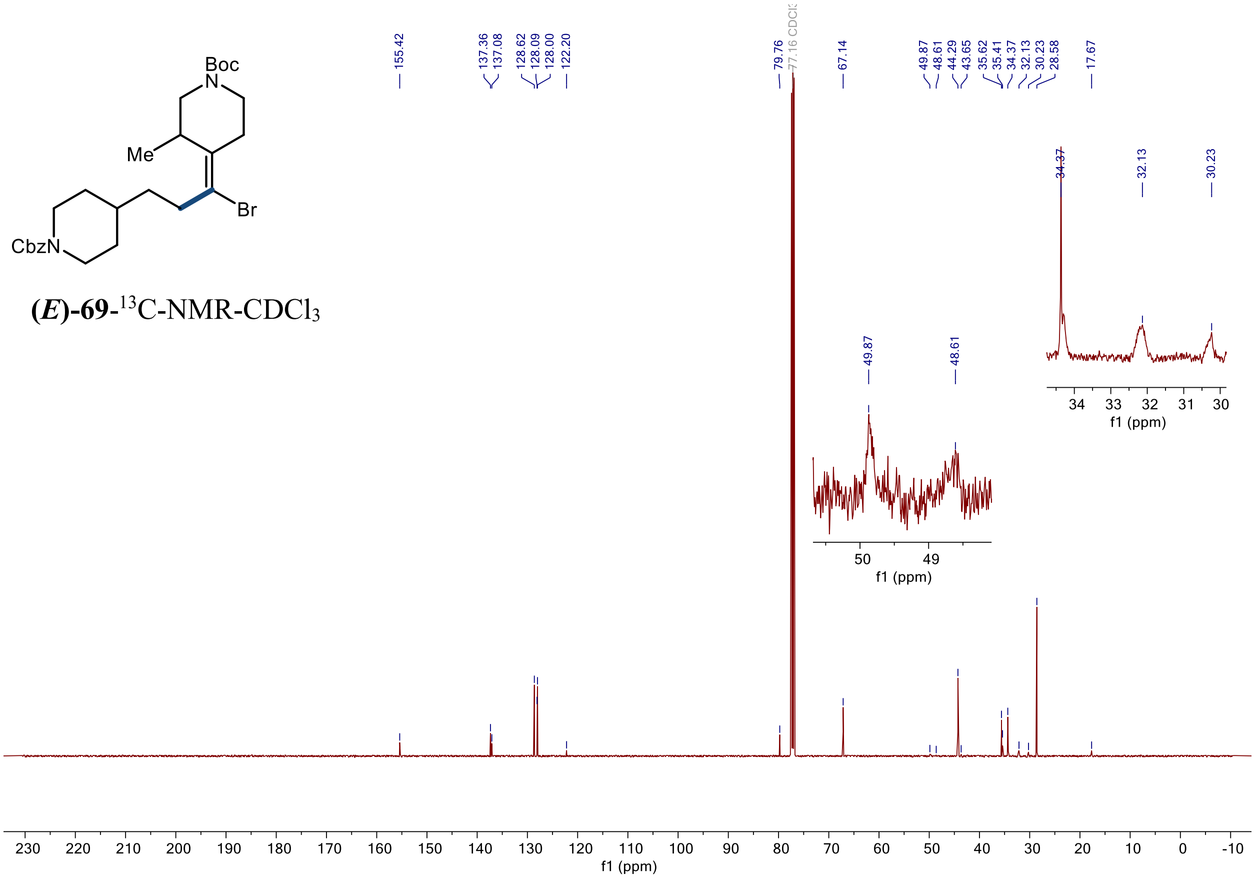

(*E*)-69-^{13}C-NMR-$CDCl_3$

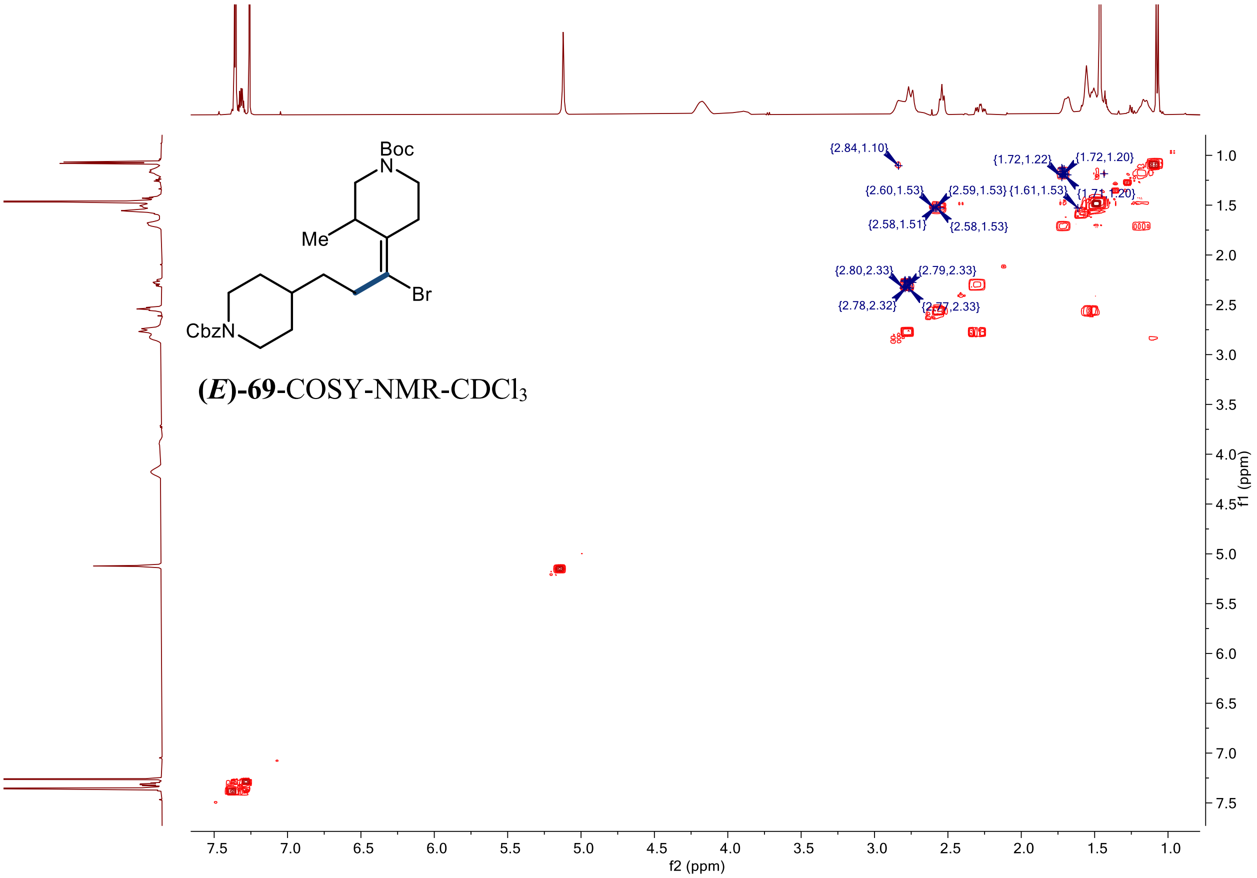

(*E*)-**69**-COSY-NMR-$CDCl_3$

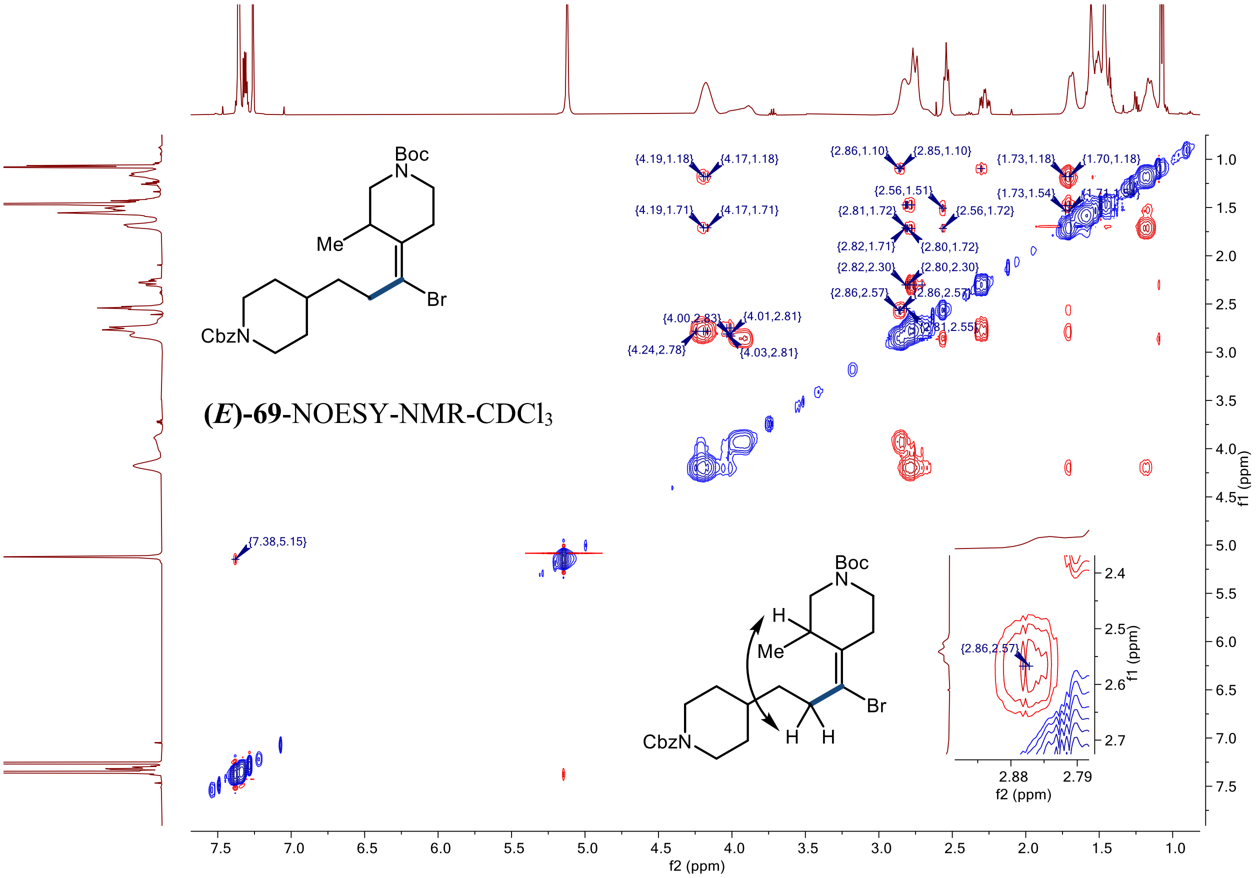

(*E*)-**69**-NOESY-NMR-$CDCl_3$

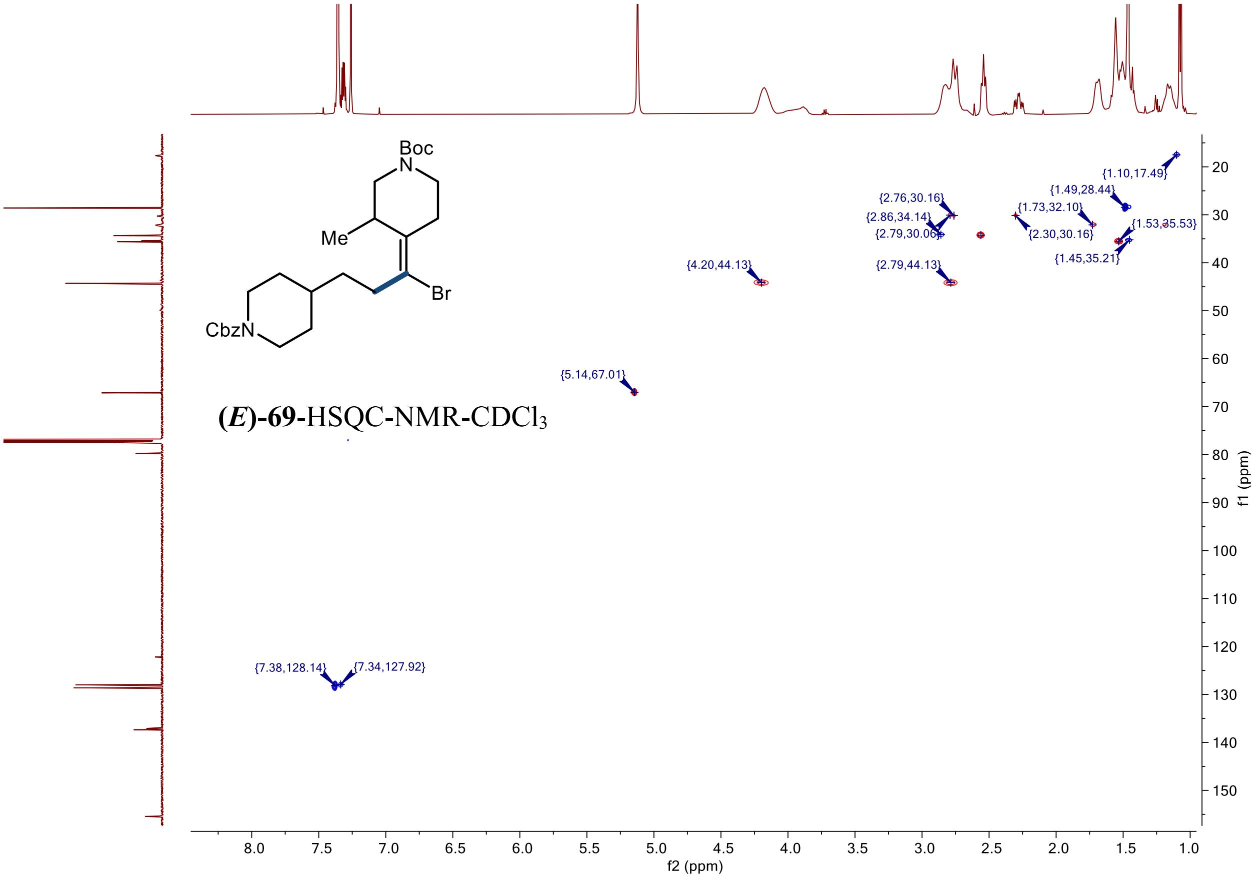

Boc
N
Me
Br
CbzN
(E)-69-HSQC-NMR-CDCl3
{1.10,17.49}
{1.49,28.44}
{2.76,30.16}
{1.73,32.10}
{2.86,34.14}
{1.53,35.53}
{2.79,30.06}
{2.30,30.16}
{1.45,35.21}
{4.20,44.13}
{2.79,44.13}
{5.14,67.01}
{7.38,128.14}
{7.34,127.92}
f2 (ppm)
f1 (ppm)

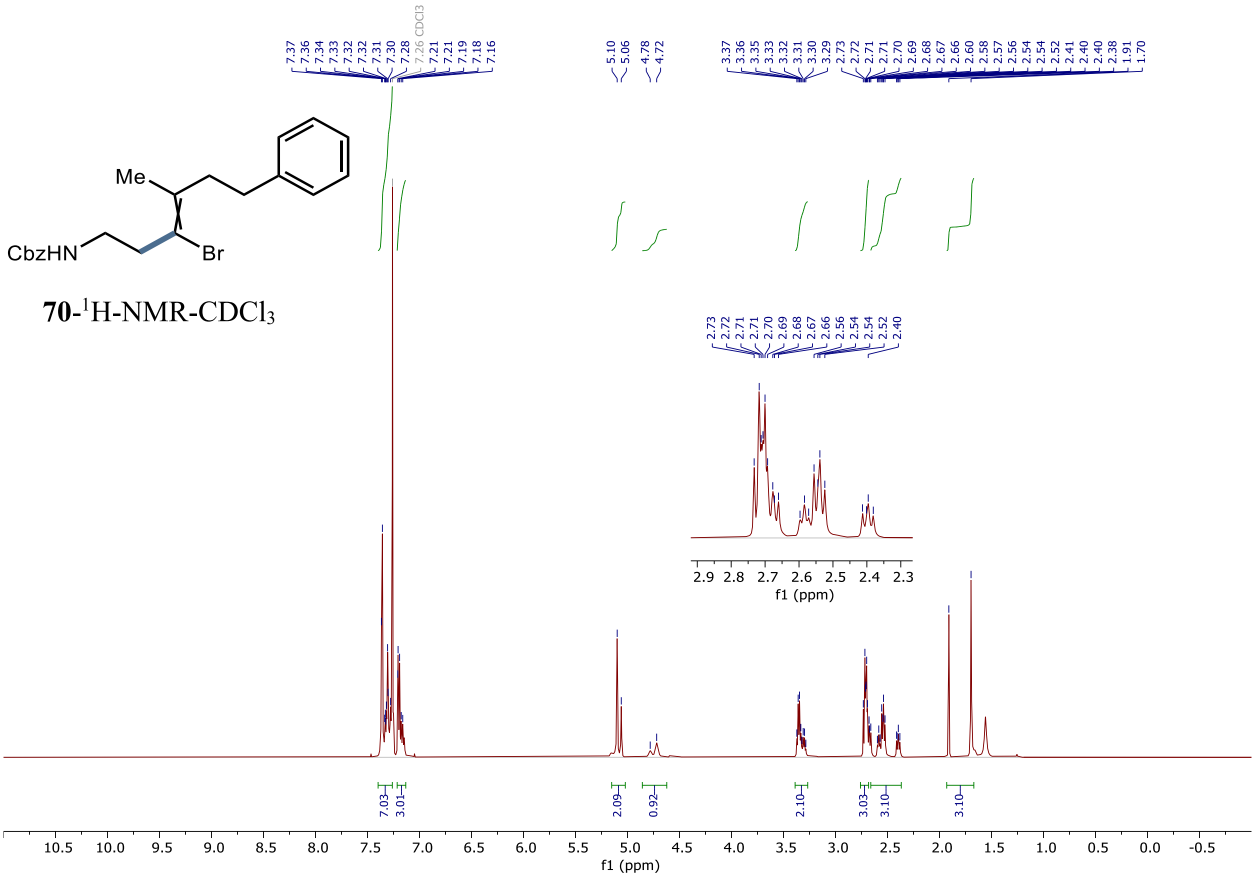

Me
CbzHN
Br
70-1H-NMR-CDCl3
7.37 7.36 7.34 7.33 7.32 7.32 7.31 7.30 7.28 7.26 CDCl3 7.21 7.21 7.21 7.19 7.18 7.16
5.10 5.06 4.78 4.72
3.37 3.36 3.35 3.33 3.32 3.31 3.30 3.29 2.73 2.72 2.71 2.71 2.70 2.69 2.68 2.67 2.66 2.60 2.58 2.57 2.56 2.54 2.54 2.52 2.41 2.40 2.40 2.38 1.91 1.70
7.03 3.01 2.09 0.92 2.10 3.03 3.10 3.10
f1 (ppm)

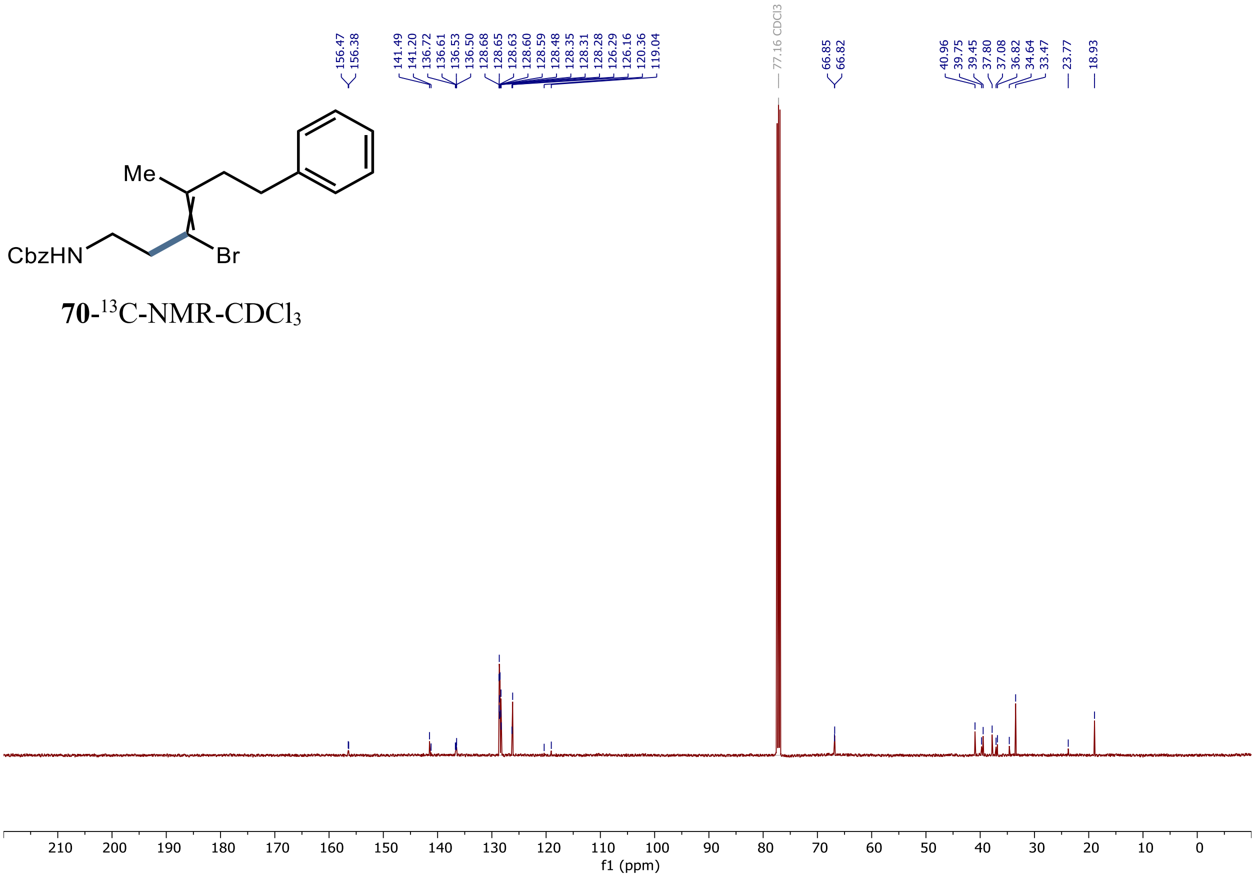

70-^{13}C-NMR-CDCl$_3$

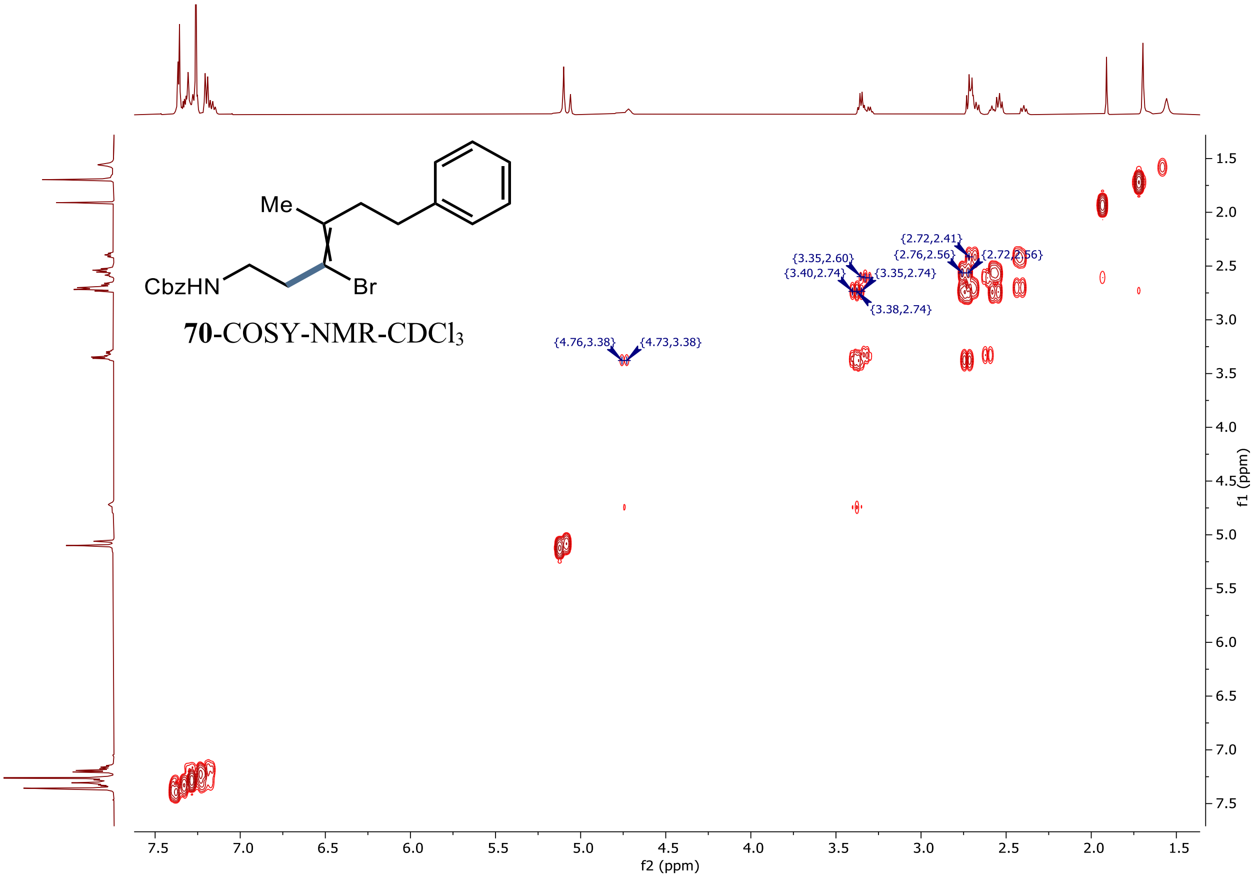

70-COSY-NMR-CDCl$_3$

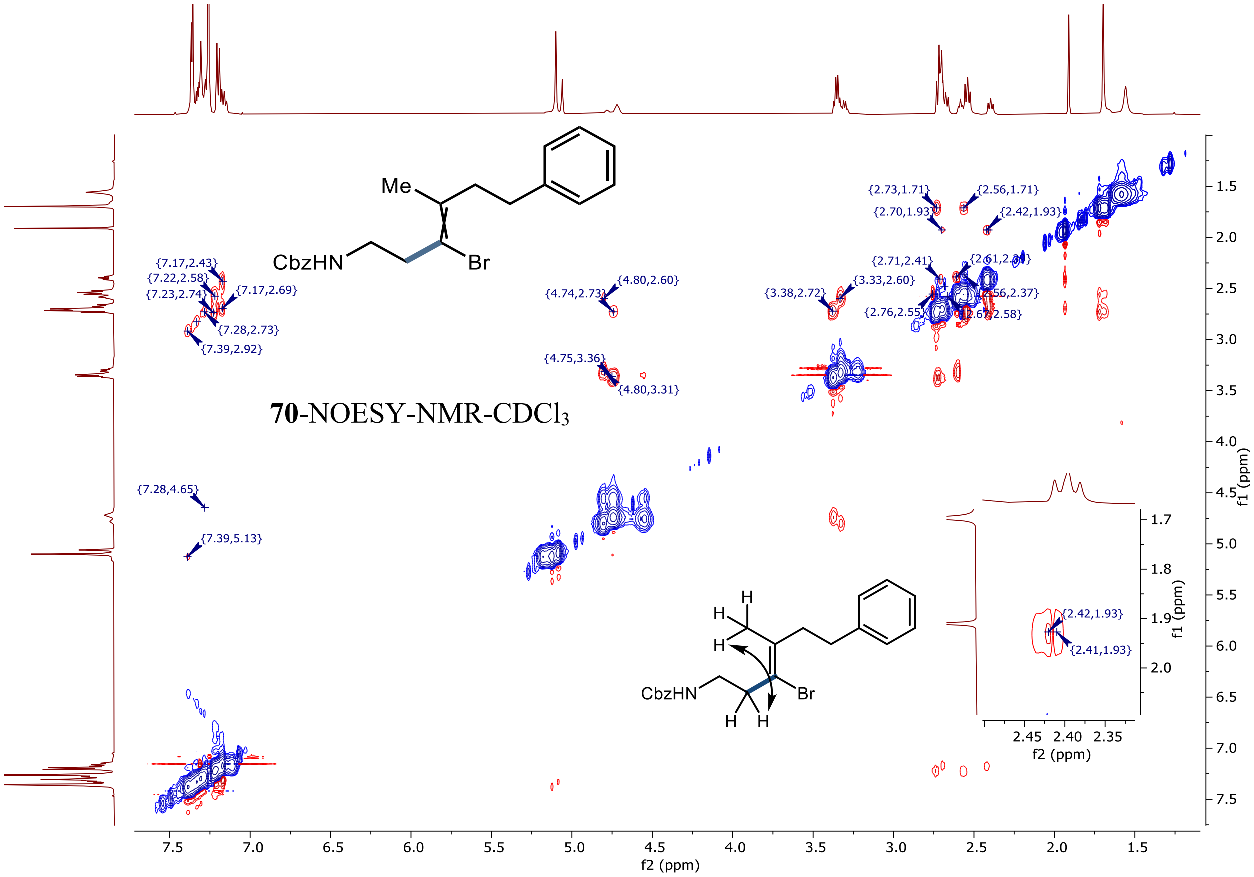

Me
CbzHN
Br
70-NOESY-NMR-CDCl3
f2 (ppm)
f1 (ppm)

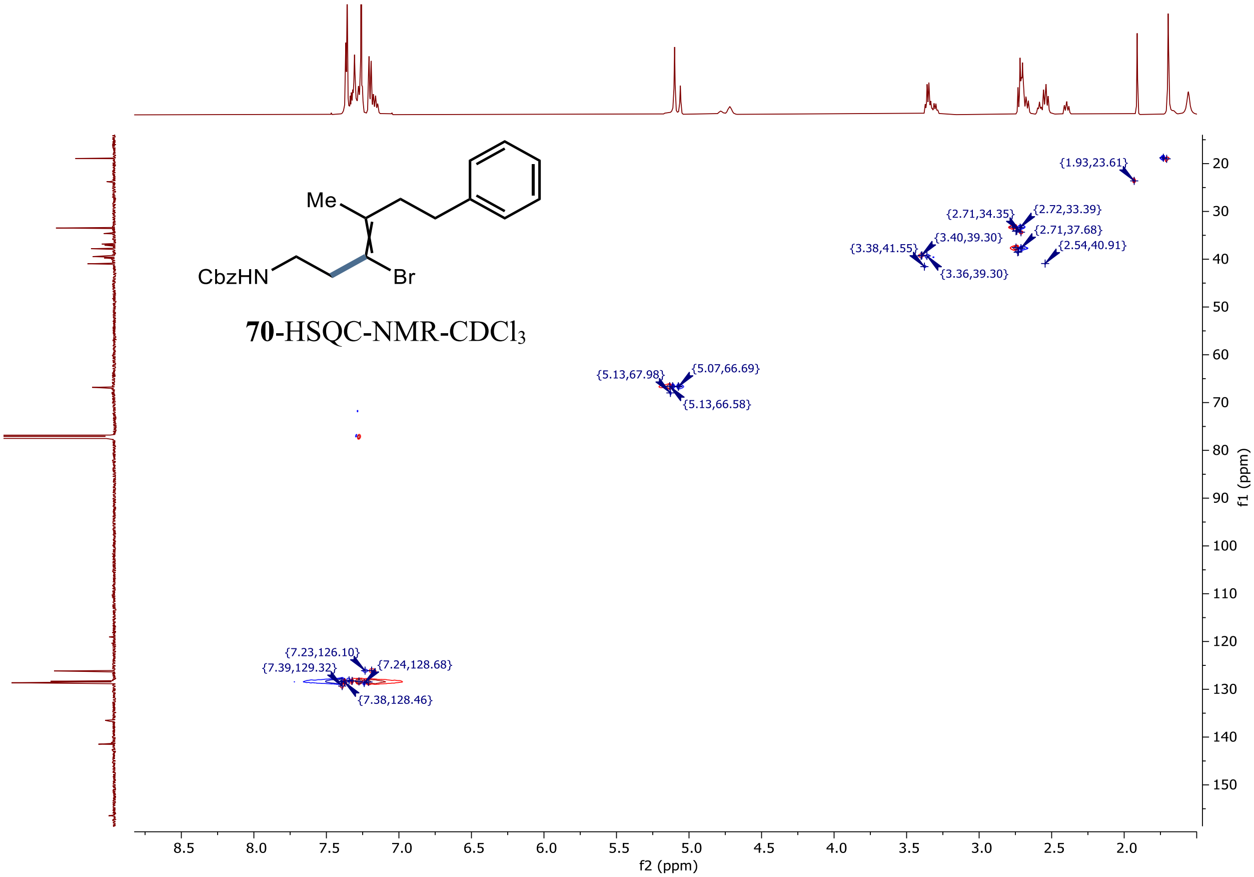

Me
CbzHN
Br
70-HSQC-NMR-CDCl3
f2 (ppm)
f1 (ppm)

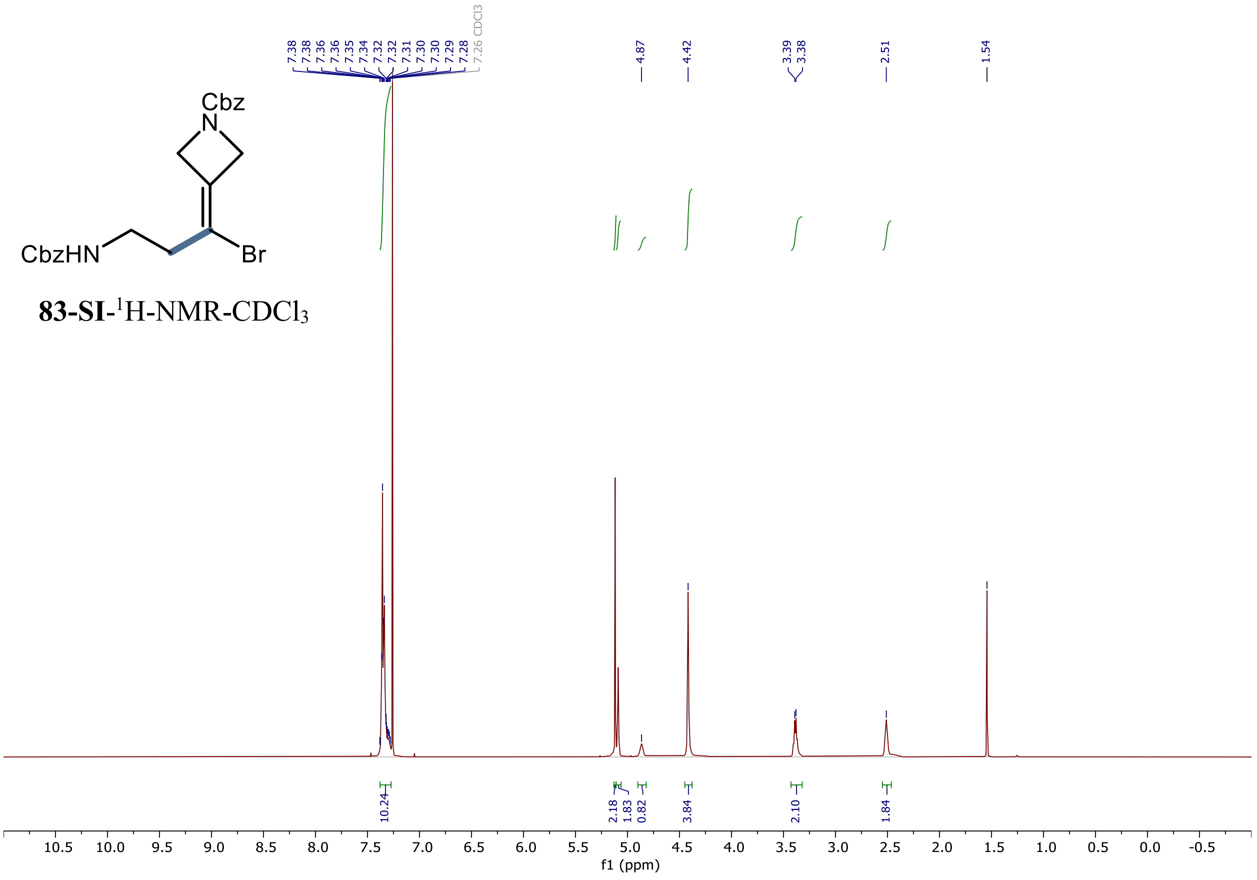

83-SI-^{1}H-NMR-$CDCl_3$

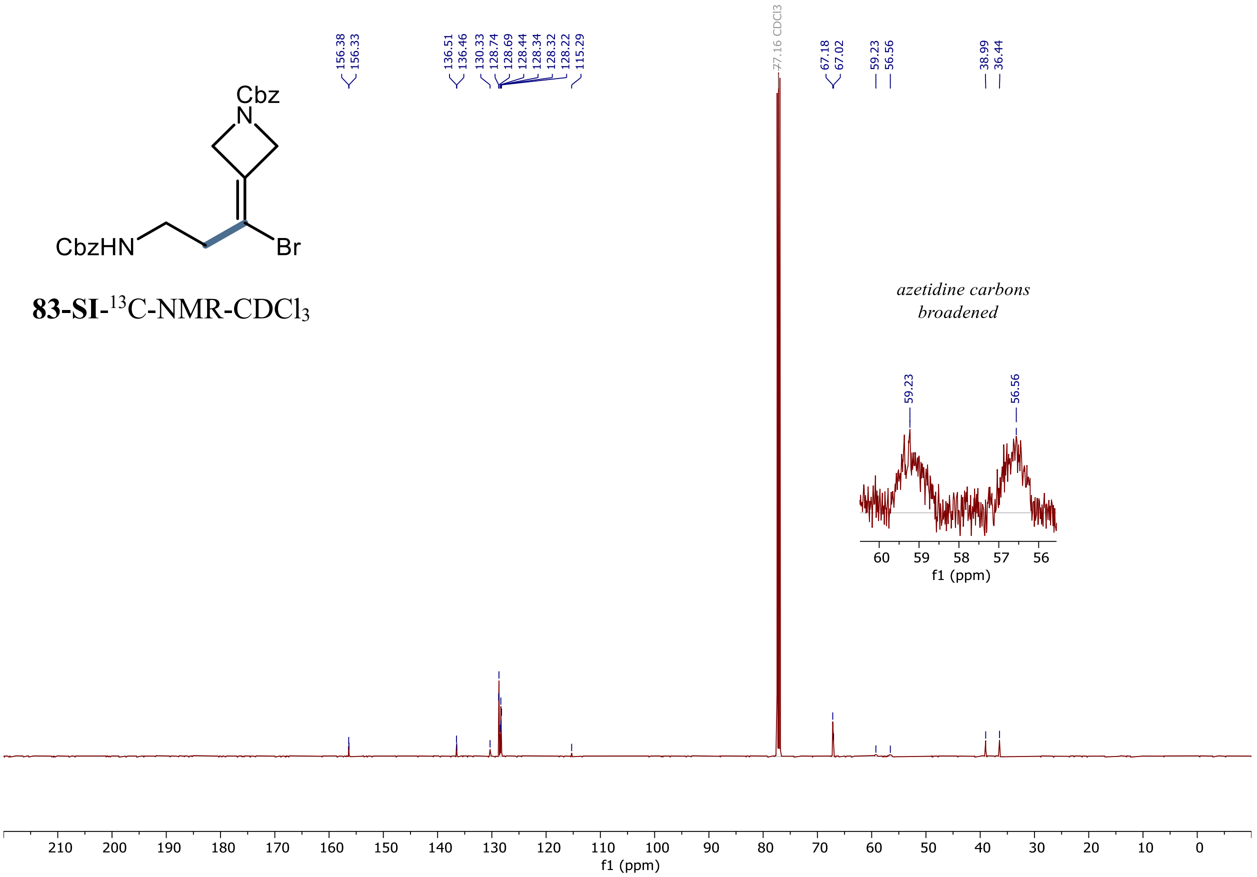

83-SI-^{13}C-NMR-$CDCl_3$

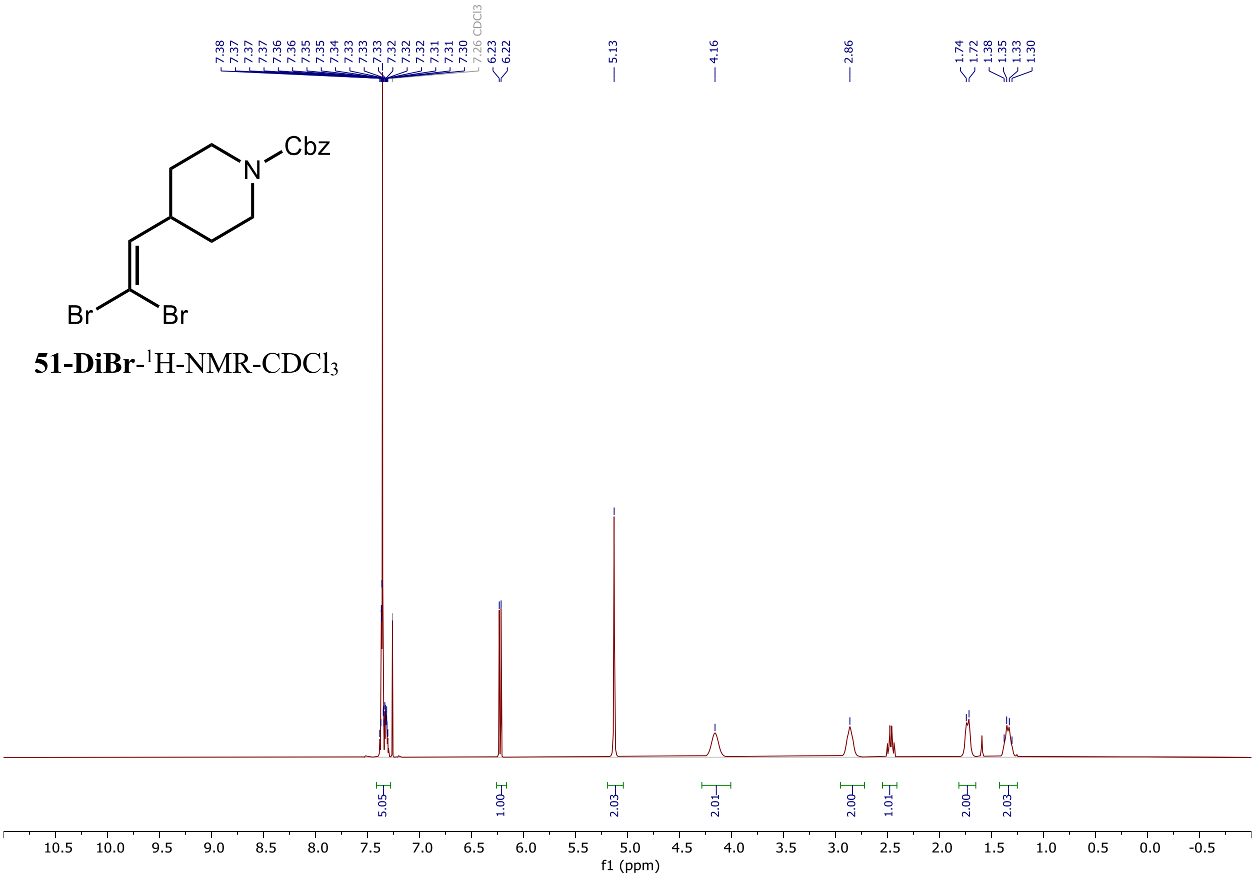

51-DiBr-^{1}H-NMR-$CDCl_3$

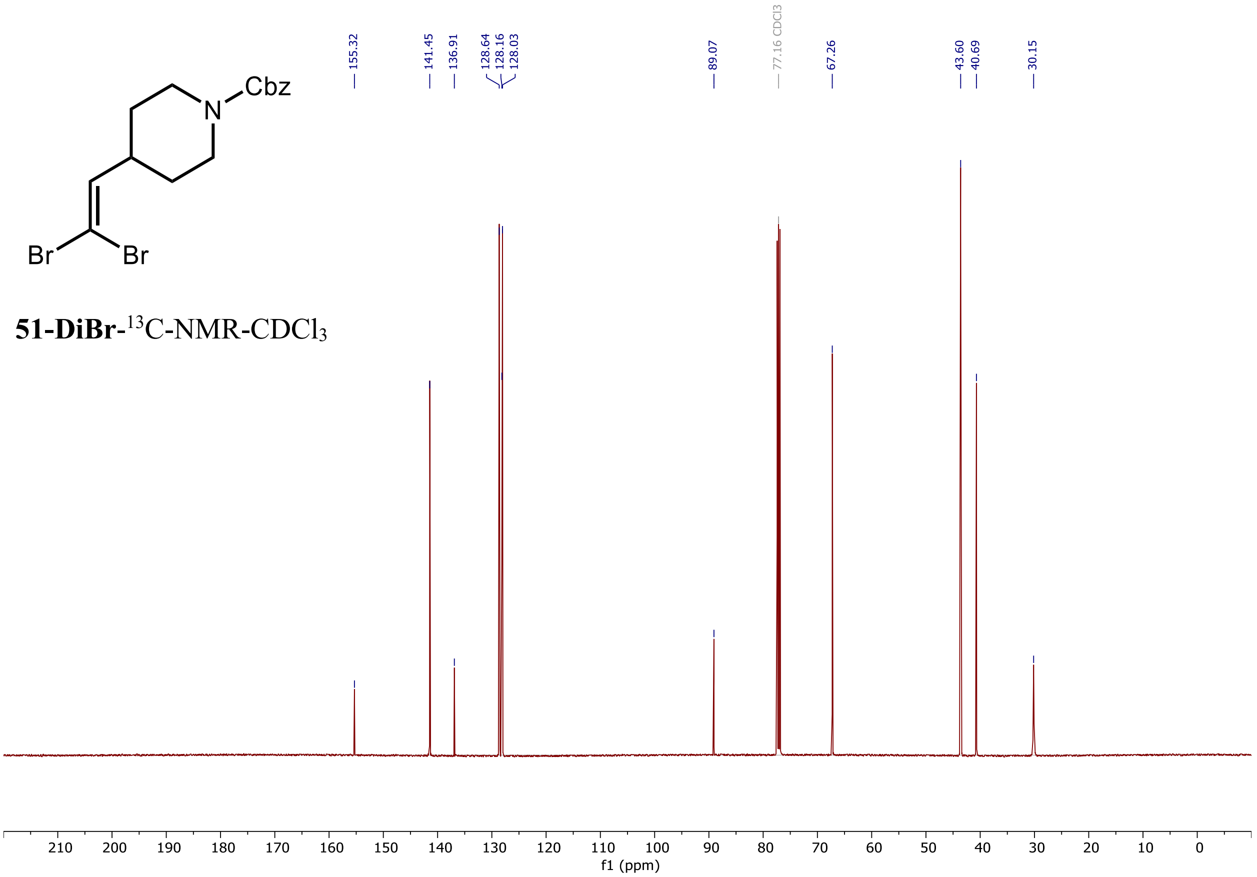

51-DiBr-^{13}C-NMR-$CDCl_3$

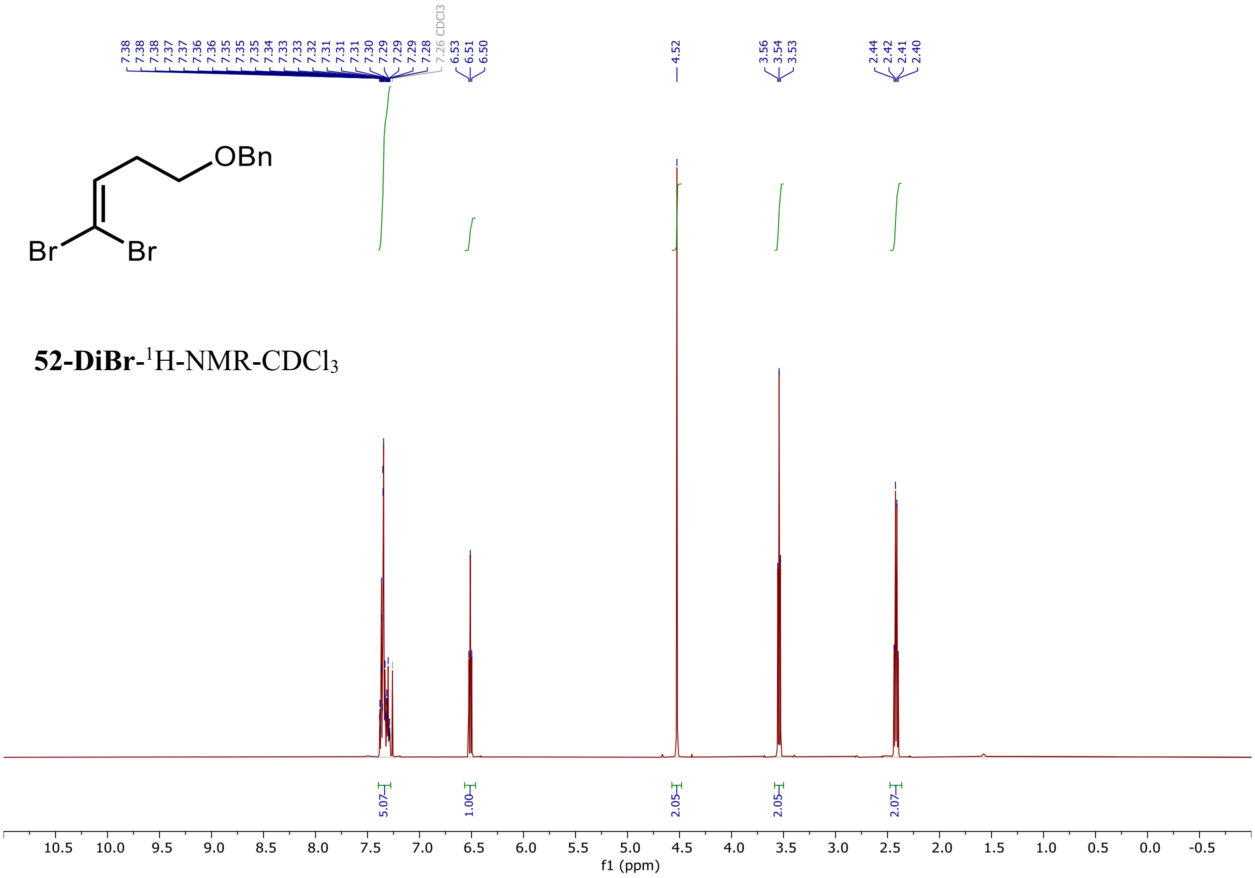

52-DiBr-^{1}H-NMR-CDCl$_3$

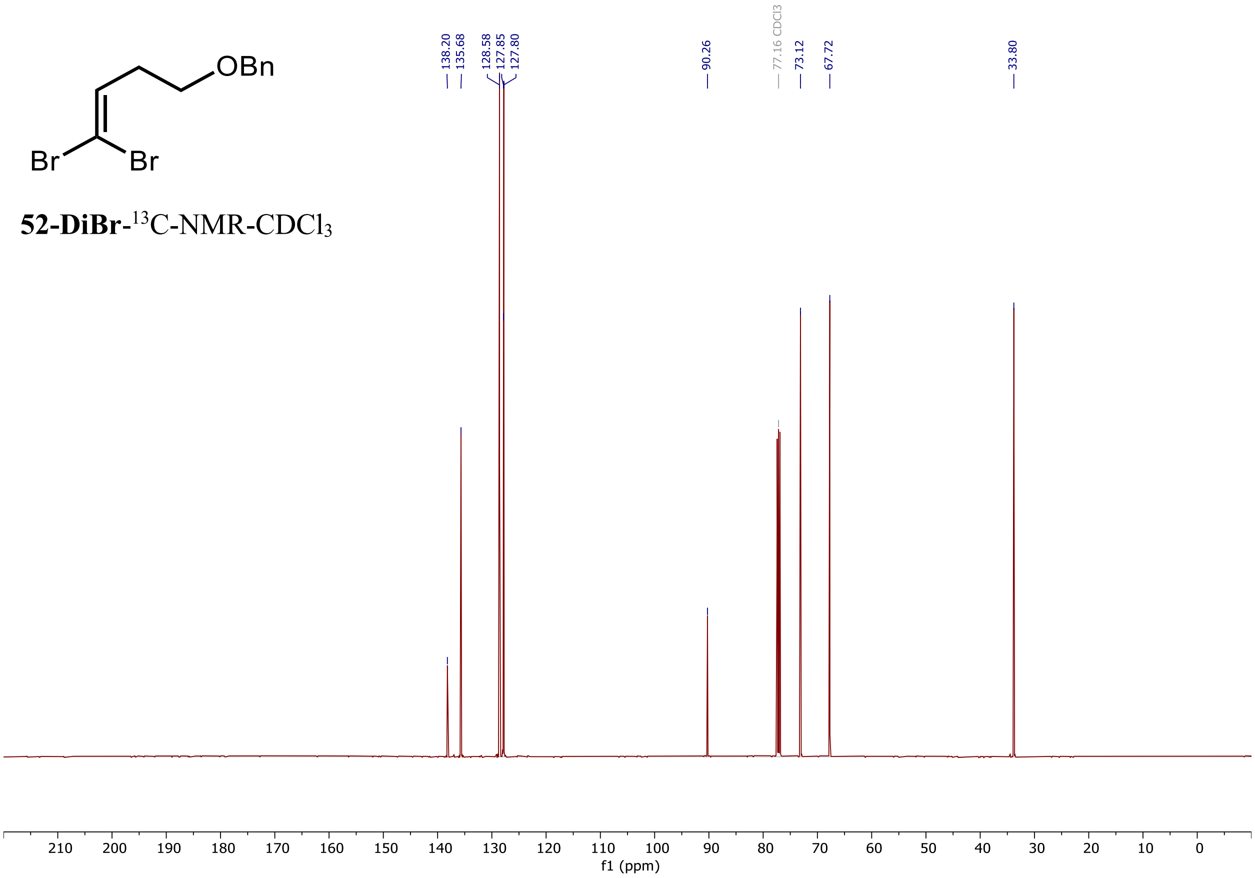

52-DiBr-^{13}C-NMR-CDCl$_3$

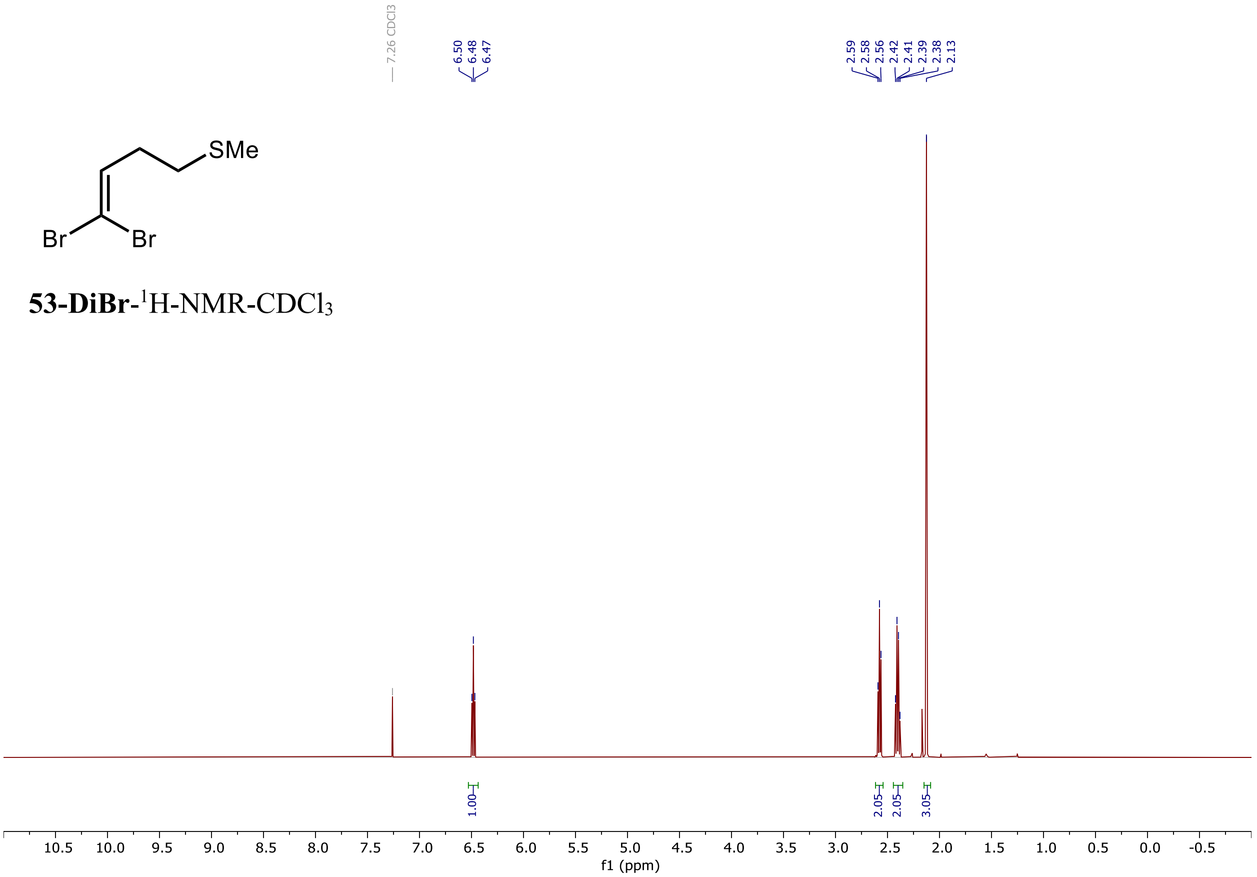
7.26 CDCl3
6.50
6.48
6.47
2.59
2.58
2.56
2.42
2.41
2.39
2.38
2.13
SMe
Br
Br
53-DiBr-1H-NMR-CDCl3
1.00
2.05
2.05
3.05
10.5 10.0 9.5 9.0 8.5 8.0 7.5 7.0 6.5 6.0 5.5 5.0 4.5 4.0 3.5 3.0 2.5 2.0 1.5 1.0 0.5 0.0 -0.5
f1 (ppm)

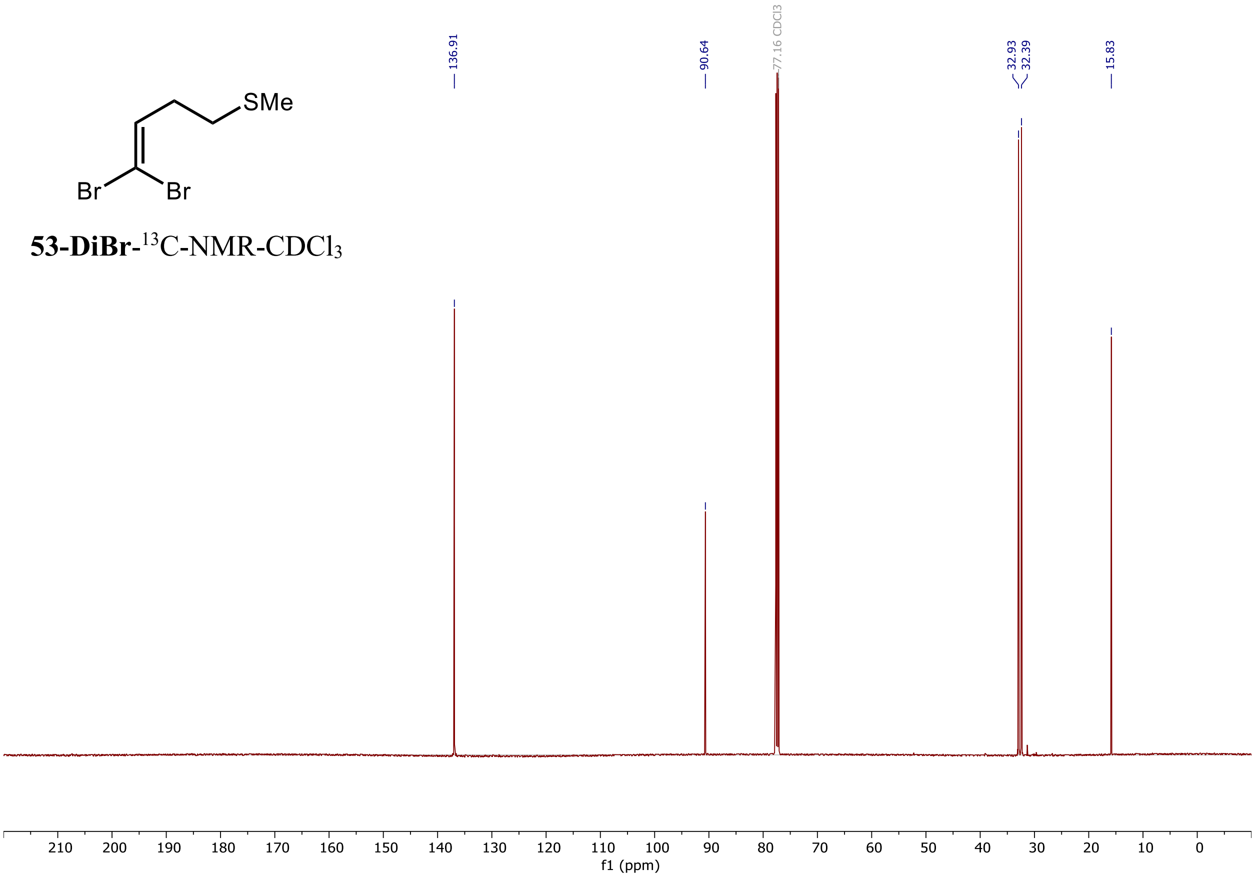
136.91
90.64
77.16 CDCl3
32.93
32.39
15.83
SMe
Br
Br
53-DiBr-13C-NMR-CDCl3
210 200 190 180 170 160 150 140 130 120 110 100 90 80 70 60 50 40 30 20 10 0
f1 (ppm)

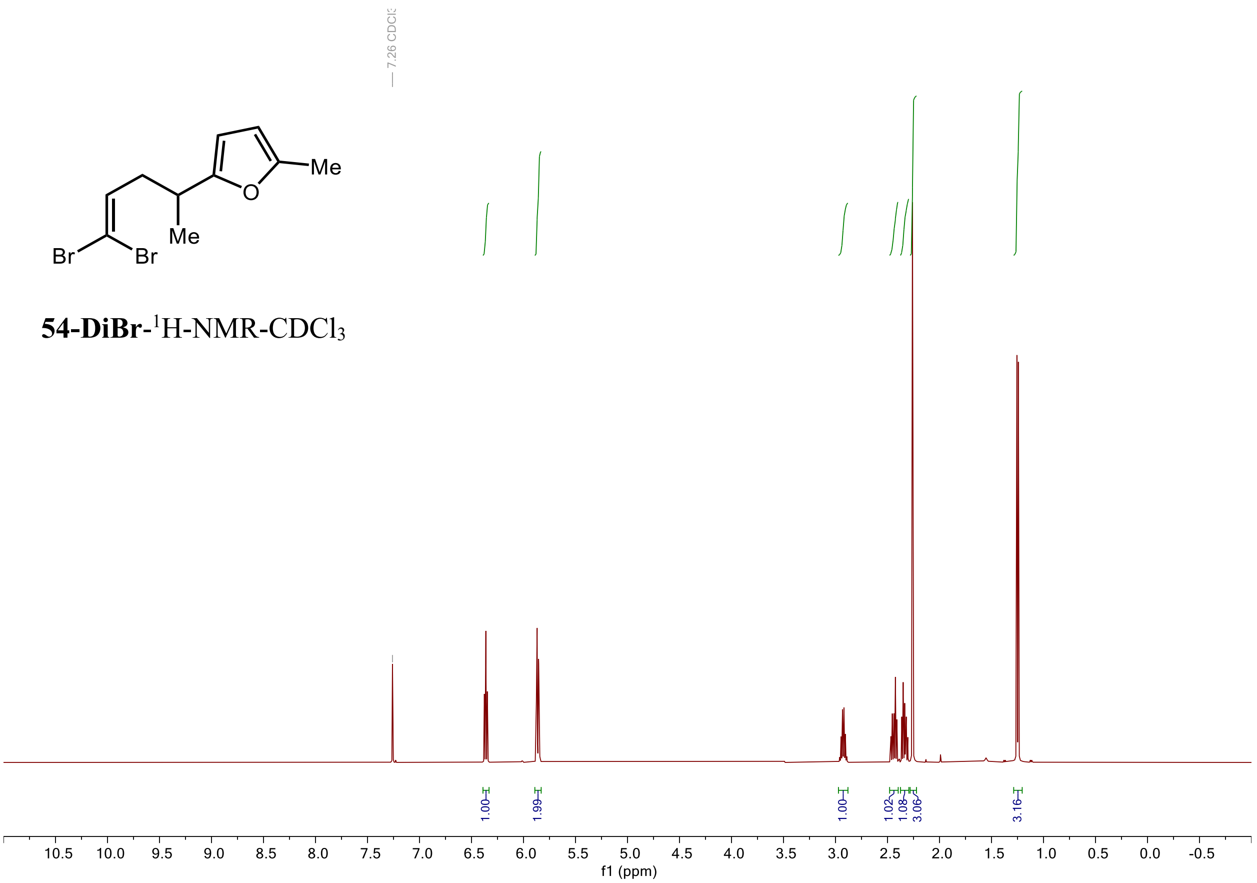

54-DiBr-^{1}H-NMR-CDCl$_3$

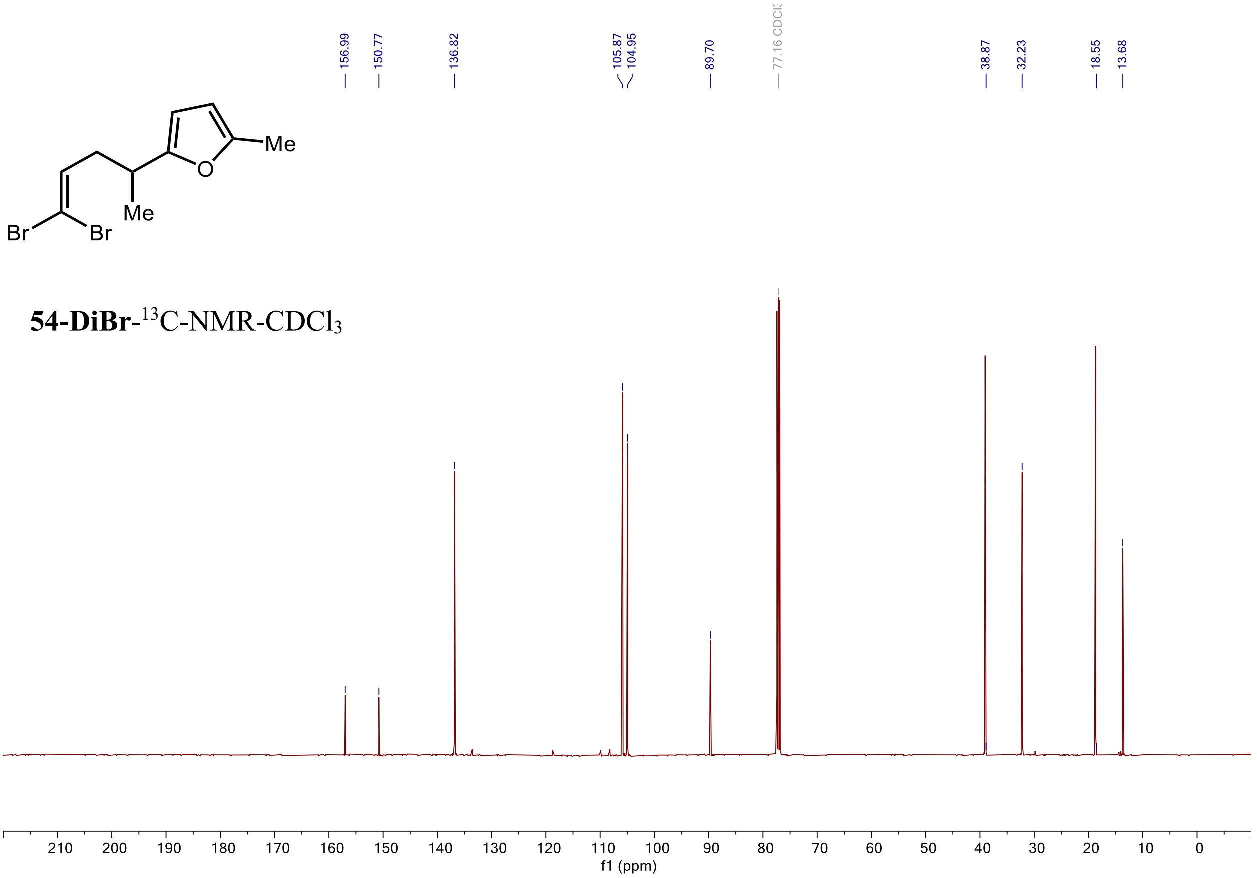

54-DiBr-^{13}C-NMR-CDCl$_3$

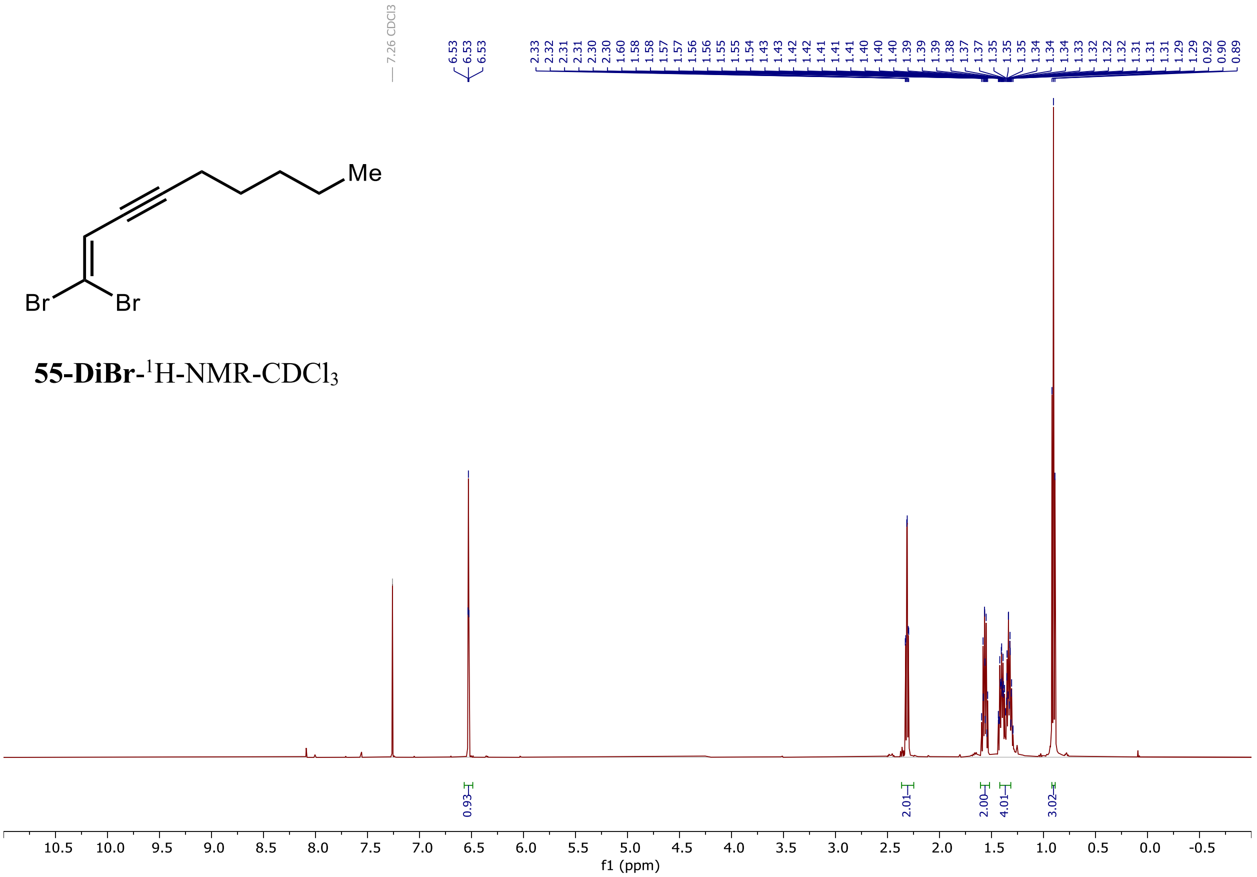

55-DiBr-^{1}H-NMR-$CDCl_3$

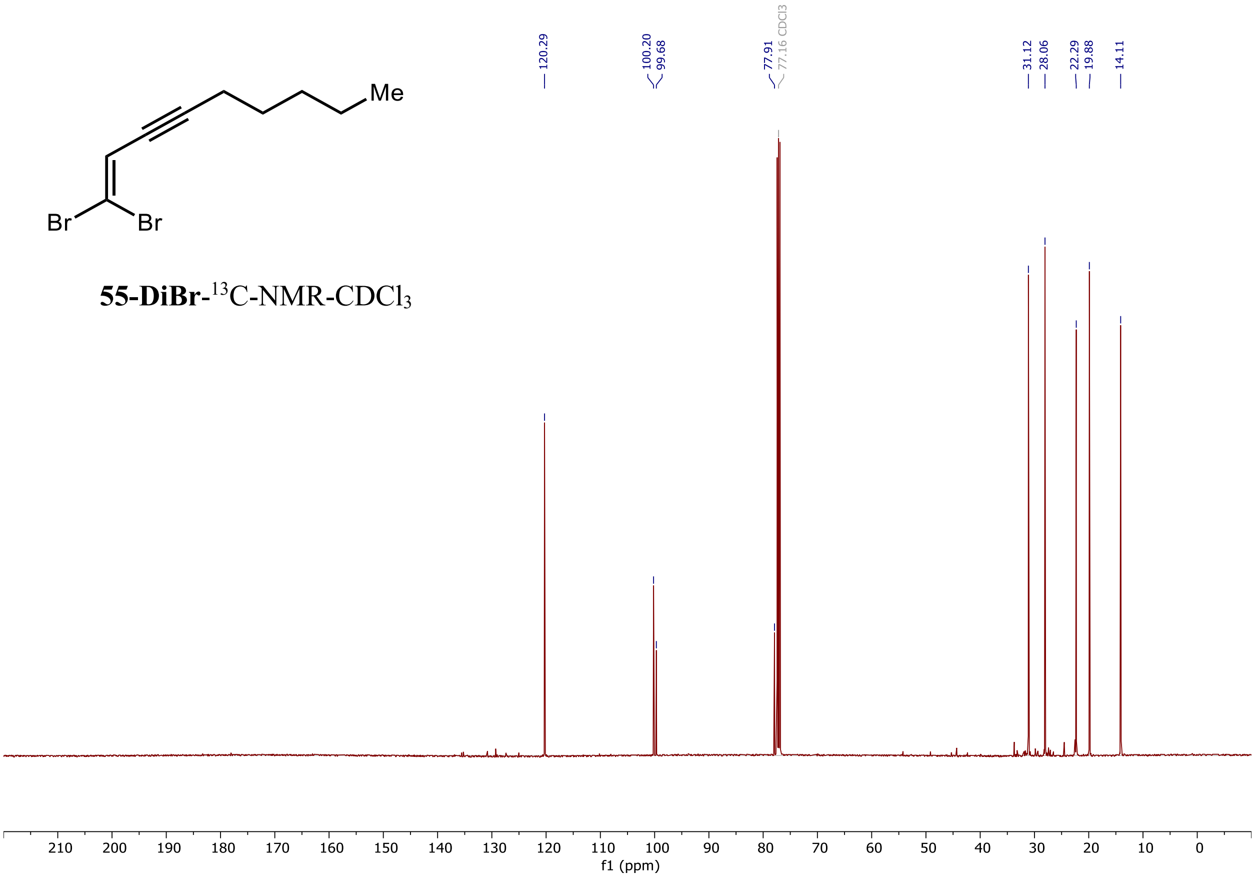

55-DiBr-^{13}C-NMR-$CDCl_3$

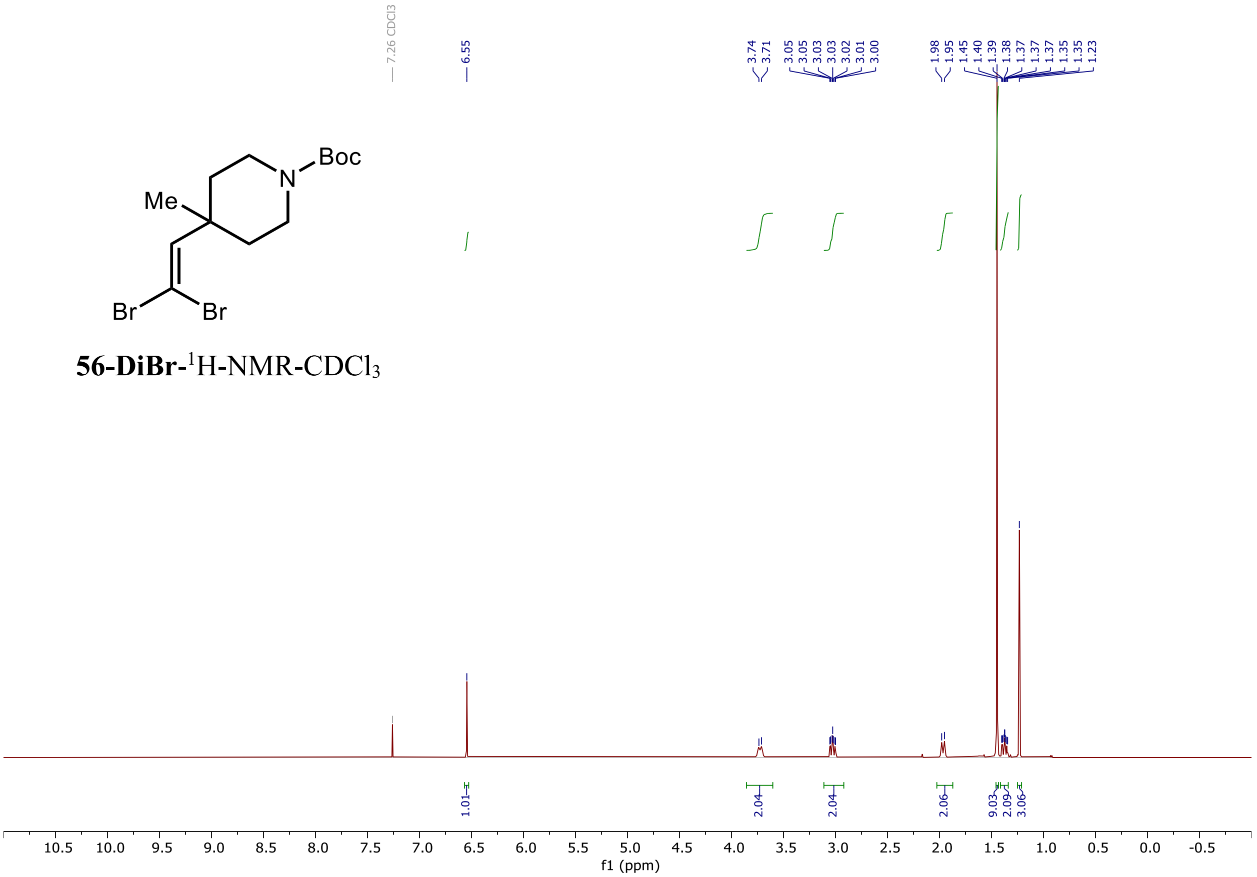

56-DiBr-^{1}H-NMR-$CDCl_3$

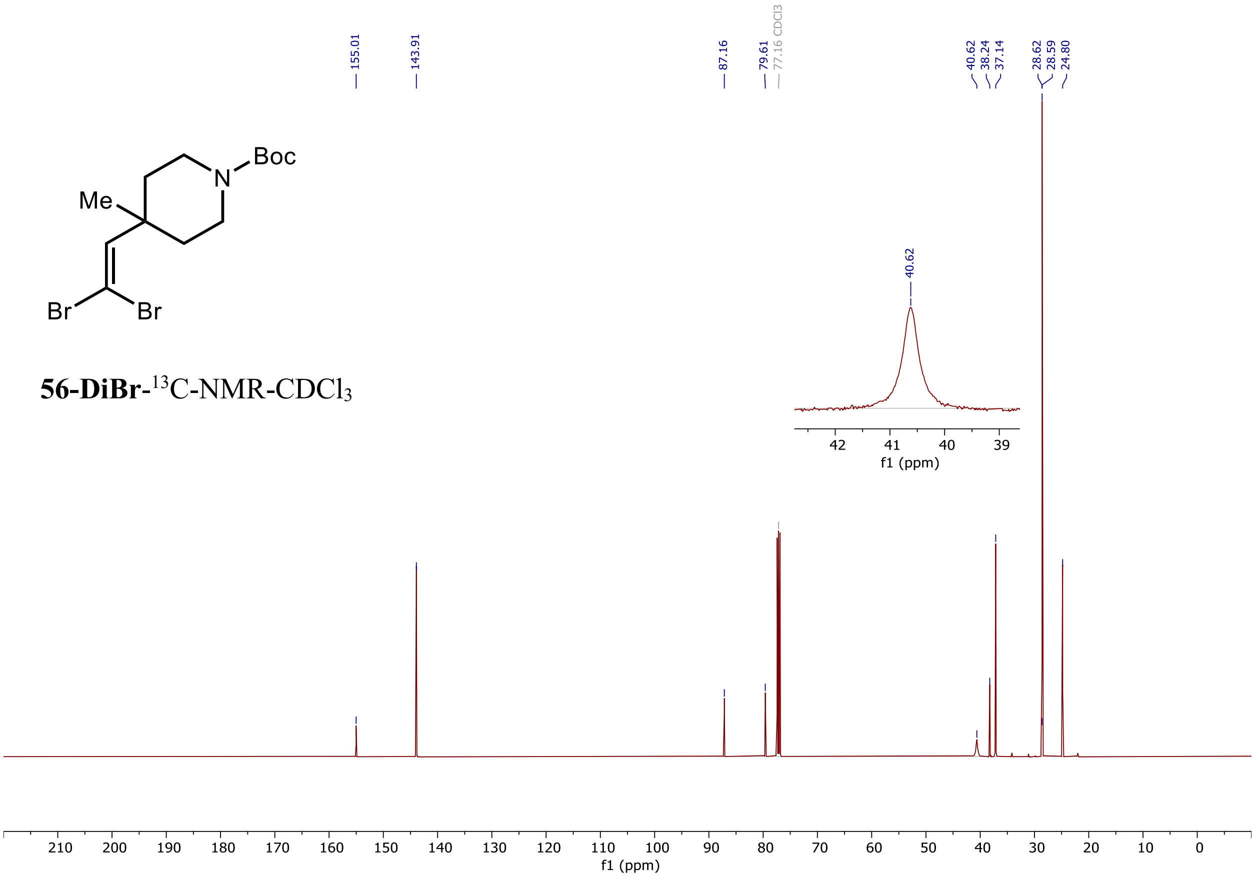

56-DiBr-^{13}C-NMR-$CDCl_3$

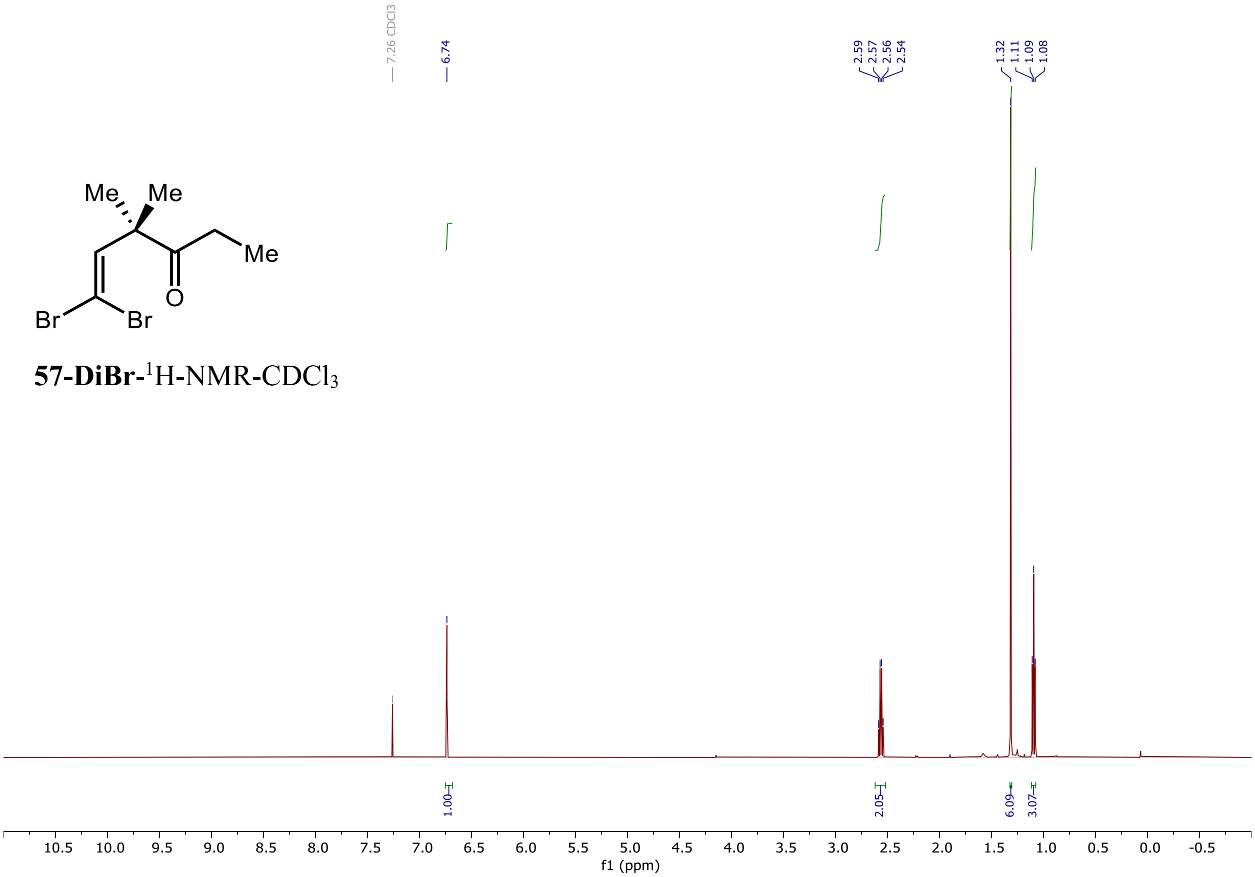

57-DiBr-^{1}H-NMR-$CDCl_3$

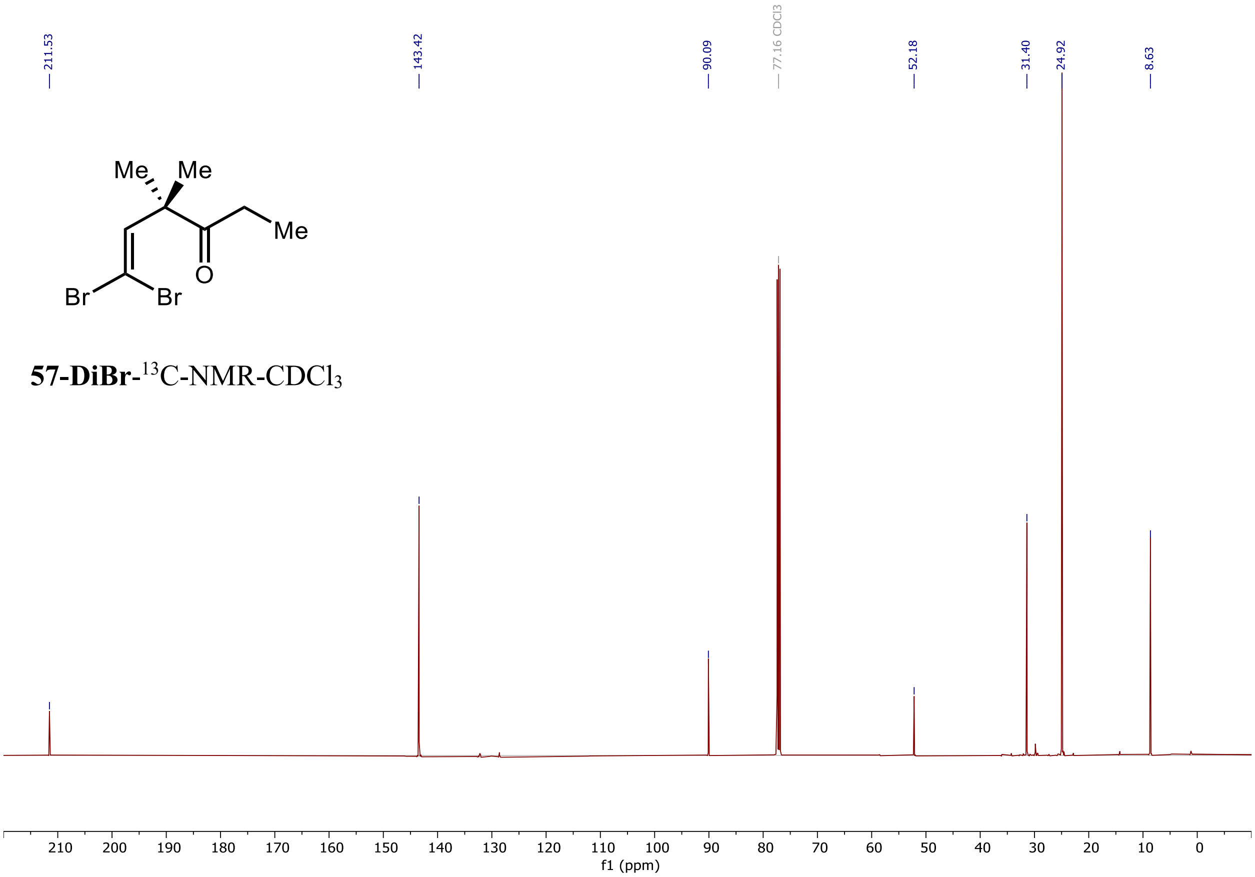

57-DiBr-^{13}C-NMR-$CDCl_3$

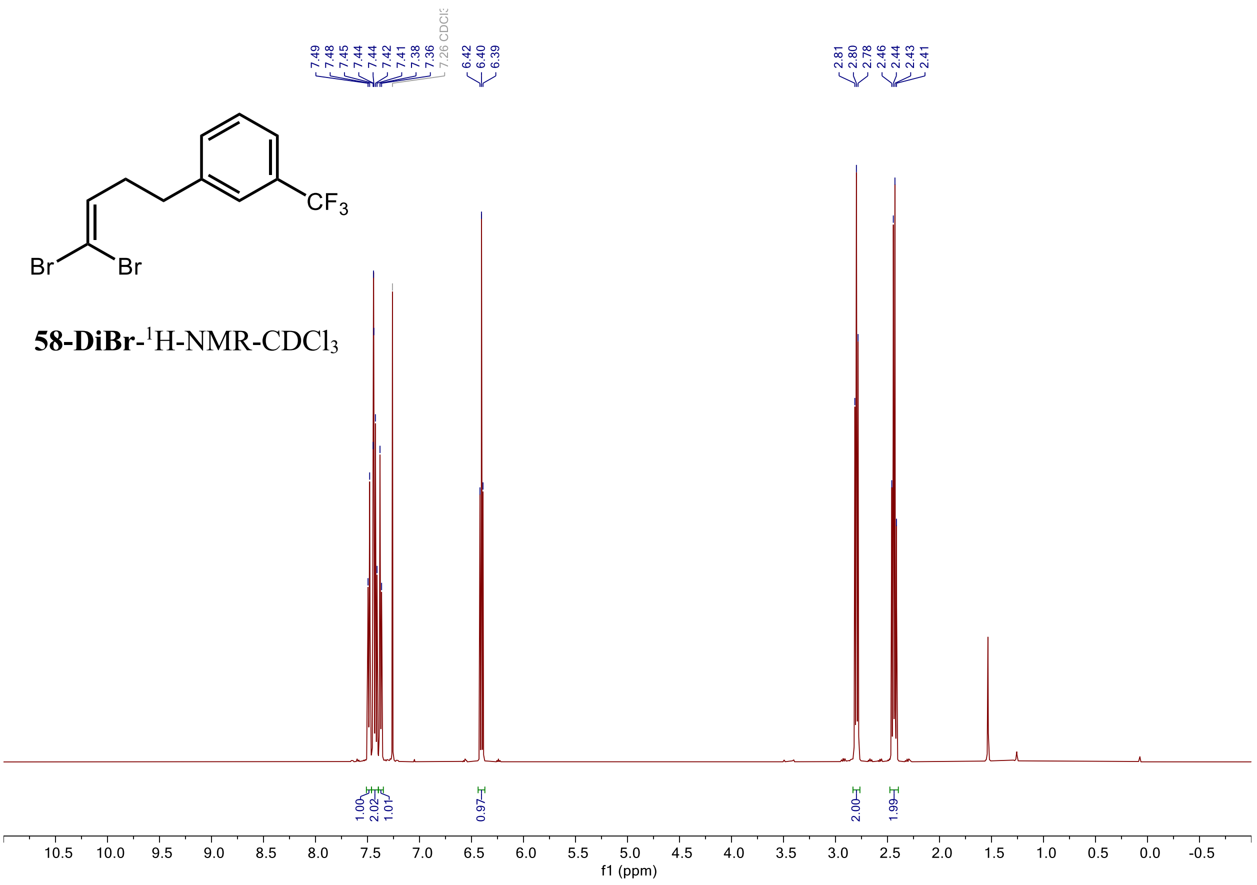
CF3
Br
Br
58-DiBr-1H-NMR-CDCl3
f1 (ppm)

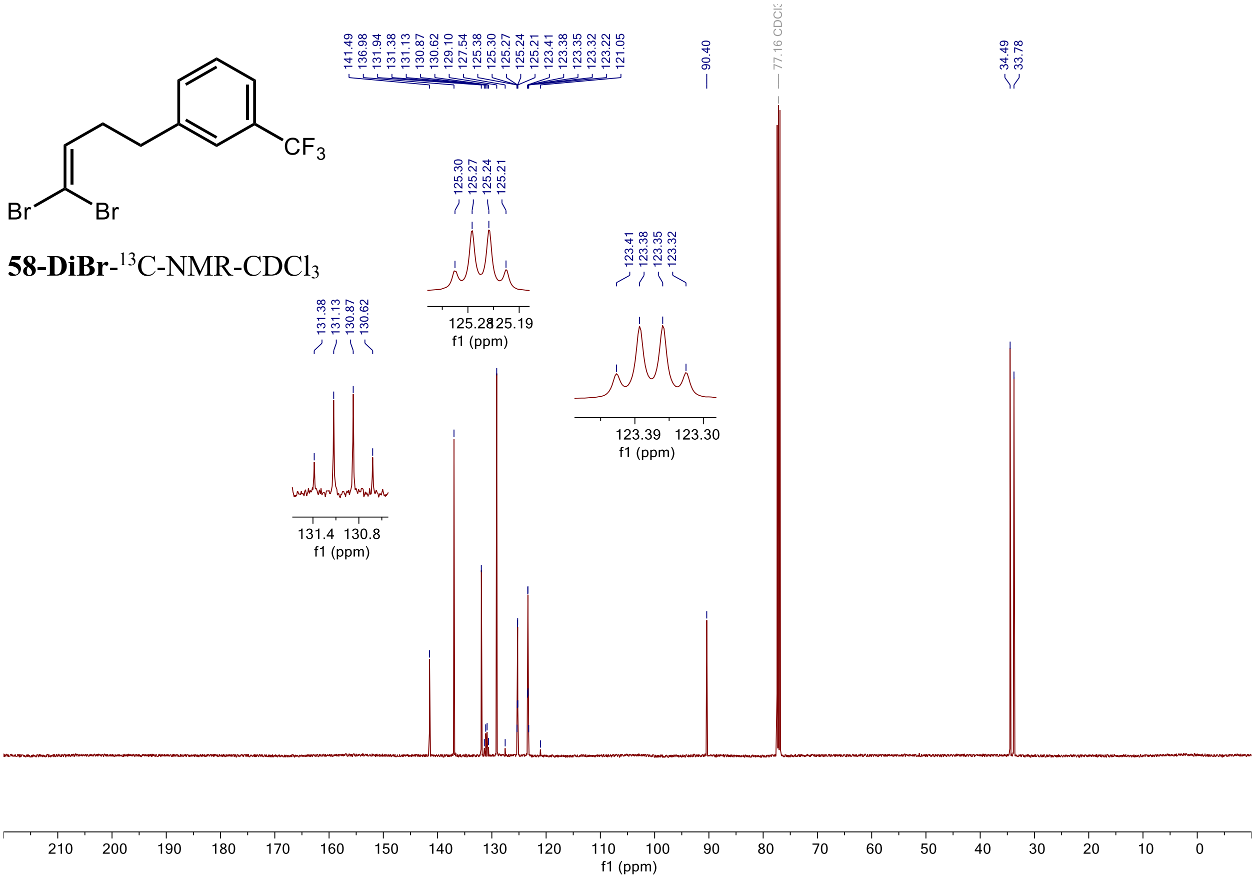
CF3
Br
Br
58-DiBr-13C-NMR-CDCl3
f1 (ppm)

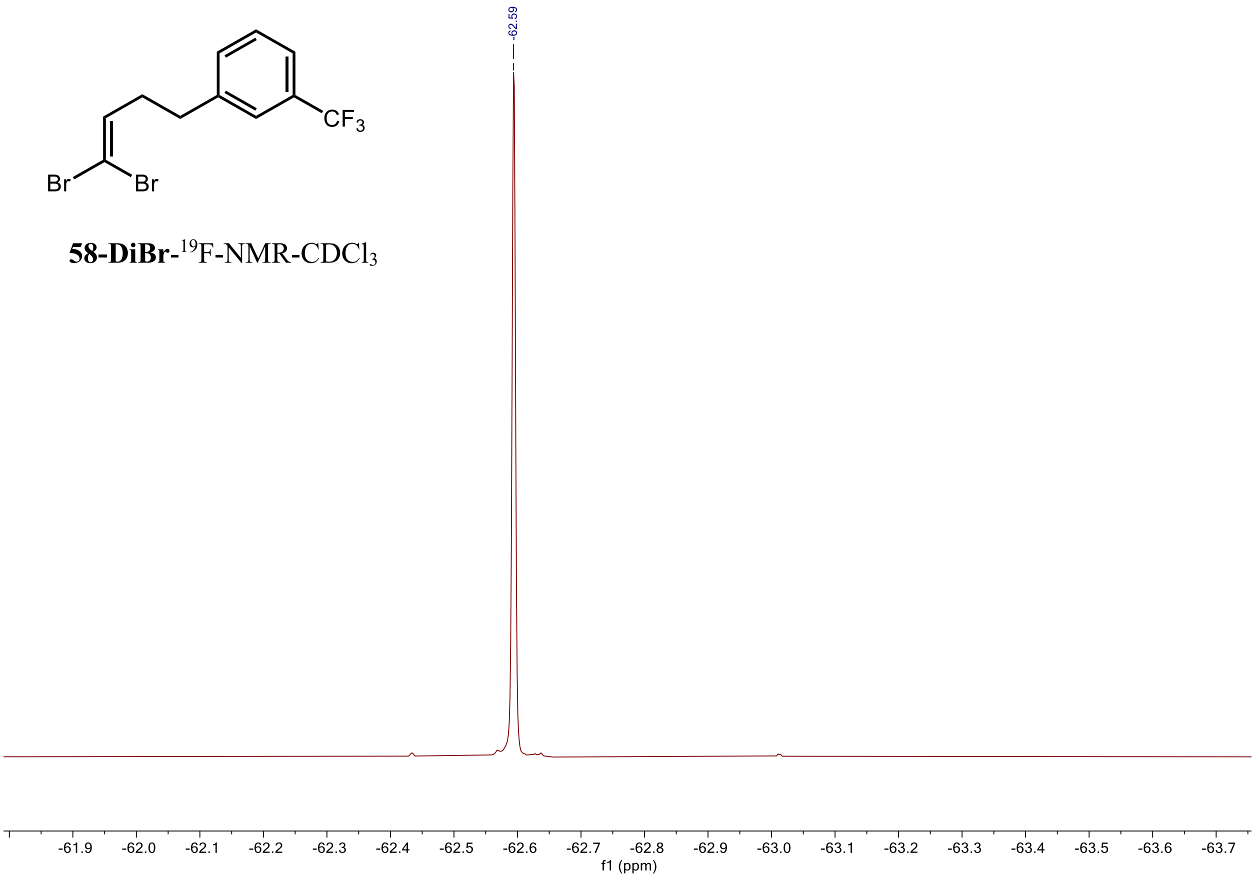

58-DiBr-^{19}F-NMR-CDCl$_3$

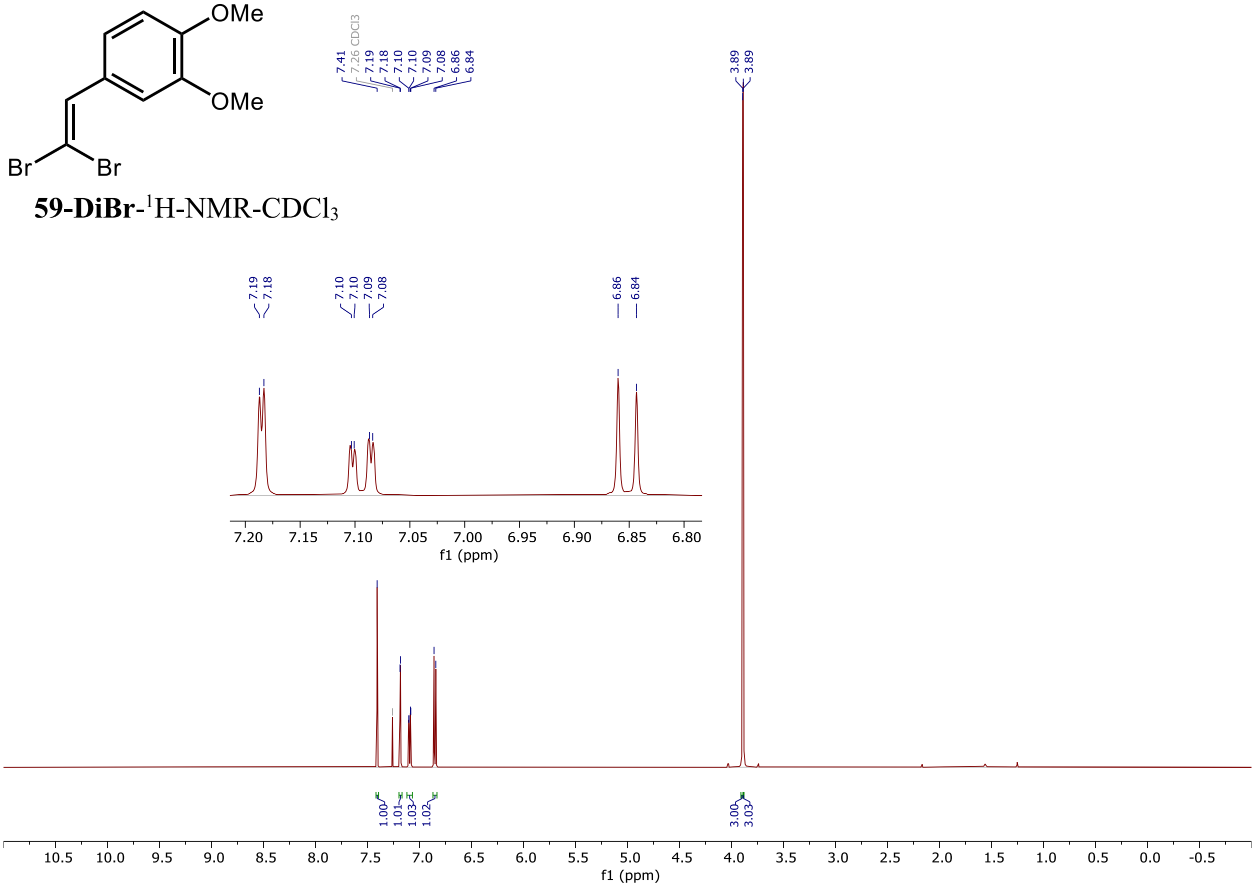

59-DiBr-^{1}H-NMR-CDCl$_3$

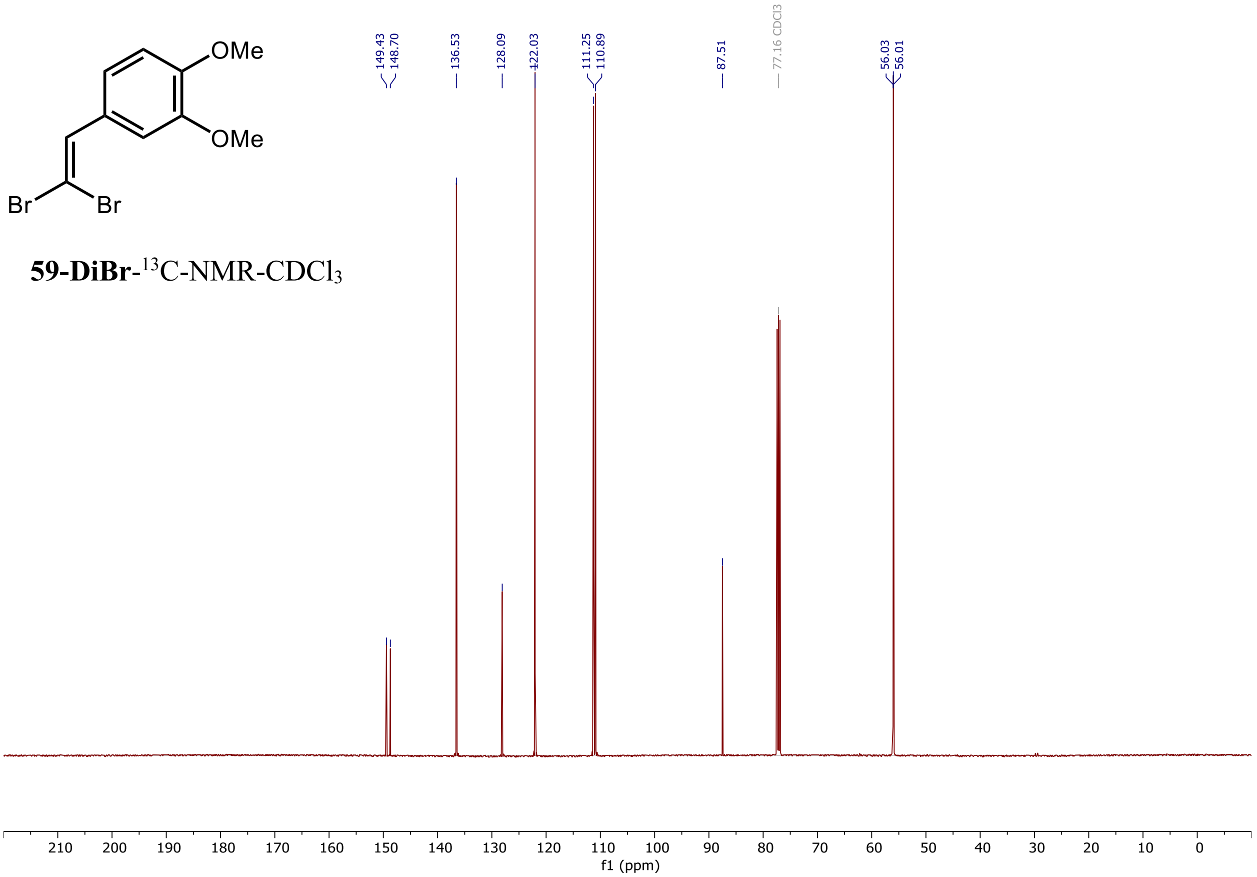

59-DiBr-^{13}C-NMR-$CDCl_3$

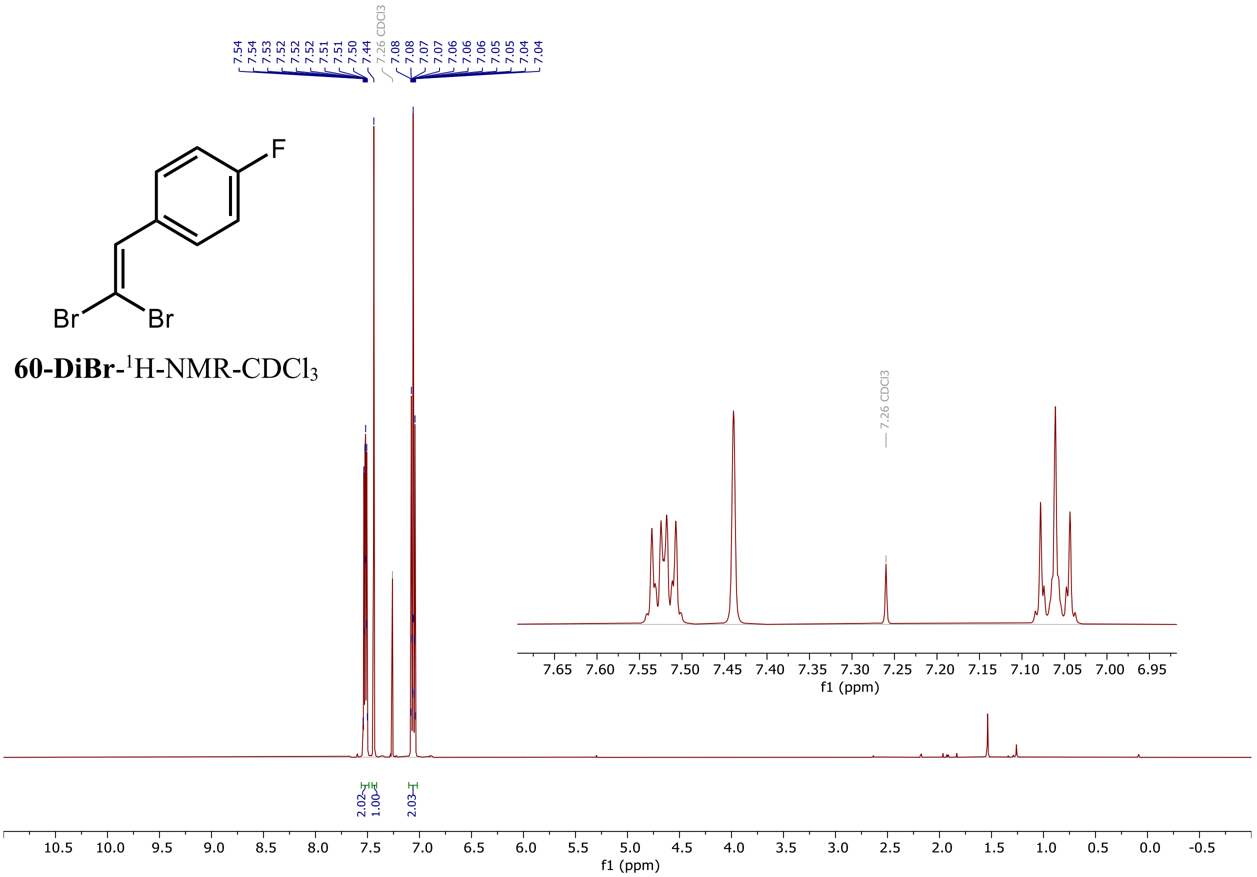

60-DiBr-^{1}H-NMR-$CDCl_3$

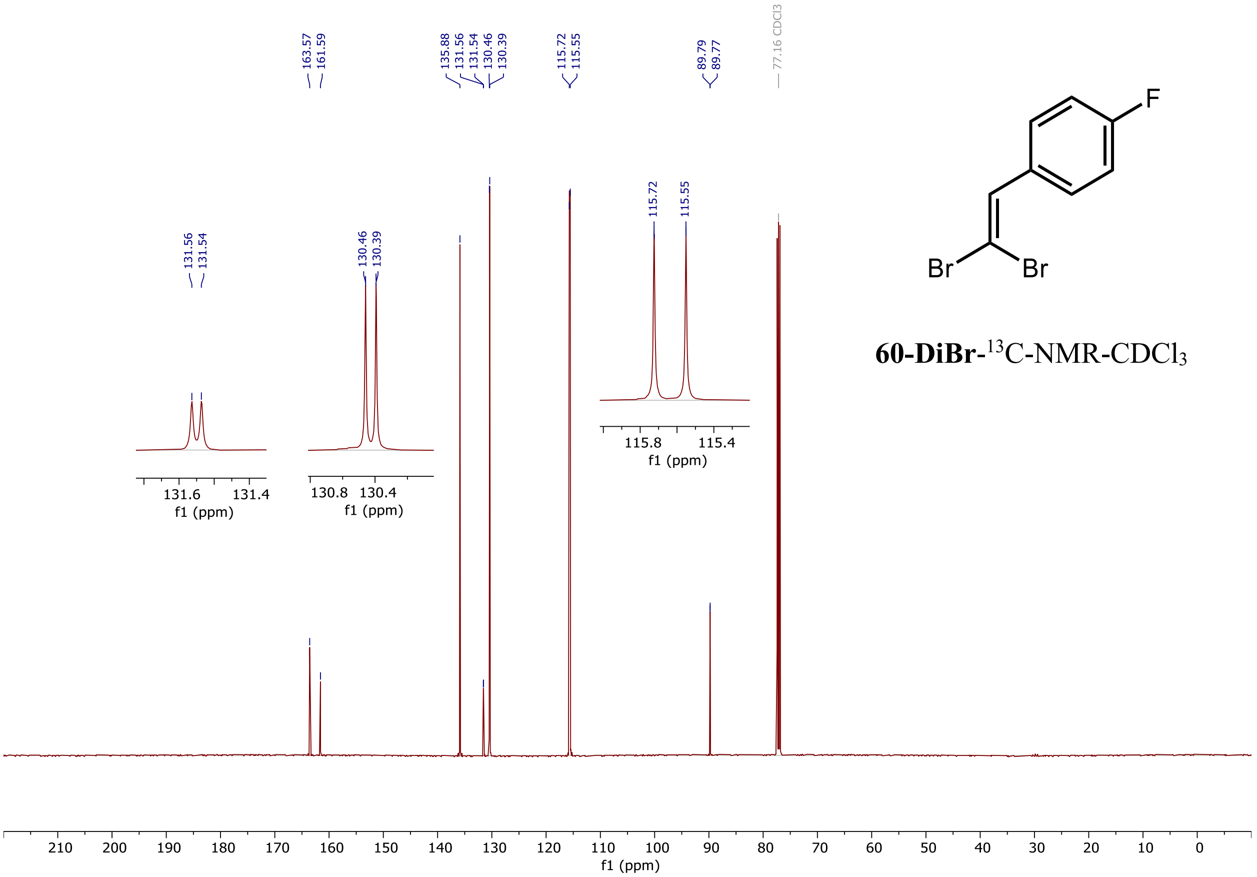

163.57
161.59
135.88
131.56
131.54
130.46
130.39
115.72
115.55
89.79
89.77
77.16 CDCl3
F
Br
Br
60-DiBr-13C-NMR-CDCl3
131.56
131.54
130.46
130.39
115.72
115.55
f1 (ppm)

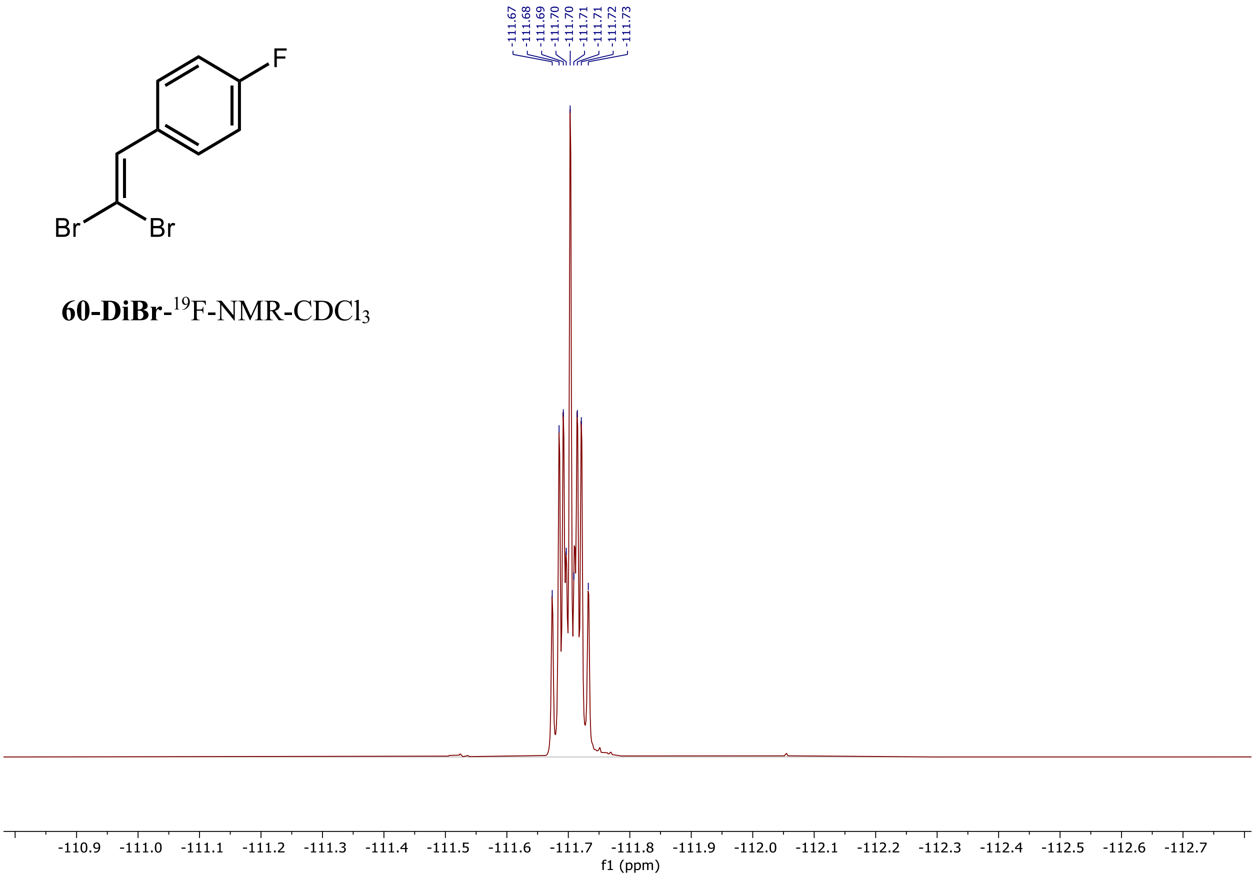

-111.67
-111.68
-111.69
-111.70
-111.70
-111.71
-111.71
-111.72
-111.73
F
Br
Br
60-DiBr-19F-NMR-CDCl3
f1 (ppm)

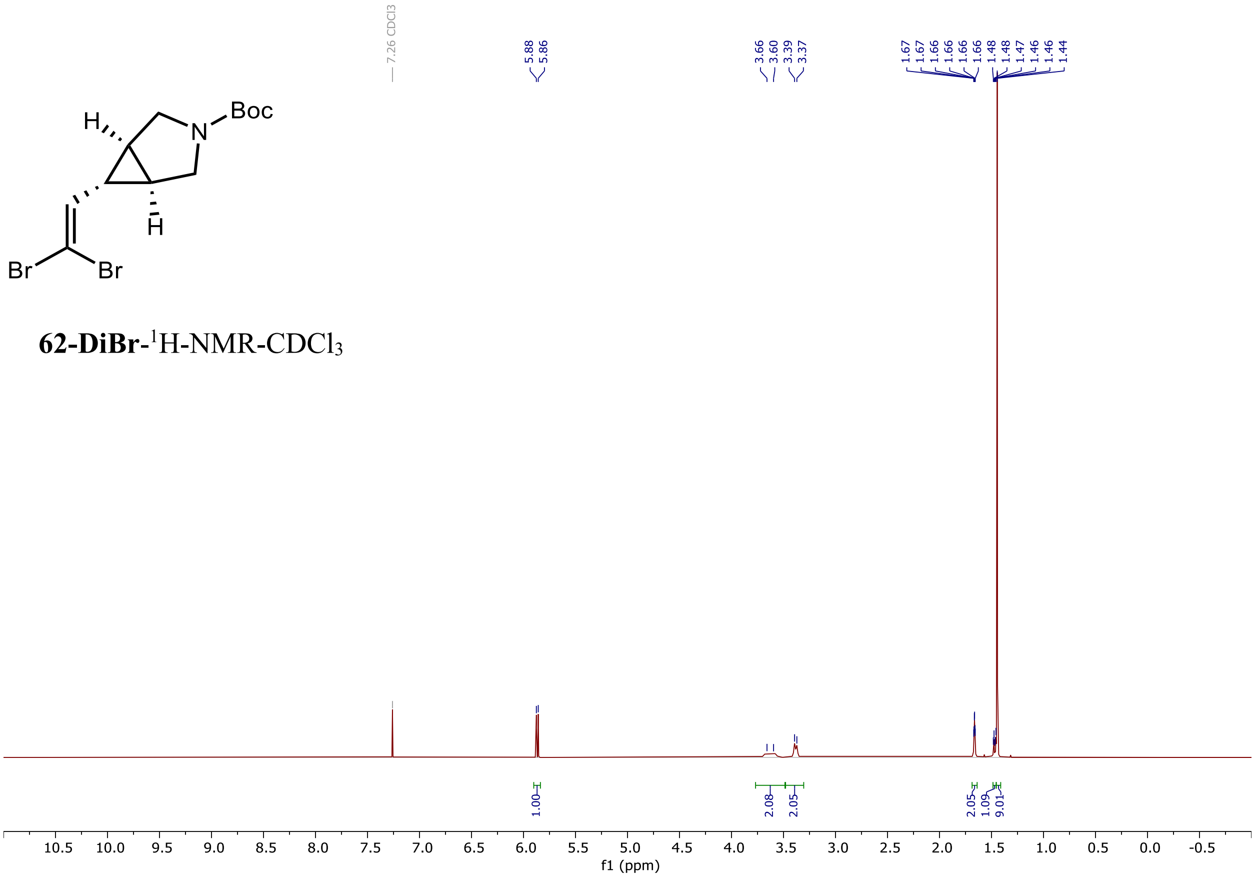

62-DiBr-^{1}H-NMR-$CDCl_3$

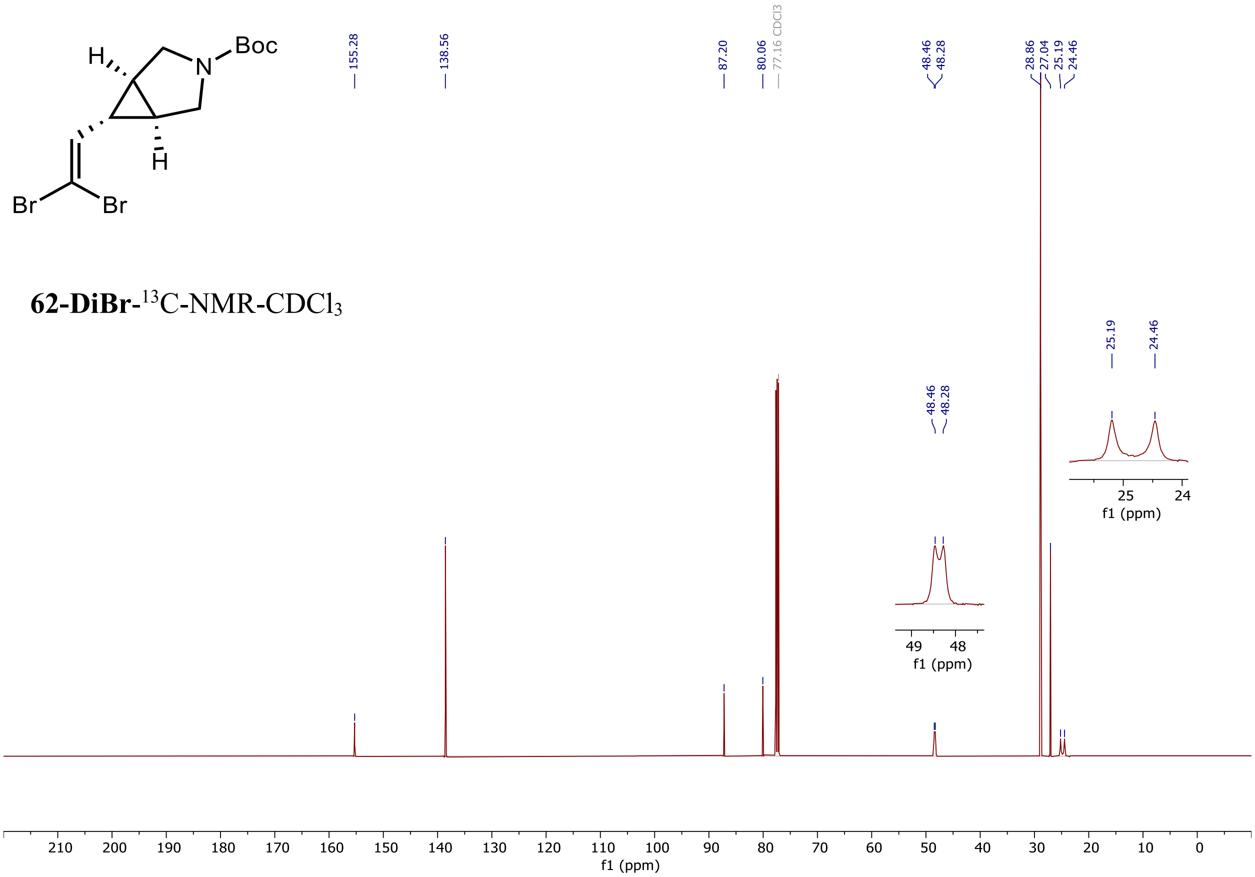

62-DiBr-^{13}C-NMR-$CDCl_3$

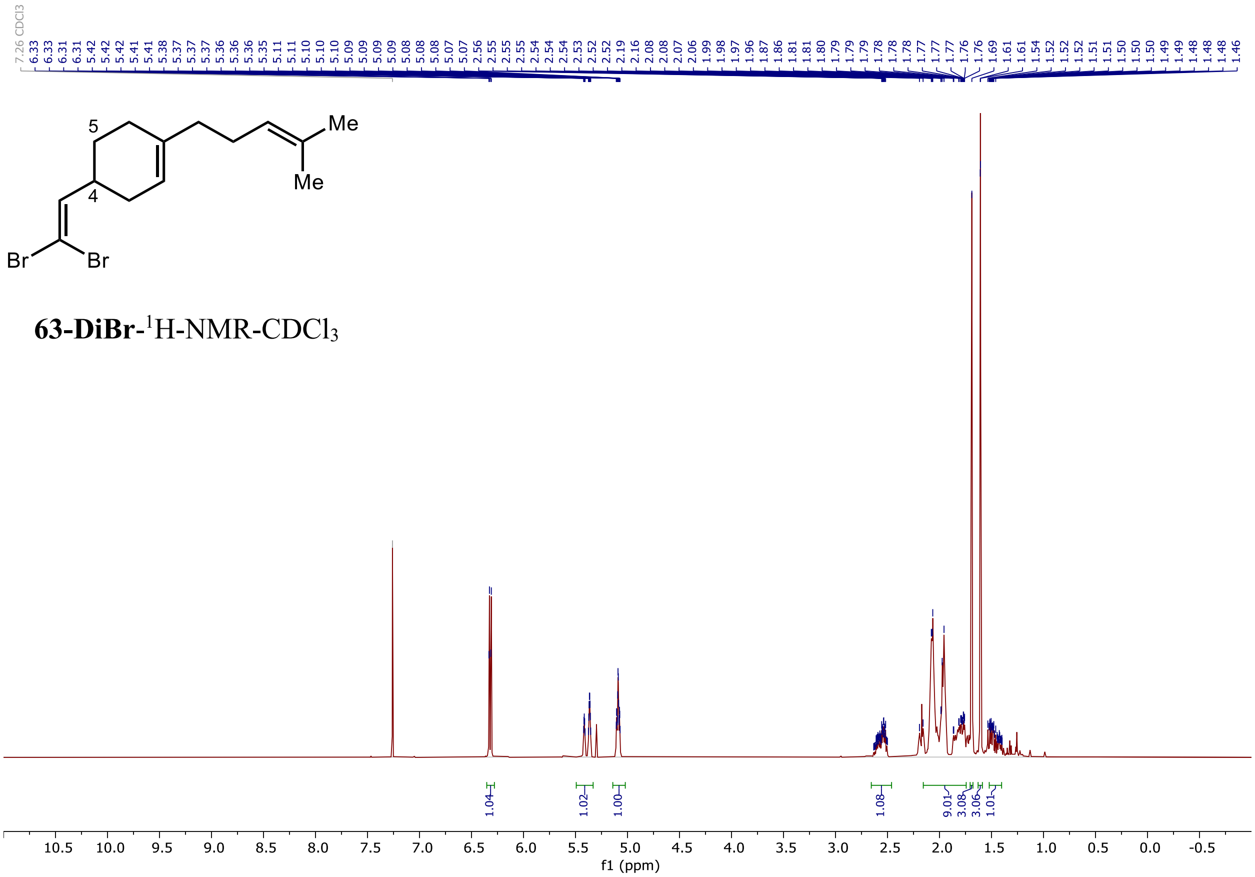

63-DiBr-^{1}H-NMR-$CDCl_3$

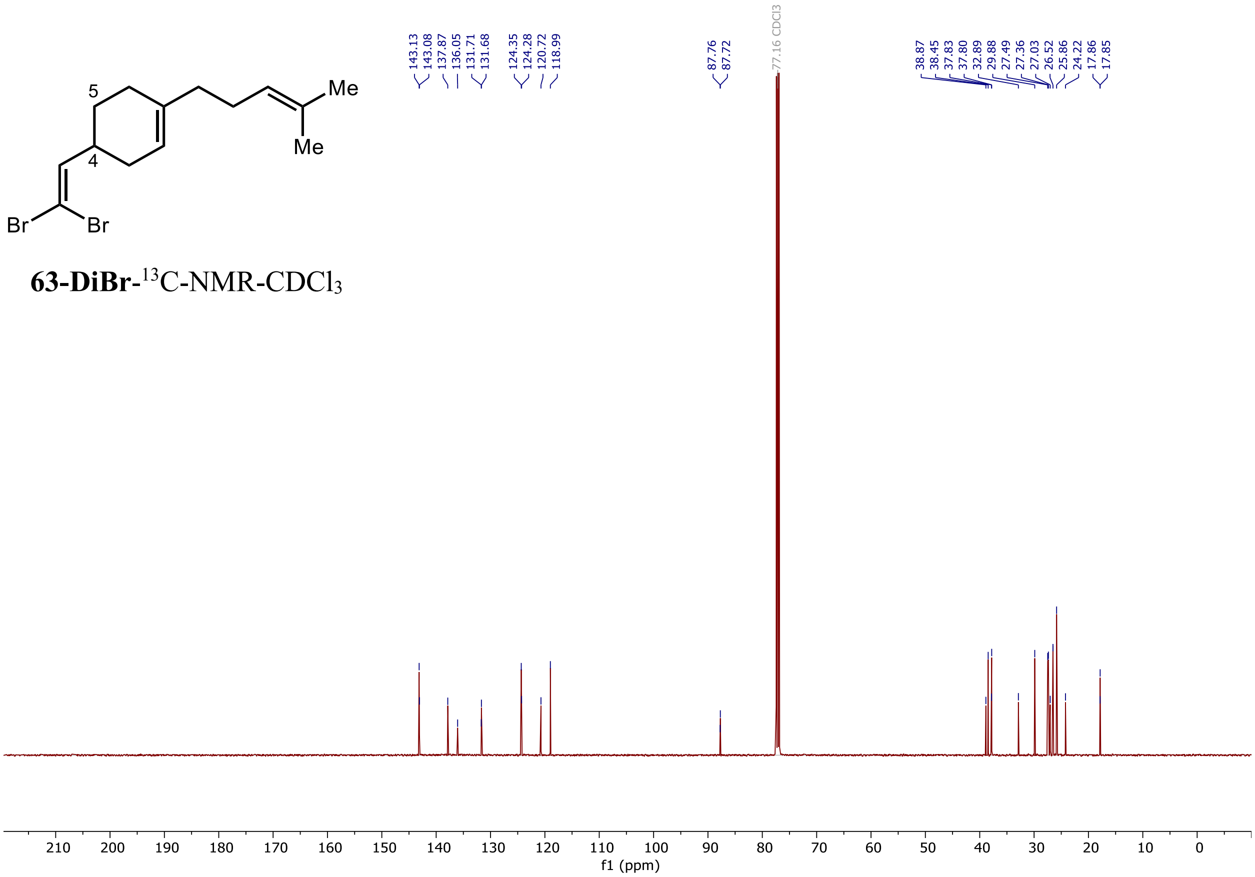

63-DiBr-^{13}C-NMR-$CDCl_3$

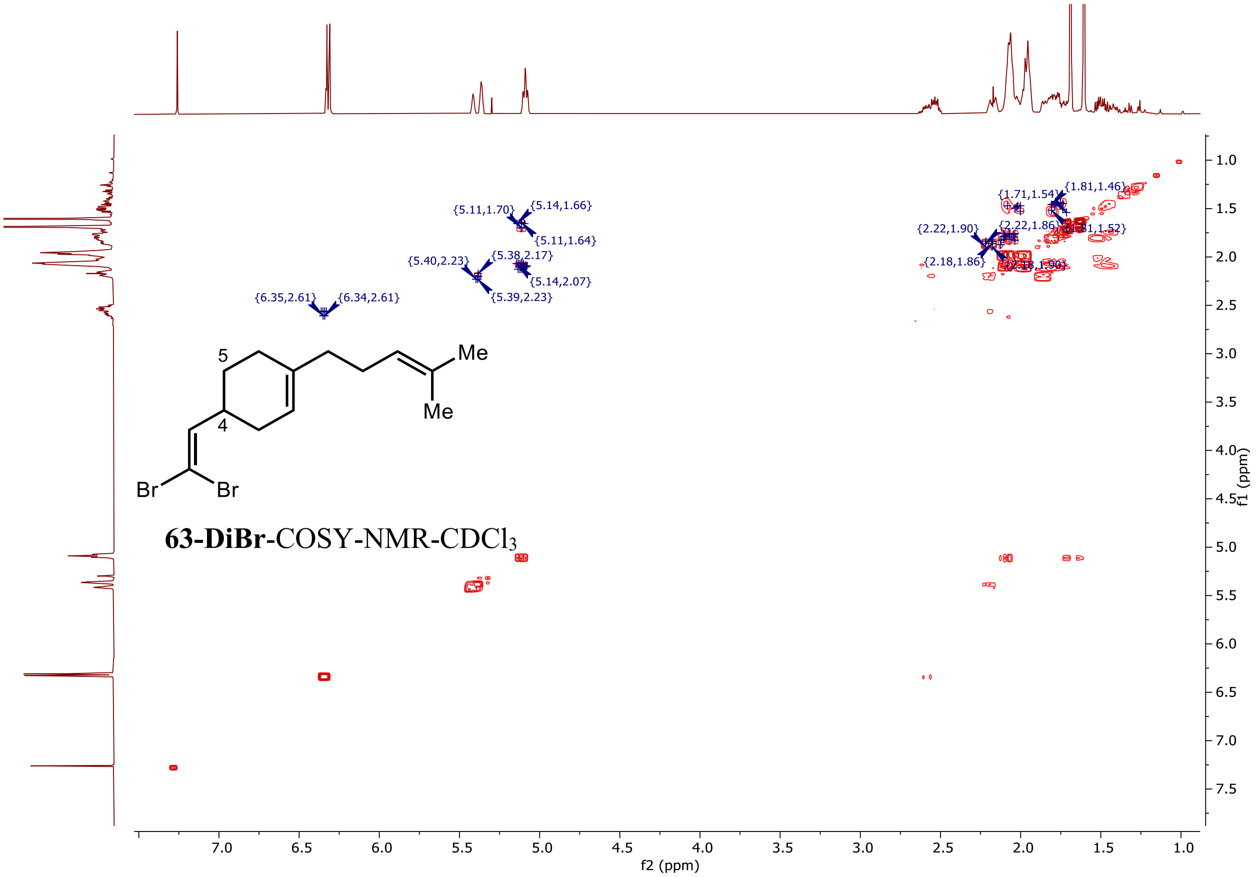
63-DiBr-COSY-NMR-CDCl3
5
4
Me
Me
Br
Br
{5.11,1.70}
{5.14,1.66}
{5.11,1.64}
{5.40,2.23}
{5.38,2.17}
{5.14,2.07}
{5.39,2.23}
{6.35,2.61}
{6.34,2.61}
{1.71,1.54}
{1.81,1.46}
{2.22,1.90}
{2.22,1.86}
{2.18,1.86}
f2 (ppm)
f1 (ppm)

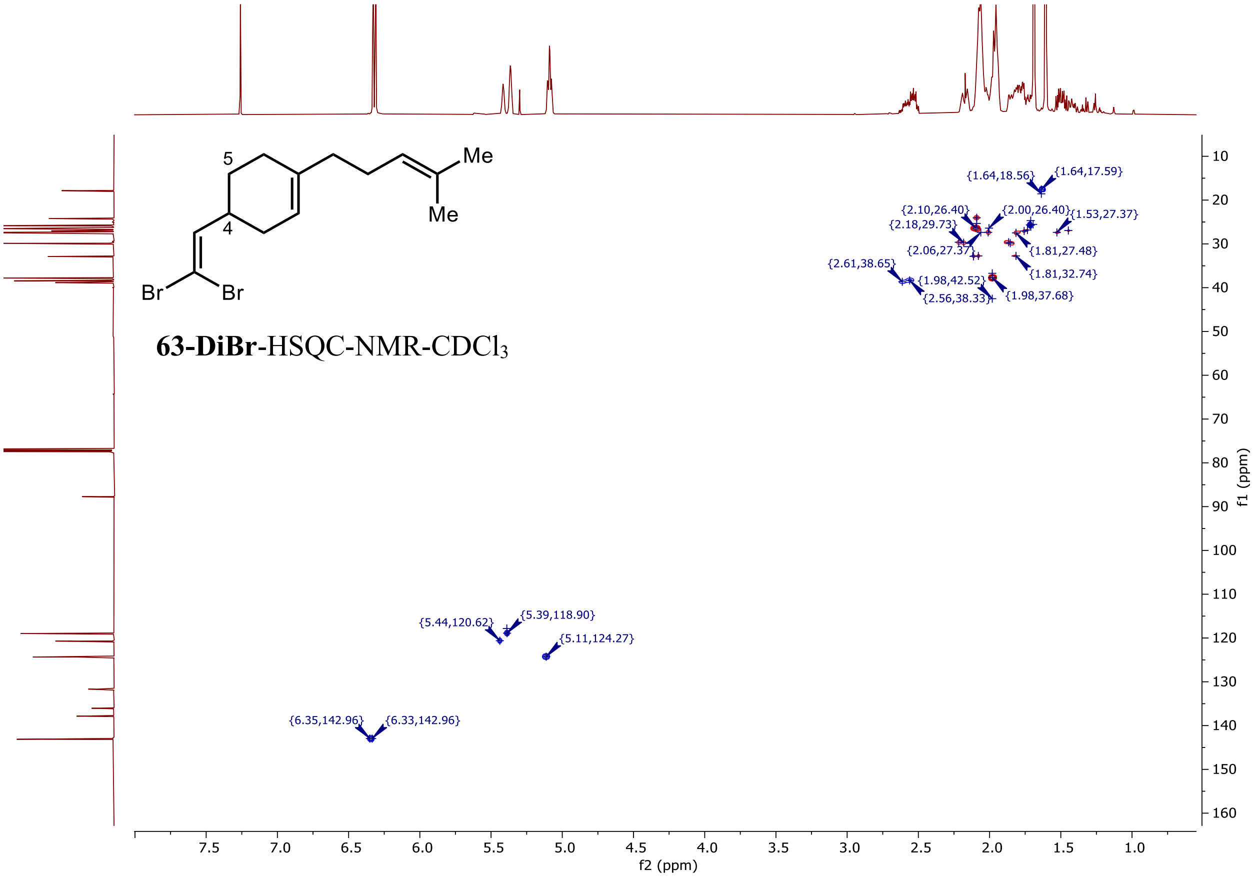
63-DiBr-HSQC-NMR-CDCl3
5
4
Me
Me
Br
Br
{1.64,18.56}
{1.64,17.59}
{2.10,26.40}
{2.00,26.40}
{1.53,27.37}
{2.18,29.73}
{2.06,27.37}
{1.81,27.48}
{2.61,38.65}
{1.81,32.74}
{1.98,42.52}
{2.56,38.33}
{1.98,37.68}
{5.44,120.62}
{5.39,118.90}
{5.11,124.27}
{6.35,142.96}
{6.33,142.96}
f2 (ppm)
f1 (ppm)

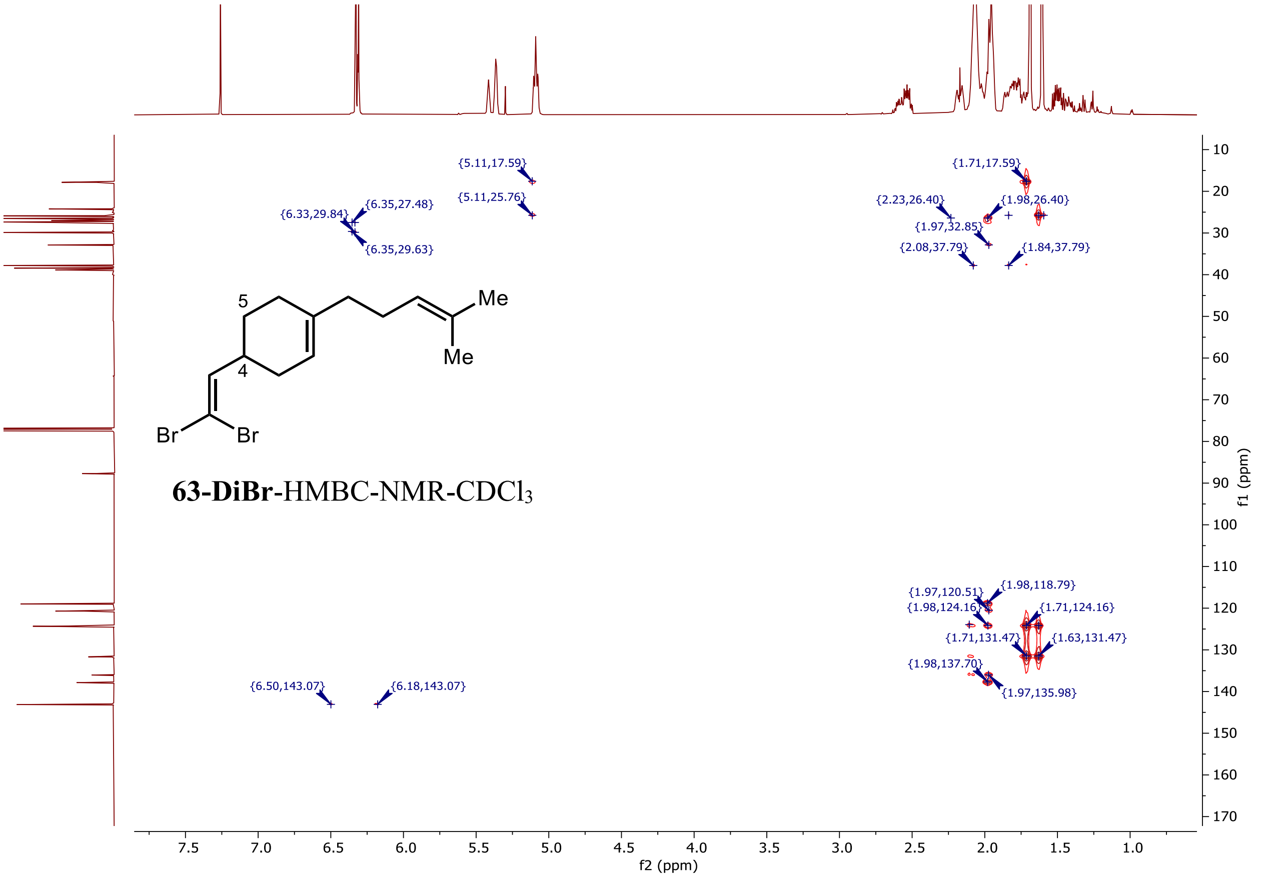

63-DiBr-HMBC-NMR-$CDCl_3$

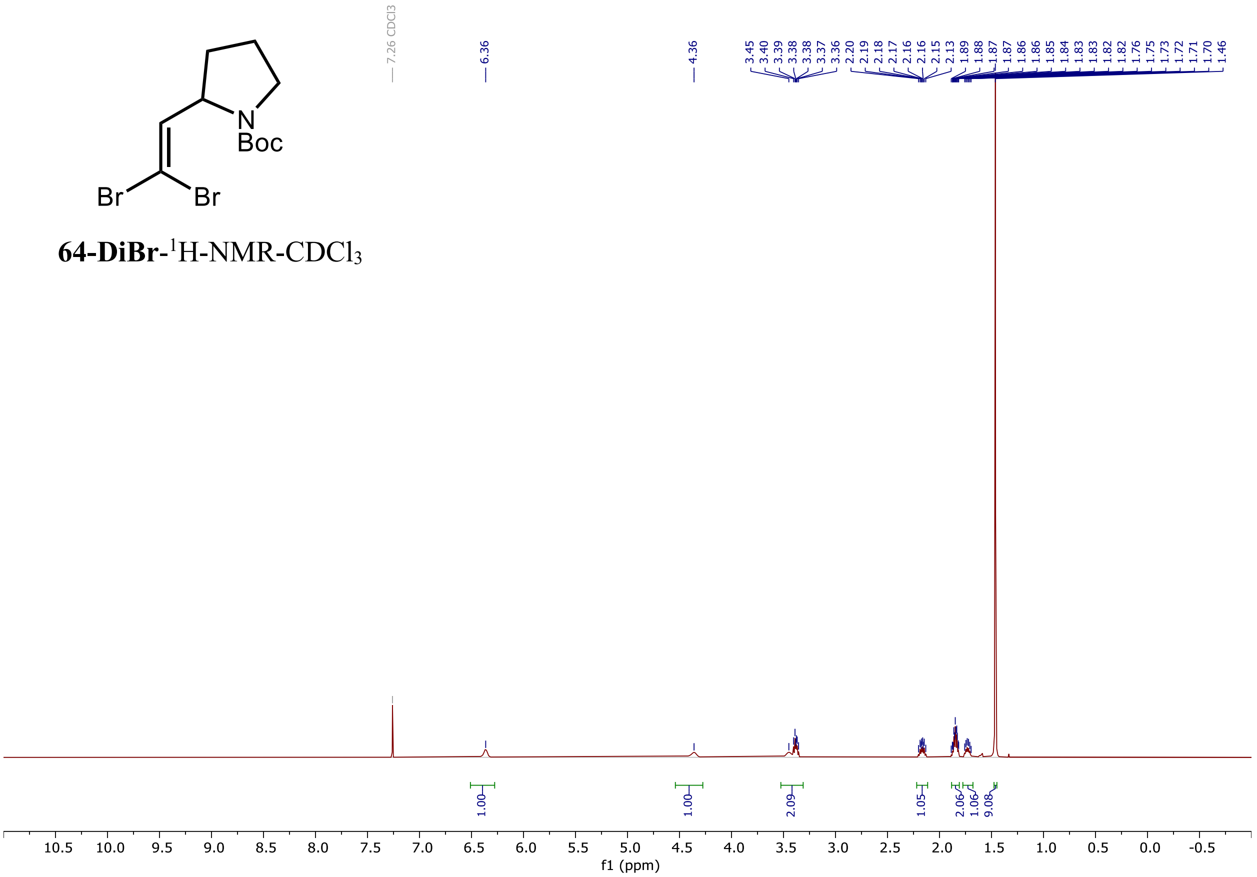

64-DiBr-^{1}H-NMR-$CDCl_3$

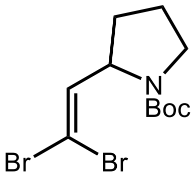

64-DiBr-^{13}C-NMR-$CDCl_3$

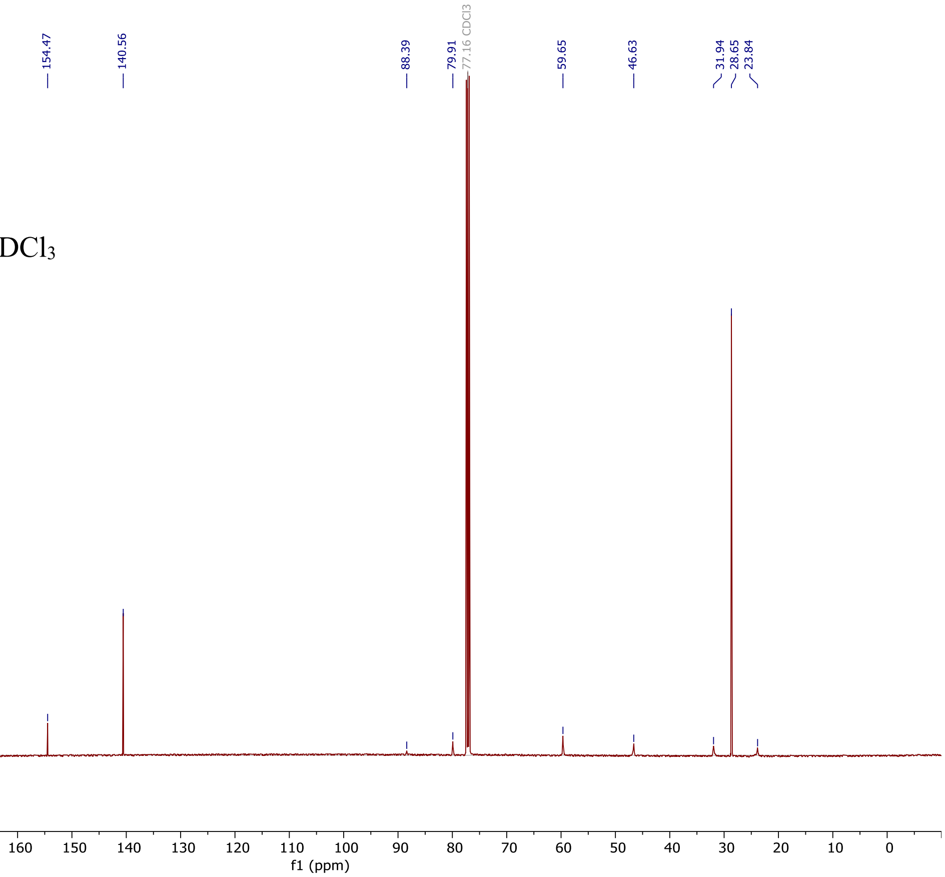

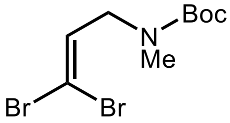

65-DiBr-^{1}H-NMR-$CDCl_3$

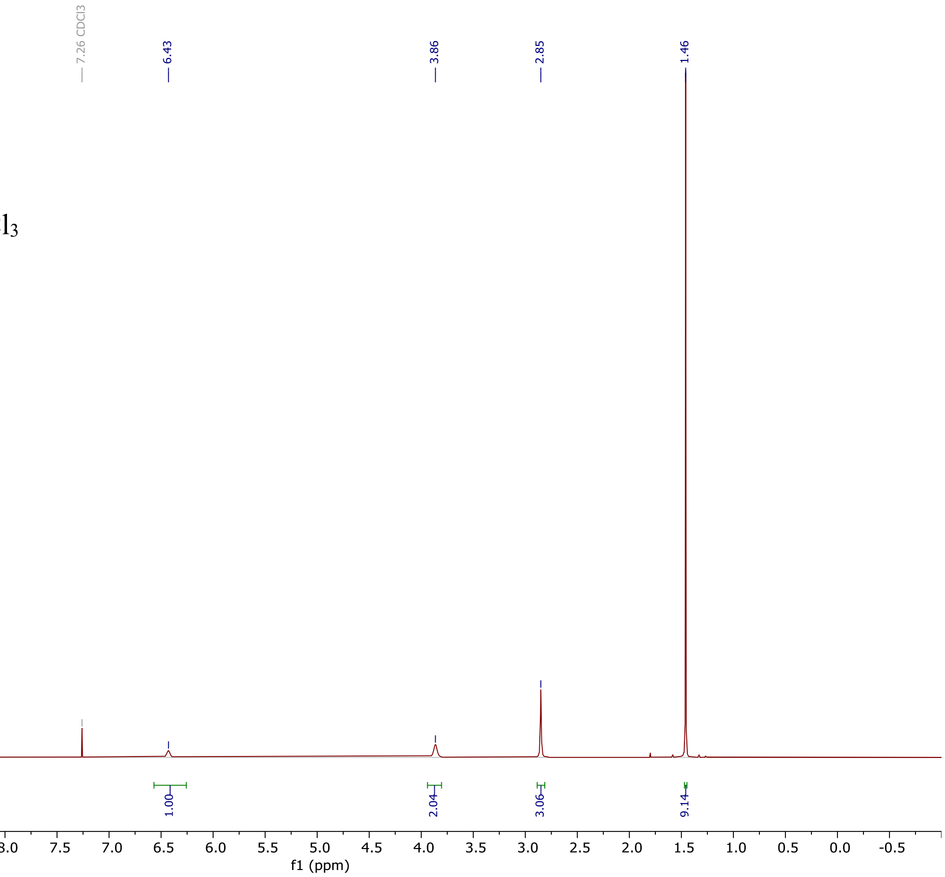

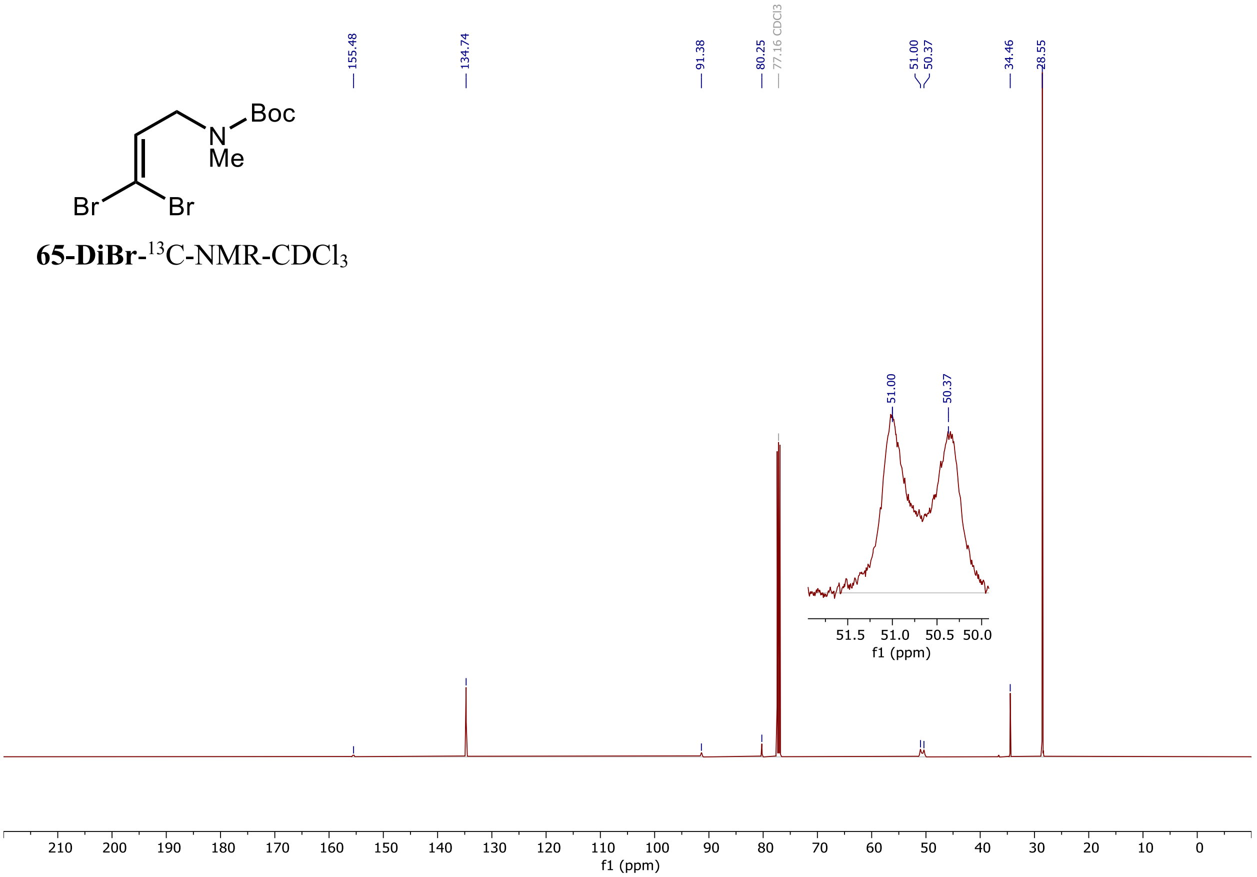

65-DiBr-^{13}C-NMR-$CDCl_3$

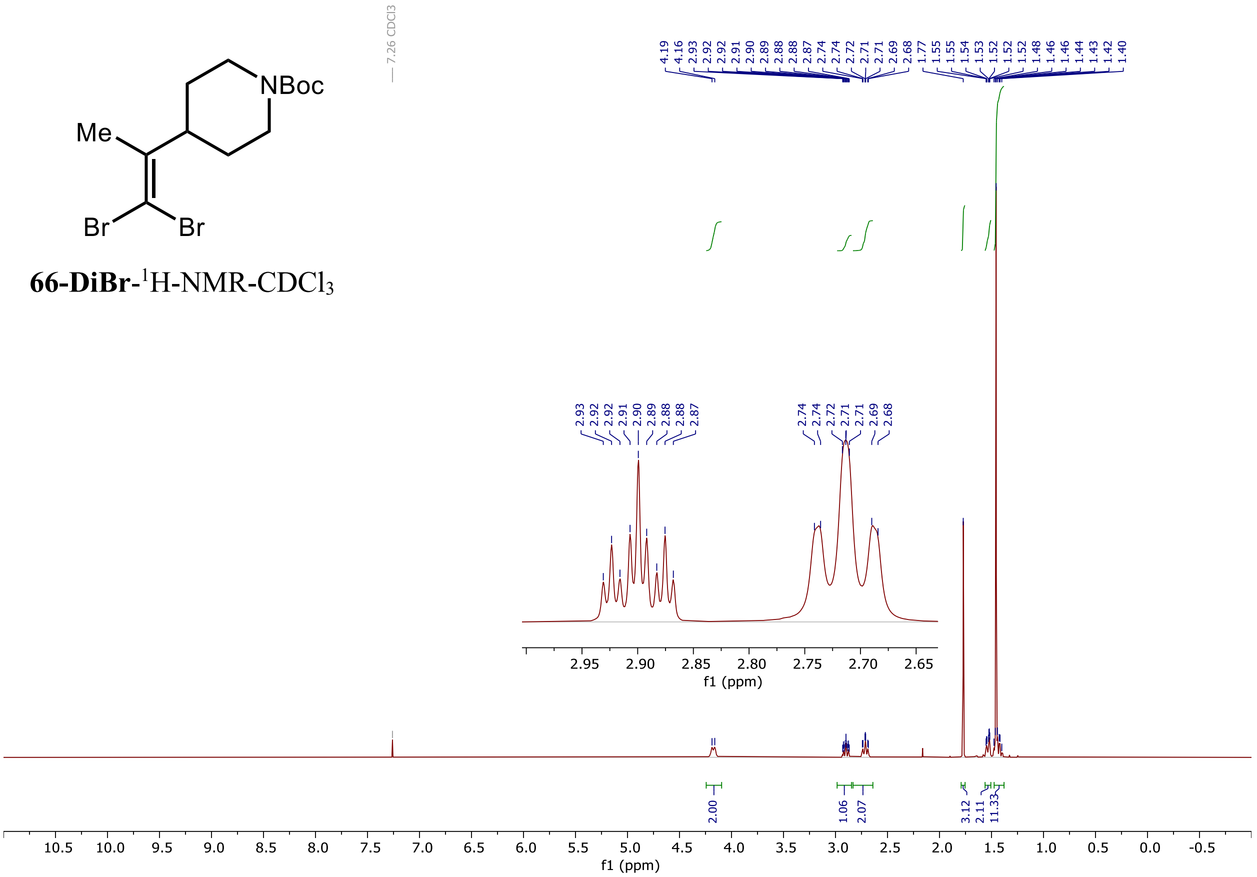

66-DiBr-^{1}H-NMR-$CDCl_3$

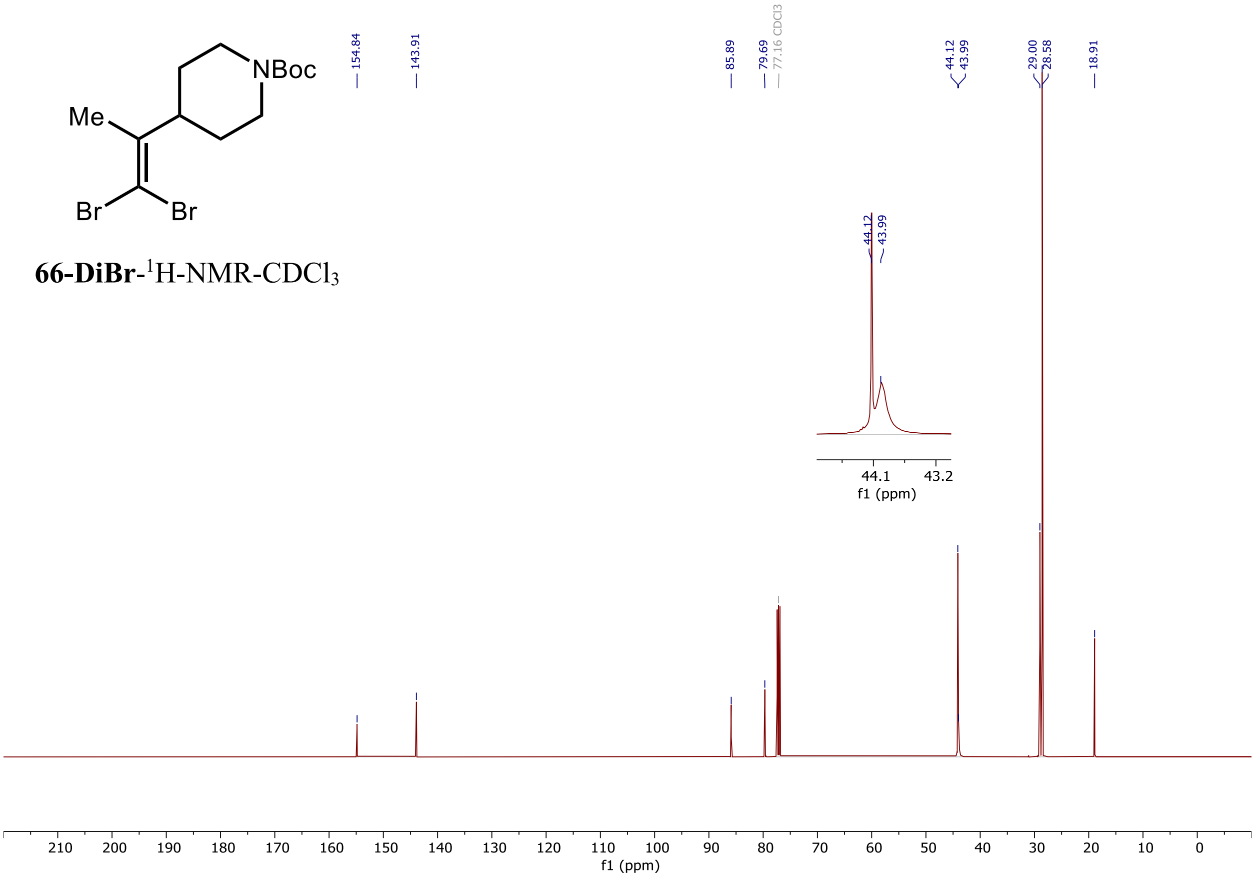

66-DiBr-^{1}H-NMR-$CDCl_3$

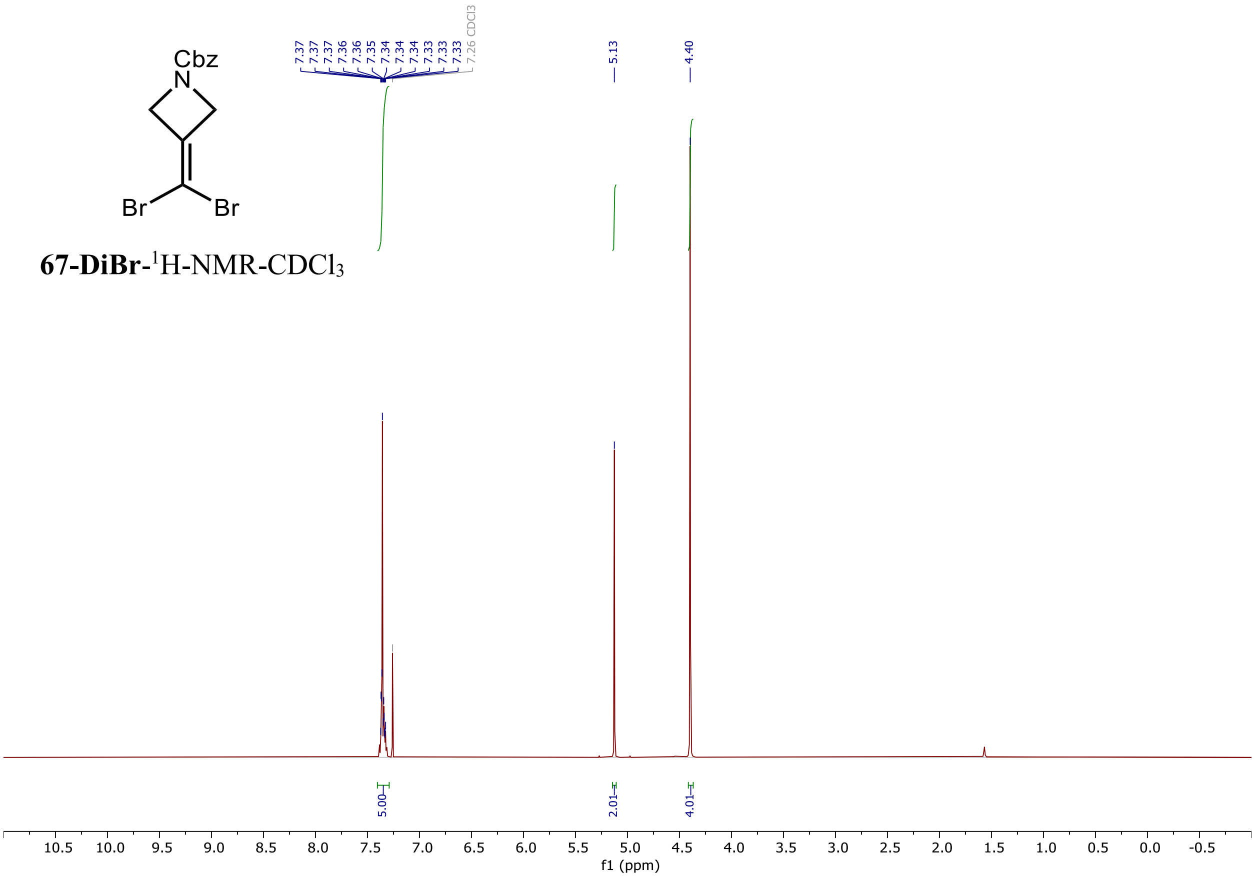

67-DiBr-^{1}H-NMR-$CDCl_3$

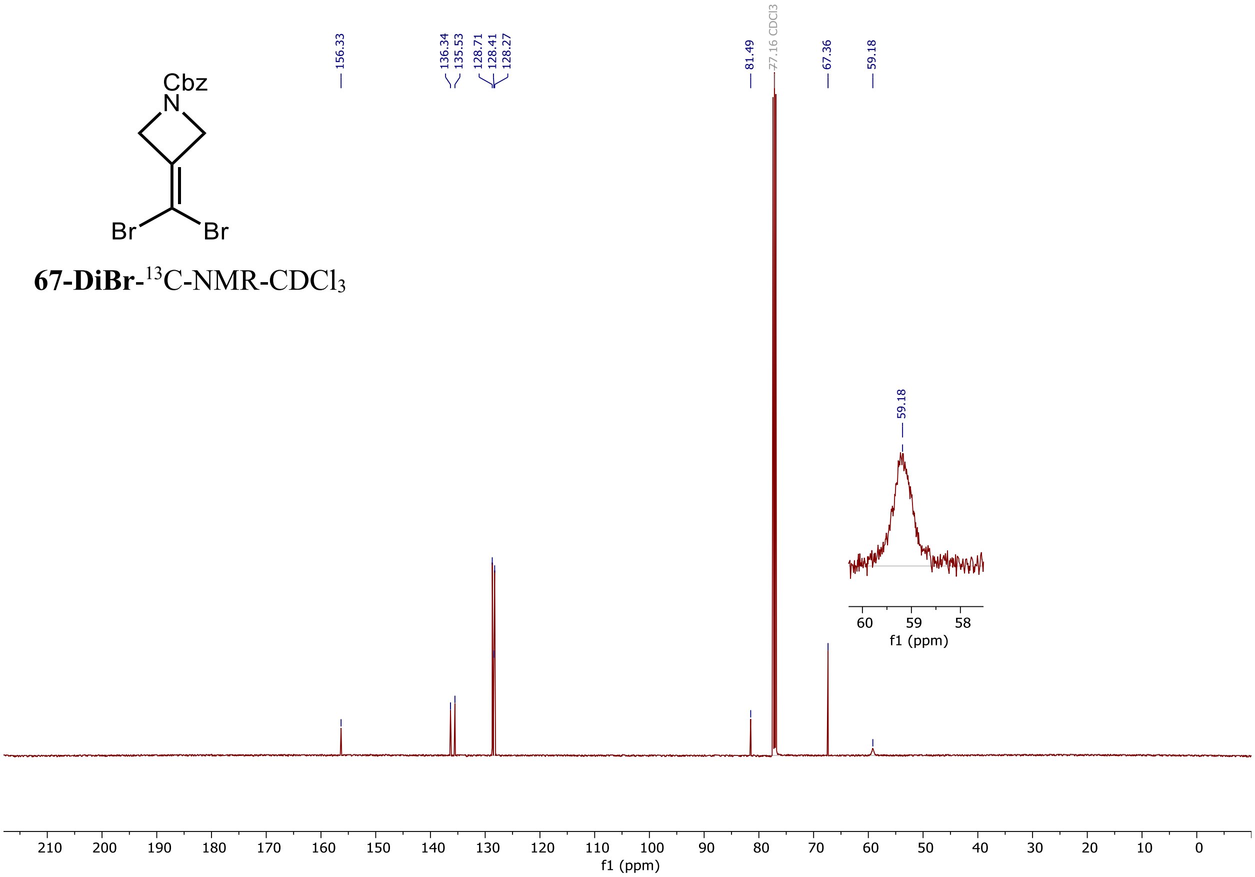

67-DiBr-^{13}C-NMR-$CDCl_3$

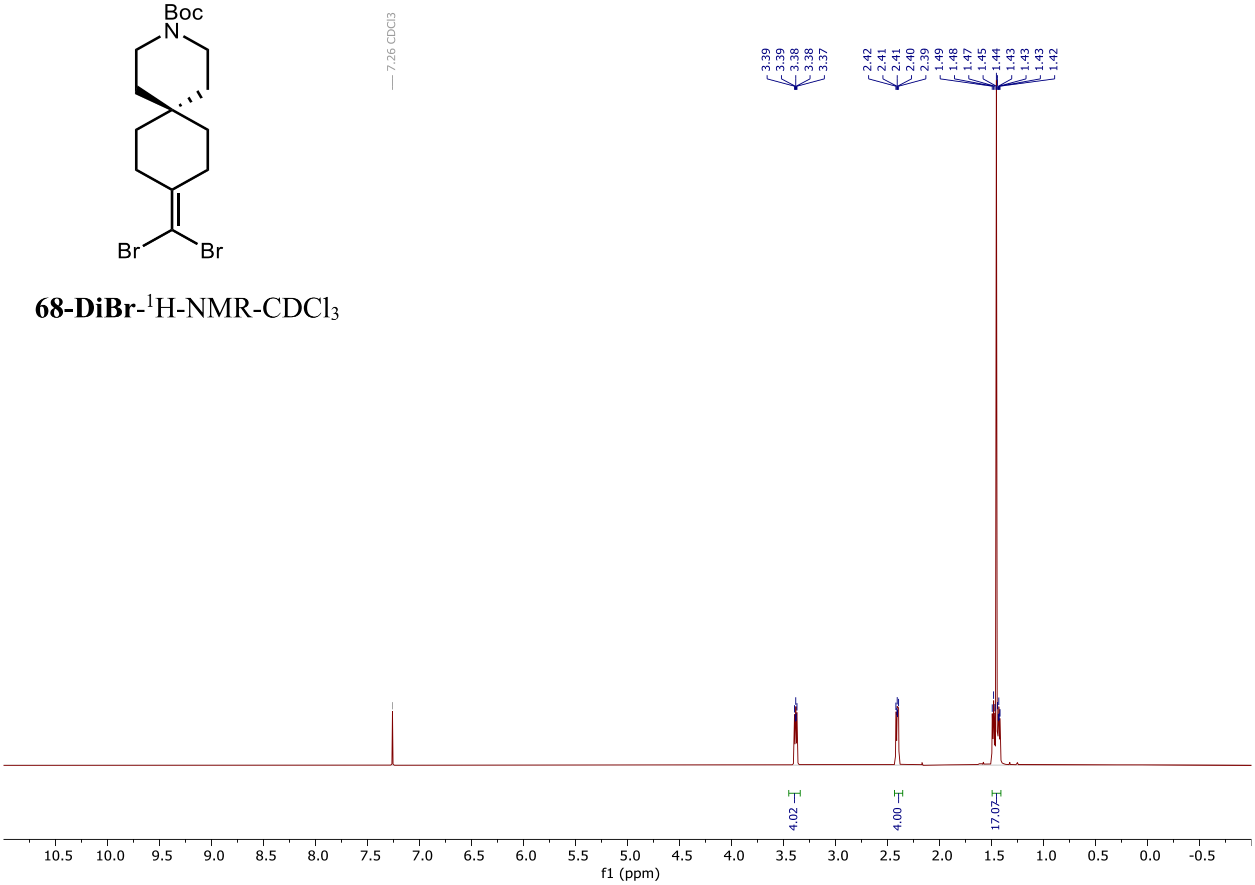

68-DiBr-^{1}H-NMR-$CDCl_3$

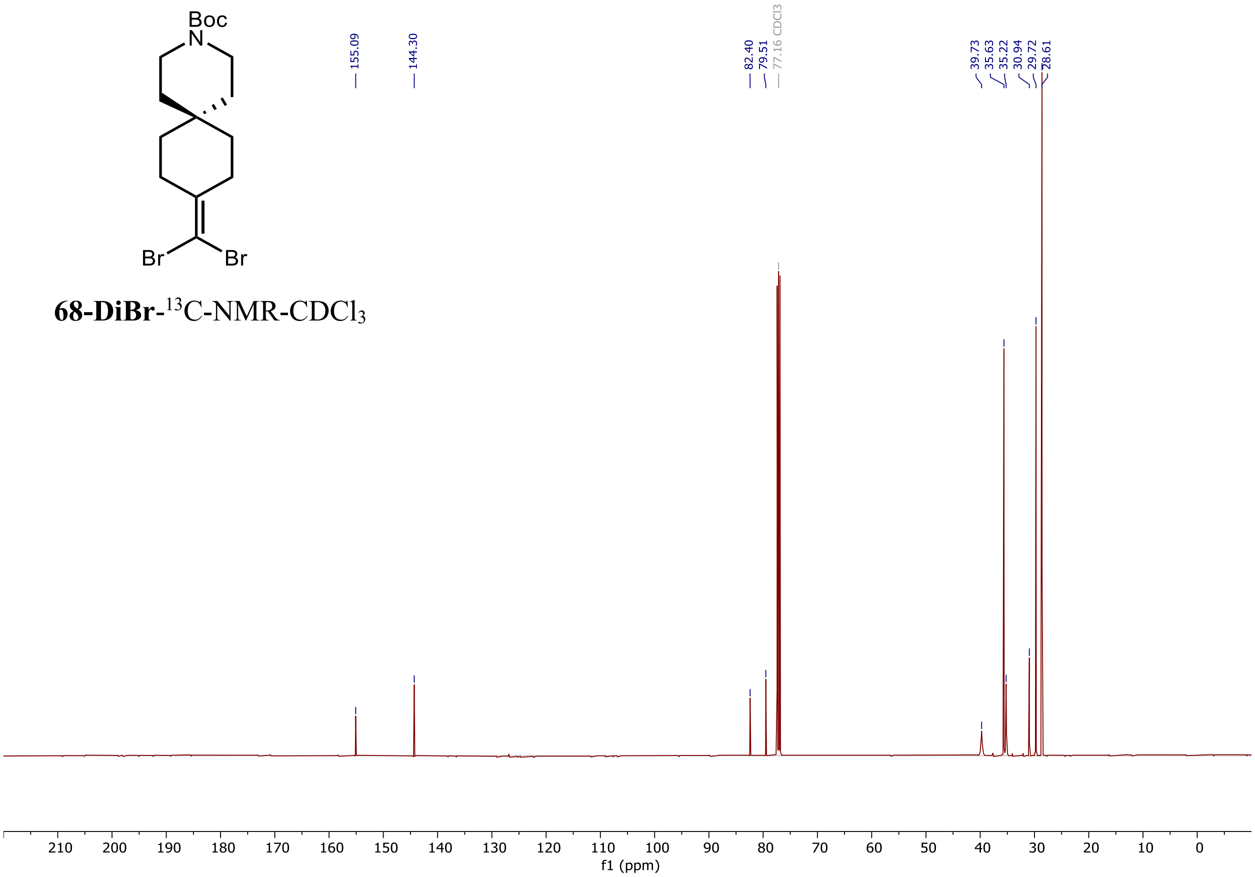

68-DiBr-^{13}C-NMR-$CDCl_3$

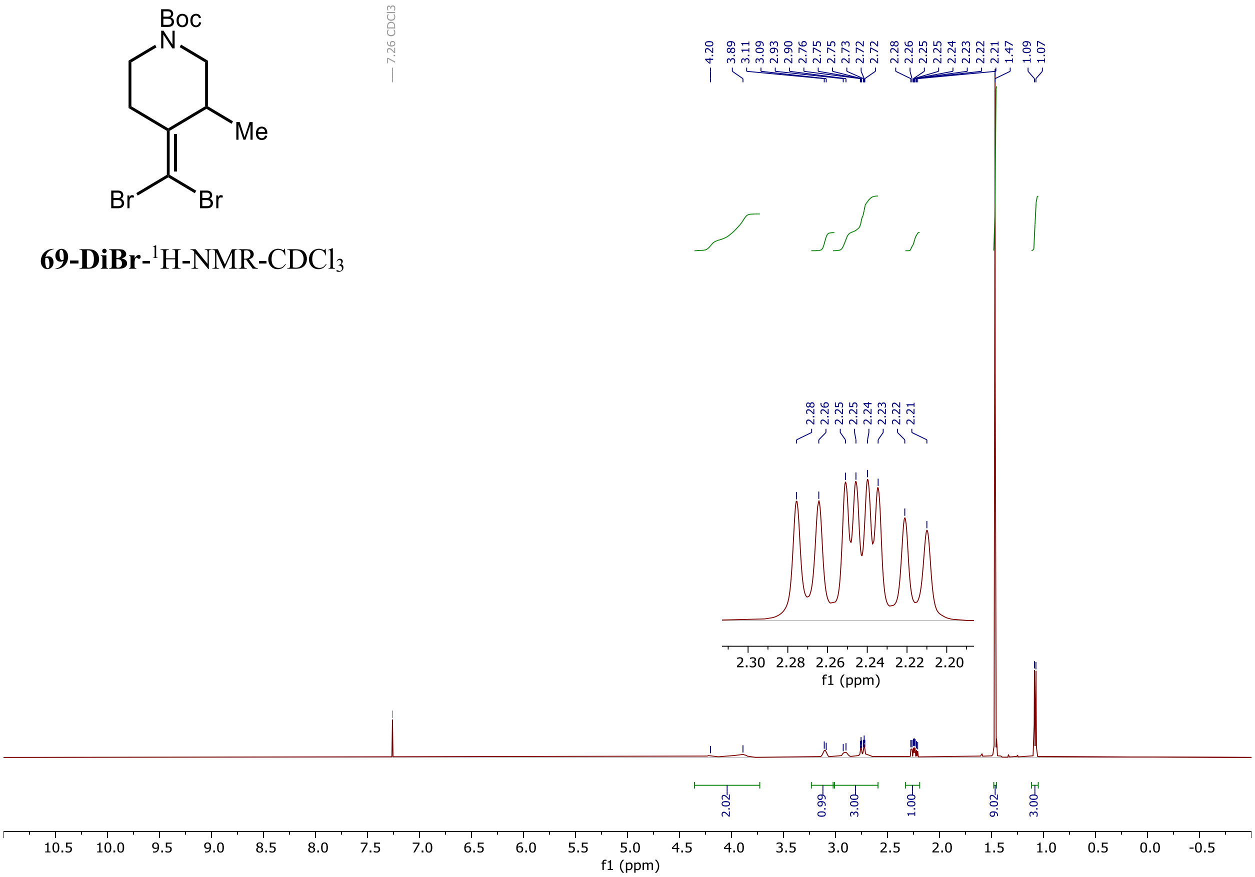

69-DiBr-^{1}H-NMR-$CDCl_3$

69-DiBr-^{13}C-NMR-CDCl$_3$

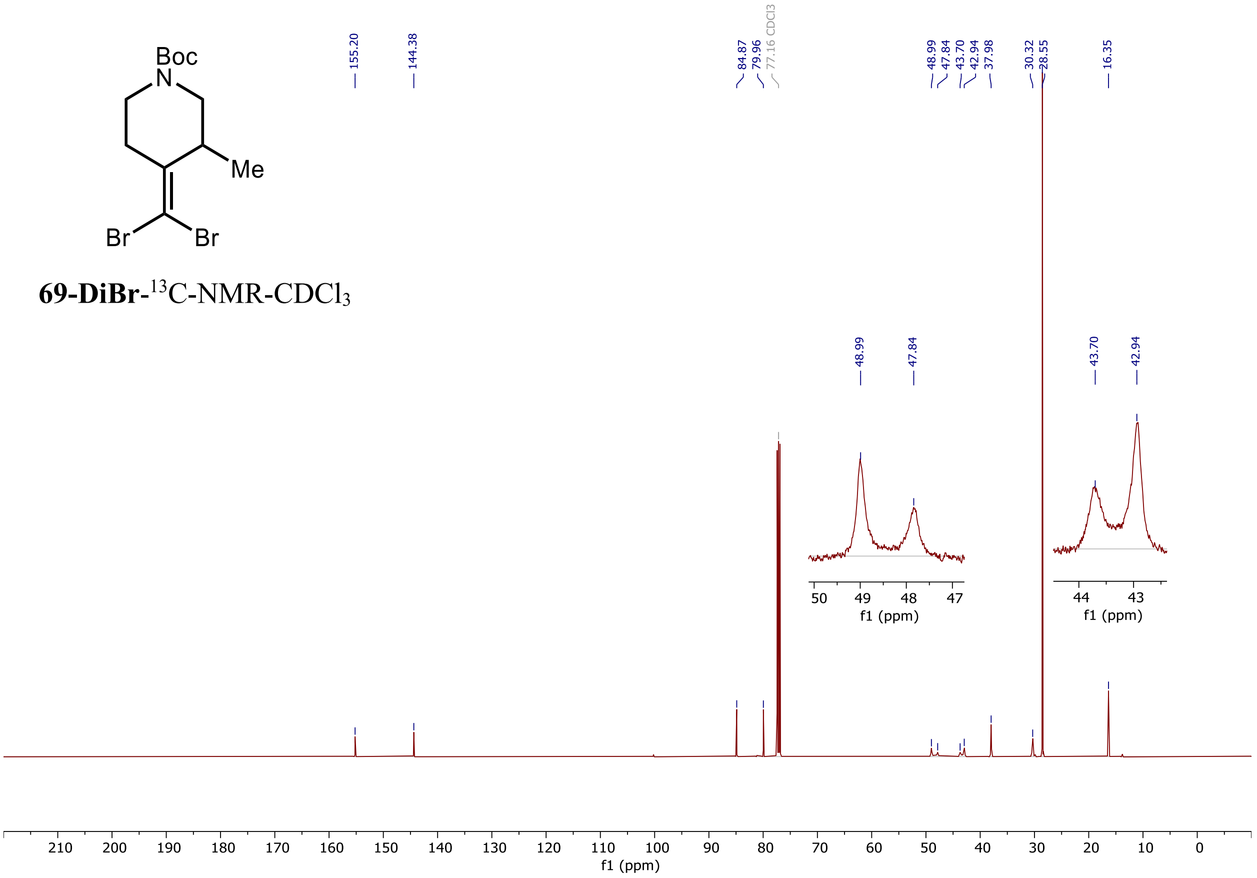

70-DiBr-^{1}H-NMR-CDCl$_3$

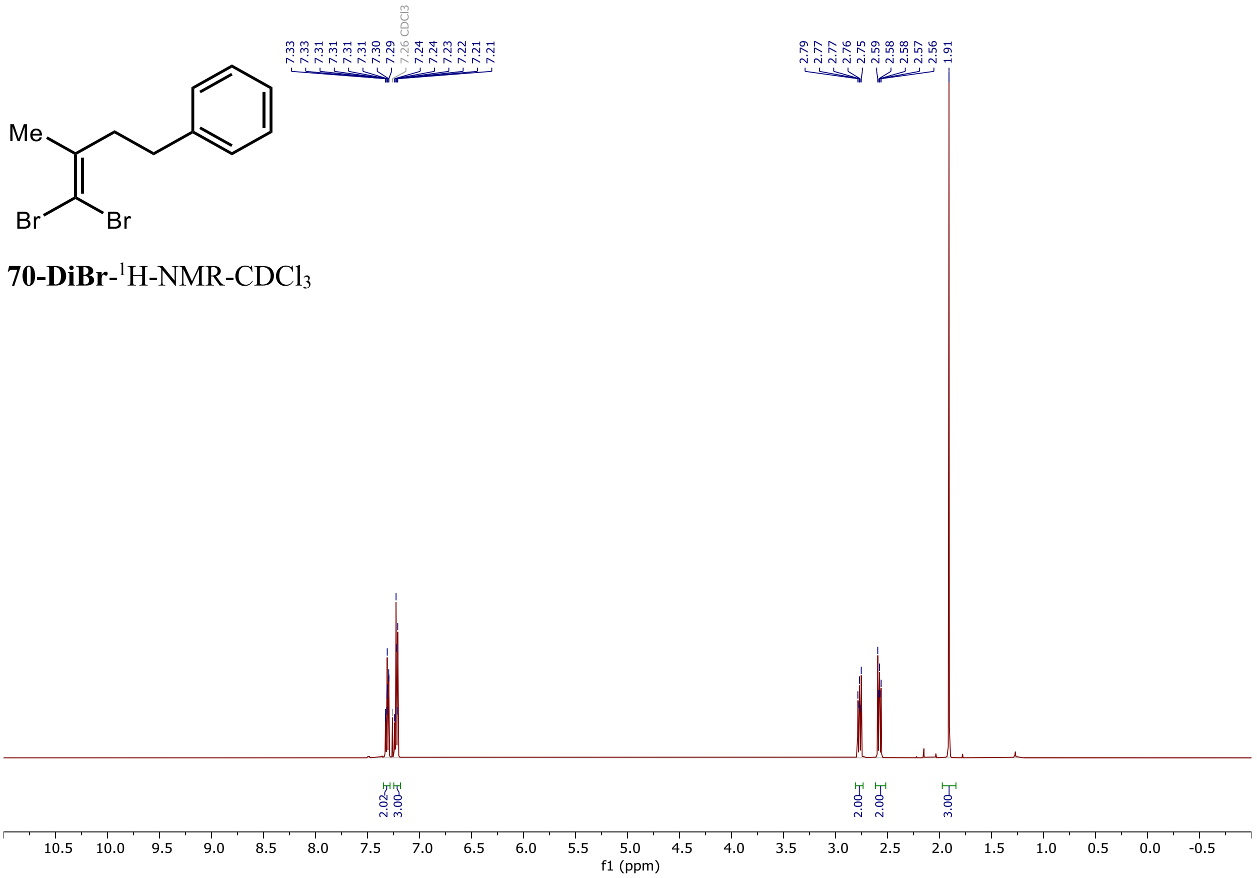

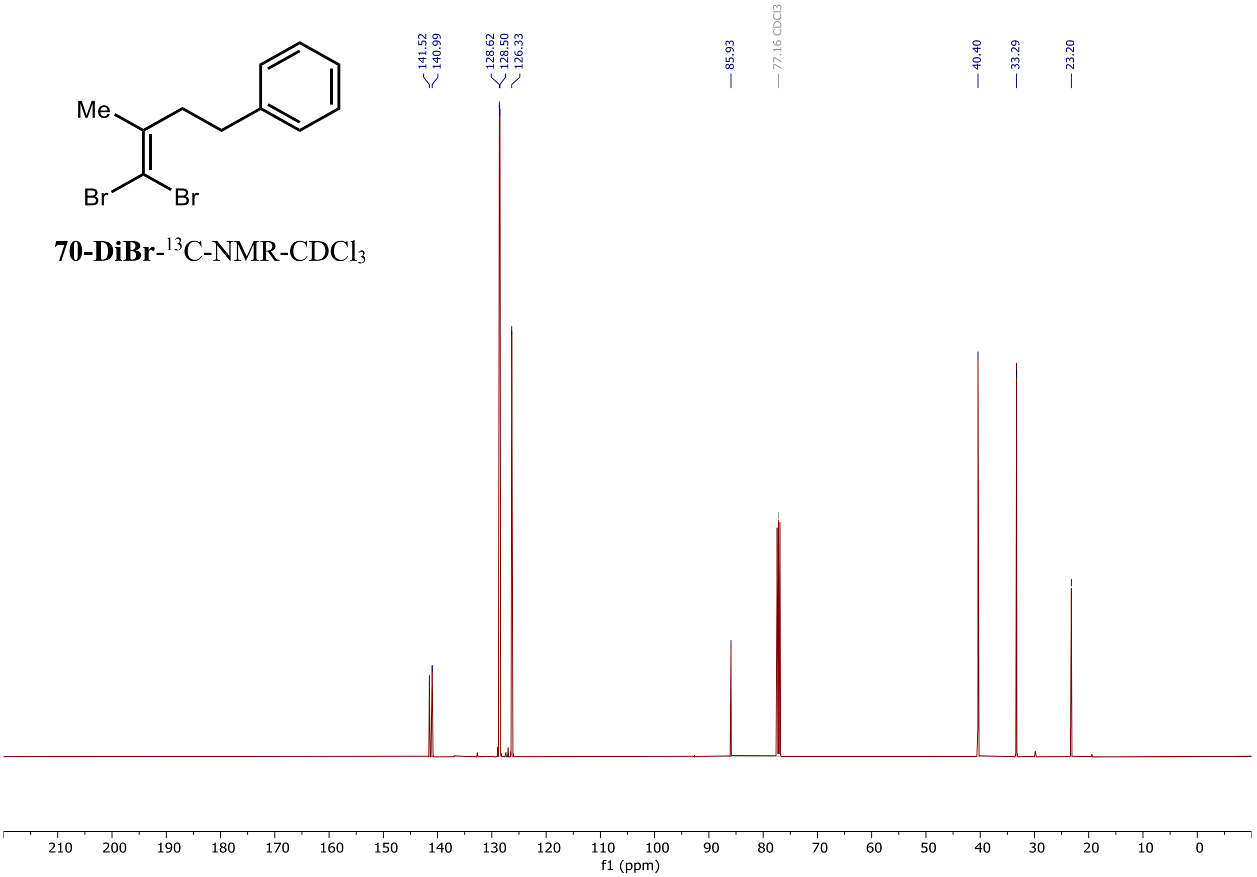

70-DiBr-^{13}C-NMR-CDCl$_3$

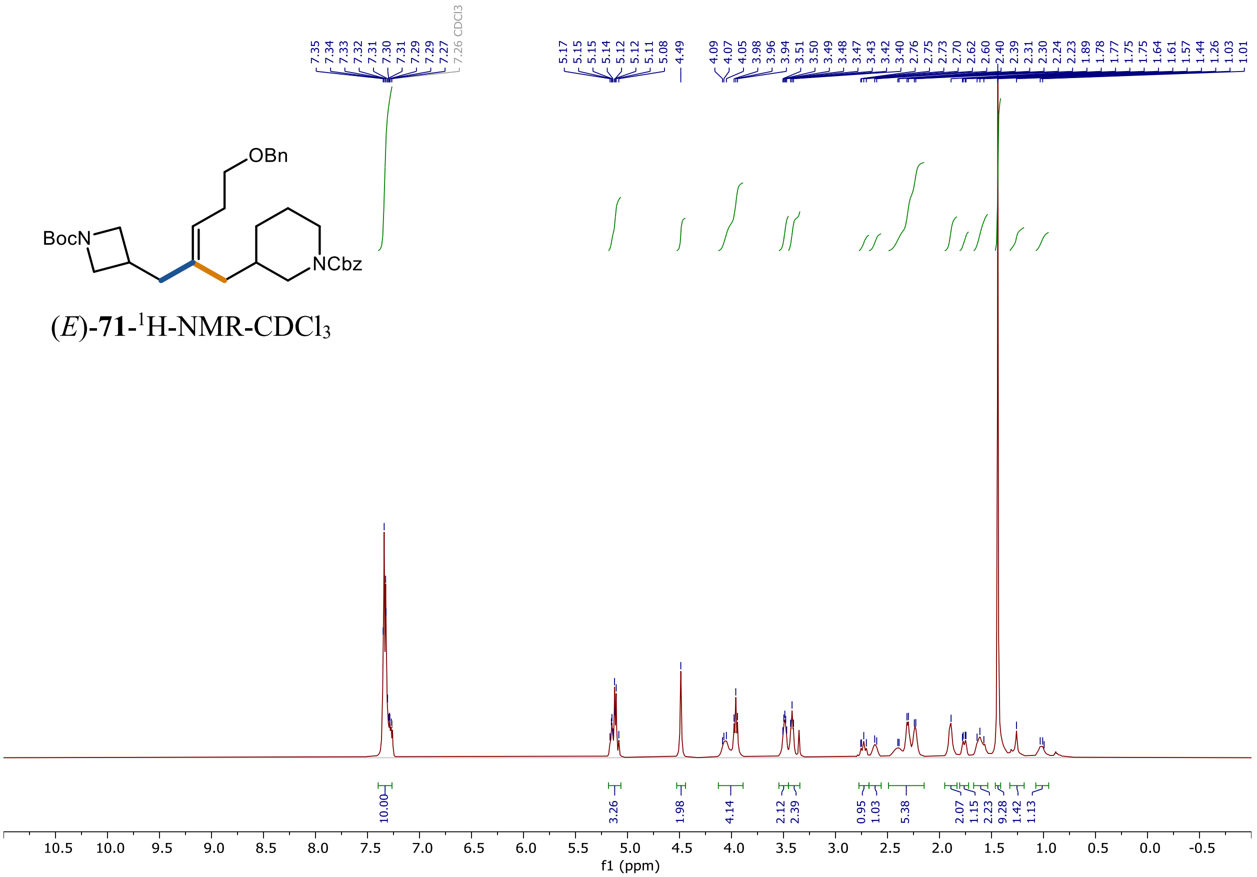

(*E*)-**71**-^{1}H-NMR-CDCl$_3$

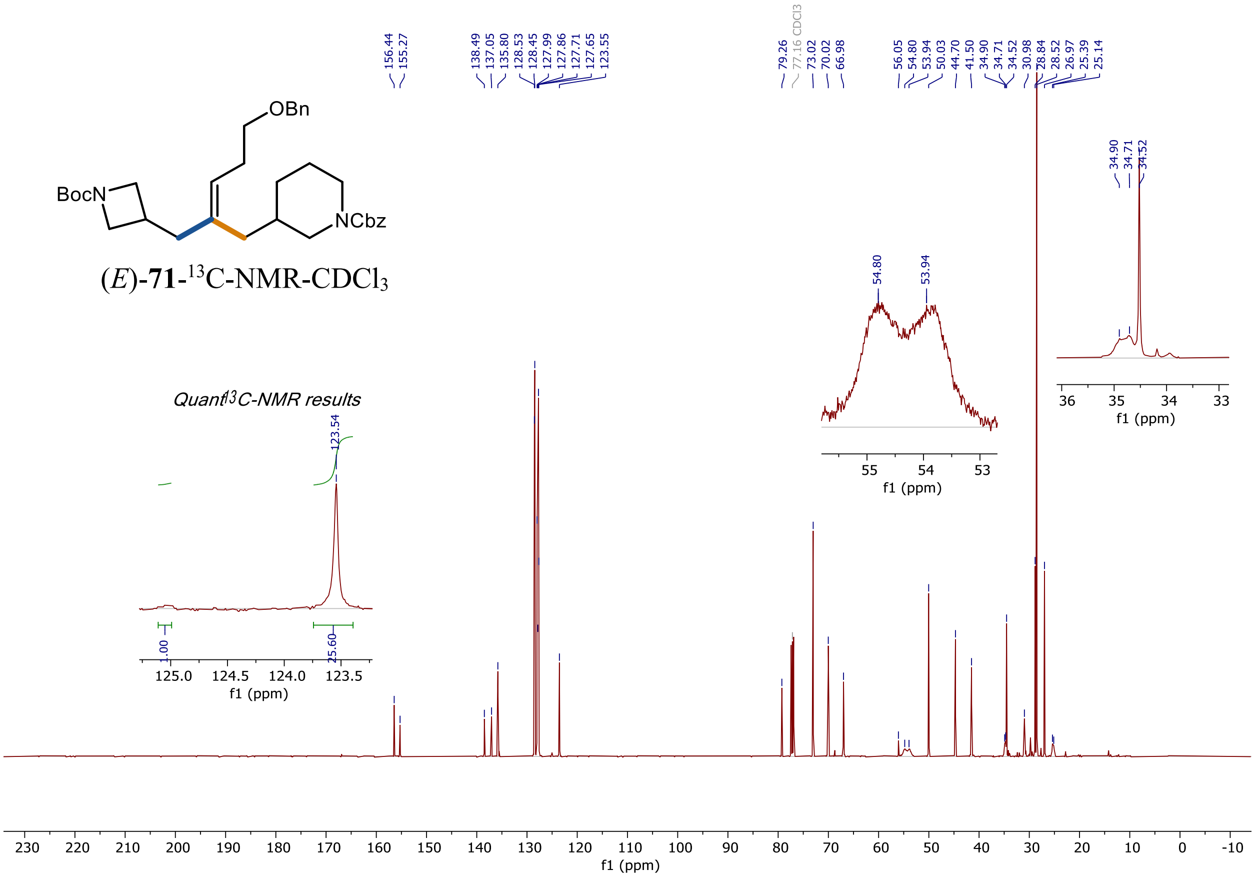
OBn
BocN
NCbz
(E)-71-13C-NMR-CDCl3
Quant13C-NMR results
f1 (ppm)

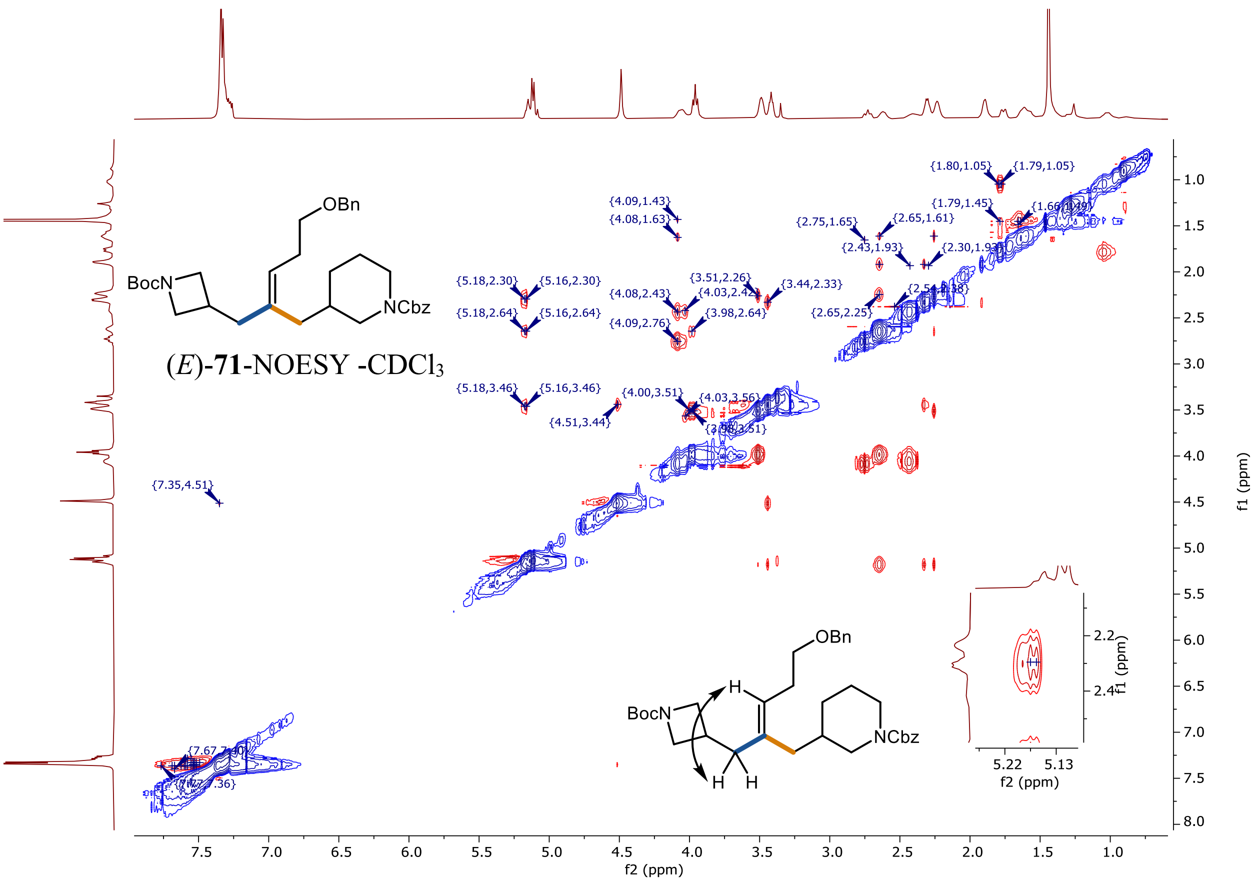
OBn
BocN
NCbz
(E)-71-NOESY -CDCl3
f2 (ppm)
f1 (ppm)

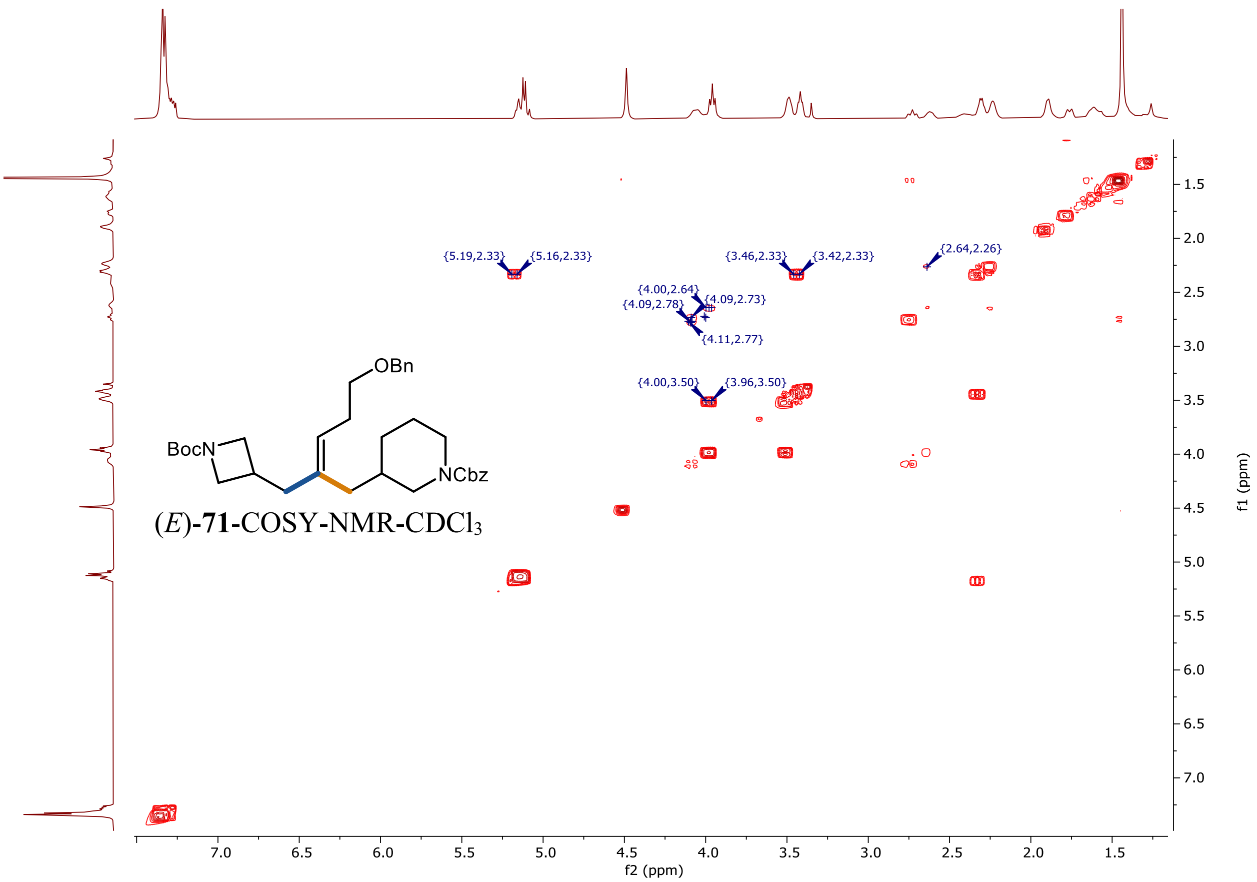
OBn
BocN
NCbz
(E)-71-COSY-NMR-CDCl3
{5.19,2.33}
{5.16,2.33}
{3.46,2.33}
{3.42,2.33}
{2.64,2.26}
{4.00,2.64}
{4.09,2.73}
{4.09,2.78}
{4.11,2.77}
{4.00,3.50}
{3.96,3.50}
f2 (ppm)
f1 (ppm)

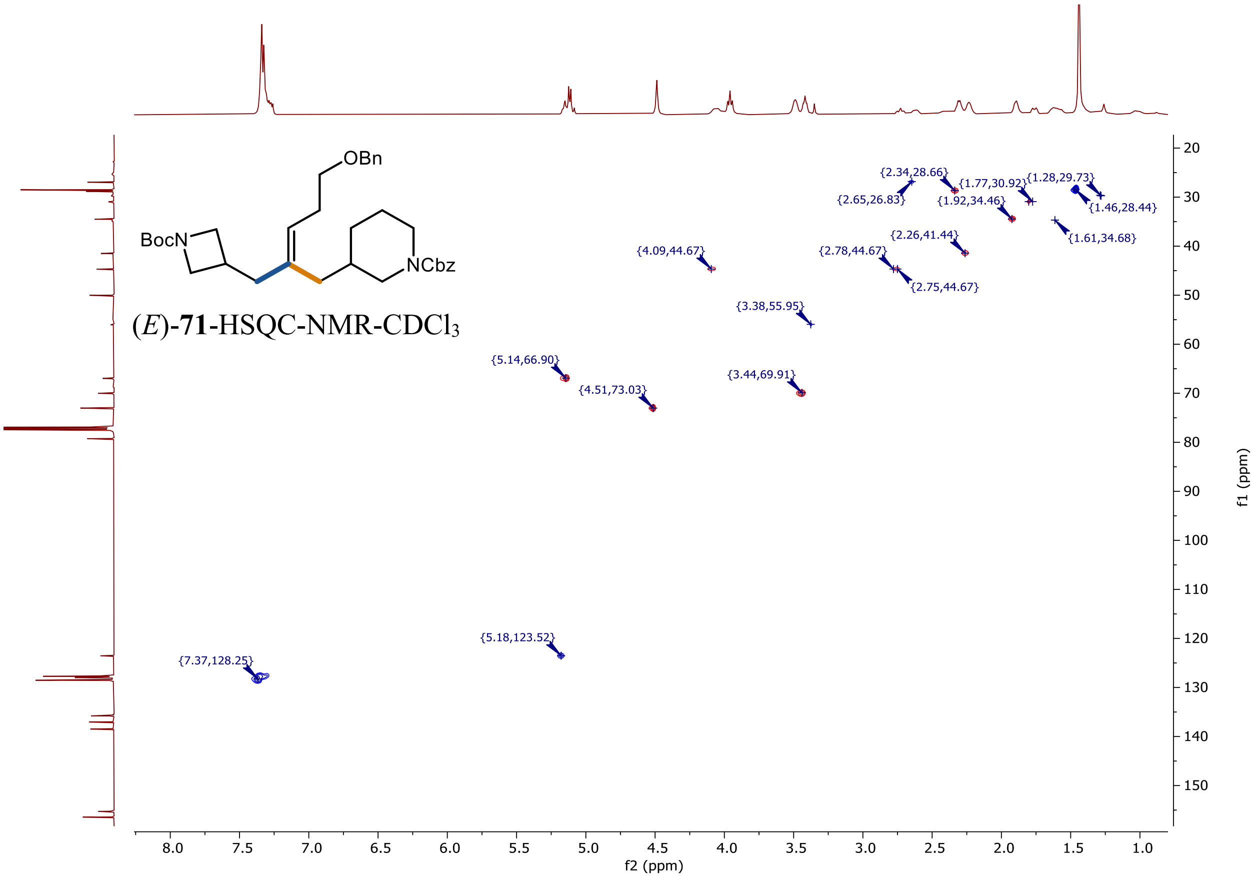
OBn
BocN
NCbz
(E)-71-HSQC-NMR-CDCl3
{2.34,28.66}
{1.77,30.92}
{1.28,29.73}
{2.65,26.83}
{1.92,34.46}
{1.46,28.44}
{2.26,41.44}
{1.61,34.68}
{4.09,44.67}
{2.78,44.67}
{2.75,44.67}
{3.38,55.95}
{5.14,66.90}
{3.44,69.91}
{4.51,73.03}
{5.18,123.52}
{7.37,128.25}
f2 (ppm)
f1 (ppm)

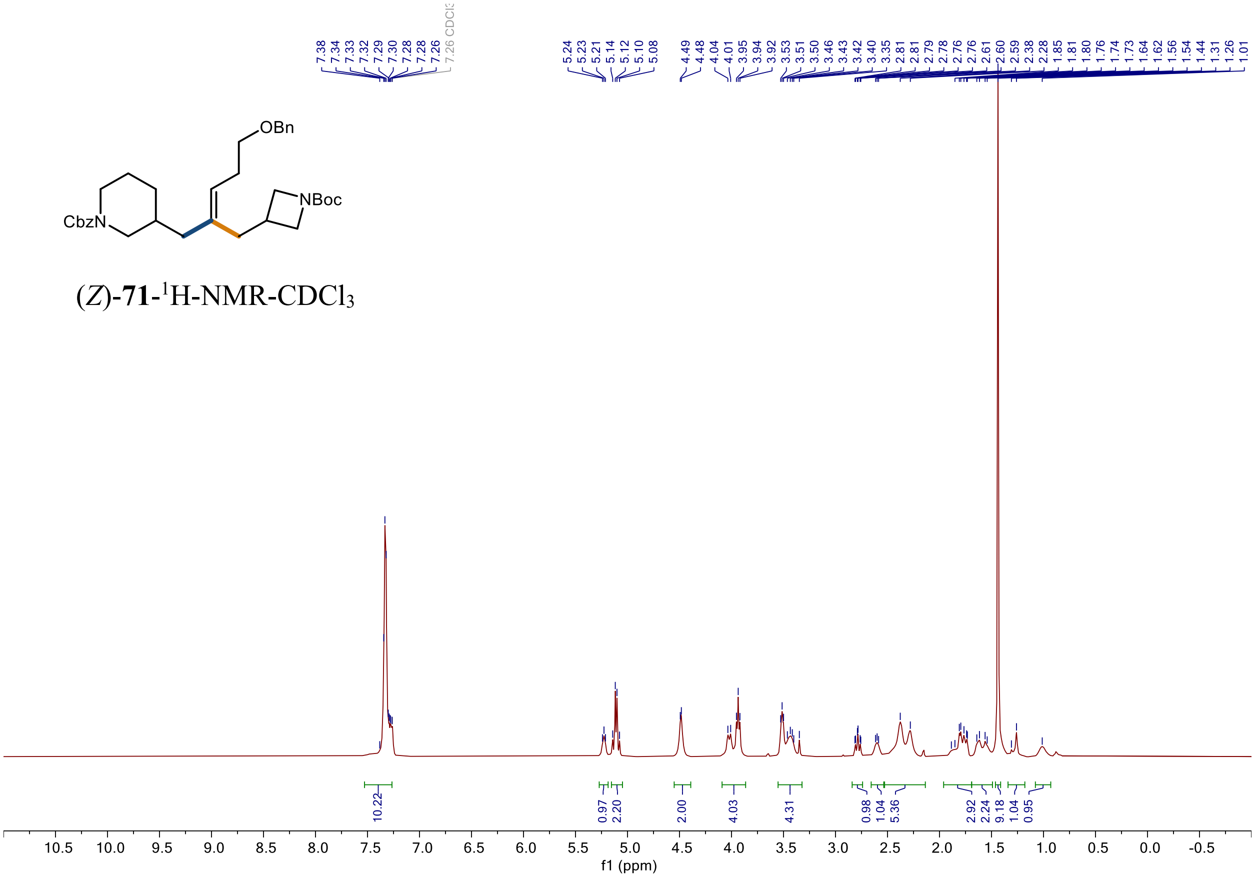

(*Z*)-**71**-^{1}H-NMR-CDCl$_3$

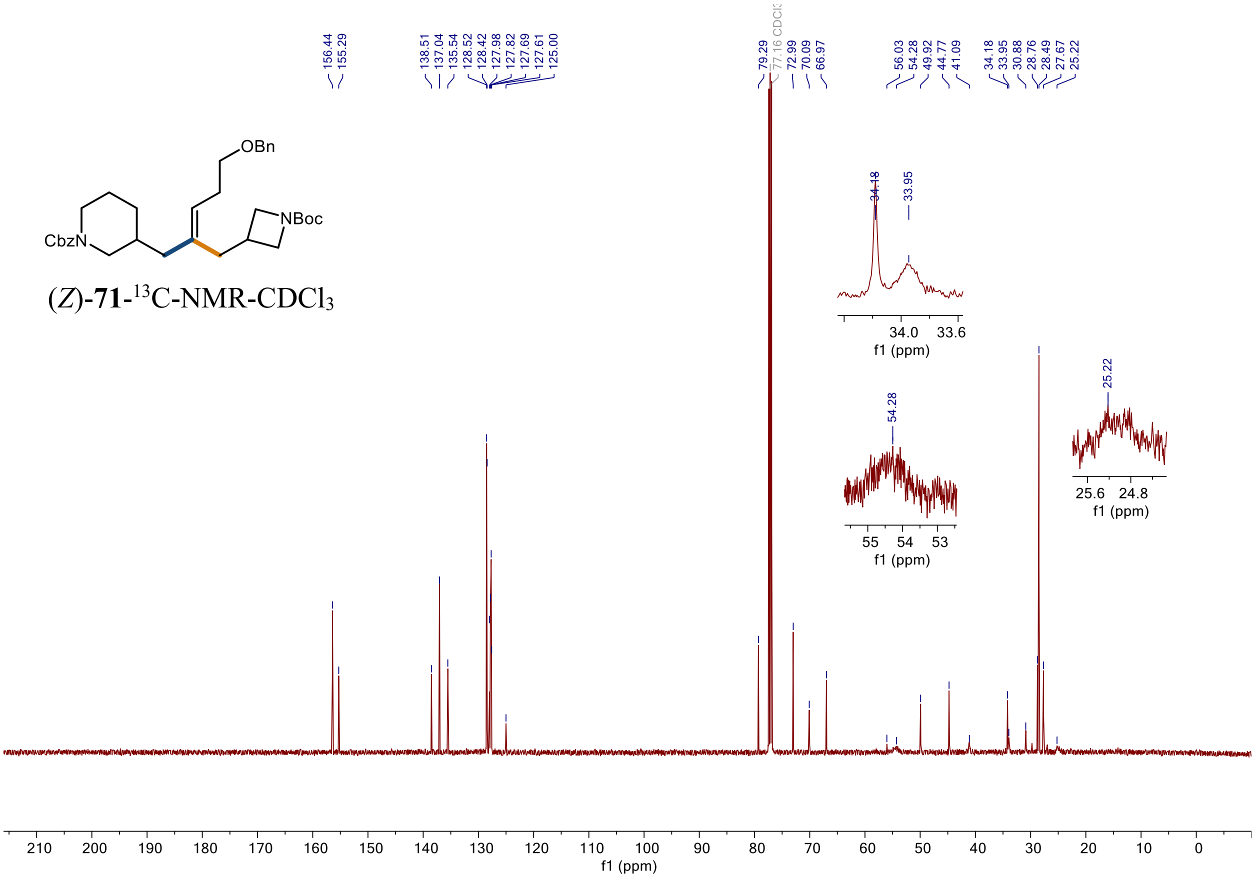

(*Z*)-**71**-^{13}C-NMR-CDCl$_3$

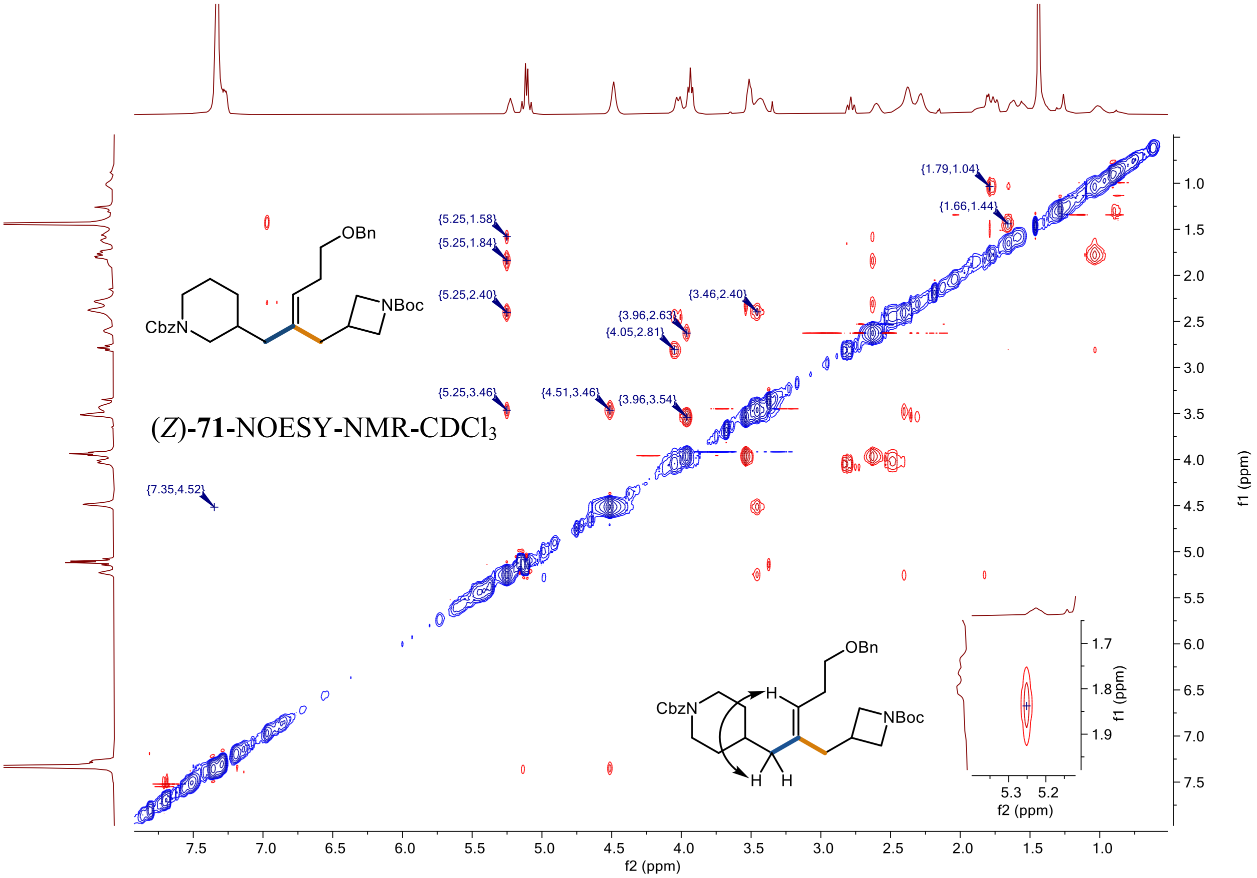

OBn
CbzN
NBoc
(Z)-71-NOESY-NMR-CDCl3
{1.79,1.04}
{1.66,1.44}
{5.25,1.58}
{5.25,1.84}
{5.25,2.40}
{3.46,2.40}
{3.96,2.63}
{4.05,2.81}
{5.25,3.46}
{4.51,3.46}
{3.96,3.54}
{7.35,4.52}
H
H H
f2 (ppm)
f1 (ppm)

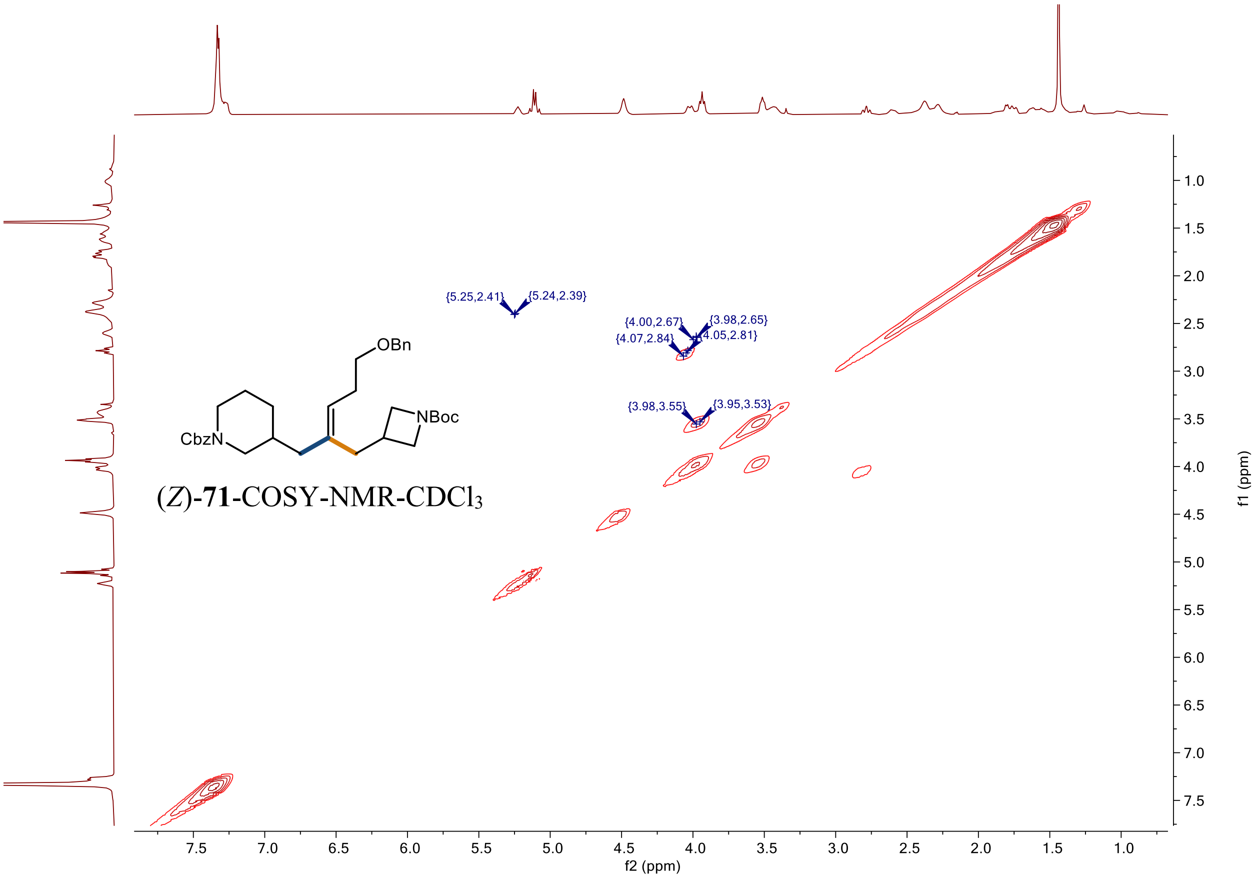

OBn
CbzN
NBoc
(Z)-71-COSY-NMR-CDCl3
{5.25,2.41}
{5.24,2.39}
{4.00,2.67}
{3.98,2.65}
{4.07,2.84}
{4.05,2.81}
{3.98,3.55}
{3.95,3.53}
f2 (ppm)
f1 (ppm)

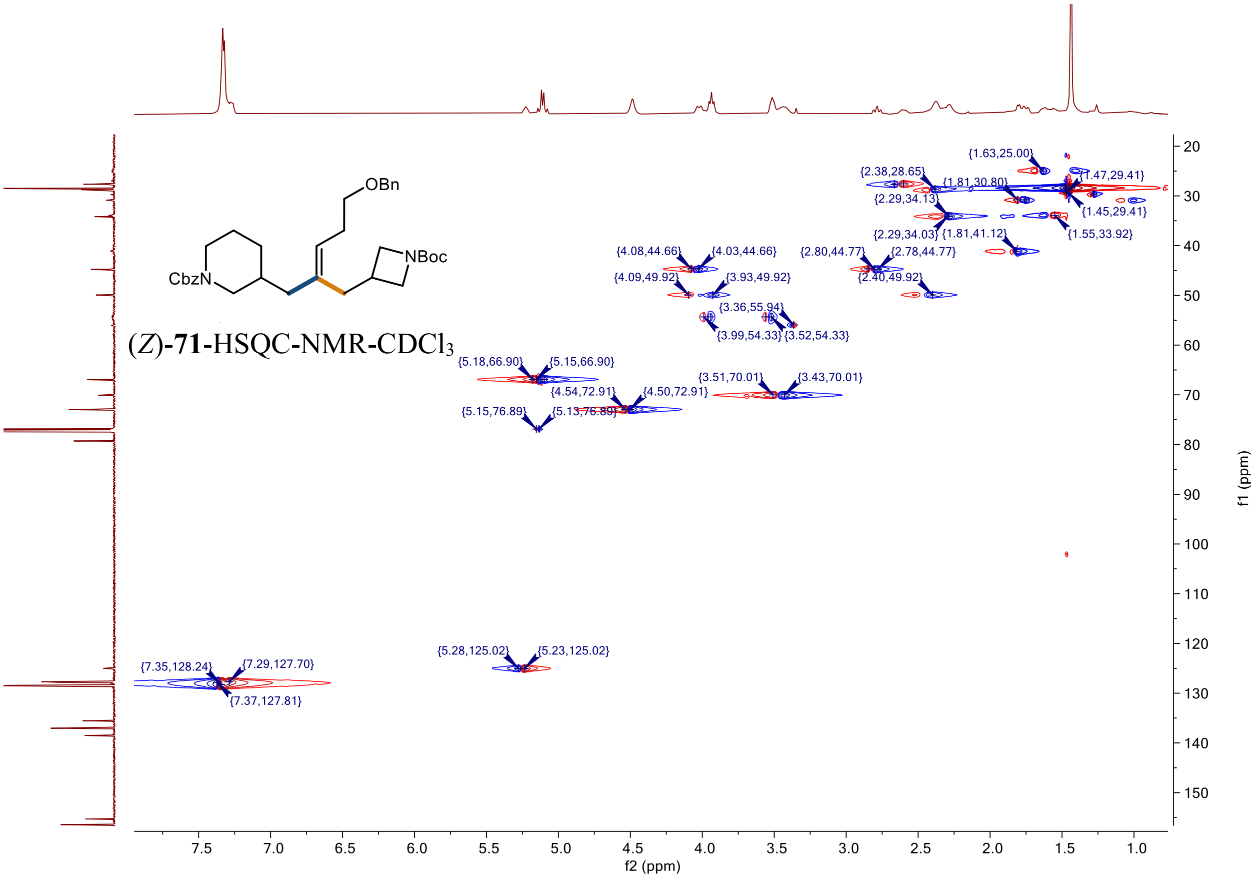

(*Z*)-**71**-HSQC-NMR-$CDCl_3$

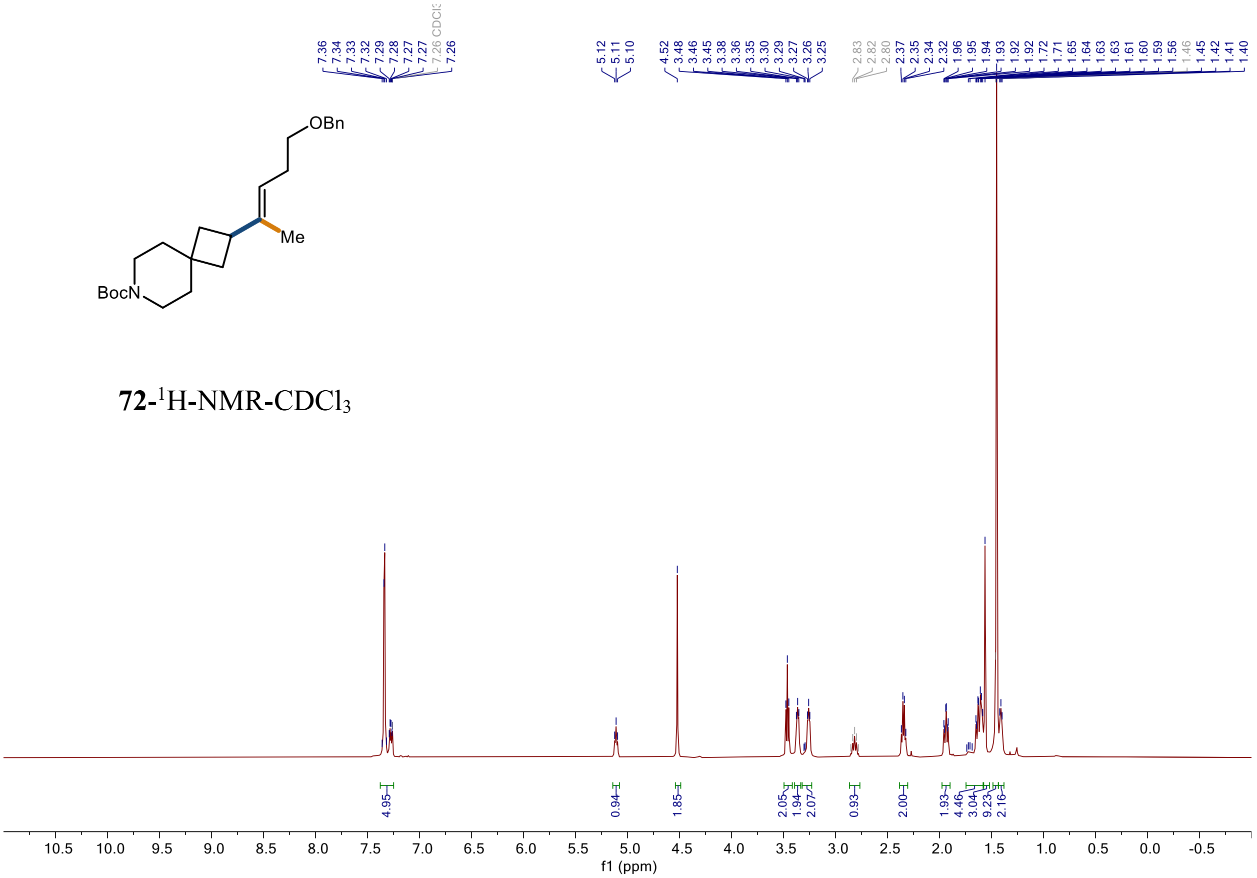

72-^{1}H-NMR-$CDCl_3$

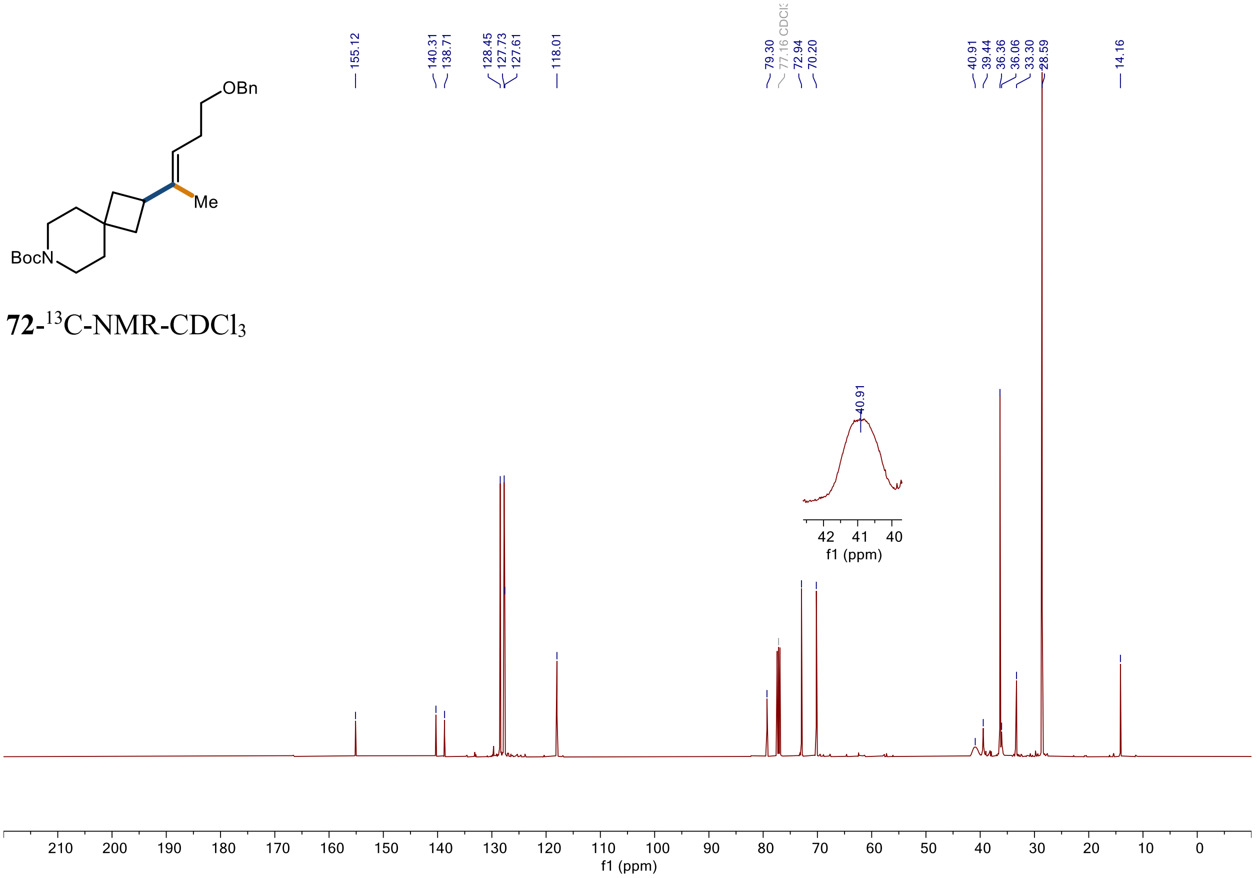

72-^{13}C-NMR-CDCl$_3$

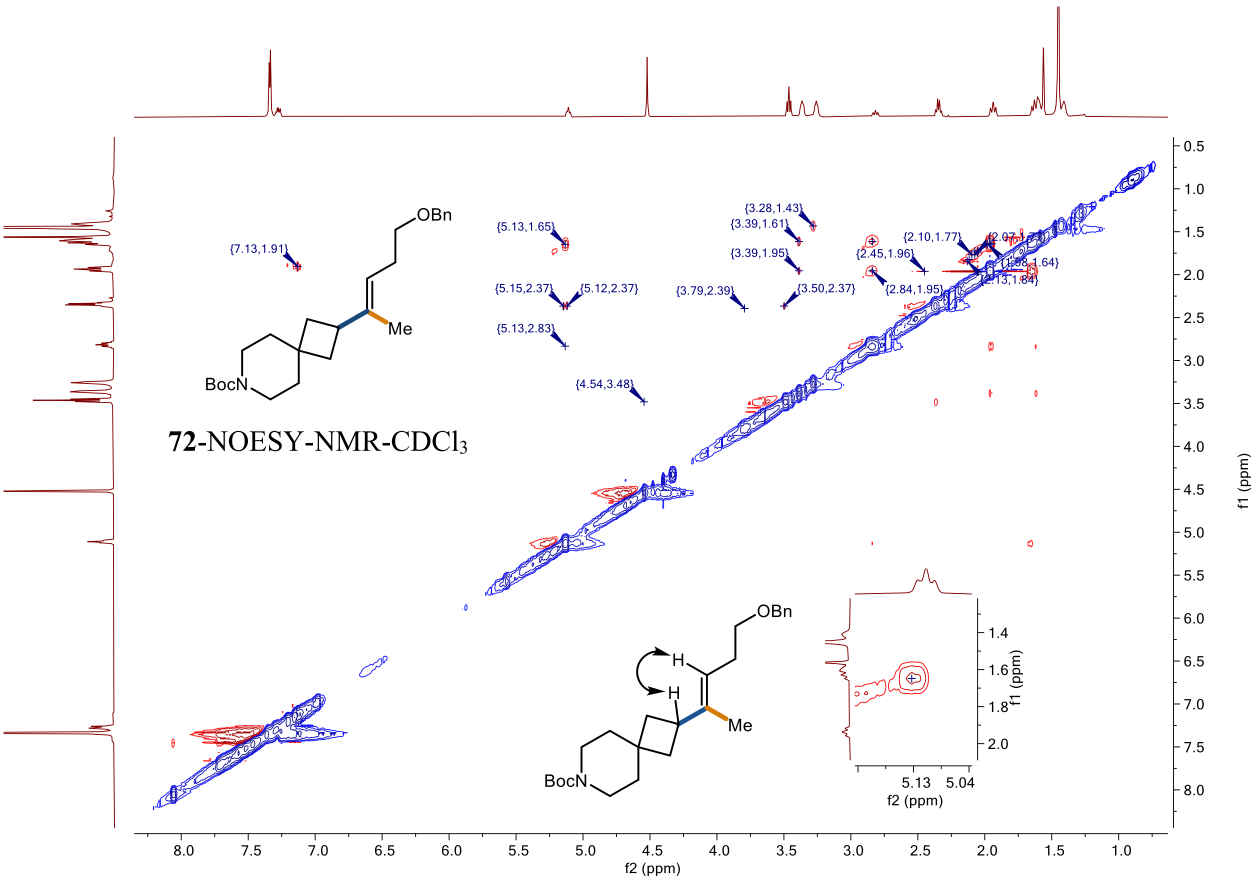

72-NOESY-NMR-CDCl$_3$

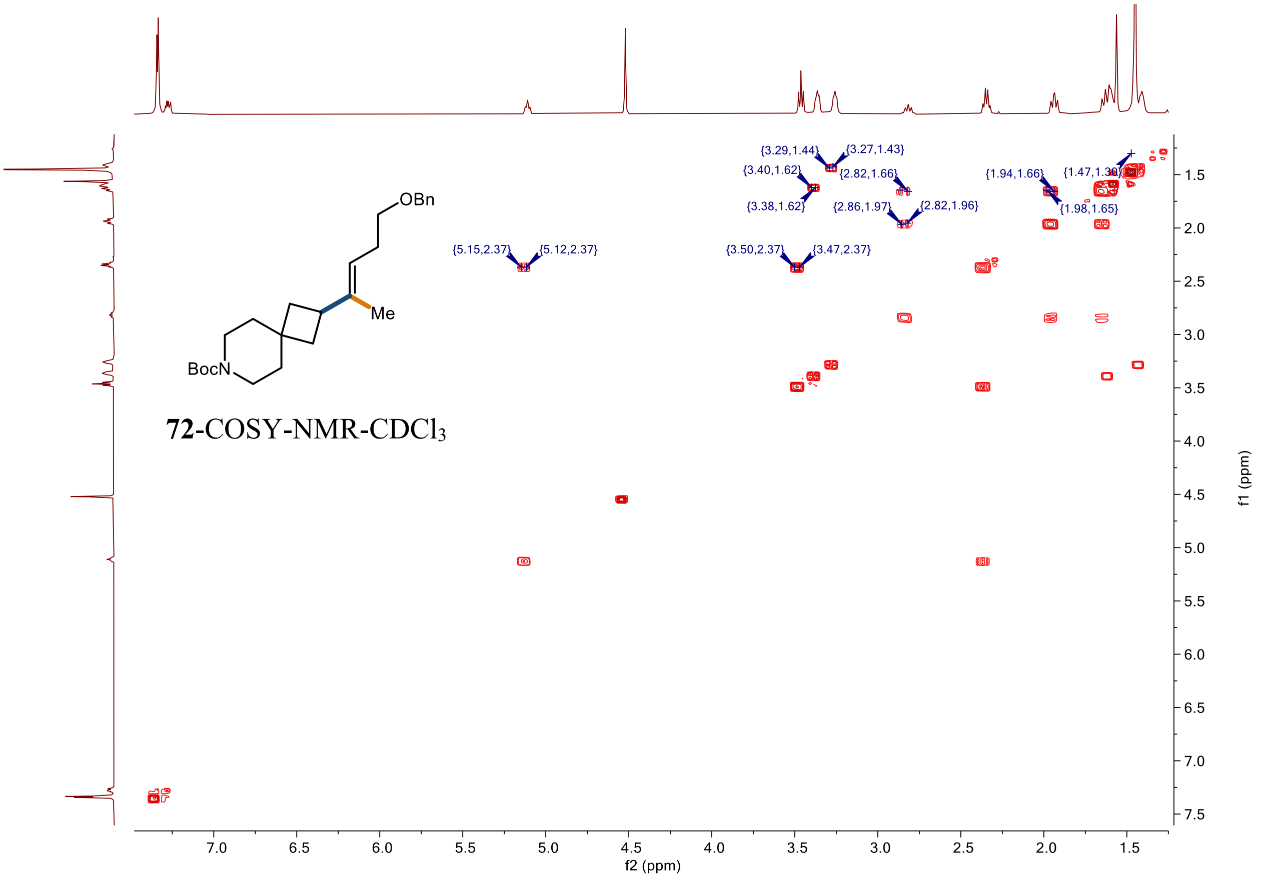

OBn
Me
BocN
72-COSY-NMR-CDCl3
f2 (ppm)
f1 (ppm)

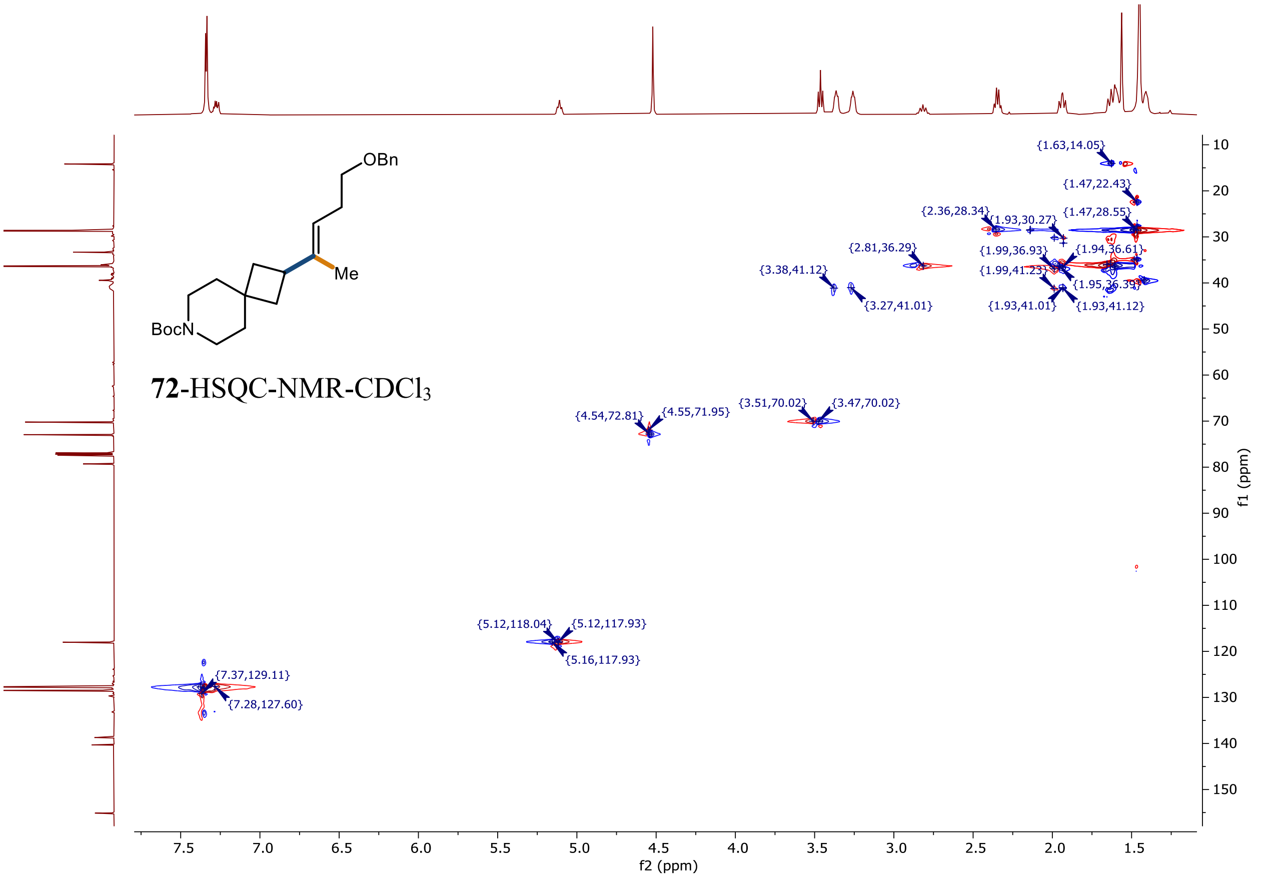

OBn
Me
BocN
72-HSQC-NMR-CDCl3
f2 (ppm)
f1 (ppm)

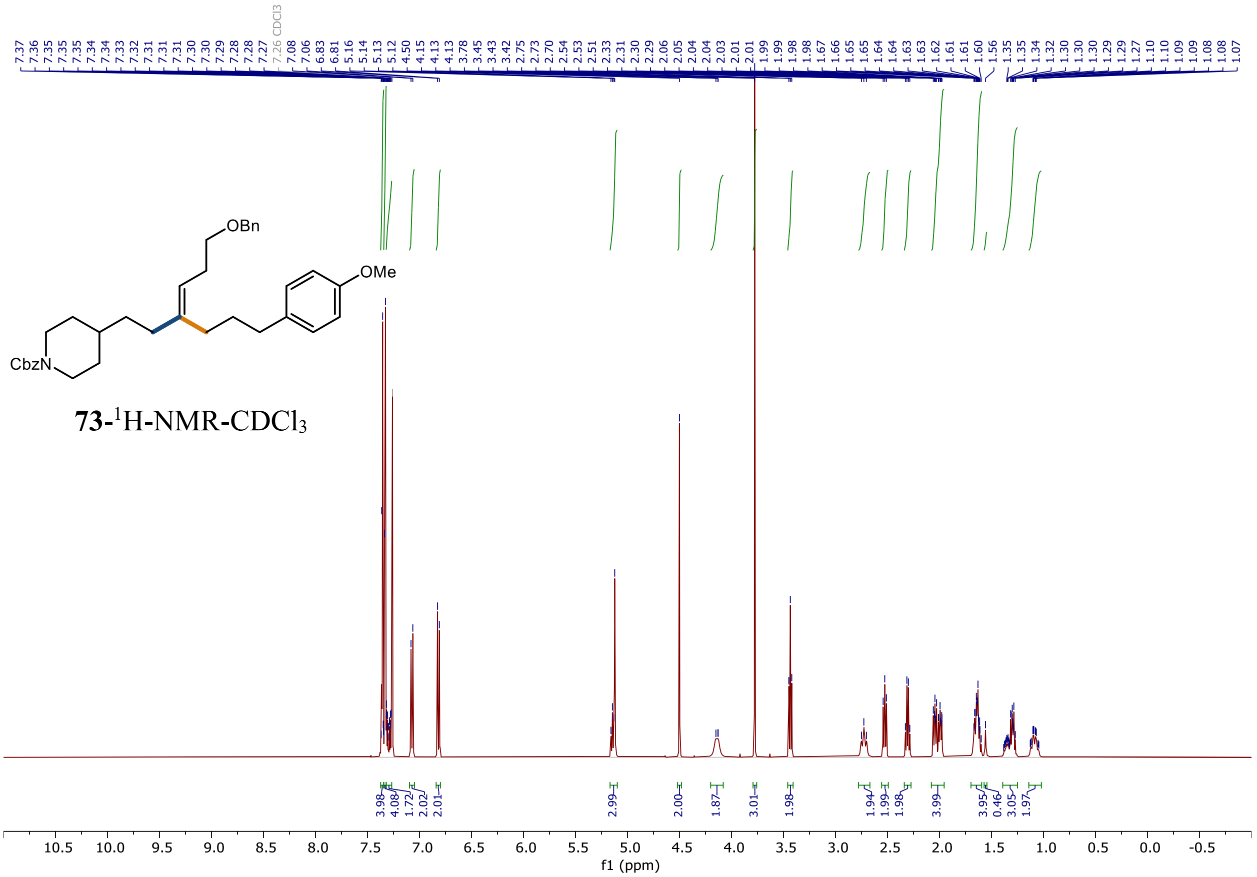
7.37 7.36 7.35 7.35 7.34 7.34 7.33 7.32 7.31 7.31 7.31 7.30 7.30 7.29 7.28 7.28 7.27 7.26 CDCl3 7.08 7.06 6.83 6.81 5.16 5.14 5.13 5.12 4.50 4.15 4.13 4.13 3.78 3.45 3.43 3.42 2.75 2.73 2.70 2.54 2.53 2.51 2.33 2.31 2.30 2.29 2.06 2.05 2.04 2.04 2.03 2.01 2.01 1.99 1.99 1.98 1.98 1.67 1.66 1.65 1.65 1.64 1.64 1.63 1.63 1.62 1.61 1.61 1.60 1.56 1.35 1.35 1.34 1.32 1.30 1.30 1.29 1.29 1.27 1.10 1.10 1.09 1.09 1.08 1.08 1.07
OBn
OMe
CbzN
73-¹H-NMR-CDCl3
3.98 4.08 1.72 2.02 2.01 2.99 2.00 1.87 3.01 1.98 1.94 1.99 1.98 3.99 3.95 0.46 3.05 1.97
10.5 10.0 9.5 9.0 8.5 8.0 7.5 7.0 6.5 6.0 5.5 5.0 4.5 4.0 3.5 3.0 2.5 2.0 1.5 1.0 0.5 0.0 -0.5
f1 (ppm)

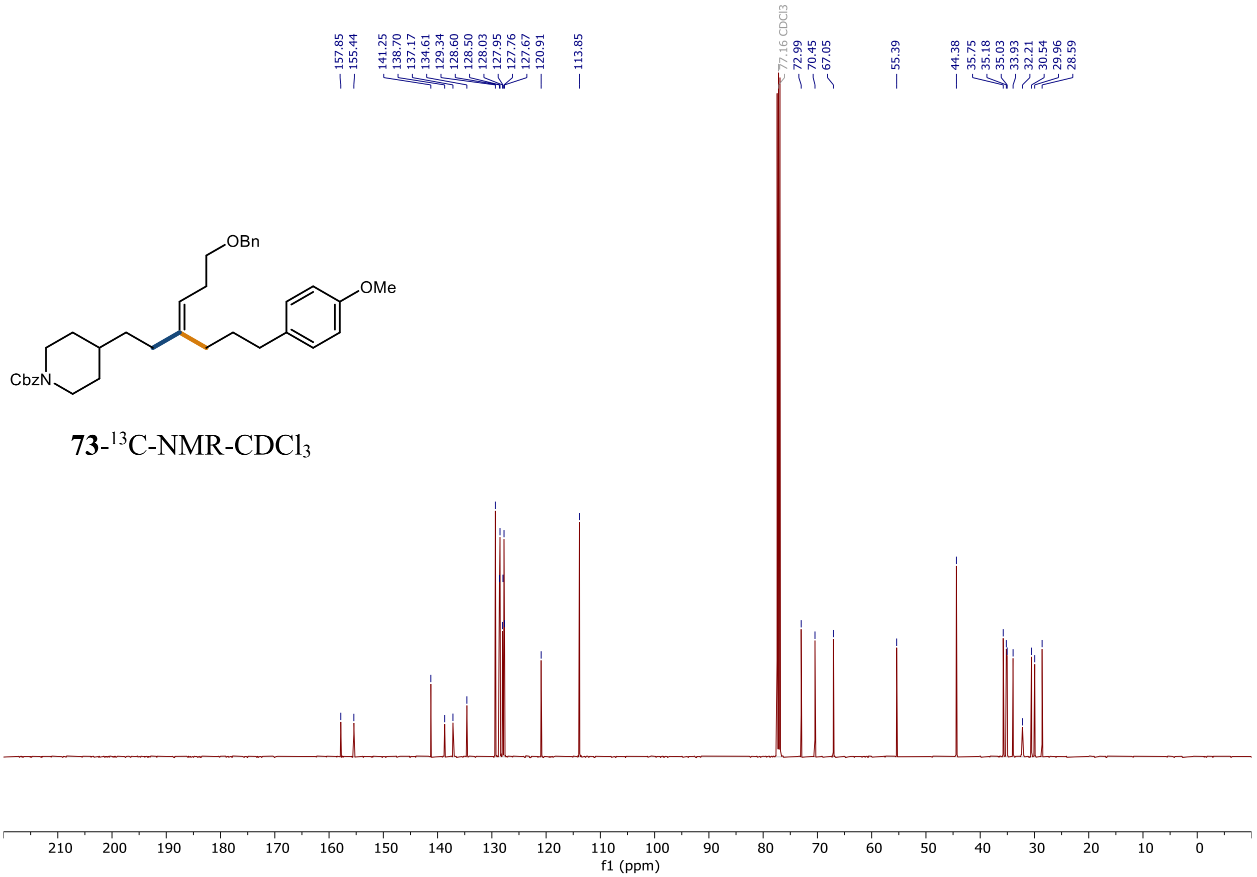
157.85 155.44 141.25 138.70 137.17 134.61 129.34 128.60 128.50 128.03 127.95 127.76 127.67 120.91 113.85 77.16 CDCl3 72.99 70.45 67.05 55.39 44.38 35.75 35.18 35.03 33.93 32.21 30.54 29.96 28.59
OBn
OMe
CbzN
73-¹³C-NMR-CDCl3
210 200 190 180 170 160 150 140 130 120 110 100 90 80 70 60 50 40 30 20 10 0
f1 (ppm)

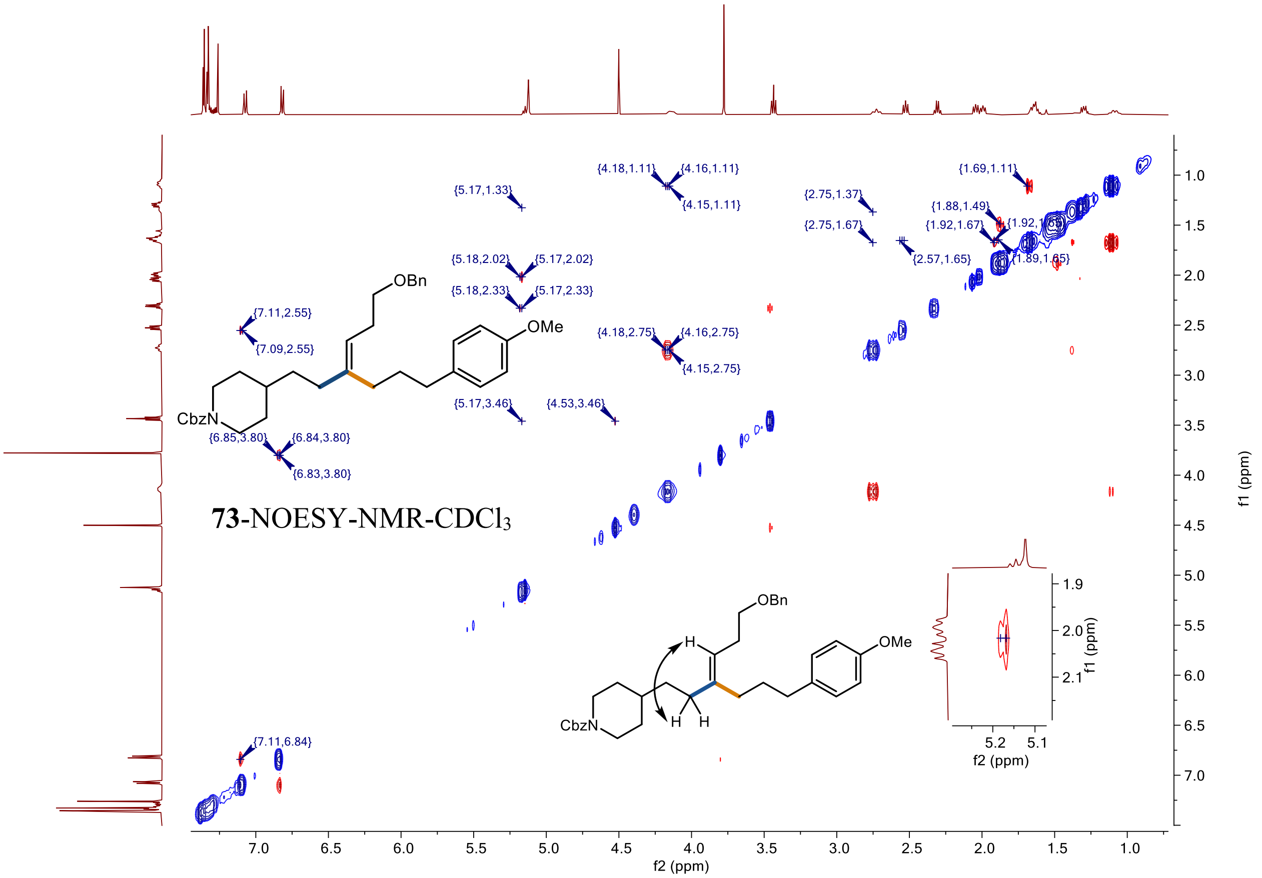
73-NOESY-NMR-CDCl3
OBn
OMe
CbzN
H
f2 (ppm)
f1 (ppm)

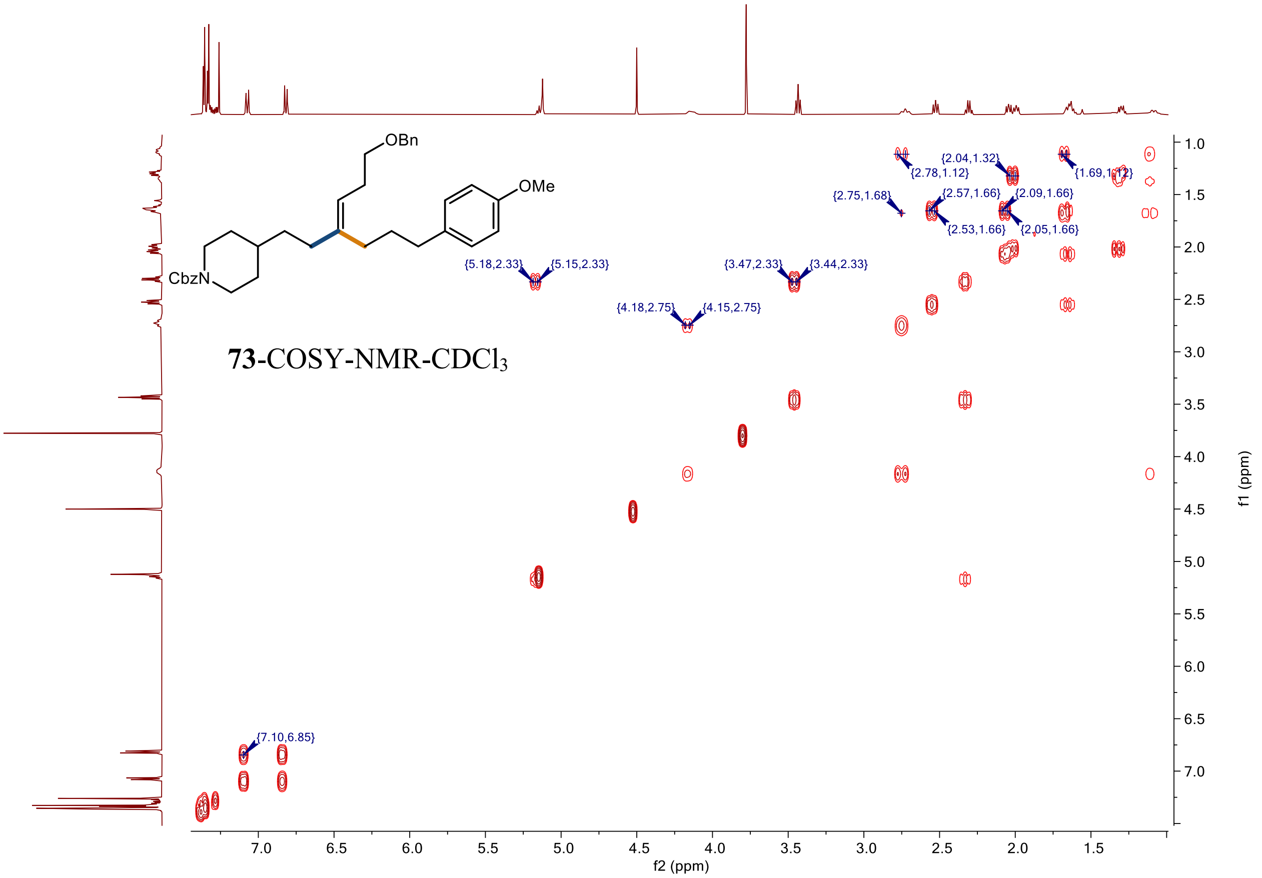
73-COSY-NMR-CDCl3
OBn
OMe
CbzN
f2 (ppm)
f1 (ppm)

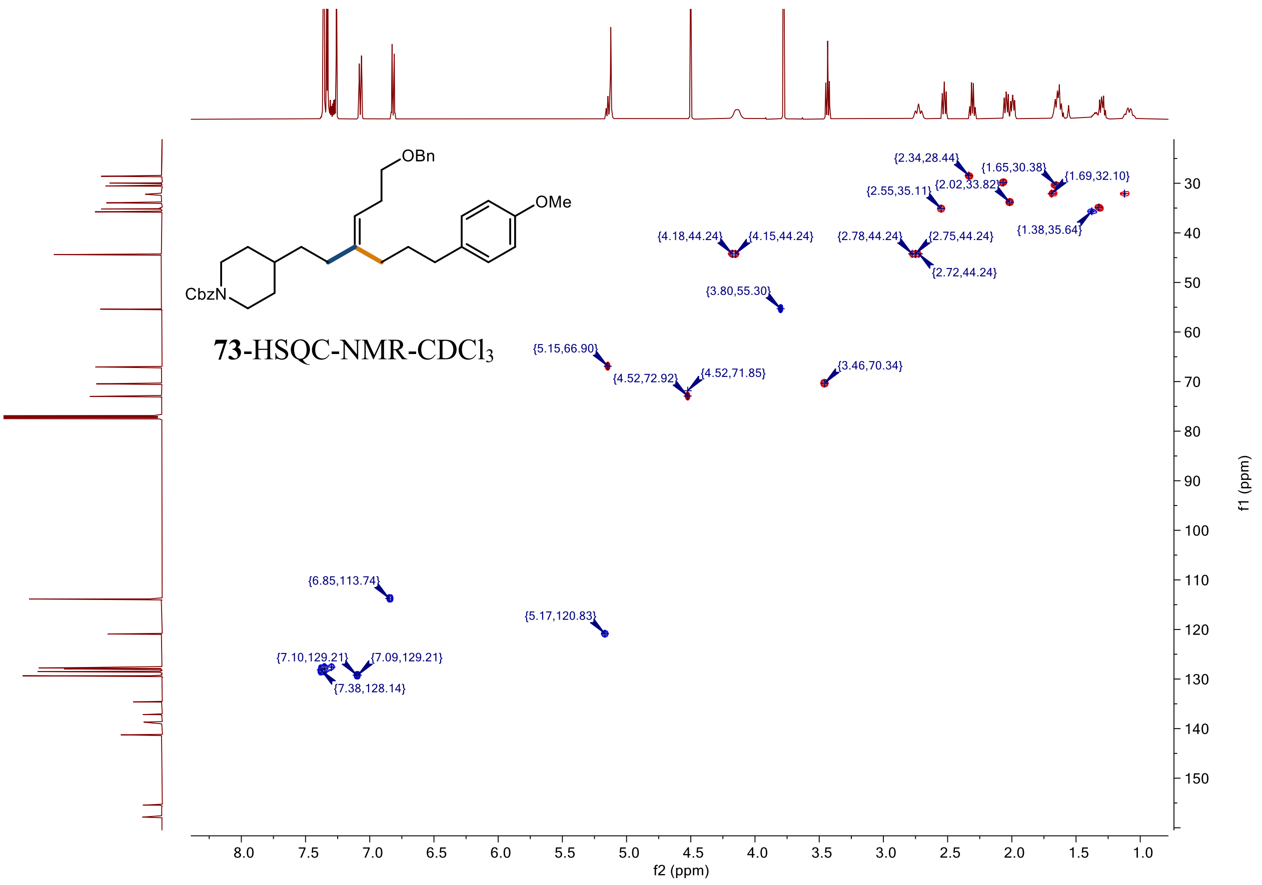
OBn
OMe
CbzN
73-HSQC-NMR-CDCl3
{2.34,28.44}
{1.65,30.38}
{1.69,32.10}
{2.02,33.82}
{2.55,35.11}
{1.38,35.64}
{4.18,44.24}
{4.15,44.24}
{2.78,44.24}
{2.75,44.24}
{2.72,44.24}
{3.80,55.30}
{5.15,66.90}
{4.52,72.92}
{4.52,71.85}
{3.46,70.34}
{6.85,113.74}
{5.17,120.83}
{7.10,129.21}
{7.09,129.21}
{7.38,128.14}
f2 (ppm)
f1 (ppm)

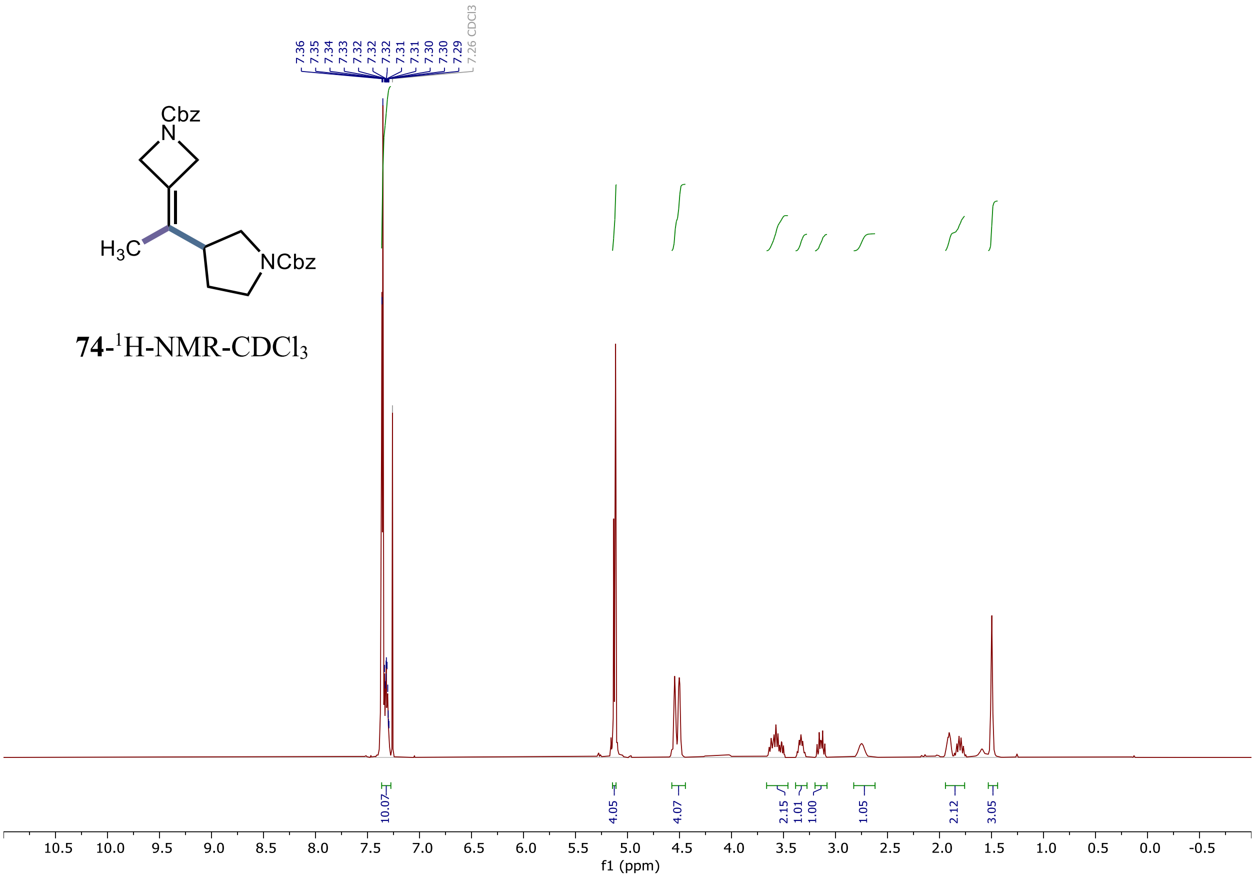
Cbz
N
H3C
NCbz
74-1H-NMR-CDCl3
7.36
7.35
7.34
7.33
7.32
7.32
7.32
7.31
7.31
7.30
7.29
7.26 CDCl3
10.07
4.05
4.07
2.15
1.01
1.00
1.05
2.12
3.05
f1 (ppm)

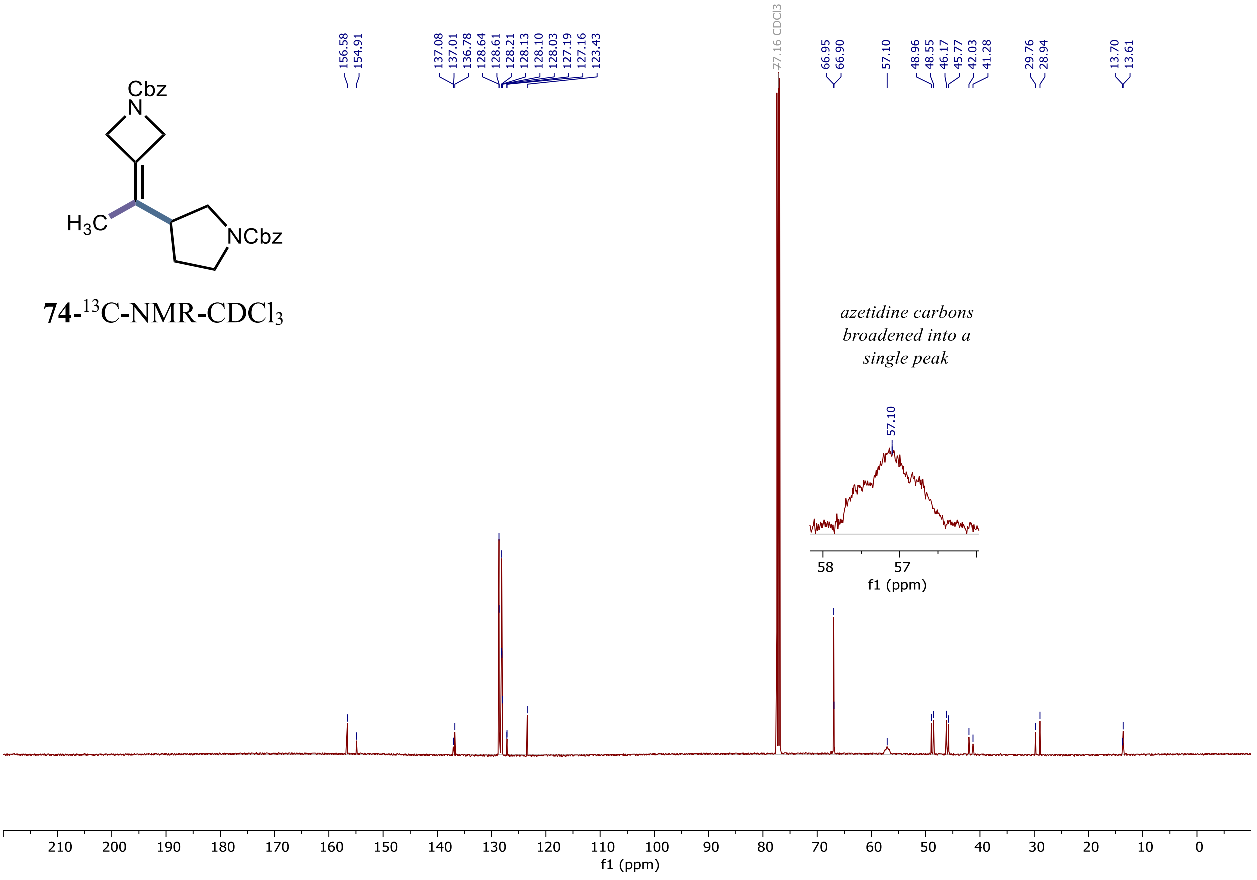

74-^{13}C-NMR-$CDCl_3$

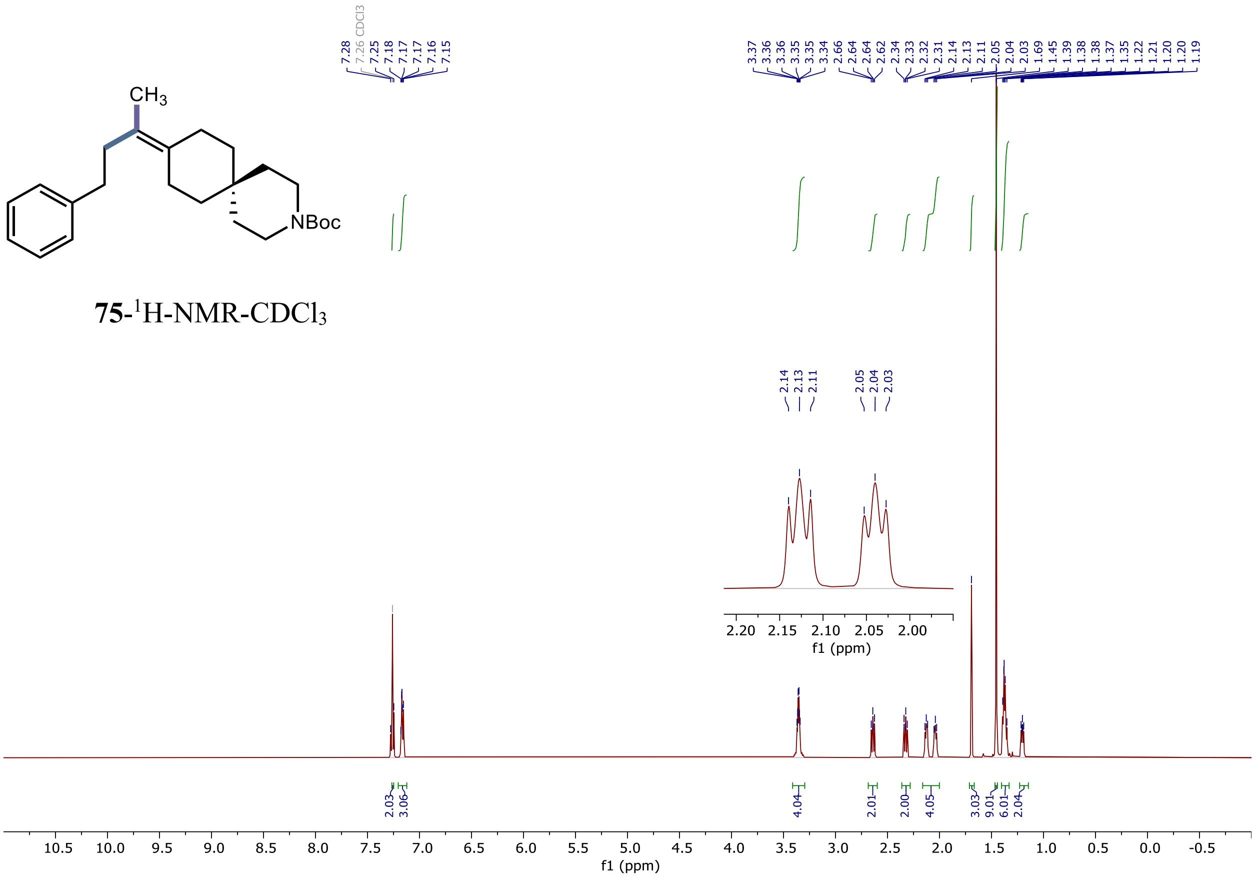

75-^{1}H-NMR-$CDCl_3$

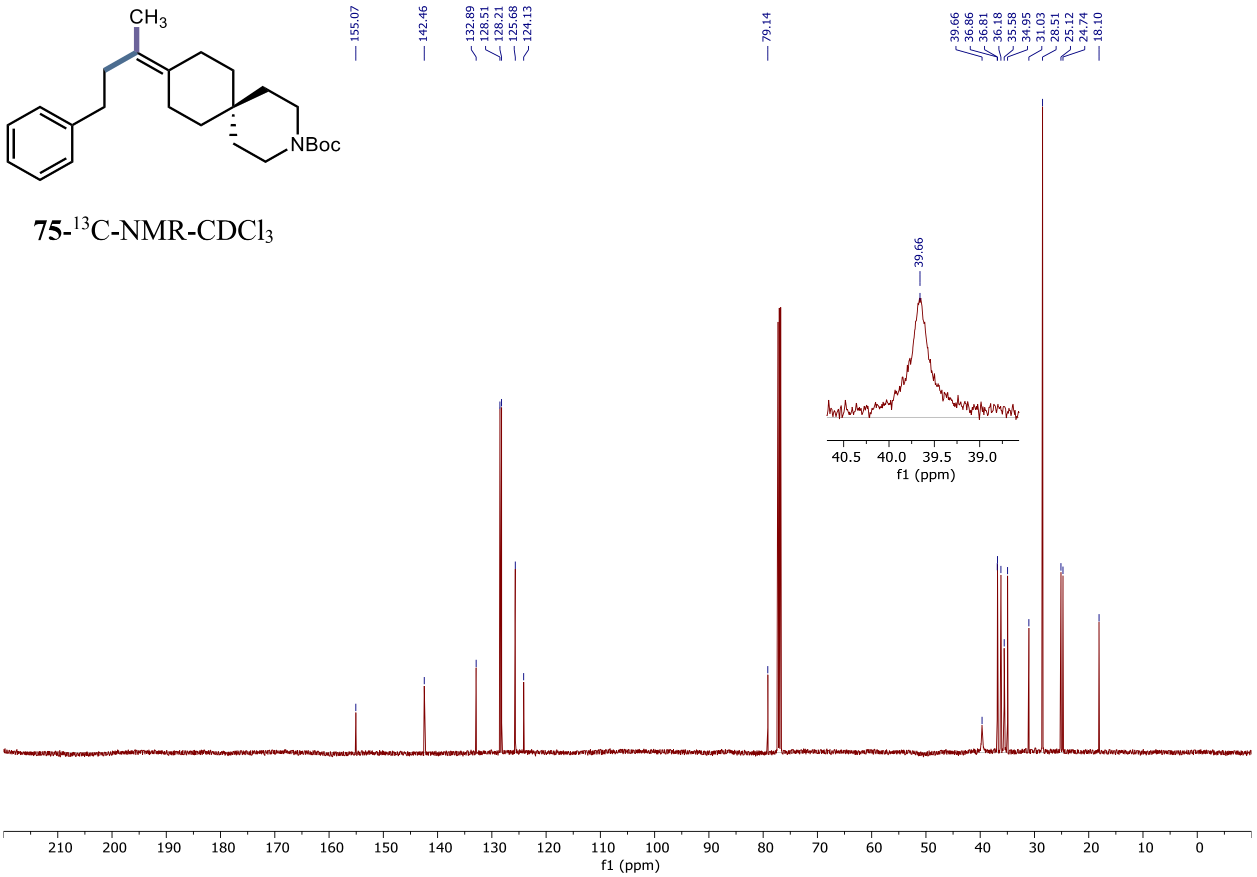

75-^{13}C-NMR-CDCl$_3$

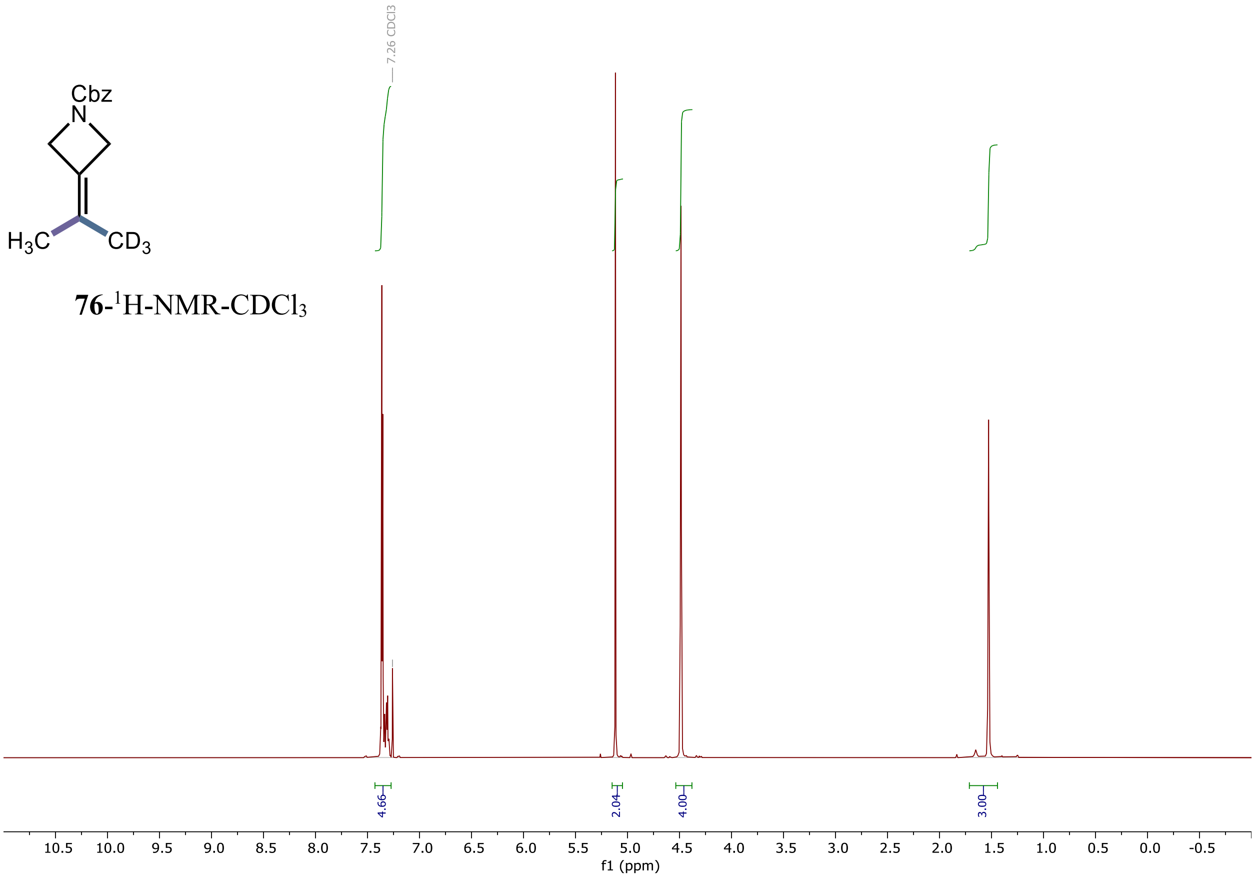

76-^{1}H-NMR-CDCl$_3$

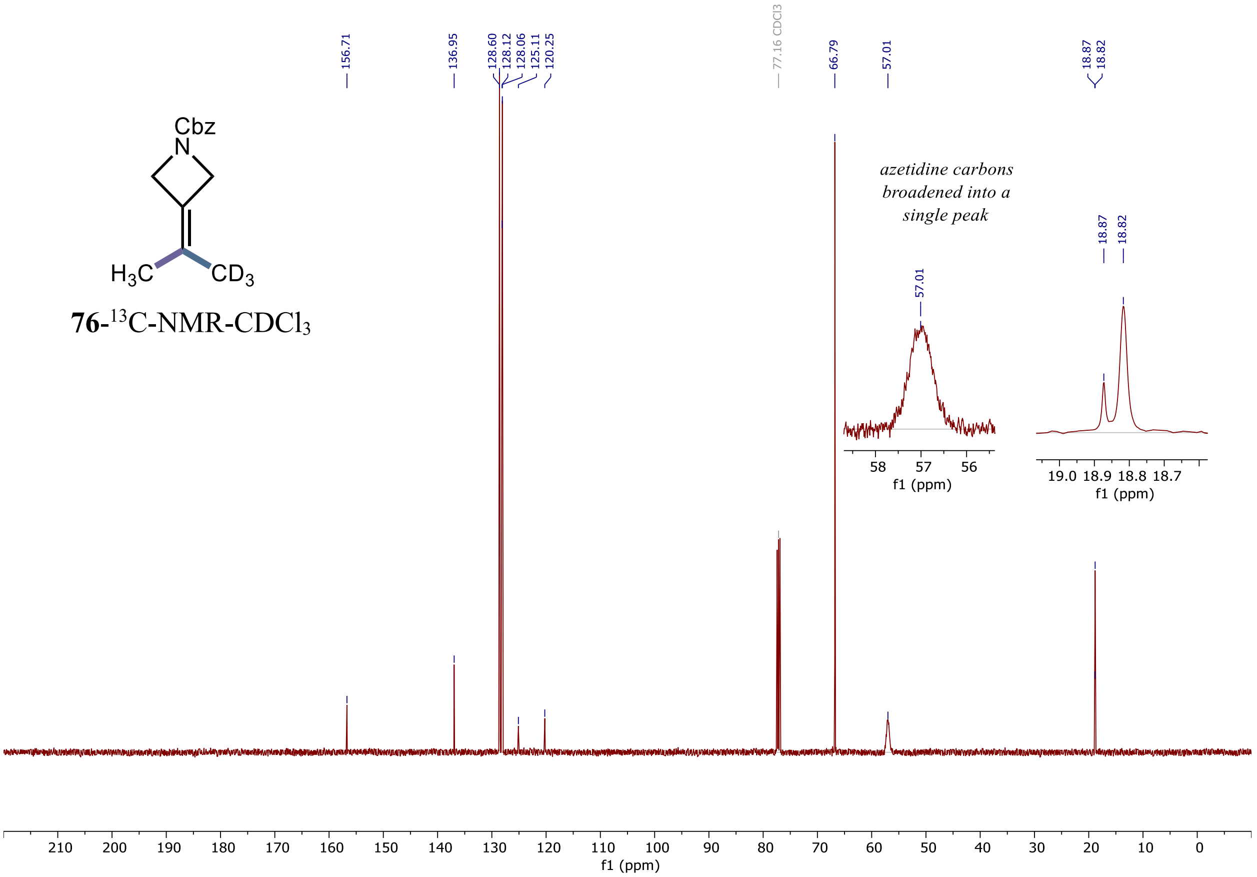
Cbz
N
H3C
CD3
76-13C-NMR-CDCl3
156.71
136.95
128.60
128.12
128.06
125.11
120.25
77.16 CDCl3
66.79
57.01
18.87
18.82
azetidine carbons broadened into a single peak
f1 (ppm)

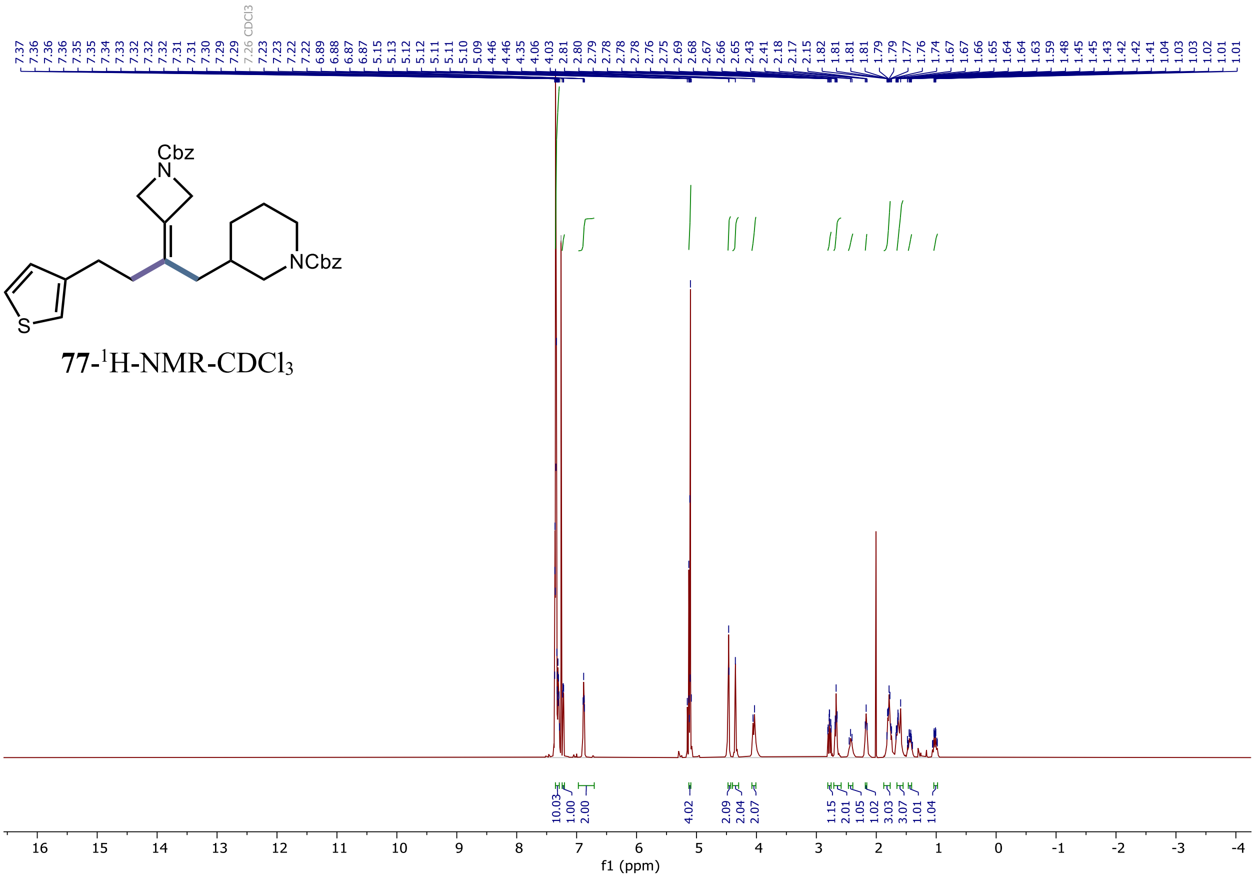
Cbz
N
NCbz
S
77-1H-NMR-CDCl3
f1 (ppm)

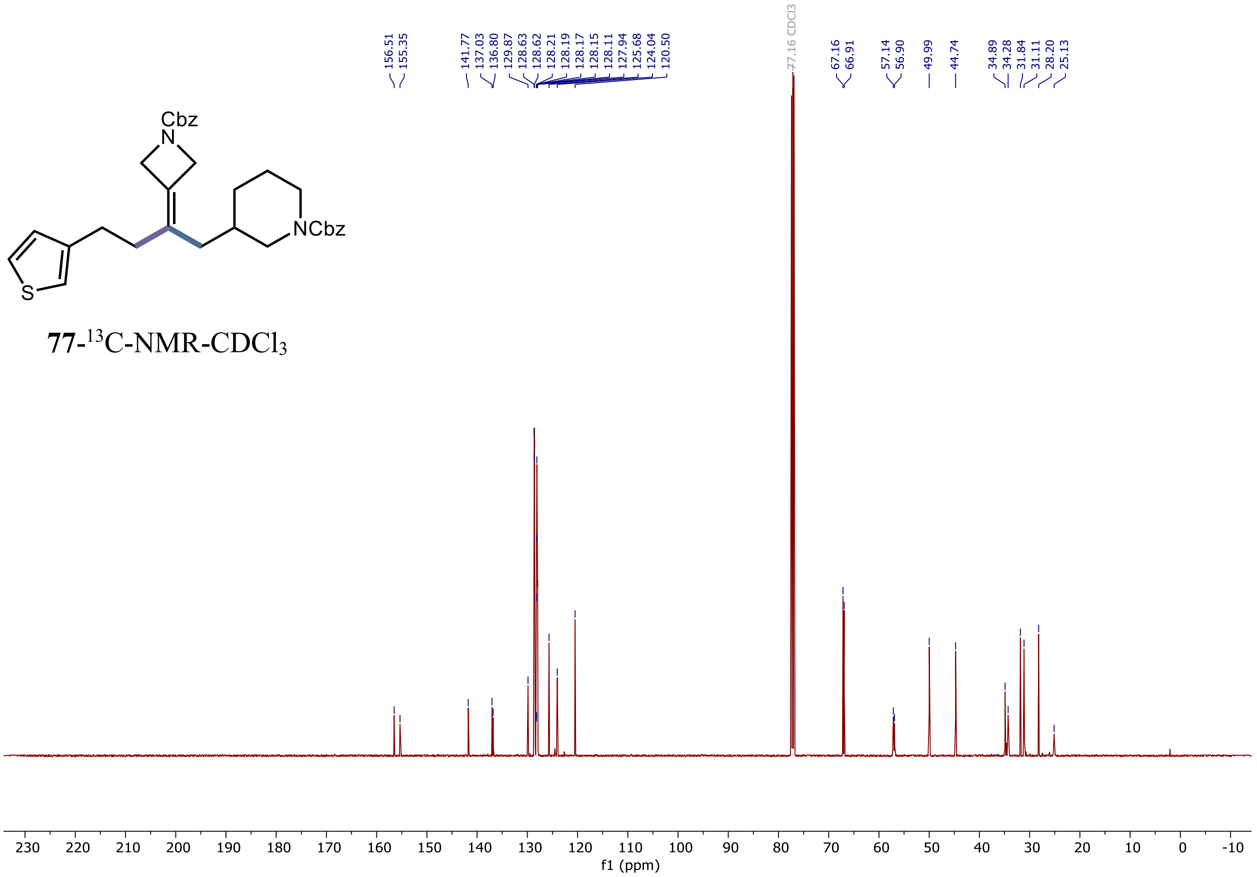

77-^{13}C-NMR-CDCl$_3$

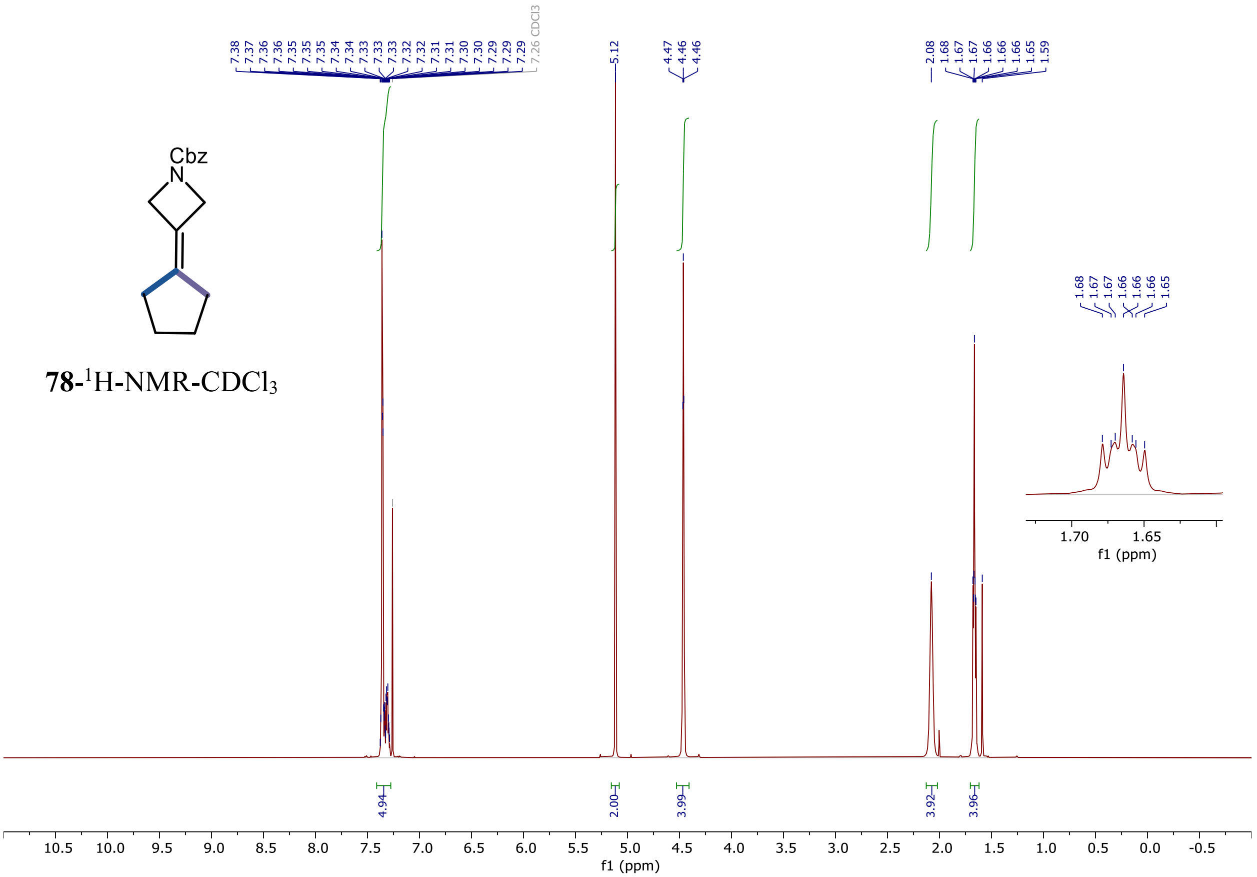

78-^{1}H-NMR-CDCl$_3$

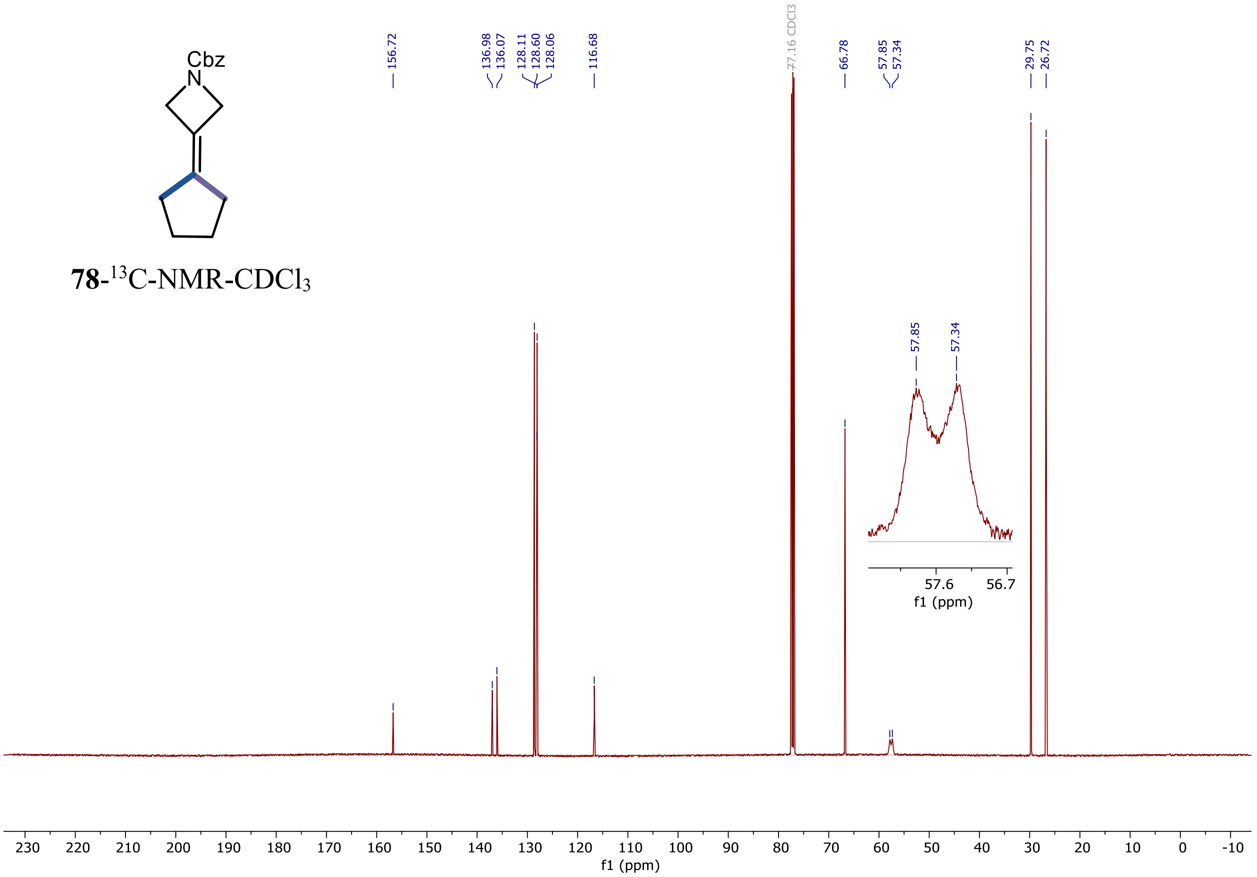

78-^{13}C-NMR-$CDCl_3$

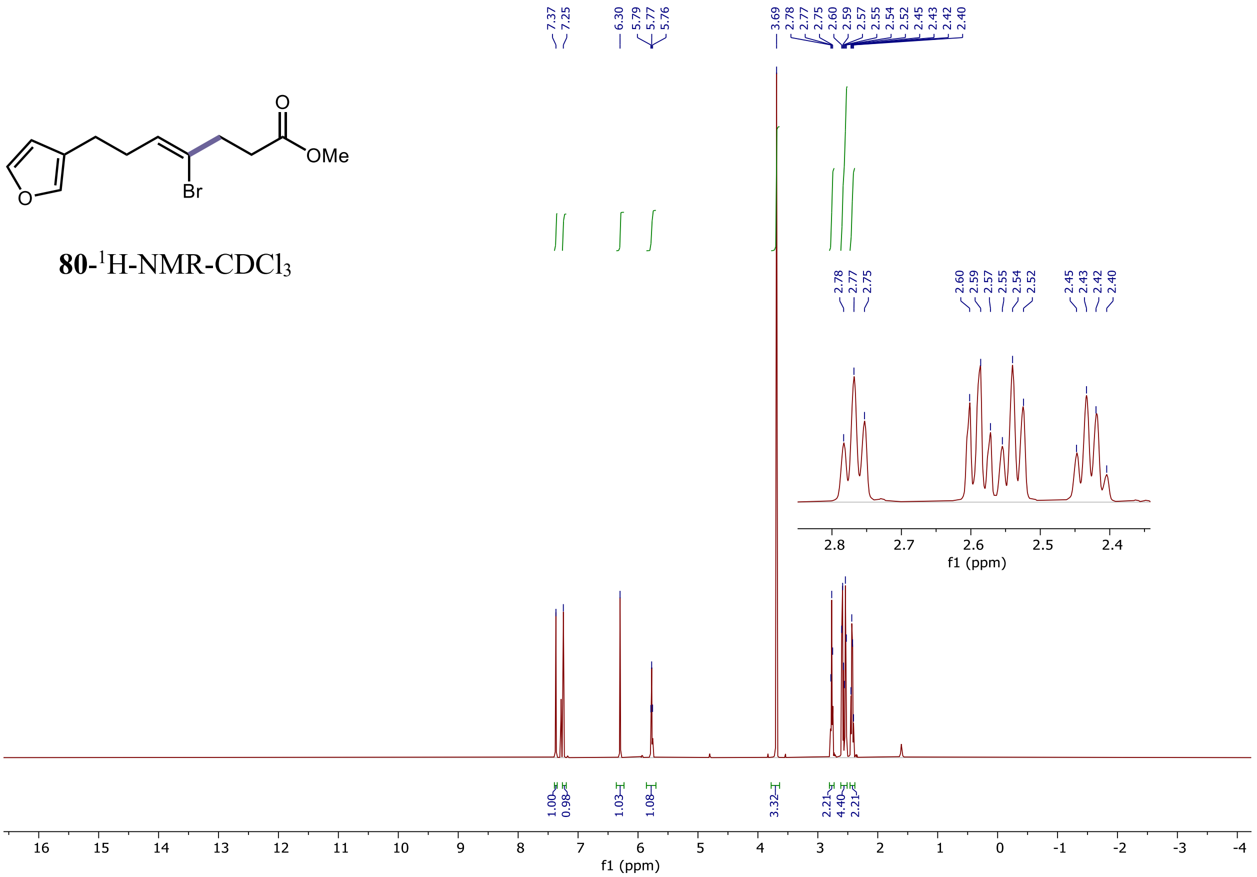

80-^{1}H-NMR-$CDCl_3$

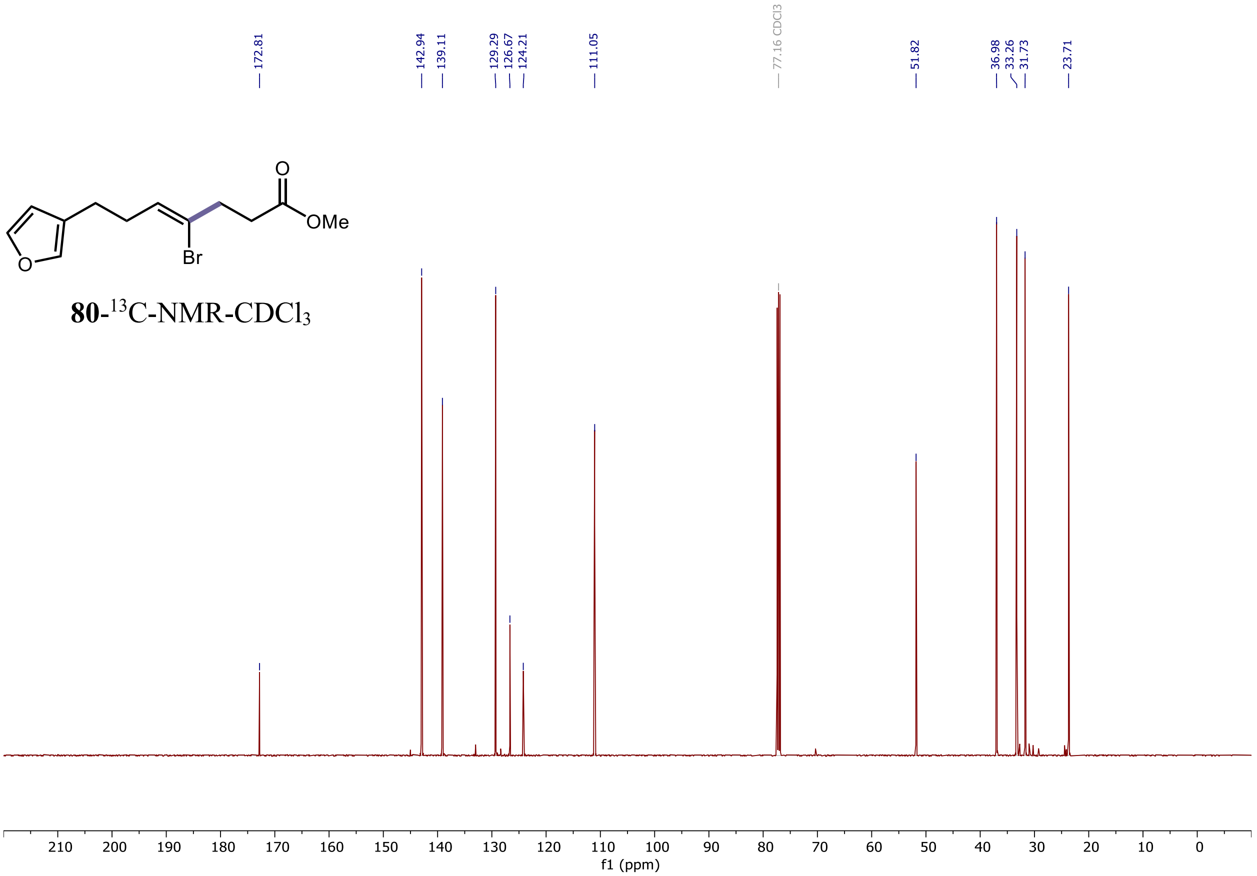
172.81
142.94
139.11
129.29
126.67
124.21
111.05
77.16 CDCl3
51.82
36.98
33.26
31.73
23.71
O
OMe
O
Br
80-^{13}C-NMR-CDCl$_3$
f1 (ppm)

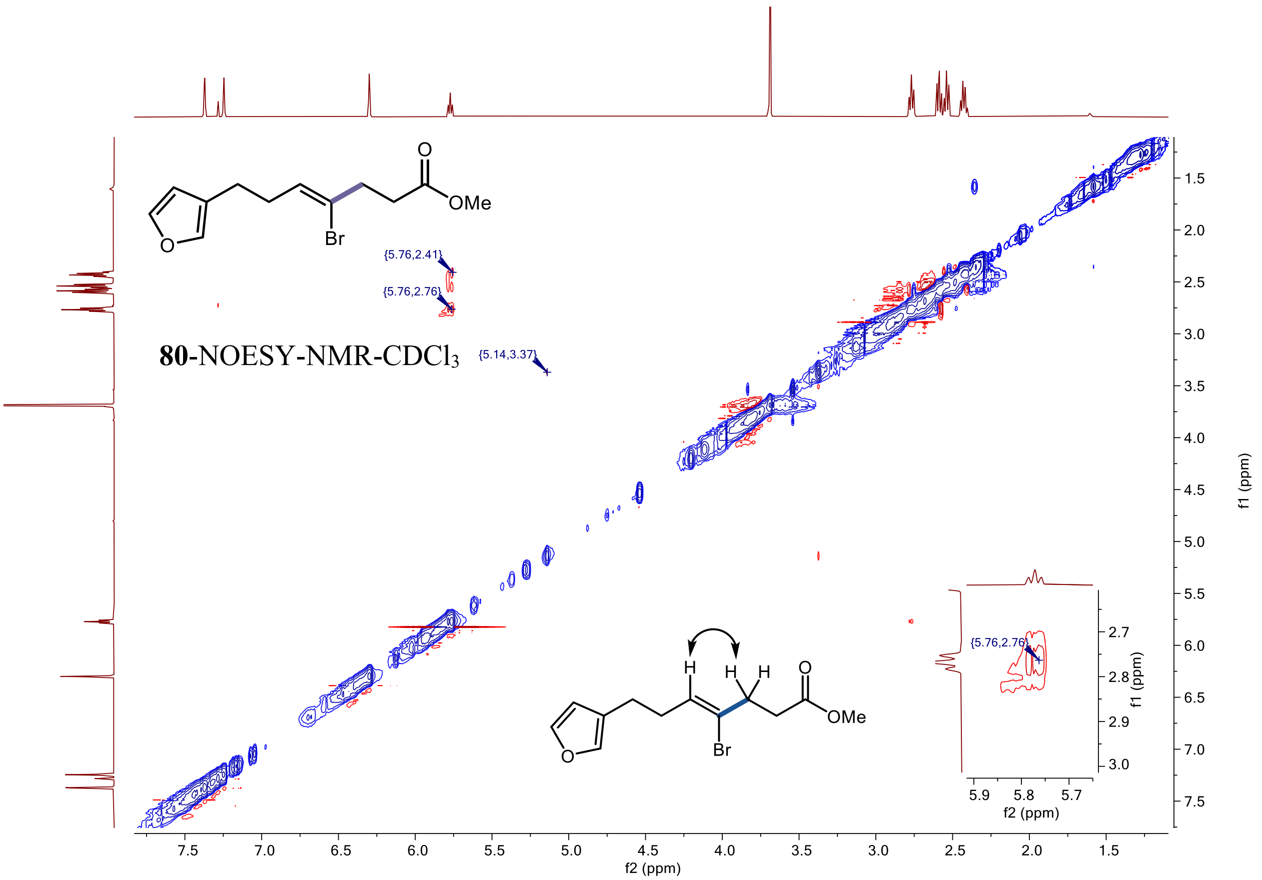
O
OMe
O
Br
{5.76,2.41}
{5.76,2.76}
{5.14,3.37}
80-NOESY-NMR-CDCl$_3$
H
H
H
{5.76,2.76}
f1 (ppm)
f2 (ppm)

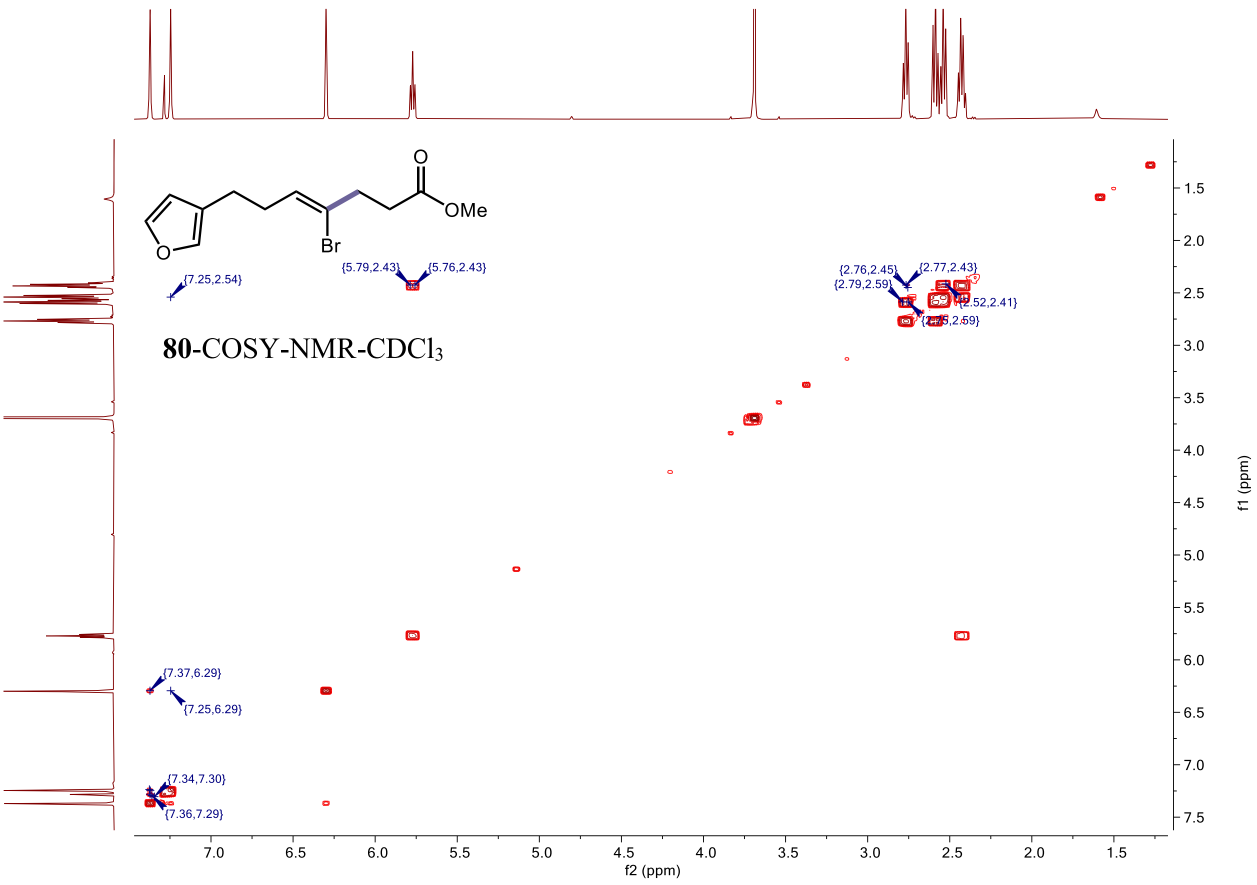
OMe
O
Br
O
80-COSY-NMR-CDCl3
f2 (ppm)
f1 (ppm)

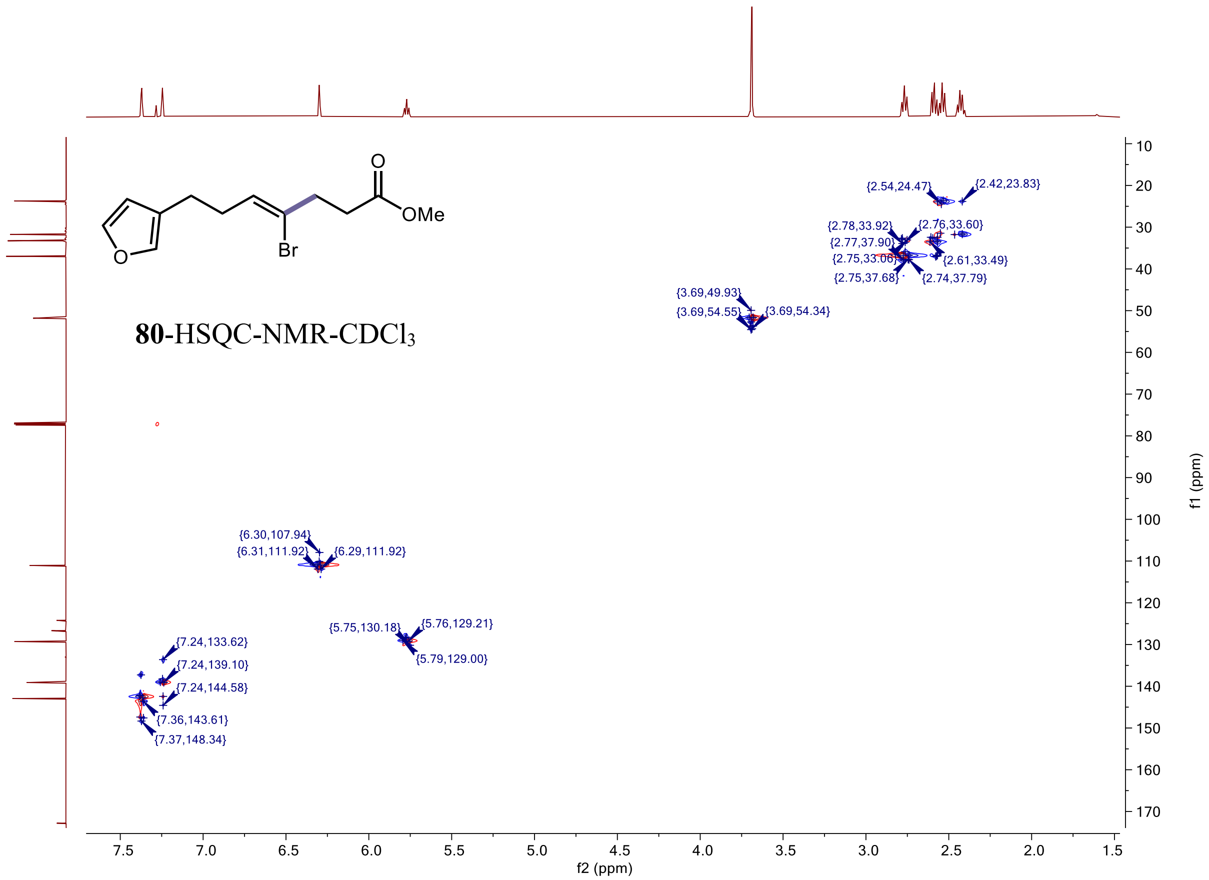
OMe
O
Br
O
80-HSQC-NMR-CDCl3
f2 (ppm)
f1 (ppm)

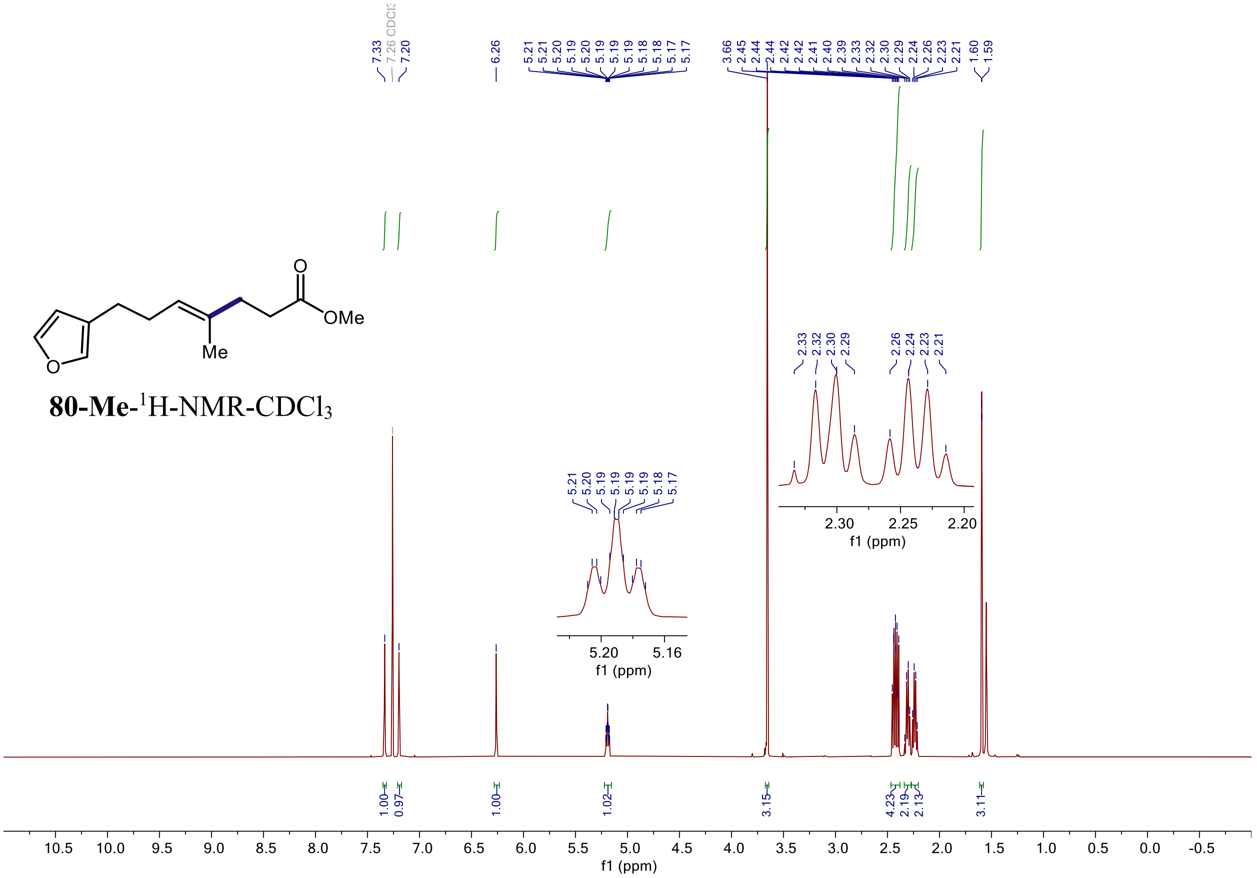
O
OMe
O
Me
80-Me-^{1}H-NMR-CDCl$_3$
7.33
7.26 CDCl3
7.20
6.26
5.21
5.21
5.20
5.19
5.20
5.19
5.19
5.19
5.18
5.18
5.17
5.17
3.66
2.45
2.44
2.44
2.42
2.42
2.41
2.40
2.39
2.33
2.32
2.30
2.29
2.24
2.26
2.23
2.21
1.60
1.59
1.00
0.97
1.00
1.02
3.15
4.23
2.19
2.13
3.11
f1 (ppm)

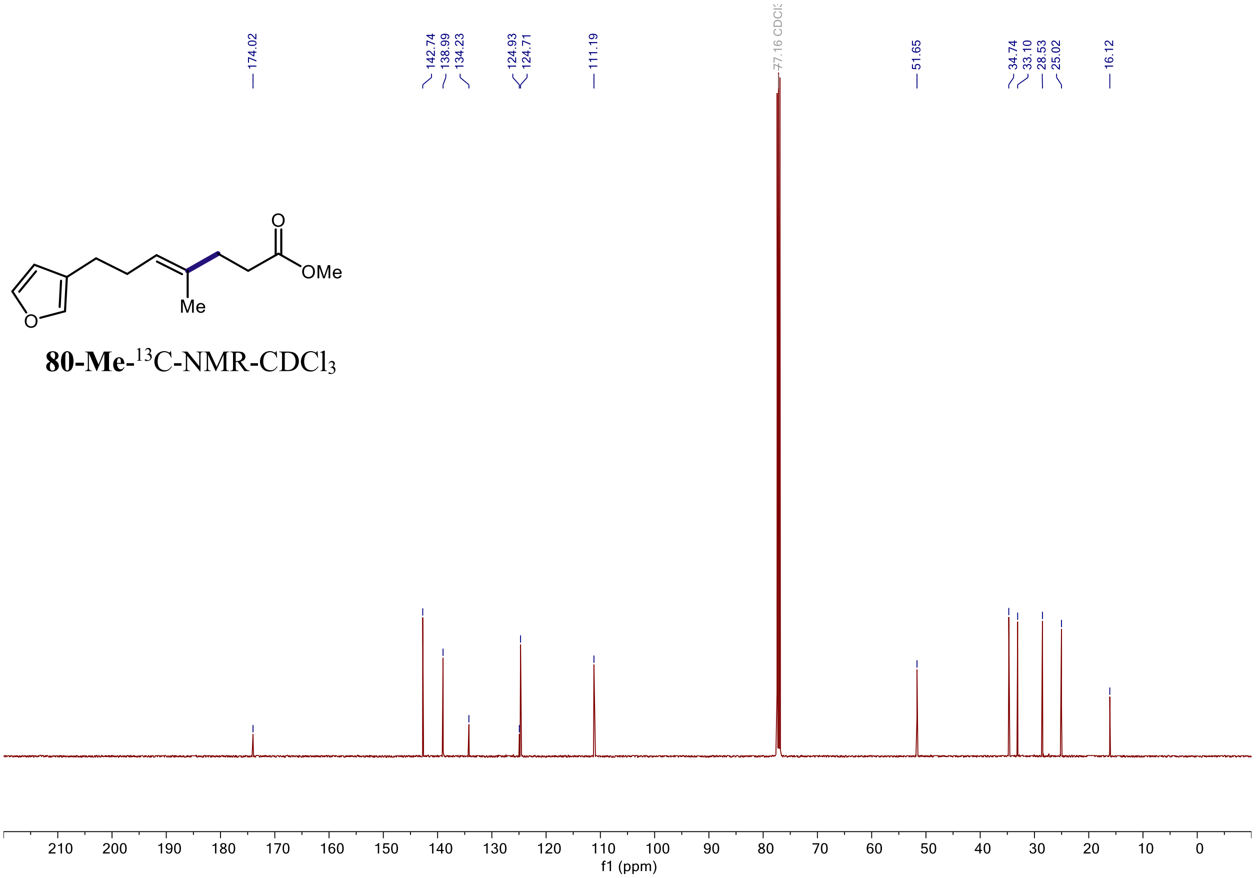
O
OMe
O
Me
80-Me-^{13}C-NMR-CDCl$_3$
174.02
142.74
138.99
134.23
124.93
124.71
111.19
77.16 CDCl3
51.65
34.74
33.10
28.53
25.02
16.12
f1 (ppm)

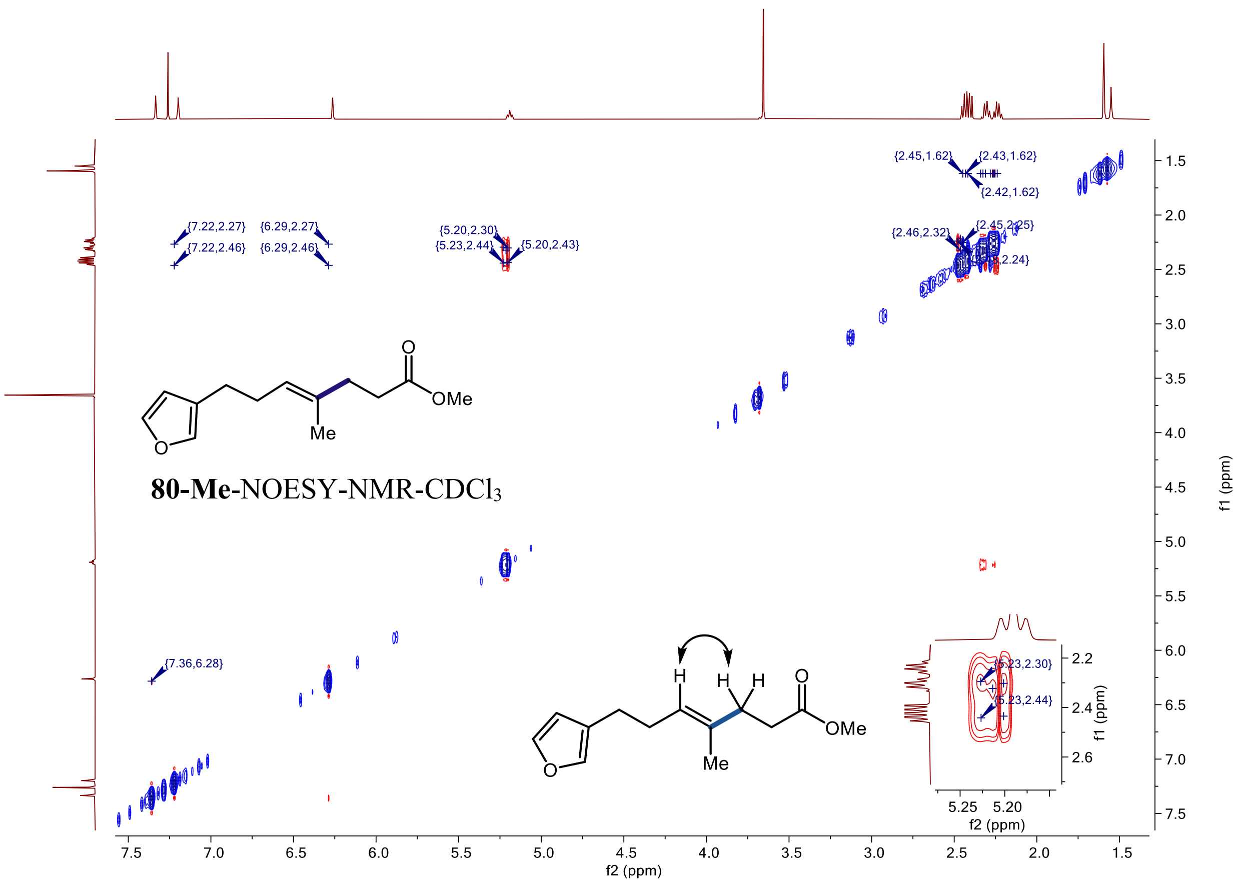
OMe
Me
80-Me-NOESY-NMR-CDCl3
f2 (ppm)
f1 (ppm)

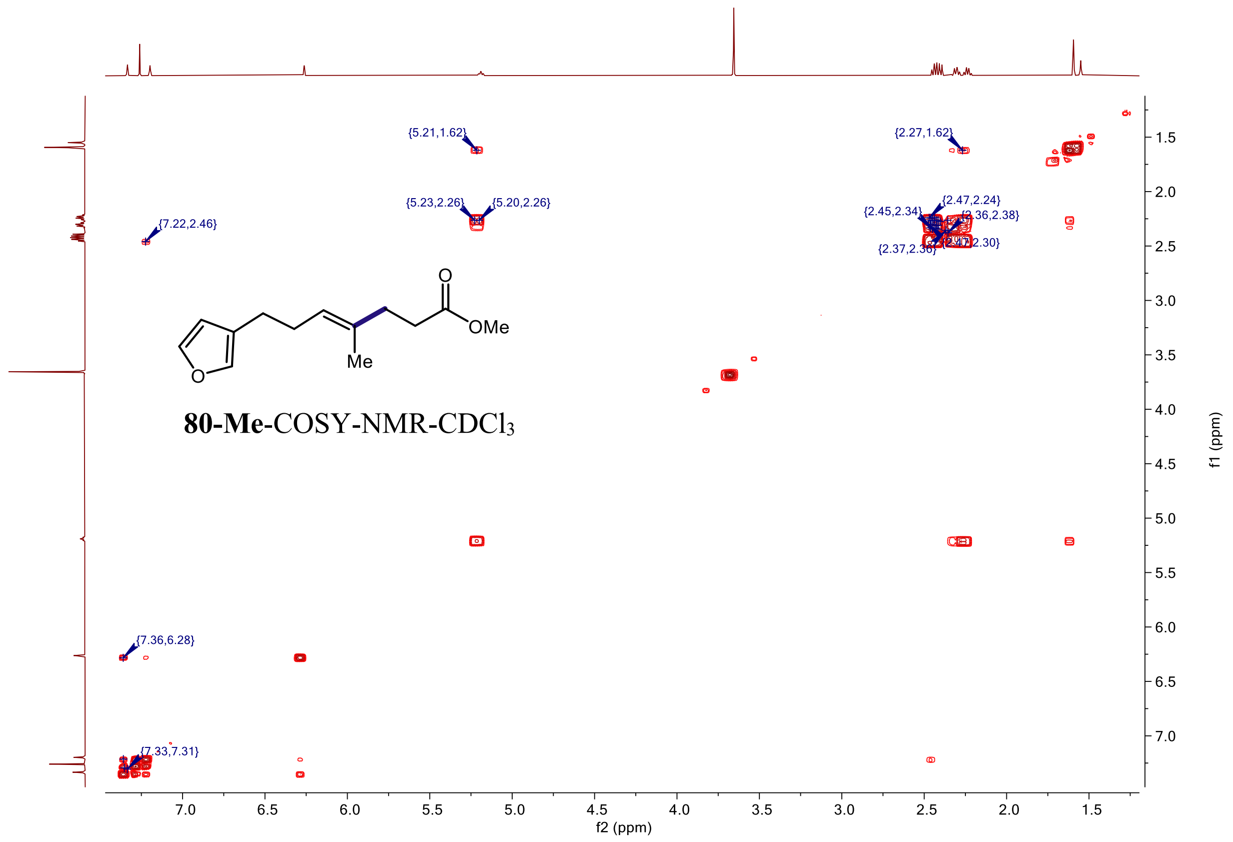
OMe
Me
80-Me-COSY-NMR-CDCl3
f2 (ppm)
f1 (ppm)

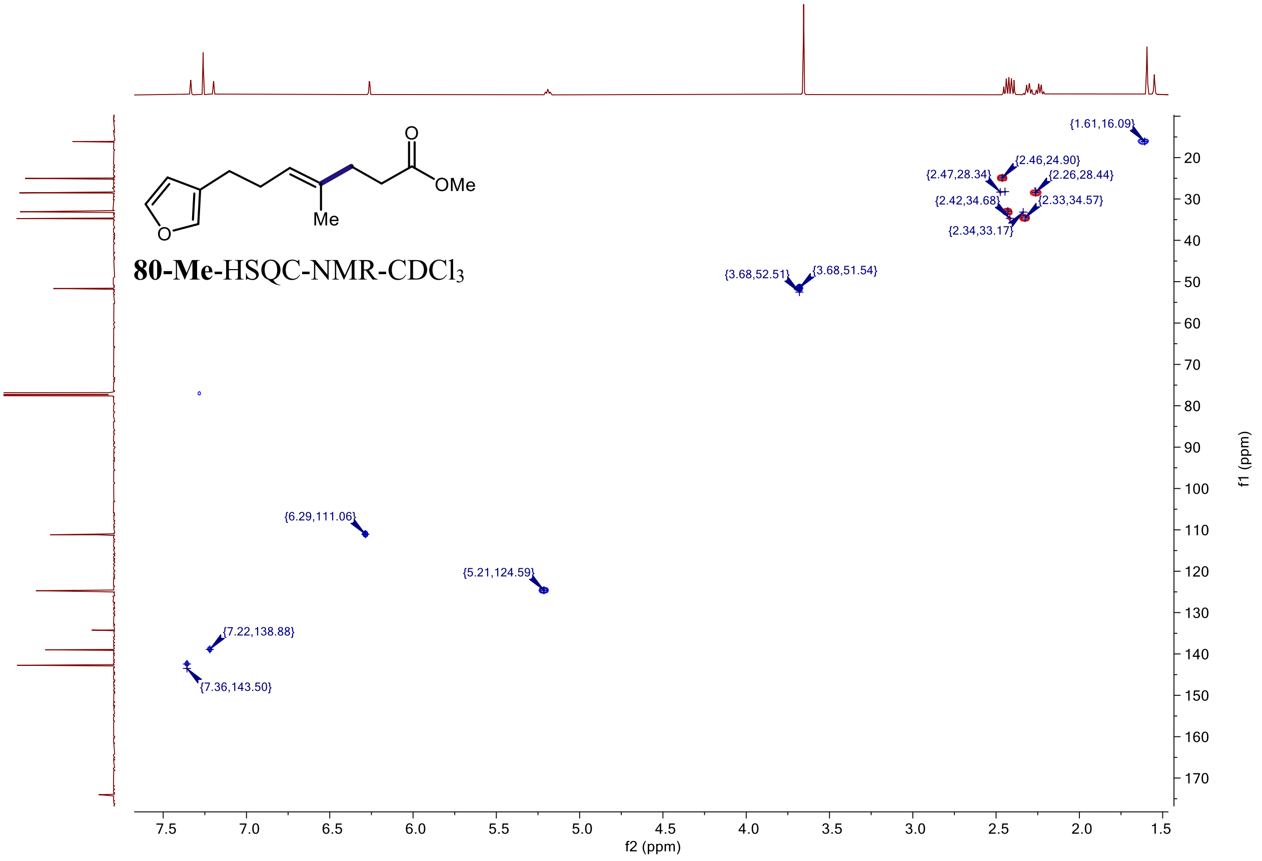

80-Me-HSQC-NMR-$CDCl_3$

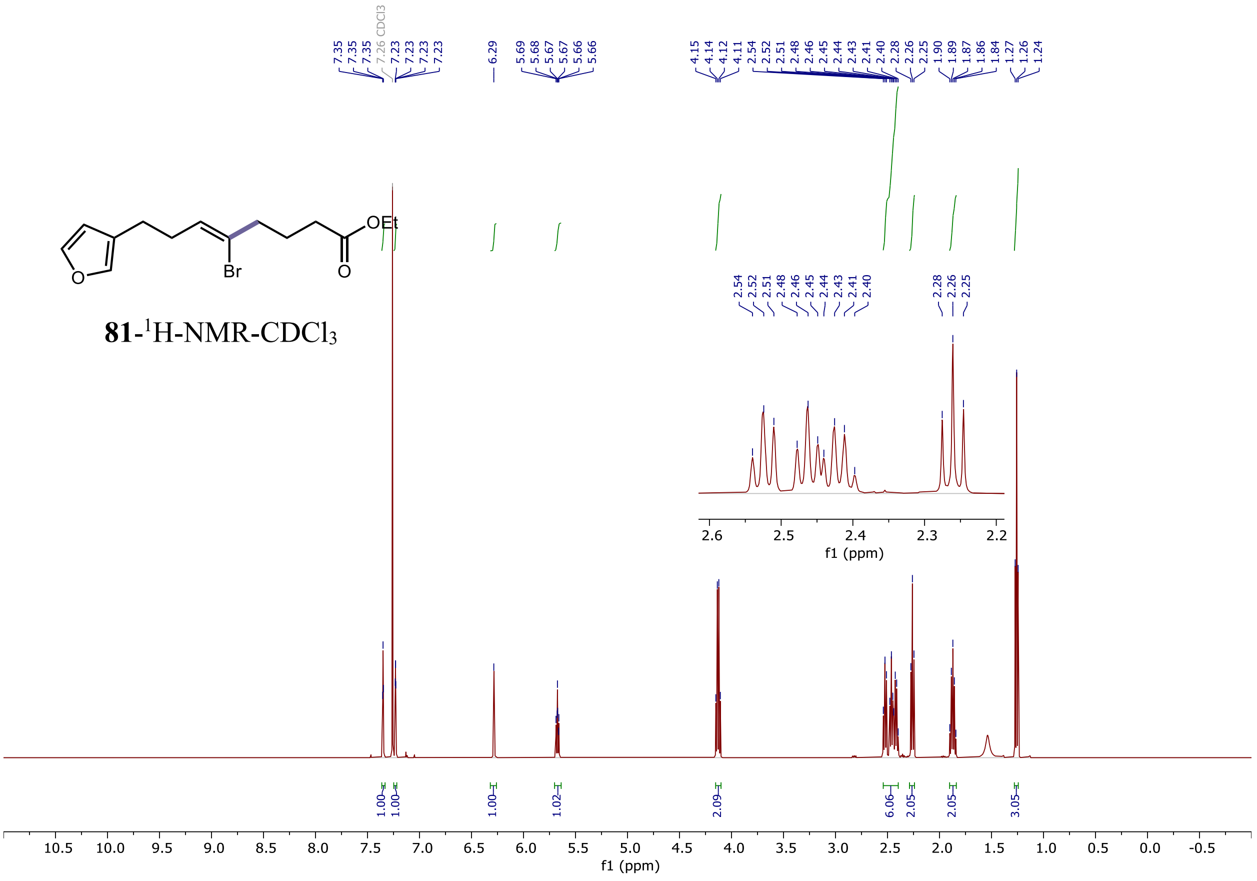

81-^{1}H-NMR-$CDCl_3$

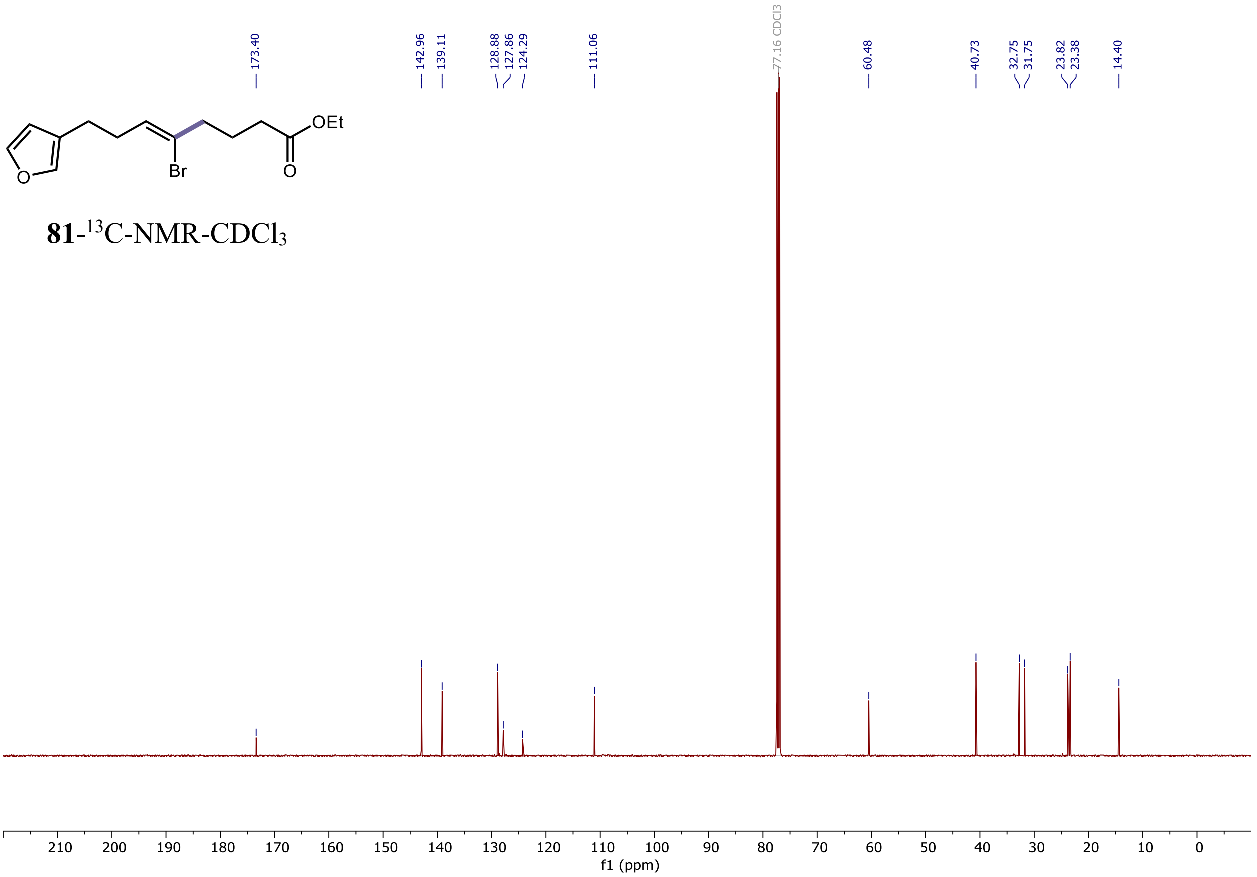

173.40
142.96
139.11
128.88
127.86
124.29
111.06
77.16 CDCl3
60.48
40.73
32.75
31.75
23.82
23.38
14.40
OEt
Br
O
81-13C-NMR-CDCl3
210
200
190
180
170
160
150
140
130
120
110
100
90
80
70
60
50
40
30
20
10
0
f1 (ppm)

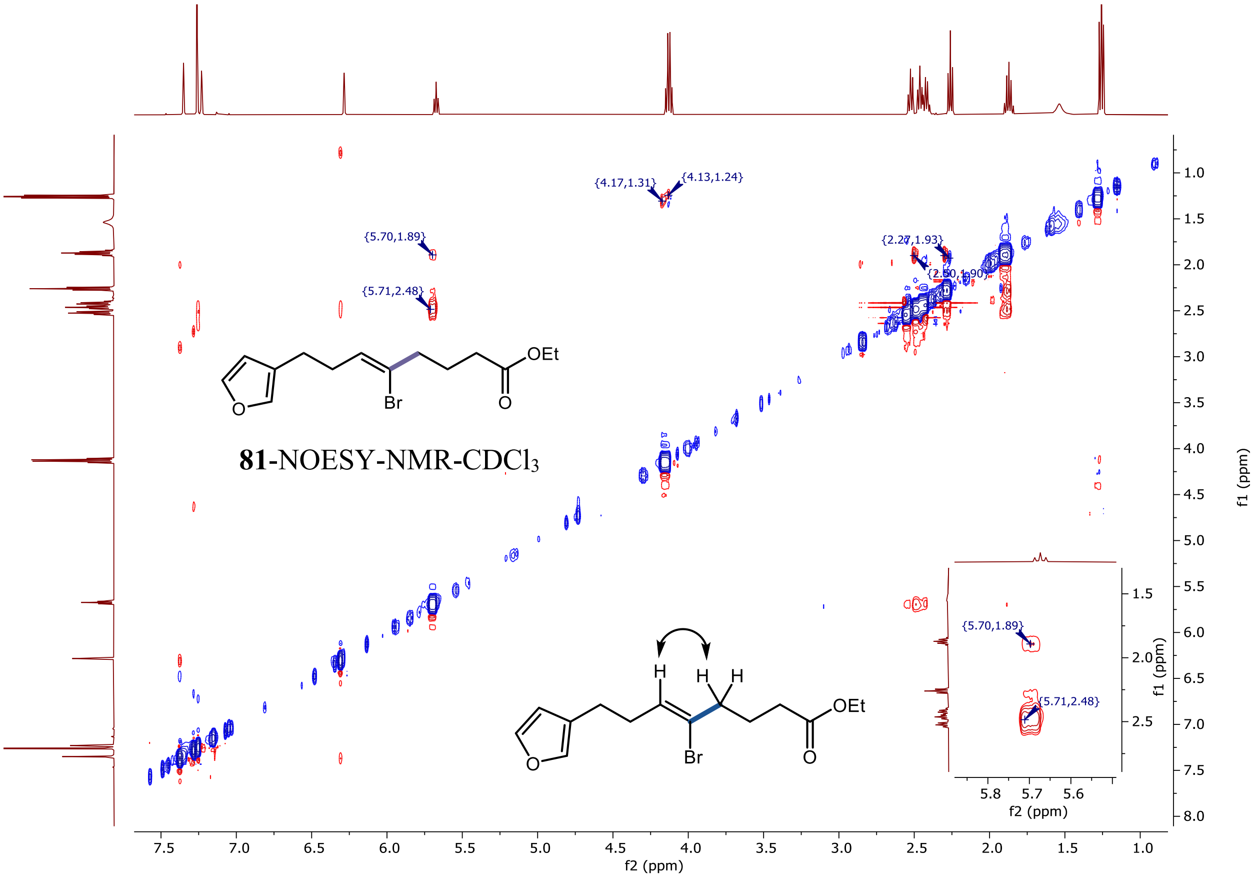

{4.17,1.31}
{4.13,1.24}
{5.70,1.89}
{5.71,2.48}
{2.27,1.93}
{2.50,1.90}
OEt
Br
O
81-NOESY-NMR-CDCl3
H
H
H
f2 (ppm)
f1 (ppm)

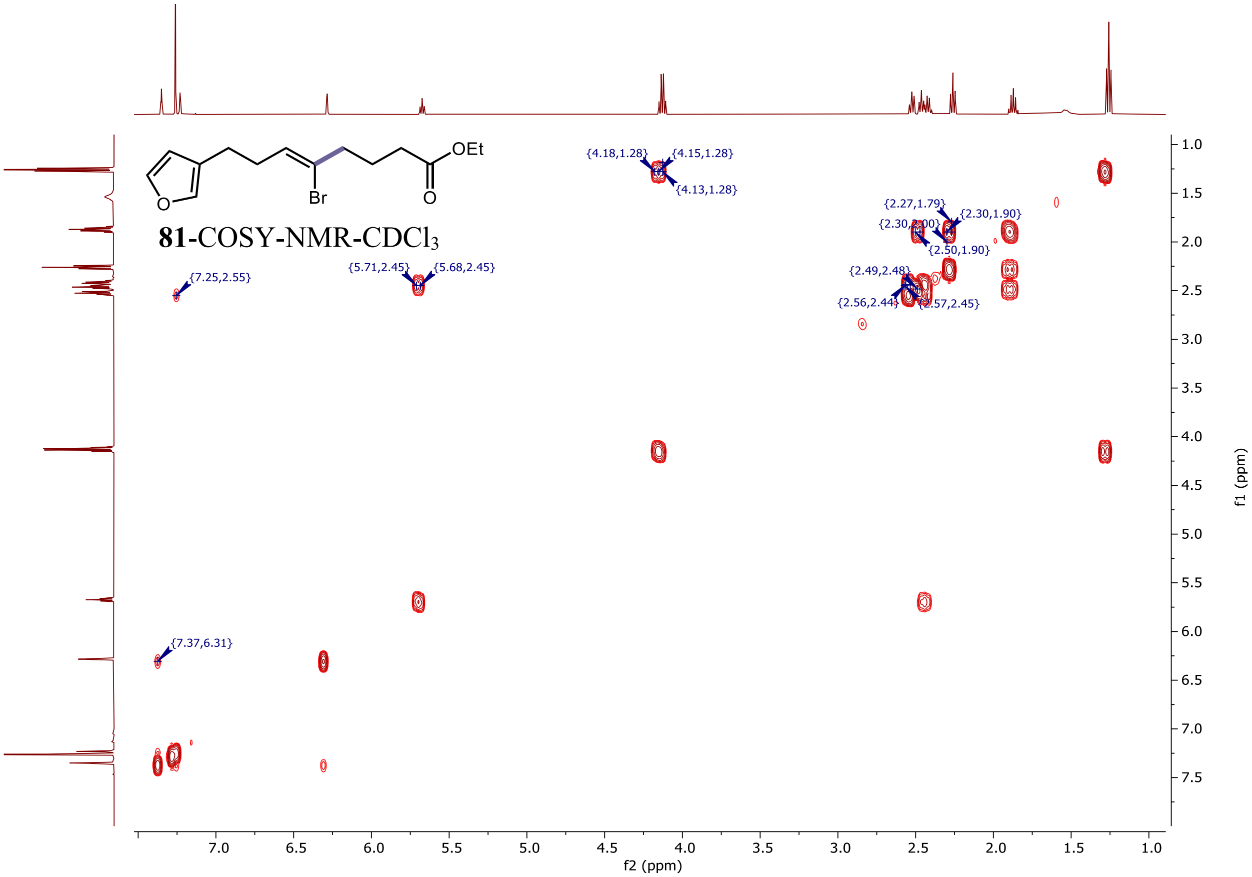
OEt
Br
O
81-COSY-NMR-CDCl3
{4.18,1.28}
{4.15,1.28}
{4.13,1.28}
{2.27,1.79}
{2.30,1.90}
{2.30,2.00}
{2.50,1.90}
{2.49,2.48}
{2.56,2.44}
{2.57,2.45}
{7.25,2.55}
{5.71,2.45}
{5.68,2.45}
{7.37,6.31}
f1 (ppm)
f2 (ppm)

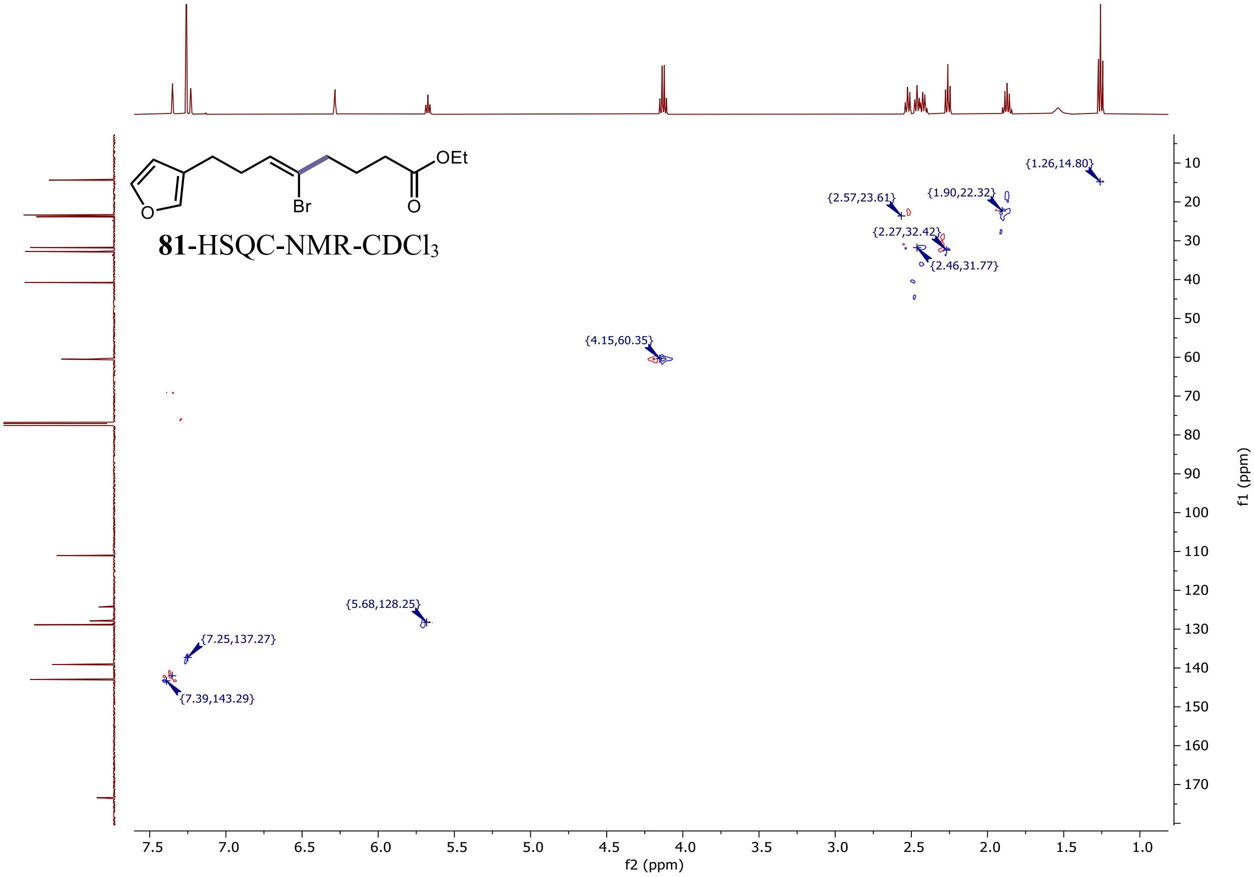
OEt
Br
O
81-HSQC-NMR-CDCl3
{1.26,14.80}
{2.57,23.61}
{1.90,22.32}
{2.27,32.42}
{2.46,31.77}
{4.15,60.35}
{5.68,128.25}
{7.25,137.27}
{7.39,143.29}
f1 (ppm)
f2 (ppm)

12) References

(1) Dong, Z.; MacMillan, D. W. C. Metallaphotoredox-Enabled Deoxygenative Arylation of Alcohols. *Nature* **2021**, *598* (7881), 451–456. https://doi.org/10.1038/s41586-021-03920-6.

(2) Xiang, J.; Yuan, R.; Wang, R.; Yi, N.; Lu, L.; Zou, H.; He, W. Method for Transforming Alkynes into (*E*)-Dibromoalkenes. *J. Org. Chem.* **2014**, *79* (23), 11378–11382. https://doi.org/10.1021/jo501776b.

(3) Geunes, E. P.; Meinhardt, J. M.; Wu, E. J.; Knowles, R. R. Photocatalytic Anti-Markovnikov Hydroamination of Alkenes with Primary Heteroaryl Amines. *J. Am. Chem. Soc.* **2023**, *145* (40), 21738–21744. https://doi.org/10.1021/jacs.3c08428.

(4) Jones, B. T.; García-Cárceles, J.; Caiger, L.; Hazelden, I. R.; Lewis, R. J.; Langer, T.; Bower, J. F. Complex Polyheterocycles and the Stereochemical Reassignment of Pileamartine A via Aza-Heck Triggered Aryl C–H Functionalization Cascades. *J. Am. Chem. Soc.* **2021**, *143* (38), 15593–15598. https://doi.org/10.1021/jacs.1c08615.

(5) Sun, D.-Y.; Han, G.-Y.; Yang, N.-N.; Lan, L.-F.; Li, X.-W.; Guo, Y.-W. Racemic Trinorsesquiterpenoids from the Beihai Sponge *Spongia Officinalis*: Structure and Biomimetic Total Synthesis. *Org. Chem. Front.* **2018**, *5* (6), 1022–1027. https://doi.org/10.1039/C7QO01091E.
